# Role of SNPs in the Biogenesis of Mature miRNAs

**DOI:** 10.1155/2021/2403418

**Published:** 2021-06-17

**Authors:** Ying Wang, Jidong Ru, Xianglian Meng, Jianhua Song, Qingfeng Jiang, Shengqing Li, Jiulei Jiang, Yi Li

**Affiliations:** ^1^College of Equipment Control, Shenyang Ligong University, No. 6, Nanping Middle Road, Hunnan New District, Shenyang, Liaoning 110159, China; ^2^Key Lab of Intelligent Optimization and Information Processing, Minnan Normal University, Fujian, Zhangzhou 363000, China; ^3^School of Computer Science and Engineering, Changshu Institute of Technology, 99 Hushan Road, Changshu, Jiangsu 215500, China; ^4^School of Textile Garment and Design, Changshu Institute of Technology, 8 No. 99, Nansanhuan Road, Changshu, Jiangsu 215500, China; ^5^School of Computer Information & Engineering, Changzhou Institute of Technology, 213032, China; ^6^Beijing Three Brothers Technology Co., Ltd, Department of Technology, Beijing Three Brothers Technology CO., LTD., Baiqiang Road, Beijing 100056, China

## Abstract

Single nucleotide polymorphisms (SNPs) play a significant role in microRNA (miRNA) generation, processing, and function and contribute to multiple phenotypes and diseases. Therefore, whole-genome analysis of how SNPs affect miRNA maturation mechanisms is important for precision medicine. The present study established an SNP-associated pre-miRNA (SNP-pre-miRNA) database, named miRSNPBase, and constructed SNP-pre-miRNA sequences. We also identified phenotypes and disease biomarker-associated isoform miRNA (isomiR) based on miRFind, which was developed in our previous study. We identified functional SNPs and isomiRs. We analyzed the biological characteristics of functional SNPs and isomiRs and studied their distribution in different ethnic groups using whole-genome analysis. Notably, we used individuals from Great Britain (GBR) as examples and identified isomiRs and isomiR-associated SNPs (iso-SNPs). We performed sequence alignments of isomiRs and miRNA sequencing data to verify the identified isomiRs and further revealed GBR ethnographic epigenetic dominant biomarkers. The SNP-pre-miRNA database consisted of 886 pre-miRNAs and 2640 SNPs. We analyzed the effects of SNP type, SNP location, and SNP-mediated free energy change during mature miRNA biogenesis and found that these factors were closely associated to mature miRNA biogenesis. Remarkably, 158 isomiRs were verified in the miRNA sequencing data for the 18 GBR samples. Our results indicated that SNPs affected the mature miRNA processing mechanism and contributed to the production of isomiRs. This mechanism may have important significance for epigenetic changes and diseases.

## 1. Background

miRNAs are small single-stranded noncoding RNAs [1] that regulate approximately 60% of the transcription process in humans [2, 3]. More than 50% of human miRNAs are located in gene fragment sections and are associated with cancers [4]. SNPs are DNA sequence polymorphisms caused by a single individual diversity variation at the genome level. More than 38 million SNPs, including 140 million small insertion/deletion events and 14,000 structure variations, exist in the human genome and contribute to human phenotypic differences and diseases as molecular markers identified in different research fields [5]. Notably, thousands of SNPs exist in miRNA sequences and their upstream and downstream flanking regions, and greater than 40% of pre-miRNAs contain one SNP [6]. SNPs within pre-miRNA regions may be responsible for several of the reported associations between SNPs, miRNAs, and complex human phenotypes and diseases.

SNPs within pre-miRNA regions affect the required secondary structure and thermodynamics and alter the miRNA maturation process, including Drosha enzyme processing, Dicer enzyme processing, and functional strand choice [7, 8], which are closely related to a variety of phenotypes and diseases [6, 9]. SNPs alter the maturation mechanism of pre-miRNAs. For example, miR-125a, which contains an SNP in the seed region, produces one mature miRNA in one arm, but the miRNA in the other arm is prevented from undergoing Drosha enzyme processing [10]. SNPs change the pre-miRNA processing sites [11]. For example, miR-934, which contains rs73558572, generates five mature miRNAs that are offset (1-2 nt) from the 3′ arm reference mature miRNA [12]. Therefore, SNPs lead to imprecise precursor cropping or dicing and affect the expression level of miRNA [13]. Sun et al. [12] demonstrated that this process was one mechanism for isomiR generation [14].

In recent years, with the discovery of a large number of SNPs, miRNAs, and isomiRs and their functions in disease risk, a series of databases, software, and tools have been developed. ISOMIREX was developed to identify miRNAs and isomiRs based on next-generation sequencing data. miR-isomiRExp analyzed miRNA expression patterns at the miRNA/isomiR level and researched the maturation and processing mechanisms of miRNA/isomiR to reveal the functional characteristics of miRNA/isomiR [15]. ISOMIR Bank collected 308,919 isomiRs within 4706 miRNAs which used next-generation sequencing data and analyzed the function of isomiRs [16]. miRNANP constructed an SNP-related miRNA database; it contained 2257 SNPs within 1596 pre-miRNAs and presented the target genes, free energy, and structural change [17]. MSDD captures the relationships between experimentally verified miRNAs, SNPs, genes, and diseases; it recorded 182 human miRNAs, 197 SNPs, 153 genes, and 525 interrelationships [18]. miRVaS provides the locations of the variants in miRNA and predicts the structure changes resulting from these variants [19]. miRvar studied the miRNA maturation mechanism that used early data; it extracted 106 SNPs located in 85 miRNA, identified mature miRNAs by phdcleAV and RISCbinder, and obtained canonical and isomiRs [20].

The above studies identified canonical and isomiR by observing the expression level of miRNA. miRvar made a preliminary study on miRNA maturation mechanism, but large data resources will help identify more functional SNPs and isomiRs. The function of isomiR studies based on the influence of SNV on miRNA maturation mechanism remains to be studied further.

SNPs play significant roles in miRNA generation, processing, and function via different molecular mechanisms and are closely related to various diseases and phenotypes [12]. However, how SNPs affect mature miRNA biogenesis is not clear. Therefore, studies of SNP-affected miRNA maturation mechanisms will provide evidence for causal SNPs and contribute to precision medicine.

**Figure 1:**
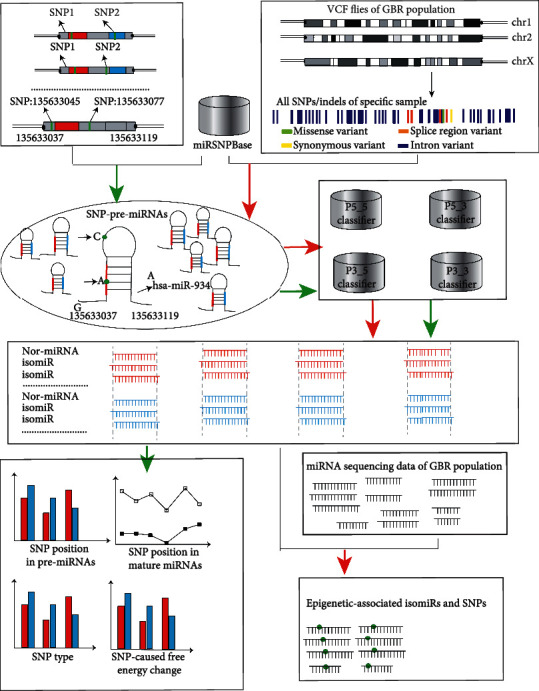
The schematics of the overall method. There are two pathways in the method. The pathways with the green arrows indicate the genome-wide analysis of the role of SNPs in the biogenesis of mature miRNAs. The pathways with red arrows are examples of the identification of isomiRs and iso-SNPs of GBR population based on our method.

The present study performed a genome-wide analysis of the role of SNPs in the biogenesis of mature miRNAs. We present a database, miRSNPBase, which provides comprehensive information about SNPs and SNP-associated miRNA loci. All pre-miRNAs and SNPs were surveyed using coordinates in the human genome. Mature sites of SNP-pre-miRNA were predicted based on miRFind [21] to identify isomiRs. We also analyzed the effects on SNP type, SNP location, and SNP-affected free energy change during the biogenesis of mature miRNAs. We verified the predicted isomiRs based on the miRNA sequencing data of 18 individuals from GBR. A schematic of the overall method is illustrated in Figure 1.

SNPs were mapped to pre-miRNAs according to coordinates based on miRBase and 1000 Genomes Project; all SNPs and associated pre-miRNAs were used to construct the miRSNPBase. There are two pathways in the method. The pathways with the green arrows indicate the genome-wide analysis of the role of SNPs in the biogenesis of mature miRNAs. For the genome-wide analysis, all SNPs and associated pre-miRNAs of miRSNPBase were used to construct SNP-pre-miRNAs, and mature miRNAs were identified based on miRFind; all isomiRs and nor-miRNA were identified by aligning with normal mature miRNA; to study the effects of SNPs on the biogenesis of mature miRNAs, the distribution of SNP position in pre-miRNAs and mature miRNAs, SNP type, and free energy change were studied. The pathways with red arrows are examples of the identification of isomiRs and iso-SNPs of GBR population based on our method. We extracted the SNPs of each chromosome for 18 GBR from the VCF files and integrated all the SNPs for each GBR sample. All SNPs were mapped to pre-miRNAs to construct the SNP-pre-miRNA for each sample, and then, mature miRNAs were identified based on miRFind; furthermore, the nor-miRNA and isomiR candidates were provided; finally, the RNA sequencing data of 1000 Genomes Project was used to validate and mine the isomiRs.

## 2. Methods

### 2.1. Data

#### 2.1.1. SNP-Associated Dataset

Information on SNPs was obtained from the 1000 Genomes Project (ftp://ftp-trace.ncbi.nih.gov/1000genomes/ftp/release/20130502/), which was constructed based on 2504 individuals from 26 populations and includes over 84.7 million SNPs, with >99% of SNPs having a frequency of >1% for a variety of ancestries. An example of the SNP information is shown in Table 1.

#### 2.1.2. miRNASNP Dataset

The miRNASNP dataset (http://bioinfo.life.hust.edu.cn/miRNASNP/#!/) is a database cataloguing 2257 SNPs in 1596 human pre-miRNAs based on miRBase [22] version 19 and the dbSNP database (version 137).

### 2.2. Validation Data

The validation data included variant call format (VCF) files, the miRNA sequencing data of GBR, and the Homo sapiens GRCh37 reference sequence (http://bioinfo.hpc.cam.ac.uk/downloads/datasets/fasta/grch37/).

The 1000 Genomes Project consists of 26 subpopulations from five major populations (Americans, Europeans, East Asians, South Asians, and Africans). The VCF file contains the final variant call set with phased genotypes for chr1-22, chrX, and chrY of 2504 individuals from 26 populations based on the phase 3 analysis of the 1000 Genomes sequence data. Eighteen GBR individuals of European origin were selected for our study. We downloaded the VCF files of GBR population-specific SNP data of the 18 individuals from the ftp server of the 1000 Genomes Project (ftp://ftp.1000genomes.ebi.ac.uk/vol1/ftp/release/20130502/) [23, 24].

The miRNA sequencing data consisted of the set of human lymphoblastic cell line samples from the GBR population. The dataset was downloaded from https://www.ebi.ac.uk/arrayexpress/experiments/E-GEUV-2/samples/.

The Homo sapiens GRCh37 reference sequence was from 1000 Genomes Project phase 2 and was downloaded from ftp://ftp.1000genomes.ebi.ac.uk/vol1/ftp/technical/reference/human_g1k_v37.fasta.gz.

#### 2.2.1. Identification of SNP-Associated miRNAs (SNP-miRNAs)

miRNA genome position is described as Attr_mir_pos_ = {Name, Coordinates, Chromosome, Start, End, Puls/trans strand}.

The attributes of the SNPs are Attr_snp_ = {Chromosome, Coordinates, SNPID, Reference, Alter, Qual, Filter, Number of allele, Frequency of allele, Amount of allele, Type of variation, Read of the variation}.

SNPs were mapped to miRNAs based on the hg38 and hg19 coordinates, and liftOver (https://genome.ucsc.edu/cgi-bin/hgLiftOver) was used to convert the coordinates from hg38 to hg19. Based on the coordinates, all SNPs were mapped to the pre-miRNAs. All pre-miRNAs and SNPs were used to construct the miRSNPBase. SNP-pre-miRNA in miRSNPBase was defined as {name, plus/trans strand, SNP positions, start of miRNA, end of miRNA, reference nucleotide, alter nucleotide, minor allele frequency}.

miRSNPBase was expanded with miRNASNP, which was developed on the basis of NCBI dbSNP (version 137).

### 2.3. Identification of Processing Sites of SNP-pre-miRNA

**Figure 2:**
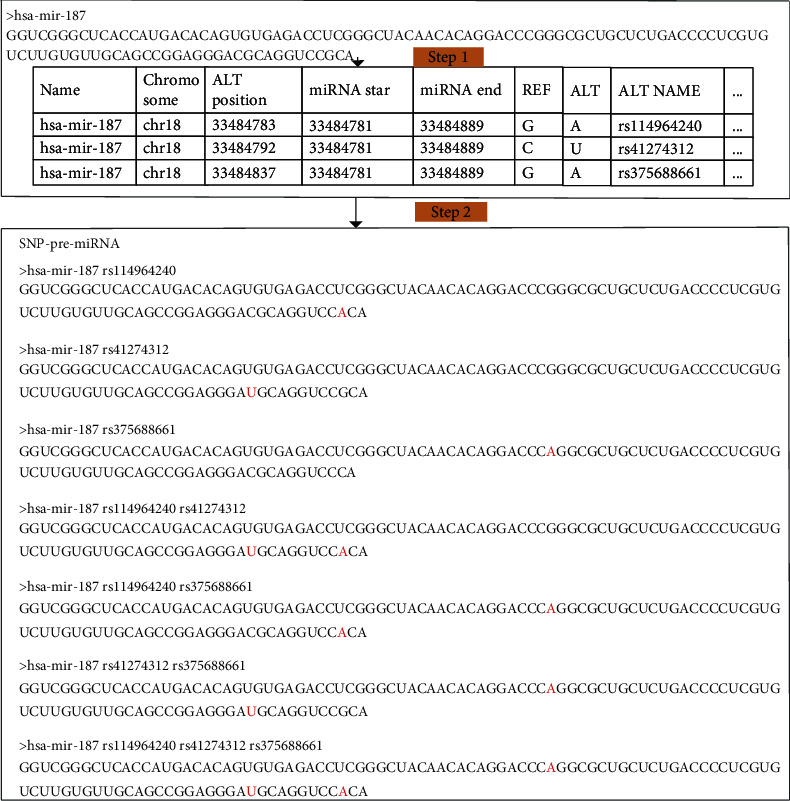
The example of constructing the SNP-pre-miRNA sequence. There are two steps in constructing the SNP-pre-miRNA sequence. (a) pre-miRNA coordinates in the human genome were extracted. (b) The variation nucleotides were mapped to the pre-miRNAs based on coordinates.

SNP-pre-miRNAs were constructed based on the combination method, and the specific nucleotides of corresponding positions in the pre-miRNAs were substituted by SNPs. The number of SNP-pre-miRNAs is shown as

(1) $$\begin{array}{c} N_{i}=C_{n}^{1}+C_{n}^{2}+\cdots +C_{n}^{k}+\cdots +C_{n}^{n}, \end{array}$$

(2) $$\begin{array}{c} C_{n}^{k}=\frac{n!}{k!\left(n-k\right)!}, \end{array}$$

where *N*_*i*_ is the number of SNP-pre-miRNAs for the *i*th pre-miRNA and *n* is the number of SNPs mapping to one pre-miRNA based on coordinates. An example of constructing an SNP-pre-miRNA sequence is illustrated in Figure 2.

**Figure 3:**
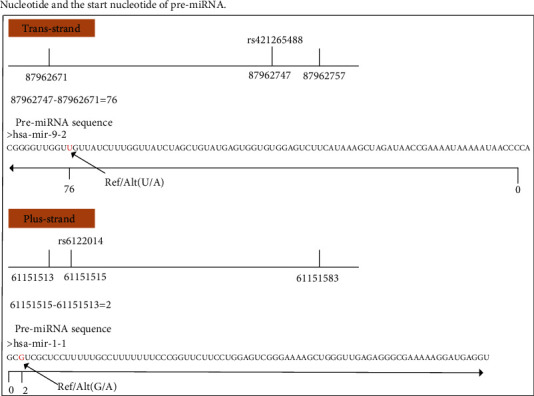
The calculation method for variation position of plus strand and trans strand. The distance between SNP and start (or end) site of pre-miRNA with smaller value was calculated based on coordinate. For the trans strand, the distance represents the length between substitutive position of nucleotide and the end nucleotide of pre-miRNA. For the plus strand, the distance represents the length between substitutive position of nucleotide and the start nucleotide of pre-miRNA.

The SNP coordinates are defined based on the plus or trans strand in this process. For two different situations, we used two different conversion methods. The calculation methods for the variation positions of the plus strand and trans strand are illustrated in Figure 3.

To systematically identify mature miRNAs of the SNP-pre-miRNAs, we used miRFind, which we developed in our previous work. miRFind was developed to identify mature miRNAs within pre-miRNAs, and it provides five mature miRNA candidates with an accuracy as high as 68%. We defined the start and end sites of the 5′ arm mature miRNA as P5_5 and P5_3 and the start and end sites of the 3′ arm mature miRNA as P3_5 and P3_3. Based on the identified mature miRNAs, we extracted the normal miRNAs (nor-miRNAs) and isomiRs. The SNP-pre-miRNAs, pre-miRNAs, and SNPs that associated with isomiRs were defined as iso-SNP-pre-miRNAs, iso-pre-miRNAs, and iso-SNPs, respectively, and the SNP-pre-miRNAs, pre-miRNAs, and SNPs that were associated with nor-miRNAs were defined as nor-SNP-pre-miRNAs, nor-pre-miRNAs, and nor-SNPs, respectively.

### 2.4. Effects of SNPs on the Biogenesis of Mature miRNAs

To study the effects of SNP position, SNP type, and SNP-affected free energy change on the mature miRNA processing mechanism, we investigated the distribution of pre-miRNAs between nor-pre-miRNA and iso-pre-miRNA based on SNP location in pre-miRNAs and the distribution of SNP-pre-miRNAs between nor-SNP-pre-miRNA and iso-SNP-pre-miRNA based on SNP type in pre-miRNAs. We researched the distribution of SNP-pre-miRNAs between nor-SNP-pre-miRNA and iso-SNP-pre-miRNA based on SNP-caused free energy changes of pre-miRNAs. The free energy of each normal pre-miRNA and SNP-pre-miRNA was calculated using RNAfold [25].

### 2.5. Identification and Verification of isomiRs Based on the GBR Population from 1000 Genomes

To verify our method and identify important biomarkers, the VCF files of GBR population-specific SNP data and related miRNA sequencing data were selected. First, we extracted the SNPs of each chromosome based on the VCF files. GenomeAnalysisTK.jar [26] (https://software.broadinstitute.org/gatk/) was used in this process to compare the samples with the human GRCh37 reference sequence and extract chromosome data and VCF files of the 23 chromosomes. Second, we integrated all of the SNPs of the 23 chromosomes. Based on the miRSNPBase, we constructed the pre-miRNA-SNP sequences. Third, we identified four processing sites of the pre-miRNA-SNP sequences and extracted the canonical miRNAs and isomiR candidates. Finally, we aligned all of the isomiRs with the miRNA sequencing data of the GBR samples. miRNAs that were found in the miRNA sequencing data were the verified isomiRs.

## 3. Results

### 3.1. Establishment of the miRSNPBase Database

A total of 1881 human pre-miRNAs were extracted from miRBase. On the basis of the coordinates in the genome, SNPs were mapped to the pre-miRNAs and flanking regions, and we found 2146 SNPs located in the pre-miRNAs. Among these SNPs, 995 SNPs were found in the dbSNP, and 1151 SNPs were not in the dbSNP. These results demonstrated that our method afforded a great degree of data integrity. Therefore, the miRNASNP data were integrated to construct our database, named miRSNPBase. The miRSNPBase included 886 pre-miRNAs and 2640 SNPs (Additional file 1: Table S1). A total of 551 pre-miRNAs had mature miRNA in the 5′ arm, and 566 pre-miRNAs had mature miRNA in the 3′ arm (Additional file 2: Table S2).

**Figure 4:**
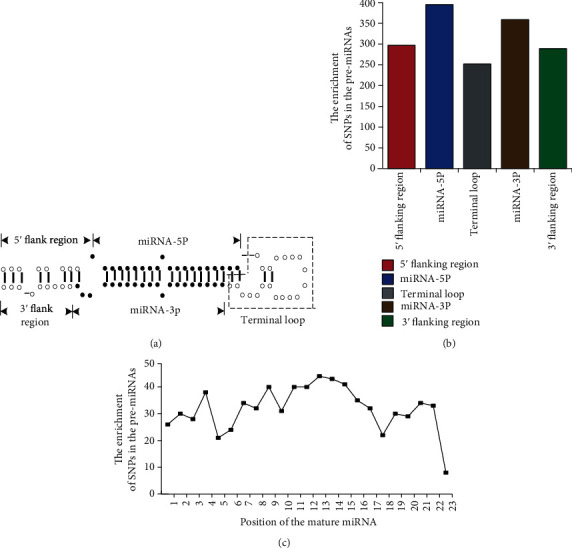
The distribution of SNPs in the pre-miRNA sequences. (a) Region definition of the pre-miRNA sequence. (b) The enrichment of SNPs in the different regions of pre-miRNAs. (c) The enrichment of SNPs in the different positions of the mature miRNA.

To compare SNP enrichment between the different regions of the pre-miRNAs, we accounted for the number of SNPs located in five regions: the 3′ flanking region, miRNA-5P, terminal loop, miRNA-3P, and 5′ flanking region of the pre-miRNA, and they correspond to section 1, section 2, section 3, section 4, and section 5, respectively. We further characterized the enrichment of SNPs located in the mature miRNAs of each pre-miRNA. The distribution of SNPs in the pre-miRNA sequences is shown in Figure 4.

As shown in Figure 4, the enrichment of SNPs in the miRNA-5P region was higher than that in the other regions. The enrichment of SNPs in the terminal loop was the lowest. In mature miRNAs, SNPs were the most enriched in the 13th nucleotide position. The lowest enrichment was in the 23rd position. Because the length of most mature miRNAs is 22 nt, the 23rd position was not considered, and the lowest enrichment of SNPs in pre-miRNAs was found in the 1st, 5th, and 17th positions. For most positions, the 13th, 14th, and 15th positions had the highest SNP enrichment.

### 3.2. Identification of Processing Sites of SNP-pre-miRNA

Each pre-miRNA and related SNPs were used to construct SNP-pre-miRNAs based on nucleotide substitution and the composition method. As shown in Figure 2, using hsa-mir-187 as an example, there are three SNP positions that may be mapped to hsa-mir-187, and we constructed 7 SNP-pre-miRNAs. We similarly constructed 10,574 SNP-pre-miRNAs using 886 pre-miRNAs and their associated 2640 SNPs. We identified the mature miRNAs of each SNP-pre-miRNA using miRFind. miRFind prediction accuracy of top five and first candidates is 55% and 33%; therefore, for improving the identification accuracy, five candidates of miRFind prediction result were considered.

Based on miRFind, mature miRNAs were divided into nor-miRNAs and isomiRs. The results of mature miRNA identification of SNP-pre-miRNAs are illustrated in Table 2.

As shown in Table 2, the distribution of iso-SNP-pre-miRNAs, iso-pre-miRNAs, and iso-SNPs was lower than that of nor-SNP-pre-miRNAs, nor-pre-miRNAs, and nor-SNPs in P5_5, and the enrichment of iso-SNP-pre-miRNAs, iso-pre-miRNAs, and iso-SNPs was higher than that of nor-SNP-pre-miRNAs, nor-pre-miRNAs, and nor-SNPs in other sites. Using P5_5 as an example, we identified 607 iso-SNP-pre-miRNAs, 143 iso-pre-miRNAs, and 427 iso-SNPs. Conversely, we identified 4235 nor-SNP-pre-miRNAs, 480 nor-pre-miRNAs, and 1250 nor-SNPs. These results indicated that the mature site of 480 pre-miRNAs was not affected by SNPs, and 1250 SNPs were not involved in the miRNA mature mechanism. In contrast, the processing sites of 143 pre-miRNAs were affected by SNPs, and 427 SNPs caused P5_5 site abnormal processing.

All the iso-pre-miRNAs, nor-pre-miRNAs, nor-SNPs, and iso-SNPs associated with the four processing sites are shown in Additional file 3: Table S3. Fifty-three pre-miRNAs and 159 SNPs did not play a role in the miRNA maturation mechanism, and 40 pre-miRNAs and 129 SNPs were involved in the miRNA mature mechanism.

**Figure 5:**
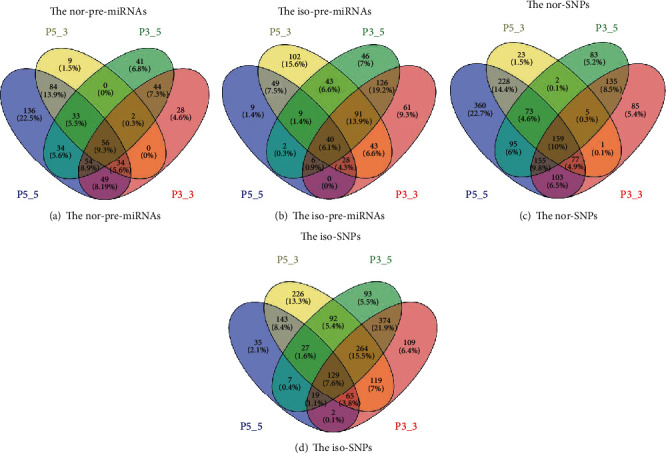
The distribution of pre-miRNAs and SNPs associated with the normal and isomiRs. (a) The pre-miRNAs that were not affected by SNPs at each site. (b) A total of 143 pre-miRNAs were affected by SNPs at each site. (c) SNPs that were not involved in the miRNA maturation mechanism at different sites. (d) SNPs that were involved in the miRNA mature mechanism on different sites.

The distribution of pre-miRNAs and SNPs associated with the nor-miRNAs and isomiRs is shown in Figure 5 and Additional file 4: Table S4.

Four processing sites of nor-pre-miRNAs, nor-SNPs, iso-pre-miRNAs, and iso-SNPs were extracted, and the results suggested that these nor-pre-miRNAs tended to remain in normal pre-miRNA processing. The nor-SNPs tended to not affect the Drosha or Dicer enzyme processing of pre-miRNAs. The iso-pre-miRNAs tended to be spliced into isomiRs, and the nor-SNPs tended to change the processing sites and produce isomiRs. Notably, all of these pre-miRNAs and SNPs were used as candidates for biological experimental studies.

### 3.3. Effects of SNPs on the Biogenesis of Mature miRNAs

**Figure 6:**
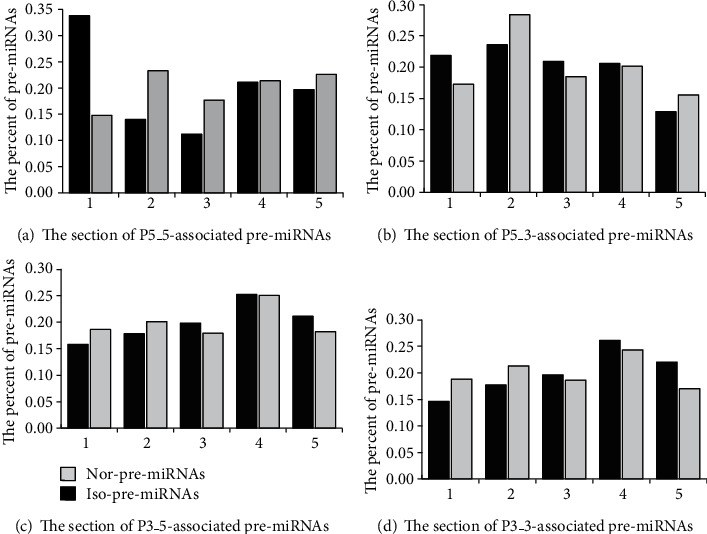
Distribution of pre-miRNAs between nor-pre-miRNA and iso-pre-miRNA based on SNP location in pre-miRNA. The SNP location in pre-miRNA includes five sections which were defined in Figure 4. In (a), pre-miRNA in which P5_5 site is changed or not by SNP effect is named P5_5-associated pre-miRNA. The pre-miRNA which site is abnormally processed is named iso-pre-miRNA. The pre-miRNA which site is normally processed is named nor-pre-miRNA.

The iso-pre-miRNAs, nor-pre-miRNA, iso-SNPs, and nor-SNPs were analyzed to determine which factors played important roles in the biogenesis of mature miRNAs. We analyzed the relationships between SNP position, SNP type, SNP-affected free energy change, and the miRNA mature mechanism. The distributions of pre-miRNAs between nor-pre-miRNA and iso-pre-miRNA based on SNP location in pre-miRNAs are described in Figure 6.

Using P5_5 as an example, the nor-pre-miRNAs had the highest and lowest enrichment when SNPs were located in section 1 and section 3, respectively. The iso-pre-miRNAs had the highest and lowest enrichment when the SNPs were located in section 4 and section 3, respectively. When SNPs were located in section 1, iso-pre-miRNAs had a higher enrichment in that region, the nor-pre-miRNAs had a lower enrichment, and their enrichments were not significantly different. When SNPs were located in section 3, iso-pre-miRNAs had a lower enrichment, nor-pre-miRNAs had a higher enrichment, and their enrichments were significantly different.

For P5_5-associated pre-miRNAs, when SNPs were located in section 1, SNP-pre-miRNAs tended to splice and produce isomiRs, and when SNPs were located in sections 2 and 3, SNP-pre-miRNAs tended to splice and produce normal miRNAs. For P5_3-associated pre-miRNAs, when SNPs were located in sections 2 and 5, SNP-pre-miRNAs tended to splice and produce normal miRNAs, and when SNPs were located in the other sections, SNP-pre-miRNAs tended to splice and produce isomiRs. For P3_5- and P3_3-associated pre-miRNAs, when the SNPs were located in sections 1 and 2, the SNP-pre-miRNAs tended to splice and produce normal miRNAs, and when SNPs were located in the other sections, SNP-pre-miRNAs tended to splice and produce isomiRs.

**Figure 7:**
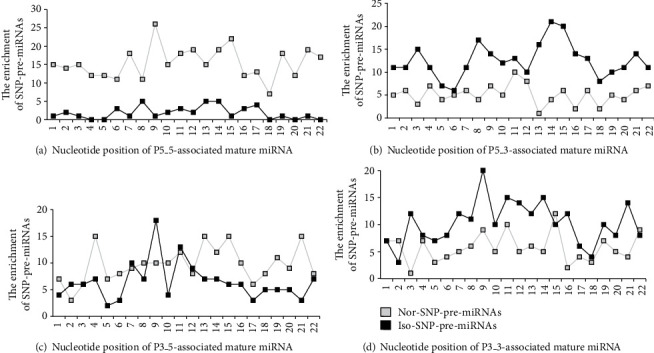
The enrichment of SNP-pre-miRNAs on SNP location in mature miRNAs. As shown in (a), mature miRNA which is associated with P5_5 site change or not is named P5_5-associated mature miRNA. SNP-pre-miRNA which site is abnormally processed is named iso-SNP-pre-miRNA. SNP-pre-miRNA which site is normally processed is named nor-SNP-pre-miRNA.

We analyzed the enrichment of SNP-pre-miRNAs on SNP location in mature miRNAs (Figure 7).

We focused on three cases: (1) the enrichment of SNPs in iso-pre-miRNAs/nor-pre-miRNAs was higher than that in nor-pre-miRNAs/iso-pre-miRNAs; (2) the enrichment of SNPs in one class of pre-miRNAs had a large degree of change in specific sites of mature miRNAs; and (3) the enrichments of SNPs in iso-pre-miRNAs and nor-pre-miRNAs had a slight change in specific sites in mature miRNAs.

For P5_5 mature miRNAs, when SNPs were located in the 7, 9, and 15 nt positions, the enrichment was higher in nor-pre-miRNAs and lower in iso-pre-miRNAs, but their enrichments were not significantly different. When the SNPs were located in the 6, 8, and 13 nt positions, iso-pre-miRNAs had a lower enrichment, the nor-pre-miRNAs had a higher enrichment, and their enrichments were significantly different. When the SNPs were located in the 17-22 nt positions, the enrichment of nor-pre-miRNAs and iso-pre-miRNAs was positively correlated. These results suggest that pre-miRNAs with SNPs in the 7, 9, and 15 nt positions tend to splice and produce normal miRNAs, and pre-miRNAs with SNPs located in the 6, 8, and 13 nt positions tend to splice and produce isomiRs. SNPs located in the 17-22 nt positions do not affect pre-miRNA processing.

For P5_3, our data suggest that pre-miRNAs with SNPs in the 1, 2, 4, 6, 11, 12, and 17 nt positions tend to splice and produce normal miRNAs and that pre-miRNAs with SNPs in the 3, 8, 13, and 14 nt positions tend to splice and produce isomiRs. In contrast, SNPs located in the 18-22 nt positions do not affect pre-miRNA processing.

For P3_5, our data suggest that pre-miRNAs with SNPs in the 4, 10, 13, 15, and 21 nt positions tend to splice and produce normal miRNAs and that pre-miRNAs with SNPs in the 2, 9, and 22 nt positions tend to splice and produce isomiRs. SNPs located in the 5, 6, and 17 nt positions do not affect pre-miRNA processing.

For P3_3, our data suggest that pre-miRNAs containing SNPs in the 2, 4, 15, and 22 nt positions tend to splice and produce normal miRNAs and that pre-miRNAs with SNPs in the 3, 4, 16, and 21 nt positions tend to splice and produce isomiRs. SNPs located in the 5, 10, 11, 18, 19, and 20 nt positions do not affect pre-miRNA processing.

In general, the enrichments of iso-pre-miRNAs and nor-pre-miRNAs were negatively correlated in most sites of mature miRNAs, which is consistent with the actual observations, i.e., as the number of SNP-pre-miRNAs to normal miRNA increases, the number of SNP-pre-miRNA to isomiRs decreases.

**Figure 8:**
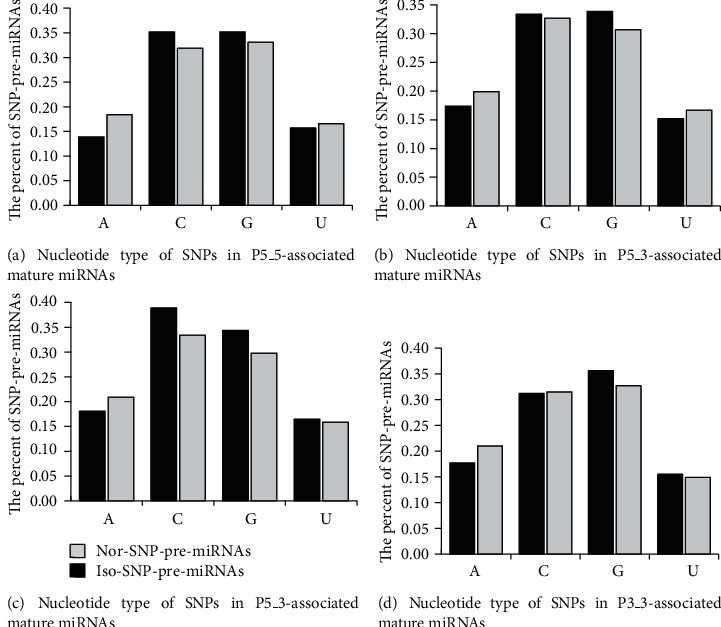
Distribution of SNP-pre-miRNAs between nor-SNP-pre-miRNA and iso-SNP-pre-miRNA based on SNP type in pre-miRNA. The SNP type in pre-miRNA includes four nucleotides.

The distributions of SNP-pre-miRNAs between nor-SNP-pre-miRNA and iso-SNP-pre-miRNA based on SNP type in pre-miRNAs are described in Figure 8.

For P5_5, when the SNP types are “C” and “G,” the percent of iso-pre-miRNAs is higher. When the SNP types are “A” and “U,” the percent of nor-pre-miRNAs is higher. When the SNP type is “A,” the difference in the percentage of iso-pre-miRNAs and nor-pre-miRNAs is the largest. These observations suggest that when SNP types are “C” and “G,” the pre-miRNAs tend to splice and produce isomiRs, and when the SNP types are “A” and “U,” the pre-miRNAs tend to splice and produce normal miRNAs.

For P5_3, the results suggest that when SNP types are “C” and “G,” the pre-miRNAs tend to splice and produce isomiRs, and when SNP types are “A” and “U,” the pre-miRNAs tend to splice and produce normal miRNAs. For P3_5, the results suggest that when SNP types are “C,” “G,” and “U,” the pre-miRNAs tend to splice and produce isomiRs, and when the SNP type is “A,” the pre-miRNAs tend to splice and produce normal miRNAs. For P3_3, the data suggest that when SNP types are “G” and “U,” the pre-miRNAs tend to splice and produce isomiRs, and when the SNP type is “A,” the pre-miRNAs tend to splice and produce normal miRNAs.

**Figure 9:**
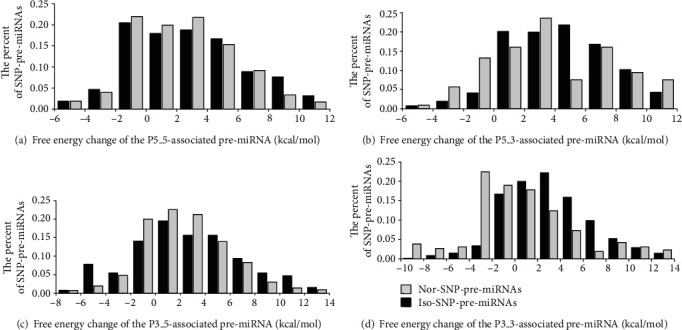
Distribution of SNP-pre-miRNAs between nor-SNP-pre-miRNA and iso-SNP-pre-miRNA based on SNP-caused free energy change of pre-miRNA.

The distributions of SNP-pre-miRNAs between nor-SNP-pre-miRNA and iso-SNP-pre-miRNA based on SNP-induced free energy changes in pre-miRNA are described in Figure 9.

The change in free energy primarily focuses on 0-8 kcal/mol. As free energy increases greater than 4 kcal/mol, SNP-pre-miRNAs tend to shear and produce isomiRs. When the free energy change decreased >2 kcal/mol, the percentages of iso-SNP-pre-miRNAs for P5_5 and P3_5 mature miRNA loci were higher than those of nor-SNP-pre-miRNAs. For the P5_3 and P3_3 mature miRNA loci, the percentages of nor-SNP-pre-miRNAs were higher than those of iso-SNP-pre-miRNAs. These results suggest that a decrease in free energy tends to alter the processing sites of the 5′ end of mature miRNAs, and the 3′ ends of mature miRNAs are less affected.

On the basis of the free energy change, the distributions of iso-SNP-pre-miRNAs and nor-SNP-pre-miRNAs associated with the 5′ end (P5_5 and P5_3) and 3′ end (P5_3 and P3_3) sites of mature miRNAs had similar characteristics, which indicates that the effects of the free energy change on the 5′ and 3′ ends of mature miRNA biogenesis are largely consistent.

For P3_3, SNP-pre-miRNAs tended to maintain normal mature miRNA biogenesis when the free energy increased and tended to splice and produce normal mature miRNAs when the free energy decreased.

### 3.4. Identification and Verification of isomiRs Based on a GBR Population from the 1000 Genomes Database

We used our method to identify the isomiRs and iso-SNPs of 18 GBR individuals of European origin. We use HG00097 as an example. Because the sample VCF data were provided by karyotype, we extracted the variation information of HG00097 from 23 chromosome files and integrated all of the variation information. All SNPs were mapped to pre-miRNAs to construct the SNP-pre-miRNAs for HG00097. We identified four sites of mature miRNA sequences. As a result, we predicted 695 isomiRs of 92 pre-miRNAs with 94 SNPs in a different guide strand with the incorporation of variations in its sequence. The pre-miRNAs, iso-SNPs, and isomiRs of HG00097 are summarized in Additional file 5: Table S5. The results suggest that some SNPs within pre-miRNAs affect miRNA biogenesis and function.

We identified the isomiRs and iso-SNPs of 18 GBR individuals of European origin, and our method predicted 209 iso-pre-miRNAs. And 71 iso-pre-miRNAs of the 18 GBR samples are shown in Additional file 6: Table S6. 2667 isomiRs of 209 pre-miRNAs and isomiRs and iso-SNPs of the 18 GBR individuals are shown in Table 3 and detailed in Additional file 7: Table S7.

We validated these results using the miRNA sequencing data. The 158 verified isomiRs of the 18 GBR samples are shown in Table 4, and the details are shown in Additional file 8: Table S8.

## 4. Discussion

As an important molecular mechanism by which SNPs significantly contribute to miRNA generation mechanisms and functions, experiments showed that the molecular structure, thermodynamic stability, and functional strand selection were affected by SNPs located in pre-miRNAs [7, 10, 27]. As a result, SNPs influenced the selection of Drosha enzyme processing, Dicer enzyme processing, and functional strands in the processing of pre-miRNAs and altered the expression levels of miRNAs [12, 28–31], which are closely related to various phenotypes and diseases. However, iso-miRNAs are hard to detect because the expression levels of iso-miRNAs are low, and the mechanism of SNP-affected mature miRNA biogenesis was not clear. Therefore, we systematically studied the role of SNPs in the biogenesis of mature miRNAs.

We constructed an SNP-associated pre-miRNA database based on the latest data from miRBase and the 1000 Genomes Project and integrated these data into the miRNASNP database to obtain our database, miRSNPBase.

We analyzed the relationships between SNP type, SNP location, and SNP-affected free energy change and the biogenesis of mature miRNAs. The results showed that these three factors played important roles in the mature miRNA generation mechanism. We identified isomiRs and iso-SNPs in 18 GBR individuals. These isomiRs were verified using the miRNA sequencing data of 18 GBR samples. As a result, we obtained epigenetic-associated isomiRs and SNPs; we compared the results with the miRNA sequencing data to identify and verify the presence of isomiRs.

## 5. Conclusions

We constructed the SNP-pre-miRNA database, named miRSNPBase, and included 886 pre-miRNAs and 2640 SNPs. By identifying the processing sites of SNP-pre-miRNA, we found that 53 pre-miRNAs and 159 SNPs did not play a role in the miRNA maturation mechanism, and 40 pre-miRNAs and 129 SNPs were involved in the miRNA mature mechanism. In addition, effects of SNPs on the miRNA mature mechanism differ depending on the location of SNPs in the pre-miRNA sequence. Moreover, the SNP type affected mature mechanism of miRNA; when the SNP types are “C” and “G,” SNP-pre-miRNA tend to process and generate isomiRs. When the SNP types are “A” and “U,” SNP-pre-miRNA tend to process and generate canonical miRNA. The decrease in free energy caused by SNPs tends to alter the processing sites of mature miRNAs. Finally, 695 isomiRs and 94 SNPs were identified based on 18 GBR individuals from the 1000 Genomes database; in particular, 158 isomiRs were verified in miRNA sequencing data.

Overall, our study suggests that SNPs affect biological characteristics and lead to changes in the Dicer sites of mature miRNAs undergoing the maturation process, which leads to the generation of isomiRs. Some isomiRs were verified based on the miRNA sequencing data of 18 GBR individuals. The identification of isomiRs in miRNA sequencing data also indicated that our method was effective. In conclusion, our results suggest that SNPs play important roles in the biogenesis of mature miRNAs.

## Figures and Tables

**Table 1:** Example of the SNP information. The format definition refers to the VCF specification; the parameters were listed as follows. Chrom: chromosome; POS: position; ID: identifier; REF: reference base(s); ALT: alternate base(s); QUAL: quality; FILTER: filter status; AN: total number of alleles in called genotypes; AC: allele count in genotypes; AF: allele frequency for each ALT allele in the same order; AN: total number of alleles in called genotypes; NS: number of samples with data; DP: read depth at this position for this sample (integer); AA: ancestral allele; VT: variation type.

Table	Parameter	Table	Parameter
Chrom	1	AC	1
POS	55,285	AF	0.000199681
ID	rs532608387	AN	5008
REF	T	NS	2504
ALT	C	DP	18,296
QUAL	100	AA	t
FILTER	PASS	VT	SNP

**Table 2:** The distribution of pre-miRNAs and SNPs associated with four processing sites.

Site	nor-SNP-pre-miRNAs	nor-pre-miRNAs	nor-SNPs	iso-SNP-pre-miRNA	iso-pre-miRNA	iso-SNPs
P5_5	4235	480	1250	607	143	427
P5_3	1825	218	569	3385	405	1065
P3_5	2089	264	707	3435	363	1005
P3_3	2026	267	721	3779	395	1081

**Table 3:** The isomiRs and iso-SNP of 18 GBR populations.

Sample	isomiR	SNP	Sample	isomiR	SNP
HG00096	697	109	HG00108	719	69
HG00097	601	105	HG00109	718	112
HG00099	426	94	HG00110	663	128
HG00100	710	119	HG00111	711	123
HG00101	646	104	HG00112	463	84
HG00102	657	113	HG00114	665	103
HG00105	683	104	HG00115	682	117
HG00106	732	128	HG00116	714	110
HG00107	315	71	HG00117	639	126

**Table 4:** The verified isomiRs of 18 GBR.

Sample	Verified isomiR	Sample	Verified isomiR
HG00096	96	HG00108	37
HG00097	19	HG00109	19
HG00099	19	HG00110	36
HG00100	67	HG00111	27
HG00101	36	HG00112	27
HG00102	32	HG00114	16
HG00105	23	HG00115	45
HG00106	23	HG00116	33
HG00107	7	HG00117	55

## Data Availability

The miRNA genome position information used in this paper can be downloaded from ftp://mirbase.org/pub/mirbase/. The information of SNPs described in this manuscript was obtained from the 1000 Genomes Project (ftp://ftp-trace.ncbi.nih.gov/1000genomes/ftp/release/20130502/). The miRNASNP dataset can be downloaded from http://bioinfo.life.hust.edu.cn/miRNASNP2/download.php. The VCF files used in this paper can be downloaded from ftp://ftp.1000genomes.ebi.ac.uk/vol1/ftp/release/20130502/. And the miRNA sequencing data described in this manuscript can be obtained from https://www.ebi.ac.uk/arrayexpress/experiments/E-GEUV-2/samples/. The Homo sapiens GRCh37 reference sequence used in this paper can be downloaded from ftp://ftp.1000genomes.ebi.ac.uk/vol1/ftp/technical/reference/human_g1k_v37.fasta.gz.

## Abbreviations

SNP: Single nucleotide polymorphism
miRNA: MicroRNA
SNP-pre-miRNA: SNP-associated pre-miRNA
isomiR: Isoform miRNA
GBR: British in England and Scotland
iso-SNPs: isomiR-associated SNPs
VCF: Variant call format
iso-pre-miRNAs: isomiR-associated pre-miRNA
nor-miRNAs: Normal miRNA
nor-SNPs: nor-miRNA-associated SNPs.

## Supplementary Materials

Supplementary Materials Additional file 1 Table S1: the database miRSNPBase (xls). Additional file 2 Table S2: the list of pre-miRNAs in miRSNPBase which is classified based on mature miRNA in the 5′ arm or 3′ arm (xls). Additional file 3 Table S3: all the iso-pre-miRNAs, nor-pre-miRNAs, nor-SNPs, and iso-SNPs associated with four splicing sites (xls). Additional file 4 Table S4: the pre-miRNAs and SNPs associated with the normal and isomiRs (xls). Additional file 5 Table S5: the pre-miRNAs, iso-SNPs, and isomiRs of HG00097 (xls). Additional file 6 Table S6: the isomiRs and iso-SNP of 18 GBR populations (xls). Additional file 7 Table S7: the verified isomiRs of 18 GBR (xls). Additional file 8 Table S8: the iso-pre-miRNA candidates and the verified iso-pre-miRNAs of 18 GBR samples (xls).
