# Supplementary material for: Role of SNPs in the Biogenesis of Mature miRNAs
Source: Biomed Res Int. 2021 Jun 17;2021:2403418. doi: 10.1155/2021/2403418 (PMC8233088; doi:10.1155/2021/2403418)
Supplement: Supplementary Materials — Additional file 1 Table S1: the database miRSNPBase (xls). Additional file 2 Table S2: the list of pre-miRNAs in miRSNPBase which is classified based on mature miRNA in the 5′ arm or 3′ arm (xls). Additional file 3 Table S3: all the iso-pre-miRNAs, nor-pre-miRNAs, nor-SNPs, and iso-SNPs associated with four splicing sites (xls). Additional file 4 Table S4: the pre-miRNAs and SNPs associated with the normal and isomiRs (xls). Additional file 5 Table S5: the pre-miRNAs, iso-SNPs, and isomiRs of HG00097 (xls). Additional file 6 Table S6: the isomiRs and iso-SNP of 18 GBR populations (xls). Additional file 7 Table S7: the verified isomiRs of 18 GBR (xls). Additional file 8 Table S8: the iso-pre-miRNA candidates and the verified iso-pre-miRNAs of 18 GBR samples (xls). [file 2403418.f1.zip › 2403418.f1/Supp Tab S1.pdf]

Name	Sequence	Chromosome	SNP position	SNP ID	Ref	Alt	Alter frequency	Strand	Position in pre-miRNA
hsa-let-7a-2	AGGUUGAGGUAGUAGGUUGUAUAGUUUAGAAUUACAUCAAGGGAGAUAACUGUACAGCCUCCUAGCUUUCCU	chr11	122017258	.	A	G	0.000199681	-	44
hsa-let-7b	CGGGGUGAGGUAGUAGGUUGUGUGGUUUCAGGGCAGUGAUGUUGCCCCUCGGAAGAUAACUAUACAACCUACUGCCUUCCCUG	chr22	46509569	.	G	A	0.000199681	+	4
hsa-let-7b	CGGGGUGAGGUAGUAGGUUGUGUGGUUUCAGGGCAGUGAUGUUGCCCCUCGGAAGAUAACUAUACAACCUACUGCCUUCCCUG	chr22	46509616	.	G	A	0.000199681	+	51
hsa-let-7e	CCCGGGCUGAGGUAGGAGGUUGUAUAGUUGAGGAGGACACCCAAGGAGAUCACUAUACGGCCUCCUAGCUUUCCCCAGG	chr19	52196045	.	C	G	0.000399361	+	7
hsa-let-7e	CCCGGGCUGAGGUAGGAGGUUGUAUAGUUGAGGAGGACACCCAAGGAGAUCACUAUACGGCCUCCUAGCUUUCCCCAGG	chr19	52196076	rs376594280	C	U	0.000599042	+	38
hsa-let-7i	CUGGCUGAGGUAGUAGUUUGUGCUGUUGGUCGGGUUGUGACAUUGCCCGCUGUGGAGAUAACUGCGCAAGCUACUGCCUUGCUA	chr12	62997470	.	C	G	0.000199681	+	5
hsa-mir-101-2	ACUGUCCUUUUUCGGUUAUCAUGGUACCGAUGCUGUAUAUCUGAAAGGUACAGUACUGUGAUAACUGAAGAAUGGUGGU	chr9	4850310	.	G	A	0.000199681	+	14
hsa-mir-101-2	ACUGUCCUUUUUCGGUUAUCAUGGUACCGAUGCUGUAUAUCUGAAAGGUACAGUACUGUGAUAACUGAAGAAUGGUGGU	chr9	4850331	.	G	A	0.000199681	+	35
hsa-mir-105-1	UGUGCAUCGUGGUCAAAUGCUCAGACUCCUGUGGUGGCUGCUCAUGCACCACGGAUGUUUGAGCAUGUGCUACGGUGUCUA	chrX	151560719	rs372715021	G	A	0.000264901	-	53
hsa-mir-105-1	UGUGCAUCGUGGUCAAAUGCUCAGACUCCUGUGGUGGCUGCUCAUGCACCACGGAUGUUUGAGCAUGUGCUACGGUGUCUA	chrX	151560699	.	C	U	0.000264901	-	73
hsa-mir-105-2	UGUGCAUCGUGGUCAAAUGCUCAGACUCCUGUGGUGGCUGCUUAUGCACCACGGAUGUUUGAGCAUGUGCUAUGGUGUCUA	chrX	151562938	rs72631816	U	A	null	null	27
hsa-mir-106b									

CCUGCCGGGGCUAAAGUGCUGACAGUGCAGAUAGUGGUCCUCUCCGUGCUACCGCACUGUGGGUACUU
GCUGCUCCAGCAGG chr7 99691653 rs370912855 C U
0.000199681 - 45
hsa-mir-106b
CCUGCCGGGGCUAAAGUGCUGACAGUGCAGAUAGUGGUCCUCUCCGUGCUACCGCACUGUGGGUACUU
GCUGCUCCAGCAGG chr7 99691652 rs72631827 G U 0.00599042
- 46
hsa-mir-107
CUCUCUGCUUUCAGCUUCUUUACAGUGUUGCCUUGUGGCAUGGAGUUCAAGCAGCAUUGUACAGGGCU
AUCAAAGCACAGAchr10 91352580 rs377494950 C U 0.000399361 -
5
hsa-mir-10a
GAUCUGUCUGUCUUCUGUAUAUACCCUGUAGAUCCGAAUUUGUGUAAGGAAUUUUGUGGUCACAAAUU
CGUAUCUAGGGGAAUAUGUAGUUGACAUAAACACUCCGCUCU chr17 46657289 rs72631828
A G null null 21
hsa-mir-10a
GAUCUGUCUGUCUUCUGUAUAUACCCUGUAGAUCCGAAUUUGUGUAAGGAAUUUUGUGGUCACAAAUU
CGUAUCUAGGGGAAUAUGUAGUUGACAUAAACACUCCGCUCU chr17 46657254 . G
A 0.000199681 - 56
hsa-mir-10b
CCAGAGGUUGUAACGUUGUCUAUAUAUACCCUGUAGAACCGAAUUUGUGUGGUAUCCGUAUAGUCACA
GAUUCGAUUCUAGGGGAAUAUAUGGUCGAUGCAAAAACUUCA chr2 177015045 . G
C 0.000199681 + 15
hsa-mir-1-1
UGGGAAACAUACUUCUUUAUAUGCCCAUAUGGACCUGCUAAGCUAUGGAAUGUAAAGAAGUAUGUAUC
UCA chr20 61151515 rs6122014 G A 0.00738818 + 3

hsa-mir-1-1
UGGGAAACAUACUUCUUUAUAUGCCCAUAUGGACCUGCUAAGCUAUGGAAUGUAAAGAAGUAUGUAUC
UCA chr20 61151539 . A U 0.000199681 + 27

hsa-mir-1-1
UGGGAAACAUACUUCUUUAUAUGCCCAUAUGGACCUGCUAAGCUAUGGAAUGUAAAGAAGUAUGUAUC
UCA chr20 61151573 . U C 0.000199681 + 61

hsa-mir-1178
GCGUUGGCUGGCAGAGGAAGGGAAGGGUCCAGGGUCAGCUGAGCAUGCCCUCAGGUUGCUCACUGUUC
UUCCCUAGAAUGUCAGGUGAUGU chr12 120151527 rs141225976 G A
0.000199681 - 3
hsa-mir-1178
GCGUUGGCUGGCAGAGGAAGGGAAGGGUCCAGGGUCAGCUGAGCAUGCCCUCAGGUUGCUCACUGUUC
UUCCCUAGAAUGUCAGGUGAUGU chr12 120151501 rs74614893 C U
0.0121805 - 29
hsa-mir-1178
GCGUUGGCUGGCAGAGGAAGGGAAGGGUCCAGGGUCAGCUGAGCAUGCCCUCAGGUUGCUCACUGUUC
UUCCCUAGAAUGUCAGGUGAUGU chr12 120151493 rs7311975 A G
null null 37

hsa-mir-1179
GGCUGGAAAGGAAGAAGCAUUCUUUCAUUGGUUGGUGUGUAUUGCCUUGUCAACCAAUAAGAGGAUGC
CAUUUAUCCUUUUCUGACUAGCU chr15 89151351 . G U
0.000599042 + 14
hsa-mir-1179
GGCUGGAAAGGAAGAAGCAUUCUUUCAUUGGUUGGUGUGUAUUGCCUUGUCAACCAAUAAGAGGAUGC
CAUUUAUCCUUUUCUGACUAGCU chr15 89151354 . G A
0.000199681 + 17
hsa-mir-1179
GGCUGGAAAGGAAGAAGCAUUCUUUCAUUGGUUGGUGUGUAUUGCCUUGUCAACCAAUAAGAGGAUGC
CAUUUAUCCUUUUCUGACUAGCU chr15 89151411 . A G
0.000399361 + 74
hsa-mir-1180
GCUGCUGGACCCACCCGGCCGGGAAUAGUGCUCCUGGUUGUUUCCGGCUCGCGUGGGUGUGUCGGCGG
C chr17 19247825 . C U 0.000599042 - 63

hsa-mir-1181
UCCACUGCUGCCGCCGUCGCCGCCACCCGAGCCGGAGCGGGCUGGGCCGCCAAGGCAAGAUGGUGGAC
UACAGCGUGUGGGchr19 10514202 rs371549320 G U 0.000599042 -
13
hsa-mir-1181
UCCACUGCUGCCGCCGUCGCCGCCACCCGAGCCGGAGCGGGCUGGGCCGCCAAGGCAAGAUGGUGGAC
UACAGCGUGUGGGchr19 10514200 . C G 0.000399361 - 15

hsa-mir-1181
UCCACUGCUGCCGCCGUCGCCGCCACCCGAGCCGGAGCGGGCUGGGCCGCCAAGGCAAGAUGGUGGAC
UACAGCGUGUGGGchr19 10514194 rs373952957 C U 0.000399361 -
21
hsa-mir-1181
UCCACUGCUGCCGCCGUCGCCGCCACCCGAGCCGGAGCGGGCUGGGCCGCCAAGGCAAGAUGGUGGAC
UACAGCGUGUGGGchr19 10514190 . A U 0.000199681 - 25

hsa-mir-1181
UCCACUGCUGCCGCCGUCGCCGCCACCCGAGCCGGAGCGGGCUGGGCCGCCAAGGCAAGAUGGUGGAC
UACAGCGUGUGGGchr19 10514159 rs2569788 C G null null 56

hsa-mir-1181
UCCACUGCUGCCGCCGUCGCCGCCACCCGAGCCGGAGCGGGCUGGGCCGCCAAGGCAAGAUGGUGGAC
UACAGCGUGUGGGchr19 10514157 rs200324204 A G 0.000199681 -
58
hsa-mir-1182
GGGACUUGUCACUGCCUGUCUCCUCCCUCUCCAGCAGCGACUGGAUUCUGGAGUCCAUCUAGAGGGUC
UUGGGAGGGAUGUGACUGUUGGGAAGCCC chr1 231155670 . G C
0.000199681 - 1
hsa-mir-1182
GGGACUUGUCACUGCCUGUCUCCUCCCUCUCCAGCAGCGACUGGAUUCUGGAGUCCAUCUAGAGGGUC
UUGGGAGGGAUGUGACUGUUGGGAAGCCC chr1 231155644 . C G

0.000199681 - 27
hsa-mir-1182
GGGACUUGUCACUGCCUGUCUCCUCCCUCUCCAGCAGCGACUGGAUUCUGGAGUCCAUCUAGAGGGUC UUGGGAGGGAUGUGACUGUUGGGAAGCCC chr1 231155581 rs375238321 G A 0.000399361 - 90
hsa-mir-1183
AUUAUUCAAAUGCUCGGAGACACAGAACAUUAGAGAAGACAGGAGUUCACUGUAGGUGAUGGUGAGAG UGGGCAUGGAGCAGGAGUGCC chr7 21510699 . A G 0.000199681 + 24
hsa-mir-1183
AUUAUUCAAAUGCUCGGAGACACAGAACAUUAGAGAAGACAGGAGUUCACUGUAGGUGAUGGUGAGAG UGGGCAUGGAGCAGGAGUGCC chr7 21510716 . A G 0.000199681 + 41
hsa-mir-1185-1
UUUGGUACUUGAAGAGAGGAUACCCUUUGUAUGUUCACUUGAUUAAUGGCGAAUAUACAGGGGGAGAC UCUUAUUUGCGUAUCAAA chr14 101509363 . C U 0.000199681 + 50
hsa-mir-1185-2
UUUGGUACUUAAAGAGAGGAUACCCUUUGUAUGUUCACUUGAUUAAUGGCGAAUAUACAGGGGGAGAC UCUCAUUUGCGUAUCAAA chr14 101510579 . A C 0.000199681 + 45
hsa-mir-1185-2
UUUGGUACUUAAAGAGAGGAUACCCUUUGUAUGUUCACUUGAUUAAUGGCGAAUAUACAGGGGGAGAC UCUCAUUUGCGUAUCAAA chr14 101510612 . C U 0.000399361 + 78
hsa-mir-1185-2
UUUGGUACUUAAAGAGAGGAUACCCUUUGUAUGUUCACUUGAUUAAUGGCGAAUAUACAGGGGGAGAC UCUCAUUUGCGUAUCAAA chr14 101510613 rs11844707 G A 0.0573083 + 79
hsa-mir-1193
GUAGCUGAGGGGAUGGUAGACCGGUGACGUGCACUUCAUUUACGAUGUAGGUCACCCGUUUGACUAUC CACCAGCGCC chr14 101496416 . C U 0.000199681 + 28

hsa-mir-1197
ACUUCCUGGUAUUUGAAGAUGCGGUUGACCAUGGUGUGUACGCUUUAUUUGUGACGUAGGACACAUGG UCUACUUCUUCUCAAUAUCA chr14 101491923 rs141611518 G U 0.000199681 + 23
hsa-mir-1197
ACUUCCUGGUAUUUGAAGAUGCGGUUGACCAUGGUGUGUACGCUUUAUUUGUGACGUAGGACACAUGG UCUACUUCUUCUCAAUAUCA chr14 101491941 rs373241810 C U 0.000199681 + 41
hsa-mir-1197
ACUUCCUGGUAUUUGAAGAUGCGGUUGACCAUGGUGUGUACGCUUUAUUUGUGACGUAGGACACAUGG UCUACUUCUUCUCAAUAUCA chr14 101491965 rs185946570 A G 0.000199681 + 65
hsa-mir-1197
ACUUCCUGGUAUUUGAAGAUGCGGUUGACCAUGGUGUGUACGCUUUAUUUGUGACGUAGGACACAUGG

UCUACUUCUUCUCAAUAUCA chr14 101491974 . U G
0.000199681 + 74
hsa-mir-1197
ACUUCCUGGUAUUUGAAGAUGCGGUUGACCAUGGUGUGUACGCUUUAUUUGUGACGUAGGACACAUGG
UCUACUUCUUCUCAAUAUCA chr14 101491981 . C U
0.000199681 + 81
hsa-mir-1199
AGCCUGCGCCGGAGCCGGGGCCUGAGCCCGGGCCGCGCAGGCCGUGAACUCGUCGAGCUGCGCGUGCG
GCCGGUGCUCAACCUGCCGGGUCCUGGCCCCGCGCUCCCGCGCGCCCUGGA chr19 14184227
rs8104055 G C 0.063099 + 55

hsa-mir-1200
UGCUACUUCUCCUGAGCCAUUCUGAGCCUCAAUCACUUGCCAGAGAGAUUGGUUCAGGAAUUUGUCAG
GGAUAGCC chr7 36959015 rs77814495 U C null null 23

hsa-mir-1200
UGCUACUUCUCCUGAGCCAUUCUGAGCCUCAAUCACUUGCCAGAGAGAUUGGUUCAGGAAUUUGUCAG
GGAUAGCC chr7 36959006 . A G 0.000599042 - 32

hsa-mir-1200
UGCUACUUCUCCUGAGCCAUUCUGAGCCUCAAUCACUUGCCAGAGAGAUUGGUUCAGGAAUUUGUCAG
GGAUAGCC chr7 36958995 rs180826747 G A 0.000199681 -
43
hsa-mir-1200
UGCUACUUCUCCUGAGCCAUUCUGAGCCUCAAUCACUUGCCAGAGAGAUUGGUUCAGGAAUUUGUCAG
GGAUAGCC chr7 36958963 . C G 0.000199681 - 75

hsa-mir-1202
CCUGCUGCAGAGGUGCCAGCUGCAGUGGGGGAGGCACUGCCAGGGCUGCCCACUCUGCUUAGCCAGCA
GGUGCCAAGAACAGG chr6 156267955 rs112508646 G A null
null 25
hsa-mir-1202
CCUGCUGCAGAGGUGCCAGCUGCAGUGGGGGAGGCACUGCCAGGGCUGCCCACUCUGCUUAGCCAGCA
GGUGCCAAGAACAGG chr6 156267978 . G U 0.000199681 +
48
hsa-mir-1203
UCCUCCCCGGAGCCAGGAUGCAGCUCAAGCCACAGCAGGGUGUUUAGCGCUCUUCAGUGGCUCCAGAU
UGUGGCGCUGGUGCAGG chr17 46233866 . C U 0.000798722 -
8
hsa-mir-1203
UCCUCCCCGGAGCCAGGAUGCAGCUCAAGCCACAGCAGGGUGUUUAGCGCUCUUCAGUGGCUCCAGAU
UGUGGCGCUGGUGCAGG chr17 46233848 rs77574787 C A null
null 26
hsa-mir-1203
UCCUCCCCGGAGCCAGGAUGCAGCUCAAGCCACAGCAGGGUGUUUAGCGCUCUUCAGUGGCUCCAGAU
UGUGGCGCUGGUGCAGG chr17 46233806 rs117091718 U C 0.00379393
- 68

hsa-mir-1204
ACCUCGUGGCCUGGUCUCCAUUAUUUGAGAUGAGUUACAUCUUGGAGGUGAGGACGUGCCUCGUGGU
chr8 128808252 . G U 0.00119808 + 45

hsa-mir-1204
ACCUCGUGGCCUGGUCUCCAUUAUUUGAGAUGAGUUACAUCUUGGAGGUGAGGACGUGCCUCGUGGU
chr8 128808253 . A U 0.00119808 + 46

hsa-mir-1205
GAAGGCCUCUGCAGGGUUUGCUUUGAGGUACUUCCUUCCUGUCAACCCUGUUCUGGAGUCUGU
chr8 128972923 . A G 0.000199681 + 45

hsa-mir-1205
GAAGGCCUCUGCAGGGUUUGCUUUGAGGUACUUCCUUCCUGUCAACCCUGUUCUGGAGUCUGU
chr8 128972925 . C G 0.000399361 + 47

hsa-mir-1205
GAAGGCCUCUGCAGGGUUUGCUUUGAGGUACUUCCUUCCUGUCAACCCUGUUCUGGAGUCUGU
chr8 128972936 . G A 0.000199681 + 58

hsa-mir-1206 CAGUGUUCAUGUAGAUGUUUAAGCUCUUGCAGUAGGUUUUUGCAAGCUAGUGAACGCUG
chr8 129021179 rs2114358 G A 0.671526 + 36

hsa-mir-1207
GCAGGGCUGGCAGGGAGGCUGGGAGGGGCUGGCUGGGUCUGGUAGUGGGCAUCAGCUGGCCCUCAUUU
CUUAAGACAGCACUUCUGU chr8 129061408 . C U 0.000399361 +
11
hsa-mir-1207
GCAGGGCUGGCAGGGAGGCUGGGAGGGGCUGGCUGGGUCUGGUAGUGGGCAUCAGCUGGCCCUCAUUU
CUUAAGACAGCACUUCUGU chr8 129061422 rs191866221 G A 0.00159744
+ 25
hsa-mir-1207
GCAGGGCUGGCAGGGAGGCUGGGAGGGGCUGGCUGGGUCUGGUAGUGGGCAUCAGCUGGCCCUCAUUU
CUUAAGACAGCACUUCUGU chr8 129061441 rs368654133 A G
0.000199681 + 44
hsa-mir-1207
GCAGGGCUGGCAGGGAGGCUGGGAGGGGCUGGCUGGGUCUGGUAGUGGGCAUCAGCUGGCCCUCAUUU
CUUAAGACAGCACUUCUGU chr8 129061462 rs148236411 A G
0.000199681 + 65
hsa-mir-1208
CACCGGCAGAAUCACUGUUCAGACAGGCGGAGACGGGUCUUUCUCGCCCUCUGAUGAGUCACCACUGU
GGUGG chr8 129162366 rs56863230 G C 0.0129792 + 5

hsa-mir-1208
CACCGGCAGAAUCACUGUUCAGACAGGCGGAGACGGGUCUUUCUCGCCCUCUGAUGAGUCACCACUGU
GGUGG chr8 129162390 . G A 0.000599042 + 29

hsa-mir-1208
CACCGGCAGAAUCACUGUUCAGACAGGCGGAGACGGGUCUUUCUCGCCCUCUGAUGAGUCACCACUGU
GGUGG chr8 129162409 . C U 0.000199681 + 48

hsa-mir-1208
CACCGGCAGAAUCACUGUUCAGACAGGCGGAGACGGGUCUUUCUCGCCCUCUGAUGAGUCACCACUGU
GGUGG chr8 129162428 . G C 0.000199681 + 67

hsa-mir-1208
CACCGGCAGAAUCACUGUUCAGACAGGCGGAGACGGGUCUUUCUCGCCCUCUGAUGAGUCACCACUGU
GGUGG chr8 129162433 rs2648841 G U null null 72

hsa-mir-122
CCUUAGCAGAGCUGUGGAGUGUGACAAUGGUGUUUGUGUCUAAACUAUCAAACGCCAUUAUCACACUA
AAUAGCUACUGCUAGGC chr18 56118358 rs41292412 C U 0.00299521
+ 53
hsa-mir-122
CCUUAGCAGAGCUGUGGAGUGUGACAAUGGUGUUUGUGUCUAAACUAUCAAACGCCAUUAUCACACUA
AAUAGCUACUGCUAGGC chr18 56118359 . G A 0.000199681 +
54
hsa-mir-1224
GUGAGGACUCGGGAGGUGGAGGGUGGUGCCGCCGGGGCCGGGCGCUGUUUCAGCUCGCUUCUCCCCCC
ACCUCCUCUCUCCUCAG chr3 183959219 rs148241223 U A 0.00159744
+ 27
hsa-mir-1224
GUGAGGACUCGGGAGGUGGAGGGUGGUGCCGCCGGGGCCGGGCGCUGUUUCAGCUCGCUUCUCCCCCC
ACCUCCUCUCUCCUCAG chr3 183959222 rs371662452 C G
0.000199681 + 30
hsa-mir-1224
GUGAGGACUCGGGAGGUGGAGGGUGGUGCCGCCGGGGCCGGGCGCUGUUUCAGCUCGCUUCUCCCCCC
ACCUCCUCUCUCCUCAG chr3 183959229 . G A 0.000199681 +
37
hsa-mir-1224
GUGAGGACUCGGGAGGUGGAGGGUGGUGCCGCCGGGGCCGGGCGCUGUUUCAGCUCGCUUCUCCCCCC
ACCUCCUCUCUCCUCAG chr3 183959232 rs374762753 G C 0.00119808
+ 40
hsa-mir-1225
GUGGGUACGGCCCAGUGGGGGGGAGAGGGACACGCCCUGGGCUCUGCCCAGGGUGCAGCCGGACUGAC
UGAGCCCCUGUGCCGCCCCCAG chr16 2140270 rs201173724 U C
0.000199681 - 16
hsa-mir-1225
GUGGGUACGGCCCAGUGGGGGGGAGAGGGACACGCCCUGGGCUCUGCCCAGGGUGCAGCCGGACUGAC
UGAGCCCCUGUGCCGCCCCCAG chr16 2140269 rs371749301 G U
0.000199681 - 17
hsa-mir-1225
GUGGGUACGGCCCAGUGGGGGGGAGAGGGACACGCCCUGGGCUCUGCCCAGGGUGCAGCCGGACUGAC
UGAGCCCCUGUGCCGCCCCCAG chr16 2140268 . G U

0.000199681 - 18
hsa-mir-1225
GUGGGUACGGCCCAGUGGGGGGGAGAGGGACACGCCCUGGGCUCUGCCCAGGGUGCAGCCGGACUGAC
UGAGCCCCUGUGCCGCCCCCAG chr16 2140267 rs201970421 G A
0.000199681 - 19
hsa-mir-1225
GUGGGUACGGCCCAGUGGGGGGGAGAGGGACACGCCCUGGGCUCUGCCCAGGGUGCAGCCGGACUGAC
UGAGCCCCUGUGCCGCCCCCAG chr16 2140240 rs199999016 G U
0.000199681 - 46
hsa-mir-1225
GUGGGUACGGCCCAGUGGGGGGGAGAGGGACACGCCCUGGGCUCUGCCCAGGGUGCAGCCGGACUGAC
UGAGCCCCUGUGCCGCCCCCAG chr16 2140204 . C U
0.000199681 - 82
hsa-mir-1226
GUGAGGGCAUGCAGGCCUGGAUGGGGCAGCUGGGAUGGUCCAAAAGGGUGGCCUCACCAGCCCUGUGU
UCCCUAG chr3 47891069 rs181697923 G A 0.000199681 +
25
hsa-mir-1226
GUGAGGGCAUGCAGGCCUGGAUGGGGCAGCUGGGAUGGUCCAAAAGGGUGGCCUCACCAGCCCUGUGU
UCCCUAG chr3 47891106 rs373981655 C U 0.000199681 +
62
hsa-mir-1226
GUGAGGGCAUGCAGGCCUGGAUGGGGCAGCUGGGAUGGUCCAAAAGGGUGGCCUCACCAGCCCUGUGU
UCCCUAG chr3 47891117 rs141645399 U C 0.000399361 +
73
hsa-mir-1227
GUGGGGCCAGGCGGUGGUGGGCACUGCUGGGGUGGGCACAGCAGCCAUGCAGAGCGGGCAUUUGACCC
CGUGCCACCCUUUUCCCCAG chr19 2234146 rs112440628 G C
null null 3
hsa-mir-1227
GUGGGGCCAGGCGGUGGUGGGCACUGCUGGGGUGGGCACAGCAGCCAUGCAGAGCGGGCAUUUGACCC
CGUGCCACCCUUUUCCCCAG chr19 2234093 rs190788838 G A
0.000399361 - 56
hsa-mir-1227
GUGGGGCCAGGCGGUGGUGGGCACUGCUGGGGUGGGCACAGCAGCCAUGCAGAGCGGGCAUUUGACCC
CGUGCCACCCUUUUCCCCAG chr19 2234086 rs74807305 U A
0.000199681 - 63
hsa-mir-1227
GUGGGGCCAGGCGGUGGUGGGCACUGCUGGGGUGGGCACAGCAGCCAUGCAGAGCGGGCAUUUGACCC
CGUGCCACCCUUUUCCCCAG chr19 2234080 rs139405773 C U
0.000199681 - 69
hsa-mir-1228
GUGGGCGGGGGCAGGUGUGUGGUGGGUGGUGGCCUGCGGUGAGCAGGGCCCUCACACCUGCCUCGCCC
CCCAG chr12 57588322 rs200703242 G A 0.000798722 + 36

hsa-mir-1228
GUGGGCGGGGGCAGGUGUGUGGUGGGUGGUGGCCUGCGGUGAGCAGGGCCCUCACACCUGCCUCGCCC

CCCAG chr12 57588323 rs139262076 C U 0.00399361 + 37

hsa-mir-1228
GUGGGCGGGGGCAGGUGUGUGGUGGGUGGUGGCCUGCGGUGAGCAGGGCCCUCACACCUGCCUCGCCC
CCCAG chr12 57588336 rs200087058 C G 0.000199681 + 50

hsa-mir-1229
GUGGGUAGGGUUUGGGGGAGAGCGUGGGCUGGGGUUCAGGGACACCCUCUCACCACUGCCCUCCCACA
G chr5 179225324 rs2291418 C U 0.0275559 - 23

hsa-mir-1229
GUGGGUAGGGUUUGGGGGAGAGCGUGGGCUGGGGUUCAGGGACACCCUCUCACCACUGCCCUCCCACA
G chr5 179225317 . U C 0.000199681 - 30

hsa-mir-1229
GUGGGUAGGGUUUGGGGGAGAGCGUGGGCUGGGGUUCAGGGACACCCUCUCACCACUGCCCUCCCACA
G chr5 179225301 . C U 0.000199681 - 46

hsa-mir-1231
GUCAGUGUCUGGGCGGACAGCUGCAGGAAAGGGAAGACCAAGGCUUGCUGUCUGUCCAGUCUGCCACC
CUACCCUGUCUGUUCUUGCCACAG chr1 201777741 . C U
0.000199681 + 3
hsa-mir-1231
GUCAGUGUCUGGGCGGACAGCUGCAGGAAAGGGAAGACCAAGGCUUGCUGUCUGUCCAGUCUGCCACC
CUACCCUGUCUGUUCUUGCCACAG chr1 201777751 rs116338160 G A
0.00958466 + 13
hsa-mir-1231
GUCAGUGUCUGGGCGGACAGCUGCAGGAAAGGGAAGACCAAGGCUUGCUGUCUGUCCAGUCUGCCACC
CUACCCUGUCUGUUCUUGCCACAG chr1 201777752 rs112032363 C A
0.00958466 + 14
hsa-mir-1231
GUCAGUGUCUGGGCGGACAGCUGCAGGAAAGGGAAGACCAAGGCUUGCUGUCUGUCCAGUCUGCCACC
CUACCCUGUCUGUUCUUGCCACAG chr1 201777753 . G A
0.000399361 + 15
hsa-mir-1231
GUCAGUGUCUGGGCGGACAGCUGCAGGAAAGGGAAGACCAAGGCUUGCUGUCUGUCCAGUCUGCCACC
CUACCCUGUCUGUUCUUGCCACAG chr1 201777819 . U C
0.000199681 + 81
hsa-mir-1233-1
GUGAGUGGGAGGCCAGGGCACGGCAGGGGGAGCUGCAGGGCUAUGGGAGGGGCCCCAGCGUCUGAGCC
CUGUCCUCCCGCAG chr15 34674345 rs71309450 - G null
null 7
hsa-mir-1233-1
GUGAGUGGGAGGCCAGGGCACGGCAGGGGGAGCUGCAGGGCUAUGGGAGGGGCCCCAGCGUCUGAGCC
CUGUCCUCCCGCAG chr15 34674326 rs347882 G C null
null 26
hsa-mir-1233-1

GUGAGUGGGAGGCCAGGGCACGGCAGGGGGAGCUGCAGGGCUAUGGGAGGGGCCCCAGCGUCUGAGCC CUGUCCUCCCGCAG chr15 34674309 rs347881 A G null null 43
hsa-mir-1233-2 GUGAGUGGGAGGCCAGGGCACGGCAGGGGGAGCUGCAGGGCUAUGGGAGGGGCCCCAGCGUCUGAGCC CUGUCCUCCCGCAG chr15 34820566 rs71309450 - G null null 7
hsa-mir-1233-2 GUGAGUGGGAGGCCAGGGCACGGCAGGGGGAGCUGCAGGGCUAUGGGAGGGGCCCCAGCGUCUGAGCC CUGUCCUCCCGCAG chr15 34820547 rs347882 G C null null 26
hsa-mir-1233-2 GUGAGUGGGAGGCCAGGGCACGGCAGGGGGAGCUGCAGGGCUAUGGGAGGGGCCCCAGCGUCUGAGCC CUGUCCUCCCGCAG chr15 34820530 rs347881 A G null null 43
hsa-mir-1234 GUGAGUGUGGGGUGGCUGGGGCGGGGGGGGCCCGGGGACGGCUUGGGCCUGCCUAGUCGGCCUGACCA CCCACCCCACAG chr8 145625542 rs76908615 GG - null null 18

hsa-mir-1234 GUGAGUGUGGGGUGGCUGGGGCGGGGGGGGCCCGGGGACGGCUUGGGCCUGCCUAGUCGGCCUGACCA CCCACCCCACAG chr8 145625537 rs66769762 GG - null null 23

hsa-mir-1234 GUGAGUGUGGGGUGGCUGGGGCGGGGGGGGCCCGGGGACGGCUUGGGCCUGCCUAGUCGGCCUGACCA CCCACCCCACAG chr8 145625536 rs2291134 G C null null 24

hsa-mir-1234 GUGAGUGUGGGGUGGCUGGGGCGGGGGGGGCCCGGGGACGGCUUGGGCCUGCCUAGUCGGCCUGACCA CCCACCCCACAG chr8 145625535 rs75169642 G C null null 25

hsa-mir-1236 GUGAGUGACAGGGGAAAUGGGGAUGGACUGGAAGUGGGCAGCAUGGAGCUGACCUUCAUCAUGGCUUG GCCAACAUAAUGCCUCUUCCCCUUGUCUCUCCAG chr6 31924708 . A G 0.000199681 - 10
hsa-mir-1236 GUGAGUGACAGGGGAAAUGGGGAUGGACUGGAAGUGGGCAGCAUGGAGCUGACCUUCAUCAUGGCUUG GCCAACAUAAUGCCUCUUCCCCUUGUCUCUCCAG chr6 31924631 rs185147690 C G 0.00199681 - 87
hsa-mir-1237 GUGGGAGGGCCCAGGCGCGGGCAGGGGUGGGGGUGGCAGAGCGCUGUCCCGGGGGCGGGGCCGAAGCG CGGCGACCGUAACUCCUUCUGCUCCGUCCCCCAG chr11 64136103 rs199916083 G U 0.000399361 + 30
hsa-mir-1237 GUGGGAGGGCCCAGGCGCGGGCAGGGGUGGGGGUGGCAGAGCGCUGUCCCGGGGGCGGGGCCGAAGCG CGGCGACCGUAACUCCUUCUGCUCCGUCCCCCAG chr11 64136130 rs113909793 G A null null 57

hsa-mir-1237
GUGGGAGGGCCCAGGCGCGGGCAGGGGUGGGGGUGGCAGAGCGCUGUCCCGGGGGCGGGGCCGAAGCG CGGCGACCGUAACUCCUUCUGCUCCGUCCCCCAG chr11 64136145 . C G 0.000199681 + 72
hsa-mir-1237
GUGGGAGGGCCCAGGCGCGGGCAGGGGUGGGGGUGGCAGAGCGCUGUCCCGGGGGCGGGGCCGAAGCG CGGCGACCGUAACUCCUUCUGCUCCGUCCCCCAG chr11 64136160 . C A 0.000199681 + 87
hsa-mir-1237
GUGGGAGGGCCCAGGCGCGGGCAGGGGUGGGGGUGGCAGAGCGCUGUCCCGGGGGCGGGGCCGAAGCG CGGCGACCGUAACUCCUUCUGCUCCGUCCCCCAG chr11 64136167 . G A 0.00159744 + 94
hsa-mir-1237
GUGGGAGGGCCCAGGCGCGGGCAGGGGUGGGGGUGGCAGAGCGCUGUCCCGGGGGCGGGGCCGAAGCG CGGCGACCGUAACUCCUUCUGCUCCGUCCCCCAG chr11 64136170 . C U 0.000199681 + 97
hsa-mir-1238
GUGAGUGGGAGCCCCAGUGUGUGGUUGGGGCCAUGGCGGGUGGGCAGCCCAGCCUCUGAGCCUUCCUC GUCUGUCUGCCCCAG chr19 10662835 rs199912582 G A 0.000599042 + 38
hsa-mir-1238
GUGAGUGGGAGCCCCAGUGUGUGGUUGGGGCCAUGGCGGGUGGGCAGCCCAGCCUCUGAGCCUUCCUC GUCUGUCUGCCCCAG chr19 10662844 rs201531397 G A 0.000199681 + 47
hsa-mir-1238
GUGAGUGGGAGCCCCAGUGUGUGGUUGGGGCCAUGGCGGGUGGGCAGCCCAGCCUCUGAGCCUUCCUC GUCUGUCUGCCCCAG chr19 10662859 . C A 0.000399361 + 62
hsa-mir-1238
GUGAGUGGGAGCCCCAGUGUGUGGUUGGGGCCAUGGCGGGUGGGCAGCCCAGCCUCUGAGCCUUCCUC GUCUGUCUGCCCCAG chr19 10662866 rs200136327 G A 0.000199681 + 69
hsa-mir-124-2
AUCAAGAUUAGAGGCUCUGCUCUCCGUGUUCACAGCGGACCUUGAUUUAAUGUCAUACAAUUAAGGCA CGCGGUGAAUGCCAAGAGCGGAGCCUACGGCUGCACUUGAA chr8 65291712 . A G 0.000199681 + 7
hsa-mir-124-2
AUCAAGAUUAGAGGCUCUGCUCUCCGUGUUCACAGCGGACCUUGAUUUAAUGUCAUACAAUUAAGGCA CGCGGUGAAUGCCAAGAGCGGAGCCUACGGCUGCACUUGAA chr8 65291793 . G U 0.000199681 + 88
hsa-mir-124-2
AUCAAGAUUAGAGGCUCUGCUCUCCGUGUUCACAGCGGACCUUGAUUUAAUGUCAUACAAUUAAGGCA CGCGGUGAAUGCCAAGAGCGGAGCCUACGGCUGCACUUGAA chr8 65291808 rs72631829 A G 0.000399361 + 103
hsa-mir-1243
CUAAAACUGGAUCAAUUAUAGGAGUGAAAUAAAGGUCCAUCUCCUGCCUAUUUAUUACUUUGCUUUGG UAAUAAAUCUAUUUUUAAAAGAACC chr4 114028104 rs148339577 A U

0.00239617 + 86
hsa-mir-124-3
UGAGGGCCCCUCUGCGUGUUCACAGCGGACCUUGAUUUAAUGUCUAUACAAUUAAGGCACGCGGUGAA
UGCCAAGAGAGGCGCCUCC chr20 61809907 rs34059726 G U null
null 56
hsa-mir-1244-1
AUCUUAUUCCGAGCAUUCCAGUAACUUUUUUGUGUAUGUACUUAGCUGUACUAUAAGUAGUUGGUUUG
UAUGAGAUGGUUAAAAA chr2 232578032 . C G 0.000199681 +
9
hsa-mir-1244-1
AUCUUAUUCCGAGCAUUCCAGUAACUUUUUUGUGUAUGUACUUAGCUGUACUAUAAGUAGUUGGUUUG
UAUGAGAUGGUUAAAAA chr2 232578048 . C U 0.000399361 +
25
hsa-mir-1244-1
AUCUUAUUCCGAGCAUUCCAGUAACUUUUUUGUGUAUGUACUUAGCUGUACUAUAAGUAGUUGGUUUG
UAUGAGAUGGUUAAAAA chr2 232578049 . U A 0.000199681 +
26
hsa-mir-1244-1
AUCUUAUUCCGAGCAUUCCAGUAACUUUUUUGUGUAUGUACUUAGCUGUACUAUAAGUAGUUGGUUUG
UAUGAGAUGGUUAAAAA chr2 232578091 . G A 0.000599042 +
68
hsa-mir-1244-1
AUCUUAUUCCGAGCAUUCCAGUAACUUUUUUGUGUAUGUACUUAGCUGUACUAUAAGUAGUUGGUUUG
UAUGAGAUGGUUAAAAA chr2 232578099 . U C 0.000199681 +
76
hsa-mir-1244-2
AUCUUAUUCCGAGCAUUCCAGUAACUUUUUUGUGUAUGUACUUAGCUGUACUAUAAGUAGUUGGUUUG
UAUGAGAUGGUUAAAAA chr5 118310287 . U A 0.000199681 +
7
hsa-mir-1244-2
AUCUUAUUCCGAGCAUUCCAGUAACUUUUUUGUGUAUGUACUUAGCUGUACUAUAAGUAGUUGGUUUG
UAUGAGAUGGUUAAAAA chr5 118310291 rs370764262 G A
0.000998403 + 11
hsa-mir-1244-2
AUCUUAUUCCGAGCAUUCCAGUAACUUUUUUGUGUAUGUACUUAGCUGUACUAUAAGUAGUUGGUUUG
UAUGAGAUGGUUAAAAA chr5 118310319 rs374764915 U G
0.000399361 + 39
hsa-mir-1244-2
AUCUUAUUCCGAGCAUUCCAGUAACUUUUUUGUGUAUGUACUUAGCUGUACUAUAAGUAGUUGGUUUG
UAUGAGAUGGUUAAAAA chr5 118310349 . U C 0.000199681 +
69
hsa-mir-1244-3
AUCUUAUUCCGAGCAUUCCAGUAACUUUUUUGUGUAUGUACUUAGCUGUACUAUAAGUAGUUGGUUUG
UAUGAGAUGGUUAAAAA chr12 9392072 rs367702648 U C
0.000199681 - 76
hsa-mir-1244-4
AUCUUAUUCCGAGCAUUCCAGUAACUUUUUUGUGUAUGUACUUAGCUGUACUAUAAGUAGUUGGUUUG

UAUGAGAUGGUUAAAAA chr12 12264915 . U C 0.000399361 + 30

hsa-mir-1244-4 AUCUUAUUCCGAGCAUUCCAGUAACUUUUUUGUGUAUGUACUUAGCUGUACUAUAAGUAGUUGGUUUGUAUGAGAUGGUUAAAAA chr12 12264938 rs373688352 A G 0.000199681 + 53

hsa-mir-1245a AUUUAUGUAUAGGCCUUUAGAUCAUCUGAUGUUGAAUACUCUUUAAGUGAUCUAAAGGCCUACAUAUAAA chr2 189842822 rs113906492 A U 0.0139776 + 5

hsa-mir-1245a AUUUAUGUAUAGGCCUUUAGAUCAUCUGAUGUUGAAUACUCUUUAAGUGAUCUAAAGGCCUACAUAUAAA chr2 189842826 . A G 0.000199681 + 9

hsa-mir-1245a AUUUAUGUAUAGGCCUUUAGAUCAUCUGAUGUUGAAUACUCUUUAAGUGAUCUAAAGGCCUACAUAUAAA chr2 189842885 . A C 0.000199681 + 68

hsa-mir-1245b UUUAUAUGUAGGCCUUUAGAUCACUUAAAGAGUAUUCAACAUCAGAUGAUCUAAAGGCCUAUACAUAAA chr2 189842885 . U G 0.000199681 - 3

hsa-mir-1245b UUUAUAUGUAGGCCUUUAGAUCACUUAAAGAGUAUUCAACAUCAGAUGAUCUAAAGGCCUAUACAUAAA chr2 189842826 . U C 0.000199681 - 62

hsa-mir-1245b UUUAUAUGUAGGCCUUUAGAUCACUUAAAGAGUAUUCAACAUCAGAUGAUCUAAAGGCCUAUACAUAAA chr2 189842822 rs113906492 U A 0.0139776 - 66

hsa-mir-1246 UGUAUCCUUGAAUGGAUUUUUGGAGCAGGAGUGGACACCUGACCCAAAGGAAAUCAAUCCAUAGGCUAGCAAU chr2 177465775 . C U 0.000199681 - 6

hsa-mir-1247 CCGCUUGCCUCGCCCAGCGCAGCCCCGGCCGCUGGGCGCACCCGUCCCGUUCGUCCCCGGACGUUGCUCUCUACCCCGGGAACGUCGAGACUGGAGCGCCCGAACUGAGCCACCUUCGCGGACCCCGAGAGCGGCG chr14 102026634 . C G 0.000399361 - 126

hsa-mir-1248 UUUACCUUCUUGUAUAAGCACUGUGCUAAAAUUGCAGACACUAGGACCAUGUCUUGGUUUUUGCAAUAAUGCUAGCAGAGUACACACAAGAAGAAAAGUAACAGCA chr3 186504477 . A U 0.000199681 + 17

hsa-mir-1248 UUUACCUUCUUGUAUAAGCACUGUGCUAAAAUUGCAGACACUAGGACCAUGUCUUGGUUUUUGCAAUAAUGCUAGCAGAGUACACACAAGAAGAAAAGUAACAGCA chr3 186504499 . C G 0.000199681 + 39

hsa-mir-1248
UUUACCUUCUUGUAUAAGCACUGUGCUAAAAUUGCAGACACUAGGACCAUGUCUUGGUUUUUGCAAUAAUGCUAGCAGAGUACACACAAGAAGAAAAGUAACAGCA chr3 186504532 rs73063489 C U 0.00159744 + 72
hsa-mir-1248
UUUACCUUCUUGUAUAAGCACUGUGCUAAAAUUGCAGACACUAGGACCAUGUCUUGGUUUUUGCAAUAAUGCUAGCAGAGUACACACAAGAAGAAAAGUAACAGCA chr3 186504537 . A G 0.000199681 + 77
hsa-mir-1249
GGGAGGAGGGAGGAGAUGGGCCAAGUUCCCUCUGGCUGGAACGCCCUUCCCCCCCUUCUUCACCUG chr22 45596885 . A U 0.000199681 - 16

hsa-mir-1250
CUGUCCCGCUGGCCUGGCAGGUGACGGUGCUGGAUGUGGCCUUUUUGCCUUUUCUAAAGGCCACAUUUUCCAGCCCAUUCAACCUUCCAGAGCCCUCUGAAGUGGCCACAGGC chr17 79107084 rs376611669 C U 0.000199681 - 25

hsa-mir-1250
CUGUCCCGCUGGCCUGGCAGGUGACGGUGCUGGAUGUGGCCUUUUUGCCUUUUCUAAAGGCCACAUUUUCCAGCCCAUUCAACCUUCCAGAGCCCUCUGAAGUGGCCACAGGC chr17 79107068 . C U 0.000199681 - 41
hsa-mir-1250
CUGUCCCGCUGGCCUGGCAGGUGACGGUGCUGGAUGUGGCCUUUUUGCCUUUUCUAAAGGCCACAUUUUCCAGCCCAUUCAACCUUCCAGAGCCCUCUGAAGUGGCCACAGGC chr17 79107061 . C U 0.000199681 - 48
hsa-mir-1250
CUGUCCCGCUGGCCUGGCAGGUGACGGUGCUGGAUGUGGCCUUUUUGCCUUUUCUAAAGGCCACAUUUUCCAGCCCAUUCAACCUUCCAGAGCCCUCUGAAGUGGCCACAGGC chr17 79107049 . G C 0.000199681 - 60
hsa-mir-1250
CUGUCCCGCUGGCCUGGCAGGUGACGGUGCUGGAUGUGGCCUUUUUGCCUUUUCUAAAGGCCACAUUUUCCAGCCCAUUCAACCUUCCAGAGCCCUCUGAAGUGGCCACAGGC chr17 79107017 . G A 0.000199681 - 92
hsa-mir-1251
GUGGACUCUAGCUGCCAAAGGCGCUUCUCCUUCUGAACAGAGCGCUUUGCUCAGCCAGUGUAGACAUGGC chr12 97885708 . C U 0.000199681 + 22

hsa-mir-1251
GUGGACUCUAGCUGCCAAAGGCGCUUCUCCUUCUGAACAGAGCGCUUUGCUCAGCCAGUGUAGACAUGGC chr12 97885715 . C U 0.000199681 + 29

hsa-mir-1251
GUGGACUCUAGCUGCCAAAGGCGCUUCUCCUUCUGAACAGAGCGCUUUGCUCAGCCAGUGUAGACAUGGC chr12 97885720 rs372195313 U C 0.000399361 + 34

hsa-mir-1251
GUGGACUCUAGCUGCCAAAGGCGCUUCUCCUUCUGAACAGAGCGCUUUGCUCAGCCAGUGUAGACAUG

GC chr12 97885730 rs368928135 G U 0.000399361 + 44

hsa-mir-1252
AGAAAGAAGGAAAUUGAAUUCAUUUAGAAAAGAGAAUUCCAAAUGAGCUUAAUUUCCUUUUUUCU
chr12 79813049 rs115256251 A G 0.00319489 + 13

hsa-mir-1252
AGAAAGAAGGAAAUUGAAUUCAUUUAGAAAAGAGAAUUCCAAAUGAGCUUAAUUUCCUUUUUUCU
chr12 79813075 . C G 0.000199681 + 39

hsa-mir-1252
AGAAAGAAGGAAAUUGAAUUCAUUUAGAAAAGAGAAUUCCAAAUGAGCUUAAUUUCCUUUUUUCU
chr12 79813083 . G C 0.000199681 + 47

hsa-mir-1253
AGCAGCAAGAGAUAGAAUCCAAAAGAGAAGAAGAUCAGCCUGCAGAUGUGGACUGCUAAAUGCAGGCU
GAUCUUCUCCCCUUUGGGAUUCUCUUAUGAGAAGCCA chr17 2651455 . A G
0.000199681 - 22
hsa-mir-1253
AGCAGCAAGAGAUAGAAUCCAAAAGAGAAGAAGAUCAGCCUGCAGAUGUGGACUGCUAAAUGCAGGCU
GAUCUUCUCCCCUUUGGGAUUCUCUUAUGAGAAGCCA chr17 2651405 rs77498226 C
A 0.00219649 - 72
hsa-mir-1253
AGCAGCAAGAGAUAGAAUCCAAAAGAGAAGAAGAUCAGCCUGCAGAUGUGGACUGCUAAAUGCAGGCU
GAUCUUCUCCCCUUUGGGAUUCUCUUAUGAGAAGCCA chr17 2651398 rs139010443 C
U 0.000399361 - 79
hsa-mir-1253
AGCAGCAAGAGAUAGAAUCCAAAAGAGAAGAAGAUCAGCCUGCAGAUGUGGACUGCUAAAUGCAGGCU
GAUCUUCUCCCCUUUGGGAUUCUCUUAUGAGAAGCCA chr17 2651389 . U C
0.000399361 - 88
hsa-mir-1253
AGCAGCAAGAGAUAGAAUCCAAAAGAGAAGAAGAUCAGCCUGCAGAUGUGGACUGCUAAAUGCAGGCU
GAUCUUCUCCCCUUUGGGAUUCUCUUAUGAGAAGCCA chr17 2651377 rs7217038 A
U 0.0123802 - 100
hsa-mir-1254-1
GGUGGGAGGAUUGCUUGAGCCUGGAAGCUGGAGCCUGCAGUGAACUAUCAUUGUGCCACUGUACUCCA
GCCUAGGCAACAAAAUGAAAUCCUGUCUA chr10 70519078 . G A
0.000599042 + 4
hsa-mir-1254-1
GGUGGGAGGAUUGCUUGAGCCUGGAAGCUGGAGCCUGCAGUGAACUAUCAUUGUGCCACUGUACUCCA
GCCUAGGCAACAAAAUGAAAUCCUGUCUA chr10 70519093 rs138812323 G A
0.000199681 + 19
hsa-mir-1254-1
GGUGGGAGGAUUGCUUGAGCCUGGAAGCUGGAGCCUGCAGUGAACUAUCAUUGUGCCACUGUACUCCA
GCCUAGGCAACAAAAUGAAAUCCUGUCUA chr10 70519169 . C U
0.000199681 + 95
hsa-mir-1254-2

CUGAGCCUGGAAGCUGGAGCCUGCAGUGAGCUAUGAUCAUGUCCCUGUACUCUAGCCUGGGCA
chr10 23682350 rs367976701 G A 0.000399361 + 17

hsa-mir-1254-2
CUGAGCCUGGAAGCUGGAGCCUGCAGUGAGCUAUGAUCAUGUCCCUGUACUCUAGCCUGGGCA
chr10 23682383 rs200793185 C U 0.000599042 + 50

hsa-mir-1255a
AUUGGAAAUCCUUUGAGUUGCUUCUCAAGGAUGAGCAAAGAAAGUAGAUUUUUUAGAUUCUAAAGAAA
CUAUCUUCUUUGCUCAUCCUUGAGAAGCAACUCCUUAUCCAUUAA chr4 102251542 . G
A 0.00139776 - 30
hsa-mir-1255a
AUUGGAAAUCCUUUGAGUUGCUUCUCAAGGAUGAGCAAAGAAAGUAGAUUUUUUAGAUUCUAAAGAAA
CUAUCUUCUUUGCUCAUCCUUGAGAAGCAACUCCUUAUCCAUUAA chr4 102251532
rs192307370 G U 0.00139776 - 40

hsa-mir-1255a
AUUGGAAAUCCUUUGAGUUGCUUCUCAAGGAUGAGCAAAGAAAGUAGAUUUUUUAGAUUCUAAAGAAA
CUAUCUUCUUUGCUCAUCCUUGAGAAGCAACUCCUUAUCCAUUAA chr4 102251501 rs28664200
A G 0.368211 - 71
hsa-mir-1255b-1
UACGGAUGAGCAAAGAAAGUGGUUUCUUAAAAUGGAAUCUACUCUUUGUGAAGAUGCUGUGAA
chr4 36428048 rs6841938 C U 0.0880591 - 3

hsa-mir-1255b-1
UACGGAUGAGCAAAGAAAGUGGUUUCUUAAAAUGGAAUCUACUCUUUGUGAAGAUGCUGUGAA
chr4 36428046 rs139535532 G U 0.000599042 - 5

hsa-mir-1255b-1
UACGGAUGAGCAAAGAAAGUGGUUUCUUAAAAUGGAAUCUACUCUUUGUGAAGAUGCUGUGAA
chr4 36428017 . G C 0.000199681 - 34

hsa-mir-1255b-1
UACGGAUGAGCAAAGAAAGUGGUUUCUUAAAAUGGAAUCUACUCUUUGUGAAGAUGCUGUGAA
chr4 36428006 rs113789096 U C 0.00419329 - 45

hsa-mir-1255b-2
UCUUACGGAUGAGCAAAGAAAGUGGUUUGCGCCUCAAGAAACCACUUUCUUUGCUCAUCCAUAAGGA
chr1 167967904 rs189709980 G C 0.000399361 + 7

hsa-mir-1255b-2
UCUUACGGAUGAGCAAAGAAAGUGGUUUGCGCCUCAAGAAACCACUUUCUUUGCUCAUCCAUAAGGA
chr1 167967953 . C A 0.000199681 + 56

hsa-mir-1255b-2
UCUUACGGAUGAGCAAAGAAAGUGGUUUGCGCCUCAAGAAACCACUUUCUUUGCUCAUCCAUAAGGA
chr1 167967958 rs79639536 A G 0.0203674 + 61

hsa-mir-1255b-2
UCUUACGGAUGAGCAAAGAAAGUGGUUUGCGCCUCAAGAAACCACUUUCUUUGCUCAUCCAUAAGGA chr1 167967963 rs142690749 G C 0.000199681 + 66

hsa-mir-1256
AGUCAGCCUGUUGAAGCUUUGAAGCUUUGAUGCCAGGCAUUGACUUCUCACUAGCUGUGAAAGUCCUAGCUAAAGAGAAGUCAAUGCAUGACAUCUUGUUUCAAUAGAUGGCUGUUUCA chr1 21314920 . G C 0.000599042 - 6
hsa-mir-1256
AGUCAGCCUGUUGAAGCUUUGAAGCUUUGAUGCCAGGCAUUGACUUCUCACUAGCUGUGAAAGUCCUAGCUAAAGAGAAGUCAAUGCAUGACAUCUUGUUUCAAUAGAUGGCUGUUUCA chr1 21314829 rs376254658 U C 0.000199681 - 97

hsa-mir-1257
GCCCUGGGCUUGUGCUUGGGGAGUGAAUGAUGGGUUCUGACCCCCAUGCACCCCUGUGGGCCCCUGGCAUCACUGGCCCCAUCCUUCACCCCUGCCAACCACGCUUGCCCUGUGCCU chr20 60528670 rs140605296 C U 0.000199681 - 49

hsa-mir-1257
GCCCUGGGCUUGUGCUUGGGGAGUGAAUGAUGGGUUCUGACCCCCAUGCACCCCUGUGGGCCCCUGGCAUCACUGGCCCCAUCCUUCACCCCUGCCAACCACGCUUGCCCUGUGCCU chr20 60528617 rs367783642 C U 0.000199681 - 102

hsa-mir-1258
CUGUGGCUUCCACGACCUAAUCCUAACUCCUGCGAGUCCCUGGAGUUAGGAUUAGGUCGUGGAAGCCACAGGA chr2 180725568 rs146754630 A G 0.0081869 - 68

hsa-mir-125a
UGCCAGUCUCUAGGUCCCUGAGACCCUUUAACCUGUGAGGACAUCCAGGGUCACAGGUGAGGUUCUUGGGAGCCUGGCGUCUGGCC chr19 52196528 rs12975333 G U null null 22
hsa-mir-125a
UGCCAGUCUCUAGGUCCCUGAGACCCUUUAACCUGUGAGGACAUCCAGGGUCACAGGUGAGGUUCUUGGGAGCCUGGCGUCUGGCC chr19 52196574 rs143525573 G A 0.000199681 + 68
hsa-mir-125b-2
ACCAGACUUUUCCUAGUCCCUGAGACCCUAACUUGUGAGGUAUUUUAGUAACAUCACAAGUCAGGCUCUUGGGACCUAGGCGGAGGGGA chr21 17962615 . A G 0.000199681 + 59
hsa-mir-125b-2
ACCAGACUUUUCCUAGUCCCUGAGACCCUAACUUGUGAGGUAUUUUAGUAACAUCACAAGUCAGGCUCUUGGGACCUAGGCGGAGGGGA chr21 17962644 . G A 0.000199681 + 88
hsa-mir-126
CGCUGGCGACGGGACAUUAUUACUUUUGGUACGCGCUGUGACACUUCAAACUCGUACCGUGAGUAAUAAUGCGCCGUCCACGGCA chr9 139565064 rs371589474 G A

0.000199681	+	11
hsa-mir-126	CGCUGGCGACGGGACAUUAUUACUUUUGGUACGCGCUGUGACACUUCAAACUCGUACCGUGAGUAAUAAUGCGCCGUCCACGGCA	chr9	139565098	.	U	A	0.000199681	+	45
hsa-mir-126	CGCUGGCGACGGGACAUUAUUACUUUUGGUACGCGCUGUGACACUUCAAACUCGUACCGUGAGUAAUAAUGCGCCGUCCACGGCA	chr9	139565119	.	A	G	0.000199681	+	66
hsa-mir-126	CGCUGGCGACGGGACAUUAUUACUUUUGGUACGCGCUGUGACACUUCAAACUCGUACCGUGAGUAAUAAUGCGCCGUCCACGGCA	chr9	139565134	rs199992070	C	U	0.000399361	+	81
hsa-mir-1260a	ACCUUUCCAGCUCAUCCCACCUCUGCCACCAAAACACUCAUCGCGGGGUCAGAGGGAGUGCCAAAAAAGGUAA	chr14	77732586	.	C	U	0.000199681	+	26

hsa-mir-1260a	ACCUUUCCAGCUCAUCCCACCUCUGCCACCAAAACACUCAUCGCGGGGUCAGAGGGAGUGCCAAAAAAGGUAA	chr14	77732603	.	G	A	0.000199681	+	43

hsa-mir-1260a	ACCUUUCCAGCUCAUCCCACCUCUGCCACCAAAACACUCAUCGCGGGGUCAGAGGGAGUGCCAAAAAAGGUAA	chr14	77732628	.	A	G	0.000798722	+	68

hsa-mir-1260b	UCUCCGUUUAUCCCACCACUGCCACCAUUAUUGCUACUGUUCAGCAGGUGCUGCUGGUGGUGAUGGUGAUAGUCUGGUGGGGGCGGUGG	chr11	96074607	rs371771349	G	U	0.000199681	+	6
hsa-mir-1260b	UCUCCGUUUAUCCCACCACUGCCACCAUUAUUGCUACUGUUCAGCAGGUGCUGCUGGUGGUGAUGGUGAUAGUCUGGUGGGGGCGGUGG	chr11	96074638	.	C	U	0.000399361	+	37
hsa-mir-1260b	UCUCCGUUUAUCCCACCACUGCCACCAUUAUUGCUACUGUUCAGCAGGUGCUGCUGGUGGUGAUGGUGAUAGUCUGGUGGGGGCGGUGG	chr11	96074649	rs34913856	G	-	null	null	48
hsa-mir-1261	UGCUAUGGAUAAGGCUUUGGCUUAUGGGGAUAUUGUGGUUGAUCUGUUCUAUCCAGAUGACUGAAACUUUCUCCAUAGCAGC	chr11	90602368	.	C	G	0.000399361	-	3
hsa-mir-1261	UGCUAUGGAUAAGGCUUUGGCUUAUGGGGAUAUUGUGGUUGAUCUGUUCUAUCCAGAUGACUGAAACUUUCUCCAUAGCAGC	chr11	90602296	rs139601788	A	G	0.00698882	-	75
hsa-mir-1262	AUCUACAAUGGUGAUGGGUGAAUUUGUAGAAGGAUGAAAGUCAAAGAAUCCUUCUGGGAACUAAUUUU

UGGCCUUCAACAAGAAUUGUGAUAU chr1 68649290 rs138343697 U C
0.00119808 - 4
hsa-mir-1262
AUCUACAAUGGUGAUGGGUGAAUUUGUAGAAGGAUGAAAGUCAAAGAAUCCUUCUGGGAACUAAUUUU
UGGCCUUCAACAAGAAUUGUGAUAU chr1 68649260 . A G
0.000399361 - 34
hsa-mir-1262
AUCUACAAUGGUGAUGGGUGAAUUUGUAGAAGGAUGAAAGUCAAAGAAUCCUUCUGGGAACUAAUUUU
UGGCCUUCAACAAGAAUUGUGAUAU chr1 68649258 rs147113488 G A
0.00658946 - 36
hsa-mir-1262
AUCUACAAUGGUGAUGGGUGAAUUUGUAGAAGGAUGAAAGUCAAAGAAUCCUUCUGGGAACUAAUUUU
UGGCCUUCAACAAGAAUUGUGAUAU chr1 68649205 . G A 0.00119808
- 89
hsa-mir-1263
CUACCCCAAAAUAUGGUACCCUGGCAUACUGAGUAUUUUAAUACUGGCAUACUCAGUAUGCCAUGUUG
CCAUAUUUUGGGGUAGCA chr3 163889342 . A G 0.00359425 -
3
hsa-mir-1263
CUACCCCAAAAUAUGGUACCCUGGCAUACUGAGUAUUUUAAUACUGGCAUACUCAGUAUGCCAUGUUG
CCAUAUUUUGGGGUAGCA chr3 163889327 . A G 0.000199681 -
18
hsa-mir-1263
CUACCCCAAAAUAUGGUACCCUGGCAUACUGAGUAUUUUAAUACUGGCAUACUCAGUAUGCCAUGUUG
CCAUAUUUUGGGGUAGCA chr3 163889284 . C U 0.000199681 -
61
hsa-mir-1264
AGGUCCUCAAUAAGUAUUUGUUGAAAGAAUAAAUAAACCAACAAGUCUUAUUUGAGCACCUGUUAUGU
G chrX 113887150 . U C 0.000264901 + 21

hsa-mir-1265
AUGGUUUGGGACUCAGGAUGUGGUCAAGUGUUGUUAAGGCAUGUUCAGGAACAAUACUUGACCACAUU
UUGAAUUCCAAACCAUAU chr10 14478575 rs368618020 A G 0.00219649
+ 1
hsa-mir-1265
AUGGUUUGGGACUCAGGAUGUGGUCAAGUGUUGUUAAGGCAUGUUCAGGAACAAUACUUGACCACAUU
UUGAAUUCCAAACCAUAU chr10 14478576 rs149384303 U C 0.00119808
+ 2
hsa-mir-1265
AUGGUUUGGGACUCAGGAUGUGGUCAAGUGUUGUUAAGGCAUGUUCAGGAACAAUACUUGACCACAUU
UUGAAUUCCAAACCAUAU chr10 14478613 . G U 0.000199681 +
39
hsa-mir-1265
AUGGUUUGGGACUCAGGAUGUGGUCAAGUGUUGUUAAGGCAUGUUCAGGAACAAUACUUGACCACAUU
UUGAAUUCCAAACCAUAU chr10 14478618 rs11259096 U C 0.103634
+ 44
hsa-mir-1267

CUCCCAAAUCUCCUGUUGAAGUGUAAUCCCCACCUCCAGCAUUGGGGAUUACAUUUCAACAUGAGAUUUGGAUGAGGA chr13 108183538 rs76080861 A G null null 59

hsa-mir-1267 CUCCCAAAUCUCCUGUUGAAGUGUAAUCCCCACCUCCAGCAUUGGGGAUUACAUUUCAACAUGAGAUUUGGAUGAGGA chr13 108183525 . A G 0.000199681 - 72

hsa-mir-1268a UAGCCGGGCGUGGUGGUGGGGGCCUGUGGUCCCAGCUACUUUGGAGGCUGAG chr15 22513275 . G A 0.000199681 - 6

hsa-mir-1268b ACCCGGGCGUGGUGGUGGGGGUGGGUGCCUGUAAUUCCAGCUAGUUGGGA chr17 78072630 . C U 0.000199681 + 4

hsa-mir-1268b ACCCGGGCGUGGUGGUGGGGGUGGGUGCCUGUAAUUCCAGCUAGUUGGGA chr17 78072635 . G A 0.000199681 + 9

hsa-mir-1268b ACCCGGGCGUGGUGGUGGGGGUGGGUGCCUGUAAUUCCAGCUAGUUGGGA chr17 78072651 . G A 0.00179712 + 25

hsa-mir-1268b ACCCGGGCGUGGUGGUGGGGGUGGGUGCCUGUAAUUCCAGCUAGUUGGGA chr17 78072653 rs59320012 G A 0.00339457 + 27

hsa-mir-1268b ACCCGGGCGUGGUGGUGGGGGUGGGUGCCUGUAAUUCCAGCUAGUUGGGA chr17 78072676 rs111756476 A U 0.0545128 + 50

hsa-mir-1269a UGGAUUGCCUAGACCAGGGAAGCCAGUUGGCAUGGCUCAGUCCAAGUCUGACCACCUGAGGAAUGCCUGGACUGAGCCGUGCUACUGGCUUCCCUGGUCUCCAGC chr4 67142564 . C U 0.000599042 + 23

hsa-mir-1269a UGGAUUGCCUAGACCAGGGAAGCCAGUUGGCAUGGCUCAGUCCAAGUCUGACCACCUGAGGAAUGCCUGGACUGAGCCGUGCUACUGGCUUCCCUGGUCUCCAGC chr4 67142583 . C U 0.000199681 + 42

hsa-mir-1269a UGGAUUGCCUAGACCAGGGAAGCCAGUUGGCAUGGCUCAGUCCAAGUCUGACCACCUGAGGAAUGCCUGGACUGAGCCGUGCUACUGGCUUCCCUGGUCUCCAGC chr4 67142619 . C U 0.000199681 + 78

hsa-mir-1269a UGGAUUGCCUAGACCAGGGAAGCCAGUUGGCAUGGCUCAGUCCAAGUCUGACCACCUGAGGAAUGCCUGGACUGAGCCGUGCUACUGGCUUCCCUGGUCUCCAGC chr4 67142620 rs73239138 G A 0.394169 + 79

hsa-mir-1269b UGAGGUUUCUGGACUGAGCCAUGCUACUGGCUUCUCUGGUUCUCCAGCUUACAGAUGGCUUAUCAUGGGACCUCU chr17 12820658 . G A 0.000199681 - 2

hsa-mir-1269b UGAGGUUUCUGGACUGAGCCAUGCUACUGGCUUCUCUGGUUCUCCAGCUUACAGAUGGCUUAUCAUGG

GACCUCU chr17 12820646 rs7210937 C G 0.352037 - 14
hsa-mir-1269b
UGAGGUUUCUGGACUGAGCCAUGCUACUGGCUUCUCUGGUUCUCCAGCUUACAGAUGGCUUAUCAUGG
GACCUCU chr17 12820632 rs12451747 U G 0.394968 - 28
hsa-mir-1272
CCAGAUCAGAUCUGGGUGCGAUGAUGAUGGCAGCAAAUUCUGAAAACGUGCUCAGUGUCUUUAUAACA
GGAAAGCCGUAAACUUAGAAAUGUAGGCUGCAGCUCGUGUGCUCUGUGGUCUGGGCUGGUA chr15 65054709 rs373550098 U C 0.000199681 - 6

hsa-mir-1272
CCAGAUCAGAUCUGGGUGCGAUGAUGAUGGCAGCAAAUUCUGAAAACGUGCUCAGUGUCUUUAUAACA
GGAAAGCCGUAAACUUAGAAAUGUAGGCUGCAGCUCGUGUGCUCUGUGGUCUGGGCUGGUA chr15 65054668 rs114965408 C U 0.00119808 - 47

hsa-mir-1272
CCAGAUCAGAUCUGGGUGCGAUGAUGAUGGCAGCAAAUUCUGAAAACGUGCUCAGUGUCUUUAUAACA
GGAAAGCCGUAAACUUAGAAAUGUAGGCUGCAGCUCGUGUGCUCUGUGGUCUGGGCUGGUA chr15 65054649 . A G 0.000199681 - 66

hsa-mir-1272
CCAGAUCAGAUCUGGGUGCGAUGAUGAUGGCAGCAAAUUCUGAAAACGUGCUCAGUGUCUUUAUAACA
GGAAAGCCGUAAACUUAGAAAUGUAGGCUGCAGCUCGUGUGCUCUGUGGUCUGGGCUGGUA chr15 65054606 . G U 0.000199681 - 109

hsa-mir-1273a
UGAGGCAGGAGAAUUGCUUGAACCCGGGUGGUGGAGGUUGCAGUGAGCCAAGAUUGCGCCACUGCACU
CCAGCCUGGGCGACAAAGCAAGACUCUUUCUUGGA chr8 101036259 rs184062845 U C 0.000199681 - 54
hsa-mir-1273c
UGCAGCCUGGGCGACAAAACGAGACCCUGUCUUUUUUUUUUUCUGAGACAGAGUCUCGUUCUGUUGCC
CAAGCUGGA chr6 155174524 rs111252952 - U null null 31

hsa-mir-1273c
UGCAGCCUGGGCGACAAAACGAGACCCUGUCUUUUUUUUUUUCUGAGACAGAGUCUCGUUCUGUUGCC
CAAGCUGGA chr6 155174534 . U C 0.000399361 + 41

hsa-mir-1273c
UGCAGCCUGGGCGACAAAACGAGACCCUGUCUUUUUUUUUUUCUGAGACAGAGUCUCGUUCUGUUGCC
CAAGCUGGA chr6 155174551 rs376685737 G A 0.000998403 + 58
hsa-mir-1273d
GAAUCGCUUGAACCCAUGAGGUUGAGGCUGCAGUGAGCCAAGAUCGUGCCACUGCACUUCAGCCUGGG
UGACAAGAGCGAAACUUC chr1 10287820 rs141221242 C A 0.0123802 + 45
hsa-mir-1273d

GAAUCGCUUGAACCCAUGAGGUUGAGGCUGCAGUGAGCCAAGAUCGUGCCACUGCACUUCAGCCUGGG UGACAAGAGCGAAACUUC chr1 10287821 . G A 0.000599042 + 46

hsa-mir-1273d GAAUCGCUUGAACCCAUGAGGUUGAGGCUGCAGUGAGCCAAGAUCGUGCCACUGCACUUCAGCCUGGG UGACAAGAGCGAAACUUC chr1 10287824 rs150712502 C U 0.0197684 + 49

hsa-mir-1273d GAAUCGCUUGAACCCAUGAGGUUGAGGCUGCAGUGAGCCAAGAUCGUGCCACUGCACUUCAGCCUGGG UGACAAGAGCGAAACUUC chr1 10287854 . G A 0.000399361 + 79

hsa-mir-1273f AGGUGGGAGGAUUGCUUGAGCCUGGGAGAUGGAGGUUGCAGUGAGCUGAGAUCACGCAACUGCACCCC CAGCCUGGGCCAUAGAGUCAGUCCUUGUCUC chr1 53394424 . C A 0.000199681 + 79

hsa-mir-1273h UACUUGGGUGACUAAGGCAGGAUUGCUUGAGCCUGGGAGGUCAAGGCUGCAGUGUCGUGGUCACAGCU UGCUGCAGACUCGACCUCCCAGGCUUAAGCAAUCCUCCUGCUCGAGUG chr16 24214442 rs187115161 G A 0.000399361 + 6

hsa-mir-1273h UACUUGGGUGACUAAGGCAGGAUUGCUUGAGCCUGGGAGGUCAAGGCUGCAGUGUCGUGGUCACAGCU UGCUGCAGACUCGACCUCCCAGGCUUAAGCAAUCCUCCUGCUCGAGUG chr16 24214486 . C U 0.000199681 + 50

hsa-mir-1273h UACUUGGGUGACUAAGGCAGGAUUGCUUGAGCCUGGGAGGUCAAGGCUGCAGUGUCGUGGUCACAGCU UGCUGCAGACUCGACCUCCCAGGCUUAAGCAAUCCUCCUGCUCGAGUG chr16 24214493 . G A 0.000599042 + 57

hsa-mir-1273h UACUUGGGUGACUAAGGCAGGAUUGCUUGAGCCUGGGAGGUCAAGGCUGCAGUGUCGUGGUCACAGCU UGCUGCAGACUCGACCUCCCAGGCUUAAGCAAUCCUCCUGCUCGAGUG chr16 24214532 . A U 0.000399361 + 96

hsa-mir-1275 CCUCUGUGAGAAAGGGUGUGGGGGAGAGGCUGUCUUGUGUCUGUAAGUAUGCCAAACUUAUUUUCCCC AAGGCAGAGGGA chr6 33967814 . G C 0.000199681 - 15

hsa-mir-1275 CCUCUGUGAGAAAGGGUGUGGGGGAGAGGCUGUCUUGUGUCUGUAAGUAUGCCAAACUUAUUUUCCCC AAGGCAGAGGGA chr6 33967806 rs370361021 G U 0.000199681 - 23

hsa-mir-1275 CCUCUGUGAGAAAGGGUGUGGGGGAGAGGCUGUCUUGUGUCUGUAAGUAUGCCAAACUUAUUUUCCCC AAGGCAGAGGGA chr6 33967800 rs377188203 G A 0.000199681 - 29

hsa-mir-1275 CCUCUGUGAGAAAGGGUGUGGGGGAGAGGCUGUCUUGUGUCUGUAAGUAUGCCAAACUUAUUUUCCCC AAGGCAGAGGGA chr6 33967796 . U A 0.000199681 - 33

hsa-mir-1275
CCUCUGUGAGAAAGGGUGUGGGGGAGAGGCUGUCUUGUGUCUGUAAGUAUGCCAAACUUAUUUUCCCC
AAGGCAGAGGGA chr6 33967787 rs76156362 U A 0.00439297 -
42
hsa-mir-1276
CCCCAGCUAGGUAAAGAGCCCUGUGGAGACACCUGGAUUCAGAGAACAUGUCUCCACUGAGCACUUGG
GCCUUGAUGGCGGCU chr15 86313794 rs34381260 - A null
null 16
hsa-mir-1276
CCCCAGCUAGGUAAAGAGCCCUGUGGAGACACCUGGAUUCAGAGAACAUGUCUCCACUGAGCACUUGG
GCCUUGAUGGCGGCU chr15 86313782 rs372186220 G A
0.000199681 - 28
hsa-mir-1276
CCCCAGCUAGGUAAAGAGCCCUGUGGAGACACCUGGAUUCAGAGAACAUGUCUCCACUGAGCACUUGG
GCCUUGAUGGCGGCU chr15 86313731 rs201382503 C U
0.000599042 - 79
hsa-mir-1277
ACCUCCCAAAUAUAUAUAUAUAUGUACGUAUGUGUAUAUAAAUGUAUACGUAGAUAUAUAUGUAUUUU
UGGUGGGUUU chrX 117520360 rs188834116 U C 0.000529801 +
4
hsa-mir-1277
ACCUCCCAAAUAUAUAUAUAUAUGUACGUAUGUGUAUAUAAAUGUAUACGUAGAUAUAUAUGUAUUUU
UGGUGGGUUU chrX 117520389 . U C 0.000264901 + 33

hsa-mir-1278
AUUUGCUCAUAGAUGAUAUGCAUAGUACUCCCAGAACUCAUUAAGUUGGUAGUACUGUGCAUAUCAUC
UAUGAGCGAAUAGchr1 193105672 . A G 0.000199681 + 40

hsa-mir-1279 AUAUUCACAAAAAUUCAUAUUGCUUCUUUCUAAUGCCAAGAAAGAAGAGUAUAAGAACUUCC
chr12 69666979 . U C 0.000798722 - 20

hsa-mir-1279 AUAUUCACAAAAAUUCAUAUUGCUUCUUUCUAAUGCCAAGAAAGAAGAGUAUAAGAACUUCC
chr12 69666969 . C U 0.000399361 - 30

hsa-mir-1279 AUAUUCACAAAAAUUCAUAUUGCUUCUUUCUAAUGCCAAGAAAGAAGAGUAUAAGAACUUCC
chr12 69666943 . A C 0.000199681 - 56

hsa-mir-1281 AGGGGGCACCGGGAGGAGGUGAGUGUCUCUUGUCGCCUCCUCCUCUCCCCCCUU
chr22 41488545 rs374817246 C U 0.000199681 + 29

hsa-mir-1281 AGGGGGCACCGGGAGGAGGUGAGUGUCUCUUGUCGCCUCCUCCUCUCCCCCCUU
chr22 41488550 . C U 0.000798722 + 34

hsa-mir-1281 AGGGGGCACCGGGAGGAGGUGAGUGUCUCUUGUCGCCUCCUCCUCUCCCCCCUU
chr22 41488558 rs367572051 C U 0.000199681 + 42

hsa-mir-1281 AGGGGGCACCGGGAGGAGGUGAGUGUCUCUUGUCGCCUCCUCCUCUCCCCCCUU
chr22 41488567 . C G 0.000998403 + 51

hsa-mir-1281 AGGGGGCACCGGGAGGAGGUGAGUGUCUCUUGUCGCCUCCUCCUCUCCCCCCUU
chr22 41488568 . C U 0.00119808 + 52

hsa-mir-128-1
UGAGCUGUUGGAUUCGGGGCCGUAGCACUGUCUGAGAGGUUUACAUUUCUCACAGUGAACCGGUCUCU
UUUUCAGCUGCUUC chr2 136422988 rs117812383 G A
0.000199681 + 22
hsa-mir-128-1
UGAGCUGUUGGAUUCGGGGCCGUAGCACUGUCUGAGAGGUUUACAUUUCUCACAGUGAACCGGUCUCU
UUUUCAGCUGCUUC chr2 136423009 . A G 0.000798722 +
43
hsa-mir-1282
CCUUCUUCUCGUUUGCCUUUUUCUGCUUCUGCUGCAUGAUCUCCGAGUCCCUGGGGGUAGAGAUGAUG
GGGCACUGGGAGGUACCAGAGGGCAAAAAGGAC chr15 44085909 rs11269 C
A null null 49
hsa-mir-1282
CCUUCUUCUCGUUUGCCUUUUUCUGCUUCUGCUGCAUGAUCUCCGAGUCCCUGGGGGUAGAGAUGAUG
GGGCACUGGGAGGUACCAGAGGGCAAAAAGGAC chr15 44085868 . G A
0.000199681 - 90
hsa-mir-1283-1
CUCAAGCUAUGAGUCUACAAAGGAAAGCGCUUUCUGUUGUCAGAAAGAAGAGAAAGCGCUUCCCUUUU
GAGGGUUACGGUUUGAGAA chr19 54191743 rs57111412 A G 0.291933
+ 9
hsa-mir-1283-1
CUCAAGCUAUGAGUCUACAAAGGAAAGCGCUUUCUGUUGUCAGAAAGAAGAGAAAGCGCUUCCCUUUU
GAGGGUUACGGUUUGAGAA chr19 54191790 rs142752420 G U
0.000399361 + 56
hsa-mir-1283-1
CUCAAGCUAUGAGUCUACAAAGGAAAGCGCUUUCUGUUGUCAGAAAGAAGAGAAAGCGCUUCCCUUUU
GAGGGUUACGGUUUGAGAA chr19 54191794 . U C 0.000199681 +
60
hsa-mir-1283-2
CUCAAGCUGUGAGUCUACAAAGGAAAGCGCUUUCUGUUGUCUGAAAGAAAAGAAAUCGCUUCCCUUUG
GAGUGUUACGGUUUGAGAA chr19 54261549 rs71363366 C G 0.00998403
+ 64
hsa-mir-1283-2
CUCAAGCUGUGAGUCUACAAAGGAAAGCGCUUUCUGUUGUCUGAAAGAAAAGAAAUCGCUUCCCUUUG
GAGUGUUACGGUUUGAGAA chr19 54261556 . G C 0.000199681 +
71
hsa-mir-1283-2
CUCAAGCUGUGAGUCUACAAAGGAAAGCGCUUUCUGUUGUCUGAAAGAAAAGAAAUCGCUUCCCUUUG
GAGUGUUACGGUUUGAGAA chr19 54261562 . C G 0.000199681 +
77
hsa-mir-1283-2

CUCAAGCUGUGAGUCUACAAAGGAAAGCGCUUUCUGUUGUCUGAAAGAAAAGAAAUCGCUUCCCUUUG GAGUGUUACGGUUUGAGAA chr19 54261563 rs373281715 G A 0.000599042 + 78
hsa-mir-1284
AUUUUGAUAUAUAAGCCAGUUUAAUGUUUUCUAUACAGACCCUGGCUUUUCUUAAAUUUUAUAUAUUG GAAAGCCCAUGUUUGUAUUGGAAACUGCUGGUUUCUUUCAUACUGAAAAUCU chr3 71591230 . A G 0.000199681 - 11
hsa-mir-1284
AUUUUGAUAUAUAAGCCAGUUUAAUGUUUUCUAUACAGACCCUGGCUUUUCUUAAAUUUUAUAUAUUG GAAAGCCCAUGUUUGUAUUGGAAACUGCUGGUUUCUUUCAUACUGAAAAUCU chr3 71591179 . U C 0.000399361 - 62
hsa-mir-1284
AUUUUGAUAUAUAAGCCAGUUUAAUGUUUUCUAUACAGACCCUGGCUUUUCUUAAAUUUUAUAUAUUG GAAAGCCCAUGUUUGUAUUGGAAACUGCUGGUUUCUUUCAUACUGAAAAUCU chr3 71591164 . A G 0.000199681 - 77
hsa-mir-1284
AUUUUGAUAUAUAAGCCAGUUUAAUGUUUUCUAUACAGACCCUGGCUUUUCUUAAAUUUUAUAUAUUG GAAAGCCCAUGUUUGUAUUGGAAACUGCUGGUUUCUUUCAUACUGAAAAUCU chr3 71591156 rs372709262 A C 0.00279553 - 85

hsa-mir-1285-2
UUUGGGAGGCCGAGGCUGGUGCAUCACUUGAGCCCAGCAAUUUGAGACCAAUCUGGGCAACAAAGUGA GACCUCCGUCUCUACAAAGA chr2 70480127 . C U 0.000199681 - 11
hsa-mir-1285-2
UUUGGGAGGCCGAGGCUGGUGCAUCACUUGAGCCCAGCAAUUUGAGACCAAUCUGGGCAACAAAGUGA GACCUCCGUCUCUACAAAGA chr2 70480123 . G A 0.000399361 - 15
hsa-mir-1285-2
UUUGGGAGGCCGAGGCUGGUGCAUCACUUGAGCCCAGCAAUUUGAGACCAAUCUGGGCAACAAAGUGA GACCUCCGUCUCUACAAAGA chr2 70480108 . G A 0.000199681 - 30
hsa-mir-1285-2
UUUGGGAGGCCGAGGCUGGUGCAUCACUUGAGCCCAGCAAUUUGAGACCAAUCUGGGCAACAAAGUGA GACCUCCGUCUCUACAAAGA chr2 70480088 . A C 0.000199681 - 50
hsa-mir-1285-2
UUUGGGAGGCCGAGGCUGGUGCAUCACUUGAGCCCAGCAAUUUGAGACCAAUCUGGGCAACAAAGUGA GACCUCCGUCUCUACAAAGA chr2 70480062 rs111851523 G A 0.00798722 - 76
hsa-mir-1285-2
UUUGGGAGGCCGAGGCUGGUGCAUCACUUGAGCCCAGCAAUUUGAGACCAAUCUGGGCAACAAAGUGA GACCUCCGUCUCUACAAAGA chr2 70480051 rs113580833 G U 0.00858626 - 87
hsa-mir-1286
UGUCCUCUGGGGACUCAGCUUGCUCUGGCUGCUGGAUUGAAUUAGCUGCAGGACCAAGAUGAGCCCUU GGUGGAGACA chr22 20236701 rs71312743 G U null null 34

hsa-mir-1287
GUUGUGCUGUCCAGGUGCUGGAUCAGUGGUUCGAGUCUGAGCCUUUAAAAGCCACUCUAGCCACAGAU
GCAGUGAUUGGAGCCAUGACAA chr10 100155033 rs200279130 C U
0.000199681 - 32
hsa-mir-1287
GUUGUGCUGUCCAGGUGCUGGAUCAGUGGUUCGAGUCUGAGCCUUUAAAAGCCACUCUAGCCACAGAU
GCAGUGAUUGGAGCCAUGACAA chr10 100155019 rs78666598 U A
null null 46
hsa-mir-1287
GUUGUGCUGUCCAGGUGCUGGAUCAGUGGUUCGAGUCUGAGCCUUUAAAAGCCACUCUAGCCACAGAU
GCAGUGAUUGGAGCCAUGACAA chr10 100154978 rs201015325 A G
0.000199681 - 87
hsa-mir-1289-1
UUCUCAAUUUUUAGUAGGAAUUAAAAACAAAACUGGUAAAUGCAGACUCUUGGUUUCCACCCCCAGAG
AAUCCCUAAACCGGGGGUGGAGUCCAGGAAUCUGCAUUUUAGAAAGUACCCAGGGUGAUUCUGAUAAUUGGGAAC
A chr20 34041842 rs139884309 A G 0.00139776 - 78

hsa-mir-1289-2
CCACGGUCCUAGUUAAAAAGGCACAUUCCUAGACCCUGCCUCAGAACUACUGAACAGAGUCACUGGGU
GUGGAGUCCAGGAAUCUGCAUUUUUACCCCUAUCGCCCCCGCC chr5 132763362
rs112928293 U A 0.000199681 - 37

hsa-mir-1289-2
CCACGGUCCUAGUUAAAAAGGCACAUUCCUAGACCCUGCCUCAGAACUACUGAACAGAGUCACUGGGU
GUGGAGUCCAGGAAUCUGCAUUUUUACCCCUAUCGCCCCCGCC chr5 132763351
rs115216563 U G 0.0299521 - 48

hsa-mir-1289-2
CCACGGUCCUAGUUAAAAAGGCACAUUCCUAGACCCUGCCUCAGAACUACUGAACAGAGUCACUGGGU
GUGGAGUCCAGGAAUCUGCAUUUUUACCCCUAUCGCCCCCGCC chr5 132763335 rs75310044
U G 0.00858626 - 64
hsa-mir-1289-2
CCACGGUCCUAGUUAAAAAGGCACAUUCCUAGACCCUGCCUCAGAACUACUGAACAGAGUCACUGGGU
GUGGAGUCCAGGAAUCUGCAUUUUUACCCCUAUCGCCCCCGCC chr5 132763305 rs74634410
A C null null 94
hsa-mir-1289-2
CCACGGUCCUAGUUAAAAAGGCACAUUCCUAGACCCUGCCUCAGAACUACUGAACAGAGUCACUGGGU
GUGGAGUCCAGGAAUCUGCAUUUUUACCCCUAUCGCCCCCGCC chr5 132763298
rs117347811 U C null null 101

hsa-mir-1289-2
CCACGGUCCUAGUUAAAAAGGCACAUUCCUAGACCCUGCCUCAGAACUACUGAACAGAGUCACUGGGU
GUGGAGUCCAGGAAUCUGCAUUUUUACCCCUAUCGCCCCCGCC chr5 132763295 rs35296450
C G null null 104
hsa-mir-1290
GAGCGUCACGUUGACACUCAAAAAGUUUCAGAUUUUGGAACAUUUCGGAUUUUGGAUUUUUGGAUCAG

GGAUGCUCAA chr1 19223639 rs75705742 C U 0.0157748 -
4
hsa-mir-1290
GAGCGUCACGUUGACACUCAAAAAGUUUCAGAUUUUGGAACAUUUCGGAUUUUGGAUUUUUGGAUCAG
GGAUGCUCAA chr1 19223571 rs147869275 U C 0.00399361 -
72
hsa-mir-1290
GAGCGUCACGUUGACACUCAAAAAGUUUCAGAUUUUGGAACAUUUCGGAUUUUGGAUUUUUGGAUCAG
GGAUGCUCAA chr1 19223566 . A C 0.000199681 - 77

hsa-mir-1291
GGUAGAAUUCCAGUGGCCCUGACUGAAGACCAGCAGUUGUACUGUGGCUGUUGGUUUCAAGCAGAGGC
CUAAAGGACUGUCUUCCUG chr12 49048277 rs77994717 U C null
null 37
hsa-mir-1291
GGUAGAAUUCCAGUGGCCCUGACUGAAGACCAGCAGUUGUACUGUGGCUGUUGGUUUCAAGCAGAGGC
CUAAAGGACUGUCUUCCUG chr12 49048263 rs115815201 U G null
null 51
hsa-mir-129-1
GGAUCUUUUUGCGGUCUGGGCUUGCUGUUCCUCUCAACAGUAGUCAGGAAGCCCUUACCCCAAAAAGU
AUCU chr7 127847960 . A U 0.000199681 + 36

hsa-mir-1292
CCUGGGAACGGGUUCCGGCAGACGCUGAGGUUGCGUUGACGCUCGCGCCCCGGCUCCCGUUCCAGG
chr20 2633434 . G A 0.000998403 + 12

hsa-mir-1292
CCUGGGAACGGGUUCCGGCAGACGCUGAGGUUGCGUUGACGCUCGCGCCCCGGCUCCCGUUCCAGG
chr20 2633462 rs201309191 C U 0.000199681 + 40

hsa-mir-1292
CCUGGGAACGGGUUCCGGCAGACGCUGAGGUUGCGUUGACGCUCGCGCCCCGGCUCCCGUUCCAGG
chr20 2633466 rs73576045 C U 0.0117812 + 44

hsa-mir-1292
CCUGGGAACGGGUUCCGGCAGACGCUGAGGUUGCGUUGACGCUCGCGCCCCGGCUCCCGUUCCAGG
chr20 2633478 . C G 0.000199681 + 56

hsa-mir-1292
CCUGGGAACGGGUUCCGGCAGACGCUGAGGUUGCGUUGACGCUCGCGCCCCGGCUCCCGUUCCAGG
chr20 2633480 . C A 0.000199681 + 58

hsa-mir-129-2
UGCCCUUCGCGAAUCUUUUUGCGGUCUGGGCUUGCUGUACAUAACUCAAUAGCCGGAAGCCCUUACCC
CAAAAAGCAUUUGCGGAGGGCG chr11 43602984 rs189052513 A G
0.000199681 + 41
hsa-mir-129-2

UGCCCUUCGCGAAUCUUUUUGCGGUCUGGGCUUGCUGUACAUAACUCAAUAGCCGGAAGCCCUUACCC
CAAAAAGCAUUUGCGGAGGGCG chr11 43602992 rs201801670 A G
0.00599042 + 49

hsa-mir-1293
AGGUUGUUCUGGGUGGUCUGGAGAUUUGUGCAGCUUGUACCUGCACAAAUCUCCGGACCACUUAGUCU
UUA chr12 50627942 rs113699072 C U 0.0379393 - 54

hsa-mir-1293
AGGUUGUUCUGGGUGGUCUGGAGAUUUGUGCAGCUUGUACCUGCACAAAUCUCCGGACCACUUAGUCU
UUA chr12 50627936 . A U 0.000399361 - 60

hsa-mir-1293
AGGUUGUUCUGGGUGGUCUGGAGAUUUGUGCAGCUUGUACCUGCACAAAUCUCCGGACCACUUAGUCU
UUA chr12 50627935 rs189740304 C U 0.000399361 - 61

hsa-mir-1294
CACCUAAUGUGUGCCAAGAUCUGUUCAUUUAUGAUCUCACCGAGUCCUGUGAGGUUGGCAUUGUUGUC
UGGCAUUGUCUGAUAUACAACAGUGCCAACCUCACAGGACUCAGUGAGGUGAAACUGAGGAUUAGGAAGGUGUA
chr5 153726707 rs367837365 G A 0.000399361 + 42

hsa-mir-1294
CACCUAAUGUGUGCCAAGAUCUGUUCAUUUAUGAUCUCACCGAGUCCUGUGAGGUUGGCAUUGUUGUC
UGGCAUUGUCUGAUAUACAACAGUGCCAACCUCACAGGACUCAGUGAGGUGAAACUGAGGAUUAGGAAGGUGUA
chr5 153726728 rs189565536 G U 0.000199681 + 63

hsa-mir-1294
CACCUAAUGUGUGCCAAGAUCUGUUCAUUUAUGAUCUCACCGAGUCCUGUGAGGUUGGCAUUGUUGUC
UGGCAUUGUCUGAUAUACAACAGUGCCAACCUCACAGGACUCAGUGAGGUGAAACUGAGGAUUAGGAAGGUGUA
chr5 153726756 rs114020978 G A 0.0123802 + 91

hsa-mir-1294
CACCUAAUGUGUGCCAAGAUCUGUUCAUUUAUGAUCUCACCGAGUCCUGUGAGGUUGGCAUUGUUGUC
UGGCAUUGUCUGAUAUACAACAGUGCCAACCUCACAGGACUCAGUGAGGUGAAACUGAGGAUUAGGAAGGUGUA
chr5 153726760 . C U 0.000199681 + 95

hsa-mir-1294
CACCUAAUGUGUGCCAAGAUCUGUUCAUUUAUGAUCUCACCGAGUCCUGUGAGGUUGGCAUUGUUGUC
UGGCAUUGUCUGAUAUACAACAGUGCCAACCUCACAGGACUCAGUGAGGUGAAACUGAGGAUUAGGAAGGUGUA
chr5 153726769 rs13186787 A G 0.0107827 + 104

hsa-mir-1294
CACCUAAUGUGUGCCAAGAUCUGUUCAUUUAUGAUCUCACCGAGUCCUGUGAGGUUGGCAUUGUUGUC
UGGCAUUGUCUGAUAUACAACAGUGCCAACCUCACAGGACUCAGUGAGGUGAAACUGAGGAUUAGGAAGGUGUA
chr5 153726803 . G A 0.000199681 + 138

hsa-mir-1295a
AGGACAUUUUGCCCAGAUCCGUGGCCUAUUCAGAAAUGUGGCCUGUGAUUAGGCCGCAGAUCUGGGUG

AAAUGUCCUCC chr1 171070937 rs191927458 G U 0.000199681 -
11
hsa-mir-1295a
AGGACAUUUUGCCCAGAUCCGUGGCCUAUUCAGAAAUGUGGCCUGUGAUUAGGCCGCAGAUCUGGGUG
AAAUGUCCUCC chr1 171070888 . A G 0.000199681 - 60

hsa-mir-1295a
AGGACAUUUUGCCCAGAUCCGUGGCCUAUUCAGAAAUGUGGCCUGUGAUUAGGCCGCAGAUCUGGGUG
AAAUGUCCUCC chr1 171070875 . G A 0.000199681 - 73

hsa-mir-1295bCACCCAGAUCUGCGGCCUAAUCACAGGCCACAUUUCUGAAUAGGCCACGGAUCUGGGCAA
chr1 171070888 . U C 0.000199681 + 9

hsa-mir-1295bCACCCAGAUCUGCGGCCUAAUCACAGGCCACAUUUCUGAAUAGGCCACGGAUCUGGGCAA
chr1 171070937 rs191927458 C A 0.000199681 + 58

hsa-mir-1297
UGUUUAUCUCUAGGGUUGAUCUAUUAGAAUUACUUAUCUGAGCCAAAGUAAUUCAAGUAAUUCAGGUG
UAGUGAAAC chr13 54886166 rs370964181 G C 0.000399361 -
18
hsa-mir-1299
CCUCAUGGCAGUGUUCUGGAAUCCUACGUGAGGGACAAUCAUUCAGACCCACGUAGCAGUGUUCUGGA
AUUCUGUGUGAGGGA chr9 69002320 . C U 0.000199681 -
2
hsa-mir-1299
CCUCAUGGCAGUGUUCUGGAAUCCUACGUGAGGGACAAUCAUUCAGACCCACGUAGCAGUGUUCUGGA
AUUCUGUGUGAGGGA chr9 69002317 rs76266132 A G null
null 5
hsa-mir-1299
CCUCAUGGCAGUGUUCUGGAAUCCUACGUGAGGGACAAUCAUUCAGACCCACGUAGCAGUGUUCUGGA
AUUCUGUGUGAGGGA chr9 69002304 rs78546776 G C null
null 18
hsa-mir-1299
CCUCAUGGCAGUGUUCUGGAAUCCUACGUGAGGGACAAUCAUUCAGACCCACGUAGCAGUGUUCUGGA
AUUCUGUGUGAGGGA chr9 69002294 rs79965448 G A null
null 28
hsa-mir-1299
CCUCAUGGCAGUGUUCUGGAAUCCUACGUGAGGGACAAUCAUUCAGACCCACGUAGCAGUGUUCUGGA
AUUCUGUGUGAGGGA chr9 69002278 . C U 0.00159744 -
44
hsa-mir-1299
CCUCAUGGCAGUGUUCUGGAAUCCUACGUGAGGGACAAUCAUUCAGACCCACGUAGCAGUGUUCUGGA
AUUCUGUGUGAGGGA chr9 69002271 rs62555121 A U null
null 51
hsa-mir-1301
GGAUUGUGGGGGGUCGCUCUAGGCACCGCAGCACUGUGCUGGGGAUGUUGCAGCUGCCUGGGAGUGAC
UUCACACAGUCCUC chr2 25551588 . A G 0.000199681 -

3
hsa-mir-1301
GGAUUGUGGGGGGUCGCUCUAGGCACCGCAGCACUGUGCUGGGGAUGUUGCAGCUGCCUGGGAGUGAC
UUCACACAGUCCUC chr2 25551583 . G C 0.000599042 -
8
hsa-mir-1301
GGAUUGUGGGGGGUCGCUCUAGGCACCGCAGCACUGUGCUGGGGAUGUUGCAGCUGCCUGGGAGUGAC
UUCACACAGUCCUC chr2 25551550 . G U 0.000199681 -
41
hsa-mir-1302-1
CAGAAAGCCCAGUUAAAUUUGAAUUUCAAGUAAACAAUGAAUAAUUGUGUAUGUAAGAAUAUCCCAUA
CAAUAUUUGGGACAUACUUAUGCUAAAAAUUAUUCCUUGCUUAUCUGAAAUUCAAAUGUAACUAGGAUUCCUGUA
chr12 113132901 rs74647838 C U 0.0275559 - 81

hsa-mir-1302-1
CAGAAAGCCCAGUUAAAUUUGAAUUUCAAGUAAACAAUGAAUAAUUGUGUAUGUAAGAAUAUCCCAUA
CAAUAUUUGGGACAUACUUAUGCUAAAAAUUAUUCCUUGCUUAUCUGAAAUUCAAAUGUAACUAGGAUUCCUGUA
chr12 113132857 rs191263402 U C 0.00219649 - 125

hsa-mir-1302-3
GGAUGCCCAGCUAGUUUGAAUUUUAGAUAAACAACGAAUAAUUUCGUAGCAUAAAUAUUUCCCAAGCU
UAGUUUGGGACAUACUUAUGCUAAAAAACAUUAUUGGUUGUUUAUCUGAGAUUCAAAAUUAAGCAUUUUA
chr2 114340663 rs6542147 C U null null 11

hsa-mir-1302-3
GGAUGCCCAGCUAGUUUGAAUUUUAGAUAAACAACGAAUAAUUUCGUAGCAUAAAUAUUUCCCAAGCU
UAGUUUGGGACAUACUUAUGCUAAAAAACAUUAUUGGUUGUUUAUCUGAGAUUCAAAAUUAAGCAUUUUA
chr2 114340631 rs74625964 U C null null 43

hsa-mir-1302-3
GGAUGCCCAGCUAGUUUGAAUUUUAGAUAAACAACGAAUAAUUUCGUAGCAUAAAUAUUUCCCAAGCU
UAGUUUGGGACAUACUUAUGCUAAAAAACAUUAUUGGUUGUUUAUCUGAGAUUCAAAAUUAAGCAUUUUA
chr2 114340628 rs7589328 G A 0.228634 - 46

hsa-mir-1302-3
GGAUGCCCAGCUAGUUUGAAUUUUAGAUAAACAACGAAUAAUUUCGUAGCAUAAAUAUUUCCCAAGCU
UAGUUUGGGACAUACUUAUGCUAAAAAACAUUAUUGGUUGUUUAUCUGAGAUUCAAAAUUAAGCAUUUUA
chr2 114340615 rs2441621 U G null null 59

hsa-mir-1302-3
GGAUGCCCAGCUAGUUUGAAUUUUAGAUAAACAACGAAUAAUUUCGUAGCAUAAAUAUUUCCCAAGCU
UAGUUUGGGACAUACUUAUGCUAAAAAACAUUAUUGGUUGUUUAUCUGAGAUUCAAAAUUAAGCAUUUUA
chr2 114340550 rs2441622 A G null null 124

hsa-mir-1302-4
AAUGCAGAAGCACAGCUAAAAUUUGAAUUUCAGAUAAACAAAUUUUUCUUAGAAUAAGUAUGUCUCCA
UGCAACAUUUGGGACAUACUUAUGCUAAAAUAUUAUUUGUGUUUCAUCUGAAAUUCAAAUUCAACUGGACAUCCU

GUAUUUU chr2 208134116 . G C 0.000199681 - 33

hsa-mir-1302-4
AAUGCAGAAGCACAGCUAAAAUUUGAAUUUCAGAUAAACAAAUUUUUCUUAGAAUAAGUAUGUCUCCA
UGCAACAUUUGGGACAUACUUAUGCUAAAAUAUUAUUUGUGUUUCAUCUGAAAUUCAAAUUCAACUGGACAUCCU
GUAUUUU chr2 208134114 rs116237969 U A 0.0275559 -
35

hsa-mir-1302-4
AAUGCAGAAGCACAGCUAAAAUUUGAAUUUCAGAUAAACAAAUUUUUCUUAGAAUAAGUAUGUCUCCA
UGCAACAUUUGGGACAUACUUAUGCUAAAAUAUUAUUUGUGUUUCAUCUGAAAUUCAAAUUCAACUGGACAUCCU
GUAUUUU chr2 208134058 . U C 0.000199681 - 91

hsa-mir-1302-4
AAUGCAGAAGCACAGCUAAAAUUUGAAUUUCAGAUAAACAAAUUUUUCUUAGAAUAAGUAUGUCUCCA
UGCAACAUUUGGGACAUACUUAUGCUAAAAUAUUAUUUGUGUUUCAUCUGAAAUUCAAAUUCAACUGGACAUCCU
GUAUUUU chr2 208134047 . U G 0.000199681 - 102

hsa-mir-1302-4
AAUGCAGAAGCACAGCUAAAAUUUGAAUUUCAGAUAAACAAAUUUUUCUUAGAAUAAGUAUGUCUCCA
UGCAACAUUUGGGACAUACUUAUGCUAAAAUAUUAUUUGUGUUUCAUCUGAAAUUCAAAUUCAACUGGACAUCCU
GUAUUUU chr2 208134017 . A G 0.000199681 - 132

hsa-mir-1302-5
UGCCCGGCCUCCCAUUAAAUUGGUUUUUCAGACAAAUCACAAAUUUGUUUAGGUAUAAGUAUAUCCCA
UGUAAUCUUUGGGACAUACUUAUGCUAAAAUAAUUGUUCCUUGUUGAUUGGAAAUUUUAAUUUUAAUUAGGUGUC
CUGUAUU chr20 49231303 . U C 0.000199681 - 20

hsa-mir-1302-5
UGCCCGGCCUCCCAUUAAAUUGGUUUUUCAGACAAAUCACAAAUUUGUUUAGGUAUAAGUAUAUCCCA
UGUAAUCUUUGGGACAUACUUAUGCUAAAAUAAUUGUUCCUUGUUGAUUGGAAAUUUUAAUUUUAAUUAGGUGUC
CUGUAUU chr20 49231248 . C A 0.000199681 - 75

hsa-mir-1302-8
CCCAUUUAAACUUGAAUUUCAUAUAAACACCGUAAUUUUCAGCAUUAGUGUAUCACAUGCAGUAUUUG
GGACAUACUUAUGCUAAAAAAUUAGGUGGUGUUGAUCUGAAAUUCCAGUGUAGAUGGGCA chr9
100125949 rs192895122 A G 0.000199681 - 15

hsa-mir-1302-8
CCCAUUUAAACUUGAAUUUCAUAUAAACACCGUAAUUUUCAGCAUUAGUGUAUCACAUGCAGUAUUUG
GGACAUACUUAUGCUAAAAAAUUAGGUGGUGUUGAUCUGAAAUUCCAGUGUAGAUGGGCA chr9
100125939 . A G 0.000199681 - 25

hsa-mir-1302-8
CCCAUUUAAACUUGAAUUUCAUAUAAACACCGUAAUUUUCAGCAUUAGUGUAUCACAUGCAGUAUUUG
GGACAUACUUAUGCUAAAAAAUUAGGUGGUGUUGAUCUGAAAUUCCAGUGUAGAUGGGCA chr9
100125865 . G A 0.000199681 - 99

hsa-mir-1302-8
CCCAUUUAAACUUGAAUUUCAUAUAAACACCGUAAUUUCAGCAUUAGUGUAUCACAUGCAGUAUUUG
GGACAUACUUAUGCUAAAAAAUUAGGUGGUGUUGAUCUGAAAUUCCAGUGUAGAUGGGCA chr9
100125848 . G A 0.000199681 - 116

hsa-mir-1303
GGCUGGGCAACAUAGCGAGACCUCAACUCUACAAUUUUUUUUUUUUUAAAUUUUAGAGACGGGGUCUU
GCUCUGUUGCCAGGCUUU chr5 154065347 rs142414368 A G
0.000399361 + 12
hsa-mir-1303
GGCUGGGCAACAUAGCGAGACCUCAACUCUACAAUUUUUUUUUUUUUAAAUUUUAGAGACGGGGUCUU
GCUCUGUUGCCAGGCUUU chr5 154065348 rs77055126 U C 0.0283546
+ 13
hsa-mir-1303
GGCUGGGCAACAUAGCGAGACCUCAACUCUACAAUUUUUUUUUUUUUAAAUUUUAGAGACGGGGUCUU
GCUCUGUUGCCAGGCUUU chr5 154065368 rs75378399 A U null
null 33
hsa-mir-1303
GGCUGGGCAACAUAGCGAGACCUCAACUCUACAAUUUUUUUUUUUUUAAAUUUUAGAGACGGGGUCUU
GCUCUGUUGCCAGGCUUU chr5 154065370 rs114588756 U A null
null 35
hsa-mir-1303
GGCUGGGCAACAUAGCGAGACCUCAACUCUACAAUUUUUUUUUUUUUAAAUUUUAGAGACGGGGUCUU
GCUCUGUUGCCAGGCUUU chr5 154065383 rs75538180 A U 0.464657
+ 48
hsa-mir-1303
GGCUGGGCAACAUAGCGAGACCUCAACUCUACAAUUUUUUUUUUUUUAAAUUUUAGAGACGGGGUCUU
GCUCUGUUGCCAGGCUUU chr5 154065385 rs33982250 A - null
null 50
hsa-mir-1304
AAACACUUGAGCCCAGCGGUUUGAGGCUACAGUGAGAUGUGAUCCUGCCACAUCUCACUGUAGCCUCG
AACCCCUGGGCUCAAGUGAUUCA chr11 93466919 rs79462725 C G
0.0163738 - 12
hsa-mir-1304
AAACACUUGAGCCCAGCGGUUUGAGGCUACAGUGAGAUGUGAUCCUGCCACAUCUCACUGUAGCCUCG
AACCCCUGGGCUCAAGUGAUUCA chr11 93466913 . G A
0.000998403 - 18
hsa-mir-1304
AAACACUUGAGCCCAGCGGUUUGAGGCUACAGUGAGAUGUGAUCCUGCCACAUCUCACUGUAGCCUCG
AACCCCUGGGCUCAAGUGAUUCA chr11 93466912 . G A
0.000199681 - 19
hsa-mir-1304
AAACACUUGAGCCCAGCGGUUUGAGGCUACAGUGAGAUGUGAUCCUGCCACAUCUCACUGUAGCCUCG
AACCCCUGGGCUCAAGUGAUUCA chr11 93466910 rs76857625 U C
null null 21
hsa-mir-1304
AAACACUUGAGCCCAGCGGUUUGAGGCUACAGUGAGAUGUGAUCCUGCCACAUCUCACUGUAGCCUCG

AACCCCUGGGCUCAAGUGAUUCA chr11 93466909 rs79759099 U C
null null 22
hsa-mir-1304
AAACACUUGAGCCCAGCGGUUUGAGGCUACAGUGAGAUGUGAUCCUGCCACAUCUCACUGUAGCCUCG
AACCCCUGGGCUCAAGUGAUUCA chr11 93466866 rs2155248 C A
0.879193 - 65
hsa-mir-1307
CAUCAAGACCCAGCUGAGUCACUGUCACUGCCUACCAAUCUCGACCGGACCUCGACCGGCUCGUCUGU
GUUGCCAAUCGACUCGGCGUGGCGUCGGUCGUGGUAGAUAGGCGGUCAUGCAUACGAAUUUUCAGCUCUUGUUCU
GGUGAC chr10 105154097 . C U 0.000199681 - 62

hsa-mir-1307
CAUCAAGACCCAGCUGAGUCACUGUCACUGCCUACCAAUCUCGACCGGACCUCGACCGGCUCGUCUGU
GUUGCCAAUCGACUCGGCGUGGCGUCGGUCGUGGUAGAUAGGCGGUCAUGCAUACGAAUUUUCAGCUCUUGUUCU
GGUGAC chr10 105154091 . U C 0.000199681 - 68

hsa-mir-1307
CAUCAAGACCCAGCUGAGUCACUGUCACUGCCUACCAAUCUCGACCGGACCUCGACCGGCUCGUCUGU
GUUGCCAAUCGACUCGGCGUGGCGUCGGUCGUGGUAGAUAGGCGGUCAUGCAUACGAAUUUUCAGCUCUUGUUCU
GGUGAC chr10 105154089 rs7911488 U C 0.303714 - 70

hsa-mir-1307
CAUCAAGACCCAGCUGAGUCACUGUCACUGCCUACCAAUCUCGACCGGACCUCGACCGGCUCGUCUGU
GUUGCCAAUCGACUCGGCGUGGCGUCGGUCGUGGUAGAUAGGCGGUCAUGCAUACGAAUUUUCAGCUCUUGUUCU
GGUGAC chr10 105154084 . A G 0.000199681 - 75

hsa-mir-1307
CAUCAAGACCCAGCUGAGUCACUGUCACUGCCUACCAAUCUCGACCGGACCUCGACCGGCUCGUCUGU
GUUGCCAAUCGACUCGGCGUGGCGUCGGUCGUGGUAGAUAGGCGGUCAUGCAUACGAAUUUUCAGCUCUUGUUCU
GGUGAC chr10 105154013 rs147668271 U C 0.000199681 - 146

hsa-mir-130b
GGCCUGCCCGACACUCUUUCCCUGUUGCACUACUAUAGGCCGCUGGGAAGCAGUGCAAUGAUGAAAGG
GCAUCGGUCAGGUC chr22 22007594 . G U 0.000199681 +
2
hsa-mir-130b
GGCCUGCCCGACACUCUUUCCCUGUUGCACUACUAUAGGCCGCUGGGAAGCAGUGCAAUGAUGAAAGG
GCAUCGGUCAGGUC chr22 22007634 rs72631822 G A 0.00259585
+ 42
hsa-mir-1321
ACAUUAUGAAGCAAGUAUUAUUAUCCCUGUUUUACAAAUAAGGAAAUAAACUCAGGGAGGUGAAUGUG
AUCAAAGAUAG chrX 85090790 . A G 0.000264901 + 6

hsa-mir-1321
ACAUUAUGAAGCAAGUAUUAUUAUCCCUGUUUUACAAAUAAGGAAAUAAACUCAGGGAGGUGAAUGUG
AUCAAAGAUAG chrX 85090809 . C U 0.000529801 + 25

hsa-mir-1321
ACAUUAUGAAGCAAGUAUUAUUAUCCCUGUUUUACAAAUAAGGAAAUAAACUCAGGGAGGUGAAUGUG
AUCAAAGAUAG chrX 85090852 rs36100620 - G null null 68

hsa-mir-1322
AGUAUCAUGAAUUAGAAACCUACUUAUUACAUAGUUUACAUAAGAAGCGUGAUGAUGCUGCUGAUGCU
GUA chr8 10682950 . A G 0.000199681 - 4

hsa-mir-1322
AGUAUCAUGAAUUAGAAACCUACUUAUUACAUAGUUUACAUAAGAAGCGUGAUGAUGCUGCUGAUGCU
GUA chr8 10682934 rs199678358 C G 0.000798722 - 20

hsa-mir-1322
AGUAUCAUGAAUUAGAAACCUACUUAUUACAUAGUUUACAUAAGAAGCGUGAUGAUGCUGCUGAUGCU
GUA chr8 10682907 rs376152593 G A 0.000199681 - 47

hsa-mir-1322
AGUAUCAUGAAUUAGAAACCUACUUAUUACAUAGUUUACAUAAGAAGCGUGAUGAUGCUGCUGAUGCU
GUA chr8 10682905 rs112004155 G A 0.0121805 - 49

hsa-mir-1322
AGUAUCAUGAAUUAGAAACCUACUUAUUACAUAGUUUACAUAAGAAGCGUGAUGAUGCUGCUGAUGCU
GUA chr8 10682898 rs189895541 U C 0.000998403 - 56

hsa-mir-1322
AGUAUCAUGAAUUAGAAACCUACUUAUUACAUAGUUUACAUAAGAAGCGUGAUGAUGCUGCUGAUGCU
GUA chr8 10682894 rs59878596 G A 0.0716853 - 60

hsa-mir-1323
ACUGAGGUCCUCAAAACUGAGGGGCAUUUUCUGUGGUUUGAAAGGAAAGUGCACCCAGUUUUGGGGAU
GUCAA chr19 54175287 rs78952474 G U null null 66

hsa-mir-1323
ACUGAGGUCCUCAAAACUGAGGGGCAUUUUCUGUGGUUUGAAAGGAAAGUGCACCCAGUUUUGGGGAU
GUCAA chr19 54175294 rs186827259 A G 0.000199681 + 73

hsa-mir-1324
CCUGAAGAGGUGCAUGAAGCCUGGUCCUGCCCUCACUGGGAACCCCCUUCCCUCUGGGUACCAGACAG
AAUUCUAUGCACUUUCCUGGAGGCUCCA chr3 75679923 . G A
0.000599042 + 10

hsa-mir-1324
CCUGAAGAGGUGCAUGAAGCCUGGUCCUGCCCUCACUGGGAACCCCCUUCCCUCUGGGUACCAGACAG
AAUUCUAUGCACUUUCCUGGAGGCUCCA chr3 75679958 . C G
0.000199681 + 45

hsa-mir-1324
CCUGAAGAGGUGCAUGAAGCCUGGUCCUGCCCUCACUGGGAACCCCCUUCCCUCUGGGUACCAGACAG
AAUUCUAUGCACUUUCCUGGAGGCUCCA chr3 75679969 . G U

0.00199681 + 56
hsa-mir-133a-1
ACAAUGCUUUGCUAGAGCUGGUAAAAUGGAACCAAAUCGCCUCUUCAAUGGAUUUGGUCCCCUUCAAC
CAGCUGUAGCUAUGCAUUGA chr18 19405743 rs369186690 A G
0.000199681 - 4
hsa-mir-133a-1
ACAAUGCUUUGCUAGAGCUGGUAAAAUGGAACCAAAUCGCCUCUUCAAUGGAUUUGGUCCCCUUCAAC
CAGCUGUAGCUAUGCAUUGA chr18 19405709 . C U
0.000199681 - 38
hsa-mir-133a-1
ACAAUGCUUUGCUAGAGCUGGUAAAAUGGAACCAAAUCGCCUCUUCAAUGGAUUUGGUCCCCUUCAAC
CAGCUGUAGCUAUGCAUUGA chr18 19405702 . U G
0.000199681 - 45
hsa-mir-133a-1
ACAAUGCUUUGCUAGAGCUGGUAAAAUGGAACCAAAUCGCCUCUUCAAUGGAUUUGGUCCCCUUCAAC
CAGCUGUAGCUAUGCAUUGA chr18 19405676 rs182673735 G A
0.000199681 - 71
hsa-mir-133a-1
ACAAUGCUUUGCUAGAGCUGGUAAAAUGGAACCAAAUCGCCUCUUCAAUGGAUUUGGUCCCCUUCAAC
CAGCUGUAGCUAUGCAUUGA chr18 19405672 rs367730853 U C
0.000599042 - 75
hsa-mir-133a-2
GGGAGCCAAAUGCUUUGCUAGAGCUGGUAAAAUGGAACCAAAUCGACUGUCCAAUGGAUUUGGUCCCC
UUCAACCAGCUGUAGCUGUGCAUUGAUGGCGCCG chr20 61162131 . C U
0.000199681 + 13
hsa-mir-133a-2
GGGAGCCAAAUGCUUUGCUAGAGCUGGUAAAAUGGAACCAAAUCGACUGUCCAAUGGAUUUGGUCCCC
UUCAACCAGCUGUAGCUGUGCAUUGAUGGCGCCG chr20 61162191 . A C
0.000199681 + 73
hsa-mir-133b
CCUCAGAAGAAAGAUGCCCCCUGCUCUGGCUGGUCAAACGGAACCAAGUCCGUCUUCCUGAGAGGUUU
GGUCCCCUUCAACCAGCUACAGCAGGGCUGGCAAUGCCCAGUCCUUGGAGA chr6 52013754
rs112599381 U C null null 34

hsa-mir-133b
CCUCAGAAGAAAGAUGCCCCCUGCUCUGGCUGGUCAAACGGAACCAAGUCCGUCUUCCUGAGAGGUUU
GGUCCCCUUCAACCAGCUACAGCAGGGCUGGCAAUGCCCAGUCCUUGGAGA chr6 52013827 .
C G 0.000199681 + 107
hsa-mir-133b
CCUCAGAAGAAAGAUGCCCCCUGCUCUGGCUGGUCAAACGGAACCAAGUCCGUCUUCCUGAGAGGUUU
GGUCCCCUUCAACCAGCUACAGCAGGGCUGGCAAUGCCCAGUCCUUGGAGA chr6 52013832
rs374103744 C U 0.000199681 + 112

hsa-mir-134
CAGGGUGUGUGACUGGUUGACCAGAGGGGCAUGCACUGUGUUCACCCUGUGGGCCACCUAGUCACCAA
CCCUC chr14 101521052 . G U 0.000199681 + 29

hsa-mir-134
CAGGGUGUGUGACUGGUUGACCAGAGGGGCAUGCACUGUGUUCACCCUGUGGGCCACCUAGUCACCAA
CCCUC chr14 101521092 . C A 0.000199681 + 69

hsa-mir-1343
GCUGGCGUCGGUGCUGGGGAGCGGCCCCCGGGUGGGCCUCUGCUCUGGCCCCUCCUGGGGCCCGCACU
CUCGCUCUGGGCCCGC chr11 34963416 rs2986407 U C 0.766773
+ 33
hsa-mir-1343
GCUGGCGUCGGUGCUGGGGAGCGGCCCCCGGGUGGGCCUCUGCUCUGGCCCCUCCUGGGGCCCGCACU
CUCGCUCUGGGCCCGC chr11 34963445 . C G 0.000199681 +
62
hsa-mir-1343
GCUGGCGUCGGUGCUGGGGAGCGGCCCCCGGGUGGGCCUCUGCUCUGGCCCCUCCUGGGGCCCGCACU
CUCGCUCUGGGCCCGC chr11 34963459 rs11032942 U C 0.1877 +
76
hsa-mir-135a-1
AGGCCUCGCUGUUCUCUAUGGCUUUUUAUUCCUAUGUGAUUCUACUGCUCACUCAUAUAGGGAUUGGA
GCCGUGGCGCACGGCGGGGACA chr3 52328298 . U C
0.000199681 - 27
hsa-mir-135a-1
AGGCCUCGCUGUUCUCUAUGGCUUUUUAUUCCUAUGUGAUUCUACUGCUCACUCAUAUAGGGAUUGGA
GCCGUGGCGCACGGCGGGGACA chr3 52328248 . G A
0.000199681 - 77
hsa-mir-135a-2
AGAUAAAUUCACUCUAGUGCUUUAUGGCUUUUUAUUCCUAUGUGAUAGUAAUAAAGUCUCAUGUAGGG
AUGGAAGCCAUGAAAUACAUUGUGAAAAAUCA chr12 97957646 rs113322127 U C
null null 57
hsa-mir-135a-2
AGAUAAAUUCACUCUAGUGCUUUAUGGCUUUUUAUUCCUAUGUGAUAGUAAUAAAGUCUCAUGUAGGG
AUGGAAGCCAUGAAAUACAUUGUGAAAAAUCA chr12 97957677 rs377622561 U C
0.000199681 + 88
hsa-mir-135b
CACUCUGCUGUGGCCUAUGGCUUUUCAUUCCUAUGUGAUUGCUGUCCCAAACUCAUGUAGGGCUAAAA
GCCAUGGGCUACAGUGAGGGGCGAGCUCC chr1 205417483 rs139405984 G C
0.000798722 - 44
hsa-mir-135b
CACUCUGCUGUGGCCUAUGGCUUUUCAUUCCUAUGUGAUUGCUGUCCCAAACUCAUGUAGGGCUAAAA
GCCAUGGGCUACAGUGAGGGGCGAGCUCC chr1 205417438 . G C
0.000199681 - 89
hsa-mir-136
UGAGCCCUCGGAGGACUCCAUUUGUUUUGAUGAUGGAUUCUUAUGCUCCAUCAUCGUCUCAAAUGAGU
CUUCAGAGGGUUCU chr14 101351048 rs189881142 G A
0.000199681 + 10
hsa-mir-136
UGAGCCCUCGGAGGACUCCAUUUGUUUUGAUGAUGGAUUCUUAUGCUCCAUCAUCGUCUCAAAUGAGU
CUUCAGAGGGUUCU chr14 101351088 rs147448304 A C

0.000199681 + 50
hsa-mir-137
GGUCCUCUGACUCUCUUCGGUGACGGGUAUUCUUGGGUGGAUAAUACGGAUUACGUUGUUAUUGCUUAAGAAUACGCGUAGUCGAGGAGAGUACCAGCGGCA chr1 98511725 . U C
0 - 3
hsa-mir-137
GGUCCUCUGACUCUCUUCGGUGACGGGUAUUCUUGGGUGGAUAAUACGGAUUACGUUGUUAUUGCUUAAGAAUACGCGUAGUCGAGGAGAGUACCAGCGGCA chr1 98511634 . C G
0.000199681 - 94
hsa-mir-138-1
CCCUGGCAUGGUGUGGUGGGGCAGCUGGUGUUGUGAAUCAGGCCGUUGCCAAUCAGAGAACGGCUACUUCACAACACCAGGGCCACACCACACUACAGG chr3 44155749 rs374104338 U C
0.000199681 + 46
hsa-mir-138-1
CCCUGGCAUGGUGUGGUGGGGCAGCUGGUGUUGUGAAUCAGGCCGUUGCCAAUCAGAGAACGGCUACUUCACAACACCAGGGCCACACCACACUACAGG chr3 44155754 . A G
0.000199681 + 51
hsa-mir-138-2
CGUUGCUGCAGCUGGUGUUGUGAAUCAGGCCGACGAGCAGCGCAUCCUCUUACCCGGCUAUUUCACGACACCAGGGUUGCAUCA chr16 56892431 rs139365823 G U
0.000399361 + 2
hsa-mir-138-2
CGUUGCUGCAGCUGGUGUUGUGAAUCAGGCCGACGAGCAGCGCAUCCUCUUACCCGGCUAUUUCACGACACCAGGGUUGCAUCA chr16 56892464 rs200558235 G A
0.000199681 + 35
hsa-mir-138-2
CGUUGCUGCAGCUGGUGUUGUGAAUCAGGCCGACGAGCAGCGCAUCCUCUUACCCGGCUAUUUCACGACACCAGGGUUGCAUCA chr16 56892470 . C A 0.000199681 +
41
hsa-mir-138-2
CGUUGCUGCAGCUGGUGUUGUGAAUCAGGCCGACGAGCAGCGCAUCCUCUUACCCGGCUAUUUCACGACACCAGGGUUGCAUCA chr16 56892507 . U C 0.000199681 +
78
hsa-mir-140
UGUGUCUCUCUCUGUGUCCUGCCAGUGGUUUUACCCUAUGGUAGGUUACGUCAUGCUGUUCUACCACAGGGUAGAACCACGGACAGGAUACCGGGGCACC chr16 69966985 . G A
0.000199681 + 2
hsa-mir-140
UGUGUCUCUCUCUGUGUCCUGCCAGUGGUUUUACCCUAUGGUAGGUUACGUCAUGCUGUUCUACCACAGGGUAGAACCACGGACAGGAUACCGGGGCACC chr16 69966994 rs112773205 U C
null null 11
hsa-mir-140
UGUGUCUCUCUCUGUGUCCUGCCAGUGGUUUUACCCUAUGGUAGGUUACGUCAUGCUGUUCUACCACAGGGUAGAACCACGGACAGGAUACCGGGGCACC chr16 69967005 rs7205289 C A
null null 22
hsa-mir-140
UGUGUCUCUCUCUGUGUCCUGCCAGUGGUUUUACCCUAUGGUAGGUUACGUCAUGCUGUUCUACCACA

GGGUAGAACCACGGACAGGAUACCGGGGCACC chr16 69967062 rs111366342 A G
null null 79
hsa-mir-141
CGGCCGGCCCUGGGUCCAUCUUCCAGUACAGUGUUGGAUGGUCUAAUUGUGAAGCUCCUAACACUGUC
UGGUAAAGAUGGCUCCCGGGUGGGUUC chr12 7073282 . C U
0.000599042 + 23
hsa-mir-141
CGGCCGGCCCUGGGUCCAUCUUCCAGUACAGUGUUGGAUGGUCUAAUUGUGAAGCUCCUAACACUGUC
UGGUAAAGAUGGCUCCCGGGUGGGUUC chr12 7073301 rs111718468 U C
null null 42
hsa-mir-141
CGGCCGGCCCUGGGUCCAUCUUCCAGUACAGUGUUGGAUGGUCUAAUUGUGAAGCUCCUAACACUGUC
UGGUAAAGAUGGCUCCCGGGUGGGUUC chr12 7073344 rs34385807 - C
null null 85
hsa-mir-142
GACAGUGCAGUCACCCAUAAAGUAGAAAGCACUACUAACAGCACUGGAGGGUGUAGUGUUUCCUACUU
UAUGGAUGAGUGUACUGUG chr17 56408639 . G A 0.000199681 -
41
hsa-mir-142
GACAGUGCAGUCACCCAUAAAGUAGAAAGCACUACUAACAGCACUGGAGGGUGUAGUGUUUCCUACUU
UAUGGAUGAGUGUACUGUG chr17 56408638 . C U 0.000199681 -
42
hsa-mir-142
GACAGUGCAGUCACCCAUAAAGUAGAAAGCACUACUAACAGCACUGGAGGGUGUAGUGUUUCCUACUU
UAUGGAUGAGUGUACUGUG chr17 56408625 . A C 0.000199681 -
55
hsa-mir-142
GACAGUGCAGUCACCCAUAAAGUAGAAAGCACUACUAACAGCACUGGAGGGUGUAGUGUUUCCUACUU
UAUGGAUGAGUGUACUGUG chr17 56408599 . U G 0.000199681 -
81
hsa-mir-143
GCGCAGCGCCCUGUCUCCCAGCCUGAGGUGCAGUGCUGCAUCUCUGGUCAGUUGGGAGUCUGAGAUGA
AGCACUGUAGCUCAGGAAGAGAGAAGUUGUUCUGCAGC chr5 148808487 . C U
0.000199681 + 7
hsa-mir-143
GCGCAGCGCCCUGUCUCCCAGCCUGAGGUGCAGUGCUGCAUCUCUGGUCAGUUGGGAGUCUGAGAUGA
AGCACUGUAGCUCAGGAAGAGAGAAGUUGUUCUGCAGC chr5 148808575 . U C
0.000199681 + 95
hsa-mir-145
CACCUUGUCCUCACGGUCCAGUUUUCCCAGGAAUCCCUUAGAUGCUAAGAUGGGGAUUCCUGGAAAUA
CUGUUCUUGAGGUCAUGGUU chr5 148810267 rs190323149 C U
0.000199681 + 59
hsa-mir-1468
GGUGGGUGGUUUCUCCGUUUGCCUGUUUCGCUGAUGUGCAUUCAACUCAUUCUCAGCAAAAUAAGCAA
AUGGAAAAUUCGUCCAUC chrX 63005918 rs145760241 U G 0.00582781
- 50
hsa-mir-1469 CUCGGCGCGGGGCGCGGGCUCCGGGUUGGGGCGAGCCAACGCCGGGG chr15

96876496 . G C 0.0107827 + 7

hsa-mir-1469 CUCGGCGCGGGGCGCGGGCUCCGGGUUGGGGCGAGCCAACGCCGGGG chr15 96876511 . C U 0.000399361 + 22

hsa-mir-146a CCGAUGUGUAUCCUCAGCUUUGAGAACUGAAUUCCAUGGGUUGUGUCAGUGUCAGACCUCUGAAAUUCAGUUCUUCAGCUGGGAUAUCUCUGUCAUCGU chr5 159912418 rs2910164 C G null null 60

hsa-mir-146a CCGAUGUGUAUCCUCAGCUUUGAGAACUGAAUUCCAUGGGUUGUGUCAGUGUCAGACCUCUGAAAUUCAGUUCUUCAGCUGGGAUAUCUCUGUCAUCGU chr5 159912436 . G U 0.000399361 + 78

hsa-mir-146b CCUGGCACUGAGAACUGAAUUCCAUAGGCUGUGAGCUCUAGCAAUGCCCUGUGGACUCAGUUCUGGUGCCCGG chr10 104196269 rs76149940 C U 0.0125799 + 1

hsa-mir-146b CCUGGCACUGAGAACUGAAUUCCAUAGGCUGUGAGCUCUAGCAAUGCCCUGUGGACUCAGUUCUGGUGCCCGG chr10 104196300 rs117717575 U G null null 32

hsa-mir-1470 GCCCUCCGCCCGUGCACCCCGGGGCAGGAGACCCCGCGGGACGCGCCGAGGUAGGGGGGAC chr19 15560380 rs113568246 G A null null 22

hsa-mir-147b UAUAAAUCUAGUGGAAACAUUUCUGCACAAACUAGAUUCUGGACACCAGUGUGCGGAAAUGCUUCUGCUACAUUUUUAGG chr15 45725255 rs56073218 C G 0.000199681 + 8

hsa-mir-147b UAUAAAUCUAGUGGAAACAUUUCUGCACAAACUAGAUUCUGGACACCAGUGUGCGGAAAUGCUUCUGCUACAUUUUUAGG chr15 45725274 . A G 0.000199681 + 27

hsa-mir-148b CAAGCACGAUUAGCAUUUGAGGUGAAGUUCUGUUAUACACUCAGGCUGUGGCUCUCUGAAAGUCAGUGCAUCACAGAACUUUGUCUCGAAAGCUUUCUA chr12 54731000 rs185315720 C U 0.000199681 + 1

hsa-mir-148b CAAGCACGAUUAGCAUUUGAGGUGAAGUUCUGUUAUACACUCAGGCUGUGGCUCUCUGAAAGUCAGUGCAUCACAGAACUUUGUCUCGAAAGCUUUCUA chr12 54731071 rs74878365 C A null null 72

hsa-mir-148b CAAGCACGAUUAGCAUUUGAGGUGAAGUUCUGUUAUACACUCAGGCUGUGGCUCUCUGAAAGUCAGUGCAUCACAGAACUUUGUCUCGAAAGCUUUCUA chr12 54731080 . U G 0.000199681 + 81

hsa-mir-149 GCCGGCGCCCGAGCUCUGGCUCCGUGUCUUCACUCCCGUGCUUGUCCGAGGAGGGAGGGAGGGACGGGGGCUGUGCUGGGGCAGCUGGA chr2 241395420 . C U

0.000798722	+	3
hsa-mir-149
GCCGGCGCCCGAGCUCUGGCUCCGUGUCUUCACUCCCGUGCUUGUCCGAGGAGGGAGGGAGGGACGGG
GGCUGUGCUGGGGCAGCUGGA	chr2	241395500	rs71428439	A	G
0.14397	+	83
hsa-mir-149
GCCGGCGCCCGAGCUCUGGCUCCGUGUCUUCACUCCCGUGCUUGUCCGAGGAGGGAGGGAGGGACGGG
GGCUGUGCUGGGGCAGCUGGA	chr2	241395503	rs2292832	U	C
0.613419	+	86
hsa-mir-151a
UUUCCUGCCCUCGAGGAGCUCACAGUCUAGUAUGUCUCAUCCCCUACUAGACUGAAGCUCCUUGAGGA
CAGGGAUGGUCAUACUCACCUC	chr8	141742750	rs149074929	U	C
0.00139776	-	3
hsa-mir-151a
UUUCCUGCCCUCGAGGAGCUCACAGUCUAGUAUGUCUCAUCCCCUACUAGACUGAAGCUCCUUGAGGA
CAGGGAUGGUCAUACUCACCUC	chr8	141742725	.	U	C
0.000199681	-	28
hsa-mir-151a
UUUCCUGCCCUCGAGGAGCUCACAGUCUAGUAUGUCUCAUCCCCUACUAGACUGAAGCUCCUUGAGGA
CAGGGAUGGUCAUACUCACCUC	chr8	141742719	.	G	U
0.000399361	-	34
hsa-mir-151b
ACCUCUGAUGUGUCAGUCUCUCUUCAGGGCUCCCGAGACACAGAAACAGACACCUGCCCUCGAGGAGC
UCACAGUCUAGACAAACAAACCCAGGGU	chr14	100575818	.	C	U
0.000798722	-	34
hsa-mir-151b
ACCUCUGAUGUGUCAGUCUCUCUUCAGGGCUCCCGAGACACAGAAACAGACACCUGCCCUCGAGGAGC
UCACAGUCUAGACAAACAAACCCAGGGU	chr14	100575791	.	C	U
0.000199681	-	61
hsa-mir-151b
ACCUCUGAUGUGUCAGUCUCUCUUCAGGGCUCCCGAGACACAGAAACAGACACCUGCCCUCGAGGAGC
UCACAGUCUAGACAAACAAACCCAGGGU	chr14	100575762	.	C	U
0.000199681	-	90
hsa-mir-151b
ACCUCUGAUGUGUCAGUCUCUCUUCAGGGCUCCCGAGACACAGAAACAGACACCUGCCCUCGAGGAGC
UCACAGUCUAGACAAACAAACCCAGGGU	chr14	100575761	rs189669291	C	A
0.000199681	-	91
hsa-mir-152
UGUCCCCCCCGGCCCAGGUUCUGUGAUACACUCCGACUCGGGCUCUGGAGCAGUCAGUGCAUGACAGA
ACUUGGGCCCGGAAGGACC	chr17	46114610	.	C	U	0.000199681	-
4
hsa-mir-152
UGUCCCCCCCGGCCCAGGUUCUGUGAUACACUCCGACUCGGGCUCUGGAGCAGUCAGUGCAUGACAGA
ACUUGGGCCCGGAAGGACC	chr17	46114580	.	C	G	0.000199681	-
34
hsa-mir-152
UGUCCCCCCCGGCCCAGGUUCUGUGAUACACUCCGACUCGGGCUCUGGAGCAGUCAGUGCAUGACAGA

ACUUGGGCCCGGAAGGACC chr17 46114572 rs200114569 G C 0.00179712 - 42

hsa-mir-153-2 AGCGGUGGCCAGUGUCAUUUUUGUGAUGUUGCAGCUAGUAAUAUGAGCCCAGUUGCAUAGUCACAAAAGUGAUCAUUGGAAACUGUG chr7 157367104 . A G 0.000199681 - 11

hsa-mir-1537 ACAGCUGUAAUUAGUCAGUUUUCUGUCCUGUCCACACAGAAAACCGUCUAGUUACAGUUGU chr1 236016347 rs192140250 G U 0.000199681 - 14

hsa-mir-1537 ACAGCUGUAAUUAGUCAGUUUUCUGUCCUGUCCACACAGAAAACCGUCUAGUUACAGUUGU chr1 236016316 . C U 0.000199681 - 45

hsa-mir-1537 ACAGCUGUAAUUAGUCAGUUUUCUGUCCUGUCCACACAGAAAACCGUCUAGUUACAGUUGU chr1 236016301 rs113301642 G A null null 60

hsa-mir-1538 GGGAACAGCAGCAACAUGGGCCUCGCUUCCUGCCGGCGCGGCCCGGGCUGCUGCUGUUCCU chr16 69599751 . C U 0.000199681 - 21

hsa-mir-1538 GGGAACAGCAGCAACAUGGGCCUCGCUUCCUGCCGGCGCGGCCCGGGCUGCUGCUGUUCCU chr16 69599735 rs191393746 C U 0.00539137 - 37

hsa-mir-1539 GGCUCUGCGGCCUGCAGGUAGCGCGAAAGUCCUGCGCGUCCCAGAUGCCC chr18 47013743 . G C 0.000599042 + 1

hsa-mir-1539 GGCUCUGCGGCCUGCAGGUAGCGCGAAAGUCCUGCGCGUCCCAGAUGCCC chr18 47013745 . C U 0.000199681 + 3

hsa-mir-1539 GGCUCUGCGGCCUGCAGGUAGCGCGAAAGUCCUGCGCGUCCCAGAUGCCC chr18 47013754 . C U 0.000199681 + 12

hsa-mir-1539 GGCUCUGCGGCCUGCAGGUAGCGCGAAAGUCCUGCGCGUCCCAGAUGCCC chr18 47013763 . G U 0.00119808 + 21

hsa-mir-1539 GGCUCUGCGGCCUGCAGGUAGCGCGAAAGUCCUGCGCGUCCCAGAUGCCC chr18 47013777 . C U 0.000599042 + 35

hsa-mir-1539 GGCUCUGCGGCCUGCAGGUAGCGCGAAAGUCCUGCGCGUCCCAGAUGCCC chr18 47013790 rs111715115 C U 0.000199681 + 48

hsa-mir-154 GUGGUACUUGAAGAUAGGUUAUCCGUGUUGCCUUCGCUUUAUUUGUGACGAAUCAUACACGGUUGACCUAUUUUUCAGUACCAA chr14 101526116 . G A 0.000199681 + 25

hsa-mir-154 GUGGUACUUGAAGAUAGGUUAUCCGUGUUGCCUUCGCUUUAUUUGUGACGAAUCAUACACGGUUGACCUAUUUUUCAGUACCAA chr14 101526127 rs41286570 G A 0.000399361 + 36

hsa-mir-154
GUGGUACUUGAAGAUAGGUUAUCCGUGUUGCCUUCGCUUUAUUUGUGACGAAUCAUACACGGUUGACC
UAUUUUUCAGUACCAA chr14 101526140 . C U 0.000199681 +
49
hsa-mir-155
CUGUUAAUGCUAAUCGUGAUAGGGGUUUUUGCCUCCAACUGACUCCUACAUAUUAGCAUUAACAG
chr21 26946325 rs140021681 U C 0.000199681 + 34

hsa-mir-1587 UUUGGGCUGGGCUGGGUUGGGCAGUUCUUCUGCUGGACUCACCUGUGACCAGC
chrX 39696856 rs191955769 C U 0.000264901 + 42

hsa-mir-1587 UUUGGGCUGGGCUGGGUUGGGCAGUUCUUCUGCUGGACUCACCUGUGACCAGC
chrX 39696863 rs145512186 C G 0.00317881 + 49

hsa-mir-15b
UUGAGGCCUUAAAGUACUGUAGCAGCACAUCAUGGUUUACAUGCUACAGUCAAGAUGCGAAUCAUUAU
UUGCUGCUCUAGAAAUUUAAGGAAAUUCAU chr3 160122421 rs146020563 A G
0.000199681 + 46
hsa-mir-15b
UUGAGGCCUUAAAGUACUGUAGCAGCACAUCAUGGUUUACAUGCUACAGUCAAGAUGCGAAUCAUUAU
UUGCUGCUCUAGAAAUUUAAGGAAAUUCAU chr3 160122434 rs192595529 G A
0.000199681 + 59
hsa-mir-16-1
GUCAGCAGUGCCUUAGCAGCACGUAAAUAUUGGCGUUAAGAUUCUAAAAUUAUCUCCAGUAUUAACUG
UGCUGCUGAAGUAAGGUUGAC chr13 50623143 rs72631826 U C
0.0081869 - 55
hsa-mir-16-1
GUCAGCAGUGCCUUAGCAGCACGUAAAUAUUGGCGUUAAGAUUCUAAAAUUAUCUCCAGUAUUAACUG
UGCUGCUGAAGUAAGGUUGAC chr13 50623110 rs371922256 A G
0.00319489 - 88
hsa-mir-17
GUCAGAAUAAUGUCAAAGUGCUUACAGUGCAGGUAGUGAUAUGUGCAUCUACUGCAGUGAAGGCACUU
GUAGCAUUAUGGUGAC chr13 92002896 . G A 0.000199681 +
38
hsa-mir-181a-2
AGAAGGGCUAUCAGGCCAGCCUUCAGAGGACUCCAAGGAACAUUCAACGCUGUCGGUGAGUUUGGGAU
UUGAAAAAACCACUGACCGUUGACUGUACCUUGGGGUCCUUA chr9 127454748 . G
A 0.000199681 + 28
hsa-mir-181b-1
CCUGUGCAGAGAUUAUUUUUUAAAAGGUCACAAUCAACAUUCAUUGCUGUCGGUGGGUUGAACUGUGU
GGACAAGCUCACUGAACAAUGAAUGCAACUGUGGCCCCGCUU chr1 198828096
rs200492369 U A 0.000399361 - 16

hsa-mir-181b-1
CCUGUGCAGAGAUUAUUUUUUAAAAGGUCACAAUCAACAUUCAUUGCUGUCGGUGGGUUGAACUGUGU
GGACAAGCUCACUGAACAAUGAAUGCAACUGUGGCCCCGCUU chr1 198828005 . G
C 0.000798722 - 107

hsa-mir-181b-2
CUGAUGGCUGCACUCAACAUUCAUUGCUGUCGGUGGGUUUGAGUCUGAAUCAACUCACUGAUCAAUGA AUGCAAACUGCGGACCAAACA chr9 127456020 . G A 0.000199681 + 32

hsa-mir-181b-2
CUGAUGGCUGCACUCAACAUUCAUUGCUGUCGGUGGGUUUGAGUCUGAAUCAACUCACUGAUCAAUGA AUGCAAACUGCGGACCAAACA chr9 127456029 rs78086449 G U null null 41

hsa-mir-181b-2
CUGAUGGCUGCACUCAACAUUCAUUGCUGUCGGUGGGUUUGAGUCUGAAUCAACUCACUGAUCAAUGA AUGCAAACUGCGGACCAAACA chr9 127456042 . C U 0.000199681 + 54

hsa-mir-181b-2
CUGAUGGCUGCACUCAACAUUCAUUGCUGUCGGUGGGUUUGAGUCUGAAUCAACUCACUGAUCAAUGA AUGCAAACUGCGGACCAAACA chr9 127456067 . C U 0.000199681 + 79

hsa-mir-181b-2
CUGAUGGCUGCACUCAACAUUCAUUGCUGUCGGUGGGUUUGAGUCUGAAUCAACUCACUGAUCAAUGA AUGCAAACUGCGGACCAAACA chr9 127456068 rs369088591 G A 0.000199681 + 80

hsa-mir-181c
CGGAAAAUUUGCCAAGGGUUUGGGGGAACAUUCAACCUGUCGGUGAGUUUGGGCAGCUCAGGCAAACC AUCGACCGUUGAGUGGACCCUGAGGCCUGGAAUUGCCAUCCU chr19 13985514 rs201394123 G A 0.000399361 + 2

hsa-mir-181d
GUCCCCUCCCCUAGGCCACAGCCGAGGUCACAAUCAACAUUCAUUGUUGUCGGUGGGUUGUGAGGACU GAGGCCAGACCCACCGGGGGAUGAAUGUCACUGUGGCUGGGCCAGACACGGCUUAAGGGGAAUGGGGAC chr19 13985689 rs373207013 G A 0.000399361 + 1

hsa-mir-181d
GUCCCCUCCCCUAGGCCACAGCCGAGGUCACAAUCAACAUUCAUUGUUGUCGGUGGGUUGUGAGGACU GAGGCCAGACCCACCGGGGGAUGAAUGUCACUGUGGCUGGGCCAGACACGGCUUAAGGGGAAUGGGGAC chr19 13985721 . A G 0.000399361 + 33

hsa-mir-181d
GUCCCCUCCCCUAGGCCACAGCCGAGGUCACAAUCAACAUUCAUUGUUGUCGGUGGGUUGUGAGGACU GAGGCCAGACCCACCGGGGGAUGAAUGUCACUGUGGCUGGGCCAGACACGGCUUAAGGGGAAUGGGGAC chr19 13985739 . C U 0.000199681 + 51

hsa-mir-181d
GUCCCCUCCCCUAGGCCACAGCCGAGGUCACAAUCAACAUUCAUUGUUGUCGGUGGGUUGUGAGGACU GAGGCCAGACCCACCGGGGGAUGAAUGUCACUGUGGCUGGGCCAGACACGGCUUAAGGGGAAUGGGGAC chr19 13985772 rs369928561 G A 0.000199681 + 84

hsa-mir-181d
GUCCCCUCCCCUAGGCCACAGCCGAGGUCACAAUCAACAUUCAUUGUUGUCGGUGGGUUGUGAGGACU

GAGGCCAGACCCACCGGGGGAUGAAUGUCACUGUGGCUGGGCCAGACACGGCUUAAGGGGAAUGGGGAC
chr19 13985805 rs377441973 C U 0.000199681 + 117

hsa-mir-181d
GUCCCCUCCCCUAGGCCACAGCCGAGGUCACAAUCAACAUUCAUUGUUGUCGGUGGGUUGUGAGGACU
GAGGCCAGACCCACCGGGGGAUGAAUGUCACUGUGGCUGGGCCAGACACGGCUUAAGGGGAAUGGGGAC
chr19 13985806 . G A 0.00119808 + 118

hsa-mir-182
GAGCUGCUUGCCUCCCCCCGUUUUUGGCAAUGGUAGAACUCACACUGGUGAGGUAACAGGAUCCGGUG
GUUCUAGACUUGCCAACUAUGGGGCGAGGACUCAGCCGGCAC chr7 129410313 . G
A 0.000199681 - 20
hsa-mir-182
GAGCUGCUUGCCUCCCCCCGUUUUUGGCAAUGGUAGAACUCACACUGGUGAGGUAACAGGAUCCGGUG
GUUCUAGACUUGCCAACUAUGGGGCGAGGACUCAGCCGGCAC chr7 129410239 rs80041074
G A 0.00259585 - 94
hsa-mir-182
GAGCUGCUUGCCUCCCCCCGUUUUUGGCAAUGGUAGAACUCACACUGGUGAGGUAACAGGAUCCGGUG
GUUCUAGACUUGCCAACUAUGGGGCGAGGACUCAGCCGGCAC chr7 129410235 rs75953509
A G null null 98
hsa-mir-182
GAGCUGCUUGCCUCCCCCCGUUUUUGGCAAUGGUAGAACUCACACUGGUGAGGUAACAGGAUCCGGUG
GUUCUAGACUUGCCAACUAUGGGGCGAGGACUCAGCCGGCAC chr7 129410228 rs77586312
C U 0.00499201 - 105
hsa-mir-182
GAGCUGCUUGCCUCCCCCCGUUUUUGGCAAUGGUAGAACUCACACUGGUGAGGUAACAGGAUCCGGUG
GUUCUAGACUUGCCAACUAUGGGGCGAGGACUCAGCCGGCAC chr7 129410227 rs76481776
G A 0.034345 - 106
hsa-mir-1825 AGAGACUGGGGUGCUGGGCUCCCCUAGACUAGGACUCCAGUGCCCUCCUCUCC
chr20 30825610 . G U 0.000199681 + 13

hsa-mir-1825 AGAGACUGGGGUGCUGGGCUCCCCUAGACUAGGACUCCAGUGCCCUCCUCUCC
chr20 30825621 rs369204612 C U 0.000199681 + 24

hsa-mir-183
CCGCAGAGUGUGACUCCUGUUCUGUGUAUGGCACUGGUAGAAUUCACUGUGAACAGUCUCAGUCAGUG
AAUUACCGAAGGGCCAUAAACAGAGCAGAGACAGAUCCACGA chr7 129414852
rs373030611 G U 0.000199681 - 3

hsa-mir-183
CCGCAGAGUGUGACUCCUGUUCUGUGUAUGGCACUGGUAGAAUUCACUGUGAACAGUCUCAGUCAGUG
AAUUACCGAAGGGCCAUAAACAGAGCAGAGACAGAUCCACGA chr7 129414843 . G
A 0.000199681 - 12
hsa-mir-183
CCGCAGAGUGUGACUCCUGUUCUGUGUAUGGCACUGGUAGAAUUCACUGUGAACAGUCUCAGUCAGUG
AAUUACCGAAGGGCCAUAAACAGAGCAGAGACAGAUCCACGA chr7 129414815 . G
A 0.000199681 - 40

hsa-mir-183
CCGCAGAGUGUGACUCCUGUUCUGUGUAUGGCACUGGUAGAAUUCACUGUGAACAGUCUCAGUCAGUG
AAUUACCGAAGGGCCAUAAACAGAGCAGAGACAGAUCCACGA chr7 129414807 . U C 0 - 48
hsa-mir-183
CCGCAGAGUGUGACUCCUGUUCUGUGUAUGGCACUGGUAGAAUUCACUGUGAACAGUCUCAGUCAGUG
AAUUACCGAAGGGCCAUAAACAGAGCAGAGACAGAUCCACGA chr7 129414806 rs41281222 G A 0.000199681 - 49
hsa-mir-183
CCGCAGAGUGUGACUCCUGUUCUGUGUAUGGCACUGGUAGAAUUCACUGUGAACAGUCUCAGUCAGUG
AAUUACCGAAGGGCCAUAAACAGAGCAGAGACAGAUCCACGA chr7 129414804 rs72631833 G U 0.00678914 - 51
hsa-mir-184
CCAGUCACGUCCCCUUAUCACUUUUCCAGCCCAGCUUUGUGACUGUAAGUGUUGGACGGAGAACUGAU
AAGGGUAGGUGAUUGA chr15 79502141 rs145763978 C G 0 + 12
hsa-mir-184
CCAGUCACGUCCCCUUAUCACUUUUCCAGCCCAGCUUUGUGACUGUAAGUGUUGGACGGAGAACUGAU
AAGGGUAGGUGAUUGA chr15 79502168 rs41280052 G U 0.00419329 + 39
hsa-mir-185
AGGGGGCGAGGGAUUGGAGAGAAAGGCAGUUCCUGAUGGUCCCCUCCCCAGGGGCUGGCUUUCCUCUG
GUCCUUCCCUCCCA chr22 20020733 rs201903819 C U 0.000199681 + 72
hsa-mir-187
GGUCGGGCUCACCAUGACACAGUGUGAGACCUCGGGCUACAACACAGGACCCGGGCGCUGCUCUGACC
CCUCGUGUCUUGUGUUGCAGCCGGAGGGACGCAGGUCCGCA chr18 33484837 rs375688661 G A 0.000399361 - 53

hsa-mir-187
GGUCGGGCUCACCAUGACACAGUGUGAGACCUCGGGCUACAACACAGGACCCGGGCGCUGCUCUGACC
CCUCGUGUCUUGUGUUGCAGCCGGAGGGACGCAGGUCCGCA chr18 33484792 rs41274312 C U 0.00579073 - 98
hsa-mir-187
GGUCGGGCUCACCAUGACACAGUGUGAGACCUCGGGCUACAACACAGGACCCGGGCGCUGCUCUGACC
CCUCGUGUCUUGUGUUGCAGCCGGAGGGACGCAGGUCCGCA chr18 33484783 rs114964240 G A 0.00359425 - 107

hsa-mir-188
UGCUCCCUCUCUCACAUCCCUUGCAUGGUGGAGGGUGAGCUUUCUGAAAACCCCUCCCACAUGCAGGG
UUUGCAGGAUGGCGAGCC chrX 49768168 rs191840972 C U 0.000264901 + 60
hsa-mir-188
UGCUCCCUCUCUCACAUCCCUUGCAUGGUGGAGGGUGAGCUUUCUGAAAACCCCUCCCACAUGCAGGG
UUUGCAGGAUGGCGAGCC chrX 49768171 rs375623427 G U 0.000264901 + 63
hsa-mir-18a

UGUUCUAAGGUGCAUCUAGUGCAGAUAGUGAAGUAGAUUAGCAUCUACUGCCCUAAGUGCUCCUUCUG
GCA chr13 92003009 rs41275866 C G null null 5

hsa-mir-1908
CGGGAAUGCCGCGGCGGGGACGGCGAUUGGUCCGUAUGUGUGGUGCCACCGGCCGCCGGCUCCGCCCC
GGCCCCCGCCCC chr11 61582708 rs174561 A G 0.279553 -
5
hsa-mir-1908
CGGGAAUGCCGCGGCGGGGACGGCGAUUGGUCCGUAUGUGUGGUGCCACCGGCCGCCGGCUCCGCCCC
GGCCCCCGCCCC chr11 61582680 . C A 0.000798722 - 33

hsa-mir-1908
CGGGAAUGCCGCGGCGGGGACGGCGAUUGGUCCGUAUGUGUGGUGCCACCGGCCGCCGGCUCCGCCCC
GGCCCCCGCCCC chr11 61582659 . C G 0.000199681 - 54

hsa-mir-1908
CGGGAAUGCCGCGGCGGGGACGGCGAUUGGUCCGUAUGUGUGGUGCCACCGGCCGCCGGCUCCGCCCC
GGCCCCCGCCCC chr11 61582649 . G A 0.000998403 - 64

hsa-mir-1909
CAUCCAGGACAAUGGUGAGUGCCGGUGCCUGCCCUGGGGCCGUCCCUGCGCAGGGGCCGGGUGCUCAC
CGCAUCUGCCCC chr19 1816176 rs199686292 U C 0.00379393 -
62
hsa-mir-1909
CAUCCAGGACAAUGGUGAGUGCCGGUGCCUGCCCUGGGGCCGUCCCUGCGCAGGGGCCGGGUGCUCAC
CGCAUCUGCCCC chr19 1816169 . C A 0.000798722 - 69

hsa-mir-190a
UGCAGGCCUCUGUGUGAUAUGUUUGAUAUAUUAGGUUGUUAUUUAAUCCAACUAUAUAUCAAACAUAU
UCCUACAGUGUCUUGCC chr15 63116226 rs146591219 C U
0.000199681 + 71
hsa-mir-190b
UGCUUCUGUGUGAUAUGUUUGAUAUUGGGUUGUUUAAUUAGGAACCAACUAAAUGUCAAACAUAUUCU
UACAGCAGCAG chr1 154166183 . A G 0.000199681 - 37

hsa-mir-1910
UGUCCCUUCAGCCAGUCCUGUGCCUGCCGCCUUUGUGCUGUCCUUGGAGGGAGGCAGAAGCAGGAUGA
CAAUGAGGGCAA chr16 85775275 rs183226620 U C 0.000199681 -
32
hsa-mir-1910
UGUCCCUUCAGCCAGUCCUGUGCCUGCCGCCUUUGUGCUGUCCUUGGAGGGAGGCAGAAGCAGGAUGA
CAAUGAGGGCAA chr16 85775260 . G C 0.000199681 - 47

hsa-mir-1911
UCGGCAUCUGCUGAGUACCGCCAUGUCUGUUGGGCAUCCACAGUCUCCCACCAGGCAUUGUGGUCUCC
GCUGACGCUUUG chrX 113997746 rs79553157 G A null null 3

hsa-mir-1911
UCGGCAUCUGCUGAGUACCGCCAUGUCUGUUGGGCAUCCACAGUCUCCCACCAGGCAUUGUGGUCUCC
GCUGACGCUUUG chrX 113997768 rs79202905 G A null null 25

hsa-mir-1911
UCGGCAUCUGCUGAGUACCGCCAUGUCUGUUGGGCAUCCACAGUCUCCCACCAGGCAUUGUGGUCUCC
GCUGACGCUUUG chrX 113997812 . G A 0.000529801 + 69

hsa-mir-1911
UCGGCAUCUGCUGAGUACCGCCAUGUCUGUUGGGCAUCCACAGUCUCCCACCAGGCAUUGUGGUCUCC
GCUGACGCUUUG chrX 113997817 . C U 0.000264901 + 74

hsa-mir-1912
CUCUAGGAUGUGCUCAUUGCAUGGGCUGUGUAUAGUAUUAUUCAAUACCCAGAGCAUGCAGUGUGAAC
AUAAUAGAGAUU chrX 113886031 . C U 0.000264901 + 13

hsa-mir-1912
CUCUAGGAUGUGCUCAUUGCAUGGGCUGUGUAUAGUAUUAUUCAAUACCCAGAGCAUGCAGUGUGAAC
AUAAUAGAGAUU chrX 113886051 rs144963985 U C 0.00344371 +
33

hsa-mir-1913
ACCUCUACCUCCCGGCAGAGGAGGCUGCAGAGGCUGGCUUUCCAAAACUCUGCCCCCUCCGCUGCUGC
CAAGUGGCUGGU chr6 166922916 rs188302425 U C 0.000199681 -
6

hsa-mir-1913
ACCUCUACCUCCCGGCAGAGGAGGCUGCAGAGGCUGGCUUUCCAAAACUCUGCCCCCUCCGCUGCUGC
CAAGUGGCUGGU chr6 166922908 . G A 0.000199681 - 14

hsa-mir-1913
ACCUCUACCUCCCGGCAGAGGAGGCUGCAGAGGCUGGCUUUCCAAAACUCUGCCCCCUCCGCUGCUGC
CAAGUGGCUGGU chr6 166922862 rs143896961 C U 0.000798722 -
60

hsa-mir-1914
CGUGUGAGCCCGCCCUGUGCCCGGCCCACUUCUGCUUCCUCUUAGCGCAGGAGGGGUCCCGCACUGGG
AGGGGCCCUCAC chr20 62572885 . C U 0.000199681 - 13

hsa-mir-1914
CGUGUGAGCCCGCCCUGUGCCCGGCCCACUUCUGCUUCCUCUUAGCGCAGGAGGGGUCCCGCACUGGG
AGGGGCCCUCAC chr20 62572874 . G A 0.000798722 - 24

hsa-mir-1914
CGUGUGAGCCCGCCCUGUGCCCGGCCCACUUCUGCUUCCUCUUAGCGCAGGAGGGGUCCCGCACUGGG
AGGGGCCCUCAC chr20 62572847 . G U 0.000199681 - 51

hsa-mir-1915
UGAGAGGCCGCACCUUGCCUUGCUGCCCGGGCCGUGCACCCGUGGGCCCCAGGGCGACGCGGCGGGGG
CGGCCCUAGCGA chr10 21785508 . C G 0.000199681 - 63

hsa-mir-192
GCCGAGACCGAGUGCACAGGGCUCUGACCUAUGAAUUGACAGCCAGUGCUCUCGUCUCCCCUCUGGCU
GCCAAUUCCAUAGGUCACAGGUAUGUUCGCCUCAAUGCCAGC chr11 64658716 . C
A 0.000199681 - 3
hsa-mir-192
GCCGAGACCGAGUGCACAGGGCUCUGACCUAUGAAUUGACAGCCAGUGCUCUCGUCUCCCCUCUGGCU
GCCAAUUCCAUAGGUCACAGGUAUGUUCGCCUCAAUGCCAGC chr11 64658715 . G
A 0.000399361 - 4
hsa-mir-192
GCCGAGACCGAGUGCACAGGGCUCUGACCUAUGAAUUGACAGCCAGUGCUCUCGUCUCCCCUCUGGCU
GCCAAUUCCAUAGGUCACAGGUAUGUUCGCCUCAAUGCCAGC chr11 64658710 . C
U 0.000199681 - 9
hsa-mir-192
GCCGAGACCGAGUGCACAGGGCUCUGACCUAUGAAUUGACAGCCAGUGCUCUCGUCUCCCCUCUGGCU
GCCAAUUCCAUAGGUCACAGGUAUGUUCGCCUCAAUGCCAGC chr11 64658705 . G
A 0.000199681 - 14
hsa-mir-192
GCCGAGACCGAGUGCACAGGGCUCUGACCUAUGAAUUGACAGCCAGUGCUCUCGUCUCCCCUCUGGCU
GCCAAUUCCAUAGGUCACAGGUAUGUUCGCCUCAAUGCCAGC chr11 64658640
rs188630835 U C 0.000998403 - 79

hsa-mir-192
GCCGAGACCGAGUGCACAGGGCUCUGACCUAUGAAUUGACAGCCAGUGCUCUCGUCUCCCCUCUGGCU
GCCAAUUCCAUAGGUCACAGGUAUGUUCGCCUCAAUGCCAGC chr11 64658623
rs139493589 C U 0.000199681 - 96

hsa-mir-193a
CGAGGAUGGGAGCUGAGGGCUGGGUCUUUGCGGGCGAGAUGAGGGUGUCGGAUCAACUGGCCUACAAA
GUCCCAGUUCUCGGCCCCCG chr17 29887033 rs60406007 G U
0.00159744 + 19
hsa-mir-194-1
AUGGUGUUAUCAAGUGUAACAGCAACUCCAUGUGGACUGUGUACCAAUUUCCAGUGGAGAUGCUGUUA
CUUUUGAUGGUUACCAA chr1 220291527 . G C 0.000199681 -
57
hsa-mir-194-1
AUGGUGUUAUCAAGUGUAACAGCAACUCCAUGUGGACUGUGUACCAAUUUCCAGUGGAGAUGCUGUUA
CUUUUGAUGGUUACCAA chr1 220291502 . C U 0.000399361 -
82
hsa-mir-194-2
UGGUUCCCGCCCCCUGUAACAGCAACUCCAUGUGGAAGUGCCCACUGGUUCCAGUGGGGCUGCUGUUA
UCUGGGGCGAGGGCCAG chr11 64658904 . C U 0.000199681 -
8
hsa-mir-194-2
UGGUUCCCGCCCCCUGUAACAGCAACUCCAUGUGGAAGUGCCCACUGGUUCCAGUGGGGCUGCUGUUA
UCUGGGGCGAGGGCCAG chr11 64658836 rs11231898 C U 0.0197684
- 76

hsa-mir-194-2
UGGUUCCCGCCCCCUGUAACAGCAACUCCAUGUGGAAGUGCCCACUGGUUCCAGUGGGGCUGCUGUUA UCUGGGGCGAGGGCCAG chr11 64658828 . A G 0.000199681 - 84
hsa-mir-195
AGCUUCCCUGGCUCUAGCAGCACAGAAAUAUUGGCACAGGGAAGCGAGUCUGCCAAUAUUGGCUGUGC UGCUCCAGGCAGGGUGGUG chr17 6920976 . C U 0.000199681 - 45
hsa-mir-195
AGCUUCCCUGGCUCUAGCAGCACAGAAAUAUUGGCACAGGGAAGCGAGUCUGCCAAUAUUGGCUGUGC UGCUCCAGGCAGGGUGGUG chr17 6920975 . G A 0.000199681 - 46
hsa-mir-195
AGCUUCCCUGGCUCUAGCAGCACAGAAAUAUUGGCACAGGGAAGCGAGUCUGCCAAUAUUGGCUGUGC UGCUCCAGGCAGGGUGGUG chr17 6920955 rs118009022 U G null null 66
hsa-mir-196a-1
GUGAAUUAGGUAGUUUCAUGUUGUUGGGCCUGGGUUUCUGAACACAACAACAUUAAACCACCCGAUUC AC chr17 46709899 rs190478598 G U 0.000399361 - 23

hsa-mir-196a-1
GUGAAUUAGGUAGUUUCAUGUUGUUGGGCCUGGGUUUCUGAACACAACAACAUUAAACCACCCGAUUC AC chr17 46709875 . A G 0.000199681 - 47

hsa-mir-196a-1
GUGAAUUAGGUAGUUUCAUGUUGUUGGGCCUGGGUUUCUGAACACAACAACAUUAAACCACCCGAUUC AC chr17 46709859 rs186449583 C U 0.000199681 - 63

hsa-mir-196a-2
UGCUCGCUCAGCUGAUCUGUGGCUUAGGUAGUUUCAUGUUGUUGGGAUUGAGUUUUGAACUCGGCAAC AAGAAACUGCCUGAGUUACAUCAGUCGGUUUUCGUCGAGGGC chr12 54385558 rs185070757 U G 0.000199681 + 37

hsa-mir-196a-2
UGCUCGCUCAGCUGAUCUGUGGCUUAGGUAGUUUCAUGUUGUUGGGAUUGAGUUUUGAACUCGGCAAC AAGAAACUGCCUGAGUUACAUCAGUCGGUUUUCGUCGAGGGC chr12 54385561 . U C 0.000199681 + 40
hsa-mir-196a-2
UGCUCGCUCAGCUGAUCUGUGGCUUAGGUAGUUUCAUGUUGUUGGGAUUGAGUUUUGAACUCGGCAAC AAGAAACUGCCUGAGUUACAUCAGUCGGUUUUCGUCGAGGGC chr12 54385584 . G A 0.000199681 + 63
hsa-mir-196a-2
UGCUCGCUCAGCUGAUCUGUGGCUUAGGUAGUUUCAUGUUGUUGGGAUUGAGUUUUGAACUCGGCAAC AAGAAACUGCCUGAGUUACAUCAGUCGGUUUUCGUCGAGGGC chr12 54385599 rs11614913 C U 0.332668 + 78
hsa-mir-196a-2
UGCUCGCUCAGCUGAUCUGUGGCUUAGGUAGUUUCAUGUUGUUGGGAUUGAGUUUUGAACUCGGCAAC

AAGAAACUGCCUGAGUUACAUCAGUCGGUUUUCGUCGAGGGC chr12 54385629 . G U 0.000199681 + 108

hsa-mir-196b ACUGGUCGGUGAUUUAGGUAGUUUCCUGUUGUUGGGAUCCACCUUUCUCUCGACAGCACGACACUGCCUUCAUUACUUCAGUUG chr7 27209165 . G A 0.000199681 - 18

hsa-mir-197 GGCUGUGCCGGGUAGAGAGGGCAGUGGGAGGUAAGAGCUCUUCACCCUUCACCACCUUCUCCACCCAGCAUGGCC chr1 110141523 rs371126356 C U 0.000199681 + 9

hsa-mir-197 GGCUGUGCCGGGUAGAGAGGGCAGUGGGAGGUAAGAGCUCUUCACCCUUCACCACCUUCUCCACCCAGCAUGGCC chr1 110141578 rs142872331 C U 0.000199681 + 64

hsa-mir-1972-2 UAUAGGCAUGUGCCACCACACCUGGCUUAAAUGUGUCAUUUAAAAAUUCAGGCCAGGCACAGUGGCUCAUGCCUGUA chr16 70064261 rs57629257 C U 0.204872 + 13

hsa-mir-1972-2 UAUAGGCAUGUGCCACCACACCUGGCUUAAAUGUGUCAUUUAAAAAUUCAGGCCAGGCACAGUGGCUCAUGCCUGUA chr16 70064272 . G U 0.000199681 + 24

hsa-mir-1972-2 UAUAGGCAUGUGCCACCACACCUGGCUUAAAUGUGUCAUUUAAAAAUUCAGGCCAGGCACAGUGGCUCAUGCCUGUA chr16 70064324 rs141946713 U C 0.000199681 + 76

hsa-mir-1973 UAUGUUCAACGGCCAUGGUAUCCUGACCGUGCAAAGGUAGCAUA chr4 117220891 . G A 0.000199681 + 11

hsa-mir-1973 UAUGUUCAACGGCCAUGGUAUCCUGACCGUGCAAAGGUAGCAUA chr4 117220909 rs183399490 G A 0.000399361 + 29

hsa-mir-198 UCAUUGGUCCAGAGGGGAGAUAGGUUCCUGUGAUUUUUCCUUCUUCUCUAUAGAAUAAAUGA chr3 120114558 rs142303836 G A 0.000399361 - 19

hsa-mir-199a-1 GCCAACCCAGUGUUCAGACUACCUGUUCAGGAGGCUCUCAAUGUGUACAGUAGUCUGCACAUUGGUUAGGC chr19 10928149 . U A 0.000199681 - 24

hsa-mir-199a-1 GCCAACCCAGUGUUCAGACUACCUGUUCAGGAGGCUCUCAAUGUGUACAGUAGUCUGCACAUUGGUUAGGC chr19 10928130 . G A 0.000199681 - 43

hsa-mir-199a-1 GCCAACCCAGUGUUCAGACUACCUGUUCAGGAGGCUCUCAAUGUGUACAGUAGUCUGCACAUUGGUUAGGC chr19 10928119 rs183664554 U C 0.0061901 - 54

hsa-mir-199a-2
AGGAAGCUUCUGGAGAUCCUGCUCCGUCGCCCCAGUGUUCAGACUACCUGUUCAGGACAAUGCCGUUG UACAGUAGUCUGCACAUUGGUUAGACUGGGCAAGGGAGAGCA chr1 172113756
rs147011290 G A 0.00119808 - 29

hsa-mir-199b
CCAGAGGACACCUCCACUCCGUCUACCCAGUGUUUAGACUAUCUGUUCAGGACUCCCAAAUUGUACAG UAGUCUGCACAUUGGUUAGGCUGGGCUGGGUUAGACCCUCGG chr9 131007109 rs72631835
C U 0.000199681 - 1

hsa-mir-199b
CCAGAGGACACCUCCACUCCGUCUACCCAGUGUUUAGACUAUCUGUUCAGGACUCCCAAAUUGUACAG UAGUCUGCACAUUGGUUAGGCUGGGCUGGGUUAGACCCUCGG chr9 131007087 . C
G 0.000199681 - 23

hsa-mir-199b
CCAGAGGACACCUCCACUCCGUCUACCCAGUGUUUAGACUAUCUGUUCAGGACUCCCAAAUUGUACAG UAGUCUGCACAUUGGUUAGGCUGGGCUGGGUUAGACCCUCGG chr9 131007052 . A
U 0.000599042 - 58

hsa-mir-199b
CCAGAGGACACCUCCACUCCGUCUACCCAGUGUUUAGACUAUCUGUUCAGGACUCCCAAAUUGUACAG UAGUCUGCACAUUGGUUAGGCUGGGCUGGGUUAGACCCUCGG chr9 131007004 . C
A 0.000199681 - 106

hsa-mir-199b
CCAGAGGACACCUCCACUCCGUCUACCCAGUGUUUAGACUAUCUGUUCAGGACUCCCAAAUUGUACAG UAGUCUGCACAUUGGUUAGGCUGGGCUGGGUUAGACCCUCGG chr9 131007001
rs146892675 G A 0.00299521 - 109

hsa-mir-19b-2
ACAUUGCUACUUACAAUUAGUUUUGCAGGUUUGCAUUUCAGCGUAUAUAUGUAUAUGUGGCUGUGCAA AUCCAUGCAAAACUGAUUGUGAUAAUGU chrX 133303758 rs193073651 C U
0.000264901 - 39

hsa-mir-19b-2
ACAUUGCUACUUACAAUUAGUUUUGCAGGUUUGCAUUUCAGCGUAUAUAUGUAUAUGUGGCUGUGCAA AUCCAUGCAAAACUGAUUGUGAUAAUGU chrX 133303744 . A G
0.000264901 - 53

hsa-mir-200a
CCGGGCCCCUGUGAGCAUCUUACCGGACAGUGCUGGAUUUCCCAGCUUGACUCUAACACUGUCUGGUA ACGAUGUUCAAAGGUGACCCGC chr1 1103284 rs202051309 C U
0.00139776 + 42

hsa-mir-200a
CCGGGCCCCUGUGAGCAUCUUACCGGACAGUGCUGGAUUUCCCAGCUUGACUCUAACACUGUCUGGUA ACGAUGUUCAAAGGUGACCCGC chr1 1103312 rs376377337 C U
0.000399361 + 70

hsa-mir-200a
CCGGGCCCCUGUGAGCAUCUUACCGGACAGUGCUGGAUUUCCCAGCUUGACUCUAACACUGUCUGGUA ACGAUGUUCAAAGGUGACCCGC chr1 1103328 . C A
0.000199681 + 86

hsa-mir-200a

CCGGGCCCCUGUGAGCAUCUUACCGGACAGUGCUGGAUUUCCCAGCUUGACUCUAACACUGUCUGGUA
ACGAUGUUCAAAGGUGACCCGC chr1 1103331 . G A
0.000599042 + 89
hsa-mir-200b
CCAGCUCGGGCAGCCGUGGCCAUCUUACUGGGCAGCAUUGGAUGGAGUCAGGUCUCUAAUACUGCCUG
GUAAUGAUGACGGCGGAGCCCUGCACG chr1 1102498 . C U
0.000599042 + 15
hsa-mir-200b
CCAGCUCGGGCAGCCGUGGCCAUCUUACUGGGCAGCAUUGGAUGGAGUCAGGUCUCUAAUACUGCCUG
GUAAUGAUGACGGCGGAGCCCUGCACG chr1 1102501 . G U
0.000399361 + 18
hsa-mir-200b
CCAGCUCGGGCAGCCGUGGCCAUCUUACUGGGCAGCAUUGGAUGGAGUCAGGUCUCUAAUACUGCCUG
GUAAUGAUGACGGCGGAGCCCUGCACG chr1 1102563 rs72563729 G A
0.0081869 + 80
hsa-mir-200b
CCAGCUCGGGCAGCCGUGGCCAUCUUACUGGGCAGCAUUGGAUGGAGUCAGGUCUCUAAUACUGCCUG
GUAAUGAUGACGGCGGAGCCCUGCACG chr1 1102567 . G A
0.000199681 + 84
hsa-mir-202
CGCCUCAGAGCCGCCCGCCGUUCCUUUUUCCUAUGCAUAUACUUCUUUGAGGAUCUGGCCUAAAGAGG
UAUAGGGCAUGGGAAAACGGGGCGGUCGGGUCCUCCCCAGCG chr10 135061124 . C
U 0.000199681 - 1
hsa-mir-202
CGCCUCAGAGCCGCCCGCCGUUCCUUUUUCCUAUGCAUAUACUUCUUUGAGGAUCUGGCCUAAAGAGG
UAUAGGGCAUGGGAAAACGGGGCGGUCGGGUCCUCCCCAGCG chr10 135061112 rs12355840
G A null null 13
hsa-mir-202
CGCCUCAGAGCCGCCCGCCGUUCCUUUUUCCUAUGCAUAUACUUCUUUGAGGAUCUGGCCUAAAGAGG
UAUAGGGCAUGGGAAAACGGGGCGGUCGGGUCCUCCCCAGCG chr10 135061111 . C
G 0.000199681 - 14
hsa-mir-202
CGCCUCAGAGCCGCCCGCCGUUCCUUUUUCCUAUGCAUAUACUUCUUUGAGGAUCUGGCCUAAAGAGG
UAUAGGGCAUGGGAAAACGGGGCGGUCGGGUCCUCCCCAGCG chr10 135061109
rs142676430 C U 0.000199681 - 16

hsa-mir-202
CGCCUCAGAGCCGCCCGCCGUUCCUUUUUCCUAUGCAUAUACUUCUUUGAGGAUCUGGCCUAAAGAGG
UAUAGGGCAUGGGAAAACGGGGCGGUCGGGUCCUCCCCAGCG chr10 135061106
rs368130172 C U 0.00199681 - 19

hsa-mir-202
CGCCUCAGAGCCGCCCGCCGUUCCUUUUUCCUAUGCAUAUACUUCUUUGAGGAUCUGGCCUAAAGAGG
UAUAGGGCAUGGGAAAACGGGGCGGUCGGGUCCUCCCCAGCG chr10 135061039
rs375116255 C U 0.000399361 - 86

hsa-mir-203a

GUGUUGGGGACUCGCGCGCUGGGUCCAGUGGUUCUUAACAGUUCAACAGUUCUGUAGCGCAAUUGUGA AAUGUUUAGGACCACUAGACCCGGCGGGCGCGGCGACAGCGA chr14 104583759 rs377122462 G U 0.000199681 + 18

hsa-mir-203a
GUGUUGGGGACUCGCGCGCUGGGUCCAGUGGUUCUUAACAGUUCAACAGUUCUGUAGCGCAAUUGUGA AAUGUUUAGGACCACUAGACCCGGCGGGCGCGGCGACAGCGA chr14 104583776 . U C 0.000199681 + 35

hsa-mir-203a
GUGUUGGGGACUCGCGCGCUGGGUCCAGUGGUUCUUAACAGUUCAACAGUUCUGUAGCGCAAUUGUGA AAUGUUUAGGACCACUAGACCCGGCGGGCGCGGCGACAGCGA chr14 104583796 . U C 0.000199681 + 55

hsa-mir-203a
GUGUUGGGGACUCGCGCGCUGGGUCCAGUGGUUCUUAACAGUUCAACAGUUCUGUAGCGCAAUUGUGA AAUGUUUAGGACCACUAGACCCGGCGGGCGCGGCGACAGCGA chr14 104583804 . U C 0.000199681 + 63

hsa-mir-203a
GUGUUGGGGACUCGCGCGCUGGGUCCAGUGGUUCUUAACAGUUCAACAGUUCUGUAGCGCAAUUGUGA AAUGUUUAGGACCACUAGACCCGGCGGGCGCGGCGACAGCGA chr14 104583828 rs374712314 A G 0.000199681 + 87

hsa-mir-203a
GUGUUGGGGACUCGCGCGCUGGGUCCAGUGGUUCUUAACAGUUCAACAGUUCUGUAGCGCAAUUGUGA AAUGUUUAGGACCACUAGACCCGGCGGGCGCGGCGACAGCGA chr14 104583838 . C G 0.000599042 + 97

hsa-mir-203b
GCGCCCGCCGGGUCUAGUGGUCCUAAACAUUUCACAAUUGCGCUACAGAACUGUUGAACUGUUAAGAA CCACUGGACCCAGCGCGC chr14 104583838 . G C 0.000599042 - 3

hsa-mir-203b
GCGCCCGCCGGGUCUAGUGGUCCUAAACAUUUCACAAUUGCGCUACAGAACUGUUGAACUGUUAAGAA CCACUGGACCCAGCGCGC chr14 104583828 rs374712314 U C 0.000199681 - 13

hsa-mir-203b
GCGCCCGCCGGGUCUAGUGGUCCUAAACAUUUCACAAUUGCGCUACAGAACUGUUGAACUGUUAAGAA CCACUGGACCCAGCGCGC chr14 104583804 . A G 0.000199681 - 37

hsa-mir-203b
GCGCCCGCCGGGUCUAGUGGUCCUAAACAUUUCACAAUUGCGCUACAGAACUGUUGAACUGUUAAGAA CCACUGGACCCAGCGCGC chr14 104583796 . A G 0.000199681 - 45

hsa-mir-203b
GCGCCCGCCGGGUCUAGUGGUCCUAAACAUUUCACAAUUGCGCUACAGAACUGUUGAACUGUUAAGAA CCACUGGACCCAGCGCGC chr14 104583776 . A G 0.000199681 - 65

hsa-mir-203b
GCGCCCGCCGGGUCUAGUGGUCCUAAACAUUUCACAAUUGCGCUACAGAACUGUUGAACUGUUAAGAA

CCACUGGACCCAGCGCGC chr14 104583759 rs377122462 C A
0.000199681 - 82
hsa-mir-204
GGCUACAGUCUUUCUUCAUGUGACUCGUGGACUUCCCUUUGUCAUCCUAUGCCUGAGAAUAUAUGAAG
GAGGCUGGGAAGGCAAAGGGACGUUCAAUUGUCAUCACUGGC chr9 73424994
rs201632779 A C 0.000199681 - 7

hsa-mir-204
GGCUACAGUCUUUCUUCAUGUGACUCGUGGACUUCCCUUUGUCAUCCUAUGCCUGAGAAUAUAUGAAG
GAGGCUGGGAAGGCAAAGGGACGUUCAAUUGUCAUCACUGGC chr9 73424988
rs112971695 U C null null 13

hsa-mir-204
GGCUACAGUCUUUCUUCAUGUGACUCGUGGACUUCCCUUUGUCAUCCUAUGCCUGAGAAUAUAUGAAG
GAGGCUGGGAAGGCAAAGGGACGUUCAAUUGUCAUCACUGGC chr9 73424983 . A
G 0.000399361 - 18
hsa-mir-204
GGCUACAGUCUUUCUUCAUGUGACUCGUGGACUUCCCUUUGUCAUCCUAUGCCUGAGAAUAUAUGAAG
GAGGCUGGGAAGGCAAAGGGACGUUCAAUUGUCAUCACUGGC chr9 73424956
rs112062096 U C null null 45

hsa-mir-204
GGCUACAGUCUUUCUUCAUGUGACUCGUGGACUUCCCUUUGUCAUCCUAUGCCUGAGAAUAUAUGAAG
GAGGCUGGGAAGGCAAAGGGACGUUCAAUUGUCAUCACUGGC chr9 73424914 . G
C 0.000199681 - 87
hsa-mir-204
GGCUACAGUCUUUCUUCAUGUGACUCGUGGACUUCCCUUUGUCAUCCUAUGCCUGAGAAUAUAUGAAG
GAGGCUGGGAAGGCAAAGGGACGUUCAAUUGUCAUCACUGGC chr9 73424910
rs374947690 G A 0.000599042 - 91

hsa-mir-205
AAAGAUCCUCAGACAAUCCAUGUGCUUCUCUUGUCCUUCAUUCCACCGGAGUCUGUCUCAUACCCAAC
CAGAUUUCAGUGGAGUGAAGUUCAGGAGGCAUGGAGCUGACA chr1 209605507 . C
U 0.000399361 + 30
hsa-mir-205
AAAGAUCCUCAGACAAUCCAUGUGCUUCUCUUGUCCUUCAUUCCACCGGAGUCUGUCUCAUACCCAAC
CAGAUUUCAGUGGAGUGAAGUUCAGGAGGCAUGGAGCUGACA chr1 209605539
rs149492792 A U 0.000399361 + 62

hsa-mir-205
AAAGAUCCUCAGACAAUCCAUGUGCUUCUCUUGUCCUUCAUUCCACCGGAGUCUGUCUCAUACCCAAC
CAGAUUUCAGUGGAGUGAAGUUCAGGAGGCAUGGAGCUGACA chr1 209605546
rs113859371 C U null null 69

hsa-mir-2053
CUUGCCAUGUAAAUACAGAUUUAAUUAACAUUUGCAACCUGUGAAGAUGCAAAACUUUAAGUGUUAAU
UAAACCUCUAUUUACAUAGCAAG chr8 113655748 . A U

0.000199681 + 27
hsa-mir-2053
CUUGCCAUGUAAAUACAGAUUUAAUUAACAUUUGCAACCUGUGAAGAUGCAAAACUUUAAGUGUUAAU
UAAACCUCUAUUUACAUAGCAAG chr8 113655752 rs10505168 U C
0.386781 + 31
hsa-mir-2053
CUUGCCAUGUAAAUACAGAUUUAAUUAACAUUUGCAACCUGUGAAGAUGCAAAACUUUAAGUGUUAAU
UAAACCUCUAUUUACAUAGCAAG chr8 113655794 . C U
0.000199681 + 73
hsa-mir-2053
CUUGCCAUGUAAAUACAGAUUUAAUUAACAUUUGCAACCUGUGAAGAUGCAAAACUUUAAGUGUUAAU
UAAACCUCUAUUUACAUAGCAAG chr8 113655806 . U A 0.00139776
+ 85
hsa-mir-206
UGCUUCCCGAGGCCACAUGCUUCUUUAUAUCCCCAUAUGGAUUACUUUGCUAUGGAAUGUAAGGAAGU
GUGUGGUUUCGGCAAGUG chr6 52009178 rs374829386 C U
0.000599042 + 32
hsa-mir-206
UGCUUCCCGAGGCCACAUGCUUCUUUAUAUCCCCAUAUGGAUUACUUUGCUAUGGAAUGUAAGGAAGU
GUGUGGUUUCGGCAAGUG chr6 52009220 . G U 0.000199681 +
74
hsa-mir-208b
CCUCUCAGGGAAGCUUUUUGCUCGAAUUAUGUUUCUGAUCCGAAUAUAAGACGAACAAAAGGUUUGUC
UGAGGGCAG chr14 23887271 . C U 0.000599042 - 2

hsa-mir-208b
CCUCUCAGGGAAGCUUUUUGCUCGAAUUAUGUUUCUGAUCCGAAUAUAAGACGAACAAAAGGUUUGUC
UGAGGGCAG chr14 23887220 . G A 0.000199681 - 53

hsa-mir-208b
CCUCUCAGGGAAGCUUUUUGCUCGAAUUAUGUUUCUGAUCCGAAUAUAAGACGAACAAAAGGUUUGUC
UGAGGGCAG chr14 23887219 rs2754157 A U null null 54

hsa-mir-20a
GUAGCACUAAAGUGCUUAUAGUGCAGGUAGUGUUUAGUUAUCUACUGCAUUAUGAGCACUUAAAGUAC
UGC chr13 92003356 rs185831554 U G 0.000199681 + 38

hsa-mir-20b
AGUACCAAAGUGCUCAUAGUGCAGGUAGUUUUGGCAUGACUCUACUGUAGUAUGGGCACUUCCAGUAC
U chrX 133303896 . G A 0.000529801 - 12

hsa-mir-21
UGUCGGGUAGCUUAUCAGACUGAUGUUGACUGUUGAAUCUCAUGGCAACACCAGUCGAUGGGCUGUCU
GACA chr17 57918678 . C U 0.000199681 + 52

hsa-mir-210
ACCCGGCAGUGCCUCCAGGCGCAGGGCAGCCCCUGCCCACCGCACACUGCGCUGCCCCAGACCCACUG

UGCGUGUGACAGCGGCUGAUCUGUGCCUGGGCAGCGCGACCC chr11 568187 . C U
0.000399361 - 12
hsa-mir-210
ACCCGGCAGUGCCUCCAGGCGCAGGGCAGCCCCUGCCCACCGCACACUGCGCUGCCCCAGACCCACUG
UGCGUGUGACAGCGGCUGAUCUGUGCCUGGGCAGCGCGACCC chr11 568176 . A G
0.000199681 - 23
hsa-mir-210
ACCCGGCAGUGCCUCCAGGCGCAGGGCAGCCCCUGCCCACCGCACACUGCGCUGCCCCAGACCCACUG
UGCGUGUGACAGCGGCUGAUCUGUGCCUGGGCAGCGCGACCC chr11 568125 . G A
0.000399361 - 74
hsa-mir-210
ACCCGGCAGUGCCUCCAGGCGCAGGGCAGCCCCUGCCCACCGCACACUGCGCUGCCCCAGACCCACUG
UGCGUGUGACAGCGGCUGAUCUGUGCCUGGGCAGCGCGACCC chr11 568106 . G C
0.000199681 - 93
hsa-mir-211
UCACCUGGCCAUGUGACUUGUGGGCUUCCCUUUGUCAUCCUUCGCCUAGGGCUCUGAGCAGGGCAGGG
ACAGCAAAGGGGUGCUCAGUUGUCACUUCCCACAGCACGGAG chr15 31357325
rs141424579 G A 0.000199681 - 20

hsa-mir-211
UCACCUGGCCAUGUGACUUGUGGGCUUCCCUUUGUCAUCCUUCGCCUAGGGCUCUGAGCAGGGCAGGG
ACAGCAAAGGGGUGCUCAGUUGUCACUUCCCACAGCACGGAG chr15 31357301
rs187960998 G A 0.000199681 - 44

hsa-mir-211
UCACCUGGCCAUGUGACUUGUGGGCUUCCCUUUGUCAUCCUUCGCCUAGGGCUCUGAGCAGGGCAGGG
ACAGCAAAGGGGUGCUCAGUUGUCACUUCCCACAGCACGGAG chr15 31357245 rs34520022
- C null null 100
hsa-mir-211
UCACCUGGCCAUGUGACUUGUGGGCUUCCCUUUGUCAUCCUUCGCCUAGGGCUCUGAGCAGGGCAGGG
ACAGCAAAGGGGUGCUCAGUUGUCACUUCCCACAGCACGGAG chr15 31357244 . C
U 0.000199681 - 101
hsa-mir-211
UCACCUGGCCAUGUGACUUGUGGGCUUCCCUUUGUCAUCCUUCGCCUAGGGCUCUGAGCAGGGCAGGG
ACAGCAAAGGGGUGCUCAGUUGUCACUUCCCACAGCACGGAG chr15 31357238
rs140017415 G A 0.00239617 - 107

hsa-mir-2110
CAGGGGUUUGGGGAAACGGCCGCUGAGUGAGGCGUCGGCUGUGUUUCUCACCGCGGUCUUUUCCUCCC
ACUCUUG chr10 115933922 rs114530801 C U null null 17

hsa-mir-2110
CAGGGGUUUGGGGAAACGGCCGCUGAGUGAGGCGUCGGCUGUGUUUCUCACCGCGGUCUUUUCCUCCC
ACUCUUG chr10 115933918 . C G 0.000199681 - 21

hsa-mir-2110
CAGGGGUUUGGGGAAACGGCCGCUGAGUGAGGCGUCGGCUGUGUUUCUCACCGCGGUCUUUUCCUCCC

ACUCUUG chr10 115933913 rs192664871 A C 0.000199681 - 26
hsa-mir-2110 CAGGGGUUUGGGGAAACGGCCGCUGAGUGAGGCGUCGGCUGUGUUUCUCACCGCGGUCUUUUCCUCCC ACUCUUG chr10 115933905 rs17091403 G A 0.043131 - 34
hsa-mir-2110 CAGGGGUUUGGGGAAACGGCCGCUGAGUGAGGCGUCGGCUGUGUUUCUCACCGCGGUCUUUUCCUCCC ACUCUUG chr10 115933890 . C U 0.000399361 - 49

hsa-mir-2110 CAGGGGUUUGGGGAAACGGCCGCUGAGUGAGGCGUCGGCUGUGUUUCUCACCGCGGUCUUUUCCUCCC ACUCUUG chr10 115933876 rs75407574 C U 0.00599042 - 63
hsa-mir-2113 UUUUCAAAGCAAUGUGUGACAGGUACAGGGACAAAUCCCGUUAAUAAGUAAGAGGAUUUGUGCUUGGC UCUGUCACAUGCCACUUUGAAAA chr6 98472426 . C A 0.000199681 + 20
hsa-mir-2113 UUUUCAAAGCAAUGUGUGACAGGUACAGGGACAAAUCCCGUUAAUAAGUAAGAGGAUUUGUGCUUGGC UCUGUCACAUGCCACUUUGAAAA chr6 98472445 rs117428639 C U 0.00259585 + 39
hsa-mir-2113 UUUUCAAAGCAAUGUGUGACAGGUACAGGGACAAAUCCCGUUAAUAAGUAAGAGGAUUUGUGCUUGGC UCUGUCACAUGCCACUUUGAAAA chr6 98472446 rs142927919 G A 0.000599042 + 40
hsa-mir-2114 CCUCCAUGCUCCUAGUCCCUUCCUUGAAGCGGUCGGAUAAUCACAUGACGAGCCUCAAGCAAGGGACU UCAAGCUGGUGG chrX 149396242 rs114424495 C G 0.0145695 + 4
hsa-mir-2114 CCUCCAUGCUCCUAGUCCCUUCCUUGAAGCGGUCGGAUAAUCACAUGACGAGCCUCAAGCAAGGGACU UCAAGCUGGUGG chrX 149396272 . C U 0.000264901 + 34

hsa-mir-2114 CCUCCAUGCUCCUAGUCCCUUCCUUGAAGCGGUCGGAUAAUCACAUGACGAGCCUCAAGCAAGGGACU UCAAGCUGGUGG chrX 149396273 rs188839575 G A 0.00476821 + 35
hsa-mir-2114 CCUCCAUGCUCCUAGUCCCUUCCUUGAAGCGGUCGGAUAAUCACAUGACGAGCCUCAAGCAAGGGACU UCAAGCUGGUGG chrX 149396288 . G A 0.000264901 + 50

hsa-mir-2115 ACUGUCAUCCCACUGCUUCCAGCUUCCAUGACUCCUGAUGGAGGAAUCACAUGAAUUCAUCAGAAUUC AUGGAGGCUAGAAGCAGUAUGAGGAUCAUUUA chr3 48357921 rs372327506 U C 0.000199681 - 29
hsa-mir-2115

ACUGUCAUCCCACUGCUUCCAGCUUCCAUGACUCCUGAUGGAGGAAUCACAUGAAUUCAUCAGAAUUC AUGGAGGCUAGAAGCAGUAUGAGGAUCAUUUA chr3 48357864 . U C 0.000199681 - 86
hsa-mir-2115
ACUGUCAUCCCACUGCUUCCAGCUUCCAUGACUCCUGAUGGAGGAAUCACAUGAAUUCAUCAGAAUUC AUGGAGGCUAGAAGCAGUAUGAGGAUCAUUUA chr3 48357853 . U C 0.000199681 - 97
hsa-mir-2116
GACCUAGGCUAGGGGUUCUUAGCAUAGGAGGUCUUCCCAUGCUAAGAAGUCCUCCCAUGCCAAGAACU CCCAGACUAGGA chr15 59463460 rs149569724 A C 0.00119808 - 2
hsa-mir-2116
GACCUAGGCUAGGGGUUCUUAGCAUAGGAGGUCUUCCCAUGCUAAGAAGUCCUCCCAUGCCAAGAACU CCCAGACUAGGA chr15 59463451 rs3838847 A - null null 11

hsa-mir-2116
GACCUAGGCUAGGGGUUCUUAGCAUAGGAGGUCUUCCCAUGCUAAGAAGUCCUCCCAUGCCAAGAACU CCCAGACUAGGA chr15 59463436 . A G 0.000399361 - 26

hsa-mir-2116
GACCUAGGCUAGGGGUUCUUAGCAUAGGAGGUCUUCCCAUGCUAAGAAGUCCUCCCAUGCCAAGAACU CCCAGACUAGGA chr15 59463389 . G A 0.000599042 - 73

hsa-mir-2117
GCUCUGAUUUACUUCUGUCCGGCAUGGUGAACAGCAGGAUUGGCUGUAGCUGUUCUCUUUGCCAAGGA CAGAUCUGAUCU chr17 41522207 rs186154593 G A 0.000199681 + 34
hsa-mir-2117
GCUCUGAUUUACUUCUGUCCGGCAUGGUGAACAGCAGGAUUGGCUGUAGCUGUUCUCUUUGCCAAGGA CAGAUCUGAUCU chr17 41522213 rs7207008 U A 0.402756 + 40
hsa-mir-2117
GCUCUGAUUUACUUCUGUCCGGCAUGGUGAACAGCAGGAUUGGCUGUAGCUGUUCUCUUUGCCAAGGA CAGAUCUGAUCU chr17 41522221 rs76511826 A G null null 48

hsa-mir-212
CGGGGCACCCCGCCCGGACAGCGCGCCGGCACCUUGGCUCUAGACUGCUUACUGCCCGGGCCGCCCUC AGUAACAGUCUCCAGUCACGGCCACCGACGCCUGGCCCCGCC chr17 1953674 . C A 0.000199681 - 1
hsa-mir-212
CGGGGCACCCCGCCCGGACAGCGCGCCGGCACCUUGGCUCUAGACUGCUUACUGCCCGGGCCGCCCUC AGUAACAGUCUCCAGUCACGGCCACCGACGCCUGGCCCCGCC chr17 1953666 . C U 0.00199681 - 9
hsa-mir-212
CGGGGCACCCCGCCCGGACAGCGCGCCGGCACCUUGGCUCUAGACUGCUUACUGCCCGGGCCGCCCUC AGUAACAGUCUCCAGUCACGGCCACCGACGCCUGGCCCCGCC chr17 1953637 . C U 0.00199681 - 38

hsa-mir-212
CGGGGCACCCCGCCCGGACAGCGCGCCGGCACCUUGGCUCUAGACUGCUUACUGCCCGGGCCGCCCUC AGUAACAGUCUCCAGUCACGGCCACCGACGCCUGGCCCCGCC chr17 1953635 . C U 0.00279553 - 40
hsa-mir-215
AUCAUUCAGAAAUGGUAUACAGGAAAAUGACCUAUGAAUUGACAGACAAUAUAGCUGAGUUUGUCUGU CAUUUCUUUAGGCCAAUAUUCUGUAUGACUGUGCUACUUCAA chr1 220291302 rs145979015 C U 0.000798722 - 3

hsa-mir-215
AUCAUUCAGAAAUGGUAUACAGGAAAAUGACCUAUGAAUUGACAGACAAUAUAGCUGAGUUUGUCUGU CAUUUCUUUAGGCCAAUAUUCUGUAUGACUGUGCUACUUCAA chr1 220291292 rs72631834 U C 0.000599042 - 13
hsa-mir-215
AUCAUUCAGAAAUGGUAUACAGGAAAAUGACCUAUGAAUUGACAGACAAUAUAGCUGAGUUUGUCUGU CAUUUCUUUAGGCCAAUAUUCUGUAUGACUGUGCUACUUCAA chr1 220291206 rs189877545 G U 0.000399361 - 99

hsa-mir-216a
GAUGGCUGUGAGUUGGCUUAAUCUCAGCUGGCAACUGUGAGAUGUUCAUACAAUCCCUCACAGUGGUC UCUGGGAUUAUGCUAAACAGAGCAAUUUCCUAGCCCUCACGA chr2 56216187 . G A 0.000199681 - 8
hsa-mir-216a
GAUGGCUGUGAGUUGGCUUAAUCUCAGCUGGCAACUGUGAGAUGUUCAUACAAUCCCUCACAGUGGUC UCUGGGAUUAUGCUAAACAGAGCAAUUUCCUAGCCCUCACGA chr2 56216156 rs370221981 G C 0.000199681 - 39

hsa-mir-216a
GAUGGCUGUGAGUUGGCUUAAUCUCAGCUGGCAACUGUGAGAUGUUCAUACAAUCCCUCACAGUGGUC UCUGGGAUUAUGCUAAACAGAGCAAUUUCCUAGCCCUCACGA chr2 56216092 . C U 0.000199681 - 103
hsa-mir-216a
GAUGGCUGUGAGUUGGCUUAAUCUCAGCUGGCAACUGUGAGAUGUUCAUACAAUCCCUCACAGUGGUC UCUGGGAUUAUGCUAAACAGAGCAAUUUCCUAGCCCUCACGA chr2 56216090 rs41291179 U A 0.0842652 - 105
hsa-mir-216b
GCAGACUGGAAAAUCUCUGCAGGCAAAUGUGAUGUCACUGAGGAAAUCACACACUUACCCGUAGAGAU UCUACAGUCUGACA chr2 56227910 . A G 0.000199681 - 21
hsa-mir-216b
GCAGACUGGAAAAUCUCUGCAGGCAAAUGUGAUGUCACUGAGGAAAUCACACACUUACCCGUAGAGAU UCUACAGUCUGACA chr2 56227905 . A C 0.000599042 - 26
hsa-mir-217
AGUAUAAUUAUUACAUAGUUUUUGAUGUCGCAGAUACUGCAUCAGGAACUGAUUGGAUAAGAAUCAGU CACCAUCAGUUCCUAAUGCAUUGCCUUCAGCAUCUAAACAAG chr2 56210210 . G C 0.000399361 - 2

hsa-mir-217
AGUAUAAUUAUUACAUAGUUUUUGAUGUCGCAGAUACUGCAUCAGGAACUGAUUGGAUAAGAAUCAGU
CACCAUCAGUUCCUAAUGCAUUGCCUUCAGCAUCUAAACAAG chr2 56210207
rs150436368 U C 0.0147764 - 5

hsa-mir-217
AGUAUAAUUAUUACAUAGUUUUUGAUGUCGCAGAUACUGCAUCAGGAACUGAUUGGAUAAGAAUCAGU
CACCAUCAGUUCCUAAUGCAUUGCCUUCAGCAUCUAAACAAG chr2 56210168
rs140509279 A G 0.000998403 - 44

hsa-mir-217
AGUAUAAUUAUUACAUAGUUUUUGAUGUCGCAGAUACUGCAUCAGGAACUGAUUGGAUAAGAAUCAGU
CACCAUCAGUUCCUAAUGCAUUGCCUUCAGCAUCUAAACAAG chr2 56210140 rs41291173
C U 0.00638978 - 72

hsa-mir-217
AGUAUAAUUAUUACAUAGUUUUUGAUGUCGCAGAUACUGCAUCAGGAACUGAUUGGAUAAGAAUCAGU
CACCAUCAGUUCCUAAUGCAUUGCCUUCAGCAUCUAAACAAG chr2 56210117 . U
G 0.000199681 - 95

hsa-mir-218-1
GUGAUAAUGUAGCGAGAUUUUCUGUUGUGCUUGAUCUAACCAUGUGGUUGCGAGGUAUGAGUAAAACA
UGGUUCCGUCAAGCACCAUGGAACGUCACGCAGCUUUCUACA chr4 20529956 . G
U 0.000199681 + 59

hsa-mir-218-1
GUGAUAAUGUAGCGAGAUUUUCUGUUGUGCUUGAUCUAACCAUGUGGUUGCGAGGUAUGAGUAAAACA
UGGUUCCGUCAAGCACCAUGGAACGUCACGCAGCUUUCUACA chr4 20529990 . G
A 0.000399361 + 93

hsa-mir-218-2
GACCAGUCGCUGCGGGGCUUUCCUUUGUGCUUGAUCUAACCAUGUGGUGGAACGAUGGAAACGGAACA
UGGUUCUGUCAAGCACCGCGGAAAGCACCGUGCUCUCCUGCA chr5 168195252 . G
A 0.000199681 - 9

hsa-mir-218-2
GACCAGUCGCUGCGGGGCUUUCCUUUGUGCUUGAUCUAACCAUGUGGUGGAACGAUGGAAACGGAACA
UGGUUCUGUCAAGCACCGCGGAAAGCACCGUGCUCUCCUGCA chr5 168195174
rs140638702 C U 0.000399361 - 87

hsa-mir-219a-1
CCGCCCCGGGCCGCGGCUCCUGAUUGUCCAAACGCAAUUCUCGAGUCUAUGGCUCCGGCCGAGAGUUG
AGUCUGGACGUCCCGAGCCGCCGCCCCCAAACCUCGAGCGGG chr6 33175642 . A
C 0.000199681 + 31

hsa-mir-219a-1
CCGCCCCGGGCCGCGGCUCCUGAUUGUCCAAACGCAAUUCUCGAGUCUAUGGCUCCGGCCGAGAGUUG
AGUCUGGACGUCCCGAGCCGCCGCCCCCAAACCUCGAGCGGG chr6 33175702 . G
U 0.000199681 + 91

hsa-mir-219a-2
ACUCAGGGGCUUCGCCACUGAUUGUCCAAACGCAAUUCUUGUACGAGUCUGCGGCCAACCGAGAAUUG
UGGCUGGACAUCUGUGGCUGAGCUCCGGG chr9 131154943 . G U
0.000199681 - 51

hsa-mir-219b
GGAGCUCAGCCACAGAUGUCCAGCCACAAUUCUCGGUUGGCCGCAGACUCGUACAAGAAUUGCGUUUG
GACAAUCAGUGGCGAAGCCC chr9 131154943 . C A
0.000199681 + 44
hsa-mir-221
UGAACAUCCAGGUCUGGGGCAUGAACCUGGCAUACAAUGUAGAUUUCUGUGUUCGUUAGGCAACAGCU
ACAUUGUCUGCUGGGUUUCAGGCUACCUGGAAACAUGUUCUC chrX 45605666
rs113054794 G U null null 29

hsa-mir-222
GCUGCUGGAAGGUGUAGGUACCCUCAAUGGCUCAGUAGCCAGUGUAGAUCCUGUCUUUCGUAAUCAGC
AGCUACAUCUGGCUACUGGGUCUCUGAUGGCAUCUUCUAGCU chrX 45606510
rs372748524 C U 0.000264901 - 21

hsa-mir-222
GCUGCUGGAAGGUGUAGGUACCCUCAAUGGCUCAGUAGCCAGUGUAGAUCCUGUCUUUCGUAAUCAGC
AGCUACAUCUGGCUACUGGGUCUCUGAUGGCAUCUUCUAGCU chrX 45606504
rs191727254 A U 0.000264901 - 27

hsa-mir-222
GCUGCUGGAAGGUGUAGGUACCCUCAAUGGCUCAGUAGCCAGUGUAGAUCCUGUCUUUCGUAAUCAGC
AGCUACAUCUGGCUACUGGGUCUCUGAUGGCAUCUUCUAGCU chrX 45606472
rs187612279 C U 0.000264901 - 59

hsa-mir-222
GCUGCUGGAAGGUGUAGGUACCCUCAAUGGCUCAGUAGCCAGUGUAGAUCCUGUCUUUCGUAAUCAGC
AGCUACAUCUGGCUACUGGGUCUCUGAUGGCAUCUUCUAGCU chrX 45606471 rs72631825
G A 0.000264901 - 60
hsa-mir-223
CCUGGCCUCCUGCAGUGCCACGCUCCGUGUAUUUGACAAGCUGAGUUGGACACUCCAUGUGGUAGAGU
GUCAGUUUGUCAAAUACCCCAAGUGCGGCACAUGCUUACCAG chrX 65238733
rs186354597 G A 0.0010596 + 22

hsa-mir-223
CCUGGCCUCCUGCAGUGCCACGCUCCGUGUAUUUGACAAGCUGAGUUGGACACUCCAUGUGGUAGAGU
GUCAGUUUGUCAAAUACCCCAAGUGCGGCACAUGCUUACCAG chrX 65238751 . G
A 0.000264901 + 40
hsa-mir-223
CCUGGCCUCCUGCAGUGCCACGCUCCGUGUAUUUGACAAGCUGAGUUGGACACUCCAUGUGGUAGAGU
GUCAGUUUGUCAAAUACCCCAAGUGCGGCACAUGCUUACCAG chrX 65238767 rs34952329
- C null null 56
hsa-mir-223
CCUGGCCUCCUGCAGUGCCACGCUCCGUGUAUUUGACAAGCUGAGUUGGACACUCCAUGUGGUAGAGU
GUCAGUUUGUCAAAUACCCCAAGUGCGGCACAUGCUUACCAG chrX 65238773 . G
A 0.000264901 + 62
hsa-mir-223
CCUGGCCUCCUGCAGUGCCACGCUCCGUGUAUUUGACAAGCUGAGUUGGACACUCCAUGUGGUAGAGU

GUCAGUUUGUCAAAUACCCCAAGUGCGGCACAUGCUUACCAG chrX 65238806 . G
A 0.000264901 + 95
hsa-mir-224
GGGCUUUCAAGUCACUAGUGGUUCCGUUUAGUAGAUGAUUGUGCAUUGUUUCAAAAUGGUGCCCUAGU
GACUACAAAGCCCchrX 151127105 rs370848505 G A 0.0013245 -
26
hsa-mir-224
GGGCUUUCAAGUCACUAGUGGUUCCGUUUAGUAGAUGAUUGUGCAUUGUUUCAAAAUGGUGCCCUAGU
GACUACAAAGCCCchrX 151127092 rs188519172 U C 0.0018543 -
39
hsa-mir-2276
GUGUUCUUCCAGUCCGCCCUCUGUCACCUUGCAGACGGCUUUCUCUCCGAAUGUCUGCAAGUGUCAGA
GGCGAGGAGUGGCAGCUGCAU chr13 24736590 rs139581948 C U
0.000399361 + 36
hsa-mir-2276
GUGUUCUUCCAGUCCGCCCUCUGUCACCUUGCAGACGGCUUUCUCUCCGAAUGUCUGCAAGUGUCAGA
GGCGAGGAGUGGCAGCUGCAU chr13 24736638 rs74399078 C G
null null 84
hsa-mir-2277
GUGCUUCCUGCGGGCUGAGCGCGGGCUGAGCGCUGCCAGUCAGCGCUCACAUUAAGGCUGACAGCGCC
CUGCCUGGCUCGGCCGGCGAAGCUC chr5 92956443 . U G
0.000599042 - 52
hsa-mir-2277
GUGCUUCCUGCGGGCUGAGCGCGGGCUGAGCGCUGCCAGUCAGCGCUCACAUUAAGGCUGACAGCGCC
CUGCCUGGCUCGGCCGGCGAAGCUC chr5 92956422 rs73772915 C G
0.00279553 - 73
hsa-mir-2277
GUGCUUCCUGCGGGCUGAGCGCGGGCUGAGCGCUGCCAGUCAGCGCUCACAUUAAGGCUGACAGCGCC
CUGCCUGGCUCGGCCGGCGAAGCUC chr5 92956420 rs181040414 G A
0.000199681 - 75
hsa-mir-2277
GUGCUUCCUGCGGGCUGAGCGCGGGCUGAGCGCUGCCAGUCAGCGCUCACAUUAAGGCUGACAGCGCC
CUGCCUGGCUCGGCCGGCGAAGCUC chr5 92956416 . C A
0.000199681 - 79
hsa-mir-2277
GUGCUUCCUGCGGGCUGAGCGCGGGCUGAGCGCUGCCAGUCAGCGCUCACAUUAAGGCUGACAGCGCC
CUGCCUGGCUCGGCCGGCGAAGCUC chr5 92956412 . C U
0.000199681 - 83
hsa-mir-2277
GUGCUUCCUGCGGGCUGAGCGCGGGCUGAGCGCUGCCAGUCAGCGCUCACAUUAAGGCUGACAGCGCC
CUGCCUGGCUCGGCCGGCGAAGCUC chr5 92956409 rs111409602 C U
0.0313498 - 86
hsa-mir-2278
GUGCUGCAGGUGUUGGAGAGCAGUGUGUGUUGCCUGGGGACUGUGUGGACUGGUAUCACCCAGACAGC
UUGCACUGACUCCAGACCCUGCCGUCAU chr9 97572244 rs356125 G A
0.0427316 + 1
hsa-mir-2355

CAGACGUGUCAUCCCCAGAUACAAUGGACAAUAUGCUAUUAUAAUCGUAUGGCAUUGUCCUUGCUGUU
UGGAGAUAAUACUGCUGAC chr2 207974756 rs144924002 U C
0.000599042 - 42
hsa-mir-2355
CAGACGUGUCAUCCCCAGAUACAAUGGACAAUAUGCUAUUAUAAUCGUAUGGCAUUGUCCUUGCUGUU
UGGAGAUAAUACUGCUGAC chr2 207974741 . G U 0.000199681 -
57
hsa-mir-2355
CAGACGUGUCAUCCCCAGAUACAAUGGACAAUAUGCUAUUAUAAUCGUAUGGCAUUGUCCUUGCUGUU
UGGAGAUAAUACUGCUGAC chr2 207974738 . C A 0.000199681 -
60
hsa-mir-2392
AUGGUCCCUCCCAAUCCAGCCAUUCCUCAGACCAGGUGGCUCCCGAGCCACCCCAGGCUGUAGGAUGG
GGGUGAGAGGUGCUAG chr14 101280828 . A G 0.000199681 +
1
hsa-mir-2392
AUGGUCCCUCCCAAUCCAGCCAUUCCUCAGACCAGGUGGCUCCCGAGCCACCCCAGGCUGUAGGAUGG
GGGUGAGAGGUGCUAG chr14 101280841 rs182974078 A G 0.00179712
+ 14
hsa-mir-2392
AUGGUCCCUCCCAAUCCAGCCAUUCCUCAGACCAGGUGGCUCCCGAGCCACCCCAGGCUGUAGGAUGG
GGGUGAGAGGUGCUAG chr14 101280856 rs73347569 A G 0.0391374
+ 29
hsa-mir-2392
AUGGUCCCUCCCAAUCCAGCCAUUCCUCAGACCAGGUGGCUCCCGAGCCACCCCAGGCUGUAGGAUGG
GGGUGAGAGGUGCUAG chr14 101280859 . C U 0.000199681 +
32
hsa-mir-2392
AUGGUCCCUCCCAAUCCAGCCAUUCCUCAGACCAGGUGGCUCCCGAGCCACCCCAGGCUGUAGGAUGG
GGGUGAGAGGUGCUAG chr14 101280892 rs118055959 A G 0.00319489
+ 65
hsa-mir-23a
GGCCGGCUGGGGUUCCUGGGGAUGGGAUUUGCUUCCUGUCACAAAUCACAUUGCCAGGGAUUUCCAAC
CGACC chr19 13947436 . G C 0.000199681 - 38

hsa-mir-23b
CUCAGGUGCUCUGGCUGCUUGGGUUCCUGGCAUGCUGAUUUGUGACUUAAGAUUAAAAUCACAUUGCC
AGGGAUUACCACGCAACCACGACCUUGGC chr9 97847498 . C G
0.000199681 + 9
hsa-mir-23b
CUCAGGUGCUCUGGCUGCUUGGGUUCCUGGCAUGCUGAUUUGUGACUUAAGAUUAAAAUCACAUUGCC
AGGGAUUACCACGCAACCACGACCUUGGC chr9 97847535 . C U
0.000199681 + 46
hsa-mir-23b
CUCAGGUGCUCUGGCUGCUUGGGUUCCUGGCAUGCUGAUUUGUGACUUAAGAUUAAAAUCACAUUGCC
AGGGAUUACCACGCAACCACGACCUUGGC chr9 97847569 . C U
0.000399361 + 80

hsa-mir-23b
CUCAGGUGCUCUGGCUGCUUGGGUUCCUGGCAUGCUGAUUUGUGACUUAAGAUUAAAAUCACAUUGCCAGGGAUUACCACGCAACCACGACCUUGGC chr9 97847573 . A U 0.000199681 + 84
hsa-mir-23c
AGUGACUUUCCAGGUGUCACACAGUGAGUGGCAUAAUCAGAGUACAAUUUGAGUCAUGCCCAUACAUCACAUUGCCAGUGAUUACCCAAGGAAAGUGACG chrX 20035242 . A G 0.000264901 - 64
hsa-mir-24-1
CUCCGGUGCCUACUGAGCUGAUAUCAGUUCUCAUUUUACACACUGGCUCAGUUCAGCAGGAACAGGAG chr9 97848319 . G C 0.000399361 + 17

hsa-mir-24-1
CUCCGGUGCCUACUGAGCUGAUAUCAGUUCUCAUUUUACACACUGGCUCAGUUCAGCAGGAACAGGAG chr9 97848343 rs181496497 C U 0.000199681 + 41

hsa-mir-24-2
CUCUGCCUCCCGUGCCUACUGAGCUGAAACACAGUUGGUUUGUGUACACUGGCUCAGUUCAGCAGGAACAGGG chr19 13947170 . U C 0.000199681 - 4

hsa-mir-2467
GGACAGGCACCUGAGGCUCUGUUAGCCUUGGCUCUGGGUCCUGCUCCUUAGAGCAGAGGCAGAGAGGCUCAGGGUCUGUCUchr2 240273490 . C U 0.000199681 - 10

hsa-mir-25
GGCCAGUGUUGAGAGGCGGAGACUUGGGCAAUUGCUGGACGCUGCCCUGGGCAUUGCACUUGUCUCGGUCUGACAGUGCCGGCC chr7 99691200 rs41274221 G A 0.000199681 - 67
hsa-mir-2681
GCCCCCUUUUCACGCAUUUGUGUUUUACCACCUCCAGGAGACUGCCCAAAGACUCUUCAGUAUCAUGGAGUUGGUAAAGCACAGAUGCAUGAAUAAUUCAACGUG chr13 102620081 . A G 0.000199681 - 16
hsa-mir-2681
GCCCCCUUUUCACGCAUUUGUGUUUUACCACCUCCAGGAGACUGCCCAAAGACUCUUCAGUAUCAUGGAGUUGGUAAAGCACAGAUGCAUGAAUAAUUCAACGUG chr13 102619995 rs180991645 C U 0.00139776 - 102
hsa-mir-2682
ACCUUCCUGAAAGAGGUUGGGGCAGGCAGUGACUGUUCAGACGUCCAAUCUCUUUGGGACGCCUCUUCAGCGCUGUCUUCCCUGCCUCUGCCUUUAGGACGAGUCUCAAA chr1 98510902 . C U 0.000199681 - 6
hsa-mir-2682
ACCUUCCUGAAAGAGGUUGGGGCAGGCAGUGACUGUUCAGACGUCCAAUCUCUUUGGGACGCCUCUUCAGCGCUGUCUUCCCUGCCUCUGCCUUUAGGACGAGUCUCAAA chr1 98510896 . A G 0.000199681 - 12
hsa-mir-2682
ACCUUCCUGAAAGAGGUUGGGGCAGGCAGUGACUGUUCAGACGUCCAAUCUCUUUGGGACGCCUCUUCAGCGCUGUCUUCCCUGCCUCUGCCUUUAGGACGAGUCUCAAA chr1 98510864 rs80017247

U A 0.000798722 - 44
hsa-mir-2682
ACCUUCCUGAAAGAGGUUGGGGCAGGCAGUGACUGUUCAGACGUCCAAUCUCUUUGGGACGCCUCUUC
AGCGCUGUCUUCCCUGCCUCUGCCUUUAGGACGAGUCUCAAA chr1 98510847 rs74904371
G A 0.0207668 - 61
hsa-mir-26a-1
GUGGCCUCGUUCAAGUAAUCCAGGAUAGGCUGUGCAGGUCCCAAUGGGCCUAUUCUUGGUUACUUGCA
CGGGGACGC chr3 38010903 rs370719000 G A 0.000599042 +
9
hsa-mir-26a-1
GUGGCCUCGUUCAAGUAAUCCAGGAUAGGCUGUGCAGGUCCCAAUGGGCCUAUUCUUGGUUACUUGCA
CGGGGACGC chr3 38010938 rs190898565 A G 0.000199681 +
44
hsa-mir-26a-1
GUGGCCUCGUUCAAGUAAUCCAGGAUAGGCUGUGCAGGUCCCAAUGGGCCUAUUCUUGGUUACUUGCA
CGGGGACGC chr3 38010964 rs182070256 G A 0.000998403 +
70
hsa-mir-26b
CCGGGACCCAGUUCAAGUAAUUCAGGAUAGGUUGUGUGCUGUCCAGCCUGUUCUCCAUUACUUGGCUC
GGGGACCGG chr2 219267370 . C U 0.000399361 + 2

hsa-mir-26b
CCGGGACCCAGUUCAAGUAAUUCAGGAUAGGUUGUGUGCUGUCCAGCCUGUUCUCCAUUACUUGGCUC
GGGGACCGG chr2 219267371 . G A 0.000199681 + 3

hsa-mir-26b
CCGGGACCCAGUUCAAGUAAUUCAGGAUAGGUUGUGUGCUGUCCAGCCUGUUCUCCAUUACUUGGCUC
GGGGACCGG chr2 219267402 . G U 0.000199681 + 34

hsa-mir-26b
CCGGGACCCAGUUCAAGUAAUUCAGGAUAGGUUGUGUGCUGUCCAGCCUGUUCUCCAUUACUUGGCUC
GGGGACCGG chr2 219267407 rs188612260 C U 0.000798722 +
39
hsa-mir-26b
CCGGGACCCAGUUCAAGUAAUUCAGGAUAGGUUGUGUGCUGUCCAGCCUGUUCUCCAUUACUUGGCUC
GGGGACCGG chr2 219267433 rs373131632 G A 0.000399361 +
65
hsa-mir-26b
CCGGGACCCAGUUCAAGUAAUUCAGGAUAGGUUGUGUGCUGUCCAGCCUGUUCUCCAUUACUUGGCUC
GGGGACCGG chr2 219267443 . C U 0.000199681 + 75

hsa-mir-27a
CUGAGGAGCAGGGCUUAGCUGCUUGUGAGCAGGGUCCACACCAAGUCGUGUUCACAGUGGCUAAGUUC
CGCCCCCCAG chr19 13947296 rs11671784 C U 0.00698882 -
36
hsa-mir-27a
CUGAGGAGCAGGGCUUAGCUGCUUGUGAGCAGGGUCCACACCAAGUCGUGUUCACAGUGGCUAAGUUC

CGCCCCCCAG chr19 13947292 rs895819 A G 0.363818 -
40
hsa-mir-27b
ACCUCUCUAACAAGGUGCAGAGCUUAGCUGAUUGGUGAACAGUGAUUGGUUUCCGCUUUGUUCACAGU
GGCUAAGUUCUGCACCUGAAGAGAAGGUG chr9 97847807 rs192552111 C U
0.000199681 + 81
hsa-mir-28
GGUCCUUGCCCUCAAGGAGCUCACAGUCUAUUGAGUUACCUUUCUGACUUUCCCACUAGAUUGUGAGC
UCCUGGAGGGCAGGCACU chr3 188406598 . A G 0.000199681 +
30
hsa-mir-28
GGUCCUUGCCCUCAAGGAGCUCACAGUCUAUUGAGUUACCUUUCUGACUUUCCCACUAGAUUGUGAGC
UCCUGGAGGGCAGGCACU chr3 188406636 rs78547906 C U 0.00379393
+ 68
hsa-mir-2861
GGCGCCUCUGCAGCUCCGGCUCCCCCUGGCCUCUCGGGAACUACAAGUCCCAGGGGGCCUGGCGGUGG
GCGGCGGGCGGAAGAGGCGGGG chr9 130548199 . C U 0.00179712
+ 3
hsa-mir-2861
GGCGCCUCUGCAGCUCCGGCUCCCCCUGGCCUCUCGGGAACUACAAGUCCCAGGGGGCCUGGCGGUGG
GCGGCGGGCGGAAGAGGCGGGG chr9 130548214 . G C
0.000199681 + 18
hsa-mir-2909
GGUGUUAGGGCCAACAUCUCUUGGUCUUUCCCCUGUGGUCCCAAGAUGGCUGUUGCAACUUAACGCCA
U chr17 35391051 rs187707102 G A 0.000199681 + 10

hsa-mir-2909
GGUGUUAGGGCCAACAUCUCUUGGUCUUUCCCCUGUGGUCCCAAGAUGGCUGUUGCAACUUAACGCCA
U chr17 35391065 . G A 0.000199681 + 24

hsa-mir-2909
GGUGUUAGGGCCAACAUCUCUUGGUCUUUCCCCUGUGGUCCCAAGAUGGCUGUUGCAACUUAACGCCA
U chr17 35391066 . U G 0.00259585 + 25

hsa-mir-2909
GGUGUUAGGGCCAACAUCUCUUGGUCUUUCCCCUGUGGUCCCAAGAUGGCUGUUGCAACUUAACGCCA
U chr17 35391105 rs140415109 C U 0.00219649 + 64

hsa-mir-296
AGGACCCUUCCAGAGGGCCCCCCCUCAAUCCUGUUGUGCCUAAUUCAGAGGGUUGGGUGGAGGCUCUC
CUGAAGGGCUCU chr20 57392715 . U C 0.000599042 - 35

hsa-mir-296
AGGACCCUUCCAGAGGGCCCCCCCUCAAUCCUGUUGUGCCUAAUUCAGAGGGUUGGGUGGAGGCUCUC
CUGAAGGGCUCU chr20 57392697 . U A 0.000399361 - 53

hsa-mir-296

AGGACCCUUCCAGAGGGCCCCCCCUCAAUCCUGUUGUGCCUAAUUCAGAGGGUUGGGUGGAGGCUCUC
CUGAAGGGCUCU chr20 57392686 rs117258475 C U 0.00499201 -
64
hsa-mir-296
AGGACCCUUCCAGAGGGCCCCCCCUCAAUCCUGUUGUGCCUAAUUCAGAGGGUUGGGUGGAGGCUCUC
CUGAAGGGCUCU chr20 57392678 . A G 0.000199681 - 72

hsa-mir-297
UGUAUGUAUGUGUGCAUGUGCAUGUAUGUGUAUAUACAUAUAUAUGUAUUAUGUACUCAUAUAUCA
chr4 111781790 . G A 0.000399361 - 14

hsa-mir-297
UGUAUGUAUGUGUGCAUGUGCAUGUAUGUGUAUAUACAUAUAUAUGUAUUAUGUACUCAUAUAUCA
chr4 111781746 rs113258805 C U null null 58

hsa-mir-297
UGUAUGUAUGUGUGCAUGUGCAUGUAUGUGUAUAUACAUAUAUAUGUAUUAUGUACUCAUAUAUCA
chr4 111781744 . U C 0.000199681 - 60

hsa-mir-297
UGUAUGUAUGUGUGCAUGUGCAUGUAUGUGUAUAUACAUAUAUAUGUAUUAUGUACUCAUAUAUCA
chr4 111781785 rs112636147 U C null null 19

hsa-mir-299
AAGAAAUGGUUUACCGUCCCACAUACAUUUUGAAUAUGUAUGUGGGAUGGUAAACCGCUUCUU
chr14 101490145 rs41286566 C U null null 15

hsa-mir-299
AAGAAAUGGUUUACCGUCCCACAUACAUUUUGAAUAUGUAUGUGGGAUGGUAAACCGCUUCUU
chr14 101490178 . U C 0.000199681 + 48

hsa-mir-300
UGCUACUUGAAGAGAGGUAAUCCUUCACGCAUUUGCUUUACUUGCAAUGAUUAUACAAGGGCAGACUC
UCUCUGGGGAGCAAA chr14 101507711 . G A 0.000199681 +
12
hsa-mir-300
UGCUACUUGAAGAGAGGUAAUCCUUCACGCAUUUGCUUUACUUGCAAUGAUUAUACAAGGGCAGACUC
UCUCUGGGGAGCAAA chr14 101507720 rs374115400 U C
0.000399361 + 21
hsa-mir-300
UGCUACUUGAAGAGAGGUAAUCCUUCACGCAUUUGCUUUACUUGCAAUGAUUAUACAAGGGCAGACUC
UCUCUGGGGAGCAAA chr14 101507727 rs12894467 C U 0.602636
+ 28
hsa-mir-300
UGCUACUUGAAGAGAGGUAAUCCUUCACGCAUUUGCUUUACUUGCAAUGAUUAUACAAGGGCAGACUC
UCUCUGGGGAGCAAA chr14 101507744 . C A 0.000199681 +
45

hsa-mir-300
UGCUACUUGAAGAGAGGUAAUCCUUCACGCAUUUGCUUUACUUGCAAUGAUUAUACAAGGGCAGACUC
UCUCUGGGGAGCAAA chr14 101507779 . C G 0.000399361 +
80
hsa-mir-301a
ACUGCUAACGAAUGCUCUGACUUUAUUGCACUACUGUACUUUACAGCUAGCAGUGCAAUAGUAUUGUC
AAAGCAUCUGAAAGCAGG chr17 57228574 rs376863738 C U
0.000199681 - 9
hsa-mir-301a
ACUGCUAACGAAUGCUCUGACUUUAUUGCACUACUGUACUUUACAGCUAGCAGUGCAAUAGUAUUGUC
AAAGCAUCUGAAAGCAGG chr17 57228573 rs370820064 G A
0.000399361 - 10
hsa-mir-302a
CCACCACUUAAACGUGGAUGUACUUGCUUUGAAACUAAAGAAGUAAGUGCUUCCAUGUUUUGGUGAUG
G chr4 113569406 . C G 0.00139776 - 2

hsa-mir-302a
CCACCACUUAAACGUGGAUGUACUUGCUUUGAAACUAAAGAAGUAAGUGCUUCCAUGUUUUGGUGAUG
G chr4 113569394 . G A 0.000199681 - 14

hsa-mir-302b
GCUCCCUUCAACUUUAACAUGGAAGUGCUUUCUGUGACUUUAAAAGUAAGUGCUUCCAUGUUUUAGUA
GGAGU chr4 113569696 . C U 0.000199681 - 18

hsa-mir-302b
GCUCCCUUCAACUUUAACAUGGAAGUGCUUUCUGUGACUUUAAAAGUAAGUGCUUCCAUGUUUUAGUA
GGAGU chr4 113569695 rs114318553 A C 0.00259585 - 19

hsa-mir-302b
GCUCCCUUCAACUUUAACAUGGAAGUGCUUUCUGUGACUUUAAAAGUAAGUGCUUCCAUGUUUUAGUA
GGAGU chr4 113569692 rs183105707 G U 0.000199681 - 22

hsa-mir-302b
GCUCCCUUCAACUUUAACAUGGAAGUGCUUUCUGUGACUUUAAAAGUAAGUGCUUCCAUGUUUUAGUA
GGAGU chr4 113569680 rs190807868 G A 0.000798722 - 34

hsa-mir-302c
CCUUUGCUUUAACAUGGGGGUACCUGCUGUGUGAAACAAAAGUAAGUGCUUCCAUGUUUCAGUGGAGG
chr4 113569519 . G U 0.000199681 - 68

hsa-mir-302d
CCUCUACUUUAACAUGGAGGCACUUGCUGUGACAUGACAAAAAUAAGUGCUUCCAUGUUUGAGUGUGG
chr4 113569211 . G C 0.000798722 - 17

hsa-mir-302d
CCUCUACUUUAACAUGGAGGCACUUGCUGUGACAUGACAAAAAUAAGUGCUUCCAUGUUUGAGUGUGG
chr4 113569207 rs138673224 C A 0.00479233 - 21

hsa-mir-302d
CCUCUACUUUAACAUGGAGGCACUUGCUGUGACAUGACAAAAAUAAGUGCUUCCAUGUUUGAGUGUGG
chr4 113569193 rs370548538 U G 0.000199681 - 35

hsa-mir-302e
UUGGGUAAGUGCUUCCAUGCUUCAGUUUCCUUACUGGUAAGAUGGAUGUAGUAAUAGCACCUACCUUA
UAGA chr11 7256001 . G U 0.000399361 + 5

hsa-mir-302e
UUGGGUAAGUGCUUCCAUGCUUCAGUUUCCUUACUGGUAAGAUGGAUGUAGUAAUAGCACCUACCUUA
UAGA chr11 7256013 . A C 0.000199681 + 17

hsa-mir-302e
UUGGGUAAGUGCUUCCAUGCUUCAGUUUCCUUACUGGUAAGAUGGAUGUAGUAAUAGCACCUACCUUA
UAGA chr11 7256057 rs143234363 C G 0.00139776 + 61

hsa-mir-302f UCUGUGUAAACCUGGCAAUUUUCACUUAAUUGCUUCCAUGUUUAUAAAAGA chr18
27878912 . C U 0.00159744 + 37

hsa-mir-3064
GGUCUGGCUGUUGUGGUGUGCAAAACUCCGUACAUUGCUAUUUUGCCACACUGCAACACCUUACAG
chr17 62496926 . A G 0.000199681 - 32

hsa-mir-3064
GGUCUGGCUGUUGUGGUGUGCAAAACUCCGUACAUUGCUAUUUUGCCACACUGCAACACCUUACAG
chr17 62496924 rs200967986 A U 0.000199681 - 34

hsa-mir-3065
CUGCCCUCUUCAACAAAAUCACUGAUGCUGGAGUCGCCUGAGUCAUCACUCAGCACCAGGAUAUUGUU
GGAGAGGACAG chr17 79099712 . G A 0.000199681 + 36

hsa-mir-3065
CUGCCCUCUUCAACAAAAUCACUGAUGCUGGAGUCGCCUGAGUCAUCACUCAGCACCAGGAUAUUGUU
GGAGAGGACAG chr17 79099736 . G A 0.000199681 + 60

hsa-mir-3065
CUGCCCUCUUCAACAAAAUCACUGAUGCUGGAGUCGCCUGAGUCAUCACUCAGCACCAGGAUAUUGUU
GGAGAGGACAG chr17 79099750 . G C 0.000199681 + 74

hsa-mir-3074
GCUCGACUCCUGUUCCUGCUGAACUGAGCCAGUGUGUAAAAUGAGAACUGAUAUCAGCUCAGUAGGCA
CCGGAGGGCGGGUchr9 97848343 rs181496497 G A 0.000199681 -
34
hsa-mir-3074
GCUCGACUCCUGUUCCUGCUGAACUGAGCCAGUGUGUAAAAUGAGAACUGAUAUCAGCUCAGUAGGCA
CCGGAGGGCGGGUchr9 97848319 . C G 0.000399361 - 58

hsa-mir-30a
GCGACUGUAAACAUCCUCGACUGGAAGCUGUGAAGCCACAGAUGGGCUUUCAGUCGGAUGUUUGCAGC
UGC chr6 72113306 rs190842689 G C 0.000199681 - 19

hsa-mir-30a
GCGACUGUAAACAUCCUCGACUGGAAGCUGUGAAGCCACAGAUGGGCUUUCAGUCGGAUGUUUGCAGC
UGC chr6 72113270 rs149150037 C U 0.000199681 - 55

hsa-mir-30a
GCGACUGUAAACAUCCUCGACUGGAAGCUGUGAAGCCACAGAUGGGCUUUCAGUCGGAUGUUUGCAGC
UGC chr6 72113269 rs370184894 G A 0.000199681 - 56

hsa-mir-30a
GCGACUGUAAACAUCCUCGACUGGAAGCUGUGAAGCCACAGAUGGGCUUUCAGUCGGAUGUUUGCAGC
UGC chr6 72113261 . G C 0.000199681 - 64

hsa-mir-30b
ACCAAGUUUCAGUUCAUGUAAACAUCCUACACUCAGCUGUAAUACAUGGAUUGGCUGGGAGGUGGAUG
UUUACUUCAGCUGACUUGGA chr8 135812836 rs111424617 C U
0.000399361 - 15
hsa-mir-30c-2
AGAUACUGUAAACAUCCUACACUCUCAGCUGUGGAAAGUAAGAAAGCUGGGAGAAGGCUGUUUACUCU
UUCU chr6 72086720 rs113749278 U C null null 15

hsa-mir-30d
GUUGUUGUAAACAUCCCCGACUGGAAGCUGUAAGACACAGCUAAGCUUUCAGUCAGAUGUUUGCUGCU
AC chr8 135817150 . A G 0.000199681 - 39

hsa-mir-30e
GGGCAGUCUUUGCUACUGUAAACAUCCUUGACUGGAAGCUGUAAGGUGUUCAGAGGAGCUUUCAGUCG
GAUGUUUACAGCGGCAGGCUGCCA chr1 41220077 rs112439044 C U
0.00379393 + 51
hsa-mir-30e
GGGCAGUCUUUGCUACUGUAAACAUCCUUGACUGGAAGCUGUAAGGUGUUCAGAGGAGCUUUCAGUCG
GAUGUUUACAGCGGCAGGCUGCCA chr1 41220094 . G A
0.000199681 + 68
hsa-mir-3116-1
CUUUAUUGAGUCCCUACUAUGUUCCAGGCACUGGGUAUCGUAGGUGCCUGGAACAUAGUAGGGACUCA
AUAAAG chr1 62544470 rs374995682 C U 0.000998403 + 13

hsa-mir-3116-1
CUUUAUUGAGUCCCUACUAUGUUCCAGGCACUGGGUAUCGUAGGUGCCUGGAACAUAGUAGGGACUCA
AUAAAG chr1 62544486 . C U 0.000998403 + 29

hsa-mir-3116-1
CUUUAUUGAGUCCCUACUAUGUUCCAGGCACUGGGUAUCGUAGGUGCCUGGAACAUAGUAGGGACUCA

AUAAAG chr1 62544490 rs141963809 G U 0.000599042 + 33

hsa-mir-3116-1
CUUUAUUGAGUCCCUACUAUGUUCCAGGCACUGGGUAUCGUAGGUGCCUGGAACAUAGUAGGGACUCA
AUAAAG chr1 62544495 . U A 0.00139776 + 38

hsa-mir-3116-1
CUUUAUUGAGUCCCUACUAUGUUCCAGGCACUGGGUAUCGUAGGUGCCUGGAACAUAGUAGGGACUCA
AUAAAG chr1 62544503 rs192207383 G A 0.000399361 + 46

hsa-mir-3116-2
UAUUGAGUCCCUACUAUGUUCCAGGCACCUACGAUACCCAGUGCCUGGAACAUAGUAGGGACUCAAUA
chr1 62544503 rs192207383 C U 0.000399361 - 26

hsa-mir-3116-2
UAUUGAGUCCCUACUAUGUUCCAGGCACCUACGAUACCCAGUGCCUGGAACAUAGUAGGGACUCAAUA
chr1 62544495 . A U 0.00139776 - 34

hsa-mir-3116-2
UAUUGAGUCCCUACUAUGUUCCAGGCACCUACGAUACCCAGUGCCUGGAACAUAGUAGGGACUCAAUA
chr1 62544490 rs141963809 C A 0.000599042 - 39

hsa-mir-3116-2
UAUUGAGUCCCUACUAUGUUCCAGGCACCUACGAUACCCAGUGCCUGGAACAUAGUAGGGACUCAAUA
chr1 62544486 . G A 0.000998403 - 43

hsa-mir-3116-2
UAUUGAGUCCCUACUAUGUUCCAGGCACCUACGAUACCCAGUGCCUGGAACAUAGUAGGGACUCAAUA
chr1 62544470 rs374995682 G A 0.000998403 - 59

hsa-mir-3117
CCCUAAAGGGCCAGACACUAUACGAGUCAUAUAAGGGAAGGCAUUAUAGGACUCAUAUAGUGCCAGGU
GUUUUGUGGG chr1 67094145 . C U 0.000199681 + 23

hsa-mir-3117
CCCUAAAGGGCCAGACACUAUACGAGUCAUAUAAGGGAAGGCAUUAUAGGACUCAUAUAGUGCCAGGU
GUUUUGUGGG chr1 67094150 . C U 0.000199681 + 28

hsa-mir-3117
CCCUAAAGGGCCAGACACUAUACGAGUCAUAUAAGGGAAGGCAUUAUAGGACUCAUAUAGUGCCAGGU
GUUUUGUGGG chr1 67094171 rs12402181 G A 0.292332 +
49

hsa-mir-3118-1
CACACAUACAAUAAUAUUCAUAAUGCAAUCACACACAAUCACCAUGUGACUGCAUUAUGAAAAUUCUU
CUAGUGUG chr1 142667289 rs75154299 C A null null 1

hsa-mir-3118-1

CACACAUACAAUAAUAUUCAUAAUGCAAUCACACACAAUCACCAUGUGACUGCAUUAUGAAAAUUCUU
CUAGUGUG chr1 142667306 rs75357324 C U null null 18

hsa-mir-3118-1
CACACAUACAAUAAUAUUCAUAAUGCAAUCACACACAAUCACCAUGUGACUGCAUUAUGAAAAUUCUU
CUAGUGUG chr1 142667330 rs76132421 U C null null 42

hsa-mir-3118-1
CACACAUACAAUAAUAUUCAUAAUGCAAUCACACACAAUCACCAUGUGACUGCAUUAUGAAAAUUCUU
CUAGUGUG chr1 142667356 rs74987789 G A null null 68

hsa-mir-3118-2
CAUACUACAAUAAUUUUCAUAAUGCAAUCACACACAAUCACCGUGUGACUGCAUUAUGAAAAUUCUUC
UAGUGUG chr1 143163772 rs78472851 G C null null 23

hsa-mir-3118-2
CAUACUACAAUAAUUUUCAUAAUGCAAUCACACACAAUCACCGUGUGACUGCAUUAUGAAAAUUCUUC
UAGUGUG chr1 143163775 rs76191636 A G null null 26

hsa-mir-3118-2
CAUACUACAAUAAUUUUCAUAAUGCAAUCACACACAAUCACCGUGUGACUGCAUUAUGAAAAUUCUUC
UAGUGUG chr15 21038150 . A U 0.000199681 + 27

hsa-mir-3118-2
CAUACUACAAUAAUUUUCAUAAUGCAAUCACACACAAUCACCGUGUGACUGCAUUAUGAAAAUUCUUC
UAGUGUG chr1 143163790 rs79594127 U C null null 41

hsa-mir-3118-2
CAUACUACAAUAAUUUUCAUAAUGCAAUCACACACAAUCACCGUGUGACUGCAUUAUGAAAAUUCUUC
UAGUGUG chr1 143163799 rs61786895 G A null null 50

hsa-mir-3118-2
CAUACUACAAUAAUUUUCAUAAUGCAAUCACACACAAUCACCGUGUGACUGCAUUAUGAAAAUUCUUC
UAGUGUG chr1 143163816 rs75563322 G A null null 67

hsa-mir-3118-3
CAUACUACAAUAAUUUUCAUAAUGCAAUCACACACAAUCACCGUGUGACUGCAUUAUGAAAAUUCUUC
UAGUGUG chr1 143424215 rs9440733 C A null null 1

hsa-mir-3118-3
CAUACUACAAUAAUUUUCAUAAUGCAAUCACACACAAUCACCGUGUGACUGCAUUAUGAAAAUUCUUC
UAGUGUG chr15 22049282 . A G 0.000399361 + 9

hsa-mir-3118-3
CAUACUACAAUAAUUUUCAUAAUGCAAUCACACACAAUCACCGUGUGACUGCAUUAUGAAAAUUCUUC
UAGUGUG chr1 143424198 rs75357324 C U null null 18

hsa-mir-3118-3
CAUACUACAAUAAUUUUCAUAAUGCAAUCACACACAAUCACCGUGUGACUGCAUUAUGAAAAUUCUUC
UAGUGUG chr1 143424165 rs11488501 G A null null 51

hsa-mir-3118-4
CAUACUACAAUAAUUUUCAUAAUGCAAUCACACACAAUCACCGUGUGACUGCAUUAUGAAAAUUCUUC
UAGUGUG chr15 22049282 . A G 0.000399361 + 9

hsa-mir-3118-4
CAUACUACAAUAAUUUUCAUAAUGCAAUCACACACAAUCACCGUGUGACUGCAUUAUGAAAAUUCUUC
UAGUGUG chr15 21038166 rs1814008 G A null null 43

hsa-mir-3118-4
CAUACUACAAUAAUUUUCAUAAUGCAAUCACACACAAUCACCGUGUGACUGCAUUAUGAAAAUUCUUC
UAGUGUG chr15 21038172 rs7167371 C G null null 49

hsa-mir-3119-1
AUUAACUCUGGCUUUUAACUUUGAUGGCAAAGGGGUAGCUAAACAAUCUAUGUCUUUGCCAUCAAAGU
UAAAAGCCAUAGUUAAU chr1 170120594 . G A 0.000399361 -
10
hsa-mir-3119-1
AUUAACUCUGGCUUUUAACUUUGAUGGCAAAGGGGUAGCUAAACAAUCUAUGUCUUUGCCAUCAAAGU
UAAAAGCCAUAGUUAAU chr1 170120575 rs58602811 A C 0.0131789
- 29
hsa-mir-3119-1
AUUAACUCUGGCUUUUAACUUUGAUGGCAAAGGGGUAGCUAAACAAUCUAUGUCUUUGCCAUCAAAGU
UAAAAGCCAUAGUUAAU chr1 170120566 rs185710757 G A
0.000199681 - 38
hsa-mir-3119-2
AUUAACUAUGGCUUUUAACUUUGAUGGCAAAGACAUAGAUUGUUUAGCUACCCCUUUGCCAUCAAAGU
UAAAAGCCAGAGUUAAU chr1 170120566 rs185710757 C U
0.000199681 + 48
hsa-mir-3119-2
AUUAACUAUGGCUUUUAACUUUGAUGGCAAAGACAUAGAUUGUUUAGCUACCCCUUUGCCAUCAAAGU
UAAAAGCCAGAGUUAAU chr1 170120575 rs58602811 U G 0.0131789
+ 57
hsa-mir-3119-2
AUUAACUAUGGCUUUUAACUUUGAUGGCAAAGACAUAGAUUGUUUAGCUACCCCUUUGCCAUCAAAGU
UAAAAGCCAGAGUUAAU chr1 170120594 . C U 0.000399361 +
76
hsa-mir-3121
AAAUGGUUAUGUCCUUUGCCUAUUCUAUUUAAGACACCCUGUACCUUAAAUAGAGUAGGCAAAGGACA
GAAACAUUU chr1 180407512 . C A 0.000199681 - 14

hsa-mir-3121
AAAUGGUUAUGUCCUUUGCCUAUUCUAUUUAAGACACCCUGUACCUUAAAUAGAGUAGGCAAAGGACA
GAAACAUUU chr1 180407505 rs138979245 U C 0.000998403 -

21
hsa-mir-3121
AAAUGGUUAUGUCCUUUGCCUAUUCUAUUUAAGACACCCUGUACCUUAAAUAGAGUAGGCAAAGGACA
GAAACAUUU chr1 180407488 . C G 0.000199681 - 38

hsa-mir-3123
AUGGAUUUGAUUGAAUGAUUCUCCCAUUUCCACAUGGAGAGUGGAGCCCAGAGAAUUGUUUAAUCAUG
UAUCCAU chr1 241295614 . G C 0.00159744 + 43

hsa-mir-3123
AUGGAUUUGAUUGAAUGAUUCUCCCAUUUCCACAUGGAGAGUGGAGCCCAGAGAAUUGUUUAAUCAUG
UAUCCAU chr1 241295617 rs78240175 G A 0.0345447 +
46
hsa-mir-3124
GCGGGCUUCGCGGGCGAAGGCAAAGUCGAUUUCCAAAAGUGACUUUCCUCACUCCCGUGAAGUCGGC
chr1 249120578 . G C 0.000199681 + 3

hsa-mir-3124
GCGGGCUUCGCGGGCGAAGGCAAAGUCGAUUUCCAAAAGUGACUUUCCUCACUCCCGUGAAGUCGGC
chr1 249120584 rs12081872 C U 0.0107827 + 9

hsa-mir-3124
GCGGGCUUCGCGGGCGAAGGCAAAGUCGAUUUCCAAAAGUGACUUUCCUCACUCCCGUGAAGUCGGC
chr1 249120591 . G U 0.000798722 + 16

hsa-mir-3124
GCGGGCUUCGCGGGCGAAGGCAAAGUCGAUUUCCAAAAGUGACUUUCCUCACUCCCGUGAAGUCGGC
chr1 249120610 . A C 0.000199681 + 35

hsa-mir-3124
GCGGGCUUCGCGGGCGAAGGCAAAGUCGAUUUCCAAAAGUGACUUUCCUCACUCCCGUGAAGUCGGC
chr1 249120631 rs115160731 C A 0.0127796 + 56

hsa-mir-3124
GCGGGCUUCGCGGGCGAAGGCAAAGUCGAUUUCCAAAAGUGACUUUCCUCACUCCCGUGAAGUCGGC
chr1 249120640 . G A 0.000199681 + 65

hsa-mir-3125
GAGAAUGGGUAGAGGAAGCUGUGGAGAGAACUCACGGUGCCUGUGGUUCGAGAUCCCCGCCUUCCUCC
UCCUUUCCUC chr2 12877500 rs72536414 - A null null 8

hsa-mir-3125
GAGAAUGGGUAGAGGAAGCUGUGGAGAGAACUCACGGUGCCUGUGGUUCGAGAUCCCCGCCUUCCUCC
UCCUUUCCUC chr2 12877501 rs78852835 G A null null 9

hsa-mir-3125
GAGAAUGGGUAGAGGAAGCUGUGGAGAGAACUCACGGUGCCUGUGGUUCGAGAUCCCCGCCUUCCUCC

UCCUUUCCUC chr2 12877502 rs33977954 - A null null 10

hsa-mir-3125
GAGAAUGGGUAGAGGAAGCUGUGGAGAGAACUCACGGUGCCUGUGGUUCGAGAUCCCCGCCUUCCUCC
UCCUUUCCUC chr2 12877520 . G U 0.000199681 + 28

hsa-mir-3125
GAGAAUGGGUAGAGGAAGCUGUGGAGAGAACUCACGGUGCCUGUGGUUCGAGAUCCCCGCCUUCCUCC
UCCUUUCCUC chr2 12877525 . C U 0.000199681 + 33

hsa-mir-3125
GAGAAUGGGUAGAGGAAGCUGUGGAGAGAACUCACGGUGCCUGUGGUUCGAGAUCCCCGCCUUCCUCC
UCCUUUCCUC chr2 12877527 . C U 0.000199681 + 35

hsa-mir-3126
AUGAUUAUAUGAGGGACAGAUGCCAGAAGCACUGGUUAUGAUUUGCAUCUGGCAUCCGUCACACAGAU
AAUUAU chr2 69330818 rs189885330 U A 0.000798722 + 5

hsa-mir-3126
AUGAUUAUAUGAGGGACAGAUGCCAGAAGCACUGGUUAUGAUUUGCAUCUGGCAUCCGUCACACAGAU
AAUUAU chr2 69330823 . U C 0.00159744 + 10

hsa-mir-3126
AUGAUUAUAUGAGGGACAGAUGCCAGAAGCACUGGUUAUGAUUUGCAUCUGGCAUCCGUCACACAGAU
AAUUAU chr2 69330824 rs182148652 G A 0.000798722 + 11

hsa-mir-3126
AUGAUUAUAUGAGGGACAGAUGCCAGAAGCACUGGUUAUGAUUUGCAUCUGGCAUCCGUCACACAGAU
AAUUAU chr2 69330825 rs187256070 A U 0.000798722 + 12

hsa-mir-3126
AUGAUUAUAUGAGGGACAGAUGCCAGAAGCACUGGUUAUGAUUUGCAUCUGGCAUCCGUCACACAGAU
AAUUAU chr2 69330871 . G A 0.000199681 + 58

hsa-mir-3126
AUGAUUAUAUGAGGGACAGAUGCCAGAAGCACUGGUUAUGAUUUGCAUCUGGCAUCCGUCACACAGAU
AAUUAU chr2 69330886 . A G 0.000199681 + 73

hsa-mir-3127
GGCCAGGCCCAUCAGGGCUUGUGGAAUGGGAAGGAGAAGGGACGCUUCCCCUUCUGCAGGCCUGCUGG
GUGUGGCU chr2 97464049 . A G 0.000199681 + 35

hsa-mir-3127
GGCCAGGCCCAUCAGGGCUUGUGGAAUGGGAAGGAGAAGGGACGCUUCCCCUUCUGCAGGCCUGCUGG
GUGUGGCU chr2 97464057 rs186329656 C U 0.00339457 +
43

hsa-mir-3128

UUCCUCUGGCAAGUAAAAAACUCUCAUUUUCCUUAAAAAAUGAGAGUUUUUUACUUGCAAUAGGAA chr2 178120681 rs61130794 C A 0.00878594 - 58

hsa-mir-3129
GUACUUGGGCAGUAGUGUAGAGAUUGGUUUGCCUGUUAAUGAAUUCAAACUAAUCUCUACACUGCUGCCCAAGAGC chr2 189997834 rs184647365 C U 0.00898562 - 4

hsa-mir-3129
GUACUUGGGCAGUAGUGUAGAGAUUGGUUUGCCUGUUAAUGAAUUCAAACUAAUCUCUACACUGCUGCCCAAGAGC chr2 189997816 rs192364638 G C 0.000599042 - 22

hsa-mir-3130-1
CUUGUCAUGUCUUACCCAGUCUCCGGUGCAGCCUGUUGUCAAGGCUGCACCGGAGACUGGGUAAGACAUGACAAG chr2 207648009 rs115772313 C U 0.0205671 - 24

hsa-mir-3130-1
CUUGUCAUGUCUUACCCAGUCUCCGGUGCAGCCUGUUGUCAAGGCUGCACCGGAGACUGGGUAAGACAUGACAAG chr2 207648001 . C G 0.000199681 - 32

hsa-mir-3130-1
CUUGUCAUGUCUUACCCAGUCUCCGGUGCAGCCUGUUGUCAAGGCUGCACCGGAGACUGGGUAAGACAUGACAAG chr2 207647981 rs2241347 G A 0.42512 - 52

hsa-mir-3130-2
CUUGUCAUGUCUUACCCAGUCUCCGGUGCAGCCUUGACAACAGGCUGCACCGGAGACUGGGUAAGACAUGACAAG chr2 207647981 rs2241347 C U 0.42512 + 24

hsa-mir-3130-2
CUUGUCAUGUCUUACCCAGUCUCCGGUGCAGCCUUGACAACAGGCUGCACCGGAGACUGGGUAAGACAUGACAAG chr2 207648001 . G C 0.000199681 + 44

hsa-mir-3130-2
CUUGUCAUGUCUUACCCAGUCUCCGGUGCAGCCUUGACAACAGGCUGCACCGGAGACUGGGUAAGACAUGACAAG chr2 207648009 rs115772313 G A 0.0205671 + 52

hsa-mir-3131
GAGUCGAGGACUGGUGGAAGGGCCUUUCCCCUCAGACCAAGGCCCUGGCCCCAGCUUCUUCUC chr2 219923421 . C G 0.000199681 - 52

hsa-mir-3131
GAGUCGAGGACUGGUGGAAGGGCCUUUCCCCUCAGACCAAGGCCCUGGCCCCAGCUUCUUCUC chr2 219923418 rs57408770 - CUU null null 55

hsa-mir-3131
GAGUCGAGGACUGGUGGAAGGGCCUUUCCCCUCAGACCAAGGCCCUGGCCCCAGCUUCUUCUC chr2 219923411 rs71974827 - C null null 62

hsa-mir-3132
GGUGGGAUGGGUAGAGAAGGAGCUCAGAGGACGGUGCGCCUUGUUUCCCUUGAGCCCUCCCUCUCUCA
UCCCACC chr2 220413813 rs12997198 C G null null 57

hsa-mir-3132
GGUGGGAUGGGUAGAGAAGGAGCUCAGAGGACGGUGCGCCUUGUUUCCCUUGAGCCCUCCCUCUCUCA
UCCCACC chr2 220413812 . U C 0.000199681 - 58

hsa-mir-3132
GGUGGGAUGGGUAGAGAAGGAGCUCAGAGGACGGUGCGCCUUGUUUCCCUUGAGCCCUCCCUCUCUCA
UCCCACC chr2 220413807 rs113790842 C U null null 63

hsa-mir-3133
CAGAAAUUGUAAAGAACUCUUAAAACCCAAUAGUAAAAAGACAACCUGUUGAGUUUAAGAGUUCUUU
AUAUAUUCUG chr2 242417357 . A G 0.000798722 + 38

hsa-mir-3134
UGUAUCCAAUGUGUAGUCUUUUAUCCCUCACAUGGAGUAAAAUAUGAUGGAUAAAAGACUACAUAUUG
GGUACA chr3 15738870 . A G 0.000199681 - 9

hsa-mir-3134
UGUAUCCAAUGUGUAGUCUUUUAUCCCUCACAUGGAGUAAAAUAUGAUGGAUAAAAGACUACAUAUUG
GGUACA chr3 15738824 . A G 0.000399361 - 55

hsa-mir-3135a
UCACUUUGGUGCCUAGGCUGAGACUGCAGUGGUGCAAUCUCAGUUCACUGCAGCCUUGACCUCCUGGG
CUCAGGUGA chr3 20179097 rs6787734 C U 0.422324 +
41

hsa-mir-3135a
UCACUUUGGUGCCUAGGCUGAGACUGCAGUGGUGCAAUCUCAGUUCACUGCAGCCUUGACCUCCUGGG
CUCAGGUGA chr3 20179098 . A G 0.000798722 + 42

hsa-mir-3135a
UCACUUUGGUGCCUAGGCUGAGACUGCAGUGGUGCAAUCUCAGUUCACUGCAGCCUUGACCUCCUGGG
CUCAGGUGA chr3 20179130 rs142342924 G A 0.108826 +
74

hsa-mir-3135b
UGCCCAGGCUGGAGCGAGUGCAGUGGUGCAGUCAGUCCUAGCUCACUGCAGCCUCGAACUCCUGGGCU
chr6 32717722 rs4351242 G A 0.109425 - 35

hsa-mir-3135b
UGCCCAGGCUGGAGCGAGUGCAGUGGUGCAGUCAGUCCUAGCUCACUGCAGCCUCGAACUCCUGGGCU
chr6 32717702 rs4285314 C U 0.574481 - 55

hsa-mir-3137
UACAGGUCUGUAGCCUGGGAGCAAUGGGGUGUAUGGUAUAGGGGUAGCCUCGUGCUCCUGGGCUACAA
ACCUGUA chr3 194855289 . G A 0.000199681 - 21

hsa-mir-3137
UACAGGUCUGUAGCCUGGGAGCAAUGGGGUGUAUGGUAUAGGGGUAGCCUCGUGCUCCUGGGCUACAA
ACCUGUA chr3 194855248 rs190632554 G A 0.00199681 -
62
hsa-mir-3138
CCCUCCUCGGCACUUCCCCCACCUCACUGCCCGGGUGCCCACAAGACUGUGGACAGUGAGGUAGAGGG
AGUGCCGAGGAGGG chr4 10080309 . C U 0.000199681 -
8
hsa-mir-3139
GGCUCAGAGUAGGAGCUCAACAGAUGCCUGUUGACUGAAUAAUAAACAGGUAUCGCAGGAGCUUUUGU
UAUGUGCC chr4 144264666 . C U 0.000399361 + 54

hsa-mir-3139
GGCUCAGAGUAGGAGCUCAACAGAUGCCUGUUGACUGAAUAAUAAACAGGUAUCGCAGGAGCUUUUGU
UAUGUGCC chr4 144264667 . G A 0.000399361 + 55

hsa-mir-3140
CCUCUUGAGGUACCUGAAUUACCAAAAGCUUUAUGUAUUCUGAAGUUAUUGAAAAUAAGAGCUUUUGG
GAAUUCAGGUAGUUCAGGAGUG chr4 153410531 . U G
0.000399361 - 38
hsa-mir-3140
CCUCUUGAGGUACCUGAAUUACCAAAAGCUUUAUGUAUUCUGAAGUUAUUGAAAAUAAGAGCUUUUGG
GAAUUCAGGUAGUUCAGGAGUG chr4 153410520 . U C 0.00119808
- 49
hsa-mir-3140
CCUCUUGAGGUACCUGAAUUACCAAAAGCUUUAUGUAUUCUGAAGUUAUUGAAAAUAAGAGCUUUUGG
GAAUUCAGGUAGUUCAGGAGUG chr4 153410482 rs116239055 A C
0.000199681 - 87
hsa-mir-3141 UCACCCGGUGAGGGCGGGUGGAGGAGGAGGGUCCCCACCAUCAGCCUUCACUGGGACGGGA
chr5 153975627 rs140135215 C U 0.000399361 - 6

hsa-mir-3141 UCACCCGGUGAGGGCGGGUGGAGGAGGAGGGUCCCCACCAUCAGCCUUCACUGGGACGGGA
chr5 153975613 . G A 0.000199681 - 20

hsa-mir-3141 UCACCCGGUGAGGGCGGGUGGAGGAGGAGGGUCCCCACCAUCAGCCUUCACUGGGACGGGA
chr5 153975576 rs936581 C U 0.091853 - 57

hsa-mir-3142
UUCAGAAAGGCCUUUCUGAACCUUCAGAAAGGCUGCUGAAUCUUCAGAAAGGCCUUUCUGAACCUUCA
GAAAGGCUGCUGAA chr5 159901426 . G A 0.000399361 +
18
hsa-mir-3142
UUCAGAAAGGCCUUUCUGAACCUUCAGAAAGGCUGCUGAAUCUUCAGAAAGGCCUUUCUGAACCUUCA
GAAAGGCUGCUGAA chr5 159901428 . A G 0.000798722 +
20
hsa-mir-3142

UUCAGAAAGGCCUUUCUGAACCUUCAGAAAGGCUGCUGAAUCUUCAGAAAGGCCUUUCUGAACCUUCA
GAAAGGCUGCUGAA chr5 159901465 . U G 0.000399361 +
57
hsa-mir-3143
UAGAUAACAUUGUAAAGCGCUUCUUUCGCGGUUGGGCUGGAGCAACUCUUUACAAUGUUUCUA
chr6 27115438 rs143774706 G C 0.00658946 + 34

hsa-mir-3143
UAGAUAACAUUGUAAAGCGCUUCUUUCGCGGUUGGGCUGGAGCAACUCUUUACAAUGUUUCUA
chr6 27115444 rs181636996 G C 0.000199681 + 40

hsa-mir-3143
UAGAUAACAUUGUAAAGCGCUUCUUUCGCGGUUGGGCUGGAGCAACUCUUUACAAUGUUUCUA
chr6 27115447 . C G 0.000199681 + 43

hsa-mir-3143
UAGAUAACAUUGUAAAGCGCUUCUUUCGCGGUUGGGCUGGAGCAACUCUUUACAAUGUUUCUA
chr6 27115458 . A G 0.000199681 + 54

hsa-mir-3143
UAGAUAACAUUGUAAAGCGCUUCUUUCGCGGUUGGGCUGGAGCAACUCUUUACAAUGUUUCUA
chr6 27115467 rs17737028 A G 0.00579073 + 63

hsa-mir-3144
AACUACACUUUAAGGGGACCAAAGAGAUAUAUAGAUAUCAGCUACCUAUAUACCUGUUCGGUCUCUUU
AAAGUGUAGUU chr6 120336327 rs68035463 C A 0.203275 +
3
hsa-mir-3144
AACUACACUUUAAGGGGACCAAAGAGAUAUAUAGAUAUCAGCUACCUAUAUACCUGUUCGGUCUCUUU
AAAGUGUAGUU chr6 120336365 . G A 0.000599042 + 41

hsa-mir-3144
AACUACACUUUAAGGGGACCAAAGAGAUAUAUAGAUAUCAGCUACCUAUAUACCUGUUCGGUCUCUUU
AAAGUGUAGUU chr6 120336384 rs67106263 G A 0.203674 +
60
hsa-mir-3145
UAUAUGAGUUCAACUCCAAACACUCAAAACUCAUUGUUGAAUGGAAUGAGAUAUUUUGAGUGUUUGGA
AUUGAACUCGUAUA chr6 138756428 rs186957505 A G 0.00219649
- 4
hsa-mir-3145
UAUAUGAGUUCAACUCCAAACACUCAAAACUCAUUGUUGAAUGGAAUGAGAUAUUUUGAGUGUUUGGA
AUUGAACUCGUAUA chr6 138756369 . U C 0.000199681 -
63
hsa-mir-3146
GCUAAGUCCCUUCUUUCUAUCCUAGUAUAACUUGAAGAAUUCAAAUAGUCAUGCUAGGAUAGAAAGAA
UGGGACUUGGC chr7 19745034 . U C 0.000199681 - 26

hsa-mir-3146
GCUAAGUCCCUUCUUUCUAUCCUAGUAUAACUUGAAGAAUUCAAAUAGUCAUGCUAGGAUAGAAAGAAUGGGACUUGGC chr7 19745016 . A G 0.000199681 - 44

hsa-mir-3146
GCUAAGUCCCUUCUUUCUAUCCUAGUAUAACUUGAAGAAUUCAAAUAGUCAUGCUAGGAUAGAAAGAAUGGGACUUGGC chr7 19745003 . G A 0.000399361 - 57

hsa-mir-3146
GCUAAGUCCCUUCUUUCUAUCCUAGUAUAACUUGAAGAAUUCAAAUAGUCAUGCUAGGAUAGAAAGAAUGGGACUUGGC chr7 19744982 rs143801795 G A 0.000599042 - 78

hsa-mir-3147
GUCCGGGUUGGGCAGUGAGGAGGGUGUGACGCCGCGAAGUGCACCUCGCCCUUGUCCAACUCGGAC chr7 57472742 . G U 0.000399361 + 12

hsa-mir-3148
GAGUUAAGAUGGAAAAAACUGGUGUGUGCUUAUUGAUGUAGCCAACAAGCAUACAUCAGUUUUUUCCAACUUAACUC chr8 29814842 . U C 0.000199681 - 23

hsa-mir-3148
GAGUUAAGAUGGAAAAAACUGGUGUGUGCUUAUUGAUGUAGCCAACAAGCAUACAUCAGUUUUUUCCAACUUAACUC chr8 29814829 . A G 0.00119808 - 36

hsa-mir-3148
GAGUUAAGAUGGAAAAAACUGGUGUGUGCUUAUUGAUGUAGCCAACAAGCAUACAUCAGUUUUUUCCAACUUAACUC chr8 29814813 rs142320250 U C 0.000998403 - 52

hsa-mir-3148
GAGUUAAGAUGGAAAAAACUGGUGUGUGCUUAUUGAUGUAGCCAACAAGCAUACAUCAGUUUUUUCCAACUUAACUC chr8 29814796 . A G 0.000199681 - 69

hsa-mir-3149
AUACAUACAUGUACACACACAUGUCAUCCACACACAUACAUAUAUAUAUGUUUGUAUGGAUAUGUGUGUGUAUGUGUGUGUAU chr8 77879071 . C U 0.000199681 - 16

hsa-mir-3149
AUACAUACAUGUACACACACAUGUCAUCCACACACAUACAUAUAUAUAUGUUUGUAUGGAUAUGUGUGUGUAUGUGUGUGUAU chr8 77879051 . A G 0.000199681 - 36

hsa-mir-3149
AUACAUACAUGUACACACACAUGUCAUCCACACACAUACAUAUAUAUAUGUUUGUAUGGAUAUGUGUGUGUAUGUGUGUGUAU chr8 77879011 . G U 0.000199681 - 76

hsa-mir-3150a
GGGAAGCAGGCCAACCUCGACGAUCUCCUCAGCACCUGAACGCCAAGGCUGGGGAGAUCCUCGAGGUUGGCCUGCUUUCC chr8 96085190 rs372692847 C U 0.000599042 +

49
hsa-mir-3150b
GAGGGAAAGCAGGCCAACCUCGAGGAUCUCCCCAGCCUUGGCGUUCAGGUGCUGAGGAGAUCGUCGAG GUUGGCCUGCUUCCCCUC chr8 96085190 rs372692847 G A
0.000599042 - 35
hsa-mir-3151
GGGGUGAUGGGUGGGGCAAUGGGAUCAGGUGCCUCAAAGGGCAUCCCACCUGAUCCCACAGCCCACCU GUCACCCC chr8 104166899 rs117403455 A C null null 58

hsa-mir-3151
GGGGUGAUGGGUGGGGCAAUGGGAUCAGGUGCCUCAAAGGGCAUCCCACCUGAUCCCACAGCCCACCU GUCACCCC chr8 104166902 rs35605502 - C null null 61

hsa-mir-3151
GGGGUGAUGGGUGGGGCAAUGGGAUCAGGUGCCUCAAAGGGCAUCCCACCUGAUCCCACAGCCCACCU GUCACCCC chr8 104166904 rs72544670 - C null null 63

hsa-mir-3152
GUGCAGAGUUAUUGCCUCUGUUCUAACACAAGACUAGGCUUCCCUGUGUUAGAAUAGGGGCAAUAACU CUGCAC chr9 18573331 . A G 0.000199681 + 28

hsa-mir-3152
GUGCAGAGUUAUUGCCUCUGUUCUAACACAAGACUAGGCUUCCCUGUGUUAGAAUAGGGGCAAUAACU CUGCAC chr9 18573360 rs13299349 G A 0.219249 + 57

hsa-mir-3152
GUGCAGAGUUAUUGCCUCUGUUCUAACACAAGACUAGGCUUCCCUGUGUUAGAAUAGGGGCAAUAACU CUGCAC chr9 18573361 . G C 0.000199681 + 58

hsa-mir-3152
GUGCAGAGUUAUUGCCUCUGUUCUAACACAAGACUAGGCUUCCCUGUGUUAGAAUAGGGGCAAUAACU CUGCAC chr9 18573374 rs113234240 G U null null 71

hsa-mir-3153
GACAAAUUUUAAAUGUCCCUGUCCCCUUCCCCCCAAUUAAAGUAGAUUGGGGGAAAGCGAGUAGGGAC AUUUAAAAUUUGUU chr9 91927158 . C U 0.000199681 +
19
hsa-mir-3153
GACAAAUUUUAAAUGUCCCUGUCCCCUUCCCCCCAAUUAAAGUAGAUUGGGGGAAAGCGAGUAGGGAC AUUUAAAAUUUGUU chr9 91927175 rs185637376 A G
0.000599042 + 36
hsa-mir-3154
GGCCCCUCCUUCUCAGCCCCAGCUCCCGCUCACCCCUGCCACGUCAAAGGAGGCAGAAGGGGAGUUGG GAGCAGAGAGGGGACC chr9 131007283 . C U 0.000399361 -
27
hsa-mir-3154
GGCCCCUCCUUCUCAGCCCCAGCUCCCGCUCACCCCUGCCACGUCAAAGGAGGCAGAAGGGGAGUUGG

GAGCAGAGAGGGGACC chr9 131007245 . U C 0.000599042 -
65
hsa-mir-3155a
UCCGGGCAUCACCUCCCACUGCAGAGCCUGGGGAGCCGGACAGCUCCCUUCCCAGGCUCUGCAGUGGG
AACUGAUGCCUGGA chr10 6194231 rs140190031 G C
0.000199681 + 73
hsa-mir-3156-1
GCAGAAGAAAGAUCUGGAAGUGGGAGACACUUUUACUAUAUAUAGUGGCUCCCACUUCCAGAUCUUUC
UCUCUGU chr10 45659490 . A U 0.000199681 + 29

hsa-mir-3156-1
GCAGAAGAAAGAUCUGGAAGUGGGAGACACUUUUACUAUAUAUAGUGGCUCCCACUUCCAGAUCUUUC
UCUCUGU chr10 45659500 . U C 0.00119808 + 39

hsa-mir-3156-2
UGCAGAAGAAAGAUCUGGAAGUGGGAGACACUUUCACUAUAUAUAGUGGCUCCCACUUCCAGAUCUUU
CUCUCUGUA chr18 14830172 . G A 0.000399361 + 8

hsa-mir-3156-2
UGCAGAAGAAAGAUCUGGAAGUGGGAGACACUUUCACUAUAUAUAGUGGCUCCCACUUCCAGAUCUUU
CUCUCUGUA chr18 14830197 . U C 0.000199681 + 33

hsa-mir-3156-2
UGCAGAAGAAAGAUCUGGAAGUGGGAGACACUUUCACUAUAUAUAGUGGCUCCCACUUCCAGAUCUUU
CUCUCUGUA chr18 14830201 . C G 0.000199681 + 37

hsa-mir-3156-2
UGCAGAAGAAAGAUCUGGAAGUGGGAGACACUUUCACUAUAUAUAGUGGCUCCCACUUCCAGAUCUUU
CUCUCUGUA chr18 14830215 rs113478966 U C null null 51

hsa-mir-3156-2
UGCAGAAGAAAGAUCUGGAAGUGGGAGACACUUUCACUAUAUAUAGUGGCUCCCACUUCCAGAUCUUU
CUCUCUGUA chr18 14830216 rs112428304 C U null null 52

hsa-mir-3156-3
UGCAGAAGAAAGAUCUGGAAGUGGGAGACACUUUCACUAUAUAUAGUGGCUCCCACUUCCUGAUCUUU
CUCUCUGUA chr21 14778721 rs2747232 U A 0.965256 -
61
hsa-mir-3156-3
UGCAGAAGAAAGAUCUGGAAGUGGGAGACACUUUCACUAUAUAUAGUGGCUCCCACUUCCUGAUCUUU
CUCUCUGUA chr21 14778711 rs112248965 C G 0.000998403 -
71
hsa-mir-3157
GGGAAGGGCUUCAGCCAGGCUAGUGCAGUCUGCUUUGUGCCAACACUGGGGUGAUGACUGCCCUAGUC
UAGCUGAAGCUUUUCCC chr10 97824145 rs141770574 C U
0.000599042 - 12
hsa-mir-3157

GGGAAGGGCUUCAGCCAGGCUAGUGCAGUCUGCUUUGUGCCAACACUGGGGUGAUGACUGCCCUAGUC UAGCUGAAGCUUUUCCC chr10 97824129 rs189826351 G C 0.000199681 - 28

hsa-mir-3157
GGGAAGGGCUUCAGCCAGGCUAGUGCAGUCUGCUUUGUGCCAACACUGGGGUGAUGACUGCCCUAGUC UAGCUGAAGCUUUUCCC chr10 97824125 . G A 0.000199681 - 32

hsa-mir-3157
GGGAAGGGCUUCAGCCAGGCUAGUGCAGUCUGCUUUGUGCCAACACUGGGGUGAUGACUGCCCUAGUC UAGCUGAAGCUUUUCCC chr10 97824075 . U C 0.000199681 - 82

hsa-mir-3158-1
AUUCAGGCCGGUCCUGCAGAGAGGAAGCCCUUCUGCUUACAGGUAUUGGAAGGGCUUCCUCUCUGCAG GACCGGCCUGAAUchr10 103361221 rs112037539 G A null null 48

hsa-mir-3158-1
AUUCAGGCCGGUCCUGCAGAGAGGAAGCCCUUCUGCUUACAGGUAUUGGAAGGGCUUCCUCUCUGCAG GACCGGCCUGAAUchr10 103361226 . G C 0.000199681 + 53

hsa-mir-3158-1
AUUCAGGCCGGUCCUGCAGAGAGGAAGCCCUUCUGCUUACAGGUAUUGGAAGGGCUUCCUCUCUGCAG GACCGGCCUGAAUchr10 103361245 . C U 0.000199681 + 72

hsa-mir-3158-2
AUUCAGGCCGGUCCUGCAGAGAGGAAGCCCUUCCAAUACCUGUAAGCAGAAGGGCUUCCUCUCUGCAG GACCGGCCUGAAUchr10 103361245 . G A 0.000199681 - 10

hsa-mir-3158-2
AUUCAGGCCGGUCCUGCAGAGAGGAAGCCCUUCCAAUACCUGUAAGCAGAAGGGCUUCCUCUCUGCAG GACCGGCCUGAAUchr10 103361226 . C G 0.000199681 - 29

hsa-mir-3158-2
AUUCAGGCCGGUCCUGCAGAGAGGAAGCCCUUCCAAUACCUGUAAGCAGAAGGGCUUCCUCUCUGCAG GACCGGCCUGAAUchr10 103361221 rs112037539 C U null null 34

hsa-mir-3159
CCAAAGUCCUAGGAUUACAAGUGUCGGCCACGGGCUGGGCACAGUGGCUCACGCCUGUAAUCCCAGCA UUUUGG chr11 18409354 rs79957895 G A 0.0235623 + 21

hsa-mir-3161
CCUCGAGAGCUGAUAAGAACAGAGGCCCAGAUUGAAGUUGAAUAGUGCUGGGCCUUUGUUUUUACCAA GUUCCCUGG chr11 48118347 rs113098367 - A null null 14

hsa-mir-3161
CCUCGAGAGCUGAUAAGAACAGAGGCCCAGAUUGAAGUUGAAUAGUGCUGGGCCUUUGUUUUUACCAA GUUCCCUGG chr11 48118349 rs11382316 - A null null 16

hsa-mir-3161
CCUCGAGAGCUGAUAAGAACAGAGGCCCAGAUUGAAGUUGAAUAGUGCUGGGCCUUUGUUUUUACCAA
GUUCCCUGG chr11 48118350 rs35834266 - A null null 17

hsa-mir-3161
CCUCGAGAGCUGAUAAGAACAGAGGCCCAGAUUGAAGUUGAAUAGUGCUGGGCCUUUGUUUUUACCAA
GUUCCCUGG chr11 48118351 rs74581179 A G null null 18

hsa-mir-3161
CCUCGAGAGCUGAUAAGAACAGAGGCCCAGAUUGAAGUUGAAUAGUGCUGGGCCUUUGUUUUUACCAA
GUUCCCUGG chr11 48118365 rs190080389 U C 0.000199681 +
32
hsa-mir-3161
CCUCGAGAGCUGAUAAGAACAGAGGCCCAGAUUGAAGUUGAAUAGUGCUGGGCCUUUGUUUUUACCAA
GUUCCCUGG chr11 48118374 rs73466882 A U 0.0163738 +
41
hsa-mir-3162
CUGACUUUUUUAGGGAGUAGAAGGGUGGGGAGCAUGAACAAUGUUUCUCACUCCCUACCCCUCCACUC
CCCAAAAAAGUCAG chr11 59362602 . G A 0.000599042 -
30
hsa-mir-3162
CUGACUUUUUUAGGGAGUAGAAGGGUGGGGAGCAUGAACAAUGUUUCUCACUCCCUACCCCUCCACUC
CCCAAAAAAGUCAG chr11 59362576 . U A 0.00119808 -
56
hsa-mir-3163
UUCCUCAUCUAUAAAAUGAGGGCAGUAAGACCUUCCUUCCUUGUCUUACUACCCCCAUUUUAUAGAUG
AGGAA chr11 66701974 . C U 0.000399361 - 4

hsa-mir-3163
UUCCUCAUCUAUAAAAUGAGGGCAGUAAGACCUUCCUUCCUUGUCUUACUACCCCCAUUUUAUAGAUG
AGGAA chr11 66701926 . C U 0.000199681 - 52

hsa-mir-3163
UUCCUCAUCUAUAAAAUGAGGGCAGUAAGACCUUCCUUCCUUGUCUUACUACCCCCAUUUUAUAGAUG
AGGAA chr11 66701915 . U C 0.000199681 - 63

hsa-mir-3164
CUUGGAAACUGUGACUUUAAGGGAAAUGGCGCACAGCAGACCCUGCAAUCAUGCCGUUUUGCUUGAAG
UCGCAGUUUCCCAGG chr11 68850698 . C U 0.000199681 +
55
hsa-mir-3164
CUUGGAAACUGUGACUUUAAGGGAAAUGGCGCACAGCAGACCCUGCAAUCAUGCCGUUUUGCUUGAAG
UCGCAGUUUCCCAGG chr11 68850706 . U C 0.000199681 +
63
hsa-mir-3164
CUUGGAAACUGUGACUUUAAGGGAAAUGGCGCACAGCAGACCCUGCAAUCAUGCCGUUUUGCUUGAAG
UCGCAGUUUCCCAGG chr11 68850720 . U C 0.00139776 +

77
hsa-mir-3166
AAAUUUUUUUGAGGCCAGUAGGCAUUGUCUGCGUUAGGAUUUCUGUAUCAUCCUCCUAACGCAGACAA
UGCCUACUGGCCUAAGAAAAAUUU chr11 87909673 rs35854553 U A
0.107029 + 4
hsa-mir-3166
AAAUUUUUUUGAGGCCAGUAGGCAUUGUCUGCGUUAGGAUUUCUGUAUCAUCCUCCUAACGCAGACAA
UGCCUACUGGCCUAAGAAAAAUUU chr11 87909701 . C U
0.000199681 + 32
hsa-mir-3166
AAAUUUUUUUGAGGCCAGUAGGCAUUGUCUGCGUUAGGAUUUCUGUAUCAUCCUCCUAACGCAGACAA
UGCCUACUGGCCUAAGAAAAAUUU chr11 87909716 rs192612757 A G
0.000199681 + 47
hsa-mir-3166
AAAUUUUUUUGAGGCCAGUAGGCAUUGUCUGCGUUAGGAUUUCUGUAUCAUCCUCCUAACGCAGACAA
UGCCUACUGGCCUAAGAAAAAUUU chr11 87909749 . C U
0.000199681 + 80
hsa-mir-3167
GGCUGUGGAGGCACCAGUAUUUCUGAAAUUCUUUUUUCUGAAAUUCUUCAGGAAGGAUUUCAGAAAUA
CUGGUGUCCCGACAGCC chr11 126858425 . C G 0.00239617 -
14
hsa-mir-3167
GGCUGUGGAGGCACCAGUAUUUCUGAAAUUCUUUUUUCUGAAAUUCUUCAGGAAGGAUUUCAGAAAUA
CUGGUGUCCCGACAGCC chr11 126858406 rs634171 U A 0.189297
- 33
hsa-mir-3167
GGCUGUGGAGGCACCAGUAUUUCUGAAAUUCUUUUUUCUGAAAUUCUUCAGGAAGGAUUUCAGAAAUA
CUGGUGUCCCGACAGCC chr11 126858392 rs670637 U C 0.189297
- 47
hsa-mir-3168
AAGAUCAUGAGUUCUACAGUCAGACAGCCUGAGUUGGAGGCUCAUCUUCACUUCUUGCUGUGUGACCC
UGGGCCAGUGACUU chr13 41675171 rs114586251 C U
0.000599042 - 66
hsa-mir-3169
AUGUGAAAACAUAGGACUGUGCUUGGCACAUAGCACAAAGUCUUAUGGUACUGUGUGCCAAGCAUAGU
CCUGUGUUUUUACAU chr13 61773937 . U C 0.000199681 -
78
hsa-mir-3170
CUGGUAACACUGGGGUUCUGAGACAGACAGUGUUAGCUCCAGAAGCAUUGCCUGUCUUAGAACCCCUA
UGUUACCAG chr13 98860801 . C U 0.000798722 + 24

hsa-mir-3170
CUGGUAACACUGGGGUUCUGAGACAGACAGUGUUAGCUCCAGAAGCAUUGCCUGUCUUAGAACCCCUA
UGUUACCAG chr13 98860808 rs147240207 U C 0.00259585 +
31
hsa-mir-3170
CUGGUAACACUGGGGUUCUGAGACAGACAGUGUUAGCUCCAGAAGCAUUGCCUGUCUUAGAACCCCUA

UGUUACCAG chr13 98860816 rs139676422 C G 0.0107827 +
39
hsa-mir-3170
CUGGUAACACUGGGGUUCUGAGACAGACAGUGUUAGCUCCAGAAGCAUUGCCUGUCUUAGAACCCCUA
UGUUACCAG chr13 98860827 rs76289985 G C 0.0107827 +
50
hsa-mir-3170
CUGGUAACACUGGGGUUCUGAGACAGACAGUGUUAGCUCCAGAAGCAUUGCCUGUCUUAGAACCCCUA
UGUUACCAG chr13 98860840 . C G 0.000399361 + 63

hsa-mir-3171
UAUAUAUAGAGAUGUAUGGAAUCUGUAUAUAUCUAUAUAUAUGUGUAUAUAUAGAUUCCAUAAAUCUA
UAUAUG chr14 28102484 rs35803920 U C null null 1

hsa-mir-3171
UAUAUAUAGAGAUGUAUGGAAUCUGUAUAUAUCUAUAUAUAUGUGUAUAUAUAGAUUCCAUAAAUCUA
UAUAUG chr14 28102484 rs67062245 AU - null null 1

hsa-mir-3171
UAUAUAUAGAGAUGUAUGGAAUCUGUAUAUAUCUAUAUAUAUGUGUAUAUAUAGAUUCCAUAAAUCUA
UAUAUG chr14 28102477 rs35170395 UA - null null 8

hsa-mir-3171
UAUAUAUAGAGAUGUAUGGAAUCUGUAUAUAUCUAUAUAUAUGUGUAUAUAUAGAUUCCAUAAAUCUA
UAUAUG chr14 28102427 rs61972532 C U 0.000798722 - 58

hsa-mir-3173
UCCCUGCCCUGCCUGUUUUCUCCUUUGUGAUUUUAUGAGAACAAAGGAGGAAAUAGGCAGGCCAGGGA
chr14 95604259 rs200801744 G A 0.000399361 - 65

hsa-mir-3175
CCUGGGGGGCGGGGAGAGAACGCAGUGACGUCUGGCCGCGUGCGCAUGUCGGGCGCUUUCUCCUCCCC
CUACCCAGG chr15 93447631 rs1439619 U G 0.414936 +
3
hsa-mir-3175
CCUGGGGGGCGGGGAGAGAACGCAGUGACGUCUGGCCGCGUGCGCAUGUCGGGCGCUUUCUCCUCCCC
CUACCCAGG chr15 93447646 rs72647784 GA - null null 18

hsa-mir-3175
CCUGGGGGGCGGGGAGAGAACGCAGUGACGUCUGGCCGCGUGCGCAUGUCGGGCGCUUUCUCCUCCCC
CUACCCAGG chr15 93447674 rs114901994 A G 0.0299521 +
46
hsa-mir-3175
CCUGGGGGGCGGGGAGAGAACGCAGUGACGUCUGGCCGCGUGCGCAUGUCGGGCGCUUUCUCCUCCCC
CUACCCAGG chr15 93447695 . C U 0.000199681 + 67

hsa-mir-3175

CCUGGGGGGCGGGGAGAGAACGCAGUGACGUCUGGCCGCGUGCGCAUGUCGGGCGCUUUCUCCUCCCC CUACCCAGG chr15 93447700 . C G 0.00139776 + 72

hsa-mir-3175 CCUGGGGGGCGGGGAGAGAACGCAGUGACGUCUGGCCGCGUGCGCAUGUCGGGCGCUUUCUCCUCCCC CUACCCAGG chr15 93447702 . C U 0.00319489 + 74

hsa-mir-3176 UGGCCUCUCCAGUCUGCAGCUCCCGGCAGCCUCGGGCCACACUCCCGGGAUCCCCAGGGACUGGCCUG GGACUACCGGGGGUGGCGGCCG chr16 593277 rs8054514 U G 0.224042 + 1

hsa-mir-3176 UGGCCUCUCCAGUCUGCAGCUCCCGGCAGCCUCGGGCCACACUCCCGGGAUCCCCAGGGACUGGCCUG GGACUACCGGGGGUGGCGGCCG chr16 593323 rs191496762 G A 0.00179712 + 47

hsa-mir-3176 UGGCCUCUCCAGUCUGCAGCUCCCGGCAGCCUCGGGCCACACUCCCGGGAUCCCCAGGGACUGGCCUG GGACUACCGGGGGUGGCGGCCG chr16 593362 rs141861277 G A 0.00419329 + 86

hsa-mir-3176 UGGCCUCUCCAGUCUGCAGCUCCCGGCAGCCUCGGGCCACACUCCCGGGAUCCCCAGGGACUGGCCUG GGACUACCGGGGGUGGCGGCCG chr16 593365 . C U 0.000399361 + 89

hsa-mir-3177 CCACGUGCCAUGUGUACACACGUGCCAGGCGCUGUCUUGAGACAUUCGCGCAGUGCACGGCACUGGGG ACACGUGGCACUGG chr16 1785015 rs145083637 C U 0.00239617 + 30

hsa-mir-3177 CCACGUGCCAUGUGUACACACGUGCCAGGCGCUGUCUUGAGACAUUCGCGCAGUGCACGGCACUGGGG ACACGUGGCACUGG chr16 1785030 . U G 0.000199681 + 45

hsa-mir-3177 CCACGUGCCAUGUGUACACACGUGCCAGGCGCUGUCUUGAGACAUUCGCGCAGUGCACGGCACUGGGG ACACGUGGCACUGG chr16 1785037 . A G 0.000199681 + 52

hsa-mir-3177 CCACGUGCCAUGUGUACACACGUGCCAGGCGCUGUCUUGAGACAUUCGCGCAGUGCACGGCACUGGGG ACACGUGGCACUGG chr16 1785038 rs28575325 G A null null 53

hsa-mir-3177 CCACGUGCCAUGUGUACACACGUGCCAGGCGCUGUCUUGAGACAUUCGCGCAGUGCACGGCACUGGGG ACACGUGGCACUGG chr16 1785042 . A G 0.000399361 + 57

hsa-mir-3177 CCACGUGCCAUGUGUACACACGUGCCAGGCGCUGUCUUGAGACAUUCGCGCAGUGCACGGCACUGGGG ACACGUGGCACUGG chr16 1785060 rs149035589 G A 0.00119808 + 75

hsa-mir-3178
GAGGCUGGGCGGGGCGCGGCCGGAUCGGUCGAGAGCGUCCUGGCUGAUGACGGUCUCCCGUGCCCACG
CCCCAAACGCAGUCUC chr16 2581992 . C A 0.000199681 -
15
hsa-mir-3178
GAGGCUGGGCGGGGCGCGGCCGGAUCGGUCGAGAGCGUCCUGGCUGAUGACGGUCUCCCGUGCCCACG
CCCCAAACGCAGUCUC chr16 2581984 . G C 0.00119808 -
23
hsa-mir-3178
GAGGCUGGGCGGGGCGCGGCCGGAUCGGUCGAGAGCGUCCUGGCUGAUGACGGUCUCCCGUGCCCACG
CCCCAAACGCAGUCUC chr16 2581955 . G C 0.000199681 -
52
hsa-mir-3178
GAGGCUGGGCGGGGCGCGGCCGGAUCGGUCGAGAGCGUCCUGGCUGAUGACGGUCUCCCGUGCCCACG
CCCCAAACGCAGUCUC chr16 2581952 rs183696444 C U
0.000199681 - 55
hsa-mir-3179-1
CAGGAUCACAGACGUUUAAAUUACACUCCUUCUGCUGUGCCUUACAGCAGUAGAAGGGGUGAAAUUUA
AACGUCUGUGAUCCUG chr16 14995387 . A G 0.000199681 +
23
hsa-mir-3179-4
CAGGAUCACAGACGUUUAAAUUACACUCCUUCUGCUGUGCCUUACAGCAGUAGAAGGGGUGAAAUUUA
AACGUCUGUGAUCCUG chr16 14995387 . U C 0.000199681 -
61
hsa-mir-3180-4
GCUCCGCCCCACGUCGCAUGCGCCCCGGGAACGCGUGGGGCGGAGCUUCCGGAGGCCCCGCUCUGCUG
CCGACCCUGUGGAGCGGAGGGUGAAGCCUCCGGAUGCCAGUCCCUCAUCGCUGGCCUGGUCGCGCUGUGGCGAAG
GGGGCGGAGC chr16 15248801 . C A 0.00259585 - 59

hsa-mir-3180-4
GCUCCGCCCCACGUCGCAUGCGCCCCGGGAACGCGUGGGGCGGAGCUUCCGGAGGCCCCGCUCUGCUG
CCGACCCUGUGGAGCGGAGGGUGAAGCCUCCGGAUGCCAGUCCCUCAUCGCUGGCCUGGUCGCGCUGUGGCGAAG
GGGGCGGAGC chr16 15248798 rs183853838 U C 0.730032 -
62
hsa-mir-3180-4
GCUCCGCCCCACGUCGCAUGCGCCCCGGGAACGCGUGGGGCGGAGCUUCCGGAGGCCCCGCUCUGCUG
CCGACCCUGUGGAGCGGAGGGUGAAGCCUCCGGAUGCCAGUCCCUCAUCGCUGGCCUGGUCGCGCUGUGGCGAAG
GGGGCGGAGC chr16 15248787 . C U 0.00119808 - 73

hsa-mir-3180-4
GCUCCGCCCCACGUCGCAUGCGCCCCGGGAACGCGUGGGGCGGAGCUUCCGGAGGCCCCGCUCUGCUG
CCGACCCUGUGGAGCGGAGGGUGAAGCCUCCGGAUGCCAGUCCCUCAUCGCUGGCCUGGUCGCGCUGUGGCGAAG
GGGGCGGAGC chr16 15248717 rs5016497 G C null null 127

hsa-mir-3180-4
GCUCCGCCCCACGUCGCAUGCGCCCCGGGAACGCGUGGGGCGGAGCUUCCGGAGGCCCCGCUCUGCUG
CCGACCCUGUGGAGCGGAGGGUGAAGCCUCCGGAUGCCAGUCCCUCAUCGCUGGCCUGGUCGCGCUGUGGCGAAG

GGGGCGGAGC chr16 15248726 . U A 0.000199681 - 134

hsa-mir-3180-4
GCUCCGCCCCACGUCGCAUGCGCCCCGGGAACGCGUGGGGCGGAGCUUCCGGAGGCCCCGCUCUGCUG
CCGACCCUGUGGAGCGGAGGGUGAAGCCUCCGGAUGCCAGUCCCUCAUCGCUGGCCUGGUCGCGCUGUGGCGAAG
GGGGCGGAGC chr16 15248720 rs75000738 G U 0.652955 -
140

hsa-mir-3180-5
GCUCCGCCCCACGUCGCAUGCGCCCCGGGAACGCGUGGGGCGGAGCUUCCGGAGGCCCCGCCCUGCUG
CCGACCCUGUGGAGCGGAGGGUGAAGCCUCCGGAUGCCAGUCCCUCAUCGCUGGCCCGGUCGCGCUGUGGCGAAG
GGGGCGGAGC chr16 2186129 rs2021041 C U null null 2

hsa-mir-3180-5
GCUCCGCCCCACGUCGCAUGCGCCCCGGGAACGCGUGGGGCGGAGCUUCCGGAGGCCCCGCCCUGCUG
CCGACCCUGUGGAGCGGAGGGUGAAGCCUCCGGAUGCCAGUCCCUCAUCGCUGGCCCGGUCGCGCUGUGGCGAAG
GGGGCGGAGC chr16 2186117 . U C 0.00339457 - 14

hsa-mir-3180-5
GCUCCGCCCCACGUCGCAUGCGCCCCGGGAACGCGUGGGGCGGAGCUUCCGGAGGCCCCGCCCUGCUG
CCGACCCUGUGGAGCGGAGGGUGAAGCCUCCGGAUGCCAGUCCCUCAUCGCUGGCCCGGUCGCGCUGUGGCGAAG
GGGGCGGAGC chr16 2186087 . A G 0.000199681 - 44

hsa-mir-3180-5
GCUCCGCCCCACGUCGCAUGCGCCCCGGGAACGCGUGGGGCGGAGCUUCCGGAGGCCCCGCCCUGCUG
CCGACCCUGUGGAGCGGAGGGUGAAGCCUCCGGAUGCCAGUCCCUCAUCGCUGGCCCGGUCGCGCUGUGGCGAAG
GGGGCGGAGC chr16 2186069 rs78681286 C U null null 62

hsa-mir-3180-5
GCUCCGCCCCACGUCGCAUGCGCCCCGGGAACGCGUGGGGCGGAGCUUCCGGAGGCCCCGCCCUGCUG
CCGACCCUGUGGAGCGGAGGGUGAAGCCUCCGGAUGCCAGUCCCUCAUCGCUGGCCCGGUCGCGCUGUGGCGAAG
GGGGCGGAGC chr16 2186054 rs2855372 G U null null 77

hsa-mir-3180-5
GCUCCGCCCCACGUCGCAUGCGCCCCGGGAACGCGUGGGGCGGAGCUUCCGGAGGCCCCGCCCUGCUG
CCGACCCUGUGGAGCGGAGGGUGAAGCCUCCGGAUGCCAGUCCCUCAUCGCUGGCCCGGUCGCGCUGUGGCGAAG
GGGGCGGAGC chr16 2186044 . G A 0.000998403 - 87

hsa-mir-3182
GCUGCUUCUGUAGUGUAGUCCGUGCAUCCGCCCUUCGAUGCUUGGGUUGGAUCAUAGAGCAGU
chr16 83541972 . G A 0.000199681 + 22

hsa-mir-3182
GCUGCUUCUGUAGUGUAGUCCGUGCAUCCGCCCUUCGAUGCUUGGGUUGGAUCAUAGAGCAGU
chr16 83541979 rs376190368 C U 0.000199681 + 29

hsa-mir-3182
GCUGCUUCUGUAGUGUAGUCCGUGCAUCCGCCCUUCGAUGCUUGGGUUGGAUCAUAGAGCAGU

chr16 83541980 rs146394071 G A 0.000199681 + 30

hsa-mir-3182
GCUGCUUCUGUAGUGUAGUCCGUGCAUCCGCCCUUCGAUGCUUGGGUUGGAUCAUAGAGCAGU
chr16 83541990 rs139736497 G A 0.000998403 + 40

hsa-mir-3183
CUCUGCCCUGCCUCUCUCGGAGUCGCUCGGAGCAGUCACGUUGACGGAAUCCUCCGGCGCCUCCUCGA
GGGAGGAGAGGCAGGG chr17 925781 . G A 0.000199681 - 19

hsa-mir-3183
CUCUGCCCUGCCUCUCUCGGAGUCGCUCGGAGCAGUCACGUUGACGGAAUCCUCCGGCGCCUCCUCGA
GGGAGGAGAGGCAGGG chr17 925776 rs111737188 C U 0.000399361 -
24

hsa-mir-3183
CUCUGCCCUGCCUCUCUCGGAGUCGCUCGGAGCAGUCACGUUGACGGAAUCCUCCGGCGCCUCCUCGA
GGGAGGAGAGGCAGGG chr17 925772 . C U 0.000199681 - 28

hsa-mir-3183
CUCUGCCCUGCCUCUCUCGGAGUCGCUCGGAGCAGUCACGUUGACGGAAUCCUCCGGCGCCUCCUCGA
GGGAGGAGAGGCAGGG chr17 925764 rs2663345 U C 0.503594 -
36

hsa-mir-3183
CUCUGCCCUGCCUCUCUCGGAGUCGCUCGGAGCAGUCACGUUGACGGAAUCCUCCGGCGCCUCCUCGA
GGGAGGAGAGGCAGGG chr17 925755 . C U 0.000199681 - 45

hsa-mir-3183
CUCUGCCCUGCCUCUCUCGGAGUCGCUCGGAGCAGUCACGUUGACGGAAUCCUCCGGCGCCUCCUCGA
GGGAGGAGAGGCAGGG chr17 925742 rs72812091 C U 0.0227636 -
58

hsa-mir-3184
AAGCAAGACUGAGGGGCCUCAGACCGAGCUUUUGGAAAAUAGAAAAGUCUCGCUCUCUGCCCCUCAGC
CUAACUU chr17 28444157 . G A 0.000199681 - 22

hsa-mir-3184
AAGCAAGACUGAGGGGCCUCAGACCGAGCUUUUGGAAAAUAGAAAAGUCUCGCUCUCUGCCCCUCAGC
CUAACUU chr17 28444104 . U A 0.000199681 - 75

hsa-mir-3185
GAAUGGAAGAAGAAGGCGGUCGGUCUGCGGGAGCCAGGCCGCAGAGCCAUCCGCCUUCUGUCCAUGUC
chr17 46801821 rs376980892 C U 0.000399361 - 17

hsa-mir-3186
AGCCUGCGGUUCCAACAGGCGUCUGUCUACGUGGCUUCAACCAAGUUCAAAGUCACGCGGAGAGAUGG
CUUUGGAACCAGGGGCU chr17 79418149 rs185669377 U C
0.000199681 - 66

hsa-mir-3188

GGCGCCUCCUGCUCUGCUGUGCCGCCAGGGCCUCCCCUAGCGCGCCUUCUGGAGAGGCUUUGUGCGGA
UACGGGGCUGGAGGCCU chr19 18392894 rs7247237 C U null
null 8
hsa-mir-3188
GGCGCCUCCUGCUCUGCUGUGCCGCCAGGGCCUCCCCUAGCGCGCCUUCUGGAGAGGCUUUGUGCGGA
UACGGGGCUGGAGGCCU chr19 18392913 rs7247767 A G null
null 27
hsa-mir-3188
GGCGCCUCCUGCUCUGCUGUGCCGCCAGGGCCUCCCCUAGCGCGCCUUCUGGAGAGGCUUUGUGCGGA
UACGGGGCUGGAGGCCU chr19 18392936 rs41510649 U C null
null 50
hsa-mir-3192
GGAAGGGAUUCUGGGAGGUUGUAGCAGUGGAAAAAGUUCUUUUCUUCCUCUGAUCGCCCUCUCAGCUC
UUUCCUUCU chr20 18451300 rs6111978 U C null null 42

hsa-mir-3192
GGAAGGGAUUCUGGGAGGUUGUAGCAGUGGAAAAAGUUCUUUUCUUCCUCUGAUCGCCCUCUCAGCUC
UUUCCUUCU chr20 18451325 rs11907020 U C null null 67

hsa-mir-3195
CCGCAGCCGCCGCGCCGGGCCCGGGUUGGCCGCUGACCCCCGCGGGGCCCCCGGCGGCCGGGGCGGGG
GCGGGGGCUGCCCCGG chr20 60639883 rs1886009 U C null
null 26
hsa-mir-3196
GGGUGGGGGCGGGGCGGCAGGGGCCUCCCCCAGUGCCAGGCCCCAUUCUGCUUCUCUCCCAGCU
chr20 61870140 rs2273488 C U null null 10

hsa-mir-3196
GGGUGGGGGCGGGGCGGCAGGGGCCUCCCCCAGUGCCAGGCCCCAUUCUGCUUCUCUCCCAGCU
chr20 61870141 rs113297757 G A null null 11

hsa-mir-3196
GGGUGGGGGCGGGGCGGCAGGGGCCUCCCCCAGUGCCAGGCCCCAUUCUGCUUCUCUCCCAGCU
chr20 61870162 rs7363975 A C null null 32

hsa-mir-3196
GGGUGGGGGCGGGGCGGCAGGGGCCUCCCCCAGUGCCAGGCCCCAUUCUGCUUCUCUCCCAGCU
chr20 61870167 rs744591 C A null null 37

hsa-mir-3199-1
GGUGACUCCAGGGACUGCCUUAGGAGAAAGUUUCUGGAAGUUCUGACAUUCCAGAAACUUUCUCCUAA
GGCAGUCCCUGGGAGUCACU chr22 28316597 rs117221779 G C
null null 4
hsa-mir-3199-1
GGUGACUCCAGGGACUGCCUUAGGAGAAAGUUUCUGGAAGUUCUGACAUUCCAGAAACUUUCUCCUAA
GGCAGUCCCUGGGAGUCACU chr22 28316592 rs75321888 - C
null null 9

hsa-mir-3199-1
GGUGACUCCAGGGACUGCCUUAGGAGAAAGUUUCUGGAAGUUCUGACAUUCCAGAAACUUUCUCCUAA
GGCAGUCCCUGGGAGUCACU chr22 28316591 rs78805657 A C
null null 10
hsa-mir-3199-1
GGUGACUCCAGGGACUGCCUUAGGAGAAAGUUUCUGGAAGUUCUGACAUUCCAGAAACUUUCUCCUAA
GGCAGUCCCUGGGAGUCACU chr22 28316513 rs118160653 U C
null null 88
hsa-mir-3199-2
GUGACUCCCAGGGACUGCCUUAGGAGAAAGUUUCUGGAAUGUCAGAACUUCCAGAAACUUUCUCCUAA
GGCAGUCCCUGGAGUCAC chr22 28316591 rs78805657 U G null
null 78
hsa-mir-3199-2
GUGACUCCCAGGGACUGCCUUAGGAGAAAGUUUCUGGAAUGUCAGAACUUCCAGAAACUUUCUCCUAA
GGCAGUCCCUGGAGUCAC chr22 28316591 rs80166589 - G null
null 78
hsa-mir-3199-2
GUGACUCCCAGGGACUGCCUUAGGAGAAAGUUUCUGGAAUGUCAGAACUUCCAGAAACUUUCUCCUAA
GGCAGUCCCUGGAGUCAC chr22 28316592 rs75321888 - G null
null 79
hsa-mir-3199-2
GUGACUCCCAGGGACUGCCUUAGGAGAAAGUUUCUGGAAUGUCAGAACUUCCAGAAACUUUCUCCUAA
GGCAGUCCCUGGAGUCAC chr22 28316597 rs117221779 C G null
null 84
hsa-mir-320a
GCUUCGCUCCCCUCCGCCUUCUCUUCCCGGUUCUUCCCGGAGUCGGGAAAAGCUGGGUUGAGAGGGCG
AAAAAGGAUGAGGU chr8 22102546 rs372063644 C U
0.000199681 - 11
hsa-mir-320a
GCUUCGCUCCCCUCCGCCUUCUCUUCCCGGUUCUUCCCGGAGUCGGGAAAAGCUGGGUUGAGAGGGCG
AAAAAGGAUGAGGU chr8 22102545 . C U 0.000798722 -
12
hsa-mir-320a
GCUUCGCUCCCCUCCGCCUUCUCUUCCCGGUUCUUCCCGGAGUCGGGAAAAGCUGGGUUGAGAGGGCG
AAAAAGGAUGAGGU chr8 22102543 rs376536600 C U
0.000399361 - 14
hsa-mir-320a
GCUUCGCUCCCCUCCGCCUUCUCUUCCCGGUUCUUCCCGGAGUCGGGAAAAGCUGGGUUGAGAGGGCG
AAAAAGGAUGAGGU chr8 22102542 rs371824376 C U
0.000199681 - 15
hsa-mir-320a
GCUUCGCUCCCCUCCGCCUUCUCUUCCCGGUUCUUCCCGGAGUCGGGAAAAGCUGGGUUGAGAGGGCG
AAAAAGGAUGAGGU chr8 22102536 rs371890344 C U
0.000199681 - 21
hsa-mir-320a
GCUUCGCUCCCCUCCGCCUUCUCUUCCCGGUUCUUCCCGGAGUCGGGAAAAGCUGGGUUGAGAGGGCG
AAAAAGGAUGAGGU chr8 22102519 rs200301891 C U

0.000998403	-	38
hsa-mir-320b-1
AAUUAAUCCCUCUCUUUCUAGUUCUUCCUAGAGUGAGGAAAAGCUGGGUUGAGAGGGCAAACAAAUUA
ACUAAUUAAUU	chr1	117214376	.	A	G	0.000199681	+	6

hsa-mir-320c-1
UUUGCAUUAAAAAUGAGGCCUUCUCUUCCCAGUUCUUCCCAGAGUCAGGAAAAGCUGGGUUGAGAGGG
UAGAAAAAAAAUGAUGUAGG	chr18	19263542	.	A	G
0.000599042	+	72
hsa-mir-320d-1	UUCUCGUCCCAGUUCUUCCCAAAGUUGAGAAAAGCUGGGUUGAGAGGA
chr13	41302007	rs181368210	C	U	0.00159744	-	5

hsa-mir-320d-1	UUCUCGUCCCAGUUCUUCCCAAAGUUGAGAAAAGCUGGGUUGAGAGGA
chr13	41301987	rs74826059	U	C	null	null	25

hsa-mir-320d-1	UUCUCGUCCCAGUUCUUCCCAAAGUUGAGAAAAGCUGGGUUGAGAGGA
chr13	41301978	.	G	C	0	-	34

hsa-mir-320d-1	UUCUCGUCCCAGUUCUUCCCAAAGUUGAGAAAAGCUGGGUUGAGAGGA
chr13	41301975	.	G	A	0.000199681	-	37

hsa-mir-320e GCCUUCUCUUCCCAGUUCUUCCUGGAGUCGGGGAAAAGCUGGGUUGAGAAGGU
chr19	47212602	.	G	U	0.000199681	-	1

hsa-mir-320e GCCUUCUCUUCCCAGUUCUUCCUGGAGUCGGGGAAAAGCUGGGUUGAGAAGGU
chr19	47212593	rs10423365	U	C	0.460863	-	10

hsa-mir-320e GCCUUCUCUUCCCAGUUCUUCCUGGAGUCGGGGAAAAGCUGGGUUGAGAAGGU
chr19	47212591	.	C	U	0.000199681	-	12

hsa-mir-323b
UGGUACUCGGAGGGAGGUUGUCCGUGGUGAGUUCGCAUUAUUUAAUGAUGCCCAAUACACGGUCGACC
UCUUUUCGGUAUCA	chr14	101522556	rs56103835	U	C	0.297923
+	1
hsa-mir-323b
UGGUACUCGGAGGGAGGUUGUCCGUGGUGAGUUCGCAUUAUUUAAUGAUGCCCAAUACACGGUCGACC
UCUUUUCGGUAUCA	chr14	101522578	rs181310917	C	U
0.000199681	+	23
hsa-mir-323b
UGGUACUCGGAGGGAGGUUGUCCGUGGUGAGUUCGCAUUAUUUAAUGAUGCCCAAUACACGGUCGACC
UCUUUUCGGUAUCA	chr14	101522582	rs117380662	G	C	0.00299521
+	27
hsa-mir-323b
UGGUACUCGGAGGGAGGUUGUCCGUGGUGAGUUCGCAUUAUUUAAUGAUGCCCAAUACACGGUCGACC
UCUUUUCGGUAUCA	chr14	101522589	rs75330474	C	U	0.0357428
+	34
hsa-mir-323b

UGGUACUCGGAGGGAGGUUGUCCGUGGUGAGUUCGCAUUAUUUAAUGAUGCCCAAUACACGGUCGACC UCUUUUCGGUAUCA chr14 101522631 . G C 0.000199681 + 76
hsa-mir-324
CUGACUAUGCCUCCCCGCAUCCCCUAGGGCAUUGGUGUAAAGCUGGAGACCCACUGCCCCAGGUGCUG CUGGGGGUUGUAGUC chr17 7126698 rs200471575 C G 0.00559105 - 1
hsa-mir-324
CUGACUAUGCCUCCCCGCAUCCCCUAGGGCAUUGGUGUAAAGCUGGAGACCCACUGCCCCAGGUGCUG CUGGGGGUUGUAGUC chr17 7126619 . A G 0.000199681 - 80
hsa-mir-325
AUACAGUGCUUGGUUCCUAGUAGGUGUCCAGUAAGUGUUUGUGACAUAAUUUGUUUAUUGAGGACCUC CUAUCAAUCAAGCACUGUGCUAGGCUCUGG chrX 76225915 rs72631830 G U null null 12
hsa-mir-325
AUACAGUGCUUGGUUCCUAGUAGGUGUCCAGUAAGUGUUUGUGACAUAAUUUGUUUAUUGAGGACCUC CUAUCAAUCAAGCACUGUGCUAGGCUCUGG chrX 76225848 . A G 0.000264901 - 79
hsa-mir-325
AUACAGUGCUUGGUUCCUAGUAGGUGUCCAGUAAGUGUUUGUGACAUAAUUUGUUUAUUGAGGACCUC CUAUCAAUCAAGCACUGUGCUAGGCUCUGG chrX 76225837 rs184629807 A G 0.000529801 - 90
hsa-mir-326
CUCAUCUGUCUGUUGGGCUGGAGGCAGGGCCUUUGUGAAGGCGGGUGGUGCUCAGAUCGCCUCUGGGC CCUUCCUCCAGCCCCGAGGCGGAUUCA chr11 75046227 rs72561778 A G 0.00159744 - 4
hsa-mir-326
CUCAUCUGUCUGUUGGGCUGGAGGCAGGGCCUUUGUGAAGGCGGGUGGUGCUCAGAUCGCCUCUGGGC CCUUCCUCCAGCCCCGAGGCGGAUUCA chr11 75046184 . G A 0.000199681 - 47
hsa-mir-328
UGGAGUGGGGGGGCAGGAGGGGCUCAGGGAGAAAGUGCAUACAGCCCCUGGCCCUCUCUGCCCUUCCG UCCCCUG chr16 67236292 rs188892061 G C 0.00219649 - 7
hsa-mir-329-1
GGUACCUGAAGAGAGGUUUUCUGGGUUUCUGUUUCUUUAAUGAGGACGAAACACACCUGGUUAACCUC UUUUCCAGUAUC chr14 101493129 rs34557733 - A null null 8

hsa-mir-329-2
GUGGUACCUGAAGAGAGGUUUUCUGGGUUUCUGUUUCUUUAUUGAGGACGAAACACACCUGGUUAACC UCUUUUCCAGUAUCAA chr14 101493478 rs377234552 U C 0.00139776 + 42
hsa-mir-330
CUUUGGCGAUCACUGCCUCUCUGGGCCUGUGUCUUAGGCUCUGCAAGAUCAACCGAGCAAAGCACACG GCCUGCAGAGAGGCAGCGCUCUGCCCchr19 46142293 . C U 0.000199681 - 53

hsa-mir-335
UGUUUUGAGCGGGGGUCAAGAGCAAUAACGAAAAAUGUUUGUCAUAAACCGUUUUUCAUUAUUGCUCCUGACCUCCUCUCAUUUGCUAUAUUCA chr7 130135961 rs369049435 C U 0.000199681 + 10
hsa-mir-337
GUAGUCAGUAGUUGGGGGGUGGGAACGGCUUCAUACAGGAGUUGAUGCACAGUUAUCCAGCUCCUAUAUGAUGCCUUUCUUCAUCCCCUUCAA chr14 101340844 rs370534178 G C 0.000199681 + 15
hsa-mir-337
GUAGUCAGUAGUUGGGGGGUGGGAACGGCUUCAUACAGGAGUUGAUGCACAGUUAUCCAGCUCCUAUAUGAUGCCUUUCUUCAUCCCCUUCAA chr14 101340862 . A G 0.000199681 + 33
hsa-mir-338
UCUCCAACAAUAUCCUGGUGCUGAGUGAUGACUCAGGCGACUCCAGCAUCAGUGAUUUUGUUGAAGA chr17 79099736 . C U 0.000199681 - 14

hsa-mir-338
UCUCCAACAAUAUCCUGGUGCUGAGUGAUGACUCAGGCGACUCCAGCAUCAGUGAUUUUGUUGAAGA chr17 79099712 . C U 0.000199681 - 38

hsa-mir-339
CGGGGCGGCCGCUCUCCCUGUCCUCCAGGAGCUCACGUGUGCCUGCCUGUGAGCGCCUCGACGACAGAGCCGGCGCCUGCCCCAGUGUCUGCGC chr7 1062662 . C U 0.000199681 - 1
hsa-mir-339
CGGGGCGGCCGCUCUCCCUGUCCUCCAGGAGCUCACGUGUGCCUGCCUGUGAGCGCCUCGACGACAGAGCCGGCGCCUGCCCCAGUGUCUGCGC chr7 1062656 rs72631831 G A 0.000199681 - 7
hsa-mir-339
CGGGGCGGCCGCUCUCCCUGUCCUCCAGGAGCUCACGUGUGCCUGCCUGUGAGCGCCUCGACGACAGAGCCGGCGCCUGCCCCAGUGUCUGCGC chr7 1062653 . C U 0.000798722 - 10
hsa-mir-339
CGGGGCGGCCGCUCUCCCUGUCCUCCAGGAGCUCACGUGUGCCUGCCUGUGAGCGCCUCGACGACAGAGCCGGCGCCUGCCCCAGUGUCUGCGC chr7 1062626 rs145196722 G A 0.000599042 - 37
hsa-mir-339
CGGGGCGGCCGCUCUCCCUGUCCUCCAGGAGCUCACGUGUGCCUGCCUGUGAGCGCCUCGACGACAGAGCCGGCGCCUGCCCCAGUGUCUGCGC chr7 1062599 rs72631820 A G 0.014976 - 64
hsa-mir-339
CGGGGCGGCCGCUCUCCCUGUCCUCCAGGAGCUCACGUGUGCCUGCCUGUGAGCGCCUCGACGACAGAGCCGGCGCCUGCCCCAGUGUCUGCGC chr7 1062574 rs13232101 C A null null 89
hsa-mir-33a
CUGUGGUGCAUUGUAGUUGCAUUGCAUGUUCUGGUGGUACCCAUGCAAUGUUUCCACAGUGCAUCACAG chr22 42296995 rs77809319 A G 0.000599042 + 48

hsa-mir-33b
GCGGGCGGCCCCGCGGUGCAUUGCUGUUGCAUUGCACGUGUGUGAGGCGGGUGCAGUGCCUCGGCAGU
GCAGCCCGGAGCCGGCCCCUGGCACCAC chr17 17717244 . C U
0.000399361 - 2
hsa-mir-33b
GCGGGCGGCCCCGCGGUGCAUUGCUGUUGCAUUGCACGUGUGUGAGGCGGGUGCAGUGCCUCGGCAGU
GCAGCCCGGAGCCGGCCCCUGGCACCAC chr17 17717243 . G A
0.000199681 - 3
hsa-mir-33b
GCGGGCGGCCCCGCGGUGCAUUGCUGUUGCAUUGCACGUGUGUGAGGCGGGUGCAGUGCCUCGGCAGU
GCAGCCCGGAGCCGGCCCCUGGCACCAC chr17 17717209 . C U
0.000399361 - 37
hsa-mir-340
UUGUACCUGGUGUGAUUAUAAAGCAAUGAGACUGAUUGUCAUAUGUCGUUUGUGGGAUCCGUCUCAGU
UACUUUAUAGCCAUACCUGGUAUCUUA chr5 179442344 . G A
0.000199681 - 54
hsa-mir-342
GAAACUGGGCUCAAGGUGAGGGGUGCUAUCUGUGAUUGAGGGACAUGGUUAAUGGAAUUGUCUCACAC
AGAAAUCGCACCCGUCACCUUGGCCUACUUA chr14 100576035 . C U
0.000199681 + 44
hsa-mir-342
GAAACUGGGCUCAAGGUGAGGGGUGCUAUCUGUGAUUGAGGGACAUGGUUAAUGGAAUUGUCUCACAC
AGAAAUCGCACCCGUCACCUUGGCCUACUUA chr14 100576059 . C A
0.000199681 + 68
hsa-mir-345
ACCCAAACCCUAGGUCUGCUGACUCCUAGUCCAGGGCUCGUGAUGGCUGGUGGGCCCUGAACGAGGGG
UCUGGAGGCCUGGGUUUGAAUAUCGACAGC chr14 100774203 rs72631832 C U
0.0081869 + 8
hsa-mir-345
ACCCAAACCCUAGGUCUGCUGACUCCUAGUCCAGGGCUCGUGAUGGCUGGUGGGCCCUGAACGAGGGG
UCUGGAGGCCUGGGUUUGAAUAUCGACAGC chr14 100774268 . G A
0.000199681 + 73
hsa-mir-345
ACCCAAACCCUAGGUCUGCUGACUCCUAGUCCAGGGCUCGUGAUGGCUGGUGGGCCCUGAACGAGGGG
UCUGGAGGCCUGGGUUUGAAUAUCGACAGC chr14 100774277 . G C
0.000199681 + 82
hsa-mir-345
ACCCAAACCCUAGGUCUGCUGACUCCUAGUCCAGGGCUCGUGAUGGCUGGUGGGCCCUGAACGAGGGG
UCUGGAGGCCUGGGUUUGAAUAUCGACAGC chr14 100774289 rs114693307 A C
null null 94
hsa-mir-346
GGUCUCUGUGUUGGGCGUCUGUCUGCCCGCAUGCCUGCCUCUCUGUUGCUCUGAAGGAGGCAGGGGCU
GGGCCUGCAGCUGCCUGGGCAGAGCGG chr10 88024462 rs373442340 U C
0.000998403 - 84
hsa-mir-346
GGUCUCUGUGUUGGGCGUCUGUCUGCCCGCAUGCCUGCCUCUCUGUUGCUCUGAAGGAGGCAGGGGCU

GGGCCUGCAGCUGCCUGGGCAGAGCGG chr10 88024452 . G A
0.000199681 - 94
hsa-mir-34a
GGCCAGCUGUGAGUGUUUCUUUGGCAGUGUCUUAGCUGGUUGUUGUGAGCAAUAGUAAGGAAGCAAUC
AGCAAGUAUACUGCCCUAGAAGUGCUGCACGUUGUGGGGCCC chr1 9211802 rs35301225
G U null null 8
hsa-mir-34a
GGCCAGCUGUGAGUGUUUCUUUGGCAGUGUCUUAGCUGGUUGUUGUGAGCAAUAGUAAGGAAGCAAUC
AGCAAGUAUACUGCCCUAGAAGUGCUGCACGUUGUGGGGCCC chr1 9211782 rs72631823
G A 0.000998403 - 55
hsa-mir-34a
GGCCAGCUGUGAGUGUUUCUUUGGCAGUGUCUUAGCUGGUUGUUGUGAGCAAUAGUAAGGAAGCAAUC
AGCAAGUAUACUGCCCUAGAAGUGCUGCACGUUGUGGGGCCC chr1 9211778 . G
A 0.000199681 - 59
hsa-mir-34a
GGCCAGCUGUGAGUGUUUCUUUGGCAGUGUCUUAGCUGGUUGUUGUGAGCAAUAGUAAGGAAGCAAUC
AGCAAGUAUACUGCCCUAGAAGUGCUGCACGUUGUGGGGCCC chr1 9211747
rs201359809 G C 0.000199681 - 90

hsa-mir-34a
GGCCAGCUGUGAGUGUUUCUUUGGCAGUGUCUUAGCUGGUUGUUGUGAGCAAUAGUAAGGAAGCAAUC
AGCAAGUAUACUGCCCUAGAAGUGCUGCACGUUGUGGGGCCC chr1 9211738 . G
A 0.000199681 - 99
hsa-mir-3605
ACUUUAUACGUGUAAUUGUGAUGAGGAUGGAUAGCAAGGAAGCCGCUCCCACCUGACCCUCACGGCCU
CCGUGUUACCUGUCCUCUAGGUGGGACGCUCG chr1 33798091 rs113525648 U C
null null 3
hsa-mir-3605
ACUUUAUACGUGUAAUUGUGAUGAGGAUGGAUAGCAAGGAAGCCGCUCCCACCUGACCCUCACGGCCU
CCGUGUUACCUGUCCUCUAGGUGGGACGCUCG chr1 33798031 rs80176156 C G
null null 68
hsa-mir-3605
ACUUUAUACGUGUAAUUGUGAUGAGGAUGGAUAGCAAGGAAGCCGCUCCCACCUGACCCUCACGGCCU
CCGUGUUACCUGUCCUCUAGGUGGGACGCUCG chr1 33798007 rs112435318 A C
null null 87
hsa-mir-3607
AAGGUUGCGGUGCAUGUGAUGAAGCAAAUCAGUAUGAAUGAAUUCAUGAUACUGUAAACGCUUUCUGA
UGUACUACUCA chr5 85916322 rs113797294 G A null null 9

hsa-mir-3609
GUAACAGUAACUUUUAUUCUCAUUUUCCUUUUCUCUACCUUGUAGAGAAGCAAAGUGAUGAGUAAUAC
UGGCUGGAGCCC chr7 98479274 rs34109026 U - null null 2

hsa-mir-361
GGAGCUUAUCAGAAUCUCCAGGGGUACUUUAUAAUUUCAAAAAGUCCCCCAGGUGUGAUUCUGAUUUG
CUUC chrX 85158670 . A C 0.000264901 - 43

hsa-mir-3610
AAGAGCCGCGGCGUAACGGCAGCCAUCUUGUUUGUUUGAGUGAAUCGGAAAGGAGGCGCCGGCUGUGG
CGGCG chr8 117886991 rs112072631 A C null null 49

hsa-mir-3610
AAGAGCCGCGGCGUAACGGCAGCCAUCUUGUUUGUUUGAGUGAAUCGGAAAGGAGGCGCCGGCUGUGG
CGGCG chr8 117886982 rs10094194 G C null null 58

hsa-mir-3612
GGGACUGGGGAUGAGGAGGCAUCUUGAGAAAUGGAAGGAAUGGGAUCUACUUCCAGUUCACUAGAGGC
GUCCUGACACCCCUAGCUC chr12 128778703 rs1683709 G A null
null 67
hsa-mir-3614
GGUUCUGUCUUGGGCCACUUGGAUCUGAAGGCUGCCCCUUUGCUCUCUGGGGUAGCCUUCAGAUCUUG
GUGUUUUGAAUUCUUACU chr17 54968700 rs118080115 A G null
null 17
hsa-mir-3615
GACUCUGGGACGCUCAGACGCCGCGCGGGGCGGGGAUUGGUCUGUGGUCCUCUCUCGGCUCCUCGCGG
CUCGCGGCGGCCGACGGUU chr17 72744798 rs745666 G C null
null 47
hsa-mir-3615
GACUCUGGGACGCUCAGACGCCGCGCGGGGCGGGGAUUGGUCUGUGGUCCUCUCUCGGCUCCUCGCGG
CUCGCGGCGGCCGACGGUU chr17 72744805 rs112977728 C U null
null 54
hsa-mir-3617
AGGUCAUAGAAAGACAUAGUUGCAAGAUGGGAUUAGAAACCAUAUGUCUCAUCAGCACCCUAUGUCCU
UUCUCUGCCCU chr20 44333749 rs80142005 C U null null 71

hsa-mir-3618
UAAGCUGAGUGCAUUGUGAUUUCCAAUAAUUGAGGCAGUGGUUCUAAAAGCUGUCUACAUUAAUGAAA
AGAGCAAUGUGGCCAGCUUG chr22 20073323 rs12159555 C G
null null 55
hsa-mir-3619
ACGGCAUCUUUGCACUCAGCAGGCAGGCUGGUGCAGCCCGUGGUGGGGGACCAUCCUGCCUGCUGUGG
GGUAAGGACGGCUGU chr22 46486996 rs73888764 A G null
null 73
hsa-mir-362
CUUGAAUCCUUGGAACCUAGGUGUGAGUGCUAUUUCAGUGCAACACACCUAUUCAAGGAUUCAAA
chrX 49773603 . A G 0.000264901 + 32

hsa-mir-3620
GUGAGGUGGGGGCCAGCAGGGAGUGGGCUGGGCUGGGCUGGGCCAAGGUACAAGGCCUCACCCUGCAU
CCCGCACCCAG chr1 228284991 rs2070960 C U null null 28

hsa-mir-3622a
AAUAGAGGGUGCACAGGCACGGGAGCUCAGGUGAGGCAGGGAGCUGAGCUCACCUGACCUCCCAUGCC
UGUGCACCCUCUAUU chr8 27559214 rs66683138 G A null

null 21
hsa-mir-3622a
AAUAGAGGGUGCACAGGCACGGGAGCUCAGGUGAGGCAGGGAGCUGAGCUCACCUGACCUCCCAUGCC UGUGCACCCUCUAUU chr8 27559261 rs13276615 C A null null 68
hsa-mir-3622b
AGUGAUAUAAUAGAGGGUGCACAGGCAUGGGAGGUCAGGUGAGCUCAGCUCCCUGCCUCACCUGAGCU CCCGUGCCUGUGCACCCUCUAUUGGCU chr8 27559261 rs13276615 G U null null 24
hsa-mir-3622b
AGUGAUAUAAUAGAGGGUGCACAGGCAUGGGAGGUCAGGUGAGCUCAGCUCCCUGCCUCACCUGAGCU CCCGUGCCUGUGCACCCUCUAUUGGCU chr8 27559214 rs66683138 C U null null 71
hsa-mir-363
UGUUGUCGGGUGGAUCACGAUGCAAUUUUGAUGAGUAUCAUAGGAGAAAAAUUGCACGGUAUCCAUCU GUAAACC chrX 133303465 rs199822597 C U 0.000529801 - 18
hsa-mir-363
UGUUGUCGGGUGGAUCACGAUGCAAUUUUGAUGAGUAUCAUAGGAGAAAAAUUGCACGGUAUCCAUCU GUAAACC chrX 133303425 rs374421612 G A 0.000264901 - 58
hsa-mir-3649
GCUUGGAACAGGCACCUGUGUGUGCCCAAGUGUUUCUAGCAAACACAGGGACCUGAGUGUCUAAGC chr12 1769533 rs117723462 A C null null 14

hsa-mir-3652
CGGCUGGAGGUGUGAGGAUCCGAACCCAGGGGUGGGGGGUGGAGGCGGCUCCUGCGAUCGAAGGGGAC UUGAGACUCACCGGCCGCACGCCAUGAGGGCCCUGUGGGUGCUGGGCCUCUGCUGCGUCCUGC chr12 104324231 rs62747560 GGGGUGG - null null 29

hsa-mir-3652
CGGCUGGAGGUGUGAGGAUCCGAACCCAGGGGUGGGGGGUGGAGGCGGCUCCUGCGAUCGAAGGGGAC UUGAGACUCACCGGCCGCACGCCAUGAGGGCCCUGUGGGUGCUGGGCCUCUGCUGCGUCCUGC chr12 104324238 rs71440517 GGGGUGG - null null 36

hsa-mir-3652
CGGCUGGAGGUGUGAGGAUCCGAACCCAGGGGUGGGGGGUGGAGGCGGCUCCUGCGAUCGAAGGGGAC UUGAGACUCACCGGCCGCACGCCAUGAGGGCCCUGUGGGUGCUGGGCCUCUGCUGCGUCCUGC chr12 104324266 rs17797090 G A null null 64

hsa-mir-3653
UCCCUGGGGACCCCUGGCAGCCCCUCCUGAUGAUUCUUCUUCCUGAGCACGCUCAUGAUGAGCAAACU GAGCCUCUAAGAAGUUGACUGAAGGGGCUGCUUCCCCAAGGA chr22 29729163 rs116331513 G U null null 94

hsa-mir-3654 UUCAUGAGCUGCAAUCUCAUCACUGGAAUGUUCCAGCGACUGGACAAGCUGAGGAA chr7 132719655 rs111673548 C G null null 21

hsa-mir-3657
UGUGUCCCAUAAUUAAAUAAUGAAAUCUGAAAUCACCAAUAAUGGGACACUAAUGUGAUUAAUGUUGUUGUGUCCCAUUAUUGGUGAUUUCAGAUUUCAUAUAUGAUUAAGGACAUA chr12 112475427 rs71083196 - G null null 93

hsa-mir-3657
UGUGUCCCAUAAUUAAAUAAUGAAAUCUGAAAUCACCAAUAAUGGGACACUAAUGUGAUUAAUGUUGUUGUGUCCCAUUAUUGGUGAUUUCAGAUUUCAUAUAUGAUUAAGGACAUA chr12 112475429 rs71083197 - C null null 97

hsa-mir-3657
UGUGUCCCAUAAUUAAAUAAUGAAAUCUGAAAUCACCAAUAAUGGGACACUAAUGUGAUUAAUGUUGUUGUGUCCCAUUAUUGGUGAUUUCAGAUUUCAUAUAUGAUUAAGGACAUA chr12 112475403 rs77419179 A G null null 117

hsa-mir-3659
UCUACAAGCAGAUACAAGGAUGCCCUUGUACACAACACACGUGCUGCUUGUAUAGACAUGAGUGUUGUCUACGAGGGCAUCCUUGUGUCUGUGUGUGUG chr1 38554942 rs553122 C U null null 40
hsa-mir-365a
ACCGCAGGGAAAAUGAGGGACUUUUGGGGGCAGAUGUGUUUCCAUUCCACUAUCAUAAUGCCCCUAAAAAUCCUUAUUGCUCUUGCA chr16 14403144 . C U 0.000199681 + 3
hsa-mir-365b
AGAGUGUUCAAGGACAGCAAGAAAAAUGAGGGACUUUCAGGGGCAGCUGUGUUUUCUGACUCAGUCAUAAUGCCCCUAAAAAUCCUUAUUGUUCUUGCAGUGUGCAUCGGG chr17 29902536 . U G 0.000199681 + 107
hsa-mir-3660
GAAAGAAGAACUGGACAAAAUUAAAAUGCUCUUCUGUCAUUGUAAUAGUUCAUAUGGGCACUGACAGGAGAGCAUUUUGACUUUGUCAAGUGUGUCUGCU chr5 89312487 rs75102645 C U null null 51
hsa-mir-3660
GAAAGAAGAACUGGACAAAAUUAAAAUGCUCUUCUGUCAUUGUAAUAGUUCAUAUGGGCACUGACAGGAGAGCAUUUUGACUUUGUCAAGUGUGUCUGCU chr5 89312451 rs34630557 - G null null 87
hsa-mir-3662
UGUGUUUUCCUCAACGCUCACAGUUACACUUCUUACUCUCAAUCCAUUCAUAUUGAAAAUGAUGAGUAGUGACUGAUGAAGCACAAAUCAGCCAA chr6 135300536 rs117905199 A G null null 35
hsa-mir-3663
CCCGGGACCUUGGUCCAGGCGCUGGUCUGCGUGGUGCUCGGGUGGAUAAGUCUGAUCUGAGCACCACACAGGCCGGGCGCCGGGACCAAGGGGGCUC chr10 118927256 rs111525499 C U null null 30
hsa-mir-3664
CUGUAAACUUGAAGGUAGGGAACUCUGUCUUCACUCAUGAGUACCUUCCAACACGAGCUCUCAGGAGUAAAGACAGAGUUCCCUACCUUCAAUGUGGAU chr11 70718466 rs75728268 C A

null null 8
hsa-mir-3667
UGAGGAUGAAAGACCCAUUGAGGAGAAGGUUCUGCUGGCUGAGAACCUUCCUCUCCAUGGGUCUUUCA
UCCUCA chr22 49937087 rs113384782 G A null null 28

hsa-mir-3667
UGAGGAUGAAAGACCCAUUGAGGAGAAGGUUCUGCUGGCUGAGAACCUUCCUCUCCAUGGGUCUUUCA
UCCUCA chr22 49937076 rs76749295 C U null null 39

hsa-mir-367
CCAUUACUGUUGCUAAUAUGCAACUCUGUUGAAUAUAAAUUGGAAUUGCACUUUAGCAAUGGUGAUGG
chr4 113569088 rs150161032 U C 0.000199681 - 10

hsa-mir-367
CCAUUACUGUUGCUAAUAUGCAACUCUGUUGAAUAUAAAUUGGAAUUGCACUUUAGCAAUGGUGAUGG
chr4 113569054 . A C 0.000199681 - 44

hsa-mir-3671
AUGUUAUUGCUGCUGCUGUCACAUUUACAUGAAAAUAAAAUGUAAAUUAUUUUAUUUCUAUCAAAUAA
GGACUAGUCUGCAGUGAUAU chr1 65523519 rs521188 U C
null null 7
hsa-mir-3671
AUGUUAUUGCUGCUGCUGUCACAUUUACAUGAAAAUAAAAUGUAAAUUAUUUUAUUUCUAUCAAAUAA
GGACUAGUCUGCAGUGAUAU chr1 65523500 rs112181011 U C
null null 26
hsa-mir-3671
AUGUUAUUGCUGCUGCUGUCACAUUUACAUGAAAAUAAAAUGUAAAUUAUUUUAUUUCUAUCAAAUAA
GGACUAGUCUGCAGUGAUAU chr1 65523495 rs115442930 G U
null null 31
hsa-mir-3674
ACAUCACUAUUGUAGAACCUAAGAUUGGCCGUUUGAGAUGUCCUUUCAAGUUUUUGCAUUUCUGAUGU
chr8 1749333 rs75404472 C U null null 43

hsa-mir-3678
GAAUCCGGUCCGUACAAACUCUGCUGUGUUGAAUGAUUGGUGAGUUUGUUUGCUCAUUGAUUGAAUCA
CUGCAGAGUUUGUACGGACCGGAUUCchr17 73402156 rs34571046 G A
null null 7
hsa-mir-3679
CGUGGUGAGGAUAUGGCAGGGAAGGGGAGUUUCCCUCUAUUCCCUUCCCCCCAGUAAUCUUCAUCAUG
chr2 134884697 rs10175383 G C null null 2

hsa-mir-3679
CGUGGUGAGGAUAUGGCAGGGAAGGGGAGUUUCCCUCUAUUCCCUUCCCCCCAGUAAUCUUCAUCAUG
chr2 134884700 rs6430498 G A null null 5

hsa-mir-3679
CGUGGUGAGGAUAUGGCAGGGAAGGGGAGUUUCCCUCUAUUCCCUUCCCCCCAGUAAUCUUCAUCAUG

chr2 134884717 rs79705373 A G null null 22

hsa-mir-3679
CGUGGUGAGGAUAUGGCAGGGAAGGGGAGUUUCCCUCUAUUCCCUUCCCCCCAGUAAUCUUCAUCAUG
chr2 134884723 rs117058134 A G null null 28

hsa-mir-3679
CGUGGUGAGGAUAUGGCAGGGAAGGGGAGUUUCCCUCUAUUCCCUUCCCCCCAGUAAUCUUCAUCAUG
chr2 134884737 rs116613504 C A null null 42

hsa-mir-3682
UAAGUUAUAUAUGUCUACUUCUACCUGUGUUAUCAUAAUAAAGGUGUCAUGAUGAUACAGGUGGAGGU
AGAAAUAUAUAACUUA chr2 54076332 rs1047873 A G null
null 11
hsa-mir-3682
UAAGUUAUAUAUGUCUACUUCUACCUGUGUUAUCAUAAUAAAGGUGUCAUGAUGAUACAGGUGGAGGU
AGAAAUAUAUAACUUA chr2 54076326 rs116380885 A G null
null 17
hsa-mir-3683
GGGUGUACACCCCCUGCGACAUUGGAAGUAGUAUCAUCUCUCCCUUGGAUGCUACGAACAAUAUCACA
GAAGGUGUACACCC chr7 7106636 rs6977967 U C null
null 41
hsa-mir-3683
GGGUGUACACCCCCUGCGACAUUGGAAGUAGUAUCAUCUCUCCCUUGGAUGCUACGAACAAUAUCACA
GAAGGUGUACACCC chr7 7106600 rs6943868 A U null
null 77
hsa-mir-3686
CUCACCUCAUUCAUUUACCUUCUCUUACAGAUCACUUUUCUGCACUGGACAGUGAUCUGUAAGAGAAA
GUAAAUGAAAGAGGUGAG chr8 130496365 rs6997249 C U null
null 24
hsa-mir-3689b
GAUCCUGUGCUCCCUGGGGGGUCUGAUCCUGUGCUUCCUGGGAGGUGUGAUAUCAUGGUUCCUGGGAG
GUGUGAUCCCGUGCUUCCUGGGAGGUGUGAUAUUGUGGUUCCUGGGAGGUGUGAUCCCGUGCUCCCUGGGAGGUG
UGAUC chr9 137742041 rs35679521 C U null null 78

hsa-mir-3689b
GAUCCUGUGCUCCCUGGGGGGUCUGAUCCUGUGCUUCCUGGGAGGUGUGAUAUCAUGGUUCCUGGGAG
GUGUGAUCCCGUGCUUCCUGGGAGGUGUGAUAUUGUGGUUCCUGGGAGGUGUGAUCCCGUGCUCCCUGGGAGGUG
UGAUC chr9 137742013 rs71483239 G C null null 106

hsa-mir-3689b
GAUCCUGUGCUCCCUGGGGGGUCUGAUCCUGUGCUUCCUGGGAGGUGUGAUAUCAUGGUUCCUGGGAG
GUGUGAUCCCGUGCUUCCUGGGAGGUGUGAUAUUGUGGUUCCUGGGAGGUGUGAUCCCGUGCUCCCUGGGAGGUG
UGAUC chr9 137741994 rs76317538 C U null null 125

hsa-mir-3689b
GAUCCUGUGCUCCCUGGGGGGUCUGAUCCUGUGCUUCCUGGGAGGUGUGAUAUCAUGGUUCCUGGGAG

GUGUGAUCCCGUGCUUCCUGGGAGGUGUGAUAUUGUGGUUCCUGGGAGGUGUGAUCCCGUGCUCCCUGGGAGGUG
UGAUC chr9 137741987 rs74405398 C U null null 127

hsa-mir-3689b
GAUCCUGUGCUCCCUGGGGGGUCUGAUCCUGUGCUUCCUGGGAGGUGUGAUAUCAUGGUUCCUGGGAG
GUGUGAUCCCGUGCUUCCUGGGAGGUGUGAUAUUGUGGUUCCUGGGAGGUGUGAUCCCGUGCUCCCUGGGAGGUG
UGAUC chr9 137741989 rs71506933 C G null null 127

hsa-mir-370
AGACAGAGAAGCCAGGUCACGUCUCUGCAGUUACACAGCUCACGAGUGCCUGCUGGGGUGGAACCUGG
UCUGUCU chr14 101377478 . A G 0.000199681 + 3

hsa-mir-3714
GAAGGCAGCAGUGCUCCCCUGUGACGUGCUCCAUCACCGGGCAGGGAAGACACCGCUGCCACCUC
chr3 16974725 rs55835744 C U null null 38

hsa-mir-3714
GAAGGCAGCAGUGCUCCCCUGUGACGUGCUCCAUCACCGGGCAGGGAAGACACCGCUGCCACCUC
chr3 16974739 rs75653184 A C null null 52

hsa-mir-371a
GUGGCACUCAAACUGUGGGGGCACUUUCUGCUCUCUGGUGAAAGUGCCGCCAUCUUUUGAGUGUUAC
chr19 54290994 . A G 0.000199681 + 66

hsa-mir-371a
GUGGCACUCAAACUGUGGGGGCACUUUCUGCUCUCUGGUGAAAGUGCCGCCAUCUUUUGAGUGUUAC
chr19 54290995 . C G 0.000199681 + 67

hsa-mir-371b
GGUAACACUCAAAAGAUGGCGGCACUUUCACCAGAGAGCAGAAAGUGCCCCCACAGUUUGAGUGCC
chr19 54290995 . G C 0.000199681 - 2

hsa-mir-371b
GGUAACACUCAAAAGAUGGCGGCACUUUCACCAGAGAGCAGAAAGUGCCCCCACAGUUUGAGUGCC
chr19 54290994 . U C 0.000199681 - 3

hsa-mir-372
GUGGGCCUCAAAUGUGGAGCACUAUUCUGAUGUCCAAGUGGAAAGUGCUGCGACAUUUGAGCGUCAC
chr19 54291161 . A U 0.000199681 + 18

hsa-mir-373
GGGAUACUCAAAAUGGGGGCGCUUUCCUUUUUGUCUGUACUGGGAAGUGCUUCGAUUUUGGGGUGUCC
C chr19 54291965 . C U 0.000199681 + 7

hsa-mir-373
GGGAUACUCAAAAUGGGGGCGCUUUCCUUUUUGUCUGUACUGGGAAGUGCUUCGAUUUUGGGGUGUCC
C chr19 54292016 rs182623921 U C 0.000199681 + 58

hsa-mir-376a-2
GGUAUUUAAAAGGUAGAUUUUCCUUCUAUGGUUACGUGUUUGAUGGUUAAUCAUAGAGGAAAAUCCAC GUUUUCAGUAUC chr14 101506441 rs373534314 G A 0.000399361 + 36

hsa-mir-376a-2
GGUAUUUAAAAGGUAGAUUUUCCUUCUAUGGUUACGUGUUUGAUGGUUAAUCAUAGAGGAAAAUCCAC GUUUUCAGUAUC chr14 101506465 . A G 0.000199681 + 60

hsa-mir-377
UUGAGCAGAGGUUGCCCUUGGUGAAUUCGCUUUAUUUAUGUUGAAUCACACAAAGGCAACUUUUGUUU G chr14 101528401 rs189165474 C U 0.000199681 + 15

hsa-mir-377
UUGAGCAGAGGUUGCCCUUGGUGAAUUCGCUUUAUUUAUGUUGAAUCACACAAAGGCAACUUUUGUUU G chr14 101528426 . G U 0.000199681 + 40

hsa-mir-378a
AGGGCUCCUGACUCCAGGUCCUGUGUGUUACCUAGAAAUAGCACUGGACUUGGAGUCAGAAGGCCU chr5 149112399 rs376185067 C G 0.000798722 + 12

hsa-mir-378c
GGAGGCCAUCACUGGACUUGGAGUCAGAAGAGUGGAGUCGGGUCAGACUUCAACUCUGACUUUGAAGG UGGUGAGUGCCUCchr10 132760931 . G A 0.000399361 - 1

hsa-mir-378c
GGAGGCCAUCACUGGACUUGGAGUCAGAAGAGUGGAGUCGGGUCAGACUUCAACUCUGACUUUGAAGG UGGUGAGUGCCUCchr10 132760893 . C U 0.00219649 - 39

hsa-mir-378d-1 ACUGUUUCUGUCCUUGUUCUUGUUGUUAUUACUGGACUUGGAGUCAGAAACAGG chr4 5925054 rs28645567 C U 0.0782748 - 2

hsa-mir-378d-2
GAAUGGUUACAAGGAGAGAACACUGGACUUGGAGUCAGAAAACUUUCAUCCAAGUCAUUCCCUGCUCU AAGUCCCAUUUCUGUUCCAUGAGAUUGUUU chr8 94928319 rs376196852 U C 0.000199681 - 29

hsa-mir-378d-2
GAAUGGUUACAAGGAGAGAACACUGGACUUGGAGUCAGAAAACUUUCAUCCAAGUCAUUCCCUGCUCU AAGUCCCAUUUCUGUUCCAUGAGAUUGUUU chr8 94928250 rs73692959 U A 0.0199681 - 98

hsa-mir-378e
CUGACUCCAGUGUCCAGGCCAGGGGCAGACAGUGGACAGAGAACAGUGCCCAAGACCACUGGACUUGG AGUCAGGACAU chr5 169455502 rs367764573 U C 0.00259585 + 11

hsa-mir-378e
CUGACUCCAGUGUCCAGGCCAGGGGCAGACAGUGGACAGAGAACAGUGCCCAAGACCACUGGACUUGG AGUCAGGACAU chr5 169455539 rs371300444 G U 0.000199681 +

48
hsa-mir-378e
CUGACUCCAGUGUCCAGGCCAGGGGCAGACAGUGGACAGAGAACAGUGCCCAAGACCACUGGACUUGG
AGUCAGGACAU chr5 169455551 rs376752141 U C 0.00259585 +
60
hsa-mir-378h
ACAGGAACACUGGACUUGGUGUCAGAUGGGAUGAGCCCUGGCUCUGUUUCCUAGCAGCAAUCUGAUCU
UGAGCUAGUCACUGG chr5 154209024 rs702742 A G 0.208267
+ 7
hsa-mir-378i
GGGAGCACUGGACUAGGAGUCAGAAGGUGGAGUUCUGGGUGCUGUUUUCCCACUCUUGGGCCCUGGGC
AUGUUCUG chr22 42319291 . G A 0.000798722 - 11

hsa-mir-378i
GGGAGCACUGGACUAGGAGUCAGAAGGUGGAGUUCUGGGUGCUGUUUUCCCACUCUUGGGCCCUGGGC
AUGUUCUG chr22 42319276 rs9607855 G A 0.000399361 -
26
hsa-mir-378i
GGGAGCACUGGACUAGGAGUCAGAAGGUGGAGUUCUGGGUGCUGUUUUCCCACUCUUGGGCCCUGGGC
AUGUUCUG chr22 42319269 rs111400080 U G 0.000199681 -
33
hsa-mir-378j
AUGCAGUGAGUCGGGGAGGAACUGGAUUUGGAGCCAGAAGAACUGGUUCUAAUAUCUACUUCCCUGUG
UAGAGUUGGGAUUUGGGAUUAUAUGAGUUAAUAUACACCAA chr17 35975072 . G
A 0.000199681 - 13
hsa-mir-378j
AUGCAGUGAGUCGGGGAGGAACUGGAUUUGGAGCCAGAAGAACUGGUUCUAAUAUCUACUUCCCUGUG
UAGAGUUGGGAUUUGGGAUUAUAUGAGUUAAUAUACACCAA chr17 35974982
rs117582922 A G 0.000599042 - 103

hsa-mir-379
AGAGAUGGUAGACUAUGGAACGUAGGCGUUAUGAUUUCUGACCUAUGUAACAUGGUCCACUAACUCU
chr14 101488414 rs61991156 A G null null 12

hsa-mir-379
AGAGAUGGUAGACUAUGGAACGUAGGCGUUAUGAUUUCUGACCUAUGUAACAUGGUCCACUAACUCU
chr14 101488424 rs72631818 G A null null 22

hsa-mir-379
AGAGAUGGUAGACUAUGGAACGUAGGCGUUAUGAUUUCUGACCUAUGUAACAUGGUCCACUAACUCU
chr14 101488430 rs115827677 G A 0.000399361 + 28

hsa-mir-379
AGAGAUGGUAGACUAUGGAACGUAGGCGUUAUGAUUUCUGACCUAUGUAACAUGGUCCACUAACUCU
chr14 101488431 rs182581788 U C 0.000199681 + 29

hsa-mir-380 AAGAUGGUUGACCAUAGAACAUGCGCUAUCUCUGUGUCGUAUGUAAUAUGGUCCACAUCUU

chr14 101491378 . G A 0.000399361 + 25

hsa-mir-380 AAGAUGGUUGACCAUAGAACAUGCGCUAUCUCUGUGUCGUAUGUAAUAUGGUCCACAUCUU
chr14 101491407 . C A 0.000199681 + 54

hsa-mir-381
UACUUAAAGCGAGGUUGCCCUUUGUAUAUUCGGUUUAUUGACAUGGAAUAUACAAGGGCAAGCUCUCU
GUGAGUA chr14 101512288 rs369671146 G A 0.000199681 +
32
hsa-mir-381
UACUUAAAGCGAGGUUGCCCUUUGUAUAUUCGGUUUAUUGACAUGGAAUAUACAAGGGCAAGCUCUCU
GUGAGUA chr14 101512304 rs375948123 A G 0.000399361 +
48
hsa-mir-382
UACUUGAAGAGAAGUUGUUCGUGGUGGAUUCGCUUUACUUAUGACGAAUCAUUCACGGACAACACUUU
UUUCAGUA chr14 101520657 rs192719529 U C 0.000199681 +
15
hsa-mir-382
UACUUGAAGAGAAGUUGUUCGUGGUGGAUUCGCUUUACUUAUGACGAAUCAUUCACGGACAACACUUU
UUUCAGUA chr14 101520687 rs377201140 C U 0 + 45

hsa-mir-383
CUCCUCAGAUCAGAAGGUGAUUGUGGCUUUGGGUGGAUAUUAAUCAGCCACAGCACUGCCUGGUCAGA
AAGAG chr8 14711013 rs112302475 A C null null 7

hsa-mir-383
CUCCUCAGAUCAGAAGGUGAUUGUGGCUUUGGGUGGAUAUUAAUCAGCCACAGCACUGCCUGGUCAGA
AAGAG chr8 14710999 rs184836993 U C 0.000199681 - 21

hsa-mir-384
UGUUAAAUCAGGAAUUUUAAACAAUUCCUAGACAAUAUGUAUAAUGUUCAUAAGUCAUUCCUAGAAAU
UGUUCAUAAUGCCUGUAACA chrX 76139740 . G A
0.000264901 - 46
hsa-mir-3908
GCCUGAGCAAUGUAGGUAGACUGUUUCUAAAAAAAUAAAAAGUUAAAAAAAUUUAUGUUAACGUGUAA
UGUGUUUACUAAUUUUUUUUUUUUUUUUUUGGAGACAGAGUCUCCCUCUGUCGCCAGGC chr12 124021017
rs111803974 C U null null 62

hsa-mir-3908
GCCUGAGCAAUGUAGGUAGACUGUUUCUAAAAAAAUAAAAAGUUAAAAAAAUUUAUGUUAACGUGUAA
UGUGUUUACUAAUUUUUUUUUUUUUUUUUUGGAGACAGAGUCUCCCUCUGUCGCCAGGC chr12 124021033
rs117234492 U A null null 78

hsa-mir-3908
GCCUGAGCAAUGUAGGUAGACUGUUUCUAAAAAAAUAAAAAGUUAAAAAAAUUUAUGUUAACGUGUAA
UGUGUUUACUAAUUUUUUUUUUUUUUUUUUGGAGACAGAGUCUCCCUCUGUCGCCAGGC chr12 124021052
rs74446573 U G null null 97

hsa-mir-3908
GCCUGAGCAAUGUAGGUAGACUGUUUCUAAAAAAAUAAAAAGUUAAAAAAAUUUAUGUUAACGUGUAA UGUGUUUACUAAUUUUUUUUUUUUUUUUUUGGAGACAGAGUCUCCCUCUGUCGCCAGGC chr12 124021054 rs61953551 G U null null 99

hsa-mir-3909
GGUAUGCUGUUGCGCUGUCCUUCCUCUGGGGAGCAGGCUCCGGGGGACAGGGAAAAGCACACAAGGAA CUUGUCCUCUAGGGCCUGCAGUCUCAUGGGAGAGUGACAUGCACCAGGACC chr22 35731697 rs9607265 G A null null 65

hsa-mir-3909
GGUAUGCUGUUGCGCUGUCCUUCCUCUGGGGAGCAGGCUCCGGGGGACAGGGAAAAGCACACAAGGAA CUUGUCCUCUAGGGCCUGCAGUCUCAUGGGAGAGUGACAUGCACCAGGACC chr22 35731712 rs34874675 G - null null 80

hsa-mir-3910-1
CUUUUGCUGUCAGUUUUUCUGUUGCUUGUCUUGGUUUUAUGCCUUUUAUAUCAAGGCACAUAAAAGGC AUAAAACCAAGACAAGCAACAAAAAAAGGAUUGAUCACAGAAG chr9 94398543 rs12344333 C U null null 11
hsa-mir-3910-1
CUUUUGCUGUCAGUUUUUCUGUUGCUUGUCUUGGUUUUAUGCCUUUUAUAUCAAGGCACAUAAAAGGC AUAAAACCAAGACAAGCAACAAAAAAAGGAUUGAUCACAGAAG chr9 94398581 rs67339585 U C null null 49
hsa-mir-3910-1
CUUUUGCUGUCAGUUUUUCUGUUGCUUGUCUUGGUUUUAUGCCUUUUAUAUCAAGGCACAUAAAAGGC AUAAAACCAAGACAAGCAACAAAAAAAGGAUUGAUCACAGAAG chr9 94398600 rs76084273 C A null null 68
hsa-mir-3910-2
UUUUUUUGUUGCUUGUCUUGGUUUUAUGCCUUUUAUGUGCCUUGAUAUAAAAGGCAUAAAACCAAGAC AAGCAACAGAAAAA chr9 94398600 rs76084273 G U null null 28
hsa-mir-3910-2
UUUUUUUGUUGCUUGUCUUGGUUUUAUGCCUUUUAUGUGCCUUGAUAUAAAAGGCAUAAAACCAAGAC AAGCAACAGAAAAA chr9 94398581 rs67339585 A G null null 47
hsa-mir-3911
GGGUGAGGAUGUGUGUGGAUCCUGGAGGAGGCAGAGAAGACAGUGAGCUUGCCAGUUCUGGUUUCCAA CACUUCCUUUCCUGCGCUUCUCGAUUCCCAGAUCUGCACCC chr9 130452987 rs112996025 C U null null 88

hsa-mir-3911
GGGUGAGGAUGUGUGUGGAUCCUGGAGGAGGCAGAGAAGACAGUGAGCUUGCCAGUUCUGGUUUCCAA CACUUCCUUUCCUGCGCUUCUCGAUUCCCAGAUCUGCACCC chr9 130452977 rs57528423 A G null null 98
hsa-mir-3911
GGGUGAGGAUGUGUGUGGAUCCUGGAGGAGGCAGAGAAGACAGUGAGCUUGCCAGUUCUGGUUUCCAA

CACUUCCUUUCCUGCGCUUCUCGAUUCCCAGAUCUGCACCC chr9 130452968 rs75848623 C A null null 107
hsa-mir-3912 AGAGAGGAAUGAACAGUUAAAUUAUAACAUGUCCAUAUUAUGGGUUAGUUGUGGACACAUACUAACGCAUAAUAUGGACAUGUUAUAAUUUAACUGUUCCUUUCU chr5 170813684 rs34887287 - U null null 81
hsa-mir-3916 AUCCCAGAGAAGAAGGAAGAAGAGGAAGAAAUGGCUGGUUCUCAGGUGAAUGUGUCUGGGUUCAGGGGAUGUGUCUCCUCUUUUCUUCUGGGAUchr1 247365286 rs113974396 UC - null null 77
hsa-mir-3919 CCUGAGCACCAUUUACUGAGUCCUUUGUUCUCUACUAGUUUGUAGUAGUUCGUAGCAGAGAACAAAGGACUCAGUAAAUGGUGCUCAGG chr3 159000436 rs112738359 C U null null 2
hsa-mir-3922 GGAAGAGUCAAGUCAAGGCCAGAGGUCCCACAGCAGGGCUGGAAAGCACACCUGUGGGACUUCUGGCCUUGACUUGACUCUUUC chr12 104985443 rs61938575 G A null null 33
hsa-mir-3924 UAAAUGAAAAAGUAGUAGUCAAAUAUGCAGAUCUAUGUCAUAUAUACAGAUAUGUAUAUGUGACUGCUACUUUUUUGUUUAchr10 59064260 rs77767763 G C null null 60

hsa-mir-3927 UGCCAAUGCCUAUCACAUAUCUGCCUGUCCUAUGACAAACAUGGCAGGUAGAUAUUUGAUAGGCAUUGGCA chr9 112273768 rs80350479 G U null null 58

hsa-mir-3928 GCUGAAGCUCUAAGGUUCCGCCUGCGGGCAGGAAGCGGAGGAACCUUGGAGCUUCGGC chr22 31556103 rs5997893 U C null null 3

hsa-mir-3928 GCUGAAGCUCUAAGGUUCCGCCUGCGGGCAGGAAGCGGAGGAACCUUGGAGCUUCGGC chr22 31556085 rs113572115 C U null null 21

hsa-mir-3936 AUGAUUCAGAGCAUCUGUCCAGUGUCUGCUGUAGAUCCCUCAAAUCCGUGUUUGGACGCUUCUGGUAAGGGGUGUAUGGCAGAUGCACCCGACAGAUGCACUUGGCAGCA chr5 131701279 rs367805 A G null null 13
hsa-mir-3936 AUGAUUCAGAGCAUCUGUCCAGUGUCUGCUGUAGAUCCCUCAAAUCCGUGUUUGGACGCUUCUGGUAAGGGGUGUAUGGCAGAUGCACCCGACAGAUGCACUUGGCAGCA chr5 131701185 rs61745813 A G null null 107
hsa-mir-3938 AGGAAUUUUUAACCCGAUCACUAGAUUAUCUACAAGGGAAUUUUUUUUUAAUUUAAAAAAUUCCCUUGUAGAUAACCCGGUGGUCAGGUUGGAUGGCUCCAUG chr3 55886608 rs28647346 C U null null 15
hsa-mir-3938 AGGAAUUUUUAACCCGAUCACUAGAUUAUCUACAAGGGAAUUUUUUUUUAAUUUAAAAAAUUCCCUUGUAGAUAACCCGGUGGUCAGGUUGGAUGGCUCCAUG chr3 55886581 rs10575780 UU

- null null 42
hsa-mir-3938
AGGAAUUUUUAACCCGAUCACUAGAUUAUCUACAAGGGAAUUUUUUUUUAAUUAAAAAAUUCCCUUG
UAGAUAACCCGGUGGUCAGGUUGGAUGGCUCCAUG chr3 55886574 rs59684995 U
A null null 49
hsa-mir-3938
AGGAAUUUUUAACCCGAUCACUAGAUUAUCUACAAGGGAAUUUUUUUUUAAUUAAAAAAUUCCCUUG
UAGAUAACCCGGUGGUCAGGUUGGAUGGCUCCAUG chr3 55886522 rs35071197 -
C null null 101
hsa-mir-3939
CUGGCUUCCAAAGGCCUCUGUGUGUUCCUGUAUGUGGGCGUGCACGUACCUGUCACAUGUGUACGCGC
AGACCACAGGAUGUCCACACUGGCUUCCAAACACAUCU chr6 167411388 rs73024234 G
C null null 13
hsa-mir-3939
CUGGCUUCCAAAGGCCUCUGUGUGUUCCUGUAUGUGGGCGUGCACGUACCUGUCACAUGUGUACGCGC
AGACCACAGGAUGUCCACACUGGCUUCCAAACACAUCU chr6 167411333 rs76608449 C
G null null 60
hsa-mir-3939
CUGGCUUCCAAAGGCCUCUGUGUGUUCCUGUAUGUGGGCGUGCACGUACCUGUCACAUGUGUACGCGC
AGACCACAGGAUGUCCACACUGGCUUCCAAACACAUCU chr6 167411334 rs75823810 G
A null null 61
hsa-mir-3939
CUGGCUUCCAAAGGCCUCUGUGUGUUCCUGUAUGUGGGCGUGCACGUACCUGUCACAUGUGUACGCGC
AGACCACAGGAUGUCCACACUGGCUUCCAAACACAUCU chr6 167411298 rs80032204 A
G null null 97
hsa-mir-3939
CUGGCUUCCAAAGGCCUCUGUGUGUUCCUGUAUGUGGGCGUGCACGUACCUGUCACAUGUGUACGCGC
AGACCACAGGAUGUCCACACUGGCUUCCAAACACAUCU chr6 167411300 rs77840042 A
G null null 99
hsa-mir-3939
CUGGCUUCCAAAGGCCUCUGUGUGUUCCUGUAUGUGGGCGUGCACGUACCUGUCACAUGUGUACGCGC
AGACCACAGGAUGUCCACACUGGCUUCCAAACACAUCU chr6 167411301 rs75692943 C
U null null 100
hsa-mir-3945
GAUGUUGAUGCACGUGACGGGGAGGGCAUAGGAGAGGGUUGAUAUAAAAUGCAAUUACAGCCUCUUAU
GCUUUCCAAAGUGGGGGAUGAUUCAAUGAU chr4 185772247 rs75297112 C U
null null 18
hsa-mir-409
UGGUACUCGGGGAGAGGUUACCCGAGCAACUUUGCAUCUGGACGACGAAUGUUGCUCGGUGAACCCCU
UUUCGGUAUCA chr14 101531654 . U A 0.000199681 + 18

hsa-mir-410
GGUACCUGAGAAGAGGUUGUCUGUGAUGAGUUCGCUUUUAUUAAUGACGAAUAUAACACAGAUGGCCU
GUUUUCAGUACC chr14 101532279 . U A 0.000199681 + 31

hsa-mir-411
UGGUACUUGGAGAGAUAGUAGACCGUAUAGCGUACGCUUUAUCUGUGACGUAUGUAACACGGUCCACU

AACCCUCAGUAUCAAAUCCAUCCCCGAG chr14 101489677 . U C
0.000199681 + 16
hsa-mir-411
UGGUACUUGGAGAGAUAGUAGACCGUAUAGCGUACGCUUUAUCUGUGACGUAUGUAACACGGUCCACU
AACCCUCAGUAUCAAAUCCAUCCCCGAG chr14 101489685 . C U
0.000199681 + 24
hsa-mir-411
UGGUACUUGGAGAGAUAGUAGACCGUAUAGCGUACGCUUUAUCUGUGACGUAUGUAACACGGUCCACU
AACCCUCAGUAUCAAAUCCAUCCCCGAG chr14 101489703 rs111906529 U C
0.0145767 + 42
hsa-mir-411
UGGUACUUGGAGAGAUAGUAGACCGUAUAGCGUACGCUUUAUCUGUGACGUAUGUAACACGGUCCACU
AACCCUCAGUAUCAAAUCCAUCCCCGAG chr14 101489714 rs111835650 U C
null null 53
hsa-mir-411
UGGUACUUGGAGAGAUAGUAGACCGUAUAGCGUACGCUUUAUCUGUGACGUAUGUAACACGGUCCACU
AACCCUCAGUAUCAAAUCCAUCCCCGAG chr14 101489728 . C U
0.000199681 + 67
hsa-mir-411
UGGUACUUGGAGAGAUAGUAGACCGUAUAGCGUACGCUUUAUCUGUGACGUAUGUAACACGGUCCACU
AACCCUCAGUAUCAAAUCCAUCCCCGAG chr14 101489745 rs150717115 A G
0.00199681 + 84
hsa-mir-412
CUGGGGUACGGGGAUGGAUGGUCGACCAGUUGGAAAGUAAUUGUUUCUAAUGUACUUCACCUGGUCCA
CUAGCCGUCCGUAUCCGCUGCAG chr14 101531806 rs115831106 C U
0.00199681 + 23
hsa-mir-412
CUGGGGUACGGGGAUGGAUGGUCGACCAGUUGGAAAGUAAUUGUUUCUAAUGUACUUCACCUGGUCCA
CUAGCCGUCCGUAUCCGCUGCAG chr14 101531849 . C U
0.000199681 + 66
hsa-mir-412
CUGGGGUACGGGGAUGGAUGGUCGACCAGUUGGAAAGUAAUUGUUUCUAAUGUACUUCACCUGGUCCA
CUAGCCGUCCGUAUCCGCUGCAG chr14 101531854 rs61992671 A G
0.189097 + 71
hsa-mir-412
CUGGGGUACGGGGAUGGAUGGUCGACCAGUUGGAAAGUAAUUGUUUCUAAUGUACUUCACCUGGUCCA
CUAGCCGUCCGUAUCCGCUGCAG chr14 101531857 rs139967426 C U
0.00239617 + 74
hsa-mir-412
CUGGGGUACGGGGAUGGAUGGUCGACCAGUUGGAAAGUAAUUGUUUCUAAUGUACUUCACCUGGUCCA
CUAGCCGUCCGUAUCCGCUGCAG chr14 101531858 . G A
0.000399361 + 75
hsa-mir-412
CUGGGGUACGGGGAUGGAUGGUCGACCAGUUGGAAAGUAAUUGUUUCUAAUGUACUUCACCUGGUCCA
CUAGCCGUCCGUAUCCGCUGCAG chr14 101531862 rs368964407 G A
0.000199681 + 79
hsa-mir-421

GCACAUUGUAGGCCUCAUUAAAUGUUUGUUGAAUGAAAAAAUGAAUCAUCAACAGACAUUAAUUGGGC GCCUGCUCUGUGAUCUC chrX 73438224 rs372055341 G A 0.00264901 - 73

hsa-mir-423 AUAAAGGAAGUUAGGCUGAGGGGCAGAGAGCGAGACUUUUCUAUUUUCCAAAAGCUCGGUCUGAGGCC CCUCAGUCUUGCUUCCUAACCCGCGCchr17 28444104 . A U 0.000199681 + 8

hsa-mir-423 AUAAAGGAAGUUAGGCUGAGGGGCAGAGAGCGAGACUUUUCUAUUUUCCAAAAGCUCGGUCUGAGGCC CCUCAGUCUUGCUUCCUAACCCGCGCchr17 28444157 . C U 0.000199681 + 61

hsa-mir-423 AUAAAGGAAGUUAGGCUGAGGGGCAGAGAGCGAGACUUUUCUAUUUUCCAAAAGCUCGGUCUGAGGCC CCUCAGUCUUGCUUCCUAACCCGCGCchr17 28444183 rs6505162 A C 0.497804 + 87

hsa-mir-424 CGAGGGGAUACAGCAGCAAUUCAUGUUUUGAAGUGUUCUAAAUGGUUCAAAACGUGAGGCGCUGCUAU ACCCCCUCGUGGGGAAGGUAGAAGGUGGGG chrX 133680673 . A G 0.000264901 - 69

hsa-mir-425 GAAAGCGCUUUGGAAUGACACGAUCACUCCCGUUGAGUGGGCACCCGAGAAGCCAUCGGGAAUGUCGU GUCCGCCCAGUGCUCUUUC chr3 49057623 rs370209295 C U 0.000199681 - 45

hsa-mir-4252 UGGGGGGCUGGCAGCUCAUCAGUCCAGGCCAUCUGGCCACUGAGUCAGCACCAGCGCCCAAUC chr1 6489914 rs3982073 A G null null 43

hsa-mir-4252 UGGGGGGCUGGCAGCUCAUCAGUCCAGGCCAUCUGGCCACUGAGUCAGCACCAGCGCCCAAUC chr1 6489910 rs3982072 A G null null 47

hsa-mir-4253 CCAGCCAUCGCCCUUGAGGGGCCCUAGGACUUACUUGUGCAGGGCAUGUCCAGGGGGUCCAGGUCUGC chr1 23189715 rs111569109 C G null null 5

hsa-mir-4253 CCAGCCAUCGCCCUUGAGGGGCCCUAGGACUUACUUGUGCAGGGCAUGUCCAGGGGGUCCAGGUCUGC chr1 23189707 rs66652227 C - null null 13

hsa-mir-4254 CUUGGGAGGAGGGUGGGGUGGCUCCUCUGCAGUGAGUAGGUCUGCCUGGAGCUACUCCACCAUCUCCC CCAGCCCC chr1 32224285 rs12731294 C A null null 52

hsa-mir-4257 GGCUUAGAAACAGUCCCUAGGUAGGAUUUGGGGAGGAGCUAAGAAGCCCCUACAGGGCCCAGAGGUGG GGACUGAGCCUUAGUUGG chr1 150524468 rs74743733 G A null null 64

hsa-mir-4258
ACGCCCCCCGCCCCGCCACCGCCUUGGAGGCUGACCUCUUACUUUCGGUCGGUCUUCUUCCCUGGGCUUGGUUUGGGGGCGGGGGAGUGUC chr1 154948201 rs112752440 G C null null 27
hsa-mir-4258
ACGCCCCCCGCCCCGCCACCGCCUUGGAGGCUGACCUCUUACUUUCGGUCGGUCUUCUUCCCUGGGCUUGGUUUGGGGGCGGGGGAGUGUC chr1 154948195 rs113220875 G C null null 33
hsa-mir-4259
GAUGGGCCCCUUGUGUCCUGAAUUGGGUGGGGGCUCUGAGUGGGGAAAGUGGGGGCCUAGGGGAGGUCACAGUUGGGUCUAGGGGUCAGGAGGGCCCAGGA chr1 159869828 rs35180578 - G null null 42
hsa-mir-4260
AACAAGGUGACUUGGGGCAUGGAGUCCCACUUCCUGGAGCCCACACCCCAGCUUGUCACACACCAAC chr1 209796849 rs111724706 G C null null 7

hsa-mir-4260
AACAAGGUGACUUGGGGCAUGGAGUCCCACUUCCUGGAGCCCACACCCCAGCUUGUCACACACCAAC chr1 209796810 rs7544976 C A null null 46

hsa-mir-4265
UGCAGUGGGUUGGAGCUUCAGCCUACACCUGUAAAGAAUUGGUCAGCCUGGGGACUGGUGAUCUCUGCAGCUGUGGGCUCAGCUCUGGGCUGGGCCUGG chr2 109757963 rs4676066 G A null null 82
hsa-mir-4267
CUCAGCAGGCUCCAGCUCGGUGGCACUGGGGGAAGGCUCCAGACCCCAGCCUCUGUCAUCCCUGCAUGGAGCCCACAUCUCC chr2 110827558 rs59793833 C U null null 62
hsa-mir-4267
CUCAGCAGGCUCCAGCUCGGUGGCACUGGGGGAAGGCUCCAGACCCCAGCCUCUGUCAUCCCUGCAUGGAGCCCACAUCUCC chr2 110827551 rs55940978 G A null null 69
hsa-mir-4268
AUGCACAUCAGGUUCUAGAGGUUUUGCCCUAGCGGCUCCUCCUCUCAGGAUGUGAUGUCACCUG chr2 220771223 rs4674470 G A null null 64

hsa-mir-4269
ACAGCGCCCUGCAGGCACAGACAGCCCUGGCUUCUGCCUCUUUCUUUGUGGAAGCCACUCUGUCAGGCCUGGGAUGGAGGGGCA chr2 240227178 rs112544913 C U null null 22
hsa-mir-4270
ACAAAUAGCUUCAGGGAGUCAGGGGAGGGCAGAAAUAGAUGGCCUUCCCCUGCUGGGAAGAAAGUGGGUC chr3 15537776 rs79922312 U C null null 40

hsa-mir-4274
GGGGCAUUUAGGGUAACUGAGCUGCUGCCGGGGCCUGGCGCUCCUCUACCUUGUCAGGUGACCCAGCAGUCCCUCCCCCUGCAUGGUGCCC chr4 7461769 rs12512664 A G

null null 15
hsa-mir-4274
GGGGCAUUUAGGGUAACUGAGCUGCUGCCGGGGCCUGGCGCUCCUCUACCUUGUCAGGUGACCCAGCA GUCCCUCCCCCUGCAUGGUGCCC chr4 7461826 rs10655902 - C null null 72
hsa-mir-4274
GGGGCAUUUAGGGUAACUGAGCUGCUGCCGGGGCCUGGCGCUCCUCUACCUUGUCAGGUGACCCAGCA GUCCCUCCCCCUGCAUGGUGCCC chr4 7461827 rs35245133 - C null null 73
hsa-mir-4274
GGGGCAUUUAGGGUAACUGAGCUGCUGCCGGGGCCUGGCGCUCCUCUACCUUGUCAGGUGACCCAGCA GUCCCUCCCCCUGCAUGGUGCCC chr4 7461828 rs75530384 U C null null 74
hsa-mir-4274
GGGGCAUUUAGGGUAACUGAGCUGCUGCCGGGGCCUGGCGCUCCUCUACCUUGUCAGGUGACCCAGCA GUCCCUCCCCCUGCAUGGUGCCC chr4 7461828 rs66511565 - AC null null 74
hsa-mir-4277
CUGGGUCGAGGCAGUUCUGAGCACAGUACACUGGGCUGCCCCCACUGCCCAGUGCCCUGCUCAGCUCA AGUCCUUGUGCCCCUC chr5 1708983 rs12523324 C U null null 1
hsa-mir-4277
CUGGGUCGAGGCAGUUCUGAGCACAGUACACUGGGCUGCCCCCACUGCCCAGUGCCCUGCUCAGCUCA AGUCCUUGUGCCCCUC chr5 1708902 rs115200817 C U null null 82
hsa-mir-4278
AUCUAACACCAGGAGAAUCCCAUAGAACAUUGACAUCAACACUAGGGGGUUUGCCCUUGUGGGGAAGA A chr5 6828032 rs116946264 C U null null 3

hsa-mir-4279 UGCUCUGUGGAGCUGAGGAGCAGAUUCUCUCUCUCUCCUCCCGGCUUCACCUCCUGAG chr5 31936263 rs34157017 - U null null 3

hsa-mir-4280
AAUCAGGGUGGAGUGUAGUUCUGAGCAGAGCCUUAAAGGAUGAGGUAUGUUCAAGACUGAAUGACACC UUUGUGAU chr5 86410766 rs116213678 G C null null 6

hsa-mir-4281 GCUGGGGGUCCCCCGACAGUGUGGAGCUGGGGCCGGGUCCCGGGGAGGGGGGUUCUGGGCAG chr5 176056492 rs34983866 C - null null 10

hsa-mir-4283-1
ACUCUGAUCCUGGGGCUCAGCGAGUUUGCAAGGGGUGUUUCUGUCCAUGGUCAGGCUUGCCAGCCUUG GUCCUUGGGCCC chr7 57023497 rs2312120 G A null null 75

hsa-mir-4283-2
ACUCUGAUCCUGGGGCUCAGCGAGUUUGCAAGGGGUGUUUCUGUCCAUGGUCAGGCUUGCCAGCCUUG GUCCUUGGGCCC chr7 63081542 rs2312120 G A null null 75

hsa-mir-4284
GUUCUGUGAGGGGCUCACAUCACCCCAUCAAAGUGGGGACUCAUGGGGAGAGGGGGUAGUUAGGAGCU
UUGAUAGAGGCGGchr7 73125664 rs11973069 C U null null 18

hsa-mir-4285
AUUAGGCGGGGCGGCGAGUCCGACUCAUAAAUAUUUUAAGGAAUGACCCGGCCCUGGGGUGCGGAAUU
GCUGCGCGGGCGGGGGC chr7 101936372 rs34606939 - G null
null 4
hsa-mir-4285
AUUAGGCGGGGCGGCGAGUCCGACUCAUAAAUAUUUUAAGGAAUGACCCGGCCCUGGGGUGCGGAAUU
GCUGCGCGGGCGGGGGC chr7 101936374 rs2960269 C G null
null 6
hsa-mir-4285
AUUAGGCGGGGCGGCGAGUCCGACUCAUAAAUAUUUUAAGGAAUGACCCGGCCCUGGGGUGCGGAAUU
GCUGCGCGGGCGGGGGC chr7 101936375 rs2906714 U C null
null 7
hsa-mir-4285
AUUAGGCGGGGCGGCGAGUCCGACUCAUAAAUAUUUUAAGGAAUGACCCGGCCCUGGGGUGCGGAAUU
GCUGCGCGGGCGGGGGC chr7 101936397 rs1293864 C A null
null 29
hsa-mir-4285
AUUAGGCGGGGCGGCGAGUCCGACUCAUAAAUAUUUUAAGGAAUGACCCGGCCCUGGGGUGCGGAAUU
GCUGCGCGGGCGGGGGC chr7 101936422 rs1293863 U C null
null 54
hsa-mir-4285
AUUAGGCGGGGCGGCGAGUCCGACUCAUAAAUAUUUUAAGGAAUGACCCGGCCCUGGGGUGCGGAAUU
GCUGCGCGGGCGGGGGC chr7 101936435 rs1293862 U A null
null 67
hsa-mir-4286
UACUUAUGGCACCCCACUCCUGGUACCAUAGUCAUAAGUUAGGAGAUGUUAGAGCUGUGAGUACCAUG
ACUUAAGUGUGGUGGCUUAAACAUG chr8 10524531 rs80245767 A C
null null 44
hsa-mir-4289
CCUUGGGAGGGCAUUGUGCAGGGCUAUCAGGCAGUUUCCUGGGCCCUGUCUGCAGAGCCUAAACAGAU
CA chr9 91360757 rs116328267 C A null null 64

hsa-mir-429
CGCCGGCCGAUGGGCGUCUUACCAGACAUGGUUAGACCUGGCCCUCUGUCUAAUACUGUCUGGUAAAA
CCGUCCAUCCGCUGC chr1 1104412 rs183812340 A G
0.000399361 + 28
hsa-mir-429
CGCCGGCCGAUGGGCGUCUUACCAGACAUGGUUAGACCUGGCCCUCUGUCUAAUACUGUCUGGUAAAA
CCGUCCAUCCGCUGC chr1 1104462 rs201269584 C U
0.000199681 + 78
hsa-mir-4293
AGAGACACCUGUUCCUUGGGAAGCUGGUGACAUUGCUAAUUCAUUUCACACCAGCCUGACAGGAACAG
CCUGACUGAA chr10 14425221 rs12220909 C G null null 56

hsa-mir-4293
AGAGACACCUGUUCCUUGGGAAGCUGGUGACAUUGCUAAUUCAUUUCACACCAGCCUGACAGGAACAG
CCUGACUGAA chr10 14425204 rs12780876 A U null null 73

hsa-mir-4294
CCGAUGCCUCGGGAGUCUACAGCAGGGCCAUGUCUGUGAGGGCCCAAGGGUGCAUGUGUCUCCCAGGU
UUCGGUGC chr10 50193625 rs12776349 C U null null 8

hsa-mir-4296
UUGGGCUUUGAUGUGGGCUCAGGCUCAGAGGGCUGAAGUGGUUGUGGGGAGGGGCUUCUGGGGACUGU
GUCCAUGUCUCUGUCGUUUU chr10 126721419 rs113642938 A G
null null 21
hsa-mir-4296
UUGGGCUUUGAUGUGGGCUCAGGCUCAGAGGGCUGAAGUGGUUGUGGGGAGGGGCUUCUGGGGACUGU
GUCCAUGUCUCUGUCGUUUU chr10 126721357 rs115700299 C U
null null 83
hsa-mir-4297
AGCACGCACGUGCCUUCCUGUCUGUGCCUGCCUUCGAAGUGCACGGCAGGGCCAGGACGGGUCGCUGU
GGGUGGGG chr10 131641629 rs114362263 G A null null 10

hsa-mir-4298
GGGGAGGUACCUGGGACAGGAGGAGGAGGCAGCCUUGCCUCAGAAACCAAACUGUCAAAAGUGUAGGU
UCCAC chr11 1880764 rs112839927 G U null null 3

hsa-mir-4298
GGGGAGGUACCUGGGACAGGAGGAGGAGGCAGCCUUGCCUCAGAAACCAAACUGUCAAAAGUGUAGGU
UCCAC chr11 1880730 rs75966923 G U null null 37

hsa-mir-4301
ACCAGCCACCUCCCACUACUUCACUUGUGAACAUUGCAUUCGUGGAGGGUGGCAGGUGCAGCUCUG
chr11 113320747 rs113391897 C G null null 64

hsa-mir-4302 UCAGGAGGGACCAGUGUGGCUCAGCGAGGUGGCUGAGUUUACUUAAGGUAUUGGAAUGAG
chr12 26026988 rs11048315 C U null null 25

hsa-mir-4305
CUGCCUUAGACCUAGACACCUCCAGUUCUGGGUUCUUAGAGGCCUAAUCCUCUACAAACUCAGUUUUC
AGACUGUGAGGGAAAUUCUCUGUCUUAUUGCUUU chr13 40238258 rs4636784 G
C null null 15
hsa-mir-4305
CUGCCUUAGACCUAGACACCUCCAGUUCUGGGUUCUUAGAGGCCUAAUCCUCUACAAACUCAGUUUUC
AGACUGUGAGGGAAAUUCUCUGUCUUAUUGCUUU chr13 40238175 rs67976778 G
A null null 98
hsa-mir-4308
UAUGGGUUCAGAGGGAACUCCAUUGGACAGAAAUUUCCUUUUGAGGAAAUCUUUCCCUGGAGUUUCUU
CUUACCUUUUUCCchr14 55344901 rs28477407 G A null null 11

hsa-mir-4309
UCUGGGGGUUCUGGAGUCUAGGAUUCCAGGAUCUGGGUUUUGAGGUCUUGGGUUGUAGGGUCUGCGGUUUGAAGCCCCUCUUG chr14 103006026 rs78860350 U A null null 46

hsa-mir-4309
UCUGGGGGUUCUGGAGUCUAGGAUUCCAGGAUCUGGGUUUUGAGGUCUUGGGUUGUAGGGUCUGCGGUUUGAAGCCCCUCUUG chr14 103006047 rs12879262 G C null null 67

hsa-mir-431
UCCUGCUUGUCCUGCGAGGUGUCUUGCAGGCCGUCAUGCAGGCCACACUGACGGUAACGUUGCAGGUCGUCUUGCAGGGCUUCUCGCAAGACGACAUCCUCAUCACCAACGACG chr14 101347355 rs76090066 C U 0.000599042 + 12

hsa-mir-431
UCCUGCUUGUCCUGCGAGGUGUCUUGCAGGCCGUCAUGCAGGCCACACUGACGGUAACGUUGCAGGUCGUCUUGCAGGGCUUCUCGCAAGACGACAUCCUCAUCACCAACGACG chr14 101347408 rs12884005 G A null null 65

hsa-mir-431
UCCUGCUUGUCCUGCGAGGUGUCUUGCAGGCCGUCAUGCAGGCCACACUGACGGUAACGUUGCAGGUCGUCUUGCAGGGCUUCUCGCAAGACGACAUCCUCAUCACCAACGACG chr14 101347448 rs138412993 A G 0.00179712 + 105

hsa-mir-431
UCCUGCUUGUCCUGCGAGGUGUCUUGCAGGCCGUCAUGCAGGCCACACUGACGGUAACGUUGCAGGUCGUCUUGCAGGGCUUCUCGCAAGACGACAUCCUCAUCACCAACGACG chr14 101347453 rs375169836 C U 0.000199681 + 110

hsa-mir-431
UCCUGCUUGUCCUGCGAGGUGUCUUGCAGGCCGUCAUGCAGGCCACACUGACGGUAACGUUGCAGGUCGUCUUGCAGGGCUUCUCGCAAGACGACAUCCUCAUCACCAACGACG chr14 101347456 . C U 0.000199681 + 113

hsa-mir-4312
GAAAGGUUGGGGGCACAGAGAGCAAGGAGCCUUCCCCAGAGGAGUCAGGCCUUGUUCCUGUCCCCAUUCCUCAGAG chr15 69094259 rs79711957 G U null null 6

hsa-mir-4312
GAAAGGUUGGGGGCACAGAGAGCAAGGAGCCUUCCCCAGAGGAGUCAGGCCUUGUUCCUGUCCCCAUUCCUCAGAG chr15 69094207 rs115780816 C U null null 58

hsa-mir-4317
AAAAGGCGAGACAUUGCCAGGGAGUUUAUUUUGUAGCUCUCUUGAUAAAAUGUUUUAGCAAACAC chr18 6374394 rs74513330 U C null null 31

hsa-mir-4317
AAAAGGCGAGACAUUGCCAGGGAGUUUAUUUUGUAGCUCUCUUGAUAAAAUGUUUUAGCAAACAC

chr18 6374387 rs115096233 U G null null 38

hsa-mir-4318
GCUUCUUAAUUAUGUCAUAAACCCACUGUGGACAAGGGCCUUGUCUUAGACAGUCACUGUGGGUACAU
GCUAGGUGCUCAAchr18 35237129 rs11873400 A G null null 32

hsa-mir-4318
GCUUCUUAAUUAUGUCAUAAACCCACUGUGGACAAGGGCCUUGUCUUAGACAGUCACUGUGGGUACAU
GCUAGGUGCUCAAchr18 35237143 rs117767519 U G null null 46

hsa-mir-432
UGACUCCUCCAGGUCUUGGAGUAGGUCAUUGGGUGGAUCCUCUAUUUCCUUACGUGGGCCACUGGAUG
GCUCCUCCAUGUCUUGGAGUAGAUCAchr14 101350873 rs373861350 G U
0.000399361 + 54

hsa-mir-4320
GACAUGUGGGGUUUGCUGUAGACAUUUCAGAUAACUCGGGAUUCUGUAGCUUCCUGGCAACUUUG
chr18 47652913 rs4430847 G A null null 21

hsa-mir-4321
CUGGUCUCCGCAGAGCCUCUGCCCCUCCCGAGACACCCGCUACCUGGUGUUAGCGGUGGACCGCCCUG
CGGGGGCCUGGC chr19 2250666 rs104894666 C U null null 29

hsa-mir-4322
ACCGCGAGUUCCGCGCCUGGCCGUGUCGCCCCACGAGGGGGACUGUGGGCUCAGCGCGUGGGGCCCGG
AGCAU chr19 10341137 rs114399468 G A null null 49

hsa-mir-4324
CGGCCCCUUUGUUAAGGGUCUCAGCUCCAGGGAACUUUAAAACCCUGAGACCCUAACCUUAAAGGUGC
UGCA chr19 49812123 rs79465964 G A null null 3

hsa-mir-4326 GCUGCUCUGCUGUUCCUCUGUCUCCCAGACUCUGGGUGGAUGGAGCAGGUCGGGGGCCA
chr20 61918164 rs6062431 C G null null 5

hsa-mir-4327
GGCCUGGGUAGGCUUGCAUGGGGGACUGGGAAGAGACCAUGAACAGGUUAGUCCAGGGAGUUCUCAUC
AAGCCUUUACUCAGUAG chr21 31747678 rs115477019 U G null
null 19

hsa-mir-4328 AACAGUUGAGUCCUGAGAACCAUUGAGAACCAGUUUUCCCAGGAUUAACUGUUCCG
chrX 78156693 rs35376760 - C null null 54

hsa-mir-433
CCGGGGAGAAGUACGGUGAGCCUGUCAUUAUUCAGAGAGGCUAGAUCCUCUGUGUUGAGAAGGAUCAU
GAUGGGCUCCUCGGUGUUCUCCAGG chr14 101348275 . U A
0.000399361 + 53

hsa-mir-448
GCCGGGAGGUUGAACAUCCUGCAUAGUGCUGCCAGGAAAUCCCUAUUUCAUAUAAGAGGGGGCUGGCU
GGUUGCAUAUGUAGGAUGUCCCAUCUCCCAGCCCACUUCGUCA chrX 114058104 . C

U 0.000264901 + 88
hsa-mir-449a
CUGUGUGUGAUGAGCUGGCAGUGUAUUGUUAGCUGGUUGAAUAUGUGAAUGGCAUCGGCUAACAUGCA ACUGCUGUCUUAUUGCAUAUACA chr5 54466378 rs112310158 C U
0.0071885 - 73
hsa-mir-449a
CUGUGUGUGAUGAGCUGGCAGUGUAUUGUUAGCUGGUUGAAUAUGUGAAUGGCAUCGGCUAACAUGCA ACUGCUGUCUUAUUGCAUAUACA chr5 54466362 rs144949536 A G
0.00139776 - 89
hsa-mir-449b
UGACCUGAAUCAGGUAGGCAGUGUAUUGUUAGCUGGCUGCUUGGGUCAAGUCAGCAGCCACAACUACC CUGCCACUUGCUUCUGGAUAAAUUCUUCU chr5 54466544 rs10061133 U C
0.122204 - 27
hsa-mir-449b
UGACCUGAAUCAGGUAGGCAGUGUAUUGUUAGCUGGCUGCUUGGGUCAAGUCAGCAGCCACAACUACC CUGCCACUUGCUUCUGGAUAAAUUCUUCU chr5 54466527 . G C
0.000199681 - 44
hsa-mir-449c
GCUGGGAUGUGUCAGGUAGGCAGUGUAUUGCUAGCGGCUGUUAAUGAUUUUAACAGUUGCUAGUUGCA CUCCUCUCUGUUGCAUUCAGAAGC chr5 54468166 rs75661995 G A
0.000998403 - 16
hsa-mir-449c
GCUGGGAUGUGUCAGGUAGGCAGUGUAUUGCUAGCGGCUGUUAAUGAUUUUAACAGUUGCUAGUUGCA CUCCUCUCUGUUGCAUUCAGAAGC chr5 54468150 rs146466616 U C
0.000599042 - 32
hsa-mir-449c
GCUGGGAUGUGUCAGGUAGGCAGUGUAUUGCUAGCGGCUGUUAAUGAUUUUAACAGUUGCUAGUUGCA CUCCUCUCUGUUGCAUUCAGAAGC chr5 54468144 . C G
0.000199681 - 38
hsa-mir-449c
GCUGGGAUGUGUCAGGUAGGCAGUGUAUUGCUAGCGGCUGUUAAUGAUUUUAACAGUUGCUAGUUGCA CUCCUCUCUGUUGCAUUCAGAAGC chr5 54468110 . C U
0.000399361 - 72
hsa-mir-449c
GCUGGGAUGUGUCAGGUAGGCAGUGUAUUGCUAGCGGCUGUUAAUGAUUUUAACAGUUGCUAGUUGCA CUCCUCUCUGUUGCAUUCAGAAGC chr5 54468124 rs35770269 U A
null null 73
hsa-mir-449c
GCUGGGAUGUGUCAGGUAGGCAGUGUAUUGCUAGCGGCUGUUAAUGAUUUUAACAGUUGCUAGUUGCA CUCCUCUCUGUUGCAUUCAGAAGC chr5 54468094 rs112705432 G A
0.0115815 - 88
hsa-mir-450a-1
AAACGAUACUAAACUGUUUUUGCGAUGUGUUCCUAAUAUGCACUAUAAAUAUAUUGGGAACAUUUUGC AUGUAUAGUUUUGUAUCAAUAUA chrX 133674439 . C U
0.000529801 - 23
hsa-mir-450a-1
AAACGAUACUAAACUGUUUUUGCGAUGUGUUCCUAAUAUGCACUAUAAAUAUAUUGGGAACAUUUUGC

AUGUAUAGUUUUGUAUCAAUAUA chrX 133674418 . U C
0.000264901 - 44
hsa-mir-450a-1
AAACGAUACUAAACUGUUUUUGCGAUGUGUUCCUAAUAUGCACUAUAAAUAUAUUGGGAACAUUUUGC
AUGUAUAGUUUUGUAUCAAUAUA chrX 133674399 rs181664033 U C
0.000529801 - 63
hsa-mir-450a-2
CCAAAGAAAGAUGCUAAACUAUUUUUGCGAUGUGUUCCUAAUAUGUAAUAUAAAUGUAUUGGGGACAU
UUUGCAUUCAUAGUUUUGUAUCAAUAAUAUGG chrX 133674612 rs141784874 U C
0.0015894 - 26
hsa-mir-450a-2
CCAAAGAAAGAUGCUAAACUAUUUUUGCGAUGUGUUCCUAAUAUGUAAUAUAAAUGUAUUGGGGACAU
UUUGCAUUCAUAGUUUUGUAUCAAUAAUAUGG chrX 133674576 rs185256782 G C
0.000264901 - 62
hsa-mir-450b
GCAGAAUUAUUUUUGCAAUAUGUUCCUGAAUAUGUAAUAUAAGUGUAUUGGGAUCAUUUUGCAUCCAU
AGUUUUGUAU chrX 133674285 . U G 0.000264901 - 8

hsa-mir-452
GCUAAGCACUUACAACUGUUUGCAGAGGAAACUGAGACUUUGUAACUAUGUCUCAGUCUCAUCUGCAA
AGAAGUAAGUGCUUUGC chrX 151128173 . A G 0.000529801 -
12
hsa-mir-454
UCUGUUUAUCACCAGAUCCUAGAACCCUAUCAAUAUUGUCUCUGCUGUGUAAAUAGUUCUGAGUAGUG
CAAUAUUGCUUAUAGGGUUUUGGUGUUUGGAAAGAACAAUGGGCAGG chr17 57215164 .
A C 0.000199681 - 70
hsa-mir-454
UCUGUUUAUCACCAGAUCCUAGAACCCUAUCAAUAUUGUCUCUGCUGUGUAAAUAGUUCUGAGUAGUG
CAAUAUUGCUUAUAGGGUUUUGGUGUUUGGAAAGAACAAUGGGCAGG chr17 57215120 .
G U 0.000199681 - 114
hsa-mir-455
UCCCUGGCGUGAGGGUAUGUGCCUUUGGACUACAUCGUGGAAGCCAGCACCAUGCAGUCCAUGGGCAU
AUACACUUGCCUCAAGGCCUAUGUCAUC chr9 116971750 . G A
0.000599042 + 37
hsa-mir-455
UCCCUGGCGUGAGGGUAUGUGCCUUUGGACUACAUCGUGGAAGCCAGCACCAUGCAGUCCAUGGGCAU
AUACACUUGCCUCAAGGCCUAUGUCAUC chr9 116971778 . G U
0.000199681 + 65
hsa-mir-466
GUGUGUGUAUAUGUGUGUUGCAUGUGUGUAUAUGUGUGUAUAUAUGUACACAUACACAUACACGCAAC
ACACAUAUAUACAUGC chr3 31203277 . G U 0.000199681 -
3
hsa-mir-466
GUGUGUGUAUAUGUGUGUUGCAUGUGUGUAUAUGUGUGUAUAUAUGUACACAUACACAUACACGCAAC
ACACAUAUAUACAUGC chr3 31203267 rs115897337 G U null
null 13
hsa-mir-466

GUGUGUGUAUAUGUGUGUUGCAUGUGUGUAUAUGUGUGUAUAUAUGUACACAUACACAUACACGCAACACACAUAUAUACAUGC chr3 31203228 . A G 0.000199681 - 52

hsa-mir-466 GUGUGUGUAUAUGUGUGUUGCAUGUGUGUAUAUGUGUGUAUAUAUGUACACAUACACAUACACGCAACACACAUAUAUACAUGC chr3 31203221 rs116476604 U C 0.00519169 - 59

hsa-mir-466 GUGUGUGUAUAUGUGUGUUGCAUGUGUGUAUAUGUGUGUAUAUAUGUACACAUACACAUACACGCAACACACAUAUAUACAUGC chr3 31203216 . G A 0.000399361 - 64

hsa-mir-466 GUGUGUGUAUAUGUGUGUUGCAUGUGUGUAUAUGUGUGUAUAUAUGUACACAUACACAUACACGCAACACACAUAUAUACAUGC chr3 31203207 rs148367480 A C 0.00299521 - 73

hsa-mir-483 GAGGGGGAAGACGGGAGGAAAGAAGGGAGUGGUUCCAUCACGCCUCCUCACUCCUCUCCUCCCGUCUUCUCCUCUC chr11 2155409 . G U 0.000199681 - 31

hsa-mir-483 GAGGGGGAAGACGGGAGGAAAGAAGGGAGUGGUUCCAUCACGCCUCCUCACUCCUCUCCUCCCGUCUUCUCCUCUC chr11 2155379 . C U 0.000199681 - 61

hsa-mir-484 AGCCUCGUCAGGCUCAGUCCCCUCCCGAUAAACCCCUAAAUAGGGACUUUCCCGGGGGGUGACCCUGGCUUUUUUGGCG chr16 15737151 . A G 0.000199681 + 1

hsa-mir-484 AGCCUCGUCAGGCUCAGUCCCCUCCCGAUAAACCCCUAAAUAGGGACUUUCCCGGGGGGUGACCCUGGCUUUUUUGGCG chr16 15737177 rs376603273 G A 0.000199681 + 27

hsa-mir-484 AGCCUCGUCAGGCUCAGUCCCCUCCCGAUAAACCCCUAAAUAGGGACUUUCCCGGGGGGUGACCCUGGCUUUUUUGGCG chr16 15737185 rs374184031 C A 0.000599042 + 35

hsa-mir-484 AGCCUCGUCAGGCUCAGUCCCCUCCCGAUAAACCCCUAAAUAGGGACUUUCCCGGGGGGUGACCCUGGCUUUUUUGGCG chr16 15737186 rs368690836 C A 0.000798722 + 36

hsa-mir-484 AGCCUCGUCAGGCUCAGUCCCCUCCCGAUAAACCCCUAAAUAGGGACUUUCCCGGGGGGUGACCCUGGCUUUUUUGGCG chr16 15737217 rs112103086 G U null null 67

hsa-mir-484 AGCCUCGUCAGGCUCAGUCCCCUCCCGAUAAACCCCUAAAUAGGGACUUUCCCGGGGGGUGACCCUGGCUUUUUUGGCG chr16 15737218 rs112906136 G A null null 68

hsa-mir-485
ACUUGGAGAGAGGCUGGCCGUGAUGAAUUCGAUUCAUCAAAGCGAGUCAUACACGGCUCUCCUCUCUU
UUAGU chr14 101521764 rs112982830 A G null null 9

hsa-mir-486-1
GCAUCCUGUACUGAGCUGCCCCGAGGCCCUUCAUGCUGCCCAGCUCGGGGCAGCUCAGUACAGGAUAC
chr8 41518025 rs150478047 C A 0.000399361 - 2

hsa-mir-486-1
GCAUCCUGUACUGAGCUGCCCCGAGGCCCUUCAUGCUGCCCAGCUCGGGGCAGCUCAGUACAGGAUAC
chr8 41518007 . C G 0.000199681 - 20

hsa-mir-486-1
GCAUCCUGUACUGAGCUGCCCCGAGGCCCUUCAUGCUGCCCAGCUCGGGGCAGCUCAGUACAGGAUAC
chr8 41518005 rs367815925 C U 0.000199681 - 22

hsa-mir-486-1
GCAUCCUGUACUGAGCUGCCCCGAGGCCCUUCAUGCUGCCCAGCUCGGGGCAGCUCAGUACAGGAUAC
chr8 41517981 . C U 0.000599042 - 46

hsa-mir-486-1
GCAUCCUGUACUGAGCUGCCCCGAGGCCCUUCAUGCUGCCCAGCUCGGGGCAGCUCAGUACAGGAUAC
chr8 41517974 . G C 0.000199681 - 53

hsa-mir-486-2
UCCUGUACUGAGCUGCCCCGAGCUGGGCAGCAUGAAGGGCCUCGGGGCAGCUCAGUACAGGAUG
chr8 41517974 . C G 0.000199681 + 13

hsa-mir-486-2
UCCUGUACUGAGCUGCCCCGAGCUGGGCAGCAUGAAGGGCCUCGGGGCAGCUCAGUACAGGAUG
chr8 41517981 . G A 0.000599042 + 20

hsa-mir-486-2
UCCUGUACUGAGCUGCCCCGAGCUGGGCAGCAUGAAGGGCCUCGGGGCAGCUCAGUACAGGAUG
chr8 41518005 rs367815925 G A 0.000199681 + 44

hsa-mir-486-2
UCCUGUACUGAGCUGCCCCGAGCUGGGCAGCAUGAAGGGCCUCGGGGCAGCUCAGUACAGGAUG
chr8 41518007 . G C 0.000199681 + 46

hsa-mir-486-2
UCCUGUACUGAGCUGCCCCGAGCUGGGCAGCAUGAAGGGCCUCGGGGCAGCUCAGUACAGGAUG
chr8 41518025 rs150478047 G U 0.000399361 + 64

hsa-mir-487a
GGUACUUGAAGAGUGGUUAUCCCUGCUGUGUUCGCUUAAUUUAUGACGAAUCAUACAGGGACAUCCAG
UUUUUCAGUAUC chr14 101518827 rs143972054 G A 0.000599042 +

45
hsa-mir-487b
UUGGUACUUGGAGAGUGGUUAUCCCUGUCCUGUUCGUUUUGCUCAUGUCGAAUCGUACAGGGUCAUCC
ACUUUUUCAGUAUCAA chr14 101512836 rs145135208 A G 0.0161741
+ 45
hsa-mir-488
GAGAAUCAUCUCUCCCAGAUAAUGGCACUCUCAAACAAGUUUCCAAAUUGUUUGAAAGGCUAUUUCUU
GGUCAGAUGACUCUC chr1 176998548 rs181176070 A G
0.000199681 - 34
hsa-mir-489
GUGGCAGCUUGGUGGUCGUAUGUGUGACGCCAUUUACUUGAACCUUUAGGAGUGACAUCACAUAUACG
GCAGCUAAACUGCUAC chr7 93113326 rs370552317 A U
0.000199681 - 6
hsa-mir-489
GUGGCAGCUUGGUGGUCGUAUGUGUGACGCCAUUUACUUGAACCUUUAGGAGUGACAUCACAUAUACG
GCAGCUAAACUGCUAC chr7 93113323 rs35930643 - U null
null 9
hsa-mir-489
GUGGCAGCUUGGUGGUCGUAUGUGUGACGCCAUUUACUUGAACCUUUAGGAGUGACAUCACAUAUACG
GCAGCUAAACUGCUAC chr7 93113314 . G A 0.000199681 -
18
hsa-mir-489
GUGGCAGCUUGGUGGUCGUAUGUGUGACGCCAUUUACUUGAACCUUUAGGAGUGACAUCACAUAUACG
GCAGCUAAACUGCUAC chr7 93113270 . A G 0.000199681 -
62
hsa-mir-490
UGGAGGCCUUGCUGGUUUGGAAAGUUCAUUGUUCGACACCAUGGAUCUCCAGGUGGGUCAAGUUUAGA
GAUGCACCAACCUGGAGGACUCCAUGCUGUUGAGCUGUUCACAAGCAGCGGACACUUCCA chr7
136587919 . G C 0.000199681 + 6

hsa-mir-490
UGGAGGCCUUGCUGGUUUGGAAAGUUCAUUGUUCGACACCAUGGAUCUCCAGGUGGGUCAAGUUUAGA
GAUGCACCAACCUGGAGGACUCCAUGCUGUUGAGCUGUUCACAAGCAGCGGACACUUCCA chr7
136587934 . A G 0.000199681 + 21

hsa-mir-490
UGGAGGCCUUGCUGGUUUGGAAAGUUCAUUGUUCGACACCAUGGAUCUCCAGGUGGGUCAAGUUUAGA
GAUGCACCAACCUGGAGGACUCCAUGCUGUUGAGCUGUUCACAAGCAGCGGACACUUCCA chr7
136588019 . U A 0.000199681 + 106

hsa-mir-491
UUGACUUAGCUGGGUAGUGGGGAACCCUUCCAUGAGGAGUAGAACACUCCUUAUGCAAGAUUCCCUUC
UACCUGGCUGGGUUGG chr9 20716104 rs140761798 U C
0.000399361 + 1
hsa-mir-491
UUGACUUAGCUGGGUAGUGGGGAACCCUUCCAUGAGGAGUAGAACACUCCUUAUGCAAGAUUCCCUUC
UACCUGGCUGGGUUGG chr9 20716128 . C U 0.000798722 +

25
hsa-mir-492
CAACUACAGCCACUACUACAGGACCAUCGAGGACCUGCGGGACAAGAUUCUUGGUGCCACCAUUGAGA ACGCCAGGAUUGUCCUGCAGAUCAACAAUGCUCAACUGGCUGCAGAUG chr12 95228179 rs200816308 A C 0.0902556 + 6

hsa-mir-492
CAACUACAGCCACUACUACAGGACCAUCGAGGACCUGCGGGACAAGAUUCUUGGUGCCACCAUUGAGA ACGCCAGGAUUGUCCUGCAGAUCAACAAUGCUCAACUGGCUGCAGAUG chr12 95228209 . U C 0.000199681 + 36
hsa-mir-492
CAACUACAGCCACUACUACAGGACCAUCGAGGACCUGCGGGACAAGAUUCUUGGUGCCACCAUUGAGA ACGCCAGGAUUGUCCUGCAGAUCAACAAUGCUCAACUGGCUGCAGAUG chr12 95228286 rs2289030 G C 0.114417 + 113

hsa-mir-493
CUGGCCUCCAGGGCUUUGUACAUGGUAGGCUUUCAUUCAUUCGUUUGCACAUUCGGUGAAGGUCUACU GUGUGCCAGGCCCUGUGCCAG chr14 101335438 . C U 0.000399361 + 42
hsa-mir-493
CUGGCCUCCAGGGCUUUGUACAUGGUAGGCUUUCAUUCAUUCGUUUGCACAUUCGGUGAAGGUCUACU GUGUGCCAGGCCCUGUGCCAG chr14 101335451 . G A 0.000199681 + 55
hsa-mir-495
UGGUACCUGAAAAGAAGUUGCCCAUGUUAUUUUCGCUUUAUAUGUGACGAAACAAACAUGGUGCACUU CUUUUUCGGUAUCA chr14 101500098 rs149411304 C U 0.000399361 + 7
hsa-mir-495
UGGUACCUGAAAAGAAGUUGCCCAUGUUAUUUUCGCUUUAUAUGUGACGAAACAAACAUGGUGCACUU CUUUUUCGGUAUCA chr14 101500126 rs367575239 G A 0.000199681 + 35
hsa-mir-495
UGGUACCUGAAAAGAAGUUGCCCAUGUUAUUUUCGCUUUAUAUGUGACGAAACAAACAUGGUGCACUU CUUUUUCGGUAUCA chr14 101500133 rs144777170 A G 0.000798722 + 42
hsa-mir-495
UGGUACCUGAAAAGAAGUUGCCCAUGUUAUUUUCGCUUUAUAUGUGACGAAACAAACAUGGUGCACUU CUUUUUCGGUAUCA chr14 101500166 rs375457031 C U 0.000199681 + 75
hsa-mir-495
UGGUACCUGAAAAGAAGUUGCCCAUGUUAUUUUCGCUUUAUAUGUGACGAAACAAACAUGGUGCACUU CUUUUUCGGUAUCA chr14 101500167 . G A 0.000199681 + 76
hsa-mir-496
CCCAAGUCAGGUACUCGAAUGGAGGUUGUCCAUGGUGUGUUCAUUUUAUUUAUGAUGAGUAUUACAUG GCCAAUCUCCUUUCGGUACUCAAUUCUUCUUGGG chr14 101526951 rs142661498 C A 0.000399361 + 42

hsa-mir-496
CCCAAGUCAGGUACUCGAAUGGAGGUUGUCCAUGGUGUGUUCAUUUUAUUUAUGAUGAGUAUUACAUG GCCAAUCUCCUUUCGGUACUCAAUUCUUCUUGGG chr14 101526957 rs189230072 A C 0.000199681 + 48
hsa-mir-496
CCCAAGUCAGGUACUCGAAUGGAGGUUGUCCAUGGUGUGUUCAUUUUAUUUAUGAUGAGUAUUACAUG GCCAAUCUCCUUUCGGUACUCAAUUCUUCUUGGG chr14 101526987 . C U 0.000599042 + 78
hsa-mir-496
CCCAAGUCAGGUACUCGAAUGGAGGUUGUCCAUGGUGUGUUCAUUUUAUUUAUGAUGAGUAUUACAUG GCCAAUCUCCUUUCGGUACUCAAUUCUUCUUGGG chr14 101526991 rs369754199 C U 0.000599042 + 82
hsa-mir-497
CCACCCCGGUCCUGCUCCCGCCCCAGCAGCACACUGUGGUUUGUACGGCACUGUGGCCACGUCCAAAC CACACUGUGGUGUUAGAGCGAGGGUGGGGGAGGCACCGCCGAGG chr17 6921289 . G A 0.000199681 - 53
hsa-mir-498
AACCCUCCUUGGGAAGUGAAGCUCAGGCUGUGAUUUCAAGCCAGGGGGCGUUUUUCUAUAACUGGAUG AAAAGCACCUCCAGAGCUUGAAGCUCACAGUUUGAGAGCAAUCGUCUAAGGAAGUU chr19 54177457 . C U 0.000199681 + 7

hsa-mir-498
AACCCUCCUUGGGAAGUGAAGCUCAGGCUGUGAUUUCAAGCCAGGGGGCGUUUUUCUAUAACUGGAUG AAAAGCACCUCCAGAGCUUGAAGCUCACAGUUUGAGAGCAAUCGUCUAAGGAAGUU chr19 54177523 . G A 0.000199681 + 73

hsa-mir-498
AACCCUCCUUGGGAAGUGAAGCUCAGGCUGUGAUUUCAAGCCAGGGGGCGUUUUUCUAUAACUGGAUG AAAAGCACCUCCAGAGCUUGAAGCUCACAGUUUGAGAGCAAUCGUCUAAGGAAGUU chr19 54177532 rs189977895 G C 0.000199681 + 82

hsa-mir-498
AACCCUCCUUGGGAAGUGAAGCUCAGGCUGUGAUUUCAAGCCAGGGGGCGUUUUUCUAUAACUGGAUG AAAAGCACCUCCAGAGCUUGAAGCUCACAGUUUGAGAGCAAUCGUCUAAGGAAGUU chr19 54177552 . G A 0.000199681 + 102

hsa-mir-498
AACCCUCCUUGGGAAGUGAAGCUCAGGCUGUGAUUUCAAGCCAGGGGGCGUUUUUCUAUAACUGGAUG AAAAGCACCUCCAGAGCUUGAAGCUCACAGUUUGAGAGCAAUCGUCUAAGGAAGUU chr19 54177559 rs371414204 A G 0.000399361 + 109

hsa-mir-498
AACCCUCCUUGGGAAGUGAAGCUCAGGCUGUGAUUUCAAGCCAGGGGGCGUUUUUCUAUAACUGGAUG AAAAGCACCUCCAGAGCUUGAAGCUCACAGUUUGAGAGCAAUCGUCUAAGGAAGUU chr19 54177564 . C G 0.000199681 + 114

hsa-mir-499a

GCCCUGUCCCCUGUGCCUUGGGCGGGCGGCUGUUAAGACUUGCAGUGAUGUUUAACUCCUCUCCACGU GAACAUCACAGCAAGUCUGUGCUGCUUCCCGUCCCUACGCUGCCUGGGCAGGGU chr20 33578201 rs370237634 C U 0.000399361 + 23

hsa-mir-499a
GCCCUGUCCCCUGUGCCUUGGGCGGGCGGCUGUUAAGACUUGCAGUGAUGUUUAACUCCUCUCCACGU GAACAUCACAGCAAGUCUGUGCUGCUUCCCGUCCCUACGCUGCCUGGGCAGGGU chr20 33578202 rs140486571 G A 0.00119808 + 24

hsa-mir-499a
GCCCUGUCCCCUGUGCCUUGGGCGGGCGGCUGUUAAGACUUGCAGUGAUGUUUAACUCCUCUCCACGU GAACAUCACAGCAAGUCUGUGCUGCUUCCCGUCCCUACGCUGCCUGGGCAGGGU chr20 33578251 rs3746444 A G 0.183506 + 73

hsa-mir-499a
GCCCUGUCCCCUGUGCCUUGGGCGGGCGGCUGUUAAGACUUGCAGUGAUGUUUAACUCCUCUCCACGU GAACAUCACAGCAAGUCUGUGCUGCUUCCCGUCCCUACGCUGCCUGGGCAGGGU chr20 33578255 rs150018420 C U 0.000798722 + 77

hsa-mir-499a
GCCCUGUCCCCUGUGCCUUGGGCGGGCGGCUGUUAAGACUUGCAGUGAUGUUUAACUCCUCUCCACGU GAACAUCACAGCAAGUCUGUGCUGCUUCCCGUCCCUACGCUGCCUGGGCAGGGU chr20 33578276 rs7267163 C U 0.0537141 + 98

hsa-mir-499b
GGAAGCAGCACAGACUUGCUGUGAUGUUCACGUGGAGAGGAGUUAAACAUCACUGCAAGUCUUAACAG CCGCC chr20 33578255 rs150018420 G A 0.000798722 - 21

hsa-mir-499b
GGAAGCAGCACAGACUUGCUGUGAUGUUCACGUGGAGAGGAGUUAAACAUCACUGCAAGUCUUAACAG CCGCC chr20 33578251 rs3746444 U C 0.183506 - 25

hsa-mir-499b
GGAAGCAGCACAGACUUGCUGUGAUGUUCACGUGGAGAGGAGUUAAACAUCACUGCAAGUCUUAACAG CCGCC chr20 33578206 . C U 0.000199681 - 70

hsa-mir-499b
GGAAGCAGCACAGACUUGCUGUGAUGUUCACGUGGAGAGGAGUUAAACAUCACUGCAAGUCUUAACAG CCGCC chr20 33578205 . G A 0.000399361 - 71

hsa-mir-500a
GCUCCCCCUCUCUAAUCCUUGCUACCUGGGUGAGAGUGCUGUCUGAAUGCAAUGCACCUGGGCAAGGA UUCUGAGAGCGAGAGC chrX 49773042 . C U 0.000264901 + 4

hsa-mir-500a
GCUCCCCCUCUCUAAUCCUUGCUACCUGGGUGAGAGUGCUGUCUGAAUGCAAUGCACCUGGGCAAGGA UUCUGAGAGCGAGAGC chrX 49773087 rs191321849 G A 0.0015894

+ 49
hsa-mir-500a
GCUCCCCCUCUCUAAUCCUUGCUACCUGGGUGAGAGUGCUGUCUGAAUGCAAUGCACCUGGGCAAGGA
UUCUGAGAGCGAGAGC chrX 49773090 . A G 0.000264901 +
52
hsa-mir-500b
CCCCCUCUCUAAUCCUUGCUACCUGGGUGAGAGUGCUUUCUGAAUGCAGUGCACCCAGGCAAGGAUUC
UGCAAGGGGGA chrX 49775296 . U A 0.000264901 + 17

hsa-mir-500b
CCCCCUCUCUAAUCCUUGCUACCUGGGUGAGAGUGCUUUCUGAAUGCAGUGCACCCAGGCAAGGAUUC
UGCAAGGGGGA chrX 49775337 . G A 0.000264901 + 58

hsa-mir-500b
CCCCCUCUCUAAUCCUUGCUACCUGGGUGAGAGUGCUUUCUGAAUGCAGUGCACCCAGGCAAGGAUUC
UGCAAGGGGGA chrX 49775351 rs151318590 A G 0.010596 +
72
hsa-mir-501
GCUCUUCCUCUCUAAUCCUUUGUCCCUGGGUGAGAGUGCUUUCUGAAUGCAAUGCACCCGGGCAAGGA
UUCUGAGAGGGUGAGC chrX 49774381 rs149912461 A G 0.0116556
+ 52
hsa-mir-501
GCUCUUCCUCUCUAAUCCUUUGUCCCUGGGUGAGAGUGCUUUCUGAAUGCAAUGCACCCGGGCAAGGA
UUCUGAGAGGGUGAGC chrX 49774383 . G A 0.000264901 +
54
hsa-mir-501
GCUCUUCCUCUCUAAUCCUUUGUCCCUGGGUGAGAGUGCUUUCUGAAUGCAAUGCACCCGGGCAAGGA
UUCUGAGAGGGUGAGC chrX 49774389 rs112489955 G A 0.0116556
+ 60
hsa-mir-502
UGCUCCCCCUCUCUAAUCCUUGCUAUCUGGGUGCUAGUGCUGGCUCAAUGCAAUGCACCUGGGCAAGG
AUUCAGAGAGGGGGAGCU chrX 49779210 . C U 0.000794702 +
5
hsa-mir-502
UGCUCCCCCUCUCUAAUCCUUGCUAUCUGGGUGCUAGUGCUGGCUCAAUGCAAUGCACCUGGGCAAGG
AUUCAGAGAGGGGGAGCU chrX 49779214 . C G 0.000264901 +
9
hsa-mir-502
UGCUCCCCCUCUCUAAUCCUUGCUAUCUGGGUGCUAGUGCUGGCUCAAUGCAAUGCACCUGGGCAAGG
AUUCAGAGAGGGGGAGCU chrX 49779234 . G U 0.000264901 +
29
hsa-mir-503
UGCCCUAGCAGCGGGAACAGUUCUGCAGUGAGCGAUCGGUGCUCUGGGGUAUUGUUUCCGCUGCCAGG
GUA chrX 133680381 rs373252206 G A 0.000264901 - 48

hsa-mir-504
GCUGCUGUUGGGAGACCCUGGUCUGCACUCUAUCUGUAUUCUUACUGAAGGGAGUGCAGGGCAGGGUU

UCCCAUACAGAGGGC chrX 137749929 . C U 0.000264901 -
26
hsa-mir-505
GAUGCACCCAGUGGGGGAGCCAGGAAGUAUUGAUGUUUCUGCCAGUUUAGCGUCAACACUUGCUGGUU
UCCUCUCUGGAGCAUC chrX 139006378 . G C 0.000264901 -
13
hsa-mir-505
GAUGCACCCAGUGGGGGAGCCAGGAAGUAUUGAUGUUUCUGCCAGUUUAGCGUCAACACUUGCUGGUU
UCCUCUCUGGAGCAUC chrX 139006339 rs143213653 G A
0.000264901 - 52
hsa-mir-506
GCCACCACCAUCAGCCAUACUAUGUGUAGUGCCUUAUUCAGGAAGGUGUUACUUAAUAGAUUAAUAUU
UGUAAGGCACCCUUCUGAGUAGAGUAAUGUGCAACAUGGACAACAUUUGUGGUGGC chrX 146312254
. A G 0.000794702 - 108

hsa-mir-507
GUGCUGUGUGUAGUGCUUCACUUCAAGAAGUGCCAUGCAUGUGUCUAGAAAUAUGUUUUGCACCUUUU
GGAGUGAAAUAAUGCACAACAGAUACchrX 146312590 . G U
0.000264901 - 6
hsa-mir-507
GUGCUGUGUGUAGUGCUUCACUUCAAGAAGUGCCAUGCAUGUGUCUAGAAAUAUGUUUUGCACCUUUU
GGAGUGAAAUAAUGCACAACAGAUACchrX 146312589 . U C
0.000529801 - 7
hsa-mir-507
GUGCUGUGUGUAGUGCUUCACUUCAAGAAGUGCCAUGCAUGUGUCUAGAAAUAUGUUUUGCACCUUUU
GGAGUGAAAUAAUGCACAACAGAUACchrX 146312560 rs190684797 U C
0.000264901 - 36
hsa-mir-507
GUGCUGUGUGUAGUGCUUCACUUCAAGAAGUGCCAUGCAUGUGUCUAGAAAUAUGUUUUGCACCUUUU
GGAGUGAAAUAAUGCACAACAGAUACchrX 146312541 . G A
0.000529801 - 55
hsa-mir-507
GUGCUGUGUGUAGUGCUUCACUUCAAGAAGUGCCAUGCAUGUGUCUAGAAAUAUGUUUUGCACCUUUU
GGAGUGAAAUAAUGCACAACAGAUACchrX 146312517 . A G
0.000794702 - 79
hsa-mir-508
CCACCUUCAGCUGAGUGUAGUGCCCUACUCCAGAGGGCGUCACUCAUGUAAACUAAAACAUGAUUGUA
GCCUUUUGGAGUAGAGUAAUACACAUCACGUAACGCAUAUUUGGUGG chrX 146318507
rs201928492 G A 0.000264901 - 39

hsa-mir-508
CCACCUUCAGCUGAGUGUAGUGCCCUACUCCAGAGGGCGUCACUCAUGUAAACUAAAACAUGAUUGUA
GCCUUUUGGAGUAGAGUAAUACACAUCACGUAACGCAUAUUUGGUGG chrX 146318448 .
G A 0.000264901 - 98
hsa-mir-509-1
CAUGCUGUGUGUGGUACCCUACUGCAGACAGUGGCAAUCAUGUAUAAUUAAAAAUGAUUGGUACGUCU
GUGGGUAGAGUACUGCAUGACACAUGchrX 146342080 rs201952682 C U

0.000794702 - 64
hsa-mir-509-1
CAUGCUGUGUGUGGUACCCUACUGCAGACAGUGGCAAUCAUGUAUAAUUAAAAAUGAUUGGUACGUCU
GUGGGUAGAGUACUGCAUGACACAUGchrX 146342071 . G A
0.000264901 - 73
hsa-mir-509-1
CAUGCUGUGUGUGGUACCCUACUGCAGACAGUGGCAAUCAUGUAUAAUUAAAAAUGAUUGGUACGUCU
GUGGGUAGAGUACUGCAUGACACAUGchrX 146342052 . A G 0.0013245
- 92
hsa-mir-509-2
CAUGCUGUGUGUGGUACCCUACUGCAGACAGUGGCAAUCAUGUAUAAUUAAAAAUGAUUGGUACGUCU
GUGGGUAGAGUACUGCAUGACAC chrX 146340360 rs201600950 G U
0.0013245 - 9
hsa-mir-509-2
CAUGCUGUGUGUGGUACCCUACUGCAGACAGUGGCAAUCAUGUAUAAUUAAAAAUGAUUGGUACGUCU
GUGGGUAGAGUACUGCAUGACAC chrX 146340339 rs36092315 A -
null null 30
hsa-mir-509-2
CAUGCUGUGUGUGGUACCCUACUGCAGACAGUGGCAAUCAUGUAUAAUUAAAAAUGAUUGGUACGUCU
GUGGGUAGAGUACUGCAUGACAC chrX 146340304 . G A
0.000264901 - 65
hsa-mir-509-2
CAUGCUGUGUGUGGUACCCUACUGCAGACAGUGGCAAUCAUGUAUAAUUAAAAAUGAUUGGUACGUCU
GUGGGUAGAGUACUGCAUGACAC chrX 146340278 . C A
0.000264901 - 91
hsa-mir-509-3
GUGGUACCCUACUGCAGACGUGGCAAUCAUGUAUAAUUAAAAAUGAUUGGUACGUCUGUGGGUAGAGU
ACUGCAU chrX 146341217 rs368447666 C G 0.000794702 -
28
hsa-mir-510
GUGGUGUCCUACUCAGGAGAGUGGCAAUCACAUGUAAUUAGGUGUGAUUGAAACCUCUAAGAGUGGAG
UAACAC chrX 146353894 rs375077546 U C 0.000794702 - 33

hsa-mir-511
CAAUAGACACCCAUCGUGUCUUUUGCUCUGCAGUCAGUAAAUAUUUUUUUGUGAAUGUGUAGCAAAAG
ACAGAAUGGUGGUCCAUUG chr10 18134042 . A U 0.000599042 +
7
hsa-mir-511
CAAUAGACACCCAUCGUGUCUUUUGCUCUGCAGUCAGUAAAUAUUUUUUUGUGAAUGUGUAGCAAAAG
ACAGAAUGGUGGUCCAUUG chr10 18134045 rs371973241 C A 0.0135783
+ 10
hsa-mir-511
CAAUAGACACCCAUCGUGUCUUUUGCUCUGCAGUCAGUAAAUAUUUUUUUGUGAAUGUGUAGCAAAAG
ACAGAAUGGUGGUCCAUUG chr10 18134091 . U A 0.000199681 +
56
hsa-mir-512-1
UCUCAGUCUGUGGCACUCAGCCUUGAGGGCACUUUCUGGUGCCAGAAUGAAAGUGCUGUCAUAGCUGA

GGUCCAAUGACUGAGG chr19 54169954 . C U 0.000199681 +
22
hsa-mir-512-1
UCUCAGUCUGUGGCACUCAGCCUUGAGGGCACUUUCUGGUGCCAGAAUGAAAGUGCUGUCAUAGCUGA
GGUCCAAUGACUGAGG chr19 54169973 . G A 0.00559105 +
41
hsa-mir-512-1
UCUCAGUCUGUGGCACUCAGCCUUGAGGGCACUUUCUGGUGCCAGAAUGAAAGUGCUGUCAUAGCUGA
GGUCCAAUGACUGAGG chr19 54170006 rs181061154 A G 0.00259585
+ 74
hsa-mir-512-1
UCUCAGUCUGUGGCACUCAGCCUUGAGGGCACUUUCUGGUGCCAGAAUGAAAGUGCUGUCAUAGCUGA
GGUCCAAUGACUGAGG chr19 54170016 . G A 0.000199681 +
84
hsa-mir-512-2
GGUACUUCUCAGUCUGUGGCACUCAGCCUUGAGGGCACUUUCUGGUGCCAGAAUGAAAGUGCUGUCAU
AGCUGAGGUCCAAUGACUGAGGCGAGCACC chr19 54172483 . G A
0.000199681 + 73
hsa-mir-512-2
GGUACUUCUCAGUCUGUGGCACUCAGCCUUGAGGGCACUUUCUGGUGCCAGAAUGAAAGUGCUGUCAU
AGCUGAGGUCCAAUGACUGAGGCGAGCACC chr19 54172501 rs199602613 C U
0.000199681 + 91
hsa-mir-512-2
GGUACUUCUCAGUCUGUGGCACUCAGCCUUGAGGGCACUUUCUGGUGCCAGAAUGAAAGUGCUGUCAU
AGCUGAGGUCCAAUGACUGAGGCGAGCACC chr19 54172508 . C U
0.000399361 + 98
hsa-mir-513a-1
GGGAUGCCACAUUCAGCCAUUCAGCGUACAGUGCCUUUCACAGGGAGGUGUCAUUUAUGUGAACUAAA
AUAUAAAUUUCACCUUUCUGAGAAGGGUAAUGUACAGCAUGCACUGCAUAUGUGGUGUCCC chrX
146295085 . C U 0.000794702 - 25

hsa-mir-513a-1
GGGAUGCCACAUUCAGCCAUUCAGCGUACAGUGCCUUUCACAGGGAGGUGUCAUUUAUGUGAACUAAA
AUAUAAAUUUCACCUUUCUGAGAAGGGUAAUGUACAGCAUGCACUGCAUAUGUGGUGUCCC chrX
146295067 rs35027589 - G null null 43

hsa-mir-513a-2
GGAUGCCACAUUCAGCCAUUCAGUGUGCAGUGCCUUUCACAGGGAGGUGUCAUUUAUGUGAACUAAAA
UAUAAAUUUCACCUUUCUGAGAAGGGUAAUGUACAGCAUGCACUGCAUAUGUGGUGUCC chrX
146307449 rs111970838 A C null null 22

hsa-mir-513a-2
GGAUGCCACAUUCAGCCAUUCAGUGUGCAGUGCCUUUCACAGGGAGGUGUCAUUUAUGUGAACUAAAA
UAUAAAUUUCACCUUUCUGAGAAGGGUAAUGUACAGCAUGCACUGCAUAUGUGGUGUCC chrX
146307414 rs193181398 U C 0.0010596 - 57

hsa-mir-513a-2

GGAUGCCACAUUCAGCCAUUCAGUGUGCAGUGCCUUUCACAGGGAGGUGUCAUUUAUGUGAACUAAAA UAUAAAUUUCACCUUUCUGAGAAGGGUAAUGUACAGCAUGCACUGCAUAUGUGGUGUCC chrX 146307411 rs372214863 G A 0.0010596 - 60

hsa-mir-513a-2
GGAUGCCACAUUCAGCCAUUCAGUGUGCAGUGCCUUUCACAGGGAGGUGUCAUUUAUGUGAACUAAAA UAUAAAUUUCACCUUUCUGAGAAGGGUAAUGUACAGCAUGCACUGCAUAUGUGGUGUCC chrX 146307375 rs376408446 A G 0.000264901 - 96

hsa-mir-513b
GUGUACAGUGCCUUUCACAAGGAGGUGUCAUUUAUGUGAACUAAAAUAUAAAUGUCACCUUUUUGAGA GGAGUAAUGUACAGCA chrX 146280644 rs138509628 U G 0.00344371 - 2

hsa-mir-513b
GUGUACAGUGCCUUUCACAAGGAGGUGUCAUUUAUGUGAACUAAAAUAUAAAUGUCACCUUUUUGAGA GGAGUAAUGUACAGCA chrX 146280626 rs370968962 A G 0.000264901 - 20

hsa-mir-513c
GCGUACAGUGCCUUUCUCAAGGAGGUGUCGUUUAUGUGAACUAAAAUAUAAAUUUCACCUUUCUGAGA AGAGUAAUGUACAGCA chrX 146271304 rs376895882 C U 0.000264901 - 2

hsa-mir-513c
GCGUACAGUGCCUUUCUCAAGGAGGUGUCGUUUAUGUGAACUAAAAUAUAAAUUUCACCUUUCUGAGA AGAGUAAUGUACAGCA chrX 146271303 rs145416750 G A 0.00476821 - 3

hsa-mir-513c
GCGUACAGUGCCUUUCUCAAGGAGGUGUCGUUUAUGUGAACUAAAAUAUAAAUUUCACCUUUCUGAGA AGAGUAAUGUACAGCA chrX 146271269 . U G 0.000264901 - 37

hsa-mir-513c
GCGUACAGUGCCUUUCUCAAGGAGGUGUCGUUUAUGUGAACUAAAAUAUAAAUUUCACCUUUCUGAGA AGAGUAAUGUACAGCA chrX 146271266 rs184216167 A G 0.000264901 - 40

hsa-mir-513c
GCGUACAGUGCCUUUCUCAAGGAGGUGUCGUUUAUGUGAACUAAAAUAUAAAUUUCACCUUUCUGAGA AGAGUAAUGUACAGCA chrX 146271235 . A G 0.000264901 - 71

hsa-mir-513c
GCGUACAGUGCCUUUCUCAAGGAGGUGUCGUUUAUGUGAACUAAAAUAUAAAUUUCACCUUUCUGAGA AGAGUAAUGUACAGCA chrX 146271231 rs193119939 A G 0.000264901 - 75

hsa-mir-514a-1
AACAUGUUGUCUGUGGUACCCUACUCUGGAGAGUGACAAUCAUGUAUAAUUAAAUUUGAUUGACACUU CUGUGAGUAGAGUAACGCAUGACACGUACG chrX 146360845 . A G 0.000529801 - 18

hsa-mir-514a-1
AACAUGUUGUCUGUGGUACCCUACUCUGGAGAGUGACAAUCAUGUAUAAUUAAAUUUGAUUGACACUU

CUGUGAGUAGAGUAACGCAUGACACGUACG chrX 146360826 rs183412647 C U
0.00238411 - 37
hsa-mir-514a-1
AACAUGUUGUCUGUGGUACCCUACUCUGGAGAGUGACAAUCAUGUAUAAUUAAAUUUGAUUGACACUU
CUGUGAGUAGAGUAACGCAUGACACGUACG chrX 146360813 rs148964961 U C
0.00688742 - 50
hsa-mir-514a-1
AACAUGUUGUCUGUGGUACCCUACUCUGGAGAGUGACAAUCAUGUAUAAUUAAAUUUGAUUGACACUU
CUGUGAGUAGAGUAACGCAUGACACGUACG chrX 146360779 rs371147586 C U
0.000794702 - 84
hsa-mir-514a-1
AACAUGUUGUCUGUGGUACCCUACUCUGGAGAGUGACAAUCAUGUAUAAUUAAAUUUGAUUGACACUU
CUGUGAGUAGAGUAACGCAUGACACGUACG chrX 146360778 rs192021879 G A
0.000264901 - 85
hsa-mir-514a-1
AACAUGUUGUCUGUGGUACCCUACUCUGGAGAGUGACAAUCAUGUAUAAUUAAAUUUGAUUGACACUU
CUGUGAGUAGAGUAACGCAUGACACGUACG chrX 146360765 . G A
0.000264901 - 98
hsa-mir-514a-3
GUUGUCUGUGGUACCCUACUCUGGAGAGUGACAAUCAUGUAUAACUAAAUUUGAUUGACACUUCUGUG
AGUAGAGUAACGCAUGACAC chrX 146366167 . G A
0.000264901 - 80
hsa-mir-515-1
UCUCAUGCAGUCAUUCUCCAAAAGAAAGCACUUUCUGUUGUCUGAAAGCAGAGUGCCUUCUUUUGGAG
CGUUACUGUUUGAGA chr19 54182261 rs188152320 A C
0.000599042 + 5
hsa-mir-515-1
UCUCAUGCAGUCAUUCUCCAAAAGAAAGCACUUUCUGUUGUCUGAAAGCAGAGUGCCUUCUUUUGGAG
CGUUACUGUUUGAGA chr19 54182325 rs200825099 C U
0.000399361 + 69
hsa-mir-515-1
UCUCAUGCAGUCAUUCUCCAAAAGAAAGCACUUUCUGUUGUCUGAAAGCAGAGUGCCUUCUUUUGGAG
CGUUACUGUUUGAGA chr19 54182326 rs374576826 G A
0.000199681 + 70
hsa-mir-516a-1
UCUCAGGCUGUGACCUUCUCGAGGAAAGAAGCACUUUCUGUUGUCUGAAAGAAAAGAAAGUGCUUCCU
UUCAGAGGGUUACGGUUUGAGA chr19 54260002 rs2569389 C U
0.000199681 + 8
hsa-mir-516a-1
UCUCAGGCUGUGACCUUCUCGAGGAAAGAAGCACUUUCUGUUGUCUGAAAGAAAAGAAAGUGCUUCCU
UUCAGAGGGUUACGGUUUGAGA chr19 54260009 rs374231641 C U
0.000399361 + 15
hsa-mir-516a-1
UCUCAGGCUGUGACCUUCUCGAGGAAAGAAGCACUUUCUGUUGUCUGAAAGAAAAGAAAGUGCUUCCU
UUCAGAGGGUUACGGUUUGAGA chr19 54260068 . A G
0.000199681 + 74
hsa-mir-516a-1

UCUCAGGCUGUGACCUUCUCGAGGAAAGAAGCACUUUCUGUUGUCUGAAAGAAAAGAAAGUGCUUCCU
UUCAGAGGGUUACGGUUUGAGA chr19 54260075 . C U
0.000199681 + 81
hsa-mir-516a-2
UCUCAGGUUGUGACCUUCUCGAGGAAAGAAGCACUUUCUGUUGUCUGAAAGAAAAGAAAGUGCUUCCU
UUCAGAGGGUUACGGUUUGAGA chr19 54264394 rs115975528 U C
0.0109824 + 8
hsa-mir-516a-2
UCUCAGGUUGUGACCUUCUCGAGGAAAGAAGCACUUUCUGUUGUCUGAAAGAAAAGAAAGUGCUUCCU
UUCAGAGGGUUACGGUUUGAGA chr19 54264421 rs368499496 U C
0.000399361 + 35
hsa-mir-516a-2
UCUCAGGUUGUGACCUUCUCGAGGAAAGAAGCACUUUCUGUUGUCUGAAAGAAAAGAAAGUGCUUCCU
UUCAGAGGGUUACGGUUUGAGA chr19 54264461 . G U
0.000199681 + 75
hsa-mir-516a-2
UCUCAGGUUGUGACCUUCUCGAGGAAAGAAGCACUUUCUGUUGUCUGAAAGAAAAGAAAGUGCUUCCU
UUCAGAGGGUUACGGUUUGAGA chr19 54264462 . G U
0.000199681 + 76
hsa-mir-516a-2
UCUCAGGUUGUGACCUUCUCGAGGAAAGAAGCACUUUCUGUUGUCUGAAAGAAAAGAAAGUGCUUCCU
UUCAGAGGGUUACGGUUUGAGA chr19 54264468 rs191772135 G U
0.00419329 + 82
hsa-mir-516b-1
UCUCAGGCUGUGACCAUCUGGAGGUAAGAAGCACUUUCUGUUUUGUGAAAGAAAAGAAAGUGCUUCCU
UUCAGAGGGUUACUCUUUGAGA chr19 54240136 rs201582431 C G
0.0215655 + 38
hsa-mir-516b-1
UCUCAGGCUGUGACCAUCUGGAGGUAAGAAGCACUUUCUGUUUUGUGAAAGAAAAGAAAGUGCUUCCU
UUCAGAGGGUUACUCUUUGAGA chr19 54240137 rs372618870 U C
0.00119808 + 39
hsa-mir-516b-1
UCUCAGGCUGUGACCAUCUGGAGGUAAGAAGCACUUUCUGUUUUGUGAAAGAAAAGAAAGUGCUUCCU
UUCAGAGGGUUACUCUUUGAGA chr19 54240142 rs183522542 U C
0.000399361 + 44
hsa-mir-516b-1
UCUCAGGCUGUGACCAUCUGGAGGUAAGAAGCACUUUCUGUUUUGUGAAAGAAAAGAAAGUGCUUCCU
UUCAGAGGGUUACUCUUUGAGA chr19 54240174 rs78861479 G A
0.014377 + 76
hsa-mir-516b-1
UCUCAGGCUGUGACCAUCUGGAGGUAAGAAGCACUUUCUGUUUUGUGAAAGAAAAGAAAGUGCUUCCU
UUCAGAGGGUUACUCUUUGAGA chr19 54240184 rs151073675 U A
0.00738818 + 86
hsa-mir-516b-2
UCUCAUGAUGUGACCAUCUGGAGGUAAGAAGCACUUUGUGUUUUGUGAAAGAAAGUGCUUCCUUUCAG
AGGGUUACUCUUUGAGA chr19 54228719 . G A 0.000199681 +
24

hsa-mir-516b-2
UCUCAUGAUGUGACCAUCUGGAGGUAAGAAGCACUUUGUGUUUUGUGAAAGAAAGUGCUUCCUUUCAG
AGGGUUACUCUUUGAGA chr19 54228742 rs10670323 - AAAGA null
null 47
hsa-mir-516b-2
UCUCAUGAUGUGACCAUCUGGAGGUAAGAAGCACUUUGUGUUUUGUGAAAGAAAGUGCUUCCUUUCAG
AGGGUUACUCUUUGAGA chr19 54228743 rs33953969 - AAAGA null
null 48
hsa-mir-516b-2
UCUCAUGAUGUGACCAUCUGGAGGUAAGAAGCACUUUGUGUUUUGUGAAAGAAAGUGCUUCCUUUCAG
AGGGUUACUCUUUGAGA chr19 54228750 . G A 0.000199681 +
55
hsa-mir-516b-2
UCUCAUGAUGUGACCAUCUGGAGGUAAGAAGCACUUUGUGUUUUGUGAAAGAAAGUGCUUCCUUUCAG
AGGGUUACUCUUUGAGA chr19 54228774 rs10583889 UU - null
null 79
hsa-mir-516b-2
UCUCAUGAUGUGACCAUCUGGAGGUAAGAAGCACUUUGUGUUUUGUGAAAGAAAGUGCUUCCUUUCAG
AGGGUUACUCUUUGAGA chr19 54228775 rs114381285 U C null
null 80
hsa-mir-517a
UCUCAGGCAGUGACCCUCUAGAUGGAAGCACUGUCUGUUGUAUAAAAGAAAAGAUCGUGCAUCCCUUU
AGAGUGUUACUGUUUGAGA chr19 54215584 rs188936188 C A
0.000199681 + 63
hsa-mir-517a
UCUCAGGCAGUGACCCUCUAGAUGGAAGCACUGUCUGUUGUAUAAAAGAAAAGAUCGUGCAUCCCUUU
AGAGUGUUACUGUUUGAGA chr19 54215608 rs377017275 A G
0.000399361 + 87
hsa-mir-517b
GUGACCCUCUAGAUGGAAGCACUGUCUGUUGUCUAAGAAAAGAUCGUGCAUCCCUUUAGAGUGUUAC
chr19 54224382 . C G 0.000199681 + 53

hsa-mir-517c
GAAGAUCUCAGGCAGUGACCCUCUAGAUGGAAGCACUGUCUGUUGUCUAAGAAAAGAUCGUGCAUCCU
UUUAGAGUGUUACUGUUUGAGAAAAUC chr19 54244647 rs182664281 C U
0.000399361 + 81
hsa-mir-518a-1
UCUCAAGCUGUGACUGCAAAGGGAAGCCCUUUCUGUUGUCUGAAAGAAGAGAAAGCGCUUCCCUUUGC
UGGAUUACGGUUUGAGA chr19 54234260 rs141873214 U C 0.00239617
+ 1
hsa-mir-518a-1
UCUCAAGCUGUGACUGCAAAGGGAAGCCCUUUCUGUUGUCUGAAAGAAGAGAAAGCGCUUCCCUUUGC
UGGAUUACGGUUUGAGA chr19 54234265 rs190044154 A U
0.000998403 + 6
hsa-mir-518a-1
UCUCAAGCUGUGACUGCAAAGGGAAGCCCUUUCUGUUGUCUGAAAGAAGAGAAAGCGCUUCCCUUUGC
UGGAUUACGGUUUGAGA chr19 54234315 rs368403519 C G

0.000199681 + 56
hsa-mir-518a-1
UCUCAAGCUGUGACUGCAAAGGGAAGCCCUUUCUGUUGUCUGAAAGAAGAGAAAGCGCUUCCCUUUGC
UGGAUUACGGUUUGAGA chr19 54234340 rs79646188 U G null
null 81
hsa-mir-518a-2
UCUCAAGCUGUGGGUCUGCAAAGGGAAGCCCUUUCUGUUGUCUAAAAGAAGAGAAAGCGCUUCCCUUU
GCUGGAUUACGGUUUGAGA chr19 54242630 rs188224089 A G
0.000399361 + 44
hsa-mir-518b
UCAUGCUGUGGCCCUCCAGAGGGAAGCGCUUUCUGUUGUCUGAAAGAAAACAAAGCGCUCCCCUUUAG
AGGUUUACGGUUUGA chr19 54206051 . C U 0.000199681 +
61
hsa-mir-518c
GCGAGAAGAUCUCAUGCUGUGACUCUCUGGAGGGAAGCACUUUCUGUUGUCUGAAAGAAAACAAAGCG
CUUCUCUUUAGAGUGUUACGGUUUGAGAAAAGC chr19 54211990 . C U
0.000399361 + 2
hsa-mir-518c
GCGAGAAGAUCUCAUGCUGUGACUCUCUGGAGGGAAGCACUUUCUGUUGUCUGAAAGAAAACAAAGCG
CUUCUCUUUAGAGUGUUACGGUUUGAGAAAAGC chr19 54212075 . C U
0.000199681 + 87
hsa-mir-518d
UCCCAUGCUGUGACCCUCUAGAGGGAAGCACUUUCUGUUGUCUGAAAGAAACCAAAGCGCUUCCCUUU
GGAGCGUUACGGUUUGAGA chr19 54238159 . C U 0.000199681 +
29
hsa-mir-518d
UCCCAUGCUGUGACCCUCUAGAGGGAAGCACUUUCUGUUGUCUGAAAGAAACCAAAGCGCUUCCCUUU
GGAGCGUUACGGUUUGAGA chr19 54238182 rs116220629 C U 0.0131789
+ 52
hsa-mir-518d
UCCCAUGCUGUGACCCUCUAGAGGGAAGCACUUUCUGUUGUCUGAAAGAAACCAAAGCGCUUCCCUUU
GGAGCGUUACGGUUUGAGA chr19 54238189 rs73602910 G A 0.00299521
+ 59
hsa-mir-518d
UCCCAUGCUGUGACCCUCUAGAGGGAAGCACUUUCUGUUGUCUGAAAGAAACCAAAGCGCUUCCCUUU
GGAGCGUUACGGUUUGAGA chr19 54238203 rs151129856 C U
0.000399361 + 73
hsa-mir-518d
UCCCAUGCUGUGACCCUCUAGAGGGAAGCACUUUCUGUUGUCUGAAAGAAACCAAAGCGCUUCCCUUU
GGAGCGUUACGGUUUGAGA chr19 54238208 rs74704964 C U 0.0147764
+ 78
hsa-mir-518e
UCUCAGGCUGUGACCCUCUAGAGGGAAGCGCUUUCUGUUGGCUAAAAGAAAAGAAAGCGCUUCCCUUC
AGAGUGUUAACGCUUUGAGA chr19 54233092 . U G
0.000199681 + 1
hsa-mir-518e
UCUCAGGCUGUGACCCUCUAGAGGGAAGCGCUUUCUGUUGGCUAAAAGAAAAGAAAGCGCUUCCCUUC

AGAGUGUUAACGCUUUGAGA chr19 54233093 . C G
0.000199681 + 2
hsa-mir-518e
UCUCAGGCUGUGACCCUCUAGAGGGAAGCGCUUUCUGUUGGCUAAAAGAAAAGAAAGCGCUUCCCUUC
AGAGUGUUAACGCUUUGAGA chr19 54233109 rs74177813 C -
null null 18
hsa-mir-518e
UCUCAGGCUGUGACCCUCUAGAGGGAAGCGCUUUCUGUUGGCUAAAAGAAAAGAAAGCGCUUCCCUUC
AGAGUGUUAACGCUUUGAGA chr19 54233112 rs34416818 - A
null null 21
hsa-mir-518e
UCUCAGGCUGUGACCCUCUAGAGGGAAGCGCUUUCUGUUGGCUAAAAGAAAAGAAAGCGCUUCCCUUC
AGAGUGUUAACGCUUUGAGA chr19 54233113 rs74177814 - G
null null 22
hsa-mir-518e
UCUCAGGCUGUGACCCUCUAGAGGGAAGCGCUUUCUGUUGGCUAAAAGAAAAGAAAGCGCUUCCCUUC
AGAGUGUUAACGCUUUGAGA chr19 54233170 . C U
0.000199681 + 79
hsa-mir-518f
UCUCAUGCUGUGACCCUCUAGAGGGAAGCACUUUCUCUUGUCUAAAAGAAAAGAAAGCGCUUCUCUUU
AGAGGAUUACUCUUUGAGA chr19 54203326 . C U 0.000199681 +
58
hsa-mir-518f
UCUCAUGCUGUGACCCUCUAGAGGGAAGCACUUUCUCUUGUCUAAAAGAAAAGAAAGCGCUUCUCUUU
AGAGGAUUACUCUUUGAGA chr19 54203333 . C G 0.000199681 +
65
hsa-mir-518f
UCUCAUGCUGUGACCCUCUAGAGGGAAGCACUUUCUCUUGUCUAAAAGAAAAGAAAGCGCUUCUCUUU
AGAGGAUUACUCUUUGAGA chr19 54203347 . U A 0.000199681 +
79
hsa-mir-519a-1
CUCAGGCUGUGACACUCUAGAGGGAAGCGCUUUCUGUUGUCUGAAAGAAAGGAAAGUGCAUCCUUUUA
GAGUGUUACUGUUUGAG chr19 54255679 rs191444009 G A
0.000199681 + 29
hsa-mir-519a-1
CUCAGGCUGUGACACUCUAGAGGGAAGCGCUUUCUGUUGUCUGAAAGAAAGGAAAGUGCAUCCUUUUA
GAGUGUUACUGUUUGAG chr19 54255689 . G U 0.000199681 +
39
hsa-mir-519a-2
UCUCAGGCUGUGUCCCUCUACAGGGAAGCGCUUUCUGUUGUCUGAAAGAAAGGAAAGUGCAUCCUUUU
AGAGUGUUACUGUUUGAGA chr19 54265617 rs137963341 A G
0.000199681 + 20
hsa-mir-519a-2
UCUCAGGCUGUGUCCCUCUACAGGGAAGCGCUUUCUGUUGUCUGAAAGAAAGGAAAGUGCAUCCUUUU
AGAGUGUUACUGUUUGAGA chr19 54265626 rs142071806 C U
0.000399361 + 29
hsa-mir-519a-2

UCUCAGGCUGUGUCCCUCUACAGGGAAGCGCUUUCUGUUGUCUGAAAGAAAGGAAAGUGCAUCCUUUU AGAGUGUUACUGUUUGAGA chr19 54265658 . A U 0.000199681 + 61

hsa-mir-519a-2
UCUCAGGCUGUGUCCCUCUACAGGGAAGCGCUUUCUGUUGUCUGAAAGAAAGGAAAGUGCAUCCUUUU AGAGUGUUACUGUUUGAGA chr19 54265670 . U G 0.000199681 + 73

hsa-mir-519b
CAUGCUGUGACCCUCUAGAGGGAAGCGCUUUCUGUUGUCUGAAAGAAAAGAAAGUGCAUCCUUUUAGA GGUUUACUGUUUGchr19 54198496 rs371426753 U G 0.000399361 + 30

hsa-mir-519b
CAUGCUGUGACCCUCUAGAGGGAAGCGCUUUCUGUUGUCUGAAAGAAAAGAAAGUGCAUCCUUUUAGA GGUUUACUGUUUGchr19 54198499 . U G 0.000599042 + 33

hsa-mir-519c
UCUCAGCCUGUGACCCUCUAGAGGGAAGCGCUUUCUGUUGUCUGAAAGAAAAGAAAGUGCAUCUUUUU AGAGGAUUACAGUUUGAGA chr19 54189751 rs371236520 C U 0.000998403 + 29

hsa-mir-519c
UCUCAGCCUGUGACCCUCUAGAGGGAAGCGCUUUCUGUUGUCUGAAAGAAAAGAAAGUGCAUCUUUUU AGAGGAUUACAGUUUGAGA chr19 54189752 rs373843431 G A 0.000199681 + 30

hsa-mir-519d
UCCCAUGCUGUGACCCUCCAAAGGGAAGCGCUUUCUGUUUGUUUUCUCUUAAACAAAGUGCCUCCCUU UAGAGUGUUACCGUUUGGGA chr19 54216615 . C A 0.000199681 + 15

hsa-mir-519d
UCCCAUGCUGUGACCCUCCAAAGGGAAGCGCUUUCUGUUUGUUUUCUCUUAAACAAAGUGCCUCCCUU UAGAGUGUUACCGUUUGGGA chr19 54216616 rs376138952 C U 0.000199681 + 16

hsa-mir-519d
UCCCAUGCUGUGACCCUCCAAAGGGAAGCGCUUUCUGUUUGUUUUCUCUUAAACAAAGUGCCUCCCUU UAGAGUGUUACCGUUUGGGA chr19 54216629 . C U 0.000199681 + 29

hsa-mir-519d
UCCCAUGCUGUGACCCUCCAAAGGGAAGCGCUUUCUGUUUGUUUUCUCUUAAACAAAGUGCCUCCCUU UAGAGUGUUACCGUUUGGGA chr19 54216650 . U A 0.000599042 + 50

hsa-mir-519d
UCCCAUGCUGUGACCCUCCAAAGGGAAGCGCUUUCUGUUUGUUUUCUCUUAAACAAAGUGCCUCCCUU UAGAGUGUUACCGUUUGGGA chr19 54216670 rs116796400 A G 0.00559105 + 70

hsa-mir-519d
UCCCAUGCUGUGACCCUCCAAAGGGAAGCGCUUUCUGUUUGUUUUCUCUUAAACAAAGUGCCUCCCUU UAGAGUGUUACCGUUUGGGA chr19 54216681 rs374419233 G A 0.000399361 + 81

hsa-mir-520a
CUCAGGCUGUGACCCUCCAGAGGGAAGUACUUUCUGUUGUCUGAGAGAAAAGAAAGUGCUUCCCUUUG
GACUGUUUCGGUUUGAG chr19 54194135 . C A 0.000399361 +
1
hsa-mir-520a
CUCAGGCUGUGACCCUCCAGAGGGAAGUACUUUCUGUUGUCUGAGAGAAAAGAAAGUGCUUCCCUUUG
GACUGUUUCGGUUUGAG chr19 54194212 . G A 0.000199681 +
78
hsa-mir-520b CCCUCUACAGGGAAGCGCUUUCUGUUGUCUGAAAGAAAAGAAAGUGCUUCCUUUUAGAGGG
chr19 54204489 rs150048439 A G 0.00898562 + 9

hsa-mir-520b CCCUCUACAGGGAAGCGCUUUCUGUUGUCUGAAAGAAAAGAAAGUGCUUCCUUUUAGAGGG
chr19 54204496 rs147743520 C U 0.000399361 + 16

hsa-mir-520b CCCUCUACAGGGAAGCGCUUUCUGUUGUCUGAAAGAAAAGAAAGUGCUUCCUUUUAGAGGG
chr19 54204497 rs199890847 G A 0.000199681 + 17

hsa-mir-520c
UCUCAGGCUGUCGUCCUCUAGAGGGAAGCACUUUCUGUUGUCUGAAAGAAAAGAAAGUGCUUCCUUUU
AGAGGGUUACCGUUUGAGA chr19 54210734 rs7255628 G C null
null 28
hsa-mir-520c
UCUCAGGCUGUCGUCCUCUAGAGGGAAGCACUUUCUGUUGUCUGAAAGAAAAGAAAGUGCUUCCUUUU
AGAGGGUUACCGUUUGAGA chr19 54210736 rs151188894 A G 0.00139776
+ 30
hsa-mir-520c
UCUCAGGCUGUCGUCCUCUAGAGGGAAGCACUUUCUGUUGUCUGAAAGAAAAGAAAGUGCUUCCUUUU
AGAGGGUUACCGUUUGAGA chr19 54210774 . U G 0.000199681 +
68
hsa-mir-520d
UCUCAAGCUGUGAGUCUACAAAGGGAAGCCCUUUCUGUUGUCUAAAAGAAAAGAAAGUGCUUCUCUUU
GGUGGGUUACGGUUUGAGA chr19 54223379 . C G 0.000199681 +
30
hsa-mir-520d
UCUCAAGCUGUGAGUCUACAAAGGGAAGCCCUUUCUGUUGUCUAAAAGAAAAGAAAGUGCUUCUCUUU
GGUGGGUUACGGUUUGAGA chr19 54223433 . G A 0.000199681 +
84
hsa-mir-520e
UCUCCUGCUGUGACCCUCAAGAUGGAAGCAGUUUCUGUUGUCUGAAAGGAAAGAAAGUGCUUCCUUUU
UGAGGGUUACUGUUUGAGA chr19 54178990 . A C 0.000199681 +
26
hsa-mir-520e
UCUCCUGCUGUGACCCUCAAGAUGGAAGCAGUUUCUGUUGUCUGAAAGGAAAGAAAGUGCUUCCUUUU
UGAGGGUUACUGUUUGAGA chr19 54179024 . C U 0.000199681 +
60
hsa-mir-520e
UCUCCUGCUGUGACCCUCAAGAUGGAAGCAGUUUCUGUUGUCUGAAAGGAAAGAAAGUGCUUCCUUUU

UGAGGGUUACUGUUUGAGA chr19 54179044 . G C 0.000199681 +
80
hsa-mir-520f
UCUCAGGCUGUGACCCUCUAAAGGGAAGCGCUUUCUGUGGUCAGAAAGAAAAGCAAGUGCUUCCUUUU
AGAGGGUUACCGUUUGGGA chr19 54185441 rs373425669 C U 0.00559105
+ 29
hsa-mir-520f
UCUCAGGCUGUGACCCUCUAAAGGGAAGCGCUUUCUGUGGUCAGAAAGAAAAGCAAGUGCUUCCUUUU
AGAGGGUUACCGUUUGGGA chr19 54185457 rs184037684 A G
0.000199681 + 45
hsa-mir-520f
UCUCAGGCUGUGACCCUCUAAAGGGAAGCGCUUUCUGUGGUCAGAAAGAAAAGCAAGUGCUUCCUUUU
AGAGGGUUACCGUUUGGGA chr19 54185481 . A G 0.000399361 +
69
hsa-mir-520f
UCUCAGGCUGUGACCCUCUAAAGGGAAGCGCUUUCUGUGGUCAGAAAGAAAAGCAAGUGCUUCCUUUU
AGAGGGUUACCGUUUGGGA chr19 54185492 rs75598818 G A 0.048123
+ 80
hsa-mir-520g
UCCCAUGCUGUGACCCUCUAGAGGAAGCACUUUCUGUUUGUUGUCUGAGAAAAAACAAAGUGCUUCCC
UUUAGAGUGUUACCGUUUGGGA chr19 54225426 . G A
0.000199681 + 7
hsa-mir-520g
UCCCAUGCUGUGACCCUCUAGAGGAAGCACUUUCUGUUUGUUGUCUGAGAAAAAACAAAGUGCUUCCC
UUUAGAGUGUUACCGUUUGGGA chr19 54225437 rs182126774 C G
0.000199681 + 18
hsa-mir-520g
UCCCAUGCUGUGACCCUCUAGAGGAAGCACUUUCUGUUUGUUGUCUGAGAAAAAACAAAGUGCUUCCC
UUUAGAGUGUUACCGUUUGGGA chr19 54225460 rs375062679 U C
0.000399361 + 41
hsa-mir-520g
UCCCAUGCUGUGACCCUCUAGAGGAAGCACUUUCUGUUUGUUGUCUGAGAAAAAACAAAGUGCUUCCC
UUUAGAGUGUUACCGUUUGGGA chr19 54225463 rs186377499 U C
0.000199681 + 44
hsa-mir-520g
UCCCAUGCUGUGACCCUCUAGAGGAAGCACUUUCUGUUUGUUGUCUGAGAAAAAACAAAGUGCUUCCC
UUUAGAGUGUUACCGUUUGGGA chr19 54225490 . U A
0.000399361 + 71
hsa-mir-520g
UCCCAUGCUGUGACCCUCUAGAGGAAGCACUUUCUGUUUGUUGUCUGAGAAAAAACAAAGUGCUUCCC
UUUAGAGUGUUACCGUUUGGGA chr19 54225501 rs112328520 C U
null null 82
hsa-mir-520h
UCCCAUGCUGUGACCCUCUAGAGGAAGCACUUUCUGUUUGUUGUCUGAGAAAAAACAAAGUGCUUCCC
UUUAGAGUUACUGUUUGGGA chr19 54245768 rs148716001 C G
0.0081869 + 3
hsa-mir-520h

UCCCAUGCUGUGACCCUCUAGAGGAAGCACUUUCUGUUUGUUGUCUGAGAAAAAACAAAGUGCUUCCC
UUUAGAGUUACUGUUUGGGA chr19 54245780 . C U
0.000399361 + 15
hsa-mir-520h
UCCCAUGCUGUGACCCUCUAGAGGAAGCACUUUCUGUUUGUUGUCUGAGAAAAAACAAAGUGCUUCCC
UUUAGAGUUACUGUUUGGGA chr19 54245788 rs56013413 G A
0.0856629 + 23
hsa-mir-520h
UCCCAUGCUGUGACCCUCUAGAGGAAGCACUUUCUGUUUGUUGUCUGAGAAAAAACAAAGUGCUUCCC
UUUAGAGUUACUGUUUGGGA chr19 54245789 . G A
0.000199681 + 24
hsa-mir-520h
UCCCAUGCUGUGACCCUCUAGAGGAAGCACUUUCUGUUUGUUGUCUGAGAAAAAACAAAGUGCUUCCC
UUUAGAGUUACUGUUUGGGA chr19 54245827 . G U
0.000199681 + 62
hsa-mir-520h
UCCCAUGCUGUGACCCUCUAGAGGAAGCACUUUCUGUUUGUUGUCUGAGAAAAAACAAAGUGCUUCCC
UUUAGAGUUACUGUUUGGGA chr19 54245841 rs111745142 - GU
null null 76
hsa-mir-521-1
UCUCAGGCUGUGACCCUCCAAAGGGAAGAACUUUCUGUUGUCUAAAAGAAAAGAACGCACUUCCCUUU
AGAGUGUUACCGUGUGAGA chr19 54251902 rs371000653 A G
0.000199681 + 13
hsa-mir-521-1
UCUCAGGCUGUGACCCUCCAAAGGGAAGAACUUUCUGUUGUCUAAAAGAAAAGAACGCACUUCCCUUU
AGAGUGUUACCGUGUGAGA chr19 54251937 rs2561251 G A null
null 48
hsa-mir-521-2
UCUCGGGCUGUGACUCUCCAAAGGGAAGAAUUUUCUCUUGUCUAAAAGAAAAGAACGCACUUCCCUUU
AGAGUGUUACCGUGUGAGA chr19 54219854 rs13382089 G U 0.00479233
\+ 7
hsa-mir-521-2
UCUCGGGCUGUGACUCUCCAAAGGGAAGAAUUUUCUCUUGUCUAAAAGAAAAGAACGCACUUCCCUUU
AGAGUGUUACCGUGUGAGA chr19 54219857 . G A 0.000199681 +
10
hsa-mir-521-2
UCUCGGGCUGUGACUCUCCAAAGGGAAGAAUUUUCUCUUGUCUAAAAGAAAAGAACGCACUUCCCUUU
AGAGUGUUACCGUGUGAGA chr19 54219870 rs200197506 G A
0.000199681 + 23
hsa-mir-521-2
UCUCGGGCUGUGACUCUCCAAAGGGAAGAAUUUUCUCUUGUCUAAAAGAAAAGAACGCACUUCCCUUU
AGAGUGUUACCGUGUGAGA chr19 54219904 rs372430140 G A
0.000399361 + 57
hsa-mir-521-2
UCUCGGGCUGUGACUCUCCAAAGGGAAGAAUUUUCUCUUGUCUAAAAGAAAAGAACGCACUUCCCUUU
AGAGUGUUACCGUGUGAGA chr19 54219912 . C U 0.000199681 +
65

hsa-mir-522
UCUCAGGCUGUGUCCCUCUAGAGGGAAGCGCUUUCUGUUGUCUGAAAGAAAAGAAAAUGGUUCCCUUU
AGAGUGUUACGCUUUGAGA chr19 54254494 rs146976572 G A
0.000199681 + 30
hsa-mir-522
UCUCAGGCUGUGUCCCUCUAGAGGGAAGCGCUUUCUGUUGUCUGAAAGAAAAGAAAAUGGUUCCCUUU
AGAGUGUUACGCUUUGAGA chr19 54254543 . G A 0.000199681 +
79
hsa-mir-523
UCUCAUGCUGUGACCCUCUAGAGGGAAGCGCUUUCUGUUGUCUGAAAGAAAAGAACGCGCUUCCCUAU
AGAGGGUUACCCUUUGAGA chr19 54201654 . C U 0.000199681 +
16
hsa-mir-523
UCUCAUGCUGUGACCCUCUAGAGGGAAGCGCUUUCUGUUGUCUGAAAGAAAAGAACGCGCUUCCCUAU
AGAGGGUUACCCUUUGAGA chr19 54201667 rs374242315 C U
0.000399361 + 29
hsa-mir-523
UCUCAUGCUGUGACCCUCUAGAGGGAAGCGCUUUCUGUUGUCUGAAAGAAAAGAACGCGCUUCCCUAU
AGAGGGUUACCCUUUGAGA chr19 54201668 rs112495387 G A null
null 30
hsa-mir-523
UCUCAUGCUGUGACCCUCUAGAGGGAAGCGCUUUCUGUUGUCUGAAAGAAAAGAACGCGCUUCCCUAU
AGAGGGUUACCCUUUGAGA chr19 54201692 rs191624794 A C
0.000399361 + 54
hsa-mir-523
UCUCAUGCUGUGACCCUCUAGAGGGAAGCGCUUUCUGUUGUCUGAAAGAAAAGAACGCGCUUCCCUAU
AGAGGGUUACCCUUUGAGA chr19 54201695 rs201232958 G A
0.000399361 + 57
hsa-mir-523
UCUCAUGCUGUGACCCUCUAGAGGGAAGCGCUUUCUGUUGUCUGAAAGAAAAGAACGCGCUUCCCUAU
AGAGGGUUACCCUUUGAGA chr19 54201703 . C U 0.000199681 +
65
hsa-mir-524
UCUCAUGCUGUGACCCUACAAAGGGAAGCACUUUCUCUUGUCCAAAGGAAAAGAAGGCGCUUCCCUUU
GGAGUGUUACGGUUUGAGA chr19 54214286 . C G 0.000599042 +
31
hsa-mir-524
UCUCAUGCUGUGACCCUACAAAGGGAAGCACUUUCUCUUGUCCAAAGGAAAAGAAGGCGCUUCCCUUU
GGAGUGUUACGGUUUGAGA chr19 54214312 rs368405432 G C
0.000199681 + 57
hsa-mir-524
UCUCAUGCUGUGACCCUACAAAGGGAAGCACUUUCUCUUGUCCAAAGGAAAAGAAGGCGCUUCCCUUU
GGAGUGUUACGGUUUGAGA chr19 54214333 . C U 0.000199681 +
78
hsa-mir-525
CUCAAGCUGUGACUCUCCAGAGGGAUGCACUUUCUCUUAUGUGAAAAAAAAGAAGGCGCUUCCCUUUA
GAGCGUUACGGUUUGGG chr19 54200810 rs377251394 G U

0.000199681 + 24
hsa-mir-525
CUCAAGCUGUGACUCUCCAGAGGGAUGCACUUUCUCUUAUGUGAAAAAAAAGAAGGCGCUUCCCUUUA
GAGCGUUACGGUUUGGG chr19 54200826 . U C 0.000199681 +
40
hsa-mir-525
CUCAAGCUGUGACUCUCCAGAGGGAUGCACUUUCUCUUAUGUGAAAAAAAAGAAGGCGCUUCCCUUUA
GAGCGUUACGGUUUGGG chr19 54200830 rs80268200 A G null
null 44
hsa-mir-525
CUCAAGCUGUGACUCUCCAGAGGGAUGCACUUUCUCUUAUGUGAAAAAAAAGAAGGCGCUUCCCUUUA
GAGCGUUACGGUUUGGG chr19 54200834 . A G 0.000199681 +
48
hsa-mir-525
CUCAAGCUGUGACUCUCCAGAGGGAUGCACUUUCUCUUAUGUGAAAAAAAAGAAGGCGCUUCCCUUUA
GAGCGUUACGGUUUGGG chr19 54200843 rs190453265 C U
0.000399361 + 57
hsa-mir-525
CUCAAGCUGUGACUCUCCAGAGGGAUGCACUUUCUCUUAUGUGAAAAAAAAGAAGGCGCUUCCCUUUA
GAGCGUUACGGUUUGGG chr19 54200853 rs374132553 U C
0.000998403 + 67
hsa-mir-526a-1
CUCAGGCUGUGACCCUCUAGAGGGAAGCACUUUCUGUUGCUUGAAAGAAGAGAAAGCGCUUCCUUUUA
GAGGAUUACUCUUUGAG chr19 54209517 rs374967660 A G
0.000199681 + 12
hsa-mir-526a-1
CUCAGGCUGUGACCCUCUAGAGGGAAGCACUUUCUGUUGCUUGAAAGAAGAGAAAGCGCUUCCUUUUA
GAGGAUUACUCUUUGAG chr19 54209527 . G A 0.000599042 +
22
hsa-mir-526a-1
CUCAGGCUGUGACCCUCUAGAGGGAAGCACUUUCUGUUGCUUGAAAGAAGAGAAAGCGCUUCCUUUUA
GAGGAUUACUCUUUGAG chr19 54209563 rs369843820 G A
0.000199681 + 58
hsa-mir-526a-2
GUGACCCUCUAGAGGGAAGCACUUUCUGUUGAAAGAAAAGAACAUGCAUCCUUUCAGAGGGUUAC
chr19 54230219 rs113349469 A U 0.00279553 + 44

hsa-mir-526b
UCAGGCUGUGACCCUCUUGAGGGAAGCACUUUCUGUUGUCUGAAAGAAGAGAAAGUGCUUCCUUUUAG
AGGCUUACUGUCUGA chr19 54197660 . C G 0.000199681 +
14
hsa-mir-526b
UCAGGCUGUGACCCUCUUGAGGGAAGCACUUUCUGUUGUCUGAAAGAAGAGAAAGUGCUUCCUUUUAG
AGGCUUACUGUCUGA chr19 54197674 . A G 0.000199681 +
28
hsa-mir-526b
UCAGGCUGUGACCCUCUUGAGGGAAGCACUUUCUGUUGUCUGAAAGAAGAGAAAGUGCUUCCUUUUAG

AGGCUUACUGUCUGA chr19 54197678 rs370046048 U A
0.000399361 + 32
hsa-mir-526b
UCAGGCUGUGACCCUCUUGAGGGAAGCACUUUCUGUUGUCUGAAAGAAGAGAAAGUGCUUCCUUUUAG
AGGCUUACUGUCUGA chr19 54197706 rs377624244 U C
0.000199681 + 60
hsa-mir-527
UCUCAAGCUGUGACUGCAAAGGGAAGCCCUUUCUGUUGUCUAAAAGAAAAGAAAGUGCUUCCCUUUGG
UGAAUUACGGUUUGAGA chr19 54257304 rs186554893 C U
0.000199681 + 33
hsa-mir-527
UCUCAAGCUGUGACUGCAAAGGGAAGCCCUUUCUGUUGUCUAAAAGAAAAGAAAGUGCUUCCCUUUGG
UGAAUUACGGUUUGAGA chr19 54257325 rs201771171 A G
0.000199681 + 54
hsa-mir-532
CGACUUGCUUUCUCUCCUCCAUGCCUUGAGUGUAGGACCGUUGGCAUCUUAAUUACCCUCCCACACCC
AAGGCUUGCAGAAGAGCGAGCCU chrX 49767769 rs371160078 C U
0.000529801 + 16
hsa-mir-532
CGACUUGCUUUCUCUCCUCCAUGCCUUGAGUGUAGGACCGUUGGCAUCUUAAUUACCCUCCCACACCC
AAGGCUUGCAGAAGAGCGAGCCU chrX 49767815 . C U
0.000264901 + 62
hsa-mir-532
CGACUUGCUUUCUCUCCUCCAUGCCUUGAGUGUAGGACCGUUGGCAUCUUAAUUACCCUCCCACACCC
AAGGCUUGCAGAAGAGCGAGCCU chrX 49767832 rs456615 A G 1
+ 79
hsa-mir-532
CGACUUGCUUUCUCUCCUCCAUGCCUUGAGUGUAGGACCGUUGGCAUCUUAAUUACCCUCCCACACCC
AAGGCUUGCAGAAGAGCGAGCCU chrX 49767835 rs456617 A G 1
+ 82
hsa-mir-532
CGACUUGCUUUCUCUCCUCCAUGCCUUGAGUGUAGGACCGUUGGCAUCUUAAUUACCCUCCCACACCC
AAGGCUUGCAGAAGAGCGAGCCU chrX 49767838 rs190537859 C U
0.000264901 + 85
hsa-mir-541
ACGUCAGGGAAAGGAUUCUGCUGUCGGUCCCACUCCAAAGUUCACAGAAUGGGUGGUGGGCACAGAAU
CUGGACUCUGCUUGUG chr14 101530833 . C U 0.000199681 +
2
hsa-mir-541
ACGUCAGGGAAAGGAUUCUGCUGUCGGUCCCACUCCAAAGUUCACAGAAUGGGUGGUGGGCACAGAAU
CUGGACUCUGCUUGUG chr14 101530834 rs369875669 G A
0.000399361 + 3
hsa-mir-541
ACGUCAGGGAAAGGAUUCUGCUGUCGGUCCCACUCCAAAGUUCACAGAAUGGGUGGUGGGCACAGAAU
CUGGACUCUGCUUGUG chr14 101530873 rs138633099 U G
0.000599042 + 42
hsa-mir-542

CAGAUCUCAGACAUCUCGGGGAUCAUCAUGUCACGAGAUACCAGUGUGCACUUGUGACAGAUUGAUAA CUGAAAGGUCUGGGAGCCACUCAUCUUCA chrX 133675375 rs190041958 C A 0.000264901 - 93
hsa-mir-543
UACUUAAUGAGAAGUUGCCCGUGUUUUUUUCGCUUUAUUUGUGACGAAACAUUCGCGGUGCACUUCUU UUUCAGUAUC chr14 101498381 . G A 0.000199681 + 58

hsa-mir-543
UACUUAAUGAGAAGUUGCCCGUGUUUUUUUCGCUUUAUUUGUGACGAAACAUUCGCGGUGCACUUCUU UUUCAGUAUC chr14 101498399 . A G 0.000199681 + 76

hsa-mir-544a
AUUUUCAUCACCUAGGGAUCUUGUUAAAAAGCAGAUUCUGAUUCAGGGACCAAGAUUCUGCAUUUUUA GCAAGUUCUCAAGUGAUGCUAAU chr14 101515025 . G U 0.000199681 + 31
hsa-mir-544a
AUUUUCAUCACCUAGGGAUCUUGUUAAAAAGCAGAUUCUGAUUCAGGGACCAAGAUUCUGCAUUUUUA GCAAGUUCUCAAGUGAUGCUAAU chr14 101515083 . A C 0.000199681 + 89
hsa-mir-544b
GGAAUUUUGUUAAAAUGCAGAAUCCAUUUCUGUAGCUCUGAGACUAGACCUGAGGUUGUGCAUUUCUA ACAAAGUGCC chr3 124451312 rs10934682 U G 0.180112 + 27
hsa-mir-544b
GGAAUUUUGUUAAAAUGCAGAAUCCAUUUCUGUAGCUCUGAGACUAGACCUGAGGUUGUGCAUUUCUA ACAAAGUGCC chr3 124451357 . A G 0 + 72

hsa-mir-545
CCCAGCCUGGCACAUUAGUAGGCCUCAGUAAAUGUUUAUUAGAUGAAUAAAUGAAUGACUCAUCAGCA AACAUUUAUUGUGUGCCUGCUAAAGUGAGCUCCACAGG chrX 73506984 . C U 0.000264901 - 61
hsa-mir-548a-1
UGCAGGGAGGUAUUAAGUUGGUGCAAAAGUAAUUGUGAUUUUUGCCAUUAAAAGUAACGACAAAACUG GCAAUUACUUUUGCACCAAACCUGGUAUU chr6 18572056 rs12197631 U G 0.139776 + 42
hsa-mir-548a-1
UGCAGGGAGGUAUUAAGUUGGUGCAAAAGUAAUUGUGAUUUUUGCCAUUAAAAGUAACGACAAAACUG GCAAUUACUUUUGCACCAAACCUGGUAUU chr6 18572070 . A G 0.000599042 + 56
hsa-mir-548a-1
UGCAGGGAGGUAUUAAGUUGGUGCAAAAGUAAUUGUGAUUUUUGCCAUUAAAAGUAACGACAAAACUG GCAAUUACUUUUGCACCAAACCUGGUAUU chr6 18572073 . G A 0.000199681 + 59
hsa-mir-548a-2
UGUGAUGUGUAUUAGGUUUGUGCAAAAGUAAUUGGGGUUUUUUGCCGUUAAAAGUAAUGGCAAAACUG GCAAUUACUUUUGCACCAAACUAAUAUAA chr6 135560369 rs373439066 A C 0.000199681 + 72

hsa-mir-548a-2
UGUGAUGUGUAUUAGGUUUGUGCAAAAGUAAUUGGGGUUUUUGCCGUUAAAAGUAAUGGCAAAACUG
GCAAUUACUUUUGCACCAAACUAAUAUAA chr6 135560387 . U C
0.000199681 + 90
hsa-mir-548a-3
CCUAGAAUGUUAUUAGGUCGGUGCAAAAGUAAUUGCGAGUUUUACCAUUACUUUCAAUGGCAAAACUG
GCAAUUACUUUUGCACCAACGUAAUACUU chr8 105496681 . U C
0.000199681 - 13
hsa-mir-548a-3
CCUAGAAUGUUAUUAGGUCGGUGCAAAAGUAAUUGCGAGUUUUACCAUUACUUUCAAUGGCAAAACUG
GCAAUUACUUUUGCACCAACGUAAUACUU chr8 105496675 rs150167306 C A
0.00299521 - 19
hsa-mir-548a-3
CCUAGAAUGUUAUUAGGUCGGUGCAAAAGUAAUUGCGAGUUUUACCAUUACUUUCAAUGGCAAAACUG
GCAAUUACUUUUGCACCAACGUAAUACUU chr8 105496638 rs71297276 AUUACUUUCA
- null null 56
hsa-mir-548a-3
CCUAGAAUGUUAUUAGGUCGGUGCAAAAGUAAUUGCGAGUUUUACCAUUACUUUCAAUGGCAAAACUG
GCAAUUACUUUUGCACCAACGUAAUACUU chr8 105496622 . A G
0.000199681 - 72
hsa-mir-548aa-1
CUUUAUUAGUCUGGUGCAAAAGAAACUGUGGUUUUUGCCAUUACUUUUACAGGCAAAAACCACAAUUA
CUUUUGCACCAACCUAAUAUAACUUGUUU chr8 124360347 . G A
0.000199681 + 74
hsa-mir-548aa-2
UUUUAUUAGGUUGGUGCAAAAGAAACUGUGGUUUUUGCCAUUACUUUCAAUGGCAAAAACCACAAUUA
CUUUUGCACCAACCUAAAUCUUCCCUCUC chr17 65467665 rs149172942 C U
0.000199681 + 61
hsa-mir-548aa-2
UUUUAUUAGGUUGGUGCAAAAGAAACUGUGGUUUUUGCCAUUACUUUCAAUGGCAAAAACCACAAUUA
CUUUUGCACCAACCUAAAUCUUCCCUCUC chr17 65467669 . A G
0.000199681 + 65
hsa-mir-548aa-2
UUUUAUUAGGUUGGUGCAAAAGAAACUGUGGUUUUUGCCAUUACUUUCAAUGGCAAAAACCACAAUUA
CUUUUGCACCAACCUAAAUCUUCCCUCUC chr17 65467701 . C U
0.000199681 + 97
hsa-mir-548ab
AUGUUGGUGCAAAAGUAAUUGUGGAUUUUGCUAUUACUUGUAUUUAUUUGUAAUGCAAAACCCGCAAU
UAGUUUUGCACCAACC chr3 103242954 rs192258471 G A
0.000399361 - 7
hsa-mir-548ab
AUGUUGGUGCAAAAGUAAUUGUGGAUUUUGCUAUUACUUGUAUUUAUUUGUAAUGCAAAACCCGCAAU
UAGUUUUGCACCAACC chr3 103242937 rs59323834 G A 0.0349441
- 24
hsa-mir-548ac
GUAUUAGGUUGGUGCAAAAGUUAUUGUGGUUUUUGCUAUUUUUUUUUAAUGGCAAAAACCGGCAAUUA
CUUUUGCACUAACCUAGUAG chr1 117102696 . A U 0.00179712

- 38
hsa-mir-548ac
GUAUUAGGUUGGUGCAAAAGUUAUUGUGGUUUUUGCUAUUUUUUUUUAAUGGCAAAAACCGGCAAUUACUUUUGCACUAACCUAGUAG chr1 117102674 . C U
0.000199681 - 60
hsa-mir-548ac
GUAUUAGGUUGGUGCAAAAGUUAUUGUGGUUUUUGCUAUUUUUUUUUAAUGGCAAAAACCGGCAAUUACUUUUGCACUAACCUAGUAG chr1 117102660 . G A
0.000199681 - 74
hsa-mir-548ac
GUAUUAGGUUGGUGCAAAAGUUAUUGUGGUUUUUGCUAUUUUUUUUUAAUGGCAAAAACCGGCAAUUACUUUUGCACUAACCUAGUAG chr1 117102649 rs1414273 G A
0.428115 - 85
hsa-mir-548ad
CUGUUAGGUUGGUGCAAAAGUAAUUGUGGUUUUUGAAAGUAACUUGGCGAAAACGACAAUGACUUUUGCACCAAUCUAAUAC chr2 35696481 . G A 0.00159744 +
11
hsa-mir-548ad
CUGUUAGGUUGGUGCAAAAGUAAUUGUGGUUUUUGAAAGUAACUUGGCGAAAACGACAAUGACUUUUGCACCAAUCUAAUAC chr2 35696518 rs147085950 C U
0.000399361 + 48
hsa-mir-548ad
CUGUUAGGUUGGUGCAAAAGUAAUUGUGGUUUUUGAAAGUAACUUGGCGAAAACGACAAUGACUUUUGCACCAAUCUAAUAC chr2 35696519 rs62143301 G A 0.0379393
+ 49
hsa-mir-548ad
CUGUUAGGUUGGUGCAAAAGUAAUUGUGGUUUUUGAAAGUAACUUGGCGAAAACGACAAUGACUUUUGCACCAAUCUAAUAC chr2 35696532 rs115818870 A G 0.0071885
+ 62
hsa-mir-548ae-1
GCAGUUUUUGCCAUUAAGUUGCGGUUUUUGCCAUUAUAAUGGCAAAAACUGCAAUUACUUUCACACCUGC chr2 185243724 . G A 0.000199681 + 23

hsa-mir-548ag-2
UGCAAAGGUAAUUGUGGUUUCUGCCAUUGAAAGUAAAGGCAAGAACCUCAAUUACCUUUGCAGC
chr20 59139674 rs186092922 C A 0.000399361 + 55

hsa-mir-548ai
GUAUUAGGUUGGUGCAAAGGUAAUUGCAGUUUUUCCCAUUUAAAAUAUGGAAAAAAAAAUCACAAUUACUUUUGCAUCAACCUAAUAA chr6 99572563 rs147263554 A G
0.000998403 + 79
hsa-mir-548aj-1
AUUGGUGUAAAAGUAAUUGCAGGUUAUGCCAUUAAAAGUAAUGGUAAAAACUGCAAUUACUUUUACACUAAC chr6 132436375 . C U 0.00119808 - 29

hsa-mir-548aj-2
AAGGUAUUAGGUUGGUGCAAAAGUAAUUGCAGUUUUUGCUAUUACUUUUAAUGGUAAAAACUGCAAUU

ACUUUUACACCAACCUAAUAUUUA chrX 37883200 rs73463468 U C
0.458808 - 40
hsa-mir-548aj-2
AAGGUAUUAGGUUGGUGCAAAAGUAAUUGCAGUUUUUGCUAUUACUUUUAAUGGUAAAAACUGCAAUU
ACUUUUACACCAACCUAAUAUUUA chrX 37883195 . C A
0.000264901 - 45
hsa-mir-548aj-2
AAGGUAUUAGGUUGGUGCAAAAGUAAUUGCAGUUUUUGCUAUUACUUUUAAUGGUAAAAACUGCAAUU
ACUUUUACACCAACCUAAUAUUUA chrX 37883165 rs113330267 A G
0.0511258 - 75
hsa-mir-548akGUGCAAAAGUAACUGCGGUUUUUGAGAAGUAAUUGAAAACCGCAAUUACUUUUGCAG
chr10 12172800 rs114111486 C A 0.00599042 - 16

hsa-mir-548akGUGCAAAAGUAACUGCGGUUUUUGAGAAGUAAUUGAAAACCGCAAUUACUUUUGCAG
chr10 12172799 . G A 0.000199681 - 17

hsa-mir-548akGUGCAAAAGUAACUGCGGUUUUUGAGAAGUAAUUGAAAACCGCAAUUACUUUUGCAG
chr10 12172788 . A C 0.000199681 - 28

hsa-mir-548akGUGCAAAAGUAACUGCGGUUUUUGAGAAGUAAUUGAAAACCGCAAUUACUUUUGCAG
chr10 12172783 . U C 0.000998403 - 33

hsa-mir-548akGUGCAAAAGUAACUGCGGUUUUUGAGAAGUAAUUGAAAACCGCAAUUACUUUUGCAG
chr10 12172775 rs7070684 C U 0.378794 - 41

hsa-mir-548al
GGUCGGUGCAAAAGUAAUUGCUGUUUUUGCCAUUAAAAAUAAUGGCAUUAAAAGUAAUGGCAAAAACG
GCAAUGACUUUUGUACCAAUCUAAUAUCU chr11 74110328 rs10437738 A G
0.0283546 + 47
hsa-mir-548al
GGUCGGUGCAAAAGUAAUUGCUGUUUUUGCCAUUAAAAAUAAUGGCAUUAAAAGUAAUGGCAAAAACG
GCAAUGACUUUUGUACCAAUCUAAUAUCU chr11 74110335 rs60917039 G A
0.0181709 + 54
hsa-mir-548al
GGUCGGUGCAAAAGUAAUUGCUGUUUUUGCCAUUAAAAAUAAUGGCAUUAAAAGUAAUGGCAAAAACG
GCAAUGACUUUUGUACCAAUCUAAUAUCU chr11 74110353 rs515924 A G
0.183906 + 72
hsa-mir-548al
GGUCGGUGCAAAAGUAAUUGCUGUUUUUGCCAUUAAAAAUAAUGGCAUUAAAAGUAAUGGCAAAAACG
GCAAUGACUUUUGUACCAAUCUAAUAUCU chr11 74110358 rs145394973 U A
0.00139776 + 77
hsa-mir-548am
AGUUGGUGCAAAAGUAAUUGCGGUUUUUGCCGUCGAAAAUAAUGGCAAAAACUGCAGUUACUUUUGUA
CCAAUG chrX 16645208 . A C 0.000264901 - 1

hsa-mir-548am
AGUUGGUGCAAAAGUAAUUGCGGUUUUUGCCGUCGAAAAUAAUGGCAAAAACUGCAGUUACUUUUGUA

CCAAUG chrX 16645199 rs181147953 A C 0.000264901 - 10

hsa-mir-548am
AGUUGGUGCAAAAGUAAUUGCGGUUUUUGCCGUCGAAAAUAAUGGCAAAAACUGCAGUUACUUUUGUACCAAUG chrX 16645178 . C U 0.000794702 - 31

hsa-mir-548am
AGUUGGUGCAAAAGUAAUUGCGGUUUUUGCCGUCGAAAAUAAUGGCAAAAACUGCAGUUACUUUUGUACCAAUG chrX 16645137 . A G 0.000264901 - 72

hsa-mir-548an
CAUUAGGUUGGUGCAAAAGGCAUUGUGGUUUUUGCCUAUAAAAGUAAUGGCAAAAACCGCAAUUCCUUUUGCACCAACCUAAU chrX 105883051 . U C 0.000264901 + 8

hsa-mir-548an
CAUUAGGUUGGUGCAAAAGGCAUUGUGGUUUUUGCCUAUAAAAGUAAUGGCAAAAACCGCAAUUCCUUUUGCACCAACCUAAU chrX 105883081 . A G 0.000264901 + 38

hsa-mir-548an
CAUUAGGUUGGUGCAAAAGGCAUUGUGGUUUUUGCCUAUAAAAGUAAUGGCAAAAACCGCAAUUCCUUUUGCACCAACCUAAU chrX 105883120 . A U 0.000264901 + 77

hsa-mir-548ao
AACUAUUCUUAGGUUGAUGCAGAAGUAACUACGGUUUUUGCAGUUGAAAGUAAUGGCAAAGACCGUGACUACUUUUGCAACAGCCUAAUAGUUUCU chr8 41128620 rs138586956 G A 0.00119808 - 43

hsa-mir-548ao
AACUAUUCUUAGGUUGAUGCAGAAGUAACUACGGUUUUUGCAGUUGAAAGUAAUGGCAAAGACCGUGACUACUUUUGCAACAGCCUAAUAGUUUCU chr8 41128599 rs79091838 C U 0.0147764 - 64

hsa-mir-548ao
AACUAUUCUUAGGUUGAUGCAGAAGUAACUACGGUUUUUGCAGUUGAAAGUAAUGGCAAAGACCGUGACUACUUUUGCAACAGCCUAAUAGUUUCU chr8 41128578 rs187877820 C U 0.000199681 - 85

hsa-mir-548ap
ACCAAUUCCUAGGUUGGUGCAAAAGUAAUUGCGGUCUUUGUCAUUAAAACCAAUAACAAAAACCACAAUUACUUUUUACUGACCUAAAGAUUAAUU chr15 86368890 rs186097449 G C 0.000599042 + 25

hsa-mir-548ap
ACCAAUUCCUAGGUUGGUGCAAAAGUAAUUGCGGUCUUUGUCAUUAAAACCAAUAACAAAAACCACAAUUACUUUUUACUGACCUAAAGAUUAAUU chr15 86368898 rs4414449 G A 0.748203 + 33

hsa-mir-548ap
ACCAAUUCCUAGGUUGGUGCAAAAGUAAUUGCGGUCUUUGUCAUUAAAACCAAUAACAAAAACCACAAUUACUUUUUACUGACCUAAAGAUUAAUU chr15 86368922 rs76468441 C U 0.0321486 + 57

hsa-mir-548ap

ACCAAUUCCUAGGUUGGUGCAAAAGUAAUUGCGGUCUUUGUCAUUAAAACCAAUAACAAAAACCACAA UUACUUUUUACUGACCUAAAGAUUAAUU chr15 86368929 rs150751643 C A 0.000399361 + 64

hsa-mir-548ap ACCAAUUCCUAGGUUGGUGCAAAAGUAAUUGCGGUCUUUGUCAUUAAAACCAAUAACAAAAACCACAA UUACUUUUUACUGACCUAAAGAUUAAUU chr15 86368959 rs4577031 A U 0.748003 + 94

hsa-mir-548aqGAAAGUAAUUGCUGUUUUUGCCAUUACUUUCAGUGGCAAAAACUGCAAUUACUUUUGC chr3 185485689 rs148741643 A G 0.000199681 - 4

hsa-mir-548aqGAAAGUAAUUGCUGUUUUUGCCAUUACUUUCAGUGGCAAAAACUGCAAUUACUUUUGC chr3 185485646 rs144265760 A G 0.00199681 - 47

hsa-mir-548arAAAAGUAAUUGCAGUUUUUGCUGUUGAACGUAGUGGUAAAACUGCAGUUAUUUUUGC chr13 115010026 rs141659366 G A 0.00898562 + 47

hsa-mir-548asAAAAGUAAUUGCGGGUUUUGCCGUUGCUUUUAAUGGUAAAACCCACAAUUAUGUUUGU chr13 93142427 rs112416833 C U 0.00119808 + 12

hsa-mir-548atAAAAGUUAUUGCGGUUUUGGCUGCCAAAAGAAAUGGCCAAAACCGCAGUAACUUUUGU chr17 40646795 . G A 0.000199681 + 5

hsa-mir-548atAAAAGUUAUUGCGGUUUUGGCUGCCAAAAGAAAUGGCCAAAACCGCAGUAACUUUUGU chr17 40646803 rs11651671 G A 0.170527 + 13

hsa-mir-548atAAAAGUUAUUGCGGUUUUGGCUGCCAAAAGAAAUGGCCAAAACCGCAGUAACUUUUGU chr17 40646816 . A G 0.000399361 + 26

hsa-mir-548atAAAAGUUAUUGCGGUUUUGGCUGCCAAAAGAAAUGGCCAAAACCGCAGUAACUUUUGU chr17 40646834 . C U 0.000199681 + 44

hsa-mir-548auAAAAGUAAUUGCGGUUUUUGCUAUUGGUUUUAAUGGCAGUUACUUUUGCACCAG chr9 96357135 rs138894217 G A 0.00119808 + 13

hsa-mir-548auAAAAGUAAUUGCGGUUUUUGCUAUUGGUUUUAAUGGCAGUUACUUUUGCACCAG chr9 96357140 . U A 0.000199681 + 18

hsa-mir-548auAAAAGUAAUUGCGGUUUUUGCUAUUGGUUUUAAUGGCAGUUACUUUUGCACCAG chr9 96357145 . A G 0.000199681 + 23

hsa-mir-548avAAAAGUACUUGCGGAUUUGCCAUCACCUUUACCUUUAAUGGCAAAACUGCAGUUACUUUUGC chr18 70520606 rs138070126 C U 0.000599042 - 12

hsa-mir-548avAAAAGUACUUGCGGAUUUGCCAUCACCUUUACCUUUAAUGGCAAAACUGCAGUUACUUUUGC chr18 70520586 . C U 0.000199681 - 32

hsa-mir-548aw

UAGGUCGGUGCAAAAGUCAUCACGGUUUUUACCAUUAAAACCGCGAUGACUUUUGCAUCAACCUA
chr9 135821099 rs56195815 C U 0.179712 + 6

hsa-mir-548aw
UAGGUCGGUGCAAAAGUCAUCACGGUUUUUACCAUUAAAACCGCGAUGACUUUUGCAUCAACCUA
chr9 135821135 rs73660564 C U 0.00599042 + 42

hsa-mir-548ay
AGAAGAUGCUUACUACUAGGUUGGUGCAAAAGUAAUUGUGGUUUUUGCAUUUAAAGUAAUGGCCAAAACCGCGAUUACUCUUGCACGAACCUAACGGUAACACUUCU chr3 32547811 . G A 0.000599042 - 71
hsa-mir-548ay
AGAAGAUGCUUACUACUAGGUUGGUGCAAAAGUAAUUGUGGUUUUUGCAUUUAAAGUAAUGGCCAAAACCGCGAUUACUCUUGCACGAACCUAACGGUAACACUUCU chr3 32547810 rs116241207 C U 0.000599042 - 72
hsa-mir-548ay
AGAAGAUGCUUACUACUAGGUUGGUGCAAAAGUAAUUGUGGUUUUUGCAUUUAAAGUAAUGGCCAAAACCGCGAUUACUCUUGCACGAACCUAACGGUAACACUUCU chr3 32547809 . G A 0.000199681 - 73
hsa-mir-548ay
AGAAGAUGCUUACUACUAGGUUGGUGCAAAAGUAAUUGUGGUUUUUGCAUUUAAAGUAAUGGCCAAAACCGCGAUUACUCUUGCACGAACCUAACGGUAACACUUCU chr3 32547795 rs138821150 G A 0.000199681 - 87
hsa-mir-548b
CAGACUAUAUAUUUAGGUUGGCGCAAAAGUAAUUGUGGUUUUGGCCUUUAUUUUCAAUGGCAAGAACCUCAGUUGCUUUUGUGCCAACCUAAUACUU chr6 119390294 . A G 0.000199681 - 15
hsa-mir-548b
CAGACUAUAUAUUUAGGUUGGCGCAAAAGUAAUUGUGGUUUUGGCCUUUAUUUUCAAUGGCAAGAACCUCAGUUGCUUUUGUGCCAACCUAAUACUU chr6 119390286 rs367701690 G A 0.000399361 - 23
hsa-mir-548b
CAGACUAUAUAUUUAGGUUGGCGCAAAAGUAAUUGUGGUUUUGGCCUUUAUUUUCAAUGGCAAGAACCUCAGUUGCUUUUGUGCCAACCUAAUACUU chr6 119390263 rs188182113 C G 0.000998403 - 46
hsa-mir-548baAAAGGUAACUGUGAUUUUUGCUAUUAGAAAGUAAUGGCAAAAACUGCAAUUACUUU
chr2 49286766 . U C 0.000199681 + 25

hsa-mir-548bb
UUAGAUUGGUGCAAAAGUAACUAUGGUUUUUGCCCAAAAACCAUAGUUACUUUUGCACCAAGCUAA
chr3 60603554 rs112971135 C A 0.000798722 - 50

hsa-mir-548bb
UUAGAUUGGUGCAAAAGUAACUAUGGUUUUUGCCCAAAAACCAUAGUUACUUUUGCACCAAGCUAA
chr3 60603553 . U G 0.000199681 - 51

hsa-mir-548bb

UUAGAUUGGUGCAAAAGUAACUAUGGUUUUUGCCCAAAAACCAUAGUUACUUUUGCACCAAGCUAA chr3 60603541 rs376966319 C G 0.000399361 - 63

hsa-mir-548c
CAUUGGCAUCUAUUAGGUUGGUGCAAAAGUAAUUGCGGUUUUUGCCAUUACUUUCAGUAGCAAAAAUCUCAAUUACUUUUGCACCAACUUAAUACUU chr12 65016300 rs17120527 A G 0.0625 + 12
hsa-mir-548c
CAUUGGCAUCUAUUAGGUUGGUGCAAAAGUAAUUGCGGUUUUUGCCAUUACUUUCAGUAGCAAAAAUCUCAAUUACUUUUGCACCAACUUAAUACUU chr12 65016325 . G A 0.000199681 + 37
hsa-mir-548d-1
AAACAAGUUAUAUUAGGUUGGUGCAAAAGUAAUUGUGGUUUUUGCCUGUAAAAGUAAUGGCAAAAACCACAGUUUCUUUUGCACCAGACUAAUAAAG chr8 124360347 . C U 0.000199681 - 24
hsa-mir-548d-2
GAGAGGGAAGAUUUAGGUUGGUGCAAAAGUAAUUGUGGUUUUUGCCAUUGAAAGUAAUGGCAAAAACCACAGUUUCUUUUGCACCAACCUAAUAAAA chr17 65467701 . G A 0.000199681 - 1
hsa-mir-548d-2
GAGAGGGAAGAUUUAGGUUGGUGCAAAAGUAAUUGUGGUUUUUGCCAUUGAAAGUAAUGGCAAAAACCACAGUUUCUUUUGCACCAACCUAAUAAAA chr17 65467669 . U C 0.000199681 - 33
hsa-mir-548d-2
GAGAGGGAAGAUUUAGGUUGGUGCAAAAGUAAUUGUGGUUUUUGCCAUUGAAAGUAAUGGCAAAAACCACAGUUUCUUUUGCACCAACCUAAUAAAA chr17 65467665 rs149172942 G A 0.000199681 - 37
hsa-mir-548f-1
AUUAGGUUGGUGCAAAAGUAAUCACAGUUUUUGACAUUACUUUCAAAGACAAAAACUGUAAUUACUUUUGGACCAACCUAAUAG chr10 56367696 . U A 0.000199681 - 22
hsa-mir-548f-2
UAAUAACUAUUAGGUUGGUGCGAACAUAAUUGCAGUUUUUAUCAUUACUUUUAAUGGCAAAAACUGUAAUUACUUUUGCACCAACCUAAUAUUUUAGU chr2 213291041 . A G 0.000199681 - 44
hsa-mir-548f-2
UAAUAACUAUUAGGUUGGUGCGAACAUAAUUGCAGUUUUUAUCAUUACUUUUAAUGGCAAAAACUGUAAUUACUUUUGCACCAACCUAAUAUUUUAGU chr2 213291038 . A G 0.000199681 - 47
hsa-mir-548f-2
UAAUAACUAUUAGGUUGGUGCGAACAUAAUUGCAGUUUUUAUCAUUACUUUUAAUGGCAAAAACUGUAAUUACUUUUGCACCAACCUAAUAUUUUAGU chr2 213291030 . U A 0.000199681 - 55
hsa-mir-548f-3
AUUAGGUUGGUGCAAACCUAAUUGCAAUUUUUGCAGUUUUUUUAAGUAAUUGCAAAAACUGUAAUUACUUUUGCACCAACCUAAUAC chr5 109849556 . G A 0.000798722 - 61

hsa-mir-548f-4
GAGUUCUAACGUAUUAGGUUGGUGCAAAAGUAAUAGUGGUUUUUGCCAUUAAAAGUAAUGACAAAAAC
UGUAAUUACUUUUGGAACAAUAUUAAUAGAAUUUCAG chr7 147075204 rs193186885 C
U 0.000199681 - 10
hsa-mir-548f-4
GAGUUCUAACGUAUUAGGUUGGUGCAAAAGUAAUAGUGGUUUUUGCCAUUAAAAGUAAUGACAAAAAC
UGUAAUUACUUUUGGAACAAUAUUAAUAGAAUUUCAG chr7 147075166 . A C
0.000399361 - 48
hsa-mir-548f-4
GAGUUCUAACGUAUUAGGUUGGUGCAAAAGUAAUAGUGGUUUUUGCCAUUAAAAGUAAUGACAAAAAC
UGUAAUUACUUUUGGAACAAUAUUAAUAGAAUUUCAG chr7 147075152 . C U
0.000199681 - 62
hsa-mir-548f-4
GAGUUCUAACGUAUUAGGUUGGUGCAAAAGUAAUAGUGGUUUUUGCCAUUAAAAGUAAUGACAAAAAC
UGUAAUUACUUUUGGAACAAUAUUAAUAGAAUUUCAG chr7 147075121 . A U
0.000199681 - 93
hsa-mir-548f-4
GAGUUCUAACGUAUUAGGUUGGUGCAAAAGUAAUAGUGGUUUUUGCCAUUAAAAGUAAUGACAAAAAC
UGUAAUUACUUUUGGAACAAUAUUAAUAGAAUUUCAG chr7 147075120 rs114864936 A
G 0.00638978 - 94
hsa-mir-548f-5
UAUUAGGUUGCUGCAAAAGUAAUCAUGUUUUUUUCCAUUGUAAGUAAUGGGAAAAACUGUAAUUACUU
UUGUACCAACCUAAUAGC chrX 32659592 rs60180387 G A 0.0270199
- 85
hsa-mir-548g
AGUUAUUAGAUUAGUGCAAAAGUAAUUGCAGUUUUUGCAUUACGUUCUAUGGCAAAACUGUAAUUACU
UUUGUACCAACAUAAUACUUC chr4 148265864 . U C
0.000199681 - 6
hsa-mir-548h-1
UCUGUCCAUUAGGUGGGUGCAAAAGUAAUCGCGGUUUUUGUCAUUACUUUUAAUGGUAAAAACUGGAA
UUACUUUUGCACUGACCUAAUAUUAAGCCAGAUA chr14 64561812 rs144194694 C
U 0.00319489 - 32
hsa-mir-548h-1
UCUGUCCAUUAGGUGGGUGCAAAAGUAAUCGCGGUUUUUGUCAUUACUUUUAAUGGUAAAAACUGGAA
UUACUUUUGCACUGACCUAAUAUUAAGCCAGAUA chr14 64561772 . C U
0.000199681 - 72
hsa-mir-548h-2
GUAUUAGGUUGGUGCAAAAGUAAUCGCGGUUUUUGUCAUUACUUUCAAUGGCAAACACCACAAUUACU
UUUGCACCAACCUAAUAUAA chr16 11400358 . C U
0.000199681 - 27
hsa-mir-548h-2
GUAUUAGGUUGGUGCAAAAGUAAUCGCGGUUUUUGUCAUUACUUUCAAUGGCAAACACCACAAUUACU
UUUGCACCAACCUAAUAUAA chr16 11400356 rs149601213 G A
0.000199681 - 29
hsa-mir-548h-2
GUAUUAGGUUGGUGCAAAAGUAAUCGCGGUUUUUGUCAUUACUUUCAAUGGCAAACACCACAAUUACU
UUUGCACCAACCUAAUAUAA chr16 11400324 rs138584205 C A

0.00119808 - 61
hsa-mir-548h-2
GUAUUAGGUUGGUGCAAAAGUAAUCGCGGUUUUUGUCAUUACUUUCAAUGGCAAACACCACAAUUACU
UUUGCACCAACCUAAUAUAA chr16 11400303 . A U
0.000199681 - 82
hsa-mir-548h-3
UCUGAUUCUGCAUGUAUUAGGUUGGUGCAAAAGUAAUCGCGGUUUUUGUCAUUGAAAGUAAUAGCAAA
AACUGCAAUUACUUUUGCACCAACCUAAAAGUAGUCACUGUCUUCAGAUA chr17 13446924
rs9913045 C U 0.430911 - 40

hsa-mir-548h-3
UCUGAUUCUGCAUGUAUUAGGUUGGUGCAAAAGUAAUCGCGGUUUUUGUCAUUGAAAGUAAUAGCAAA
AACUGCAAUUACUUUUGCACCAACCUAAAAGUAGUCACUGUCUUCAGAUA chr17 13446906 .
G A 0.000199681 - 58
hsa-mir-548h-3
UCUGAUUCUGCAUGUAUUAGGUUGGUGCAAAAGUAAUCGCGGUUUUUGUCAUUGAAAGUAAUAGCAAA
AACUGCAAUUACUUUUGCACCAACCUAAAAGUAGUCACUGUCUUCAGAUA chr17 13446893 .
C U 0.00459265 - 71
hsa-mir-548h-3
UCUGAUUCUGCAUGUAUUAGGUUGGUGCAAAAGUAAUCGCGGUUUUUGUCAUUGAAAGUAAUAGCAAA
AACUGCAAUUACUUUUGCACCAACCUAAAAGUAGUCACUGUCUUCAGAUA chr17 13446849
rs189427199 G U 0.000199681 - 115

hsa-mir-548h-3
UCUGAUUCUGCAUGUAUUAGGUUGGUGCAAAAGUAAUCGCGGUUUUUGUCAUUGAAAGUAAUAGCAAA
AACUGCAAUUACUUUUGCACCAACCUAAAAGUAGUCACUGUCUUCAGAUA chr17 13446848 .
A U 0.000399361 - 116
hsa-mir-548h-3
UCUGAUUCUGCAUGUAUUAGGUUGGUGCAAAAGUAAUCGCGGUUUUUGUCAUUGAAAGUAAUAGCAAA
AACUGCAAUUACUUUUGCACCAACCUAAAAGUAGUCACUGUCUUCAGAUA chr17 13446846
rs140470503 A G 0.00579073 - 118

hsa-mir-548h-4
GCUAUUAGGUUGGUGCAAAAGUAAUCGCGGUUUUUGUCAUUACUUUAAUUACUUUACGUUUCAUUAAU
GACAAAAACCGCAAUUACUUUUGCACCAACCUAAUACUUGCUA chr8 26906471
rs115507344 U C 0.00199681 - 10

hsa-mir-548h-4
GCUAUUAGGUUGGUGCAAAAGUAAUCGCGGUUUUUGUCAUUACUUUAAUUACUUUACGUUUCAUUAAU
GACAAAAACCGCAAUUACUUUUGCACCAACCUAAUACUUGCUA chr8 26906452 . G
A 0.000599042 - 29
hsa-mir-548h-4
GCUAUUAGGUUGGUGCAAAAGUAAUCGCGGUUUUUGUCAUUACUUUAAUUACUUUACGUUUCAUUAAU
GACAAAAACCGCAAUUACUUUUGCACCAACCUAAUACUUGCUA chr8 26906437
rs184537764 U C 0.0395367 - 44

hsa-mir-548h-4

GCUAUUAGGUUGGUGCAAAAGUAAUCGCGGUUUUUGUCAUUACUUUAAUUACUUUACGUUUCAUUAAUGACAAAAACCGCAAUUACUUUUGCACCAACCUAAUACUUGCUA chr8 26906423 . G A 0.000199681 - 58

hsa-mir-548h-4
GCUAUUAGGUUGGUGCAAAAGUAAUCGCGGUUUUUGUCAUUACUUUAAUUACUUUACGUUUCAUUAAUGACAAAAACCGCAAUUACUUUUGCACCAACCUAAUACUUGCUA chr8 26906403 . C U 0.000199681 - 78

hsa-mir-548h-4
GCUAUUAGGUUGGUGCAAAAGUAAUCGCGGUUUUUGUCAUUACUUUAAUUACUUUACGUUUCAUUAAUGACAAAAACCGCAAUUACUUUUGCACCAACCUAAUACUUGCUA chr8 26906402 rs73235381 G A 0.0393371 - 79

hsa-mir-548h-5
ACAAAAGUAAUCGCGGUUUUUGUCAUUACUUUUAACUGUAAAAACCACGGUUGCUUUUGC chr6 132113344 . U C 0.00219649 + 33

hsa-mir-548h-5
ACAAAAGUAAUCGCGGUUUUUGUCAUUACUUUUAACUGUAAAAACCACGGUUGCUUUUGC chr6 132113349 . G A 0.000399361 + 38

hsa-mir-548h-5
ACAAAAGUAAUCGCGGUUUUUGUCAUUACUUUUAACUGUAAAAACCACGGUUGCUUUUGC chr6 132113371 . C U 0.000199681 + 60

hsa-mir-548i-1
CAGAUGGCUCUGAAGUUUGCACCCUAUUAGGUUGGUGCAAAAGUAAUUGCGGAUUUUGCCAUUAAAAGUAAUGGCAAAAAUAGCAAUUAUUUUUGUACCAGCCUAGUAUCUUUUCUCCUUCUACCAAACUUUGUCCCUGAGCCAUCUCA chr3 125509374 rs34864809 - C null null 22

hsa-mir-548i-1
CAGAUGGCUCUGAAGUUUGCACCCUAUUAGGUUGGUGCAAAAGUAAUUGCGGAUUUUGCCAUUAAAAGUAAUGGCAAAAAUAGCAAUUAUUUUUGUACCAGCCUAGUAUCUUUUCUCCUUCUACCAAACUUUGUCCCUGAGCCAUCUCA chr3 125509346 rs200103239 C U 0.00199681 - 50

hsa-mir-548i-1
CAGAUGGCUCUGAAGUUUGCACCCUAUUAGGUUGGUGCAAAAGUAAUUGCGGAUUUUGCCAUUAAAAGUAAUGGCAAAAAUAGCAAUUAUUUUUGUACCAGCCUAGUAUCUUUUCUCCUUCUACCAAACUUUGUCCCUGAGCCAUCUCA chr3 125509345 . G A 0.000199681 - 51

hsa-mir-548i-1
CAGAUGGCUCUGAAGUUUGCACCCUAUUAGGUUGGUGCAAAAGUAAUUGCGGAUUUUGCCAUUAAAAGUAAUGGCAAAAAUAGCAAUUAUUUUUGUACCAGCCUAGUAUCUUUUCUCCUUCUACCAAACUUUGUCCCUGAGCCAUCUCA chr3 125509344 . G U 0.000199681 - 52

hsa-mir-548i-1
CAGAUGGCUCUGAAGUUUGCACCCUAUUAGGUUGGUGCAAAAGUAAUUGCGGAUUUUGCCAUUAAAAGUAAUGGCAAAAAUAGCAAUUAUUUUUGUACCAGCCUAGUAUCUUUUCUCCUUCUACCAAACUUUGUCCCUGAGCCAUCUCA chr3 125509306 . U C 0.000199681 - 90

hsa-mir-548i-1
CAGAUGGCUCUGAAGUUUGCACCCUAUUAGGUUGGUGCAAAAGUAAUUGCGGAUUUUGCCAUUAAAAG
UAAUGGCAAAAAUAGCAAUUAUUUUUGUACCAGCCUAGUAUCUUUUCUCCUUCUACCAAACUUUGUCCCUGAGCC
AUCUCA chr3 125509294 . C U 0.000199681 - 102

hsa-mir-548i-1
CAGAUGGCUCUGAAGUUUGCACCCUAUUAGGUUGGUGCAAAAGUAAUUGCGGAUUUUGCCAUUAAAAG
UAAUGGCAAAAAUAGCAAUUAUUUUUGUACCAGCCUAGUAUCUUUUCUCCUUCUACCAAACUUUGUCCCUGAGCC
AUCUCA chr3 125509270 rs369044448 A G 0.00219649 - 126

hsa-mir-548i-1
CAGAUGGCUCUGAAGUUUGCACCCUAUUAGGUUGGUGCAAAAGUAAUUGCGGAUUUUGCCAUUAAAAG
UAAUGGCAAAAAUAGCAAUUAUUUUUGUACCAGCCUAGUAUCUUUUCUCCUUCUACCAAACUUUGUCCCUGAGCC
AUCUCA chr3 125509254 rs200238772 C A 0.00199681 - 142

hsa-mir-548i-1
CAGAUGGCUCUGAAGUUUGCACCCUAUUAGGUUGGUGCAAAAGUAAUUGCGGAUUUUGCCAUUAAAAG
UAAUGGCAAAAAUAGCAAUUAUUUUUGUACCAGCCUAGUAUCUUUUCUCCUUCUACCAAACUUUGUCCCUGAGCC
AUCUCA chr3 125509250 rs189554431 C U 0.000399361 - 146

hsa-mir-548i-2
UAGAUGGCUCCGAAGUUUGCAUCCUAUUAGUUUGGUGCAAAAGUAAUUGCGGAUUUUGCCAUUAAAAG
UAAUGGCAAAAAUAGCAAUUAUUUUUGUACCAGCCUAGUAUCUUUUCUCCUUCUAACAAAGUUCGUCCCUGAUCC
AUCUCA chr4 9557920 rs199641628 U G 0.00239617 - 18

hsa-mir-548i-2
UAGAUGGCUCCGAAGUUUGCAUCCUAUUAGUUUGGUGCAAAAGUAAUUGCGGAUUUUGCCAUUAAAAG
UAAUGGCAAAAAUAGCAAUUAUUUUUGUACCAGCCUAGUAUCUUUUCUCCUUCUAACAAAGUUCGUCCCUGAUCC
AUCUCA chr4 9557888 rs113384898 C U 0.00199681 - 50

hsa-mir-548i-2
UAGAUGGCUCCGAAGUUUGCAUCCUAUUAGUUUGGUGCAAAAGUAAUUGCGGAUUUUGCCAUUAAAAG
UAAUGGCAAAAAUAGCAAUUAUUUUUGUACCAGCCUAGUAUCUUUUCUCCUUCUAACAAAGUUCGUCCCUGAUCC
AUCUCA chr4 9557857 rs111766467 U C 0.00179712 - 81

hsa-mir-548i-2
UAGAUGGCUCCGAAGUUUGCAUCCUAUUAGUUUGGUGCAAAAGUAAUUGCGGAUUUUGCCAUUAAAAG
UAAUGGCAAAAAUAGCAAUUAUUUUUGUACCAGCCUAGUAUCUUUUCUCCUUCUAACAAAGUUCGUCCCUGAUCC
AUCUCA chr4 9557851 rs140817192 U C 0.00559105 - 87

hsa-mir-548i-2
UAGAUGGCUCCGAAGUUUGCAUCCUAUUAGUUUGGUGCAAAAGUAAUUGCGGAUUUUGCCAUUAAAAG
UAAUGGCAAAAAUAGCAAUUAUUUUUGUACCAGCCUAGUAUCUUUUCUCCUUCUAACAAAGUUCGUCCCUGAUCC
AUCUCA chr4 9557830 rs372885052 A G 0.000199681 - 108

hsa-mir-548i-3

CAGAUGGCUCCGAAGUUUACAUCCUAUUAGGUUUGUGCAAAAGUAAUUGCGGAUUUUGCCAUUAAAAG UAAUGGCAAAAAUAGCAAUUAUUUUUGUACCAGCCUAGUAUCUUUUCUCCUUCUACCAAACUUUGUCCCUGAGCC AUCUCA chr8 7946598 rs113893828 A G null null 14

hsa-mir-548i-3
CAGAUGGCUCCGAAGUUUACAUCCUAUUAGGUUUGUGCAAAAGUAAUUGCGGAUUUUGCCAUUAAAAG UAAUGGCAAAAAUAGCAAUUAUUUUUGUACCAGCCUAGUAUCUUUUCUCCUUCUACCAAACUUUGUCCCUGAGCC AUCUCA chr8 7946560 . G U 0.0439297 - 52

hsa-mir-548i-3
CAGAUGGCUCCGAAGUUUACAUCCUAUUAGGUUUGUGCAAAAGUAAUUGCGGAUUUUGCCAUUAAAAG UAAUGGCAAAAAUAGCAAUUAUUUUUGUACCAGCCUAGUAUCUUUUCUCCUUCUACCAAACUUUGUCCCUGAGCC AUCUCA chr8 7946514 . C G 0.000199681 - 98

hsa-mir-548i-4
AGGUUGGUGCAAAAGUAAUUGCGGAUUUUGCCAUACUUUUAACGGCAAAAACCACAAAUAUUAUUGCA CCAACCUAU chrX 83480814 . G A 0.000264901 - 23

hsa-mir-548i-4
AGGUUGGUGCAAAAGUAAUUGCGGAUUUUGCCAUACUUUUAACGGCAAAAACCACAAAUAUUAUUGCA CCAACCUAU chrX 83480764 rs72632467 C U 0.0728477 - 73

hsa-mir-548j
GGGCAGCCAGUGAAUAGUUAGCUGGUGCAAAAGUAAUUGCGGUCUUUGGUAUUACUUUCAGUGGCAAA AACUGCAUUACUUUUGCACCAGCCUACUAGAACGCUGAGUUCAG chr22 26951279 . U C 0.000199681 - 11

hsa-mir-548j
GGGCAGCCAGUGAAUAGUUAGCUGGUGCAAAAGUAAUUGCGGUCUUUGGUAUUACUUUCAGUGGCAAA AACUGCAUUACUUUUGCACCAGCCUACUAGAACGCUGAGUUCAG chr22 26951249 . G A 0.000199681 - 41

hsa-mir-548j
GGGCAGCCAGUGAAUAGUUAGCUGGUGCAAAAGUAAUUGCGGUCUUUGGUAUUACUUUCAGUGGCAAA AACUGCAUUACUUUUGCACCAGCCUACUAGAACGCUGAGUUCAG chr22 26951246 . C G 0.000199681 - 44

hsa-mir-548j
GGGCAGCCAGUGAAUAGUUAGCUGGUGCAAAAGUAAUUGCGGUCUUUGGUAUUACUUUCAGUGGCAAA AACUGCAUUACUUUUGCACCAGCCUACUAGAACGCUGAGUUCAG chr22 26951215 rs12161068 A G 0.0327476 - 75

hsa-mir-548j
GGGCAGCCAGUGAAUAGUUAGCUGGUGCAAAAGUAAUUGCGGUCUUUGGUAUUACUUUCAGUGGCAAA AACUGCAUUACUUUUGCACCAGCCUACUAGAACGCUGAGUUCAG chr22 26951188 . G U 0.000199681 - 102

hsa-mir-548j
GGGCAGCCAGUGAAUAGUUAGCUGGUGCAAAAGUAAUUGCGGUCUUUGGUAUUACUUUCAGUGGCAAA AACUGCAUUACUUUUGCACCAGCCUACUAGAACGCUGAGUUCAG chr22 26951185 rs4822739 G C 0.141174 - 105

hsa-mir-548k

CUUUUCUCAAGUAUUGCUGUUAGGUUGGUGCAAAAGUACUUGCGGAUUUUGCUUUACUUUUAAUGGCAAAAACCGCAAUUAUUUUUGCUUCAACCUAAUAUGAUGCAAAAUUGGCU chr11 70130103 . C A 0.000199681 + 43

hsa-mir-548k CUUUUCUCAAGUAUUGCUGUUAGGUUGGUGCAAAAGUACUUGCGGAUUUUGCUUUACUUUUAAUGGCAAAAACCGCAAUUAUUUUUGCUUCAACCUAAUAUGAUGCAAAAUUGGCU chr11 70130104 rs368851728 G A 0.00199681 + 44

hsa-mir-548k CUUUUCUCAAGUAUUGCUGUUAGGUUGGUGCAAAAGUACUUGCGGAUUUUGCUUUACUUUUAAUGGCAAAAACCGCAAUUAUUUUUGCUUCAACCUAAUAUGAUGCAAAAUUGGCU chr11 70130135 rs373477890 G A 0.000399361 + 75

hsa-mir-548k CUUUUCUCAAGUAUUGCUGUUAGGUUGGUGCAAAAGUACUUGCGGAUUUUGCUUUACUUUUAAUGGCAAAAACCGCAAUUAUUUUUGCUUCAACCUAAUAUGAUGCAAAAUUGGCU chr11 70130159 rs191249039 U C 0.000199681 + 99

hsa-mir-548k CUUUUCUCAAGUAUUGCUGUUAGGUUGGUGCAAAAGUACUUGCGGAUUUUGCUUUACUUUUAAUGGCAAAAACCGCAAUUAUUUUUGCUUCAACCUAAUAUGAUGCAAAAUUGGCU chr11 70130164 . U C 0.000199681 + 104

hsa-mir-5481 UAUUAGGUUGGUGCAAAAGUAUUUGCGGGUUUUGUCGUAGAAAGUAAUGGCAAAAACUGCAGUUACUUGUGCACCAACCAAAUGCU chr11 94199735 rs143312616 U A 0.000199681 - 12

hsa-mir-5481 UAUUAGGUUGGUGCAAAAGUAUUUGCGGGUUUUGUCGUAGAAAGUAAUGGCAAAAACUGCAGUUACUUGUGCACCAACCAAAUGCU chr11 94199721 rs13447640 C U 0.0519169 - 26

hsa-mir-5481 UAUUAGGUUGGUGCAAAAGUAUUUGCGGGUUUUGUCGUAGAAAGUAAUGGCAAAAACUGCAGUUACUUGUGCACCAACCAAAUGCU chr11 94199711 rs148377174 C U 0.000199681 - 36

hsa-mir-5481 UAUUAGGUUGGUGCAAAAGUAUUUGCGGGUUUUGUCGUAGAAAGUAAUGGCAAAAACUGCAGUUACUUGUGCACCAACCAAAUGCU chr11 94199710 rs11020790 G A 0.0541134 - 37

hsa-mir-5481 UAUUAGGUUGGUGCAAAAGUAUUUGCGGGUUUUGUCGUAGAAAGUAAUGGCAAAAACUGCAGUUACUUGUGCACCAACCAAAUGCU chr11 94199696 rs191135297 C U 0.000399361 - 51

hsa-mir-5481 UAUUAGGUUGGUGCAAAAGUAUUUGCGGGUUUUGUCGUAGAAAGUAAUGGCAAAAACUGCAGUUACUUGUGCACCAACCAAAUGCU chr11 94199691 rs186423401 A G 0.000199681 - 56

hsa-mir-548m

AUAUUAGGUUGGUGCAAAGGUAUUUGUGGUUUUUGUCAUUAAAGUAAUGCAAAAGCCACAAAUACCUU
UGCACCAACCUAAUAUUA chrX 94318185 . A G 0.000264901 -
41
hsa-mir-548n
AGGUUGGUGCAAAAGUAAUUGUGGAUUUUGUCGUUAAAAAUAGCAAAACCCGCAAUUACUUUUGCACC
AACCUAA chr7 34980383 . G U 0.000199681 - 64

hsa-mir-548o
UGGUGAAAAUGUGUUGAUUGUAAUGGUUCCUAUUCUGAUCAAUAAACAUGGUUUGAGCCUAGUUACAA
UGAUCUAAAAUUCACGGUCCAAAACUGCAGUUACUUUUGCACCAAC chr7 102046296
rs185456116 A C 0.000199681 - 7

hsa-mir-548o
UGGUGAAAAUGUGUUGAUUGUAAUGGUUCCUAUUCUGAUCAAUAAACAUGGUUUGAGCCUAGUUACAA
UGAUCUAAAAUUCACGGUCCAAAACUGCAGUUACUUUUGCACCAAC chr7 102046276
rs145823581 U C 0.000399361 - 27

hsa-mir-548o
UGGUGAAAAUGUGUUGAUUGUAAUGGUUCCUAUUCUGAUCAAUAAACAUGGUUUGAGCCUAGUUACAA
UGAUCUAAAAUUCACGGUCCAAAACUGCAGUUACUUUUGCACCAAC chr7 102046262 .
A U 0.000199681 - 41
hsa-mir-548o
UGGUGAAAAUGUGUUGAUUGUAAUGGUUCCUAUUCUGAUCAAUAAACAUGGUUUGAGCCUAGUUACAA
UGAUCUAAAAUUCACGGUCCAAAACUGCAGUUACUUUUGCACCAAC chr7 102046237
rs117420576 C A 0.00239617 - 66

hsa-mir-548o
UGGUGAAAAUGUGUUGAUUGUAAUGGUUCCUAUUCUGAUCAAUAAACAUGGUUUGAGCCUAGUUACAA
UGAUCUAAAAUUCACGGUCCAAAACUGCAGUUACUUUUGCACCAAC chr7 102046219
rs141762298 G A 0.000399361 - 84

hsa-mir-548o
UGGUGAAAAUGUGUUGAUUGUAAUGGUUCCUAUUCUGAUCAAUAAACAUGGUUUGAGCCUAGUUACAA
UGAUCUAAAAUUCACGGUCCAAAACUGCAGUUACUUUUGCACCAAC chr7 102046217
rs181862774 U C 0.000798722 - 86

hsa-mir-548p
AUUAGGUUGGUAUAAAAUUAAUUGCAGUUUUUGUCAUUACUUUCAAUAGCAAAAACUGCAGUUACUUU
UGCACCAAUGUAAUAC chr5 100152259 . U C 0.00199681 -
11
hsa-mir-548p
AUUAGGUUGGUAUAAAAUUAAUUGCAGUUUUUGUCAUUACUUUCAAUAGCAAAAACUGCAGUUACUUU
UGCACCAAUGUAAUAC chr5 100152234 rs141552882 A G
0.000399361 - 36
hsa-mir-548p
AUUAGGUUGGUAUAAAAUUAAUUGCAGUUUUUGUCAUUACUUUCAAUAGCAAAAACUGCAGUUACUUU
UGCACCAAUGUAAUAC chr5 100152212 . G U 0.000599042 -

58
hsa-mir-548p
AUUAGGUUGGUAUAAAAUUAAUUGCAGUUUUUGUCAUUACUUUCAAUAGCAAAAACUGCAGUUACUUU
UGCACCAAUGUAAUAC chr5 100152201 . U G 0.000199681 -
69
hsa-mir-548t
AGGGUGGUGCAAAAGUGAUCGUGGUUUUUGCAAUUUUUUAAUGACAAAAACCACAAUUACUUUUGCAC
CAACCU chr4 174189331 rs185250675 G A 0.000599042 + 21

hsa-mir-548t
AGGGUGGUGCAAAAGUGAUCGUGGUUUUUGCAAUUUUUUAAUGACAAAAACCACAAUUACUUUUGCAC
CAACCU chr4 174189360 rs73872515 A C 0.0081869 + 50

hsa-mir-548t
AGGGUGGUGCAAAAGUGAUCGUGGUUUUUGCAAUUUUUUAAUGACAAAAACCACAAUUACUUUUGCAC
CAACCU chr4 174189381 . A G 0.00139776 + 71

hsa-mir-548u
AUUAGGAUGGUGCAAAAGUAAUGUGGUUUUUUUCUUUACUUUUAAUGGCAAAGACUGCAAUUACUUUU
GCGCCAACCUAAUchr6 57254939 rs4994089 G U null null 10

hsa-mir-548u
AUUAGGAUGGUGCAAAAGUAAUGUGGUUUUUUUCUUUACUUUUAAUGGCAAAGACUGCAAUUACUUUU
GCGCCAACCUAAUchr6 57254951 rs33917213 - U null null 22

hsa-mir-548u
AUUAGGAUGGUGCAAAAGUAAUGUGGUUUUUUUCUUUACUUUUAAUGGCAAAGACUGCAAUUACUUUU
GCGCCAACCUAAUchr6 57254952 rs67187462 - U null null 23

hsa-mir-548u
AUUAGGAUGGUGCAAAAGUAAUGUGGUUUUUUUCUUUACUUUUAAUGGCAAAGACUGCAAUUACUUUU
GCGCCAACCUAAUchr6 57254955 rs2894842 G A null null 26

hsa-mir-548u
AUUAGGAUGGUGCAAAAGUAAUGUGGUUUUUUUCUUUACUUUUAAUGGCAAAGACUGCAAUUACUUUU
GCGCCAACCUAAUchr6 57254986 rs2397267 G A null null 57

hsa-mir-548u
AUUAGGAUGGUGCAAAAGUAAUGUGGUUUUUUUCUUUACUUUUAAUGGCAAAGACUGCAAUUACUUUU
GCGCCAACCUAAUchr6 57255001 rs2894843 C A null null 72

hsa-mir-548v
AAUACUAGGUUUGAGCAAAAGUAAUUGCGGUUUUGCCAUCAUGCCAAAAGCUACAGUUACUUUUGCAC
CAGCCUAAUAUU chr8 17539162 . C U 0.000199681 - 5

hsa-mir-548w
GGUUGGUGCAAAAGUAACUGCGGUUUUUGCCUUUCAACAUAAUGGCAAAACCCACAAUUACUUUUGCA

CCAAUC chr16 26036570 . A G 0.000199681 + 13

hsa-mir-548x
AGGUUAGUGCAAAAGUAAUUGCAGUUUUUGCGUUACUUUCAAUCGUAAAAACUGCAAUUACUUUCACA
CCAAUCU chr21 20058441 . A G 0.000199681 - 42

hsa-mir-548x
AGGUUAGUGCAAAAGUAAUUGCAGUUUUUGCGUUACUUUCAAUCGUAAAAACUGCAAUUACUUUCACA
CCAAUCU chr21 20058423 . A G 0.000199681 - 60

hsa-mir-548x-2
AUGCCAAAUAUUAGGUUGGCACAAAAGUAAUUGUGGCUUUUGCCAUUAAAAGUAAUGGUAAAAACUGC
AAUUACUUUCGUGCCAACCUAAUAUUUGUGUG chr13 66540539 rs187794270 A G
0.000998403 - 23
hsa-mir-548x-2
AUGCCAAAUAUUAGGUUGGCACAAAAGUAAUUGUGGCUUUUGCCAUUAAAAGUAAUGGUAAAAACUGC
AAUUACUUUCGUGCCAACCUAAUAUUUGUGUG chr13 66540532 . A G
0.000399361 - 30
hsa-mir-548x-2
AUGCCAAAUAUUAGGUUGGCACAAAAGUAAUUGUGGCUUUUGCCAUUAAAAGUAAUGGUAAAAACUGC
AAUUACUUUCGUGCCAACCUAAUAUUUGUGUG chr13 66540526 . G C
0.000199681 - 36
hsa-mir-548x-2
AUGCCAAAUAUUAGGUUGGCACAAAAGUAAUUGUGGCUUUUGCCAUUAAAAGUAAUGGUAAAAACUGC
AAUUACUUUCGUGCCAACCUAAUAUUUGUGUG chr13 66540515 . U G
0.000399361 - 47
hsa-mir-548x-2
AUGCCAAAUAUUAGGUUGGCACAAAAGUAAUUGUGGCUUUUGCCAUUAAAAGUAAUGGUAAAAACUGC
AAUUACUUUCGUGCCAACCUAAUAUUUGUGUG chr13 66540506 . U C
0.000399361 - 56
hsa-mir-548x-2
AUGCCAAAUAUUAGGUUGGCACAAAAGUAAUUGUGGCUUUUGCCAUUAAAAGUAAUGGUAAAAACUGC
AAUUACUUUCGUGCCAACCUAAUAUUUGUGUG chr13 66540484 . C U
0.000199681 - 78
hsa-mir-548y
GCCUAAACUAUUAGGUUGGUGCAAAAGUAAUCACUGUUUUUGCCAUUACUCUCAGUGGCAAAAACCGU
GAUUACUUUUGCACCAACCUAGUAACACCUUCACUGUGGGGG chr14 48230294 . G
U 0.000199681 - 14
hsa-mir-548y
GCCUAAACUAUUAGGUUGGUGCAAAAGUAAUCACUGUUUUUGCCAUUACUCUCAGUGGCAAAAACCGU
GAUUACUUUUGCACCAACCUAGUAACACCUUCACUGUGGGGG chr14 48230259 . C
U 0.000399361 - 49
hsa-mir-548y
GCCUAAACUAUUAGGUUGGUGCAAAAGUAAUCACUGUUUUUGCCAUUACUCUCAGUGGCAAAAACCGU
GAUUACUUUUGCACCAACCUAGUAACACCUUCACUGUGGGGG chr14 48230257 . C
U 0.000599042 - 51
hsa-mir-548z

AAGUAUUAAGUUGGUGCAAAAGUAAUUGAGAUUUUUGCUACUGAAAGUAAUGGCAAAAACCGCAAUUACUUUUGCACCAACCUAAUAGAUGCCAAUG chr12 65016325 . C U 0.000199681 - 61

hsa-mir-548z AAGUAUUAAGUUGGUGCAAAAGUAAUUGAGAUUUUUGCUACUGAAAGUAAUGGCAAAAACCGCAAUUACUUUUGCACCAACCUAAUAGAUGCCAAUG chr12 65016300 rs17120527 U C 0.0625 - 86

hsa-mir-549a AGACAUGCAACUCAAGAAUAUAUUGAGAGCUCAUCCAUAGUUGUCACUGUCUCAAAUCAGUGACAACUAUGGAUGAGCUCUUAAUAUAUCCCAGGC chr15 81134346 rs141006148 A G 0.000399361 - 69

hsa-mir-549a AGACAUGCAACUCAAGAAUAUAUUGAGAGCUCAUCCAUAGUUGUCACUGUCUCAAAUCAGUGACAACUAUGGAUGAGCUCUUAAUAUAUCCCAGGC chr15 81134338 . G A 0.000399361 - 77

hsa-mir-550a-1 UGAUGCUUUGCUGGCUGGUGCAGUGCCUGAGGGAGUAAGAGCCCUGUUGUUGUAAGAUAGUGUCUUACUCCCUCAGGCACAUCUCCAACAAGUCUCU chr7 30329423 . G U 0.000199681 + 14

hsa-mir-550a-1 UGAUGCUUUGCUGGCUGGUGCAGUGCCUGAGGGAGUAAGAGCCCUGUUGUUGUAAGAUAGUGUCUUACUCCCUCAGGCACAUCUCCAACAAGUCUCU chr7 30329444 rs187080847 G A 0.000599042 + 35

hsa-mir-550a-1 UGAUGCUUUGCUGGCUGGUGCAGUGCCUGAGGGAGUAAGAGCCCUGUUGUUGUAAGAUAGUGUCUUACUCCCUCAGGCACAUCUCCAACAAGUCUCU chr7 30329451 rs74864340 C U null null 42

hsa-mir-550a-1 UGAUGCUUUGCUGGCUGGUGCAGUGCCUGAGGGAGUAAGAGCCCUGUUGUUGUAAGAUAGUGUCUUACUCCCUCAGGCACAUCUCCAACAAGUCUCU chr7 30329463 . A C 0.000199681 + 54

hsa-mir-550a-1 UGAUGCUUUGCUGGCUGGUGCAGUGCCUGAGGGAGUAAGAGCCCUGUUGUUGUAAGAUAGUGUCUUACUCCCUCAGGCACAUCUCCAACAAGUCUCU chr7 30329467 rs71528599 U C null null 58

hsa-mir-550a-2 UGAUGCUUUGCUGGCUGGUGCAGUGCCUGAGGGAGUAAGAGCCCUGUUGUUGUCAGAUAGUGUCUUACUCCCUCAGGCACAUCUCCAGCGAGUCUCU chr7 32772606 . G U 0.000199681 + 14

hsa-mir-550a-2 UGAUGCUUUGCUGGCUGGUGCAGUGCCUGAGGGAGUAAGAGCCCUGUUGUUGUCAGAUAGUGUCUUACUCCCUCAGGCACAUCUCCAGCGAGUCUCU chr7 32772637 rs113464681 UGU - null null 45

hsa-mir-550a-2 UGAUGCUUUGCUGGCUGGUGCAGUGCCUGAGGGAGUAAGAGCCCUGUUGUUGUCAGAUAGUGUCUUACUCCCUCAGGCACAUCUCCAGCGAGUCUCU chr7 32772678 . C U 0.000199681 + 86

hsa-mir-550a-2
UGAUGCUUUGCUGGCUGGUGCAGUGCCUGAGGGAGUAAGAGCCCUGUUGUUGUCAGAUAGUGUCUUAC
UCCCUCAGGCACAUCUCCAGCGAGUCUCU chr7 32772680 . G A
0.000599042 + 88
hsa-mir-550a-2
UGAUGCUUUGCUGGCUGGUGCAGUGCCUGAGGGAGUAAGAGCCCUGUUGUUGUCAGAUAGUGUCUUAC
UCCCUCAGGCACAUCUCCAGCGAGUCUCU chr7 32772682 . G A
0.000599042 + 90
hsa-mir-550a-3
GAUGCUUUGCUGGCUGGUGCAGUGCCUGAGGGAGUAAGAGUCCUGUUGUUGUAAGAUAGUGUCUUACU
CCCUCAGGCACAUCUCCAACAAGUCUC chr7 29720358 rs2696977 A G
0.0209665 - 87
hsa-mir-550b-1
AGAGACUUGUUGGAGAUGUGCCUGAGGGAGUAAGACACUAUCUUACAACAACAGGGCUCUUACUCCCU
CAGGCACUGCACCAGCCAGCAAAGCAUCA chr7 30329467 rs71528599 A G
null null 40
hsa-mir-550b-1
AGAGACUUGUUGGAGAUGUGCCUGAGGGAGUAAGACACUAUCUUACAACAACAGGGCUCUUACUCCCU
CAGGCACUGCACCAGCCAGCAAAGCAUCA chr7 30329463 . U G
0.000199681 - 44
hsa-mir-550b-1
AGAGACUUGUUGGAGAUGUGCCUGAGGGAGUAAGACACUAUCUUACAACAACAGGGCUCUUACUCCCU
CAGGCACUGCACCAGCCAGCAAAGCAUCA chr7 30329451 rs74864340 G A
null null 56
hsa-mir-550b-1
AGAGACUUGUUGGAGAUGUGCCUGAGGGAGUAAGACACUAUCUUACAACAACAGGGCUCUUACUCCCU
CAGGCACUGCACCAGCCAGCAAAGCAUCA chr7 30329444 rs187080847 C U
0.000599042 - 63
hsa-mir-550b-1
AGAGACUUGUUGGAGAUGUGCCUGAGGGAGUAAGACACUAUCUUACAACAACAGGGCUCUUACUCCCU
CAGGCACUGCACCAGCCAGCAAAGCAUCA chr7 30329423 . C A
0.000199681 - 84
hsa-mir-550b-2
AGAGACUCGCUGGAGAUGUGCCUGAGGGAGUAAGACACUAUCUGACAACAACAGGGCUCUUACUCCCU
CAGGCACUGCACCAGCCAGCAAAGCAUCA chr7 32772682 . C U
0.000599042 - 8
hsa-mir-550b-2
AGAGACUCGCUGGAGAUGUGCCUGAGGGAGUAAGACACUAUCUGACAACAACAGGGCUCUUACUCCCU
CAGGCACUGCACCAGCCAGCAAAGCAUCA chr7 32772680 . C U
0.000599042 - 10
hsa-mir-550b-2
AGAGACUCGCUGGAGAUGUGCCUGAGGGAGUAAGACACUAUCUGACAACAACAGGGCUCUUACUCCCU
CAGGCACUGCACCAGCCAGCAAAGCAUCA chr7 32772678 . G A
0.000199681 - 12
hsa-mir-550b-2
AGAGACUCGCUGGAGAUGUGCCUGAGGGAGUAAGACACUAUCUGACAACAACAGGGCUCUUACUCCCU
CAGGCACUGCACCAGCCAGCAAAGCAUCA chr7 32772637 rs113464681 ACA -

null null 53
hsa-mir-550b-2
AGAGACUCGCUGGAGAUGUGCCUGAGGGAGUAAGACACUAUCUGACAACAACAGGGCUCUUACUCCCU CAGGCACUGCACCAGCCAGCAAAGCAUCA chr7 32772606 . C A
0.000199681 - 84
hsa-mir-551a
GGGGACUGCCGGGUGACCCUGGAAAUCCAGAGUGGGUGGGGCCAGUCUGACCGUUUCUAGGCGACCCA CUCUUGGUUUCCAGGGUUGCCCUGGAAA chr1 3477292 rs187195064 G A
0.00599042 - 63
hsa-mir-551b
AGAUGUGCUCUCCUGGCCCAUGAAAUCAAGCGUGGGUGAGACCUGGUGCAGAACGGGAAGGCGACCCA UACUUGGUUUCAGAGGCUGUGAGAAUAA chr3 168269657 . G A
0.000199681 + 16
hsa-mir-552
AACCAUUCAAAUAUACCACAGUUUGUUUAACCUUUUGCCUGUUGGUUGAAGAUGCCUUUCAACAGGUG ACUGGUUAGACAAACUGUGGUAUAUACA chr1 35135286 . A G
0.000199681 - 10
hsa-mir-552
AACCAUUCAAAUAUACCACAGUUUGUUUAACCUUUUGCCUGUUGGUUGAAGAUGCCUUUCAACAGGUG ACUGGUUAGACAAACUGUGGUAUAUACA chr1 35135274 rs189946363 U C
0.000199681 - 22
hsa-mir-552
AACCAUUCAAAUAUACCACAGUUUGUUUAACCUUUUGCCUGUUGGUUGAAGAUGCCUUUCAACAGGUG ACUGGUUAGACAAACUGUGGUAUAUACA chr1 35135270 rs76067517 U C
0.00479233 - 26
hsa-mir-552
AACCAUUCAAAUAUACCACAGUUUGUUUAACCUUUUGCCUGUUGGUUGAAGAUGCCUUUCAACAGGUG ACUGGUUAGACAAACUGUGGUAUAUACA chr1 35135204 rs184377285 A G
0.000399361 - 92
hsa-mir-553
CUUCAAUUUUAUUUUAAAACGGUGAGAUUUUGUUUUGUCUGAGAAAAUCUCGCUGUUUUAGACUGAGG
chr1 100746814 rs190622705 A G 0.000998403 + 18

hsa-mir-553
CUUCAAUUUUAUUUUAAAACGGUGAGAUUUUGUUUUGUCUGAGAAAAUCUCGCUGUUUUAGACUGAGG
chr1 100746835 . C U 0.000199681 + 39

hsa-mir-553
CUUCAAUUUUAUUUUAAAACGGUGAGAUUUUGUUUUGUCUGAGAAAAUCUCGCUGUUUUAGACUGAGG
chr1 100746847 . C U 0.000199681 + 51

hsa-mir-553
CUUCAAUUUUAUUUUAAAACGGUGAGAUUUUGUUUUGUCUGAGAAAAUCUCGCUGUUUUAGACUGAGG
chr1 100746848 . G U 0.000199681 + 52

hsa-mir-553
CUUCAAUUUUAUUUUAAAACGGUGAGAUUUUGUUUUGUCUGAGAAAAUCUCGCUGUUUUAGACUGAGG

chr1 100746855 rs112891767 U C null null 59

hsa-mir-554
ACCUGAGUAACCUUUGCUAGUCCUGACUCAGCCAGUACUGGUCUUAGACUGGUGAUGGGUCAGGGUUC
AUAUUUUGGCAUCUCUCUCUGGGCAUCU chr1 151518284 rs79661940 U G
0.0225639 + 13
hsa-mir-554
ACCUGAGUAACCUUUGCUAGUCCUGACUCAGCCAGUACUGGUCUUAGACUGGUGAUGGGUCAGGGUUC
AUAUUUUGGCAUCUCUCUCUGGGCAUCU chr1 151518300 . C U
0.000199681 + 29
hsa-mir-554
ACCUGAGUAACCUUUGCUAGUCCUGACUCAGCCAGUACUGGUCUUAGACUGGUGAUGGGUCAGGGUUC
AUAUUUUGGCAUCUCUCUCUGGGCAUCU chr1 151518318 rs184748971 G A
0.000199681 + 47
hsa-mir-554
ACCUGAGUAACCUUUGCUAGUCCUGACUCAGCCAGUACUGGUCUUAGACUGGUGAUGGGUCAGGGUUC
AUAUUUUGGCAUCUCUCUCUGGGCAUCU chr1 151518322 rs189668852 G A
0.000399361 + 51
hsa-mir-555
GGAGUGAACUCAGAUGUGGAGCACUACCUUUGUGAGCAGUGUGACCCAAGGCCUGUGGACAGGGUAAG
CUGAACCUCUGAUAAAACUCUGAUCUAU chr1 155316214 rs113306711 A G
null null 23
hsa-mir-556
GAUAGUAAUAAGAAAGAUGAGCUCAUUGUAAUAUGAGCUUCAUUUAUACAUUUCAUAUUACCAUUAGC
UCAUCUUUUUUAUUACUACCUUCAACA chr1 162312411 . U C
0.000199681 + 76
hsa-mir-556
GAUAGUAAUAAGAAAGAUGAGCUCAUUGUAAUAUGAGCUUCAUUUAUACAUUUCAUAUUACCAUUAGC
UCAUCUUUUUUAUUACUACCUUCAACA chr1 162312417 . U A
0.000199681 + 82
hsa-mir-557
AGAAUGGGCAAAUGAACAGUAAAUUUGGAGGCCUGGGGCCCUCCCUGCUGCUGGAGAAGUGUUUGCAC
GGGUGGGCCUUGUCUUUGAAAGGAGGUGGA chr1 168344770 . C U
0.000199681 + 9
hsa-mir-557
AGAAUGGGCAAAUGAACAGUAAAUUUGGAGGCCUGGGGCCCUCCCUGCUGCUGGAGAAGUGUUUGCAC
GGGUGGGCCUUGUCUUUGAAAGGAGGUGGA chr1 168344829 rs78825966 C U
0.0557109 + 68
hsa-mir-558
GUGUGUGUGUGUGUGUGUGUGGUUAUUUUGGUAUAGUAGCUCUAGACUCUAUUAUAGUUUCCUGAGCUGC
UGUACCAAAAUACCACAAACGGGCUGchr2 32757230 rs72089144 - GUGU
null null 11
hsa-mir-558
GUGUGUGUGUGUGUGUGUGUGGUUAUUUUGGUAUAGUAGCUCUAGACUCUAUUAUAGUUUCCUGAGCUGC
UGUACCAAAAUACCACAAACGGGCUGchr2 32757231 rs71953654 - UGUGUGUGUG
null null 12
hsa-mir-558

GUGUGUGUGUGUGUGUGUGUGGUUAUUUUGGUAUAGUAGCUCUAGACUCUAUUAUAGUUUCCUGAGCUGC UGUACCAAAAUACCACAAACGGGCUGchr2 32757238 rs35999329 - UGUG null null 19

hsa-mir-558
GUGUGUGUGUGUGUGUGUGUGGUUAUUUUGGUAUAGUAGCUCUAGACUCUAUUAUAGUUUCCUGAGCUGC UGUACCAAAAUACCACAAACGGGCUGchr2 32757239 rs72215615 - UGUG null null 20

hsa-mir-558
GUGUGUGUGUGUGUGUGUGUGGUUAUUUUGGUAUAGUAGCUCUAGACUCUAUUAUAGUUUCCUGAGCUGC UGUACCAAAAUACCACAAACGGGCUGchr2 32757311 rs376848732 C U 0.000599042 + 92

hsa-mir-559
GCUCCAGUAACAUCUUAAAGUAAAUAUGCACCAAAAUUACUUUUGGUAAAUACAGUUUUGGUGCAUAU UUACUUUAGGAUGUUACUGGAGCUCCCA chr2 47604848 . A C 0.000199681 + 35

hsa-mir-559
GCUCCAGUAACAUCUUAAAGUAAAUAUGCACCAAAAUUACUUUUGGUAAAUACAGUUUUGGUGCAUAU UUACUUUAGGAUGUUACUGGAGCUCCCA chr2 47604856 rs114803590 U C 0.0119808 + 43

hsa-mir-559
GCUCCAGUAACAUCUUAAAGUAAAUAUGCACCAAAAUUACUUUUGGUAAAUACAGUUUUGGUGCAUAU UUACUUUAGGAUGUUACUGGAGCUCCCA chr2 47604865 rs372928176 A G 0.000199681 + 52

hsa-mir-559
GCUCCAGUAACAUCUUAAAGUAAAUAUGCACCAAAAUUACUUUUGGUAAAUACAGUUUUGGUGCAUAU UUACUUUAGGAUGUUACUGGAGCUCCCA chr2 47604866 rs58450758 C U 0.209065 + 53

hsa-mir-559
GCUCCAGUAACAUCUUAAAGUAAAUAUGCACCAAAAUUACUUUUGGUAAAUACAGUUUUGGUGCAUAU UUACUUUAGGAUGUUACUGGAGCUCCCA chr2 47604884 rs188620729 A G 0.000199681 + 71

hsa-mir-559
GCUCCAGUAACAUCUUAAAGUAAAUAUGCACCAAAAUUACUUUUGGUAAAUACAGUUUUGGUGCAUAU UUACUUUAGGAUGUUACUGGAGCUCCCA chr2 47604893 rs146825879 U C 0.000199681 + 80

hsa-mir-561
CUUCAUCCACCAGUCCUCCAGGAACAUCAAGGAUCUUAAACUUUGCCAGAGCUACAAAGGCAAAGUUU AAGAUCCUUGAAGUUCCUGGGGGAACCAU chr2 189162257 rs112456539 A G null null 39

hsa-mir-561
CUUCAUCCACCAGUCCUCCAGGAACAUCAAGGAUCUUAAACUUUGCCAGAGCUACAAAGGCAAAGUUU AAGAUCCUUGAAGUUCCUGGGGGAACCAU chr2 189162272 . A G 0.000199681 + 54

hsa-mir-561
CUUCAUCCACCAGUCCUCCAGGAACAUCAAGGAUCUUAAACUUUGCCAGAGCUACAAAGGCAAAGUUU AAGAUCCUUGAAGUUCCUGGGGGAACCAU chr2 189162305 rs184508542 G C 0.000199681 + 87

hsa-mir-561
CUUCAUCCACCAGUCCUCCAGGAACAUCAAGGAUCUUAAACUUUGCCAGAGCUACAAAGGCAAAGUUU AAGAUCCUUGAAGUUCCUGGGGGAACCAU chr2 189162311 . A G 0.000199681 + 93
hsa-mir-562
AGUGAAAUUGCUAGGUCAUAUGGUCAGUCUACUUUUAGAGUAAUUGUGAAACUGUUUUUCAAAGUAGC UGUACCAUUUGCACUCCCUGUGGCAAU chr2 233037428 . A G 0.000199681 + 66
hsa-mir-563
AGCAAAGAAGUGUGUUGCCCUCUAGGAAAUGUGUGUUGCUCUGAUGUAAUUAGGUUGACAUACGUUUC CCUGGUAGCCA chr3 15915297 . C U 0.000199681 + 20

hsa-mir-563
AGCAAAGAAGUGUGUUGCCCUCUAGGAAAUGUGUGUUGCUCUGAUGUAAUUAGGUUGACAUACGUUUC CCUGGUAGCCA chr3 15915306 rs145590767 A G 0.00159744 + 29
hsa-mir-563
AGCAAAGAAGUGUGUUGCCCUCUAGGAAAUGUGUGUUGCUCUGAUGUAAUUAGGUUGACAUACGUUUC CCUGGUAGCCA chr3 15915311 . U C 0.000199681 + 34

hsa-mir-563
AGCAAAGAAGUGUGUUGCCCUCUAGGAAAUGUGUGUUGCUCUGAUGUAAUUAGGUUGACAUACGUUUC CCUGGUAGCCA chr3 15915340 . C U 0.000199681 + 63

hsa-mir-563
AGCAAAGAAGUGUGUUGCCCUCUAGGAAAUGUGUGUUGCUCUGAUGUAAUUAGGUUGACAUACGUUUC CCUGGUAGCCA chr3 15915341 rs200447087 G A 0.000399361 + 64
hsa-mir-564
CGGGCAGCGGGUGCCAGGCACGGUGUCAGCAGGCAACAUGGCCGAGAGGCCGGGGCCUCCGGGCGGCG CCGUGUCCGCGACCGCGUACCCUGACchr3 44903385 rs114636202 A G 0.0239617 + 6
hsa-mir-564
CGGGCAGCGGGUGCCAGGCACGGUGUCAGCAGGCAACAUGGCCGAGAGGCCGGGGCCUCCGGGCGGCG CCGUGUCCGCGACCGCGUACCCUGACchr3 44903399 . A U 0.000199681 + 20
hsa-mir-564
CGGGCAGCGGGUGCCAGGCACGGUGUCAGCAGGCAACAUGGCCGAGAGGCCGGGGCCUCCGGGCGGCG CCGUGUCCGCGACCGCGUACCCUGACchr3 44903401 . G U 0.000399361 + 22
hsa-mir-564
CGGGCAGCGGGUGCCAGGCACGGUGUCAGCAGGCAACAUGGCCGAGAGGCCGGGGCCUCCGGGCGGCG CCGUGUCCGCGACCGCGUACCCUGACchr3 44903406 . C G 0.000199681 + 27
hsa-mir-564
CGGGCAGCGGGUGCCAGGCACGGUGUCAGCAGGCAACAUGGCCGAGAGGCCGGGGCCUCCGGGCGGCG CCGUGUCCGCGACCGCGUACCCUGACchr3 44903433 . G A

0.000199681 + 54
hsa-mir-564
CGGGCAGCGGGUGCCAGGCACGGUGUCAGCAGGCAACAUGGCCGAGAGGCCGGGGCCUCCGGGCGGCG
CCGUGUCCGCGACCGCGUACCCUGACchr3 44903434 rs2292181 G C
0.0545128 + 55
hsa-mir-566
GCUAGGCGUGGUGGCGGGCGCCUGUGAUCCCAACUACUCAGGAGGCUGGGGCAGCAGAAUCGCUUGAA
CCCGGGAGGCGAAGGUUGCAGUGAGCchr3 50210782 . G A
0.000199681 + 24
hsa-mir-566
GCUAGGCGUGGUGGCGGGCGCCUGUGAUCCCAACUACUCAGGAGGCUGGGGCAGCAGAAUCGCUUGAA
CCCGGGAGGCGAAGGUUGCAGUGAGCchr3 50210836 rs187238646 C U
0.000199681 + 78
hsa-mir-567
GGAUUCUUAUAGGACAGUAUGUUCUUCCAGGACAGAACAUUCUUUGCUAUUUUGUACUGGAAGAACAU
GCAAAACUAAAAAAAAAAAAAAGUUAUUGCU chr3 111831684 rs146499813 A C
0.000199681 + 37
hsa-mir-567
GGAUUCUUAUAGGACAGUAUGUUCUUCCAGGACAGAACAUUCUUUGCUAUUUUGUACUGGAAGAACAU
GCAAAACUAAAAAAAAAAAAAAGUUAUUGCU chr3 111831701 rs373126449 G A
0.000399361 + 54
hsa-mir-567
GGAUUCUUAUAGGACAGUAUGUUCUUCCAGGACAGAACAUUCUUUGCUAUUUUGUACUGGAAGAACAU
GCAAAACUAAAAAAAAAAAAAAGUUAUUGCU chr3 111831722 rs115707679 C A
null null 75
hsa-mir-567
GGAUUCUUAUAGGACAGUAUGUUCUUCCAGGACAGAACAUUCUUUGCUAUUUUGUACUGGAAGAACAU
GCAAAACUAAAAAAAAAAAAAAGUUAUUGCU chr3 111831724 rs67780840 - A
null null 77
hsa-mir-567
GGAUUCUUAUAGGACAGUAUGUUCUUCCAGGACAGAACAUUCUUUGCUAUUUUGUACUGGAAGAACAU
GCAAAACUAAAAAAAAAAAAAAGUUAUUGCU chr3 111831736 rs76778672 AA -
null null 89
hsa-mir-567
GGAUUCUUAUAGGACAGUAUGUUCUUCCAGGACAGAACAUUCUUUGCUAUUUUGUACUGGAAGAACAU
GCAAAACUAAAAAAAAAAAAAAGUUAUUGCU chr3 111831737 rs71704738 -
AAAA null null 90
hsa-mir-568
GAUAUACACUAUAUUAUGUAUAAAUGUAUACACACUUCCUAUAUGUAUCCACAUAUAUAUAGUGUAUA
UAUUAUACAUGUAUAGGUGUGUAUAUG chr3 114035388 rs149509568 U C
0.000399361 - 29
hsa-mir-568
GAUAUACACUAUAUUAUGUAUAAAUGUAUACACACUUCCUAUAUGUAUCCACAUAUAUAUAGUGUAUA
UAUUAUACAUGUAUAGGUGUGUAUAUG chr3 114035387 rs28632138 A C
null null 30
hsa-mir-569
GGUAUUGUUAGAUUAAUUUUGUGGGACAUUAACAACAGCAUCAGAAGCAACAUCAGCUUUAGUUAAUG

AAUCCUGGAAAGUUAAGUGACUUUAUUU chr3 170824459 rs116867000 U C
0.00119808 - 90
hsa-mir-569
GGUAUUGUUAGAUUAAUUUUGUGGGACAUUAACAACAGCAUCAGAAGCAACAUCAGCUUUAGUUAAUG
AAUCCUGGAAAGUUAAGUGACUUUAUUU chr3 170824453 rs73037390 U G
0.0215655 - 96
hsa-mir-570
CUAGAUAAGUUAUUAGGUGGGUGCAAAGGUAAUUGCAGUUUUUCCCAUUAUUUUAAUUGCGAAAACAG
CAAUUACCUUUGCACCAACCUGAUGGAGU chr3 195426295 rs373870815 C U
0.000199681 + 24
hsa-mir-570
CUAGAUAAGUUAUUAGGUGGGUGCAAAGGUAAUUGCAGUUUUUCCCAUUAUUUUAAUUGCGAAAACAG
CAAUUACCUUUGCACCAACCUGAUGGAGU chr3 195426299 . G A
0.000199681 + 28
hsa-mir-570
CUAGAUAAGUUAUUAGGUGGGUGCAAAGGUAAUUGCAGUUUUUCCCAUUAUUUUAAUUGCGAAAACAG
CAAUUACCUUUGCACCAACCUGAUGGAGU chr3 195426305 rs9860655 U C
0.0730831 + 34
hsa-mir-570
CUAGAUAAGUUAUUAGGUGGGUGCAAAGGUAAUUGCAGUUUUUCCCAUUAUUUUAAUUGCGAAAACAG
CAAUUACCUUUGCACCAACCUGAUGGAGU chr3 195426325 . U C
0.000399361 + 54
hsa-mir-570
CUAGAUAAGUUAUUAGGUGGGUGCAAAGGUAAUUGCAGUUUUUCCCAUUAUUUUAAUUGCGAAAACAG
CAAUUACCUUUGCACCAACCUGAUGGAGU chr3 195426331 . C U
0.000199681 + 60
hsa-mir-570
CUAGAUAAGUUAUUAGGUGGGUGCAAAGGUAAUUGCAGUUUUUCCCAUUAUUUUAAUUGCGAAAACAG
CAAUUACCUUUGCACCAACCUGAUGGAGU chr3 195426360 . U C
0.000199681 + 89
hsa-mir-571
CCUCAGUAAGACCAAGCUCAGUGUGCCAUUUCCUUGUCUGUAGCCAUGUCUAUGGGCUCUUGAGUUGG
CCAUCUGAGUGAGGGCCUGCUUAUUCUA chr4 343998 rs148718934 U C
0.000399361 + 53
hsa-mir-571
CCUCAGUAAGACCAAGCUCAGUGUGCCAUUUCCUUGUCUGUAGCCAUGUCUAUGGGCUCUUGAGUUGG
CCAUCUGAGUGAGGGCCUGCUUAUUCUA chr4 344032 . G C
0.000199681 + 87
hsa-mir-572
GUCGAGGCCGUGGCCCGGAAGUGGUCGGGGCCGCUGCGGGCGGAAGGGCGCCUGUGCUUCGUCCGCUC
GGCGGUGGCCCAGCCAGGCCCGCGGGA chr4 11370454 . G C
0.000399361 + 4
hsa-mir-572
GUCGAGGCCGUGGCCCGGAAGUGGUCGGGGCCGCUGCGGGCGGAAGGGCGCCUGUGCUUCGUCCGCUC
GGCGGUGGCCCAGCCAGGCCCGCGGGA chr4 11370463 . G A
0.000798722 + 13
hsa-mir-572

GUCGAGGCCGUGGCCCGGAAGUGGUCGGGGCCGCUGCGGGCGGAAGGGCGCCUGUGCUUCGUCCGCUC GGCGGUGGCCCAGCCAGGCCCGCGGGA chr4 11370488 . G A 0.000199681 + 38
hsa-mir-572
GUCGAGGCCGUGGCCCGGAAGUGGUCGGGGCCGCUGCGGGCGGAAGGGCGCCUGUGCUUCGUCCGCUC GGCGGUGGCCCAGCCAGGCCCGCGGGA chr4 11370489 . G A 0.000199681 + 39
hsa-mir-572
GUCGAGGCCGUGGCCCGGAAGUGGUCGGGGCCGCUGCGGGCGGAAGGGCGCCUGUGCUUCGUCCGCUC GGCGGUGGCCCAGCCAGGCCCGCGGGA chr4 11370514 . C U 0.000199681 + 64
hsa-mir-573
UUUAGCGGUUUCUCCCUGAAGUGAUGUGUAACUGAUCAGGAUCUACUCAUGUCGUCUUUGGUAAAGUU AUGUCGCUUGUCAGGGUGAGGAGAGUUUUUG chr4 24521910 rs77543406 A U null null 4
hsa-mir-573
UUUAGCGGUUUCUCCCUGAAGUGAUGUGUAACUGAUCAGGAUCUACUCAUGUCGUCUUUGGUAAAGUU AUGUCGCUUGUCAGGGUGAGGAGAGUUUUUG chr4 24521904 rs78830737 U C null null 10
hsa-mir-573
UUUAGCGGUUUCUCCCUGAAGUGAUGUGUAACUGAUCAGGAUCUACUCAUGUCGUCUUUGGUAAAGUU AUGUCGCUUGUCAGGGUGAGGAGAGUUUUUG chr4 24521902 rs76014664 C U null null 12
hsa-mir-573
UUUAGCGGUUUCUCCCUGAAGUGAUGUGUAACUGAUCAGGAUCUACUCAUGUCGUCUUUGGUAAAGUU AUGUCGCUUGUCAGGGUGAGGAGAGUUUUUG chr4 24521871 rs192259662 C U 0.000199681 - 43
hsa-mir-573
UUUAGCGGUUUCUCCCUGAAGUGAUGUGUAACUGAUCAGGAUCUACUCAUGUCGUCUUUGGUAAAGUU AUGUCGCUUGUCAGGGUGAGGAGAGUUUUUG chr4 24521822 rs113070835 A G null null 92
hsa-mir-574
GGGACCUGCGUGGGUGCGGGCGUGUGAGUGUGUGUGUGUGAGUGUGUGUCGCUCCGGGUCCACGCUCA UGCACACACCCACACGCCCACACUCAGG chr4 38869669 rs376863423 C U 0.000399361 + 17
hsa-mir-574
GGGACCUGCGUGGGUGCGGGCGUGUGAGUGUGUGUGUGUGAGUGUGUGUCGCUCCGGGUCCACGCUCA UGCACACACCCACACGCCCACACUCAGG chr4 38869703 . G A 0.000798722 + 51
hsa-mir-574
GGGACCUGCGUGGGUGCGGGCGUGUGAGUGUGUGUGUGUGAGUGUGUGUCGCUCCGGGUCCACGCUCA UGCACACACCCACACGCCCACACUCAGG chr4 38869731 rs371124579 C U 0.00119808 + 79
hsa-mir-574
GGGACCUGCGUGGGUGCGGGCGUGUGAGUGUGUGUGUGUGAGUGUGUGUCGCUCCGGGUCCACGCUCA UGCACACACCCACACGCCCACACUCAGG chr4 38869742 rs368340617 A G 0.000399361 + 90

hsa-mir-575
AAUUCAGCCCUGCCACUGGCUUAUGUCAUGACCUUGGGCUACUCAGGCUGUCUGCACAAUGAGCCAGU
UGGACAGGAGCAGUGCCACUCAACUCchr4 83674520 rs149186367 C U
0.00259585 - 64
hsa-mir-576
UACAAUCCAACGAGGAUUCUAAUUUCUCCACGUCUUUGGUAAUAAGGUUUGGCAAAGAUGUGGAAAAA
UUGGAAUCCUCAUUCGAUUGGUUAUAACCA chr4 110409864 . C U
0.000199681 + 11
hsa-mir-576
UACAAUCCAACGAGGAUUCUAAUUUCUCCACGUCUUUGGUAAUAAGGUUUGGCAAAGAUGUGGAAAAA
UUGGAAUCCUCAUUCGAUUGGUUAUAACCA chr4 110409867 rs185489298 G A
0.000998403 + 14
hsa-mir-576
UACAAUCCAACGAGGAUUCUAAUUUCUCCACGUCUUUGGUAAUAAGGUUUGGCAAAGAUGUGGAAAAA
UUGGAAUCCUCAUUCGAUUGGUUAUAACCA chr4 110409923 rs71603032 U G
null null 70
hsa-mir-576
UACAAUCCAACGAGGAUUCUAAUUUCUCCACGUCUUUGGUAAUAAGGUUUGGCAAAGAUGUGGAAAAA
UUGGAAUCCUCAUUCGAUUGGUUAUAACCA chr4 110409933 rs77639117 A U
0.0409345 + 80
hsa-mir-576
UACAAUCCAACGAGGAUUCUAAUUUCUCCACGUCUUUGGUAAUAAGGUUUGGCAAAGAUGUGGAAAAA
UUGGAAUCCUCAUUCGAUUGGUUAUAACCA chr4 110409936 . C U
0.000199681 + 83
hsa-mir-576
UACAAUCCAACGAGGAUUCUAAUUUCUCCACGUCUUUGGUAAUAAGGUUUGGCAAAGAUGUGGAAAAA
UUGGAAUCCUCAUUCGAUUGGUUAUAACCA chr4 110409938 . A G
0.000199681 + 85
hsa-mir-577
UGGGGGAGUGAAGAGUAGAUAAAAUAUUGGUACCUGAUGAAUCUGAGGCCAGGUUUCAAUACUUUAUC
UGCUCUUCAUUUCCCCAUAUCUACUUAC chr4 115577921 rs79560193 A G
null null 7
hsa-mir-577
UGGGGGAGUGAAGAGUAGAUAAAAUAUUGGUACCUGAUGAAUCUGAGGCCAGGUUUCAAUACUUUAUC
UGCUCUUCAUUUCCCCAUAUCUACUUAC chr4 115577996 rs189506651 C U
0.000998403 + 82
hsa-mir-577
UGGGGGAGUGAAGAGUAGAUAAAAUAUUGGUACCUGAUGAAUCUGAGGCCAGGUUUCAAUACUUUAUC
UGCUCUUCAUUUCCCCAUAUCUACUUAC chr4 115577997 rs34115976 C G
0.104034 + 83
hsa-mir-578
AGAUAAAUCUAUAGACAAAAUACAAUCCCGGACAACAAGAAGCUCCUAUAGCUCCUGUAGCUUCUUGU
GCUCUAGGAUUGUAUUUUGUUUAUAUAU chr4 166307424 rs200005096 G C
0.00359425 + 31
hsa-mir-578
AGAUAAAUCUAUAGACAAAAUACAAUCCCGGACAACAAGAAGCUCCUAUAGCUCCUGUAGCUUCUUGU
GCUCUAGGAUUGUAUUUUGUUUAUAUAU chr4 166307429 rs369159029 C G

0.000199681 + 36
hsa-mir-578
AGAUAAAUCUAUAGACAAAAUACAAUCCCGGACAACAAGAAGCUCCUAUAGCUCCUGUAGCUUCUUGU
GCUCUAGGAUUGUAUUUUGUUUAUAUAU chr4 166307447 rs200991991 C G
0.000199681 + 54
hsa-mir-579
CAUAUUAGGUUAAUGCAAAAGUAAUCGCGGUUUGUGCCAGAUGACGAUUUGAAUUAAUAAAUUCAUUU
GGUAUAAACCGCGAUUAUUUUUGCAUCAAC chr5 32394503 rs376231726 G A
0.00119808 - 79
hsa-mir-580
AUAAAAUUUCCAAUUGGAACCUAAUGAUUCAUCAGACUCAGAUAUUUAAGUUAACAGUAUUUGAGAAU
GAUGAAUCAUUAGGUUCCGGUCAGAAAUU chr5 36148058 . C U
0.000199681 - 33
hsa-mir-580
AUAAAAUUUCCAAUUGGAACCUAAUGAUUCAUCAGACUCAGAUAUUUAAGUUAACAGUAUUUGAGAAU
GAUGAAUCAUUAGGUUCCGGUCAGAAAUU chr5 36148057 rs115089112 A G
0.00978435 - 34
hsa-mir-580
AUAAAAUUUCCAAUUGGAACCUAAUGAUUCAUCAGACUCAGAUAUUUAAGUUAACAGUAUUUGAGAAU
GAUGAAUCAUUAGGUUCCGGUCAGAAAUU chr5 36148054 rs73080005 C A
0.000399361 - 37
hsa-mir-580
AUAAAAUUUCCAAUUGGAACCUAAUGAUUCAUCAGACUCAGAUAUUUAAGUUAACAGUAUUUGAGAAU
GAUGAAUCAUUAGGUUCCGGUCAGAAAUU chr5 36148005 . C U
0.000599042 - 86
hsa-mir-581
GUUAUGUGAAGGUAUUCUUGUGUUCUCUAGAUCAGUGCUUUUAGAAAAUUUGUGUGAUCUAAAGAACA
CAAAGAAUACCUACACAGAACCACCUGC chr5 53247400 rs810917 G A
null null 30
hsa-mir-581
GUUAUGUGAAGGUAUUCUUGUGUUCUCUAGAUCAGUGCUUUUAGAAAAUUUGUGUGAUCUAAAGAACA
CAAAGAAUACCUACACAGAACCACCUGC chr5 53247394 rs1694089 U G
null null 36
hsa-mir-581
GUUAUGUGAAGGUAUUCUUGUGUUCUCUAGAUCAGUGCUUUUAGAAAAUUUGUGUGAUCUAAAGAACA
CAAAGAAUACCUACACAGAACCACCUGC chr5 53247386 rs788517 G A
null null 44
hsa-mir-581
GUUAUGUGAAGGUAUUCUUGUGUUCUCUAGAUCAGUGCUUUUAGAAAAUUUGUGUGAUCUAAAGAACA
CAAAGAAUACCUACACAGAACCACCUGC chr5 53247360 rs375983171 A G
0.000199681 - 70
hsa-mir-581
GUUAUGUGAAGGUAUUCUUGUGUUCUCUAGAUCAGUGCUUUUAGAAAAUUUGUGUGAUCUAAAGAACA
CAAAGAAUACCUACACAGAACCACCUGC chr5 53247340 rs149830925 C G
0.000199681 - 90
hsa-mir-583
AACUCACACAUUAACCAAAGAGGAAGGUCCCAUUACUGCAGGGAUCUUAGCAGUACUGGGACCUACCU

CUUUGGU chr5 95414851 rs10697860 - AUAAA null null 10

hsa-mir-583
AACUCACACAUUAACCAAAGAGGAAGGUCCCAUUACUGCAGGGAUCUUAGCAGUACUGGGACCUACCU
CUUUGGU chr5 95414858 . A G 0.000599042 + 17

hsa-mir-583
AACUCACACAUUAACCAAAGAGGAAGGUCCCAUUACUGCAGGGAUCUUAGCAGUACUGGGACCUACCU
CUUUGGU chr5 95414863 . G A 0.000399361 + 22

hsa-mir-583
AACUCACACAUUAACCAAAGAGGAAGGUCCCAUUACUGCAGGGAUCUUAGCAGUACUGGGACCUACCU
CUUUGGU chr5 95414898 . U C 0.000199681 + 57

hsa-mir-583
AACUCACACAUUAACCAAAGAGGAAGGUCCCAUUACUGCAGGGAUCUUAGCAGUACUGGGACCUACCU
CUUUGGU chr5 95414909 rs184340760 U C 0.000599042 +
68
hsa-mir-584
UAGGGUGACCAGCCAUUAUGGUUUGCCUGGGACUGAGGAAUUUGCUGGGAUAUGUCAGUUCCAGGCCA
ACCAGGCUGGUUGGUCUCCCUGAAGCAAC chr5 148441924 rs375362381 G C
0.000199681 - 49
hsa-mir-584
UAGGGUGACCAGCCAUUAUGGUUUGCCUGGGACUGAGGAAUUUGCUGGGAUAUGUCAGUUCCAGGCCA
ACCAGGCUGGUUGGUCUCCCUGAAGCAAC chr5 148441899 . G A
0.000199681 - 74
hsa-mir-585
UGGGGUGUCUGUGCUAUGGCAGCCCUAGCACACAGAUACGCCCAGAGAAAGCCUGAACGUUGGGCGUA
UCUGUAUGCUAGGGCUGCUGUAACAAchr5 168690697 rs184601084 G A
0.000199681 - 2
hsa-mir-585
UGGGGUGUCUGUGCUAUGGCAGCCCUAGCACACAGAUACGCCCAGAGAAAGCCUGAACGUUGGGCGUA
UCUGUAUGCUAGGGCUGCUGUAACAAchr5 168690664 rs140379047 G C
0.00539137 - 35
hsa-mir-585
UGGGGUGUCUGUGCUAUGGCAGCCCUAGCACACAGAUACGCCCAGAGAAAGCCUGAACGUUGGGCGUA
UCUGUAUGCUAGGGCUGCUGUAACAAchr5 168690659 rs377680713 G U
0.000998403 - 40
hsa-mir-585
UGGGGUGUCUGUGCUAUGGCAGCCCUAGCACACAGAUACGCCCAGAGAAAGCCUGAACGUUGGGCGUA
UCUGUAUGCUAGGGCUGCUGUAACAAchr5 168690635 rs62376935 G A
0.0940495 - 64
hsa-mir-585
UGGGGUGUCUGUGCUAUGGCAGCCCUAGCACACAGAUACGCCCAGAGAAAGCCUGAACGUUGGGCGUA
UCUGUAUGCUAGGGCUGCUGUAACAAchr5 168690612 rs62376934 U C
0.808506 - 87
hsa-mir-585

UGGGGUGUCUGUGCUAUGGCAGCCCUAGCACACAGAUACGCCCAGAGAAAGCCUGAACGUUGGGCGUA
UCUGUAUGCUAGGGCUGCUGUAACAAchr5 168690611 rs115473017 G A
0.00858626 - 88
hsa-mir-586
AUGGGGUAAAACCAUUAUGCAUUGUAUUUUUAGGUCCCAAUACAUGUGGGCCCUAAAAAUACAAUGCA
UAAUGGUUUUUCACUCUUUAUCUUCUUAU chr6 45165499 . A G
0.000199681 - 9
hsa-mir-586
AUGGGGUAAAACCAUUAUGCAUUGUAUUUUUAGGUCCCAAUACAUGUGGGCCCUAAAAAUACAAUGCA
UAAUGGUUUUUCACUCUUUAUCUUCUUAU chr6 45165473 rs73735310 U C
0.00738818 - 35
hsa-mir-586
AUGGGGUAAAACCAUUAUGCAUUGUAUUUUUAGGUCCCAAUACAUGUGGGCCCUAAAAAUACAAUGCA
UAAUGGUUUUUCACUCUUUAUCUUCUUAU chr6 45165472 . C U
0.000199681 - 36
hsa-mir-587
CUCCUAUGCACCCUCUUUCCAUAGGUGAUGAGUCACAGGGCUCAGGGAAUGUGUCUGCACCUGUGACU
CAUCACCAGUGGAAAGCCCAUCCCAUAU chr6 107232039 rs373030429 G A
0.000399361 + 40
hsa-mir-587
CUCCUAUGCACCCUCUUUCCAUAGGUGAUGAGUCACAGGGCUCAGGGAAUGUGUCUGCACCUGUGACU
CAUCACCAGUGGAAAGCCCAUCCCAUAU chr6 107232080 . A C
0.000199681 + 81
hsa-mir-587
CUCCUAUGCACCCUCUUUCCAUAGGUGAUGAGUCACAGGGCUCAGGGAAUGUGUCUGCACCUGUGACU
CAUCACCAGUGGAAAGCCCAUCCCAUAU chr6 107232088 rs368413100 U C
0.000998403 + 89
hsa-mir-588
AGCUUAGGUACCAAUUUGGCCACAAUGGGUUAGAACACUAUUCCAUUGUGUUCUUACCCACCAUGGCC
AAAAUUGGGCCUAAG chr6 126805859 . G C 0.000399361 +
83
hsa-mir-589
UCCAGCCUGUGCCCAGCAGCCCCUGAGAACCACGUCUGCUCUGAGCUGGGUACUGCCUGUUCAGAACA
AAUGCCGGUUCCCAGACGCUGCCAGCUGGCC chr7 5535548 . U C
0.00279553 - 1
hsa-mir-589
UCCAGCCUGUGCCCAGCAGCCCCUGAGAACCACGUCUGCUCUGAGCUGGGUACUGCCUGUUCAGAACA
AAUGCCGGUUCCCAGACGCUGCCAGCUGGCC chr7 5535537 rs368361028 C U
0.000199681 - 12
hsa-mir-589
UCCAGCCUGUGCCCAGCAGCCCCUGAGAACCACGUCUGCUCUGAGCUGGGUACUGCCUGUUCAGAACA
AAUGCCGGUUCCCAGACGCUGCCAGCUGGCC chr7 5535527 . C U
0.000998403 - 22
hsa-mir-589
UCCAGCCUGUGCCCAGCAGCCCCUGAGAACCACGUCUGCUCUGAGCUGGGUACUGCCUGUUCAGAACA
AAUGCCGGUUCCCAGACGCUGCCAGCUGGCC chr7 5535475 rs375178432 C U
0.000199681 - 74

hsa-mir-589
UCCAGCCUGUGCCCAGCAGCCCCUGAGAACCACGUCUGCUCUGAGCUGGGUACUGCCUGUUCAGAACA AAUGCCGGUUCCCAGACGCUGCCAGCUGGCC chr7 5535469 rs372099307 C U 0.000998403 - 80
hsa-mir-589
UCCAGCCUGUGCCCAGCAGCCCCUGAGAACCACGUCUGCUCUGAGCUGGGUACUGCCUGUUCAGAACA AAUGCCGGUUCCCAGACGCUGCCAGCUGGCC chr7 5535464 rs368679861 C U 0.000798722 - 85
hsa-mir-590
UAGCCAGUCAGAAAUGAGCUUAUUCAUAAAAGUGCAGUAUGGUGAAGUCAAUCUGUAAUUUUAUGUAU AAGCUAGUCUCUGAUUGAAACAUGCAGCA chr7 73605546 rs189727189 C U 0.00159744 + 19
hsa-mir-590
UAGCCAGUCAGAAAUGAGCUUAUUCAUAAAAGUGCAGUAUGGUGAAGUCAAUCUGUAAUUUUAUGUAU AAGCUAGUCUCUGAUUGAAACAUGCAGCA chr7 73605551 rs192756765 U A 0.000199681 + 24
hsa-mir-590
UAGCCAGUCAGAAAUGAGCUUAUUCAUAAAAGUGCAGUAUGGUGAAGUCAAUCUGUAAUUUUAUGUAU AAGCUAGUCUCUGAUUGAAACAUGCAGCA chr7 73605578 rs147169000 A G 0.000998403 + 51
hsa-mir-590
UAGCCAGUCAGAAAUGAGCUUAUUCAUAAAAGUGCAGUAUGGUGAAGUCAAUCUGUAAUUUUAUGUAU AAGCUAGUCUCUGAUUGAAACAUGCAGCA chr7 73605599 rs6971711 C U 0.0261581 + 72
hsa-mir-590
UAGCCAGUCAGAAAUGAGCUUAUUCAUAAAAGUGCAGUAUGGUGAAGUCAAUCUGUAAUUUUAUGUAU AAGCUAGUCUCUGAUUGAAACAUGCAGCA chr7 73605603 . U G 0.000399361 + 76
hsa-mir-591
UCUUAUCAAUGAGGUAGACCAUGGGUUCUCAUUGUAAUAGUGUAGAAUGUUGGUUAACUGUGGACUCC CUGGCUCUGUCUCAAAUCUACUGAUUC chr7 95849010 rs189095137 U G 0.000399361 - 59
hsa-mir-592
UAUUAUGCCAUGACAUUGUGUCAAUAUGCGAUGAUGUGUUGUGAUGGCACAGCGUCAUCACGUGGUGA CGCAACAUCAUGACGUAAGACGUCACAAC chr7 126698158 rs181133328 A G 0.000199681 - 81
hsa-mir-592
UAUUAUGCCAUGACAUUGUGUCAAUAUGCGAUGAUGUGUUGUGAUGGCACAGCGUCAUCACGUGGUGA CGCAACAUCAUGACGUAAGACGUCACAAC chr7 126698143 rs139323492 A G 0.000199681 - 96
hsa-mir-592
UAUUAUGCCAUGACAUUGUGUCAAUAUGCGAUGAUGUGUUGUGAUGGCACAGCGUCAUCACGUGGUGA CGCAACAUCAUGACGUAAGACGUCACAAC chr7 126698142 rs375234833 C U 0.000199681 - 97
hsa-mir-593
CCCCCAGAAUCUGUCAGGCACCAGCCAGGCAUUGCUCAGCCCGUUUCCCUCUGGGGGAGCAAGGAGUG GUGCUGGGUUUGUCUCUGCUGGGGUUUCUCCU chr7 127721934 rs73721294 C U

0.0269569 + 22
hsa-mir-593
CCCCCAGAAUCUGUCAGGCACCAGCCAGGCAUUGCUCAGCCCGUUUCCCUCUGGGGGAGCAAGGAGUG
GUGCUGGGUUUGUCUCUGCUGGGGUUUCUCCU chr7 127721971 rs184901939 G U
0.000199681 + 59
hsa-mir-593
CCCCCAGAAUCUGUCAGGCACCAGCCAGGCAUUGCUCAGCCCGUUUCCCUCUGGGGGAGCAAGGAGUG
GUGCUGGGUUUGUCUCUGCUGGGGUUUCUCCU chr7 127721972 . C U
0.000199681 + 60
hsa-mir-593
CCCCCAGAAUCUGUCAGGCACCAGCCAGGCAUUGCUCAGCCCGUUUCCCUCUGGGGGAGCAAGGAGUG
GUGCUGGGUUUGUCUCUGCUGGGGUUUCUCCU chr7 127721986 rs372874678 G A
0.000399361 + 74
hsa-mir-595
ACGGAAGCCUGCACGCAUUUAACACCAGCACGCUCAAUGUAGUCUUGUAAGGAACAGGUUGAAGUGUG
CCGUGGUGUGUCUGGAGGAAGCGCCUGU chr7 158325503 rs4909237 G A
0.160543 - 3
hsa-mir-595
ACGGAAGCCUGCACGCAUUUAACACCAGCACGCUCAAUGUAGUCUUGUAAGGAACAGGUUGAAGUGUG
CCGUGGUGUGUCUGGAGGAAGCGCCUGU chr7 158325499 rs374577536 G U
0.000599042 - 7
hsa-mir-595
ACGGAAGCCUGCACGCAUUUAACACCAGCACGCUCAAUGUAGUCUUGUAAGGAACAGGUUGAAGUGUG
CCGUGGUGUGUCUGGAGGAAGCGCCUGU chr7 158325492 rs202194191 C U
0.000599042 - 14
hsa-mir-595
ACGGAAGCCUGCACGCAUUUAACACCAGCACGCUCAAUGUAGUCUUGUAAGGAACAGGUUGAAGUGUG
CCGUGGUGUGUCUGGAGGAAGCGCCUGU chr7 158325415 rs114151270 G A
0.0133786 - 91
hsa-mir-595
ACGGAAGCCUGCACGCAUUUAACACCAGCACGCUCAAUGUAGUCUUGUAAGGAACAGGUUGAAGUGUG
CCGUGGUGUGUCUGGAGGAAGCGCCUGU chr7 158325411 rs117344178 G A
0.00898562 - 95
hsa-mir-596
AGCACGGCCUCUCCGAAGCCUGCCCGGCUCCUCGGGAACCUGCCUCCCGCAUGGCAGCUGCUGCCCUU
CGGAGGCCG chr8 1765402 rs372395112 G U 0.000599042 +
6
hsa-mir-596
AGCACGGCCUCUCCGAAGCCUGCCCGGCUCCUCGGGAACCUGCCUCCCGCAUGGCAGCUGCUGCCCUU
CGGAGGCCG chr8 1765409 rs116450906 C U 0.0153754 +
13
hsa-mir-596
AGCACGGCCUCUCCGAAGCCUGCCCGGCUCCUCGGGAACCUGCCUCCCGCAUGGCAGCUGCUGCCCUU
CGGAGGCCG chr8 1765425 rs61388742 U C 0.0615016 +
29
hsa-mir-596
AGCACGGCCUCUCCGAAGCCUGCCCGGCUCCUCGGGAACCUGCCUCCCGCAUGGCAGCUGCUGCCCUU

CGGAGGCCG chr8 1765433 . A G 0.000199681 + 37

hsa-mir-596
AGCACGGCCUCUCCGAAGCCUGCCCGGCUCCUCGGGAACCUGCCUCCCGCAUGGCAGCUGCUGCCCUU
CGGAGGCCG chr8 1765435 rs111606748 C U null null 39

hsa-mir-596
AGCACGGCCUCUCCGAAGCCUGCCCGGCUCCUCGGGAACCUGCCUCCCGCAUGGCAGCUGCUGCCCUU
CGGAGGCCG chr8 1765461 rs375379855 C G 0.000199681 +
65
hsa-mir-597
UACUUACUCUACGUGUGUGUCACUCGAUGACCACUGUGAAGACAGUAAAAUGUACAGUGGUUCUCUUG
UGGCUCAAGCGUAAUGUAGAGUACUGGUC chr8 9599212 rs112056940 C U
0.00758786 + 31
hsa-mir-597
UACUUACUCUACGUGUGUGUCACUCGAUGACCACUGUGAAGACAGUAAAAUGUACAGUGGUUCUCUUG
UGGCUCAAGCGUAAUGUAGAGUACUGGUC chr8 9599253 rs374031731 C G
0.000199681 + 72
hsa-mir-597
UACUUACUCUACGUGUGUGUCACUCGAUGACCACUGUGAAGACAGUAAAAUGUACAGUGGUUCUCUUG
UGGCUCAAGCGUAAUGUAGAGUACUGGUC chr8 9599255 rs146125159 C U
0.000199681 + 74
hsa-mir-597
UACUUACUCUACGUGUGUGUCACUCGAUGACCACUGUGAAGACAGUAAAAUGUACAGUGGUUCUCUUG
UGGCUCAAGCGUAAUGUAGAGUACUGGUC chr8 9599259 rs368118876 C U
0.000399361 + 78
hsa-mir-597
UACUUACUCUACGUGUGUGUCACUCGAUGACCACUGUGAAGACAGUAAAAUGUACAGUGGUUCUCUUG
UGGCUCAAGCGUAAUGUAGAGUACUGGUC chr8 9599276 rs79397096 G A
0.00359425 + 95
hsa-mir-598
GCUUGAUGAUGCUGCUGAUGCUGGCGGUGAUCCCGAUGGUGUGAGCUGGAAAUGGGGUGCUACGUCAU
CGUUGUCAUCGUCAUCAUCAUCAUCCGAG chr8 10892785 . U C
0.000399361 - 28
hsa-mir-598
GCUUGAUGAUGCUGCUGAUGCUGGCGGUGAUCCCGAUGGUGUGAGCUGGAAAUGGGGUGCUACGUCAU
CGUUGUCAUCGUCAUCAUCAUCAUCCGAG chr8 10892767 . C A
0.000199681 - 46
hsa-mir-598
GCUUGAUGAUGCUGCUGAUGCUGGCGGUGAUCCCGAUGGUGUGAGCUGGAAAUGGGGUGCUACGUCAU
CGUUGUCAUCGUCAUCAUCAUCAUCCGAG chr8 10892749 . G A
0.000199681 - 64
hsa-mir-599
AAAGACAUGCUGUCCACAGUGUGUUUGAUAAGCUGACAUGGGACAGGGAUUCUUUUCACUGUUGUGUC
AGUUUAUCAAACCCAUACUUGGAUGAC chr8 100548917 rs186714401 G A
0.000199681 - 42
hsa-mir-600

AAGUCACGUGCUGUGGCUCCAGCUUCAUAGGAAGGCUCUUGUCUGUCAGGCAGUGGAGUUACUUACAG ACAAGAGCCUUGCUCAGGCCAGCCCUGCCC chr9 125873916 rs147291680 C U 0.00179712 - 7
hsa-mir-600
AAGUCACGUGCUGUGGCUCCAGCUUCAUAGGAAGGCUCUUGUCUGUCAGGCAGUGGAGUUACUUACAG ACAAGAGCCUUGCUCAGGCCAGCCCUGCCC chr9 125873833 . G A 0.000199681 - 90
hsa-mir-601
UGCAUGAGUUCGUCUUGGUCUAGGAUUGUUGGAGGAGUCAGAAAAACUACCCCAGGGAUCCUGAAGUC CUUUGGGUGGA chr9 126164871 rs184395895 G A 0.000199681 - 12
hsa-mir-602
UUCUCACCCCCGCCUGACACGGGCGACAGCUGCGGCCCGCUGUGUUCACUCGGGCCGAGUGCGUCUCC UGUCAGGCAAGGGAGAGCAGAGCCCCCCUG chr9 140732882 . G A 0.00219649 + 12
hsa-mir-602
UUCUCACCCCCGCCUGACACGGGCGACAGCUGCGGCCCGCUGUGUUCACUCGGGCCGAGUGCGUCUCC UGUCAGGCAAGGGAGAGCAGAGCCCCCCUG chr9 140732888 rs201175632 C U 0.00239617 + 18
hsa-mir-602
UUCUCACCCCCGCCUGACACGGGCGACAGCUGCGGCCCGCUGUGUUCACUCGGGCCGAGUGCGUCUCC UGUCAGGCAAGGGAGAGCAGAGCCCCCCUG chr9 140732890 rs202075878 C U 0.000199681 + 20
hsa-mir-602
UUCUCACCCCCGCCUGACACGGGCGACAGCUGCGGCCCGCUGUGUUCACUCGGGCCGAGUGCGUCUCC UGUCAGGCAAGGGAGAGCAGAGCCCCCCUG chr9 140732927 . G A 0.000199681 + 57
hsa-mir-602
UUCUCACCCCCGCCUGACACGGGCGACAGCUGCGGCCCGCUGUGUUCACUCGGGCCGAGUGCGUCUCC UGUCAGGCAAGGGAGAGCAGAGCCCCCCUG chr9 140732933 . G A 0.000199681 + 63
hsa-mir-602
UUCUCACCCCCGCCUGACACGGGCGACAGCUGCGGCCCGCUGUGUUCACUCGGGCCGAGUGCGUCUCC UGUCAGGCAAGGGAGAGCAGAGCCCCCCUG chr9 140732963 rs116636751 C U 0.00459265 + 93
hsa-mir-603
GAUUGAUGCUGUUGGUUUGGUGCAAAAGUAAUUGCAGUGCUUCCCAUUUAAAAGUAAUGGCACACACU GCAAUUACUUUUGCUCCAACUUAAUACUU chr10 24564632 rs79500031 G U null null 19
hsa-mir-603
GAUUGAUGCUGUUGGUUUGGUGCAAAAGUAAUUGCAGUGCUUCCCAUUUAAAAGUAAUGGCACACACU GCAAUUACUUUUGCUCCAACUUAAUACUU chr10 24564653 rs11014002 C U 0.0836661 + 40
hsa-mir-603
GAUUGAUGCUGUUGGUUUGGUGCAAAAGUAAUUGCAGUGCUUCCCAUUUAAAAGUAAUGGCACACACU GCAAUUACUUUUGCUCCAACUUAAUACUU chr10 24564674 . C G 0.000199681 + 61

hsa-mir-604
AGAGCAUCGUGCUUGACCUUCCACGCUCUCGUGUCCACUAGCAGGCAGGUUUUCUGACACAGGCUGCG
GAAUUCAGGACAGUGCAUCAUGGAGAchr10 29834018 rs376672879 G A
0.000199681 - 9
hsa-mir-604
AGAGCAUCGUGCUUGACCUUCCACGCUCUCGUGUCCACUAGCAGGCAGGUUUUCUGACACAGGCUGCG
GAAUUCAGGACAGUGCAUCAUGGAGAchr10 29834003 rs2368392 C U
0.323083 - 24
hsa-mir-604
AGAGCAUCGUGCUUGACCUUCCACGCUCUCGUGUCCACUAGCAGGCAGGUUUUCUGACACAGGCUGCG
GAAUUCAGGACAGUGCAUCAUGGAGAchr10 29833998 rs2368393 U C
0.329473 - 29
hsa-mir-604
AGAGCAUCGUGCUUGACCUUCCACGCUCUCGUGUCCACUAGCAGGCAGGUUUUCUGACACAGGCUGCG
GAAUUCAGGACAGUGCAUCAUGGAGAchr10 29833960 rs374251261 C U
0.000199681 - 67
hsa-mir-604
AGAGCAUCGUGCUUGACCUUCCACGCUCUCGUGUCCACUAGCAGGCAGGUUUUCUGACACAGGCUGCG
GAAUUCAGGACAGUGCAUCAUGGAGAchr10 29833959 rs186844507 G A
0.0297524 - 68
hsa-mir-604
AGAGCAUCGUGCUUGACCUUCCACGCUCUCGUGUCCACUAGCAGGCAGGUUUUCUGACACAGGCUGCG
GAAUUCAGGACAGUGCAUCAUGGAGAchr10 29833942 rs374132597 A G
0.000599042 - 85
hsa-mir-605
GCCCUAGCUUGGUUCUAAAUCCCAUGGUGCCUUCUCCUUGGGAAAAACAGAGAAGGCACUAUGAGAUU
UAGAAUCAAGUUAGG chr10 53059349 rs113212828 A G 0.00159744
+ 17
hsa-mir-605
GCCCUAGCUUGGUUCUAAAUCCCAUGGUGCCUUCUCCUUGGGAAAAACAGAGAAGGCACUAUGAGAUU
UAGAAUCAAGUUAGG chr10 53059356 rs76759855 A G null
null 24
hsa-mir-605
GCCCUAGCUUGGUUCUAAAUCCCAUGGUGCCUUCUCCUUGGGAAAAACAGAGAAGGCACUAUGAGAUU
UAGAAUCAAGUUAGG chr10 53059393 rs367648737 A G
0.000199681 + 61
hsa-mir-605
GCCCUAGCUUGGUUCUAAAUCCCAUGGUGCCUUCUCCUUGGGAAAAACAGAGAAGGCACUAUGAGAUU
UAGAAUCAAGUUAGG chr10 53059406 rs2043556 U C 0.259585
+ 74
hsa-mir-606
UGUAUCCUUGGUUUUUAGUAGUUUUACUAUGAUGAGGUGUGCCAUCCACCCCAUCAUAGUAAACUACU
GAAAAUCAAAGAUACAAGUGCCUGACCA chr10 77312216 . U A
0.000199681 + 1
hsa-mir-606
UGUAUCCUUGGUUUUUAGUAGUUUUACUAUGAUGAGGUGUGCCAUCCACCCCAUCAUAGUAAACUACU
GAAAAUCAAAGAUACAAGUGCCUGACCA chr10 77312284 rs34610391 - A

null null 69
hsa-mir-606
UGUAUCCUUGGUUUUUAGUAGUUUUACUAUGAUGAGGUGUGCCAUCCACCCCAUCAUAGUAAACUACU
GAAAAUCAAAGAUACAAGUGCCUGACCA chr10 77312301 . G A
0.000399361 + 86
hsa-mir-607
UUGCCUAAAGUCACACAGGUUAUAGAUCUGGAUUGGAACCCAGGGAGCCAGACUGCCUGGGUUCAAAU
CCAGAUCUAUAACUUGUGUGACUUUGGG chr10 98588497 . G C
0.000199681 - 25
hsa-mir-607
UUGCCUAAAGUCACACAGGUUAUAGAUCUGGAUUGGAACCCAGGGAGCCAGACUGCCUGGGUUCAAAU
CCAGAUCUAUAACUUGUGUGACUUUGGG chr10 98588496 rs12780546 A U
null null 26
hsa-mir-607
UUGCCUAAAGUCACACAGGUUAUAGAUCUGGAUUGGAACCCAGGGAGCCAGACUGCCUGGGUUCAAAU
CCAGAUCUAUAACUUGUGUGACUUUGGG chr10 98588495 rs12778876 U A
null null 27
hsa-mir-607
UUGCCUAAAGUCACACAGGUUAUAGAUCUGGAUUGGAACCCAGGGAGCCAGACUGCCUGGGUUCAAAU
CCAGAUCUAUAACUUGUGUGACUUUGGG chr10 98588480 rs368230098 A C
0.00159744 - 42
hsa-mir-607
UUGCCUAAAGUCACACAGGUUAUAGAUCUGGAUUGGAACCCAGGGAGCCAGACUGCCUGGGUUCAAAU
CCAGAUCUAUAACUUGUGUGACUUUGGG chr10 98588464 . U G
0.000199681 - 58
hsa-mir-607
UUGCCUAAAGUCACACAGGUUAUAGAUCUGGAUUGGAACCCAGGGAGCCAGACUGCCUGGGUUCAAAU
CCAGAUCUAUAACUUGUGUGACUUUGGG chr10 98588440 rs192456466 U C
0.00399361 - 82
hsa-mir-608
GGGCCAAGGUGGGCCAGGGGUGGUGUUGGGACAGCUCCGUUUAAAAAGGCAUCUCCAAGAGCUUCCAU
CAAAGGCUGCCUCUUGGUGCAGCACAGGUAGA chr10 102734749 . G C
0.000199681 + 8
hsa-mir-608
GGGCCAAGGUGGGCCAGGGGUGGUGUUGGGACAGCUCCGUUUAAAAAGGCAUCUCCAAGAGCUUCCAU
CAAAGGCUGCCUCUUGGUGCAGCACAGGUAGA chr10 102734771 . G U
0.000199681 + 30
hsa-mir-608
GGGCCAAGGUGGGCCAGGGGUGGUGUUGGGACAGCUCCGUUUAAAAAGGCAUCUCCAAGAGCUUCCAU
CAAAGGCUGCCUCUUGGUGCAGCACAGGUAGA chr10 102734778 rs4919510 C G
0.363818 + 37
hsa-mir-608
GGGCCAAGGUGGGCCAGGGGUGGUGUUGGGACAGCUCCGUUUAAAAAGGCAUCUCCAAGAGCUUCCAU
CAAAGGCUGCCUCUUGGUGCAGCACAGGUAGA chr10 102734811 . A G
0.000998403 + 70
hsa-mir-609
UGCUCGGCUGUUCCUAGGGUGUUUCUCUCAUCUCUGGUCUAUAAUGGGUUAAAUAGUAGAGAUGAGGG

CAACACCCUAGGAACAGCAGAGGAACC chr10 105978637 . C U
0.000998403 - 5
hsa-mir-609
UGCUCGGCUGUUCCUAGGGUGUUUCUCUCAUCUCUGGUCUAUAAUGGGUUAAAUAGUAGAGAUGAGGG
CAACACCCUAGGAACAGCAGAGGAACC chr10 105978601 rs201403671 A G
0.00319489 - 41
hsa-mir-609
UGCUCGGCUGUUCCUAGGGUGUUUCUCUCAUCUCUGGUCUAUAAUGGGUUAAAUAGUAGAGAUGAGGG
CAACACCCUAGGAACAGCAGAGGAACC chr10 105978597 . U C
0.000199681 - 45
hsa-mir-609
UGCUCGGCUGUUCCUAGGGUGUUUCUCUCAUCUCUGGUCUAUAAUGGGUUAAAUAGUAGAGAUGAGGG
CAACACCCUAGGAACAGCAGAGGAACC chr10 105978581 rs74154754 G C
0.00638978 - 61
hsa-mir-609
UGCUCGGCUGUUCCUAGGGUGUUUCUCUCAUCUCUGGUCUAUAAUGGGUUAAAUAGUAGAGAUGAGGG
CAACACCCUAGGAACAGCAGAGGAACC chr10 105978547 . C U
0.000199681 - 95
hsa-mir-610
UCUAUUUGUCUUAGGUGAGCUAAAUGUGUGCUGGGACACAUUUGAGCCAAAUGUCCCAGCACACAUUU
AGCUCACAUAAGAAAAAUGGACUCUAGU chr11 28078365 . A G
0.000199681 + 4
hsa-mir-610
UCUAUUUGUCUUAGGUGAGCUAAAUGUGUGCUGGGACACAUUUGAGCCAAAUGUCCCAGCACACAUUU
AGCUCACAUAAGAAAAAUGGACUCUAGU chr11 28078453 . C A
0.000399361 + 92
hsa-mir-610
UCUAUUUGUCUUAGGUGAGCUAAAUGUGUGCUGGGACACAUUUGAGCCAAAUGUCCCAGCACACAUUU
AGCUCACAUAAGAAAAAUGGACUCUAGU chr11 28078454 . U C
0.000199681 + 93
hsa-mir-611
AAAAUGGUGAGAGCGUUGAGGGGAGUUCCAGACGGAGAUGCGAGGACCCCUCGGGGUCUGACCCACA
chr11 61560031 rs144890118 A G 0.00359425 - 3

hsa-mir-611
AAAAUGGUGAGAGCGUUGAGGGGAGUUCCAGACGGAGAUGCGAGGACCCCUCGGGGUCUGACCCACA
chr11 61560026 rs111585800 U A null null 8

hsa-mir-611
AAAAUGGUGAGAGCGUUGAGGGGAGUUCCAGACGGAGAUGCGAGGACCCCUCGGGGUCUGACCCACA
chr11 61560021 rs200755503 G U 0.000199681 - 13

hsa-mir-611
AAAAUGGUGAGAGCGUUGAGGGGAGUUCCAGACGGAGAUGCGAGGACCCCUCGGGGUCUGACCCACA
chr11 61559991 . A G 0.000199681 - 43

hsa-mir-611

AAAAUGGUGAGAGCGUUGAGGGGAGUUCCAGACGGAGAUGCGAGGACCCCUCGGGGUCUGACCCACA chr11 61559981 rs187944546 G A 0.000199681 - 53

hsa-mir-612
UCCCAUCUGGACCCUGCUGGGCAGGGCUUCUGAGCUCCUUAGCACUAGCAGGAGGGGCUCCAGGGGCC CUCCCUCCAUGGCAGCCAGGACAGGACUCUCA chr11 65211940 rs550894 C A 0.215455 + 12
hsa-mir-612
UCCCAUCUGGACCCUGCUGGGCAGGGCUUCUGAGCUCCUUAGCACUAGCAGGAGGGGCUCCAGGGGCC CUCCCUCCAUGGCAGCCAGGACAGGACUCUCA chr11 65211979 rs12803915 G A 0.138179 + 51
hsa-mir-612
UCCCAUCUGGACCCUGCUGGGCAGGGCUUCUGAGCUCCUUAGCACUAGCAGGAGGGGCUCCAGGGGCC CUCCCUCCAUGGCAGCCAGGACAGGACUCUCA chr11 65211994 rs368138070 G U 0.000998403 + 66
hsa-mir-613
GGUGAGUGCGUUUCCAAGUGUGAAGGGACCCUUCCUGUAGUGUCUUAUAUACAAUACAGUAGGAAUGU UCCUUCUUUGCCACUCAUACACCUUUA chr12 12917583 rs373130703 G A 0.00119808 + 1
hsa-mir-613
GGUGAGUGCGUUUCCAAGUGUGAAGGGACCCUUCCUGUAGUGUCUUAUAUACAAUACAGUAGGAAUGU UCCUUCUUUGCCACUCAUACACCUUUA chr12 12917592 rs376973992 G A 0.000199681 + 10
hsa-mir-613
GGUGAGUGCGUUUCCAAGUGUGAAGGGACCCUUCCUGUAGUGUCUUAUAUACAAUACAGUAGGAAUGU UCCUUCUUUGCCACUCAUACACCUUUA chr12 12917608 rs147268651 G A 0.000199681 + 26
hsa-mir-613
GGUGAGUGCGUUUCCAAGUGUGAAGGGACCCUUCCUGUAGUGUCUUAUAUACAAUACAGUAGGAAUGU UCCUUCUUUGCCACUCAUACACCUUUA chr12 12917640 . A G 0.000199681 + 58
hsa-mir-613
GGUGAGUGCGUUUCCAAGUGUGAAGGGACCCUUCCUGUAGUGUCUUAUAUACAAUACAGUAGGAAUGU UCCUUCUUUGCCACUCAUACACCUUUA chr12 12917673 rs369614079 C G 0.000199681 + 91
hsa-mir-615
CUCGGGAGGGGCGGGAGGGGGGUCCCCGGUGCUCGGAUCUCGAGGGUGCUUAUUGUUCGGUCCGAGCC UGGGUCUCCCUCUUCCCCCCAACCCCCC chr12 54427753 . G U 0.00239617 + 20
hsa-mir-615
CUCGGGAGGGGCGGGAGGGGGGUCCCCGGUGCUCGGAUCUCGAGGGUGCUUAUUGUUCGGUCCGAGCC UGGGUCUCCCUCUUCCCCCCAACCCCCC chr12 54427779 . G A 0.000199681 + 46
hsa-mir-615
CUCGGGAGGGGCGGGAGGGGGGUCCCCGGUGCUCGGAUCUCGAGGGUGCUUAUUGUUCGGUCCGAGCC UGGGUCUCCCUCUUCCCCCCAACCCCCC chr12 54427797 . G A 0.000599042 + 64

hsa-mir-615
CUCGGGAGGGGCGGGAGGGGGGUCCCCGGUGCUCGGAUCUCGAGGGUGCUUAUUGUUCGGUCCGAGCC
UGGGUCUCCCUCUUCCCCCCAACCCCCC chr12 54427827 . C U
0.000399361 + 94
hsa-mir-616
UUAGGUAAUUCCUCCACUCAAAACCCUUCAGUGACUUCCAUGACAUGAAAUAGGAAGUCAUUGGAGGG
UUUGAGCAGAGGAAUGACCUGUUUUAAAA chr12 57912973 . U C
0.000199681 - 70
hsa-mir-617
CAUCAUAAGGAGCCUAGACUUCCCAUUUGAAGGUGGCCAUUUCCUACCACCUUCAAAUGGUAAGUCCA
GGCUCCUUCUGAUUCAAUAAAUGAGGAGC chr12 81226407 . A U
0.000798722 - 2
hsa-mir-617
CAUCAUAAGGAGCCUAGACUUCCCAUUUGAAGGUGGCCAUUUCCUACCACCUUCAAAUGGUAAGUCCA
GGCUCCUUCUGAUUCAAUAAAUGAGGAGC chr12 81226358 rs113739044 C U
null null 51
hsa-mir-617
CAUCAUAAGGAGCCUAGACUUCCCAUUUGAAGGUGGCCAUUUCCUACCACCUUCAAAUGGUAAGUCCA
GGCUCCUUCUGAUUCAAUAAAUGAGGAGC chr12 81226326 rs12815353 C G
null null 83
hsa-mir-618
CUCUUGUUCACAGCCAAACUCUACUUGUCCUUCUGAGUGUAAUUACGUACAUGCAGUAGCUCAGGAGA
CAAGCAGGUUUACCCUGUGGAUGAGUCUGA chr12 81329579 . U C
0.000199681 - 34
hsa-mir-618
CUCUUGUUCACAGCCAAACUCUACUUGUCCUUCUGAGUGUAAUUACGUACAUGCAGUAGCUCAGGAGA
CAAGCAGGUUUACCCUGUGGAUGAGUCUGA chr12 81329566 rs374241904 G A
0.000199681 - 47
hsa-mir-618
CUCUUGUUCACAGCCAAACUCUACUUGUCCUUCUGAGUGUAAUUACGUACAUGCAGUAGCUCAGGAGA
CAAGCAGGUUUACCCUGUGGAUGAGUCUGA chr12 81329536 rs2682818 U G
0.757588 - 77
hsa-mir-618
CUCUUGUUCACAGCCAAACUCUACUUGUCCUUCUGAGUGUAAUUACGUACAUGCAGUAGCUCAGGAGA
CAAGCAGGUUUACCCUGUGGAUGAGUCUGA chr12 81329527 rs145551269 U C
0.000199681 - 86
hsa-mir-619
CGCCCACCUCAGCCUCCCAAAAUGCUGGGAUUACAGGCAUGAGCCACUGCGGUCGACCAUGACCUGGA
CAUGUUUGUGCCCAGUACUGUCAGUUUGCAG chr12 109230732 rs34651680 - C
null null 51
hsa-mir-619
CGCCCACCUCAGCCUCCCAAAAUGCUGGGAUUACAGGCAUGAGCCACUGCGGUCGACCAUGACCUGGA
CAUGUUUGUGCCCAGUACUGUCAGUUUGCAG chr12 109230729 rs189214156 C U
0.000199681 - 54
hsa-mir-619
CGCCCACCUCAGCCUCCCAAAAUGCUGGGAUUACAGGCAUGAGCCACUGCGGUCGACCAUGACCUGGA
CAUGUUUGUGCCCAGUACUGUCAGUUUGCAG chr12 109230717 . G A

0.000199681 - 66
hsa-mir-619
CGCCCACCUCAGCCUCCCAAAAUGCUGGGAUUACAGGCAUGAGCCACUGCGGUCGACCAUGACCUGGA
CAUGUUUGUGCCCAGUACUGUCAGUUUGCAG chr12 109230697 . C U
0.000199681 - 86
hsa-mir-620
AUAUAUAUCUAUAUCUAGCUCCGUAUAUAUAUAUAUAUAUAUAUAGAUAUCUCCAUAUAUAUGGAGAU
AGAUAUAGAAAUAAAACAAGCAAAGAA chr12 116586436 rs34551929 U -
null null 24
hsa-mir-620
AUAUAUAUCUAUAUCUAGCUCCGUAUAUAUAUAUAUAUAUAUAUAGAUAUCUCCAUAUAUAUGGAGAU
AGAUAUAGAAAUAAAACAAGCAAAGAA chr12 116586436 rs34380284 C -
null null 24
hsa-mir-620
AUAUAUAUCUAUAUCUAGCUCCGUAUAUAUAUAUAUAUAUAUAUAGAUAUCUCCAUAUAUAUGGAGAU
AGAUAUAGAAAUAAAACAAGCAAAGAA chr12 116586426 rs5801168 AU -
null null 34
hsa-mir-620
AUAUAUAUCUAUAUCUAGCUCCGUAUAUAUAUAUAUAUAUAUAUAGAUAUCUCCAUAUAUAUGGAGAU
AGAUAUAGAAAUAAAACAAGCAAAGAA chr12 116586415 rs10549054 UA -
null null 45
hsa-mir-620
AUAUAUAUCUAUAUCUAGCUCCGUAUAUAUAUAUAUAUAUAUAUAGAUAUCUCCAUAUAUAUGGAGAU
AGAUAUAGAAAUAAAACAAGCAAAGAA chr12 116586414 rs77703604 G U
0.0113818 - 46
hsa-mir-620
AUAUAUAUCUAUAUCUAGCUCCGUAUAUAUAUAUAUAUAUAUAUAGAUAUCUCCAUAUAUAUGGAGAU
AGAUAUAGAAAUAAAACAAGCAAAGAA chr12 116586405 rs139328587 A G
0.000199681 - 55
hsa-mir-621
UAGAUUGAGGAAGGGGCUGAGUGGUAGGCGGUGCUGCUGUGCUCUGAUGAAGACCCAUGUGGCUAGCA
ACAGCGCUUACCUUUUGUCUCUGGGUCC chr13 41384930 . C U
0.000199681 + 29
hsa-mir-621
UAGAUUGAGGAAGGGGCUGAGUGGUAGGCGGUGCUGCUGUGCUCUGAUGAAGACCCAUGUGGCUAGCA
ACAGCGCUUACCUUUUGUCUCUGGGUCC chr13 41384931 rs188415420 G A
0.000399361 + 30
hsa-mir-621
UAGAUUGAGGAAGGGGCUGAGUGGUAGGCGGUGCUGCUGUGCUCUGAUGAAGACCCAUGUGGCUAGCA
ACAGCGCUUACCUUUUGUCUCUGGGUCC chr13 41384940 rs138641855 G A
0.00419329 + 39
hsa-mir-621
UAGAUUGAGGAAGGGGCUGAGUGGUAGGCGGUGCUGCUGUGCUCUGAUGAAGACCCAUGUGGCUAGCA
ACAGCGCUUACCUUUUGUCUCUGGGUCC chr13 41384974 . C U
0.000399361 + 73
hsa-mir-621
UAGAUUGAGGAAGGGGCUGAGUGGUAGGCGGUGCUGCUGUGCUCUGAUGAAGACCCAUGUGGCUAGCA

ACAGCGCUUACCUUUUGUCUCUGGGUCC chr13 41384997 rs181274589 C G
0.00419329 + 96
hsa-mir-622
AGAGAAGCUGGACAAGUACUGGUCUCAGCAGAUUGAGGAGAGCACCACAGUGGUCAUCACACAGUCUG
CUGAGGUUGGAGCUGCUGAGAUGACACU chr13 90883443 . C G
0.000199681 + 8
hsa-mir-622
AGAGAAGCUGGACAAGUACUGGUCUCAGCAGAUUGAGGAGAGCACCACAGUGGUCAUCACACAGUCUG
CUGAGGUUGGAGCUGCUGAGAUGACACU chr13 90883483 . C U 0
+ 48
hsa-mir-622
AGAGAAGCUGGACAAGUACUGGUCUCAGCAGAUUGAGGAGAGCACCACAGUGGUCAUCACACAGUCUG
CUGAGGUUGGAGCUGCUGAGAUGACACU chr13 90883502 . U G
0.000199681 + 67
hsa-mir-622
AGAGAAGCUGGACAAGUACUGGUCUCAGCAGAUUGAGGAGAGCACCACAGUGGUCAUCACACAGUCUG
CUGAGGUUGGAGCUGCUGAGAUGACACU chr13 90883517 rs111371406 U C
0.00279553 + 82
hsa-mir-623
GUACACAGUAGAAGCAUCCCUUGCAGGGGCUGUUGGGUUGCAUCCUAAGCUGUGCUGGAGCUUCCCGA
UGUACUCUGUAGAUGUCUUUGCACCUUCUG chr13 100008417 rs117579039 U G
null null 33
hsa-mir-623
GUACACAGUAGAAGCAUCCCUUGCAGGGGCUGUUGGGUUGCAUCCUAAGCUGUGCUGGAGCUUCCCGA
UGUACUCUGUAGAUGUCUUUGCACCUUCUG chr13 100008451 rs192917405 G U
0.000599042 + 67
hsa-mir-624
AAUGCUGUUUCAAGGUAGUACCAGUACCUUGUGUUCAGUGGAACCAAGGUAAACACAAGGUAUUGGUA
UUACCUUGAGAUAGCAUUACACCUAAGUG chr14 31483883 rs73251987 G C
0.0123802 - 66
hsa-mir-625
AGGGUAGAGGGAUGAGGGGGAAAGUUCUAUAGUCCUGUAAUUAGAUCUCAGGACUAUAGAACUUUCCC
CCUCAUCCCUCUGCCCU chr14 65937848 rs376464832 A G
0.000199681 + 29
hsa-mir-625
AGGGUAGAGGGAUGAGGGGGAAAGUUCUAUAGUCCUGUAAUUAGAUCUCAGGACUAUAGAACUUUCCC
CCUCAUCCCUCUGCCCU chr14 65937884 . U C 0.000199681 +
65
hsa-mir-625
AGGGUAGAGGGAUGAGGGGGAAAGUUCUAUAGUCCUGUAAUUAGAUCUCAGGACUAUAGAACUUUCCC
CCUCAUCCCUCUGCCCU chr14 65937889 rs12894182 C A null
null 70
hsa-mir-625
AGGGUAGAGGGAUGAGGGGGAAAGUUCUAUAGUCCUGUAAUUAGAUCUCAGGACUAUAGAACUUUCCC
CCUCAUCCCUCUGCCCU chr14 65937900 . G C 0.000199681 +
81
hsa-mir-626

ACUGAUAUAUUUGUCUUAUUUGAGAGCUGAGGAGUAUUUUAUGCAAUCUGAAUGAUCUCAGCUGUCU GAAAAUGUCUUCAAUUUUAAAGGCUUchr15 41983790 rs377390756 U C
0.000199681 + 8
hsa-mir-627
UACUUAUUACUGGUAGUGAGUCUCUAAGAAAAGAGGAGGUGGUUGUUUUCCUCCUCUUUUCUUUGAGA CUCACUACCAAUAAUAAGAAAUACUACUA chr15 42491856 rs371592505 A G
0.000998403 - 9
hsa-mir-627
UACUUAUUACUGGUAGUGAGUCUCUAAGAAAAGAGGAGGUGGUUGUUUUCCUCCUCUUUUCUUUGAGA CUCACUACCAAUAAUAAGAAAUACUACUA chr15 42491848 rs2620381 U G
0.0840655 - 17
hsa-mir-627
UACUUAUUACUGGUAGUGAGUCUCUAAGAAAAGAGGAGGUGGUUGUUUUCCUCCUCUUUUCUUUGAGA CUCACUACCAAUAAUAAGAAAUACUACUA chr15 42491832 rs76362052 G A
null null 33
hsa-mir-627
UACUUAUUACUGGUAGUGAGUCUCUAAGAAAAGAGGAGGUGGUUGUUUUCCUCCUCUUUUCUUUGAGA CUCACUACCAAUAAUAAGAAAUACUACUA chr15 42491820 . G A
0.000199681 - 45
hsa-mir-628
AUAGCUGUUGUGUCACUUCCUCAUGCUGACAUAUUUACUAGAGGGUAAAAUUAAUAACCUUCUAGUAA GAGUGGCAGUCGAAGGGAAGGGCUCAU chr15 55665200 rs200771662 A G
0.000199681 - 33
hsa-mir-628
AUAGCUGUUGUGUCACUUCCUCAUGCUGACAUAUUUACUAGAGGGUAAAAUUAAUAACCUUCUAGUAA GAGUGGCAGUCGAAGGGAAGGGCUCAU chr15 55665154 rs80185076 C G
null null 79
hsa-mir-628
AUAGCUGUUGUGUCACUUCCUCAUGCUGACAUAUUUACUAGAGGGUAAAAUUAAUAACCUUCUAGUAA GAGUGGCAGUCGAAGGGAAGGGCUCAU chr15 55665139 . A G
0.000199681 - 94
hsa-mir-629
UCCCUUUCCCAGGGGAGGGGCUGGGUUUACGUUGGGAGAACUUUUACGGUGAACCAGGAGGUUCUCCC AACGUAAGCCCAGCCCCUCCCCUCUGCCU chr15 70371794 . G A
0.000199681 - 14
hsa-mir-629
UCCCUUUCCCAGGGGAGGGGCUGGGUUUACGUUGGGAGAACUUUUACGGUGAACCAGGAGGUUCUCCC AACGUAAGCCCAGCCCCUCCCCUCUGCCU chr15 70371777 rs78212770 G C
0.0607029 - 31
hsa-mir-629
UCCCUUUCCCAGGGGAGGGGCUGGGUUUACGUUGGGAGAACUUUUACGGUGAACCAGGAGGUUCUCCC AACGUAAGCCCAGCCCCUCCCCUCUGCCU chr15 70371772 rs111899904 G A
0.00579073 - 36
hsa-mir-629
UCCCUUUCCCAGGGGAGGGGCUGGGUUUACGUUGGGAGAACUUUUACGGUGAACCAGGAGGUUCUCCC AACGUAAGCCCAGCCCCUCCCCUCUGCCU chr15 70371761 rs377691713 C U
0.000399361 - 47

hsa-mir-629
UCCCUUUCCCAGGGGAGGGGCUGGGUUUACGUUGGGAGAACUUUUACGGUGAACCAGGAGGUUCUCCC
AACGUAAGCCCAGCCCCUCCCCUCUGCCU chr15 70371740 . C G
0.000199681 - 68
hsa-mir-630
AACUUAACAUCAUGCUACCUCUUUGUAUCAUAUUUUGUUAUUCUGGUCACAGAAUGACCUAGUAUUCU
GUACCAGGGAAGGUAGUUCUUAACUAUAU chr15 72879630 . C U
0.000199681 + 73
hsa-mir-630
AACUUAACAUCAUGCUACCUCUUUGUAUCAUAUUUUGUUAUUCUGGUCACAGAAUGACCUAGUAUUCU
GUACCAGGGAAGGUAGUUCUUAACUAUAU chr15 72879649 . C G
0.000199681 + 92
hsa-mir-630
AACUUAACAUCAUGCUACCUCUUUGUAUCAUAUUUUGUUAUUCUGGUCACAGAAUGACCUAGUAUUCU
GUACCAGGGAAGGUAGUUCUUAACUAUAU chr15 72879653 rs113971639 A G
null null 96
hsa-mir-630
AACUUAACAUCAUGCUACCUCUUUGUAUCAUAUUUUGUUAUUCUGGUCACAGAAUGACCUAGUAUUCU
GUACCAGGGAAGGUAGUUCUUAACUAUAU chr15 72879654 rs67603382 - UUG
null null 97
hsa-mir-631
GUGGGGAGCCUGGUUAGACCUGGCCCAGACCUCAGCUACACAAGCUGAUGGACUGAGUCAGGGGCCAC
ACUCUCC chr15 75645967 rs5745925 GA - null null 60

hsa-mir-631
GUGGGGAGCCUGGUUAGACCUGGCCCAGACCUCAGCUACACAAGCUGAUGGACUGAGUCAGGGGCCAC
ACUCUCC chr15 75645962 . C U 0.000199681 - 65

hsa-mir-632
CGCCUCCUACCGCAGUGCUUGACGGGAGGCGGAGCGGGGAACGAGGCCGUCGGCCAUUUUGUGUCUGC
UUCCUGUGGGACGUGGUGGUAGCCGUchr17 30677169 rs371936677 C G
0.000199681 + 42
hsa-mir-632
CGCCUCCUACCGCAGUGCUUGACGGGAGGCGGAGCGGGGAACGAGGCCGUCGGCCAUUUUGUGUCUGC
UUCCUGUGGGACGUGGUGGUAGCCGUchr17 30677175 . C G
0.000199681 + 48
hsa-mir-632
CGCCUCCUACCGCAGUGCUUGACGGGAGGCGGAGCGGGGAACGAGGCCGUCGGCCAUUUUGUGUCUGC
UUCCUGUGGGACGUGGUGGUAGCCGUchr17 30677197 . U C
0.000199681 + 70
hsa-mir-632
CGCCUCCUACCGCAGUGCUUGACGGGAGGCGGAGCGGGGAACGAGGCCGUCGGCCAUUUUGUGUCUGC
UUCCUGUGGGACGUGGUGGUAGCCGUchr17 30677213 . G C
0.000199681 + 86
hsa-mir-633
AACCUCUCUUAGCCUCUGUUUCUUUAUUGCGGUAGAUACUAUUAACCUAAAAUGAGAAGGCUAAUAGU
AUCUACCACAAUAAAAUUGUUGUGAGGAUA chr17 61021604 rs75781970 G U

null null 29
hsa-mir-633
AACCUCUCUUAGCCUCUGUUUCUUUAUUGCGGUAGAUACUAUUAACCUAAAAUGAGAAGGCUAAUAGU
AUCUACCACAAUAAAAUUGUUGUGAGGAUA chr17 61021605 . C U
0.000199681 + 30
hsa-mir-633
AACCUCUCUUAGCCUCUGUUUCUUUAUUGCGGUAGAUACUAUUAACCUAAAAUGAGAAGGCUAAUAGU
AUCUACCACAAUAAAAUUGUUGUGAGGAUA chr17 61021611 rs17759989 A G
0.0233626 + 36
hsa-mir-633
AACCUCUCUUAGCCUCUGUUUCUUUAUUGCGGUAGAUACUAUUAACCUAAAAUGAGAAGGCUAAUAGU
AUCUACCACAAUAAAAUUGUUGUGAGGAUA chr17 61021625 rs181392999 A C
0.000199681 + 50
hsa-mir-633
AACCUCUCUUAGCCUCUGUUUCUUUAUUGCGGUAGAUACUAUUAACCUAAAAUGAGAAGGCUAAUAGU
AUCUACCACAAUAAAAUUGUUGUGAGGAUA chr17 61021673 . A U
0.000199681 + 98
hsa-mir-634
AAACCCACACCACUGCAUUUUGGCCAUCGAGGGUUGGGGCUUGGUGUCAUGCCCCAAGAUAACCAGCA
CCCCAACUUUGGACAGCAUGGAUUAGUCU chr17 64783222 . G U
0.000199681 + 33
hsa-mir-634
AAACCCACACCACUGCAUUUUGGCCAUCGAGGGUUGGGGCUUGGUGUCAUGCCCCAAGAUAACCAGCA
CCCCAACUUUGGACAGCAUGGAUUAGUCU chr17 64783240 . G A
0.000599042 + 51
hsa-mir-635
CAGAGAGGAGCUGCCACUUGGGCACUGAAACAAUGUCCAUUAGGCUUUGUUAUGGAAACUUCUCCUGA
UCAUUGUUUUGUGUCCAUUGAGCUUCCAAU chr17 66420605 rs192867879 A G
0.000199681 - 85
hsa-mir-635
CAGAGAGGAGCUGCCACUUGGGCACUGAAACAAUGUCCAUUAGGCUUUGUUAUGGAAACUUCUCCUGA
UCAUUGUUUUGUGUCCAUUGAGCUUCCAAU chr17 66420592 rs77279010 U C
0.0267572 - 98
hsa-mir-636
UGGCGGCCUGGGCGGGAGCGCGCGGGCGGGGCCGGCCCCGCUGCCUGGAAUUAACCCCGCUGUGCUUG
CUCGUCCCGCCCGCAGCCCUAGGCGGCGUCG chr17 74732624 rs377064670 C U
0.00119808 - 7
hsa-mir-636
UGGCGGCCUGGGCGGGAGCGCGCGGGCGGGGCCGGCCCCGCUGCCUGGAAUUAACCCCGCUGUGCUUG
CUCGUCCCGCCCGCAGCCCUAGGCGGCGUCG chr17 74732617 . G C
0.000199681 - 14
hsa-mir-636
UGGCGGCCUGGGCGGGAGCGCGCGGGCGGGGCCGGCCCCGCUGCCUGGAAUUAACCCCGCUGUGCUUG
CUCGUCCCGCCCGCAGCCCUAGGCGGCGUCG chr17 74732598 rs372808564 C U
0.00219649 - 33
hsa-mir-636
UGGCGGCCUGGGCGGGAGCGCGCGGGCGGGGCCGGCCCCGCUGCCUGGAAUUAACCCCGCUGUGCUUG

CUCGUCCCGCCCGCAGCCCUAGGCGGCGUCG chr17 74732595 . C U
0.000199681 - 36
hsa-mir-636
UGGCGGCCUGGGCGGGAGCGCGCGGGCGGGGCCGGCCCCGCUGCCUGGAAUUAACCCCGCUGUGCUUG
CUCGUCCCGCCCGCAGCCCUAGGCGGCGUCG chr17 74732560 . C G
0.000199681 - 71
hsa-mir-637
UGGCUAAGGUGUUGGCUCGGGCUCCCCACUGCAGUUACCCUCCCCUCGGCGUUACUGAGCACUGGGGG
CUUUCGGGCUCUGCGUCUGCACAGAUACUUC chr19 3961463 . G A
0.000199681 - 48
hsa-mir-637
UGGCUAAGGUGUUGGCUCGGGCUCCCCACUGCAGUUACCCUCCCCUCGGCGUUACUGAGCACUGGGGG
CUUUCGGGCUCUGCGUCUGCACAGAUACUUC chr19 3961438 rs202144891 C U
0.000599042 - 73
hsa-mir-637
UGGCUAAGGUGUUGGCUCGGGCUCCCCACUGCAGUUACCCUCCCCUCGGCGUUACUGAGCACUGGGGG
CUUUCGGGCUCUGCGUCUGCACAGAUACUUC chr19 3961435 . G A
0.000199681 - 76
hsa-mir-637
UGGCUAAGGUGUUGGCUCGGGCUCCCCACUGCAGUUACCCUCCCCUCGGCGUUACUGAGCACUGGGGG
CUUUCGGGCUCUGCGUCUGCACAGAUACUUC chr19 3961431 rs149472696 U C
0.00279553 - 80
hsa-mir-637
UGGCUAAGGUGUUGGCUCGGGCUCCCCACUGCAGUUACCCUCCCCUCGGCGUUACUGAGCACUGGGGG
CUUUCGGGCUCUGCGUCUGCACAGAUACUUC chr19 3961429 . C U
0.000199681 - 82
hsa-mir-638
GUGAGCGGGCGCGGCAGGGAUCGCGGGCGGGUGGCGGCCUAGGGCGCGGAGGGCGGACCGGGAAUGGC
GCGCCGUGCGCCGCCGGCGUAACUGCGGCGCU chr19 10829107 . C U
0.000199681 + 28
hsa-mir-638
GUGAGCGGGCGCGGCAGGGAUCGCGGGCGGGUGGCGGCCUAGGGCGCGGAGGGCGGACCGGGAAUGGC
GCGCCGUGCGCCGCCGGCGUAACUGCGGCGCU chr19 10829114 . C U
0.000199681 + 35
hsa-mir-638
GUGAGCGGGCGCGGCAGGGAUCGCGGGCGGGUGGCGGCCUAGGGCGCGGAGGGCGGACCGGGAAUGGC
GCGCCGUGCGCCGCCGGCGUAACUGCGGCGCU chr19 10829162 . C U
0.000199681 + 83
hsa-mir-638
GUGAGCGGGCGCGGCAGGGAUCGCGGGCGGGUGGCGGCCUAGGGCGCGGAGGGCGGACCGGGAAUGGC
GCGCCGUGCGCCGCCGGCGUAACUGCGGCGCU chr19 10829163 . G A
0.000199681 + 84
hsa-mir-639
UGGCCGACGGGGCGCGCGCGGCCUGGAGGGGCGGGGCGGACGCAGAGCCGCGUUUAGUCUAUCGCUGC
GGUUGCGAGCGCUGUAGGGAGCCUGUGCUG chr19 14640378 rs114121047 U C
0.0195687 + 24
hsa-mir-639

UGGCCGACGGGGCGCGCGCGGCCUGGAGGGGCGGGGCGGACGCAGAGCCGCGUUUAGUCUAUCGCUGC GGUUGCGAGCGCUGUAGGGAGCCUGUGCUG chr19 14640399 rs372602559 G C 0.0061901 + 45
hsa-mir-639 UGGCCGACGGGGCGCGCGCGGCCUGGAGGGGCGGGGCGGACGCAGAGCCGCGUUUAGUCUAUCGCUGC GGUUGCGAGCGCUGUAGGGAGCCUGUGCUG chr19 14640403 rs45556632 C G null null 49
hsa-mir-639 UGGCCGACGGGGCGCGCGCGGCCUGGAGGGGCGGGGCGGACGCAGAGCCGCGUUUAGUCUAUCGCUGC GGUUGCGAGCGCUGUAGGGAGCCUGUGCUG chr19 14640418 rs377544418 G C 0.000199681 + 64
hsa-mir-639 UGGCCGACGGGGCGCGCGCGGCCUGGAGGGGCGGGGCGGACGCAGAGCCGCGUUUAGUCUAUCGCUGC GGUUGCGAGCGCUGUAGGGAGCCUGUGCUG chr19 14640439 rs35149836 G A null null 85
hsa-mir-639 UGGCCGACGGGGCGCGCGCGGCCUGGAGGGGCGGGGCGGACGCAGAGCCGCGUUUAGUCUAUCGCUGC GGUUGCGAGCGCUGUAGGGAGCCUGUGCUG chr19 14640449 rs142882191 G A 0.000199681 + 95
hsa-mir-640 GUGACCCUGGGCAAGUUCCUGAAGAUCAGACACAUCAGAUCCCUUAUCUGUAAAAUGGGCAUGAUCCA GGAACCUGCCUCUACGGUUGCCUUGGGG chr19 19545876 . C U 0.000199681 + 5
hsa-mir-640 GUGACCCUGGGCAAGUUCCUGAAGAUCAGACACAUCAGAUCCCUUAUCUGUAAAAUGGGCAUGAUCCA GGAACCUGCCUCUACGGUUGCCUUGGGG chr19 19545895 . G U 0.000399361 + 24
hsa-mir-640 GUGACCCUGGGCAAGUUCCUGAAGAUCAGACACAUCAGAUCCCUUAUCUGUAAAAUGGGCAUGAUCCA GGAACCUGCCUCUACGGUUGCCUUGGGG chr19 19545931 . C U 0.000199681 + 60
hsa-mir-640 GUGACCCUGGGCAAGUUCCUGAAGAUCAGACACAUCAGAUCCCUUAUCUGUAAAAUGGGCAUGAUCCA GGAACCUGCCUCUACGGUUGCCUUGGGG chr19 19545932 rs111726405 A G null null 61
hsa-mir-641 UGGGUGAAAGGAAGGAAAGACAUAGGAUAGAGUCACCUCUGUCCUCUGUCCUCUACCUAUAGAGGUGA CUGUCCUAUGUCUUUCCUUCCUCUUACCCCU chr19 40788533 . A G 0.000399361 - 16
hsa-mir-641 UGGGUGAAAGGAAGGAAAGACAUAGGAUAGAGUCACCUCUGUCCUCUGUCCUCUACCUAUAGAGGUGA CUGUCCUAUGUCUUUCCUUCCUCUUACCCCU chr19 40788489 . U G 0.000199681 - 60
hsa-mir-641 UGGGUGAAAGGAAGGAAAGACAUAGGAUAGAGUCACCUCUGUCCUCUGUCCUCUACCUAUAGAGGUGA CUGUCCUAUGUCUUUCCUUCCUCUUACCCCU chr19 40788477 rs147213560 U G 0.000798722 - 72

hsa-mir-642a
AUCUGAGUUGGGAGGGUCCCUCUCCAAAUGUGUCUUGGGGUGGGGGAUCAAGACACAUUUGGAGAGGG
AACCUCCCAACUCGGCCUCUGCCAUCAUU chr19 46178206 rs78902025 U G
0.000199681 + 21
hsa-mir-642a
AUCUGAGUUGGGAGGGUCCCUCUCCAAAUGUGUCUUGGGGUGGGGGAUCAAGACACAUUUGGAGAGGG
AACCUCCCAACUCGGCCUCUGCCAUCAUU chr19 46178217 rs111664333 G A
null null 32
hsa-mir-642a
AUCUGAGUUGGGAGGGUCCCUCUCCAAAUGUGUCUUGGGGUGGGGGAUCAAGACACAUUUGGAGAGGG
AACCUCCCAACUCGGCCUCUGCCAUCAUU chr19 46178238 rs200255173 A G
0.000199681 + 53
hsa-mir-642a
AUCUGAGUUGGGAGGGUCCCUCUCCAAAUGUGUCUUGGGGUGGGGGAUCAAGACACAUUUGGAGAGGG
AACCUCCCAACUCGGCCUCUGCCAUCAUU chr19 46178255 rs377713506 A C
0.00419329 + 70
hsa-mir-642a
AUCUGAGUUGGGAGGGUCCCUCUCCAAAUGUGUCUUGGGGUGGGGGAUCAAGACACAUUUGGAGAGGG
AACCUCCCAACUCGGCCUCUGCCAUCAUU chr19 46178267 . G A
0.000399361 + 82
hsa-mir-642a
AUCUGAGUUGGGAGGGUCCCUCUCCAAAUGUGUCUUGGGGUGGGGGAUCAAGACACAUUUGGAGAGGG
AACCUCCCAACUCGGCCUCUGCCAUCAUU chr19 46178268 . G A
0.000199681 + 83
hsa-mir-642b
GAGUUGGGAGGUUCCCUCUCCAAAUGUGUCUUGAUCCCCCACCCCAAGACACAUUUGGAGAGGGACCC
UCCCAACUC chr19 46178255 rs377713506 U G 0.00419329 -
12
hsa-mir-642b
GAGUUGGGAGGUUCCCUCUCCAAAUGUGUCUUGAUCCCCCACCCCAAGACACAUUUGGAGAGGGACCC
UCCCAACUC chr19 46178238 rs200255173 U C 0.000199681 -
29
hsa-mir-642b
GAGUUGGGAGGUUCCCUCUCCAAAUGUGUCUUGAUCCCCCACCCCAAGACACAUUUGGAGAGGGACCC
UCCCAACUC chr19 46178217 rs111664333 C U null null 50

hsa-mir-642b
GAGUUGGGAGGUUCCCUCUCCAAAUGUGUCUUGAUCCCCCACCCCAAGACACAUUUGGAGAGGGACCC
UCCCAACUC chr19 46178206 rs78902025 A C 0.000199681 -
61
hsa-mir-643
ACCAAGUGAUAUUCAUUGUCUACCUGAGCUAGAAUACAAGUAGUUGGCGUCUUCAGAGACACUUGUAU
GCUAGCUCAGGUAGAUAUUGAAUGAAAAA chr19 52785062 . U A
0.000199681 + 13
hsa-mir-644a
UUUUUUUUUAGUAUUUUUCCAUCAGUGUUCAUAAGGAAUGUUGCUCUGUAGUUUUCUUAUAGUGUGGC
UUUCUUAGAGCAAAGAUGGUUCCCUAchr20 33054160 rs181082862 A G

0.000199681	+	31
hsa-mir-644a
UUUUUUUUUAGUAUUUUUCCAUCAGUGUUCAUAAGGAAUGUUGCUCUGUAGUUUUCUUAUAGUGUGGC
UUUCUUAGAGCAAAGAUGGUUCCCUAchr20	33054163	rs186999403	A	C
0.000199681	+	34
hsa-mir-644a
UUUUUUUUUAGUAUUUUUCCAUCAGUGUUCAUAAGGAAUGUUGCUCUGUAGUUUUCUUAUAGUGUGGC
UUUCUUAGAGCAAAGAUGGUUCCCUAchr20	33054187	.	U	C
0.000199681	+	58
hsa-mir-644a
UUUUUUUUUAGUAUUUUUCCAUCAGUGUUCAUAAGGAAUGUUGCUCUGUAGUUUUCUUAUAGUGUGGC
UUUCUUAGAGCAAAGAUGGUUCCCUAchr20	33054223	rs191742048	A	C
0.000798722	+	94
hsa-mir-645
CAGUUCCUAACAGGCCUCAGACCAGUACCGGUCUGUGGCCUGGGGGUUGAGGACCCCUGCUCUAGGCU
GGUACUGCUGAUGCUUAAAAAGAGAGchr20	49202351	rs111893316	C	U
0.000199681	+	29
hsa-mir-645
CAGUUCCUAACAGGCCUCAGACCAGUACCGGUCUGUGGCCUGGGGGUUGAGGACCCCUGCUCUAGGCU
GGUACUGCUGAUGCUUAAAAAGAGAGchr20	49202354	rs35645123	-	A
null	null	32
hsa-mir-645
CAGUUCCUAACAGGCCUCAGACCAGUACCGGUCUGUGGCCUGGGGGUUGAGGACCCCUGCUCUAGGCU
GGUACUGCUGAUGCUUAAAAAGAGAGchr20	49202375	.	A	G
0.000599042	+	53
hsa-mir-646
GAUCAGGAGUCUGCCAGUGGAGUCAGCACACCUGCUUUUCACCUGUGAUCCCAGGAGAGGAAGCAGCU
GCCUCUGAGGCCUCAGGCUCAGUGGCchr20	58883534	rs6513496	U	C
0.272764	+	3
hsa-mir-646
GAUCAGGAGUCUGCCAGUGGAGUCAGCACACCUGCUUUUCACCUGUGAUCCCAGGAGAGGAAGCAGCU
GCCUCUGAGGCCUCAGGCUCAGUGGCchr20	58883553	.	G	A
0.000199681	+	22
hsa-mir-646
GAUCAGGAGUCUGCCAGUGGAGUCAGCACACCUGCUUUUCACCUGUGAUCCCAGGAGAGGAAGCAGCU
GCCUCUGAGGCCUCAGGCUCAGUGGCchr20	58883601	rs112880289	C	U
null	null	70
hsa-mir-646
GAUCAGGAGUCUGCCAGUGGAGUCAGCACACCUGCUUUUCACCUGUGAUCCCAGGAGAGGAAGCAGCU
GCCUCUGAGGCCUCAGGCUCAGUGGCchr20	58883602	.	C	G
0.000399361	+	71
hsa-mir-646
GAUCAGGAGUCUGCCAGUGGAGUCAGCACACCUGCUUUUCACCUGUGAUCCCAGGAGAGGAAGCAGCU
GCCUCUGAGGCCUCAGGCUCAGUGGCchr20	58883605	rs6513497	U	G
0.190895	+	74
hsa-mir-646
GAUCAGGAGUCUGCCAGUGGAGUCAGCACACCUGCUUUUCACCUGUGAUCCCAGGAGAGGAAGCAGCU

GCCUCUGAGGCCUCAGGCUCAGUGGCchr20	58883611	.	C	U
0.000199681	+	80
hsa-mir-647
AGGAAGUGUUGGCCUGUGGCUGCACUCACUUCCUUCAGCCCCAGGAAGCCUUGGUCGGGGGCAGGAGG
GAGGGUCAGGCAGGGCUGGGGGCCUGAC	chr20	62574021	rs367570577	G	C
0.00319489	-	59
hsa-mir-647
AGGAAGUGUUGGCCUGUGGCUGCACUCACUUCCUUCAGCCCCAGGAAGCCUUGGUCGGGGGCAGGAGG
GAGGGUCAGGCAGGGCUGGGGGCCUGAC	chr20	62574016	rs191451021	G	A
0.00299521	-	64
hsa-mir-647
AGGAAGUGUUGGCCUGUGGCUGCACUCACUUCCUUCAGCCCCAGGAAGCCUUGGUCGGGGGCAGGAGG
GAGGGUCAGGCAGGGCUGGGGGCCUGAC	chr20	62574006	rs73147065	U	C
0.230631	-	74
hsa-mir-647
AGGAAGUGUUGGCCUGUGGCUGCACUCACUUCCUUCAGCCCCAGGAAGCCUUGGUCGGGGGCAGGAGG
GAGGGUCAGGCAGGGCUGGGGGCCUGAC	chr20	62573997	.	G	A
0.000199681	-	83
hsa-mir-648
AUCACAGACACCUCCAAGUGUGCAGGGCACUGGUGGGGGCCGGGGCAGGCCCAGCGAAAGUGCAGGAC
CUGGCACUUAGUCGGAAGUGAGGGUGchr22	18463709	.	U	C
0.000199681	-	19
hsa-mir-648
AUCACAGACACCUCCAAGUGUGCAGGGCACUGGUGGGGGCCGGGGCAGGCCCAGCGAAAGUGCAGGAC
CUGGCACUUAGUCGGAAGUGAGGGUGchr22	18463706	rs377373465	G	A
0.000199681	-	22
hsa-mir-648
AUCACAGACACCUCCAAGUGUGCAGGGCACUGGUGGGGGCCGGGGCAGGCCCAGCGAAAGUGCAGGAC
CUGGCACUUAGUCGGAAGUGAGGGUGchr22	18463686	.	G	A
0.000199681	-	42
hsa-mir-648
AUCACAGACACCUCCAAGUGUGCAGGGCACUGGUGGGGGCCGGGGCAGGCCCAGCGAAAGUGCAGGAC
CUGGCACUUAGUCGGAAGUGAGGGUGchr22	18463663	.	G	A
0.000599042	-	65
hsa-mir-648
AUCACAGACACCUCCAAGUGUGCAGGGCACUGGUGGGGGCCGGGGCAGGCCCAGCGAAAGUGCAGGAC
CUGGCACUUAGUCGGAAGUGAGGGUGchr22	18463645	.	G	C
0.000199681	-	83
hsa-mir-648
AUCACAGACACCUCCAAGUGUGCAGGGCACUGGUGGGGGCCGGGGCAGGCCCAGCGAAAGUGCAGGAC
CUGGCACUUAGUCGGAAGUGAGGGUGchr22	18463636	.	G	C	0.00159744
-	92
hsa-mir-649
GGCCUAGCCAAAUACUGUAUUUUUGAUCGACAUUUGGUUGAAAAAUAUCUAUGUAUUAGUAAACCUGU
GUUGUUCAAGAGUCCACUGUGUUUUGCUG	chr22	21388533	.	G	A
0.000199681	-	29
hsa-mir-649

GGCCUAGCCAAAUACUGUAUUUUUGAUCGACAUUUGGUUGAAAAAUAUCUAUGUAUUAGUAAACCUGU
GUUGUUCAAGAGUCCACUGUGUUUUGCUG chr22 21388510 rs183799425 U A
0.000399361 - 52
hsa-mir-650
CAGUGCUGGGGUCUCAGGAGGCAGCGCUCUCAGGACGUCACCACCAUGGCCUGGGCUCUGCUCCUCCU
CACCCUCCUCACUCAGGGCACAGGUGAU chr22 23165295 . G A
0.000798722 + 26
hsa-mir-650
CAGUGCUGGGGUCUCAGGAGGCAGCGCUCUCAGGACGUCACCACCAUGGCCUGGGCUCUGCUCCUCCU
CACCCUCCUCACUCAGGGCACAGGUGAU chr22 23165301 rs200746309 A G
0.000599042 + 32
hsa-mir-650
CAGUGCUGGGGUCUCAGGAGGCAGCGCUCUCAGGACGUCACCACCAUGGCCUGGGCUCUGCUCCUCCU
CACCCUCCUCACUCAGGGCACAGGUGAU chr22 23165333 rs201141491 C A
0.00519169 + 64
hsa-mir-650
CAGUGCUGGGGUCUCAGGAGGCAGCGCUCUCAGGACGUCACCACCAUGGCCUGGGCUCUGCUCCUCCU
CACCCUCCUCACUCAGGGCACAGGUGAU chr22 23165340 rs5996397 C G
0.152556 + 71
hsa-mir-650
CAGUGCUGGGGUCUCAGGAGGCAGCGCUCUCAGGACGUCACCACCAUGGCCUGGGCUCUGCUCCUCCU
CACCCUCCUCACUCAGGGCACAGGUGAU chr22 23165356 rs373942695 C U
0.000199681 + 87
hsa-mir-650
CAGUGCUGGGGUCUCAGGAGGCAGCGCUCUCAGGACGUCACCACCAUGGCCUGGGCUCUGCUCCUCCU
CACCCUCCUCACUCAGGGCACAGGUGAU chr22 23165365 . U C
0.00259585 + 96
hsa-mir-651
AAUCUAUCACUGCUUUUUAGGAUAAGCUUGACUUUUGUUCAAAUAAAAAUGCAAAAGGAAAGUGUAUC
CUAAAAGGCAAUGACAGUUUAAUGUGUUU chrX 8095011 rs144909301 A G
0.00874172 + 6
hsa-mir-651
AAUCUAUCACUGCUUUUUAGGAUAAGCUUGACUUUUGUUCAAAUAAAAAUGCAAAAGGAAAGUGUAUC
CUAAAAGGCAAUGACAGUUUAAUGUGUUU chrX 8095036 rs111336920 A G
null null 31
hsa-mir-652
ACGAAUGGCUAUGCACUGCACAACCCUAGGAGAGGGUGCCAUUCACAUAGACUAUAAUUGAAUGGCGC
CACUAGGGUUGUGCAGUGCACAACCUACAC chrX 109298558 rs184631864 C U
0.00397351 + 2
hsa-mir-653
UUCAUUCCUUCAGUGUUGAAACAAUCUCUACUGAACCAGCUUCAAACAAGUUCACUGGAGUUUGUUUC
AAUAUUGCAAGAAUGAUAAGAUGGAAGC chr7 93112137 . C G
0.000199681 - 31
hsa-mir-653
UUCAUUCCUUCAGUGUUGAAACAAUCUCUACUGAACCAGCUUCAAACAAGUUCACUGGAGUUUGUUUC
AAUAUUGCAAGAAUGAUAAGAUGGAAGC chr7 93112083 . U C
0.000199681 - 85

hsa-mir-654
GGGUAAGUGGAAAGAUGGUGGGCCGCAGAACAUGUGCUGAGUUCGUGCCAUAUGUCUGCUGACCAUCACCUUUAGAAGCCCchr14 101506577 . G A 0.000199681 + 22

hsa-mir-654
GGGUAAGUGGAAAGAUGGUGGGCCGCAGAACAUGUGCUGAGUUCGUGCCAUAUGUCUGCUGACCAUCACCUUUAGAAGCCCchr14 101506580 . G A 0 + 25

hsa-mir-654
GGGUAAGUGGAAAGAUGGUGGGCCGCAGAACAUGUGCUGAGUUCGUGCCAUAUGUCUGCUGACCAUCACCUUUAGAAGCCCchr14 101506596 rs377283150 G C 0.000599042 + 41

hsa-mir-654
GGGUAAGUGGAAAGAUGGUGGGCCGCAGAACAUGUGCUGAGUUCGUGCCAUAUGUCUGCUGACCAUCACCUUUAGAAGCCCchr14 101506600 rs368487359 G A 0.000599042 + 45

hsa-mir-655
AACUAUGCAAGGAUAUUUGAGGAGAGGUUAUCCGUGUUAUGUUCGCUUCAUUCAUCAUGAAUAAUACAUGGUUAACCUCUUUUUGAAUAUCAGACUC chr14 101515920 . G A 0.000399361 + 34

hsa-mir-655
AACUAUGCAAGGAUAUUUGAGGAGAGGUUAUCCGUGUUAUGUUCGCUUCAUUCAUCAUGAAUAAUACAUGGUUAACCUCUUUUUGAAUAUCAGACUC chr14 101515923 . U C 0.000199681 + 37

hsa-mir-655
AACUAUGCAAGGAUAUUUGAGGAGAGGUUAUCCGUGUUAUGUUCGCUUCAUUCAUCAUGAAUAAUACAUGGUUAACCUCUUUUUGAAUAUCAGACUC chr14 101515943 . A C 0.000199681 + 57

hsa-mir-656
CUGAAAUAGGUUGCCUGUGAGGUGUUCACUUUCUAUAUGAUGAAUAUUAUACAGUCAACCUCUUUCCGAUAUCGAAUC chr14 101533074 . C A 0.000199681 + 14

hsa-mir-656
CUGAAAUAGGUUGCCUGUGAGGUGUUCACUUUCUAUAUGAUGAAUAUUAUACAGUCAACCUCUUUCCGAUAUCGAAUC chr14 101533093 rs58834075 C U 0.0926518 + 33

hsa-mir-656
CUGAAAUAGGUUGCCUGUGAGGUGUUCACUUUCUAUAUGAUGAAUAUUAUACAGUCAACCUCUUUCCGAUAUCGAAUC chr14 101533116 . C U 0.000399361 + 56

hsa-mir-656
CUGAAAUAGGUUGCCUGUGAGGUGUUCACUUUCUAUAUGAUGAAUAUUAUACAGUCAACCUCUUUCCGAUAUCGAAUC chr14 101533123 rs185468172 U C 0.000199681 + 63

hsa-mir-656
CUGAAAUAGGUUGCCUGUGAGGUGUUCACUUUCUAUAUGAUGAAUAUUAUACAGUCAACCUCUUUCCGAUAUCGAAUC chr14 101533133 rs190189102 C U 0.000199681 +

73
hsa-mir-656
CUGAAAUAGGUUGCCUGUGAGGUGUUCACUUUCUAUAUGAUGAAUAUUAUACAGUCAACCUCUUUCCG
AUAUCGAAUC chr14 101533134 rs371434052 G A 0.00199681 +
74
hsa-mir-657
GUGUAGUAGAGCUAGGAGGAGAGGGUCCUGGAGAAGCGUGGACCGGUCCGGGUGGGUUCCGGCAGGUU
CUCACCCUCUCUAGGCCCCAUUCUCCUCUG chr17 79099150 rs369112104 G C
0.000399361 - 24
hsa-mir-657
GUGUAGUAGAGCUAGGAGGAGAGGGUCCUGGAGAAGCGUGGACCGGUCCGGGUGGGUUCCGGCAGGUU
CUCACCCUCUCUAGGCCCCAUUCUCCUCUG chr17 79099130 rs372805254 C U
0.000399361 - 44
hsa-mir-657
GUGUAGUAGAGCUAGGAGGAGAGGGUCCUGGAGAAGCGUGGACCGGUCCGGGUGGGUUCCGGCAGGUU
CUCACCCUCUCUAGGCCCCAUUCUCCUCUG chr17 79099125 rs146763174 C U
0.00179712 - 49
hsa-mir-657
GUGUAGUAGAGCUAGGAGGAGAGGGUCCUGGAGAAGCGUGGACCGGUCCGGGUGGGUUCCGGCAGGUU
CUCACCCUCUCUAGGCCCCAUUCUCCUCUG chr17 79099114 rs377125988 C U
0.000199681 - 60
hsa-mir-657
GUGUAGUAGAGCUAGGAGGAGAGGGUCCUGGAGAAGCGUGGACCGGUCCGGGUGGGUUCCGGCAGGUU
CUCACCCUCUCUAGGCCCCAUUCUCCUCUG chr17 79099097 . C G
0.000399361 - 77
hsa-mir-657
GUGUAGUAGAGCUAGGAGGAGAGGGUCCUGGAGAAGCGUGGACCGGUCCGGGUGGGUUCCGGCAGGUU
CUCACCCUCUCUAGGCCCCAUUCUCCUCUG chr17 79099079 rs371946056 U G
0.000199681 - 95
hsa-mir-658
GCUCGGUUGCCGUGGUUGCGGGCCCUGCCCGCCCGCCAGCUCGCUGACAGCACGACUCAGGGCGGAGG
GAAGUAGGUCCGUUGGUCGGUCGGGAACGAGG chr22 38240378 . G U
0.000199681 - 1
hsa-mir-658
GCUCGGUUGCCGUGGUUGCGGGCCCUGCCCGCCCGCCAGCUCGCUGACAGCACGACUCAGGGCGGAGG
GAAGUAGGUCCGUUGGUCGGUCGGGAACGAGG chr22 38240368 rs141002682 C G
0.0169728 - 11
hsa-mir-658
GCUCGGUUGCCGUGGUUGCGGGCCCUGCCCGCCCGCCAGCUCGCUGACAGCACGACUCAGGGCGGAGG
GAAGUAGGUCCGUUGGUCGGUCGGGAACGAGG chr22 38240324 . A G
0.000199681 - 55
hsa-mir-658
GCUCGGUUGCCGUGGUUGCGGGCCCUGCCCGCCCGCCAGCUCGCUGACAGCACGACUCAGGGCGGAGG
GAAGUAGGUCCGUUGGUCGGUCGGGAACGAGG chr22 38240315 . G C
0.000199681 - 64
hsa-mir-658
GCUCGGUUGCCGUGGUUGCGGGCCCUGCCCGCCCGCCAGCUCGCUGACAGCACGACUCAGGGCGGAGG

GAAGUAGGUCCGUUGGUCGGUCGGGAACGAGG chr22 38240300 . C G
0.000199681 - 79
hsa-mir-658
GCUCGGUUGCCGUGGUUGCGGGCCCUGCCCGCCCGCCAGCUCGCUGACAGCACGACUCAGGGCGGAGG
GAAGUAGGUCCGUUGGUCGGUCGGGAACGAGG chr22 38240299 . G C
0.000199681 - 80
hsa-mir-658
GCUCGGUUGCCGUGGUUGCGGGCCCUGCCCGCCCGCCAGCUCGCUGACAGCACGACUCAGGGCGGAGG
GAAGUAGGUCCGUUGGUCGGUCGGGAACGAGG chr22 38240289 rs182859563 C A
0.00299521 - 90
hsa-mir-658
GCUCGGUUGCCGUGGUUGCGGGCCCUGCCCGCCCGCCAGCUCGCUGACAGCACGACUCAGGGCGGAGG
GAAGUAGGUCCGUUGGUCGGUCGGGAACGAGG chr22 38240280 rs369972422 G A
0.00359425 - 99
hsa-mir-658
GCUCGGUUGCCGUGGUUGCGGGCCCUGCCCGCCCGCCAGCUCGCUGACAGCACGACUCAGGGCGGAGG
GAAGUAGGUCCGUUGGUCGGUCGGGAACGAGG chr22 38240279 . G A
0.000199681 - 100
hsa-mir-659
UACCGACCCUCGAUUUGGUUCAGGACCUUCCCUGAACCAAGGAAGAGUCACAGUCUCUUCCUUGGUUC
AGGGAGGGUCCCCAACAAUGUCCUCAUGG chr22 38243770 . G A
0.000199681 - 12
hsa-mir-659
UACCGACCCUCGAUUUGGUUCAGGACCUUCCCUGAACCAAGGAAGAGUCACAGUCUCUUCCUUGGUUC
AGGGAGGGUCCCCAACAAUGUCCUCAUGG chr22 38243743 . A C
0.000199681 - 39
hsa-mir-659
UACCGACCCUCGAUUUGGUUCAGGACCUUCCCUGAACCAAGGAAGAGUCACAGUCUCUUCCUUGGUUC
AGGGAGGGUCCCCAACAAUGUCCUCAUGG chr22 38243739 . A C
0.000199681 - 43
hsa-mir-659
UACCGACCCUCGAUUUGGUUCAGGACCUUCCCUGAACCAAGGAAGAGUCACAGUCUCUUCCUUGGUUC
AGGGAGGGUCCCCAACAAUGUCCUCAUGG chr22 38243727 . C G
0.000199681 - 55
hsa-mir-660
CUGCUCCUUCUCCCAUACCCAUUGCAUAUCGGAGUUGUGAAUUCUCAAAACACCUCCUGUGUGCAUGG
AUUACAGGAGGGUGAGCCUUGUCAUCGUG chrX 49777907 rs180785970 G C
0.000529801 + 59
hsa-mir-661
GGAGAGGCUGUGCUGUGGGGCAGGCGCAGGCCUGAGCCCUGGUUUCGGGCUGCCUGGGUCUCUGGCCU
GCGCGUGACUUUGGGGUGGCU chr8 145019437 . U G
0.000998403 - 11
hsa-mir-661
GGAGAGGCUGUGCUGUGGGGCAGGCGCAGGCCUGAGCCCUGGUUUCGGGCUGCCUGGGUCUCUGGCCU
GCGCGUGACUUUGGGGUGGCU chr8 145019401 rs372639714 G A
0.000399361 - 47
hsa-mir-661

GGAGAGGCUGUGCUGUGGGGCAGGCGCAGGCCUGAGCCCUGGUUUCGGGCUGCCUGGGUCUCUGGCCU
GCGCGUGACUUUGGGGUGGCU chr8 145019377 . G A
0.000199681 - 71
hsa-mir-661
GGAGAGGCUGUGCUGUGGGGCAGGCGCAGGCCUGAGCCCUGGUUUCGGGCUGCCUGGGUCUCUGGCCU
GCGCGUGACUUUGGGGUGGCU chr8 145019376 . C U
0.000399361 - 72
hsa-mir-662
GCUGUUGAGGCUGCGCAGCCAGGCCCUGACGGUGGGGUGGCUGCGGGCCUUCUGAAGGUCUCCCACGU
UGUGGCCCAGCAGCGCAGUCACGUUGC chr16 820192 rs137952851 G A
0.0121805 + 10
hsa-mir-662
GCUGUUGAGGCUGCGCAGCCAGGCCCUGACGGUGGGGUGGCUGCGGGCCUUCUGAAGGUCUCCCACGU
UGUGGCCCAGCAGCGCAGUCACGUUGC chr16 820197 rs367967080 G A
0.000599042 + 15
hsa-mir-662
GCUGUUGAGGCUGCGCAGCCAGGCCCUGACGGUGGGGUGGCUGCGGGCCUUCUGAAGGUCUCCCACGU
UGUGGCCCAGCAGCGCAGUCACGUUGC chr16 820215 rs74656628 U A
0.13738 + 33
hsa-mir-662
GCUGUUGAGGCUGCGCAGCCAGGCCCUGACGGUGGGGUGGCUGCGGGCCUUCUGAAGGUCUCCCACGU
UGUGGCCCAGCAGCGCAGUCACGUUGC chr16 820249 rs9745376 G A
0.0543131 + 67
hsa-mir-662
GCUGUUGAGGCUGCGCAGCCAGGCCCUGACGGUGGGGUGGCUGCGGGCCUUCUGAAGGUCUCCCACGU
UGUGGCCCAGCAGCGCAGUCACGUUGC chr16 820265 rs140176098 G A
0.00119808 + 83
hsa-mir-663a
CCUUCCGGCGUCCCAGGCGGGGCGCCGCGGGACCGCCCUCGUGUCUGUGGCGGUGGGAUCCCGCGGCC
GUGUUUUCCUGGUGGCCCGGCCAUG chr20 26188908 . G A
0.000199681 - 7
hsa-mir-663a
CCUUCCGGCGUCCCAGGCGGGGCGCCGCGGGACCGCCCUCGUGUCUGUGGCGGUGGGAUCCCGCGGCC
GUGUUUUCCUGGUGGCCCGGCCAUG chr20 26188906 rs183843457 C G
0.0165735 - 9
hsa-mir-663a
CCUUCCGGCGUCCCAGGCGGGGCGCCGCGGGACCGCCCUCGUGUCUGUGGCGGUGGGAUCCCGCGGCC
GUGUUUUCCUGGUGGCCCGGCCAUG chr20 26188880 . G U
0.000199681 - 35
hsa-mir-663a
CCUUCCGGCGUCCCAGGCGGGGCGCCGCGGGACCGCCCUCGUGUCUGUGGCGGUGGGAUCCCGCGGCC
GUGUUUUCCUGGUGGCCCGGCCAUG chr20 26188865 . G A
0.000199681 - 50
hsa-mir-663a
CCUUCCGGCGUCCCAGGCGGGGCGCCGCGGGACCGCCCUCGUGUCUGUGGCGGUGGGAUCCCGCGGCC
GUGUUUUCCUGGUGGCCCGGCCAUG chr20 26188825 . C U
0.000399361 - 90

hsa-mir-663b
GGUGCCGAGGGCCGUCCGGCAUCCUAGGCGGGUCGCUGCGGUACCUCCCUCCUGUCUGUGGCGGUGGG
AUCCCGUGGCCGUGUUUUCCUGGUGGCCCGGCCGUGCCUGAGGUUUC chr2 133014640
rs78867621 G A null null 14

hsa-mir-663b
GGUGCCGAGGGCCGUCCGGCAUCCUAGGCGGGUCGCUGCGGUACCUCCCUCCUGUCUGUGGCGGUGGG
AUCCCGUGGCCGUGUUUUCCUGGUGGCCCGGCCGUGCCUGAGGUUUC chr2 133014633
rs115758595 A G null null 21

hsa-mir-663b
GGUGCCGAGGGCCGUCCGGCAUCCUAGGCGGGUCGCUGCGGUACCUCCCUCCUGUCUGUGGCGGUGGG
AUCCCGUGGCCGUGUUUUCCUGGUGGCCCGGCCGUGCCUGAGGUUUC chr2 133014619
rs78327226 G A null null 35

hsa-mir-663b
GGUGCCGAGGGCCGUCCGGCAUCCUAGGCGGGUCGCUGCGGUACCUCCCUCCUGUCUGUGGCGGUGGG
AUCCCGUGGCCGUGUUUUCCUGGUGGCCCGGCCGUGCCUGAGGUUUC chr2 133014612
rs74853538 U G null null 42

hsa-mir-663b
GGUGCCGAGGGCCGUCCGGCAUCCUAGGCGGGUCGCUGCGGUACCUCCCUCCUGUCUGUGGCGGUGGG
AUCCCGUGGCCGUGUUUUCCUGGUGGCCCGGCCGUGCCUGAGGUUUC chr2 133014579
rs62165009 U C null null 75

hsa-mir-664a
GAACAUUGAAACUGGCUAGGGAAAAUGAUUGGAUAGAAACUAUUAUUCUAUUCAUUUAUCCCCAGCCU
ACAAAAUGAAAAAA chr1 220373943 rs113256801 G C 0.00339457
- 19
hsa-mir-664a
GAACAUUGAAACUGGCUAGGGAAAAUGAUUGGAUAGAAACUAUUAUUCUAUUCAUUUAUCCCCAGCCU
ACAAAAUGAAAAAA chr1 220373939 . A G 0.000199681 -
23
hsa-mir-664a
GAACAUUGAAACUGGCUAGGGAAAAUGAUUGGAUAGAAACUAUUAUUCUAUUCAUUUAUCCCCAGCCU
ACAAAAUGAAAAAA chr1 220373934 . A C 0.000199681 -
28
hsa-mir-664a
GAACAUUGAAACUGGCUAGGGAAAAUGAUUGGAUAGAAACUAUUAUUCUAUUCAUUUAUCCCCAGCCU
ACAAAAUGAAAAAA chr1 220373933 . U C 0.000199681 -
29
hsa-mir-664a
GAACAUUGAAACUGGCUAGGGAAAAUGAUUGGAUAGAAACUAUUAUUCUAUUCAUUUAUCCCCAGCCU
ACAAAAUGAAAAAA chr1 220373922 rs201407438 C U
0.000599042 - 40
hsa-mir-664b UGGGCUAAGGGAGAUGAUUGGGUAGAAAGUAUUAUUCUAUUCAUUUGCCUCCCAGCCUACA
chrX 153996899 rs138465224 G C 0.0015894 + 29

hsa-mir-664b UGGGCUAAGGGAGAUGAUUGGGUAGAAAGUAUUAUUCUAUUCAUUUGCCUCCCAGCCUACA chrX 153996903 . U G 0.000264901 + 33

hsa-mir-664b UGGGCUAAGGGAGAUGAUUGGGUAGAAAGUAUUAUUCUAUUCAUUUGCCUCCCAGCCUACA chrX 153996905 rs188563482 U C 0.000529801 + 35

hsa-mir-665 UCUCCUCGAGGGGUCUCUGCCUCUACCCAGGACUCUUUCAUGACCAGGAGGCUGAGGCCCCUCACAGGCGGC chr14 101341373 . C A 0.000199681 + 4

hsa-mir-665 UCUCCUCGAGGGGUCUCUGCCUCUACCCAGGACUCUUUCAUGACCAGGAGGCUGAGGCCCCUCACAGGCGGC chr14 101341407 . U A 0.000798722 + 38

hsa-mir-665 UCUCCUCGAGGGGUCUCUGCCUCUACCCAGGACUCUUUCAUGACCAGGAGGCUGAGGCCCCUCACAGGCGGC chr14 101341408 . C U 0.000798722 + 39

hsa-mir-668 GGUAAGUGCGCCUCGGGUGAGCAUGCACUUAAUGUGGGUGUAUGUCACUCGGCUCGGCCCACUACC chr14 101521608 rs141238795 C U 0.000199681 + 14

hsa-mir-668 GGUAAGUGCGCCUCGGGUGAGCAUGCACUUAAUGUGGGUGUAUGUCACUCGGCUCGGCCCACUACC chr14 101521620 . C U 0.000199681 + 26

hsa-mir-670 GUUUAGGGGUGGACCUGAUGUCCCUGAGUGUAUGUGGUGAACCUGAAUUUGCCUUGGGUUUCCUCAUAUUCAUUCAGGAGUGUCAGUUGCCCCUUCAC chr11 43581287 rs185097505 G U 0.00119808 + 82

hsa-mir-671 GCAGGUGAACUGGCAGGCCAGGAAGAGGAGGAAGCCCUGGAGGGGCUGGAGGUGAUGGAUGUUUUCCUCCGGUUCUCAGGGCUCCACCUCUUUCGGGCCGUAGAGCCAGGGCUGGUGC chr7 150935522 . G A 0.000199681 + 16

hsa-mir-671 GCAGGUGAACUGGCAGGCCAGGAAGAGGAGGAAGCCCUGGAGGGGCUGGAGGUGAUGGAUGUUUUCCUCCGGUUCUCAGGGCUCCACCUCUUUCGGGCCGUAGAGCCAGGGCUGGUGC chr7 150935577 rs200733440 G A 0.000199681 + 71

hsa-mir-671 GCAGGUGAACUGGCAGGCCAGGAAGAGGAGGAAGCCCUGGAGGGGCUGGAGGUGAUGGAUGUUUUCCUCCGGUUCUCAGGGCUCCACCUCUUUCGGGCCGUAGAGCCAGGGCUGGUGC chr7 150935583 rs199522292 C A 0 + 77

hsa-mir-675 CCCAGGGUCUGGUGCGGAGAGGGCCCACAGUGGACUUGGUGACGCUGUAUGCCCUCACCGCUCAGCCC

CUGGG chr11 2018056 . G A 0.000199681 - 6

hsa-mir-675
CCCAGGGUCUGGUGCGGAGAGGGCCCACAGUGGACUUGGUGACGCUGUAUGCCCUCACCGCUCAGCCC
CUGGG chr11 2018019 . C U 0.000199681 - 43

hsa-mir-675
CCCAGGGUCUGGUGCGGAGAGGGCCCACAGUGGACUUGGUGACGCUGUAUGCCCUCACCGCUCAGCCC
CUGGG chr11 2018004 . C U 0.000599042 - 58

hsa-mir-675
CCCAGGGUCUGGUGCGGAGAGGGCCCACAGUGGACUUGGUGACGCUGUAUGCCCUCACCGCUCAGCCC
CUGGG chr11 2018002 rs376650475 G A 0.000199681 - 60

hsa-mir-676
GCAUGACUCUUCAACCUCAGGACUUGCAGAAUUAAUGGAAUGCUGUCCUAAGGUUGUUGAGUUGUGC
chrX 69242707 rs188608568 G A 0.000529801 + 1

hsa-mir-676
GCAUGACUCUUCAACCUCAGGACUUGCAGAAUUAAUGGAAUGCUGUCCUAAGGUUGUUGAGUUGUGC
chrX 69242767 . G A 0.000529801 + 61

hsa-mir-708
AACUGCCCUCAAGGAGCUUACAAUCUAGCUGGGGGUAAAUGACUUGCACAUGAACACAACUAGACUGU
GAGCUUCUAGAGGGCAGGGA chr11 79113119 . G U
0.000199681 - 35
hsa-mir-7-1
UUGGAUGUUGGCCUAGUUCUGUGUGGAAGACUAGUGAUUUUGUUGUUUUUAGAUAACUAAAUCGACAA
CAAAUCACAGUCUGCCAUAUGGCACAGGCCAUGCCUCUACAG chr9 86584720 . A
G 0.000399361 - 53
hsa-mir-7-1
UUGGAUGUUGGCCUAGUUCUGUGUGGAAGACUAGUGAUUUUGUUGUUUUUAGAUAACUAAAUCGACAA
CAAAUCACAGUCUGCCAUAUGGCACAGGCCAUGCCUCUACAG chr9 86584707 rs76662330
C A null null 66
hsa-mir-7-1
UUGGAUGUUGGCCUAGUUCUGUGUGGAAGACUAGUGAUUUUGUUGUUUUUAGAUAACUAAAUCGACAA
CAAAUCACAGUCUGCCAUAUGGCACAGGCCAUGCCUCUACAG chr9 86584675 . C
G 0.000399361 - 98
hsa-mir-711
ACUGACUUUGAGUCUCUCCUCAGGGUGCUGCAGGCAAAGCUGGGGACCCAGGGAGAGACGUAAGUGAG
GGGAGAUG chr3 48616390 rs199808370 C U 0.000199681 -
21
hsa-mir-711
ACUGACUUUGAGUCUCUCCUCAGGGUGCUGCAGGCAAAGCUGGGGACCCAGGGAGAGACGUAAGUGAG
GGGAGAUG chr3 48616372 . G A 0.000199681 - 39

hsa-mir-7-2

CUGGAUACAGAGUGGACCGGCUGGCCCCAUCUGGAAGACUAGUGAUUUUGUUGUUGUCUUACUGCGCU CAACAACAAAUCCCAGUCUACCUAAUGGUGCCAGCCAUCGCA chr15 89155064 rs141312113 A G 0.000599042 + 9

hsa-mir-7-2
CUGGAUACAGAGUGGACCGGCUGGCCCCAUCUGGAAGACUAGUGAUUUUGUUGUUGUCUUACUGCGCU CAACAACAAAUCCCAGUCUACCUAAUGGUGCCAGCCAUCGCA chr15 89155073 rs41276930 C U 0.00599042 + 18

hsa-mir-7-2
CUGGAUACAGAGUGGACCGGCUGGCCCCAUCUGGAAGACUAGUGAUUUUGUUGUUGUCUUACUGCGCU CAACAACAAAUCCCAGUCUACCUAAUGGUGCCAGCCAUCGCA chr15 89155084 rs75737367 A C null null 29

hsa-mir-7-2
CUGGAUACAGAGUGGACCGGCUGGCCCCAUCUGGAAGACUAGUGAUUUUGUUGUUGUCUUACUGCGCU CAACAACAAAUCCCAGUCUACCUAAUGGUGCCAGCCAUCGCA chr15 89155121 rs147579757 G A 0.0109824 + 66

hsa-mir-7-2
CUGGAUACAGAGUGGACCGGCUGGCCCCAUCUGGAAGACUAGUGAUUUUGUUGUUGUCUUACUGCGCU CAACAACAAAUCCCAGUCUACCUAAUGGUGCCAGCCAUCGCA chr15 89155162 rs186890720 C U 0.000199681 + 107

hsa-mir-7-2
CUGGAUACAGAGUGGACCGGCUGGCCCCAUCUGGAAGACUAGUGAUUUUGUUGUUGUCUUACUGCGCU CAACAACAAAUCCCAGUCUACCUAAUGGUGCCAGCCAUCGCA chr15 89155163 rs376166526 G A 0.00359425 + 108

hsa-mir-7-3
AGAUUAGAGUGGCUGUGGUCUAGUGCUGUGUGGAAGACUAGUGAUUUUGUUGUUCUGAUGUACUACGA CAACAAGUCACAGCCGGCCUCAUAGCGCAGACUCCCUUCGAC chr19 4770697 rs76199191 U G null null 16

hsa-mir-7-3
AGAUUAGAGUGGCUGUGGUCUAGUGCUGUGUGGAAGACUAGUGAUUUUGUUGUUCUGAUGUACUACGA CAACAAGUCACAGCCGGCCUCAUAGCGCAGACUCCCUUCGAC chr19 4770709 . G C 0.000399361 + 28

hsa-mir-7-3
AGAUUAGAGUGGCUGUGGUCUAGUGCUGUGUGGAAGACUAGUGAUUUUGUUGUUCUGAUGUACUACGA CAACAAGUCACAGCCGGCCUCAUAGCGCAGACUCCCUUCGAC chr19 4770747 rs146575140 C U 0.00219649 + 66

hsa-mir-7-3
AGAUUAGAGUGGCUGUGGUCUAGUGCUGUGUGGAAGACUAGUGAUUUUGUUGUUCUGAUGUACUACGA CAACAAGUCACAGCCGGCCUCAUAGCGCAGACUCCCUUCGAC chr19 4770789 . G A 0.000399361 + 108

hsa-mir-744
UUGGGCAAGGUGCGGGGCUAGGGCUAACAGCAGUCUUACUGAAGGUUUCCUGGAAACCACGCACAUGC UGUUGCCACUAACCUCAACCUUACUCGGUC chr17 11985275 . C U

0.000199681	+	60
hsa-mir-758
GCCUGGAUACAUGAGAUGGUUGACCAGAGAGCACACGCUUUAUUUGUGCCGUUUGUGACCUGGUCCAC
UAACCCUCAGUAUCUAAUGC	chr14	101492444	.	C	U
0.000199681	+	88
hsa-mir-759
UAAUAAAUUAAAUGCCUAAACUGGCAGAGUGCAAACAAUUUUGACUCAGAUCUAAAUGUUUGCACUGG
CUGUUUAAACAUUUAAUUUGUUA	chr13	53384209	rs144233096	C	G
0.00259585	+	25
hsa-mir-759
UAAUAAAUUAAAUGCCUAAACUGGCAGAGUGCAAACAAUUUUGACUCAGAUCUAAAUGUUUGCACUGG
CUGUUUAAACAUUUAAUUUGUUA	chr13	53384256	rs77079459	U	G
null	null	72
hsa-mir-759
UAAUAAAUUAAAUGCCUAAACUGGCAGAGUGCAAACAAUUUUGACUCAGAUCUAAAUGUUUGCACUGG
CUGUUUAAACAUUUAAUUUGUUA	chr13	53384262	.	C	U
0.000199681	+	78
hsa-mir-760
GGCGCGUCGCCCCCCUCAGUCCACCAGAGCCCGGAUACCUCAGAAAUUCGGCUCUGGGUCUGUGGGGA
GCGAAAUGCAAC	chr1	94312442	.	U	A	0.000199681	+	55

hsa-mir-761	GGAGGAGCAGCAGGGUGAAACUGACACAGUUCUGGUGAGUUUCACUUUGCUGCUCCUCC
chr1	52302043	.	C	U	0.000199681	-	32

hsa-mir-762
GGCCCGGCUCCGGGUCUCGGCCCGUACAGUCCGGCCGGCCAUGCUGGCGGGGCUGGGGCCGGGGCCGA
GCCCGCGGCGGGGCC	chr16	30905269	.	G	A	0.000199681	+
46
hsa-mir-762
GGCCCGGCUCCGGGUCUCGGCCCGUACAGUCCGGCCGGCCAUGCUGGCGGGGCUGGGGCCGGGGCCGA
GCCCGCGGCGGGGCC	chr16	30905298	.	G	U	0.000798722	+
75
hsa-mir-766
GCAUCCUCAGGACCUGGGCUUGGGUGGUAGGAGGAAUUGGUGCUGGUCUUUCAUUUUGGAUUUGACUC
CAGCCCCACAGCCUCAGCCACCCCAGCCAAUUGUCAUAGGAGC	chrX	118780790
rs375057002	G	U	0.000264901	-	22

hsa-mir-766
GCAUCCUCAGGACCUGGGCUUGGGUGGUAGGAGGAAUUGGUGCUGGUCUUUCAUUUUGGAUUUGACUC
CAGCCCCACAGCCUCAGCCACCCCAGCCAAUUGUCAUAGGAGC	chrX	118780738	.	C
U	0.000529801	-	74
hsa-mir-767
GCUUUUAUAUUGUAGGUUUUUGCUCAUGCACCAUGGUUGUCUGAGCAUGCAGCAUGCUUGUCUGCUCA
UACCCCAUGGUUUCUGAGCAGGAACCUUCAUUGUCUACUGC	chrX	151561912	.	G
A	0.000264901	-	90
hsa-mir-769
GCCUUGGUGCUGAUUCCUGGGCUCUGACCUGAGACCUCUGGGUUCUGAGCUGUGAUGUUGCUCUCGAG

CUGGGAUCUCCGGGGUCUUGGUUCAGGGCCGGGGCCUCUGGGUUCCAAGC chr19 46522190 .
G A 0.000199681 + 1
hsa-mir-769
GCCUUGGUGCUGAUUCCUGGGCUCUGACCUGAGACCUCUGGGUUCUGAGCUGUGAUGUUGCUCUCGAG
CUGGGAUCUCCGGGGUCUUGGUUCAGGGCCGGGGCCUCUGGGUUCCAAGC chr19 46522201 .
G A 0.000199681 + 12
hsa-mir-769
GCCUUGGUGCUGAUUCCUGGGCUCUGACCUGAGACCUCUGGGUUCUGAGCUGUGAUGUUGCUCUCGAG
CUGGGAUCUCCGGGGUCUUGGUUCAGGGCCGGGGCCUCUGGGUUCCAAGC chr19 46522255
rs185689285 G A 0.00139776 + 66

hsa-mir-769
GCCUUGGUGCUGAUUCCUGGGCUCUGACCUGAGACCUCUGGGUUCUGAGCUGUGAUGUUGCUCUCGAG
CUGGGAUCUCCGGGGUCUUGGUUCAGGGCCGGGGCCUCUGGGUUCCAAGC chr19 46522298
rs190748158 G A 0.00199681 + 109

hsa-mir-770
AGGAGCCACCUUCCGAGCCUCCAGUACCACGUGUCAGGGCCACAUGAGCUGGGCCUCGUGGGCCUGAU
GUGGUGCUGGGGCCUCAGGGGUCUGCUCUU chr14 101318740 rs376495137 C U
0.000199681 + 14
hsa-mir-770
AGGAGCCACCUUCCGAGCCUCCAGUACCACGUGUCAGGGCCACAUGAGCUGGGCCUCGUGGGCCUGAU
GUGGUGCUGGGGCCUCAGGGGUCUGCUCUU chr14 101318783 . C U
0.000199681 + 57
hsa-mir-770
AGGAGCCACCUUCCGAGCCUCCAGUACCACGUGUCAGGGCCACAUGAGCUGGGCCUCGUGGGCCUGAU
GUGGUGCUGGGGCCUCAGGGGUCUGCUCUU chr14 101318784 . G C
0.000199681 + 58
hsa-mir-770
AGGAGCCACCUUCCGAGCCUCCAGUACCACGUGUCAGGGCCACAUGAGCUGGGCCUCGUGGGCCUGAU
GUGGUGCUGGGGCCUCAGGGGUCUGCUCUU chr14 101318807 . C U
0.000199681 + 81
hsa-mir-802
GUUCUGUUAUUUGCAGUCAGUAACAAAGAUUCAUCCUUGUGUCCAUCAUGCAACAAGGAGAAUCUUUG
UCACUUAGUGUAAUUAAUAGCUGGACchr21 37093013 rs376162684 G A
0.000199681 + 1
hsa-mir-802
GUUCUGUUAUUUGCAGUCAGUAACAAAGAUUCAUCCUUGUGUCCAUCAUGCAACAAGGAGAAUCUUUG
UCACUUAGUGUAAUUAAUAGCUGGACchr21 37093078 . U C
0.000199681 + 66
hsa-mir-802
GUUCUGUUAUUUGCAGUCAGUAACAAAGAUUCAUCCUUGUGUCCAUCAUGCAACAAGGAGAAUCUUUG
UCACUUAGUGUAAUUAAUAGCUGGACchr21 37093089 rs184815008 U C
0.000199681 + 77
hsa-mir-802
GUUCUGUUAUUUGCAGUCAGUAACAAAGAUUCAUCCUUGUGUCCAUCAUGCAACAAGGAGAAUCUUUG
UCACUUAGUGUAAUUAAUAGCUGGACchr21 37093097 . A G

0.000199681 + 85
hsa-mir-873
GUGUGCAUUUGCAGGAACUUGUGAGUCUCCUAUUGAAAAUGAACAGGAGACUGAUGAGUUCCCGGGAA
CACCCACAA chr9 28888922 rs199861280 A G 0.000399361 -
32
hsa-mir-874
UUAGCCCUGCGGCCCCACGCACCAGGGUAAGAGAGACUCUCGCUUCCUGCCCUGGCCCGAGGGACCGA
CUGGCUGGGC chr5 136983326 . C U 0.000199681 - 13

hsa-mir-874
UUAGCCCUGCGGCCCCACGCACCAGGGUAAGAGAGACUCUCGCUUCCUGCCCUGGCCCGAGGGACCGA
CUGGCUGGGC chr5 136983319 . C U 0.000199681 - 20

hsa-mir-874
UUAGCCCUGCGGCCCCACGCACCAGGGUAAGAGAGACUCUCGCUUCCUGCCCUGGCCCGAGGGACCGA
CUGGCUGGGC chr5 136983281 . C U 0.000199681 - 58

hsa-mir-874
UUAGCCCUGCGGCCCCACGCACCAGGGUAAGAGAGACUCUCGCUUCCUGCCCUGGCCCGAGGGACCGA
CUGGCUGGGC chr5 136983275 . A G 0.000199681 - 64

hsa-mir-876
UGAAGUGCUGUGGAUUUCUUUGUGAAUCACCAUAUCUAAGCUAAUGUGGUGGUGGUUUACAAAGUAAU
UCAUAGUGCUUCAchr9 28863695 rs373286402 G A 0.000199681 -
10
hsa-mir-876
UGAAGUGCUGUGGAUUUCUUUGUGAAUCACCAUAUCUAAGCUAAUGUGGUGGUGGUUUACAAAGUAAU
UCAUAGUGCUUCAchr9 28863633 . U C 0.00259585 - 72

hsa-mir-876
UGAAGUGCUGUGGAUUUCUUUGUGAAUCACCAUAUCUAAGCUAAUGUGGUGGUGGUUUACAAAGUAAU
UCAUAGUGCUUCAchr9 28863628 rs372901000 C U 0.000599042 -
77
hsa-mir-877
GUAGAGGAGAUGGCGCAGGGGACACGGGCAAAGACUUGGGGGUUCCUGGGACCCUCAGACGUGUGUCC
UCUUCUCCCUCCUCCCAG chr6 30552168 . C U 0.000199681 +
60
hsa-mir-877
GUAGAGGAGAUGGCGCAGGGGACACGGGCAAAGACUUGGGGGUUCCUGGGACCCUCAGACGUGUGUCC
UCUUCUCCCUCCUCCCAG chr6 30552187 rs372113020 C U 0.00199681
+ 79
hsa-mir-885
CCGCACUCUCUCCAUUACACUACCCUGCCUCUUCUCCAUGAGAGGCAGCGGGGUGUAGUGGAUAGAGC
ACGGGU chr3 10436238 . U C 0.000199681 - 9

hsa-mir-885
CCGCACUCUCUCCAUUACACUACCCUGCCUCUUCUCCAUGAGAGGCAGCGGGGUGUAGUGGAUAGAGC

ACGGGU chr3 10436219 . C U 0.000199681 - 28

hsa-mir-885
CCGCACUCUCUCCAUUACACUACCCUGCCUCUUCUCCAUGAGAGGCAGCGGGGUGUAGUGGAUAGAGC
ACGGGU chr3 10436201 rs376907509 C U 0.000199681 - 46

hsa-mir-885
CCGCACUCUCUCCAUUACACUACCCUGCCUCUUCUCCAUGAGAGGCAGCGGGGUGUAGUGGAUAGAGC
ACGGGU chr3 10436194 rs189766364 G C 0.000199681 - 53

hsa-mir-885
CCGCACUCUCUCCAUUACACUACCCUGCCUCUUCUCCAUGAGAGGCAGCGGGGUGUAGUGGAUAGAGC
ACGGGU chr3 10436187 . G A 0.000199681 - 60

hsa-mir-885
CCGCACUCUCUCCAUUACACUACCCUGCCUCUUCUCCAUGAGAGGCAGCGGGGUGUAGUGGAUAGAGC
ACGGGU chr3 10436180 . G A 0.000199681 - 67

hsa-mir-887
GUGCAGAUCCUUGGGAGCCCUGUUAGACUCUGGAUUUUACACUUGGAGUGAACGGGCGCCAUCCCGAG
GCUUUGCACAG chr5 15935316 . G C 0.000199681 + 26

hsa-mir-887
GUGCAGAUCCUUGGGAGCCCUGUUAGACUCUGGAUUUUACACUUGGAGUGAACGGGCGCCAUCCCGAG
GCUUUGCACAG chr5 15935347 . C U 0.000199681 + 57

hsa-mir-887
GUGCAGAUCCUUGGGAGCCCUGUUAGACUCUGGAUUUUACACUUGGAGUGAACGGGCGCCAUCCCGAG
GCUUUGCACAG chr5 15935348 . G C 0.000199681 + 58

hsa-mir-887
GUGCAGAUCCUUGGGAGCCCUGUUAGACUCUGGAUUUUACACUUGGAGUGAACGGGCGCCAUCCCGAG
GCUUUGCACAG chr5 15935351 . A G 0.000199681 + 61

hsa-mir-888
GGCAGUGCUCUACUCAAAAAGCUGUCAGUCACUUAGAUUACAUGUGACUGACACCUCUUUGGGUGAAG
GAAGGCUCA chrX 145076376 . C A 0.0015894 - 3

hsa-mir-888
GGCAGUGCUCUACUCAAAAAGCUGUCAGUCACUUAGAUUACAUGUGACUGACACCUCUUUGGGUGAAG
GAAGGCUCA chrX 145076356 rs112850228 U C null null 23

hsa-mir-888
GGCAGUGCUCUACUCAAAAAGCUGUCAGUCACUUAGAUUACAUGUGACUGACACCUCUUUGGGUGAAG
GAAGGCUCA chrX 145076355 rs143634721 G U 0.0124503 -
24
hsa-mir-888

GGCAGUGCUCUACUCAAAAAGCUGUCAGUCACUUAGAUUACAUGUGACUGACACCUCUUUGGGUGAAG
GAAGGCUCA chrX 145076302 rs5965660 A C 0.107285 -
77
hsa-mir-890
GGAAGUGCCCUACUUGGAAAGGCAUCAGUUGCUUAGAUUACAUGUAACUAUUCCCUUUCUGAGUAGAG
UAAGUCUUA chrX 145075848 rs369372319 G A 0.000264901 -
22
hsa-mir-890
GGAAGUGCCCUACUUGGAAAGGCAUCAGUUGCUUAGAUUACAUGUAACUAUUCCCUUUCUGAGUAGAG
UAAGUCUUA chrX 145075804 rs138166791 G C 0.00503311 -
66
hsa-mir-891a
CCUUAAUCCUUGCAACGAACCUGAGCCACUGAUUCAGUAAAAUACUCAGUGGCACAUGUUUGUUGUGA
GGGUCAAAAGA chrX 145109356 rs5965990 C U 0.0188079 -
35
hsa-mir-891a
CCUUAAUCCUUGCAACGAACCUGAGCCACUGAUUCAGUAAAAUACUCAGUGGCACAUGUUUGUUGUGA
GGGUCAAAAGA chrX 145109328 rs188075884 U C 0.000264901 -
63
hsa-mir-891b
CCUUAAUCCUUGCAACUUACCUGAGUCAUUGAUUCAGUAAAACAUUCAAUGGCACAUGUUUGUUGUUA
GGGUCAAAAGA chrX 145082615 rs143246735 C G 0.00291391 -
35
hsa-mir-892a
GCAGUGCCUUACUCAGAAAGGUGCCAGUCACUUACACUACAUGUCACUGUGUCCUUUCUGCGUAGAGU
AAGGCUC chrX 145078244 rs183818844 A G 0.000264901 -
18
hsa-mir-892a
GCAGUGCCUUACUCAGAAAGGUGCCAGUCACUUACACUACAUGUCACUGUGUCCUUUCUGCGUAGAGU
AAGGCUC chrX 145078200 . G A 0.000264901 - 62

hsa-mir-892b
UGCAAUGCCCUACUCAGAAAGGUGCCAUUUAUGUAGAUUUUAUGUCACUGGCUCCUUUCUGGGUAGAG
CAAGGCUCA chrX 145078733 rs146806052 U C 0.0188079 -
60
hsa-mir-892c
GGCAGUGCCCUAUUCAGAAAGGUGCCAGUCACUUAGAUUACAUGUCACUGUUUCCUUUCUGAGUGGAG
UAGGGCUUA chrX 145074342 . C G 0.000264901 - 3

hsa-mir-892c
GGCAGUGCCCUAUUCAGAAAGGUGCCAGUCACUUAGAUUACAUGUCACUGUUUCCUUUCUGAGUGGAG
UAGGGCUUA chrX 145074289 . U C 0.000529801 - 56

hsa-mir-892c
GGCAGUGCCCUAUUCAGAAAGGUGCCAGUCACUUAGAUUACAUGUCACUGUUUCCUUUCUGAGUGGAG
UAGGGCUUA chrX 145074284 . G A 0.000794702 - 61

hsa-mir-892c
GGCAGUGCCCUAUUCAGAAAGGUGCCAGUCACUUAGAUUACAUGUCACUGUUUCCUUUCUGAGUGGAG
UAGGGCUUA chrX 145074283 . A G 0.000794702 - 62

hsa-mir-892c
GGCAGUGCCCUAUUCAGAAAGGUGCCAGUCACUUAGAUUACAUGUCACUGUUUCCUUUCUGAGUGGAG
UAGGGCUUA chrX 145074272 rs147601604 G A 0.00291391 -
73
hsa-mir-9-1
CGGGGUUGGUUGUUAUCUUUGGUUAUCUAGCUGUAUGAGUGGUGUGGAGUCUUCAUAAAGCUAGAUAA
CCGAAAGUAAAAAUAACCCCA chr1 156390147 . G A
0.000199681 - 75
hsa-mir-9-2
GGAAGCGAGUUGUUAUCUUUGGUUAUCUAGCUGUAUGAGUGUAUUGGUCUUCAUAAAGCUAGAUAACC
GAAAGUAAAAACUCCUUCA chr5 87962747 rs41265488 U A
0.000199681 - 11
hsa-mir-920
GUAGUUGUUCUACAGAAGACCUGGAUGUGUAGGAGCUAAGACACACUCCAGGGGAGCUGUGGAAGCAG
UAACACG chr12 24365358 rs66686007 GUUGU - null null 4

hsa-mir-920
GUAGUUGUUCUACAGAAGACCUGGAUGUGUAGGAGCUAAGACACACUCCAGGGGAGCUGUGGAAGCAG
UAACACG chr12 24365383 . G A 0.000199681 + 29

hsa-mir-922
AUGGCGUUUUCCCUCUCCCUGUCCUGGACUGGGGUCAGACUGUGCCCCGAGGAGAAGCAGCAGAGAAU
AGGACUACGUCAUchr3 197401443 . C U 0.000199681 - 5

hsa-mir-922
AUGGCGUUUUCCCUCUCCCUGUCCUGGACUGGGGUCAGACUGUGCCCCGAGGAGAAGCAGCAGAGAAU
AGGACUACGUCAUchr3 197401442 rs150407249 G A 0.000199681 -
6
hsa-mir-922
AUGGCGUUUUCCCUCUCCCUGUCCUGGACUGGGGUCAGACUGUGCCCCGAGGAGAAGCAGCAGAGAAU
AGGACUACGUCAUchr3 197401415 rs114814977 G U 0.0139776 -
33
hsa-mir-922
AUGGCGUUUUCCCUCUCCCUGUCCUGGACUGGGGUCAGACUGUGCCCCGAGGAGAAGCAGCAGAGAAU
AGGACUACGUCAUchr3 197401372 rs182056447 C U 0.000599042 -
76
hsa-mir-922
AUGGCGUUUUCCCUCUCCCUGUCCUGGACUGGGGUCAGACUGUGCCCCGAGGAGAAGCAGCAGAGAAU
AGGACUACGUCAUchr3 197401371 rs140463854 G A 0.000199681 -
77
hsa-mir-924 AAUAGAGUCUUGUGAUGUCUUGCUUAAGGGCCAUCCAACCUAGAGUCUACAAC
chr18 37202138 . A G 0.000199681 - 2

hsa-mir-924 AAUAGAGUCUUGUGAUGUCUUGCUUAAGGGCCAUCCAACCUAGAGUCUACAAC
chr18 37202113 . A C 0.000199681 - 27

hsa-mir-924 AAUAGAGUCUUGUGAUGUCUUGCUUAAGGGCCAUCCAACCUAGAGUCUACAAC
chr18 37202112 . G A 0.000199681 - 28

hsa-mir-924 AAUAGAGUCUUGUGAUGUCUUGCUUAAGGGCCAUCCAACCUAGAGUCUACAAC
chr18 37202092 . U C 0.000199681 - 48

hsa-mir-92a-1
CUUUCUACACAGGUUGGGAUCGGUUGCAAUGCUGUGUUUCUGUAUGGUAUUGCACUUGUCCCGGCCUG
UUGAGUUUGG chr13 92003588 rs72631821 C U null null 21

hsa-mir-92a-1
CUUUCUACACAGGUUGGGAUCGGUUGCAAUGCUGUGUUUCUGUAUGGUAUUGCACUUGUCCCGGCCUG
UUGAGUUUGG chr13 92003589 rs9589207 G A 0.0239617 +
22

hsa-mir-92a-2
UCAUCCCUGGGUGGGGAUUUGUUGCAUUACUUGUGUUCUAUAUAAAGUAUUGCACUUGUCCCGGCCUG
UGGAAGA chrX 133303579 . G A 0.000264901 - 64

hsa-mir-92b
CGGGCCCCGGGCGGGCGGGAGGGACGGGACGCGGUGCAGUGUUGUUUUUUCCCCCGCCAAUAUUGCAC
UCGUCCCGGCCUCCGGCCCCCCCGGCCC chr1 155165044 rs12759620 G C
null null 77

hsa-mir-93
CUGGGGGCUCCAAAGUGCUGUUCGUGCAGGUAGUGUGAUUACCCAACCUACUGCUGAGCUAGCACUUC
CCGAGCCCCCGG chr7 99691429 rs72631824 C U 0.00239617 -
42

hsa-mir-93
CUGGGGGCUCCAAAGUGCUGUUCGUGCAGGUAGUGUGAUUACCCAACCUACUGCUGAGCUAGCACUUC
CCGAGCCCCCGG chr7 99691401 . C U 0.000199681 - 70

hsa-mir-93
CUGGGGGCUCCAAAGUGCUGUUCGUGCAGGUAGUGUGAUUACCCAACCUACUGCUGAGCUAGCACUUC
CCGAGCCCCCGG chr7 99691396 . C U 0.000199681 - 75

hsa-mir-93
CUGGGGGCUCCAAAGUGCUGUUCGUGCAGGUAGUGUGAUUACCCAACCUACUGCUGAGCUAGCACUUC
CCGAGCCCCCGG chr7 99691393 rs372015556 C U 0.000199681 -
78

hsa-mir-9-3
GGAGGCCCGUUUCUCUCUUUGGUUAUCUAGCUGUAUGAGUGCCACAGAGCCGUCAUAAAGCUAGAUAA
CCGAAAGUAGAAAUGAUUCUCA chr15 89911256 . G C 0 +
9

hsa-mir-933
ACUUGGGUCAGUUCAGAGGUCCUCGGGGCGCGCGUCGAGUCAGCCGUGUGCGCAGGGAGACCUCUCCC

ACCCACAGU chr2 176032428 . A U 0.000199681 - 10

hsa-mir-933
ACUUGGGUCAGUUCAGAGGUCCUCGGGGCGCGCGUCGAGUCAGCCGUGUGCGCAGGGAGACCUCUCCC
ACCCACAGU chr2 176032424 . C A 0.000199681 - 14

hsa-mir-933
ACUUGGGUCAGUUCAGAGGUCCUCGGGGCGCGCGUCGAGUCAGCCGUGUGCGCAGGGAGACCUCUCCC
ACCCACAGU chr2 176032415 rs373954778 U C 0.000199681 -
23
hsa-mir-933
ACUUGGGUCAGUUCAGAGGUCCUCGGGGCGCGCGUCGAGUCAGCCGUGUGCGCAGGGAGACCUCUCCC
ACCCACAGU chr2 176032408 rs182717619 G U 0.000599042 -
30
hsa-mir-933
ACUUGGGUCAGUUCAGAGGUCCUCGGGGCGCGCGUCGAGUCAGCCGUGUGCGCAGGGAGACCUCUCCC
ACCCACAGU chr2 176032384 rs139770589 A G 0.0061901 -
54
hsa-mir-933
ACUUGGGUCAGUUCAGAGGUCCUCGGGGCGCGCGUCGAGUCAGCCGUGUGCGCAGGGAGACCUCUCCC
ACCCACAGU chr2 176032376 rs79402775 C U 0.0593051 -
62
hsa-mir-934
AGAAAUAAGGCUUCUGUCUACUACUGGAGACACUGGUAGUAUAAAACCCAGAGUCUCCAGUAAUGGAC
GGGAGCCUUAUUUCU chrX 135633045 rs73558572 G A 0.0299338
+ 9
hsa-mir-934
AGAAAUAAGGCUUCUGUCUACUACUGGAGACACUGGUAGUAUAAAACCCAGAGUCUCCAGUAAUGGAC
GGGAGCCUUAUUUCU chrX 135633077 . A C 0.000264901 +
41
hsa-mir-936
UCAAGGCCACUGGGACAGUAGAGGGAGGAAUCGCAGAAAUCACUCCAGGAGCAACUGAGAGACCUUGC
UUCUACUUUACCAGGUCCUGCUGGCCCAGA chr10 105807928 rs79924817 A G
0.00798722 - 17
hsa-mir-936
UCAAGGCCACUGGGACAGUAGAGGGAGGAAUCGCAGAAAUCACUCCAGGAGCAACUGAGAGACCUUGC
UUCUACUUUACCAGGUCCUGCUGGCCCAGA chr10 105807912 rs189063749 G A
0.000399361 - 33
hsa-mir-936
UCAAGGCCACUGGGACAGUAGAGGGAGGAAUCGCAGAAAUCACUCCAGGAGCAACUGAGAGACCUUGC
UUCUACUUUACCAGGUCCUGCUGGCCCAGA chr10 105807909 rs140657237 G A
0.00379393 - 36
hsa-mir-936
UCAAGGCCACUGGGACAGUAGAGGGAGGAAUCGCAGAAAUCACUCCAGGAGCAACUGAGAGACCUUGC
UUCUACUUUACCAGGUCCUGCUGGCCCAGA chr10 105807864 rs112526889 A G
null null 81
hsa-mir-936

UCAAGGCCACUGGGACAGUAGAGGGAGGAAUCGCAGAAAUCACUCCAGGAGCAACUGAGAGACCUUGC UUCUACUUUACCAGGUCCUGCUGGCCCAGA chr10 105807858 rs145823228 U C 0.000199681 - 87
hsa-mir-936
UCAAGGCCACUGGGACAGUAGAGGGAGGAAUCGCAGAAAUCACUCCAGGAGCAACUGAGAGACCUUGC UUCUACUUUACCAGGUCCUGCUGGCCCAGA chr10 105807855 rs149841873 U G 0.00179712 - 90
hsa-mir-937
AGCACUGCCCCCGGUGAGUCAGGGUGGGGCUGGCCCCCUGCUUCGUGCCCAUCCGCGCUCUGACUCUC UGCCCACCUGCAGGAGCU chr8 144895179 rs201884898 C U 0.000199681 - 34
hsa-mir-937
AGCACUGCCCCCGGUGAGUCAGGGUGGGGCUGGCCCCCUGCUUCGUGCCCAUCCGCGCUCUGACUCUC UGCCCACCUGCAGGAGCU chr8 144895170 rs199722919 U C 0.000199681 - 43
hsa-mir-937
AGCACUGCCCCCGGUGAGUCAGGGUGGGGCUGGCCCCCUGCUUCGUGCCCAUCCGCGCUCUGACUCUC UGCCCACCUGCAGGAGCU chr8 144895169 . C U 0.000399361 - 44
hsa-mir-937
AGCACUGCCCCCGGUGAGUCAGGGUGGGGCUGGCCCCCUGCUUCGUGCCCAUCCGCGCUCUGACUCUC UGCCCACCUGCAGGAGCU chr8 144895168 rs200654758 G A 0.000199681 - 45
hsa-mir-937
AGCACUGCCCCCGGUGAGUCAGGGUGGGGCUGGCCCCCUGCUUCGUGCCCAUCCGCGCUCUGACUCUC UGCCCACCUGCAGGAGCU chr8 144895164 rs375199812 C G 0.000399361 - 49
hsa-mir-937
AGCACUGCCCCCGGUGAGUCAGGGUGGGGCUGGCCCCCUGCUUCGUGCCCAUCCGCGCUCUGACUCUC UGCCCACCUGCAGGAGCU chr8 144895159 . C U 0.000199681 - 54
hsa-mir-938
GAAGGUGUACCAUGUGCCCUUAAAGGUGAACCCAGUGCACCUUCAUGAACCGUGGUACACCUUUAAGA ACUUGGUAUGCCUUC chr10 29891262 . G A 0.000199681 - 14
hsa-mir-938
GAAGGUGUACCAUGUGCCCUUAAAGGUGAACCCAGUGCACCUUCAUGAACCGUGGUACACCUUUAAGA ACUUGGUAUGCCUUC chr10 29891260 rs12416605 G A 0.125 - 16
hsa-mir-938
GAAGGUGUACCAUGUGCCCUUAAAGGUGAACCCAGUGCACCUUCAUGAACCGUGGUACACCUUUAAGA ACUUGGUAUGCCUUC chr10 29891245 . C U 0.000199681 - 31
hsa-mir-938
GAAGGUGUACCAUGUGCCCUUAAAGGUGAACCCAGUGCACCUUCAUGAACCGUGGUACACCUUUAAGA ACUUGGUAUGCCUUC chr10 29891235 . C U 0.000199681 - 41

hsa-mir-939
UGUGGGCAGGGCCCUGGGGAGCUGAGGCUCUGGGGGUGGCCGGGGCUGACCCUGGGCCUCUGCUCCCC
AGUGUCUGACCGCG chr8 145619442 . G A 0.000399361 -
4
hsa-mir-939
UGUGGGCAGGGCCCUGGGGAGCUGAGGCUCUGGGGGUGGCCGGGGCUGACCCUGGGCCUCUGCUCCCC
AGUGUCUGACCGCG chr8 145619405 rs112257111 C U null
null 41
hsa-mir-939
UGUGGGCAGGGCCCUGGGGAGCUGAGGCUCUGGGGGUGGCCGGGGCUGACCCUGGGCCUCUGCUCCCC
AGUGUCUGACCGCG chr8 145619386 . C U 0.000199681 -
60
hsa-mir-939
UGUGGGCAGGGCCCUGGGGAGCUGAGGCUCUGGGGGUGGCCGGGGCUGACCCUGGGCCUCUGCUCCCC
AGUGUCUGACCGCG chr8 145619377 rs35486628 - C null
null 69
hsa-mir-939
UGUGGGCAGGGCCCUGGGGAGCUGAGGCUCUGGGGGUGGCCGGGGCUGACCCUGGGCCUCUGCUCCCC
AGUGUCUGACCGCG chr8 145619365 rs149434817 C U
0.000399361 - 81
hsa-mir-940
GUGAGGUGUGGGCCCGGCCCCAGGAGCGGGGCCUGGGCAGCCCCGUGUGUUGAGGAAGGAAGGCAGGG
CCCCCGCUCCCCGGGCCUGACCCCACchr16 2321762 rs372445685 C U
0.000399361 + 15
hsa-mir-940
GUGAGGUGUGGGCCCGGCCCCAGGAGCGGGGCCUGGGCAGCCCCGUGUGUUGAGGAAGGAAGGCAGGG
CCCCCGCUCCCCGGGCCUGACCCCACchr16 2321774 rs373947923 C U
0.000199681 + 27
hsa-mir-940
GUGAGGUGUGGGCCCGGCCCCAGGAGCGGGGCCUGGGCAGCCCCGUGUGUUGAGGAAGGAAGGCAGGG
CCCCCGCUCCCCGGGCCUGACCCCACchr16 2321792 . G A
0.000399361 + 45
hsa-mir-940
GUGAGGUGUGGGCCCGGCCCCAGGAGCGGGGCCUGGGCAGCCCCGUGUGUUGAGGAAGGAAGGCAGGG
CCCCCGCUCCCCGGGCCUGACCCCACchr16 2321809 rs149527765 G A
0.000199681 + 62
hsa-mir-940
GUGAGGUGUGGGCCCGGCCCCAGGAGCGGGGCCUGGGCAGCCCCGUGUGUUGAGGAAGGAAGGCAGGG
CCCCCGCUCCCCGGGCCUGACCCCACchr16 2321820 rs35356504 C -
null null 73
hsa-mir-940
GUGAGGUGUGGGCCCGGCCCCAGGAGCGGGGCCUGGGCAGCCCCGUGUGUUGAGGAAGGAAGGCAGGG
CCCCCGCUCCCCGGGCCUGACCCCACchr16 2321827 rs373106490 C U
0.000399361 + 80
hsa-mir-941-1
UGUGGACAUGUGCCCAGGGCCCGGGACAGCGCCACGGAAGAGGACGCACCCGGCUGUGUGCACAUGUG
CCCA chr20 62550880 rs6089780 G A null null 3

hsa-mir-941-1
UGUGGACAUGUGCCCAGGGCCCGGGACAGCGCCACGGAAGAGGACGCACCCGGCUGUGUGCACAUGUG
CCCA chr20 62550780 rs4809383 C U null null 3

hsa-mir-941-1
UGUGGACAUGUGCCCAGGGCCCGGGACAGCGCCACGGAAGAGGACGCACCCGGCUGUGUGCACAUGUG
CCCA chr20 62550890 rs6090019 C U null null 13

hsa-mir-941-1
UGUGGACAUGUGCCCAGGGCCCGGGACAGCGCCACGGAAGAGGACGCACCCGGCUGUGUGCACAUGUG
CCCA chr20 62550824 rs2427556 G A 0.455072 + 23

hsa-mir-941-1
UGUGGACAUGUGCCCAGGGCCCGGGACAGCGCCACGGAAGAGGACGCACCCGGCUGUGUGCACAUGUG
CCCA chr20 62550859 rs111479365 G A null null 82

hsa-mir-941-1
UGUGGACAUGUGCCCAGGGCCCGGGACAGCGCCACGGAAGAGGACGCACCCGGCUGUGUGCACAUGUG
CCCA chr20 62550862 rs113698386 C G null null 85

hsa-mir-941-3
UGUGCACAUGUGCCCAGGGCCCGGGACAGCGCCACGGAAGAGGACGCACCCGGCUGUGUGCACAUGUG
CCCA chr20 62551110 rs113587130 G A null null 26

hsa-mir-941-3
UGUGCACAUGUGCCCAGGGCCCGGGACAGCGCCACGGAAGAGGACGCACCCGGCUGUGUGCACAUGUG
CCCA chr20 62551113 rs113258585 C G null null 29

hsa-mir-941-3
UGUGCACAUGUGCCCAGGGCCCGGGACAGCGCCACGGAAGAGGACGCACCCGGCUGUGUGCACAUGUG
CCCA chr20 62551199 rs7360929 U C null null 29

hsa-mir-941-3
UGUGCACAUGUGCCCAGGGCCCGGGACAGCGCCACGGAAGAGGACGCACCCGGCUGUGUGCACAUGUG
CCCA chr20 62551160 rs113672516 G A null null 76

hsa-mir-941-3
UGUGCACAUGUGCCCAGGGCCCGGGACAGCGCCACGGAAGAGGACGCACCCGGCUGUGUGCACAUGUG
CCCA chr20 62551166 rs111993072 G A null null 82

hsa-mir-941-3
UGUGCACAUGUGCCCAGGGCCCGGGACAGCGCCACGGAAGAGGACGCACCCGGCUGUGUGCACAUGUG
CCCA chr20 62551169 rs34604519 C G null null 85

hsa-mir-941-4
UGUGCACAUGUGCCCAGGGCCCGGGACAGCGCCACGGAAGAGGACGCACCCGGCUGUGUGCACAUGUG

CCCA chr20 62551199 rs7360929 U C null null 3

hsa-mir-941-4
UGUGCACAUGUGCCCAGGGCCCGGGACAGCGCCACGGAAGAGGACGCACCCGGCUGUGUGCACAUGUG
CCCA chr20 62551216 rs76606291 G A null null 20

hsa-mir-941-4
UGUGCACAUGUGCCCAGGGCCCGGGACAGCGCCACGGAAGAGGACGCACCCGGCUGUGUGCACAUGUG
CCCA chr20 62551272 rs35544770 G A null null 26

hsa-mir-941-4
UGUGCACAUGUGCCCAGGGCCCGGGACAGCGCCACGGAAGAGGACGCACCCGGCUGUGUGCACAUGUG
CCCA chr20 62551255 rs112205797 C U null null 59

hsa-mir-941-4
UGUGCACAUGUGCCCAGGGCCCGGGACAGCGCCACGGAAGAGGACGCACCCGGCUGUGUGCACAUGUG
CCCA chr20 62551278 rs111586745 G A null null 82

hsa-mir-941-4
UGUGCACAUGUGCCCAGGGCCCGGGACAGCGCCACGGAAGAGGACGCACCCGGCUGUGUGCACAUGUG
CCCA chr20 62551222 rs111696059 G A null null 85

hsa-mir-941-5
UGUGCACAUGUGCCCAGGGCCCGGGACAGCGCCACGGAAGAGGACGCACCCGGCUGUGUGCACAUGUG
CCCA chr20 62551225 rs12625445 C G 0.00219649 + 5

hsa-mir-941-5
UGUGCACAUGUGCCCAGGGCCCGGGACAGCGCCACGGAAGAGGACGCACCCGGCUGUGUGCACAUGUG
CCCA chr20 62551265 . C U 0.000599042 + 45

hsa-mir-942
AUUAGGAGAGUAUCUUCUCUGUUUUGGCCAUGUGUGUACUCACAGCCCCUCACACAUGGCCGAAACAG
AGAAGUUACUUUCCUAAU chr1 117637290 rs116426341 G U null
null 26
hsa-mir-942
AUUAGGAGAGUAUCUUCUCUGUUUUGGCCAUGUGUGUACUCACAGCCCCUCACACAUGGCCGAAACAG
AGAAGUUACUUUCCUAAU chr1 117637302 rs371128981 A G
0.000199681 + 38
hsa-mir-942
AUUAGGAGAGUAUCUUCUCUGUUUUGGCCAUGUGUGUACUCACAGCCCCUCACACAUGGCCGAAACAG
AGAAGUUACUUUCCUAAU chr1 117637325 rs115372145 C U 0.0131789
+ 61
hsa-mir-942
AUUAGGAGAGUAUCUUCUCUGUUUUGGCCAUGUGUGUACUCACAGCCCCUCACACAUGGCCGAAACAG
AGAAGUUACUUUCCUAAU chr1 117637326 . G A 0.000199681 +
62
hsa-mir-943

GGGACGUUCUGAGCUCGGGGUGGGGGACGUUUGCCGGUCACUGCUGCUGGCGCCCUGACUGUUGCCGU
CCUCCAGCCCCACUCAAAGGCAUCCCchr4 1988193 rs1077020 A G
0.250399 - 12
hsa-mir-943
GGGACGUUCUGAGCUCGGGGUGGGGGACGUUUGCCGGUCACUGCUGCUGGCGCCCUGACUGUUGCCGU
CCUCCAGCCCCACUCAAAGGCAUCCCchr4 1988176 rs368905227 G A
0.000399361 - 29
hsa-mir-943
GGGACGUUCUGAGCUCGGGGUGGGGGACGUUUGCCGGUCACUGCUGCUGGCGCCCUGACUGUUGCCGU
CCUCCAGCCCCACUCAAAGGCAUCCCchr4 1988170 rs201974342 C U
0.000199681 - 35
hsa-mir-943
GGGACGUUCUGAGCUCGGGGUGGGGGACGUUUGCCGGUCACUGCUGCUGGCGCCCUGACUGUUGCCGU
CCUCCAGCCCCACUCAAAGGCAUCCCchr4 1988144 rs186568343 G U
0.000599042 - 61
hsa-mir-943
GGGACGUUCUGAGCUCGGGGUGGGGGACGUUUGCCGGUCACUGCUGCUGGCGCCCUGACUGUUGCCGU
CCUCCAGCCCCACUCAAAGGCAUCCCchr4 1988119 rs200107238 A G
0.000599042 - 86
hsa-mir-943
GGGACGUUCUGAGCUCGGGGUGGGGGACGUUUGCCGGUCACUGCUGCUGGCGCCCUGACUGUUGCCGU
CCUCCAGCCCCACUCAAAGGCAUCCCchr4 1988116 rs142924150 C U
0.000399361 - 89
hsa-mir-944
GUUCCAGACACAUCUCAUCUGAUAUACAAUAUUUUCUUAAAUUGUAUAAAGAGAAAUUAUUGUACAUC
GGAUGAGCUGUGUCUGGGAU chr3 189547735 rs75715827 U C
0.0339457 + 25
hsa-mir-944
GUUCCAGACACAUCUCAUCUGAUAUACAAUAUUUUCUUAAAUUGUAUAAAGAGAAAUUAUUGUACAUC
GGAUGAGCUGUGUCUGGGAU chr3 189547748 . U C
0.000199681 + 38
hsa-mir-944
GUUCCAGACACAUCUCAUCUGAUAUACAAUAUUUUCUUAAAUUGUAUAAAGAGAAAUUAUUGUACAUC
GGAUGAGCUGUGUCUGGGAU chr3 189547761 rs186331124 G C
0.000199681 + 51
hsa-mir-944
GUUCCAGACACAUCUCAUCUGAUAUACAAUAUUUUCUUAAAUUGUAUAAAGAGAAAUUAUUGUACAUC
GGAUGAGCUGUGUCUGGGAU chr3 189547778 rs77473380 C U
0.00239617 + 68
hsa-mir-944
GUUCCAGACACAUCUCAUCUGAUAUACAAUAUUUUCUUAAAUUGUAUAAAGAGAAAUUAUUGUACAUC
GGAUGAGCUGUGUCUGGGAU chr3 189547779 rs190786647 G C
0.000199681 + 69
hsa-mir-95
AACACAGUGGGCACUCAAUAAAUGUCUGUUGAAUUGAAAUGCGUUACAUUCAACGGGUAUUUAUUGAG
CACCCACUCUGUGchr4 8007104 rs188573591 C G 0.000199681 -
5

hsa-mir-95
AACACAGUGGGCACUCAAUAAAUGUCUGUUGAAUUGAAAUGCGUUACAUUCAACGGGUAUUUAUUGAG
CACCCACUCUGUGchr4 8007067 rs376530569 C U 0.00199681 -
42
hsa-mir-95
AACACAGUGGGCACUCAAUAAAUGUCUGUUGAAUUGAAAUGCGUUACAUUCAACGGGUAUUUAUUGAG
CACCCACUCUGUGchr4 8007066 rs183460242 G A 0.000199681 -
43
hsa-mir-95
AACACAGUGGGCACUCAAUAAAUGUCUGUUGAAUUGAAAUGCGUUACAUUCAACGGGUAUUUAUUGAG
CACCCACUCUGUGchr4 8007039 . A G 0.000199681 - 70

hsa-mir-95
AACACAGUGGGCACUCAAUAAAUGUCUGUUGAAUUGAAAUGCGUUACAUUCAACGGGUAUUUAUUGAG
CACCCACUCUGUGchr4 8007037 rs371266077 C U 0.000599042 -
72
hsa-mir-96
UGGCCGAUUUUGGCACUAGCACAUUUUUGCUUGUGUCUCUCCGCUCUGAGCAAUCAUGUGCAGUGCCA
AUAUGGGAAA chr7 129414574 rs41274239 U C 0.000998403 -
36
hsa-mir-96
UGGCCGAUUUUGGCACUAGCACAUUUUUGCUUGUGUCUCUCCGCUCUGAGCAAUCAUGUGCAGUGCCA
AUAUGGGAAA chr7 129414568 rs73159662 C U 0.00179712 -
42
hsa-mir-96
UGGCCGAUUUUGGCACUAGCACAUUUUUGCUUGUGUCUCUCCGCUCUGAGCAAUCAUGUGCAGUGCCA
AUAUGGGAAA chr7 129414553 . U C 0.000199681 - 57

hsa-mir-98
AGGAUUCUGCUCAUGCCAGGGUGAGGUAGUAAGUUGUAUUGUUGUGGGGUAGGGAUAUUAGGCCCCAA
UUAGAAGAUAACUAUACAACUUACUACUUUCCCUGGUGUGUGGCAUAUUCA chrX 53583294 .
G A 0.000529801 - 9
hsa-mir-99a
CCCAUUGGCAUAAACCCGUAGAUCCGAUCUUGUGGUGAAGUGGACCGCACAAGCUCGCUUCUAUGGGU
CUGUGUCAGUGUGchr21 17911447 . A U 0.000199681 + 39

hsa-mir-99a
CCCAUUGGCAUAAACCCGUAGAUCCGAUCUUGUGGUGAAGUGGACCGCACAAGCUCGCUUCUAUGGGU
CUGUGUCAGUGUGchr21 17911448 rs374130229 G A 0.000199681 +
40
hsa-mir-99b
GGCACCCACCCGUAGAACCGACCUUGCGGGGCCUUCGCCGCACACAAGCUCGUGUCUGUGGGUCCGUG
UC chr19 52195904 rs190452290 G A 0.000599042 + 40
