# Supplementary material for: Role of SNPs in the Biogenesis of Mature miRNAs
Source: Biomed Res Int. 2021 Jun 17;2021:2403418. doi: 10.1155/2021/2403418 (PMC8233088; doi:10.1155/2021/2403418)
Supplement: Supplementary Materials — Additional file 1 Table S1: the database miRSNPBase (xls). Additional file 2 Table S2: the list of pre-miRNAs in miRSNPBase which is classified based on mature miRNA in the 5′ arm or 3′ arm (xls). Additional file 3 Table S3: all the iso-pre-miRNAs, nor-pre-miRNAs, nor-SNPs, and iso-SNPs associated with four splicing sites (xls). Additional file 4 Table S4: the pre-miRNAs and SNPs associated with the normal and isomiRs (xls). Additional file 5 Table S5: the pre-miRNAs, iso-SNPs, and isomiRs of HG00097 (xls). Additional file 6 Table S6: the isomiRs and iso-SNP of 18 GBR populations (xls). Additional file 7 Table S7: the verified isomiRs of 18 GBR (xls). Additional file 8 Table S8: the iso-pre-miRNA candidates and the verified iso-pre-miRNAs of 18 GBR samples (xls). [file 2403418.f1.zip › 2403418.f1/Supp Tab S2.pdf]

5'SNP-pre-miRNA	hsa-let-7a-2
5'SNP-pre-miRNA	hsa-let-7b
5'SNP-pre-miRNA	hsa-let-7e
5'SNP-pre-miRNA	hsa-let-7i
5'SNP-pre-miRNA	hsa-mir-105-1
5'SNP-pre-miRNA	hsa-mir-105-2
5'SNP-pre-miRNA	hsa-mir-106b
5'SNP-pre-miRNA	hsa-mir-10a
5'SNP-pre-miRNA	hsa-mir-10b
5'SNP-pre-miRNA	hsa-mir-1178
5'SNP-pre-miRNA	hsa-mir-1179
5'SNP-pre-miRNA	hsa-mir-1180
5'SNP-pre-miRNA	hsa-mir-1181
5'SNP-pre-miRNA	hsa-mir-1185-1
5'SNP-pre-miRNA	hsa-mir-1185-2
5'SNP-pre-miRNA	hsa-mir-1193
5'SNP-pre-miRNA	hsa-mir-1199
5'SNP-pre-miRNA	hsa-mir-1200
5'SNP-pre-miRNA	hsa-mir-1202
5'SNP-pre-miRNA	hsa-mir-1203
5'SNP-pre-miRNA	hsa-mir-1204
5'SNP-pre-miRNA	hsa-mir-1205
5'SNP-pre-miRNA	hsa-mir-1206
5'SNP-pre-miRNA	hsa-mir-1207
5'SNP-pre-miRNA	hsa-mir-1208
5'SNP-pre-miRNA	hsa-mir-122
5'SNP-pre-miRNA	hsa-mir-1224
5'SNP-pre-miRNA	hsa-mir-1225
5'SNP-pre-miRNA	hsa-mir-1226
5'SNP-pre-miRNA	hsa-mir-1227
5'SNP-pre-miRNA	hsa-mir-1228
5'SNP-pre-miRNA	hsa-mir-1229
5'SNP-pre-miRNA	hsa-mir-1231
5'SNP-pre-miRNA	hsa-mir-1233-1
5'SNP-pre-miRNA	hsa-mir-1233-2
5'SNP-pre-miRNA	hsa-mir-1236
5'SNP-pre-miRNA	hsa-mir-1237
5'SNP-pre-miRNA	hsa-mir-1238
5'SNP-pre-miRNA	hsa-mir-124-2
5'SNP-pre-miRNA	hsa-mir-1243
5'SNP-pre-miRNA	hsa-mir-124-3
5'SNP-pre-miRNA	hsa-mir-1245b
5'SNP-pre-miRNA	hsa-mir-1246
5'SNP-pre-miRNA	hsa-mir-1247
5'SNP-pre-miRNA	hsa-mir-1248
5'SNP-pre-miRNA	hsa-mir-1250
5'SNP-pre-miRNA	hsa-mir-1251

5’SNP-pre-miRNA	hsa-mir-1252
5’SNP-pre-miRNA	hsa-mir-1253
5’SNP-pre-miRNA	hsa-mir-1254-1
5’SNP-pre-miRNA	hsa-mir-1254-2
5’SNP-pre-miRNA	hsa-mir-1255a
5’SNP-pre-miRNA	hsa-mir-1255b-1
5’SNP-pre-miRNA	hsa-mir-1255b-2
5’SNP-pre-miRNA	hsa-mir-1257
5’SNP-pre-miRNA	hsa-mir-125a
5’SNP-pre-miRNA	hsa-mir-125b-2
5’SNP-pre-miRNA	hsa-mir-126
5’SNP-pre-miRNA	hsa-mir-1260a
5’SNP-pre-miRNA	hsa-mir-1260b
5’SNP-pre-miRNA	hsa-mir-1261
5’SNP-pre-miRNA	hsa-mir-1262
5’SNP-pre-miRNA	hsa-mir-1263
5’SNP-pre-miRNA	hsa-mir-1265
5’SNP-pre-miRNA	hsa-mir-1267
5’SNP-pre-miRNA	hsa-mir-1268a
5’SNP-pre-miRNA	hsa-mir-1268b
5’SNP-pre-miRNA	hsa-mir-1269b
5’SNP-pre-miRNA	hsa-mir-1272
5’SNP-pre-miRNA	hsa-mir-1273c
5’SNP-pre-miRNA	hsa-mir-1273d
5’SNP-pre-miRNA	hsa-mir-1273f
5’SNP-pre-miRNA	hsa-mir-1273h
5’SNP-pre-miRNA	hsa-mir-1275
5’SNP-pre-miRNA	hsa-mir-1276
5’SNP-pre-miRNA	hsa-mir-1277
5’SNP-pre-miRNA	hsa-mir-1279
5’SNP-pre-miRNA	hsa-mir-128-1
5’SNP-pre-miRNA	hsa-mir-1282
5’SNP-pre-miRNA	hsa-mir-1283-1
5’SNP-pre-miRNA	hsa-mir-1283-2
5’SNP-pre-miRNA	hsa-mir-1284
5’SNP-pre-miRNA	hsa-mir-1287
5’SNP-pre-miRNA	hsa-mir-1291
5’SNP-pre-miRNA	hsa-mir-129-1
5’SNP-pre-miRNA	hsa-mir-1292
5’SNP-pre-miRNA	hsa-mir-129-2
5’SNP-pre-miRNA	hsa-mir-1293
5’SNP-pre-miRNA	hsa-mir-1295b
5’SNP-pre-miRNA	hsa-mir-1301
5’SNP-pre-miRNA	hsa-mir-1304
5’SNP-pre-miRNA	hsa-mir-1307
5’SNP-pre-miRNA	hsa-mir-130b
5’SNP-pre-miRNA	hsa-mir-1323

5’SNP-pre-miRNA	hsa-mir-133a-1
5’SNP-pre-miRNA	hsa-mir-133a-2
5’SNP-pre-miRNA	hsa-mir-134
5’SNP-pre-miRNA	hsa-mir-1343
5’SNP-pre-miRNA	hsa-mir-135a-1
5’SNP-pre-miRNA	hsa-mir-135a-2
5’SNP-pre-miRNA	hsa-mir-135b
5’SNP-pre-miRNA	hsa-mir-136
5’SNP-pre-miRNA	hsa-mir-138-1
5’SNP-pre-miRNA	hsa-mir-138-2
5’SNP-pre-miRNA	hsa-mir-140
5’SNP-pre-miRNA	hsa-mir-141
5’SNP-pre-miRNA	hsa-mir-142
5’SNP-pre-miRNA	hsa-mir-143
5’SNP-pre-miRNA	hsa-mir-145
5’SNP-pre-miRNA	hsa-mir-1468
5’SNP-pre-miRNA	hsa-mir-1469
5’SNP-pre-miRNA	hsa-mir-146a
5’SNP-pre-miRNA	hsa-mir-146b
5’SNP-pre-miRNA	hsa-mir-1470
5’SNP-pre-miRNA	hsa-mir-148b
5’SNP-pre-miRNA	hsa-mir-149
5’SNP-pre-miRNA	hsa-mir-151a
5’SNP-pre-miRNA	hsa-mir-152
5’SNP-pre-miRNA	hsa-mir-153-2
5’SNP-pre-miRNA	hsa-mir-1537
5’SNP-pre-miRNA	hsa-mir-154
5’SNP-pre-miRNA	hsa-mir-155
5’SNP-pre-miRNA	hsa-mir-1587
5’SNP-pre-miRNA	hsa-mir-15b
5’SNP-pre-miRNA	hsa-mir-16-1
5’SNP-pre-miRNA	hsa-mir-17
5’SNP-pre-miRNA	hsa-mir-181a-2
5’SNP-pre-miRNA	hsa-mir-181b-1
5’SNP-pre-miRNA	hsa-mir-181b-2
5’SNP-pre-miRNA	hsa-mir-181c
5’SNP-pre-miRNA	hsa-mir-181d
5’SNP-pre-miRNA	hsa-mir-182
5’SNP-pre-miRNA	hsa-mir-183
5’SNP-pre-miRNA	hsa-mir-185
5’SNP-pre-miRNA	hsa-mir-187
5’SNP-pre-miRNA	hsa-mir-188
5’SNP-pre-miRNA	hsa-mir-18a
5’SNP-pre-miRNA	hsa-mir-1908
5’SNP-pre-miRNA	hsa-mir-1909
5’SNP-pre-miRNA	hsa-mir-190a
5’SNP-pre-miRNA	hsa-mir-190b

5’SNP-pre-miRNA	hsa-mir-1910
5’SNP-pre-miRNA	hsa-mir-1911
5’SNP-pre-miRNA	hsa-mir-1914
5’SNP-pre-miRNA	hsa-mir-1915
5’SNP-pre-miRNA	hsa-mir-192
5’SNP-pre-miRNA	hsa-mir-193a
5’SNP-pre-miRNA	hsa-mir-194-1
5’SNP-pre-miRNA	hsa-mir-194-2
5’SNP-pre-miRNA	hsa-mir-195
5’SNP-pre-miRNA	hsa-mir-196a-1
5’SNP-pre-miRNA	hsa-mir-196a-2
5’SNP-pre-miRNA	hsa-mir-196b
5’SNP-pre-miRNA	hsa-mir-197
5’SNP-pre-miRNA	hsa-mir-198
5’SNP-pre-miRNA	hsa-mir-199a-1
5’SNP-pre-miRNA	hsa-mir-199a-2
5’SNP-pre-miRNA	hsa-mir-199b
5’SNP-pre-miRNA	hsa-mir-19b-2
5’SNP-pre-miRNA	hsa-mir-200a
5’SNP-pre-miRNA	hsa-mir-200b
5’SNP-pre-miRNA	hsa-mir-202
5’SNP-pre-miRNA	hsa-mir-203b
5’SNP-pre-miRNA	hsa-mir-204
5’SNP-pre-miRNA	hsa-mir-205
5’SNP-pre-miRNA	hsa-mir-208b
5’SNP-pre-miRNA	hsa-mir-20a
5’SNP-pre-miRNA	hsa-mir-20b
5’SNP-pre-miRNA	hsa-mir-21
5’SNP-pre-miRNA	hsa-mir-210
5’SNP-pre-miRNA	hsa-mir-211
5’SNP-pre-miRNA	hsa-mir-2110
5’SNP-pre-miRNA	hsa-mir-2114
5’SNP-pre-miRNA	hsa-mir-2115
5’SNP-pre-miRNA	hsa-mir-2116
5’SNP-pre-miRNA	hsa-mir-212
5’SNP-pre-miRNA	hsa-mir-215
5’SNP-pre-miRNA	hsa-mir-216a
5’SNP-pre-miRNA	hsa-mir-216b
5’SNP-pre-miRNA	hsa-mir-218-1
5’SNP-pre-miRNA	hsa-mir-218-2
5’SNP-pre-miRNA	hsa-mir-219a-1
5’SNP-pre-miRNA	hsa-mir-219a-2
5’SNP-pre-miRNA	hsa-mir-219b
5’SNP-pre-miRNA	hsa-mir-221
5’SNP-pre-miRNA	hsa-mir-222
5’SNP-pre-miRNA	hsa-mir-223
5’SNP-pre-miRNA	hsa-mir-224

5’SNP-pre-miRNA	hsa-mir-2276
5’SNP-pre-miRNA	hsa-mir-2277
5’SNP-pre-miRNA	hsa-mir-2278
5’SNP-pre-miRNA	hsa-mir-2355
5’SNP-pre-miRNA	hsa-mir-23a
5’SNP-pre-miRNA	hsa-mir-23b
5’SNP-pre-miRNA	hsa-mir-24-1
5’SNP-pre-miRNA	hsa-mir-24-2
5’SNP-pre-miRNA	hsa-mir-2467
5’SNP-pre-miRNA	hsa-mir-25
5’SNP-pre-miRNA	hsa-mir-2681
5’SNP-pre-miRNA	hsa-mir-2682
5’SNP-pre-miRNA	hsa-mir-26a-1
5’SNP-pre-miRNA	hsa-mir-26b
5’SNP-pre-miRNA	hsa-mir-27a
5’SNP-pre-miRNA	hsa-mir-27b
5’SNP-pre-miRNA	hsa-mir-28
5’SNP-pre-miRNA	hsa-mir-2909
5’SNP-pre-miRNA	hsa-mir-296
5’SNP-pre-miRNA	hsa-mir-297
5’SNP-pre-miRNA	hsa-mir-299
5’SNP-pre-miRNA	hsa-mir-301a
5’SNP-pre-miRNA	hsa-mir-302a
5’SNP-pre-miRNA	hsa-mir-302b
5’SNP-pre-miRNA	hsa-mir-302c
5’SNP-pre-miRNA	hsa-mir-302d
5’SNP-pre-miRNA	hsa-mir-302e
5’SNP-pre-miRNA	hsa-mir-3064
5’SNP-pre-miRNA	hsa-mir-3065
5’SNP-pre-miRNA	hsa-mir-3074
5’SNP-pre-miRNA	hsa-mir-30a
5’SNP-pre-miRNA	hsa-mir-30b
5’SNP-pre-miRNA	hsa-mir-30c-2
5’SNP-pre-miRNA	hsa-mir-30d
5’SNP-pre-miRNA	hsa-mir-30e
5’SNP-pre-miRNA	hsa-mir-3117
5’SNP-pre-miRNA	hsa-mir-3119-1
5’SNP-pre-miRNA	hsa-mir-3119-2
5’SNP-pre-miRNA	hsa-mir-3121
5’SNP-pre-miRNA	hsa-mir-3124
5’SNP-pre-miRNA	hsa-mir-3125
5’SNP-pre-miRNA	hsa-mir-3126
5’SNP-pre-miRNA	hsa-mir-3127
5’SNP-pre-miRNA	hsa-mir-3128
5’SNP-pre-miRNA	hsa-mir-3129
5’SNP-pre-miRNA	hsa-mir-3130-1
5’SNP-pre-miRNA	hsa-mir-3130-2

5'SNP-pre-miRNA	hsa-mir-3131
5'SNP-pre-miRNA	hsa-mir-3132
5'SNP-pre-miRNA	hsa-mir-3133
5'SNP-pre-miRNA	hsa-mir-3135a
5'SNP-pre-miRNA	hsa-mir-3135b
5'SNP-pre-miRNA	hsa-mir-3137
5'SNP-pre-miRNA	hsa-mir-3139
5'SNP-pre-miRNA	hsa-mir-3140
5'SNP-pre-miRNA	hsa-mir-3141
5'SNP-pre-miRNA	hsa-mir-3143
5'SNP-pre-miRNA	hsa-mir-3144
5'SNP-pre-miRNA	hsa-mir-3145
5'SNP-pre-miRNA	hsa-mir-3147
5'SNP-pre-miRNA	hsa-mir-3148
5'SNP-pre-miRNA	hsa-mir-3150a
5'SNP-pre-miRNA	hsa-mir-3150b
5'SNP-pre-miRNA	hsa-mir-3151
5'SNP-pre-miRNA	hsa-mir-3152
5'SNP-pre-miRNA	hsa-mir-3156-1
5'SNP-pre-miRNA	hsa-mir-3156-2
5'SNP-pre-miRNA	hsa-mir-3156-3
5'SNP-pre-miRNA	hsa-mir-3157
5'SNP-pre-miRNA	hsa-mir-3158-1
5'SNP-pre-miRNA	hsa-mir-3158-2
5'SNP-pre-miRNA	hsa-mir-3159
5'SNP-pre-miRNA	hsa-mir-3161
5'SNP-pre-miRNA	hsa-mir-3162
5'SNP-pre-miRNA	hsa-mir-3163
5'SNP-pre-miRNA	hsa-mir-3164
5'SNP-pre-miRNA	hsa-mir-3168
5'SNP-pre-miRNA	hsa-mir-3169
5'SNP-pre-miRNA	hsa-mir-3170
5'SNP-pre-miRNA	hsa-mir-3171
5'SNP-pre-miRNA	hsa-mir-3173
5'SNP-pre-miRNA	hsa-mir-3175
5'SNP-pre-miRNA	hsa-mir-3177
5'SNP-pre-miRNA	hsa-mir-3178
5'SNP-pre-miRNA	hsa-mir-3182
5'SNP-pre-miRNA	hsa-mir-3183
5'SNP-pre-miRNA	hsa-mir-3184
5'SNP-pre-miRNA	hsa-mir-3185
5'SNP-pre-miRNA	hsa-mir-3186
5'SNP-pre-miRNA	hsa-mir-3192
5'SNP-pre-miRNA	hsa-mir-3195
5'SNP-pre-miRNA	hsa-mir-3196
5'SNP-pre-miRNA	hsa-mir-3199-1
5'SNP-pre-miRNA	hsa-mir-3199-2

5’SNP-pre-miRNA	hsa-mir-323b
5’SNP-pre-miRNA	hsa-mir-324
5’SNP-pre-miRNA	hsa-mir-325
5’SNP-pre-miRNA	hsa-mir-328
5’SNP-pre-miRNA	hsa-mir-329-1
5’SNP-pre-miRNA	hsa-mir-329-2
5’SNP-pre-miRNA	hsa-mir-330
5’SNP-pre-miRNA	hsa-mir-335
5’SNP-pre-miRNA	hsa-mir-337
5’SNP-pre-miRNA	hsa-mir-338
5’SNP-pre-miRNA	hsa-mir-339
5’SNP-pre-miRNA	hsa-mir-33a
5’SNP-pre-miRNA	hsa-mir-33b
5’SNP-pre-miRNA	hsa-mir-340
5’SNP-pre-miRNA	hsa-mir-342
5’SNP-pre-miRNA	hsa-mir-345
5’SNP-pre-miRNA	hsa-mir-346
5’SNP-pre-miRNA	hsa-mir-34a
5’SNP-pre-miRNA	hsa-mir-3605
5’SNP-pre-miRNA	hsa-mir-3607
5’SNP-pre-miRNA	hsa-mir-361
5’SNP-pre-miRNA	hsa-mir-3612
5’SNP-pre-miRNA	hsa-mir-3614
5’SNP-pre-miRNA	hsa-mir-3617
5’SNP-pre-miRNA	hsa-mir-3619
5’SNP-pre-miRNA	hsa-mir-362
5’SNP-pre-miRNA	hsa-mir-3620
5’SNP-pre-miRNA	hsa-mir-3622a
5’SNP-pre-miRNA	hsa-mir-3622b
5’SNP-pre-miRNA	hsa-mir-363
5’SNP-pre-miRNA	hsa-mir-3652
5’SNP-pre-miRNA	hsa-mir-365a
5’SNP-pre-miRNA	hsa-mir-365b
5’SNP-pre-miRNA	hsa-mir-3663
5’SNP-pre-miRNA	hsa-mir-3664
5’SNP-pre-miRNA	hsa-mir-3667
5’SNP-pre-miRNA	hsa-mir-367
5’SNP-pre-miRNA	hsa-mir-3674
5’SNP-pre-miRNA	hsa-mir-3678
5’SNP-pre-miRNA	hsa-mir-3679
5’SNP-pre-miRNA	hsa-mir-3682
5’SNP-pre-miRNA	hsa-mir-3683
5’SNP-pre-miRNA	hsa-mir-3689b
5’SNP-pre-miRNA	hsa-mir-370
5’SNP-pre-miRNA	hsa-mir-3714
5’SNP-pre-miRNA	hsa-mir-371a
5’SNP-pre-miRNA	hsa-mir-371b

5’SNP-pre-miRNA	hsa-mir-372
5’SNP-pre-miRNA	hsa-mir-373
5’SNP-pre-miRNA	hsa-mir-376a-2
5’SNP-pre-miRNA	hsa-mir-377
5’SNP-pre-miRNA	hsa-mir-378a
5’SNP-pre-miRNA	hsa-mir-378c
5’SNP-pre-miRNA	hsa-mir-378d-2
5’SNP-pre-miRNA	hsa-mir-378h
5’SNP-pre-miRNA	hsa-mir-378i
5’SNP-pre-miRNA	hsa-mir-378j
5’SNP-pre-miRNA	hsa-mir-379
5’SNP-pre-miRNA	hsa-mir-380
5’SNP-pre-miRNA	hsa-mir-381
5’SNP-pre-miRNA	hsa-mir-382
5’SNP-pre-miRNA	hsa-mir-383
5’SNP-pre-miRNA	hsa-mir-3908
5’SNP-pre-miRNA	hsa-mir-3911
5’SNP-pre-miRNA	hsa-mir-3912
5’SNP-pre-miRNA	hsa-mir-3916
5’SNP-pre-miRNA	hsa-mir-3922
5’SNP-pre-miRNA	hsa-mir-3927
5’SNP-pre-miRNA	hsa-mir-3928
5’SNP-pre-miRNA	hsa-mir-3945
5’SNP-pre-miRNA	hsa-mir-409
5’SNP-pre-miRNA	hsa-mir-410
5’SNP-pre-miRNA	hsa-mir-411
5’SNP-pre-miRNA	hsa-mir-412
5’SNP-pre-miRNA	hsa-mir-423
5’SNP-pre-miRNA	hsa-mir-424
5’SNP-pre-miRNA	hsa-mir-425
5’SNP-pre-miRNA	hsa-mir-4258
5’SNP-pre-miRNA	hsa-mir-4260
5’SNP-pre-miRNA	hsa-mir-4267
5’SNP-pre-miRNA	hsa-mir-4269
5’SNP-pre-miRNA	hsa-mir-4270
5’SNP-pre-miRNA	hsa-mir-4277
5’SNP-pre-miRNA	hsa-mir-4280
5’SNP-pre-miRNA	hsa-mir-4283-1
5’SNP-pre-miRNA	hsa-mir-4283-2
5’SNP-pre-miRNA	hsa-mir-4284
5’SNP-pre-miRNA	hsa-mir-4285
5’SNP-pre-miRNA	hsa-mir-4286
5’SNP-pre-miRNA	hsa-mir-4289
5’SNP-pre-miRNA	hsa-mir-4294
5’SNP-pre-miRNA	hsa-mir-4296
5’SNP-pre-miRNA	hsa-mir-4297
5’SNP-pre-miRNA	hsa-mir-4298

5’SNP-pre-miRNA	hsa-mir-4301
5’SNP-pre-miRNA	hsa-mir-4302
5’SNP-pre-miRNA	hsa-mir-4305
5’SNP-pre-miRNA	hsa-mir-4309
5’SNP-pre-miRNA	hsa-mir-431
5’SNP-pre-miRNA	hsa-mir-4317
5’SNP-pre-miRNA	hsa-mir-432
5’SNP-pre-miRNA	hsa-mir-4326
5’SNP-pre-miRNA	hsa-mir-4327
5’SNP-pre-miRNA	hsa-mir-433
5’SNP-pre-miRNA	hsa-mir-449a
5’SNP-pre-miRNA	hsa-mir-449b
5’SNP-pre-miRNA	hsa-mir-449c
5’SNP-pre-miRNA	hsa-mir-450a-1
5’SNP-pre-miRNA	hsa-mir-450a-2
5’SNP-pre-miRNA	hsa-mir-450b
5’SNP-pre-miRNA	hsa-mir-452
5’SNP-pre-miRNA	hsa-mir-454
5’SNP-pre-miRNA	hsa-mir-455
5’SNP-pre-miRNA	hsa-mir-483
5’SNP-pre-miRNA	hsa-mir-484
5’SNP-pre-miRNA	hsa-mir-485
5’SNP-pre-miRNA	hsa-mir-486-2
5’SNP-pre-miRNA	hsa-mir-487a
5’SNP-pre-miRNA	hsa-mir-487b
5’SNP-pre-miRNA	hsa-mir-488
5’SNP-pre-miRNA	hsa-mir-489
5’SNP-pre-miRNA	hsa-mir-490
5’SNP-pre-miRNA	hsa-mir-491
5’SNP-pre-miRNA	hsa-mir-492
5’SNP-pre-miRNA	hsa-mir-493
5’SNP-pre-miRNA	hsa-mir-495
5’SNP-pre-miRNA	hsa-mir-497
5’SNP-pre-miRNA	hsa-mir-499a
5’SNP-pre-miRNA	hsa-mir-499b
5’SNP-pre-miRNA	hsa-mir-500a
5’SNP-pre-miRNA	hsa-mir-500b
5’SNP-pre-miRNA	hsa-mir-501
5’SNP-pre-miRNA	hsa-mir-502
5’SNP-pre-miRNA	hsa-mir-503
5’SNP-pre-miRNA	hsa-mir-504
5’SNP-pre-miRNA	hsa-mir-505
5’SNP-pre-miRNA	hsa-mir-506
5’SNP-pre-miRNA	hsa-mir-508
5’SNP-pre-miRNA	hsa-mir-509-1
5’SNP-pre-miRNA	hsa-mir-509-2
5’SNP-pre-miRNA	hsa-mir-509-3

5'SNP-pre-miRNA	hsa-mir-510
5'SNP-pre-miRNA	hsa-mir-511
5'SNP-pre-miRNA	hsa-mir-512-1
5'SNP-pre-miRNA	hsa-mir-512-2
5'SNP-pre-miRNA	hsa-mir-513a-1
5'SNP-pre-miRNA	hsa-mir-513a-2
5'SNP-pre-miRNA	hsa-mir-513b
5'SNP-pre-miRNA	hsa-mir-513c
5'SNP-pre-miRNA	hsa-mir-514a-1
5'SNP-pre-miRNA	hsa-mir-514a-3
5'SNP-pre-miRNA	hsa-mir-515-1
5'SNP-pre-miRNA	hsa-mir-516a-1
5'SNP-pre-miRNA	hsa-mir-516a-2
5'SNP-pre-miRNA	hsa-mir-516b-1
5'SNP-pre-miRNA	hsa-mir-516b-2
5'SNP-pre-miRNA	hsa-mir-517a
5'SNP-pre-miRNA	hsa-mir-517b
5'SNP-pre-miRNA	hsa-mir-517c
5'SNP-pre-miRNA	hsa-mir-518a-1
5'SNP-pre-miRNA	hsa-mir-518a-2
5'SNP-pre-miRNA	hsa-mir-518c
5'SNP-pre-miRNA	hsa-mir-518d
5'SNP-pre-miRNA	hsa-mir-518e
5'SNP-pre-miRNA	hsa-mir-518f
5'SNP-pre-miRNA	hsa-mir-519a-1
5'SNP-pre-miRNA	hsa-mir-519b
5'SNP-pre-miRNA	hsa-mir-519c
5'SNP-pre-miRNA	hsa-mir-519d
5'SNP-pre-miRNA	hsa-mir-520a
5'SNP-pre-miRNA	hsa-mir-520c
5'SNP-pre-miRNA	hsa-mir-520d
5'SNP-pre-miRNA	hsa-mir-520f
5'SNP-pre-miRNA	hsa-mir-520g
5'SNP-pre-miRNA	hsa-mir-522
5'SNP-pre-miRNA	hsa-mir-523
5'SNP-pre-miRNA	hsa-mir-524
5'SNP-pre-miRNA	hsa-mir-525
5'SNP-pre-miRNA	hsa-mir-526a-1
5'SNP-pre-miRNA	hsa-mir-526a-2
5'SNP-pre-miRNA	hsa-mir-526b
5'SNP-pre-miRNA	hsa-mir-527
5'SNP-pre-miRNA	hsa-mir-532
5'SNP-pre-miRNA	hsa-mir-541
5'SNP-pre-miRNA	hsa-mir-542
5'SNP-pre-miRNA	hsa-mir-545
5'SNP-pre-miRNA	hsa-mir-548a-3
5'SNP-pre-miRNA	hsa-mir-548ab

5’SNP-pre-miRNA	hsa-mir-548ag-2
5’SNP-pre-miRNA	hsa-mir-548ai
5’SNP-pre-miRNA	hsa-mir-548aj-2
5’SNP-pre-miRNA	hsa-mir-548ak
5’SNP-pre-miRNA	hsa-mir-548am
5’SNP-pre-miRNA	hsa-mir-548an
5’SNP-pre-miRNA	hsa-mir-548ao
5’SNP-pre-miRNA	hsa-mir-548ap
5’SNP-pre-miRNA	hsa-mir-548aq
5’SNP-pre-miRNA	hsa-mir-548ar
5’SNP-pre-miRNA	hsa-mir-548as
5’SNP-pre-miRNA	hsa-mir-548at
5’SNP-pre-miRNA	hsa-mir-548au
5’SNP-pre-miRNA	hsa-mir-548av
5’SNP-pre-miRNA	hsa-mir-548aw
5’SNP-pre-miRNA	hsa-mir-548ay
5’SNP-pre-miRNA	hsa-mir-548b
5’SNP-pre-miRNA	hsa-mir-548ba
5’SNP-pre-miRNA	hsa-mir-548c
5’SNP-pre-miRNA	hsa-mir-548d-1
5’SNP-pre-miRNA	hsa-mir-548d-2
5’SNP-pre-miRNA	hsa-mir-548f-1
5’SNP-pre-miRNA	hsa-mir-548g
5’SNP-pre-miRNA	hsa-mir-548h-1
5’SNP-pre-miRNA	hsa-mir-548h-2
5’SNP-pre-miRNA	hsa-mir-548h-3
5’SNP-pre-miRNA	hsa-mir-548h-4
5’SNP-pre-miRNA	hsa-mir-548h-5
5’SNP-pre-miRNA	hsa-mir-659
5’SNP-pre-miRNA	hsa-mir-660
5’SNP-pre-miRNA	hsa-mir-663a
5’SNP-pre-miRNA	hsa-mir-664a
5’SNP-pre-miRNA	hsa-mir-664b
5’SNP-pre-miRNA	hsa-mir-668
5’SNP-pre-miRNA	hsa-mir-670
5’SNP-pre-miRNA	hsa-mir-671
5’SNP-pre-miRNA	hsa-mir-675
5’SNP-pre-miRNA	hsa-mir-676
5’SNP-pre-miRNA	hsa-mir-708
5’SNP-pre-miRNA	hsa-mir-7-1
5’SNP-pre-miRNA	hsa-mir-7-2
5’SNP-pre-miRNA	hsa-mir-7-3
5’SNP-pre-miRNA	hsa-mir-744
5’SNP-pre-miRNA	hsa-mir-758
5’SNP-pre-miRNA	hsa-mir-759
5’SNP-pre-miRNA	hsa-mir-761
5’SNP-pre-miRNA	hsa-mir-766

5’SNP-pre-miRNA	hsa-mir-767
5’SNP-pre-miRNA	hsa-mir-769
5’SNP-pre-miRNA	hsa-mir-770
5’SNP-pre-miRNA	hsa-mir-802
5’SNP-pre-miRNA	hsa-mir-873
5’SNP-pre-miRNA	hsa-mir-874
5’SNP-pre-miRNA	hsa-mir-876
5’SNP-pre-miRNA	hsa-mir-877
5’SNP-pre-miRNA	hsa-mir-885
5’SNP-pre-miRNA	hsa-mir-887
5’SNP-pre-miRNA	hsa-mir-888
5’SNP-pre-miRNA	hsa-mir-890
5’SNP-pre-miRNA	hsa-mir-891a
5’SNP-pre-miRNA	hsa-mir-891b
5’SNP-pre-miRNA	hsa-mir-892c
5’SNP-pre-miRNA	hsa-mir-9-1
5’SNP-pre-miRNA	hsa-mir-9-2
5’SNP-pre-miRNA	hsa-mir-924
5’SNP-pre-miRNA	hsa-mir-92a-1
5’SNP-pre-miRNA	hsa-mir-92a-2
5’SNP-pre-miRNA	hsa-mir-92b
5’SNP-pre-miRNA	hsa-mir-93
5’SNP-pre-miRNA	hsa-mir-9-3
5’SNP-pre-miRNA	hsa-mir-934
5’SNP-pre-miRNA	hsa-mir-936
5’SNP-pre-miRNA	hsa-mir-937
5’SNP-pre-miRNA	hsa-mir-938
5’SNP-pre-miRNA	hsa-mir-939
5’SNP-pre-miRNA	hsa-mir-942
5’SNP-pre-miRNA	hsa-mir-95
5’SNP-pre-miRNA	hsa-mir-96
5’SNP-pre-miRNA	hsa-mir-98
5’SNP-pre-miRNA	hsa-mir-99a
5’SNP-pre-miRNA	hsa-mir-99b
3’SNP-pre-miRNA	>hsa-let-7a-2
3’SNP-pre-miRNA	>hsa-let-7b
3’SNP-pre-miRNA	>hsa-let-7e
3’SNP-pre-miRNA	>hsa-let-7i
3’SNP-pre-miRNA	>hsa-mir-101-2
3’SNP-pre-miRNA	>hsa-mir-105-1
3’SNP-pre-miRNA	>hsa-mir-105-2
3’SNP-pre-miRNA	>hsa-mir-106b
3’SNP-pre-miRNA	>hsa-mir-107
3’SNP-pre-miRNA	>hsa-mir-10a
3’SNP-pre-miRNA	>hsa-mir-10b
3’SNP-pre-miRNA	>hsa-mir-1-1
3’SNP-pre-miRNA	>hsa-mir-1178

3'SNP-pre-miRNA	>hsa-mir-1180
3'SNP-pre-miRNA	>hsa-mir-1182
3'SNP-pre-miRNA	>hsa-mir-1183
3'SNP-pre-miRNA	>hsa-mir-1185-1
3'SNP-pre-miRNA	>hsa-mir-1185-2
3'SNP-pre-miRNA	>hsa-mir-1197
3'SNP-pre-miRNA	>hsa-mir-1199
3'SNP-pre-miRNA	>hsa-mir-1207
3'SNP-pre-miRNA	>hsa-mir-122
3'SNP-pre-miRNA	>hsa-mir-1224
3'SNP-pre-miRNA	>hsa-mir-1225
3'SNP-pre-miRNA	>hsa-mir-1226
3'SNP-pre-miRNA	>hsa-mir-1227
3'SNP-pre-miRNA	>hsa-mir-1228
3'SNP-pre-miRNA	>hsa-mir-1229
3'SNP-pre-miRNA	>hsa-mir-1233-1
3'SNP-pre-miRNA	>hsa-mir-1233-2
3'SNP-pre-miRNA	>hsa-mir-1234
3'SNP-pre-miRNA	>hsa-mir-1236
3'SNP-pre-miRNA	>hsa-mir-1237
3'SNP-pre-miRNA	>hsa-mir-1238
3'SNP-pre-miRNA	>hsa-mir-124-2
3'SNP-pre-miRNA	>hsa-mir-124-3
3'SNP-pre-miRNA	>hsa-mir-1244-1
3'SNP-pre-miRNA	>hsa-mir-1244-2
3'SNP-pre-miRNA	>hsa-mir-1244-3
3'SNP-pre-miRNA	>hsa-mir-1245a
3'SNP-pre-miRNA	>hsa-mir-1245b
3'SNP-pre-miRNA	>hsa-mir-1247
3'SNP-pre-miRNA	>hsa-mir-1249
3'SNP-pre-miRNA	>hsa-mir-1250
3'SNP-pre-miRNA	>hsa-mir-1251
3'SNP-pre-miRNA	>hsa-mir-1252
3'SNP-pre-miRNA	>hsa-mir-1255b-2
3'SNP-pre-miRNA	>hsa-mir-1256
3'SNP-pre-miRNA	>hsa-mir-1258
3'SNP-pre-miRNA	>hsa-mir-125a
3'SNP-pre-miRNA	>hsa-mir-125b-2
3'SNP-pre-miRNA	>hsa-mir-126
3'SNP-pre-miRNA	>hsa-mir-1264
3'SNP-pre-miRNA	>hsa-mir-1269a
3'SNP-pre-miRNA	>hsa-mir-1273a
3'SNP-pre-miRNA	>hsa-mir-1273h
3'SNP-pre-miRNA	>hsa-mir-1277
3'SNP-pre-miRNA	>hsa-mir-1278
3'SNP-pre-miRNA	>hsa-mir-1281
3'SNP-pre-miRNA	>hsa-mir-128-1

3'SNP-pre-miRNA	>hsa-mir-1285-2
3'SNP-pre-miRNA	>hsa-mir-1286
3'SNP-pre-miRNA	>hsa-mir-1287
3'SNP-pre-miRNA	>hsa-mir-1289-1
3'SNP-pre-miRNA	>hsa-mir-1289-2
3'SNP-pre-miRNA	>hsa-mir-1290
3'SNP-pre-miRNA	>hsa-mir-129-1
3'SNP-pre-miRNA	>hsa-mir-1292
3'SNP-pre-miRNA	>hsa-mir-129-2
3'SNP-pre-miRNA	>hsa-mir-1294
3'SNP-pre-miRNA	>hsa-mir-1295a
3'SNP-pre-miRNA	>hsa-mir-1295b
3'SNP-pre-miRNA	>hsa-mir-1297
3'SNP-pre-miRNA	>hsa-mir-1299
3'SNP-pre-miRNA	>hsa-mir-1301
3'SNP-pre-miRNA	>hsa-mir-1302-1
3'SNP-pre-miRNA	>hsa-mir-1302-3
3'SNP-pre-miRNA	>hsa-mir-1302-4
3'SNP-pre-miRNA	>hsa-mir-1302-5
3'SNP-pre-miRNA	>hsa-mir-1302-8
3'SNP-pre-miRNA	>hsa-mir-1303
3'SNP-pre-miRNA	>hsa-mir-1304
3'SNP-pre-miRNA	>hsa-mir-1307
3'SNP-pre-miRNA	>hsa-mir-130b
3'SNP-pre-miRNA	>hsa-mir-1321
3'SNP-pre-miRNA	>hsa-mir-1322
3'SNP-pre-miRNA	>hsa-mir-1324
3'SNP-pre-miRNA	>hsa-mir-133a-1
3'SNP-pre-miRNA	>hsa-mir-133a-2
3'SNP-pre-miRNA	>hsa-mir-133b
3'SNP-pre-miRNA	>hsa-mir-134
3'SNP-pre-miRNA	>hsa-mir-1343
3'SNP-pre-miRNA	>hsa-mir-135a-1
3'SNP-pre-miRNA	>hsa-mir-135b
3'SNP-pre-miRNA	>hsa-mir-136
3'SNP-pre-miRNA	>hsa-mir-137
3'SNP-pre-miRNA	>hsa-mir-138-1
3'SNP-pre-miRNA	>hsa-mir-138-2
3'SNP-pre-miRNA	>hsa-mir-140
3'SNP-pre-miRNA	>hsa-mir-141
3'SNP-pre-miRNA	>hsa-mir-142
3'SNP-pre-miRNA	>hsa-mir-143
3'SNP-pre-miRNA	>hsa-mir-145
3'SNP-pre-miRNA	>hsa-mir-1468
3'SNP-pre-miRNA	>hsa-mir-146a
3'SNP-pre-miRNA	>hsa-mir-146b
3'SNP-pre-miRNA	>hsa-mir-147b

3’SNP-pre-miRNA >hsa-mir-148b
3’SNP-pre-miRNA >hsa-mir-149
3’SNP-pre-miRNA >hsa-mir-151a
3’SNP-pre-miRNA >hsa-mir-151b
3’SNP-pre-miRNA >hsa-mir-152
3’SNP-pre-miRNA >hsa-mir-153-2
3’SNP-pre-miRNA >hsa-mir-1537
3’SNP-pre-miRNA >hsa-mir-1538
3’SNP-pre-miRNA >hsa-mir-154
3’SNP-pre-miRNA >hsa-mir-155
3’SNP-pre-miRNA >hsa-mir-15b
3’SNP-pre-miRNA >hsa-mir-16-1
3’SNP-pre-miRNA >hsa-mir-17
3’SNP-pre-miRNA >hsa-mir-181a-2
3’SNP-pre-miRNA >hsa-mir-181b-1
3’SNP-pre-miRNA >hsa-mir-181c
3’SNP-pre-miRNA >hsa-mir-181d
3’SNP-pre-miRNA >hsa-mir-182
3’SNP-pre-miRNA >hsa-mir-1825
3’SNP-pre-miRNA >hsa-mir-183
3’SNP-pre-miRNA >hsa-mir-184
3’SNP-pre-miRNA >hsa-mir-185
3’SNP-pre-miRNA >hsa-mir-187
3’SNP-pre-miRNA >hsa-mir-188
3’SNP-pre-miRNA >hsa-mir-18a
3’SNP-pre-miRNA >hsa-mir-1908
3’SNP-pre-miRNA >hsa-mir-1909
3’SNP-pre-miRNA >hsa-mir-190a
3’SNP-pre-miRNA >hsa-mir-1910
3’SNP-pre-miRNA >hsa-mir-1911
3’SNP-pre-miRNA >hsa-mir-1912
3’SNP-pre-miRNA >hsa-mir-1913
3’SNP-pre-miRNA >hsa-mir-1914
3’SNP-pre-miRNA >hsa-mir-1915
3’SNP-pre-miRNA >hsa-mir-192
3’SNP-pre-miRNA >hsa-mir-193a
3’SNP-pre-miRNA >hsa-mir-194-2
3’SNP-pre-miRNA >hsa-mir-195
3’SNP-pre-miRNA >hsa-mir-196a-2
3’SNP-pre-miRNA >hsa-mir-196b
3’SNP-pre-miRNA >hsa-mir-197
3’SNP-pre-miRNA >hsa-mir-1972-2
3’SNP-pre-miRNA >hsa-mir-1973
3’SNP-pre-miRNA >hsa-mir-199a-1
3’SNP-pre-miRNA >hsa-mir-199a-2
3’SNP-pre-miRNA >hsa-mir-199b
3’SNP-pre-miRNA >hsa-mir-19b-2

3’SNP-pre-miRNA >hsa-mir-200a
3’SNP-pre-miRNA >hsa-mir-200b
3’SNP-pre-miRNA >hsa-mir-202
3’SNP-pre-miRNA >hsa-mir-203a
3’SNP-pre-miRNA >hsa-mir-203b
3’SNP-pre-miRNA >hsa-mir-204
3’SNP-pre-miRNA >hsa-mir-205
3’SNP-pre-miRNA >hsa-mir-2053
3’SNP-pre-miRNA >hsa-mir-206
3’SNP-pre-miRNA >hsa-mir-208b
3’SNP-pre-miRNA >hsa-mir-20a
3’SNP-pre-miRNA >hsa-mir-20b
3’SNP-pre-miRNA >hsa-mir-21
3’SNP-pre-miRNA >hsa-mir-210
3’SNP-pre-miRNA >hsa-mir-211
3’SNP-pre-miRNA >hsa-mir-2113
3’SNP-pre-miRNA >hsa-mir-2114
3’SNP-pre-miRNA >hsa-mir-2115
3’SNP-pre-miRNA >hsa-mir-2116
3’SNP-pre-miRNA >hsa-mir-2117
3’SNP-pre-miRNA >hsa-mir-212
3’SNP-pre-miRNA >hsa-mir-215
3’SNP-pre-miRNA >hsa-mir-216a
3’SNP-pre-miRNA >hsa-mir-216b
3’SNP-pre-miRNA >hsa-mir-217
3’SNP-pre-miRNA >hsa-mir-218-1
3’SNP-pre-miRNA >hsa-mir-218-2
3’SNP-pre-miRNA >hsa-mir-219a-1
3’SNP-pre-miRNA >hsa-mir-219a-2
3’SNP-pre-miRNA >hsa-mir-219b
3’SNP-pre-miRNA >hsa-mir-221
3’SNP-pre-miRNA >hsa-mir-222
3’SNP-pre-miRNA >hsa-mir-223
3’SNP-pre-miRNA >hsa-mir-224
3’SNP-pre-miRNA >hsa-mir-2276
3’SNP-pre-miRNA >hsa-mir-2277
3’SNP-pre-miRNA >hsa-mir-2355
3’SNP-pre-miRNA >hsa-mir-2392
3’SNP-pre-miRNA >hsa-mir-23a
3’SNP-pre-miRNA >hsa-mir-23b
3’SNP-pre-miRNA >hsa-mir-23c
3’SNP-pre-miRNA >hsa-mir-24-1
3’SNP-pre-miRNA >hsa-mir-24-2
3’SNP-pre-miRNA >hsa-mir-2467
3’SNP-pre-miRNA >hsa-mir-25
3’SNP-pre-miRNA >hsa-mir-2681
3’SNP-pre-miRNA >hsa-mir-2682

3’SNP-pre-miRNA	>hsa-mir-26a-1
3’SNP-pre-miRNA	>hsa-mir-26b
3’SNP-pre-miRNA	>hsa-mir-27a
3’SNP-pre-miRNA	>hsa-mir-27b
3’SNP-pre-miRNA	>hsa-mir-28
3’SNP-pre-miRNA	>hsa-mir-2861
3’SNP-pre-miRNA	>hsa-mir-296
3’SNP-pre-miRNA	>hsa-mir-299
3’SNP-pre-miRNA	>hsa-mir-300
3’SNP-pre-miRNA	>hsa-mir-301a
3’SNP-pre-miRNA	>hsa-mir-302a
3’SNP-pre-miRNA	>hsa-mir-302b
3’SNP-pre-miRNA	>hsa-mir-302c
3’SNP-pre-miRNA	>hsa-mir-302d
3’SNP-pre-miRNA	>hsa-mir-302f
3’SNP-pre-miRNA	>hsa-mir-3064
3’SNP-pre-miRNA	>hsa-mir-3065
3’SNP-pre-miRNA	>hsa-mir-3074
3’SNP-pre-miRNA	>hsa-mir-30a
3’SNP-pre-miRNA	>hsa-mir-30b
3’SNP-pre-miRNA	>hsa-mir-30c-2
3’SNP-pre-miRNA	>hsa-mir-30d
3’SNP-pre-miRNA	>hsa-mir-30e
3’SNP-pre-miRNA	>hsa-mir-3116-1
3’SNP-pre-miRNA	>hsa-mir-3116-2
3’SNP-pre-miRNA	>hsa-mir-3117
3’SNP-pre-miRNA	>hsa-mir-3118-1
3’SNP-pre-miRNA	>hsa-mir-3118-2
3’SNP-pre-miRNA	>hsa-mir-3118-3
3’SNP-pre-miRNA	>hsa-mir-3118-4
3’SNP-pre-miRNA	>hsa-mir-3121
3’SNP-pre-miRNA	>hsa-mir-3123
3’SNP-pre-miRNA	>hsa-mir-3124
3’SNP-pre-miRNA	>hsa-mir-3126
3’SNP-pre-miRNA	>hsa-mir-3127
3’SNP-pre-miRNA	>hsa-mir-3129
3’SNP-pre-miRNA	>hsa-mir-3130-1
3’SNP-pre-miRNA	>hsa-mir-3130-2
3’SNP-pre-miRNA	>hsa-mir-3134
3’SNP-pre-miRNA	>hsa-mir-3138
3’SNP-pre-miRNA	>hsa-mir-3140
3’SNP-pre-miRNA	>hsa-mir-3142
3’SNP-pre-miRNA	>hsa-mir-3144
3’SNP-pre-miRNA	>hsa-mir-3145
3’SNP-pre-miRNA	>hsa-mir-3146
3’SNP-pre-miRNA	>hsa-mir-3149
3’SNP-pre-miRNA	>hsa-mir-3150a

3’SNP-pre-miRNA >hsa-mir-3150b
3’SNP-pre-miRNA >hsa-mir-3151
3’SNP-pre-miRNA >hsa-mir-3152
3’SNP-pre-miRNA >hsa-mir-3153
3’SNP-pre-miRNA >hsa-mir-3154
3’SNP-pre-miRNA >hsa-mir-3155a
3’SNP-pre-miRNA >hsa-mir-3156-1
3’SNP-pre-miRNA >hsa-mir-3156-2
3’SNP-pre-miRNA >hsa-mir-3157
3’SNP-pre-miRNA >hsa-mir-3158-1
3’SNP-pre-miRNA >hsa-mir-3158-2
3’SNP-pre-miRNA >hsa-mir-3162
3’SNP-pre-miRNA >hsa-mir-3166
3’SNP-pre-miRNA >hsa-mir-3167
3’SNP-pre-miRNA >hsa-mir-3173
3’SNP-pre-miRNA >hsa-mir-3176
3’SNP-pre-miRNA >hsa-mir-3177
3’SNP-pre-miRNA >hsa-mir-3179-1
3’SNP-pre-miRNA >hsa-mir-3180-4
3’SNP-pre-miRNA >hsa-mir-3180-5
3’SNP-pre-miRNA >hsa-mir-3184
3’SNP-pre-miRNA >hsa-mir-3186
3’SNP-pre-miRNA >hsa-mir-3188
3’SNP-pre-miRNA >hsa-mir-3192
3’SNP-pre-miRNA >hsa-mir-320a
3’SNP-pre-miRNA >hsa-mir-320b-1
3’SNP-pre-miRNA >hsa-mir-320c-1
3’SNP-pre-miRNA >hsa-mir-320e
3’SNP-pre-miRNA >hsa-mir-323b
3’SNP-pre-miRNA >hsa-mir-324
3’SNP-pre-miRNA >hsa-mir-326
3’SNP-pre-miRNA >hsa-mir-328
3’SNP-pre-miRNA >hsa-mir-329-1
3’SNP-pre-miRNA >hsa-mir-329-2
3’SNP-pre-miRNA >hsa-mir-330
3’SNP-pre-miRNA >hsa-mir-335
3’SNP-pre-miRNA >hsa-mir-337
3’SNP-pre-miRNA >hsa-mir-338
3’SNP-pre-miRNA >hsa-mir-339
3’SNP-pre-miRNA >hsa-mir-33a
3’SNP-pre-miRNA >hsa-mir-33b
3’SNP-pre-miRNA >hsa-mir-340
3’SNP-pre-miRNA >hsa-mir-342
3’SNP-pre-miRNA >hsa-mir-345
3’SNP-pre-miRNA >hsa-mir-34a
3’SNP-pre-miRNA >hsa-mir-3605
3’SNP-pre-miRNA >hsa-mir-3607

3'SNP-pre-miRNA	>hsa-mir-3609
3'SNP-pre-miRNA	>hsa-mir-361
3'SNP-pre-miRNA	>hsa-mir-3610
3'SNP-pre-miRNA	>hsa-mir-3614
3'SNP-pre-miRNA	>hsa-mir-3615
3'SNP-pre-miRNA	>hsa-mir-3617
3'SNP-pre-miRNA	>hsa-mir-3618
3'SNP-pre-miRNA	>hsa-mir-3619
3'SNP-pre-miRNA	>hsa-mir-362
3'SNP-pre-miRNA	>hsa-mir-3620
3'SNP-pre-miRNA	>hsa-mir-3622a
3'SNP-pre-miRNA	>hsa-mir-3622b
3'SNP-pre-miRNA	>hsa-mir-363
3'SNP-pre-miRNA	>hsa-mir-3649
3'SNP-pre-miRNA	>hsa-mir-3653
3'SNP-pre-miRNA	>hsa-mir-3654
3'SNP-pre-miRNA	>hsa-mir-3657
3'SNP-pre-miRNA	>hsa-mir-3659
3'SNP-pre-miRNA	>hsa-mir-365a
3'SNP-pre-miRNA	>hsa-mir-365b
3'SNP-pre-miRNA	>hsa-mir-3660
3'SNP-pre-miRNA	>hsa-mir-3662
3'SNP-pre-miRNA	>hsa-mir-3663
3'SNP-pre-miRNA	>hsa-mir-3664
3'SNP-pre-miRNA	>hsa-mir-3667
3'SNP-pre-miRNA	>hsa-mir-367
3'SNP-pre-miRNA	>hsa-mir-3671
3'SNP-pre-miRNA	>hsa-mir-3678
3'SNP-pre-miRNA	>hsa-mir-3679
3'SNP-pre-miRNA	>hsa-mir-3682
3'SNP-pre-miRNA	>hsa-mir-3686
3'SNP-pre-miRNA	>hsa-mir-3689b
3'SNP-pre-miRNA	>hsa-mir-370
3'SNP-pre-miRNA	>hsa-mir-371a
3'SNP-pre-miRNA	>hsa-mir-371b
3'SNP-pre-miRNA	>hsa-mir-372
3'SNP-pre-miRNA	>hsa-mir-373
3'SNP-pre-miRNA	>hsa-mir-376a-2
3'SNP-pre-miRNA	>hsa-mir-377
3'SNP-pre-miRNA	>hsa-mir-378a
3'SNP-pre-miRNA	>hsa-mir-378e
3'SNP-pre-miRNA	>hsa-mir-379
3'SNP-pre-miRNA	>hsa-mir-380
3'SNP-pre-miRNA	>hsa-mir-381
3'SNP-pre-miRNA	>hsa-mir-382
3'SNP-pre-miRNA	>hsa-mir-383
3'SNP-pre-miRNA	>hsa-mir-384

3'SNP-pre-miRNA	>hsa-mir-3909
3'SNP-pre-miRNA	>hsa-mir-3910-1
3'SNP-pre-miRNA	>hsa-mir-3910-2
3'SNP-pre-miRNA	>hsa-mir-3912
3'SNP-pre-miRNA	>hsa-mir-3919
3'SNP-pre-miRNA	>hsa-mir-3922
3'SNP-pre-miRNA	>hsa-mir-3924
3'SNP-pre-miRNA	>hsa-mir-3927
3'SNP-pre-miRNA	>hsa-mir-3928
3'SNP-pre-miRNA	>hsa-mir-3936
3'SNP-pre-miRNA	>hsa-mir-3938
3'SNP-pre-miRNA	>hsa-mir-3939
3'SNP-pre-miRNA	>hsa-mir-409
3'SNP-pre-miRNA	>hsa-mir-410
3'SNP-pre-miRNA	>hsa-mir-411
3'SNP-pre-miRNA	>hsa-mir-412
3'SNP-pre-miRNA	>hsa-mir-421
3'SNP-pre-miRNA	>hsa-mir-423
3'SNP-pre-miRNA	>hsa-mir-424
3'SNP-pre-miRNA	>hsa-mir-425
3'SNP-pre-miRNA	>hsa-mir-4252
3'SNP-pre-miRNA	>hsa-mir-4253
3'SNP-pre-miRNA	>hsa-mir-4254
3'SNP-pre-miRNA	>hsa-mir-4257
3'SNP-pre-miRNA	>hsa-mir-4259
3'SNP-pre-miRNA	>hsa-mir-4265
3'SNP-pre-miRNA	>hsa-mir-4268
3'SNP-pre-miRNA	>hsa-mir-4274
3'SNP-pre-miRNA	>hsa-mir-4278
3'SNP-pre-miRNA	>hsa-mir-4279
3'SNP-pre-miRNA	>hsa-mir-4281
3'SNP-pre-miRNA	>hsa-mir-429
3'SNP-pre-miRNA	>hsa-mir-4293
3'SNP-pre-miRNA	>hsa-mir-4308
3'SNP-pre-miRNA	>hsa-mir-431
3'SNP-pre-miRNA	>hsa-mir-4312
3'SNP-pre-miRNA	>hsa-mir-4318
3'SNP-pre-miRNA	>hsa-mir-432
3'SNP-pre-miRNA	>hsa-mir-4320
3'SNP-pre-miRNA	>hsa-mir-4321
3'SNP-pre-miRNA	>hsa-mir-4322
3'SNP-pre-miRNA	>hsa-mir-4324
3'SNP-pre-miRNA	>hsa-mir-433
3'SNP-pre-miRNA	>hsa-mir-448
3'SNP-pre-miRNA	>hsa-mir-449b
3'SNP-pre-miRNA	>hsa-mir-449c
3'SNP-pre-miRNA	>hsa-mir-450a-1

3’SNP-pre-miRNA	>hsa-mir-450a-2
3’SNP-pre-miRNA	>hsa-mir-450b
3’SNP-pre-miRNA	>hsa-mir-452
3’SNP-pre-miRNA	>hsa-mir-454
3’SNP-pre-miRNA	>hsa-mir-455
3’SNP-pre-miRNA	>hsa-mir-466
3’SNP-pre-miRNA	>hsa-mir-483
3’SNP-pre-miRNA	>hsa-mir-485
3’SNP-pre-miRNA	>hsa-mir-486-2
3’SNP-pre-miRNA	>hsa-mir-487a
3’SNP-pre-miRNA	>hsa-mir-487b
3’SNP-pre-miRNA	>hsa-mir-488
3’SNP-pre-miRNA	>hsa-mir-489
3’SNP-pre-miRNA	>hsa-mir-490
3’SNP-pre-miRNA	>hsa-mir-491
3’SNP-pre-miRNA	>hsa-mir-493
3’SNP-pre-miRNA	>hsa-mir-495
3’SNP-pre-miRNA	>hsa-mir-496
3’SNP-pre-miRNA	>hsa-mir-497
3’SNP-pre-miRNA	>hsa-mir-498
3’SNP-pre-miRNA	>hsa-mir-499a
3’SNP-pre-miRNA	>hsa-mir-499b
3’SNP-pre-miRNA	>hsa-mir-500a
3’SNP-pre-miRNA	>hsa-mir-500b
3’SNP-pre-miRNA	>hsa-mir-501
3’SNP-pre-miRNA	>hsa-mir-502
3’SNP-pre-miRNA	>hsa-mir-503
3’SNP-pre-miRNA	>hsa-mir-504
3’SNP-pre-miRNA	>hsa-mir-505
3’SNP-pre-miRNA	>hsa-mir-506
3’SNP-pre-miRNA	>hsa-mir-507
3’SNP-pre-miRNA	>hsa-mir-508
3’SNP-pre-miRNA	>hsa-mir-509-1
3’SNP-pre-miRNA	>hsa-mir-509-2
3’SNP-pre-miRNA	>hsa-mir-509-3
3’SNP-pre-miRNA	>hsa-mir-510
3’SNP-pre-miRNA	>hsa-mir-511
3’SNP-pre-miRNA	>hsa-mir-512-1
3’SNP-pre-miRNA	>hsa-mir-512-2
3’SNP-pre-miRNA	>hsa-mir-513a-1
3’SNP-pre-miRNA	>hsa-mir-513a-2
3’SNP-pre-miRNA	>hsa-mir-513b
3’SNP-pre-miRNA	>hsa-mir-513c
3’SNP-pre-miRNA	>hsa-mir-514a-1
3’SNP-pre-miRNA	>hsa-mir-514a-3
3’SNP-pre-miRNA	>hsa-mir-515-1
3’SNP-pre-miRNA	>hsa-mir-516a-1

3’SNP-pre-miRNA	>hsa-mir-516a-2
3’SNP-pre-miRNA	>hsa-mir-516b-1
3’SNP-pre-miRNA	>hsa-mir-516b-2
3’SNP-pre-miRNA	>hsa-mir-517a
3’SNP-pre-miRNA	>hsa-mir-517b
3’SNP-pre-miRNA	>hsa-mir-517c
3’SNP-pre-miRNA	>hsa-mir-518a-1
3’SNP-pre-miRNA	>hsa-mir-518a-2
3’SNP-pre-miRNA	>hsa-mir-518b
3’SNP-pre-miRNA	>hsa-mir-518c
3’SNP-pre-miRNA	>hsa-mir-518d
3’SNP-pre-miRNA	>hsa-mir-518e
3’SNP-pre-miRNA	>hsa-mir-518f
3’SNP-pre-miRNA	>hsa-mir-519a-1
3’SNP-pre-miRNA	>hsa-mir-519a-2
3’SNP-pre-miRNA	>hsa-mir-519b
3’SNP-pre-miRNA	>hsa-mir-519c
3’SNP-pre-miRNA	>hsa-mir-519d
3’SNP-pre-miRNA	>hsa-mir-520a
3’SNP-pre-miRNA	>hsa-mir-520b
3’SNP-pre-miRNA	>hsa-mir-520c
3’SNP-pre-miRNA	>hsa-mir-520d
3’SNP-pre-miRNA	>hsa-mir-520e
3’SNP-pre-miRNA	>hsa-mir-520f
3’SNP-pre-miRNA	>hsa-mir-520g
3’SNP-pre-miRNA	>hsa-mir-520h
3’SNP-pre-miRNA	>hsa-mir-521-1
3’SNP-pre-miRNA	>hsa-mir-521-2
3’SNP-pre-miRNA	>hsa-mir-522
3’SNP-pre-miRNA	>hsa-mir-523
3’SNP-pre-miRNA	>hsa-mir-524
3’SNP-pre-miRNA	>hsa-mir-525
3’SNP-pre-miRNA	>hsa-mir-526b
3’SNP-pre-miRNA	>hsa-mir-532
3’SNP-pre-miRNA	>hsa-mir-541
3’SNP-pre-miRNA	>hsa-mir-542
3’SNP-pre-miRNA	>hsa-mir-543
3’SNP-pre-miRNA	>hsa-mir-544a
3’SNP-pre-miRNA	>hsa-mir-544b
3’SNP-pre-miRNA	>hsa-mir-545
3’SNP-pre-miRNA	>hsa-mir-548a-1
3’SNP-pre-miRNA	>hsa-mir-548a-2
3’SNP-pre-miRNA	>hsa-mir-548a-3
3’SNP-pre-miRNA	>hsa-mir-548aa-1
3’SNP-pre-miRNA	>hsa-mir-548aa-2
3’SNP-pre-miRNA	>hsa-mir-548ac
3’SNP-pre-miRNA	>hsa-mir-548ad

3'SNP-pre-miRNA	>hsa-mir-548ae-1
3'SNP-pre-miRNA	>hsa-mir-548aj-1
3'SNP-pre-miRNA	>hsa-mir-548aj-2
3'SNP-pre-miRNA	>hsa-mir-548al
3'SNP-pre-miRNA	>hsa-mir-548am
3'SNP-pre-miRNA	>hsa-mir-548ao
3'SNP-pre-miRNA	>hsa-mir-548ap
3'SNP-pre-miRNA	>hsa-mir-548aq
3'SNP-pre-miRNA	>hsa-mir-548ar
3'SNP-pre-miRNA	>hsa-mir-548as
3'SNP-pre-miRNA	>hsa-mir-548at
3'SNP-pre-miRNA	>hsa-mir-548au
3'SNP-pre-miRNA	>hsa-mir-548av
3'SNP-pre-miRNA	>hsa-mir-548ay
3'SNP-pre-miRNA	>hsa-mir-548b
3'SNP-pre-miRNA	>hsa-mir-548c
3'SNP-pre-miRNA	>hsa-mir-548d-1
3'SNP-pre-miRNA	>hsa-mir-548d-2
3'SNP-pre-miRNA	>hsa-mir-548f-1
3'SNP-pre-miRNA	>hsa-mir-548f-2
3'SNP-pre-miRNA	>hsa-mir-548f-3
3'SNP-pre-miRNA	>hsa-mir-548f-4
3'SNP-pre-miRNA	>hsa-mir-548f-5
3'SNP-pre-miRNA	>hsa-mir-548g
3'SNP-pre-miRNA	>hsa-mir-548h-4
3'SNP-pre-miRNA	>hsa-mir-548i-1
3'SNP-pre-miRNA	>hsa-mir-658
3'SNP-pre-miRNA	>hsa-mir-659
3'SNP-pre-miRNA	>hsa-mir-660
3'SNP-pre-miRNA	>hsa-mir-661
3'SNP-pre-miRNA	>hsa-mir-662
3'SNP-pre-miRNA	>hsa-mir-663b
3'SNP-pre-miRNA	>hsa-mir-664a
3'SNP-pre-miRNA	>hsa-mir-664b
3'SNP-pre-miRNA	>hsa-mir-665
3'SNP-pre-miRNA	>hsa-mir-668
3'SNP-pre-miRNA	>hsa-mir-670
3'SNP-pre-miRNA	>hsa-mir-671
3'SNP-pre-miRNA	>hsa-mir-675
3'SNP-pre-miRNA	>hsa-mir-676
3'SNP-pre-miRNA	>hsa-mir-708
3'SNP-pre-miRNA	>hsa-mir-7-1
3'SNP-pre-miRNA	>hsa-mir-711
3'SNP-pre-miRNA	>hsa-mir-7-2
3'SNP-pre-miRNA	>hsa-mir-744
3'SNP-pre-miRNA	>hsa-mir-758
3'SNP-pre-miRNA	>hsa-mir-760

3’SNP-pre-miRNA	>hsa-mir-762
3’SNP-pre-miRNA	>hsa-mir-766
3’SNP-pre-miRNA	>hsa-mir-767
3’SNP-pre-miRNA	>hsa-mir-769
3’SNP-pre-miRNA	>hsa-mir-873
3’SNP-pre-miRNA	>hsa-mir-874
3’SNP-pre-miRNA	>hsa-mir-876
3’SNP-pre-miRNA	>hsa-mir-877
3’SNP-pre-miRNA	>hsa-mir-885
3’SNP-pre-miRNA	>hsa-mir-887
3’SNP-pre-miRNA	>hsa-mir-888
3’SNP-pre-miRNA	>hsa-mir-891a
3’SNP-pre-miRNA	>hsa-mir-892a
3’SNP-pre-miRNA	>hsa-mir-892b
3’SNP-pre-miRNA	>hsa-mir-892c
3’SNP-pre-miRNA	>hsa-mir-9-1
3’SNP-pre-miRNA	>hsa-mir-9-2
3’SNP-pre-miRNA	>hsa-mir-920
3’SNP-pre-miRNA	>hsa-mir-922
3’SNP-pre-miRNA	>hsa-mir-92a-1
3’SNP-pre-miRNA	>hsa-mir-92a-2
3’SNP-pre-miRNA	>hsa-mir-92b
3’SNP-pre-miRNA	>hsa-mir-93
3’SNP-pre-miRNA	>hsa-mir-9-3
3’SNP-pre-miRNA	>hsa-mir-933
3’SNP-pre-miRNA	>hsa-mir-937
3’SNP-pre-miRNA	>hsa-mir-939
3’SNP-pre-miRNA	>hsa-mir-940
3’SNP-pre-miRNA	>hsa-mir-942
3’SNP-pre-miRNA	>hsa-mir-943
3’SNP-pre-miRNA	>hsa-mir-944
3’SNP-pre-miRNA	>hsa-mir-95
3’SNP-pre-miRNA	>hsa-mir-96
3’SNP-pre-miRNA	>hsa-mir-98
3’SNP-pre-miRNA	>hsa-mir-99a
3’SNP-pre-miRNA	>hsa-mir-99b
