# Supplementary material for: Role of SNPs in the Biogenesis of Mature miRNAs
Source: Biomed Res Int. 2021 Jun 17;2021:2403418. doi: 10.1155/2021/2403418 (PMC8233088; doi:10.1155/2021/2403418)
Supplement: Supplementary Materials — Additional file 1 Table S1: the database miRSNPBase (xls). Additional file 2 Table S2: the list of pre-miRNAs in miRSNPBase which is classified based on mature miRNA in the 5′ arm or 3′ arm (xls). Additional file 3 Table S3: all the iso-pre-miRNAs, nor-pre-miRNAs, nor-SNPs, and iso-SNPs associated with four splicing sites (xls). Additional file 4 Table S4: the pre-miRNAs and SNPs associated with the normal and isomiRs (xls). Additional file 5 Table S5: the pre-miRNAs, iso-SNPs, and isomiRs of HG00097 (xls). Additional file 6 Table S6: the isomiRs and iso-SNP of 18 GBR populations (xls). Additional file 7 Table S7: the verified isomiRs of 18 GBR (xls). Additional file 8 Table S8: the iso-pre-miRNA candidates and the verified iso-pre-miRNAs of 18 GBR samples (xls). [file 2403418.f1.zip › 2403418.f1/Supp Tab S3.pdf]

Sits	Category
P5_5	nor-pre-miRNA>hsa-let-7a-2
P5_5	nor-pre-miRNA>hsa-let-7b
P5_5	nor-pre-miRNA>hsa-let-7e
P5_5	nor-pre-miRNA>hsa-let-7i
P5_5	nor-pre-miRNA>hsa-mir-105-1
P5_5	nor-pre-miRNA>hsa-mir-105-2
P5_5	nor-pre-miRNA>hsa-mir-106b
P5_5	nor-pre-miRNA>hsa-mir-10a
P5_5	nor-pre-miRNA>hsa-mir-10b
P5_5	nor-pre-miRNA>hsa-mir-1179
P5_5	nor-pre-miRNA>hsa-mir-1180
P5_5	nor-pre-miRNA>hsa-mir-1181
P5_5	nor-pre-miRNA>hsa-mir-1185-1
P5_5	nor-pre-miRNA>hsa-mir-1185-2
P5_5	nor-pre-miRNA>hsa-mir-1193
P5_5	nor-pre-miRNA>hsa-mir-1200
P5_5	nor-pre-miRNA>hsa-mir-1203
P5_5	nor-pre-miRNA>hsa-mir-1204
P5_5	nor-pre-miRNA>hsa-mir-1205
P5_5	nor-pre-miRNA>hsa-mir-1207
P5_5	nor-pre-miRNA>hsa-mir-1208
P5_5	nor-pre-miRNA>hsa-mir-122
P5_5	nor-pre-miRNA>hsa-mir-1225
P5_5	nor-pre-miRNA>hsa-mir-1226
P5_5	nor-pre-miRNA>hsa-mir-1227
P5_5	nor-pre-miRNA>hsa-mir-1228
P5_5	nor-pre-miRNA>hsa-mir-1229
P5_5	nor-pre-miRNA>hsa-mir-1231
P5_5	nor-pre-miRNA>hsa-mir-1233-1
P5_5	nor-pre-miRNA>hsa-mir-1233-2
P5_5	nor-pre-miRNA>hsa-mir-1236
P5_5	nor-pre-miRNA>hsa-mir-1238
P5_5	nor-pre-miRNA>hsa-mir-124-2
P5_5	nor-pre-miRNA>hsa-mir-1243
P5_5	nor-pre-miRNA>hsa-mir-124-3
P5_5	nor-pre-miRNA>hsa-mir-1245b
P5_5	nor-pre-miRNA>hsa-mir-1246
P5_5	nor-pre-miRNA>hsa-mir-1248
P5_5	nor-pre-miRNA>hsa-mir-1250
P5_5	nor-pre-miRNA>hsa-mir-1251
P5_5	nor-pre-miRNA>hsa-mir-1252
P5_5	nor-pre-miRNA>hsa-mir-1253
P5_5	nor-pre-miRNA>hsa-mir-1254-1
P5_5	nor-pre-miRNA>hsa-mir-1254-2
P5_5	nor-pre-miRNA>hsa-mir-1255a
P5_5	nor-pre-miRNA>hsa-mir-1255b-1

P5_5 nor-pre-miRNA>hsa-mir-1255b-2
P5_5 nor-pre-miRNA>hsa-mir-1257
P5_5 nor-pre-miRNA>hsa-mir-125a
P5_5 nor-pre-miRNA>hsa-mir-125b-2
P5_5 nor-pre-miRNA>hsa-mir-126
P5_5 nor-pre-miRNA>hsa-mir-1260b
P5_5 nor-pre-miRNA>hsa-mir-1261
P5_5 nor-pre-miRNA>hsa-mir-1262
P5_5 nor-pre-miRNA>hsa-mir-1263
P5_5 nor-pre-miRNA>hsa-mir-1265
P5_5 nor-pre-miRNA>hsa-mir-1268b
P5_5 nor-pre-miRNA>hsa-mir-1269b
P5_5 nor-pre-miRNA>hsa-mir-1272
P5_5 nor-pre-miRNA>hsa-mir-1273c
P5_5 nor-pre-miRNA>hsa-mir-1273d
P5_5 nor-pre-miRNA>hsa-mir-1273f
P5_5 nor-pre-miRNA>hsa-mir-1273h
P5_5 nor-pre-miRNA>hsa-mir-1275
P5_5 nor-pre-miRNA>hsa-mir-1276
P5_5 nor-pre-miRNA>hsa-mir-1277
P5_5 nor-pre-miRNA>hsa-mir-128-1
P5_5 nor-pre-miRNA>hsa-mir-1282
P5_5 nor-pre-miRNA>hsa-mir-1283-1
P5_5 nor-pre-miRNA>hsa-mir-1283-2
P5_5 nor-pre-miRNA>hsa-mir-1284
P5_5 nor-pre-miRNA>hsa-mir-1287
P5_5 nor-pre-miRNA>hsa-mir-1291
P5_5 nor-pre-miRNA>hsa-mir-129-1
P5_5 nor-pre-miRNA>hsa-mir-1292
P5_5 nor-pre-miRNA>hsa-mir-129-2
P5_5 nor-pre-miRNA>hsa-mir-1293
P5_5 nor-pre-miRNA>hsa-mir-1295b
P5_5 nor-pre-miRNA>hsa-mir-1301
P5_5 nor-pre-miRNA>hsa-mir-1304
P5_5 nor-pre-miRNA>hsa-mir-1307
P5_5 nor-pre-miRNA>hsa-mir-130b
P5_5 nor-pre-miRNA>hsa-mir-1323
P5_5 nor-pre-miRNA>hsa-mir-133a-1
P5_5 nor-pre-miRNA>hsa-mir-133a-2
P5_5 nor-pre-miRNA>hsa-mir-134
P5_5 nor-pre-miRNA>hsa-mir-1343
P5_5 nor-pre-miRNA>hsa-mir-135a-1
P5_5 nor-pre-miRNA>hsa-mir-135a-2
P5_5 nor-pre-miRNA>hsa-mir-135b
P5_5 nor-pre-miRNA>hsa-mir-136
P5_5 nor-pre-miRNA>hsa-mir-138-1
P5_5 nor-pre-miRNA>hsa-mir-138-2

P5_5 nor-pre-miRNA>hsa-mir-140
P5_5 nor-pre-miRNA>hsa-mir-141
P5_5 nor-pre-miRNA>hsa-mir-142
P5_5 nor-pre-miRNA>hsa-mir-143
P5_5 nor-pre-miRNA>hsa-mir-145
P5_5 nor-pre-miRNA>hsa-mir-1468
P5_5 nor-pre-miRNA>hsa-mir-1469
P5_5 nor-pre-miRNA>hsa-mir-146a
P5_5 nor-pre-miRNA>hsa-mir-146b
P5_5 nor-pre-miRNA>hsa-mir-1470
P5_5 nor-pre-miRNA>hsa-mir-148b
P5_5 nor-pre-miRNA>hsa-mir-149
P5_5 nor-pre-miRNA>hsa-mir-152
P5_5 nor-pre-miRNA>hsa-mir-153-2
P5_5 nor-pre-miRNA>hsa-mir-1537
P5_5 nor-pre-miRNA>hsa-mir-154
P5_5 nor-pre-miRNA>hsa-mir-155
P5_5 nor-pre-miRNA>hsa-mir-1587
P5_5 nor-pre-miRNA>hsa-mir-15b
P5_5 nor-pre-miRNA>hsa-mir-16-1
P5_5 nor-pre-miRNA>hsa-mir-17
P5_5 nor-pre-miRNA>hsa-mir-181b-1
P5_5 nor-pre-miRNA>hsa-mir-181b-2
P5_5 nor-pre-miRNA>hsa-mir-181c
P5_5 nor-pre-miRNA>hsa-mir-181d
P5_5 nor-pre-miRNA>hsa-mir-182
P5_5 nor-pre-miRNA>hsa-mir-183
P5_5 nor-pre-miRNA>hsa-mir-185
P5_5 nor-pre-miRNA>hsa-mir-187
P5_5 nor-pre-miRNA>hsa-mir-188
P5_5 nor-pre-miRNA>hsa-mir-18a
P5_5 nor-pre-miRNA>hsa-mir-1908
P5_5 nor-pre-miRNA>hsa-mir-1909
P5_5 nor-pre-miRNA>hsa-mir-190a
P5_5 nor-pre-miRNA>hsa-mir-190b
P5_5 nor-pre-miRNA>hsa-mir-1910
P5_5 nor-pre-miRNA>hsa-mir-1911
P5_5 nor-pre-miRNA>hsa-mir-1914
P5_5 nor-pre-miRNA>hsa-mir-1915
P5_5 nor-pre-miRNA>hsa-mir-192
P5_5 nor-pre-miRNA>hsa-mir-193a
P5_5 nor-pre-miRNA>hsa-mir-194-1
P5_5 nor-pre-miRNA>hsa-mir-195
P5_5 nor-pre-miRNA>hsa-mir-196a-1
P5_5 nor-pre-miRNA>hsa-mir-196a-2
P5_5 nor-pre-miRNA>hsa-mir-196b
P5_5 nor-pre-miRNA>hsa-mir-197

P5_5 nor-pre-miRNA>hsa-mir-198
P5_5 nor-pre-miRNA>hsa-mir-199a-1
P5_5 nor-pre-miRNA>hsa-mir-199a-2
P5_5 nor-pre-miRNA>hsa-mir-199b
P5_5 nor-pre-miRNA>hsa-mir-19b-2
P5_5 nor-pre-miRNA>hsa-mir-200a
P5_5 nor-pre-miRNA>hsa-mir-200b
P5_5 nor-pre-miRNA>hsa-mir-202
P5_5 nor-pre-miRNA>hsa-mir-203b
P5_5 nor-pre-miRNA>hsa-mir-205
P5_5 nor-pre-miRNA>hsa-mir-208b
P5_5 nor-pre-miRNA>hsa-mir-20a
P5_5 nor-pre-miRNA>hsa-mir-20b
P5_5 nor-pre-miRNA>hsa-mir-21
P5_5 nor-pre-miRNA>hsa-mir-210
P5_5 nor-pre-miRNA>hsa-mir-211
P5_5 nor-pre-miRNA>hsa-mir-2110
P5_5 nor-pre-miRNA>hsa-mir-2114
P5_5 nor-pre-miRNA>hsa-mir-215
P5_5 nor-pre-miRNA>hsa-mir-216a
P5_5 nor-pre-miRNA>hsa-mir-216b
P5_5 nor-pre-miRNA>hsa-mir-218-1
P5_5 nor-pre-miRNA>hsa-mir-218-2
P5_5 nor-pre-miRNA>hsa-mir-219a-1
P5_5 nor-pre-miRNA>hsa-mir-219a-2
P5_5 nor-pre-miRNA>hsa-mir-219b
P5_5 nor-pre-miRNA>hsa-mir-221
P5_5 nor-pre-miRNA>hsa-mir-222
P5_5 nor-pre-miRNA>hsa-mir-223
P5_5 nor-pre-miRNA>hsa-mir-224
P5_5 nor-pre-miRNA>hsa-mir-2276
P5_5 nor-pre-miRNA>hsa-mir-2277
P5_5 nor-pre-miRNA>hsa-mir-2278
P5_5 nor-pre-miRNA>hsa-mir-2355
P5_5 nor-pre-miRNA>hsa-mir-23a
P5_5 nor-pre-miRNA>hsa-mir-23b
P5_5 nor-pre-miRNA>hsa-mir-24-1
P5_5 nor-pre-miRNA>hsa-mir-2467
P5_5 nor-pre-miRNA>hsa-mir-25
P5_5 nor-pre-miRNA>hsa-mir-2681
P5_5 nor-pre-miRNA>hsa-mir-2682
P5_5 nor-pre-miRNA>hsa-mir-26a-1
P5_5 nor-pre-miRNA>hsa-mir-26b
P5_5 nor-pre-miRNA>hsa-mir-27a
P5_5 nor-pre-miRNA>hsa-mir-27b
P5_5 nor-pre-miRNA>hsa-mir-28
P5_5 nor-pre-miRNA>hsa-mir-2909

P5_5 nor-pre-miRNA>hsa-mir-296
P5_5 nor-pre-miRNA>hsa-mir-297
P5_5 nor-pre-miRNA>hsa-mir-299
P5_5 nor-pre-miRNA>hsa-mir-301a
P5_5 nor-pre-miRNA>hsa-mir-302a
P5_5 nor-pre-miRNA>hsa-mir-302b
P5_5 nor-pre-miRNA>hsa-mir-302c
P5_5 nor-pre-miRNA>hsa-mir-302d
P5_5 nor-pre-miRNA>hsa-mir-302e
P5_5 nor-pre-miRNA>hsa-mir-3064
P5_5 nor-pre-miRNA>hsa-mir-3065
P5_5 nor-pre-miRNA>hsa-mir-3074
P5_5 nor-pre-miRNA>hsa-mir-30a
P5_5 nor-pre-miRNA>hsa-mir-30b
P5_5 nor-pre-miRNA>hsa-mir-30c-2
P5_5 nor-pre-miRNA>hsa-mir-30d
P5_5 nor-pre-miRNA>hsa-mir-30e
P5_5 nor-pre-miRNA>hsa-mir-3117
P5_5 nor-pre-miRNA>hsa-mir-3119-1
P5_5 nor-pre-miRNA>hsa-mir-3119-2
P5_5 nor-pre-miRNA>hsa-mir-3121
P5_5 nor-pre-miRNA>hsa-mir-3124
P5_5 nor-pre-miRNA>hsa-mir-3125
P5_5 nor-pre-miRNA>hsa-mir-3126
P5_5 nor-pre-miRNA>hsa-mir-3127
P5_5 nor-pre-miRNA>hsa-mir-3128
P5_5 nor-pre-miRNA>hsa-mir-3130-1
P5_5 nor-pre-miRNA>hsa-mir-3130-2
P5_5 nor-pre-miRNA>hsa-mir-3131
P5_5 nor-pre-miRNA>hsa-mir-3132
P5_5 nor-pre-miRNA>hsa-mir-3133
P5_5 nor-pre-miRNA>hsa-mir-3135a
P5_5 nor-pre-miRNA>hsa-mir-3135b
P5_5 nor-pre-miRNA>hsa-mir-3137
P5_5 nor-pre-miRNA>hsa-mir-3139
P5_5 nor-pre-miRNA>hsa-mir-3140
P5_5 nor-pre-miRNA>hsa-mir-3143
P5_5 nor-pre-miRNA>hsa-mir-3145
P5_5 nor-pre-miRNA>hsa-mir-3147
P5_5 nor-pre-miRNA>hsa-mir-3148
P5_5 nor-pre-miRNA>hsa-mir-3150a
P5_5 nor-pre-miRNA>hsa-mir-3150b
P5_5 nor-pre-miRNA>hsa-mir-3151
P5_5 nor-pre-miRNA>hsa-mir-3152
P5_5 nor-pre-miRNA>hsa-mir-3156-1
P5_5 nor-pre-miRNA>hsa-mir-3156-2
P5_5 nor-pre-miRNA>hsa-mir-3156-3

P5_5 nor-pre-miRNA>hsa-mir-3157
P5_5 nor-pre-miRNA>hsa-mir-3158-1
P5_5 nor-pre-miRNA>hsa-mir-3158-2
P5_5 nor-pre-miRNA>hsa-mir-3159
P5_5 nor-pre-miRNA>hsa-mir-3161
P5_5 nor-pre-miRNA>hsa-mir-3162
P5_5 nor-pre-miRNA>hsa-mir-3163
P5_5 nor-pre-miRNA>hsa-mir-3164
P5_5 nor-pre-miRNA>hsa-mir-3168
P5_5 nor-pre-miRNA>hsa-mir-3169
P5_5 nor-pre-miRNA>hsa-mir-3170
P5_5 nor-pre-miRNA>hsa-mir-3171
P5_5 nor-pre-miRNA>hsa-mir-3173
P5_5 nor-pre-miRNA>hsa-mir-3175
P5_5 nor-pre-miRNA>hsa-mir-3177
P5_5 nor-pre-miRNA>hsa-mir-3178
P5_5 nor-pre-miRNA>hsa-mir-3182
P5_5 nor-pre-miRNA>hsa-mir-3183
P5_5 nor-pre-miRNA>hsa-mir-3184
P5_5 nor-pre-miRNA>hsa-mir-3185
P5_5 nor-pre-miRNA>hsa-mir-3186
P5_5 nor-pre-miRNA>hsa-mir-3192
P5_5 nor-pre-miRNA>hsa-mir-3199-1
P5_5 nor-pre-miRNA>hsa-mir-3199-2
P5_5 nor-pre-miRNA>hsa-mir-323b
P5_5 nor-pre-miRNA>hsa-mir-324
P5_5 nor-pre-miRNA>hsa-mir-325
P5_5 nor-pre-miRNA>hsa-mir-328
P5_5 nor-pre-miRNA>hsa-mir-329-1
P5_5 nor-pre-miRNA>hsa-mir-329-2
P5_5 nor-pre-miRNA>hsa-mir-330
P5_5 nor-pre-miRNA>hsa-mir-335
P5_5 nor-pre-miRNA>hsa-mir-337
P5_5 nor-pre-miRNA>hsa-mir-338
P5_5 nor-pre-miRNA>hsa-mir-339
P5_5 nor-pre-miRNA>hsa-mir-33a
P5_5 nor-pre-miRNA>hsa-mir-33b
P5_5 nor-pre-miRNA>hsa-mir-340
P5_5 nor-pre-miRNA>hsa-mir-342
P5_5 nor-pre-miRNA>hsa-mir-345
P5_5 nor-pre-miRNA>hsa-mir-346
P5_5 nor-pre-miRNA>hsa-mir-34a
P5_5 nor-pre-miRNA>hsa-mir-3605
P5_5 nor-pre-miRNA>hsa-mir-3607
P5_5 nor-pre-miRNA>hsa-mir-361
P5_5 nor-pre-miRNA>hsa-mir-3612
P5_5 nor-pre-miRNA>hsa-mir-3617

P5_5 nor-pre-miRNA>hsa-mir-3619
P5_5 nor-pre-miRNA>hsa-mir-362
P5_5 nor-pre-miRNA>hsa-mir-3622a
P5_5 nor-pre-miRNA>hsa-mir-3622b
P5_5 nor-pre-miRNA>hsa-mir-363
P5_5 nor-pre-miRNA>hsa-mir-3652
P5_5 nor-pre-miRNA>hsa-mir-365a
P5_5 nor-pre-miRNA>hsa-mir-365b
P5_5 nor-pre-miRNA>hsa-mir-3663
P5_5 nor-pre-miRNA>hsa-mir-3664
P5_5 nor-pre-miRNA>hsa-mir-3667
P5_5 nor-pre-miRNA>hsa-mir-367
P5_5 nor-pre-miRNA>hsa-mir-3674
P5_5 nor-pre-miRNA>hsa-mir-3678
P5_5 nor-pre-miRNA>hsa-mir-3679
P5_5 nor-pre-miRNA>hsa-mir-3682
P5_5 nor-pre-miRNA>hsa-mir-3689b
P5_5 nor-pre-miRNA>hsa-mir-370
P5_5 nor-pre-miRNA>hsa-mir-3714
P5_5 nor-pre-miRNA>hsa-mir-371a
P5_5 nor-pre-miRNA>hsa-mir-371b
P5_5 nor-pre-miRNA>hsa-mir-372
P5_5 nor-pre-miRNA>hsa-mir-373
P5_5 nor-pre-miRNA>hsa-mir-376a-2
P5_5 nor-pre-miRNA>hsa-mir-377
P5_5 nor-pre-miRNA>hsa-mir-378a
P5_5 nor-pre-miRNA>hsa-mir-378c
P5_5 nor-pre-miRNA>hsa-mir-378h
P5_5 nor-pre-miRNA>hsa-mir-378i
P5_5 nor-pre-miRNA>hsa-mir-379
P5_5 nor-pre-miRNA>hsa-mir-380
P5_5 nor-pre-miRNA>hsa-mir-381
P5_5 nor-pre-miRNA>hsa-mir-382
P5_5 nor-pre-miRNA>hsa-mir-383
P5_5 nor-pre-miRNA>hsa-mir-3908
P5_5 nor-pre-miRNA>hsa-mir-3911
P5_5 nor-pre-miRNA>hsa-mir-3912
P5_5 nor-pre-miRNA>hsa-mir-3922
P5_5 nor-pre-miRNA>hsa-mir-3928
P5_5 nor-pre-miRNA>hsa-mir-3945
P5_5 nor-pre-miRNA>hsa-mir-409
P5_5 nor-pre-miRNA>hsa-mir-410
P5_5 nor-pre-miRNA>hsa-mir-411
P5_5 nor-pre-miRNA>hsa-mir-412
P5_5 nor-pre-miRNA>hsa-mir-423
P5_5 nor-pre-miRNA>hsa-mir-424
P5_5 nor-pre-miRNA>hsa-mir-4260

P5_5 nor-pre-miRNA>hsa-mir-4267
P5_5 nor-pre-miRNA>hsa-mir-4269
P5_5 nor-pre-miRNA>hsa-mir-4277
P5_5 nor-pre-miRNA>hsa-mir-4280
P5_5 nor-pre-miRNA>hsa-mir-4283-1
P5_5 nor-pre-miRNA>hsa-mir-4283-2
P5_5 nor-pre-miRNA>hsa-mir-4284
P5_5 nor-pre-miRNA>hsa-mir-4286
P5_5 nor-pre-miRNA>hsa-mir-4294
P5_5 nor-pre-miRNA>hsa-mir-4296
P5_5 nor-pre-miRNA>hsa-mir-4297
P5_5 nor-pre-miRNA>hsa-mir-4298
P5_5 nor-pre-miRNA>hsa-mir-4305
P5_5 nor-pre-miRNA>hsa-mir-4309
P5_5 nor-pre-miRNA>hsa-mir-431
P5_5 nor-pre-miRNA>hsa-mir-432
P5_5 nor-pre-miRNA>hsa-mir-4327
P5_5 nor-pre-miRNA>hsa-mir-449a
P5_5 nor-pre-miRNA>hsa-mir-449b
P5_5 nor-pre-miRNA>hsa-mir-449c
P5_5 nor-pre-miRNA>hsa-mir-450a-1
P5_5 nor-pre-miRNA>hsa-mir-450a-2
P5_5 nor-pre-miRNA>hsa-mir-450b
P5_5 nor-pre-miRNA>hsa-mir-452
P5_5 nor-pre-miRNA>hsa-mir-454
P5_5 nor-pre-miRNA>hsa-mir-455
P5_5 nor-pre-miRNA>hsa-mir-483
P5_5 nor-pre-miRNA>hsa-mir-484
P5_5 nor-pre-miRNA>hsa-mir-485
P5_5 nor-pre-miRNA>hsa-mir-486-2
P5_5 nor-pre-miRNA>hsa-mir-487a
P5_5 nor-pre-miRNA>hsa-mir-487b
P5_5 nor-pre-miRNA>hsa-mir-488
P5_5 nor-pre-miRNA>hsa-mir-489
P5_5 nor-pre-miRNA>hsa-mir-490
P5_5 nor-pre-miRNA>hsa-mir-491
P5_5 nor-pre-miRNA>hsa-mir-492
P5_5 nor-pre-miRNA>hsa-mir-493
P5_5 nor-pre-miRNA>hsa-mir-495
P5_5 nor-pre-miRNA>hsa-mir-497
P5_5 nor-pre-miRNA>hsa-mir-499a
P5_5 nor-pre-miRNA>hsa-mir-499b
P5_5 nor-pre-miRNA>hsa-mir-500a
P5_5 nor-pre-miRNA>hsa-mir-500b
P5_5 nor-pre-miRNA>hsa-mir-501
P5_5 nor-pre-miRNA>hsa-mir-502
P5_5 nor-pre-miRNA>hsa-mir-503

P5_5 nor-pre-miRNA>hsa-mir-505
P5_5 nor-pre-miRNA>hsa-mir-506
P5_5 nor-pre-miRNA>hsa-mir-508
P5_5 nor-pre-miRNA>hsa-mir-509-1
P5_5 nor-pre-miRNA>hsa-mir-509-2
P5_5 nor-pre-miRNA>hsa-mir-509-3
P5_5 nor-pre-miRNA>hsa-mir-510
P5_5 nor-pre-miRNA>hsa-mir-511
P5_5 nor-pre-miRNA>hsa-mir-512-1
P5_5 nor-pre-miRNA>hsa-mir-512-2
P5_5 nor-pre-miRNA>hsa-mir-513a-1
P5_5 nor-pre-miRNA>hsa-mir-513a-2
P5_5 nor-pre-miRNA>hsa-mir-513b
P5_5 nor-pre-miRNA>hsa-mir-513c
P5_5 nor-pre-miRNA>hsa-mir-514a-1
P5_5 nor-pre-miRNA>hsa-mir-514a-3
P5_5 nor-pre-miRNA>hsa-mir-515-1
P5_5 nor-pre-miRNA>hsa-mir-516a-1
P5_5 nor-pre-miRNA>hsa-mir-516a-2
P5_5 nor-pre-miRNA>hsa-mir-516b-1
P5_5 nor-pre-miRNA>hsa-mir-516b-2
P5_5 nor-pre-miRNA>hsa-mir-517a
P5_5 nor-pre-miRNA>hsa-mir-517b
P5_5 nor-pre-miRNA>hsa-mir-517c
P5_5 nor-pre-miRNA>hsa-mir-518a-1
P5_5 nor-pre-miRNA>hsa-mir-518a-2
P5_5 nor-pre-miRNA>hsa-mir-518c
P5_5 nor-pre-miRNA>hsa-mir-518d
P5_5 nor-pre-miRNA>hsa-mir-518f
P5_5 nor-pre-miRNA>hsa-mir-519a-1
P5_5 nor-pre-miRNA>hsa-mir-519b
P5_5 nor-pre-miRNA>hsa-mir-519c
P5_5 nor-pre-miRNA>hsa-mir-519d
P5_5 nor-pre-miRNA>hsa-mir-520a
P5_5 nor-pre-miRNA>hsa-mir-520c
P5_5 nor-pre-miRNA>hsa-mir-520d
P5_5 nor-pre-miRNA>hsa-mir-520f
P5_5 nor-pre-miRNA>hsa-mir-520g
P5_5 nor-pre-miRNA>hsa-mir-522
P5_5 nor-pre-miRNA>hsa-mir-523
P5_5 nor-pre-miRNA>hsa-mir-524
P5_5 nor-pre-miRNA>hsa-mir-525
P5_5 nor-pre-miRNA>hsa-mir-526a-1
P5_5 nor-pre-miRNA>hsa-mir-526a-2
P5_5 nor-pre-miRNA>hsa-mir-526b
P5_5 nor-pre-miRNA>hsa-mir-527
P5_5 nor-pre-miRNA>hsa-mir-532

P5_5 nor-pre-miRNA>hsa-mir-541
P5_5 nor-pre-miRNA>hsa-mir-542
P5_5 nor-pre-miRNA>hsa-mir-545
P5_5 nor-pre-miRNA>hsa-mir-548a-3
P5_5 nor-pre-miRNA>hsa-mir-548ab
P5_5 nor-pre-miRNA>hsa-mir-548ag-2
P5_5 nor-pre-miRNA>hsa-mir-548ai
P5_5 nor-pre-miRNA>hsa-mir-548aj-2
P5_5 nor-pre-miRNA>hsa-mir-548ak
P5_5 nor-pre-miRNA>hsa-mir-548am
P5_5 nor-pre-miRNA>hsa-mir-548an
P5_5 nor-pre-miRNA>hsa-mir-548ao
P5_5 nor-pre-miRNA>hsa-mir-548ap
P5_5 nor-pre-miRNA>hsa-mir-548aq
P5_5 nor-pre-miRNA>hsa-mir-548ar
P5_5 nor-pre-miRNA>hsa-mir-548as
P5_5 nor-pre-miRNA>hsa-mir-548at
P5_5 nor-pre-miRNA>hsa-mir-548av
P5_5 nor-pre-miRNA>hsa-mir-548aw
P5_5 nor-pre-miRNA>hsa-mir-548ay
P5_5 nor-pre-miRNA>hsa-mir-548b
P5_5 nor-pre-miRNA>hsa-mir-548ba
P5_5 nor-pre-miRNA>hsa-mir-548c
P5_5 nor-pre-miRNA>hsa-mir-548d-1
P5_5 nor-pre-miRNA>hsa-mir-548d-2
P5_5 nor-pre-miRNA>hsa-mir-548f-1
P5_5 nor-pre-miRNA>hsa-mir-548g
P5_5 nor-pre-miRNA>hsa-mir-548h-1
P5_5 nor-pre-miRNA>hsa-mir-548h-2
P5_5 nor-pre-miRNA>hsa-mir-548h-3
P5_5 nor-pre-miRNA>hsa-mir-548h-4
P5_5 nor-pre-miRNA>hsa-mir-548h-5
P5_5 nor-pre-miRNA>hsa-mir-659
P5_5 nor-pre-miRNA>hsa-mir-663a
P5_5 nor-pre-miRNA>hsa-mir-664a
P5_5 nor-pre-miRNA>hsa-mir-664b
P5_5 nor-pre-miRNA>hsa-mir-671
P5_5 nor-pre-miRNA>hsa-mir-675
P5_5 nor-pre-miRNA>hsa-mir-7-1
P5_5 nor-pre-miRNA>hsa-mir-7-2
P5_5 nor-pre-miRNA>hsa-mir-7-3
P5_5 nor-pre-miRNA>hsa-mir-769
P5_5 nor-pre-miRNA>hsa-mir-770
P5_5 nor-pre-miRNA>hsa-mir-802
P5_5 nor-pre-miRNA>hsa-mir-874
P5_5 nor-pre-miRNA>hsa-mir-876
P5_5 nor-pre-miRNA>hsa-mir-885

P5_5 nor-pre-miRNA>hsa-mir-888
P5_5 nor-pre-miRNA>hsa-mir-892c
P5_5 nor-pre-miRNA>hsa-mir-924
P5_5 nor-pre-miRNA>hsa-mir-93
P5_5 nor-pre-miRNA>hsa-mir-936
P5_5 nor-pre-miRNA>hsa-mir-937
P5_5 nor-pre-miRNA>hsa-mir-938
P5_5 nor-pre-miRNA>hsa-mir-939
P5_5 nor-pre-miRNA>hsa-mir-942
P5_5 nor-pre-miRNA>hsa-mir-95
P5_5 nor-pre-miRNA>hsa-mir-96
P5_5 nor-SNP 568106
P5_5 nor-SNP 568125
P5_5 nor-SNP 568176
P5_5 nor-SNP 568187
P5_5 nor-SNP 925742
P5_5 nor-SNP 925755
P5_5 nor-SNP 925764
P5_5 nor-SNP 925772
P5_5 nor-SNP 925776
P5_5 nor-SNP 925781
P5_5 nor-SNP 1062574
P5_5 nor-SNP 1062599
P5_5 nor-SNP 1062626
P5_5 nor-SNP 1062653
P5_5 nor-SNP 1062656
P5_5 nor-SNP 1062662
P5_5 nor-SNP 1102498
P5_5 nor-SNP 1102501
P5_5 nor-SNP 1102563
P5_5 nor-SNP 1102567
P5_5 nor-SNP 1103284
P5_5 nor-SNP 1103312
P5_5 nor-SNP 1103328
P5_5 nor-SNP 1103331
P5_5 nor-SNP 1708902
P5_5 nor-SNP 1708983
P5_5 nor-SNP 1749333
P5_5 nor-SNP 1785015
P5_5 nor-SNP 1785030
P5_5 nor-SNP 1785037
P5_5 nor-SNP 1785038
P5_5 nor-SNP 1785042
P5_5 nor-SNP 1785060
P5_5 nor-SNP 1816169
P5_5 nor-SNP 1816176
P5_5 nor-SNP 1880764

P5_5	nor-SNP	1988193
P5_5	nor-SNP	2018002
P5_5	nor-SNP	2018004
P5_5	nor-SNP	2018019
P5_5	nor-SNP	2018056
P5_5	nor-SNP	2140204
P5_5	nor-SNP	2140240
P5_5	nor-SNP	2140267
P5_5	nor-SNP	2140268
P5_5	nor-SNP	2140269
P5_5	nor-SNP	2155379
P5_5	nor-SNP	2155409
P5_5	nor-SNP	2234086
P5_5	nor-SNP	2234093
P5_5	nor-SNP	2581952
P5_5	nor-SNP	2581955
P5_5	nor-SNP	2581984
P5_5	nor-SNP	2581992
P5_5	nor-SNP	2633434
P5_5	nor-SNP	2633462
P5_5	nor-SNP	2633466
P5_5	nor-SNP	2633478
P5_5	nor-SNP	2633480
P5_5	nor-SNP	2651377
P5_5	nor-SNP	2651389
P5_5	nor-SNP	2651398
P5_5	nor-SNP	2651405
P5_5	nor-SNP	2651455
P5_5	nor-SNP	4770697
P5_5	nor-SNP	4770709
P5_5	nor-SNP	4770747
P5_5	nor-SNP	4770789
P5_5	nor-SNP	6920955
P5_5	nor-SNP	6920975
P5_5	nor-SNP	6920976
P5_5	nor-SNP	6921289
P5_5	nor-SNP	7073282
P5_5	nor-SNP	7073301
P5_5	nor-SNP	7073344
P5_5	nor-SNP	7126619
P5_5	nor-SNP	7126698
P5_5	nor-SNP	7256001
P5_5	nor-SNP	7256013
P5_5	nor-SNP	7256057
P5_5	nor-SNP	8007037
P5_5	nor-SNP	8007039
P5_5	nor-SNP	8007066

P5_5	nor-SNP	8007067
P5_5	nor-SNP	9211738
P5_5	nor-SNP	9211747
P5_5	nor-SNP	9211778
P5_5	nor-SNP	9211782
P5_5	nor-SNP	9211802
P5_5	nor-SNP	10287820
P5_5	nor-SNP	10287821
P5_5	nor-SNP	10287824
P5_5	nor-SNP	10287854
P5_5	nor-SNP	10436180
P5_5	nor-SNP	10436187
P5_5	nor-SNP	10436194
P5_5	nor-SNP	10436201
P5_5	nor-SNP	10436219
P5_5	nor-SNP	10436238
P5_5	nor-SNP	10514157
P5_5	nor-SNP	10514190
P5_5	nor-SNP	10514194
P5_5	nor-SNP	10514200
P5_5	nor-SNP	10514202
P5_5	nor-SNP	10524531
P5_5	nor-SNP	10662844
P5_5	nor-SNP	10662859
P5_5	nor-SNP	10662866
P5_5	nor-SNP	10928119
P5_5	nor-SNP	10928130
P5_5	nor-SNP	10928149
P5_5	nor-SNP	11400303
P5_5	nor-SNP	11400324
P5_5	nor-SNP	11400356
P5_5	nor-SNP	11400358
P5_5	nor-SNP	12172775
P5_5	nor-SNP	12172783
P5_5	nor-SNP	12172788
P5_5	nor-SNP	12172799
P5_5	nor-SNP	12172800
P5_5	nor-SNP	12820658
P5_5	nor-SNP	12877500
P5_5	nor-SNP	12877501
P5_5	nor-SNP	12877502
P5_5	nor-SNP	12877520
P5_5	nor-SNP	12877525
P5_5	nor-SNP	12877527
P5_5	nor-SNP	13446846
P5_5	nor-SNP	13446848
P5_5	nor-SNP	13446849

P5_5	nor-SNP	13446893
P5_5	nor-SNP	13446906
P5_5	nor-SNP	13446924
P5_5	nor-SNP	13947292
P5_5	nor-SNP	13947296
P5_5	nor-SNP	13947436
P5_5	nor-SNP	13985514
P5_5	nor-SNP	13985689
P5_5	nor-SNP	13985721
P5_5	nor-SNP	13985739
P5_5	nor-SNP	13985772
P5_5	nor-SNP	13985805
P5_5	nor-SNP	13985806
P5_5	nor-SNP	14403144
P5_5	nor-SNP	14478575
P5_5	nor-SNP	14478576
P5_5	nor-SNP	14478613
P5_5	nor-SNP	14478618
P5_5	nor-SNP	14710999
P5_5	nor-SNP	14711013
P5_5	nor-SNP	14778711
P5_5	nor-SNP	14778721
P5_5	nor-SNP	14830172
P5_5	nor-SNP	14830197
P5_5	nor-SNP	14830201
P5_5	nor-SNP	14830215
P5_5	nor-SNP	14830216
P5_5	nor-SNP	15560380
P5_5	nor-SNP	15737151
P5_5	nor-SNP	15737177
P5_5	nor-SNP	15737185
P5_5	nor-SNP	15737186
P5_5	nor-SNP	15737217
P5_5	nor-SNP	15737218
P5_5	nor-SNP	16645137
P5_5	nor-SNP	16645178
P5_5	nor-SNP	16645199
P5_5	nor-SNP	16645208
P5_5	nor-SNP	16974725
P5_5	nor-SNP	16974739
P5_5	nor-SNP	17717209
P5_5	nor-SNP	17717243
P5_5	nor-SNP	17717244
P5_5	nor-SNP	17962615
P5_5	nor-SNP	17962644
P5_5	nor-SNP	18134042
P5_5	nor-SNP	18134045

P5_5	nor-SNP	18134091
P5_5	nor-SNP	18409354
P5_5	nor-SNP	18451300
P5_5	nor-SNP	18451325
P5_5	nor-SNP	18573331
P5_5	nor-SNP	18573360
P5_5	nor-SNP	18573361
P5_5	nor-SNP	18573374
P5_5	nor-SNP	19247825
P5_5	nor-SNP	19405672
P5_5	nor-SNP	19405676
P5_5	nor-SNP	19405702
P5_5	nor-SNP	19405709
P5_5	nor-SNP	19405743
P5_5	nor-SNP	20020733
P5_5	nor-SNP	20179097
P5_5	nor-SNP	20179098
P5_5	nor-SNP	20179130
P5_5	nor-SNP	20529956
P5_5	nor-SNP	20529990
P5_5	nor-SNP	20716104
P5_5	nor-SNP	20716128
P5_5	nor-SNP	21785508
P5_5	nor-SNP	22007594
P5_5	nor-SNP	22007634
P5_5	nor-SNP	23682350
P5_5	nor-SNP	23682383
P5_5	nor-SNP	23887219
P5_5	nor-SNP	23887220
P5_5	nor-SNP	23887271
P5_5	nor-SNP	24214442
P5_5	nor-SNP	24214486
P5_5	nor-SNP	24214493
P5_5	nor-SNP	24214532
P5_5	nor-SNP	24736590
P5_5	nor-SNP	24736638
P5_5	nor-SNP	25551550
P5_5	nor-SNP	25551583
P5_5	nor-SNP	25551588
P5_5	nor-SNP	26188825
P5_5	nor-SNP	26188865
P5_5	nor-SNP	26188880
P5_5	nor-SNP	26188906
P5_5	nor-SNP	26188908
P5_5	nor-SNP	26906402
P5_5	nor-SNP	26906403
P5_5	nor-SNP	26906423

P5_5	nor-SNP	26906437
P5_5	nor-SNP	26906452
P5_5	nor-SNP	26906471
P5_5	nor-SNP	26946325
P5_5	nor-SNP	27115438
P5_5	nor-SNP	27115444
P5_5	nor-SNP	27115447
P5_5	nor-SNP	27115458
P5_5	nor-SNP	27115467
P5_5	nor-SNP	27209165
P5_5	nor-SNP	27559214
P5_5	nor-SNP	27559261
P5_5	nor-SNP	28102427
P5_5	nor-SNP	28102477
P5_5	nor-SNP	28102484
P5_5	nor-SNP	28316513
P5_5	nor-SNP	28316591
P5_5	nor-SNP	28316592
P5_5	nor-SNP	28316597
P5_5	nor-SNP	28444104
P5_5	nor-SNP	28444157
P5_5	nor-SNP	28444183
P5_5	nor-SNP	28863628
P5_5	nor-SNP	28863633
P5_5	nor-SNP	28863695
P5_5	nor-SNP	29814796
P5_5	nor-SNP	29814813
P5_5	nor-SNP	29814829
P5_5	nor-SNP	29814842
P5_5	nor-SNP	29887033
P5_5	nor-SNP	29891235
P5_5	nor-SNP	29891245
P5_5	nor-SNP	29891260
P5_5	nor-SNP	29891262
P5_5	nor-SNP	29902536
P5_5	nor-SNP	31357238
P5_5	nor-SNP	31357244
P5_5	nor-SNP	31357245
P5_5	nor-SNP	31357301
P5_5	nor-SNP	31357325
P5_5	nor-SNP	31556085
P5_5	nor-SNP	31747678
P5_5	nor-SNP	31924631
P5_5	nor-SNP	31924708
P5_5	nor-SNP	32547795
P5_5	nor-SNP	32547809
P5_5	nor-SNP	32547810

P5_5	nor-SNP	32547811
P5_5	nor-SNP	32717702
P5_5	nor-SNP	32717722
P5_5	nor-SNP	33175642
P5_5	nor-SNP	33175702
P5_5	nor-SNP	33484792
P5_5	nor-SNP	33484837
P5_5	nor-SNP	33578201
P5_5	nor-SNP	33578202
P5_5	nor-SNP	33578205
P5_5	nor-SNP	33578206
P5_5	nor-SNP	33578251
P5_5	nor-SNP	33578255
P5_5	nor-SNP	33578276
P5_5	nor-SNP	33798007
P5_5	nor-SNP	33798031
P5_5	nor-SNP	33798091
P5_5	nor-SNP	33967787
P5_5	nor-SNP	33967814
P5_5	nor-SNP	34674326
P5_5	nor-SNP	34674345
P5_5	nor-SNP	34820530
P5_5	nor-SNP	34820566
P5_5	nor-SNP	34963416
P5_5	nor-SNP	34963445
P5_5	nor-SNP	34963459
P5_5	nor-SNP	35391051
P5_5	nor-SNP	35391065
P5_5	nor-SNP	35391066
P5_5	nor-SNP	35391105
P5_5	nor-SNP	36428006
P5_5	nor-SNP	36428017
P5_5	nor-SNP	36428046
P5_5	nor-SNP	36428048
P5_5	nor-SNP	36958963
P5_5	nor-SNP	36958995
P5_5	nor-SNP	36959006
P5_5	nor-SNP	36959015
P5_5	nor-SNP	37093013
P5_5	nor-SNP	37093078
P5_5	nor-SNP	37093089
P5_5	nor-SNP	37093097
P5_5	nor-SNP	37202092
P5_5	nor-SNP	37202112
P5_5	nor-SNP	37202113
P5_5	nor-SNP	37202138
P5_5	nor-SNP	37883165

P5_5	nor-SNP	37883195
P5_5	nor-SNP	37883200
P5_5	nor-SNP	38010903
P5_5	nor-SNP	38010938
P5_5	nor-SNP	38010964
P5_5	nor-SNP	38243727
P5_5	nor-SNP	38243739
P5_5	nor-SNP	38243743
P5_5	nor-SNP	38243770
P5_5	nor-SNP	39696856
P5_5	nor-SNP	39696863
P5_5	nor-SNP	40238175
P5_5	nor-SNP	40238258
P5_5	nor-SNP	40646795
P5_5	nor-SNP	40646803
P5_5	nor-SNP	40646816
P5_5	nor-SNP	40646834
P5_5	nor-SNP	41128578
P5_5	nor-SNP	41128599
P5_5	nor-SNP	41128620
P5_5	nor-SNP	41220077
P5_5	nor-SNP	41220094
P5_5	nor-SNP	41517974
P5_5	nor-SNP	41517981
P5_5	nor-SNP	41518005
P5_5	nor-SNP	41518007
P5_5	nor-SNP	41518025
P5_5	nor-SNP	41675171
P5_5	nor-SNP	42296995
P5_5	nor-SNP	42319269
P5_5	nor-SNP	42319276
P5_5	nor-SNP	42319291
P5_5	nor-SNP	43602984
P5_5	nor-SNP	43602992
P5_5	nor-SNP	44085868
P5_5	nor-SNP	44155749
P5_5	nor-SNP	44155754
P5_5	nor-SNP	44333749
P5_5	nor-SNP	45605666
P5_5	nor-SNP	45606471
P5_5	nor-SNP	45606472
P5_5	nor-SNP	45606504
P5_5	nor-SNP	45606510
P5_5	nor-SNP	45659490
P5_5	nor-SNP	45659500
P5_5	nor-SNP	46114572
P5_5	nor-SNP	46114580

P5_5	nor-SNP	46114610
P5_5	nor-SNP	46142293
P5_5	nor-SNP	46233806
P5_5	nor-SNP	46233848
P5_5	nor-SNP	46233866
P5_5	nor-SNP	46486996
P5_5	nor-SNP	46509569
P5_5	nor-SNP	46509616
P5_5	nor-SNP	46522190
P5_5	nor-SNP	46522201
P5_5	nor-SNP	46522255
P5_5	nor-SNP	46522298
P5_5	nor-SNP	46657254
P5_5	nor-SNP	46657289
P5_5	nor-SNP	46709859
P5_5	nor-SNP	46709875
P5_5	nor-SNP	46709899
P5_5	nor-SNP	46801821
P5_5	nor-SNP	47891069
P5_5	nor-SNP	47891106
P5_5	nor-SNP	47891117
P5_5	nor-SNP	48118347
P5_5	nor-SNP	48118349
P5_5	nor-SNP	48118350
P5_5	nor-SNP	48118351
P5_5	nor-SNP	48118365
P5_5	nor-SNP	48118374
P5_5	nor-SNP	49048263
P5_5	nor-SNP	49048277
P5_5	nor-SNP	49767769
P5_5	nor-SNP	49767815
P5_5	nor-SNP	49767832
P5_5	nor-SNP	49767835
P5_5	nor-SNP	49767838
P5_5	nor-SNP	49768168
P5_5	nor-SNP	49768171
P5_5	nor-SNP	49773042
P5_5	nor-SNP	49773087
P5_5	nor-SNP	49773090
P5_5	nor-SNP	49773603
P5_5	nor-SNP	49774381
P5_5	nor-SNP	49774383
P5_5	nor-SNP	49774389
P5_5	nor-SNP	49775296
P5_5	nor-SNP	49775337
P5_5	nor-SNP	49775351
P5_5	nor-SNP	49779210

P5_5	nor-SNP	49779214
P5_5	nor-SNP	49779234
P5_5	nor-SNP	49937076
P5_5	nor-SNP	49937087
P5_5	nor-SNP	50193625
P5_5	nor-SNP	50623110
P5_5	nor-SNP	50623143
P5_5	nor-SNP	50627935
P5_5	nor-SNP	50627936
P5_5	nor-SNP	50627942
P5_5	nor-SNP	52196045
P5_5	nor-SNP	52196076
P5_5	nor-SNP	52196528
P5_5	nor-SNP	52196574
P5_5	nor-SNP	52328248
P5_5	nor-SNP	52328298
P5_5	nor-SNP	53394424
P5_5	nor-SNP	54076326
P5_5	nor-SNP	54076332
P5_5	nor-SNP	54169954
P5_5	nor-SNP	54169973
P5_5	nor-SNP	54170006
P5_5	nor-SNP	54170016
P5_5	nor-SNP	54172483
P5_5	nor-SNP	54172501
P5_5	nor-SNP	54172508
P5_5	nor-SNP	54175287
P5_5	nor-SNP	54175294
P5_5	nor-SNP	54182261
P5_5	nor-SNP	54182325
P5_5	nor-SNP	54182326
P5_5	nor-SNP	54185441
P5_5	nor-SNP	54185457
P5_5	nor-SNP	54185481
P5_5	nor-SNP	54185492
P5_5	nor-SNP	54189751
P5_5	nor-SNP	54189752
P5_5	nor-SNP	54191794
P5_5	nor-SNP	54194135
P5_5	nor-SNP	54194212
P5_5	nor-SNP	54197660
P5_5	nor-SNP	54197674
P5_5	nor-SNP	54197678
P5_5	nor-SNP	54197706
P5_5	nor-SNP	54198496
P5_5	nor-SNP	54198499
P5_5	nor-SNP	54200810

P5_5	nor-SNP	54200826
P5_5	nor-SNP	54200830
P5_5	nor-SNP	54200834
P5_5	nor-SNP	54200843
P5_5	nor-SNP	54200853
P5_5	nor-SNP	54201654
P5_5	nor-SNP	54201667
P5_5	nor-SNP	54201668
P5_5	nor-SNP	54201692
P5_5	nor-SNP	54201695
P5_5	nor-SNP	54201703
P5_5	nor-SNP	54203326
P5_5	nor-SNP	54203333
P5_5	nor-SNP	54203347
P5_5	nor-SNP	54209517
P5_5	nor-SNP	54209527
P5_5	nor-SNP	54209563
P5_5	nor-SNP	54210734
P5_5	nor-SNP	54210736
P5_5	nor-SNP	54210774
P5_5	nor-SNP	54211990
P5_5	nor-SNP	54212075
P5_5	nor-SNP	54214286
P5_5	nor-SNP	54214312
P5_5	nor-SNP	54214333
P5_5	nor-SNP	54215584
P5_5	nor-SNP	54215608
P5_5	nor-SNP	54216615
P5_5	nor-SNP	54216616
P5_5	nor-SNP	54216629
P5_5	nor-SNP	54216650
P5_5	nor-SNP	54216670
P5_5	nor-SNP	54216681
P5_5	nor-SNP	54223379
P5_5	nor-SNP	54223433
P5_5	nor-SNP	54224382
P5_5	nor-SNP	54225426
P5_5	nor-SNP	54225437
P5_5	nor-SNP	54225460
P5_5	nor-SNP	54225463
P5_5	nor-SNP	54225490
P5_5	nor-SNP	54225501
P5_5	nor-SNP	54228719
P5_5	nor-SNP	54228742
P5_5	nor-SNP	54228743
P5_5	nor-SNP	54228750
P5_5	nor-SNP	54228774

P5_5	nor-SNP	54228775
P5_5	nor-SNP	54230219
P5_5	nor-SNP	54234260
P5_5	nor-SNP	54234265
P5_5	nor-SNP	54234315
P5_5	nor-SNP	54234340
P5_5	nor-SNP	54238159
P5_5	nor-SNP	54238182
P5_5	nor-SNP	54238189
P5_5	nor-SNP	54238203
P5_5	nor-SNP	54238208
P5_5	nor-SNP	54240136
P5_5	nor-SNP	54240137
P5_5	nor-SNP	54240142
P5_5	nor-SNP	54240174
P5_5	nor-SNP	54240184
P5_5	nor-SNP	54242630
P5_5	nor-SNP	54244647
P5_5	nor-SNP	54254494
P5_5	nor-SNP	54254543
P5_5	nor-SNP	54255679
P5_5	nor-SNP	54255689
P5_5	nor-SNP	54257304
P5_5	nor-SNP	54257325
P5_5	nor-SNP	54260002
P5_5	nor-SNP	54260009
P5_5	nor-SNP	54260068
P5_5	nor-SNP	54260075
P5_5	nor-SNP	54261549
P5_5	nor-SNP	54261556
P5_5	nor-SNP	54261562
P5_5	nor-SNP	54261563
P5_5	nor-SNP	54264394
P5_5	nor-SNP	54264421
P5_5	nor-SNP	54264461
P5_5	nor-SNP	54264462
P5_5	nor-SNP	54264468
P5_5	nor-SNP	54290994
P5_5	nor-SNP	54290995
P5_5	nor-SNP	54291161
P5_5	nor-SNP	54291965
P5_5	nor-SNP	54292016
P5_5	nor-SNP	54385558
P5_5	nor-SNP	54385561
P5_5	nor-SNP	54385584
P5_5	nor-SNP	54385599
P5_5	nor-SNP	54385629

P5_5	nor-SNP	54466362
P5_5	nor-SNP	54466378
P5_5	nor-SNP	54466527
P5_5	nor-SNP	54466544
P5_5	nor-SNP	54468094
P5_5	nor-SNP	54468110
P5_5	nor-SNP	54468124
P5_5	nor-SNP	54468144
P5_5	nor-SNP	54468150
P5_5	nor-SNP	54468166
P5_5	nor-SNP	54731000
P5_5	nor-SNP	54731071
P5_5	nor-SNP	54731080
P5_5	nor-SNP	56118358
P5_5	nor-SNP	56118359
P5_5	nor-SNP	56216090
P5_5	nor-SNP	56216092
P5_5	nor-SNP	56216156
P5_5	nor-SNP	56216187
P5_5	nor-SNP	56227905
P5_5	nor-SNP	56227910
P5_5	nor-SNP	56367696
P5_5	nor-SNP	56408599
P5_5	nor-SNP	56408625
P5_5	nor-SNP	56408639
P5_5	nor-SNP	56892431
P5_5	nor-SNP	56892464
P5_5	nor-SNP	56892470
P5_5	nor-SNP	56892507
P5_5	nor-SNP	57023497
P5_5	nor-SNP	57215120
P5_5	nor-SNP	57215164
P5_5	nor-SNP	57228573
P5_5	nor-SNP	57228574
P5_5	nor-SNP	57392678
P5_5	nor-SNP	57392686
P5_5	nor-SNP	57392697
P5_5	nor-SNP	57392715
P5_5	nor-SNP	57472742
P5_5	nor-SNP	57588322
P5_5	nor-SNP	57588323
P5_5	nor-SNP	57588336
P5_5	nor-SNP	57918678
P5_5	nor-SNP	59139674
P5_5	nor-SNP	59362576
P5_5	nor-SNP	60528617
P5_5	nor-SNP	60528670

P5_5	nor-SNP	61162131
P5_5	nor-SNP	61162191
P5_5	nor-SNP	61582659
P5_5	nor-SNP	61582680
P5_5	nor-SNP	61582708
P5_5	nor-SNP	61773937
P5_5	nor-SNP	61809907
P5_5	nor-SNP	62496924
P5_5	nor-SNP	62496926
P5_5	nor-SNP	62572847
P5_5	nor-SNP	62572874
P5_5	nor-SNP	62572885
P5_5	nor-SNP	62997470
P5_5	nor-SNP	63005918
P5_5	nor-SNP	63081542
P5_5	nor-SNP	63116226
P5_5	nor-SNP	64561772
P5_5	nor-SNP	64561812
P5_5	nor-SNP	64658623
P5_5	nor-SNP	64658640
P5_5	nor-SNP	64658705
P5_5	nor-SNP	64658710
P5_5	nor-SNP	64658715
P5_5	nor-SNP	64658716
P5_5	nor-SNP	65016300
P5_5	nor-SNP	65016325
P5_5	nor-SNP	65054606
P5_5	nor-SNP	65054649
P5_5	nor-SNP	65054668
P5_5	nor-SNP	65054709
P5_5	nor-SNP	65238733
P5_5	nor-SNP	65238751
P5_5	nor-SNP	65238767
P5_5	nor-SNP	65238773
P5_5	nor-SNP	65238806
P5_5	nor-SNP	65291712
P5_5	nor-SNP	65291793
P5_5	nor-SNP	65291808
P5_5	nor-SNP	65467665
P5_5	nor-SNP	65467669
P5_5	nor-SNP	65467701
P5_5	nor-SNP	66701915
P5_5	nor-SNP	66701926
P5_5	nor-SNP	66701974
P5_5	nor-SNP	67094145
P5_5	nor-SNP	67094150
P5_5	nor-SNP	67094171

P5_5	nor-SNP	67236292
P5_5	nor-SNP	68649205
P5_5	nor-SNP	68649258
P5_5	nor-SNP	68649260
P5_5	nor-SNP	68649290
P5_5	nor-SNP	68850698
P5_5	nor-SNP	68850706
P5_5	nor-SNP	68850720
P5_5	nor-SNP	69330818
P5_5	nor-SNP	69330823
P5_5	nor-SNP	69330824
P5_5	nor-SNP	69330825
P5_5	nor-SNP	69330871
P5_5	nor-SNP	69330886
P5_5	nor-SNP	69966985
P5_5	nor-SNP	69966994
P5_5	nor-SNP	69967005
P5_5	nor-SNP	69967062
P5_5	nor-SNP	70519078
P5_5	nor-SNP	70519093
P5_5	nor-SNP	70519169
P5_5	nor-SNP	70718466
P5_5	nor-SNP	71591156
P5_5	nor-SNP	71591164
P5_5	nor-SNP	71591179
P5_5	nor-SNP	71591230
P5_5	nor-SNP	72086720
P5_5	nor-SNP	72113261
P5_5	nor-SNP	72113269
P5_5	nor-SNP	72113270
P5_5	nor-SNP	72113306
P5_5	nor-SNP	73125664
P5_5	nor-SNP	73402156
P5_5	nor-SNP	73506984
P5_5	nor-SNP	76225837
P5_5	nor-SNP	76225848
P5_5	nor-SNP	76225915
P5_5	nor-SNP	78072635
P5_5	nor-SNP	78072651
P5_5	nor-SNP	78072653
P5_5	nor-SNP	78072676
P5_5	nor-SNP	79099712
P5_5	nor-SNP	79099736
P5_5	nor-SNP	79099750
P5_5	nor-SNP	79107017
P5_5	nor-SNP	79107049
P5_5	nor-SNP	79107061

P5_5	nor-SNP	79107068
P5_5	nor-SNP	79107084
P5_5	nor-SNP	79418149
P5_5	nor-SNP	79813049
P5_5	nor-SNP	79813075
P5_5	nor-SNP	79813083
P5_5	nor-SNP	83541972
P5_5	nor-SNP	83541979
P5_5	nor-SNP	83541980
P5_5	nor-SNP	83541990
P5_5	nor-SNP	85158670
P5_5	nor-SNP	85775260
P5_5	nor-SNP	85775275
P5_5	nor-SNP	85916322
P5_5	nor-SNP	86313731
P5_5	nor-SNP	86313782
P5_5	nor-SNP	86313794
P5_5	nor-SNP	86368890
P5_5	nor-SNP	86368898
P5_5	nor-SNP	86368922
P5_5	nor-SNP	86368929
P5_5	nor-SNP	86368959
P5_5	nor-SNP	86410766
P5_5	nor-SNP	86584675
P5_5	nor-SNP	86584707
P5_5	nor-SNP	86584720
P5_5	nor-SNP	88024452
P5_5	nor-SNP	88024462
P5_5	nor-SNP	89151351
P5_5	nor-SNP	89151354
P5_5	nor-SNP	89151411
P5_5	nor-SNP	89155064
P5_5	nor-SNP	89155073
P5_5	nor-SNP	89155084
P5_5	nor-SNP	89155121
P5_5	nor-SNP	89155162
P5_5	nor-SNP	89155163
P5_5	nor-SNP	90602296
P5_5	nor-SNP	90602368
P5_5	nor-SNP	92002896
P5_5	nor-SNP	92003009
P5_5	nor-SNP	92003356
P5_5	nor-SNP	92956409
P5_5	nor-SNP	92956412
P5_5	nor-SNP	92956416
P5_5	nor-SNP	92956420
P5_5	nor-SNP	92956422

P5_5	nor-SNP	92956443
P5_5	nor-SNP	93113270
P5_5	nor-SNP	93113314
P5_5	nor-SNP	93113323
P5_5	nor-SNP	93113326
P5_5	nor-SNP	93142427
P5_5	nor-SNP	93447631
P5_5	nor-SNP	93447646
P5_5	nor-SNP	93447674
P5_5	nor-SNP	93447695
P5_5	nor-SNP	93447700
P5_5	nor-SNP	93447702
P5_5	nor-SNP	93466866
P5_5	nor-SNP	93466909
P5_5	nor-SNP	93466910
P5_5	nor-SNP	93466912
P5_5	nor-SNP	93466913
P5_5	nor-SNP	93466919
P5_5	nor-SNP	95228179
P5_5	nor-SNP	95228286
P5_5	nor-SNP	95604259
P5_5	nor-SNP	96074607
P5_5	nor-SNP	96074638
P5_5	nor-SNP	96074649
P5_5	nor-SNP	96085190
P5_5	nor-SNP	97464049
P5_5	nor-SNP	97464057
P5_5	nor-SNP	97572244
P5_5	nor-SNP	97824075
P5_5	nor-SNP	97824125
P5_5	nor-SNP	97824129
P5_5	nor-SNP	97824145
P5_5	nor-SNP	97847498
P5_5	nor-SNP	97847569
P5_5	nor-SNP	97847573
P5_5	nor-SNP	97847807
P5_5	nor-SNP	97848319
P5_5	nor-SNP	97848343
P5_5	nor-SNP	97885708
P5_5	nor-SNP	97885715
P5_5	nor-SNP	97885720
P5_5	nor-SNP	97885730
P5_5	nor-SNP	97957646
P5_5	nor-SNP	97957677
P5_5	nor-SNP	98510847
P5_5	nor-SNP	98510864
P5_5	nor-SNP	98510896

P5_5	nor-SNP	98510902
P5_5	nor-SNP	98860801
P5_5	nor-SNP	98860808
P5_5	nor-SNP	98860816
P5_5	nor-SNP	98860827
P5_5	nor-SNP	98860840
P5_5	nor-SNP	99572563
P5_5	nor-SNP	99691200
P5_5	nor-SNP	99691393
P5_5	nor-SNP	99691396
P5_5	nor-SNP	99691401
P5_5	nor-SNP	99691429
P5_5	nor-SNP	99691652
P5_5	nor-SNP	99691653
P5_5	nor-SNP	100154978
P5_5	nor-SNP	100155019
P5_5	nor-SNP	100155033
P5_5	nor-SNP	100576035
P5_5	nor-SNP	100774203
P5_5	nor-SNP	100774268
P5_5	nor-SNP	100774277
P5_5	nor-SNP	100774289
P5_5	nor-SNP	101318740
P5_5	nor-SNP	101318783
P5_5	nor-SNP	101318784
P5_5	nor-SNP	101318807
P5_5	nor-SNP	101335438
P5_5	nor-SNP	101335451
P5_5	nor-SNP	101340844
P5_5	nor-SNP	101340862
P5_5	nor-SNP	101347355
P5_5	nor-SNP	101347408
P5_5	nor-SNP	101350873
P5_5	nor-SNP	101351048
P5_5	nor-SNP	101351088
P5_5	nor-SNP	101377478
P5_5	nor-SNP	101488414
P5_5	nor-SNP	101488424
P5_5	nor-SNP	101488430
P5_5	nor-SNP	101488431
P5_5	nor-SNP	101489677
P5_5	nor-SNP	101489685
P5_5	nor-SNP	101489703
P5_5	nor-SNP	101489714
P5_5	nor-SNP	101489728
P5_5	nor-SNP	101489745
P5_5	nor-SNP	101490145

P5_5	nor-SNP	101490178
P5_5	nor-SNP	101491378
P5_5	nor-SNP	101491407
P5_5	nor-SNP	101493129
P5_5	nor-SNP	101493478
P5_5	nor-SNP	101496416
P5_5	nor-SNP	101500098
P5_5	nor-SNP	101500126
P5_5	nor-SNP	101500133
P5_5	nor-SNP	101500166
P5_5	nor-SNP	101500167
P5_5	nor-SNP	101506441
P5_5	nor-SNP	101506465
P5_5	nor-SNP	101509363
P5_5	nor-SNP	101510579
P5_5	nor-SNP	101510612
P5_5	nor-SNP	101510613
P5_5	nor-SNP	101512288
P5_5	nor-SNP	101512304
P5_5	nor-SNP	101512836
P5_5	nor-SNP	101518827
P5_5	nor-SNP	101520657
P5_5	nor-SNP	101520687
P5_5	nor-SNP	101521052
P5_5	nor-SNP	101521092
P5_5	nor-SNP	101521764
P5_5	nor-SNP	101522556
P5_5	nor-SNP	101522578
P5_5	nor-SNP	101522582
P5_5	nor-SNP	101522589
P5_5	nor-SNP	101522631
P5_5	nor-SNP	101526116
P5_5	nor-SNP	101528401
P5_5	nor-SNP	101528426
P5_5	nor-SNP	101530833
P5_5	nor-SNP	101530834
P5_5	nor-SNP	101530873
P5_5	nor-SNP	101531654
P5_5	nor-SNP	101531806
P5_5	nor-SNP	101531849
P5_5	nor-SNP	101531854
P5_5	nor-SNP	101531857
P5_5	nor-SNP	101531858
P5_5	nor-SNP	101531862
P5_5	nor-SNP	101532279
P5_5	nor-SNP	102251501
P5_5	nor-SNP	102251532

P5_5	nor-SNP	102251542
P5_5	nor-SNP	102619995
P5_5	nor-SNP	102620081
P5_5	nor-SNP	103006026
P5_5	nor-SNP	103006047
P5_5	nor-SNP	103242937
P5_5	nor-SNP	103242954
P5_5	nor-SNP	103361221
P5_5	nor-SNP	103361226
P5_5	nor-SNP	103361245
P5_5	nor-SNP	104166899
P5_5	nor-SNP	104166902
P5_5	nor-SNP	104166904
P5_5	nor-SNP	104196269
P5_5	nor-SNP	104196300
P5_5	nor-SNP	104324231
P5_5	nor-SNP	104324238
P5_5	nor-SNP	104324266
P5_5	nor-SNP	104583759
P5_5	nor-SNP	104583776
P5_5	nor-SNP	104583796
P5_5	nor-SNP	104583804
P5_5	nor-SNP	104583828
P5_5	nor-SNP	104583838
P5_5	nor-SNP	104985443
P5_5	nor-SNP	105154013
P5_5	nor-SNP	105154084
P5_5	nor-SNP	105154089
P5_5	nor-SNP	105154091
P5_5	nor-SNP	105154097
P5_5	nor-SNP	105496622
P5_5	nor-SNP	105496638
P5_5	nor-SNP	105496675
P5_5	nor-SNP	105807855
P5_5	nor-SNP	105807858
P5_5	nor-SNP	105807864
P5_5	nor-SNP	105807909
P5_5	nor-SNP	105807912
P5_5	nor-SNP	105883051
P5_5	nor-SNP	105883081
P5_5	nor-SNP	105883120
P5_5	nor-SNP	110141523
P5_5	nor-SNP	110141578
P5_5	nor-SNP	110827551
P5_5	nor-SNP	110827558
P5_5	nor-SNP	111781744
P5_5	nor-SNP	111781746

P5_5	nor-SNP	111781785
P5_5	nor-SNP	111781790
P5_5	nor-SNP	113569054
P5_5	nor-SNP	113569088
P5_5	nor-SNP	113569193
P5_5	nor-SNP	113569207
P5_5	nor-SNP	113569211
P5_5	nor-SNP	113569394
P5_5	nor-SNP	113569406
P5_5	nor-SNP	113569519
P5_5	nor-SNP	113569680
P5_5	nor-SNP	113569692
P5_5	nor-SNP	113569695
P5_5	nor-SNP	113569696
P5_5	nor-SNP	113997746
P5_5	nor-SNP	113997768
P5_5	nor-SNP	113997812
P5_5	nor-SNP	113997817
P5_5	nor-SNP	114028104
P5_5	nor-SNP	115010026
P5_5	nor-SNP	115933876
P5_5	nor-SNP	115933890
P5_5	nor-SNP	115933905
P5_5	nor-SNP	115933913
P5_5	nor-SNP	115933918
P5_5	nor-SNP	115933922
P5_5	nor-SNP	116971750
P5_5	nor-SNP	116971778
P5_5	nor-SNP	117520360
P5_5	nor-SNP	117520389
P5_5	nor-SNP	117637290
P5_5	nor-SNP	117637302
P5_5	nor-SNP	117637325
P5_5	nor-SNP	117637326
P5_5	nor-SNP	118927256
P5_5	nor-SNP	119390263
P5_5	nor-SNP	119390286
P5_5	nor-SNP	119390294
P5_5	nor-SNP	120114558
P5_5	nor-SNP	122017258
P5_5	nor-SNP	124021017
P5_5	nor-SNP	124021033
P5_5	nor-SNP	124021052
P5_5	nor-SNP	124021054
P5_5	nor-SNP	124360347
P5_5	nor-SNP	126721357
P5_5	nor-SNP	126721419

P5_5	nor-SNP	127456020
P5_5	nor-SNP	127456029
P5_5	nor-SNP	127456042
P5_5	nor-SNP	127456067
P5_5	nor-SNP	127456068
P5_5	nor-SNP	127847960
P5_5	nor-SNP	128778703
P5_5	nor-SNP	128808252
P5_5	nor-SNP	128808253
P5_5	nor-SNP	128972923
P5_5	nor-SNP	128972925
P5_5	nor-SNP	129061441
P5_5	nor-SNP	129061462
P5_5	nor-SNP	129162366
P5_5	nor-SNP	129162409
P5_5	nor-SNP	129162428
P5_5	nor-SNP	129162433
P5_5	nor-SNP	129410227
P5_5	nor-SNP	129410228
P5_5	nor-SNP	129410235
P5_5	nor-SNP	129410239
P5_5	nor-SNP	129410313
P5_5	nor-SNP	129414568
P5_5	nor-SNP	129414574
P5_5	nor-SNP	129414804
P5_5	nor-SNP	129414806
P5_5	nor-SNP	129414807
P5_5	nor-SNP	129414815
P5_5	nor-SNP	129414843
P5_5	nor-SNP	129414852
P5_5	nor-SNP	130135961
P5_5	nor-SNP	130452968
P5_5	nor-SNP	130452977
P5_5	nor-SNP	130452987
P5_5	nor-SNP	131007001
P5_5	nor-SNP	131007004
P5_5	nor-SNP	131007052
P5_5	nor-SNP	131007087
P5_5	nor-SNP	131007109
P5_5	nor-SNP	131154943
P5_5	nor-SNP	131641629
P5_5	nor-SNP	132113344
P5_5	nor-SNP	132113349
P5_5	nor-SNP	132113371
P5_5	nor-SNP	132760893
P5_5	nor-SNP	132760931
P5_5	nor-SNP	133303425

P5_5	nor-SNP	133303465
P5_5	nor-SNP	133303744
P5_5	nor-SNP	133303758
P5_5	nor-SNP	133303896
P5_5	nor-SNP	133674285
P5_5	nor-SNP	133674399
P5_5	nor-SNP	133674418
P5_5	nor-SNP	133674439
P5_5	nor-SNP	133674576
P5_5	nor-SNP	133674612
P5_5	nor-SNP	133675375
P5_5	nor-SNP	133680381
P5_5	nor-SNP	133680673
P5_5	nor-SNP	134884697
P5_5	nor-SNP	134884700
P5_5	nor-SNP	134884717
P5_5	nor-SNP	134884723
P5_5	nor-SNP	134884737
P5_5	nor-SNP	135061039
P5_5	nor-SNP	135061106
P5_5	nor-SNP	135061109
P5_5	nor-SNP	135061111
P5_5	nor-SNP	135061112
P5_5	nor-SNP	135061124
P5_5	nor-SNP	135812836
P5_5	nor-SNP	135817150
P5_5	nor-SNP	135821099
P5_5	nor-SNP	135821135
P5_5	nor-SNP	136422988
P5_5	nor-SNP	136423009
P5_5	nor-SNP	136587919
P5_5	nor-SNP	136587934
P5_5	nor-SNP	136588019
P5_5	nor-SNP	136983275
P5_5	nor-SNP	136983319
P5_5	nor-SNP	137741987
P5_5	nor-SNP	137741989
P5_5	nor-SNP	137741994
P5_5	nor-SNP	137742013
P5_5	nor-SNP	137742041
P5_5	nor-SNP	138756369
P5_5	nor-SNP	138756428
P5_5	nor-SNP	139006339
P5_5	nor-SNP	139006378
P5_5	nor-SNP	139565064
P5_5	nor-SNP	139565098
P5_5	nor-SNP	139565119

P5_5	nor-SNP	139565134
P5_5	nor-SNP	144264666
P5_5	nor-SNP	144264667
P5_5	nor-SNP	144895159
P5_5	nor-SNP	144895164
P5_5	nor-SNP	144895168
P5_5	nor-SNP	144895169
P5_5	nor-SNP	144895170
P5_5	nor-SNP	144895179
P5_5	nor-SNP	145074272
P5_5	nor-SNP	145074283
P5_5	nor-SNP	145074284
P5_5	nor-SNP	145074289
P5_5	nor-SNP	145074342
P5_5	nor-SNP	145076302
P5_5	nor-SNP	145076355
P5_5	nor-SNP	145076356
P5_5	nor-SNP	145076376
P5_5	nor-SNP	145619365
P5_5	nor-SNP	145619377
P5_5	nor-SNP	145619386
P5_5	nor-SNP	145619405
P5_5	nor-SNP	145619442
P5_5	nor-SNP	146271231
P5_5	nor-SNP	146271235
P5_5	nor-SNP	146271266
P5_5	nor-SNP	146271269
P5_5	nor-SNP	146271303
P5_5	nor-SNP	146271304
P5_5	nor-SNP	146280626
P5_5	nor-SNP	146280644
P5_5	nor-SNP	146295067
P5_5	nor-SNP	146295085
P5_5	nor-SNP	146307375
P5_5	nor-SNP	146307411
P5_5	nor-SNP	146307414
P5_5	nor-SNP	146307449
P5_5	nor-SNP	146312254
P5_5	nor-SNP	146318448
P5_5	nor-SNP	146318507
P5_5	nor-SNP	146340278
P5_5	nor-SNP	146340304
P5_5	nor-SNP	146340339
P5_5	nor-SNP	146340360
P5_5	nor-SNP	146341217
P5_5	nor-SNP	146342052
P5_5	nor-SNP	146342071

P5_5	nor-SNP	146342080
P5_5	nor-SNP	146353894
P5_5	nor-SNP	146360765
P5_5	nor-SNP	146360778
P5_5	nor-SNP	146360779
P5_5	nor-SNP	146360813
P5_5	nor-SNP	146360826
P5_5	nor-SNP	146360845
P5_5	nor-SNP	146366167
P5_5	nor-SNP	148265864
P5_5	nor-SNP	148808487
P5_5	nor-SNP	148808575
P5_5	nor-SNP	148810267
P5_5	nor-SNP	149112399
P5_5	nor-SNP	149396242
P5_5	nor-SNP	149396272
P5_5	nor-SNP	149396273
P5_5	nor-SNP	149396288
P5_5	nor-SNP	150935522
P5_5	nor-SNP	150935577
P5_5	nor-SNP	150935583
P5_5	nor-SNP	151127092
P5_5	nor-SNP	151127105
P5_5	nor-SNP	151128173
P5_5	nor-SNP	151560699
P5_5	nor-SNP	151560719
P5_5	nor-SNP	151562938
P5_5	nor-SNP	153410482
P5_5	nor-SNP	153410520
P5_5	nor-SNP	153410531
P5_5	nor-SNP	153996899
P5_5	nor-SNP	153996903
P5_5	nor-SNP	153996905
P5_5	nor-SNP	154166183
P5_5	nor-SNP	154209024
P5_5	nor-SNP	155174524
P5_5	nor-SNP	155174534
P5_5	nor-SNP	155174551
P5_5	nor-SNP	157367104
P5_5	nor-SNP	159912436
P5_5	nor-SNP	160122421
P5_5	nor-SNP	160122434
P5_5	nor-SNP	163889284
P5_5	nor-SNP	163889327
P5_5	nor-SNP	163889342
P5_5	nor-SNP	167967904
P5_5	nor-SNP	167967953

P5_5	nor-SNP	167967958
P5_5	nor-SNP	167967963
P5_5	nor-SNP	168195174
P5_5	nor-SNP	170120566
P5_5	nor-SNP	170120575
P5_5	nor-SNP	170120594
P5_5	nor-SNP	170813684
P5_5	nor-SNP	171070888
P5_5	nor-SNP	171070937
P5_5	nor-SNP	172113756
P5_5	nor-SNP	176998548
P5_5	nor-SNP	177015045
P5_5	nor-SNP	177465775
P5_5	nor-SNP	178120681
P5_5	nor-SNP	179225301
P5_5	nor-SNP	179225317
P5_5	nor-SNP	179225324
P5_5	nor-SNP	179442344
P5_5	nor-SNP	180407488
P5_5	nor-SNP	180407505
P5_5	nor-SNP	180407512
P5_5	nor-SNP	185485646
P5_5	nor-SNP	185772247
P5_5	nor-SNP	186504477
P5_5	nor-SNP	186504499
P5_5	nor-SNP	186504532
P5_5	nor-SNP	186504537
P5_5	nor-SNP	188406598
P5_5	nor-SNP	188406636
P5_5	nor-SNP	189842822
P5_5	nor-SNP	189842826
P5_5	nor-SNP	189842885
P5_5	nor-SNP	194855248
P5_5	nor-SNP	194855289
P5_5	nor-SNP	198828005
P5_5	nor-SNP	198828096
P5_5	nor-SNP	201777741
P5_5	nor-SNP	205417438
P5_5	nor-SNP	205417483
P5_5	nor-SNP	207647981
P5_5	nor-SNP	207648001
P5_5	nor-SNP	207648009
P5_5	nor-SNP	207974738
P5_5	nor-SNP	207974741
P5_5	nor-SNP	207974756
P5_5	nor-SNP	209605507
P5_5	nor-SNP	209605539

P5_5	nor-SNP	209605546
P5_5	nor-SNP	209796810
P5_5	nor-SNP	219267370
P5_5	nor-SNP	219267371
P5_5	nor-SNP	219267402
P5_5	nor-SNP	219267407
P5_5	nor-SNP	219267433
P5_5	nor-SNP	219267443
P5_5	nor-SNP	219923411
P5_5	nor-SNP	219923418
P5_5	nor-SNP	219923421
P5_5	nor-SNP	220291206
P5_5	nor-SNP	220291292
P5_5	nor-SNP	220291302
P5_5	nor-SNP	220291502
P5_5	nor-SNP	220291527
P5_5	nor-SNP	220373922
P5_5	nor-SNP	220373933
P5_5	nor-SNP	220373934
P5_5	nor-SNP	220373939
P5_5	nor-SNP	220373943
P5_5	nor-SNP	220413807
P5_5	nor-SNP	220413812
P5_5	nor-SNP	220413813
P5_5	nor-SNP	236016301
P5_5	nor-SNP	236016316
P5_5	nor-SNP	236016347
P5_5	nor-SNP	240227178
P5_5	nor-SNP	240273490
P5_5	nor-SNP	241395420
P5_5	nor-SNP	241395500
P5_5	nor-SNP	241395503
P5_5	nor-SNP	242417357
P5_5	nor-SNP	249120578
P5_5	nor-SNP	249120584
P5_5	nor-SNP	249120591
P5_5	nor-SNP	249120610
P5_5	nor-SNP	249120631
P5_5	nor-SNP	249120640
P5_5	iso-pre-miRNA>hsa-mir-1237	
P5_5	iso-pre-miRNA>hsa-mir-7-3	
P5_5	iso-pre-miRNA>hsa-mir-769	
P5_5	iso-pre-miRNA>hsa-mir-671	
P5_5	iso-pre-miRNA>hsa-mir-766	
P5_5	iso-pre-miRNA>hsa-mir-492	
P5_5	iso-pre-miRNA>hsa-mir-767	
P5_5	iso-pre-miRNA>hsa-mir-1178	

P5_5 iso-pre-miRNA>hsa-mir-1250
P5_5 iso-pre-miRNA>hsa-mir-7-1
P5_5 iso-pre-miRNA>hsa-mir-759
P5_5 iso-pre-miRNA>hsa-mir-659
P5_5 iso-pre-miRNA>hsa-mir-98
P5_5 iso-pre-miRNA>hsa-mir-2115
P5_5 iso-pre-miRNA>hsa-mir-670
P5_5 iso-pre-miRNA>hsa-mir-770
P5_5 iso-pre-miRNA>hsa-mir-92b
P5_5 iso-pre-miRNA>hsa-mir-1304
P5_5 iso-pre-miRNA>hsa-mir-3620
P5_5 iso-pre-miRNA>hsa-mir-802
P5_5 iso-pre-miRNA>hsa-mir-1254-1
P5_5 iso-pre-miRNA>hsa-mir-1275
P5_5 iso-pre-miRNA>hsa-mir-2277
P5_5 iso-pre-miRNA>hsa-mir-660
P5_5 iso-pre-miRNA>hsa-mir-9-1
P5_5 iso-pre-miRNA>hsa-mir-9-2
P5_5 iso-pre-miRNA>hsa-mir-9-3
P5_5 iso-pre-miRNA>hsa-mir-194-2
P5_5 iso-pre-miRNA>hsa-mir-663a
P5_5 iso-pre-miRNA>hsa-mir-758
P5_5 iso-pre-miRNA>hsa-mir-934
P5_5 iso-pre-miRNA>hsa-mir-936
P5_5 iso-pre-miRNA>hsa-mir-938
P5_5 iso-pre-miRNA>hsa-mir-939
P5_5 iso-pre-miRNA>hsa-mir-95
P5_5 iso-pre-miRNA>hsa-mir-125a
P5_5 iso-pre-miRNA>hsa-mir-128-1
P5_5 iso-pre-miRNA>hsa-mir-203b
P5_5 iso-pre-miRNA>hsa-mir-518e
P5_5 iso-pre-miRNA>hsa-mir-3916
P5_5 iso-pre-miRNA>hsa-mir-412
P5_5 iso-pre-miRNA>hsa-mir-1283-1
P5_5 iso-pre-miRNA>hsa-mir-937
P5_5 iso-pre-miRNA>hsa-mir-1181
P5_5 iso-pre-miRNA>hsa-mir-2116
P5_5 iso-pre-miRNA>hsa-mir-1279
P5_5 iso-pre-miRNA>hsa-mir-1199
P5_5 iso-pre-miRNA>hsa-mir-378d-2
P5_5 iso-pre-miRNA>hsa-mir-1914
P5_5 iso-pre-miRNA>hsa-mir-942
P5_5 iso-pre-miRNA>hsa-mir-99a
P5_5 iso-pre-miRNA>hsa-mir-1276
P5_5 iso-pre-miRNA>hsa-mir-3158-2
P5_5 iso-pre-miRNA>hsa-mir-1260a
P5_5 iso-pre-miRNA>hsa-mir-1908

P5_5 iso-pre-miRNA>hsa-mir-664a
P5_5 iso-pre-miRNA>hsa-mir-708
P5_5 iso-pre-miRNA>hsa-mir-744
P5_5 iso-pre-miRNA>hsa-mir-873
P5_5 iso-pre-miRNA>hsa-mir-876
P5_5 iso-pre-miRNA>hsa-mir-885
P5_5 iso-pre-miRNA>hsa-mir-888
P5_5 iso-pre-miRNA>hsa-mir-890
P5_5 iso-pre-miRNA>hsa-mir-891a
P5_5 iso-pre-miRNA>hsa-mir-891b
P5_5 iso-pre-miRNA>hsa-mir-892c
P5_5 iso-pre-miRNA>hsa-mir-92a-1
P5_5 iso-pre-miRNA>hsa-mir-93
P5_5 iso-pre-miRNA>hsa-mir-675
P5_5 iso-pre-miRNA>hsa-mir-874
P5_5 iso-pre-miRNA>hsa-mir-887
P5_5 iso-pre-miRNA>hsa-mir-4302
P5_5 iso-pre-miRNA>hsa-mir-433
P5_5 iso-pre-miRNA>hsa-mir-1267
P5_5 iso-pre-miRNA>hsa-mir-154
P5_5 iso-pre-miRNA>hsa-mir-3144
P5_5 iso-pre-miRNA>hsa-mir-3175
P5_5 iso-pre-miRNA>hsa-mir-4289
P5_5 iso-pre-miRNA>hsa-mir-516a-2
P5_5 iso-pre-miRNA>hsa-mir-1269b
P5_5 iso-pre-miRNA>hsa-mir-92a-2
P5_5 iso-pre-miRNA>hsa-mir-96
P5_5 iso-pre-miRNA>hsa-mir-3196
P5_5 iso-pre-miRNA>hsa-mir-523
P5_5 iso-pre-miRNA>hsa-mir-3125
P5_5 iso-pre-miRNA>hsa-mir-2110
P5_5 iso-pre-miRNA>hsa-mir-676
P5_5 iso-pre-miRNA>hsa-mir-4317
P5_5 iso-pre-miRNA>hsa-mir-4309
P5_5 iso-pre-miRNA>hsa-mir-3141
P5_5 iso-pre-miRNA>hsa-mir-23b
P5_5 iso-pre-miRNA>hsa-mir-3177
P5_5 iso-pre-miRNA>hsa-mir-342
P5_5 iso-pre-miRNA>hsa-mir-668
P5_5 iso-pre-miRNA>hsa-mir-761
P5_5 iso-pre-miRNA>hsa-mir-3199-1
P5_5 iso-pre-miRNA>hsa-mir-146a
P5_5 iso-pre-miRNA>hsa-mir-4260
P5_5 iso-pre-miRNA>hsa-mir-4270
P5_5 iso-pre-miRNA>hsa-mir-1255b-2
P5_5 iso-pre-miRNA>hsa-mir-3129
P5_5 iso-pre-miRNA>hsa-mir-1273h

P5_5 iso-pre-miRNA>hsa-mir-4258
P5_5 iso-pre-miRNA>hsa-mir-1207
P5_5 iso-pre-miRNA>hsa-mir-1231
P5_5 iso-pre-miRNA>hsa-mir-1252
P5_5 iso-pre-miRNA>hsa-mir-3170
P5_5 iso-pre-miRNA>hsa-mir-1205
P5_5 iso-pre-miRNA>hsa-mir-4301
P5_5 iso-pre-miRNA>hsa-mir-924
P5_5 iso-pre-miRNA>hsa-mir-3683
P5_5 iso-pre-miRNA>hsa-mir-142
P5_5 iso-pre-miRNA>hsa-mir-3124
P5_5 iso-pre-miRNA>hsa-mir-484
P5_5 iso-pre-miRNA>hsa-mir-1255b-1
P5_5 iso-pre-miRNA>hsa-mir-4326
P5_5 iso-pre-miRNA>hsa-mir-200a
P5_5 iso-pre-miRNA>hsa-mir-3178
P5_5 iso-pre-miRNA>hsa-mir-1202
P5_5 iso-pre-miRNA>hsa-mir-126
P5_5 iso-pre-miRNA>hsa-mir-504
P5_5 iso-pre-miRNA>hsa-mir-664b
P5_5 iso-pre-miRNA>hsa-mir-877
P5_5 iso-pre-miRNA>hsa-mir-1225
P5_5 iso-pre-miRNA>hsa-mir-3195
P5_5 iso-pre-miRNA>hsa-mir-378a
P5_5 iso-pre-miRNA>hsa-mir-3143
P5_5 iso-pre-miRNA>hsa-mir-1587
P5_5 iso-pre-miRNA>hsa-mir-3927
P5_5 iso-pre-miRNA>hsa-mir-1282
P5_5 iso-pre-miRNA>hsa-mir-1292
P5_5 iso-pre-miRNA>hsa-mir-188
P5_5 iso-pre-miRNA>hsa-mir-197
P5_5 iso-pre-miRNA>hsa-mir-1260b
P5_5 iso-pre-miRNA>hsa-mir-1224
P5_5 iso-pre-miRNA>hsa-mir-1203
P5_5 iso-pre-miRNA>hsa-mir-339
P5_5 iso-pre-miRNA>hsa-mir-4298
P5_5 iso-pre-miRNA>hsa-mir-548ak
P5_5 iso-pre-miRNA>hsa-mir-1233-1
P5_5 iso-pre-miRNA>hsa-mir-1233-2
P5_5 iso-pre-miRNA>hsa-mir-196a-2
P5_5 iso-pre-miRNA>hsa-mir-216a
P5_5 iso-SNP 64136167
P5_5 iso-SNP 64136145
P5_5 iso-SNP 64136130
P5_5 iso-SNP 64136103
P5_5 iso-SNP 64136160
P5_5 iso-SNP 64136170

P5_5	iso-SNP	4770697
P5_5	iso-SNP	4770709
P5_5	iso-SNP	46522190
P5_5	iso-SNP	46522201
P5_5	iso-SNP	150935522
P5_5	iso-SNP	150935577
P5_5	iso-SNP	118780790
P5_5	iso-SNP	118780738
P5_5	iso-SNP	95228209
P5_5	iso-SNP	95228179
P5_5	iso-SNP	151561912
P5_5	iso-SNP	120151501
P5_5	iso-SNP	79107061
P5_5	iso-SNP	79107049
P5_5	iso-SNP	86584720
P5_5	iso-SNP	86584707
P5_5	iso-SNP	53384209
P5_5	iso-SNP	53384256
P5_5	iso-SNP	79107068
P5_5	iso-SNP	120151527
P5_5	iso-SNP	79107084
P5_5	iso-SNP	38243770
P5_5	iso-SNP	38243743
P5_5	iso-SNP	53583294
P5_5	iso-SNP	48357921
P5_5	iso-SNP	43581287
P5_5	iso-SNP	101318740
P5_5	iso-SNP	101318783
P5_5	iso-SNP	155165044
P5_5	iso-SNP	93466919
P5_5	iso-SNP	93466912
P5_5	iso-SNP	93466910
P5_5	iso-SNP	93466913
P5_5	iso-SNP	93466909
P5_5	iso-SNP	228284991
P5_5	iso-SNP	93466866
P5_5	iso-SNP	37093013
P5_5	iso-SNP	37093078
P5_5	iso-SNP	70519078
P5_5	iso-SNP	33967814
P5_5	iso-SNP	33967800
P5_5	iso-SNP	92956416
P5_5	iso-SNP	92956420
P5_5	iso-SNP	49777907
P5_5	iso-SNP	156390147
P5_5	iso-SNP	87962747
P5_5	iso-SNP	89911256

P5_5	iso-SNP	64658904
P5_5	iso-SNP	64658836
P5_5	iso-SNP	26188908
P5_5	iso-SNP	26188906
P5_5	iso-SNP	101492444
P5_5	iso-SNP	135633045
P5_5	iso-SNP	105807928
P5_5	iso-SNP	105807912
P5_5	iso-SNP	29891262
P5_5	iso-SNP	29891260
P5_5	iso-SNP	145619442
P5_5	iso-SNP	145619405
P5_5	iso-SNP	8007104
P5_5	iso-SNP	8007066
P5_5	iso-SNP	8007067
P5_5	iso-SNP	52196528
P5_5	iso-SNP	136423009
P5_5	iso-SNP	135633077
P5_5	iso-SNP	104583838
P5_5	iso-SNP	29891245
P5_5	iso-SNP	54233093
P5_5	iso-SNP	54233092
P5_5	iso-SNP	105807909
P5_5	iso-SNP	247365286
P5_5	iso-SNP	101531806
P5_5	iso-SNP	54191790
P5_5	iso-SNP	144895179
P5_5	iso-SNP	144895170
P5_5	iso-SNP	10514202
P5_5	iso-SNP	59463460
P5_5	iso-SNP	10514190
P5_5	iso-SNP	10514194
P5_5	iso-SNP	54191743
P5_5	iso-SNP	120151493
P5_5	iso-SNP	33967796
P5_5	iso-SNP	10514200
P5_5	iso-SNP	64658828
P5_5	iso-SNP	69666979
P5_5	iso-SNP	14184227
P5_5	iso-SNP	59463451
P5_5	iso-SNP	94928319
P5_5	iso-SNP	62572885
P5_5	iso-SNP	117637302
P5_5	iso-SNP	117637290
P5_5	iso-SNP	17911447
P5_5	iso-SNP	144895169
P5_5	iso-SNP	144895168

P5_5	iso-SNP	62572847
P5_5	iso-SNP	62572874
P5_5	iso-SNP	59463389
P5_5	iso-SNP	59463436
P5_5	iso-SNP	48357853
P5_5	iso-SNP	105807864
P5_5	iso-SNP	105807858
P5_5	iso-SNP	105807855
P5_5	iso-SNP	86313731
P5_5	iso-SNP	103361226
P5_5	iso-SNP	77732628
P5_5	iso-SNP	86313794
P5_5	iso-SNP	61582649
P5_5	iso-SNP	61582659
P5_5	iso-SNP	79107017
P5_5	iso-SNP	220373943
P5_5	iso-SNP	220373939
P5_5	iso-SNP	79113119
P5_5	iso-SNP	11985275
P5_5	iso-SNP	28888922
P5_5	iso-SNP	28863695
P5_5	iso-SNP	28863633
P5_5	iso-SNP	10436238
P5_5	iso-SNP	10436219
P5_5	iso-SNP	145076376
P5_5	iso-SNP	145076356
P5_5	iso-SNP	145075848
P5_5	iso-SNP	145075804
P5_5	iso-SNP	145109356
P5_5	iso-SNP	145109328
P5_5	iso-SNP	145082615
P5_5	iso-SNP	145074342
P5_5	iso-SNP	145074289
P5_5	iso-SNP	92003588
P5_5	iso-SNP	92003589
P5_5	iso-SNP	99691429
P5_5	iso-SNP	99691401
P5_5	iso-SNP	220373934
P5_5	iso-SNP	220373922
P5_5	iso-SNP	52196574
P5_5	iso-SNP	10514159
P5_5	iso-SNP	220373933
P5_5	iso-SNP	48357864
P5_5	iso-SNP	92956422
P5_5	iso-SNP	92956443
P5_5	iso-SNP	54233109
P5_5	iso-SNP	8007039

P5_5	iso-SNP	77732586
P5_5	iso-SNP	2018056
P5_5	iso-SNP	2018019
P5_5	iso-SNP	136983326
P5_5	iso-SNP	136983319
P5_5	iso-SNP	136983281
P5_5	iso-SNP	136983275
P5_5	iso-SNP	15935316
P5_5	iso-SNP	15935347
P5_5	iso-SNP	26026988
P5_5	iso-SNP	101348275
P5_5	iso-SNP	108183538
P5_5	iso-SNP	101526116
P5_5	iso-SNP	8007037
P5_5	iso-SNP	120336327
P5_5	iso-SNP	120336365
P5_5	iso-SNP	93447631
P5_5	iso-SNP	91360757
P5_5	iso-SNP	93447646
P5_5	iso-SNP	54264394
P5_5	iso-SNP	12820646
P5_5	iso-SNP	133303579
P5_5	iso-SNP	129414574
P5_5	iso-SNP	129414553
P5_5	iso-SNP	129414568
P5_5	iso-SNP	12820658
P5_5	iso-SNP	69666969
P5_5	iso-SNP	61870141
P5_5	iso-SNP	54201668
P5_5	iso-SNP	12877500
P5_5	iso-SNP	15935351
P5_5	iso-SNP	69666943
P5_5	iso-SNP	103361245
P5_5	iso-SNP	77732603
P5_5	iso-SNP	115933918
P5_5	iso-SNP	69242707
P5_5	iso-SNP	69242767
P5_5	iso-SNP	115933876
P5_5	iso-SNP	115933922
P5_5	iso-SNP	61870162
P5_5	iso-SNP	6374387
P5_5	iso-SNP	6374394
P5_5	iso-SNP	103006047
P5_5	iso-SNP	153975613
P5_5	iso-SNP	97847498
P5_5	iso-SNP	61870140
P5_5	iso-SNP	54233112

P5_5	iso-SNP	1785030
P5_5	iso-SNP	115933913
P5_5	iso-SNP	100576059
P5_5	iso-SNP	12820632
P5_5	iso-SNP	101521608
P5_5	iso-SNP	101521620
P5_5	iso-SNP	52302043
P5_5	iso-SNP	153975627
P5_5	iso-SNP	54233113
P5_5	iso-SNP	28316592
P5_5	iso-SNP	136422988
P5_5	iso-SNP	159912418
P5_5	iso-SNP	86313782
P5_5	iso-SNP	153975576
P5_5	iso-SNP	209796849
P5_5	iso-SNP	15537776
P5_5	iso-SNP	167967953
P5_5	iso-SNP	145074283
P5_5	iso-SNP	189997834
P5_5	iso-SNP	24214442
P5_5	iso-SNP	108183525
P5_5	iso-SNP	1785015
P5_5	iso-SNP	154948201
P5_5	iso-SNP	129061408
P5_5	iso-SNP	101526140
P5_5	iso-SNP	101526127
P5_5	iso-SNP	53384262
P5_5	iso-SNP	54201654
P5_5	iso-SNP	201777751
P5_5	iso-SNP	201777753
P5_5	iso-SNP	201777741
P5_5	iso-SNP	201777752
P5_5	iso-SNP	79813049
P5_5	iso-SNP	12877520
P5_5	iso-SNP	12877502
P5_5	iso-SNP	28316597
P5_5	iso-SNP	12877501
P5_5	iso-SNP	120336384
P5_5	iso-SNP	54201692
P5_5	iso-SNP	54201667
P5_5	iso-SNP	98860808
P5_5	iso-SNP	98860801
P5_5	iso-SNP	128972925
P5_5	iso-SNP	128972923
P5_5	iso-SNP	61870167
P5_5	iso-SNP	113320747
P5_5	iso-SNP	79813075

P5_5	iso-SNP	37202138
P5_5	iso-SNP	37202113
P5_5	iso-SNP	7106600
P5_5	iso-SNP	7106636
P5_5	iso-SNP	56408638
P5_5	iso-SNP	249120578
P5_5	iso-SNP	15737177
P5_5	iso-SNP	15737151
P5_5	iso-SNP	15935348
P5_5	iso-SNP	54233170
P5_5	iso-SNP	36428048
P5_5	iso-SNP	36428046
P5_5	iso-SNP	24214493
P5_5	iso-SNP	24214532
P5_5	iso-SNP	61918164
P5_5	iso-SNP	61582708
P5_5	iso-SNP	36428006
P5_5	iso-SNP	36428017
P5_5	iso-SNP	1103284
P5_5	iso-SNP	79813083
P5_5	iso-SNP	2581984
P5_5	iso-SNP	24214486
P5_5	iso-SNP	61582680
P5_5	iso-SNP	156267978
P5_5	iso-SNP	139565098
P5_5	iso-SNP	189997816
P5_5	iso-SNP	128972936
P5_5	iso-SNP	145074272
P5_5	iso-SNP	137749929
P5_5	iso-SNP	145074284
P5_5	iso-SNP	139565064
P5_5	iso-SNP	153996899
P5_5	iso-SNP	153996903
P5_5	iso-SNP	30552168
P5_5	iso-SNP	30552187
P5_5	iso-SNP	2140269
P5_5	iso-SNP	2140270
P5_5	iso-SNP	2140240
P5_5	iso-SNP	2140268
P5_5	iso-SNP	2140267
P5_5	iso-SNP	60639883
P5_5	iso-SNP	149112399
P5_5	iso-SNP	27115444
P5_5	iso-SNP	27115438
P5_5	iso-SNP	54264462
P5_5	iso-SNP	249120584
P5_5	iso-SNP	27115458

P5_5	iso-SNP	167967904
P5_5	iso-SNP	56408639
P5_5	iso-SNP	156267955
P5_5	iso-SNP	39696863
P5_5	iso-SNP	112273768
P5_5	iso-SNP	1785038
P5_5	iso-SNP	1785037
P5_5	iso-SNP	44085909
P5_5	iso-SNP	70519169
P5_5	iso-SNP	101531857
P5_5	iso-SNP	101531854
P5_5	iso-SNP	101531862
P5_5	iso-SNP	101531849
P5_5	iso-SNP	2633462
P5_5	iso-SNP	49768168
P5_5	iso-SNP	110141523
P5_5	iso-SNP	110141578
P5_5	iso-SNP	100576035
P5_5	iso-SNP	54264421
P5_5	iso-SNP	70519093
P5_5	iso-SNP	129061422
P5_5	iso-SNP	96074607
P5_5	iso-SNP	183959222
P5_5	iso-SNP	46233848
P5_5	iso-SNP	27115467
P5_5	iso-SNP	154948195
P5_5	iso-SNP	10514157
P5_5	iso-SNP	12172775
P5_5	iso-SNP	12172788
P5_5	iso-SNP	12172799
P5_5	iso-SNP	34674309
P5_5	iso-SNP	34820530
P5_5	iso-SNP	54385599
P5_5	iso-SNP	56216090
P5_5	iso-SNP	56216156
P5_5	iso-SNP	4770747
P5_5	iso-SNP	46522255
P5_5	iso-SNP	150935583
P5_5	iso-SNP	95228286
P5_5	iso-SNP	86584675
P5_5	iso-SNP	38243739
P5_5	iso-SNP	101318784
P5_5	iso-SNP	37093089
P5_5	iso-SNP	92956412
P5_5	iso-SNP	26188880
P5_5	iso-SNP	145619386
P5_5	iso-SNP	104583828

P5_5	iso-SNP	29891235
P5_5	iso-SNP	101531858
P5_5	iso-SNP	54191794
P5_5	iso-SNP	33967787
P5_5	iso-SNP	94928250
P5_5	iso-SNP	1988193
P5_5	iso-SNP	117637325
P5_5	iso-SNP	144895159
P5_5	iso-SNP	144895164
P5_5	iso-SNP	28863628
P5_5	iso-SNP	10436201
P5_5	iso-SNP	145076355
P5_5	iso-SNP	99691396
P5_5	iso-SNP	2018004
P5_5	iso-SNP	93447674
P5_5	iso-SNP	93447702
P5_5	iso-SNP	93447700
P5_5	iso-SNP	93447695
P5_5	iso-SNP	115933905
P5_5	iso-SNP	97847535
P5_5	iso-SNP	28316591
P5_5	iso-SNP	159912436
P5_5	iso-SNP	209796810
P5_5	iso-SNP	167967958
P5_5	iso-SNP	129061441
P5_5	iso-SNP	129061462
P5_5	iso-SNP	201777819
P5_5	iso-SNP	54201695
P5_5	iso-SNP	98860827
P5_5	iso-SNP	37202112
P5_5	iso-SNP	56408625
P5_5	iso-SNP	56408599
P5_5	iso-SNP	15737186
P5_5	iso-SNP	15737185
P5_5	iso-SNP	1103312
P5_5	iso-SNP	2581955
P5_5	iso-SNP	139565119
P5_5	iso-SNP	12877527
P5_5	iso-SNP	153996905
P5_5	iso-SNP	249120631
P5_5	iso-SNP	249120610
P5_5	iso-SNP	249120591
P5_5	iso-SNP	167967963
P5_5	iso-SNP	1785042
P5_5	iso-SNP	2633466
P5_5	iso-SNP	49768171
P5_5	iso-SNP	1785060

P5_5	iso-SNP	27115447
P5_5	iso-SNP	96074638
P5_5	iso-SNP	183959232
P5_5	iso-SNP	46233806
P5_5	iso-SNP	44085868
P5_5	iso-SNP	12172783
P5_5	iso-SNP	54385629
P5_5	iso-SNP	56216092
P5_5	iso-SNP	4770789
P5_5	iso-SNP	46522298
P5_5	iso-SNP	38243727
P5_5	iso-SNP	101318807
P5_5	iso-SNP	37093097
P5_5	iso-SNP	92956409
P5_5	iso-SNP	26188865
P5_5	iso-SNP	145619377
P5_5	iso-SNP	104583804
P5_5	iso-SNP	117637326
P5_5	iso-SNP	10436194
P5_5	iso-SNP	145076302
P5_5	iso-SNP	99691393
P5_5	iso-SNP	2018002
P5_5	iso-SNP	115933890
P5_5	iso-SNP	97847569
P5_5	iso-SNP	28316513
P5_5	iso-SNP	98860840
P5_5	iso-SNP	37202092
P5_5	iso-SNP	15737217
P5_5	iso-SNP	1103328
P5_5	iso-SNP	2581952
P5_5	iso-SNP	139565134
P5_5	iso-SNP	249120640
P5_5	iso-SNP	12877525
P5_5	iso-SNP	2633478
P5_5	iso-SNP	15737218
P5_5	iso-SNP	2140204
P5_5	iso-SNP	96074649
P5_5	iso-SNP	26188825
P5_5	iso-SNP	145619365
P5_5	iso-SNP	104583759
P5_5	iso-SNP	10436187
P5_5	iso-SNP	54201703
P5_5	iso-SNP	97847573
P5_5	iso-SNP	2633480
P5_5	iso-SNP	10436180
P5_3	nor-pre-miRNA>hsa-let-7a-2	
P5_3	nor-pre-miRNA>hsa-let-7e	

P5_3 nor-pre-miRNA>hsa-mir-1180
P5_3 nor-pre-miRNA>hsa-mir-1181
P5_3 nor-pre-miRNA>hsa-mir-1205
P5_3 nor-pre-miRNA>hsa-mir-1208
P5_3 nor-pre-miRNA>hsa-mir-122
P5_3 nor-pre-miRNA>hsa-mir-1225
P5_3 nor-pre-miRNA>hsa-mir-1231
P5_3 nor-pre-miRNA>hsa-mir-1236
P5_3 nor-pre-miRNA>hsa-mir-1238
P5_3 nor-pre-miRNA>hsa-mir-1250
P5_3 nor-pre-miRNA>hsa-mir-1254-1
P5_3 nor-pre-miRNA>hsa-mir-1255b-1
P5_3 nor-pre-miRNA>hsa-mir-1255b-2
P5_3 nor-pre-miRNA>hsa-mir-125b-2
P5_3 nor-pre-miRNA>hsa-mir-1260a
P5_3 nor-pre-miRNA>hsa-mir-1260b
P5_3 nor-pre-miRNA>hsa-mir-1263
P5_3 nor-pre-miRNA>hsa-mir-1265
P5_3 nor-pre-miRNA>hsa-mir-1267
P5_3 nor-pre-miRNA>hsa-mir-1268a
P5_3 nor-pre-miRNA>hsa-mir-1268b
P5_3 nor-pre-miRNA>hsa-mir-1273c
P5_3 nor-pre-miRNA>hsa-mir-1273d
P5_3 nor-pre-miRNA>hsa-mir-1275
P5_3 nor-pre-miRNA>hsa-mir-1277
P5_3 nor-pre-miRNA>hsa-mir-1284
P5_3 nor-pre-miRNA>hsa-mir-1291
P5_3 nor-pre-miRNA>hsa-mir-1292
P5_3 nor-pre-miRNA>hsa-mir-1293
P5_3 nor-pre-miRNA>hsa-mir-1301
P5_3 nor-pre-miRNA>hsa-mir-1304
P5_3 nor-pre-miRNA>hsa-mir-1323
P5_3 nor-pre-miRNA>hsa-mir-133a-1
P5_3 nor-pre-miRNA>hsa-mir-133a-2
P5_3 nor-pre-miRNA>hsa-mir-134
P5_3 nor-pre-miRNA>hsa-mir-1343
P5_3 nor-pre-miRNA>hsa-mir-135a-2
P5_3 nor-pre-miRNA>hsa-mir-135b
P5_3 nor-pre-miRNA>hsa-mir-136
P5_3 nor-pre-miRNA>hsa-mir-145
P5_3 nor-pre-miRNA>hsa-mir-1468
P5_3 nor-pre-miRNA>hsa-mir-1469
P5_3 nor-pre-miRNA>hsa-mir-146b
P5_3 nor-pre-miRNA>hsa-mir-152
P5_3 nor-pre-miRNA>hsa-mir-1537
P5_3 nor-pre-miRNA>hsa-mir-154
P5_3 nor-pre-miRNA>hsa-mir-155

P5_3 nor-pre-miRNA>hsa-mir-16-1
P5_3 nor-pre-miRNA>hsa-mir-181b-2
P5_3 nor-pre-miRNA>hsa-mir-181d
P5_3 nor-pre-miRNA>hsa-mir-182
P5_3 nor-pre-miRNA>hsa-mir-183
P5_3 nor-pre-miRNA>hsa-mir-188
P5_3 nor-pre-miRNA>hsa-mir-1908
P5_3 nor-pre-miRNA>hsa-mir-1909
P5_3 nor-pre-miRNA>hsa-mir-190a
P5_3 nor-pre-miRNA>hsa-mir-1910
P5_3 nor-pre-miRNA>hsa-mir-1911
P5_3 nor-pre-miRNA>hsa-mir-1914
P5_3 nor-pre-miRNA>hsa-mir-1915
P5_3 nor-pre-miRNA>hsa-mir-192
P5_3 nor-pre-miRNA>hsa-mir-193a
P5_3 nor-pre-miRNA>hsa-mir-196a-1
P5_3 nor-pre-miRNA>hsa-mir-196b
P5_3 nor-pre-miRNA>hsa-mir-200a
P5_3 nor-pre-miRNA>hsa-mir-202
P5_3 nor-pre-miRNA>hsa-mir-203b
P5_3 nor-pre-miRNA>hsa-mir-205
P5_3 nor-pre-miRNA>hsa-mir-21
P5_3 nor-pre-miRNA>hsa-mir-211
P5_3 nor-pre-miRNA>hsa-mir-2110
P5_3 nor-pre-miRNA>hsa-mir-2114
P5_3 nor-pre-miRNA>hsa-mir-2115
P5_3 nor-pre-miRNA>hsa-mir-215
P5_3 nor-pre-miRNA>hsa-mir-216a
P5_3 nor-pre-miRNA>hsa-mir-216b
P5_3 nor-pre-miRNA>hsa-mir-219a-1
P5_3 nor-pre-miRNA>hsa-mir-221
P5_3 nor-pre-miRNA>hsa-mir-224
P5_3 nor-pre-miRNA>hsa-mir-2277
P5_3 nor-pre-miRNA>hsa-mir-23a
P5_3 nor-pre-miRNA>hsa-mir-23b
P5_3 nor-pre-miRNA>hsa-mir-24-1
P5_3 nor-pre-miRNA>hsa-mir-24-2
P5_3 nor-pre-miRNA>hsa-mir-2467
P5_3 nor-pre-miRNA>hsa-mir-26a-1
P5_3 nor-pre-miRNA>hsa-mir-26b
P5_3 nor-pre-miRNA>hsa-mir-28
P5_3 nor-pre-miRNA>hsa-mir-2909
P5_3 nor-pre-miRNA>hsa-mir-296
P5_3 nor-pre-miRNA>hsa-mir-297
P5_3 nor-pre-miRNA>hsa-mir-299
P5_3 nor-pre-miRNA>hsa-mir-302a
P5_3 nor-pre-miRNA>hsa-mir-302e

P5_3 nor-pre-miRNA>hsa-mir-3074
P5_3 nor-pre-miRNA>hsa-mir-30a
P5_3 nor-pre-miRNA>hsa-mir-30b
P5_3 nor-pre-miRNA>hsa-mir-30c-2
P5_3 nor-pre-miRNA>hsa-mir-30d
P5_3 nor-pre-miRNA>hsa-mir-3119-2
P5_3 nor-pre-miRNA>hsa-mir-3121
P5_3 nor-pre-miRNA>hsa-mir-3125
P5_3 nor-pre-miRNA>hsa-mir-3126
P5_3 nor-pre-miRNA>hsa-mir-3127
P5_3 nor-pre-miRNA>hsa-mir-3130-1
P5_3 nor-pre-miRNA>hsa-mir-3130-2
P5_3 nor-pre-miRNA>hsa-mir-3133
P5_3 nor-pre-miRNA>hsa-mir-3135a
P5_3 nor-pre-miRNA>hsa-mir-3137
P5_3 nor-pre-miRNA>hsa-mir-3140
P5_3 nor-pre-miRNA>hsa-mir-3147
P5_3 nor-pre-miRNA>hsa-mir-3148
P5_3 nor-pre-miRNA>hsa-mir-3157
P5_3 nor-pre-miRNA>hsa-mir-3158-1
P5_3 nor-pre-miRNA>hsa-mir-3158-2
P5_3 nor-pre-miRNA>hsa-mir-3159
P5_3 nor-pre-miRNA>hsa-mir-3161
P5_3 nor-pre-miRNA>hsa-mir-3162
P5_3 nor-pre-miRNA>hsa-mir-3163
P5_3 nor-pre-miRNA>hsa-mir-3164
P5_3 nor-pre-miRNA>hsa-mir-3169
P5_3 nor-pre-miRNA>hsa-mir-3170
P5_3 nor-pre-miRNA>hsa-mir-3173
P5_3 nor-pre-miRNA>hsa-mir-3178
P5_3 nor-pre-miRNA>hsa-mir-3184
P5_3 nor-pre-miRNA>hsa-mir-3185
P5_3 nor-pre-miRNA>hsa-mir-3196
P5_3 nor-pre-miRNA>hsa-mir-324
P5_3 nor-pre-miRNA>hsa-mir-325
P5_3 nor-pre-miRNA>hsa-mir-329-2
P5_3 nor-pre-miRNA>hsa-mir-330
P5_3 nor-pre-miRNA>hsa-mir-335
P5_3 nor-pre-miRNA>hsa-mir-337
P5_3 nor-pre-miRNA>hsa-mir-338
P5_3 nor-pre-miRNA>hsa-mir-339
P5_3 nor-pre-miRNA>hsa-mir-345
P5_3 nor-pre-miRNA>hsa-mir-346
P5_3 nor-pre-miRNA>hsa-mir-34a
P5_3 nor-pre-miRNA>hsa-mir-361
P5_3 nor-pre-miRNA>hsa-mir-3619
P5_3 nor-pre-miRNA>hsa-mir-362

P5_3 nor-pre-miRNA>hsa-mir-3664
P5_3 nor-pre-miRNA>hsa-mir-367
P5_3 nor-pre-miRNA>hsa-mir-3679
P5_3 nor-pre-miRNA>hsa-mir-3689b
P5_3 nor-pre-miRNA>hsa-mir-371a
P5_3 nor-pre-miRNA>hsa-mir-377
P5_3 nor-pre-miRNA>hsa-mir-380
P5_3 nor-pre-miRNA>hsa-mir-3911
P5_3 nor-pre-miRNA>hsa-mir-3927
P5_3 nor-pre-miRNA>hsa-mir-3928
P5_3 nor-pre-miRNA>hsa-mir-409
P5_3 nor-pre-miRNA>hsa-mir-412
P5_3 nor-pre-miRNA>hsa-mir-423
P5_3 nor-pre-miRNA>hsa-mir-424
P5_3 nor-pre-miRNA>hsa-mir-4260
P5_3 nor-pre-miRNA>hsa-mir-4270
P5_3 nor-pre-miRNA>hsa-mir-4284
P5_3 nor-pre-miRNA>hsa-mir-4302
P5_3 nor-pre-miRNA>hsa-mir-4317
P5_3 nor-pre-miRNA>hsa-mir-4326
P5_3 nor-pre-miRNA>hsa-mir-449a
P5_3 nor-pre-miRNA>hsa-mir-450a-1
P5_3 nor-pre-miRNA>hsa-mir-450a-2
P5_3 nor-pre-miRNA>hsa-mir-450b
P5_3 nor-pre-miRNA>hsa-mir-455
P5_3 nor-pre-miRNA>hsa-mir-484
P5_3 nor-pre-miRNA>hsa-mir-486-2
P5_3 nor-pre-miRNA>hsa-mir-488
P5_3 nor-pre-miRNA>hsa-mir-493
P5_3 nor-pre-miRNA>hsa-mir-495
P5_3 nor-pre-miRNA>hsa-mir-499a
P5_3 nor-pre-miRNA>hsa-mir-499b
P5_3 nor-pre-miRNA>hsa-mir-505
P5_3 nor-pre-miRNA>hsa-mir-506
P5_3 nor-pre-miRNA>hsa-mir-510
P5_3 nor-pre-miRNA>hsa-mir-513b
P5_3 nor-pre-miRNA>hsa-mir-513c
P5_3 nor-pre-miRNA>hsa-mir-514a-1
P5_3 nor-pre-miRNA>hsa-mir-514a-3
P5_3 nor-pre-miRNA>hsa-mir-516b-2
P5_3 nor-pre-miRNA>hsa-mir-517b
P5_3 nor-pre-miRNA>hsa-mir-519d
P5_3 nor-pre-miRNA>hsa-mir-520g
P5_3 nor-pre-miRNA>hsa-mir-525
P5_3 nor-pre-miRNA>hsa-mir-526b
P5_3 nor-pre-miRNA>hsa-mir-532
P5_3 nor-pre-miRNA>hsa-mir-541

P5_3	nor-pre-miRNA>hsa-mir-542	
P5_3	nor-pre-miRNA>hsa-mir-545	
P5_3	nor-pre-miRNA>hsa-mir-548ak	
P5_3	nor-pre-miRNA>hsa-mir-548am	
P5_3	nor-pre-miRNA>hsa-mir-548ap	
P5_3	nor-pre-miRNA>hsa-mir-548aq	
P5_3	nor-pre-miRNA>hsa-mir-548as	
P5_3	nor-pre-miRNA>hsa-mir-548at	
P5_3	nor-pre-miRNA>hsa-mir-548aw	
P5_3	nor-pre-miRNA>hsa-mir-548ba	
P5_3	nor-pre-miRNA>hsa-mir-548f-1	
P5_3	nor-pre-miRNA>hsa-mir-548h-3	
P5_3	nor-pre-miRNA>hsa-mir-548h-5	
P5_3	nor-pre-miRNA>hsa-mir-663a	
P5_3	nor-pre-miRNA>hsa-mir-664a	
P5_3	nor-pre-miRNA>hsa-mir-664b	
P5_3	nor-pre-miRNA>hsa-mir-671	
P5_3	nor-pre-miRNA>hsa-mir-675	
P5_3	nor-pre-miRNA>hsa-mir-769	
P5_3	nor-pre-miRNA>hsa-mir-802	
P5_3	nor-pre-miRNA>hsa-mir-874	
P5_3	nor-pre-miRNA>hsa-mir-885	
P5_3	nor-pre-miRNA>hsa-mir-924	
P5_3	nor-pre-miRNA>hsa-mir-936	
P5_3	nor-pre-miRNA>hsa-mir-938	
P5_3	nor-pre-miRNA>hsa-mir-939	
P5_3	nor-pre-miRNA>hsa-mir-942	
P5_3	nor-pre-miRNA>hsa-mir-96	
P5_3	nor-SNP	7256001
P5_3	nor-SNP	7256057
P5_3	nor-SNP	31924631
P5_3	nor-SNP	36428017
P5_3	nor-SNP	36428006
P5_3	nor-SNP	101491407
P5_3	nor-SNP	19247825
P5_3	nor-SNP	2581984
P5_3	nor-SNP	2581955
P5_3	nor-SNP	2581952
P5_3	nor-SNP	2581992
P5_3	nor-SNP	113569054
P5_3	nor-SNP	113569088
P5_3	nor-SNP	46709899
P5_3	nor-SNP	46709875
P5_3	nor-SNP	46709859
P5_3	nor-SNP	97848319
P5_3	nor-SNP	97848343
P5_3	nor-SNP	101490145

P5_3	nor-SNP	101490178
P5_3	nor-SNP	209796849
P5_3	nor-SNP	209796810
P5_3	nor-SNP	104196269
P5_3	nor-SNP	194855289
P5_3	nor-SNP	194855248
P5_3	nor-SNP	117520360
P5_3	nor-SNP	50627942
P5_3	nor-SNP	50627936
P5_3	nor-SNP	50627935
P5_3	nor-SNP	69330818
P5_3	nor-SNP	69330824
P5_3	nor-SNP	69330825
P5_3	nor-SNP	69330871
P5_3	nor-SNP	69330886
P5_3	nor-SNP	242417357
P5_3	nor-SNP	29814842
P5_3	nor-SNP	29814813
P5_3	nor-SNP	29814796
P5_3	nor-SNP	66701974
P5_3	nor-SNP	66701926
P5_3	nor-SNP	66701915
P5_3	nor-SNP	108183538
P5_3	nor-SNP	108183525
P5_3	nor-SNP	54175287
P5_3	nor-SNP	48118347
P5_3	nor-SNP	59362576
P5_3	nor-SNP	56367696
P5_3	nor-SNP	10436238
P5_3	nor-SNP	10436219
P5_3	nor-SNP	10436201
P5_3	nor-SNP	10436194
P5_3	nor-SNP	10436187
P5_3	nor-SNP	10436180
P5_3	nor-SNP	97464049
P5_3	nor-SNP	97464057
P5_3	nor-SNP	103361245
P5_3	nor-SNP	103361226
P5_3	nor-SNP	103361221
P5_3	nor-SNP	61773937
P5_3	nor-SNP	28444104
P5_3	nor-SNP	28444157
P5_3	nor-SNP	163889327
P5_3	nor-SNP	163889342
P5_3	nor-SNP	33967800
P5_3	nor-SNP	62572885
P5_3	nor-SNP	149396242

P5_3	nor-SNP	149396272
P5_3	nor-SNP	149396273
P5_3	nor-SNP	149396288
P5_3	nor-SNP	13947170
P5_3	nor-SNP	176998548
P5_3	nor-SNP	50623143
P5_3	nor-SNP	50623110
P5_3	nor-SNP	34963416
P5_3	nor-SNP	34963445
P5_3	nor-SNP	34963459
P5_3	nor-SNP	63116226
P5_3	nor-SNP	27209165
P5_3	nor-SNP	105807912
P5_3	nor-SNP	29891262
P5_3	nor-SNP	29891260
P5_3	nor-SNP	29891245
P5_3	nor-SNP	29891235
P5_3	nor-SNP	19405709
P5_3	nor-SNP	19405743
P5_3	nor-SNP	101493478
P5_3	nor-SNP	46486996
P5_3	nor-SNP	54228743
P5_3	nor-SNP	54228750
P5_3	nor-SNP	54228719
P5_3	nor-SNP	148810267
P5_3	nor-SNP	86368898
P5_3	nor-SNP	86368922
P5_3	nor-SNP	86368890
P5_3	nor-SNP	93466919
P5_3	nor-SNP	93466913
P5_3	nor-SNP	93466912
P5_3	nor-SNP	93466910
P5_3	nor-SNP	93466909
P5_3	nor-SNP	93466866
P5_3	nor-SNP	97847498
P5_3	nor-SNP	97847569
P5_3	nor-SNP	97847573
P5_3	nor-SNP	49767769
P5_3	nor-SNP	49767815
P5_3	nor-SNP	49767832
P5_3	nor-SNP	49767835
P5_3	nor-SNP	49767838
P5_3	nor-SNP	29887033
P5_3	nor-SNP	61162131
P5_3	nor-SNP	61162191
P5_3	nor-SNP	9211782
P5_3	nor-SNP	9211802

P5_3	nor-SNP	146360765
P5_3	nor-SNP	146360845
P5_3	nor-SNP	146360813
P5_3	nor-SNP	146360779
P5_3	nor-SNP	146360826
P5_3	nor-SNP	97957646
P5_3	nor-SNP	97957677
P5_3	nor-SNP	129410313
P5_3	nor-SNP	129410239
P5_3	nor-SNP	129410235
P5_3	nor-SNP	129410228
P5_3	nor-SNP	129410227
P5_3	nor-SNP	73506984
P5_3	nor-SNP	129414852
P5_3	nor-SNP	129414843
P5_3	nor-SNP	129414815
P5_3	nor-SNP	71591230
P5_3	nor-SNP	71591179
P5_3	nor-SNP	71591164
P5_3	nor-SNP	71591156
P5_3	nor-SNP	46522190
P5_3	nor-SNP	46522201
P5_3	nor-SNP	46522255
P5_3	nor-SNP	209605507
P5_3	nor-SNP	209605539
P5_3	nor-SNP	209605546
P5_3	nor-SNP	220373939
P5_3	nor-SNP	220373943
P5_3	nor-SNP	26026988
P5_3	nor-SNP	77732628
P5_3	nor-SNP	77732603
P5_3	nor-SNP	15737185
P5_3	nor-SNP	15737186
P5_3	nor-SNP	15737151
P5_3	nor-SNP	127456029
P5_3	nor-SNP	127456020
P5_3	nor-SNP	132113349
P5_3	nor-SNP	61870141
P5_3	nor-SNP	61870162
P5_3	nor-SNP	49048277
P5_3	nor-SNP	78072676
P5_3	nor-SNP	115933905
P5_3	nor-SNP	115933922
P5_3	nor-SNP	115933913
P5_3	nor-SNP	130135961
P5_3	nor-SNP	12172783
P5_3	nor-SNP	12172775

P5_3	nor-SNP	12172788
P5_3	nor-SNP	85158670
P5_3	nor-SNP	115933918
P5_3	nor-SNP	115933890
P5_3	nor-SNP	115933876
P5_3	nor-SNP	57472742
P5_3	nor-SNP	38010903
P5_3	nor-SNP	38010964
P5_3	nor-SNP	129414568
P5_3	nor-SNP	207648009
P5_3	nor-SNP	207648001
P5_3	nor-SNP	207647981
P5_3	nor-SNP	33967806
P5_3	nor-SNP	62572874
P5_3	nor-SNP	146280644
P5_3	nor-SNP	146280626
P5_3	nor-SNP	146271304
P5_3	nor-SNP	146271303
P5_3	nor-SNP	146271269
P5_3	nor-SNP	146271266
P5_3	nor-SNP	146271235
P5_3	nor-SNP	146271231
P5_3	nor-SNP	133674439
P5_3	nor-SNP	133674418
P5_3	nor-SNP	133674399
P5_3	nor-SNP	146366167
P5_3	nor-SNP	220291302
P5_3	nor-SNP	135817150
P5_3	nor-SNP	6374394
P5_3	nor-SNP	117637290
P5_3	nor-SNP	117637326
P5_3	nor-SNP	117637302
P5_3	nor-SNP	49768168
P5_3	nor-SNP	49768171
P5_3	nor-SNP	54200810
P5_3	nor-SNP	54200826
P5_3	nor-SNP	54200830
P5_3	nor-SNP	54200843
P5_3	nor-SNP	54200853
P5_3	nor-SNP	54200834
P5_3	nor-SNP	116971750
P5_3	nor-SNP	116971778
P5_3	nor-SNP	135812836
P5_3	nor-SNP	88024462
P5_3	nor-SNP	64658716
P5_3	nor-SNP	64658715
P5_3	nor-SNP	64658710

P5_3	nor-SNP	64658640
P5_3	nor-SNP	129414807
P5_3	nor-SNP	129414806
P5_3	nor-SNP	129414804
P5_3	nor-SNP	146353894
P5_3	nor-SNP	48118349
P5_3	nor-SNP	48118350
P5_3	nor-SNP	48118351
P5_3	nor-SNP	48118365
P5_3	nor-SNP	48118374
P5_3	nor-SNP	37093078
P5_3	nor-SNP	37093089
P5_3	nor-SNP	37093097
P5_3	nor-SNP	37093013
P5_3	nor-SNP	31924708
P5_3	nor-SNP	35391065
P5_3	nor-SNP	35391051
P5_3	nor-SNP	20179097
P5_3	nor-SNP	20179098
P5_3	nor-SNP	20179130
P5_3	nor-SNP	10514202
P5_3	nor-SNP	17962615
P5_3	nor-SNP	95604259
P5_3	nor-SNP	52196045
P5_3	nor-SNP	52196076
P5_3	nor-SNP	18409354
P5_3	nor-SNP	163889284
P5_3	nor-SNP	56118358
P5_3	nor-SNP	56118359
P5_3	nor-SNP	15737177
P5_3	nor-SNP	104583828
P5_3	nor-SNP	104196300
P5_3	nor-SNP	19405702
P5_3	nor-SNP	45605666
P5_3	nor-SNP	73125664
P5_3	nor-SNP	26188908
P5_3	nor-SNP	26188825
P5_3	nor-SNP	26188906
P5_3	nor-SNP	26188880
P5_3	nor-SNP	38010938
P5_3	nor-SNP	130452987
P5_3	nor-SNP	130452977
P5_3	nor-SNP	130452968
P5_3	nor-SNP	33578202
P5_3	nor-SNP	33578201
P5_3	nor-SNP	29814829
P5_3	nor-SNP	170120566

P5_3	nor-SNP	135821099
P5_3	nor-SNP	92956420
P5_3	nor-SNP	92956416
P5_3	nor-SNP	15537776
P5_3	nor-SNP	15737217
P5_3	nor-SNP	92956443
P5_3	nor-SNP	151127092
P5_3	nor-SNP	151127105
P5_3	nor-SNP	33578255
P5_3	nor-SNP	33578251
P5_3	nor-SNP	101351088
P5_3	nor-SNP	61918164
P5_3	nor-SNP	2018056
P5_3	nor-SNP	2018004
P5_3	nor-SNP	2018002
P5_3	nor-SNP	10514200
P5_3	nor-SNP	117520389
P5_3	nor-SNP	33578206
P5_3	nor-SNP	78072653
P5_3	nor-SNP	16645178
P5_3	nor-SNP	54216629
P5_3	nor-SNP	133674612
P5_3	nor-SNP	133674576
P5_3	nor-SNP	127456042
P5_3	nor-SNP	167967953
P5_3	nor-SNP	167967958
P5_3	nor-SNP	167967963
P5_3	nor-SNP	101530833
P5_3	nor-SNP	146312254
P5_3	nor-SNP	54290994
P5_3	nor-SNP	14478575
P5_3	nor-SNP	14478613
P5_3	nor-SNP	188406598
P5_3	nor-SNP	135061124
P5_3	nor-SNP	135061112
P5_3	nor-SNP	135061111
P5_3	nor-SNP	135061109
P5_3	nor-SNP	135061106
P5_3	nor-SNP	135061039
P5_3	nor-SNP	36428048
P5_3	nor-SNP	6374387
P5_3	nor-SNP	46142293
P5_3	nor-SNP	49773603
P5_3	nor-SNP	180407512
P5_3	nor-SNP	180407505
P5_3	nor-SNP	180407488
P5_3	nor-SNP	137742013

P5_3	nor-SNP	137741994
P5_3	nor-SNP	137741987
P5_3	nor-SNP	137741989
P5_3	nor-SNP	57918678
P5_3	nor-SNP	97847535
P5_3	nor-SNP	15737218
P5_3	nor-SNP	104583796
P5_3	nor-SNP	104583804
P5_3	nor-SNP	101521092
P5_3	nor-SNP	63005918
P5_3	nor-SNP	26946325
P5_3	nor-SNP	104583838
P5_3	nor-SNP	104583776
P5_3	nor-SNP	104583759
P5_3	nor-SNP	112273768
P5_3	nor-SNP	21785508
P5_3	nor-SNP	72113306
P5_3	nor-SNP	72113270
P5_3	nor-SNP	72113269
P5_3	nor-SNP	72113261
P5_3	nor-SNP	153410520
P5_3	nor-SNP	9211778
P5_3	nor-SNP	9211747
P5_3	nor-SNP	9211738
P5_3	nor-SNP	97824129
P5_3	nor-SNP	97824125
P5_3	nor-SNP	97824075
P5_3	nor-SNP	101531654
P5_3	nor-SNP	219267370
P5_3	nor-SNP	54175294
P5_3	nor-SNP	79107068
P5_3	nor-SNP	129162366
P5_3	nor-SNP	129162428
P5_3	nor-SNP	54197674
P5_3	nor-SNP	54197678
P5_3	nor-SNP	101351048
P5_3	nor-SNP	33578276
P5_3	nor-SNP	88024452
P5_3	nor-SNP	98860808
P5_3	nor-SNP	98860801
P5_3	nor-SNP	101528426
P5_3	nor-SNP	101528401
P5_3	nor-SNP	7126698
P5_3	nor-SNP	7126619
P5_3	nor-SNP	16645137
P5_3	nor-SNP	46801821
P5_3	nor-SNP	2633462

P5_3	nor-SNP	139006378
P5_3	nor-SNP	139006339
P5_3	nor-SNP	79099736
P5_3	nor-SNP	155174524
P5_3	nor-SNP	155174534
P5_3	nor-SNP	155174551
P5_3	nor-SNP	145619405
P5_3	nor-SNP	145619442
P5_3	nor-SNP	145619377
P5_3	nor-SNP	145619386
P5_3	nor-SNP	13947436
P5_3	nor-SNP	54216615
P5_3	nor-SNP	1062662
P5_3	nor-SNP	101530873
P5_3	nor-SNP	101530834
P5_3	nor-SNP	167967904
P5_3	nor-SNP	133674285
P5_3	nor-SNP	49048263
P5_3	nor-SNP	1062653
P5_3	nor-SNP	1062626
P5_3	nor-SNP	100774203
P5_3	nor-SNP	100774268
P5_3	nor-SNP	100774277
P5_3	nor-SNP	100774289
P5_3	nor-SNP	188406636
P5_3	nor-SNP	70519078
P5_3	nor-SNP	101335438
P5_3	nor-SNP	101335451
P5_3	nor-SNP	97824145
P5_3	nor-SNP	57392715
P5_3	nor-SNP	57392686
P5_3	nor-SNP	134884697
P5_3	nor-SNP	79107049
P5_3	nor-SNP	79107061
P5_3	nor-SNP	79107017
P5_3	nor-SNP	14478576
P5_3	nor-SNP	137742041
P5_3	nor-SNP	1103284
P5_3	nor-SNP	79107084
P5_3	nor-SNP	127456067
P5_3	nor-SNP	127456068
P5_3	nor-SNP	56216187
P5_3	nor-SNP	56216092
P5_3	nor-SNP	56216090
P5_3	nor-SNP	41517981
P5_3	nor-SNP	41517974
P5_3	nor-SNP	2633434

P5_3	nor-SNP	133680673
P5_3	nor-SNP	153410531
P5_3	nor-SNP	33175642
P5_3	nor-SNP	33175702
P5_3	nor-SNP	205417483
P5_3	nor-SNP	205417438
P5_3	nor-SNP	54225460
P5_3	nor-SNP	54225463
P5_3	nor-SNP	54225426
P5_3	nor-SNP	54216650
P5_3	nor-SNP	19405676
P5_3	nor-SNP	19405672
P5_3	nor-SNP	101526127
P5_3	nor-SNP	101526140
P5_3	nor-SNP	101526116
P5_3	nor-SNP	113569406
P5_3	nor-SNP	72086720
P5_3	nor-SNP	101531854
P5_3	nor-SNP	101531857
P5_3	nor-SNP	134884700
P5_3	nor-SNP	76225915
P5_3	nor-SNP	76225848
P5_3	nor-SNP	76225837
P5_3	nor-SNP	236016301
P5_3	nor-SNP	85775275
P5_3	nor-SNP	101340844
P5_3	nor-SNP	101340862
P5_3	nor-SNP	128972936
P5_3	nor-SNP	128972923
P5_3	nor-SNP	128972925
P5_3	nor-SNP	101531849
P5_3	nor-SNP	101531858
P5_3	nor-SNP	129162390
P5_3	nor-SNP	36428046
P5_3	nor-SNP	61870167
P5_3	nor-SNP	61870140
P5_3	nor-SNP	113997768
P5_3	nor-SNP	113997812
P5_3	nor-SNP	113997817
P5_3	nor-SNP	133675375
P5_3	nor-SNP	46114572
P5_3	nor-SNP	46114610
P5_3	nor-SNP	46114580
P5_3	nor-SNP	150935577
P5_3	nor-SNP	150935522
P5_3	nor-SNP	150935583
P5_3	nor-SNP	25551588

P5_3	nor-SNP	25551583
P5_3	nor-SNP	25551550
P5_3	nor-SNP	70718466
P5_3	nor-SNP	56227910
P5_3	nor-SNP	56227905
P5_3	nor-SNP	1816176
P5_3	nor-SNP	1816169
P5_3	nor-SNP	28444183
P5_3	nor-SNP	136983319
P5_3	nor-SNP	61582680
P5_3	nor-SNP	61582708
P5_3	nor-SNP	134884717
P5_3	nor-SNP	240273490
P5_3	nor-SNP	111781790
P5_3	nor-SNP	111781746
P5_3	nor-SNP	111781744
P5_3	nor-SNP	111781785
P5_3	nor-SNP	13985739
P5_3	nor-SNP	13985772
P5_3	nor-SNP	68850698
P5_3	nor-SNP	68850706
P5_3	nor-SNP	68850720
P5_3	nor-SNP	101500133
P5_3	nor-SNP	101500166
P5_3	nor-SNP	101500167
P5_3	nor-SNP	54197706
P5_3	nor-SNP	10287821
P5_3	nor-SNP	10287820
P5_3	nor-SNP	31357244
P5_3	nor-SNP	31357238
P5_3	nor-SNP	31357325
P5_3	nor-SNP	31357245
P5_3	nor-SNP	201777741
P5_3	nor-SNP	12877500
P5_3	nor-SNP	96074638
P5_3	nor-SNP	13446893
P5_3	nor-SNP	13446924
P5_3	nor-SNP	2633466
P5_3	nor-SNP	10662844
P5_3	nor-SNP	10662859
P5_3	nor-SNP	10662866
P5_3	nor-SNP	54466378
P5_3	nor-SNP	54466362
P5_3	nor-SNP	48357864
P5_3	nor-SNP	48357853
P5_3	nor-SNP	13985689
P5_3	nor-SNP	122017258

P5_3	nor-SNP	2140269
P5_3	nor-SNP	33967787
P5_3	nor-SNP	62572847
P5_3	nor-SNP	105807909
P5_3	nor-SNP	54228775
P5_3	nor-SNP	86368929
P5_3	nor-SNP	146360778
P5_3	nor-SNP	46522298
P5_3	nor-SNP	220373934
P5_3	nor-SNP	220373933
P5_3	nor-SNP	220373922
P5_3	nor-SNP	132113371
P5_3	nor-SNP	129414553
P5_3	nor-SNP	220291292
P5_3	nor-SNP	117637325
P5_3	nor-SNP	1988193
P5_3	nor-SNP	64658705
P5_3	nor-SNP	64658623
P5_3	nor-SNP	35391105
P5_3	nor-SNP	10514194
P5_3	nor-SNP	10514190
P5_3	nor-SNP	10514159
P5_3	nor-SNP	10514157
P5_3	nor-SNP	26188865
P5_3	nor-SNP	170120575
P5_3	nor-SNP	170120594
P5_3	nor-SNP	135821135
P5_3	nor-SNP	92956409
P5_3	nor-SNP	33967796
P5_3	nor-SNP	54216681
P5_3	nor-SNP	219267443
P5_3	nor-SNP	219267433
P5_3	nor-SNP	219267402
P5_3	nor-SNP	219267407
P5_3	nor-SNP	98860816
P5_3	nor-SNP	2633478
P5_3	nor-SNP	145619365
P5_3	nor-SNP	1062656
P5_3	nor-SNP	1062599
P5_3	nor-SNP	1062574
P5_3	nor-SNP	1103312
P5_3	nor-SNP	1103328
P5_3	nor-SNP	1103331
P5_3	nor-SNP	219267371
P5_3	nor-SNP	41518025
P5_3	nor-SNP	56216156
P5_3	nor-SNP	54225501

P5_3	nor-SNP	69330823
P5_3	nor-SNP	113569394
P5_3	nor-SNP	101531862
P5_3	nor-SNP	70519093
P5_3	nor-SNP	136983275
P5_3	nor-SNP	57392697
P5_3	nor-SNP	61582659
P5_3	nor-SNP	61582649
P5_3	nor-SNP	134884723
P5_3	nor-SNP	134884737
P5_3	nor-SNP	13985806
P5_3	nor-SNP	10287854
P5_3	nor-SNP	201777751
P5_3	nor-SNP	12877525
P5_3	nor-SNP	12877502
P5_3	nor-SNP	96074649
P5_3	nor-SNP	13446846
P5_3	nor-SNP	2633480
P5_3	nor-SNP	13985721
P5_3	nor-SNP	13985805
P5_3	nor-SNP	2140267
P5_3	nor-SNP	2140268
P5_3	nor-SNP	105807864
P5_3	nor-SNP	86368959
P5_3	nor-SNP	105807855
P5_3	nor-SNP	105807858
P5_3	nor-SNP	98860827
P5_3	nor-SNP	201777753
P5_3	nor-SNP	12877527
P5_3	nor-SNP	2140240
P5_3	nor-SNP	153410482
P5_3	nor-SNP	98860840
P5_3	iso-pre-miRNA>hsa-let-7b	
P5_3	iso-pre-miRNA>hsa-let-7i	
P5_3	iso-pre-miRNA>hsa-mir-105-1	
P5_3	iso-pre-miRNA>hsa-mir-105-2	
P5_3	iso-pre-miRNA>hsa-mir-106b	
P5_3	iso-pre-miRNA>hsa-mir-10a	
P5_3	iso-pre-miRNA>hsa-mir-10b	
P5_3	iso-pre-miRNA>hsa-mir-1178	
P5_3	iso-pre-miRNA>hsa-mir-1179	
P5_3	iso-pre-miRNA>hsa-mir-1181	
P5_3	iso-pre-miRNA>hsa-mir-1185-1	
P5_3	iso-pre-miRNA>hsa-mir-1185-2	
P5_3	iso-pre-miRNA>hsa-mir-1193	
P5_3	iso-pre-miRNA>hsa-mir-1199	
P5_3	iso-pre-miRNA>hsa-mir-1200	

P5_3 iso-pre-miRNA>hsa-mir-1202
P5_3 iso-pre-miRNA>hsa-mir-1203
P5_3 iso-pre-miRNA>hsa-mir-1204
P5_3 iso-pre-miRNA>hsa-mir-1205
P5_3 iso-pre-miRNA>hsa-mir-1207
P5_3 iso-pre-miRNA>hsa-mir-1208
P5_3 iso-pre-miRNA>hsa-mir-1224
P5_3 iso-pre-miRNA>hsa-mir-1225
P5_3 iso-pre-miRNA>hsa-mir-1226
P5_3 iso-pre-miRNA>hsa-mir-1227
P5_3 iso-pre-miRNA>hsa-mir-1228
P5_3 iso-pre-miRNA>hsa-mir-1229
P5_3 iso-pre-miRNA>hsa-mir-1231
P5_3 iso-pre-miRNA>hsa-mir-1233-1
P5_3 iso-pre-miRNA>hsa-mir-1233-2
P5_3 iso-pre-miRNA>hsa-mir-1236
P5_3 iso-pre-miRNA>hsa-mir-1237
P5_3 iso-pre-miRNA>hsa-mir-124-2
P5_3 iso-pre-miRNA>hsa-mir-1243
P5_3 iso-pre-miRNA>hsa-mir-124-3
P5_3 iso-pre-miRNA>hsa-mir-1245b
P5_3 iso-pre-miRNA>hsa-mir-1246
P5_3 iso-pre-miRNA>hsa-mir-1248
P5_3 iso-pre-miRNA>hsa-mir-1251
P5_3 iso-pre-miRNA>hsa-mir-1252
P5_3 iso-pre-miRNA>hsa-mir-1253
P5_3 iso-pre-miRNA>hsa-mir-1254-1
P5_3 iso-pre-miRNA>hsa-mir-1254-2
P5_3 iso-pre-miRNA>hsa-mir-1255a
P5_3 iso-pre-miRNA>hsa-mir-1255b-1
P5_3 iso-pre-miRNA>hsa-mir-1257
P5_3 iso-pre-miRNA>hsa-mir-125a
P5_3 iso-pre-miRNA>hsa-mir-125b-2
P5_3 iso-pre-miRNA>hsa-mir-126
P5_3 iso-pre-miRNA>hsa-mir-1260a
P5_3 iso-pre-miRNA>hsa-mir-1260b
P5_3 iso-pre-miRNA>hsa-mir-1261
P5_3 iso-pre-miRNA>hsa-mir-1262
P5_3 iso-pre-miRNA>hsa-mir-1265
P5_3 iso-pre-miRNA>hsa-mir-1269b
P5_3 iso-pre-miRNA>hsa-mir-1272
P5_3 iso-pre-miRNA>hsa-mir-1273d
P5_3 iso-pre-miRNA>hsa-mir-1273f
P5_3 iso-pre-miRNA>hsa-mir-1273h
P5_3 iso-pre-miRNA>hsa-mir-1275
P5_3 iso-pre-miRNA>hsa-mir-1276
P5_3 iso-pre-miRNA>hsa-mir-1279

P5_3 iso-pre-miRNA>hsa-mir-128-1
P5_3 iso-pre-miRNA>hsa-mir-1282
P5_3 iso-pre-miRNA>hsa-mir-1283-1
P5_3 iso-pre-miRNA>hsa-mir-1283-2
P5_3 iso-pre-miRNA>hsa-mir-1287
P5_3 iso-pre-miRNA>hsa-mir-129-1
P5_3 iso-pre-miRNA>hsa-mir-1292
P5_3 iso-pre-miRNA>hsa-mir-129-2
P5_3 iso-pre-miRNA>hsa-mir-1295b
P5_3 iso-pre-miRNA>hsa-mir-1304
P5_3 iso-pre-miRNA>hsa-mir-1307
P5_3 iso-pre-miRNA>hsa-mir-130b
P5_3 iso-pre-miRNA>hsa-mir-134
P5_3 iso-pre-miRNA>hsa-mir-135a-1
P5_3 iso-pre-miRNA>hsa-mir-138-1
P5_3 iso-pre-miRNA>hsa-mir-138-2
P5_3 iso-pre-miRNA>hsa-mir-140
P5_3 iso-pre-miRNA>hsa-mir-141
P5_3 iso-pre-miRNA>hsa-mir-142
P5_3 iso-pre-miRNA>hsa-mir-143
P5_3 iso-pre-miRNA>hsa-mir-146a
P5_3 iso-pre-miRNA>hsa-mir-1470
P5_3 iso-pre-miRNA>hsa-mir-148b
P5_3 iso-pre-miRNA>hsa-mir-149
P5_3 iso-pre-miRNA>hsa-mir-152
P5_3 iso-pre-miRNA>hsa-mir-153-2
P5_3 iso-pre-miRNA>hsa-mir-1587
P5_3 iso-pre-miRNA>hsa-mir-15b
P5_3 iso-pre-miRNA>hsa-mir-17
P5_3 iso-pre-miRNA>hsa-mir-181b-1
P5_3 iso-pre-miRNA>hsa-mir-181c
P5_3 iso-pre-miRNA>hsa-mir-181d
P5_3 iso-pre-miRNA>hsa-mir-183
P5_3 iso-pre-miRNA>hsa-mir-185
P5_3 iso-pre-miRNA>hsa-mir-187
P5_3 iso-pre-miRNA>hsa-mir-18a
P5_3 iso-pre-miRNA>hsa-mir-1908
P5_3 iso-pre-miRNA>hsa-mir-190b
P5_3 iso-pre-miRNA>hsa-mir-1910
P5_3 iso-pre-miRNA>hsa-mir-1911
P5_3 iso-pre-miRNA>hsa-mir-1914
P5_3 iso-pre-miRNA>hsa-mir-192
P5_3 iso-pre-miRNA>hsa-mir-194-1
P5_3 iso-pre-miRNA>hsa-mir-194-2
P5_3 iso-pre-miRNA>hsa-mir-195
P5_3 iso-pre-miRNA>hsa-mir-196a-2
P5_3 iso-pre-miRNA>hsa-mir-197

P5_3 iso-pre-miRNA>hsa-mir-198
P5_3 iso-pre-miRNA>hsa-mir-199a-1
P5_3 iso-pre-miRNA>hsa-mir-199a-2
P5_3 iso-pre-miRNA>hsa-mir-199b
P5_3 iso-pre-miRNA>hsa-mir-19b-2
P5_3 iso-pre-miRNA>hsa-mir-200a
P5_3 iso-pre-miRNA>hsa-mir-200b
P5_3 iso-pre-miRNA>hsa-mir-202
P5_3 iso-pre-miRNA>hsa-mir-203b
P5_3 iso-pre-miRNA>hsa-mir-208b
P5_3 iso-pre-miRNA>hsa-mir-20a
P5_3 iso-pre-miRNA>hsa-mir-20b
P5_3 iso-pre-miRNA>hsa-mir-210
P5_3 iso-pre-miRNA>hsa-mir-211
P5_3 iso-pre-miRNA>hsa-mir-2110
P5_3 iso-pre-miRNA>hsa-mir-2115
P5_3 iso-pre-miRNA>hsa-mir-2116
P5_3 iso-pre-miRNA>hsa-mir-215
P5_3 iso-pre-miRNA>hsa-mir-218-1
P5_3 iso-pre-miRNA>hsa-mir-218-2
P5_3 iso-pre-miRNA>hsa-mir-219a-2
P5_3 iso-pre-miRNA>hsa-mir-219b
P5_3 iso-pre-miRNA>hsa-mir-222
P5_3 iso-pre-miRNA>hsa-mir-223
P5_3 iso-pre-miRNA>hsa-mir-2276
P5_3 iso-pre-miRNA>hsa-mir-2277
P5_3 iso-pre-miRNA>hsa-mir-2278
P5_3 iso-pre-miRNA>hsa-mir-2355
P5_3 iso-pre-miRNA>hsa-mir-25
P5_3 iso-pre-miRNA>hsa-mir-2681
P5_3 iso-pre-miRNA>hsa-mir-2682
P5_3 iso-pre-miRNA>hsa-mir-26b
P5_3 iso-pre-miRNA>hsa-mir-27a
P5_3 iso-pre-miRNA>hsa-mir-27b
P5_3 iso-pre-miRNA>hsa-mir-2909
P5_3 iso-pre-miRNA>hsa-mir-296
P5_3 iso-pre-miRNA>hsa-mir-301a
P5_3 iso-pre-miRNA>hsa-mir-302a
P5_3 iso-pre-miRNA>hsa-mir-302b
P5_3 iso-pre-miRNA>hsa-mir-302c
P5_3 iso-pre-miRNA>hsa-mir-302d
P5_3 iso-pre-miRNA>hsa-mir-302e
P5_3 iso-pre-miRNA>hsa-mir-3064
P5_3 iso-pre-miRNA>hsa-mir-3065
P5_3 iso-pre-miRNA>hsa-mir-30e
P5_3 iso-pre-miRNA>hsa-mir-3117
P5_3 iso-pre-miRNA>hsa-mir-3119-1

P5_3 iso-pre-miRNA>hsa-mir-3119-2
P5_3 iso-pre-miRNA>hsa-mir-3124
P5_3 iso-pre-miRNA>hsa-mir-3125
P5_3 iso-pre-miRNA>hsa-mir-3126
P5_3 iso-pre-miRNA>hsa-mir-3128
P5_3 iso-pre-miRNA>hsa-mir-3129
P5_3 iso-pre-miRNA>hsa-mir-3131
P5_3 iso-pre-miRNA>hsa-mir-3132
P5_3 iso-pre-miRNA>hsa-mir-3135b
P5_3 iso-pre-miRNA>hsa-mir-3139
P5_3 iso-pre-miRNA>hsa-mir-3140
P5_3 iso-pre-miRNA>hsa-mir-3141
P5_3 iso-pre-miRNA>hsa-mir-3143
P5_3 iso-pre-miRNA>hsa-mir-3144
P5_3 iso-pre-miRNA>hsa-mir-3145
P5_3 iso-pre-miRNA>hsa-mir-3150a
P5_3 iso-pre-miRNA>hsa-mir-3150b
P5_3 iso-pre-miRNA>hsa-mir-3151
P5_3 iso-pre-miRNA>hsa-mir-3152
P5_3 iso-pre-miRNA>hsa-mir-3156-1
P5_3 iso-pre-miRNA>hsa-mir-3156-2
P5_3 iso-pre-miRNA>hsa-mir-3156-3
P5_3 iso-pre-miRNA>hsa-mir-3162
P5_3 iso-pre-miRNA>hsa-mir-3168
P5_3 iso-pre-miRNA>hsa-mir-3170
P5_3 iso-pre-miRNA>hsa-mir-3171
P5_3 iso-pre-miRNA>hsa-mir-3175
P5_3 iso-pre-miRNA>hsa-mir-3177
P5_3 iso-pre-miRNA>hsa-mir-3178
P5_3 iso-pre-miRNA>hsa-mir-3182
P5_3 iso-pre-miRNA>hsa-mir-3183
P5_3 iso-pre-miRNA>hsa-mir-3184
P5_3 iso-pre-miRNA>hsa-mir-3186
P5_3 iso-pre-miRNA>hsa-mir-3192
P5_3 iso-pre-miRNA>hsa-mir-3195
P5_3 iso-pre-miRNA>hsa-mir-3199-1
P5_3 iso-pre-miRNA>hsa-mir-3199-2
P5_3 iso-pre-miRNA>hsa-mir-323b
P5_3 iso-pre-miRNA>hsa-mir-328
P5_3 iso-pre-miRNA>hsa-mir-329-1
P5_3 iso-pre-miRNA>hsa-mir-338
P5_3 iso-pre-miRNA>hsa-mir-33a
P5_3 iso-pre-miRNA>hsa-mir-33b
P5_3 iso-pre-miRNA>hsa-mir-340
P5_3 iso-pre-miRNA>hsa-mir-342
P5_3 iso-pre-miRNA>hsa-mir-3605
P5_3 iso-pre-miRNA>hsa-mir-3607

P5_3 iso-pre-miRNA>hsa-mir-3612
P5_3 iso-pre-miRNA>hsa-mir-3617
P5_3 iso-pre-miRNA>hsa-mir-3620
P5_3 iso-pre-miRNA>hsa-mir-3622a
P5_3 iso-pre-miRNA>hsa-mir-3622b
P5_3 iso-pre-miRNA>hsa-mir-363
P5_3 iso-pre-miRNA>hsa-mir-3652
P5_3 iso-pre-miRNA>hsa-mir-365a
P5_3 iso-pre-miRNA>hsa-mir-365b
P5_3 iso-pre-miRNA>hsa-mir-3663
P5_3 iso-pre-miRNA>hsa-mir-3667
P5_3 iso-pre-miRNA>hsa-mir-3674
P5_3 iso-pre-miRNA>hsa-mir-3678
P5_3 iso-pre-miRNA>hsa-mir-3679
P5_3 iso-pre-miRNA>hsa-mir-3682
P5_3 iso-pre-miRNA>hsa-mir-3683
P5_3 iso-pre-miRNA>hsa-mir-370
P5_3 iso-pre-miRNA>hsa-mir-3714
P5_3 iso-pre-miRNA>hsa-mir-371a
P5_3 iso-pre-miRNA>hsa-mir-371b
P5_3 iso-pre-miRNA>hsa-mir-372
P5_3 iso-pre-miRNA>hsa-mir-373
P5_3 iso-pre-miRNA>hsa-mir-376a-2
P5_3 iso-pre-miRNA>hsa-mir-377
P5_3 iso-pre-miRNA>hsa-mir-378a
P5_3 iso-pre-miRNA>hsa-mir-378c
P5_3 iso-pre-miRNA>hsa-mir-378d-2
P5_3 iso-pre-miRNA>hsa-mir-378h
P5_3 iso-pre-miRNA>hsa-mir-378i
P5_3 iso-pre-miRNA>hsa-mir-379
P5_3 iso-pre-miRNA>hsa-mir-381
P5_3 iso-pre-miRNA>hsa-mir-382
P5_3 iso-pre-miRNA>hsa-mir-383
P5_3 iso-pre-miRNA>hsa-mir-3908
P5_3 iso-pre-miRNA>hsa-mir-3912
P5_3 iso-pre-miRNA>hsa-mir-3916
P5_3 iso-pre-miRNA>hsa-mir-3922
P5_3 iso-pre-miRNA>hsa-mir-3945
P5_3 iso-pre-miRNA>hsa-mir-410
P5_3 iso-pre-miRNA>hsa-mir-411
P5_3 iso-pre-miRNA>hsa-mir-412
P5_3 iso-pre-miRNA>hsa-mir-423
P5_3 iso-pre-miRNA>hsa-mir-425
P5_3 iso-pre-miRNA>hsa-mir-4258
P5_3 iso-pre-miRNA>hsa-mir-4260
P5_3 iso-pre-miRNA>hsa-mir-4267
P5_3 iso-pre-miRNA>hsa-mir-4269

P5_3 iso-pre-miRNA>hsa-mir-4277
P5_3 iso-pre-miRNA>hsa-mir-4280
P5_3 iso-pre-miRNA>hsa-mir-4283-1
P5_3 iso-pre-miRNA>hsa-mir-4283-2
P5_3 iso-pre-miRNA>hsa-mir-4286
P5_3 iso-pre-miRNA>hsa-mir-4289
P5_3 iso-pre-miRNA>hsa-mir-4294
P5_3 iso-pre-miRNA>hsa-mir-4296
P5_3 iso-pre-miRNA>hsa-mir-4297
P5_3 iso-pre-miRNA>hsa-mir-4298
P5_3 iso-pre-miRNA>hsa-mir-4301
P5_3 iso-pre-miRNA>hsa-mir-4305
P5_3 iso-pre-miRNA>hsa-mir-4309
P5_3 iso-pre-miRNA>hsa-mir-431
P5_3 iso-pre-miRNA>hsa-mir-432
P5_3 iso-pre-miRNA>hsa-mir-4327
P5_3 iso-pre-miRNA>hsa-mir-433
P5_3 iso-pre-miRNA>hsa-mir-449b
P5_3 iso-pre-miRNA>hsa-mir-449c
P5_3 iso-pre-miRNA>hsa-mir-452
P5_3 iso-pre-miRNA>hsa-mir-454
P5_3 iso-pre-miRNA>hsa-mir-483
P5_3 iso-pre-miRNA>hsa-mir-485
P5_3 iso-pre-miRNA>hsa-mir-486-2
P5_3 iso-pre-miRNA>hsa-mir-487a
P5_3 iso-pre-miRNA>hsa-mir-487b
P5_3 iso-pre-miRNA>hsa-mir-489
P5_3 iso-pre-miRNA>hsa-mir-490
P5_3 iso-pre-miRNA>hsa-mir-491
P5_3 iso-pre-miRNA>hsa-mir-492
P5_3 iso-pre-miRNA>hsa-mir-495
P5_3 iso-pre-miRNA>hsa-mir-497
P5_3 iso-pre-miRNA>hsa-mir-499b
P5_3 iso-pre-miRNA>hsa-mir-500a
P5_3 iso-pre-miRNA>hsa-mir-500b
P5_3 iso-pre-miRNA>hsa-mir-501
P5_3 iso-pre-miRNA>hsa-mir-502
P5_3 iso-pre-miRNA>hsa-mir-503
P5_3 iso-pre-miRNA>hsa-mir-504
P5_3 iso-pre-miRNA>hsa-mir-508
P5_3 iso-pre-miRNA>hsa-mir-509-1
P5_3 iso-pre-miRNA>hsa-mir-509-2
P5_3 iso-pre-miRNA>hsa-mir-509-3
P5_3 iso-pre-miRNA>hsa-mir-511
P5_3 iso-pre-miRNA>hsa-mir-512-1
P5_3 iso-pre-miRNA>hsa-mir-512-2
P5_3 iso-pre-miRNA>hsa-mir-513a-1

P5_3 iso-pre-miRNA>hsa-mir-513a-2
P5_3 iso-pre-miRNA>hsa-mir-514a-1
P5_3 iso-pre-miRNA>hsa-mir-515-1
P5_3 iso-pre-miRNA>hsa-mir-516a-1
P5_3 iso-pre-miRNA>hsa-mir-516a-2
P5_3 iso-pre-miRNA>hsa-mir-516b-1
P5_3 iso-pre-miRNA>hsa-mir-516b-2
P5_3 iso-pre-miRNA>hsa-mir-517a
P5_3 iso-pre-miRNA>hsa-mir-517c
P5_3 iso-pre-miRNA>hsa-mir-518a-1
P5_3 iso-pre-miRNA>hsa-mir-518a-2
P5_3 iso-pre-miRNA>hsa-mir-518c
P5_3 iso-pre-miRNA>hsa-mir-518d
P5_3 iso-pre-miRNA>hsa-mir-518e
P5_3 iso-pre-miRNA>hsa-mir-518f
P5_3 iso-pre-miRNA>hsa-mir-519a-1
P5_3 iso-pre-miRNA>hsa-mir-519b
P5_3 iso-pre-miRNA>hsa-mir-519c
P5_3 iso-pre-miRNA>hsa-mir-519d
P5_3 iso-pre-miRNA>hsa-mir-520a
P5_3 iso-pre-miRNA>hsa-mir-520c
P5_3 iso-pre-miRNA>hsa-mir-520d
P5_3 iso-pre-miRNA>hsa-mir-520f
P5_3 iso-pre-miRNA>hsa-mir-520g
P5_3 iso-pre-miRNA>hsa-mir-522
P5_3 iso-pre-miRNA>hsa-mir-523
P5_3 iso-pre-miRNA>hsa-mir-524
P5_3 iso-pre-miRNA>hsa-mir-525
P5_3 iso-pre-miRNA>hsa-mir-526a-1
P5_3 iso-pre-miRNA>hsa-mir-526a-2
P5_3 iso-pre-miRNA>hsa-mir-526b
P5_3 iso-pre-miRNA>hsa-mir-527
P5_3 iso-pre-miRNA>hsa-mir-541
P5_3 iso-pre-miRNA>hsa-mir-548a-3
P5_3 iso-pre-miRNA>hsa-mir-548ab
P5_3 iso-pre-miRNA>hsa-mir-548ag-2
P5_3 iso-pre-miRNA>hsa-mir-548ai
P5_3 iso-pre-miRNA>hsa-mir-548aj-2
P5_3 iso-pre-miRNA>hsa-mir-548am
P5_3 iso-pre-miRNA>hsa-mir-548an
P5_3 iso-pre-miRNA>hsa-mir-548ao
P5_3 iso-pre-miRNA>hsa-mir-548ap
P5_3 iso-pre-miRNA>hsa-mir-548ar
P5_3 iso-pre-miRNA>hsa-mir-548at
P5_3 iso-pre-miRNA>hsa-mir-548aw
P5_3 iso-pre-miRNA>hsa-mir-548ay
P5_3 iso-pre-miRNA>hsa-mir-548b

P5_3 iso-pre-miRNA>hsa-mir-548c
P5_3 iso-pre-miRNA>hsa-mir-548d-1
P5_3 iso-pre-miRNA>hsa-mir-548d-2
P5_3 iso-pre-miRNA>hsa-mir-548g
P5_3 iso-pre-miRNA>hsa-mir-548h-1
P5_3 iso-pre-miRNA>hsa-mir-548h-2
P5_3 iso-pre-miRNA>hsa-mir-548h-3
P5_3 iso-pre-miRNA>hsa-mir-548h-4
P5_3 iso-pre-miRNA>hsa-mir-548h-5
P5_3 iso-pre-miRNA>hsa-mir-659
P5_3 iso-pre-miRNA>hsa-mir-660
P5_3 iso-pre-miRNA>hsa-mir-663a
P5_3 iso-pre-miRNA>hsa-mir-664a
P5_3 iso-pre-miRNA>hsa-mir-664b
P5_3 iso-pre-miRNA>hsa-mir-668
P5_3 iso-pre-miRNA>hsa-mir-670
P5_3 iso-pre-miRNA>hsa-mir-671
P5_3 iso-pre-miRNA>hsa-mir-675
P5_3 iso-pre-miRNA>hsa-mir-676
P5_3 iso-pre-miRNA>hsa-mir-708
P5_3 iso-pre-miRNA>hsa-mir-7-1
P5_3 iso-pre-miRNA>hsa-mir-7-2
P5_3 iso-pre-miRNA>hsa-mir-7-3
P5_3 iso-pre-miRNA>hsa-mir-744
P5_3 iso-pre-miRNA>hsa-mir-758
P5_3 iso-pre-miRNA>hsa-mir-759
P5_3 iso-pre-miRNA>hsa-mir-761
P5_3 iso-pre-miRNA>hsa-mir-766
P5_3 iso-pre-miRNA>hsa-mir-767
P5_3 iso-pre-miRNA>hsa-mir-769
P5_3 iso-pre-miRNA>hsa-mir-770
P5_3 iso-pre-miRNA>hsa-mir-802
P5_3 iso-pre-miRNA>hsa-mir-873
P5_3 iso-pre-miRNA>hsa-mir-874
P5_3 iso-pre-miRNA>hsa-mir-876
P5_3 iso-pre-miRNA>hsa-mir-877
P5_3 iso-pre-miRNA>hsa-mir-885
P5_3 iso-pre-miRNA>hsa-mir-887
P5_3 iso-pre-miRNA>hsa-mir-888
P5_3 iso-pre-miRNA>hsa-mir-890
P5_3 iso-pre-miRNA>hsa-mir-891a
P5_3 iso-pre-miRNA>hsa-mir-891b
P5_3 iso-pre-miRNA>hsa-mir-892c
P5_3 iso-pre-miRNA>hsa-mir-9-1
P5_3 iso-pre-miRNA>hsa-mir-9-2
P5_3 iso-pre-miRNA>hsa-mir-924
P5_3 iso-pre-miRNA>hsa-mir-92a-1

P5_3	iso-pre-miRNA>hsa-mir-92a-2	
P5_3	iso-pre-miRNA>hsa-mir-92b	
P5_3	iso-pre-miRNA>hsa-mir-93	
P5_3	iso-pre-miRNA>hsa-mir-9-3	
P5_3	iso-pre-miRNA>hsa-mir-934	
P5_3	iso-pre-miRNA>hsa-mir-936	
P5_3	iso-pre-miRNA>hsa-mir-937	
P5_3	iso-pre-miRNA>hsa-mir-938	
P5_3	iso-pre-miRNA>hsa-mir-939	
P5_3	iso-pre-miRNA>hsa-mir-942	
P5_3	iso-pre-miRNA>hsa-mir-95	
P5_3	iso-pre-miRNA>hsa-mir-96	
P5_3	iso-pre-miRNA>hsa-mir-98	
P5_3	iso-pre-miRNA>hsa-mir-99a	
P5_3	iso-SNP	568106
P5_3	iso-SNP	568125
P5_3	iso-SNP	568176
P5_3	iso-SNP	568187
P5_3	iso-SNP	925742
P5_3	iso-SNP	925755
P5_3	iso-SNP	925764
P5_3	iso-SNP	925772
P5_3	iso-SNP	925776
P5_3	iso-SNP	925781
P5_3	iso-SNP	1102498
P5_3	iso-SNP	1102501
P5_3	iso-SNP	1102563
P5_3	iso-SNP	1102567
P5_3	iso-SNP	1103312
P5_3	iso-SNP	1103328
P5_3	iso-SNP	1103331
P5_3	iso-SNP	1708902
P5_3	iso-SNP	1708983
P5_3	iso-SNP	1749333
P5_3	iso-SNP	1785015
P5_3	iso-SNP	1785030
P5_3	iso-SNP	1785037
P5_3	iso-SNP	1785038
P5_3	iso-SNP	1785042
P5_3	iso-SNP	1785060
P5_3	iso-SNP	1880764
P5_3	iso-SNP	1988193
P5_3	iso-SNP	2018002
P5_3	iso-SNP	2018004
P5_3	iso-SNP	2018019
P5_3	iso-SNP	2018056
P5_3	iso-SNP	2140204

P5_3	iso-SNP	2140240
P5_3	iso-SNP	2140267
P5_3	iso-SNP	2140268
P5_3	iso-SNP	2140269
P5_3	iso-SNP	2140270
P5_3	iso-SNP	2155379
P5_3	iso-SNP	2155409
P5_3	iso-SNP	2234086
P5_3	iso-SNP	2234093
P5_3	iso-SNP	2581955
P5_3	iso-SNP	2581984
P5_3	iso-SNP	2581992
P5_3	iso-SNP	2633434
P5_3	iso-SNP	2633462
P5_3	iso-SNP	2633478
P5_3	iso-SNP	2633480
P5_3	iso-SNP	2651377
P5_3	iso-SNP	2651389
P5_3	iso-SNP	2651398
P5_3	iso-SNP	2651405
P5_3	iso-SNP	2651455
P5_3	iso-SNP	4770697
P5_3	iso-SNP	4770709
P5_3	iso-SNP	4770747
P5_3	iso-SNP	4770789
P5_3	iso-SNP	6920955
P5_3	iso-SNP	6920975
P5_3	iso-SNP	6920976
P5_3	iso-SNP	6921289
P5_3	iso-SNP	7073282
P5_3	iso-SNP	7073301
P5_3	iso-SNP	7073344
P5_3	iso-SNP	7106600
P5_3	iso-SNP	7106636
P5_3	iso-SNP	7256001
P5_3	iso-SNP	7256013
P5_3	iso-SNP	7256057
P5_3	iso-SNP	8007037
P5_3	iso-SNP	8007039
P5_3	iso-SNP	8007066
P5_3	iso-SNP	8007067
P5_3	iso-SNP	8007104
P5_3	iso-SNP	10287820
P5_3	iso-SNP	10287821
P5_3	iso-SNP	10287824
P5_3	iso-SNP	10287854
P5_3	iso-SNP	10436180

P5_3	iso-SNP	10436187
P5_3	iso-SNP	10436194
P5_3	iso-SNP	10436201
P5_3	iso-SNP	10436219
P5_3	iso-SNP	10436238
P5_3	iso-SNP	10514157
P5_3	iso-SNP	10514159
P5_3	iso-SNP	10514190
P5_3	iso-SNP	10514194
P5_3	iso-SNP	10514200
P5_3	iso-SNP	10514202
P5_3	iso-SNP	10524531
P5_3	iso-SNP	10928119
P5_3	iso-SNP	10928130
P5_3	iso-SNP	10928149
P5_3	iso-SNP	11400303
P5_3	iso-SNP	11400324
P5_3	iso-SNP	11400356
P5_3	iso-SNP	11400358
P5_3	iso-SNP	11985275
P5_3	iso-SNP	12820632
P5_3	iso-SNP	12820646
P5_3	iso-SNP	12820658
P5_3	iso-SNP	12877500
P5_3	iso-SNP	12877501
P5_3	iso-SNP	12877502
P5_3	iso-SNP	12877520
P5_3	iso-SNP	12877525
P5_3	iso-SNP	12877527
P5_3	iso-SNP	13446846
P5_3	iso-SNP	13446848
P5_3	iso-SNP	13446849
P5_3	iso-SNP	13446893
P5_3	iso-SNP	13446906
P5_3	iso-SNP	13446924
P5_3	iso-SNP	13947292
P5_3	iso-SNP	13947296
P5_3	iso-SNP	13985514
P5_3	iso-SNP	13985721
P5_3	iso-SNP	13985739
P5_3	iso-SNP	13985772
P5_3	iso-SNP	13985805
P5_3	iso-SNP	13985806
P5_3	iso-SNP	14184227
P5_3	iso-SNP	14403144
P5_3	iso-SNP	14478575
P5_3	iso-SNP	14478576

P5_3	iso-SNP	14478613
P5_3	iso-SNP	14478618
P5_3	iso-SNP	14710999
P5_3	iso-SNP	14711013
P5_3	iso-SNP	14778711
P5_3	iso-SNP	14778721
P5_3	iso-SNP	14830172
P5_3	iso-SNP	14830197
P5_3	iso-SNP	14830201
P5_3	iso-SNP	14830215
P5_3	iso-SNP	14830216
P5_3	iso-SNP	15560380
P5_3	iso-SNP	15935316
P5_3	iso-SNP	15935347
P5_3	iso-SNP	15935348
P5_3	iso-SNP	15935351
P5_3	iso-SNP	16645137
P5_3	iso-SNP	16645178
P5_3	iso-SNP	16645199
P5_3	iso-SNP	16645208
P5_3	iso-SNP	16974725
P5_3	iso-SNP	16974739
P5_3	iso-SNP	17717209
P5_3	iso-SNP	17717243
P5_3	iso-SNP	17717244
P5_3	iso-SNP	17911447
P5_3	iso-SNP	17962615
P5_3	iso-SNP	17962644
P5_3	iso-SNP	18134042
P5_3	iso-SNP	18134045
P5_3	iso-SNP	18134091
P5_3	iso-SNP	18451300
P5_3	iso-SNP	18451325
P5_3	iso-SNP	18573331
P5_3	iso-SNP	18573360
P5_3	iso-SNP	18573361
P5_3	iso-SNP	18573374
P5_3	iso-SNP	20020733
P5_3	iso-SNP	20529956
P5_3	iso-SNP	20529990
P5_3	iso-SNP	20716104
P5_3	iso-SNP	20716128
P5_3	iso-SNP	22007594
P5_3	iso-SNP	22007634
P5_3	iso-SNP	23682350
P5_3	iso-SNP	23682383
P5_3	iso-SNP	23887219

P5_3	iso-SNP	23887220
P5_3	iso-SNP	23887271
P5_3	iso-SNP	24214442
P5_3	iso-SNP	24214486
P5_3	iso-SNP	24214493
P5_3	iso-SNP	24214532
P5_3	iso-SNP	24736590
P5_3	iso-SNP	24736638
P5_3	iso-SNP	26188825
P5_3	iso-SNP	26188865
P5_3	iso-SNP	26188880
P5_3	iso-SNP	26188906
P5_3	iso-SNP	26188908
P5_3	iso-SNP	26906402
P5_3	iso-SNP	26906403
P5_3	iso-SNP	26906423
P5_3	iso-SNP	26906437
P5_3	iso-SNP	26906452
P5_3	iso-SNP	26906471
P5_3	iso-SNP	27115438
P5_3	iso-SNP	27115444
P5_3	iso-SNP	27115447
P5_3	iso-SNP	27115458
P5_3	iso-SNP	27115467
P5_3	iso-SNP	27559214
P5_3	iso-SNP	27559261
P5_3	iso-SNP	28102427
P5_3	iso-SNP	28102477
P5_3	iso-SNP	28102484
P5_3	iso-SNP	28316513
P5_3	iso-SNP	28316591
P5_3	iso-SNP	28316592
P5_3	iso-SNP	28316597
P5_3	iso-SNP	28444104
P5_3	iso-SNP	28444157
P5_3	iso-SNP	28444183
P5_3	iso-SNP	28863628
P5_3	iso-SNP	28863633
P5_3	iso-SNP	28863695
P5_3	iso-SNP	28888922
P5_3	iso-SNP	29891235
P5_3	iso-SNP	29891245
P5_3	iso-SNP	29891260
P5_3	iso-SNP	29891262
P5_3	iso-SNP	29902536
P5_3	iso-SNP	30552168
P5_3	iso-SNP	30552187

P5_3	iso-SNP	31357238
P5_3	iso-SNP	31357244
P5_3	iso-SNP	31357245
P5_3	iso-SNP	31357301
P5_3	iso-SNP	31357325
P5_3	iso-SNP	31747678
P5_3	iso-SNP	31924708
P5_3	iso-SNP	32547795
P5_3	iso-SNP	32547809
P5_3	iso-SNP	32547810
P5_3	iso-SNP	32547811
P5_3	iso-SNP	32717702
P5_3	iso-SNP	32717722
P5_3	iso-SNP	33484783
P5_3	iso-SNP	33484837
P5_3	iso-SNP	33578205
P5_3	iso-SNP	33578206
P5_3	iso-SNP	33578251
P5_3	iso-SNP	33578255
P5_3	iso-SNP	33798007
P5_3	iso-SNP	33798031
P5_3	iso-SNP	33798091
P5_3	iso-SNP	33967787
P5_3	iso-SNP	33967796
P5_3	iso-SNP	33967800
P5_3	iso-SNP	33967806
P5_3	iso-SNP	34674326
P5_3	iso-SNP	34674345
P5_3	iso-SNP	34820547
P5_3	iso-SNP	34820566
P5_3	iso-SNP	35391051
P5_3	iso-SNP	35391065
P5_3	iso-SNP	35391066
P5_3	iso-SNP	35391105
P5_3	iso-SNP	36428006
P5_3	iso-SNP	36428017
P5_3	iso-SNP	36428046
P5_3	iso-SNP	36958963
P5_3	iso-SNP	36958995
P5_3	iso-SNP	36959006
P5_3	iso-SNP	36959015
P5_3	iso-SNP	37093013
P5_3	iso-SNP	37093078
P5_3	iso-SNP	37093089
P5_3	iso-SNP	37093097
P5_3	iso-SNP	37202092
P5_3	iso-SNP	37202112

P5_3	iso-SNP	37202113
P5_3	iso-SNP	37202138
P5_3	iso-SNP	37883165
P5_3	iso-SNP	37883195
P5_3	iso-SNP	37883200
P5_3	iso-SNP	38243727
P5_3	iso-SNP	38243739
P5_3	iso-SNP	38243743
P5_3	iso-SNP	38243770
P5_3	iso-SNP	39696856
P5_3	iso-SNP	39696863
P5_3	iso-SNP	40238175
P5_3	iso-SNP	40238258
P5_3	iso-SNP	40646803
P5_3	iso-SNP	40646816
P5_3	iso-SNP	40646834
P5_3	iso-SNP	41128578
P5_3	iso-SNP	41128599
P5_3	iso-SNP	41128620
P5_3	iso-SNP	41220077
P5_3	iso-SNP	41220094
P5_3	iso-SNP	41517974
P5_3	iso-SNP	41517981
P5_3	iso-SNP	41518005
P5_3	iso-SNP	41518007
P5_3	iso-SNP	41518025
P5_3	iso-SNP	41675171
P5_3	iso-SNP	42296995
P5_3	iso-SNP	42319269
P5_3	iso-SNP	42319276
P5_3	iso-SNP	42319291
P5_3	iso-SNP	43581287
P5_3	iso-SNP	43602984
P5_3	iso-SNP	43602992
P5_3	iso-SNP	44085868
P5_3	iso-SNP	44085909
P5_3	iso-SNP	44155749
P5_3	iso-SNP	44155754
P5_3	iso-SNP	44333749
P5_3	iso-SNP	45606471
P5_3	iso-SNP	45606472
P5_3	iso-SNP	45606504
P5_3	iso-SNP	45606510
P5_3	iso-SNP	45659490
P5_3	iso-SNP	45659500
P5_3	iso-SNP	46114580
P5_3	iso-SNP	46114610

P5_3	iso-SNP	46233806
P5_3	iso-SNP	46233848
P5_3	iso-SNP	46233866
P5_3	iso-SNP	46509569
P5_3	iso-SNP	46509616
P5_3	iso-SNP	46522190
P5_3	iso-SNP	46522201
P5_3	iso-SNP	46522255
P5_3	iso-SNP	46522298
P5_3	iso-SNP	46657254
P5_3	iso-SNP	46657289
P5_3	iso-SNP	47891069
P5_3	iso-SNP	47891106
P5_3	iso-SNP	47891117
P5_3	iso-SNP	48357853
P5_3	iso-SNP	48357864
P5_3	iso-SNP	48357921
P5_3	iso-SNP	49057623
P5_3	iso-SNP	49773042
P5_3	iso-SNP	49773087
P5_3	iso-SNP	49773090
P5_3	iso-SNP	49774381
P5_3	iso-SNP	49774383
P5_3	iso-SNP	49774389
P5_3	iso-SNP	49775296
P5_3	iso-SNP	49775337
P5_3	iso-SNP	49775351
P5_3	iso-SNP	49777907
P5_3	iso-SNP	49779210
P5_3	iso-SNP	49779214
P5_3	iso-SNP	49779234
P5_3	iso-SNP	49937076
P5_3	iso-SNP	49937087
P5_3	iso-SNP	50193625
P5_3	iso-SNP	52196528
P5_3	iso-SNP	52196574
P5_3	iso-SNP	52302043
P5_3	iso-SNP	52328248
P5_3	iso-SNP	52328298
P5_3	iso-SNP	53384209
P5_3	iso-SNP	53384256
P5_3	iso-SNP	53384262
P5_3	iso-SNP	53394424
P5_3	iso-SNP	53583294
P5_3	iso-SNP	54076326
P5_3	iso-SNP	54076332
P5_3	iso-SNP	54169954

P5_3	iso-SNP	54169973
P5_3	iso-SNP	54170006
P5_3	iso-SNP	54170016
P5_3	iso-SNP	54172483
P5_3	iso-SNP	54172501
P5_3	iso-SNP	54172508
P5_3	iso-SNP	54182261
P5_3	iso-SNP	54182325
P5_3	iso-SNP	54182326
P5_3	iso-SNP	54185441
P5_3	iso-SNP	54185457
P5_3	iso-SNP	54185481
P5_3	iso-SNP	54185492
P5_3	iso-SNP	54189751
P5_3	iso-SNP	54189752
P5_3	iso-SNP	54191743
P5_3	iso-SNP	54191790
P5_3	iso-SNP	54191794
P5_3	iso-SNP	54194135
P5_3	iso-SNP	54194212
P5_3	iso-SNP	54197660
P5_3	iso-SNP	54197674
P5_3	iso-SNP	54197678
P5_3	iso-SNP	54197706
P5_3	iso-SNP	54198496
P5_3	iso-SNP	54198499
P5_3	iso-SNP	54200810
P5_3	iso-SNP	54200830
P5_3	iso-SNP	54200853
P5_3	iso-SNP	54201654
P5_3	iso-SNP	54201667
P5_3	iso-SNP	54201668
P5_3	iso-SNP	54201692
P5_3	iso-SNP	54201695
P5_3	iso-SNP	54201703
P5_3	iso-SNP	54203326
P5_3	iso-SNP	54203333
P5_3	iso-SNP	54203347
P5_3	iso-SNP	54209517
P5_3	iso-SNP	54209527
P5_3	iso-SNP	54209563
P5_3	iso-SNP	54210734
P5_3	iso-SNP	54210736
P5_3	iso-SNP	54210774
P5_3	iso-SNP	54211990
P5_3	iso-SNP	54212075
P5_3	iso-SNP	54214286

P5_3	iso-SNP	54214312
P5_3	iso-SNP	54214333
P5_3	iso-SNP	54215584
P5_3	iso-SNP	54215608
P5_3	iso-SNP	54216615
P5_3	iso-SNP	54216616
P5_3	iso-SNP	54216629
P5_3	iso-SNP	54216650
P5_3	iso-SNP	54216670
P5_3	iso-SNP	54216681
P5_3	iso-SNP	54223379
P5_3	iso-SNP	54223433
P5_3	iso-SNP	54225426
P5_3	iso-SNP	54225437
P5_3	iso-SNP	54225460
P5_3	iso-SNP	54225463
P5_3	iso-SNP	54225490
P5_3	iso-SNP	54225501
P5_3	iso-SNP	54228719
P5_3	iso-SNP	54228742
P5_3	iso-SNP	54228743
P5_3	iso-SNP	54228750
P5_3	iso-SNP	54228774
P5_3	iso-SNP	54228775
P5_3	iso-SNP	54230219
P5_3	iso-SNP	54233092
P5_3	iso-SNP	54233093
P5_3	iso-SNP	54233109
P5_3	iso-SNP	54233112
P5_3	iso-SNP	54233113
P5_3	iso-SNP	54233170
P5_3	iso-SNP	54234260
P5_3	iso-SNP	54234265
P5_3	iso-SNP	54234315
P5_3	iso-SNP	54234340
P5_3	iso-SNP	54238159
P5_3	iso-SNP	54238182
P5_3	iso-SNP	54238189
P5_3	iso-SNP	54238203
P5_3	iso-SNP	54238208
P5_3	iso-SNP	54240136
P5_3	iso-SNP	54240137
P5_3	iso-SNP	54240142
P5_3	iso-SNP	54240174
P5_3	iso-SNP	54240184
P5_3	iso-SNP	54242630
P5_3	iso-SNP	54244647

P5_3	iso-SNP	54254494
P5_3	iso-SNP	54254543
P5_3	iso-SNP	54255679
P5_3	iso-SNP	54255689
P5_3	iso-SNP	54257304
P5_3	iso-SNP	54257325
P5_3	iso-SNP	54260002
P5_3	iso-SNP	54260009
P5_3	iso-SNP	54260068
P5_3	iso-SNP	54260075
P5_3	iso-SNP	54261549
P5_3	iso-SNP	54261556
P5_3	iso-SNP	54261562
P5_3	iso-SNP	54261563
P5_3	iso-SNP	54264394
P5_3	iso-SNP	54264421
P5_3	iso-SNP	54264461
P5_3	iso-SNP	54264462
P5_3	iso-SNP	54264468
P5_3	iso-SNP	54290994
P5_3	iso-SNP	54290995
P5_3	iso-SNP	54291161
P5_3	iso-SNP	54291965
P5_3	iso-SNP	54292016
P5_3	iso-SNP	54385558
P5_3	iso-SNP	54385561
P5_3	iso-SNP	54385584
P5_3	iso-SNP	54385599
P5_3	iso-SNP	54385629
P5_3	iso-SNP	54466527
P5_3	iso-SNP	54466544
P5_3	iso-SNP	54468094
P5_3	iso-SNP	54468110
P5_3	iso-SNP	54468124
P5_3	iso-SNP	54468144
P5_3	iso-SNP	54468150
P5_3	iso-SNP	54468166
P5_3	iso-SNP	54731000
P5_3	iso-SNP	54731071
P5_3	iso-SNP	54731080
P5_3	iso-SNP	56408599
P5_3	iso-SNP	56408625
P5_3	iso-SNP	56408638
P5_3	iso-SNP	56408639
P5_3	iso-SNP	56892431
P5_3	iso-SNP	56892464
P5_3	iso-SNP	56892470

P5_3	iso-SNP	56892507
P5_3	iso-SNP	57023497
P5_3	iso-SNP	57215120
P5_3	iso-SNP	57215164
P5_3	iso-SNP	57228573
P5_3	iso-SNP	57228574
P5_3	iso-SNP	57392678
P5_3	iso-SNP	57392686
P5_3	iso-SNP	57392697
P5_3	iso-SNP	57392715
P5_3	iso-SNP	57588322
P5_3	iso-SNP	57588323
P5_3	iso-SNP	57588336
P5_3	iso-SNP	59139674
P5_3	iso-SNP	59362576
P5_3	iso-SNP	59362602
P5_3	iso-SNP	59463389
P5_3	iso-SNP	59463436
P5_3	iso-SNP	59463451
P5_3	iso-SNP	59463460
P5_3	iso-SNP	60528617
P5_3	iso-SNP	60528670
P5_3	iso-SNP	60639883
P5_3	iso-SNP	61582649
P5_3	iso-SNP	61582659
P5_3	iso-SNP	61582708
P5_3	iso-SNP	61809907
P5_3	iso-SNP	62496924
P5_3	iso-SNP	62496926
P5_3	iso-SNP	62572847
P5_3	iso-SNP	62572874
P5_3	iso-SNP	62997470
P5_3	iso-SNP	63081542
P5_3	iso-SNP	64136103
P5_3	iso-SNP	64136130
P5_3	iso-SNP	64136145
P5_3	iso-SNP	64136160
P5_3	iso-SNP	64136167
P5_3	iso-SNP	64136170
P5_3	iso-SNP	64561772
P5_3	iso-SNP	64561812
P5_3	iso-SNP	64658623
P5_3	iso-SNP	64658640
P5_3	iso-SNP	64658705
P5_3	iso-SNP	64658710
P5_3	iso-SNP	64658715
P5_3	iso-SNP	64658716

P5_3	iso-SNP	64658828
P5_3	iso-SNP	64658836
P5_3	iso-SNP	64658904
P5_3	iso-SNP	65016300
P5_3	iso-SNP	65016325
P5_3	iso-SNP	65054606
P5_3	iso-SNP	65054649
P5_3	iso-SNP	65054668
P5_3	iso-SNP	65054709
P5_3	iso-SNP	65238733
P5_3	iso-SNP	65238751
P5_3	iso-SNP	65238767
P5_3	iso-SNP	65238773
P5_3	iso-SNP	65238806
P5_3	iso-SNP	65291712
P5_3	iso-SNP	65291793
P5_3	iso-SNP	65291808
P5_3	iso-SNP	65467665
P5_3	iso-SNP	65467669
P5_3	iso-SNP	65467701
P5_3	iso-SNP	67094145
P5_3	iso-SNP	67094150
P5_3	iso-SNP	67094171
P5_3	iso-SNP	67236292
P5_3	iso-SNP	68649205
P5_3	iso-SNP	68649258
P5_3	iso-SNP	68649260
P5_3	iso-SNP	68649290
P5_3	iso-SNP	69242707
P5_3	iso-SNP	69242767
P5_3	iso-SNP	69330818
P5_3	iso-SNP	69330823
P5_3	iso-SNP	69330824
P5_3	iso-SNP	69330825
P5_3	iso-SNP	69330871
P5_3	iso-SNP	69330886
P5_3	iso-SNP	69666943
P5_3	iso-SNP	69666969
P5_3	iso-SNP	69666979
P5_3	iso-SNP	69966985
P5_3	iso-SNP	69966994
P5_3	iso-SNP	69967005
P5_3	iso-SNP	69967062
P5_3	iso-SNP	70519078
P5_3	iso-SNP	70519093
P5_3	iso-SNP	70519169
P5_3	iso-SNP	73402156

P5_3	iso-SNP	77732586
P5_3	iso-SNP	77732603
P5_3	iso-SNP	77732628
P5_3	iso-SNP	79099712
P5_3	iso-SNP	79099736
P5_3	iso-SNP	79099750
P5_3	iso-SNP	79113119
P5_3	iso-SNP	79418149
P5_3	iso-SNP	79813049
P5_3	iso-SNP	79813075
P5_3	iso-SNP	79813083
P5_3	iso-SNP	83541972
P5_3	iso-SNP	83541979
P5_3	iso-SNP	83541980
P5_3	iso-SNP	83541990
P5_3	iso-SNP	85775260
P5_3	iso-SNP	85775275
P5_3	iso-SNP	85916322
P5_3	iso-SNP	86313731
P5_3	iso-SNP	86313782
P5_3	iso-SNP	86313794
P5_3	iso-SNP	86368890
P5_3	iso-SNP	86368898
P5_3	iso-SNP	86368922
P5_3	iso-SNP	86368929
P5_3	iso-SNP	86368959
P5_3	iso-SNP	86410766
P5_3	iso-SNP	86584675
P5_3	iso-SNP	86584707
P5_3	iso-SNP	86584720
P5_3	iso-SNP	87962747
P5_3	iso-SNP	89151351
P5_3	iso-SNP	89151354
P5_3	iso-SNP	89151411
P5_3	iso-SNP	89155064
P5_3	iso-SNP	89155073
P5_3	iso-SNP	89155084
P5_3	iso-SNP	89155121
P5_3	iso-SNP	89155162
P5_3	iso-SNP	89155163
P5_3	iso-SNP	89911256
P5_3	iso-SNP	90602296
P5_3	iso-SNP	90602368
P5_3	iso-SNP	91360757
P5_3	iso-SNP	92002896
P5_3	iso-SNP	92003009
P5_3	iso-SNP	92003356

P5_3	iso-SNP	92003588
P5_3	iso-SNP	92003589
P5_3	iso-SNP	92956409
P5_3	iso-SNP	92956412
P5_3	iso-SNP	92956416
P5_3	iso-SNP	92956420
P5_3	iso-SNP	92956422
P5_3	iso-SNP	92956443
P5_3	iso-SNP	93113270
P5_3	iso-SNP	93113314
P5_3	iso-SNP	93113323
P5_3	iso-SNP	93113326
P5_3	iso-SNP	93447631
P5_3	iso-SNP	93447646
P5_3	iso-SNP	93447674
P5_3	iso-SNP	93447695
P5_3	iso-SNP	93447700
P5_3	iso-SNP	93447702
P5_3	iso-SNP	93466866
P5_3	iso-SNP	93466910
P5_3	iso-SNP	93466912
P5_3	iso-SNP	93466919
P5_3	iso-SNP	94928250
P5_3	iso-SNP	94928319
P5_3	iso-SNP	95228179
P5_3	iso-SNP	95228209
P5_3	iso-SNP	95228286
P5_3	iso-SNP	96074607
P5_3	iso-SNP	96074638
P5_3	iso-SNP	96074649
P5_3	iso-SNP	96085190
P5_3	iso-SNP	97572244
P5_3	iso-SNP	97847807
P5_3	iso-SNP	97885708
P5_3	iso-SNP	97885715
P5_3	iso-SNP	97885720
P5_3	iso-SNP	97885730
P5_3	iso-SNP	98510847
P5_3	iso-SNP	98510864
P5_3	iso-SNP	98510896
P5_3	iso-SNP	98510902
P5_3	iso-SNP	98860801
P5_3	iso-SNP	98860808
P5_3	iso-SNP	98860816
P5_3	iso-SNP	98860827
P5_3	iso-SNP	98860840
P5_3	iso-SNP	99572563

P5_3	iso-SNP	99691200
P5_3	iso-SNP	99691393
P5_3	iso-SNP	99691396
P5_3	iso-SNP	99691401
P5_3	iso-SNP	99691429
P5_3	iso-SNP	99691652
P5_3	iso-SNP	99691653
P5_3	iso-SNP	100154978
P5_3	iso-SNP	100155019
P5_3	iso-SNP	100155033
P5_3	iso-SNP	100576035
P5_3	iso-SNP	100576059
P5_3	iso-SNP	101318740
P5_3	iso-SNP	101318783
P5_3	iso-SNP	101318784
P5_3	iso-SNP	101318807
P5_3	iso-SNP	101347355
P5_3	iso-SNP	101347408
P5_3	iso-SNP	101348275
P5_3	iso-SNP	101350873
P5_3	iso-SNP	101377478
P5_3	iso-SNP	101488414
P5_3	iso-SNP	101488424
P5_3	iso-SNP	101488430
P5_3	iso-SNP	101488431
P5_3	iso-SNP	101489677
P5_3	iso-SNP	101489685
P5_3	iso-SNP	101489703
P5_3	iso-SNP	101489714
P5_3	iso-SNP	101489728
P5_3	iso-SNP	101489745
P5_3	iso-SNP	101492444
P5_3	iso-SNP	101493129
P5_3	iso-SNP	101496416
P5_3	iso-SNP	101500098
P5_3	iso-SNP	101500126
P5_3	iso-SNP	101500133
P5_3	iso-SNP	101500166
P5_3	iso-SNP	101500167
P5_3	iso-SNP	101506441
P5_3	iso-SNP	101506465
P5_3	iso-SNP	101509363
P5_3	iso-SNP	101510579
P5_3	iso-SNP	101510612
P5_3	iso-SNP	101510613
P5_3	iso-SNP	101512288
P5_3	iso-SNP	101512304

P5_3	iso-SNP	101512836
P5_3	iso-SNP	101518827
P5_3	iso-SNP	101520657
P5_3	iso-SNP	101520687
P5_3	iso-SNP	101521052
P5_3	iso-SNP	101521092
P5_3	iso-SNP	101521608
P5_3	iso-SNP	101521620
P5_3	iso-SNP	101521764
P5_3	iso-SNP	101522556
P5_3	iso-SNP	101522578
P5_3	iso-SNP	101522582
P5_3	iso-SNP	101522589
P5_3	iso-SNP	101522631
P5_3	iso-SNP	101528401
P5_3	iso-SNP	101530833
P5_3	iso-SNP	101530834
P5_3	iso-SNP	101530873
P5_3	iso-SNP	101531806
P5_3	iso-SNP	101531849
P5_3	iso-SNP	101531854
P5_3	iso-SNP	101531857
P5_3	iso-SNP	101531858
P5_3	iso-SNP	101531862
P5_3	iso-SNP	101532279
P5_3	iso-SNP	102251501
P5_3	iso-SNP	102251532
P5_3	iso-SNP	102251542
P5_3	iso-SNP	102619995
P5_3	iso-SNP	102620081
P5_3	iso-SNP	103006026
P5_3	iso-SNP	103006047
P5_3	iso-SNP	103242937
P5_3	iso-SNP	103242954
P5_3	iso-SNP	104166899
P5_3	iso-SNP	104166902
P5_3	iso-SNP	104166904
P5_3	iso-SNP	104324231
P5_3	iso-SNP	104324238
P5_3	iso-SNP	104324266
P5_3	iso-SNP	104583759
P5_3	iso-SNP	104583804
P5_3	iso-SNP	104985443
P5_3	iso-SNP	105154013
P5_3	iso-SNP	105154084
P5_3	iso-SNP	105154089
P5_3	iso-SNP	105154091

P5_3	iso-SNP	105154097
P5_3	iso-SNP	105496622
P5_3	iso-SNP	105496638
P5_3	iso-SNP	105496675
P5_3	iso-SNP	105807855
P5_3	iso-SNP	105807858
P5_3	iso-SNP	105807864
P5_3	iso-SNP	105807909
P5_3	iso-SNP	105807912
P5_3	iso-SNP	105807928
P5_3	iso-SNP	105883051
P5_3	iso-SNP	105883081
P5_3	iso-SNP	105883120
P5_3	iso-SNP	110141523
P5_3	iso-SNP	110141578
P5_3	iso-SNP	110827551
P5_3	iso-SNP	110827558
P5_3	iso-SNP	113320747
P5_3	iso-SNP	113569193
P5_3	iso-SNP	113569207
P5_3	iso-SNP	113569211
P5_3	iso-SNP	113569394
P5_3	iso-SNP	113569519
P5_3	iso-SNP	113569680
P5_3	iso-SNP	113569692
P5_3	iso-SNP	113569695
P5_3	iso-SNP	113569696
P5_3	iso-SNP	113997746
P5_3	iso-SNP	113997768
P5_3	iso-SNP	113997812
P5_3	iso-SNP	113997817
P5_3	iso-SNP	114028104
P5_3	iso-SNP	115933876
P5_3	iso-SNP	115933913
P5_3	iso-SNP	115933922
P5_3	iso-SNP	117637290
P5_3	iso-SNP	117637302
P5_3	iso-SNP	117637325
P5_3	iso-SNP	117637326
P5_3	iso-SNP	118780738
P5_3	iso-SNP	118780790
P5_3	iso-SNP	118927256
P5_3	iso-SNP	119390263
P5_3	iso-SNP	119390286
P5_3	iso-SNP	119390294
P5_3	iso-SNP	120114558
P5_3	iso-SNP	120151493

P5_3	iso-SNP	120151501
P5_3	iso-SNP	120151527
P5_3	iso-SNP	120336327
P5_3	iso-SNP	120336365
P5_3	iso-SNP	120336384
P5_3	iso-SNP	124021017
P5_3	iso-SNP	124021033
P5_3	iso-SNP	124021052
P5_3	iso-SNP	124021054
P5_3	iso-SNP	124360347
P5_3	iso-SNP	126721357
P5_3	iso-SNP	126721419
P5_3	iso-SNP	127847960
P5_3	iso-SNP	128778703
P5_3	iso-SNP	128808252
P5_3	iso-SNP	128808253
P5_3	iso-SNP	128972923
P5_3	iso-SNP	128972925
P5_3	iso-SNP	128972936
P5_3	iso-SNP	129061408
P5_3	iso-SNP	129061422
P5_3	iso-SNP	129061441
P5_3	iso-SNP	129061462
P5_3	iso-SNP	129162366
P5_3	iso-SNP	129162390
P5_3	iso-SNP	129162428
P5_3	iso-SNP	129162433
P5_3	iso-SNP	129414553
P5_3	iso-SNP	129414568
P5_3	iso-SNP	129414574
P5_3	iso-SNP	129414804
P5_3	iso-SNP	129414806
P5_3	iso-SNP	129414843
P5_3	iso-SNP	131007001
P5_3	iso-SNP	131007004
P5_3	iso-SNP	131007052
P5_3	iso-SNP	131007087
P5_3	iso-SNP	131007109
P5_3	iso-SNP	131154943
P5_3	iso-SNP	131641629
P5_3	iso-SNP	132113371
P5_3	iso-SNP	132760893
P5_3	iso-SNP	132760931
P5_3	iso-SNP	133303425
P5_3	iso-SNP	133303465
P5_3	iso-SNP	133303579
P5_3	iso-SNP	133303744

P5_3	iso-SNP	133303758
P5_3	iso-SNP	133303896
P5_3	iso-SNP	133680381
P5_3	iso-SNP	134884697
P5_3	iso-SNP	134884700
P5_3	iso-SNP	134884717
P5_3	iso-SNP	134884723
P5_3	iso-SNP	134884737
P5_3	iso-SNP	135061039
P5_3	iso-SNP	135061109
P5_3	iso-SNP	135061111
P5_3	iso-SNP	135061124
P5_3	iso-SNP	135633045
P5_3	iso-SNP	135633077
P5_3	iso-SNP	135821135
P5_3	iso-SNP	136422988
P5_3	iso-SNP	136423009
P5_3	iso-SNP	136587919
P5_3	iso-SNP	136587934
P5_3	iso-SNP	136588019
P5_3	iso-SNP	136983275
P5_3	iso-SNP	136983281
P5_3	iso-SNP	136983319
P5_3	iso-SNP	136983326
P5_3	iso-SNP	137749929
P5_3	iso-SNP	138756369
P5_3	iso-SNP	138756428
P5_3	iso-SNP	139565064
P5_3	iso-SNP	139565098
P5_3	iso-SNP	139565119
P5_3	iso-SNP	139565134
P5_3	iso-SNP	144264666
P5_3	iso-SNP	144264667
P5_3	iso-SNP	144895159
P5_3	iso-SNP	144895164
P5_3	iso-SNP	144895168
P5_3	iso-SNP	144895169
P5_3	iso-SNP	144895170
P5_3	iso-SNP	144895179
P5_3	iso-SNP	145074272
P5_3	iso-SNP	145074283
P5_3	iso-SNP	145074284
P5_3	iso-SNP	145074289
P5_3	iso-SNP	145074342
P5_3	iso-SNP	145075804
P5_3	iso-SNP	145075848
P5_3	iso-SNP	145076302

P5_3	iso-SNP	145076355
P5_3	iso-SNP	145076356
P5_3	iso-SNP	145076376
P5_3	iso-SNP	145082615
P5_3	iso-SNP	145109328
P5_3	iso-SNP	145109356
P5_3	iso-SNP	145619365
P5_3	iso-SNP	145619377
P5_3	iso-SNP	145619386
P5_3	iso-SNP	145619405
P5_3	iso-SNP	145619442
P5_3	iso-SNP	146295067
P5_3	iso-SNP	146295085
P5_3	iso-SNP	146307375
P5_3	iso-SNP	146307411
P5_3	iso-SNP	146307414
P5_3	iso-SNP	146307449
P5_3	iso-SNP	146318448
P5_3	iso-SNP	146318507
P5_3	iso-SNP	146340278
P5_3	iso-SNP	146340304
P5_3	iso-SNP	146340339
P5_3	iso-SNP	146340360
P5_3	iso-SNP	146341217
P5_3	iso-SNP	146342052
P5_3	iso-SNP	146342071
P5_3	iso-SNP	146342080
P5_3	iso-SNP	146360765
P5_3	iso-SNP	146360778
P5_3	iso-SNP	146360779
P5_3	iso-SNP	146360813
P5_3	iso-SNP	146360826
P5_3	iso-SNP	146360845
P5_3	iso-SNP	148265864
P5_3	iso-SNP	148808487
P5_3	iso-SNP	148808575
P5_3	iso-SNP	149112399
P5_3	iso-SNP	150935522
P5_3	iso-SNP	150935577
P5_3	iso-SNP	150935583
P5_3	iso-SNP	151128173
P5_3	iso-SNP	151560699
P5_3	iso-SNP	151560719
P5_3	iso-SNP	151561912
P5_3	iso-SNP	151562938
P5_3	iso-SNP	153410482
P5_3	iso-SNP	153410520

P5_3	iso-SNP	153410531
P5_3	iso-SNP	153975576
P5_3	iso-SNP	153975613
P5_3	iso-SNP	153996899
P5_3	iso-SNP	153996903
P5_3	iso-SNP	153996905
P5_3	iso-SNP	154166183
P5_3	iso-SNP	154209024
P5_3	iso-SNP	154948195
P5_3	iso-SNP	154948201
P5_3	iso-SNP	155165044
P5_3	iso-SNP	156267955
P5_3	iso-SNP	156267978
P5_3	iso-SNP	156390147
P5_3	iso-SNP	157367104
P5_3	iso-SNP	159912418
P5_3	iso-SNP	159912436
P5_3	iso-SNP	160122421
P5_3	iso-SNP	160122434
P5_3	iso-SNP	168195174
P5_3	iso-SNP	170120566
P5_3	iso-SNP	170120575
P5_3	iso-SNP	170120594
P5_3	iso-SNP	170813684
P5_3	iso-SNP	171070937
P5_3	iso-SNP	172113756
P5_3	iso-SNP	177015045
P5_3	iso-SNP	177465775
P5_3	iso-SNP	178120681
P5_3	iso-SNP	179225301
P5_3	iso-SNP	179225317
P5_3	iso-SNP	179225324
P5_3	iso-SNP	179442344
P5_3	iso-SNP	183959219
P5_3	iso-SNP	183959222
P5_3	iso-SNP	183959229
P5_3	iso-SNP	183959232
P5_3	iso-SNP	185772247
P5_3	iso-SNP	186504477
P5_3	iso-SNP	186504499
P5_3	iso-SNP	186504532
P5_3	iso-SNP	186504537
P5_3	iso-SNP	189842822
P5_3	iso-SNP	189842826
P5_3	iso-SNP	189842885
P5_3	iso-SNP	189997816
P5_3	iso-SNP	189997834

P5_3	iso-SNP	198828005
P5_3	iso-SNP	198828096
P5_3	iso-SNP	201777741
P5_3	iso-SNP	201777751
P5_3	iso-SNP	201777752
P5_3	iso-SNP	201777753
P5_3	iso-SNP	201777819
P5_3	iso-SNP	207974738
P5_3	iso-SNP	207974741
P5_3	iso-SNP	207974756
P5_3	iso-SNP	209796810
P5_3	iso-SNP	219267370
P5_3	iso-SNP	219267371
P5_3	iso-SNP	219267402
P5_3	iso-SNP	219267407
P5_3	iso-SNP	219267433
P5_3	iso-SNP	219267443
P5_3	iso-SNP	219923411
P5_3	iso-SNP	219923418
P5_3	iso-SNP	219923421
P5_3	iso-SNP	220291206
P5_3	iso-SNP	220291292
P5_3	iso-SNP	220291302
P5_3	iso-SNP	220291502
P5_3	iso-SNP	220291527
P5_3	iso-SNP	220373922
P5_3	iso-SNP	220373933
P5_3	iso-SNP	220373934
P5_3	iso-SNP	220373939
P5_3	iso-SNP	220373943
P5_3	iso-SNP	220413807
P5_3	iso-SNP	220413812
P5_3	iso-SNP	220413813
P5_3	iso-SNP	228284991
P5_3	iso-SNP	240227178
P5_3	iso-SNP	241395420
P5_3	iso-SNP	241395500
P5_3	iso-SNP	241395503
P5_3	iso-SNP	247365286
P5_3	iso-SNP	249120578
P5_3	iso-SNP	249120584
P5_3	iso-SNP	249120591
P5_3	iso-SNP	249120610
P5_3	iso-SNP	249120631
P5_3	iso-SNP	249120640
P3_5	nor-pre-miRNA>hsa-mir-106b	
P3_5	nor-pre-miRNA>hsa-mir-10a	

P3_5 nor-pre-miRNA>hsa-mir-1180
P3_5 nor-pre-miRNA>hsa-mir-124-2
P3_5 nor-pre-miRNA>hsa-mir-124-3
P3_5 nor-pre-miRNA>hsa-mir-1249
P3_5 nor-pre-miRNA>hsa-mir-1264
P3_5 nor-pre-miRNA>hsa-mir-1285-2
P3_5 nor-pre-miRNA>hsa-mir-1302-3
P3_5 nor-pre-miRNA>hsa-mir-1303
P3_5 nor-pre-miRNA>hsa-mir-1304
P3_5 nor-pre-miRNA>hsa-mir-130b
P3_5 nor-pre-miRNA>hsa-mir-1343
P3_5 nor-pre-miRNA>hsa-mir-140
P3_5 nor-pre-miRNA>hsa-mir-143
P3_5 nor-pre-miRNA>hsa-mir-148b
P3_5 nor-pre-miRNA>hsa-mir-1537
P3_5 nor-pre-miRNA>hsa-mir-16-1
P3_5 nor-pre-miRNA>hsa-mir-181c
P3_5 nor-pre-miRNA>hsa-mir-182
P3_5 nor-pre-miRNA>hsa-mir-183
P3_5 nor-pre-miRNA>hsa-mir-1912
P3_5 nor-pre-miRNA>hsa-mir-196b
P3_5 nor-pre-miRNA>hsa-mir-1972-2
P3_5 nor-pre-miRNA>hsa-mir-210
P3_5 nor-pre-miRNA>hsa-mir-2113
P3_5 nor-pre-miRNA>hsa-mir-2115
P3_5 nor-pre-miRNA>hsa-mir-215
P3_5 nor-pre-miRNA>hsa-mir-23b
P3_5 nor-pre-miRNA>hsa-mir-24-1
P3_5 nor-pre-miRNA>hsa-mir-2467
P3_5 nor-pre-miRNA>hsa-mir-2681
P3_5 nor-pre-miRNA>hsa-mir-2682
P3_5 nor-pre-miRNA>hsa-mir-296
P3_5 nor-pre-miRNA>hsa-mir-299
P3_5 nor-pre-miRNA>hsa-mir-302f
P3_5 nor-pre-miRNA>hsa-mir-30c-2
P3_5 nor-pre-miRNA>hsa-mir-3116-1
P3_5 nor-pre-miRNA>hsa-mir-3117
P3_5 nor-pre-miRNA>hsa-mir-3130-1
P3_5 nor-pre-miRNA>hsa-mir-3130-2
P3_5 nor-pre-miRNA>hsa-mir-3144
P3_5 nor-pre-miRNA>hsa-mir-329-2
P3_5 nor-pre-miRNA>hsa-mir-361
P3_5 nor-pre-miRNA>hsa-mir-3619
P3_5 nor-pre-miRNA>hsa-mir-3667
P3_5 nor-pre-miRNA>hsa-mir-373
P3_5 nor-pre-miRNA>hsa-mir-3910-1
P3_5 nor-pre-miRNA>hsa-mir-3928

P3_5 nor-pre-miRNA>hsa-mir-4253
P3_5 nor-pre-miRNA>hsa-mir-4281
P3_5 nor-pre-miRNA>hsa-mir-429
P3_5 nor-pre-miRNA>hsa-mir-449b
P3_5 nor-pre-miRNA>hsa-mir-455
P3_5 nor-pre-miRNA>hsa-mir-500a
P3_5 nor-pre-miRNA>hsa-mir-500b
P3_5 nor-pre-miRNA>hsa-mir-507
P3_5 nor-pre-miRNA>hsa-mir-508
P3_5 nor-pre-miRNA>hsa-mir-513a-1
P3_5 nor-pre-miRNA>hsa-mir-513a-2
P3_5 nor-pre-miRNA>hsa-mir-532
P3_5 nor-pre-miRNA>hsa-mir-544a
P3_5 nor-pre-miRNA>hsa-mir-548b
P3_5 nor-pre-miRNA>hsa-mir-548d-1
P3_5 nor-pre-miRNA>hsa-mir-664a
P3_5 nor-pre-miRNA>hsa-mir-888
P3_5 nor-pre-miRNA>hsa-mir-892c
P3_5 nor-pre-miRNA>hsa-mir-933
P3_5 nor-pre-miRNA>hsa-mir-769
P3_5 nor-pre-miRNA>hsa-mir-1224
P3_5 nor-pre-miRNA>hsa-mir-96
P3_5 nor-pre-miRNA>hsa-mir-135b
P3_5 nor-pre-miRNA>hsa-mir-3609
P3_5 nor-pre-miRNA>hsa-mir-197
P3_5 nor-pre-miRNA>hsa-mir-302c
P3_5 nor-pre-miRNA>hsa-mir-326
P3_5 nor-pre-miRNA>hsa-mir-3679
P3_5 nor-pre-miRNA>hsa-mir-1207
P3_5 nor-pre-miRNA>hsa-mir-1295b
P3_5 nor-pre-miRNA>hsa-mir-133b
P3_5 nor-pre-miRNA>hsa-mir-3607
P3_5 nor-pre-miRNA>hsa-mir-380
P3_5 nor-pre-miRNA>hsa-mir-449c
P3_5 nor-pre-miRNA>hsa-mir-1199
P3_5 nor-pre-miRNA>hsa-mir-188
P3_5 nor-pre-miRNA>hsa-mir-3186
P3_5 nor-pre-miRNA>hsa-mir-329-1
P3_5 nor-pre-miRNA>hsa-mir-3622b
P3_5 nor-pre-miRNA>hsa-mir-3682
P3_5 nor-pre-miRNA>hsa-mir-377
P3_5 nor-pre-miRNA>hsa-mir-3924
P3_5 nor-pre-miRNA>hsa-mir-411
P3_5 nor-pre-miRNA>hsa-mir-4278
P3_5 nor-pre-miRNA>hsa-mir-659
P3_5 nor-pre-miRNA>hsa-mir-1182
P3_5 nor-pre-miRNA>hsa-mir-1185-1

P3_5 nor-pre-miRNA>hsa-mir-1297
P3_5 nor-pre-miRNA>hsa-mir-18a
P3_5 nor-pre-miRNA>hsa-mir-192
P3_5 nor-pre-miRNA>hsa-mir-3074
P3_5 nor-pre-miRNA>hsa-mir-3127
P3_5 nor-pre-miRNA>hsa-mir-3153
P3_5 nor-pre-miRNA>hsa-mir-3192
P3_5 nor-pre-miRNA>hsa-mir-3910-2
P3_5 nor-pre-miRNA>hsa-mir-3938
P3_5 nor-pre-miRNA>hsa-mir-4321
P3_5 nor-pre-miRNA>hsa-mir-1178
P3_5 nor-pre-miRNA>hsa-mir-1236
P3_5 nor-pre-miRNA>hsa-mir-1252
P3_5 nor-pre-miRNA>hsa-mir-1538
P3_5 nor-pre-miRNA>hsa-mir-1914
P3_5 nor-pre-miRNA>hsa-mir-208b
P3_5 nor-pre-miRNA>hsa-mir-223
P3_5 nor-pre-miRNA>hsa-mir-3124
P3_5 nor-pre-miRNA>hsa-mir-3662
P3_5 nor-pre-miRNA>hsa-mir-3909
P3_5 nor-pre-miRNA>hsa-mir-548a-1
P3_5 nor-pre-miRNA>hsa-mir-548aa-1
P3_5 nor-pre-miRNA>hsa-mir-3615
P3_5 nor-pre-miRNA>hsa-mir-518e
P3_5 nor-pre-miRNA>hsa-mir-383
P3_5 nor-pre-miRNA>hsa-mir-520g
P3_5 nor-pre-miRNA>hsa-mir-520h
P3_5 nor-pre-miRNA>hsa-mir-545
P3_5 nor-pre-miRNA>hsa-mir-548g
P3_5 nor-pre-miRNA>hsa-mir-302b
P3_5 nor-pre-miRNA>hsa-mir-942
P3_5 nor-pre-miRNA>hsa-mir-92a-1
P3_5 nor-pre-miRNA>hsa-mir-3142
P3_5 nor-pre-miRNA>hsa-mir-4293
P3_5 nor-pre-miRNA>hsa-mir-4274
P3_5 nor-pre-miRNA>hsa-mir-122
P3_5 nor-pre-miRNA>hsa-mir-3064
P3_5 nor-pre-miRNA>hsa-mir-3121
P3_5 nor-pre-miRNA>hsa-mir-1915
P3_5 nor-pre-miRNA>hsa-mir-3188
P3_5 nor-pre-miRNA>hsa-mir-3176
P3_5 nor-pre-miRNA>hsa-let-7a-2
P3_5 nor-pre-miRNA>hsa-mir-203b
P3_5 nor-pre-miRNA>hsa-mir-362
P3_5 nor-pre-miRNA>hsa-mir-1-1
P3_5 nor-pre-miRNA>hsa-mir-1185-2
P3_5 nor-pre-miRNA>hsa-mir-514a-1

P3_5 nor-pre-miRNA>hsa-mir-514a-3
P3_5 nor-pre-miRNA>hsa-mir-154
P3_5 nor-pre-miRNA>hsa-mir-1278
P3_5 nor-pre-miRNA>hsa-mir-3152
P3_5 nor-pre-miRNA>hsa-mir-3154
P3_5 nor-pre-miRNA>hsa-mir-432
P3_5 nor-pre-miRNA>hsa-mir-141
P3_5 nor-pre-miRNA>hsa-mir-525
P3_5 nor-pre-miRNA>hsa-mir-26a-1
P3_5 nor-pre-miRNA>hsa-mir-26b
P3_5 nor-pre-miRNA>hsa-mir-506
P3_5 nor-pre-miRNA>hsa-mir-24-2
P3_5 nor-pre-miRNA>hsa-mir-324
P3_5 nor-pre-miRNA>hsa-mir-339
P3_5 nor-pre-miRNA>hsa-mir-3617
P3_5 nor-pre-miRNA>hsa-mir-4320
P3_5 nor-pre-miRNA>hsa-mir-519d
P3_5 nor-pre-miRNA>hsa-mir-548a-3
P3_5 nor-pre-miRNA>hsa-mir-548am
P3_5 nor-pre-miRNA>hsa-mir-548f-2
P3_5 nor-pre-miRNA>hsa-mir-664b
P3_5 nor-pre-miRNA>hsa-mir-93
P3_5 nor-pre-miRNA>hsa-mir-523
P3_5 nor-pre-miRNA>hsa-mir-943
P3_5 nor-pre-miRNA>hsa-mir-342
P3_5 nor-pre-miRNA>hsa-mir-3134
P3_5 nor-pre-miRNA>hsa-mir-515-1
P3_5 nor-pre-miRNA>hsa-mir-4279
P3_5 nor-pre-miRNA>hsa-mir-367
P3_5 nor-pre-miRNA>hsa-mir-518d
P3_5 nor-pre-miRNA>hsa-mir-133a-1
P3_5 nor-pre-miRNA>hsa-mir-3126
P3_5 nor-pre-miRNA>hsa-mir-675
P3_5 nor-pre-miRNA>hsa-mir-502
P3_5 nor-pre-miRNA>hsa-mir-372
P3_5 nor-pre-miRNA>hsa-mir-944
P3_5 nor-pre-miRNA>hsa-mir-136
P3_5 nor-pre-miRNA>hsa-mir-302a
P3_5 nor-pre-miRNA>hsa-mir-4254
P3_5 nor-pre-miRNA>hsa-mir-505
P3_5 nor-pre-miRNA>hsa-mir-20a
P3_5 nor-pre-miRNA>hsa-mir-27a
P3_5 nor-pre-miRNA>hsa-mir-3158-2
P3_5 nor-pre-miRNA>hsa-mir-520d
P3_5 nor-pre-miRNA>hsa-mir-1250
P3_5 nor-pre-miRNA>hsa-mir-940
P3_5 nor-pre-miRNA>hsa-mir-1307

P3_5 nor-pre-miRNA>hsa-mir-1245a
P3_5 nor-pre-miRNA>hsa-mir-25
P3_5 nor-pre-miRNA>hsa-mir-211
P3_5 nor-pre-miRNA>hsa-mir-181d
P3_5 nor-pre-miRNA>hsa-mir-548aq
P3_5 nor-pre-miRNA>hsa-mir-200b
P3_5 nor-pre-miRNA>hsa-mir-200a
P3_5 nor-pre-miRNA>hsa-mir-3145
P3_5 nor-pre-miRNA>hsa-mir-219b
P3_5 nor-pre-miRNA>hsa-mir-1321
P3_5 nor-pre-miRNA>hsa-mir-151b
P3_5 nor-pre-miRNA>hsa-mir-548a-2
P3_5 nor-pre-miRNA>hsa-mir-202
P3_5 nor-pre-miRNA>hsa-mir-2355
P3_5 nor-pre-miRNA>hsa-mir-3167
P3_5 nor-pre-miRNA>hsa-mir-3156-2
P3_5 nor-pre-miRNA>hsa-mir-548ac
P3_5 nor-pre-miRNA>hsa-mir-548aj-1
P3_5 nor-pre-miRNA>hsa-mir-548aj-2
P3_5 nor-pre-miRNA>hsa-mir-548as
P3_5 nor-pre-miRNA>hsa-mir-548d-2
P3_5 nor-pre-miRNA>hsa-mir-548c
P3_5 nor-pre-miRNA>hsa-mir-199b
P3_5 nor-pre-miRNA>hsa-mir-34a
P3_5 nor-pre-miRNA>hsa-mir-320a
P3_5 nor-pre-miRNA>hsa-mir-101-2
P3_5 nor-pre-miRNA>hsa-mir-887
P3_5 nor-pre-miRNA>hsa-mir-135a-1
P3_5 nor-pre-miRNA>hsa-mir-517b
P3_5 nor-pre-miRNA>hsa-mir-2277
P3_5 nor-pre-miRNA>hsa-mir-1197
P3_5 nor-pre-miRNA>hsa-mir-371b
P3_5 nor-pre-miRNA>hsa-mir-3149
P3_5 nor-pre-miRNA>hsa-mir-503
P3_5 nor-pre-miRNA>hsa-mir-496
P3_5 nor-pre-miRNA>hsa-mir-1289-1
P3_5 nor-pre-miRNA>hsa-mir-548ao
P3_5 nor-pre-miRNA>hsa-mir-493
P3_5 nor-pre-miRNA>hsa-mir-1251
P3_5 nor-pre-miRNA>hsa-mir-216b
P3_5 nor-pre-miRNA>hsa-mir-19b-2
P3_5 nor-pre-miRNA>hsa-mir-466
P3_5 nor-pre-miRNA>hsa-mir-1301
P3_5 nor-pre-miRNA>hsa-mir-203a
P3_5 nor-pre-miRNA>hsa-mir-548ay
P3_5 nor-pre-miRNA>hsa-mir-524
P3_5 nor-pre-miRNA>hsa-mir-137

P3_5	nor-pre-miRNA>hsa-mir-371a	
P3_5	nor-pre-miRNA>hsa-mir-2861	
P3_5	nor-pre-miRNA>hsa-mir-1911	
P3_5	nor-pre-miRNA>hsa-mir-548ar	
P3_5	nor-pre-miRNA>hsa-mir-548f-3	
P3_5	nor-pre-miRNA>hsa-mir-1244-2	
P3_5	nor-pre-miRNA>hsa-mir-1244-1	
P3_5	nor-pre-miRNA>hsa-mir-1244-3	
P3_5	nor-pre-miRNA>hsa-mir-3123	
P3_5	nor-pre-miRNA>hsa-mir-548aa-2	
P3_5	nor-pre-miRNA>hsa-mir-548ap	
P3_5	nor-pre-miRNA>hsa-mir-548f-4	
P3_5	nor-pre-miRNA>hsa-mir-125b-2	
P3_5	nor-pre-miRNA>hsa-mir-874	
P3_5	nor-pre-miRNA>hsa-mir-146b	
P3_5	nor-pre-miRNA>hsa-mir-520b	
P3_5	nor-pre-miRNA>hsa-mir-424	
P3_5	nor-pre-miRNA>hsa-mir-1910	
P3_5	nor-pre-miRNA>hsa-mir-2114	
P3_5	nor-pre-miRNA>hsa-mir-501	
P3_5	nor-pre-miRNA>hsa-mir-1258	
P3_5	nor-pre-miRNA>hsa-mir-338	
P3_5	nor-pre-miRNA>hsa-mir-1255b-2	
P3_5	nor-pre-miRNA>hsa-mir-1286	
P3_5	nor-pre-miRNA>hsa-mir-518a-1	
P3_5	nor-pre-miRNA>hsa-mir-3605	
P3_5	nor-pre-miRNA>hsa-mir-17	
P3_5	nor-SNP	99691653
P3_5	nor-SNP	46657289
P3_5	nor-SNP	46657254
P3_5	nor-SNP	19247825
P3_5	nor-SNP	65291712
P3_5	nor-SNP	65291793
P3_5	nor-SNP	65291808
P3_5	nor-SNP	61809907
P3_5	nor-SNP	45596885
P3_5	nor-SNP	70480088
P3_5	nor-SNP	70480127
P3_5	nor-SNP	70480123
P3_5	nor-SNP	70480108
P3_5	nor-SNP	114340663
P3_5	nor-SNP	154065347
P3_5	nor-SNP	154065348
P3_5	nor-SNP	154065368
P3_5	nor-SNP	154065383
P3_5	nor-SNP	93466913
P3_5	nor-SNP	93466919

P3_5	nor-SNP	22007594
P3_5	nor-SNP	34963416
P3_5	nor-SNP	34963445
P3_5	nor-SNP	34963459
P3_5	nor-SNP	69966985
P3_5	nor-SNP	69966994
P3_5	nor-SNP	69967005
P3_5	nor-SNP	69967062
P3_5	nor-SNP	148808487
P3_5	nor-SNP	148808575
P3_5	nor-SNP	54731071
P3_5	nor-SNP	54731080
P3_5	nor-SNP	236016347
P3_5	nor-SNP	236016316
P3_5	nor-SNP	236016301
P3_5	nor-SNP	50623143
P3_5	nor-SNP	50623110
P3_5	nor-SNP	13985514
P3_5	nor-SNP	129410313
P3_5	nor-SNP	129410239
P3_5	nor-SNP	129410235
P3_5	nor-SNP	129410227
P3_5	nor-SNP	129414804
P3_5	nor-SNP	129414852
P3_5	nor-SNP	129414843
P3_5	nor-SNP	129414815
P3_5	nor-SNP	113886051
P3_5	nor-SNP	27209165
P3_5	nor-SNP	70064272
P3_5	nor-SNP	70064261
P3_5	nor-SNP	568187
P3_5	nor-SNP	568176
P3_5	nor-SNP	568125
P3_5	nor-SNP	568106
P3_5	nor-SNP	98472426
P3_5	nor-SNP	98472445
P3_5	nor-SNP	98472446
P3_5	nor-SNP	48357864
P3_5	nor-SNP	48357853
P3_5	nor-SNP	220291302
P3_5	nor-SNP	220291292
P3_5	nor-SNP	220291206
P3_5	nor-SNP	97847535
P3_5	nor-SNP	97847498
P3_5	nor-SNP	97848319
P3_5	nor-SNP	97848343
P3_5	nor-SNP	240273490

P3_5	nor-SNP	102620081
P3_5	nor-SNP	102619995
P3_5	nor-SNP	98510902
P3_5	nor-SNP	98510896
P3_5	nor-SNP	98510864
P3_5	nor-SNP	98510847
P3_5	nor-SNP	57392715
P3_5	nor-SNP	101490145
P3_5	nor-SNP	101490178
P3_5	nor-SNP	72086720
P3_5	nor-SNP	62544490
P3_5	nor-SNP	67094145
P3_5	nor-SNP	67094150
P3_5	nor-SNP	67094171
P3_5	nor-SNP	207648001
P3_5	nor-SNP	207648009
P3_5	nor-SNP	207647981
P3_5	nor-SNP	120336327
P3_5	nor-SNP	101493478
P3_5	nor-SNP	85158670
P3_5	nor-SNP	46486996
P3_5	nor-SNP	49937087
P3_5	nor-SNP	49937076
P3_5	nor-SNP	54291965
P3_5	nor-SNP	54292016
P3_5	nor-SNP	94398543
P3_5	nor-SNP	94398581
P3_5	nor-SNP	94398600
P3_5	nor-SNP	31556085
P3_5	nor-SNP	23189715
P3_5	nor-SNP	176056492
P3_5	nor-SNP	1104412
P3_5	nor-SNP	1104462
P3_5	nor-SNP	54466544
P3_5	nor-SNP	54466527
P3_5	nor-SNP	116971750
P3_5	nor-SNP	49773090
P3_5	nor-SNP	49773042
P3_5	nor-SNP	49773087
P3_5	nor-SNP	49775296
P3_5	nor-SNP	49775337
P3_5	nor-SNP	49775351
P3_5	nor-SNP	146312590
P3_5	nor-SNP	146312589
P3_5	nor-SNP	146312560
P3_5	nor-SNP	146312517
P3_5	nor-SNP	146318507

P3_5	nor-SNP	146318448
P3_5	nor-SNP	146295085
P3_5	nor-SNP	146295067
P3_5	nor-SNP	146307449
P3_5	nor-SNP	146307414
P3_5	nor-SNP	146307411
P3_5	nor-SNP	146307375
P3_5	nor-SNP	49767769
P3_5	nor-SNP	49767815
P3_5	nor-SNP	49767832
P3_5	nor-SNP	49767835
P3_5	nor-SNP	49767838
P3_5	nor-SNP	101515083
P3_5	nor-SNP	101515025
P3_5	nor-SNP	119390294
P3_5	nor-SNP	119390286
P3_5	nor-SNP	124360347
P3_5	nor-SNP	220373943
P3_5	nor-SNP	220373939
P3_5	nor-SNP	220373934
P3_5	nor-SNP	220373933
P3_5	nor-SNP	220373922
P3_5	nor-SNP	145076355
P3_5	nor-SNP	145074283
P3_5	nor-SNP	176032428
P3_5	nor-SNP	176032376
P3_5	nor-SNP	176032424
P3_5	nor-SNP	176032408
P3_5	nor-SNP	46522190
P3_5	nor-SNP	46522201
P3_5	nor-SNP	99691652
P3_5	nor-SNP	183959219
P3_5	nor-SNP	183959222
P3_5	nor-SNP	183959229
P3_5	nor-SNP	129414807
P3_5	nor-SNP	129414568
P3_5	nor-SNP	205417483
P3_5	nor-SNP	205417438
P3_5	nor-SNP	62544495
P3_5	nor-SNP	62544486
P3_5	nor-SNP	98479274
P3_5	nor-SNP	110141523
P3_5	nor-SNP	113569519
P3_5	nor-SNP	75046227
P3_5	nor-SNP	134884717
P3_5	nor-SNP	134884700
P3_5	nor-SNP	129061408

P3_5	nor-SNP	129061422
P3_5	nor-SNP	129061441
P3_5	nor-SNP	129061462
P3_5	nor-SNP	171070888
P3_5	nor-SNP	171070937
P3_5	nor-SNP	52013832
P3_5	nor-SNP	52013827
P3_5	nor-SNP	75046184
P3_5	nor-SNP	85916322
P3_5	nor-SNP	101491407
P3_5	nor-SNP	54468166
P3_5	nor-SNP	54468150
P3_5	nor-SNP	54468144
P3_5	nor-SNP	54468110
P3_5	nor-SNP	54468124
P3_5	nor-SNP	54468094
P3_5	nor-SNP	14184227
P3_5	nor-SNP	49768168
P3_5	nor-SNP	49768171
P3_5	nor-SNP	79418149
P3_5	nor-SNP	101493129
P3_5	nor-SNP	27559261
P3_5	nor-SNP	27559214
P3_5	nor-SNP	134884697
P3_5	nor-SNP	54076332
P3_5	nor-SNP	54076326
P3_5	nor-SNP	101528401
P3_5	nor-SNP	59064260
P3_5	nor-SNP	101489677
P3_5	nor-SNP	101489685
P3_5	nor-SNP	101489703
P3_5	nor-SNP	101489714
P3_5	nor-SNP	101489728
P3_5	nor-SNP	101489745
P3_5	nor-SNP	6828032
P3_5	nor-SNP	38243770
P3_5	nor-SNP	38243743
P3_5	nor-SNP	231155670
P3_5	nor-SNP	101509363
P3_5	nor-SNP	54886166
P3_5	nor-SNP	54731000
P3_5	nor-SNP	92003009
P3_5	nor-SNP	64658715
P3_5	nor-SNP	64658710
P3_5	nor-SNP	64658640
P3_5	nor-SNP	64658716
P3_5	nor-SNP	62544470

P3_5	nor-SNP	97464049
P3_5	nor-SNP	97464057
P3_5	nor-SNP	91927158
P3_5	nor-SNP	91927175
P3_5	nor-SNP	18451300
P3_5	nor-SNP	101528426
P3_5	nor-SNP	55886608
P3_5	nor-SNP	55886574
P3_5	nor-SNP	55886522
P3_5	nor-SNP	2250666
P3_5	nor-SNP	145074284
P3_5	nor-SNP	176032415
P3_5	nor-SNP	120151527
P3_5	nor-SNP	120151501
P3_5	nor-SNP	120151493
P3_5	nor-SNP	31924708
P3_5	nor-SNP	79813049
P3_5	nor-SNP	79813083
P3_5	nor-SNP	69599751
P3_5	nor-SNP	69599735
P3_5	nor-SNP	62572874
P3_5	nor-SNP	62572847
P3_5	nor-SNP	23887271
P3_5	nor-SNP	23887220
P3_5	nor-SNP	23887219
P3_5	nor-SNP	65238733
P3_5	nor-SNP	65238751
P3_5	nor-SNP	65238767
P3_5	nor-SNP	65238773
P3_5	nor-SNP	65238806
P3_5	nor-SNP	249120578
P3_5	nor-SNP	135300536
P3_5	nor-SNP	35731712
P3_5	nor-SNP	35731697
P3_5	nor-SNP	18572056
P3_5	nor-SNP	72744798
P3_5	nor-SNP	62544503
P3_5	nor-SNP	54233092
P3_5	nor-SNP	54233109
P3_5	nor-SNP	54233112
P3_5	nor-SNP	14711013
P3_5	nor-SNP	14710999
P3_5	nor-SNP	54225460
P3_5	nor-SNP	54225463
P3_5	nor-SNP	54225426
P3_5	nor-SNP	54245788
P3_5	nor-SNP	54245789

P3_5	nor-SNP	54245768
P3_5	nor-SNP	73506984
P3_5	nor-SNP	148265864
P3_5	nor-SNP	113569696
P3_5	nor-SNP	113569695
P3_5	nor-SNP	113569692
P3_5	nor-SNP	113569680
P3_5	nor-SNP	117637302
P3_5	nor-SNP	92003589
P3_5	nor-SNP	159901428
P3_5	nor-SNP	14425221
P3_5	nor-SNP	14425204
P3_5	nor-SNP	7461769
P3_5	nor-SNP	56118359
P3_5	nor-SNP	62496926
P3_5	nor-SNP	62496924
P3_5	nor-SNP	180407512
P3_5	nor-SNP	180407505
P3_5	nor-SNP	180407488
P3_5	nor-SNP	21785508
P3_5	nor-SNP	18392936
P3_5	nor-SNP	18392894
P3_5	nor-SNP	18392913
P3_5	nor-SNP	593365
P3_5	nor-SNP	593277
P3_5	nor-SNP	593362
P3_5	nor-SNP	122017258
P3_5	nor-SNP	104583838
P3_5	nor-SNP	104583828
P3_5	nor-SNP	104583804
P3_5	nor-SNP	104583776
P3_5	nor-SNP	104583759
P3_5	nor-SNP	104583796
P3_5	nor-SNP	52013754
P3_5	nor-SNP	593323
P3_5	nor-SNP	49773603
P3_5	nor-SNP	61151539
P3_5	nor-SNP	61151515
P3_5	nor-SNP	101510579
P3_5	nor-SNP	101510612
P3_5	nor-SNP	101510613
P3_5	nor-SNP	146360845
P3_5	nor-SNP	146366167
P3_5	nor-SNP	70480051
P3_5	nor-SNP	101526140
P3_5	nor-SNP	101526116
P3_5	nor-SNP	101526127

P3_5	nor-SNP	193105672
P3_5	nor-SNP	18573331
P3_5	nor-SNP	18573360
P3_5	nor-SNP	18573361
P3_5	nor-SNP	18573374
P3_5	nor-SNP	131007283
P3_5	nor-SNP	131007245
P3_5	nor-SNP	101350873
P3_5	nor-SNP	145074289
P3_5	nor-SNP	7073301
P3_5	nor-SNP	7073282
P3_5	nor-SNP	54200843
P3_5	nor-SNP	54200810
P3_5	nor-SNP	54200826
P3_5	nor-SNP	54200830
P3_5	nor-SNP	54200834
P3_5	nor-SNP	38010938
P3_5	nor-SNP	38010903
P3_5	nor-SNP	219267402
P3_5	nor-SNP	219267407
P3_5	nor-SNP	219267433
P3_5	nor-SNP	219267371
P3_5	nor-SNP	249120640
P3_5	nor-SNP	249120610
P3_5	nor-SNP	249120591
P3_5	nor-SNP	146312254
P3_5	nor-SNP	13947170
P3_5	nor-SNP	7126698
P3_5	nor-SNP	7126619
P3_5	nor-SNP	1062662
P3_5	nor-SNP	1062653
P3_5	nor-SNP	1062626
P3_5	nor-SNP	44333749
P3_5	nor-SNP	47652913
P3_5	nor-SNP	54216629
P3_5	nor-SNP	54216615
P3_5	nor-SNP	105496675
P3_5	nor-SNP	16645208
P3_5	nor-SNP	16645178
P3_5	nor-SNP	16645137
P3_5	nor-SNP	16645199
P3_5	nor-SNP	213291038
P3_5	nor-SNP	213291041
P3_5	nor-SNP	153996899
P3_5	nor-SNP	153996903
P3_5	nor-SNP	153996905
P3_5	nor-SNP	99691429

P3_5	nor-SNP	99691393
P3_5	nor-SNP	99691401
P3_5	nor-SNP	99691396
P3_5	nor-SNP	54201654
P3_5	nor-SNP	1988119
P3_5	nor-SNP	1988176
P3_5	nor-SNP	1988170
P3_5	nor-SNP	1988144
P3_5	nor-SNP	100576035
P3_5	nor-SNP	54216650
P3_5	nor-SNP	15738870
P3_5	nor-SNP	15738824
P3_5	nor-SNP	54182261
P3_5	nor-SNP	54182325
P3_5	nor-SNP	54182326
P3_5	nor-SNP	31936263
P3_5	nor-SNP	57392678
P3_5	nor-SNP	57392686
P3_5	nor-SNP	113569088
P3_5	nor-SNP	54238182
P3_5	nor-SNP	54238159
P3_5	nor-SNP	19405743
P3_5	nor-SNP	19405676
P3_5	nor-SNP	19405672
P3_5	nor-SNP	69330818
P3_5	nor-SNP	69330824
P3_5	nor-SNP	69330825
P3_5	nor-SNP	69330871
P3_5	nor-SNP	2018056
P3_5	nor-SNP	2018004
P3_5	nor-SNP	2018002
P3_5	nor-SNP	49779210
P3_5	nor-SNP	49779214
P3_5	nor-SNP	49779234
P3_5	nor-SNP	54291161
P3_5	nor-SNP	189547748
P3_5	nor-SNP	189547779
P3_5	nor-SNP	189547761
P3_5	nor-SNP	101351088
P3_5	nor-SNP	113569406
P3_5	nor-SNP	32224285
P3_5	nor-SNP	139006339
P3_5	nor-SNP	139006378
P3_5	nor-SNP	92003356
P3_5	nor-SNP	13947296
P3_5	nor-SNP	13947292
P3_5	nor-SNP	145076302

P3_5	nor-SNP	54233093
P3_5	nor-SNP	54233113
P3_5	nor-SNP	54233170
P3_5	nor-SNP	103361221
P3_5	nor-SNP	54223379
P3_5	nor-SNP	159901465
P3_5	nor-SNP	159901426
P3_5	nor-SNP	79107084
P3_5	nor-SNP	2321762
P3_5	nor-SNP	2321774
P3_5	nor-SNP	2321792
P3_5	nor-SNP	7461828
P3_5	nor-SNP	7461826
P3_5	nor-SNP	7461827
P3_5	nor-SNP	38010964
P3_5	nor-SNP	117637290
P3_5	nor-SNP	117637325
P3_5	nor-SNP	105154097
P3_5	nor-SNP	189842822
P3_5	nor-SNP	99691200
P3_5	nor-SNP	105154091
P3_5	nor-SNP	105154089
P3_5	nor-SNP	31357301
P3_5	nor-SNP	31357325
P3_5	nor-SNP	13985689
P3_5	nor-SNP	185485646
P3_5	nor-SNP	100576059
P3_5	nor-SNP	105154013
P3_5	nor-SNP	54238189
P3_5	nor-SNP	54238203
P3_5	nor-SNP	54238208
P3_5	nor-SNP	145074342
P3_5	nor-SNP	1102498
P3_5	nor-SNP	1102501
P3_5	nor-SNP	1102563
P3_5	nor-SNP	1102567
P3_5	nor-SNP	1103284
P3_5	nor-SNP	138756428
P3_5	nor-SNP	138756369
P3_5	nor-SNP	131154943
P3_5	nor-SNP	85090790
P3_5	nor-SNP	85090809
P3_5	nor-SNP	85090852
P3_5	nor-SNP	100575818
P3_5	nor-SNP	100575791
P3_5	nor-SNP	100575762
P3_5	nor-SNP	100575761

P3_5	nor-SNP	249120584
P3_5	nor-SNP	135560369
P3_5	nor-SNP	135560387
P3_5	nor-SNP	135061124
P3_5	nor-SNP	207974741
P3_5	nor-SNP	207974738
P3_5	nor-SNP	126858425
P3_5	nor-SNP	14830172
P3_5	nor-SNP	105496638
P3_5	nor-SNP	105496622
P3_5	nor-SNP	117102696
P3_5	nor-SNP	117102674
P3_5	nor-SNP	117102660
P3_5	nor-SNP	117102649
P3_5	nor-SNP	132436375
P3_5	nor-SNP	37883200
P3_5	nor-SNP	37883195
P3_5	nor-SNP	37883165
P3_5	nor-SNP	93142427
P3_5	nor-SNP	65467701
P3_5	nor-SNP	65467669
P3_5	nor-SNP	65467665
P3_5	nor-SNP	54200853
P3_5	nor-SNP	65016300
P3_5	nor-SNP	65016325
P3_5	nor-SNP	131007001
P3_5	nor-SNP	131007109
P3_5	nor-SNP	131007087
P3_5	nor-SNP	131007004
P3_5	nor-SNP	219267370
P3_5	nor-SNP	219267443
P3_5	nor-SNP	9211782
P3_5	nor-SNP	9211802
P3_5	nor-SNP	31924631
P3_5	nor-SNP	54216616
P3_5	nor-SNP	54216670
P3_5	nor-SNP	54216681
P3_5	nor-SNP	103361226
P3_5	nor-SNP	22102542
P3_5	nor-SNP	22102546
P3_5	nor-SNP	22102545
P3_5	nor-SNP	22102543
P3_5	nor-SNP	4850310
P3_5	nor-SNP	4850331
P3_5	nor-SNP	146360765
P3_5	nor-SNP	146360813
P3_5	nor-SNP	146360779

P3_5	nor-SNP	146360826
P3_5	nor-SNP	15935347
P3_5	nor-SNP	15935348
P3_5	nor-SNP	15935351
P3_5	nor-SNP	52328298
P3_5	nor-SNP	52328248
P3_5	nor-SNP	54224382
P3_5	nor-SNP	92956422
P3_5	nor-SNP	92956420
P3_5	nor-SNP	92956416
P3_5	nor-SNP	92956412
P3_5	nor-SNP	92956409
P3_5	nor-SNP	101491923
P3_5	nor-SNP	54290995
P3_5	nor-SNP	54290994
P3_5	nor-SNP	120336365
P3_5	nor-SNP	120336384
P3_5	nor-SNP	77879011
P3_5	nor-SNP	62572885
P3_5	nor-SNP	133680381
P3_5	nor-SNP	101351048
P3_5	nor-SNP	101526951
P3_5	nor-SNP	70064324
P3_5	nor-SNP	54223433
P3_5	nor-SNP	103361245
P3_5	nor-SNP	15935316
P3_5	nor-SNP	34041842
P3_5	nor-SNP	79107068
P3_5	nor-SNP	79107061
P3_5	nor-SNP	79107049
P3_5	nor-SNP	79107017
P3_5	nor-SNP	13985739
P3_5	nor-SNP	13985772
P3_5	nor-SNP	41128620
P3_5	nor-SNP	41128599
P3_5	nor-SNP	41128578
P3_5	nor-SNP	101335451
P3_5	nor-SNP	97885708
P3_5	nor-SNP	97885720
P3_5	nor-SNP	213291030
P3_5	nor-SNP	56227910
P3_5	nor-SNP	56227905
P3_5	nor-SNP	189842826
P3_5	nor-SNP	133303758
P3_5	nor-SNP	31203221
P3_5	nor-SNP	31203267
P3_5	nor-SNP	31203228

P3_5	nor-SNP	31203216
P3_5	nor-SNP	31203207
P3_5	nor-SNP	97885715
P3_5	nor-SNP	135061112
P3_5	nor-SNP	135061111
P3_5	nor-SNP	135061109
P3_5	nor-SNP	135061106
P3_5	nor-SNP	135061039
P3_5	nor-SNP	54201667
P3_5	nor-SNP	25551588
P3_5	nor-SNP	32547811
P3_5	nor-SNP	32547810
P3_5	nor-SNP	32547809
P3_5	nor-SNP	32547795
P3_5	nor-SNP	54214286
P3_5	nor-SNP	98511725
P3_5	nor-SNP	98511634
P3_5	nor-SNP	130548199
P3_5	nor-SNP	113997768
P3_5	nor-SNP	113997812
P3_5	nor-SNP	113997817
P3_5	nor-SNP	101335438
P3_5	nor-SNP	7073344
P3_5	nor-SNP	115010026
P3_5	nor-SNP	109849556
P3_5	nor-SNP	118310291
P3_5	nor-SNP	118310287
P3_5	nor-SNP	232578032
P3_5	nor-SNP	232578048
P3_5	nor-SNP	232578099
P3_5	nor-SNP	118310319
P3_5	nor-SNP	118310349
P3_5	nor-SNP	9392072
P3_5	nor-SNP	241295617
P3_5	nor-SNP	207974756
P3_5	nor-SNP	86368890
P3_5	nor-SNP	86368898
P3_5	nor-SNP	86368922
P3_5	nor-SNP	86368929
P3_5	nor-SNP	86368959
P3_5	nor-SNP	147075204
P3_5	nor-SNP	147075166
P3_5	nor-SNP	147075152
P3_5	nor-SNP	147075121
P3_5	nor-SNP	147075120
P3_5	nor-SNP	1988116
P3_5	nor-SNP	17962644

P3_5	nor-SNP	17962615
P3_5	nor-SNP	136983319
P3_5	nor-SNP	136983281
P3_5	nor-SNP	136983275
P3_5	nor-SNP	104196300
P3_5	nor-SNP	54201668
P3_5	nor-SNP	54204489
P3_5	nor-SNP	54204496
P3_5	nor-SNP	54204497
P3_5	nor-SNP	133680673
P3_5	nor-SNP	249120631
P3_5	nor-SNP	85775275
P3_5	nor-SNP	22102536
P3_5	nor-SNP	149396242
P3_5	nor-SNP	105154084
P3_5	nor-SNP	101491965
P3_5	nor-SNP	97847569
P3_5	nor-SNP	97847573
P3_5	nor-SNP	113569394
P3_5	nor-SNP	49774381
P3_5	nor-SNP	180725568
P3_5	nor-SNP	101526957
P3_5	nor-SNP	101526987
P3_5	nor-SNP	101526991
P3_5	nor-SNP	77879071
P3_5	nor-SNP	79099736
P3_5	nor-SNP	167967953
P3_5	nor-SNP	167967958
P3_5	nor-SNP	167967963
P3_5	nor-SNP	20236701
P3_5	nor-SNP	56118358
P3_5	nor-SNP	54234260
P3_5	nor-SNP	69330823
P3_5	nor-SNP	33798007
P3_5	nor-SNP	33798091
P3_5	nor-SNP	33798031
P3_5	nor-SNP	92002896
P3_5	nor-SNP	114340628
P3_5	nor-SNP	114340631
P3_5	nor-SNP	154065385
P3_5	nor-SNP	93466912
P3_5	nor-SNP	93466910
P3_5	nor-SNP	93466909
P3_5	nor-SNP	93466866
P3_5	nor-SNP	22007634
P3_5	nor-SNP	129410228
P3_5	nor-SNP	116971778

P3_5	nor-SNP	145074272
P3_5	nor-SNP	46522298
P3_5	nor-SNP	183959232
P3_5	nor-SNP	129414553
P3_5	nor-SNP	110141578
P3_5	nor-SNP	134884723
P3_5	nor-SNP	38243739
P3_5	nor-SNP	38243727
P3_5	nor-SNP	46522255
P3_5	nor-SNP	231155581
P3_5	nor-SNP	231155644
P3_5	nor-SNP	64658623
P3_5	nor-SNP	176032384
P3_5	nor-SNP	18572073
P3_5	nor-SNP	54225501
P3_5	nor-SNP	54245841
P3_5	nor-SNP	61151573
P3_5	nor-SNP	146360778
P3_5	nor-SNP	134884737
P3_5	nor-SNP	1062656
P3_5	nor-SNP	1062599
P3_5	nor-SNP	1062574
P3_5	nor-SNP	54201695
P3_5	nor-SNP	54201703
P3_5	nor-SNP	189547735
P3_5	nor-SNP	189547778
P3_5	nor-SNP	8007104
P3_5	nor-SNP	2321820
P3_5	nor-SNP	117637326
P3_5	nor-SNP	2321809
P3_5	nor-SNP	31357245
P3_5	nor-SNP	31357244
P3_5	nor-SNP	31357238
P3_5	nor-SNP	13985721
P3_5	nor-SNP	13985805
P3_5	nor-SNP	13985806
P3_5	nor-SNP	1103312
P3_5	nor-SNP	1103328
P3_5	nor-SNP	126858392
P3_5	nor-SNP	126858406
P3_5	nor-SNP	14830197
P3_5	nor-SNP	14830201
P3_5	nor-SNP	14830215
P3_5	nor-SNP	14830216
P3_5	nor-SNP	9211778
P3_5	nor-SNP	9211747
P3_5	nor-SNP	9211738

P3_5	nor-SNP	1103331
P3_5	nor-SNP	72744805
P3_5	nor-SNP	57392697
P3_5	nor-SNP	2321827
P3_5	nor-SNP	189842885
P3_5	nor-SNP	133303744
P3_5	nor-SNP	54201692
P3_5	nor-SNP	25551550
P3_5	nor-SNP	54214333
P3_5	nor-SNP	232578049
P3_5	nor-SNP	119390263
P3_5	nor-SNP	22102519
P3_5	nor-SNP	149396272
P3_5	nor-SNP	113569054
P3_5	nor-SNP	101491974
P3_5	nor-SNP	101491981
P3_5	nor-SNP	49774383
P3_5	nor-SNP	54234265
P3_5	nor-SNP	69330886
P3_5	nor-SNP	114340615
P3_5	nor-SNP	129414806
P3_5	nor-SNP	146312541
P3_5	nor-SNP	70480062
P3_5	nor-SNP	1988193
P3_5	nor-SNP	149396273
P3_5	nor-SNP	49774389
P3_5	nor-SNP	54234315
P3_5	nor-SNP	54234340
P3_5	nor-SNP	114340550
P3_5	iso-pre-miRNA>hsa-mir-192	
P3_5	iso-pre-miRNA>hsa-mir-888	
P3_5	iso-pre-miRNA>hsa-mir-216a	
P3_5	iso-pre-miRNA>hsa-mir-3142	
P3_5	iso-pre-miRNA>hsa-mir-218-2	
P3_5	iso-pre-miRNA>hsa-mir-147b	
P3_5	iso-pre-miRNA>hsa-mir-662	
P3_5	iso-pre-miRNA>hsa-mir-548at	
P3_5	iso-pre-miRNA>hsa-mir-1290	
P3_5	iso-pre-miRNA>hsa-mir-320e	
P3_5	iso-pre-miRNA>hsa-mir-3689b	
P3_5	iso-pre-miRNA>hsa-mir-1281	
P3_5	iso-pre-miRNA>hsa-mir-892c	
P3_5	iso-pre-miRNA>hsa-mir-3679	
P3_5	iso-pre-miRNA>hsa-mir-1825	
P3_5	iso-pre-miRNA>hsa-mir-1251	
P3_5	iso-pre-miRNA>hsa-mir-548al	
P3_5	iso-pre-miRNA>hsa-mir-1292	

P3_5 iso-pre-miRNA>hsa-mir-302d
P3_5 iso-pre-miRNA>hsa-mir-486-2
P3_5 iso-pre-miRNA>hsa-mir-4268
P3_5 iso-pre-miRNA>hsa-mir-1229
P3_5 iso-pre-miRNA>hsa-mir-30a
P3_5 iso-pre-miRNA>hsa-mir-4322
P3_5 iso-pre-miRNA>hsa-mir-338
P3_5 iso-pre-miRNA>hsa-mir-155
P3_5 iso-pre-miRNA>hsa-mir-1-1
P3_5 iso-pre-miRNA>hsa-mir-320b-1
P3_5 iso-pre-miRNA>hsa-mir-199a-1
P3_5 iso-pre-miRNA>hsa-mir-380
P3_5 iso-pre-miRNA>hsa-mir-1252
P3_5 iso-pre-miRNA>hsa-mir-4252
P3_5 iso-pre-miRNA>hsa-mir-363
P3_5 iso-pre-miRNA>hsa-mir-134
P3_5 iso-pre-miRNA>hsa-mir-4312
P3_5 iso-pre-miRNA>hsa-mir-379
P3_5 iso-pre-miRNA>hsa-mir-483
P3_5 iso-pre-miRNA>hsa-mir-3124
P3_5 iso-pre-miRNA>hsa-mir-1245b
P3_5 iso-pre-miRNA>hsa-mir-3151
P3_5 iso-pre-miRNA>hsa-mir-1255b-2
P3_5 iso-pre-miRNA>hsa-mir-937
P3_5 iso-pre-miRNA>hsa-mir-1228
P3_5 iso-pre-miRNA>hsa-mir-3065
P3_5 iso-pre-miRNA>hsa-mir-1245a
P3_5 iso-pre-miRNA>hsa-mir-3173
P3_5 iso-pre-miRNA>hsa-mir-20b
P3_5 iso-pre-miRNA>hsa-mir-381
P3_5 iso-pre-miRNA>hsa-mir-548av
P3_5 iso-pre-miRNA>hsa-mir-887
P3_5 iso-pre-miRNA>hsa-mir-4318
P3_5 iso-pre-miRNA>hsa-let-7e
P3_5 iso-pre-miRNA>hsa-mir-1322
P3_5 iso-pre-miRNA>hsa-mir-3177
P3_5 iso-pre-miRNA>hsa-mir-3649
P3_5 iso-pre-miRNA>hsa-mir-3162
P3_5 iso-pre-miRNA>hsa-mir-3146
P3_5 iso-pre-miRNA>hsa-mir-548ae-1
P3_5 iso-pre-miRNA>hsa-mir-378a
P3_5 iso-pre-miRNA>hsa-mir-509-3
P3_5 iso-pre-miRNA>hsa-mir-1234
P3_5 iso-pre-miRNA>hsa-mir-1226
P3_5 iso-pre-miRNA>hsa-let-7b
P3_5 iso-pre-miRNA>hsa-mir-200a
P3_5 iso-pre-miRNA>hsa-mir-523

P3_5 iso-pre-miRNA>hsa-mir-922
P3_5 iso-pre-miRNA>hsa-mir-543
P3_5 iso-pre-miRNA>hsa-mir-1301
P3_5 iso-pre-miRNA>hsa-mir-874
P3_5 iso-pre-miRNA>hsa-mir-146b
P3_5 iso-pre-miRNA>hsa-mir-33a
P3_5 iso-pre-miRNA>hsa-mir-3927
P3_5 iso-pre-miRNA>hsa-mir-367
P3_5 iso-pre-miRNA>hsa-mir-425
P3_5 iso-pre-miRNA>hsa-mir-378e
P3_5 iso-pre-miRNA>hsa-mir-301a
P3_5 iso-pre-miRNA>hsa-mir-197
P3_5 iso-pre-miRNA>hsa-mir-1913
P3_5 iso-pre-miRNA>hsa-mir-328
P3_5 iso-pre-miRNA>hsa-mir-30d
P3_5 iso-pre-miRNA>hsa-mir-675
P3_5 iso-pre-miRNA>hsa-mir-510
P3_5 iso-pre-miRNA>hsa-mir-376a-2
P3_5 iso-pre-miRNA>hsa-mir-130b
P3_5 iso-pre-miRNA>hsa-mir-21
P3_5 iso-pre-miRNA>hsa-mir-504
P3_5 iso-pre-miRNA>hsa-mir-499b
P3_5 iso-pre-miRNA>hsa-mir-544b
P3_5 iso-pre-miRNA>hsa-mir-129-1
P3_5 iso-pre-miRNA>hsa-mir-26b
P3_5 iso-pre-miRNA>hsa-mir-548ad
P3_5 iso-pre-miRNA>hsa-mir-3116-2
P3_5 iso-pre-miRNA>hsa-mir-1277
P3_5 iso-pre-miRNA>hsa-mir-2117
P3_5 iso-pre-miRNA>hsa-mir-382
P3_5 iso-pre-miRNA>hsa-mir-3610
P3_5 iso-pre-miRNA>hsa-mir-942
P3_5 iso-pre-miRNA>hsa-mir-96
P3_5 iso-pre-miRNA>hsa-mir-943
P3_5 iso-pre-miRNA>hsa-mir-521-2
P3_5 iso-pre-miRNA>hsa-mir-300
P3_5 iso-pre-miRNA>hsa-mir-3138
P3_5 iso-pre-miRNA>hsa-mir-195
P3_5 iso-pre-miRNA>hsa-mir-125a
P3_5 iso-pre-miRNA>hsa-mir-224
P3_5 iso-pre-miRNA>hsa-mir-1911
P3_5 iso-pre-miRNA>hsa-mir-495
P3_5 iso-pre-miRNA>hsa-mir-1909
P3_5 iso-pre-miRNA>hsa-mir-142
P3_5 iso-pre-miRNA>hsa-mir-933
P3_5 iso-pre-miRNA>hsa-mir-512-1
P3_5 iso-pre-miRNA>hsa-mir-3118-3

P3_5 iso-pre-miRNA>hsa-mir-487a
P3_5 iso-pre-miRNA>hsa-mir-409
P3_5 iso-pre-miRNA>hsa-mir-3118-4
P3_5 iso-pre-miRNA>hsa-mir-3184
P3_5 iso-pre-miRNA>hsa-mir-95
P3_5 iso-pre-miRNA>hsa-mir-3118-2
P3_5 iso-pre-miRNA>hsa-mir-3622a
P3_5 iso-pre-miRNA>hsa-mir-133a-1
P3_5 iso-pre-miRNA>hsa-mir-892a
P3_5 iso-pre-miRNA>hsa-let-7i
P3_5 iso-pre-miRNA>hsa-mir-2392
P3_5 iso-pre-miRNA>hsa-mir-28
P3_5 iso-pre-miRNA>hsa-mir-365a
P3_5 iso-pre-miRNA>hsa-mir-203b
P3_5 iso-pre-miRNA>hsa-mir-1908
P3_5 iso-pre-miRNA>hsa-mir-4308
P3_5 iso-pre-miRNA>hsa-mir-525
P3_5 iso-pre-miRNA>hsa-mir-93
P3_5 iso-pre-miRNA>hsa-mir-2114
P3_5 iso-pre-miRNA>hsa-mir-3156-1
P3_5 iso-pre-miRNA>hsa-mir-320a
P3_5 iso-pre-miRNA>hsa-mir-518f
P3_5 iso-pre-miRNA>hsa-mir-3140
P3_5 iso-pre-miRNA>hsa-mir-4257
P3_5 iso-pre-miRNA>hsa-mir-521-1
P3_5 iso-pre-miRNA>hsa-mir-3192
P3_5 iso-pre-miRNA>hsa-mir-370
P3_5 iso-pre-miRNA>hsa-mir-939
P3_5 iso-pre-miRNA>hsa-mir-105-1
P3_5 iso-pre-miRNA>hsa-mir-105-2
P3_5 iso-pre-miRNA>hsa-mir-107
P3_5 iso-pre-miRNA>hsa-mir-23a
P3_5 iso-pre-miRNA>hsa-mir-128-1
P3_5 iso-pre-miRNA>hsa-mir-4324
P3_5 iso-pre-miRNA>hsa-mir-3156-2
P3_5 iso-pre-miRNA>hsa-mir-665
P3_5 iso-pre-miRNA>hsa-mir-3126
P3_5 iso-pre-miRNA>hsa-mir-3150b
P3_5 iso-pre-miRNA>hsa-mir-526b
P3_5 iso-pre-miRNA>hsa-mir-3118-1
P3_5 iso-pre-miRNA>hsa-mir-518a-1
P3_5 iso-pre-miRNA>hsa-mir-4259
P3_5 iso-pre-miRNA>hsa-mir-129-2
P3_5 iso-pre-miRNA>hsa-mir-138-2
P3_5 iso-pre-miRNA>hsa-mir-548f-5
P3_5 iso-pre-miRNA>hsa-mir-3158-1
P3_5 iso-pre-miRNA>hsa-mir-544a

P3_5 iso-pre-miRNA>hsa-mir-489
P3_5 iso-pre-miRNA>hsa-mir-2861
P3_5 iso-pre-miRNA>hsa-mir-876
P3_5 iso-pre-miRNA>hsa-mir-1910
P3_5 iso-pre-miRNA>hsa-mir-185
P3_5 iso-pre-miRNA>hsa-mir-1914
P3_5 iso-pre-miRNA>hsa-mir-519b
P3_5 iso-pre-miRNA>hsa-mir-513b
P3_5 iso-pre-miRNA>hsa-mir-520a
P3_5 iso-pre-miRNA>hsa-mir-513c
P3_5 iso-pre-miRNA>hsa-mir-126
P3_5 iso-pre-miRNA>hsa-mir-323b
P3_5 iso-pre-miRNA>hsa-mir-512-2
P3_5 iso-pre-miRNA>hsa-mir-412
P3_5 iso-pre-miRNA>hsa-mir-1244-1
P3_5 iso-pre-miRNA>hsa-mir-3167
P3_5 iso-pre-miRNA>hsa-mir-125b-2
P3_5 iso-pre-miRNA>hsa-mir-466
P3_5 iso-pre-miRNA>hsa-mir-517a
P3_5 iso-pre-miRNA>hsa-mir-509-2
P3_5 iso-pre-miRNA>hsa-mir-335
P3_5 iso-pre-miRNA>hsa-mir-885
P3_5 iso-pre-miRNA>hsa-mir-421
P3_5 iso-pre-miRNA>hsa-mir-33b
P3_5 iso-pre-miRNA>hsa-mir-3919
P3_5 iso-pre-miRNA>hsa-mir-296
P3_5 iso-pre-miRNA>hsa-mir-548am
P3_5 iso-pre-miRNA>hsa-mir-154
P3_5 iso-pre-miRNA>hsa-mir-184
P3_5 iso-pre-miRNA>hsa-mir-1225
P3_5 iso-pre-miRNA>hsa-mir-1324
P3_5 iso-pre-miRNA>hsa-mir-3922
P3_5 iso-pre-miRNA>hsa-mir-149
P3_5 iso-pre-miRNA>hsa-mir-541
P3_5 iso-pre-miRNA>hsa-mir-3155a
P3_5 iso-pre-miRNA>hsa-mir-3671
P3_5 iso-pre-miRNA>hsa-mir-450b
P3_5 iso-pre-miRNA>hsa-mir-2053
P3_5 iso-pre-miRNA>hsa-mir-152
P3_5 iso-pre-miRNA>hsa-mir-500a
P3_5 iso-pre-miRNA>hsa-mir-345
P3_5 iso-pre-miRNA>hsa-mir-3188
P3_5 iso-pre-miRNA>hsa-mir-3149
P3_5 iso-pre-miRNA>hsa-mir-488
P3_5 iso-pre-miRNA>hsa-mir-3150a
P3_5 iso-pre-miRNA>hsa-mir-153-2
P3_5 iso-pre-miRNA>hsa-mir-1299

P3_5 iso-pre-miRNA>hsa-mir-431
P3_5 iso-pre-miRNA>hsa-mir-188
P3_5 iso-pre-miRNA>hsa-mir-542
P3_5 iso-pre-miRNA>hsa-mir-455
P3_5 iso-pre-miRNA>hsa-mir-493
P3_5 iso-pre-miRNA>hsa-mir-1912
P3_5 iso-pre-miRNA>hsa-mir-206
P3_5 iso-pre-miRNA>hsa-mir-509-1
P3_5 iso-pre-miRNA>hsa-mir-340
P3_5 iso-pre-miRNA>hsa-mir-511
P3_5 iso-pre-miRNA>hsa-mir-190a
P3_5 iso-pre-miRNA>hsa-mir-519a-1
P3_5 iso-pre-miRNA>hsa-mir-2276
P3_5 iso-pre-miRNA>hsa-mir-485
P3_5 iso-pre-miRNA>hsa-mir-3158-2
P3_5 iso-pre-miRNA>hsa-mir-491
P3_5 iso-pre-miRNA>hsa-mir-940
P3_5 iso-pre-miRNA>hsa-mir-1224
P3_5 iso-pre-miRNA>hsa-mir-516b-2
P3_5 iso-pre-miRNA>hsa-mir-423
P3_5 iso-pre-miRNA>hsa-mir-3686
P3_5 iso-pre-miRNA>hsa-mir-519a-2
P3_5 iso-pre-miRNA>hsa-mir-522
P3_5 iso-pre-miRNA>hsa-mir-2116
P3_5 iso-pre-miRNA>hsa-mir-1468
P3_5 iso-pre-miRNA>hsa-mir-520f
P3_5 iso-pre-miRNA>hsa-mir-519c
P3_5 iso-pre-miRNA>hsa-mir-518a-2
P3_5 iso-pre-miRNA>hsa-mir-320c-1
P3_5 iso-pre-miRNA>hsa-mir-524
P3_5 iso-pre-miRNA>hsa-mir-518b
P3_5 iso-pre-miRNA>hsa-mir-520c
P3_5 iso-pre-miRNA>hsa-mir-4274
P3_5 iso-pre-miRNA>hsa-mir-516b-1
P3_5 iso-pre-miRNA>hsa-mir-454
P3_5 iso-pre-miRNA>hsa-mir-3157
P3_5 iso-pre-miRNA>hsa-mir-1303
P3_5 iso-pre-miRNA>hsa-mir-507
P3_5 iso-pre-miRNA>hsa-mir-501
P3_5 iso-pre-miRNA>hsa-mir-1295a
P3_5 iso-pre-miRNA>hsa-mir-3618
P3_5 iso-pre-miRNA>hsa-mir-1183
P3_5 iso-pre-miRNA>hsa-mir-1238
P3_5 iso-pre-miRNA>hsa-mir-3678
P3_5 iso-pre-miRNA>hsa-mir-452
P3_5 iso-pre-miRNA>hsa-mir-1233-1
P3_5 iso-pre-miRNA>hsa-mir-1233-2

P3_5 iso-pre-miRNA>hsa-mir-3179-1
P3_5 iso-pre-miRNA>hsa-mir-30e
P3_5 iso-pre-miRNA>hsa-mir-520g
P3_5 iso-pre-miRNA>hsa-mir-520h
P3_5 iso-pre-miRNA>hsa-mir-661
P3_5 iso-pre-miRNA>hsa-mir-548a-1
P3_5 iso-pre-miRNA>hsa-mir-15b
P3_5 iso-pre-miRNA>hsa-mir-146a
P3_5 iso-pre-miRNA>hsa-mir-3660
P3_5 iso-pre-miRNA>hsa-mir-219a-2
P3_5 iso-pre-miRNA>hsa-mir-514a-1
P3_5 iso-pre-miRNA>hsa-mir-3176
P3_5 iso-pre-miRNA>hsa-mir-194-2
P3_5 iso-pre-miRNA>hsa-mir-1237
P3_5 iso-pre-miRNA>hsa-mir-3936
P3_5 iso-pre-miRNA>hsa-mir-19b-2
P3_5 iso-pre-miRNA>hsa-mir-199b
P3_5 iso-pre-miRNA>hsa-mir-1250
P3_5 iso-pre-miRNA>hsa-mir-3129
P3_5 iso-pre-miRNA>hsa-mir-516a-1
P3_5 iso-pre-miRNA>hsa-mir-410
P3_5 iso-pre-miRNA>hsa-mir-330
P3_5 iso-pre-miRNA>hsa-mir-3615
P3_5 iso-pre-miRNA>hsa-mir-1304
P3_5 iso-pre-miRNA>hsa-mir-519d
P3_5 iso-pre-miRNA>hsa-mir-3938
P3_5 iso-pre-miRNA>hsa-mir-193a
P3_5 iso-pre-miRNA>hsa-mir-516a-2
P3_5 iso-pre-miRNA>hsa-mir-30b
P3_5 iso-pre-miRNA>hsa-mir-1289-2
P3_5 iso-pre-miRNA>hsa-mir-4265
P3_5 iso-pre-miRNA>hsa-mir-27b
P3_5 iso-pre-miRNA>hsa-mir-3166
P3_5 iso-pre-miRNA>hsa-mir-7-1
P3_5 iso-pre-miRNA>hsa-mir-196a-2
P3_5 iso-pre-miRNA>hsa-mir-337
P3_5 iso-pre-miRNA>hsa-mir-548b
P3_5 iso-pre-miRNA>hsa-mir-3123
P3_5 iso-pre-miRNA>hsa-mir-487b
P3_5 iso-pre-miRNA>hsa-mir-448
P3_5 iso-pre-miRNA>hsa-mir-497
P3_5 iso-pre-miRNA>hsa-mir-3939
P3_5 iso-pre-miRNA>hsa-mir-944
P3_5 iso-pre-miRNA>hsa-mir-1287
P3_5 iso-pre-miRNA>hsa-mir-450a-1
P3_5 iso-pre-miRNA>hsa-mir-145
P3_5 iso-pre-miRNA>hsa-mir-183

P3_5 iso-pre-miRNA>hsa-mir-1197
P3_5 iso-pre-miRNA>hsa-mir-548f-4
P3_5 iso-pre-miRNA>hsa-mir-365b
P3_5 iso-pre-miRNA>hsa-mir-517c
P3_5 iso-pre-miRNA>hsa-mir-2277
P3_5 iso-pre-miRNA>hsa-mir-34a
P3_5 iso-pre-miRNA>hsa-mir-203a
P3_5 iso-pre-miRNA>hsa-mir-3912
P3_5 iso-pre-miRNA>hsa-mir-3664
P3_5 iso-pre-miRNA>hsa-mir-23b
P3_5 iso-pre-miRNA>hsa-mir-548f-1
P3_5 iso-pre-miRNA>hsa-mir-450a-2
P3_5 iso-pre-miRNA>hsa-mir-133a-2
P3_5 iso-pre-miRNA>hsa-mir-663b
P3_5 iso-pre-miRNA>hsa-mir-1273a
P3_5 iso-pre-miRNA>hsa-mir-202
P3_5 iso-pre-miRNA>hsa-mir-219a-1
P3_5 iso-pre-miRNA>hsa-mir-1182
P3_5 iso-pre-miRNA>hsa-mir-211
P3_5 iso-pre-miRNA>hsa-mir-518c
P3_5 iso-pre-miRNA>hsa-mir-221
P3_5 iso-pre-miRNA>hsa-mir-3659
P3_5 iso-pre-miRNA>hsa-mir-151b
P3_5 iso-pre-miRNA>hsa-mir-3663
P3_5 iso-pre-miRNA>hsa-mir-3657
P3_5 iso-pre-miRNA>hsa-mir-1269a
P3_5 iso-pre-miRNA>hsa-mir-218-1
P3_5 iso-pre-miRNA>hsa-mir-138-1
P3_5 iso-pre-miRNA>hsa-mir-548h-4
P3_5 iso-pre-miRNA>hsa-mir-182
P3_5 iso-pre-miRNA>hsa-mir-671
P3_5 iso-pre-miRNA>hsa-mir-187
P3_5 iso-pre-miRNA>hsa-mir-3620
P3_5 iso-pre-miRNA>hsa-mir-3605
P3_5 iso-pre-miRNA>hsa-mir-199a-2
P3_5 iso-pre-miRNA>hsa-mir-1273h
P3_5 iso-pre-miRNA>hsa-mir-769
P3_5 iso-pre-miRNA>hsa-mir-10b
P3_5 iso-pre-miRNA>hsa-mir-1285-2
P3_5 iso-pre-miRNA>hsa-mir-520e
P3_5 iso-pre-miRNA>hsa-mir-1302-8
P3_5 iso-pre-miRNA>hsa-mir-1256
P3_5 iso-pre-miRNA>hsa-mir-217
P3_5 iso-pre-miRNA>hsa-mir-3653
P3_5 iso-pre-miRNA>hsa-mir-181b-1
P3_5 iso-pre-miRNA>hsa-mir-384
P3_5 iso-pre-miRNA>hsa-mir-490

P3_5	iso-pre-miRNA>hsa-mir-222	
P3_5	iso-pre-miRNA>hsa-mir-498	
P3_5	iso-pre-miRNA>hsa-mir-499a	
P3_5	iso-pre-miRNA>hsa-mir-181d	
P3_5	iso-pre-miRNA>hsa-mir-1302-3	
P3_5	iso-pre-miRNA>hsa-mir-205	
P3_5	iso-pre-miRNA>hsa-mir-433	
P3_5	iso-pre-miRNA>hsa-mir-1307	
P3_5	iso-pre-miRNA>hsa-mir-3180-5	
P3_5	iso-pre-miRNA>hsa-mir-3180-4	
P3_5	iso-pre-miRNA>hsa-mir-1302-1	
P3_5	iso-pre-miRNA>hsa-mir-1294	
P3_5	iso-pre-miRNA>hsa-mir-1302-5	
P3_5	iso-pre-miRNA>hsa-mir-1302-4	
P3_5	iso-pre-miRNA>hsa-mir-23c	
P3_5	iso-pre-miRNA>hsa-mir-1247	
P3_5	iso-SNP	64658715
P3_5	iso-SNP	64658716
P3_5	iso-SNP	145076356
P3_5	iso-SNP	56216092
P3_5	iso-SNP	56216156
P3_5	iso-SNP	159901426
P3_5	iso-SNP	168195174
P3_5	iso-SNP	159901428
P3_5	iso-SNP	45725255
P3_5	iso-SNP	45725274
P3_5	iso-SNP	820197
P3_5	iso-SNP	40646795
P3_5	iso-SNP	19223566
P3_5	iso-SNP	19223571
P3_5	iso-SNP	19223639
P3_5	iso-SNP	47212591
P3_5	iso-SNP	137742013
P3_5	iso-SNP	137741994
P3_5	iso-SNP	137741987
P3_5	iso-SNP	137741989
P3_5	iso-SNP	40646803
P3_5	iso-SNP	41488550
P3_5	iso-SNP	47212593
P3_5	iso-SNP	137742041
P3_5	iso-SNP	134884723
P3_5	iso-SNP	134884700
P3_5	iso-SNP	134884697
P3_5	iso-SNP	97885708
P3_5	iso-SNP	74110335
P3_5	iso-SNP	74110328
P3_5	iso-SNP	134884737

P3_5	iso-SNP	41488568
P3_5	iso-SNP	2633462
P3_5	iso-SNP	97885715
P3_5	iso-SNP	113569211
P3_5	iso-SNP	113569207
P3_5	iso-SNP	113569193
P3_5	iso-SNP	41517974
P3_5	iso-SNP	41518005
P3_5	iso-SNP	41518007
P3_5	iso-SNP	41518025
P3_5	iso-SNP	220771223
P3_5	iso-SNP	179225301
P3_5	iso-SNP	179225324
P3_5	iso-SNP	72113306
P3_5	iso-SNP	72113270
P3_5	iso-SNP	72113269
P3_5	iso-SNP	72113261
P3_5	iso-SNP	10341137
P3_5	iso-SNP	79099712
P3_5	iso-SNP	79099736
P3_5	iso-SNP	26946325
P3_5	iso-SNP	41517981
P3_5	iso-SNP	61151515
P3_5	iso-SNP	61151573
P3_5	iso-SNP	2633478
P3_5	iso-SNP	117214376
P3_5	iso-SNP	10928149
P3_5	iso-SNP	10928119
P3_5	iso-SNP	10928130
P3_5	iso-SNP	101491378
P3_5	iso-SNP	79813075
P3_5	iso-SNP	79813049
P3_5	iso-SNP	6489914
P3_5	iso-SNP	6489910
P3_5	iso-SNP	133303465
P3_5	iso-SNP	133303425
P3_5	iso-SNP	101521092
P3_5	iso-SNP	69094259
P3_5	iso-SNP	69094207
P3_5	iso-SNP	101488431
P3_5	iso-SNP	101488414
P3_5	iso-SNP	101488424
P3_5	iso-SNP	101488430
P3_5	iso-SNP	2155409
P3_5	iso-SNP	249120591
P3_5	iso-SNP	249120610
P3_5	iso-SNP	249120578

P3_5	iso-SNP	189842885
P3_5	iso-SNP	104166899
P3_5	iso-SNP	104166902
P3_5	iso-SNP	104166904
P3_5	iso-SNP	167967904
P3_5	iso-SNP	144895169
P3_5	iso-SNP	57588322
P3_5	iso-SNP	79099750
P3_5	iso-SNP	189842826
P3_5	iso-SNP	189842822
P3_5	iso-SNP	95604259
P3_5	iso-SNP	133303896
P3_5	iso-SNP	101512288
P3_5	iso-SNP	101512304
P3_5	iso-SNP	15935347
P3_5	iso-SNP	35237143
P3_5	iso-SNP	52196045
P3_5	iso-SNP	52196076
P3_5	iso-SNP	10682907
P3_5	iso-SNP	1785038
P3_5	iso-SNP	1785015
P3_5	iso-SNP	1769533
P3_5	iso-SNP	1785037
P3_5	iso-SNP	59362602
P3_5	iso-SNP	10682950
P3_5	iso-SNP	19745034
P3_5	iso-SNP	19745003
P3_5	iso-SNP	10682894
P3_5	iso-SNP	10682934
P3_5	iso-SNP	19745016
P3_5	iso-SNP	185243724
P3_5	iso-SNP	149112399
P3_5	iso-SNP	146341217
P3_5	iso-SNP	145625542
P3_5	iso-SNP	145625537
P3_5	iso-SNP	47891069
P3_5	iso-SNP	46509616
P3_5	iso-SNP	46509569
P3_5	iso-SNP	1103312
P3_5	iso-SNP	1103328
P3_5	iso-SNP	1103331
P3_5	iso-SNP	54201692
P3_5	iso-SNP	54201654
P3_5	iso-SNP	54201667
P3_5	iso-SNP	54201668
P3_5	iso-SNP	47891106
P3_5	iso-SNP	197401372

P3_5	iso-SNP	197401443
P3_5	iso-SNP	197401442
P3_5	iso-SNP	197401415
P3_5	iso-SNP	101498399
P3_5	iso-SNP	101498381
P3_5	iso-SNP	25551588
P3_5	iso-SNP	25551550
P3_5	iso-SNP	136983326
P3_5	iso-SNP	136983319
P3_5	iso-SNP	104196269
P3_5	iso-SNP	15935316
P3_5	iso-SNP	47891117
P3_5	iso-SNP	42296995
P3_5	iso-SNP	112273768
P3_5	iso-SNP	113569054
P3_5	iso-SNP	25551583
P3_5	iso-SNP	145625535
P3_5	iso-SNP	145625536
P3_5	iso-SNP	49057623
P3_5	iso-SNP	169455539
P3_5	iso-SNP	169455502
P3_5	iso-SNP	169455551
P3_5	iso-SNP	57228574
P3_5	iso-SNP	57228573
P3_5	iso-SNP	110141578
P3_5	iso-SNP	166922916
P3_5	iso-SNP	67236292
P3_5	iso-SNP	179225317
P3_5	iso-SNP	101521052
P3_5	iso-SNP	135817150
P3_5	iso-SNP	2018019
P3_5	iso-SNP	2018056
P3_5	iso-SNP	146353894
P3_5	iso-SNP	101506465
P3_5	iso-SNP	101506441
P3_5	iso-SNP	166922908
P3_5	iso-SNP	22007634
P3_5	iso-SNP	57918678
P3_5	iso-SNP	137749929
P3_5	iso-SNP	33578205
P3_5	iso-SNP	33578255
P3_5	iso-SNP	33578206
P3_5	iso-SNP	124451312
P3_5	iso-SNP	127847960
P3_5	iso-SNP	219267371
P3_5	iso-SNP	35696518
P3_5	iso-SNP	35696481

P3_5	iso-SNP	62544486
P3_5	iso-SNP	62544495
P3_5	iso-SNP	62544490
P3_5	iso-SNP	219267370
P3_5	iso-SNP	117520360
P3_5	iso-SNP	117520389
P3_5	iso-SNP	219267402
P3_5	iso-SNP	219267407
P3_5	iso-SNP	41522207
P3_5	iso-SNP	101520657
P3_5	iso-SNP	101520687
P3_5	iso-SNP	33578251
P3_5	iso-SNP	124451357
P3_5	iso-SNP	62544503
P3_5	iso-SNP	117886991
P3_5	iso-SNP	62544470
P3_5	iso-SNP	35696519
P3_5	iso-SNP	35696532
P3_5	iso-SNP	117637325
P3_5	iso-SNP	117637326
P3_5	iso-SNP	117637302
P3_5	iso-SNP	129414568
P3_5	iso-SNP	145074342
P3_5	iso-SNP	145074272
P3_5	iso-SNP	1988176
P3_5	iso-SNP	1988144
P3_5	iso-SNP	54219854
P3_5	iso-SNP	54219870
P3_5	iso-SNP	54219904
P3_5	iso-SNP	54219912
P3_5	iso-SNP	101507720
P3_5	iso-SNP	10080309
P3_5	iso-SNP	6920975
P3_5	iso-SNP	54219857
P3_5	iso-SNP	52196528
P3_5	iso-SNP	52196574
P3_5	iso-SNP	2155379
P3_5	iso-SNP	151127092
P3_5	iso-SNP	151127105
P3_5	iso-SNP	101507711
P3_5	iso-SNP	113997746
P3_5	iso-SNP	101500126
P3_5	iso-SNP	101500098
P3_5	iso-SNP	1816176
P3_5	iso-SNP	56408638
P3_5	iso-SNP	56408639
P3_5	iso-SNP	101507744

P3_5	iso-SNP	101507727
P3_5	iso-SNP	145074283
P3_5	iso-SNP	176032424
P3_5	iso-SNP	54170016
P3_5	iso-SNP	54169954
P3_5	iso-SNP	54169973
P3_5	iso-SNP	54170006
P3_5	iso-SNP	143424215
P3_5	iso-SNP	101518827
P3_5	iso-SNP	101531654
P3_5	iso-SNP	1816169
P3_5	iso-SNP	143424198
P3_5	iso-SNP	21038172
P3_5	iso-SNP	28444157
P3_5	iso-SNP	8007067
P3_5	iso-SNP	8007039
P3_5	iso-SNP	8007037
P3_5	iso-SNP	8007066
P3_5	iso-SNP	143163772
P3_5	iso-SNP	143163775
P3_5	iso-SNP	21038150
P3_5	iso-SNP	143163790
P3_5	iso-SNP	143163799
P3_5	iso-SNP	143163816
P3_5	iso-SNP	27559214
P3_5	iso-SNP	27559261
P3_5	iso-SNP	22049282
P3_5	iso-SNP	176032428
P3_5	iso-SNP	28444104
P3_5	iso-SNP	19405702
P3_5	iso-SNP	19405743
P3_5	iso-SNP	145078244
P3_5	iso-SNP	62997470
P3_5	iso-SNP	101280841
P3_5	iso-SNP	101280828
P3_5	iso-SNP	188406598
P3_5	iso-SNP	14403144
P3_5	iso-SNP	188406636
P3_5	iso-SNP	104583796
P3_5	iso-SNP	101280856
P3_5	iso-SNP	101280892
P3_5	iso-SNP	61582680
P3_5	iso-SNP	104583838
P3_5	iso-SNP	55344901
P3_5	iso-SNP	54200834
P3_5	iso-SNP	54200810
P3_5	iso-SNP	54200826

P3_5	iso-SNP	54200830
P3_5	iso-SNP	99691401
P3_5	iso-SNP	99691396
P3_5	iso-SNP	99691429
P3_5	iso-SNP	149396272
P3_5	iso-SNP	61582708
P3_5	iso-SNP	61582659
P3_5	iso-SNP	61582649
P3_5	iso-SNP	45659500
P3_5	iso-SNP	22102519
P3_5	iso-SNP	22102546
P3_5	iso-SNP	22102543
P3_5	iso-SNP	22102542
P3_5	iso-SNP	143424165
P3_5	iso-SNP	54203326
P3_5	iso-SNP	54203333
P3_5	iso-SNP	54203347
P3_5	iso-SNP	153410531
P3_5	iso-SNP	150524468
P3_5	iso-SNP	54251937
P3_5	iso-SNP	18451325
P3_5	iso-SNP	18451300
P3_5	iso-SNP	45659490
P3_5	iso-SNP	144895170
P3_5	iso-SNP	101377478
P3_5	iso-SNP	145619405
P3_5	iso-SNP	145619442
P3_5	iso-SNP	145619377
P3_5	iso-SNP	145619386
P3_5	iso-SNP	151560719
P3_5	iso-SNP	151560699
P3_5	iso-SNP	151562938
P3_5	iso-SNP	91352580
P3_5	iso-SNP	13947436
P3_5	iso-SNP	136422988
P3_5	iso-SNP	136423009
P3_5	iso-SNP	49812123
P3_5	iso-SNP	14830197
P3_5	iso-SNP	14830201
P3_5	iso-SNP	14830215
P3_5	iso-SNP	14830216
P3_5	iso-SNP	101341408
P3_5	iso-SNP	101341373
P3_5	iso-SNP	101341407
P3_5	iso-SNP	69330823
P3_5	iso-SNP	54251902
P3_5	iso-SNP	69330818

P3_5	iso-SNP	96085190
P3_5	iso-SNP	54197660
P3_5	iso-SNP	54197674
P3_5	iso-SNP	54197678
P3_5	iso-SNP	54197706
P3_5	iso-SNP	21038166
P3_5	iso-SNP	69330886
P3_5	iso-SNP	69330825
P3_5	iso-SNP	69330824
P3_5	iso-SNP	142667306
P3_5	iso-SNP	142667330
P3_5	iso-SNP	142667356
P3_5	iso-SNP	117886982
P3_5	iso-SNP	54234260
P3_5	iso-SNP	159869828
P3_5	iso-SNP	43602984
P3_5	iso-SNP	56892431
P3_5	iso-SNP	56892464
P3_5	iso-SNP	56892470
P3_5	iso-SNP	56892507
P3_5	iso-SNP	32659592
P3_5	iso-SNP	57588323
P3_5	iso-SNP	103361221
P3_5	iso-SNP	101515025
P3_5	iso-SNP	57588336
P3_5	iso-SNP	93113326
P3_5	iso-SNP	93113323
P3_5	iso-SNP	93113314
P3_5	iso-SNP	93113270
P3_5	iso-SNP	130548214
P3_5	iso-SNP	130548199
P3_5	iso-SNP	149396242
P3_5	iso-SNP	28863695
P3_5	iso-SNP	28863633
P3_5	iso-SNP	28863628
P3_5	iso-SNP	85775275
P3_5	iso-SNP	20020733
P3_5	iso-SNP	2633466
P3_5	iso-SNP	2633480
P3_5	iso-SNP	2633434
P3_5	iso-SNP	62572885
P3_5	iso-SNP	149396273
P3_5	iso-SNP	149396288
P3_5	iso-SNP	54198496
P3_5	iso-SNP	146280644
P3_5	iso-SNP	146280626
P3_5	iso-SNP	54194135

P3_5	iso-SNP	146271304
P3_5	iso-SNP	139565064
P3_5	iso-SNP	139565098
P3_5	iso-SNP	139565119
P3_5	iso-SNP	139565134
P3_5	iso-SNP	142667289
P3_5	iso-SNP	101522578
P3_5	iso-SNP	101522582
P3_5	iso-SNP	101522589
P3_5	iso-SNP	101522631
P3_5	iso-SNP	144895179
P3_5	iso-SNP	54172508
P3_5	iso-SNP	54172483
P3_5	iso-SNP	54172501
P3_5	iso-SNP	101531849
P3_5	iso-SNP	101531854
P3_5	iso-SNP	101531857
P3_5	iso-SNP	101531858
P3_5	iso-SNP	232578091
P3_5	iso-SNP	232578032
P3_5	iso-SNP	232578048
P3_5	iso-SNP	126858425
P3_5	iso-SNP	17962615
P3_5	iso-SNP	85775260
P3_5	iso-SNP	31203277
P3_5	iso-SNP	144895159
P3_5	iso-SNP	54215584
P3_5	iso-SNP	54215608
P3_5	iso-SNP	144895168
P3_5	iso-SNP	146340339
P3_5	iso-SNP	146340360
P3_5	iso-SNP	130135961
P3_5	iso-SNP	1785030
P3_5	iso-SNP	10436187
P3_5	iso-SNP	10436238
P3_5	iso-SNP	10436194
P3_5	iso-SNP	10436201
P3_5	iso-SNP	10436219
P3_5	iso-SNP	146340304
P3_5	iso-SNP	146340278
P3_5	iso-SNP	73438224
P3_5	iso-SNP	17717244
P3_5	iso-SNP	17717243
P3_5	iso-SNP	17717209
P3_5	iso-SNP	101531806
P3_5	iso-SNP	56408625
P3_5	iso-SNP	56408599

P3_5	iso-SNP	159000436
P3_5	iso-SNP	57392697
P3_5	iso-SNP	16645208
P3_5	iso-SNP	101526116
P3_5	iso-SNP	126858406
P3_5	iso-SNP	79502141
P3_5	iso-SNP	2140267
P3_5	iso-SNP	2140270
P3_5	iso-SNP	2140268
P3_5	iso-SNP	74110353
P3_5	iso-SNP	74110358
P3_5	iso-SNP	75679969
P3_5	iso-SNP	75679923
P3_5	iso-SNP	75679958
P3_5	iso-SNP	56216187
P3_5	iso-SNP	56216090
P3_5	iso-SNP	101280859
P3_5	iso-SNP	104985443
P3_5	iso-SNP	241395420
P3_5	iso-SNP	101530833
P3_5	iso-SNP	6194231
P3_5	iso-SNP	65523519
P3_5	iso-SNP	101530834
P3_5	iso-SNP	133674285
P3_5	iso-SNP	126858392
P3_5	iso-SNP	65523500
P3_5	iso-SNP	43602992
P3_5	iso-SNP	113655748
P3_5	iso-SNP	113655752
P3_5	iso-SNP	113655794
P3_5	iso-SNP	113655806
P3_5	iso-SNP	46114572
P3_5	iso-SNP	46114610
P3_5	iso-SNP	49773087
P3_5	iso-SNP	49773042
P3_5	iso-SNP	57392715
P3_5	iso-SNP	100774203
P3_5	iso-SNP	100774268
P3_5	iso-SNP	100774277
P3_5	iso-SNP	100774289
P3_5	iso-SNP	79502168
P3_5	iso-SNP	18392894
P3_5	iso-SNP	18392913
P3_5	iso-SNP	77879071
P3_5	iso-SNP	54234265
P3_5	iso-SNP	176998548
P3_5	iso-SNP	54234315

P3_5	iso-SNP	54234340
P3_5	iso-SNP	157367104
P3_5	iso-SNP	69002317
P3_5	iso-SNP	69002320
P3_5	iso-SNP	69002304
P3_5	iso-SNP	69002278
P3_5	iso-SNP	101347355
P3_5	iso-SNP	101347408
P3_5	iso-SNP	104583828
P3_5	iso-SNP	104583804
P3_5	iso-SNP	241395500
P3_5	iso-SNP	241395503
P3_5	iso-SNP	49768168
P3_5	iso-SNP	133675375
P3_5	iso-SNP	116971778
P3_5	iso-SNP	101335438
P3_5	iso-SNP	145619365
P3_5	iso-SNP	113886031
P3_5	iso-SNP	52009178
P3_5	iso-SNP	146342080
P3_5	iso-SNP	146342071
P3_5	iso-SNP	179442344
P3_5	iso-SNP	18134045
P3_5	iso-SNP	18134042
P3_5	iso-SNP	63116226
P3_5	iso-SNP	18134091
P3_5	iso-SNP	54194212
P3_5	iso-SNP	54255679
P3_5	iso-SNP	24736638
P3_5	iso-SNP	101521764
P3_5	iso-SNP	24736590
P3_5	iso-SNP	103361245
P3_5	iso-SNP	52009220
P3_5	iso-SNP	20716104
P3_5	iso-SNP	20716128
P3_5	iso-SNP	103361226
P3_5	iso-SNP	2321762
P3_5	iso-SNP	2321809
P3_5	iso-SNP	2321792
P3_5	iso-SNP	183959232
P3_5	iso-SNP	183959229
P3_5	iso-SNP	2321774
P3_5	iso-SNP	54228742
P3_5	iso-SNP	54228719
P3_5	iso-SNP	101522556
P3_5	iso-SNP	130496365
P3_5	iso-SNP	54265617

P3_5	iso-SNP	54265626
P3_5	iso-SNP	54265658
P3_5	iso-SNP	54265670
P3_5	iso-SNP	54254543
P3_5	iso-SNP	54254494
P3_5	iso-SNP	59463460
P3_5	iso-SNP	59463436
P3_5	iso-SNP	59463389
P3_5	iso-SNP	63005918
P3_5	iso-SNP	54185441
P3_5	iso-SNP	54185457
P3_5	iso-SNP	54185481
P3_5	iso-SNP	54185492
P3_5	iso-SNP	54189751
P3_5	iso-SNP	54189752
P3_5	iso-SNP	54201695
P3_5	iso-SNP	54201703
P3_5	iso-SNP	6920976
P3_5	iso-SNP	6920955
P3_5	iso-SNP	54242630
P3_5	iso-SNP	19263542
P3_5	iso-SNP	28444183
P3_5	iso-SNP	54214312
P3_5	iso-SNP	54214333
P3_5	iso-SNP	54214286
P3_5	iso-SNP	54206051
P3_5	iso-SNP	54210734
P3_5	iso-SNP	54210736
P3_5	iso-SNP	54210774
P3_5	iso-SNP	69002294
P3_5	iso-SNP	7461769
P3_5	iso-SNP	69002271
P3_5	iso-SNP	54240174
P3_5	iso-SNP	54240184
P3_5	iso-SNP	54240136
P3_5	iso-SNP	57215120
P3_5	iso-SNP	57215164
P3_5	iso-SNP	7461826
P3_5	iso-SNP	7461827
P3_5	iso-SNP	2321820
P3_5	iso-SNP	146342052
P3_5	iso-SNP	97824145
P3_5	iso-SNP	97824129
P3_5	iso-SNP	97824125
P3_5	iso-SNP	154065370
P3_5	iso-SNP	154065368
P3_5	iso-SNP	154065347

P3_5	iso-SNP	35237129
P3_5	iso-SNP	154065348
P3_5	iso-SNP	146312590
P3_5	iso-SNP	49774381
P3_5	iso-SNP	97824075
P3_5	iso-SNP	145076355
P3_5	iso-SNP	146312541
P3_5	iso-SNP	146312589
P3_5	iso-SNP	146312560
P3_5	iso-SNP	145076376
P3_5	iso-SNP	54228750
P3_5	iso-SNP	54228743
P3_5	iso-SNP	101530873
P3_5	iso-SNP	59362576
P3_5	iso-SNP	49774383
P3_5	iso-SNP	171070937
P3_5	iso-SNP	171070888
P3_5	iso-SNP	171070875
P3_5	iso-SNP	49774389
P3_5	iso-SNP	20073323
P3_5	iso-SNP	21510699
P3_5	iso-SNP	21510716
P3_5	iso-SNP	10662844
P3_5	iso-SNP	10662859
P3_5	iso-SNP	73402156
P3_5	iso-SNP	10662866
P3_5	iso-SNP	41522213
P3_5	iso-SNP	151128173
P3_5	iso-SNP	34674345
P3_5	iso-SNP	34674326
P3_5	iso-SNP	34820566
P3_5	iso-SNP	34820547
P3_5	iso-SNP	54228775
P3_5	iso-SNP	41522221
P3_5	iso-SNP	153410482
P3_5	iso-SNP	153410520
P3_5	iso-SNP	14995387
P3_5	iso-SNP	54240142
P3_5	iso-SNP	41220094
P3_5	iso-SNP	54225437
P3_5	iso-SNP	54225460
P3_5	iso-SNP	54225463
P3_5	iso-SNP	54225490
P3_5	iso-SNP	54225501
P3_5	iso-SNP	54245788
P3_5	iso-SNP	54245789
P3_5	iso-SNP	54245827

P3_5	iso-SNP	54245841
P3_5	iso-SNP	54228774
P3_5	iso-SNP	145019437
P3_5	iso-SNP	145019401
P3_5	iso-SNP	145019377
P3_5	iso-SNP	145019376
P3_5	iso-SNP	54245780
P3_5	iso-SNP	54225426
P3_5	iso-SNP	77879051
P3_5	iso-SNP	19405709
P3_5	iso-SNP	101531862
P3_5	iso-SNP	18572070
P3_5	iso-SNP	18572056
P3_5	iso-SNP	46114580
P3_5	iso-SNP	160122421
P3_5	iso-SNP	31203228
P3_5	iso-SNP	159912436
P3_5	iso-SNP	31203221
P3_5	iso-SNP	89312487
P3_5	iso-SNP	89312451
P3_5	iso-SNP	131154943
P3_5	iso-SNP	146360826
P3_5	iso-SNP	146360813
P3_5	iso-SNP	146360779
P3_5	iso-SNP	146360778
P3_5	iso-SNP	593362
P3_5	iso-SNP	160122434
P3_5	iso-SNP	159912418
P3_5	iso-SNP	64658828
P3_5	iso-SNP	64136103
P3_5	iso-SNP	64136145
P3_5	iso-SNP	64136160
P3_5	iso-SNP	64136167
P3_5	iso-SNP	64136170
P3_5	iso-SNP	131701185
P3_5	iso-SNP	131701279
P3_5	iso-SNP	133303744
P3_5	iso-SNP	19744982
P3_5	iso-SNP	131007109
P3_5	iso-SNP	131007052
P3_5	iso-SNP	131007087
P3_5	iso-SNP	79107068
P3_5	iso-SNP	189997834
P3_5	iso-SNP	189997816
P3_5	iso-SNP	54260009
P3_5	iso-SNP	54260002
P3_5	iso-SNP	131007004

P3_5	iso-SNP	101532279
P3_5	iso-SNP	46142293
P3_5	iso-SNP	54240137
P3_5	iso-SNP	72744805
P3_5	iso-SNP	93466919
P3_5	iso-SNP	93466912
P3_5	iso-SNP	93466910
P3_5	iso-SNP	93466909
P3_5	iso-SNP	93466866
P3_5	iso-SNP	54216615
P3_5	iso-SNP	55886581
P3_5	iso-SNP	55886608
P3_5	iso-SNP	154065385
P3_5	iso-SNP	29887033
P3_5	iso-SNP	54260068
P3_5	iso-SNP	54260075
P3_5	iso-SNP	54264394
P3_5	iso-SNP	54264421
P3_5	iso-SNP	54264461
P3_5	iso-SNP	54264468
P3_5	iso-SNP	64658836
P3_5	iso-SNP	59463451
P3_5	iso-SNP	101500133
P3_5	iso-SNP	101500166
P3_5	iso-SNP	101500167
P3_5	iso-SNP	54264462
P3_5	iso-SNP	135812836
P3_5	iso-SNP	132763295
P3_5	iso-SNP	132763362
P3_5	iso-SNP	132763335
P3_5	iso-SNP	109757963
P3_5	iso-SNP	132763305
P3_5	iso-SNP	132763298
P3_5	iso-SNP	97847807
P3_5	iso-SNP	87909673
P3_5	iso-SNP	86584720
P3_5	iso-SNP	54385558
P3_5	iso-SNP	54385561
P3_5	iso-SNP	54385584
P3_5	iso-SNP	54385599
P3_5	iso-SNP	54385629
P3_5	iso-SNP	101340844
P3_5	iso-SNP	101340862
P3_5	iso-SNP	87909716
P3_5	iso-SNP	87909749
P3_5	iso-SNP	87909701
P3_5	iso-SNP	119390286

P3_5	iso-SNP	119390294
P3_5	iso-SNP	41220077
P3_5	iso-SNP	146271303
P3_5	iso-SNP	146271269
P3_5	iso-SNP	146271266
P3_5	iso-SNP	146271235
P3_5	iso-SNP	146271231
P3_5	iso-SNP	54216616
P3_5	iso-SNP	54216650
P3_5	iso-SNP	54216629
P3_5	iso-SNP	241295614
P3_5	iso-SNP	119390263
P3_5	iso-SNP	101512836
P3_5	iso-SNP	114058104
P3_5	iso-SNP	6921289
P3_5	iso-SNP	167411298
P3_5	iso-SNP	167411388
P3_5	iso-SNP	189547778
P3_5	iso-SNP	189547779
P3_5	iso-SNP	189547761
P3_5	iso-SNP	18572073
P3_5	iso-SNP	189547748
P3_5	iso-SNP	54245768
P3_5	iso-SNP	100155033
P3_5	iso-SNP	100155019
P3_5	iso-SNP	100154978
P3_5	iso-SNP	133674439
P3_5	iso-SNP	133674418
P3_5	iso-SNP	133674399
P3_5	iso-SNP	167411334
P3_5	iso-SNP	167411300
P3_5	iso-SNP	167411301
P3_5	iso-SNP	148810267
P3_5	iso-SNP	820192
P3_5	iso-SNP	132763351
P3_5	iso-SNP	129414843
P3_5	iso-SNP	129414806
P3_5	iso-SNP	129414852
P3_5	iso-SNP	129414815
P3_5	iso-SNP	129414807
P3_5	iso-SNP	101491923
P3_5	iso-SNP	101491974
P3_5	iso-SNP	101491981
P3_5	iso-SNP	167411333
P3_5	iso-SNP	147075166
P3_5	iso-SNP	147075204
P3_5	iso-SNP	29902536

P3_5	iso-SNP	820215
P3_5	iso-SNP	820249
P3_5	iso-SNP	54244647
P3_5	iso-SNP	92956443
P3_5	iso-SNP	9211802
P3_5	iso-SNP	9211778
P3_5	iso-SNP	9211747
P3_5	iso-SNP	9211738
P3_5	iso-SNP	104583759
P3_5	iso-SNP	104583776
P3_5	iso-SNP	170813684
P3_5	iso-SNP	54198499
P3_5	iso-SNP	1785042
P3_5	iso-SNP	1785060
P3_5	iso-SNP	70718466
P3_5	iso-SNP	97847498
P3_5	iso-SNP	56367696
P3_5	iso-SNP	133674612
P3_5	iso-SNP	133674576
P3_5	iso-SNP	61162131
P3_5	iso-SNP	61162191
P3_5	iso-SNP	133014640
P3_5	iso-SNP	133014633
P3_5	iso-SNP	101036259
P3_5	iso-SNP	135061111
P3_5	iso-SNP	135061109
P3_5	iso-SNP	33175642
P3_5	iso-SNP	33175702
P3_5	iso-SNP	231155644
P3_5	iso-SNP	231155581
P3_5	iso-SNP	101491941
P3_5	iso-SNP	31357325
P3_5	iso-SNP	54211990
P3_5	iso-SNP	54212075
P3_5	iso-SNP	45605666
P3_5	iso-SNP	135061124
P3_5	iso-SNP	38554942
P3_5	iso-SNP	31357245
P3_5	iso-SNP	31357244
P3_5	iso-SNP	31357238
P3_5	iso-SNP	100575791
P3_5	iso-SNP	118927256
P3_5	iso-SNP	112475403
P3_5	iso-SNP	112475427
P3_5	iso-SNP	112475429
P3_5	iso-SNP	100575818
P3_5	iso-SNP	67142564

P3_5	iso-SNP	67142583
P3_5	iso-SNP	67142619
P3_5	iso-SNP	67142620
P3_5	iso-SNP	593323
P3_5	iso-SNP	133014619
P3_5	iso-SNP	232578049
P3_5	iso-SNP	20529956
P3_5	iso-SNP	20529990
P3_5	iso-SNP	133014579
P3_5	iso-SNP	133014612
P3_5	iso-SNP	44155749
P3_5	iso-SNP	26906471
P3_5	iso-SNP	26906452
P3_5	iso-SNP	26906437
P3_5	iso-SNP	26906423
P3_5	iso-SNP	26906403
P3_5	iso-SNP	26906402
P3_5	iso-SNP	44155754
P3_5	iso-SNP	129410228
P3_5	iso-SNP	129410313
P3_5	iso-SNP	129410239
P3_5	iso-SNP	129410235
P3_5	iso-SNP	150935522
P3_5	iso-SNP	33484837
P3_5	iso-SNP	54255689
P3_5	iso-SNP	33484783
P3_5	iso-SNP	228284991
P3_5	iso-SNP	33798031
P3_5	iso-SNP	172113756
P3_5	iso-SNP	24214442
P3_5	iso-SNP	24214486
P3_5	iso-SNP	24214493
P3_5	iso-SNP	24214532
P3_5	iso-SNP	46522201
P3_5	iso-SNP	46522255
P3_5	iso-SNP	46522298
P3_5	iso-SNP	64658710
P3_5	iso-SNP	177015045
P3_5	iso-SNP	92956422
P3_5	iso-SNP	70480088
P3_5	iso-SNP	70480127
P3_5	iso-SNP	70480108
P3_5	iso-SNP	64136130
P3_5	iso-SNP	54178990
P3_5	iso-SNP	54179024
P3_5	iso-SNP	54179044
P3_5	iso-SNP	100125949

P3_5	iso-SNP	100125939
P3_5	iso-SNP	100125865
P3_5	iso-SNP	100125848
P3_5	iso-SNP	21314920
P3_5	iso-SNP	21314829
P3_5	iso-SNP	56210210
P3_5	iso-SNP	56210207
P3_5	iso-SNP	29729163
P3_5	iso-SNP	198828005
P3_5	iso-SNP	198828096
P3_5	iso-SNP	76139740
P3_5	iso-SNP	136587919
P3_5	iso-SNP	136587934
P3_5	iso-SNP	136588019
P3_5	iso-SNP	45606472
P3_5	iso-SNP	45606471
P3_5	iso-SNP	45606510
P3_5	iso-SNP	54177532
P3_5	iso-SNP	54177552
P3_5	iso-SNP	54177523
P3_5	iso-SNP	54177559
P3_5	iso-SNP	54177564
P3_5	iso-SNP	54177457
P3_5	iso-SNP	33578276
P3_5	iso-SNP	33578201
P3_5	iso-SNP	45606504
P3_5	iso-SNP	13985721
P3_5	iso-SNP	13985739
P3_5	iso-SNP	13985772
P3_5	iso-SNP	13985805
P3_5	iso-SNP	13985806
P3_5	iso-SNP	114340663
P3_5	iso-SNP	209605539
P3_5	iso-SNP	209605546
P3_5	iso-SNP	2140269
P3_5	iso-SNP	65523495
P3_5	iso-SNP	101348275
P3_5	iso-SNP	105154084
P3_5	iso-SNP	105154091
P3_5	iso-SNP	105154089
P3_5	iso-SNP	105154097
P3_5	iso-SNP	209605507
P3_5	iso-SNP	2186069
P3_5	iso-SNP	2186129
P3_5	iso-SNP	2186117
P3_5	iso-SNP	2186087
P3_5	iso-SNP	2186054

P3_5	iso-SNP	15248787
P3_5	iso-SNP	15248717
P3_5	iso-SNP	15248726
P3_5	iso-SNP	15248720
P3_5	iso-SNP	15248801
P3_5	iso-SNP	15248798
P3_5	iso-SNP	2186044
P3_5	iso-SNP	33578202
P3_5	iso-SNP	113132901
P3_5	iso-SNP	113132857
P3_5	iso-SNP	153726707
P3_5	iso-SNP	153726728
P3_5	iso-SNP	153726756
P3_5	iso-SNP	153726760
P3_5	iso-SNP	153726769
P3_5	iso-SNP	49231303
P3_5	iso-SNP	49231248
P3_5	iso-SNP	208134116
P3_5	iso-SNP	208134114
P3_5	iso-SNP	208134058
P3_5	iso-SNP	208134047
P3_5	iso-SNP	208134017
P3_5	iso-SNP	20035242
P3_5	iso-SNP	64658705
P3_5	iso-SNP	40646816
P3_5	iso-SNP	41488558
P3_5	iso-SNP	41488567
P3_5	iso-SNP	97885730
P3_5	iso-SNP	97885720
P3_5	iso-SNP	101491407
P3_5	iso-SNP	79813083
P3_5	iso-SNP	134884717
P3_5	iso-SNP	249120640
P3_5	iso-SNP	167967953
P3_5	iso-SNP	167967958
P3_5	iso-SNP	167967963
P3_5	iso-SNP	15935351
P3_5	iso-SNP	136983281
P3_5	iso-SNP	136983275
P3_5	iso-SNP	104196300
P3_5	iso-SNP	166922862
P3_5	iso-SNP	2018004
P3_5	iso-SNP	2018002
P3_5	iso-SNP	197401371
P3_5	iso-SNP	219267433
P3_5	iso-SNP	219267443
P3_5	iso-SNP	1988193

P3_5	iso-SNP	145074289
P3_5	iso-SNP	1988170
P3_5	iso-SNP	1988119
P3_5	iso-SNP	113997768
P3_5	iso-SNP	113997812
P3_5	iso-SNP	113997817
P3_5	iso-SNP	176032376
P3_5	iso-SNP	129414574
P3_5	iso-SNP	19405676
P3_5	iso-SNP	19405672
P3_5	iso-SNP	101507779
P3_5	iso-SNP	54200843
P3_5	iso-SNP	22102536
P3_5	iso-SNP	54200853
P3_5	iso-SNP	144895164
P3_5	iso-SNP	69330871
P3_5	iso-SNP	62572874
P3_5	iso-SNP	31203216
P3_5	iso-SNP	31203267
P3_5	iso-SNP	10436180
P3_5	iso-SNP	57392678
P3_5	iso-SNP	57392686
P3_5	iso-SNP	16645199
P3_5	iso-SNP	2140204
P3_5	iso-SNP	2140240
P3_5	iso-SNP	232578099
P3_5	iso-SNP	49768171
P3_5	iso-SNP	101335451
P3_5	iso-SNP	113886051
P3_5	iso-SNP	2321827
P3_5	iso-SNP	154065383
P3_5	iso-SNP	146312517
P3_5	iso-SNP	145076302
P3_5	iso-SNP	77879011
P3_5	iso-SNP	31203207
P3_5	iso-SNP	146360765
P3_5	iso-SNP	79107049
P3_5	iso-SNP	79107061
P3_5	iso-SNP	131007001
P3_5	iso-SNP	55886574
P3_5	iso-SNP	55886522
P3_5	iso-SNP	86584707
P3_5	iso-SNP	54216670
P3_5	iso-SNP	241295617
P3_5	iso-SNP	8007104
P3_5	iso-SNP	147075152
P3_5	iso-SNP	820265

P3_5	iso-SNP	92956420
P3_5	iso-SNP	92956416
P3_5	iso-SNP	92956412
P3_5	iso-SNP	92956409
P3_5	iso-SNP	97847569
P3_5	iso-SNP	97847573
P3_5	iso-SNP	135061039
P3_5	iso-SNP	101491965
P3_5	iso-SNP	100575762
P3_5	iso-SNP	129410227
P3_5	iso-SNP	150935577
P3_5	iso-SNP	64658640
P3_5	iso-SNP	64658623
P3_5	iso-SNP	70480062
P3_5	iso-SNP	56210140
P3_5	iso-SNP	56210168
P3_5	iso-SNP	56210117
P3_5	iso-SNP	114340631
P3_5	iso-SNP	114340550
P3_5	iso-SNP	114340615
P3_5	iso-SNP	153726803
P3_5	iso-SNP	40646834
P3_5	iso-SNP	145074284
P3_5	iso-SNP	189547735
P3_5	iso-SNP	1988116
P3_5	iso-SNP	16645178
P3_5	iso-SNP	79107017
P3_5	iso-SNP	54216681
P3_5	iso-SNP	147075121
P3_5	iso-SNP	147075120
P3_5	iso-SNP	100575761
P3_5	iso-SNP	129414804
P3_5	iso-SNP	105154013
P3_5	iso-SNP	70480051
P3_3	nor-pre-miRNA>hsa-mir-101-2	
P3_3	nor-pre-miRNA>hsa-mir-10a	
P3_3	nor-pre-miRNA>hsa-mir-10b	
P3_3	nor-pre-miRNA>hsa-mir-1178	
P3_3	nor-pre-miRNA>hsa-mir-1197	
P3_3	nor-pre-miRNA>hsa-mir-1207	
P3_3	nor-pre-miRNA>hsa-mir-1233-1	
P3_3	nor-pre-miRNA>hsa-mir-1233-2	
P3_3	nor-pre-miRNA>hsa-mir-124-3	
P3_3	nor-pre-miRNA>hsa-mir-1245b	
P3_3	nor-pre-miRNA>hsa-mir-1286	
P3_3	nor-pre-miRNA>hsa-mir-1292	
P3_3	nor-pre-miRNA>hsa-mir-1295a	

P3_3 nor-pre-miRNA>hsa-mir-1303
P3_3 nor-pre-miRNA>hsa-mir-130b
P3_3 nor-pre-miRNA>hsa-mir-133a-1
P3_3 nor-pre-miRNA>hsa-mir-136
P3_3 nor-pre-miRNA>hsa-mir-140
P3_3 nor-pre-miRNA>hsa-mir-141
P3_3 nor-pre-miRNA>hsa-mir-145
P3_3 nor-pre-miRNA>hsa-mir-154
P3_3 nor-pre-miRNA>hsa-mir-15b
P3_3 nor-pre-miRNA>hsa-mir-183
P3_3 nor-pre-miRNA>hsa-mir-190a
P3_3 nor-pre-miRNA>hsa-mir-1914
P3_3 nor-pre-miRNA>hsa-mir-193a
P3_3 nor-pre-miRNA>hsa-mir-200b
P3_3 nor-pre-miRNA>hsa-mir-215
P3_3 nor-pre-miRNA>hsa-mir-2277
P3_3 nor-pre-miRNA>hsa-mir-25
P3_3 nor-pre-miRNA>hsa-mir-2681
P3_3 nor-pre-miRNA>hsa-mir-27a
P3_3 nor-pre-miRNA>hsa-mir-296
P3_3 nor-pre-miRNA>hsa-mir-299
P3_3 nor-pre-miRNA>hsa-mir-302a
P3_3 nor-pre-miRNA>hsa-mir-3118-3
P3_3 nor-pre-miRNA>hsa-mir-3118-4
P3_3 nor-pre-miRNA>hsa-mir-3126
P3_3 nor-pre-miRNA>hsa-mir-3144
P3_3 nor-pre-miRNA>hsa-mir-3152
P3_3 nor-pre-miRNA>hsa-mir-3153
P3_3 nor-pre-miRNA>hsa-mir-3158-2
P3_3 nor-pre-miRNA>hsa-mir-3184
P3_3 nor-pre-miRNA>hsa-mir-3192
P3_3 nor-pre-miRNA>hsa-mir-320a
P3_3 nor-pre-miRNA>hsa-mir-320e
P3_3 nor-pre-miRNA>hsa-mir-324
P3_3 nor-pre-miRNA>hsa-mir-335
P3_3 nor-pre-miRNA>hsa-mir-338
P3_3 nor-pre-miRNA>hsa-mir-361
P3_3 nor-pre-miRNA>hsa-mir-3667
P3_3 nor-pre-miRNA>hsa-mir-367
P3_3 nor-pre-miRNA>hsa-mir-3679
P3_3 nor-pre-miRNA>hsa-mir-3682
P3_3 nor-pre-miRNA>hsa-mir-373
P3_3 nor-pre-miRNA>hsa-mir-3912
P3_3 nor-pre-miRNA>hsa-mir-409
P3_3 nor-pre-miRNA>hsa-mir-410
P3_3 nor-pre-miRNA>hsa-mir-429
P3_3 nor-pre-miRNA>hsa-mir-491

P3_3 nor-pre-miRNA>hsa-mir-499a
P3_3 nor-pre-miRNA>hsa-mir-507
P3_3 nor-pre-miRNA>hsa-mir-509-1
P3_3 nor-pre-miRNA>hsa-mir-518a-1
P3_3 nor-pre-miRNA>hsa-mir-532
P3_3 nor-pre-miRNA>hsa-mir-548b
P3_3 nor-pre-miRNA>hsa-mir-548d-1
P3_3 nor-pre-miRNA>hsa-mir-885
P3_3 nor-pre-miRNA>hsa-mir-888
P3_3 nor-pre-miRNA>hsa-mir-892c
P3_3 nor-pre-miRNA>hsa-mir-937
P3_3 nor-pre-miRNA>hsa-mir-944
P3_3 nor-pre-miRNA>hsa-mir-19b-2
P3_3 nor-pre-miRNA>hsa-mir-7-1
P3_3 nor-pre-miRNA>hsa-mir-197
P3_3 nor-pre-miRNA>hsa-mir-379
P3_3 nor-pre-miRNA>hsa-mir-509-2
P3_3 nor-pre-miRNA>hsa-mir-519a-1
P3_3 nor-pre-miRNA>hsa-mir-664b
P3_3 nor-pre-miRNA>hsa-mir-125a
P3_3 nor-pre-miRNA>hsa-mir-1295b
P3_3 nor-pre-miRNA>hsa-mir-196a-2
P3_3 nor-pre-miRNA>hsa-mir-339
P3_3 nor-pre-miRNA>hsa-mir-3622a
P3_3 nor-pre-miRNA>hsa-mir-381
P3_3 nor-pre-miRNA>hsa-mir-16-1
P3_3 nor-pre-miRNA>hsa-mir-1911
P3_3 nor-pre-miRNA>hsa-mir-2682
P3_3 nor-pre-miRNA>hsa-mir-3127
P3_3 nor-pre-miRNA>hsa-mir-3129
P3_3 nor-pre-miRNA>hsa-mir-500b
P3_3 nor-pre-miRNA>hsa-mir-509-3
P3_3 nor-pre-miRNA>hsa-mir-548aq
P3_3 nor-pre-miRNA>hsa-mir-659
P3_3 nor-pre-miRNA>hsa-mir-1245a
P3_3 nor-pre-miRNA>hsa-mir-1277
P3_3 nor-pre-miRNA>hsa-mir-133a-2
P3_3 nor-pre-miRNA>hsa-mir-151b
P3_3 nor-pre-miRNA>hsa-mir-1915
P3_3 nor-pre-miRNA>hsa-mir-301a
P3_3 nor-pre-miRNA>hsa-mir-30b
P3_3 nor-pre-miRNA>hsa-mir-30e
P3_3 nor-pre-miRNA>hsa-mir-3186
P3_3 nor-pre-miRNA>hsa-mir-329-1
P3_3 nor-pre-miRNA>hsa-mir-518d
P3_3 nor-pre-miRNA>hsa-mir-520h
P3_3 nor-pre-miRNA>hsa-mir-933

P3_3 nor-pre-miRNA>hsa-mir-1252
P3_3 nor-pre-miRNA>hsa-mir-135a-1
P3_3 nor-pre-miRNA>hsa-mir-1912
P3_3 nor-pre-miRNA>hsa-mir-202
P3_3 nor-pre-miRNA>hsa-mir-2861
P3_3 nor-pre-miRNA>hsa-mir-3150a
P3_3 nor-pre-miRNA>hsa-mir-3150b
P3_3 nor-pre-miRNA>hsa-mir-362
P3_3 nor-pre-miRNA>hsa-mir-370
P3_3 nor-pre-miRNA>hsa-mir-3910-2
P3_3 nor-pre-miRNA>hsa-mir-3928
P3_3 nor-pre-miRNA>hsa-mir-450a-1
P3_3 nor-pre-miRNA>hsa-mir-513b
P3_3 nor-pre-miRNA>hsa-mir-513c
P3_3 nor-pre-miRNA>hsa-mir-548ap
P3_3 nor-pre-miRNA>hsa-mir-548av
P3_3 nor-pre-miRNA>hsa-mir-887
P3_3 nor-pre-miRNA>hsa-mir-943
P3_3 nor-pre-miRNA>hsa-mir-517b
P3_3 nor-pre-miRNA>hsa-mir-517c
P3_3 nor-pre-miRNA>hsa-mir-1281
P3_3 nor-pre-miRNA>hsa-mir-23a
P3_3 nor-pre-miRNA>hsa-mir-505
P3_3 nor-pre-miRNA>hsa-mir-4254
P3_3 nor-pre-miRNA>hsa-mir-664a
P3_3 nor-pre-miRNA>hsa-mir-512-1
P3_3 nor-pre-miRNA>hsa-mir-512-2
P3_3 nor-pre-miRNA>hsa-mir-2115
P3_3 nor-pre-miRNA>hsa-mir-206
P3_3 nor-pre-miRNA>hsa-mir-3140
P3_3 nor-pre-miRNA>hsa-mir-371a
P3_3 nor-pre-miRNA>hsa-mir-1908
P3_3 nor-pre-miRNA>hsa-mir-501
P3_3 nor-pre-miRNA>hsa-mir-3188
P3_3 nor-pre-miRNA>hsa-mir-195
P3_3 nor-pre-miRNA>hsa-mir-203b
P3_3 nor-pre-miRNA>hsa-mir-3167
P3_3 nor-pre-miRNA>hsa-mir-1285-2
P3_3 nor-pre-miRNA>hsa-mir-3657
P3_3 nor-pre-miRNA>hsa-mir-671
P3_3 nor-pre-miRNA>hsa-mir-1250
P3_3 nor-pre-miRNA>hsa-mir-548aa-1
P3_3 nor-pre-miRNA>hsa-mir-146a
P3_3 nor-pre-miRNA>hsa-mir-219b
P3_3 nor-pre-miRNA>hsa-mir-3064
P3_3 nor-pre-miRNA>hsa-mir-1278
P3_3 nor-pre-miRNA>hsa-mir-383

P3_3 nor-pre-miRNA>hsa-mir-1-1
P3_3 nor-pre-miRNA>hsa-mir-126
P3_3 nor-pre-miRNA>hsa-mir-300
P3_3 nor-pre-miRNA>hsa-mir-3149
P3_3 nor-pre-miRNA>hsa-mir-1468
P3_3 nor-pre-miRNA>hsa-mir-525
P3_3 nor-pre-miRNA>hsa-mir-200a
P3_3 nor-pre-miRNA>hsa-mir-1322
P3_3 nor-pre-miRNA>hsa-mir-423
P3_3 nor-pre-miRNA>hsa-mir-1304
P3_3 nor-pre-miRNA>hsa-mir-2116
P3_3 nor-pre-miRNA>hsa-mir-26b
P3_3 nor-pre-miRNA>hsa-mir-3156-1
P3_3 nor-pre-miRNA>hsa-mir-3156-2
P3_3 nor-pre-miRNA>hsa-mir-3173
P3_3 nor-pre-miRNA>hsa-mir-3617
P3_3 nor-pre-miRNA>hsa-mir-450a-2
P3_3 nor-pre-miRNA>hsa-mir-548a-3
P3_3 nor-pre-miRNA>hsa-mir-548am
P3_3 nor-pre-miRNA>hsa-mir-548f-4
P3_3 nor-pre-miRNA>hsa-mir-1228
P3_3 nor-pre-miRNA>hsa-mir-1244-1
P3_3 nor-pre-miRNA>hsa-mir-939
P3_3 nor-pre-miRNA>hsa-mir-1273h
P3_3 nor-pre-miRNA>hsa-mir-526b
P3_3 nor-pre-miRNA>hsa-mir-3615
P3_3 nor-pre-miRNA>hsa-mir-487b
P3_3 nor-pre-miRNA>hsa-mir-874
P3_3 nor-pre-miRNA>hsa-mir-548ae-1
P3_3 nor-pre-miRNA>hsa-mir-3909
P3_3 nor-pre-miRNA>hsa-mir-192
P3_3 nor-pre-miRNA>hsa-mir-940
P3_3 nor-pre-miRNA>hsa-mir-138-1
P3_3 nor-pre-miRNA>hsa-mir-3686
P3_3 nor-pre-miRNA>hsa-mir-769
P3_3 nor-pre-miRNA>hsa-mir-153-2
P3_3 nor-pre-miRNA>hsa-mir-466
P3_3 nor-pre-miRNA>hsa-mir-3166
P3_3 nor-pre-miRNA>hsa-mir-125b-2
P3_3 nor-pre-miRNA>hsa-mir-1538
P3_3 nor-pre-miRNA>hsa-mir-3176
P3_3 nor-pre-miRNA>hsa-mir-342
P3_3 nor-pre-miRNA>hsa-mir-181b-1
P3_3 nor-pre-miRNA>hsa-mir-520b
P3_3 nor-pre-miRNA>hsa-mir-1249
P3_3 nor-pre-miRNA>hsa-mir-518a-2
P3_3 nor-pre-miRNA>hsa-mir-519d

P3_3 nor-pre-miRNA>hsa-mir-544b
P3_3 nor-pre-miRNA>hsa-mir-378a
P3_3 nor-pre-miRNA>hsa-mir-3116-1
P3_3 nor-pre-miRNA>hsa-mir-486-2
P3_3 nor-pre-miRNA>hsa-mir-224
P3_3 nor-pre-miRNA>hsa-mir-455
P3_3 nor-pre-miRNA>hsa-mir-330
P3_3 nor-pre-miRNA>hsa-mir-3660
P3_3 nor-pre-miRNA>hsa-mir-548a-1
P3_3 nor-pre-miRNA>hsa-mir-548c
P3_3 nor-pre-miRNA>hsa-mir-143
P3_3 nor-pre-miRNA>hsa-mir-345
P3_3 nor-pre-miRNA>hsa-mir-548ao
P3_3 nor-pre-miRNA>hsa-mir-185
P3_3 nor-pre-miRNA>hsa-mir-523
P3_3 nor-pre-miRNA>hsa-mir-3659
P3_3 nor-pre-miRNA>hsa-mir-548g
P3_3 nor-pre-miRNA>hsa-mir-184
P3_3 nor-pre-miRNA>hsa-mir-1910
P3_3 nor-pre-miRNA>hsa-mir-17
P3_3 nor-pre-miRNA>hsa-mir-371b
P3_3 nor-pre-miRNA>hsa-mir-24-1
P3_3 nor-pre-miRNA>hsa-mir-1255b-2
P3_3 nor-pre-miRNA>hsa-mir-449c
P3_3 nor-pre-miRNA>hsa-mir-3671
P3_3 nor-pre-miRNA>hsa-mir-93
P3_3 nor-pre-miRNA>hsa-mir-518b
P3_3 nor-pre-miRNA>hsa-mir-1269a
P3_3 nor-pre-miRNA>hsa-mir-2113
P3_3 nor-pre-miRNA>hsa-mir-449b
P3_3 nor-pre-miRNA>hsa-mir-1307
P3_3 nor-pre-miRNA>hsa-mir-662
P3_3 nor-pre-miRNA>hsa-mir-24-2
P3_3 nor-pre-miRNA>hsa-mir-380
P3_3 nor-pre-miRNA>hsa-mir-187
P3_3 nor-pre-miRNA>hsa-mir-210
P3_3 nor-pre-miRNA>hsa-mir-4322
P3_3 nor-pre-miRNA>hsa-mir-520c
P3_3 nor-pre-miRNA>hsa-mir-522
P3_3 nor-pre-miRNA>hsa-mir-1289-2
P3_3 nor-pre-miRNA>hsa-mir-519a-2
P3_3 nor-pre-miRNA>hsa-mir-3124
P3_3 nor-pre-miRNA>hsa-mir-219a-1
P3_3 nor-pre-miRNA>hsa-mir-521-1
P3_3 nor-pre-miRNA>hsa-mir-519b
P3_3 nor-pre-miRNA>hsa-mir-548at
P3_3 nor-pre-miRNA>hsa-mir-450b

P3_3	nor-pre-miRNA>hsa-mir-548ar	
P3_3	nor-pre-miRNA>hsa-mir-548as	
P3_3	nor-pre-miRNA>hsa-mir-199b	
P3_3	nor-pre-miRNA>hsa-mir-199a-2	
P3_3	nor-pre-miRNA>hsa-mir-3142	
P3_3	nor-pre-miRNA>hsa-mir-3158-1	
P3_3	nor-pre-miRNA>hsa-mir-485	
P3_3	nor-pre-miRNA>hsa-mir-514a-1	
P3_3	nor-pre-miRNA>hsa-mir-520g	
P3_3	nor-pre-miRNA>hsa-mir-3938	
P3_3	nor-pre-miRNA>hsa-mir-34a	
P3_3	nor-pre-miRNA>hsa-mir-548ac	
P3_3	nor-pre-miRNA>hsa-mir-1913	
P3_3	nor-pre-miRNA>hsa-mir-3074	
P3_3	nor-pre-miRNA>hsa-mir-105-1	
P3_3	nor-pre-miRNA>hsa-mir-105-2	
P3_3	nor-pre-miRNA>hsa-mir-3664	
P3_3	nor-pre-miRNA>hsa-mir-196b	
P3_3	nor-pre-miRNA>hsa-mir-3618	
P3_3	nor-SNP	4850310
P3_3	nor-SNP	4850331
P3_3	nor-SNP	46657254
P3_3	nor-SNP	177015045
P3_3	nor-SNP	120151527
P3_3	nor-SNP	120151501
P3_3	nor-SNP	120151493
P3_3	nor-SNP	101491923
P3_3	nor-SNP	101491974
P3_3	nor-SNP	101491981
P3_3	nor-SNP	129061408
P3_3	nor-SNP	34674309
P3_3	nor-SNP	34820530
P3_3	nor-SNP	61809907
P3_3	nor-SNP	189842885
P3_3	nor-SNP	20236701
P3_3	nor-SNP	2633462
P3_3	nor-SNP	171070937
P3_3	nor-SNP	154065383
P3_3	nor-SNP	154065347
P3_3	nor-SNP	154065348
P3_3	nor-SNP	154065368
P3_3	nor-SNP	22007594
P3_3	nor-SNP	22007634
P3_3	nor-SNP	19405743
P3_3	nor-SNP	19405676
P3_3	nor-SNP	19405672
P3_3	nor-SNP	101351088

P3_3	nor-SNP	101351048
P3_3	nor-SNP	69966985
P3_3	nor-SNP	69966994
P3_3	nor-SNP	69967005
P3_3	nor-SNP	69967062
P3_3	nor-SNP	7073301
P3_3	nor-SNP	7073282
P3_3	nor-SNP	148810267
P3_3	nor-SNP	101526116
P3_3	nor-SNP	160122434
P3_3	nor-SNP	160122421
P3_3	nor-SNP	129414804
P3_3	nor-SNP	129414852
P3_3	nor-SNP	129414815
P3_3	nor-SNP	63116226
P3_3	nor-SNP	62572885
P3_3	nor-SNP	62572874
P3_3	nor-SNP	29887033
P3_3	nor-SNP	1102498
P3_3	nor-SNP	1102501
P3_3	nor-SNP	1102563
P3_3	nor-SNP	220291302
P3_3	nor-SNP	220291292
P3_3	nor-SNP	220291206
P3_3	nor-SNP	92956420
P3_3	nor-SNP	92956416
P3_3	nor-SNP	99691200
P3_3	nor-SNP	102620081
P3_3	nor-SNP	102619995
P3_3	nor-SNP	13947296
P3_3	nor-SNP	13947292
P3_3	nor-SNP	57392715
P3_3	nor-SNP	57392697
P3_3	nor-SNP	57392686
P3_3	nor-SNP	57392678
P3_3	nor-SNP	101490145
P3_3	nor-SNP	101490178
P3_3	nor-SNP	113569406
P3_3	nor-SNP	113569394
P3_3	nor-SNP	143424215
P3_3	nor-SNP	22049282
P3_3	nor-SNP	143424198
P3_3	nor-SNP	143424165
P3_3	nor-SNP	21038172
P3_3	nor-SNP	69330824
P3_3	nor-SNP	69330825
P3_3	nor-SNP	69330818

P3_3	nor-SNP	120336327
P3_3	nor-SNP	120336365
P3_3	nor-SNP	120336384
P3_3	nor-SNP	18573331
P3_3	nor-SNP	18573360
P3_3	nor-SNP	18573361
P3_3	nor-SNP	18573374
P3_3	nor-SNP	91927158
P3_3	nor-SNP	91927175
P3_3	nor-SNP	103361221
P3_3	nor-SNP	103361226
P3_3	nor-SNP	28444157
P3_3	nor-SNP	18451325
P3_3	nor-SNP	18451300
P3_3	nor-SNP	22102545
P3_3	nor-SNP	47212593
P3_3	nor-SNP	47212591
P3_3	nor-SNP	7126698
P3_3	nor-SNP	7126619
P3_3	nor-SNP	130135961
P3_3	nor-SNP	79099736
P3_3	nor-SNP	85158670
P3_3	nor-SNP	49937087
P3_3	nor-SNP	49937076
P3_3	nor-SNP	113569088
P3_3	nor-SNP	113569054
P3_3	nor-SNP	134884697
P3_3	nor-SNP	54076332
P3_3	nor-SNP	54291965
P3_3	nor-SNP	54292016
P3_3	nor-SNP	170813684
P3_3	nor-SNP	101531654
P3_3	nor-SNP	101532279
P3_3	nor-SNP	1104412
P3_3	nor-SNP	1104462
P3_3	nor-SNP	20716104
P3_3	nor-SNP	20716128
P3_3	nor-SNP	33578201
P3_3	nor-SNP	33578251
P3_3	nor-SNP	33578255
P3_3	nor-SNP	33578276
P3_3	nor-SNP	33578202
P3_3	nor-SNP	146312590
P3_3	nor-SNP	146342080
P3_3	nor-SNP	146342071
P3_3	nor-SNP	146342052
P3_3	nor-SNP	54234260

P3_3	nor-SNP	49767769
P3_3	nor-SNP	49767815
P3_3	nor-SNP	49767832
P3_3	nor-SNP	49767835
P3_3	nor-SNP	49767838
P3_3	nor-SNP	119390294
P3_3	nor-SNP	119390286
P3_3	nor-SNP	124360347
P3_3	nor-SNP	10436238
P3_3	nor-SNP	10436201
P3_3	nor-SNP	10436194
P3_3	nor-SNP	10436180
P3_3	nor-SNP	10436219
P3_3	nor-SNP	145076376
P3_3	nor-SNP	145076356
P3_3	nor-SNP	145076355
P3_3	nor-SNP	145074289
P3_3	nor-SNP	145074342
P3_3	nor-SNP	144895170
P3_3	nor-SNP	189547748
P3_3	nor-SNP	189547778
P3_3	nor-SNP	133303758
P3_3	nor-SNP	133303744
P3_3	nor-SNP	86584720
P3_3	nor-SNP	86584675
P3_3	nor-SNP	110141578
P3_3	nor-SNP	101488414
P3_3	nor-SNP	101488424
P3_3	nor-SNP	101488430
P3_3	nor-SNP	101488431
P3_3	nor-SNP	146340360
P3_3	nor-SNP	146340339
P3_3	nor-SNP	146340304
P3_3	nor-SNP	146340278
P3_3	nor-SNP	54255679
P3_3	nor-SNP	54255689
P3_3	nor-SNP	153996899
P3_3	nor-SNP	144895168
P3_3	nor-SNP	144895169
P3_3	nor-SNP	144895179
P3_3	nor-SNP	52196574
P3_3	nor-SNP	171070888
P3_3	nor-SNP	101526127
P3_3	nor-SNP	101526140
P3_3	nor-SNP	129414807
P3_3	nor-SNP	54385584
P3_3	nor-SNP	54385558

P3_3	nor-SNP	54385561
P3_3	nor-SNP	1062662
P3_3	nor-SNP	1062653
P3_3	nor-SNP	1062626
P3_3	nor-SNP	27559214
P3_3	nor-SNP	27559261
P3_3	nor-SNP	101512288
P3_3	nor-SNP	101512304
P3_3	nor-SNP	50623143
P3_3	nor-SNP	50623110
P3_3	nor-SNP	113997768
P3_3	nor-SNP	113997812
P3_3	nor-SNP	113997817
P3_3	nor-SNP	98510902
P3_3	nor-SNP	98510896
P3_3	nor-SNP	98510864
P3_3	nor-SNP	98510847
P3_3	nor-SNP	97464049
P3_3	nor-SNP	97464057
P3_3	nor-SNP	189997834
P3_3	nor-SNP	189997816
P3_3	nor-SNP	49775296
P3_3	nor-SNP	49775337
P3_3	nor-SNP	49775351
P3_3	nor-SNP	146341217
P3_3	nor-SNP	185485646
P3_3	nor-SNP	38243743
P3_3	nor-SNP	189842822
P3_3	nor-SNP	117520360
P3_3	nor-SNP	117520389
P3_3	nor-SNP	61162131
P3_3	nor-SNP	61162191
P3_3	nor-SNP	100575791
P3_3	nor-SNP	100575818
P3_3	nor-SNP	129414843
P3_3	nor-SNP	21785508
P3_3	nor-SNP	57228574
P3_3	nor-SNP	57228573
P3_3	nor-SNP	135812836
P3_3	nor-SNP	41220077
P3_3	nor-SNP	79418149
P3_3	nor-SNP	22102543
P3_3	nor-SNP	22102542
P3_3	nor-SNP	22102546
P3_3	nor-SNP	101493129
P3_3	nor-SNP	54238182
P3_3	nor-SNP	54238159

P3_3	nor-SNP	54245768
P3_3	nor-SNP	176032424
P3_3	nor-SNP	176032428
P3_3	nor-SNP	79813049
P3_3	nor-SNP	79813075
P3_3	nor-SNP	79813083
P3_3	nor-SNP	2633466
P3_3	nor-SNP	2633478
P3_3	nor-SNP	2633480
P3_3	nor-SNP	52328298
P3_3	nor-SNP	52328248
P3_3	nor-SNP	113886031
P3_3	nor-SNP	135061124
P3_3	nor-SNP	135061112
P3_3	nor-SNP	135061111
P3_3	nor-SNP	135061109
P3_3	nor-SNP	135061106
P3_3	nor-SNP	135061039
P3_3	nor-SNP	130548199
P3_3	nor-SNP	130548214
P3_3	nor-SNP	96085190
P3_3	nor-SNP	49773603
P3_3	nor-SNP	101377478
P3_3	nor-SNP	94398600
P3_3	nor-SNP	94398581
P3_3	nor-SNP	133674439
P3_3	nor-SNP	133674418
P3_3	nor-SNP	133674399
P3_3	nor-SNP	146280644
P3_3	nor-SNP	146280626
P3_3	nor-SNP	146271304
P3_3	nor-SNP	146271303
P3_3	nor-SNP	146271269
P3_3	nor-SNP	146271266
P3_3	nor-SNP	146271235
P3_3	nor-SNP	146271231
P3_3	nor-SNP	86368890
P3_3	nor-SNP	86368898
P3_3	nor-SNP	86368922
P3_3	nor-SNP	86368929
P3_3	nor-SNP	86368959
P3_3	nor-SNP	15935347
P3_3	nor-SNP	15935348
P3_3	nor-SNP	15935351
P3_3	nor-SNP	15935316
P3_3	nor-SNP	176032408
P3_3	nor-SNP	176032384

P3_3	nor-SNP	176032415
P3_3	nor-SNP	1988119
P3_3	nor-SNP	1988176
P3_3	nor-SNP	1988170
P3_3	nor-SNP	1988144
P3_3	nor-SNP	46657289
P3_3	nor-SNP	54224382
P3_3	nor-SNP	54244647
P3_3	nor-SNP	103361245
P3_3	nor-SNP	22102519
P3_3	nor-SNP	41488550
P3_3	nor-SNP	13947436
P3_3	nor-SNP	139006339
P3_3	nor-SNP	32224285
P3_3	nor-SNP	220373943
P3_3	nor-SNP	220373939
P3_3	nor-SNP	144895164
P3_3	nor-SNP	144895159
P3_3	nor-SNP	54169954
P3_3	nor-SNP	54169973
P3_3	nor-SNP	54170006
P3_3	nor-SNP	54170016
P3_3	nor-SNP	54172483
P3_3	nor-SNP	54172501
P3_3	nor-SNP	86584707
P3_3	nor-SNP	48357864
P3_3	nor-SNP	48357853
P3_3	nor-SNP	52009178
P3_3	nor-SNP	52009220
P3_3	nor-SNP	153410531
P3_3	nor-SNP	153410482
P3_3	nor-SNP	153410520
P3_3	nor-SNP	52196528
P3_3	nor-SNP	129414806
P3_3	nor-SNP	54290994
P3_3	nor-SNP	54290995
P3_3	nor-SNP	61582680
P3_3	nor-SNP	61582708
P3_3	nor-SNP	38243770
P3_3	nor-SNP	49774383
P3_3	nor-SNP	49774389
P3_3	nor-SNP	49774381
P3_3	nor-SNP	18392936
P3_3	nor-SNP	18392894
P3_3	nor-SNP	18392913
P3_3	nor-SNP	6920975
P3_3	nor-SNP	104583804

P3_3	nor-SNP	104583796
P3_3	nor-SNP	104583838
P3_3	nor-SNP	126858425
P3_3	nor-SNP	126858406
P3_3	nor-SNP	126858392
P3_3	nor-SNP	101491941
P3_3	nor-SNP	70480123
P3_3	nor-SNP	70480127
P3_3	nor-SNP	54385599
P3_3	nor-SNP	54385629
P3_3	nor-SNP	112475403
P3_3	nor-SNP	112475427
P3_3	nor-SNP	112475429
P3_3	nor-SNP	150935522
P3_3	nor-SNP	150935577
P3_3	nor-SNP	150935583
P3_3	nor-SNP	79107084
P3_3	nor-SNP	54245780
P3_3	nor-SNP	159912436
P3_3	nor-SNP	131154943
P3_3	nor-SNP	62496926
P3_3	nor-SNP	62496924
P3_3	nor-SNP	193105672
P3_3	nor-SNP	14710999
P3_3	nor-SNP	61151515
P3_3	nor-SNP	139565098
P3_3	nor-SNP	139565119
P3_3	nor-SNP	139565134
P3_3	nor-SNP	101507720
P3_3	nor-SNP	101507727
P3_3	nor-SNP	77879011
P3_3	nor-SNP	77879071
P3_3	nor-SNP	77879051
P3_3	nor-SNP	63005918
P3_3	nor-SNP	113997746
P3_3	nor-SNP	79099712
P3_3	nor-SNP	146312589
P3_3	nor-SNP	146312560
P3_3	nor-SNP	146312541
P3_3	nor-SNP	146312517
P3_3	nor-SNP	54200810
P3_3	nor-SNP	54200826
P3_3	nor-SNP	54200830
P3_3	nor-SNP	54200843
P3_3	nor-SNP	54200853
P3_3	nor-SNP	54200834
P3_3	nor-SNP	189842826

P3_3	nor-SNP	2633434
P3_3	nor-SNP	1103284
P3_3	nor-SNP	10682934
P3_3	nor-SNP	159912418
P3_3	nor-SNP	28444104
P3_3	nor-SNP	28444183
P3_3	nor-SNP	93466919
P3_3	nor-SNP	93466912
P3_3	nor-SNP	93466910
P3_3	nor-SNP	93466909
P3_3	nor-SNP	93466866
P3_3	nor-SNP	62572847
P3_3	nor-SNP	59463451
P3_3	nor-SNP	59463436
P3_3	nor-SNP	59463389
P3_3	nor-SNP	219267402
P3_3	nor-SNP	219267407
P3_3	nor-SNP	219267370
P3_3	nor-SNP	45659500
P3_3	nor-SNP	14830172
P3_3	nor-SNP	14830197
P3_3	nor-SNP	14830201
P3_3	nor-SNP	14830215
P3_3	nor-SNP	14830216
P3_3	nor-SNP	95604259
P3_3	nor-SNP	44333749
P3_3	nor-SNP	133674612
P3_3	nor-SNP	133674576
P3_3	nor-SNP	105496675
P3_3	nor-SNP	16645208
P3_3	nor-SNP	16645137
P3_3	nor-SNP	16645199
P3_3	nor-SNP	16645178
P3_3	nor-SNP	147075166
P3_3	nor-SNP	147075204
P3_3	nor-SNP	92956422
P3_3	nor-SNP	92956412
P3_3	nor-SNP	92956409
P3_3	nor-SNP	134884700
P3_3	nor-SNP	232578032
P3_3	nor-SNP	145619405
P3_3	nor-SNP	24214442
P3_3	nor-SNP	24214486
P3_3	nor-SNP	24214493
P3_3	nor-SNP	24214532
P3_3	nor-SNP	54197674
P3_3	nor-SNP	54197678

P3_3	nor-SNP	6920976
P3_3	nor-SNP	6920955
P3_3	nor-SNP	72744798
P3_3	nor-SNP	101512836
P3_3	nor-SNP	139006378
P3_3	nor-SNP	136983326
P3_3	nor-SNP	136983319
P3_3	nor-SNP	136983281
P3_3	nor-SNP	185243724
P3_3	nor-SNP	35731712
P3_3	nor-SNP	35731697
P3_3	nor-SNP	64658715
P3_3	nor-SNP	64658710
P3_3	nor-SNP	64658640
P3_3	nor-SNP	64658716
P3_3	nor-SNP	2321762
P3_3	nor-SNP	57588323
P3_3	nor-SNP	61151539
P3_3	nor-SNP	44155749
P3_3	nor-SNP	130496365
P3_3	nor-SNP	104583828
P3_3	nor-SNP	104583776
P3_3	nor-SNP	104583759
P3_3	nor-SNP	61151573
P3_3	nor-SNP	54197706
P3_3	nor-SNP	46522201
P3_3	nor-SNP	46522255
P3_3	nor-SNP	14711013
P3_3	nor-SNP	54197660
P3_3	nor-SNP	157367104
P3_3	nor-SNP	31203277
P3_3	nor-SNP	87909673
P3_3	nor-SNP	17962615
P3_3	nor-SNP	69599751
P3_3	nor-SNP	69599735
P3_3	nor-SNP	593277
P3_3	nor-SNP	100576035
P3_3	nor-SNP	198828005
P3_3	nor-SNP	198828096
P3_3	nor-SNP	54204489
P3_3	nor-SNP	54204496
P3_3	nor-SNP	54204497
P3_3	nor-SNP	45596885
P3_3	nor-SNP	54242630
P3_3	nor-SNP	54216629
P3_3	nor-SNP	54216670
P3_3	nor-SNP	54216681

P3_3	nor-SNP	54216650
P3_3	nor-SNP	124451312
P3_3	nor-SNP	124451357
P3_3	nor-SNP	149112399
P3_3	nor-SNP	10682950
P3_3	nor-SNP	54216616
P3_3	nor-SNP	54245841
P3_3	nor-SNP	54245789
P3_3	nor-SNP	54245788
P3_3	nor-SNP	154065370
P3_3	nor-SNP	62544470
P3_3	nor-SNP	41517981
P3_3	nor-SNP	41517974
P3_3	nor-SNP	151127105
P3_3	nor-SNP	151127092
P3_3	nor-SNP	116971750
P3_3	nor-SNP	116971778
P3_3	nor-SNP	2321809
P3_3	nor-SNP	101507711
P3_3	nor-SNP	593323
P3_3	nor-SNP	46142293
P3_3	nor-SNP	89312487
P3_3	nor-SNP	89312451
P3_3	nor-SNP	18572056
P3_3	nor-SNP	105496638
P3_3	nor-SNP	105496622
P3_3	nor-SNP	119390263
P3_3	nor-SNP	65016300
P3_3	nor-SNP	65016325
P3_3	nor-SNP	1988116
P3_3	nor-SNP	2321792
P3_3	nor-SNP	62544490
P3_3	nor-SNP	148808487
P3_3	nor-SNP	148808575
P3_3	nor-SNP	59463460
P3_3	nor-SNP	2321774
P3_3	nor-SNP	100774203
P3_3	nor-SNP	100774268
P3_3	nor-SNP	100774277
P3_3	nor-SNP	100774289
P3_3	nor-SNP	41128599
P3_3	nor-SNP	41128578
P3_3	nor-SNP	139565064
P3_3	nor-SNP	20020733
P3_3	nor-SNP	54201654
P3_3	nor-SNP	46522190
P3_3	nor-SNP	54245827

P3_3	nor-SNP	72744805
P3_3	nor-SNP	38554942
P3_3	nor-SNP	148265864
P3_3	nor-SNP	79502168
P3_3	nor-SNP	85775275
P3_3	nor-SNP	69330823
P3_3	nor-SNP	189547761
P3_3	nor-SNP	92002896
P3_3	nor-SNP	97848319
P3_3	nor-SNP	97848343
P3_3	nor-SNP	167967904
P3_3	nor-SNP	167967953
P3_3	nor-SNP	167967958
P3_3	nor-SNP	167967963
P3_3	nor-SNP	54468150
P3_3	nor-SNP	54468144
P3_3	nor-SNP	54468110
P3_3	nor-SNP	54468094
P3_3	nor-SNP	65523500
P3_3	nor-SNP	99691429
P3_3	nor-SNP	99691401
P3_3	nor-SNP	99691396
P3_3	nor-SNP	99691393
P3_3	nor-SNP	54206051
P3_3	nor-SNP	67142564
P3_3	nor-SNP	67142583
P3_3	nor-SNP	67142619
P3_3	nor-SNP	67142620
P3_3	nor-SNP	98472446
P3_3	nor-SNP	98472426
P3_3	nor-SNP	46522298
P3_3	nor-SNP	54466527
P3_3	nor-SNP	54466544
P3_3	nor-SNP	105154084
P3_3	nor-SNP	105154089
P3_3	nor-SNP	820192
P3_3	nor-SNP	820197
P3_3	nor-SNP	820215
P3_3	nor-SNP	70480051
P3_3	nor-SNP	70480108
P3_3	nor-SNP	13947170
P3_3	nor-SNP	105154097
P3_3	nor-SNP	101491407
P3_3	nor-SNP	33484837
P3_3	nor-SNP	31203228
P3_3	nor-SNP	31203221
P3_3	nor-SNP	31203216

P3_3	nor-SNP	31203207
P3_3	nor-SNP	105154013
P3_3	nor-SNP	105154091
P3_3	nor-SNP	568187
P3_3	nor-SNP	568176
P3_3	nor-SNP	568125
P3_3	nor-SNP	568106
P3_3	nor-SNP	101491378
P3_3	nor-SNP	98472445
P3_3	nor-SNP	10341137
P3_3	nor-SNP	54210734
P3_3	nor-SNP	54210736
P3_3	nor-SNP	54210774
P3_3	nor-SNP	54254494
P3_3	nor-SNP	54254543
P3_3	nor-SNP	132763305
P3_3	nor-SNP	132763362
P3_3	nor-SNP	132763335
P3_3	nor-SNP	54265617
P3_3	nor-SNP	18572070
P3_3	nor-SNP	249120578
P3_3	nor-SNP	54265626
P3_3	nor-SNP	54265658
P3_3	nor-SNP	54265670
P3_3	nor-SNP	33175642
P3_3	nor-SNP	33175702
P3_3	nor-SNP	54251902
P3_3	nor-SNP	33484783
P3_3	nor-SNP	593365
P3_3	nor-SNP	593362
P3_3	nor-SNP	54198496
P3_3	nor-SNP	54198499
P3_3	nor-SNP	40646803
P3_3	nor-SNP	133674285
P3_3	nor-SNP	115010026
P3_3	nor-SNP	93142427
P3_3	nor-SNP	153996903
P3_3	nor-SNP	153996905
P3_3	nor-SNP	131007087
P3_3	nor-SNP	131007052
P3_3	nor-SNP	131007004
P3_3	nor-SNP	131007109
P3_3	nor-SNP	172113756
P3_3	nor-SNP	159901428
P3_3	nor-SNP	159901465
P3_3	nor-SNP	54201692
P3_3	nor-SNP	54201667

P3_3	nor-SNP	54201668
P3_3	nor-SNP	101521764
P3_3	nor-SNP	159901426
P3_3	nor-SNP	146360765
P3_3	nor-SNP	146360845
P3_3	nor-SNP	146360779
P3_3	nor-SNP	146360813
P3_3	nor-SNP	54216615
P3_3	nor-SNP	54225437
P3_3	nor-SNP	54225426
P3_3	nor-SNP	145619442
P3_3	nor-SNP	145619386
P3_3	nor-SNP	55886581
P3_3	nor-SNP	55886608
P3_3	nor-SNP	136983275
P3_3	nor-SNP	9211782
P3_3	nor-SNP	9211802
P3_3	nor-SNP	117102674
P3_3	nor-SNP	117102660
P3_3	nor-SNP	117102649
P3_3	nor-SNP	113886051
P3_3	nor-SNP	171070875
P3_3	nor-SNP	166922916
P3_3	nor-SNP	151560699
P3_3	nor-SNP	151562938
P3_3	nor-SNP	70718466
P3_3	nor-SNP	27209165
P3_3	nor-SNP	20073323
P3_3	nor-SNP	69330871
P3_3	nor-SNP	2321820
P3_3	nor-SNP	129061422
P3_3	nor-SNP	129061441
P3_3	nor-SNP	7073344
P3_3	nor-SNP	69330886
P3_3	nor-SNP	54234265
P3_3	nor-SNP	54234315
P3_3	nor-SNP	54234340
P3_3	nor-SNP	10436187
P3_3	nor-SNP	145076302
P3_3	nor-SNP	145074283
P3_3	nor-SNP	145074272
P3_3	nor-SNP	145074284
P3_3	nor-SNP	189547779
P3_3	nor-SNP	1062656
P3_3	nor-SNP	1062599
P3_3	nor-SNP	1062574
P3_3	nor-SNP	38243727

P3_3	nor-SNP	100575762
P3_3	nor-SNP	41220094
P3_3	nor-SNP	54238189
P3_3	nor-SNP	54238203
P3_3	nor-SNP	54238208
P3_3	nor-SNP	176032376
P3_3	nor-SNP	189547735
P3_3	nor-SNP	22102536
P3_3	nor-SNP	41488567
P3_3	nor-SNP	41488558
P3_3	nor-SNP	220373934
P3_3	nor-SNP	61582649
P3_3	nor-SNP	101491965
P3_3	nor-SNP	70480088
P3_3	nor-SNP	79107068
P3_3	nor-SNP	79107061
P3_3	nor-SNP	79107017
P3_3	nor-SNP	101507779
P3_3	nor-SNP	79107049
P3_3	nor-SNP	1103312
P3_3	nor-SNP	1103328
P3_3	nor-SNP	1103331
P3_3	nor-SNP	10682907
P3_3	nor-SNP	219267443
P3_3	nor-SNP	147075121
P3_3	nor-SNP	147075120
P3_3	nor-SNP	232578091
P3_3	nor-SNP	145619377
P3_3	nor-SNP	64658623
P3_3	nor-SNP	44155754
P3_3	nor-SNP	87909701
P3_3	nor-SNP	87909716
P3_3	nor-SNP	87909749
P3_3	nor-SNP	41518025
P3_3	nor-SNP	2321827
P3_3	nor-SNP	38243739
P3_3	nor-SNP	62544495
P3_3	nor-SNP	64658705
P3_3	nor-SNP	65523495
P3_3	nor-SNP	820249
P3_3	nor-SNP	820265
P3_3	nor-SNP	134884717
P3_3	nor-SNP	85775260
P3_3	nor-SNP	132763298
P3_3	nor-SNP	18572073
P3_3	nor-SNP	249120591
P3_3	nor-SNP	249120610

P3_3	nor-SNP	10682894
P3_3	nor-SNP	54251937
P3_3	nor-SNP	40646816
P3_3	nor-SNP	219267371
P3_3	nor-SNP	129061462
P3_3	nor-SNP	146360778
P3_3	nor-SNP	54201695
P3_3	nor-SNP	54201703
P3_3	nor-SNP	54225490
P3_3	nor-SNP	54225460
P3_3	nor-SNP	54225463
P3_3	nor-SNP	145619365
P3_3	nor-SNP	55886574
P3_3	nor-SNP	55886522
P3_3	nor-SNP	9211778
P3_3	nor-SNP	9211747
P3_3	nor-SNP	9211738
P3_3	nor-SNP	147075152
P3_3	nor-SNP	166922862
P3_3	nor-SNP	1102567
P3_3	nor-SNP	8007104
P3_3	nor-SNP	100575761
P3_3	nor-SNP	41488568
P3_3	nor-SNP	220373933
P3_3	nor-SNP	232578099
P3_3	nor-SNP	70480062
P3_3	nor-SNP	134884723
P3_3	nor-SNP	132763295
P3_3	nor-SNP	249120640
P3_3	nor-SNP	40646834
P3_3	nor-SNP	219267433
P3_3	nor-SNP	54225501
P3_3	nor-SNP	220373922
P3_3	nor-SNP	131007001
P3_3	iso-pre-miRNA>hsa-mir-663b	
P3_3	iso-pre-miRNA>hsa-mir-1273a	
P3_3	iso-pre-miRNA>hsa-mir-1237	
P3_3	iso-pre-miRNA>hsa-let-7b	
P3_3	iso-pre-miRNA>hsa-mir-4259	
P3_3	iso-pre-miRNA>hsa-mir-1225	
P3_3	iso-pre-miRNA>hsa-mir-1244-1	
P3_3	iso-pre-miRNA>hsa-mir-1244-2	
P3_3	iso-pre-miRNA>hsa-mir-1244-3	
P3_3	iso-pre-miRNA>hsa-mir-1324	
P3_3	iso-pre-miRNA>hsa-mir-1236	
P3_3	iso-pre-miRNA>hsa-mir-1226	
P3_3	iso-pre-miRNA>hsa-let-7i	

P3_3 iso-pre-miRNA>hsa-mir-1238
P3_3 iso-pre-miRNA>hsa-mir-1289-2
P3_3 iso-pre-miRNA>hsa-mir-378e
P3_3 iso-pre-miRNA>hsa-mir-1227
P3_3 iso-pre-miRNA>hsa-mir-4265
P3_3 iso-pre-miRNA>hsa-mir-1233-1
P3_3 iso-pre-miRNA>hsa-mir-1233-2
P3_3 iso-pre-miRNA>hsa-mir-1228
P3_3 iso-pre-miRNA>hsa-mir-2392
P3_3 iso-pre-miRNA>hsa-mir-1299
P3_3 iso-pre-miRNA>hsa-mir-3157
P3_3 iso-pre-miRNA>hsa-mir-548al
P3_3 iso-pre-miRNA>hsa-mir-922
P3_3 iso-pre-miRNA>hsa-mir-1234
P3_3 iso-pre-miRNA>hsa-let-7e
P3_3 iso-pre-miRNA>hsa-mir-541
P3_3 iso-pre-miRNA>hsa-mir-548h-4
P3_3 iso-pre-miRNA>hsa-mir-3679
P3_3 iso-pre-miRNA>hsa-mir-138-2
P3_3 iso-pre-miRNA>hsa-mir-200a
P3_3 iso-pre-miRNA>hsa-mir-3156-1
P3_3 iso-pre-miRNA>hsa-mir-1224
P3_3 iso-pre-miRNA>hsa-mir-1229
P3_3 iso-pre-miRNA>hsa-mir-218-2
P3_3 iso-pre-miRNA>hsa-mir-221
P3_3 iso-pre-miRNA>hsa-mir-3154
P3_3 iso-pre-miRNA>hsa-mir-486-2
P3_3 iso-pre-miRNA>hsa-mir-500a
P3_3 iso-pre-miRNA>hsa-mir-4274
P3_3 iso-pre-miRNA>hsa-mir-454
P3_3 iso-pre-miRNA>hsa-mir-3678
P3_3 iso-pre-miRNA>hsa-mir-27b
P3_3 iso-pre-miRNA>hsa-mir-449c
P3_3 iso-pre-miRNA>hsa-mir-129-1
P3_3 iso-pre-miRNA>hsa-mir-1289-1
P3_3 iso-pre-miRNA>hsa-mir-412
P3_3 iso-pre-miRNA>hsa-mir-3140
P3_3 iso-pre-miRNA>hsa-mir-4318
P3_3 iso-pre-miRNA>hsa-mir-2276
P3_3 iso-pre-miRNA>hsa-mir-106b
P3_3 iso-pre-miRNA>hsa-mir-326
P3_3 iso-pre-miRNA>hsa-mir-3177
P3_3 iso-pre-miRNA>hsa-let-7a-2
P3_3 iso-pre-miRNA>hsa-mir-3922
P3_3 iso-pre-miRNA>hsa-mir-3609
P3_3 iso-pre-miRNA>hsa-mir-18a
P3_3 iso-pre-miRNA>hsa-mir-662

P3_3 iso-pre-miRNA>hsa-mir-219a-2
P3_3 iso-pre-miRNA>hsa-mir-876
P3_3 iso-pre-miRNA>hsa-mir-30e
P3_3 iso-pre-miRNA>hsa-mir-148b
P3_3 iso-pre-miRNA>hsa-mir-523
P3_3 iso-pre-miRNA>hsa-mir-516b-1
P3_3 iso-pre-miRNA>hsa-mir-516a-2
P3_3 iso-pre-miRNA>hsa-mir-30d
P3_3 iso-pre-miRNA>hsa-mir-520a
P3_3 iso-pre-miRNA>hsa-mir-26a-1
P3_3 iso-pre-miRNA>hsa-mir-142
P3_3 iso-pre-miRNA>hsa-mir-519b
P3_3 iso-pre-miRNA>hsa-mir-518c
P3_3 iso-pre-miRNA>hsa-mir-300
P3_3 iso-pre-miRNA>hsa-mir-942
P3_3 iso-pre-miRNA>hsa-mir-448
P3_3 iso-pre-miRNA>hsa-mir-1182
P3_3 iso-pre-miRNA>hsa-mir-328
P3_3 iso-pre-miRNA>hsa-mir-134
P3_3 iso-pre-miRNA>hsa-mir-4257
P3_3 iso-pre-miRNA>hsa-mir-199b
P3_3 iso-pre-miRNA>hsa-mir-548aa-2
P3_3 iso-pre-miRNA>hsa-mir-203b
P3_3 iso-pre-miRNA>hsa-mir-431
P3_3 iso-pre-miRNA>hsa-mir-152
P3_3 iso-pre-miRNA>hsa-mir-1290
P3_3 iso-pre-miRNA>hsa-mir-3188
P3_3 iso-pre-miRNA>hsa-mir-2053
P3_3 iso-pre-miRNA>hsa-mir-3166
P3_3 iso-pre-miRNA>hsa-mir-95
P3_3 iso-pre-miRNA>hsa-mir-129-2
P3_3 iso-pre-miRNA>hsa-mir-133b
P3_3 iso-pre-miRNA>hsa-mir-3192
P3_3 iso-pre-miRNA>hsa-mir-96
P3_3 iso-pre-miRNA>hsa-mir-211
P3_3 iso-pre-miRNA>hsa-mir-483
P3_3 iso-pre-miRNA>hsa-mir-1251
P3_3 iso-pre-miRNA>hsa-mir-28
P3_3 iso-pre-miRNA>hsa-mir-3936
P3_3 iso-pre-miRNA>hsa-mir-30a
P3_3 iso-pre-miRNA>hsa-mir-181d
P3_3 iso-pre-miRNA>hsa-mir-34a
P3_3 iso-pre-miRNA>hsa-mir-3151
P3_3 iso-pre-miRNA>hsa-mir-1287
P3_3 iso-pre-miRNA>hsa-mir-2355
P3_3 iso-pre-miRNA>hsa-mir-1285-2
P3_3 iso-pre-miRNA>hsa-mir-184

P3_3 iso-pre-miRNA>hsa-mir-664a
P3_3 iso-pre-miRNA>hsa-mir-1322
P3_3 iso-pre-miRNA>hsa-mir-3116-1
P3_3 iso-pre-miRNA>hsa-mir-384
P3_3 iso-pre-miRNA>hsa-mir-1245a
P3_3 iso-pre-miRNA>hsa-mir-452
P3_3 iso-pre-miRNA>hsa-mir-518f
P3_3 iso-pre-miRNA>hsa-mir-146b
P3_3 iso-pre-miRNA>hsa-mir-3671
P3_3 iso-pre-miRNA>hsa-mir-216b
P3_3 iso-pre-miRNA>hsa-mir-4293
P3_3 iso-pre-miRNA>hsa-mir-516a-1
P3_3 iso-pre-miRNA>hsa-mir-503
P3_3 iso-pre-miRNA>hsa-mir-3124
P3_3 iso-pre-miRNA>hsa-mir-3065
P3_3 iso-pre-miRNA>hsa-mir-149
P3_3 iso-pre-miRNA>hsa-mir-3653
P3_3 iso-pre-miRNA>hsa-mir-188
P3_3 iso-pre-miRNA>hsa-mir-3145
P3_3 iso-pre-miRNA>hsa-mir-3155a
P3_3 iso-pre-miRNA>hsa-mir-23b
P3_3 iso-pre-miRNA>hsa-mir-466
P3_3 iso-pre-miRNA>hsa-mir-125b-2
P3_3 iso-pre-miRNA>hsa-mir-3138
P3_3 iso-pre-miRNA>hsa-mir-521-2
P3_3 iso-pre-miRNA>hsa-mir-510
P3_3 iso-pre-miRNA>hsa-mir-514a-1
P3_3 iso-pre-miRNA>hsa-mir-542
P3_3 iso-pre-miRNA>hsa-mir-2682
P3_3 iso-pre-miRNA>hsa-mir-548d-2
P3_3 iso-pre-miRNA>hsa-mir-197
P3_3 iso-pre-miRNA>hsa-mir-323b
P3_3 iso-pre-miRNA>hsa-mir-33a
P3_3 iso-pre-miRNA>hsa-mir-216a
P3_3 iso-pre-miRNA>hsa-mir-489
P3_3 iso-pre-miRNA>hsa-mir-520e
P3_3 iso-pre-miRNA>hsa-mir-138-1
P3_3 iso-pre-miRNA>hsa-mir-2277
P3_3 iso-pre-miRNA>hsa-mir-3162
P3_3 iso-pre-miRNA>hsa-mir-365a
P3_3 iso-pre-miRNA>hsa-mir-449b
P3_3 iso-pre-miRNA>hsa-mir-135b
P3_3 iso-pre-miRNA>hsa-mir-504
P3_3 iso-pre-miRNA>hsa-mir-3620
P3_3 iso-pre-miRNA>hsa-mir-940
P3_3 iso-pre-miRNA>hsa-mir-140
P3_3 iso-pre-miRNA>hsa-mir-182

P3_3 iso-pre-miRNA>hsa-mir-4312
P3_3 iso-pre-miRNA>hsa-mir-519d
P3_3 iso-pre-miRNA>hsa-mir-939
P3_3 iso-pre-miRNA>hsa-mir-3663
P3_3 iso-pre-miRNA>hsa-mir-1307
P3_3 iso-pre-miRNA>hsa-mir-340
P3_3 iso-pre-miRNA>hsa-mir-524
P3_3 iso-pre-miRNA>hsa-mir-520f
P3_3 iso-pre-miRNA>hsa-mir-520h
P3_3 iso-pre-miRNA>hsa-mir-516b-2
P3_3 iso-pre-miRNA>hsa-mir-1303
P3_3 iso-pre-miRNA>hsa-mir-199a-1
P3_3 iso-pre-miRNA>hsa-mir-3134
P3_3 iso-pre-miRNA>hsa-mir-487a
P3_3 iso-pre-miRNA>hsa-mir-1250
P3_3 iso-pre-miRNA>hsa-mir-411
P3_3 iso-pre-miRNA>hsa-mir-548f-2
P3_3 iso-pre-miRNA>hsa-mir-3938
P3_3 iso-pre-miRNA>hsa-mir-499b
P3_3 iso-pre-miRNA>hsa-mir-507
P3_3 iso-pre-miRNA>hsa-mir-548ac
P3_3 iso-pre-miRNA>hsa-mir-675
P3_3 iso-pre-miRNA>hsa-mir-3649
P3_3 iso-pre-miRNA>hsa-mir-425
P3_3 iso-pre-miRNA>hsa-mir-107
P3_3 iso-pre-miRNA>hsa-mir-337
P3_3 iso-pre-miRNA>hsa-mir-3689b
P3_3 iso-pre-miRNA>hsa-mir-223
P3_3 iso-pre-miRNA>hsa-mir-365b
P3_3 iso-pre-miRNA>hsa-mir-521-1
P3_3 iso-pre-miRNA>hsa-mir-203a
P3_3 iso-pre-miRNA>hsa-mir-377
P3_3 iso-pre-miRNA>hsa-mir-4321
P3_3 iso-pre-miRNA>hsa-mir-548ao
P3_3 iso-pre-miRNA>hsa-mir-26b
P3_3 iso-pre-miRNA>hsa-mir-329-2
P3_3 iso-pre-miRNA>hsa-mir-3939
P3_3 iso-pre-miRNA>hsa-mir-33b
P3_3 iso-pre-miRNA>hsa-mir-548at
P3_3 iso-pre-miRNA>hsa-mir-141
P3_3 iso-pre-miRNA>hsa-mir-137
P3_3 iso-pre-miRNA>hsa-mir-665
P3_3 iso-pre-miRNA>hsa-mir-548a-1
P3_3 iso-pre-miRNA>hsa-mir-3927
P3_3 iso-pre-miRNA>hsa-mir-3605
P3_3 iso-pre-miRNA>hsa-mir-128-1
P3_3 iso-pre-miRNA>hsa-mir-30c-2

P3_3 iso-pre-miRNA>hsa-mir-519c
P3_3 iso-pre-miRNA>hsa-mir-517a
P3_3 iso-pre-miRNA>hsa-mir-3123
P3_3 iso-pre-miRNA>hsa-mir-543
P3_3 iso-pre-miRNA>hsa-mir-490
P3_3 iso-pre-miRNA>hsa-mir-302b
P3_3 iso-pre-miRNA>hsa-mir-513a-2
P3_3 iso-pre-miRNA>hsa-mir-1343
P3_3 iso-pre-miRNA>hsa-mir-105-1
P3_3 iso-pre-miRNA>hsa-mir-320a
P3_3 iso-pre-miRNA>hsa-mir-1537
P3_3 iso-pre-miRNA>hsa-mir-218-1
P3_3 iso-pre-miRNA>hsa-mir-133a-1
P3_3 iso-pre-miRNA>hsa-mir-3662
P3_3 iso-pre-miRNA>hsa-mir-302d
P3_3 iso-pre-miRNA>hsa-mir-1197
P3_3 iso-pre-miRNA>hsa-mir-1908
P3_3 iso-pre-miRNA>hsa-mir-3176
P3_3 iso-pre-miRNA>hsa-mir-4278
P3_3 iso-pre-miRNA>hsa-mir-424
P3_3 iso-pre-miRNA>hsa-mir-518a-1
P3_3 iso-pre-miRNA>hsa-mir-1972-2
P3_3 iso-pre-miRNA>hsa-mir-1183
P3_3 iso-pre-miRNA>hsa-mir-3619
P3_3 iso-pre-miRNA>hsa-mir-511
P3_3 iso-pre-miRNA>hsa-mir-518d
P3_3 iso-pre-miRNA>hsa-mir-147b
P3_3 iso-pre-miRNA>hsa-mir-520d
P3_3 iso-pre-miRNA>hsa-mir-495
P3_3 iso-pre-miRNA>hsa-mir-4308
P3_3 iso-pre-miRNA>hsa-mir-2117
P3_3 iso-pre-miRNA>hsa-mir-520g
P3_3 iso-pre-miRNA>hsa-mir-3657
P3_3 iso-pre-miRNA>hsa-mir-514a-3
P3_3 iso-pre-miRNA>hsa-mir-302c
P3_3 iso-pre-miRNA>hsa-mir-499a
P3_3 iso-pre-miRNA>hsa-mir-508
P3_3 iso-pre-miRNA>hsa-mir-382
P3_3 iso-pre-miRNA>hsa-mir-3158-2
P3_3 iso-pre-miRNA>hsa-mir-493
P3_3 iso-pre-miRNA>hsa-mir-3146
P3_3 iso-pre-miRNA>hsa-mir-506
P3_3 iso-pre-miRNA>hsa-mir-3130-1
P3_3 iso-pre-miRNA>hsa-mir-3130-2
P3_3 iso-pre-miRNA>hsa-mir-3117
P3_3 iso-pre-miRNA>hsa-mir-3682
P3_3 iso-pre-miRNA>hsa-mir-3116-2

P3_3 iso-pre-miRNA>hsa-mir-3610
P3_3 iso-pre-miRNA>hsa-mir-363
P3_3 iso-pre-miRNA>hsa-mir-421
P3_3 iso-pre-miRNA>hsa-mir-548a-2
P3_3 iso-pre-miRNA>hsa-mir-372
P3_3 iso-pre-miRNA>hsa-mir-545
P3_3 iso-pre-miRNA>hsa-mir-518e
P3_3 iso-pre-miRNA>hsa-mir-3184
P3_3 iso-pre-miRNA>hsa-mir-205
P3_3 iso-pre-miRNA>hsa-mir-888
P3_3 iso-pre-miRNA>hsa-mir-548f-4
P3_3 iso-pre-miRNA>hsa-mir-433
P3_3 iso-pre-miRNA>hsa-mir-1909
P3_3 iso-pre-miRNA>hsa-mir-548aj-1
P3_3 iso-pre-miRNA>hsa-mir-943
P3_3 iso-pre-miRNA>hsa-mir-320c-1
P3_3 iso-pre-miRNA>hsa-mir-122
P3_3 iso-pre-miRNA>hsa-mir-933
P3_3 iso-pre-miRNA>hsa-mir-1321
P3_3 iso-pre-miRNA>hsa-mir-548ad
P3_3 iso-pre-miRNA>hsa-mir-200b
P3_3 iso-pre-miRNA>hsa-mir-151b
P3_3 iso-pre-miRNA>hsa-mir-20a
P3_3 iso-pre-miRNA>hsa-mir-376a-2
P3_3 iso-pre-miRNA>hsa-mir-3924
P3_3 iso-pre-miRNA>hsa-mir-1185-2
P3_3 iso-pre-miRNA>hsa-mir-181c
P3_3 iso-pre-miRNA>hsa-mir-1281
P3_3 iso-pre-miRNA>hsa-mir-2114
P3_3 iso-pre-miRNA>hsa-mir-183
P3_3 iso-pre-miRNA>hsa-mir-1297
P3_3 iso-pre-miRNA>hsa-mir-4268
P3_3 iso-pre-miRNA>hsa-mir-136
P3_3 iso-pre-miRNA>hsa-mir-1199
P3_3 iso-pre-miRNA>hsa-mir-1258
P3_3 iso-pre-miRNA>hsa-mir-3118-4
P3_3 iso-pre-miRNA>hsa-mir-892c
P3_3 iso-pre-miRNA>hsa-mir-497
P3_3 iso-pre-miRNA>hsa-mir-515-1
P3_3 iso-pre-miRNA>hsa-mir-1913
P3_3 iso-pre-miRNA>hsa-mir-3118-1
P3_3 iso-pre-miRNA>hsa-mir-548aj-2
P3_3 iso-pre-miRNA>hsa-mir-887
P3_3 iso-pre-miRNA>hsa-mir-4252
P3_3 iso-pre-miRNA>hsa-mir-1302-1
P3_3 iso-pre-miRNA>hsa-mir-502
P3_3 iso-pre-miRNA>hsa-mir-1207

P3_3 iso-pre-miRNA>hsa-mir-21
P3_3 iso-pre-miRNA>hsa-mir-1304
P3_3 iso-pre-miRNA>hsa-mir-4320
P3_3 iso-pre-miRNA>hsa-mir-208b
P3_3 iso-pre-miRNA>hsa-mir-548f-3
P3_3 iso-pre-miRNA>hsa-mir-3118-2
P3_3 iso-pre-miRNA>hsa-mir-3149
P3_3 iso-pre-miRNA>hsa-mir-3910-1
P3_3 iso-pre-miRNA>hsa-mir-4281
P3_3 iso-pre-miRNA>hsa-mir-885
P3_3 iso-pre-miRNA>hsa-mir-1302-3
P3_3 iso-pre-miRNA>hsa-mir-4253
P3_3 iso-pre-miRNA>hsa-mir-525
P3_3 iso-pre-miRNA>hsa-mir-124-2
P3_3 iso-pre-miRNA>hsa-mir-4324
P3_3 iso-pre-miRNA>hsa-mir-1302-8
P3_3 iso-pre-miRNA>hsa-mir-222
P3_3 iso-pre-miRNA>hsa-mir-496
P3_3 iso-pre-miRNA>hsa-mir-3126
P3_3 iso-pre-miRNA>hsa-mir-23c
P3_3 iso-pre-miRNA>hsa-mir-1301
P3_3 iso-pre-miRNA>hsa-mir-1185-1
P3_3 iso-pre-miRNA>hsa-mir-2467
P3_3 iso-pre-miRNA>hsa-mir-512-2
P3_3 iso-pre-miRNA>hsa-mir-944
P3_3 iso-pre-miRNA>hsa-mir-548f-1
P3_3 iso-pre-miRNA>hsa-mir-1302-5
P3_3 iso-pre-miRNA>hsa-mir-937
P3_3 iso-pre-miRNA>hsa-mir-1910
P3_3 iso-pre-miRNA>hsa-mir-548ay
P3_3 iso-pre-miRNA>hsa-mir-488
P3_3 iso-pre-miRNA>hsa-mir-3179-1
P3_3 iso-pre-miRNA>hsa-mir-513a-1
P3_3 iso-pre-miRNA>hsa-mir-548f-5
P3_3 iso-pre-miRNA>hsa-mir-194-2
P3_3 iso-pre-miRNA>hsa-mir-2115
P3_3 iso-pre-miRNA>hsa-mir-3622b
P3_3 iso-pre-miRNA>hsa-mir-4279
P3_3 iso-pre-miRNA>hsa-mir-544a
P3_3 iso-pre-miRNA>hsa-mir-661
P3_3 iso-pre-miRNA>hsa-mir-432
P3_3 iso-pre-miRNA>hsa-mir-3919
P3_3 iso-pre-miRNA>hsa-mir-1302-4
P3_3 iso-pre-miRNA>hsa-mir-155
P3_3 iso-pre-miRNA>hsa-mir-342
P3_3 iso-pre-miRNA>hsa-mir-320b-1
P3_3 iso-pre-miRNA>hsa-mir-20b

P3_3 iso-pre-miRNA>hsa-mir-1180
P3_3 iso-pre-miRNA>hsa-mir-93
P3_3 iso-pre-miRNA>hsa-mir-3607
P3_3 iso-pre-miRNA>hsa-mir-3121
P3_3 iso-pre-miRNA>hsa-mir-891a
P3_3 iso-pre-miRNA>hsa-mir-874
P3_3 iso-pre-miRNA>hsa-mir-7-2
P3_3 iso-pre-miRNA>hsa-mir-192
P3_3 iso-pre-miRNA>hsa-mir-498
P3_3 iso-pre-miRNA>hsa-mir-1294
P3_3 iso-pre-miRNA>hsa-mir-1256
P3_3 iso-pre-miRNA>hsa-mir-548aq
P3_3 iso-pre-miRNA>hsa-mir-3928
P3_3 iso-pre-miRNA>hsa-mir-217
P3_3 iso-pre-miRNA>hsa-mir-302f
P3_3 iso-pre-miRNA>hsa-mir-3180-4
P3_3 iso-pre-miRNA>hsa-mir-3180-5
P3_3 iso-pre-miRNA>hsa-mir-92a-1
P3_3 iso-pre-miRNA>hsa-mir-196a-2
P3_3 iso-pre-miRNA>hsa-mir-1264
P3_3 iso-pre-miRNA>hsa-mir-1247
P3_3 iso-pre-miRNA>hsa-mir-1825
P3_3 iso-pre-miRNA>hsa-mir-548i-1
P3_3 iso-pre-miRNA>hsa-mir-664b
P3_3 iso-pre-miRNA>hsa-mir-1292
P3_3 iso-pre-miRNA>hsa-mir-676
P3_3 iso-pre-miRNA>hsa-mir-668
P3_3 iso-pre-miRNA>hsa-mir-892a
P3_3 iso-pre-miRNA>hsa-mir-873
P3_3 iso-pre-miRNA>hsa-mir-892b
P3_3 iso-pre-miRNA>hsa-mir-760
P3_3 iso-pre-miRNA>hsa-mir-92a-2
P3_3 iso-pre-miRNA>hsa-mir-920
P3_3 iso-pre-miRNA>hsa-mir-762
P3_3 iso-pre-miRNA>hsa-mir-99a
P3_3 iso-pre-miRNA>hsa-mir-660
P3_3 iso-pre-miRNA>hsa-mir-758
P3_3 iso-pre-miRNA>hsa-mir-9-2
P3_3 iso-pre-miRNA>hsa-mir-9-1
P3_3 iso-pre-miRNA>hsa-mir-9-3
P3_3 iso-pre-miRNA>hsa-mir-708
P3_3 iso-pre-miRNA>hsa-mir-670
P3_3 iso-pre-miRNA>hsa-mir-92b
P3_3 iso-pre-miRNA>hsa-mir-659
P3_3 iso-pre-miRNA>hsa-mir-767
P3_3 iso-pre-miRNA>hsa-mir-658
P3_3 iso-pre-miRNA>hsa-mir-877

P3_3	iso-pre-miRNA>hsa-mir-766	
P3_3	iso-pre-miRNA>hsa-mir-7-1	
P3_3	iso-pre-miRNA>hsa-mir-671	
P3_3	iso-pre-miRNA>hsa-mir-744	
P3_3	iso-pre-miRNA>hsa-mir-769	
P3_3	iso-pre-miRNA>hsa-mir-98	
P3_3	iso-SNP	133014640
P3_3	iso-SNP	133014633
P3_3	iso-SNP	133014619
P3_3	iso-SNP	133014612
P3_3	iso-SNP	133014579
P3_3	iso-SNP	101036259
P3_3	iso-SNP	64136145
P3_3	iso-SNP	64136160
P3_3	iso-SNP	64136167
P3_3	iso-SNP	64136170
P3_3	iso-SNP	64136103
P3_3	iso-SNP	64136130
P3_3	iso-SNP	46509569
P3_3	iso-SNP	159869828
P3_3	iso-SNP	2140267
P3_3	iso-SNP	2140268
P3_3	iso-SNP	232578091
P3_3	iso-SNP	232578048
P3_3	iso-SNP	232578032
P3_3	iso-SNP	232578099
P3_3	iso-SNP	118310291
P3_3	iso-SNP	118310319
P3_3	iso-SNP	118310349
P3_3	iso-SNP	9392072
P3_3	iso-SNP	46509616
P3_3	iso-SNP	2140270
P3_3	iso-SNP	75679969
P3_3	iso-SNP	75679923
P3_3	iso-SNP	75679958
P3_3	iso-SNP	31924708
P3_3	iso-SNP	47891106
P3_3	iso-SNP	62997470
P3_3	iso-SNP	10662844
P3_3	iso-SNP	10662859
P3_3	iso-SNP	132763362
P3_3	iso-SNP	132763335
P3_3	iso-SNP	132763305
P3_3	iso-SNP	132763298
P3_3	iso-SNP	169455539
P3_3	iso-SNP	2234093
P3_3	iso-SNP	169455502

P3_3	iso-SNP	109757963
P3_3	iso-SNP	34674345
P3_3	iso-SNP	34674326
P3_3	iso-SNP	34820566
P3_3	iso-SNP	34820547
P3_3	iso-SNP	57588322
P3_3	iso-SNP	169455551
P3_3	iso-SNP	101280841
P3_3	iso-SNP	132763295
P3_3	iso-SNP	69002320
P3_3	iso-SNP	69002317
P3_3	iso-SNP	69002304
P3_3	iso-SNP	69002278
P3_3	iso-SNP	232578049
P3_3	iso-SNP	118310287
P3_3	iso-SNP	97824075
P3_3	iso-SNP	97824129
P3_3	iso-SNP	97824125
P3_3	iso-SNP	69002294
P3_3	iso-SNP	74110328
P3_3	iso-SNP	74110353
P3_3	iso-SNP	74110358
P3_3	iso-SNP	101280828
P3_3	iso-SNP	197401372
P3_3	iso-SNP	197401442
P3_3	iso-SNP	197401443
P3_3	iso-SNP	145625537
P3_3	iso-SNP	145625536
P3_3	iso-SNP	145625535
P3_3	iso-SNP	10662866
P3_3	iso-SNP	52196045
P3_3	iso-SNP	52196076
P3_3	iso-SNP	132763351
P3_3	iso-SNP	101530833
P3_3	iso-SNP	101530834
P3_3	iso-SNP	26906437
P3_3	iso-SNP	26906423
P3_3	iso-SNP	26906471
P3_3	iso-SNP	2140269
P3_3	iso-SNP	197401415
P3_3	iso-SNP	134884700
P3_3	iso-SNP	145625542
P3_3	iso-SNP	101280856
P3_3	iso-SNP	101280892
P3_3	iso-SNP	56892464
P3_3	iso-SNP	56892431
P3_3	iso-SNP	1103331

P3_3	iso-SNP	1103312
P3_3	iso-SNP	1103328
P3_3	iso-SNP	45659490
P3_3	iso-SNP	57588336
P3_3	iso-SNP	57588323
P3_3	iso-SNP	183959219
P3_3	iso-SNP	183959222
P3_3	iso-SNP	183959229
P3_3	iso-SNP	183959232
P3_3	iso-SNP	31924631
P3_3	iso-SNP	179225324
P3_3	iso-SNP	168195174
P3_3	iso-SNP	45605666
P3_3	iso-SNP	131007283
P3_3	iso-SNP	131007245
P3_3	iso-SNP	41518005
P3_3	iso-SNP	41517974
P3_3	iso-SNP	41517981
P3_3	iso-SNP	49773087
P3_3	iso-SNP	49773042
P3_3	iso-SNP	7461769
P3_3	iso-SNP	57215164
P3_3	iso-SNP	73402156
P3_3	iso-SNP	101280859
P3_3	iso-SNP	97847807
P3_3	iso-SNP	54468110
P3_3	iso-SNP	54468150
P3_3	iso-SNP	54468144
P3_3	iso-SNP	54468124
P3_3	iso-SNP	127847960
P3_3	iso-SNP	34041842
P3_3	iso-SNP	179225317
P3_3	iso-SNP	101531854
P3_3	iso-SNP	101531857
P3_3	iso-SNP	153410531
P3_3	iso-SNP	35237143
P3_3	iso-SNP	54468166
P3_3	iso-SNP	24736590
P3_3	iso-SNP	99691652
P3_3	iso-SNP	75046227
P3_3	iso-SNP	1785015
P3_3	iso-SNP	1785037
P3_3	iso-SNP	1785042
P3_3	iso-SNP	1785060
P3_3	iso-SNP	1785038
P3_3	iso-SNP	122017258
P3_3	iso-SNP	47891117

P3_3	iso-SNP	47891069
P3_3	iso-SNP	104985443
P3_3	iso-SNP	74110335
P3_3	iso-SNP	97824145
P3_3	iso-SNP	98479274
P3_3	iso-SNP	92003009
P3_3	iso-SNP	820215
P3_3	iso-SNP	820192
P3_3	iso-SNP	820265
P3_3	iso-SNP	131154943
P3_3	iso-SNP	28863695
P3_3	iso-SNP	28863633
P3_3	iso-SNP	28863628
P3_3	iso-SNP	41220094
P3_3	iso-SNP	134884697
P3_3	iso-SNP	54731000
P3_3	iso-SNP	54201692
P3_3	iso-SNP	54201654
P3_3	iso-SNP	54201667
P3_3	iso-SNP	54201668
P3_3	iso-SNP	54240142
P3_3	iso-SNP	54264462
P3_3	iso-SNP	54264394
P3_3	iso-SNP	54264421
P3_3	iso-SNP	135817150
P3_3	iso-SNP	54194212
P3_3	iso-SNP	54240136
P3_3	iso-SNP	54240174
P3_3	iso-SNP	54240184
P3_3	iso-SNP	38010903
P3_3	iso-SNP	56408638
P3_3	iso-SNP	54198496
P3_3	iso-SNP	54211990
P3_3	iso-SNP	54212075
P3_3	iso-SNP	101507720
P3_3	iso-SNP	117637290
P3_3	iso-SNP	114058104
P3_3	iso-SNP	69002271
P3_3	iso-SNP	7461828
P3_3	iso-SNP	7461826
P3_3	iso-SNP	7461827
P3_3	iso-SNP	231155670
P3_3	iso-SNP	67236292
P3_3	iso-SNP	101521092
P3_3	iso-SNP	231155581
P3_3	iso-SNP	150524468
P3_3	iso-SNP	131007001

P3_3	iso-SNP	131007087
P3_3	iso-SNP	131007004
P3_3	iso-SNP	65467665
P3_3	iso-SNP	65467669
P3_3	iso-SNP	38010938
P3_3	iso-SNP	104583838
P3_3	iso-SNP	101347355
P3_3	iso-SNP	101347408
P3_3	iso-SNP	46114610
P3_3	iso-SNP	46114580
P3_3	iso-SNP	56892470
P3_3	iso-SNP	56892507
P3_3	iso-SNP	19223571
P3_3	iso-SNP	19223566
P3_3	iso-SNP	19223639
P3_3	iso-SNP	18392894
P3_3	iso-SNP	18392913
P3_3	iso-SNP	41518007
P3_3	iso-SNP	65467701
P3_3	iso-SNP	113655752
P3_3	iso-SNP	113655748
P3_3	iso-SNP	87909701
P3_3	iso-SNP	87909716
P3_3	iso-SNP	87909749
P3_3	iso-SNP	8007067
P3_3	iso-SNP	43602992
P3_3	iso-SNP	43602984
P3_3	iso-SNP	52013832
P3_3	iso-SNP	52013827
P3_3	iso-SNP	18451300
P3_3	iso-SNP	101531849
P3_3	iso-SNP	101531858
P3_3	iso-SNP	129414568
P3_3	iso-SNP	101531806
P3_3	iso-SNP	99691653
P3_3	iso-SNP	31357244
P3_3	iso-SNP	31357238
P3_3	iso-SNP	31357245
P3_3	iso-SNP	1785030
P3_3	iso-SNP	38010964
P3_3	iso-SNP	2155409
P3_3	iso-SNP	97885708
P3_3	iso-SNP	97885715
P3_3	iso-SNP	188406598
P3_3	iso-SNP	131701185
P3_3	iso-SNP	131701279
P3_3	iso-SNP	134884723

P3_3	iso-SNP	72113306
P3_3	iso-SNP	72113270
P3_3	iso-SNP	72113269
P3_3	iso-SNP	72113261
P3_3	iso-SNP	49773090
P3_3	iso-SNP	131007052
P3_3	iso-SNP	75046184
P3_3	iso-SNP	54194135
P3_3	iso-SNP	13985689
P3_3	iso-SNP	13985739
P3_3	iso-SNP	13985772
P3_3	iso-SNP	9211802
P3_3	iso-SNP	9211747
P3_3	iso-SNP	9211738
P3_3	iso-SNP	104583796
P3_3	iso-SNP	104583828
P3_3	iso-SNP	104583804
P3_3	iso-SNP	104166899
P3_3	iso-SNP	104166902
P3_3	iso-SNP	104166904
P3_3	iso-SNP	9211778
P3_3	iso-SNP	100155033
P3_3	iso-SNP	100155019
P3_3	iso-SNP	100154978
P3_3	iso-SNP	207974738
P3_3	iso-SNP	70480127
P3_3	iso-SNP	70480108
P3_3	iso-SNP	79502141
P3_3	iso-SNP	220373943
P3_3	iso-SNP	220373922
P3_3	iso-SNP	220373939
P3_3	iso-SNP	220373934
P3_3	iso-SNP	10682907
P3_3	iso-SNP	62544470
P3_3	iso-SNP	76139740
P3_3	iso-SNP	70480123
P3_3	iso-SNP	189842826
P3_3	iso-SNP	151128173
P3_3	iso-SNP	54203326
P3_3	iso-SNP	54203333
P3_3	iso-SNP	54203347
P3_3	iso-SNP	104196269
P3_3	iso-SNP	65523519
P3_3	iso-SNP	56227910
P3_3	iso-SNP	56227905
P3_3	iso-SNP	14425204
P3_3	iso-SNP	14425221

P3_3	iso-SNP	54260002
P3_3	iso-SNP	54260009
P3_3	iso-SNP	54260068
P3_3	iso-SNP	54260075
P3_3	iso-SNP	54264461
P3_3	iso-SNP	54264468
P3_3	iso-SNP	133680381
P3_3	iso-SNP	131007109
P3_3	iso-SNP	134884737
P3_3	iso-SNP	249120610
P3_3	iso-SNP	249120591
P3_3	iso-SNP	231155644
P3_3	iso-SNP	54240137
P3_3	iso-SNP	79099712
P3_3	iso-SNP	79099736
P3_3	iso-SNP	79099750
P3_3	iso-SNP	241395420
P3_3	iso-SNP	249120640
P3_3	iso-SNP	41518025
P3_3	iso-SNP	97885720
P3_3	iso-SNP	29729163
P3_3	iso-SNP	49768168
P3_3	iso-SNP	241395500
P3_3	iso-SNP	241395503
P3_3	iso-SNP	138756428
P3_3	iso-SNP	138756369
P3_3	iso-SNP	6194231
P3_3	iso-SNP	189842822
P3_3	iso-SNP	97847498
P3_3	iso-SNP	31203267
P3_3	iso-SNP	70480088
P3_3	iso-SNP	17962644
P3_3	iso-SNP	17962615
P3_3	iso-SNP	249120578
P3_3	iso-SNP	207974741
P3_3	iso-SNP	10080309
P3_3	iso-SNP	54219854
P3_3	iso-SNP	54219857
P3_3	iso-SNP	54219870
P3_3	iso-SNP	54219904
P3_3	iso-SNP	54219912
P3_3	iso-SNP	146353894
P3_3	iso-SNP	146360845
P3_3	iso-SNP	133675375
P3_3	iso-SNP	98510896
P3_3	iso-SNP	98510902
P3_3	iso-SNP	35237129

P3_3	iso-SNP	110141523
P3_3	iso-SNP	101522578
P3_3	iso-SNP	101522582
P3_3	iso-SNP	101522589
P3_3	iso-SNP	101522631
P3_3	iso-SNP	42296995
P3_3	iso-SNP	134884717
P3_3	iso-SNP	249120584
P3_3	iso-SNP	56216187
P3_3	iso-SNP	56216092
P3_3	iso-SNP	101521052
P3_3	iso-SNP	97847535
P3_3	iso-SNP	93113323
P3_3	iso-SNP	93113326
P3_3	iso-SNP	54178990
P3_3	iso-SNP	54179024
P3_3	iso-SNP	54179044
P3_3	iso-SNP	44155754
P3_3	iso-SNP	93113314
P3_3	iso-SNP	93113270
P3_3	iso-SNP	92956443
P3_3	iso-SNP	59362576
P3_3	iso-SNP	13985721
P3_3	iso-SNP	14403144
P3_3	iso-SNP	54466544
P3_3	iso-SNP	26906452
P3_3	iso-SNP	26906403
P3_3	iso-SNP	26906402
P3_3	iso-SNP	205417438
P3_3	iso-SNP	205417483
P3_3	iso-SNP	101522556
P3_3	iso-SNP	137749929
P3_3	iso-SNP	228284991
P3_3	iso-SNP	2321774
P3_3	iso-SNP	2321762
P3_3	iso-SNP	69966994
P3_3	iso-SNP	129410313
P3_3	iso-SNP	69094259
P3_3	iso-SNP	54216629
P3_3	iso-SNP	54216615
P3_3	iso-SNP	145619386
P3_3	iso-SNP	145619442
P3_3	iso-SNP	118927256
P3_3	iso-SNP	10682950
P3_3	iso-SNP	105154091
P3_3	iso-SNP	105154089
P3_3	iso-SNP	179442344

P3_3	iso-SNP	54214286
P3_3	iso-SNP	54214312
P3_3	iso-SNP	54214333
P3_3	iso-SNP	2321809
P3_3	iso-SNP	2321792
P3_3	iso-SNP	101531862
P3_3	iso-SNP	54185441
P3_3	iso-SNP	54185457
P3_3	iso-SNP	54185481
P3_3	iso-SNP	54185492
P3_3	iso-SNP	8007066
P3_3	iso-SNP	8007039
P3_3	iso-SNP	54245768
P3_3	iso-SNP	54228742
P3_3	iso-SNP	54228719
P3_3	iso-SNP	154065347
P3_3	iso-SNP	10928149
P3_3	iso-SNP	10928130
P3_3	iso-SNP	10928119
P3_3	iso-SNP	15738824
P3_3	iso-SNP	101518827
P3_3	iso-SNP	79107068
P3_3	iso-SNP	101489677
P3_3	iso-SNP	101489685
P3_3	iso-SNP	101489703
P3_3	iso-SNP	101489714
P3_3	iso-SNP	101489728
P3_3	iso-SNP	101489745
P3_3	iso-SNP	213291038
P3_3	iso-SNP	55886608
P3_3	iso-SNP	55886574
P3_3	iso-SNP	55886522
P3_3	iso-SNP	33578205
P3_3	iso-SNP	33578255
P3_3	iso-SNP	33578251
P3_3	iso-SNP	33578206
P3_3	iso-SNP	146312590
P3_3	iso-SNP	117102696
P3_3	iso-SNP	2018019
P3_3	iso-SNP	1769533
P3_3	iso-SNP	8007037
P3_3	iso-SNP	145619405
P3_3	iso-SNP	820249
P3_3	iso-SNP	49057623
P3_3	iso-SNP	91352580
P3_3	iso-SNP	179225301
P3_3	iso-SNP	101340844

P3_3	iso-SNP	101340862
P3_3	iso-SNP	137742041
P3_3	iso-SNP	105154097
P3_3	iso-SNP	49768171
P3_3	iso-SNP	13985805
P3_3	iso-SNP	13985806
P3_3	iso-SNP	65238733
P3_3	iso-SNP	65238751
P3_3	iso-SNP	65238767
P3_3	iso-SNP	65238773
P3_3	iso-SNP	65238806
P3_3	iso-SNP	105154084
P3_3	iso-SNP	52013754
P3_3	iso-SNP	29902536
P3_3	iso-SNP	54251902
P3_3	iso-SNP	56408639
P3_3	iso-SNP	104583759
P3_3	iso-SNP	104583776
P3_3	iso-SNP	101528401
P3_3	iso-SNP	2250666
P3_3	iso-SNP	41128620
P3_3	iso-SNP	2321827
P3_3	iso-SNP	219267370
P3_3	iso-SNP	137742013
P3_3	iso-SNP	137741994
P3_3	iso-SNP	137741987
P3_3	iso-SNP	137741989
P3_3	iso-SNP	101493478
P3_3	iso-SNP	167411333
P3_3	iso-SNP	167411388
P3_3	iso-SNP	129410228
P3_3	iso-SNP	129410239
P3_3	iso-SNP	129410235
P3_3	iso-SNP	31357325
P3_3	iso-SNP	17717244
P3_3	iso-SNP	17717243
P3_3	iso-SNP	17717209
P3_3	iso-SNP	113655794
P3_3	iso-SNP	113655806
P3_3	iso-SNP	10682894
P3_3	iso-SNP	40646795
P3_3	iso-SNP	7073282
P3_3	iso-SNP	7073344
P3_3	iso-SNP	46114572
P3_3	iso-SNP	98511634
P3_3	iso-SNP	129410227
P3_3	iso-SNP	219267402

P3_3	iso-SNP	219267407
P3_3	iso-SNP	219267433
P3_3	iso-SNP	69094207
P3_3	iso-SNP	62544495
P3_3	iso-SNP	62544486
P3_3	iso-SNP	101341373
P3_3	iso-SNP	101341407
P3_3	iso-SNP	18572070
P3_3	iso-SNP	112273768
P3_3	iso-SNP	33798031
P3_3	iso-SNP	33798091
P3_3	iso-SNP	136422988
P3_3	iso-SNP	136423009
P3_3	iso-SNP	72086720
P3_3	iso-SNP	54189751
P3_3	iso-SNP	54189752
P3_3	iso-SNP	54201695
P3_3	iso-SNP	54201703
P3_3	iso-SNP	54215608
P3_3	iso-SNP	54251937
P3_3	iso-SNP	241295617
P3_3	iso-SNP	101498399
P3_3	iso-SNP	101498381
P3_3	iso-SNP	136587919
P3_3	iso-SNP	136587934
P3_3	iso-SNP	136588019
P3_3	iso-SNP	113569696
P3_3	iso-SNP	113569695
P3_3	iso-SNP	113569692
P3_3	iso-SNP	113569680
P3_3	iso-SNP	146307414
P3_3	iso-SNP	146307449
P3_3	iso-SNP	101341408
P3_3	iso-SNP	34963445
P3_3	iso-SNP	34963459
P3_3	iso-SNP	34963416
P3_3	iso-SNP	151560719
P3_3	iso-SNP	22102545
P3_3	iso-SNP	236016347
P3_3	iso-SNP	236016316
P3_3	iso-SNP	236016301
P3_3	iso-SNP	117637325
P3_3	iso-SNP	20529956
P3_3	iso-SNP	20529990
P3_3	iso-SNP	207974756
P3_3	iso-SNP	19405702
P3_3	iso-SNP	19405743

P3_3	iso-SNP	135300536
P3_3	iso-SNP	113569211
P3_3	iso-SNP	113569207
P3_3	iso-SNP	113569193
P3_3	iso-SNP	24736638
P3_3	iso-SNP	101491965
P3_3	iso-SNP	101491923
P3_3	iso-SNP	61582680
P3_3	iso-SNP	61582708
P3_3	iso-SNP	593277
P3_3	iso-SNP	33798007
P3_3	iso-SNP	6828032
P3_3	iso-SNP	133680673
P3_3	iso-SNP	54234265
P3_3	iso-SNP	54234315
P3_3	iso-SNP	54234340
P3_3	iso-SNP	54216616
P3_3	iso-SNP	70064261
P3_3	iso-SNP	70064272
P3_3	iso-SNP	70064324
P3_3	iso-SNP	62544490
P3_3	iso-SNP	154065348
P3_3	iso-SNP	21510716
P3_3	iso-SNP	21510699
P3_3	iso-SNP	31357301
P3_3	iso-SNP	46486996
P3_3	iso-SNP	18134042
P3_3	iso-SNP	54238159
P3_3	iso-SNP	54238189
P3_3	iso-SNP	54238203
P3_3	iso-SNP	54238208
P3_3	iso-SNP	117637326
P3_3	iso-SNP	117637302
P3_3	iso-SNP	45725255
P3_3	iso-SNP	45725274
P3_3	iso-SNP	54215584
P3_3	iso-SNP	54223379
P3_3	iso-SNP	54223433
P3_3	iso-SNP	593362
P3_3	iso-SNP	101500098
P3_3	iso-SNP	101500133
P3_3	iso-SNP	101500166
P3_3	iso-SNP	101500167
P3_3	iso-SNP	820197
P3_3	iso-SNP	18572073
P3_3	iso-SNP	55344901
P3_3	iso-SNP	98511725

P3_3	iso-SNP	41522207
P3_3	iso-SNP	54225426
P3_3	iso-SNP	54225437
P3_3	iso-SNP	54225460
P3_3	iso-SNP	54225463
P3_3	iso-SNP	54225490
P3_3	iso-SNP	54225501
P3_3	iso-SNP	112475427
P3_3	iso-SNP	112475429
P3_3	iso-SNP	146366167
P3_3	iso-SNP	56216090
P3_3	iso-SNP	113569519
P3_3	iso-SNP	33578202
P3_3	iso-SNP	33578201
P3_3	iso-SNP	2018004
P3_3	iso-SNP	2018002
P3_3	iso-SNP	146360826
P3_3	iso-SNP	146360813
P3_3	iso-SNP	146360779
P3_3	iso-SNP	146360778
P3_3	iso-SNP	146318507
P3_3	iso-SNP	146318448
P3_3	iso-SNP	101520657
P3_3	iso-SNP	103361245
P3_3	iso-SNP	101335438
P3_3	iso-SNP	101335451
P3_3	iso-SNP	19745034
P3_3	iso-SNP	19745016
P3_3	iso-SNP	19745003
P3_3	iso-SNP	146312254
P3_3	iso-SNP	207648009
P3_3	iso-SNP	207647981
P3_3	iso-SNP	41522213
P3_3	iso-SNP	41522221
P3_3	iso-SNP	67094145
P3_3	iso-SNP	67094150
P3_3	iso-SNP	67094171
P3_3	iso-SNP	54076326
P3_3	iso-SNP	62544503
P3_3	iso-SNP	117886982
P3_3	iso-SNP	133303465
P3_3	iso-SNP	133303425
P3_3	iso-SNP	104196300
P3_3	iso-SNP	61582659
P3_3	iso-SNP	61582649
P3_3	iso-SNP	73438224
P3_3	iso-SNP	135560369

P3_3	iso-SNP	135560387
P3_3	iso-SNP	54291161
P3_3	iso-SNP	101528426
P3_3	iso-SNP	73506984
P3_3	iso-SNP	101500126
P3_3	iso-SNP	103361226
P3_3	iso-SNP	167411298
P3_3	iso-SNP	54233109
P3_3	iso-SNP	54233112
P3_3	iso-SNP	54233113
P3_3	iso-SNP	54233170
P3_3	iso-SNP	54228750
P3_3	iso-SNP	28444104
P3_3	iso-SNP	28444157
P3_3	iso-SNP	209605507
P3_3	iso-SNP	209605539
P3_3	iso-SNP	209605546
P3_3	iso-SNP	145076302
P3_3	iso-SNP	147075204
P3_3	iso-SNP	147075152
P3_3	iso-SNP	147075121
P3_3	iso-SNP	147075120
P3_3	iso-SNP	101348275
P3_3	iso-SNP	1816176
P3_3	iso-SNP	132436375
P3_3	iso-SNP	1988176
P3_3	iso-SNP	1988144
P3_3	iso-SNP	19263542
P3_3	iso-SNP	54228743
P3_3	iso-SNP	54245788
P3_3	iso-SNP	54245789
P3_3	iso-SNP	56118358
P3_3	iso-SNP	56118359
P3_3	iso-SNP	176032376
P3_3	iso-SNP	176032428
P3_3	iso-SNP	85090790
P3_3	iso-SNP	85090809
P3_3	iso-SNP	85090852
P3_3	iso-SNP	220373933
P3_3	iso-SNP	79107049
P3_3	iso-SNP	79107061
P3_3	iso-SNP	15738870
P3_3	iso-SNP	35696481
P3_3	iso-SNP	1102567
P3_3	iso-SNP	1102501
P3_3	iso-SNP	1102563
P3_3	iso-SNP	100575818

P3_3	iso-SNP	22102546
P3_3	iso-SNP	22102543
P3_3	iso-SNP	22102536
P3_3	iso-SNP	207648001
P3_3	iso-SNP	2018056
P3_3	iso-SNP	92003356
P3_3	iso-SNP	101507744
P3_3	iso-SNP	101507727
P3_3	iso-SNP	101507711
P3_3	iso-SNP	167411300
P3_3	iso-SNP	167411334
P3_3	iso-SNP	189842885
P3_3	iso-SNP	101506441
P3_3	iso-SNP	101506465
P3_3	iso-SNP	59064260
P3_3	iso-SNP	101510579
P3_3	iso-SNP	101510612
P3_3	iso-SNP	101510613
P3_3	iso-SNP	13985514
P3_3	iso-SNP	41488550
P3_3	iso-SNP	149396272
P3_3	iso-SNP	129414843
P3_3	iso-SNP	54886166
P3_3	iso-SNP	220771223
P3_3	iso-SNP	117886991
P3_3	iso-SNP	101351048
P3_3	iso-SNP	14184227
P3_3	iso-SNP	180725568
P3_3	iso-SNP	154065385
P3_3	iso-SNP	154065370
P3_3	iso-SNP	154065368
P3_3	iso-SNP	54216650
P3_3	iso-SNP	100575791
P3_3	iso-SNP	100575762
P3_3	iso-SNP	100575761
P3_3	iso-SNP	22049282
P3_3	iso-SNP	145074289
P3_3	iso-SNP	6921289
P3_3	iso-SNP	56408625
P3_3	iso-SNP	56408599
P3_3	iso-SNP	54182261
P3_3	iso-SNP	54182325
P3_3	iso-SNP	54182326
P3_3	iso-SNP	166922908
P3_3	iso-SNP	166922916
P3_3	iso-SNP	167411301
P3_3	iso-SNP	18572056

P3_3	iso-SNP	79107017
P3_3	iso-SNP	22102542
P3_3	iso-SNP	129414852
P3_3	iso-SNP	149396273
P3_3	iso-SNP	149396288
P3_3	iso-SNP	176032424
P3_3	iso-SNP	142667289
P3_3	iso-SNP	37883200
P3_3	iso-SNP	15935316
P3_3	iso-SNP	6489914
P3_3	iso-SNP	6489910
P3_3	iso-SNP	113132857
P3_3	iso-SNP	101530873
P3_3	iso-SNP	49779210
P3_3	iso-SNP	49779214
P3_3	iso-SNP	49779234
P3_3	iso-SNP	31203277
P3_3	iso-SNP	219267443
P3_3	iso-SNP	219267371
P3_3	iso-SNP	129061422
P3_3	iso-SNP	129061441
P3_3	iso-SNP	129061462
P3_3	iso-SNP	57918678
P3_3	iso-SNP	93466919
P3_3	iso-SNP	54233093
P3_3	iso-SNP	54233092
P3_3	iso-SNP	47652913
P3_3	iso-SNP	23887271
P3_3	iso-SNP	23887220
P3_3	iso-SNP	23887219
P3_3	iso-SNP	109849556
P3_3	iso-SNP	213291041
P3_3	iso-SNP	21038150
P3_3	iso-SNP	143163790
P3_3	iso-SNP	143163772
P3_3	iso-SNP	149396242
P3_3	iso-SNP	77879071
P3_3	iso-SNP	145074284
P3_3	iso-SNP	94398581
P3_3	iso-SNP	94398543
P3_3	iso-SNP	65523495
P3_3	iso-SNP	176056492
P3_3	iso-SNP	145074342
P3_3	iso-SNP	10436238
P3_3	iso-SNP	10436194
P3_3	iso-SNP	10436219
P3_3	iso-SNP	10436201

P3_3	iso-SNP	93466913
P3_3	iso-SNP	114340663
P3_3	iso-SNP	19744982
P3_3	iso-SNP	593323
P3_3	iso-SNP	97847569
P3_3	iso-SNP	97847573
P3_3	iso-SNP	23189715
P3_3	iso-SNP	77879051
P3_3	iso-SNP	54200830
P3_3	iso-SNP	54200810
P3_3	iso-SNP	65291712
P3_3	iso-SNP	65291793
P3_3	iso-SNP	65291808
P3_3	iso-SNP	37883195
P3_3	iso-SNP	37883165
P3_3	iso-SNP	145074283
P3_3	iso-SNP	69966985
P3_3	iso-SNP	145076355
P3_3	iso-SNP	49812123
P3_3	iso-SNP	100125949
P3_3	iso-SNP	100125939
P3_3	iso-SNP	100125865
P3_3	iso-SNP	100125848
P3_3	iso-SNP	45606504
P3_3	iso-SNP	45606510
P3_3	iso-SNP	101526951
P3_3	iso-SNP	69330886
P3_3	iso-SNP	69330825
P3_3	iso-SNP	69330824
P3_3	iso-SNP	20035242
P3_3	iso-SNP	92956422
P3_3	iso-SNP	25551588
P3_3	iso-SNP	25551583
P3_3	iso-SNP	25551550
P3_3	iso-SNP	101509363
P3_3	iso-SNP	146307411
P3_3	iso-SNP	146307375
P3_3	iso-SNP	213291030
P3_3	iso-SNP	94398600
P3_3	iso-SNP	240273490
P3_3	iso-SNP	54172508
P3_3	iso-SNP	54172483
P3_3	iso-SNP	54172501
P3_3	iso-SNP	189547778
P3_3	iso-SNP	189547779
P3_3	iso-SNP	189547761
P3_3	iso-SNP	188406636

P3_3	iso-SNP	56367696
P3_3	iso-SNP	49231248
P3_3	iso-SNP	49231303
P3_3	iso-SNP	144895179
P3_3	iso-SNP	144895169
P3_3	iso-SNP	144895168
P3_3	iso-SNP	144895170
P3_3	iso-SNP	189547748
P3_3	iso-SNP	57215120
P3_3	iso-SNP	54228775
P3_3	iso-SNP	85775260
P3_3	iso-SNP	35696518
P3_3	iso-SNP	35696519
P3_3	iso-SNP	35696532
P3_3	iso-SNP	32547811
P3_3	iso-SNP	32547810
P3_3	iso-SNP	32547809
P3_3	iso-SNP	32547795
P3_3	iso-SNP	176998548
P3_3	iso-SNP	59362602
P3_3	iso-SNP	45606472
P3_3	iso-SNP	45606471
P3_3	iso-SNP	54731071
P3_3	iso-SNP	54731080
P3_3	iso-SNP	14995387
P3_3	iso-SNP	143163775
P3_3	iso-SNP	143163799
P3_3	iso-SNP	143163816
P3_3	iso-SNP	146295085
P3_3	iso-SNP	146295067
P3_3	iso-SNP	142667306
P3_3	iso-SNP	142667330
P3_3	iso-SNP	142667356
P3_3	iso-SNP	10436187
P3_3	iso-SNP	32659592
P3_3	iso-SNP	64658904
P3_3	iso-SNP	48357921
P3_3	iso-SNP	145619365
P3_3	iso-SNP	18134045
P3_3	iso-SNP	27559261
P3_3	iso-SNP	27559214
P3_3	iso-SNP	241295614
P3_3	iso-SNP	1816169
P3_3	iso-SNP	31936263
P3_3	iso-SNP	101515025
P3_3	iso-SNP	101515083
P3_3	iso-SNP	113132901

P3_3	iso-SNP	64658836
P3_3	iso-SNP	64658828
P3_3	iso-SNP	145019437
P3_3	iso-SNP	145019401
P3_3	iso-SNP	145019377
P3_3	iso-SNP	101350873
P3_3	iso-SNP	159000436
P3_3	iso-SNP	145619377
P3_3	iso-SNP	18134091
P3_3	iso-SNP	208134116
P3_3	iso-SNP	208134114
P3_3	iso-SNP	208134058
P3_3	iso-SNP	208134047
P3_3	iso-SNP	208134017
P3_3	iso-SNP	54076332
P3_3	iso-SNP	101526957
P3_3	iso-SNP	101526987
P3_3	iso-SNP	101526991
P3_3	iso-SNP	19405709
P3_3	iso-SNP	26946325
P3_3	iso-SNP	145019376
P3_3	iso-SNP	100576059
P3_3	iso-SNP	100576035
P3_3	iso-SNP	117214376
P3_3	iso-SNP	1102498
P3_3	iso-SNP	249120631
P3_3	iso-SNP	133303896
P3_3	iso-SNP	19247825
P3_3	iso-SNP	69330818
P3_3	iso-SNP	2155379
P3_3	iso-SNP	176032415
P3_3	iso-SNP	99691429
P3_3	iso-SNP	99691396
P3_3	iso-SNP	176032408
P3_3	iso-SNP	85916322
P3_3	iso-SNP	101520687
P3_3	iso-SNP	21038166
P3_3	iso-SNP	180407505
P3_3	iso-SNP	180407488
P3_3	iso-SNP	180407512
P3_3	iso-SNP	145076356
P3_3	iso-SNP	145109356
P3_3	iso-SNP	136983326
P3_3	iso-SNP	54228774
P3_3	iso-SNP	89155064
P3_3	iso-SNP	64658715
P3_3	iso-SNP	64658716

P3_3	iso-SNP	54177532
P3_3	iso-SNP	54177552
P3_3	iso-SNP	54177559
P3_3	iso-SNP	54177564
P3_3	iso-SNP	153726756
P3_3	iso-SNP	153726760
P3_3	iso-SNP	153726707
P3_3	iso-SNP	21314920
P3_3	iso-SNP	21314829
P3_3	iso-SNP	153726728
P3_3	iso-SNP	153726769
P3_3	iso-SNP	54177457
P3_3	iso-SNP	56210210
P3_3	iso-SNP	56210207
P3_3	iso-SNP	54177523
P3_3	iso-SNP	15248787
P3_3	iso-SNP	15248717
P3_3	iso-SNP	2186069
P3_3	iso-SNP	2186044
P3_3	iso-SNP	2186129
P3_3	iso-SNP	2186117
P3_3	iso-SNP	2186087
P3_3	iso-SNP	15248801
P3_3	iso-SNP	92003588
P3_3	iso-SNP	15248798
P3_3	iso-SNP	2186054
P3_3	iso-SNP	15248726
P3_3	iso-SNP	15248720
P3_3	iso-SNP	56216156
P3_3	iso-SNP	54385561
P3_3	iso-SNP	145074272
P3_3	iso-SNP	89155073
P3_3	iso-SNP	31556085
P3_3	iso-SNP	41488568
P3_3	iso-SNP	153996899
P3_3	iso-SNP	153996903
P3_3	iso-SNP	69242707
P3_3	iso-SNP	69242767
P3_3	iso-SNP	101521608
P3_3	iso-SNP	101521620
P3_3	iso-SNP	145078244
P3_3	iso-SNP	145078200
P3_3	iso-SNP	28888922
P3_3	iso-SNP	145076376
P3_3	iso-SNP	145078733
P3_3	iso-SNP	136983319
P3_3	iso-SNP	94312442

P3_3	iso-SNP	10682898
P3_3	iso-SNP	133303579
P3_3	iso-SNP	92003589
P3_3	iso-SNP	15935347
P3_3	iso-SNP	145109328
P3_3	iso-SNP	24365358
P3_3	iso-SNP	24365383
P3_3	iso-SNP	8007104
P3_3	iso-SNP	30905269
P3_3	iso-SNP	30905298
P3_3	iso-SNP	99691401
P3_3	iso-SNP	17911447
P3_3	iso-SNP	49777907
P3_3	iso-SNP	129414574
P3_3	iso-SNP	129414553
P3_3	iso-SNP	101492444
P3_3	iso-SNP	87962747
P3_3	iso-SNP	189547735
P3_3	iso-SNP	1988193
P3_3	iso-SNP	1988170
P3_3	iso-SNP	156390147
P3_3	iso-SNP	89911256
P3_3	iso-SNP	79113119
P3_3	iso-SNP	43581287
P3_3	iso-SNP	155165044
P3_3	iso-SNP	38243770
P3_3	iso-SNP	38243743
P3_3	iso-SNP	151561912
P3_3	iso-SNP	30552168
P3_3	iso-SNP	30552187
P3_3	iso-SNP	118780790
P3_3	iso-SNP	118780738
P3_3	iso-SNP	86584720
P3_3	iso-SNP	86584707
P3_3	iso-SNP	150935522
P3_3	iso-SNP	150935577
P3_3	iso-SNP	11985275
P3_3	iso-SNP	46522190
P3_3	iso-SNP	46522201
P3_3	iso-SNP	53583294
P3_3	iso-SNP	2140204
P3_3	iso-SNP	2140240
P3_3	iso-SNP	2234086
P3_3	iso-SNP	197401371
P3_3	iso-SNP	45659500
P3_3	iso-SNP	153410520
P3_3	iso-SNP	54468094

P3_3	iso-SNP	54198499
P3_3	iso-SNP	97885730
P3_3	iso-SNP	70480051
P3_3	iso-SNP	65523500
P3_3	iso-SNP	31203228
P3_3	iso-SNP	31203221
P3_3	iso-SNP	31203216
P3_3	iso-SNP	31203207
P3_3	iso-SNP	98510864
P3_3	iso-SNP	110141578
P3_3	iso-SNP	92956420
P3_3	iso-SNP	92956416
P3_3	iso-SNP	92956412
P3_3	iso-SNP	92956409
P3_3	iso-SNP	69967005
P3_3	iso-SNP	54216681
P3_3	iso-SNP	10682934
P3_3	iso-SNP	54245780
P3_3	iso-SNP	146312589
P3_3	iso-SNP	117102674
P3_3	iso-SNP	117102660
P3_3	iso-SNP	117102649
P3_3	iso-SNP	41128599
P3_3	iso-SNP	41128578
P3_3	iso-SNP	40646803
P3_3	iso-SNP	2321820
P3_3	iso-SNP	151560699
P3_3	iso-SNP	19405676
P3_3	iso-SNP	19405672
P3_3	iso-SNP	101491974
P3_3	iso-SNP	101491981
P3_3	iso-SNP	593365
P3_3	iso-SNP	54216670
P3_3	iso-SNP	79502168
P3_3	iso-SNP	146360765
P3_3	iso-SNP	1988119
P3_3	iso-SNP	54245841
P3_3	iso-SNP	41488558
P3_3	iso-SNP	129414807
P3_3	iso-SNP	129414815
P3_3	iso-SNP	154065383
P3_3	iso-SNP	22102519
P3_3	iso-SNP	70480062
P3_3	iso-SNP	15935348
P3_3	iso-SNP	93466912
P3_3	iso-SNP	93466910
P3_3	iso-SNP	93466909

P3_3	iso-SNP	93466866
P3_3	iso-SNP	114340628
P3_3	iso-SNP	114340631
P3_3	iso-SNP	114340615
P3_3	iso-SNP	114340550
P3_3	iso-SNP	54200853
P3_3	iso-SNP	69330871
P3_3	iso-SNP	144895159
P3_3	iso-SNP	144895164
P3_3	iso-SNP	48357853
P3_3	iso-SNP	48357864
P3_3	iso-SNP	69330823
P3_3	iso-SNP	99691393
P3_3	iso-SNP	176032384
P3_3	iso-SNP	21038172
P3_3	iso-SNP	136983281
P3_3	iso-SNP	89155084
P3_3	iso-SNP	64658705
P3_3	iso-SNP	64658710
P3_3	iso-SNP	153726803
P3_3	iso-SNP	56210168
P3_3	iso-SNP	56210117
P3_3	iso-SNP	56210140
P3_3	iso-SNP	54385599
P3_3	iso-SNP	153996905
P3_3	iso-SNP	10436180
P3_3	iso-SNP	38243739
P3_3	iso-SNP	86584675
P3_3	iso-SNP	150935583
P3_3	iso-SNP	46522255
P3_3	iso-SNP	153410482
P3_3	iso-SNP	101507779
P3_3	iso-SNP	98510847
P3_3	iso-SNP	69967062
P3_3	iso-SNP	54245827
P3_3	iso-SNP	105154013
P3_3	iso-SNP	40646816
P3_3	iso-SNP	146312541
P3_3	iso-SNP	1988116
P3_3	iso-SNP	129414806
P3_3	iso-SNP	166922862
P3_3	iso-SNP	146312560
P3_3	iso-SNP	89155121
P3_3	iso-SNP	64658623
P3_3	iso-SNP	64658640
P3_3	iso-SNP	136983275
P3_3	iso-SNP	15935351

P3_3	iso-SNP	38243727
P3_3	iso-SNP	46522298
P3_3	iso-SNP	40646834
P3_3	iso-SNP	129414804
P3_3	iso-SNP	89155162
P3_3	iso-SNP	89155163
