# Supplementary material for: Role of SNPs in the Biogenesis of Mature miRNAs
Source: Biomed Res Int. 2021 Jun 17;2021:2403418. doi: 10.1155/2021/2403418 (PMC8233088; doi:10.1155/2021/2403418)
Supplement: Supplementary Materials — Additional file 1 Table S1: the database miRSNPBase (xls). Additional file 2 Table S2: the list of pre-miRNAs in miRSNPBase which is classified based on mature miRNA in the 5′ arm or 3′ arm (xls). Additional file 3 Table S3: all the iso-pre-miRNAs, nor-pre-miRNAs, nor-SNPs, and iso-SNPs associated with four splicing sites (xls). Additional file 4 Table S4: the pre-miRNAs and SNPs associated with the normal and isomiRs (xls). Additional file 5 Table S5: the pre-miRNAs, iso-SNPs, and isomiRs of HG00097 (xls). Additional file 6 Table S6: the isomiRs and iso-SNP of 18 GBR populations (xls). Additional file 7 Table S7: the verified isomiRs of 18 GBR (xls). Additional file 8 Table S8: the iso-pre-miRNA candidates and the verified iso-pre-miRNAs of 18 GBR samples (xls). [file 2403418.f1.zip › 2403418.f1/Supp Tab S4.pdf]

nor-pre-miRNA	nor-SNP	iso-pre-miRNA	iso-SNP
>hsa-mir-1250	1062574	>hsa-mir-1237	1103312
>hsa-mir-1255b-2	1062599	>hsa-mir-769	1103328
>hsa-mir-125b-2	1062626	>hsa-mir-671	1785015
>hsa-mir-1304	1062653	>hsa-mir-7-1	1785030
>hsa-mir-133a-1	1062656	>hsa-mir-1304	1785037
>hsa-mir-136	1062662	>hsa-mir-3620	1785038
>hsa-mir-154	1103284	>hsa-mir-2277	1785042
>hsa-mir-16-1	1103312	>hsa-mir-194-2	1785060
>hsa-mir-183	1103328	>hsa-mir-939	1988193
>hsa-mir-1910	1103331	>hsa-mir-95	2018002
>hsa-mir-1911	7126619	>hsa-mir-128-1	2018004
>hsa-mir-1914	7126698	>hsa-mir-203b	2018019
>hsa-mir-1915	9211738	>hsa-mir-412	2018056
>hsa-mir-192	9211747	>hsa-mir-937	2140204
>hsa-mir-196b	9211778	>hsa-mir-942	2140240
>hsa-mir-200a	9211782	>hsa-mir-1908	2140267
>hsa-mir-202	9211802	>hsa-mir-876	2140268
>hsa-mir-203b	16645137	>hsa-mir-885	2140269
>hsa-mir-215	16645178	>hsa-mir-888	2140270
>hsa-mir-2277	17962615	>hsa-mir-892c	8007037
>hsa-mir-24-1	19405672	>hsa-mir-93	8007039
>hsa-mir-26b	19405676	>hsa-mir-675	8007066
>hsa-mir-296	19405743	>hsa-mir-874	8007067
>hsa-mir-299	21785508	>hsa-mir-887	8007104
>hsa-mir-302a	27209165	>hsa-mir-433	10436180
>hsa-mir-3074	46522190	>hsa-mir-516a-2	10436187
>hsa-mir-3126	46522201	>hsa-mir-96	10436194
>hsa-mir-3127	46522255	>hsa-mir-523	10436201
>hsa-mir-3158-2	46522298	>hsa-mir-3177	10436219
>hsa-mir-324	49767769	>hsa-mir-142	10436238
>hsa-mir-338	49767815	>hsa-mir-3124	15935316
>hsa-mir-339	49767832	>hsa-mir-200a	15935347
>hsa-mir-34a	49767835	>hsa-mir-504	15935351
>hsa-mir-361	49767838	>hsa-mir-1225	28863628
>hsa-mir-362	49773603	>hsa-mir-1292	28863633
>hsa-mir-367	50623110	>hsa-mir-197	28863695
>hsa-mir-3679	50623143	>hsa-mir-1224	46522201
>hsa-mir-371a	54200810	>hsa-mir-1233-1	46522255
>hsa-mir-380	54200826	>hsa-mir-1233-2	46522298
>hsa-mir-3928	54200830	>hsa-mir-196a-2	54201654
>hsa-mir-455	54200834		54201667
>hsa-mir-505	54200843		54201668
>hsa-mir-514a-1	54200853		54201692
>hsa-mir-517b	54216615		54201695
>hsa-mir-519d	54216629		54201703
>hsa-mir-520g	54216650		54264394

>hsa-mir-525 54216681		54264421
>hsa-mir-532 54225426		54264462
>hsa-mir-548am	54225460	54385599
>hsa-mir-548ap	54225463	56408599
>hsa-mir-548aq	54225501	56408625
>hsa-mir-548as	54290994	56408638
>hsa-mir-664a57392686		56408639
>hsa-mir-664b57392697		61582649
>hsa-mir-769 57392715		61582659
>hsa-mir-874 62572847		61582708
	62572874	64136103
	62572885	64136130
	64658623	64136145
	64658640	64136160
	64658710	64136167
	64658715	64136170
	64658716	64658828
	69330818	64658836
	69330823	86584707
	69330824	86584720
	69330825	92956409
	69330871	92956412
	69330886	92956416
	79099736	92956420
	79107017	92956422
	79107049	92956443
	79107061	93466866
	79107068	93466910
	79107084	93466912
	85158670	93466919
	85775275	99691396
	86368890	99691401
	86368898	99691429
	86368922	101348275
	86368929	101531806
	86368959	101531849
	92956409	101531854
	92956416	101531857
	92956420	101531858
	93466866	101531862
	93466909	104583759
	93466910	104583804
	93466912	110141578
	93466919	117637302
	97464049	117637325
	97464057	117637326
	97848319	129414568

97848343
101351048
101351088
101490145
101490178
101491407
101526116
103361221
103361226
103361245
104583759
104583776
104583796
104583804
104583828
104583838
113569054
113569088
113569394
113569406
113997768
113997812
113997817
116971750
116971778
129414804
129414806
129414807
129414815
129414843
129414852
134884697
134884700
134884717
134884723
135061039
135061106
135061109
135061111
135061112
135061124
136983275
136983319
139006339
139006378
146360765
146360778
129414574
136422988
136423009
136983275
136983281
136983319
136983326
137749929
144895159
144895164
144895168
144895169
144895170
144895179
145074272
145074283
145074284
145074289
145074342
145076302
145076355
145076356
145076376
145619365
145619377
145619386
145619405
145619442
150935522
150935577
183959232
228284991
249120578
249120591
249120610
249120640

146360779
146360813
146360845
167967953
167967958
167967963
219267370
219267371
219267402
219267407
219267433
219267443
220291292
220291302
220373922
220373933
220373934
220373939
220373943
