# Supplementary material for: Role of SNPs in the Biogenesis of Mature miRNAs
Source: Biomed Res Int. 2021 Jun 17;2021:2403418. doi: 10.1155/2021/2403418 (PMC8233088; doi:10.1155/2021/2403418)
Supplement: Supplementary Materials — Additional file 1 Table S1: the database miRSNPBase (xls). Additional file 2 Table S2: the list of pre-miRNAs in miRSNPBase which is classified based on mature miRNA in the 5′ arm or 3′ arm (xls). Additional file 3 Table S3: all the iso-pre-miRNAs, nor-pre-miRNAs, nor-SNPs, and iso-SNPs associated with four splicing sites (xls). Additional file 4 Table S4: the pre-miRNAs and SNPs associated with the normal and isomiRs (xls). Additional file 5 Table S5: the pre-miRNAs, iso-SNPs, and isomiRs of HG00097 (xls). Additional file 6 Table S6: the isomiRs and iso-SNP of 18 GBR populations (xls). Additional file 7 Table S7: the verified isomiRs of 18 GBR (xls). Additional file 8 Table S8: the iso-pre-miRNA candidates and the verified iso-pre-miRNAs of 18 GBR samples (xls). [file 2403418.f1.zip › 2403418.f1/Supp Tab S5.pdf]

pre-miRNA isomiR isomiR isomiR isomiR isomiR SNP position snp

>hsa-mir-548aj-2 AAAAACTGCAATTACTTTTACA TGGTAAAAACTGCAATTACTTT
AACTGCAATTACTTTTACACCA AATTACTTTTACACCAACCTAA 37883200
rs73463468
>hsa-mir-548ad AAAACGACAATGACTTTTGCAC ACTTTTGCACCAATCTAATAC
CTTTTGCACCAATCTAATAC GCAAAAACGACAATGACTTTTG
CAAAAACGACAATGACTTTTGC 35696519 rs62143301
>hsa-mir-202 AAAGAGGTATAGGGCATGGGAA AAGAGGTATAGGGCATGGGAAA
GGGAAAACGGGGCGGTCGGGTC TAAAGAGGTATAGGGCATGGGA 135061112
rs12355840
>hsa-mir-548ap AACAAAAACCACAATTACTTTT CAAAAACCACAATTACTTTTTA
CAATTACTTTTTACTGACCTAA rs4414449

>hsa-mir-548ap AACAAAAACCACAATTACTTTT AACAAAAACCACAATTACTTTT
CAAAAACCACAATTACTTTTTA TTACTTTTTACTGACCTAAAGA 86368959
rs4577031
>hsa-mir-548ap AACAAAAACCACAATTACTTTT CAAAAACCACAATTACTTTTTA
CAATTACTTTTTACTGACCTAA 86368959 rs4577031
rs4414449
>hsa-mir-548ad AACGACAATGACTTTTGCACCA GGCAAAAACGACAATGACTTTT
TGGCAAAAACGACAATGACTTT AAAAACGACAATGACTTTTGCA 35696519
rs62143301
>hsa-mir-548al AACGGCAGTGACTTTTGTACCA TGGCAAAAACGGCAGTGACTTT
TAAAAGTAATGGCAAAAACGGC AAAAGTAATGGCAAAAACGGCA 74110353
rs515924
>hsa-mir-423 AAGCTCGGTCTGAGGCCCCTCA AGGCCCCTCAGTCTTGCTTCCT
TCTGAGGCCCCTCAGTCTTGCT GTCTGAGGCCCCTCAGTCTTGC 28444183
rs6505162
>hsa-mir-3144AAGGGGACCAAAGAGATATATA TAAGGGGACCAAAGAGATATAT
TTAAGGGGACCAAAGAGATATA TTTAAGGGGACCAAAGAGATAT
ATACACTTTAAGGGGACCAAAG 120336327 rs68035463
>hsa-mir-3671AATAAGGACTAGTCTGCAGTGA TTTATTTCTATCAAATAAGGAC
TTTTATTTCTATCAAATAAGGA GGACTAGTCTGCAGTGATAT
AAATAAGGACTAGTCTGCAGTG 65523519 rs521188
>hsa-mir-622 ACACAGTCTGCTGAGGTTGGAG CTGCTGAGGTTGGAGCCGCTGA
ACACAGTCTGCTGAGGTTGGAG rs111371406

>hsa-mir-24-2ACACTGGCTCAGTTCAGCAGGA CACTGGCTCAGTTCAGCAGGAA
TGGCTCAGTTCAGCAGGAACAG CTGGCTCAGTTCAGCAGGAACA 13947170
.
>hsa-mir-597 ACAGTGGTTCTCTTGTGGCTTA GGCTTAAGCGTAATGTAGAGTA
AATGTACAGTGGTTCTCTTGTG TGTACAGTGGTTCTCTTGTGGC 9599255
rs146125159
>hsa-mir-3141ACCCGGTGAGGGCGGGTGGAGG CGGTGAGGGCGGGTGGAGGAGG
CCGGTGAGGGCGGGTGGAGGAG CCCGGTGAGGGCGGGTGGAGGA
CACCCGGTGAGGGCGGGTGGAG 153975576 rs936581

>hsa-mir-1307ACCGGACCTCGACCGGCTCGTC CGGACCTCGACCGGCTCGTCTG CCGGACCTCGACCGGCTCGTCT ATCTCGACCGGACCTCGACCGG AATCTCGACCGGACCTCGACCG 105154089 rs7911488
>hsa-mir-486-2 ACTGAGCTGCCCCGAGCTGGGC CTGAGCTGCCCCGAGCTGGGCA CTGTACTGAGCTGCCCCGAGCT CCTGTACTGAGCTGCCCCGAGC GTACTGAGCTGCCCCGAGCTGG 41518007 .
>hsa-mir-3118-1 ACTGCATTATGAAAATTCTTCT ATTATGAAAATTCTTCTAGTGT GCATTATGAAAATTCTTCTAGT CTGCATTATGAAAATTCTTCTA 142667330 rs76132421
>hsa-mir-3125AGAATGGATAGAGGAAGCTGTG GAGGAAGCTGTGGAGAGAACTC AGAGGAAGCTGTGGAGAGAACT GCTGTGGAGAGAACTCACGGTG GGAAGCTGTGGAGAGAACTCAC 12877501 rs78852835
>hsa-mir-642bAGATACATTTGGAGAGGGACCC TTGGAGAGGGACCCTCCCAACT TTTGGAGAGGGACCCTCCCAAC ATACATTTGGAGAGGGACCCTC 46178217 rs111664333
>hsa-mir-378hAGATGGGATGAGCCCTGGCTCT TCAGATGGGATGAGCCCTGGCT TGTCAGATGGGATGAGCCCTGG GATGGGATGAGCCCTGGCTCTG CAGATGGGATGAGCCCTGGCTC 154209024 rs702742
>hsa-mir-646 AGCAGCTGCCTCTGAGGCCTCA CTGAGGCCTCAGGCTCAGTGGC CTCTGAGGCCTCAGGCTCAGTG GCCTCTGAGGCCTCAGGCTCAG TCTGAGGCCTCAGGCTCAGTGG 58883534 rs6513496
>hsa-mir-1269b AGCCATGCTACGGGCTTCTCTG ACTGAGCCATGCTACGGGCTTC AGGTTTCTGGACTGAGCCATGC TGAGGTTTCTGGACTGAGCCAT TTTCTGGACTGAGCCATGCTAC 12820632 rs12451747
>hsa-mir-3135b AGCGAGTGCAGTGGTGCAGTCA AGGCTGGAGCGAGTGCAGTGGT CTGGAGCGAGTGCAGTGGTGCA CAGGCTGGAGCGAGTGCAGTGG CCAGGCTGGAGCGAGTGCAGTG 32717702 rs4285314
>hsa-mir-3180-4 AGCGGAGGGTGAAGCCTCCGGA CGGAGGGTGAAGCCTCCGGATG GAGCGGAGGGTGAAGCCTCCGG GCGGAGGGTGAAGCCTCCGGAT 15248798 rs183853838
>hsa-mir-3180-4 AGCGGAGGGTGAAGCCTCCGGA CGGAGGGTGAAGCCTCCGGATG GAGCGGAGGGTGAAGCCTCCGG GCGGAGGGTGAAGCCTCCGGAT 15248720 rs75000738 15248798 rs183853838
>hsa-mir-3125AGCTGTGGAGAGAACTCACGGT AAGCTGTGGAGAGAACTCACGG TAGAGGAAGCTGTGGAGAGAAC TGGATAGAGGAAGCTGTGGAGA AGGAAGCTGTGGAGAGAACTCA 12877501 rs78852835
>hsa-mir-30d AGCTTTCAGTCAGATGTTTGCT GGCTAAGCTTTCAGTCAGATGT GCTAAGCTTTCAGTCAGATGTT TTCAGTCAGATGTTTGCTGCTA 135817150 .
>hsa-mir-519a-2 AGGAAAGTGCATCCTTTTAGAG AGTGCATCCTTTTAGAGGGTTA GGAAAGTGCATCCTTTTAGAGG GAAAGGAAAGTGCATCCTTTTA 54265670 .
>hsa-mir-646 AGGAAGCAGCTGCCTCTGAGGC GCTGCCTCTGAGGCCTCAGGCT CTGAGGCCTCAGGCTCAGTGGC TCTGAGGCCTCAGGCTCAGTGG 58883534 rs6513496
>hsa-mir-149 AGGGAGGGAGGGACGGGGGCTG GGGCTGTGCTGGGGCAGCCGGA GAGGGAGGGAGGGACGGGGGCT GGGACGGGGGCTGTGCTGGGGC

GGGAGGGAGGGACGGGGGCTGT 241395503 rs2292832
>hsa-mir-658 AGGTCGGTTGGTCGGTCGGGAA GTCGGTTGGTCGGTCGGGAACG
TAGGTCGGTTGGTCGGTCGGGA
>hsa-mir-548ap AGTAATTGCAGTCTTTGTCATT AAGTAATTGCAGTCTTTGTCAT
AAAGTAATTGCAGTCTTTGTCA AAAAGTAATTGCAGTCTTTGTC
CAAAAGTAATTGCAGTCTTTGT 86368898 rs4414449
>hsa-mir-548ap AGTAATTGCAGTCTTTGTCATT AAGTAATTGCAGTCTTTGTCAT
AAAGTAATTGCAGTCTTTGTCA AAAAGTAATTGCAGTCTTTGTC
CAAAAGTAATTGCAGTCTTTGT 86368898 rs4414449 86368959
rs4577031
>hsa-mir-548aj-2 AGTAATTGCAGTTTTTGCCATT AAGTAATTGCAGTTTTTGCCAT
AAAGTAATTGCAGTTTTTGCCA TGCAAAAGTAATTGCAGTTTTT
AAAAGTAATTGCAGTTTTTGCC 37883200 rs73463468
>hsa-mir-548ap AGTAATTGCGGTCTTTGTCATT AAGTAATTGCGGTCTTTGTCAT
AAAGTAATTGCGGTCTTTGTCA AAAAGTAATTGCGGTCTTTGTC
CAAAAGTAATTGCGGTCTTTGT 86368959 rs4577031
>hsa-mir-630 AGTATTCTGTACCAGGGAAGGT ACCTAGTATTCTGTACCAGGGA
CCAGGGAAGGTAGTTCTTAACT CAGGGAAGGTAGTTCTTAACTA 72879653
rs113971639
>hsa-mir-3686AGTGATCTGTAAGAGAAAGTAA TCTGTAAGAGAAAGTAAATGAA
GTAAGAGAAAGTAAATGAAAGA ACAGTGATCTGTAAGAGAAAGT 130496365
rs6997249
>hsa-mir-196a-2 AGTTTCATGTTGTTGGGATTGA AGGTAGTTTCATGTTGTTGGGA
TAGTTTCATGTTGTTGGGATTG GTAGTTTCATGTTGTTGGGATT
GGTAGTTTCATGTTGTTGGGAT 54385599 rs11614913
>hsa-mir-323bATACACGGTCGACCTCTTTTCG TACACGGTCGACCTCTTTTCGG
ACACGGTCGACCTCTTTTCGGT rs56103835

>hsa-mir-3144ATACCTGTTCAGTCTCTTTAAA TTCAGTCTCTTTAAAGTGTAGT
CCTGTTCAGTCTCTTTAAAGTG TGTTCAGTCTCTTTAAAGTGTA 120336384
rs67106263
>hsa-mir-3144ATACCTGTTCAGTCTCTTTAAA TTCAGTCTCTTTAAAGTGTAGT
CTGTTCAGTCTCTTTAAAGTGT TATACCTGTTCAGTCTCTTTAA 120336327
rs68035463 rs67106263
>hsa-mir-3144ATACCTGTTCGGTCTCTTTAAA CTGTTCGGTCTCTTTAAAGTGT
GTTCGGTCTCTTTAAAGTGTAG TGTTCGGTCTCTTTAAAGTGTA 120336327
rs68035463
>hsa-mir-548h-4 ATCGCGGTTTTTGTCATTACCT CGGTTTTTGTCATTACCTTAAT
AATCGCGGTTTTTGTCATTACC CGCGGTTTTTGTCATTACCTTA
AGTAATCGCGGTTTTTGTCATT 26906437 rs184537764
>hsa-mir-548h-4 ATCGCGGTTTTTGTCATTACCT CGGTTTTTGTCATTACCTTAAT
AATCGCGGTTTTTGTCATTACC CGCGGTTTTTGTCATTACCTTA
AGTAATCGCGGTTTTTGTCATT 26906402 rs73235381 26906437
rs184537764
>hsa-mir-548h-4 ATCGCGGTTTTTGTCATTACTT CGGTTTTTGTCATTACTTTAAT
AATCGCGGTTTTTGTCATTACT CGCGGTTTTTGTCATTACTTTA
AGTAATCGCGGTTTTTGTCATT 26906402 rs73235381

>hsa-mir-642aATTTGGAGAGGGAACCTCCCAA AGACACATTTGGAGAGGGAACC
ACACATTTGGAGAGGGAACCTC CACATTTGGAGAGGGAACCTCC 46178217
rs111664333
>hsa-mir-548ac CAAAAACCGGCAATTACTTTTG GGCAAAAACCGGCAATTACTTT
TTACTTTTGCACTAACCTAATA ACCGGCAATTACTTTTGCACTA 117102649
rs1414273
>hsa-mir-412 CACCTGGTCCACTGGCCGTCCG ACCTGGTCCACTGGCCGTCCGT
CTGGCCGTCCGTATCCGCTGCA TCACCTGGTCCACTGGCCGTCC 101531854
rs61992671
>hsa-mir-412 CACCTGGTTCACTGGCCGTCCG ACCTGGTTCACTGGCCGTCCGT
CTGGCCGTCCGTATCCGCTGCA TCACCTGGTTCACTGGCCGTCC 101531849
. rs61992671
>hsa-mir-509-2 CAGACAGTGGCAATCATGTATA GCAGACAGTGGCAATCATGTAT
CTGCAGACAGTGGCAATCATGT ACTGCAGACAGTGGCAATCATG
TGCAGACAGTGGCAATCATGTA 146340360 rs201600950
>hsa-mir-515-1 CAGAGTGCCTTCTTTTGGAGCA GAGTGCCTTCTTTTGGAGCATT
TGCCTTCTTTTGGAGCATTACT GTGCCTTCTTTTGGAGCATTAC 54182326
rs374576826
>hsa-mir-449bCAGCAGCCACAACTACCCTGCC CACAACTACCCTGCCACTTGCT
GCAGCCACAACTACCCTGCCAC AGCAGCCACAACTACCCTGCCA 54466544
rs10061133
>hsa-mir-4265CAGCTGTGGGCTCAACTCTGGG ATCTCTGCAGCTGTGGGCTCAA
GATCTCTGCAGCTGTGGGCTCA CTGCAGCTGTGGGCTCAACTCT 109757963
rs4676066
>hsa-mir-622 CAGTCTGCTGAGGTTGGAGCCG AGGTTGGAGCCGCTGAGATGAC
TCATCACACAGTCTGCTGAGGT GCTGAGGTTGGAGCCGCTGAGA
GAGGTTGGAGCCGCTGAGATGA 90883517 rs111371406
>hsa-mir-1227CATTTGACCCCGTGCCACCCTT ATTTGACCCCGTGCCACCCTTT
AGGCATTTGACCCCGTGCCACC GACCCCGTGCCACCCTTTTCCC 2234093
rs190788838
>hsa-mir-3151CCACCTGATCCCACACCCCACC CACCTGATCCCACACCCCACCT
CCCACCTGATCCCACACCCCAC TGATCCCACACCCCACCTGTCA 104166902
rs35605502
>hsa-mir-1343CCCCTCCTGGGGCCCGCACTCT CCCTCCTGGGGCCCGCACTCTC
CCTGGGGCCCGCACTCTCGCTC TGGGGCCCGCACTCTCGCTCTG 34963416
rs2986407
>hsa-mir-3166CCTACTGGCCTAAGAAAAATTT AGACAATGCCTACTGGCCTAAG
ATGCCTACTGGCCTAAGAAAAA CAATGCCTACTGGCCTAAGAAA 87909673
rs35854553
>hsa-mir-4268CCTCTCAGGATGTGATGTCACC CTCCTCTCAGGATGTGATGTCA
CTCCTCCTCTCAGGATGTGATG rs4674470

>hsa-mir-4254CCTGGAGATACTCCACCATCTC AGATACTCCACCATCTCCCCCA
GGAGATACTCCACCATCTCCCC rs12731294

>hsa-mir-1273h CCTGGGAGGTCAAGGCTGTAGT TGGGAGGTCAAGGCTGTAGTGT
ATTGCTTGAGCCTGGGAGGTCA GCCTGGGAGGTCAAGGCTGTAG

TTGAGCCTGGGAGGTCAAGGCT 24214486 .
>hsa-mir-182 CGGTGGTTCTAGACTTGCCAAC CCGGTGGTTCTAGACTTGCCAA
TCCGGTGGTTCTAGACTTGCCA GCCAACTATGGGGCGAGGACTC 129410227
rs76481776
>hsa-mir-412 CGTCCGTATCCGCTGCAG CCGTCCGTATCCGCTGCAG TCACCTGGTCCACTGGCCGTCC
ACCTGGTCCACTGGCCGTCCGT CACCTGGTCCACTGGCCGTCCG 101531854
rs61992671
>hsa-mir-412 CGTCCGTATCCGCTGCAG CCGTCCGTATCCGCTGCAG TCACCTGGTTCACTGGCCGTCC
ACCTGGTTCACTGGCCGTCCGT CACCTGGTTCACTGGCCGTCCG 101531849 .
101531854 rs61992671
>hsa-mir-509-2 CGTCTGTGGGTAGAGTACTGCA AATGATTGGTACGTCTGTGGGT
TGGTACGTCTGTGGGTAGAGTA TGGGTAGAGTACTGCATGACAC 146340360
rs201600950
>hsa-mir-3117CTCATATAGTGCCAGGTGTTTT GACTCATATAGTGCCAGGTGTT
TCATATAGTGCCAGGTGTTTTG ATAAGACTCATATAGTGCCAGG 67094171
rs12402181
>hsa-mir-3180-4 CTCCGGATGCCAGTCCCTCATC GGAGGGTGAAGCCTCCGGATGC
AGCGGAGGGTGAAGCCTCCGGA CTGGCCTGGTCGCGCTGTGGCT
GAGCGGAGGGTGAAGCCTCCGG 15248720 rs75000738
>hsa-mir-196a-2 CTCGGCAACAAGAAACTGTCTG CAAGAAACTGTCTGAGTTACAT
CAACAAGAAACTGTCTGAGTTA ACAAGAAACTGTCTGAGTTACA 54385599
rs11614913
>hsa-mir-486-2 CTCGGCGCAGCTCAGTACAGGA AGGGCCTCGGCGCAGCTCAGTA
TCGGCGCAGCTCAGTACAGGAT GGGCCTCGGCGCAGCTCAGTAC 41518007
.
>hsa-mir-4268CTCTCAGGATGTGATGTCACCT CCTCTCAGGATGTGATGTCACC
GCTCCTCCTCTCAGGATGTGAT TCCTCTCAGGATGTGATGTCAC
CTCCTCCTCTCAGGATGTGATG 220771223 rs4674470
>hsa-mir-3615CTCTCTCGGCTCCTCGCGGCTC GGCTCCTCGCGGCTCGCGGCGG
CGGCTCCTCGCGGCTCGCGGCG TCGGCTCCTCGCGGCTCGCGGC 72744798
rs745666
>hsa-mir-3151CTGATCCCACACCCCACCTGTC TGATCCCACACCCCACCTGTCA
GATCCCACACCCCACCTGTCAC GGGCATCCCACCTGATCCCACA
TCCCACCTGATCCCACACCCCA 104166902 rs35605502
>hsa-mir-1304CTGTAGCATCGAACCCCTGGGC GAACCCCTGGGCTCAAGTGATT
CTCACTGTAGCATCGAACCCCT CGAACCCCTGGGCTCAAGTGAT 93466866
rs2155248
>hsa-mir-3922CTGTGGGACTTCTGGCCTTGAC ACCTGTGGGACTTCTGGCCTTG
GGGACTTCTGGCCTTGACTTGA TGGGACTTCTGGCCTTGACTTG 104985443
rs61938575
>hsa-mir-412 CTTCACCTGGTTCACTAGCCGT ACCTGGTTCACTAGCCGTCCGT
TGTACTTCACCTGGTTCACTAG CTGGTTCACTAGCCGTCCGTAT
GTACTTCACCTGGTTCACTAGC 101531849 .
>hsa-mir-320eGAAAAGCTGGGTTGAGAAGGT AAAAGCTGGGTTGAGAAGGT
GGAAAAGCTGGGTTGAGAAGGT GGGAAAAGCTGGGTTGAGAAGG rs10423365

>hsa-mir-940 GAAGGCAGGGCCCC-GCTCCCC G CCC-GCTCCCCGGGCCTGACCC

rs35356504
>hsa-mir-548h-4 GACAAAAACCACAATTACTTTT AAAAACCACAATTACTTTTGCA TGACAAAAACCACAATTACTTT ACAAAAACCACAATTACTTTTG 26906402 rs73235381
>hsa-mir-548h-4 GACAAAAACCACAATTACTTTT AAAAACCACAATTACTTTTGCA TGACAAAAACCACAATTACTTT ACAAAAACCACAATTACTTTTG 26906402 rs73235381 26906437
>hsa-mir-548h-4 GACAAAAACCGCAATTACTTTT TGACAAAAACCGCAATTACTTT AAAAACCGCAATTACTTTTGCA AATGACAAAAACCGCAATTACT 26906437 rs184537764
>hsa-mir-4274GACCCAGCAGTCCCTCCCCCTG CCCAGCAGTCCCTCCCCCTGCA TGACCCAGCAGTCCCTCCCCCT TCAGGTGACCCAGCAGTCCCTC 7461769 rs12512664
>hsa-mir-1307GACTCGGCGTGGCGTCGGTCGT CGTGGCGTCGGTCGTGGTAGAT ATCGACTCGGCGTGGCGTCGGT GTGGCGTCGGTCGTGGTAGATA 105154089 rs7911488
>hsa-mir-629 GAGGTTCTCCCAACGTAAGCCC AGGTTCTCCCAACGTAAGCCCA TCTCCCAACGTAAGCCCAGCCC CAGGAGGTTCTCCCAACGTAAG 70371761 rs377691713
>hsa-mir-509-2 GATTGGTACGTCTGTGGGTAGA GTAGAGTACTGCATGACAC TAGAGTACTGCATGACAC CTGTGGGTAGAGTACTGCATGA TCTGTGGGTAGAGTACTGCATG 146340360 rs201600950
>hsa-mir-548ak GCAAAAGTAACTGCGGTTTTTG TGCAAAAGTAACTGCGGTTTTT CAAAAGTAACTGCGGTTTTTGA GTGCAAAAGTAACTGCGGTTTT rs7070684
>hsa-mir-222 GCAGCTACATCTGGCTACTGGG TACTGGGTCTCTGATGGCATCT GCTACTGGGTCTCTGATGGCAT CTGGCTACTGGGTCTCTGATGG 45606504 rs191727254
>hsa-mir-1343GCCCCTCCTGGGGCCCGCACTC CCCCTCCTGGGGCCCGCACTCT GGGGCCCGCACTCTCGCTCTGG CCCTCCTGGGGCCCGCACTCTC TGGGGCCCGCACTCTCGCTCTG 34963416 rs2986407
>hsa-mir-3180-4 GCGGAGGGTGAAGCCTCCGGAT CGCTGGCCTGGTCGCGCTGTGG TCGCTGGCCTGGTCGCGCTGTG AAGCCTCCGGATGCCAGTCCCT 15248720 rs75000738
>hsa-mir-3196GCGGGGCGGCAGGGGCCTCCCC GGCGGGGCGGCAGGGGCCTCCC GGGCGGGGCGGCAGGGGCCTCC TGGGGGCGGGGCGGCAGGGGCC GGGGCGGGGCGGCAGGGGCCTC 61870167 rs744591
>hsa-mir-2682GGACACCTCTTCAGCGCTGTCT CAGCGCTGTCTTCCCTGCCTCT TTCAGCGCTGTCTTCCCTGCCT CACCTCTTCAGCGCTGTCTTCC 98510847 rs74904371
>hsa-mir-3180-4 GGAGGGTGAAGCCTCCGGATGC GGTGAAGCCTCCGGATGCCAGT GCGGAGGGTGAAGCCTCCGGAT AGCGGAGGGTGAAGCCTCCGGA GCCTGGTCGCGCTGTGGCGAAG 15248798 rs183853838
>hsa-mir-3180-4 GGAGGGTGAAGCCTCCGGATGC GGTGAAGCCTCCGGATGCCAGT GCGGAGGGTGAAGCCTCCGGAT AGCGGAGGGTGAAGCCTCCGGA CTGGCCTGGTCGCGCTGTGGCT 15248720 rs75000738 15248798 rs183853838

>hsa-mir-548a-3 GGCAAAACTGGCAGTTACTTTT GCAAAACTGGCAGTTACTTTTG
AAAACTGGCAGTTACTTTTGCA AACTGGCAGTTACTTTTGCACC 105496622
.
>hsa-mir-1227GGCATTTGACCCCGTGCCACCC AGGCATTTGACCCCGTGCCACC
AGGCATTTGACCCCGTGCCACC TGACCCCGTGCCACCCTTTTCC
ATTTGACCCCGTGCCACCCTTT 2234093 rs190788838
>hsa-mir-608 GGCCAAGGTGGGCCAGGGGTGG AAGGTGGGCCAGGGGTGGTGTT
GGGGTGGTGTTGGGACAGCTGC TGGTGTTGGGACAGCTGCGTTT
GGTGGGCCAGGGGTGGTGTTGG 102734778 rs4919510
>hsa-mir-149 GGGAGGGAGGGACGGGGGCTGT GGAGGGACGGGGGCTGTGCTGG
AGGGACGGGGGCTGTGCTGGGG GAGGAGGGAGGGAGGGACGGGG 241395503
rs2292832
>hsa-mir-1197GTAGGACACATGGTCTACTTCT ACACATGGTCTACTTCTTCTCA
ACATGGTCTACTTCTTCTCAAT TAGGACACATGGTCTACTTCTT 101491923
rs141611518
>hsa-mir-658 GTAGGTCGGTTGGTCGGTCGGG G TAGGTCGGTTGGTCGGTCGGGA
38240368 rs141002682
>hsa-mir-658 GTAGGTCGGTTGGTCGGTCGGG TAGGTCGGTTGGTCGGTCGGGA
38240368 rs141002682 38240315 .
>hsa-mir-449bGTATCGTTAGCTGGCTGCTTGG AGTGTATCGTTAGCTGGCTGCT
CAGTGTATCGTTAGCTGGCTGC GTGTATCGTTAGCTGGCTGCTT
TGTATCGTTAGCTGGCTGCTTG 54466544 rs10061133
>hsa-mir-658 GTCCGTTGGTCGGTCGGGAACG G TCCGTTGGTCGGTCGGGAACGA

>hsa-mir-658 GTCCGTTGGTCGGTCGGGAACG .
rs141002682
>hsa-mir-573 GTGTAACTGATCAGGATCTACT TGTGTAACTGATCAGGATCTAC
GATGTGTAACTGATCAGGATCT TGATGTGTAACTGATCAGGATC
GTGATGTGTAACTGATCAGGAT 24521902 rs76014664
>hsa-mir-573 GTGTAACTGATCAGGATCTACT TGTGTAACTGATCAGGATCTAC
GATGTGTAACTGATCAGGATCT TGATGTGTAACTGATCAGGATC
GTGATGTGTAACTGATCAGGAT 24521904 rs78830737
>hsa-mir-573 GTGTAACTGATCAGGATCTACT TGTGTAACTGATCAGGATCTAC
GATGTGTAACTGATCAGGATCT TGATGTGTAACTGATCAGGATC
GTGATGTGTAACTGATCAGGAT 24521902 rs76014664 24521904
rs78830737
>hsa-mir-3117TAAAGGGCCAGACACTATACGA GGGCCAGACACTATACGAGTCA
GCCAGACACTATACGAGTCATA GGCCAGACACTATACGAGTCAT
CCCTAAAGGGCCAGACACTATA 67094171 rs12402181
>hsa-mir-3144TAAGGGGACCAAAGAGATATAT TTAAGGGGACCAAAGAGATATA
AAGGGGACCAAAGAGATATATA CTACACTTTAAGGGGACCAAAG
TTTAAGGGGACCAAAGAGATAT 120336384 rs67106263
>hsa-mir-3144TAAGGGGACCAAAGAGATATAT AAGGGGACCAAAGAGATATATA
TTAAGGGGACCAAAGAGATATA ATACACTTTAAGGGGACCAAAG
TTTAAGGGGACCAAAGAGATAT 120336327 rs68035463 120336384
rs67106263
>hsa-mir-3144TAAGGGGACCAAAGAGATATAT TTAAGGGGACCAAAGAGATATA

TTTAAGGGGACCAAAGAGATAT TACACTTTAAGGGGACCAAAGA
AAATACACTTTAAGGGGACCAA 120336327 rs68035463
>hsa-mir-3144TAAGGGGACCAAAGAGATATAT TTAAGGGGACCAAAGAGATATA
TTTAAGGGGACCAAAGAGATAT TACACTTTAAGGGGACCAAAGA
AACTACACTTTAAGGGGACCAA 120336384 rs67106263
>hsa-mir-3144TAAGGGGACCAAAGAGATATAT TTAAGGGGACCAAAGAGATATA
TTTAAGGGGACCAAAGAGATAT TACACTTTAAGGGGACCAAAGA
AAATACACTTTAAGGGGACCAA 120336327 rs68035463 120336384
rs67106263
>hsa-mir-3936TAAGGGGTGTATGGCAGATGCA TTCTGGTAAGGGGTGTATGGCA
CACCCGACAGATGCACTTGGCA TGTATGGCAGATGCACCCGACA 131701279
rs367805
>hsa-mir-629 TACGTTGGGAGAACTTTTATGG TTACGTTGGGAGAACTTTTATG
TTTACGTTGGGAGAACTTTTAT TGGGTTTACGTTGGGAGAACTT
GTTTACGTTGGGAGAACTTTTA 70371761 rs377691713
>hsa-mir-936 TAGAGGGAGGAATCGCAGAAAT TCAAGGCCACTGGGACAGTAGA
TGGGACAGTAGAGGGAGGAATC GGAGGAATCGCAGAAATCACTC
GGGAGGAATCGCAGAAATCACT 105807858 rs145823228
>hsa-mir-300 TATACAAGGGCAGACTCTCTCT TGATTATACAAGGGCAGACTCT
ATTATACAAGGGCAGACTCTCT rs12894467

>hsa-mir-580 TATTTGAGAATGATGAATCATT TGAATCATTAGGTTCCGGTCAG
ATGAATCATTAGGTTCCGGTCA TTTGAGAATGATGAATCATTAG
GAGAATGATGAATCATTAGGTT 36148057 rs115089112
>hsa-mir-3922TCAAGGCCAGAGGTCCCACAAC TCAAGTCAAGGCCAGAGGTCCC
GCCAGAGGTCCCACAACAGGGC GGCCAGAGGTCCCACAACAGGG
GTCAAGGCCAGAGGTCCCACAA 104985443 rs61938575
>hsa-mir-3141TCACCCGGTGAGGGCGGGTGGA CCGGTGAGGGCGGGTGGAGGAG
CGGTGAGGGCGGGTGGAGGAGG CACCCGGTGAGGGCGGGTGGAG
CCCGGTGAGGGCGGGTGGAGGA 153975576 rs936581
>hsa-mir-412 TCACCTGGTTCACTAGCCGTCC TCACCTGGTTCACTAGCCGTCC
ATGTACTTCACCTGGTTCACTA CTTCACCTGGTTCACTAGCCGT 101531849
.
>hsa-mir-564 TCAGCAGGCAACATGGCCGAGA TGTCAGCAGGCAACATGGCCGA
GTCAGCAGGCAACATGGCCGAG GTGTCAGCAGGCAACATGGCCG
GGTGTCAGCAGGCAACATGGCC 44903434 rs2292181
>hsa-mir-222 TCAGTAGCCAGTGTAGATCCTG TGGCTCAGTAGCCAGTGTAGAT
TTGGCTCAGTAGCCAGTGTAGA TCATTGGCTCAGTAGCCAGTGT
TACCCTCATTGGCTCAGTAGCC 45606504 rs191727254
>hsa-mir-515-1 TCCAAAAGAAAGCACTTTCTGT TCTCCAAAAGAAAGCACTTTCT
TTCTCCAAAAGAAAGCACTTTC TCATTCTCCAAAAGAAAGCACT
TGCAGTCATTCTCCAAAAGAAA 54182326 rs374576826
>hsa-mir-532 TCCCACACCCAAGGCTTGCAGA CTCCCACACCCAAGGCTTGCAG
CCTCCCACACCCAAGGCTTGCA CACCCAAGGCTTGCAGAAGAGC 49767832
rs456615
>hsa-mir-532 TCCCACACCCAAGGCTTGCAGA CTCCCACACCCAAGGCTTGCAG
CCTCCCACACCCAAGGCTTGCA CACCCAAGGCTTGCAGAAGAGC 49767835

rs456617
>hsa-mir-532 TCCCACACCCAAGGCTTGCAGA CTCCCACACCCAAGGCTTGCAG CCTCCCACACCCAAGGCTTGCA CACCCAAGGCTTGCAGAAGAGC 49767832 rs456615 49767835 rs456617
>hsa-mir-663aTCCCAGGCGGGGCGCCGCGGGA TCCGGCGTCCCAGGCGGGGCGC TTCCGGCGTCCCAGGCGGGGCG GCGCCGCGGGACCTCCCTCGTG GGCGCCGCGGGACCTCCCTCGT 26188880 .
>hsa-mir-596 TCCGAAGCCTGCCCGGCCCCTC GCCTGCCCGGCCCCTCGGGAAC TCTCCGAAGCCTGCCCGGCCCC CTGCCCGGCCCCTCGGGAACCT CCTGCCCGGCCCCTCGGGAACC 1765425 rs61388742
>hsa-mir-149 TCCGTGTCTTCACTCCCGTGCT TGGCTCCGTGTCTTCACTCCCG TCTGGCTCCGTGTCTTCACTCC CCGTGTCTTCACTCCCGTGCTT AGCTCTGGCTCCGTGTCTTCAC 241395503 rs2292832
>hsa-mir-618 TCCTTCTGAGTGTAATTACGTA TGTCCTTCTGAGTGTAATTACG TTGTCCTTCTGAGTGTAATTAC TACTTGTCCTTCTGAGTGTAAT GTCCTTCTGAGTGTAATTACGT 81329527 rs145551269
>hsa-mir-618 TCCTTCTGAGTGTAATTACGTA TGTCCTTCTGAGTGTAATTACG TTGTCCTTCTGAGTGTAATTAC TACTTGTCCTTCTGAGTGTAAT GTCCTTCTGAGTGTAATTACGT 81329536 rs2682818
>hsa-mir-618 TCCTTCTGAGTGTAATTACGTA TGTCCTTCTGAGTGTAATTACG TTGTCCTTCTGAGTGTAATTAC TACTTGTCCTTCTGAGTGTAAT GTCCTTCTGAGTGTAATTACGT 81329527 rs145551269 81329536 rs2682818
>hsa-mir-492 TCGAGGACCTGCGGGACAAGAT TACAGGACCATCGAGGACCTGC TACTACAGGACCATCGAGGACC TCCAGCCACTACTACAGGACCA GACCTGCGGGACAAGATTCTTG 95228179 rs200816308
>hsa-mir-3183TCGGAGTCGCTCGGAGCAGCCA TCTCGGAGTCGCTCGGAGCAGC TCTCTCGGAGTCGCTCGGAGCA TCTGCCCTGCCTCTCTCGGAGT TGCCCTGCCTCTCTCGGAGTCG 925764 rs2663345
>hsa-mir-3671TCTATCAAATAAGGACTAGTCT AAATAAGGACTAGTCTGCAGTG TTTATTTCTATCAAATAAGGAC CAAATAAGGACTAGTCTGCAGT 65523519 rs521188
>hsa-mir-4326TCTGCTGTTCCTCTGTCTCCCA TGGTCTGCTGTTCCTCTGTCTC CTGGTCTGCTGTTCCTCTGTCT GCTGTTCCTCTGTCTCCCAGAC TGCTGTTCCTCTGTCTCCCAGA 61918164 rs6062431
>hsa-mir-4305TCTGGGTTCTTAGAGGCCTAAT TTCTGGGTTCTTAGAGGCCTAA GTTCTGGGTTCTTAGAGGCCTA TCCAGTTCTGGGTTCTTAGAGG CAGTTCTGGGTTCTTAGAGGCC 40238175 rs67976778
>hsa-mir-3118-1 TGAAAATTCTTCTAGTGTG ATGAAAATTCTTCTAGTGTG TGCATTATGAAAATTCTTCTAG TTATGAAAATTCTTCTAGTGTG ATTATGAAAATTCTTCTAGTGT 142667330 rs76132421
>hsa-mir-635 TGAAACAATGTCCATTAGGCTT GAAACAATGTCCATTAGGCTTT ACAATGTCCATTAGGCTTTGTT AACAATGTCCATTAGGCTTTGT CTGAAACAATGTCCATTAGGCT 66420592 rs77279010
>hsa-mir-1255a TGAGCAAAGAAAGTAGATTTTT GCAAAGAAAGTAGATTTTTTAG TCAAGGATGAGCAAAGAAAGTA GAGCAAAGAAAGTAGATTTTTT TCTCAAGGATGAGCAAAGAAAG 102251501 rs28664200

>hsa-mir-3151TGATGGGTGGGGCAATGGGATC TGGGTGGGGCAATGGGATCAGG TGGGGCAATGGGATCAGGTGCC GGGGTGATGGGTGGGGCAATGG GGGTGATGGGTGGGGCAATGGG 104166902 rs35605502
>hsa-mir-548a-3 TGCAAAAGTAATTGCGAGTTTT TCGGTGCAAAAGTAATTGCGAG TAGGTCGGTGCAAAAGTAATTG TTAGGTCGGTGCAAAAGTAATT TATTAGGTCGGTGCAAAAGTAA 105496622 .
>hsa-mir-3156-2 TGCAGAAGAAAGATCTGGAAGT GAAAGATCTGGAAGTGGGAGAC GAAGAAAGATCTGGAAGTGGGA GCAGAAGAAAGATCTGGAAGTG CAGAAGAAAGATCTGGAAGTGG 14830215 rs113478966
>hsa-mir-3156-3 TGCAGAAGAAAGATCTGGAAGT GCAGAAGAAAGATCTGGAAGTG GAAGAAAGATCTGGAAGTGGGA GAAAGATCTGGAAGTGGGAGAC AGAAGAAAGATCTGGAAGTGGG 14778721 rs2747232
>hsa-mir-1200TGCTACTTCTCCTGAGCCATTC TGAGCCATTCTGAGCCTCAGTC TACTTCTCCTGAGCCATTCTGA TCCTGAGCCATTCTGAGCCTCA TTCTCCTGAGCCATTCTGAGCC 36959006 .
>hsa-mir-1273h TGCTGCAGACTCGACCTCCCAG TGCAGACTCGACCTCCCAGGCT CTGCAGACTCGACCTCCCAGGC AGACTCGACCTCCCAGGCTTAA 24214486 .
>hsa-mir-1254-2 TGGAAGCTGGAGCCTGCAGTGA TGAGCCTGGAAGCTGGAGCCTG GAAGCTGGAGCCTGCAGTGAGC GGAAGCTGGAGCCTGCAGTGAG GCCTGGAAGCTGGAGCCTGCAG 23682383 rs200793185
>hsa-mir-3156-2 TGGCCCCCACTTCCAGATCTTT CCCCCACTTCCAGATCTTTCTC ACTTCCAGATCTTTCTCTCTGT CCCCACTTCCAGATCTTTCTCT 14830215 rs113478966
>hsa-mir-637 TGGCTAAGGTGTTGGCTCGGGC TGGCTAAGGTGTTGGCTCGGGC

>hsa-mir-608 TGGGACAGCTGCGTTTAAAAAG TTGGGACAGCTGCGTTTAAAAA GGACAGCTGCGTTTAAAAAGGC TGTTGGGACAGCTGCGTTTAAA GGGACAGCTGCGTTTAAAAAGG 102734778 rs4919510
>hsa-mir-1273h TGGGAGGTCAAGGCTGTAGTGT TGAGCCTGGGAGGTCAAGGCTG TTGAGCCTGGGAGGTCAAGGCT TGCTTGAGCCTGGGAGGTCAAG TTGCTTGAGCCTGGGAGGTCAA 24214486 .
>hsa-mir-345 TGGGCCCTGAACGAGGGGTCTG GTGGGCCCTGAACGAGGGGTCT GCCCTGAACGAGGGGTCTGGAG GGCCCTGAACGAGGGGTCTGGA 100774203 rs72631832
>hsa-mir-2682TGGGGCAGGCAGTGACTGTTCA TTGGGGCAGGCAGTGACTGTTC GGCAGTGACTGTTCAGACGTCC TGAAAGAGGTTGGGGCAGGCAG GCAGGCAGTGACTGTTCAGACG 98510847 rs74904371
>hsa-mir-412 TGGGGTACGGGGATGGATGGTC GGATGGATGGTCGACCAGTTGG GATGGATGGTCGACCAGTTGGA TCGACCAGTTGGAAAGTAATTG ACGGGGATGGATGGTCGACCAG 101531854 rs61992671
>hsa-mir-412 TGGGGTACGGGGATGGATGGTC GGATGGATGGTCGACCAGTTGG GGGTACGGGGATGGATGGTCGA GATGGATGGTCGACCAGTTGGA TCGACCAGTTGGAAAGTAATTG 101531849 . 101531854 rs61992671

>hsa-mir-412 TGGGGTACGGGGATGGATGGTC TCGACCAGTTGGAAAGTAATTG TGGTCGACCAGTTGGAAAGTAA TACGGGGATGGATGGTCGACCA

TGGATGGTCGACCAGTTGGAAA 101531854 rs61992671
>hsa-mir-412 TGGGGTACGGGGATGGATGGTC TCGACCAGTTGGAAAGTAATTG
TGGTCGACCAGTTGGAAAGTAA TACGGGGATGGATGGTCGACCA
TGGATGGTCGACCAGTTGGAAA 101531849 . 101531854 rs61992671

>hsa-mir-1227TGGTGGGCACTGCTGGGGTGGG TGGGGCCAGGCGGTGGTGGGCA
AGGCGGTGGTGGGCACTGCTGG GGTGGGCACTGCTGGGGTGGGC
GTGGTGGGCACTGCTGGGGTGG 2234093 rs190788838
>hsa-mir-323bTGTCCGTGGTGAGTTCGCATTA TTGTCCGTGGTGAGTTCGCATT
TACTCGGAGGGAGGTTGTCCGT TCGGAGGGAGGTTGTCCGTGGT
AGGTTGTCCGTGGTGAGTTCGC 101522556 rs56103835
>hsa-mir-2053TGTTAATTAAACCTCTATTTAC ACTTTAAGTGTTAATTAAACCT
TTTAAGTGTTAATTAAACCTCT TTAAGTGTTAATTAAACCTCTA 113655752
rs10505168
>hsa-mir-642bTTCCCTCTCCAAATGTGTCTTG TTGGGAGGTTCCCTCTCCAAAT
TGGGAGGTTCCCTCTCCAAATG GAGTTGGGAGGTTCCCTCTCCA
GTTGGGAGGTTCCCTCTCCAAA 46178217 rs111664333
>hsa-mir-4277TTCTGAGCACAGTACACTGGGC TCGAGGCAGTTCTGAGCACAGT
TGGGTCGAGGCAGTTCTGAGCA GTTCTGAGCACAGTACACTGGG
GCAGTTCTGAGCACAGTACACT 1708902 rs115200817
>hsa-mir-4277TTCTGAGCACAGTACACTGGGC TCGAGGCAGTTCTGAGCACAGT
TGGGTCGAGGCAGTTCTGAGCA TTGGGTCGAGGCAGTTCTGAGC
GTTCTGAGCACAGTACACTGGG 1708983 rs12523324
>hsa-mir-4277TTCTGAGCACAGTACACTGGGC TCGAGGCAGTTCTGAGCACAGT
TGGGTCGAGGCAGTTCTGAGCA TTGGGTCGAGGCAGTTCTGAGC
GTTCTGAGCACAGTACACTGGG 1708902 rs115200817 1708983
rs12523324
>hsa-mir-585 TTGGGCGTATCTGTATGCTAGG TATCTGTATGCTAGGGCTGCCG
TGGGCGTATCTGTATGCTAGGG GCGTATCTGTATGCTAGGGCTG 168690612
rs62376934
>hsa-mir-553 TTTAAAACGGTGAGATTTTGTT TTTTAAAACGGTGAGATTTTGT
ATTTTAAAACGGTGAGATTTTG TATTTTAAAACGGTGAGATTTT
TTATTTTAAAACGGTGAGATTT 100746848 .
>hsa-mir-553 TTTAAGACGGTGAGATTTTGTT TTATTTTAAGACGGTGAGATTT
TATTTTAAGACGGTGAGATTTT TTTTATTTTAAGACGGTGAGAT
ATTTTAAGACGGTGAGATTTTG 100746814 rs190622705
>hsa-mir-553 TTTAAGACGGTGAGATTTTGTT TTTTAAGACGGTGAGATTTTGT
ATTTTAAGACGGTGAGATTTTG TATTTTAAGACGGTGAGATTTT
TTATTTTAAGACGGTGAGATTT 100746814 rs190622705
>hsa-mir-553 TTTAAGACGGTGAGATTTTGTT TTTTAAGACGGTGAGATTTTGT
ATTTTAAGACGGTGAGATTTTG TATTTTAAGACGGTGAGATTTT
TTATTTTAAGACGGTGAGATTT 100746814 rs190622705 100746848 .

>hsa-mir-580 TTTGAGAATGATGAATCATTAG GATGAATCATTAGGTTCCGGTC
AATGATGAATCATTAGGTTCCG AGAATGATGAATCATTAGGTTC 36148057
rs115089112
>hsa-mir-553 TTTTAAAACGGTGAGATTTTGT TATTTTAAAACGGTGAGATTTT

TTTTATTTTAAAACGGTGAGAT TTTAAAACGGTGAGATTTTGTT
ATTTTATTTTAAAACGGTGAGA 100746848 .
>hsa-mir-553 TTTTAAGACGGTGAGATTTTGT TATTTTAAGACGGTGAGATTTT
TTTAAGACGGTGAGATTTTGTT TTTTATTTTAAGACGGTGAGAT
ATTTTATTTTAAGACGGTGAGA 100746814 rs190622705 100746848 .
