# Supplementary material for: Role of SNPs in the Biogenesis of Mature miRNAs
Source: Biomed Res Int. 2021 Jun 17;2021:2403418. doi: 10.1155/2021/2403418 (PMC8233088; doi:10.1155/2021/2403418)
Supplement: Supplementary Materials — Additional file 1 Table S1: the database miRSNPBase (xls). Additional file 2 Table S2: the list of pre-miRNAs in miRSNPBase which is classified based on mature miRNA in the 5′ arm or 3′ arm (xls). Additional file 3 Table S3: all the iso-pre-miRNAs, nor-pre-miRNAs, nor-SNPs, and iso-SNPs associated with four splicing sites (xls). Additional file 4 Table S4: the pre-miRNAs and SNPs associated with the normal and isomiRs (xls). Additional file 5 Table S5: the pre-miRNAs, iso-SNPs, and isomiRs of HG00097 (xls). Additional file 6 Table S6: the isomiRs and iso-SNP of 18 GBR populations (xls). Additional file 7 Table S7: the verified isomiRs of 18 GBR (xls). Additional file 8 Table S8: the iso-pre-miRNA candidates and the verified iso-pre-miRNAs of 18 GBR samples (xls). [file 2403418.f1.zip › 2403418.f1/Supp Tab S6.pdf]

>hsa-mir-1273h
>hsa-mir-1273d

>hsa-mir-564
>hsa-mir-320e
>hsa-mir-663a
>hsa-mir-635
>hsa-mir-1254-2
>hsa-mir-499b
>hsa-mir-1255b-2
>hsa-mir-423
>hsa-mir-3615
>hsa-mir-940
>hsa-mir-1303
>hsa-mir-1307
>hsa-mir-149
>hsa-mir-222
>hsa-mir-486-2
>hsa-mir-548a-3
>hsa-mir-629
>hsa-mir-637
>hsa-mir-658
>hsa-mir-1304
>hsa-mir-24-2
>hsa-mir-532
>hsa-mir-642a
>hsa-mir-1294
>hsa-mir-580
>hsa-mir-604
>hsa-mir-30d
>hsa-mir-3166
>hsa-mir-520h
>hsa-mir-577
>hsa-mir-196a-2
>hsa-mir-3199-2
>hsa-mir-449c
>hsa-mir-500b
>hsa-mir-573
>hsa-mir-650
>hsa-mir-1227
>hsa-mir-3176
>hsa-mir-3188
>hsa-mir-3679
>hsa-mir-3909
>hsa-mir-3922
>hsa-mir-563
>hsa-mir-744

>hsa-mir-877
>hsa-mir-1200
>hsa-mir-27a
>hsa-mir-548ac
>hsa-mir-548at
>hsa-mir-199b
>hsa-mir-3620
>hsa-mir-576
>hsa-mir-612
>hsa-mir-943
>hsa-mir-944
>hsa-mir-1908
>hsa-mir-1255b-1
>hsa-mir-1244-3
>hsa-mir-590
>hsa-mir-1972-2
>hsa-mir-1268a
>hsa-mir-3144
>hsa-mir-3652
>hsa-mir-548ap
>hsa-mir-138-1
>hsa-mir-548j
>hsa-mir-1229
>hsa-mir-1343
>hsa-mir-151b
