# Supplementary material for: Role of SNPs in the Biogenesis of Mature miRNAs
Source: Biomed Res Int. 2021 Jun 17;2021:2403418. doi: 10.1155/2021/2403418 (PMC8233088; doi:10.1155/2021/2403418)
Supplement: Supplementary Materials — Additional file 1 Table S1: the database miRSNPBase (xls). Additional file 2 Table S2: the list of pre-miRNAs in miRSNPBase which is classified based on mature miRNA in the 5′ arm or 3′ arm (xls). Additional file 3 Table S3: all the iso-pre-miRNAs, nor-pre-miRNAs, nor-SNPs, and iso-SNPs associated with four splicing sites (xls). Additional file 4 Table S4: the pre-miRNAs and SNPs associated with the normal and isomiRs (xls). Additional file 5 Table S5: the pre-miRNAs, iso-SNPs, and isomiRs of HG00097 (xls). Additional file 6 Table S6: the isomiRs and iso-SNP of 18 GBR populations (xls). Additional file 7 Table S7: the verified isomiRs of 18 GBR (xls). Additional file 8 Table S8: the iso-pre-miRNA candidates and the verified iso-pre-miRNAs of 18 GBR samples (xls). [file 2403418.f1.zip › 2403418.f1/Supp Tab S7.pdf]

HG00097 pre-miRNA isomiR isomiR isomiR isomiR isomiR SNP position snp
HG00097 >hsa-mir-548aj-2 AAAAACTGCAATTACTTTTACA TGGTAAAAACTGCAATTACTTT AACTGCAATTACTTTTACACCA AATTACTTTTACACCAACCTAA 37883200 rs73463468

HG00097 >hsa-mir-548ad AAAACGACAATGACTTTTGCAC ACTTTTGCACCAATCTAATAC CTTTTGCACCAATCTAATAC GCAAAAACGACAATGACTTTTG CAAAAACGACAATGACTTTTGC 35696519 rs62143301
HG00097 >hsa-mir-202 AAAGAGGTATAGGGCATGGGAA AAGAGGTATAGGGCATGGGAAA GGGAAAACGGGGCGGTCGGGTC TAAAGAGGTATAGGGCATGGGA 135061112 rs12355840
HG00097 >hsa-mir-548ap AACAAAAACCACAATTACTTTT CAAAAACCACAATTACTTTTTA CAATTACTTTTTACTGACCTAA rs4414449
HG00097 >hsa-mir-548ap AACAAAAACCACAATTACTTTT AACAAAAACCACAATTACTTTT CAAAAACCACAATTACTTTTTA TTACTTTTTACTGACCTAAAGA 86368959 rs4577031

HG00097 >hsa-mir-548ap AACAAAAACCACAATTACTTTT CAAAAACCACAATTACTTTTTA CAATTACTTTTTACTGACCTAA 86368959 rs4577031 rs4414449
HG00097 >hsa-mir-548ad AACGACAATGACTTTTGCACCA GGCAAAAACGACAATGACTTTT TGGCAAAAACGACAATGACTTT AAAAACGACAATGACTTTTGCA 35696519 rs62143301

HG00097 >hsa-mir-548al AACGGCAGTGACTTTTGTACCA TGGCAAAAACGGCAGTGACTTT TAAAAGTAATGGCAAAAACGGC AAAAGTAATGGCAAAAACGGCA 74110353 rs515924

HG00097 >hsa-mir-423 AAGCTCGGTCTGAGGCCCCTCA AGGCCCCTCAGTCTTGCTTCCT TCTGAGGCCCCTCAGTCTTGCT GTCTGAGGCCCCTCAGTCTTGC 28444183 rs6505162
HG00097 >hsa-mir-3144AAGGGGACCAAAGAGATATATA TAAGGGGACCAAAGAGATATAT TTAAGGGGACCAAAGAGATATA TTTAAGGGGACCAAAGAGATAT ATACACTTTAAGGGGACCAAAG 120336327 rs68035463

HG00097 >hsa-mir-3671AATAAGGACTAGTCTGCAGTGA TTTATTTCTATCAAATAAGGAC TTTTATTTCTATCAAATAAGGA GGACTAGTCTGCAGTGATAT AAATAAGGACTAGTCTGCAGTG 65523519 rs521188

HG00097 >hsa-mir-622 ACACAGTCTGCTGAGGTTGGAG CTGCTGAGGTTGGAGCCGCTGA ACACAGTCTGCTGAGGTTGGAG rs111371406

HG00097 >hsa-mir-24-2ACACTGGCTCAGTTCAGCAGGA CACTGGCTCAGTTCAGCAGGAA TGGCTCAGTTCAGCAGGAACAG CTGGCTCAGTTCAGCAGGAACA 13947170

.
HG00097 >hsa-mir-597 ACAGTGGTTCTCTTGTGGCTTA GGCTTAAGCGTAATGTAGAGTA AATGTACAGTGGTTCTCTTGTG TGTACAGTGGTTCTCTTGTGGC 9599255 rs146125159
HG00097 >hsa-mir-3141ACCCGGTGAGGGCGGGTGGAGG CGGTGAGGGCGGGTGGAGGAGG CCGGTGAGGGCGGGTGGAGGAG CCCGGTGAGGGCGGGTGGAGGA CACCCGGTGAGGGCGGGTGGAG 153975576 rs936581

HG00097 >hsa-mir-1307ACCGGACCTCGACCGGCTCGTC CGGACCTCGACCGGCTCGTCTG CCGGACCTCGACCGGCTCGTCT ATCTCGACCGGACCTCGACCGG AATCTCGACCGGACCTCGACCG 105154089 rs7911488

HG00097 >hsa-mir-486-2 ACTGAGCTGCCCCGAGCTGGGC CTGAGCTGCCCCGAGCTGGGCA CTGTACTGAGCTGCCCCGAGCT CCTGTACTGAGCTGCCCCGAGC GTACTGAGCTGCCCCGAGCTGG 41518007 .

HG00097 >hsa-mir-3118-1 ACTGCATTATGAAAATTCTTCT ATTATGAAAATTCTTCTAGTGT GCATTATGAAAATTCTTCTAGT CTGCATTATGAAAATTCTTCTA 142667330 rs76132421

HG00097 >hsa-mir-3125AGAATGGATAGAGGAAGCTGTG GAGGAAGCTGTGGAGAGAACTC AGAGGAAGCTGTGGAGAGAACT GCTGTGGAGAGAACTCACGGTG GGAAGCTGTGGAGAGAACTCAC 12877501 rs78852835

HG00097 >hsa-mir-642bAGATACATTTGGAGAGGGACCC TTGGAGAGGGACCCTCCCAACT TTTGGAGAGGGACCCTCCCAAC ATACATTTGGAGAGGGACCCTC 46178217 rs111664333
HG00097 >hsa-mir-378hAGATGGGATGAGCCCTGGCTCT TCAGATGGGATGAGCCCTGGCT TGTCAGATGGGATGAGCCCTGG GATGGGATGAGCCCTGGCTCTG CAGATGGGATGAGCCCTGGCTC 154209024 rs702742

HG00097 >hsa-mir-646 AGCAGCTGCCTCTGAGGCCTCA CTGAGGCCTCAGGCTCAGTGGC CTCTGAGGCCTCAGGCTCAGTG GCCTCTGAGGCCTCAGGCTCAG TCTGAGGCCTCAGGCTCAGTGG 58883534 rs6513496

HG00097 >hsa-mir-1269b AGCCATGCTACGGGCTTCTCTG ACTGAGCCATGCTACGGGCTTC AGGTTTCTGGACTGAGCCATGC TGAGGTTTCTGGACTGAGCCAT TTTCTGGACTGAGCCATGCTAC 12820632 rs12451747
HG00097 >hsa-mir-3135b AGCGAGTGCAGTGGTGCAGTCA AGGCTGGAGCGAGTGCAGTGGT CTGGAGCGAGTGCAGTGGTGCA CAGGCTGGAGCGAGTGCAGTGG CCAGGCTGGAGCGAGTGCAGTG 32717702 rs4285314
HG00097 >hsa-mir-3180-4 AGCGGAGGGTGAAGCCTCCGGA CGGAGGGTGAAGCCTCCGGATG GAGCGGAGGGTGAAGCCTCCGG GCGGAGGGTGAAGCCTCCGGAT 15248798 rs183853838

HG00097 >hsa-mir-3180-4 AGCGGAGGGTGAAGCCTCCGGA
CGGAGGGTGAAGCCTCCGGATG GAGCGGAGGGTGAAGCCTCCGG
GCGGAGGGTGAAGCCTCCGGAT 15248720 rs75000738 15248798
rs183853838
HG00097 >hsa-mir-3125AGCTGTGGAGAGAACTCACGGT AAGCTGTGGAGAGAACTCACGG
TAGAGGAAGCTGTGGAGAGAAC TGGATAGAGGAAGCTGTGGAGA
AGGAAGCTGTGGAGAGAACTCA 12877501 rs78852835

HG00097 >hsa-mir-30d AGCTTTCAGTCAGATGTTTGCT GGCTAAGCTTTCAGTCAGATGT
GCTAAGCTTTCAGTCAGATGTT TTCAGTCAGATGTTTGCTGCTA 135817150
.
HG00097 >hsa-mir-519a-2 AGGAAAGTGCATCCTTTTAGAG
AGTGCATCCTTTTAGAGGGTTA GGAAAGTGCATCCTTTTAGAGG
GAAAGGAAAGTGCATCCTTTTA 54265670 .

HG00097 >hsa-mir-646 AGGAAGCAGCTGCCTCTGAGGC GCTGCCTCTGAGGCCTCAGGCT
CTGAGGCCTCAGGCTCAGTGGC TCTGAGGCCTCAGGCTCAGTGG 58883534
rs6513496
HG00097 >hsa-mir-149 AGGGAGGGAGGGACGGGGGCTG GGGCTGTGCTGGGGCAGCCGGA
GAGGGAGGGAGGGACGGGGGCT GGGACGGGGGCTGTGCTGGGGC
GGGAGGGAGGGACGGGGGCTGT 241395503 rs2292832

HG00097 >hsa-mir-658 AGGTCGGTTGGTCGGTCGGGAA GTCGGTTGGTCGGTCGGGAACG
TAGGTCGGTTGGTCGGTCGGGA

HG00097 >hsa-mir-548ap AGTAATTGCAGTCTTTGTCATT
AAGTAATTGCAGTCTTTGTCAT AAAGTAATTGCAGTCTTTGTCA
AAAAGTAATTGCAGTCTTTGTC CAAAAGTAATTGCAGTCTTTGT 86368898
rs4414449
HG00097 >hsa-mir-548ap AGTAATTGCAGTCTTTGTCATT
AAGTAATTGCAGTCTTTGTCAT AAAGTAATTGCAGTCTTTGTCA
AAAAGTAATTGCAGTCTTTGTC CAAAAGTAATTGCAGTCTTTGT 86368898
rs4414449 86368959 rs4577031
HG00097 >hsa-mir-548aj-2 AGTAATTGCAGTTTTTGCCATT
AAGTAATTGCAGTTTTTGCCAT AAAGTAATTGCAGTTTTTGCCA
TGCAAAAGTAATTGCAGTTTTT AAAAGTAATTGCAGTTTTTGCC 37883200
rs73463468
HG00097 >hsa-mir-548ap AGTAATTGCGGTCTTTGTCATT
AAGTAATTGCGGTCTTTGTCAT AAAGTAATTGCGGTCTTTGTCA
AAAAGTAATTGCGGTCTTTGTC CAAAAGTAATTGCGGTCTTTGT 86368959
rs4577031
HG00097 >hsa-mir-630 AGTATTCTGTACCAGGGAAGGT ACCTAGTATTCTGTACCAGGGA
CCAGGGAAGGTAGTTCTTAACT CAGGGAAGGTAGTTCTTAACTA 72879653
rs113971639
HG00097 >hsa-mir-3686AGTGATCTGTAAGAGAAAGTAA TCTGTAAGAGAAAGTAAATGAA
GTAAGAGAAAGTAAATGAAAGA ACAGTGATCTGTAAGAGAAAGT 130496365
rs6997249

HG00097 >hsa-mir-196a-2 AGTTTCATGTTGTTGGGATTGA
AGGTAGTTTCATGTTGTTGGGA TAGTTTCATGTTGTTGGGATTG
GTAGTTTCATGTTGTTGGGATT GGTAGTTTCATGTTGTTGGGAT 54385599
rs11614913
HG00097 >hsa-mir-323bATACACGGTCGACCTCTTTTCG TACACGGTCGACCTCTTTTCGG
ACACGGTCGACCTCTTTTCGGT rs56103835

HG00097 >hsa-mir-3144ATACCTGTTCAGTCTCTTTAAA TTCAGTCTCTTTAAAGTGTAGT
CCTGTTCAGTCTCTTTAAAGTG TGTTCAGTCTCTTTAAAGTGTA 120336384
rs67106263
HG00097 >hsa-mir-3144ATACCTGTTCAGTCTCTTTAAA TTCAGTCTCTTTAAAGTGTAGT
CTGTTCAGTCTCTTTAAAGTGT TATACCTGTTCAGTCTCTTTAA 120336327
rs68035463 rs67106263
HG00097 >hsa-mir-3144ATACCTGTTCGGTCTCTTTAAA CTGTTCGGTCTCTTTAAAGTGT
GTTCGGTCTCTTTAAAGTGTAG TGTTCGGTCTCTTTAAAGTGTA 120336327
rs68035463
HG00097 >hsa-mir-548h-4 ATCGCGGTTTTTGTCATTACCT
CGGTTTTTGTCATTACCTTAAT AATCGCGGTTTTTGTCATTACC
CGCGGTTTTTGTCATTACCTTA AGTAATCGCGGTTTTTGTCATT 26906437
rs184537764
HG00097 >hsa-mir-548h-4 ATCGCGGTTTTTGTCATTACCT
CGGTTTTTGTCATTACCTTAAT AATCGCGGTTTTTGTCATTACC
CGCGGTTTTTGTCATTACCTTA AGTAATCGCGGTTTTTGTCATT 26906402
rs73235381 26906437 rs184537764
HG00097 >hsa-mir-548h-4 ATCGCGGTTTTTGTCATTACTT
CGGTTTTTGTCATTACTTTAAT AATCGCGGTTTTTGTCATTACT
CGCGGTTTTTGTCATTACTTTA AGTAATCGCGGTTTTTGTCATT 26906402
rs73235381
HG00097 >hsa-mir-642aATTTGGAGAGGGAACCTCCCAA AGACACATTTGGAGAGGGAACC
ACACATTTGGAGAGGGAACCTC CACATTTGGAGAGGGAACCTCC 46178217
rs111664333
HG00097 >hsa-mir-548ac CAAAAACCGGCAATTACTTTTG
GGCAAAAACCGGCAATTACTTT TTACTTTTGCACTAACCTAATA
ACCGGCAATTACTTTTGCACTA 117102649 rs1414273

HG00097 >hsa-mir-412 CACCTGGTCCACTGGCCGTCCG ACCTGGTCCACTGGCCGTCCGT
CTGGCCGTCCGTATCCGCTGCA TCACCTGGTCCACTGGCCGTCC 101531854
rs61992671
HG00097 >hsa-mir-412 CACCTGGTTCACTGGCCGTCCG ACCTGGTTCACTGGCCGTCCGT
CTGGCCGTCCGTATCCGCTGCA TCACCTGGTTCACTGGCCGTCC 101531849
. rs61992671
HG00097 >hsa-mir-509-2 CAGACAGTGGCAATCATGTATA
GCAGACAGTGGCAATCATGTAT CTGCAGACAGTGGCAATCATGT
ACTGCAGACAGTGGCAATCATG TGCAGACAGTGGCAATCATGTA 146340360
rs201600950
HG00097 >hsa-mir-515-1 CAGAGTGCCTTCTTTTGGAGCA
GAGTGCCTTCTTTTGGAGCATT TGCCTTCTTTTGGAGCATTACT

GTGCCTTCTTTTGGAGCATTAC 54182326 rs374576826

HG00097 >hsa-mir-449bCAGCAGCCACAACTACCCTGCC CACAACTACCCTGCCACTTGCT GCAGCCACAACTACCCTGCCAC AGCAGCCACAACTACCCTGCCA 54466544 rs10061133

HG00097 >hsa-mir-4265CAGCTGTGGGCTCAACTCTGGG ATCTCTGCAGCTGTGGGCTCAA GATCTCTGCAGCTGTGGGCTCA CTGCAGCTGTGGGCTCAACTCT 109757963 rs4676066

HG00097 >hsa-mir-622 CAGTCTGCTGAGGTTGGAGCCG AGGTTGGAGCCGCTGAGATGAC TCATCACACAGTCTGCTGAGGT GCTGAGGTTGGAGCCGCTGAGA GAGGTTGGAGCCGCTGAGATGA 90883517 rs111371406

HG00097 >hsa-mir-1227CATTTGACCCCGTGCCACCCTT ATTTGACCCCGTGCCACCCTTT AGGCATTTGACCCCGTGCCACC GACCCCGTGCCACCCTTTTCCC 2234093 rs190788838

HG00097 >hsa-mir-3151CCACCTGATCCCACACCCCACC CACCTGATCCCACACCCCACCT CCCACCTGATCCCACACCCCAC TGATCCCACACCCCACCTGTCA 104166902 rs35605502

HG00097 >hsa-mir-1343CCCCTCCTGGGGCCCGCACTCT CCCTCCTGGGGCCCGCACTCTC CCTGGGGCCCGCACTCTCGCTC TGGGGCCCGCACTCTCGCTCTG 34963416 rs2986407

HG00097 >hsa-mir-3166CCTACTGGCCTAAGAAAAATTT AGACAATGCCTACTGGCCTAAG ATGCCTACTGGCCTAAGAAAAA CAATGCCTACTGGCCTAAGAAA 87909673 rs35854553

HG00097 >hsa-mir-4268CCTCTCAGGATGTGATGTCACC CTCCTCTCAGGATGTGATGTCA CTCCTCCTCTCAGGATGTGATG rs4674470

HG00097 >hsa-mir-4254CCTGGAGATACTCCACCATCTC AGATACTCCACCATCTCCCCCA GGAGATACTCCACCATCTCCCC rs12731294

HG00097 >hsa-mir-1273h CCTGGGAGGTCAAGGCTGTAGT TGGGAGGTCAAGGCTGTAGTGT ATTGCTTGAGCCTGGGAGGTCA GCCTGGGAGGTCAAGGCTGTAG TTGAGCCTGGGAGGTCAAGGCT 24214486 .

HG00097 >hsa-mir-182 CGGTGGTTCTAGACTTGCCAAC CCGGTGGTTCTAGACTTGCCAA TCCGGTGGTTCTAGACTTGCCA GCCAACTATGGGGCGAGGACTC 129410227 rs76481776

HG00097 >hsa-mir-412 CGTCCGTATCCGCTGCAG CCGTCCGTATCCGCTGCAG TCACCTGGTCCACTGGCCGTCC ACCTGGTCCACTGGCCGTCCGT CACCTGGTCCACTGGCCGTCCG 101531854 rs61992671

HG00097 >hsa-mir-412 CGTCCGTATCCGCTGCAG CCGTCCGTATCCGCTGCAG TCACCTGGTTCACTGGCCGTCC ACCTGGTTCACTGGCCGTCCGT CACCTGGTTCACTGGCCGTCCG 101531849 . 101531854 rs61992671

HG00097 >hsa-mir-509-2 CGTCTGTGGGTAGAGTACTGCA AATGATTGGTACGTCTGTGGGT TGGTACGTCTGTGGGTAGAGTA

TGGGTAGAGTACTGCATGACAC 146340360 rs201600950

HG00097 >hsa-mir-3117CTCATATAGTGCCAGGTGTTTT GACTCATATAGTGCCAGGTGTT TCATATAGTGCCAGGTGTTTTG ATAAGACTCATATAGTGCCAGG 67094171 rs12402181
HG00097 >hsa-mir-3180-4 CTCCGGATGCCAGTCCCTCATC GGAGGGTGAAGCCTCCGGATGC AGCGGAGGGTGAAGCCTCCGGA CTGGCCTGGTCGCGCTGTGGCT GAGCGGAGGGTGAAGCCTCCGG 15248720 rs75000738
HG00097 >hsa-mir-196a-2 CTCGGCAACAAGAAACTGTCTG CAAGAAACTGTCTGAGTTACAT CAACAAGAAACTGTCTGAGTTA ACAAGAAACTGTCTGAGTTACA 54385599 rs11614913

HG00097 >hsa-mir-486-2 CTCGGCGCAGCTCAGTACAGGA AGGGCCTCGGCGCAGCTCAGTA TCGGCGCAGCTCAGTACAGGAT GGGCCTCGGCGCAGCTCAGTAC 41518007 .

HG00097 >hsa-mir-4268CTCTCAGGATGTGATGTCACCT CCTCTCAGGATGTGATGTCACC GCTCCTCCTCTCAGGATGTGAT TCCTCTCAGGATGTGATGTCAC CTCCTCCTCTCAGGATGTGATG 220771223 rs4674470

HG00097 >hsa-mir-3615CTCTCTCGGCTCCTCGCGGCTC GGCTCCTCGCGGCTCGCGGCGG CGGCTCCTCGCGGCTCGCGGCG TCGGCTCCTCGCGGCTCGCGGC 72744798 rs745666
HG00097 >hsa-mir-3151CTGATCCCACACCCCACCTGTC TGATCCCACACCCCACCTGTCA GATCCCACACCCCACCTGTCAC GGGCATCCCACCTGATCCCACA TCCCACCTGATCCCACACCCCA 104166902 rs35605502

HG00097 >hsa-mir-1304CTGTAGCATCGAACCCCTGGGC GAACCCCTGGGCTCAAGTGATT CTCACTGTAGCATCGAACCCCT CGAACCCCTGGGCTCAAGTGAT 93466866 rs2155248
HG00097 >hsa-mir-3922CTGTGGGACTTCTGGCCTTGAC ACCTGTGGGACTTCTGGCCTTG GGGACTTCTGGCCTTGACTTGA TGGGACTTCTGGCCTTGACTTG 104985443 rs61938575
HG00097 >hsa-mir-412 CTTCACCTGGTTCACTAGCCGT ACCTGGTTCACTAGCCGTCCGT TGTACTTCACCTGGTTCACTAG CTGGTTCACTAGCCGTCCGTAT GTACTTCACCTGGTTCACTAGC 101531849 .

HG00097 >hsa-mir-320eGAAAAGCTGGGTTGAGAAGGT AAAAGCTGGGTTGAGAAGGT GGAAAAGCTGGGTTGAGAAGGT GGGAAAAGCTGGGTTGAGAAGG rs10423365

HG00097 >hsa-mir-940 GAAGGCAGGGCCCC-GCTCCCC G CCC-GCTCCCCGGGCCTGACCC rs35356504

HG00097 >hsa-mir-548h-4 GACAAAAACCACAATTACTTTT AAAAACCACAATTACTTTTGCA TGACAAAAACCACAATTACTTT ACAAAAACCACAATTACTTTTG 26906402 rs73235381

HG00097 >hsa-mir-548h-4 GACAAAAACCACAATTACTTTT
AAAAACCACAATTACTTTTGCA TGACAAAAACCACAATTACTTT
ACAAAAACCACAATTACTTTTG 26906402 rs73235381 26906437

HG00097 >hsa-mir-548h-4 GACAAAAACCGCAATTACTTTT
TGACAAAAACCGCAATTACTTT AAAAACCGCAATTACTTTTGCA
AATGACAAAAACCGCAATTACT 26906437 rs184537764

HG00097 >hsa-mir-4274GACCCAGCAGTCCCTCCCCCTG CCCAGCAGTCCCTCCCCCTGCA
TGACCCAGCAGTCCCTCCCCCT TCAGGTGACCCAGCAGTCCCTC 7461769
rs12512664
HG00097 >hsa-mir-1307GACTCGGCGTGGCGTCGGTCGT CGTGGCGTCGGTCGTGGTAGAT
ATCGACTCGGCGTGGCGTCGGT GTGGCGTCGGTCGTGGTAGATA 105154089
rs7911488
HG00097 >hsa-mir-629 GAGGTTCTCCCAACGTAAGCCC AGGTTCTCCCAACGTAAGCCCA
TCTCCCAACGTAAGCCCAGCCC CAGGAGGTTCTCCCAACGTAAG 70371761
rs377691713
HG00097 >hsa-mir-509-2 GATTGGTACGTCTGTGGGTAGA
GTAGAGTACTGCATGACAC TAGAGTACTGCATGACAC CTGTGGGTAGAGTACTGCATGA
TCTGTGGGTAGAGTACTGCATG 146340360 rs201600950

HG00097 >hsa-mir-548ak GCAAAAGTAACTGCGGTTTTTG
TGCAAAAGTAACTGCGGTTTTT CAAAAGTAACTGCGGTTTTTGA
GTGCAAAAGTAACTGCGGTTTT rs7070684

HG00097 >hsa-mir-222 GCAGCTACATCTGGCTACTGGG TACTGGGTCTCTGATGGCATCT
GCTACTGGGTCTCTGATGGCAT CTGGCTACTGGGTCTCTGATGG 45606504
rs191727254
HG00097 >hsa-mir-1343GCCCCTCCTGGGGCCCGCACTC CCCCTCCTGGGGCCCGCACTCT
GGGGCCCGCACTCTCGCTCTGG CCCTCCTGGGGCCCGCACTCTC
TGGGGCCCGCACTCTCGCTCTG 34963416 rs2986407

HG00097 >hsa-mir-3180-4 GCGGAGGGTGAAGCCTCCGGAT
CGCTGGCCTGGTCGCGCTGTGG TCGCTGGCCTGGTCGCGCTGTG
AAGCCTCCGGATGCCAGTCCCT 15248720 rs75000738

HG00097 >hsa-mir-3196GCGGGGCGGCAGGGGCCTCCCC GGCGGGGCGGCAGGGGCCTCCC
GGGCGGGGCGGCAGGGGCCTCC TGGGGGCGGGGCGGCAGGGGCC
GGGGCGGGGCGGCAGGGGCCTC 61870167 rs744591

HG00097 >hsa-mir-2682GGACACCTCTTCAGCGCTGTCT CAGCGCTGTCTTCCCTGCCTCT
TTCAGCGCTGTCTTCCCTGCCT CACCTCTTCAGCGCTGTCTTCC 98510847
rs74904371
HG00097 >hsa-mir-3180-4 GGAGGGTGAAGCCTCCGGATGC
GGTGAAGCCTCCGGATGCCAGT GCGGAGGGTGAAGCCTCCGGAT
AGCGGAGGGTGAAGCCTCCGGA GCCTGGTCGCGCTGTGGCGAAG 15248798

rs183853838
HG00097 >hsa-mir-3180-4 GGAGGGTGAAGCCTCCGGATGC
GGTGAAGCCTCCGGATGCCAGT GCGGAGGGTGAAGCCTCCGGAT
AGCGGAGGGTGAAGCCTCCGGA CTGGCCTGGTCGCGCTGTGGCT 15248720
rs75000738 15248798 rs183853838
HG00097 >hsa-mir-548a-3 GGCAAAACTGGCAGTTACTTTT
GCAAAACTGGCAGTTACTTTTG AAAACTGGCAGTTACTTTTGCA
AACTGGCAGTTACTTTTGCACC 105496622 .

HG00097 >hsa-mir-1227GGCATTTGACCCCGTGCCACCC AGGCATTTGACCCCGTGCCACC
AGGCATTTGACCCCGTGCCACC TGACCCCGTGCCACCCTTTTCC
ATTTGACCCCGTGCCACCCTTT 2234093 rs190788838

HG00097 >hsa-mir-608 GGCCAAGGTGGGCCAGGGGTGG AAGGTGGGCCAGGGGTGGTGTT
GGGGTGGTGTTGGGACAGCTGC TGGTGTTGGGACAGCTGCGTTT
GGTGGGCCAGGGGTGGTGTTGG 102734778 rs4919510

HG00097 >hsa-mir-149 GGGAGGGAGGGACGGGGGCTGT GGAGGGACGGGGGCTGTGCTGG
AGGGACGGGGGCTGTGCTGGGG GAGGAGGGAGGGAGGGACGGGG 241395503
rs2292832
HG00097 >hsa-mir-1197GTAGGACACATGGTCTACTTCT ACACATGGTCTACTTCTTCTCA
ACATGGTCTACTTCTTCTCAAT TAGGACACATGGTCTACTTCTT 101491923
rs141611518
HG00097 >hsa-mir-658 GTAGGTCGGTTGGTCGGTCGGG G
TAGGTCGGTTGGTCGGTCGGGA 38240368 rs141002682

HG00097 >hsa-mir-658 GTAGGTCGGTTGGTCGGTCGGG TAGGTCGGTTGGTCGGTCGGGA
38240368 rs141002682 38240315 .

HG00097 >hsa-mir-449bGTATCGTTAGCTGGCTGCTTGG AGTGTATCGTTAGCTGGCTGCT
CAGTGTATCGTTAGCTGGCTGC GTGTATCGTTAGCTGGCTGCTT
TGTATCGTTAGCTGGCTGCTTG 54466544 rs10061133

HG00097 >hsa-mir-658 GTCCGTTGGTCGGTCGGGAACG G
TCCGTTGGTCGGTCGGGAACGA

HG00097 >hsa-mir-658 GTCCGTTGGTCGGTCGGGAACG
. rs141002682
HG00097 >hsa-mir-573 GTGTAACTGATCAGGATCTACT TGTGTAACTGATCAGGATCTAC
GATGTGTAACTGATCAGGATCT TGATGTGTAACTGATCAGGATC
GTGATGTGTAACTGATCAGGAT 24521902 rs76014664

HG00097 >hsa-mir-573 GTGTAACTGATCAGGATCTACT TGTGTAACTGATCAGGATCTAC
GATGTGTAACTGATCAGGATCT TGATGTGTAACTGATCAGGATC
GTGATGTGTAACTGATCAGGAT 24521904 rs78830737

HG00097 >hsa-mir-573 GTGTAACTGATCAGGATCTACT TGTGTAACTGATCAGGATCTAC

GATGTGTAACTGATCAGGATCT TGATGTGTAACTGATCAGGATC
GTGATGTGTAACTGATCAGGAT 24521902 rs76014664 24521904
rs78830737
HG00097 >hsa-mir-3117TAAAGGGCCAGACACTATACGA GGGCCAGACACTATACGAGTCA
GCCAGACACTATACGAGTCATA GGCCAGACACTATACGAGTCAT
CCCTAAAGGGCCAGACACTATA 67094171 rs12402181

HG00097 >hsa-mir-3144TAAGGGGACCAAAGAGATATAT TTAAGGGGACCAAAGAGATATA
AAGGGGACCAAAGAGATATATA CTACACTTTAAGGGGACCAAAG
TTTAAGGGGACCAAAGAGATAT 120336384 rs67106263

HG00097 >hsa-mir-3144TAAGGGGACCAAAGAGATATAT AAGGGGACCAAAGAGATATATA
TTAAGGGGACCAAAGAGATATA ATACACTTTAAGGGGACCAAAG
TTTAAGGGGACCAAAGAGATAT 120336327 rs68035463 120336384
rs67106263
HG00097 >hsa-mir-3144TAAGGGGACCAAAGAGATATAT TTAAGGGGACCAAAGAGATATA
TTTAAGGGGACCAAAGAGATAT TACACTTTAAGGGGACCAAAGA
AAATACACTTTAAGGGGACCAA 120336327 rs68035463

HG00097 >hsa-mir-3144TAAGGGGACCAAAGAGATATAT TTAAGGGGACCAAAGAGATATA
TTTAAGGGGACCAAAGAGATAT TACACTTTAAGGGGACCAAAGA
AACTACACTTTAAGGGGACCAA 120336384 rs67106263

HG00097 >hsa-mir-3144TAAGGGGACCAAAGAGATATAT TTAAGGGGACCAAAGAGATATA
TTTAAGGGGACCAAAGAGATAT TACACTTTAAGGGGACCAAAGA
AAATACACTTTAAGGGGACCAA 120336327 rs68035463 120336384
rs67106263
HG00097 >hsa-mir-3936TAAGGGGTGTATGGCAGATGCA TTCTGGTAAGGGGTGTATGGCA
CACCCGACAGATGCACTTGGCA TGTATGGCAGATGCACCCGACA 131701279
rs367805
HG00097 >hsa-mir-629 TACGTTGGGAGAACTTTTATGG TTACGTTGGGAGAACTTTTATG
TTTACGTTGGGAGAACTTTTAT TGGGTTTACGTTGGGAGAACTT
GTTTACGTTGGGAGAACTTTTA 70371761 rs377691713

HG00097 >hsa-mir-936 TAGAGGGAGGAATCGCAGAAAT TCAAGGCCACTGGGACAGTAGA
TGGGACAGTAGAGGGAGGAATC GGAGGAATCGCAGAAATCACTC
GGGAGGAATCGCAGAAATCACT 105807858 rs145823228

HG00097 >hsa-mir-300 TATACAAGGGCAGACTCTCTCT TGATTATACAAGGGCAGACTCT
ATTATACAAGGGCAGACTCTCT rs12894467

HG00097 >hsa-mir-580 TATTTGAGAATGATGAATCATT TGAATCATTAGGTTCCGGTCAG
ATGAATCATTAGGTTCCGGTCA TTTGAGAATGATGAATCATTAG
GAGAATGATGAATCATTAGGTT 36148057 rs115089112

HG00097 >hsa-mir-3922TCAAGGCCAGAGGTCCCACAAC TCAAGTCAAGGCCAGAGGTCCC
GCCAGAGGTCCCACAACAGGGC GGCCAGAGGTCCCACAACAGGG

GTCAAGGCCAGAGGTCCCACAA 104985443 rs61938575

HG00097 >hsa-mir-3141TCACCCGGTGAGGGCGGGTGGA CCGGTGAGGGCGGGTGGAGGAG CGGTGAGGGCGGGTGGAGGAGG CACCCGGTGAGGGCGGGTGGAG CCCGGTGAGGGCGGGTGGAGGA 153975576 rs936581

HG00097 >hsa-mir-412 TCACCTGGTTCACTAGCCGTCC TCACCTGGTTCACTAGCCGTCC ATGTACTTCACCTGGTTCACTA CTTCACCTGGTTCACTAGCCGT 101531849 .

HG00097 >hsa-mir-564 TCAGCAGGCAACATGGCCGAGA TGTCAGCAGGCAACATGGCCGA GTCAGCAGGCAACATGGCCGAG GTGTCAGCAGGCAACATGGCCG GGTGTCAGCAGGCAACATGGCC 44903434 rs2292181

HG00097 >hsa-mir-222 TCAGTAGCCAGTGTAGATCCTG TGGCTCAGTAGCCAGTGTAGAT TTGGCTCAGTAGCCAGTGTAGA TCATTGGCTCAGTAGCCAGTGT TACCCTCATTGGCTCAGTAGCC 45606504 rs191727254

HG00097 >hsa-mir-515-1 TCCAAAAGAAAGCACTTTCTGT TCTCCAAAAGAAAGCACTTTCT TTCTCCAAAAGAAAGCACTTTC TCATTCTCCAAAAGAAAGCACT TGCAGTCATTCTCCAAAAGAAA 54182326 rs374576826

HG00097 >hsa-mir-532 TCCCACACCCAAGGCTTGCAGA CTCCCACACCCAAGGCTTGCAG CCTCCCACACCCAAGGCTTGCA CACCCAAGGCTTGCAGAAGAGC 49767832 rs456615

HG00097 >hsa-mir-532 TCCCACACCCAAGGCTTGCAGA CTCCCACACCCAAGGCTTGCAG CCTCCCACACCCAAGGCTTGCA CACCCAAGGCTTGCAGAAGAGC 49767835 rs456617

HG00097 >hsa-mir-532 TCCCACACCCAAGGCTTGCAGA CTCCCACACCCAAGGCTTGCAG CCTCCCACACCCAAGGCTTGCA CACCCAAGGCTTGCAGAAGAGC 49767832 rs456615 49767835 rs456617

HG00097 >hsa-mir-663aTCCCAGGCGGGGCGCCGCGGGA TCCGGCGTCCCAGGCGGGGCGC TTCCGGCGTCCCAGGCGGGGCG GCGCCGCGGGACCTCCCTCGTG GGCGCCGCGGGACCTCCCTCGT 26188880 .

HG00097 >hsa-mir-596 TCCGAAGCCTGCCCGGCCCCTC GCCTGCCCGGCCCCTCGGGAAC TCTCCGAAGCCTGCCCGGCCCC CTGCCCGGCCCCTCGGGAACCT CCTGCCCGGCCCCTCGGGAACC 1765425 rs61388742

HG00097 >hsa-mir-149 TCCGTGTCTTCACTCCCGTGCT TGGCTCCGTGTCTTCACTCCCG TCTGGCTCCGTGTCTTCACTCC CCGTGTCTTCACTCCCGTGCTT AGCTCTGGCTCCGTGTCTTCAC 241395503 rs2292832

HG00097 >hsa-mir-618 TCCTTCTGAGTGTAATTACGTA TGTCCTTCTGAGTGTAATTACG TTGTCCTTCTGAGTGTAATTAC TACTTGTCCTTCTGAGTGTAAT GTCCTTCTGAGTGTAATTACGT 81329527 rs145551269

HG00097 >hsa-mir-618 TCCTTCTGAGTGTAATTACGTA TGTCCTTCTGAGTGTAATTACG

TTGTCCTTCTGAGTGTAATTAC TACTTGTCCTTCTGAGTGTAAT
GTCCTTCTGAGTGTAATTACGT 81329536 rs2682818

HG00097 >hsa-mir-618 TCCTTCTGAGTGTAATTACGTA TGTCCTTCTGAGTGTAATTACG
TTGTCCTTCTGAGTGTAATTAC TACTTGTCCTTCTGAGTGTAAT
GTCCTTCTGAGTGTAATTACGT 81329527 rs145551269 81329536
rs2682818
HG00097 >hsa-mir-492 TCGAGGACCTGCGGGACAAGAT TACAGGACCATCGAGGACCTGC
TACTACAGGACCATCGAGGACC TCCAGCCACTACTACAGGACCA
GACCTGCGGGACAAGATTCTTG 95228179 rs200816308

HG00097 >hsa-mir-3183TCGGAGTCGCTCGGAGCAGCCA TCTCGGAGTCGCTCGGAGCAGC
TCTCTCGGAGTCGCTCGGAGCA TCTGCCCTGCCTCTCTCGGAGT
TGCCCTGCCTCTCTCGGAGTCG 925764 rs2663345

HG00097 >hsa-mir-3671TCTATCAAATAAGGACTAGTCT AAATAAGGACTAGTCTGCAGTG
TTTATTTCTATCAAATAAGGAC CAAATAAGGACTAGTCTGCAGT 65523519
rs521188
HG00097 >hsa-mir-4326TCTGCTGTTCCTCTGTCTCCCA TGGTCTGCTGTTCCTCTGTCTC
CTGGTCTGCTGTTCCTCTGTCT GCTGTTCCTCTGTCTCCCAGAC
TGCTGTTCCTCTGTCTCCCAGA 61918164 rs6062431

HG00097 >hsa-mir-4305TCTGGGTTCTTAGAGGCCTAAT TTCTGGGTTCTTAGAGGCCTAA
GTTCTGGGTTCTTAGAGGCCTA TCCAGTTCTGGGTTCTTAGAGG
CAGTTCTGGGTTCTTAGAGGCC 40238175 rs67976778

HG00097 >hsa-mir-3118-1 TGAAAATTCTTCTAGTGTG ATGAAAATTCTTCTAGTGTG
TGCATTATGAAAATTCTTCTAG TTATGAAAATTCTTCTAGTGTG
ATTATGAAAATTCTTCTAGTGT 142667330 rs76132421

HG00097 >hsa-mir-635 TGAAACAATGTCCATTAGGCTT GAAACAATGTCCATTAGGCTTT
ACAATGTCCATTAGGCTTTGTT AACAATGTCCATTAGGCTTTGT
CTGAAACAATGTCCATTAGGCT 66420592 rs77279010

HG00097 >hsa-mir-1255a TGAGCAAAGAAAGTAGATTTTT
GCAAAGAAAGTAGATTTTTTAG TCAAGGATGAGCAAAGAAAGTA
GAGCAAAGAAAGTAGATTTTTT TCTCAAGGATGAGCAAAGAAAG 102251501
rs28664200
HG00097 >hsa-mir-3151TGATGGGTGGGGCAATGGGATC TGGGTGGGGCAATGGGATCAGG
TGGGGCAATGGGATCAGGTGCC GGGGTGATGGGTGGGGCAATGG
GGGTGATGGGTGGGGCAATGGG 104166902 rs35605502

HG00097 >hsa-mir-548a-3 TGCAAAAGTAATTGCGAGTTTT
TCGGTGCAAAAGTAATTGCGAG TAGGTCGGTGCAAAAGTAATTG
TTAGGTCGGTGCAAAAGTAATT TATTAGGTCGGTGCAAAAGTAA 105496622 .

HG00097 >hsa-mir-3156-2 TGCAGAAGAAAGATCTGGAAGT

GAAAGATCTGGAAGTGGGAGAC GAAGAAAGATCTGGAAGTGGGA
GCAGAAGAAAGATCTGGAAGTG CAGAAGAAAGATCTGGAAGTGG 14830215
rs113478966
HG00097 >hsa-mir-3156-3 TGCAGAAGAAAGATCTGGAAGT
GCAGAAGAAAGATCTGGAAGTG GAAGAAAGATCTGGAAGTGGGA
GAAAGATCTGGAAGTGGGAGAC AGAAGAAAGATCTGGAAGTGGG 14778721
rs2747232
HG00097 >hsa-mir-1200TGCTACTTCTCCTGAGCCATTC TGAGCCATTCTGAGCCTCAGTC
TACTTCTCCTGAGCCATTCTGA TCCTGAGCCATTCTGAGCCTCA
TTCTCCTGAGCCATTCTGAGCC 36959006 .

HG00097 >hsa-mir-1273h TGCTGCAGACTCGACCTCCCAG
TGCAGACTCGACCTCCCAGGCT CTGCAGACTCGACCTCCCAGGC
AGACTCGACCTCCCAGGCTTAA 24214486 .

HG00097 >hsa-mir-1254-2 TGGAAGCTGGAGCCTGCAGTGA
TGAGCCTGGAAGCTGGAGCCTG GAAGCTGGAGCCTGCAGTGAGC
GGAAGCTGGAGCCTGCAGTGAG GCCTGGAAGCTGGAGCCTGCAG 23682383
rs200793185
HG00097 >hsa-mir-3156-2 TGGCCCCCACTTCCAGATCTTT
CCCCCACTTCCAGATCTTTCTC ACTTCCAGATCTTTCTCTCTGT
CCCCACTTCCAGATCTTTCTCT 14830215 rs113478966

HG00097 >hsa-mir-637 TGGCTAAGGTGTTGGCTCGGGC TGGCTAAGGTGTTGGCTCGGGC

HG00097 >hsa-mir-608 TGGGACAGCTGCGTTTAAAAAG TTGGGACAGCTGCGTTTAAAAA
GGACAGCTGCGTTTAAAAAGGC TGTTGGGACAGCTGCGTTTAAA
GGGACAGCTGCGTTTAAAAAGG 102734778 rs4919510

HG00097 >hsa-mir-1273h TGGGAGGTCAAGGCTGTAGTGT
TGAGCCTGGGAGGTCAAGGCTG TTGAGCCTGGGAGGTCAAGGCT
TGCTTGAGCCTGGGAGGTCAAG TTGCTTGAGCCTGGGAGGTCAA 24214486 .

HG00097 >hsa-mir-345 TGGGCCCTGAACGAGGGGTCTG GTGGGCCCTGAACGAGGGGTCT
GCCCTGAACGAGGGGTCTGGAG GGCCCTGAACGAGGGGTCTGGA 100774203
rs72631832
HG00097 >hsa-mir-2682TGGGGCAGGCAGTGACTGTTCA TTGGGGCAGGCAGTGACTGTTC
GGCAGTGACTGTTCAGACGTCC TGAAAGAGGTTGGGGCAGGCAG
GCAGGCAGTGACTGTTCAGACG 98510847 rs74904371

HG00097 >hsa-mir-412 TGGGGTACGGGGATGGATGGTC GGATGGATGGTCGACCAGTTGG
GATGGATGGTCGACCAGTTGGA TCGACCAGTTGGAAAGTAATTG
ACGGGGATGGATGGTCGACCAG 101531854 rs61992671

HG00097 >hsa-mir-412 TGGGGTACGGGGATGGATGGTC GGATGGATGGTCGACCAGTTGG
GGGTACGGGGATGGATGGTCGA GATGGATGGTCGACCAGTTGGA
TCGACCAGTTGGAAAGTAATTG 101531849 . 101531854 rs61992671

HG00097 >hsa-mir-412 TGGGGTACGGGGATGGATGGTC TCGACCAGTTGGAAAGTAATTG
TGGTCGACCAGTTGGAAAGTAA TACGGGGATGGATGGTCGACCA
TGGATGGTCGACCAGTTGGAAA 101531854 rs61992671

HG00097 >hsa-mir-412 TGGGGTACGGGGATGGATGGTC TCGACCAGTTGGAAAGTAATTG
TGGTCGACCAGTTGGAAAGTAA TACGGGGATGGATGGTCGACCA
TGGATGGTCGACCAGTTGGAAA 101531849 . 101531854 rs61992671

HG00097 >hsa-mir-1227TGGTGGGCACTGCTGGGGTGGG TGGGGCCAGGCGGTGGTGGGCA
AGGCGGTGGTGGGCACTGCTGG GGTGGGCACTGCTGGGGTGGGC
GTGGTGGGCACTGCTGGGGTGG 2234093 rs190788838

HG00097 >hsa-mir-323bTGTCCGTGGTGAGTTCGCATTA TTGTCCGTGGTGAGTTCGCATT
TACTCGGAGGGAGGTTGTCCGT TCGGAGGGAGGTTGTCCGTGGT
AGGTTGTCCGTGGTGAGTTCGC 101522556 rs56103835

HG00097 >hsa-mir-2053TGTTAATTAAACCTCTATTTAC ACTTTAAGTGTTAATTAAACCT
TTTAAGTGTTAATTAAACCTCT TTAAGTGTTAATTAAACCTCTA 113655752
rs10505168
HG00097 >hsa-mir-642bTTCCCTCTCCAAATGTGTCTTG TTGGGAGGTTCCCTCTCCAAAT
TGGGAGGTTCCCTCTCCAAATG GAGTTGGGAGGTTCCCTCTCCA
GTTGGGAGGTTCCCTCTCCAAA 46178217 rs111664333

HG00097 >hsa-mir-4277TTCTGAGCACAGTACACTGGGC TCGAGGCAGTTCTGAGCACAGT
TGGGTCGAGGCAGTTCTGAGCA GTTCTGAGCACAGTACACTGGG
GCAGTTCTGAGCACAGTACACT 1708902 rs115200817

HG00097 >hsa-mir-4277TTCTGAGCACAGTACACTGGGC TCGAGGCAGTTCTGAGCACAGT
TGGGTCGAGGCAGTTCTGAGCA TTGGGTCGAGGCAGTTCTGAGC
GTTCTGAGCACAGTACACTGGG 1708983 rs12523324

HG00097 >hsa-mir-4277TTCTGAGCACAGTACACTGGGC TCGAGGCAGTTCTGAGCACAGT
TGGGTCGAGGCAGTTCTGAGCA TTGGGTCGAGGCAGTTCTGAGC
GTTCTGAGCACAGTACACTGGG 1708902 rs115200817 1708983
rs12523324
HG00097 >hsa-mir-585 TTGGGCGTATCTGTATGCTAGG TATCTGTATGCTAGGGCTGCCG
TGGGCGTATCTGTATGCTAGGG GCGTATCTGTATGCTAGGGCTG 168690612
rs62376934
HG00097 >hsa-mir-553 TTTAAAACGGTGAGATTTTGTT TTTTAAAACGGTGAGATTTTGT
ATTTTAAAACGGTGAGATTTTG TATTTTAAAACGGTGAGATTTT
TTATTTTAAAACGGTGAGATTT 100746848 .

HG00097 >hsa-mir-553 TTTAAGACGGTGAGATTTTGTT TTATTTTAAGACGGTGAGATTT
TATTTTAAGACGGTGAGATTTT TTTTATTTTAAGACGGTGAGAT
ATTTTAAGACGGTGAGATTTTG 100746814 rs190622705

HG00097 >hsa-mir-553 TTTAAGACGGTGAGATTTTGTT TTTTAAGACGGTGAGATTTTGT ATTTTAAGACGGTGAGATTTTG TATTTTAAGACGGTGAGATTTT TTATTTTAAGACGGTGAGATTT 100746814 rs190622705

HG00097 >hsa-mir-553 TTTAAGACGGTGAGATTTTGTT TTTTAAGACGGTGAGATTTTGT ATTTTAAGACGGTGAGATTTTG TATTTTAAGACGGTGAGATTTT TTATTTTAAGACGGTGAGATTT 100746814 rs190622705 100746848 .

HG00097 >hsa-mir-580 TTTGAGAATGATGAATCATTAG GATGAATCATTAGGTTCCGGTC AATGATGAATCATTAGGTTCCG AGAATGATGAATCATTAGGTTC 36148057 rs115089112

HG00097 >hsa-mir-553 TTTTAAAACGGTGAGATTTTGT TATTTTAAAACGGTGAGATTTT TTTTATTTTAAAACGGTGAGAT TTTAAAACGGTGAGATTTTGTT ATTTTATTTTAAAACGGTGAGA 100746848 .

HG00097 >hsa-mir-553 TTTTAAGACGGTGAGATTTTGT TATTTTAAGACGGTGAGATTTT TTTAAGACGGTGAGATTTTGTT TTTTATTTTAAGACGGTGAGAT ATTTTATTTTAAGACGGTGAGA 100746814 rs190622705 100746848 .

HG00096 >hsa-mir-1273h CCTGGGAGGTCAAGGCTGTAGT TGGGAGGTCAAGGCTGTAGTGT GCCTGGGAGGTCAAGGCTGTAG ATTGCTTGAGCCTGGGAGGTCA TTGAGCCTGGGAGGTCAAGGCT 24214486 .

HG00096 >hsa-mir-3196TGGGGGCAGGGCGGCAGGGGCC GGTGGGGGCAGGGCGGCAGGGG GGGGGCAGGGCGGCAGGGGCCT GTGGGGGCAGGGCGGCAGGGGC GGGTGGGGGCAGGGCGGCAGGG 61870141 rs113297757

HG00096 >hsa-mir-3196GCGGGGCGGCAGGGGCCTCCCC GGGCGGGGCGGCAGGGGCCTCC GGCGGGGCGGCAGGGGCCTCCC TGGGGGCGGGGCGGCAGGGGCC GGGGCGGGGCGGCAGGGGCCTC 61870167 rs744591

HG00096 >hsa-mir-3196GCAGGGCGGCAGGGGCCTCCCC GGGCAGGGCGGCAGGGGCCTCC TGGGGGCAGGGCGGCAGGGGCC GGTGGGGGCAGGGCGGCAGGGG GGGGGCAGGGCGGCAGGGGCCT 61870141 rs113297757 61870167 rs744591

HG00096 >hsa-mir-412 TGGGGTACGGGGATGGATGGTC GGATGGATGGTCGACCAGTTGG GATGGATGGTCGACCAGTTGGA TCGACCAGTTGGAAAGTAATTG GGGTACGGGGATGGATGGTCGA 101531854 rs61992671

HG00096 >hsa-mir-4309CTGGGGGTTCTGGAGTCTAGGA TGGAGTCTAGGATTCCAGGATC GGAGTCTAGGATTCCAGGATCT GGGGGTTCTGGAGTCTAGGATT GGGTTCTGGAGTCTAGGATTCC 103006047 rs12879262

HG00096 >hsa-mir-4326TCTGCTGTTCCTCTGTCTCCCA TGGTCTGCTGTTCCTCTGTCTC CTGGTCTGCTGTTCCTCTGTCT GCTGTTCCTCTGTCTCCCAGAC TGCTGTTCCTCTGTCTCCCAGA 61918164 rs6062431

HG00096 >hsa-mir-548ak GCAAAAGTAACTGCGGTTTTTG
TGCAAAAGTAACTGCGGTTTTT GTGCAAAAGTAACTGCGGTTTT
CAAAAGTAACTGCGGTTTTTGA rs7070684

HG00096 >hsa-mir-553 TTTAAGACGGTGAGATTTTGTT TTATTTTAAGACGGTGAGATTT
TATTTTAAGACGGTGAGATTTT ATTTTAAGACGGTGAGATTTTG
TTTTATTTTAAGACGGTGAGAT 100746814 rs190622705

HG00096 >hsa-mir-553 TTTTAAAACGGTGAGATTTTGT TTTAAAACGGTGAGATTTTGTT
TTATTTTAAAACGGTGAGATTT ATTTTAAAACGGTGAGATTTTG
TATTTTAAAACGGTGAGATTTT 100746835 .

HG00096 >hsa-mir-553 TTTAAAACGGTGAGATTTTGTT TTATTTTAAAACGGTGAGATTT
ATTTTAAAACGGTGAGATTTTG TATTTTAAAACGGTGAGATTTT
TTTATTTTAAAACGGTGAGATT 100746848 .

HG00096 >hsa-mir-553 TTTTATTTTAAAACGGTGAGAT TTTAAAACGGTGAGATTTTGTT
TTTTAAAACGGTGAGATTTTGT TTATTTTAAAACGGTGAGATTT
AATTTTATTTTAAAACGGTGAG 100746855 rs112891767

HG00096 >hsa-mir-553 TTTAAGACGGTGAGATTTTGTT TTTTAAGACGGTGAGATTTTGT
TTATTTTAAGACGGTGAGATTT TATTTTAAGACGGTGAGATTTT
ATTTTAAGACGGTGAGATTTTG 100746814 rs190622705 100746835 .

HG00096 >hsa-mir-553 TTTAAGACGGTGAGATTTTGTT TTATTTTAAGACGGTGAGATTT
TATTTTAAGACGGTGAGATTTT ATTTTAAGACGGTGAGATTTTG
TTTTATTTTAAGACGGTGAGAT 100746814 rs190622705 100746848 .

HG00096 >hsa-mir-553 TTTAAGACGGTGAGATTTTGTT TTTTATTTTAAGACGGTGAGAT
TTTTAAGACGGTGAGATTTTGT AATTTTATTTTAAGACGGTGAG
TTATTTTAAGACGGTGAGATTT 100746814 rs190622705 100746855
rs112891767
HG00096 >hsa-mir-553 TTTTAAAACGGTGAGATTTTGT TATTTTAAAACGGTGAGATTTT
TTTTATTTTAAAACGGTGAGAT TTTAAAACGGTGAGATTTTGTT
ATTTTATTTTAAAACGGTGAGA 100746835 . 100746848 .

HG00096 >hsa-mir-553 TTTTAAAACGGTGAGATTTTGT TTTTATTTTAAAACGGTGAGAT
TTATTTTAAAACGGTGAGATTT AATTTTATTTTAAAACGGTGAG
TTTAAAACGGTGAGATTTTGTT 100746835 . 100746855
rs112891767
HG00096 >hsa-mir-553 TTTTATTTTAAAACGGTGAGAT TTTAAAACGGTGAGATTTTGTT
TTTTAAAACGGTGAGATTTTGT TTATTTTAAAACGGTGAGATTT
AATTTTATTTTAAAACGGTGAG 100746848 . 100746855
rs112891767
HG00096 >hsa-mir-553 TTTTAAGACGGTGAGATTTTGT TATTTTAAGACGGTGAGATTTT
TTTAAGACGGTGAGATTTTGTT TTTTATTTTAAGACGGTGAGAT
ATTTTATTTTAAGACGGTGAGA 100746814 rs190622705 100746835 .

100746848 .
HG00096 >hsa-mir-553 TTTTAAAACGGTGAGATTTTGT TTTTATTTTAAAACGGTGAGAT
TTTAAAACGGTGAGATTTTGTT TATTTTAAAACGGTGAGATTTT
ATTTTATTTTAAAACGGTGAGA 100746835 . 100746848 .
100746855 rs112891767
HG00096 >hsa-mir-553 TTTTAAGACGGTGAGATTTTGT TTTTATTTTAAGACGGTGAGAT
TTTAAGACGGTGAGATTTTGTT TATTTTAAGACGGTGAGATTTT
ATTTTATTTTAAGACGGTGAGA 100746814 rs190622705 100746835 .
100746848 . 100746855 rs112891767
HG00096 >hsa-mir-1283-1 AAAGGAAAGCGCTTTCTGTTGT
ACAAAGGAAAGCGCTTTCTGTT AGTCTACAAAGGAAAGCGCTTT
CAAAGGAAAGCGCTTTCTGTTG CTACAAAGGAAAGCGCTTTCTG 54191743
rs57111412
HG00096 >hsa-mir-486-2 ACTGAGCTGCCCCGAGCTGGGC
CTGAGCTGCCCCGAGCTGGGCA CTGTACTGAGCTGCCCCGAGCT
CCTGTACTGAGCTGCCCCGAGC GTACTGAGCTGCCCCGAGCTGG 41518007 .

HG00096 >hsa-mir-378hAGATGGGATGAGCCCTGGCTCT TCAGATGGGATGAGCCCTGGCT
TGTCAGATGGGATGAGCCCTGG GATGGGATGAGCCCTGGCTCTG
CAGATGGGATGAGCCCTGGCTC 154209024 rs702742

HG00096 >hsa-mir-1269b AGCCATGCTACGGGCTTCTCTG
ACTGAGCCATGCTACGGGCTTC AGGTTTCTGGACTGAGCCATGC
TGAGGTTTCTGGACTGAGCCAT TTTCTGGACTGAGCCATGCTAC 12820632
rs12451747
HG00096 >hsa-mir-3135b AGCGAGTGCAGTGGTGCAGTCA
AGGCTGGAGCGAGTGCAGTGGT CTGGAGCGAGTGCAGTGGTGCA
CAGGCTGGAGCGAGTGCAGTGG CCAGGCTGGAGCGAGTGCAGTG 32717722
rs4351242
HG00096 >hsa-mir-548ap AGTAATTGCAGTCTTTGTCATT
AAGTAATTGCAGTCTTTGTCAT AAAGTAATTGCAGTCTTTGTCA
AAAAGTAATTGCAGTCTTTGTC CAAAAGTAATTGCAGTCTTTGT 86368898
rs4414449
HG00096 >hsa-mir-548ap AGTAATTGCAGTCTTTGTCATT
AAGTAATTGCAGTCTTTGTCAT AAAGTAATTGCAGTCTTTGTCA
AAAAGTAATTGCAGTCTTTGTC CAAAAGTAATTGCAGTCTTTGT 86368898
rs4414449 86368959 rs4577031
HG00096 >hsa-mir-548ap AGTAATTGCGGTCTTTGTCATT
AAGTAATTGCGGTCTTTGTCAT AAAGTAATTGCGGTCTTTGTCA
AAAAGTAATTGCGGTCTTTGTC CAAAAGTAATTGCGGTCTTTGT 86368959
rs4577031
HG00096 >hsa-mir-1273d GAGGTTGAGGCTGCAGTGAGCC
TGAGGTTGAGGCTGCAGTGAGC CATGAGGTTGAGGCTGCAGTGA
CCATGAGGTTGAGGCTGCAGTG ATGAGGTTGAGGCTGCAGTGAG 10287824
rs150712502
HG00096 >hsa-mir-573 GTGTAACTGATCAGGATCTACT TGTGTAACTGATCAGGATCTAC
GATGTGTAACTGATCAGGATCT TGATGTGTAACTGATCAGGATC

GTGATGTGTAACTGATCAGGAT 24521902 rs76014664

HG00096 >hsa-mir-3117TAAAGGGCCAGACACTATACGA GGGCCAGACACTATACGAGTCA GCCAGACACTATACGAGTCATA GGCCAGACACTATACGAGTCAT CCCTAAAGGGCCAGACACTATA 67094171 rs12402181

HG00096 >hsa-mir-559 TAAATATGCACCAAAATTACTT ATATGCACCAAAATTACTTCTG AATATGCACCAAAATTACTTCT AAATATGCACCAAAATTACTTC TAAAGTAAATATGCACCAAAAT 47604856 rs114803590

HG00096 >hsa-mir-629 TACGTTGGGAGAACTTTTATGG TTACGTTGGGAGAACTTTTATG TTTACGTTGGGAGAACTTTTAT TGGGTTTACGTTGGGAGAACTT GTTTACGTTGGGAGAACTTTTA 70371761 rs377691713

HG00096 >hsa-mir-888 TACTCAAAAAGCTTTCAGTCAC TCTACTCAAAAAGCTTTCAGTC TGCTCTACTCAAAAAGCTTTCA GCTCTACTCAAAAAGCTTTCAG GGCAGTGCTCTACTCAAAAAGC 145076355 rs143634721

HG00096 >hsa-mir-449cTAGGCAGTGTATTGCTAGCGGC TCAGATAGGCAGTGTATTGCTA TGTCAGATAGGCAGTGTATTGC TGTGTCAGATAGGCAGTGTATT TGGGATGTGTCAGATAGGCAGT 54468166 rs75661995

HG00096 >hsa-mir-590 TATTCATAAAAGTGCAGTATGG TTATTCATAAAAGTGCAGTATG TTTATTCATAAAAGTGCAGTAT AGTTTATTCATAAAAGTGCAGT TGAGTTTATTCATAAAAGTGCA 73605546 rs189727189

HG00096 >hsa-mir-564 TCAGCAGGCAACATGGCCGAGA TGTCAGCAGGCAACATGGCCGA GTCAGCAGGCAACATGGCCGAG TGCCAGGCACGGTGTCAGCAGG GTGTCAGCAGGCAACATGGCCG 44903433 .

HG00096 >hsa-mir-515-1 TCCAAAAGAAAGCACTTTCTGT TCTCCAAAAGAAAGCACTTTCT TTCTCCAAAAGAAAGCACTTTC TCATTCTCCAAAAGAAAGCACT TGCAGTCATTCTCCAAAAGAAA 54182326 rs374576826
HG00096 >hsa-mir-663aTCCCAGGCGGGGCGCCGCGGGA TCCGGCGTCCCAGGCGGGGCGC TTCCGGCGTCCCAGGCGGGGCG GCGCCGCGGGACCTCCCTCGTG GGCGCCGCGGGACCTCCCTCGT 26188880 .

HG00096 >hsa-mir-596 TCCGAAGCCTGCCCGGCCCCTC GCCTGCCCGGCCCCTCGGGAAC TCTCCGAAGCCTGCCCGGCCCC CTGCCCGGCCCCTCGGGAACCT CCTGCCCGGCCCCTCGGGAACC 1765425 rs61388742

HG00096 >hsa-mir-149 TCCGTGTCTTCACTCCCGTGCT TGGCTCCGTGTCTTCACTCCCG TCTGGCTCCGTGTCTTCACTCC CCGTGTCTTCACTCCCGTGCTT AGCTCTGGCTCCGTGTCTTCAC 241395503 rs2292832

HG00096 >hsa-mir-3183TCGGAGTCGCTCGGAGCAGCCA TCTCGGAGTCGCTCGGAGCAGC

TCTCTCGGAGTCGCTCGGAGCA TCTGCCCTGCCTCTCTCGGAGT
TGCCCTGCCTCTCTCGGAGTCG 925764 rs2663345

HG00096 >hsa-mir-520cTCTCAGGCTGTCGTCCTCTAGA TCAGGCTGTCGTCCTCTAGAGG
TGTCGTCCTCTAGAGGGAAGCA TCGTCCTCTAGAGGGAAGCACT
TCCTCTAGAGGGAAGCACTTTC 54210774 .

HG00096 >hsa-mir-4305TCTGGGTTCTTAGAGGCCTAAT TTCTGGGTTCTTAGAGGCCTAA
GTTCTGGGTTCTTAGAGGCCTA TCCAGTTCTGGGTTCTTAGAGG
CAGTTCTGGGTTCTTAGAGGCC 40238175 rs67976778

HG00096 >hsa-mir-635 TGAAACAATGTCCATTAGGCTT GAAACAATGTCCATTAGGCTTT
ACAATGTCCATTAGGCTTTGTT AACAATGTCCATTAGGCTTTGT
CTGAAACAATGTCCATTAGGCT 66420592 rs77279010

HG00096 >hsa-mir-27a TGAGGAGCAGGGCTTAGCTGCT TTAGCTGCTTGTGAGCAGGGTC
GAGGAGCAGGGCTTAGCTGCTT GGAGCAGGGCTTAGCTGCTTGT
GAGCAGGGCTTAGCTGCTTGTG 13947292 rs895819

HG00096 >hsa-mir-3151TGATGGGTGGGGCAATGGGATC TGGGTGGGGCAATGGGATCAGG
TGGGGCAATGGGATCAGGTGCC GGGGTGATGGGTGGGGCAATGG
GGGTGATGGGTGGGGCAATGGG 104166902 rs35605502

HG00096 >hsa-mir-570 TGCAAAGGTAATCGCAGTTTTT TGGGTGCAAAGGTAATCGCAGT
GTAATCGCAGTTTTTCCCATTA GGTAATCGCAGTTTTTCCCATT
TAGGTGGGTGCAAAGGTAATCG 195426305 rs9860655

HG00096 >hsa-mir-3156-2 TGCAGAAGAAAGATCTGGAAGT
GAAAGATCTGGAAGTGGGAGAC GAAGAAAGATCTGGAAGTGGGA
GCAGAAGAAAGATCTGGAAGTG CAGAAGAAAGATCTGGAAGTGG 14830215
rs113478966
HG00096 >hsa-mir-3156-3 TGCAGAAGAAAGATCTGGAAGT
GCAGAAGAAAGATCTGGAAGTG GAAGAAAGATCTGGAAGTGGGA
GAAAGATCTGGAAGTGGGAGAC AGAAGAAAGATCTGGAAGTGGG 14778721
rs2747232
HG00096 >hsa-mir-3152TGCCTCTGTTCTAACACAAGAC TTGCCTCTGTTCTAACACAAGA
TATTGCCTCTGTTCTAACACAA TTATTGCCTCTGTTCTAACACA
TGCAGAGTTATTGCCTCTGTTC 18573360 rs13299349

HG00096 >hsa-mir-3182TGCTTCTGTAGTGTAGTCCGTG TTCTGTAGTGTAGTCCGTGCAT
TCTGTAGTGTAGTCCGTGCATC GCTGCTTCTGTAGTGTAGTCCG
GCTTCTGTAGTGTAGTCCGTGC 83541990 rs139736497

HG00096 >hsa-mir-1254-2 TGGAAGCTGGAGCCTGCAGTGA
TGAGCCTGGAAGCTGGAGCCTG GAAGCTGGAGCCTGCAGTGAGC
GGAAGCTGGAGCCTGCAGTGAG GCCTGGAAGCTGGAGCCTGCAG 23682383
rs200793185

HG00096 >hsa-mir-378d-2 TGGACTTGGAGTCAGAAAACTT
GACTTGGAGTCAGAAAACTTTC GGACTTGGAGTCAGAAAACTTT
GAACACTGGACTTGGAGTCAGA TACAAGGAGAGAACACTGGACT 94928250
rs73692959
HG00096 >hsa-mir-516b-2 TGGAGGTAAGAAGCACTTTGTG
TCTGGAGGTAAGAAGCACTTTG TGACCATCTGGAGGTAAGAAGC
TGTGACCATCTGGAGGTAAGAA TGATGTGACCATCTGGAGGTAA 54228742
rs10670323
HG00096 >hsa-mir-4309TGGAGTCTAGGATTCCAGGATC GGAGTCTAGGATTCCAGGATCT
TCTGGGGGTTCTGGAGTCTAGG TTCTGGAGTCTAGGATTCCAGG
TGGGGGTTCTGGAGTCTAGGAT 103006047 rs12879262

HG00096 >hsa-mir-1273h TGGGAGGTCAAGGCTGTAGTGT
TGAGCCTGGGAGGTCAAGGCTG TTGAGCCTGGGAGGTCAAGGCT
TGCTTGAGCCTGGGAGGTCAAG TTGCTTGAGCCTGGGAGGTCAA 24214486 .

HG00096 >hsa-mir-1227TGGGGCCAGGCGGTGGTGGGCA TGGTGGGCACTGCTGGGGTGGG
AGGCGGTGGTGGGCACTGCTGG GTGGGGCCAGGCGGTGGTGGGC
GGGGCCAGGCGGTGGTGGGCAC 2234093 rs190788838

HG00096 >hsa-mir-412 TGGGGTACGGGGATGGATGGTC TCGACCAGTTGGAAAGTAATTG
TGGTCGACCAGTTGGAAAGTAA TACGGGGATGGATGGTCGACCA
TGGATGGTCGACCAGTTGGAAA 101531854 rs61992671

HG00096 >hsa-mir-323bTGTCCGTGGTGAGTTCGCATTA TTGTCCGTGGTGAGTTCGCATT
TACTCGGAGGGAGGTTGTCCGT TCGGAGGGAGGTTGTCCGTGGT
AGGTTGTCCGTGGTGAGTTCGC 101522556 rs56103835

HG00096 >hsa-mir-27a TTAGCTGCTTGTGAGCAGGGTT TGAGGAGCAGGGCTTAGCTGCT
GCTTAGCTGCTTGTGAGCAGGG GGCTTAGCTGCTTGTGAGCAGG
GAGGAGCAGGGCTTAGCTGCTT 13947296 rs11671784

HG00096 >hsa-mir-27a TTAGCTGCTTGTGAGCAGGGTT TGAGGAGCAGGGCTTAGCTGCT
GCTTAGCTGCTTGTGAGCAGGG GGCTTAGCTGCTTGTGAGCAGG
GAGGAGCAGGGCTTAGCTGCTT 13947292 rs895819 13947296
rs11671784
HG00096 >hsa-mir-642bTTCCCTCTCCAAATGTGTCTTG TTGGGAGGTTCCCTCTCCAAAT
TGGGAGGTTCCCTCTCCAAATG GAGTTGGGAGGTTCCCTCTCCA
GTTGGGAGGTTCCCTCTCCAAA 46178217 rs111664333

HG00096 >hsa-mir-4277TTCTGAGCACAGTACACTGGGC TCGAGGCAGTTCTGAGCACAGT
TGGGTCGAGGCAGTTCTGAGCA GTTCTGAGCACAGTACACTGGG
GCAGTTCTGAGCACAGTACACT 1708902 rs115200817

HG00096 >hsa-mir-4277TTCTGAGCACAGTACACTGGGC TCGAGGCAGTTCTGAGCACAGT
TGGGTCGAGGCAGTTCTGAGCA TTGGGTCGAGGCAGTTCTGAGC
GTTCTGAGCACAGTACACTGGG 1708983 rs12523324

HG00096 >hsa-mir-4277TTCTGAGCACAGTACACTGGGC TCGAGGCAGTTCTGAGCACAGT TGGGTCGAGGCAGTTCTGAGCA TTGGGTCGAGGCAGTTCTGAGC GTTCTGAGCACAGTACACTGGG 1708902 rs115200817 1708983 rs12523324
HG00096 >hsa-mir-553 TTTAAAACGGTGAGATTTTGTT TTTTAAAACGGTGAGATTTTGT ATTTTAAAACGGTGAGATTTTG TATTTTAAAACGGTGAGATTTT TTATTTTAAAACGGTGAGATTT 100746835 .

HG00096 >hsa-mir-553 TTTAAAACGGTGAGATTTTGTT TTTTAAAACGGTGAGATTTTGT ATTTTAAAACGGTGAGATTTTG TATTTTAAAACGGTGAGATTTT TTATTTTAAAACGGTGAGATTT 100746848 .

HG00096 >hsa-mir-553 TTTAAAACGGTGAGATTTTGTT TTTTAAAACGGTGAGATTTTGT ATTTTAAAACGGTGAGATTTTG TATTTTAAAACGGTGAGATTTT TTATTTTAAAACGGTGAGATTT 100746855 rs112891767

HG00096 >hsa-mir-553 TTTAAAACGGTGAGATTTTGTT TTTTAAAACGGTGAGATTTTGT ATTTTAAAACGGTGAGATTTTG TATTTTAAAACGGTGAGATTTT TTATTTTAAAACGGTGAGATTT 100746835 . 100746848 .

HG00096 >hsa-mir-553 TTTAAAACGGTGAGATTTTGTT TTTTAAAACGGTGAGATTTTGT ATTTTAAAACGGTGAGATTTTG TATTTTAAAACGGTGAGATTTT TTATTTTAAAACGGTGAGATTT 100746835 . 100746855 rs112891767
HG00096 >hsa-mir-553 TTTAAAACGGTGAGATTTTGTT TTTTAAAACGGTGAGATTTTGT ATTTTAAAACGGTGAGATTTTG TATTTTAAAACGGTGAGATTTT TTATTTTAAAACGGTGAGATTT 100746848 . 100746855 rs112891767
HG00096 >hsa-mir-553 TTTAAAACGGTGAGATTTTGTT TTTTAAAACGGTGAGATTTTGT ATTTTAAAACGGTGAGATTTTG TATTTTAAAACGGTGAGATTTT TTATTTTAAAACGGTGAGATTT 100746835 . 100746848 . 100746855 rs112891767
HG00096 >hsa-mir-553 TTTAAGACGGTGAGATTTTGTT TTTTAAGACGGTGAGATTTTGT ATTTTAAGACGGTGAGATTTTG TATTTTAAGACGGTGAGATTTT TTATTTTAAGACGGTGAGATTT 100746814 rs190622705

HG00096 >hsa-mir-553 TTTAAGACGGTGAGATTTTGTT TTTTAAGACGGTGAGATTTTGT ATTTTAAGACGGTGAGATTTTG TATTTTAAGACGGTGAGATTTT TTATTTTAAGACGGTGAGATTT 100746814 rs190622705 100746835 .

HG00096 >hsa-mir-553 TTTAAGACGGTGAGATTTTGTT TTTTAAGACGGTGAGATTTTGT ATTTTAAGACGGTGAGATTTTG TATTTTAAGACGGTGAGATTTT TTATTTTAAGACGGTGAGATTT 100746814 rs190622705 100746848 .

HG00096 >hsa-mir-553 TTTAAGACGGTGAGATTTTGTT TTTTAAGACGGTGAGATTTTGT ATTTTAAGACGGTGAGATTTTG TATTTTAAGACGGTGAGATTTT

TTATTTTAAGACGGTGAGATTT 100746814 rs190622705 100746855
rs112891767
HG00096 >hsa-mir-553 TTTAAGACGGTGAGATTTTGTT TTTTAAGACGGTGAGATTTTGT
ATTTTAAGACGGTGAGATTTTG TATTTTAAGACGGTGAGATTTT
TTATTTTAAGACGGTGAGATTT 100746814 rs190622705 100746835 .
100746848 .
HG00096 >hsa-mir-553 TTTAAGACGGTGAGATTTTGTT TTTTAAGACGGTGAGATTTTGT
ATTTTAAGACGGTGAGATTTTG TATTTTAAGACGGTGAGATTTT
TTATTTTAAGACGGTGAGATTT 100746814 rs190622705 100746835 .
100746848 . 100746855 rs112891767
HG00096 >hsa-mir-1227AGGCATTTGACCCCGTGCCACC GGCATTTGACCCCGTGCCACCC
AGGCATTTGACCCCGTGCCACC TGACCCCGTGCCACCCTTTTCC
AGGCATTTGACCCCGTGCCACC 2234093 rs190788838

HG00096 >hsa-mir-149 AGGGAGGGAGGGACGGGGGCTG GGGCTGTGCTGGGGCAGCCGGA
GGGACGGGGGCTGTGCTGGGGC GAGGGAGGGAGGGACGGGGGCT
GGGAGGGAGGGACGGGGGCTGT 241395503 rs2292832

HG00096 >hsa-mir-658 AGGTCGGTTGGTCGGTCGGGAA GTCGGTTGGTCGGTCGGGAACG
TAGGTCGGTTGGTCGGTCGGGA

HG00096 >hsa-mir-622 CAGTCTGCTGAGGTTGGAGCCG AGGTTGGAGCCGCTGAGATGAC
TCATCACACAGTCTGCTGAGGT GCTGAGGTTGGAGCCGCTGAGA
GAGGTTGGAGCCGCTGAGATGA 90883517 rs111371406

HG00096 >hsa-mir-412 CGTCCGTATCCGCTGCAG CCGTCCGTATCCGCTGCAG
TCACCTGGTCCACTGGCCGTCC ACCTGGTCCACTGGCCGTCCGT
CACCTGGTCCACTGGCCGTCCG 101531854 rs61992671

HG00096 >hsa-mir-3180-4 CTCCGGATGCCAGTCCCTCATC
GGAGGGTGAAGCCTCCGGATGC AGCGGAGGGTGAAGCCTCCGGA
CTGGCCTGGTCGCGCTGTGGCT GAGCGGAGGGTGAAGCCTCCGG 15248720
rs75000738
HG00096 >hsa-mir-4268CTCTCAGGATGTGATGTCACCT CCTCTCAGGATGTGATGTCACC
GCTCCTCCTCTCAGGATGTGAT TCCTCTCAGGATGTGATGTCAC
CTCCTCCTCTCAGGATGTGATG 220771223 rs4674470

HG00096 >hsa-mir-3151CTGATCCCACACCCCACCTGTC TGATCCCACACCCCACCTGTCA
GATCCCACACCCCACCTGTCAC GGGCATCCCACCTGATCCCACA
TCCCACCTGATCCCACACCCCA 104166902 rs35605502

HG00096 >hsa-mir-658 TAGGTCGGTTGGTCGGTCGGGA

HG00096 >hsa-mir-658 GTCCGTTGGTCGGTCGGGAACG TCCGTTGGTCGGTCGGGAACGA

HG00096 >hsa-mir-320eGAAAAGCTGGGTTGAGAAGGT AAAAGCTGGGTTGAGAAGGT
GGAAAAGCTGGGTTGAGAAGGT GGGAAAAGCTGGGTTGAGAAGG

rs10423365
HG00096 >hsa-mir-3180-4 GGAGGGTGAAGCCTCCGGATGC
GGTGAAGCCTCCGGATGCCAGT GCGGAGGGTGAAGCCTCCGGAT
AGCGGAGGGTGAAGCCTCCGGA GCCTGGTCGCGCTGTGGCGAAG 15248798
rs183853838
HG00096 >hsa-mir-3180-4 GGAGGGTGAAGCCTCCGGATGC
GGTGAAGCCTCCGGATGCCAGT GCGGAGGGTGAAGCCTCCGGAT
AGCGGAGGGTGAAGCCTCCGGA CTGGCCTGGTCGCGCTGTGGCT 15248720
rs75000738 15248798 rs183853838
HG00096 >hsa-mir-580 TATTTGAGAATGATGAATCATT TGAATCATTAGGTTCCGGTCAG
ATGAATCATTAGGTTCCGGTCA TTTGAGAATGATGAATCATTAG
GAGAATGATGAATCATTAGGTT 36148057 rs115089112

HG00096 >hsa-mir-3118-1 TGAAAATTCTTCTAGTGTG ATGAAAATTCTTCTAGTGTG
TGCATTATGAAAATTCTTCTAG TTATGAAAATTCTTCTAGTGTG
CTGCATTATGAAAATTCTTCTA 142667330 rs76132421

HG00096 >hsa-mir-516b-2 TGCTTCCTTTCAGAGGGTTACT
TCAGAGGGTTACTCTTTGAGA AAAGAAAGTGCTTCCTTTCAGA
CAGAGGGTTACTCTTTGAGA AAAAGAAAGTGCTTCCTTTCAG 54228742
rs10670323
HG00096 >hsa-mir-320c-1 AAAAGCTGGGTTGAGAGGGTAG
AGCTGGGTTGAGAGGGTAGGAA CTGGGTTGAGAGGGTAGGAAAA
AGGGTAGGAAAAAAATGATGTA 19263542 .

HG00096 >hsa-mir-499bAAACATCACTGCAAGTCTTAAC
rs3746444
HG00096 >hsa-mir-548ap AACAAAAACCACAATTACTTTT
CAAAAACCACAATTACTTTTTA CAATTACTTTTTACTGACCTAA
rs4414449
HG00096 >hsa-mir-548ap AACAAAAACCACAATTACTTTT
CAAAAACCACAATTACTTTTTA TTACTTTTTACTGACCTAAAGA
rs4577031
HG00096 >hsa-mir-548ap AACAAAAACCACAATTACTTTT
CAAAAACCACAATTACTTTTTA CAATTACTTTTTACTGACCTAA
rs4414449 86368959 rs4577031
HG00096 >hsa-mir-1255b-2 AACCACTTTCTTTGCTCATCCG
AAACCACTTTCTTTGCTCATCC CTTTCTTTGCTCATCCGTAAGG
rs79639536
HG00096 >hsa-mir-516b-2 AAGAAAGTGCTTCCTTTCAGAG
AAAGAAAGTGCTTCCTTTCAGA AAGAAAAGAAAGTGCTTCCTTT
AAAGTGCTTCCTTTCAGAGGGT 54228742 rs10670323

HG00096 >hsa-mir-520cAAGAAAGTGCTTCCTTTGAGAG AGTGCTTCCTTTGAGAGGGTTA
GTGCTTCCTTTGAGAGGGTTAC TTTGAGAGGGTTACCGTTTGAG 54210774
.
HG00096 >hsa-mir-423 AAGCTCGGTCTGAGGCCCCTCA AGGCCCCTCAGTCTTGCTTCCT

TCTGAGGCCCCTCAGTCTTGCT GTCTGAGGCCCCTCAGTCTTGC 28444183 rs6505162

HG00096 >hsa-mir-3910-1 AAGGCATAAAACCAAGACAAGC CATAAAACCAAGACAAGCAACA GGCATAAAACCAAGACAAGCAA GCATAAAACCAAGACAAGCAAC 94398581 rs67339585

HG00096 >hsa-mir-622 ACACAGTCTGCTGAGGTTGGAG CTGCTGAGGTTGGAGCCGCTGA ACACAGTCTGCTGAGGTTGGAG rs111371406

HG00096 >hsa-mir-449cACAGTTGCTAGTTGCACTCCTC AACAGTTGCTAGTTGCACTCCT GTTGCTAGTTGCACTCCTCTCT GTTGCACTCCTCTCTGTTGCAT 54468166 rs75661995

HG00096 >hsa-mir-3118-1 ACTGCATTATGAAAATTCTTCT ATTATGAAAATTCTTCTAGTGT GCATTATGAAAATTCTTCTAGT CTGCATTATGAAAATTCTTCTA 142667330 rs76132421

HG00096 >hsa-mir-642bAGATACATTTGGAGAGGGACCC TTGGAGAGGGACCCTCCCAACT TTTGGAGAGGGACCCTCCCAAC ATACATTTGGAGAGGGACCCTC 46178217 rs111664333

HG00096 >hsa-mir-3180-4 AGCGGAGGGTGAAGCCTCCGGA CGGAGGGTGAAGCCTCCGGATG GAGCGGAGGGTGAAGCCTCCGG GCGGAGGGTGAAGCCTCCGGAT 15248798 rs183853838

HG00096 >hsa-mir-3180-4 AGCGGAGGGTGAAGCCTCCGGA CGGAGGGTGAAGCCTCCGGATG GAGCGGAGGGTGAAGCCTCCGG GCGGAGGGTGAAGCCTCCGGAT 15248720 rs75000738 15248798 rs183853838

HG00096 >hsa-mir-30d AGCTTTCAGTCAGATGTTTGCT GGCTAAGCTTTCAGTCAGATGT GCTAAGCTTTCAGTCAGATGTT TTCAGTCAGATGTTTGCTGCTA 135817150 .

HG00096 >hsa-mir-519a-2 AGGAAAGTGCATCCTTTTAGAG AGTGCATCCTTTTAGAGGGTTA GGAAAGTGCATCCTTTTAGAGG GAAAGGAAAGTGCATCCTTTTA 54265670 .

HG00096 >hsa-mir-630 AGTATTCTGTACCAGGGAAGGT ACCTAGTATTCTGTACCAGGGA CCAGGGAAGGTAGTTCTTAACT CAGGGAAGGTAGTTCTTAACTA 72879653 rs113971639

HG00096 >hsa-mir-513cATAAATTTCACCTTTCTGAGAA TTTCTGAGAAGAGTAATGTACA CCTTTCTGAGAAGAGTAATGTA TTTCACCTTTCTGAGAAGAGTA 146271303 rs145416750

HG00096 >hsa-mir-323bATACACGGTCGACCTCTTTTCG TACACGGTCGACCTCTTTTCGG ACACGGTCGACCTCTTTTCGGT rs56103835

HG00096 >hsa-mir-642aATTTGGAGAGGGAACCTCCCAA AGACACATTTGGAGAGGGAACC ACACATTTGGAGAGGGAACCTC CACATTTGGAGAGGGAACCTCC 46178217 rs111664333

HG00096 >hsa-mir-412 CACCTGGTCCACTGGCCGTCCG ACCTGGTCCACTGGCCGTCCGT

CTGGCCGTCCGTATCCGCTGCA TCACCTGGTCCACTGGCCGTCC 101531854 rs61992671
HG00096 >hsa-mir-515-1 CAGAGTGCCTTCTTTTGGAGCA GAGTGCCTTCTTTTGGAGCATT TGCCTTCTTTTGGAGCATTACT GTGCCTTCTTTTGGAGCATTAC 54182326 rs374576826

HG00096 >hsa-mir-4265CAGCTGTGGGCTCAACTCTGGG ATCTCTGCAGCTGTGGGCTCAA GATCTCTGCAGCTGTGGGCTCA CTGCAGCTGTGGGCTCAACTCT 109757963 rs4676066
HG00096 >hsa-mir-1227CATTTGACCCCGTGCCACCCTT ATTTGACCCCGTGCCACCCTTT AGGCATTTGACCCCGTGCCACC GACCCCGTGCCACCCTTTTCCC 2234093 rs190788838
HG00096 >hsa-mir-3151CCACCTGATCCCACACCCCACC CACCTGATCCCACACCCCACCT CCCACCTGATCCCACACCCCAC TGATCCCACACCCCACCTGTCA 104166902 rs35605502
HG00096 >hsa-mir-4268CCTCTCAGGATGTGATGTCACC CTCCTCTCAGGATGTGATGTCA TCCTCTCAGGATGTGATGTCAC rs4674470

HG00096 >hsa-mir-4254CCTGGAGATACTCCACCATCTC AGATACTCCACCATCTCCCCCA GGAGATACTCCACCATCTCCCC rs12731294

HG00096 >hsa-mir-3152CCTGTGTTAGAATAAGGGCAAT TTAGAATAAGGGCAATAACTCT AGAATAAGGGCAATAACTCTGC TGTGTTAGAATAAGGGCAATAA 18573360 rs13299349
HG00096 >hsa-mir-3117CTCATATAGTGCCAGGTGTTTT GACTCATATAGTGCCAGGTGTT TCATATAGTGCCAGGTGTTTTG ATAAGACTCATATAGTGCCAGG 67094171 rs12402181
HG00096 >hsa-mir-486-2 CTCGGCGCAGCTCAGTACAGGA AGGGCCTCGGCGCAGCTCAGTA TCGGCGCAGCTCAGTACAGGAT GGGCCTCGGCGCAGCTCAGTAC 41518007 .

HG00096 >hsa-mir-3615CTCTCTCGGCTCCTCGCGGCTC GGCTCCTCGCGGCTCGCGGCGG CGGCTCCTCGCGGCTCGCGGCG TCGGCTCCTCGCGGCTCGCGGC 72744798 rs745666
HG00096 >hsa-mir-888 CTCTTTGGGTGAAGGAAGGCTC CTGACACCTCTTTGGGTGAAGG GACTGACACCTCTTTGGGTGAA CCTCTTTGGGTGAAGGAAGGCT 145076355 rs143634721
HG00096 >hsa-mir-940 GAAGGCAGGGCCCC-GCTCCCC G CCC-GCTCCCCGGGCCTGACCC rs35356504

HG00096 >hsa-mir-4274GACCCAGCAGTCCCTCCCCCTG CCCAGCAGTCCCTCCCCCTGCA TGACCCAGCAGTCCCTCCCCCT TCAGGTGACCCAGCAGTCCCTC 7461769 rs12512664
HG00096 >hsa-mir-629 GAGGTTCTCCCAACGTAAGCCC AGGTTCTCCCAACGTAAGCCCA TCTCCCAACGTAAGCCCAGCCC CAGGAGGTTCTCCCAACGTAAG 70371761 rs377691713
HG00096 >hsa-mir-3180-4 GCGGAGGGTGAAGCCTCCGGAT

CGCTGGCCTGGTCGCGCTGTGG TCGCTGGCCTGGTCGCGCTGTG
AAGCCTCCGGATGCCAGTCCCT 15248720 rs75000738

HG00096 >hsa-mir-149 GGGAGGGAGGGACGGGGGCTGT GGAGGGACGGGGGCTGTGCTGG
AGGGACGGGGGCTGTGCTGGGG GAGGAGGGAGGGAGGGACGGGG 241395503
rs2292832
HG00096 >hsa-mir-1197GTAGGACACATGGTCTACTTCT ACACATGGTCTACTTCTTCTCA
ACATGGTCTACTTCTTCTCAAT TAGGACACATGGTCTACTTCTT 101491923
rs141611518
HG00096 >hsa-mir-549aGTGACAACTATGGATGAACTCT ACAACTATGGATGAACTCTTAA
ACTATGGATGAACTCTTAATAT TATGGATGAACTCTTAATATAT 81134338
.
HG00096 >hsa-mir-27a GTGTTCACAGTGGCTAAGTTCC TCGTGTTCACAGTGGCTAAGTT
AGTGGCTAAGTTCCGCCCCCCA CACAGTGGCTAAGTTCCGCCCC 13947292
rs895819
HG00096 >hsa-mir-27a GTGTTCACAGTGGCTAAGTTCC CGTGTTCACAGTGGCTAAGTTC
TGTTCACAGTGGCTAAGTTCCG GTCGTGTTCACAGTGGCTAAGT 13947296
rs11671784
HG00096 >hsa-mir-27a GTGTTCACAGTGGCTAAGTTCC CGTGTTCACAGTGGCTAAGTTC
TGTTCACAGTGGCTAAGTTCCG GTCGTGTTCACAGTGGCTAAGT 13947292
rs895819 13947296 rs11671784
HG00096 >hsa-mir-3910-2 TAAAAGGCATAAAACCAAGACA
TAAAACCAAGACAAGCAACAGA AGGCATAAAACCAAGACAAGCA
GGCATAAAACCAAGACAAGCAA 94398581 rs67339585

HG00096 >hsa-mir-3936TAAGGGGTGTATGGCAGATGCA TTCTGGTAAGGGGTGTATGGCA
CACCCGACAGATGCACTTGGCA TGTATGGCAGATGCACCCGACA 131701279
rs367805
HG00096 >hsa-mir-300 TATACAAGGGCAGACTCTCTCT TGATTATACAAGGGCAGACTCT
ATTATACAAGGGCAGACTCTCT rs12894467

HG00096 >hsa-mir-499aTCACAGCAAGTCTGTGCTGCTT ACAGCAAGTCTGTGCTGCTTCC
CGTCACAGCAAGTCTGTGCTGC TCCCTACGCTGCCTGGGCAGGG 33578251
rs3746444
HG00096 >hsa-mir-4308TCTTTCCCTGGAGTTTCTTCTT AATCTTTCCCTGGAGTTTCTTC
GAAATCTTTCCCTGGAGTTTCT CCTGGAGTTTCTTCTTACCTTT 55344901
rs28477407
HG00096 >hsa-mir-570 TGCGAAAACAGCAATTACCTTT GAAAACAGCAATTACCTTTGCA
ACAGCAATTACCTTTGCACCAA AAACAGCAATTACCTTTGCACC 195426305
rs9860655
HG00096 >hsa-mir-1273h TGCTGCAGACTCGACCTCCCAG
TGCAGACTCGACCTCCCAGGCT CTGCAGACTCGACCTCCCAGGC
AGACTCGACCTCCCAGGCTTAA 24214486 .

HG00096 >hsa-mir-3156-2 TGGCCCCCACTTCCAGATCTTT
CCCCCACTTCCAGATCTTTCTC ACTTCCAGATCTTTCTCTCTGT
CCCCACTTCCAGATCTTTCTCT 14830215 rs113478966

HG00096 >hsa-mir-345 TGGGCCCTGAACGAGGGGTCTG GTGGGCCCTGAACGAGGGGTCT
GCCCTGAACGAGGGGTCTGGAG GGCCCTGAACGAGGGGTCTGGA 100774203
rs72631832
HG00096 >hsa-mir-590 TGTAATTTTATGTATAAGCTAG AATCTGTAATTTTATGTATAAG
GTATAAGCTAGTCTCTGATTGA TTTTATGTATAAGCTAGTCTCT 73605546
rs189727189
HG00096 >hsa-mir-2053TGTTAATTAAACCTCTATTTAC ACTTTAAGTGTTAATTAAACCT
TTTAAGTGTTAATTAAACCTCT GTTAATTAAACCTCTATTTACA 113655752
rs10505168
HG00096 >hsa-mir-585 TTGGGCGTATCTGTATGCTAGG TATCTGTATGCTAGGGCTGCCG
TGGGCGTATCTGTATGCTAGGG GCGTATCTGTATGCTAGGGCTG 168690612
rs62376934
HG00096 >hsa-mir-1303TTTAGAGACGGGGTCTTGCTCT TAGAGACGGGGTCTTGCTCTGT
ACGGGGTCTTGCTCTGTTGCCA GGGTCTTGCTCTGTTGCCAGGC 154065347
rs142414368
HG00096 >hsa-mir-1303TTTAGAGACGGGGTCTTGCTCT TAGAGACGGGGTCTTGCTCTGT
TTAGAGACGGGGTCTTGCTCTG ACGGGGTCTTGCTCTGTTGCCA 154065383
rs75538180
HG00096 >hsa-mir-1303TTTAGAGACGGGGTCTTGCTCT TAGAGACGGGGTCTTGCTCTGT
TTAGAGACGGGGTCTTGCTCTG ACGGGGTCTTGCTCTGTTGCCA 154065347
rs142414368 154065383 rs75538180
HG00096 >hsa-mir-580 TTTGAGAATGATGAATCATTAG GATGAATCATTAGGTTCCGGTC
AATGATGAATCATTAGGTTCCG AGAATGATGAATCATTAGGTTC 36148057
rs115089112
HG00099 >hsa-mir-1273h CCTGGGAGGTCAAGGCTGTAGT
TGGGAGGTCAAGGCTGTAGTGT GCCTGGGAGGTCAAGGCTGTAG
ATTGCTTGAGCCTGGGAGGTCA TTGAGCCTGGGAGGTCAAGGCT 24214486 .

HG00099 >hsa-mir-1908CCGCGGCGGGGACGGCGATTGG GCGGCGGGGACGGCGATTGGTC
CGCGGCGGGGACGGCGATTGGT CGGGGACGGCGATTGGTCCGTA
GGGGACGGCGATTGGTCCGTAT 61582708 rs174561

HG00099 >hsa-mir-3141TCACCCGGTGAGGGCGGGTGGA CCGGTGAGGGCGGGTGGAGGAG
CGGTGAGGGCGGGTGGAGGAGG CACCCGGTGAGGGCGGGTGGAG
CCCGGTGAGGGCGGGTGGAGGA 153975576 rs936581

HG00099 >hsa-mir-3196GCGGGGCGGCAGGGGCCTCCCC GGCGGGGCGGCAGGGGCCTCCC
GGGCGGGGCGGCAGGGGCCTCC TGGGGGCGGGGCGGCAGGGGCC
GGGGCGGGGCGGCAGGGGCCTC 61870167 rs744591

HG00099 >hsa-mir-412 TGGGGTACGGGGATGGATGGTC GGATGGATGGTCGACCAGTTGG
GATGGATGGTCGACCAGTTGGA TCGACCAGTTGGAAAGTAATTG
ACGGGGATGGATGGTCGACCAG 101531854 rs61992671

HG00099 >hsa-mir-412 TGGGGTACGGGGATGGATGGTC GGATGGATGGTCGACCAGTTGG
GGGTACGGGGATGGATGGTCGA GATGGATGGTCGACCAGTTGGA

TCGACCAGTTGGAAAGTAATTG 101531849 . 101531854 rs61992671

HG00099 >hsa-mir-553 TTTTAAAACGGTGAGATTTTGT TATTTTAAAACGGTGAGATTTT
TTTTATTTTAAAACGGTGAGAT TTTAAAACGGTGAGATTTTGTT
ATTTTATTTTAAAACGGTGAGA 100746848 .

HG00099 >hsa-mir-608 GGCCAAGGTGGGCCAGGGGTGG AAGGTGGGCCAGGGGTGGTGTT
GGGGTGGTGTTGGGACAGCTGC TGGTGTTGGGACAGCTGCGTTT
GGTGGGCCAGGGGTGGTGTTGG 102734778 rs4919510

HG00099 >hsa-mir-892cTGCCCTATTCAGAAAGGTGCCA TCAGAAAGGTGCCAGTCACTTA
AGTGCCCTATTCAGAAAGGTGC GCCCTATTCAGAAAGGTGCCAG
GTGCCCTATTCAGAAAGGTGCC 145074284 .

HG00099 >hsa-mir-1273h CCTGGGAGGTCAAGGCTGTAGT
TGGGAGGTCAAGGCTGTAGTGT GCCTGGGAGGTCAAGGCTGTAG
ATTGCTTGAGCCTGGGAGGTCA TTGAGCCTGGGAGGTCAAGGCT 24214486 .

HG00099 >hsa-mir-1908CCGCGGCGGGGACGGCGATTGG GCGGCGGGGACGGCGATTGGTC
CGCGGCGGGGACGGCGATTGGT CGGGGACGGCGATTGGTCCGTA
GGGGACGGCGATTGGTCCGTAT 61582708 rs174561

HG00099 >hsa-mir-3141TCACCCGGTGAGGGCGGGTGGA CCGGTGAGGGCGGGTGGAGGAG
CGGTGAGGGCGGGTGGAGGAGG CACCCGGTGAGGGCGGGTGGAG
CCCGGTGAGGGCGGGTGGAGGA 153975576 rs936581

HG00099 >hsa-mir-3196GCGGGGCGGCAGGGGCCTCCCC GGCGGGGCGGCAGGGGCCTCCC
GGGCGGGGCGGCAGGGGCCTCC TGGGGGCGGGGCGGCAGGGGCC
GGGGCGGGGCGGCAGGGGCCTC 61870167 rs744591

HG00099 >hsa-mir-412 TGGGGTACGGGGATGGATGGTC GGATGGATGGTCGACCAGTTGG
GATGGATGGTCGACCAGTTGGA TCGACCAGTTGGAAAGTAATTG
ACGGGGATGGATGGTCGACCAG 101531854 rs61992671

HG00099 >hsa-mir-412 TGGGGTACGGGGATGGATGGTC GGATGGATGGTCGACCAGTTGG
GGGTACGGGGATGGATGGTCGA GATGGATGGTCGACCAGTTGGA
TCGACCAGTTGGAAAGTAATTG 101531849 . 101531854 rs61992671

HG00099 >hsa-mir-553 TTTTAAAACGGTGAGATTTTGT TATTTTAAAACGGTGAGATTTT
TTTTATTTTAAAACGGTGAGAT TTTAAAACGGTGAGATTTTGTT
ATTTTATTTTAAAACGGTGAGA 100746848 .

HG00099 >hsa-mir-608 GGCCAAGGTGGGCCAGGGGTGG AAGGTGGGCCAGGGGTGGTGTT
GGGGTGGTGTTGGGACAGCTGC TGGTGTTGGGACAGCTGCGTTT
GGTGGGCCAGGGGTGGTGTTGG 102734778 rs4919510

HG00099 >hsa-mir-892cTGCCCTATTCAGAAAGGTGCCA TCAGAAAGGTGCCAGTCACTTA

AGTGCCCTATTCAGAAAGGTGC GCCCTATTCAGAAAGGTGCCAG
GTGCCCTATTCAGAAAGGTGCC 145074284 .

HG00099 >hsa-mir-1294ACAACAGTGCCAACCTCACGGG ACAGTGCCAACCTCACGGGACT
AACAGTGCCAACCTCACGGGAC TACAACAGTGCCAACCTCACGG
AGTGCCAACCTCACGGGACTCA 153726769 rs13186787

HG00099 >hsa-mir-646 AGCAGCTGCCTCTGAGGCCTCA CTGAGGCCTCAGGCTCAGTGGC
CTCTGAGGCCTCAGGCTCAGTG GCCTCTGAGGCCTCAGGCTCAG
TCTGAGGCCTCAGGCTCAGTGG 58883534 rs6513496

HG00099 >hsa-mir-149 AGGGAGGGAGGGACGGGGGCTG GGGCTGTGCTGGGGCAGCCGGA
GAGGGAGGGAGGGACGGGGGCT GGGACGGGGGCTGTGCTGGGGC
GGGAGGGAGGGACGGGGGCTGT 241395503 rs2292832

HG00099 >hsa-mir-658 AGGTCGGTTGGTCGGTCGGGAA GTCGGTTGGTCGGTCGGGAACG
TAGGTCGGTTGGTCGGTCGGGA . .

HG00099 >hsa-mir-604 CACAGGCTGCGGAATTCAGGAC ACACAGGCTGCGGAATTCAGGA
ACAGGCTGCGGAATTCAGGACA GGCTGCGGAATTCAGGACAGTG
CTGCGGAATTCAGGACAGTGCA 29833998 rs2368393

HG00099 >hsa-mir-604 CACAGGCTGCGGAATTCAGGAC ACACAGGCTGCGGAATTCAGGA
ACAGGCTGCGGAATTCAGGACA GGCTGCGGAATTCAGGACAGTG
CTGCGGAATTCAGGACAGTGCA 29833998 rs2368393 29834003
rs2368392
HG00099 >hsa-mir-412 CGTCCGTATCCGCTGCAG CCGTCCGTATCCGCTGCAG
TCACCTGGTCCACTGGCCGTCC ACCTGGTCCACTGGCCGTCCGT
CACCTGGTCCACTGGCCGTCCG 101531854 rs61992671

HG00099 >hsa-mir-412 CGTCCGTATCCGCTGCAG CCGTCCGTATCCGCTGCAG
TCACCTGGTTCACTGGCCGTCC ACCTGGTTCACTGGCCGTCCGT
CACCTGGTTCACTGGCCGTCCG 101531849 . 101531854 rs61992671

HG00099 >hsa-mir-3180-4 CTCCGGATGCCAGTCCCTCATC
GGAGGGTGAAGCCTCCGGATGC AGCGGAGGGTGAAGCCTCCGGA
CTGGCCTGGTCGCGCTGTGGCT GAGCGGAGGGTGAAGCCTCCGG 15248720
rs75000738
HG00099 >hsa-mir-4268CTCTCAGGATGTGATGTCACCT CCTCTCAGGATGTGATGTCACC
GCTCCTCCTCTCAGGATGTGAT TCCTCTCAGGATGTGATGTCAC
CTCCTCCTCTCAGGATGTGATG 220771223 rs4674470

HG00099 >hsa-mir-3151CTGATCCCACACCCCACCTGTC TGATCCCACACCCCACCTGTCA
GATCCCACACCCCACCTGTCAC GGGCATCCCACCTGATCCCACA
TCCCACCTGATCCCACACCCCA 104166902 rs35605502

HG00099 >hsa-mir-412 CTTCACCTGGTTCACTAGCCGT ACCTGGTTCACTAGCCGTCCGT

TGTACTTCACCTGGTTCACTAG CTGGTTCACTAGCCGTCCGTAT
GTACTTCACCTGGTTCACTAGC 101531849 .

HG00099 >hsa-mir-320eGAAAAGCTGGGTTGAGAAGGT AAAAGCTGGGTTGAGAAGGT
GGAAAAGCTGGGTTGAGAAGGT GGGAAAAGCTGGGTTGAGAAGG
rs10423365
HG00099 >hsa-mir-1343GCCCCTCCTGGGGCCCGCACTC CCCCTCCTGGGGCCCGCACTCT
GGGGCCCGCACTCTCGCTCTGG CCCTCCTGGGGCCCGCACTCTC
TGGGGCCCGCACTCTCGCTCTG 34963416 rs2986407

HG00099 >hsa-mir-3180-4 GGAGGGTGAAGCCTCCGGATGC
GGTGAAGCCTCCGGATGCCAGT GCGGAGGGTGAAGCCTCCGGAT
AGCGGAGGGTGAAGCCTCCGGA GCCTGGTCGCGCTGTGGCGAAG 15248798
rs183853838
HG00099 >hsa-mir-3180-4 GGAGGGTGAAGCCTCCGGATGC
GGTGAAGCCTCCGGATGCCAGT GCGGAGGGTGAAGCCTCCGGAT
AGCGGAGGGTGAAGCCTCCGGA CTGGCCTGGTCGCGCTGTGGCT 15248720
rs75000738 15248798 rs183853838
HG00099 >hsa-mir-1227GGCATTTGACCCCGTGCCACCC AGGCATTTGACCCCGTGCCACC
AGGCATTTGACCCCGTGCCACC TGACCCCGTGCCACCCTTTTCC
ATTTGACCCCGTGCCACCCTTT 2234093 rs190788838

HG00099 >hsa-mir-580 TATTTGAGAATGATGAATCATT TGAATCATTAGGTTCCGGTCAG
ATGAATCATTAGGTTCCGGTCA TTTGAGAATGATGAATCATTAG
GAGAATGATGAATCATTAGGTT 36148057 rs115089112

HG00099 >hsa-mir-3118-1 TGAAAATTCTTCTAGTGTG ATGAAAATTCTTCTAGTGTG
TGCATTATGAAAATTCTTCTAG TTATGAAAATTCTTCTAGTGTG
ATTATGAAAATTCTTCTAGTGT 142667330 rs76132421

HG00099 >hsa-mir-637 TGGCTAAGGTGTTGGCTCGGGC TGGCTAAGGTGTTGGCTCGGGC
.
HG00099 >hsa-mir-320c-1 AAAAGCTGGGTTGAGAGGGTAG
AGCTGGGTTGAGAGGGTAGGAA CTGGGTTGAGAGGGTAGGAAAA
AGGGTAGGAAAAAAATGATGTA 2234093 rs190788838

HG00099 >hsa-mir-499bAAACATCACTGCAAGTCTTAAC rs3746444
79107017 .
HG00099 >hsa-mir-202 AAAGAGGTATAGGGCATGGGAA AAGAGGTATAGGGCATGGGAAA
GGGAAAACGGGGCGGTCGGGTC TAAAGAGGTATAGGGCATGGGA

HG00099 >hsa-mir-520hAAAGTGCTTCCCTTTAGAGTTA rs148716001

HG00099 >hsa-mir-548ap AACAAAAACCACAATTACTTTT
CAAAAACCACAATTACTTTTTA CAATTACTTTTTACTGACCTAA
rs4414449 24214486 .
HG00099 >hsa-mir-548ap AACAAAAACCACAATTACTTTT

CAAAAACCACAATTACTTTTTA TTACTTTTTACTGACCTAAAGA
rs4577031 153726769 rs13186787
HG00099 >hsa-mir-548ap AACAAAAACCACAATTACTTTT
CAAAAACCACAATTACTTTTTA CAATTACTTTTTACTGACCTAA
rs4414449 154065347 rs142414368
HG00099 >hsa-mir-1294AACAGTGCCAACCTCACGGGAC TATACAACAGTGCCAACCTCAC
ATACAACAGTGCCAACCTCACG TACAACAGTGCCAACCTCACGG
154065383 rs75538180
HG00099 >hsa-mir-1255b-2 AACCACTTTCTTTGCTCATCCG
AAACCACTTTCTTTGCTCATCC CTTTCTTTGCTCATCCGTAAGG
rs79639536 154065347 rs142414368 154065383 rs75538180

HG00099 >hsa-mir-423 AAGCTCGGTCTGAGGCCCCTCA AGGCCCCTCAGTCTTGCTTCCT
TCTGAGGCCCCTCAGTCTTGCT GTCTGAGGCCCCTCAGTCTTGC 93466866
rs2155248
HG00099 >hsa-mir-597 ACAGTGGTTCTCTTGTGGCTTA GGCTTAAGCGTAATGTAGAGTA
AATGTACAGTGGTTCTCTTGTG TGTACAGTGGTTCTCTTGTGGC 52013832
rs374103744
HG00099 >hsa-mir-3118-1 ACTGCATTATGAAAATTCTTCT
ATTATGAAAATTCTTCTAGTGT GCATTATGAAAATTCTTCTAGT
CTGCATTATGAAAATTCTTCTA 34963416 rs2986407

HG00099 >hsa-mir-3166AGACAATGCCTACTGGCCTAAG AACGCAGACAATGCCTACTGGC
ATGCCTACTGGCCTAAGAAAAA CAATGCCTACTGGCCTAAGAAA 241395503
rs2292832
HG00099 >hsa-mir-642bAGATACATTTGGAGAGGGACCC TTGGAGAGGGACCCTCCCAACT
TTTGGAGAGGGACCCTCCCAAC ATACATTTGGAGAGGGACCCTC

HG00099 >hsa-mir-3180-4 AGCGGAGGGTGAAGCCTCCGGA
CGGAGGGTGAAGCCTCCGGATG GAGCGGAGGGTGAAGCCTCCGG
GCGGAGGGTGAAGCCTCCGGAT 61582708 rs174561

HG00099 >hsa-mir-3180-4 AGCGGAGGGTGAAGCCTCCGGA
CGGAGGGTGAAGCCTCCGGATG GAGCGGAGGGTGAAGCCTCCGG
GCGGAGGGTGAAGCCTCCGGAT 54385599 rs11614913

HG00099 >hsa-mir-30d AGCTTTCAGTCAGATGTTTGCT GGCTAAGCTTTCAGTCAGATGT
GCTAAGCTTTCAGTCAGATGTT TTCAGTCAGATGTTTGCTGCTA 135061112
rs12355840
HG00099 >hsa-mir-519a-2 AGGAAAGTGCATCCTTTTAGAG
AGTGCATCCTTTTAGAGGGTTA GGAAAGTGCATCCTTTTAGAGG
GAAAGGAAAGTGCATCCTTTTA 113655752 rs10505168

HG00099 >hsa-mir-646 AGGAAGCAGCTGCCTCTGAGGC GCTGCCTCTGAGGCCTCAGGCT
CTGAGGCCTCAGGCTCAGTGGC TCTGAGGCCTCAGGCTCAGTGG 41522213
rs7207008
HG00099 >hsa-mir-1250AGGCCACATTTTCCAGCCCATT CTTCCAGAACCCTCTGAAGTGG

CCATTCAACCTTCCAGAACCCT AGCCCATTCAACCTTCCAGAAC

HG00099 >hsa-mir-630 AGTATTCTGTACCAGGGAAGGT ACCTAGTATTCTGTACCAGGGA CCAGGGAAGGTAGTTCTTAACT CAGGGAAGGTAGTTCTTAACTA 45606504 rs191727254

HG00099 >hsa-mir-3686AGTGATCTGTAAGAGAAAGTAA TCTGTAAGAGAAAGTAAATGAA GTAAGAGAAAGTAAATGAAAGA ACAGTGATCTGTAAGAGAAAGT 98510847 rs74904371

HG00099 >hsa-mir-513cATAAATTTCACCTTTCTGAGAA TTTCTGAGAAGAGTAATGTACA CCTTTCTGAGAAGAGTAATGTA TTTCACCTTTCTGAGAAGAGTA 135817150 .

HG00099 >hsa-mir-642aATTTGGAGAGGGAACCTCCCAA AGACACATTTGGAGAGGGAACC ACACATTTGGAGAGGGAACCTC CACATTTGGAGAGGGAACCTCC 67094171 rs12402181

HG00099 >hsa-mir-604 CACAGGCTGCGGAATTCAGGAC TGACACAGGCTGCGGAATTCAG GACACAGGCTGCGGAATTCAGG TTTCTGACACAGGCTGCGGAAT 142667330 rs76132421

HG00099 >hsa-mir-412 CACCTGGTCCACTGGCCGTCCG ACCTGGTCCACTGGCCGTCCGT CTGGCCGTCCGTATCCGCTGCA TCACCTGGTCCACTGGCCGTCC 207647981 rs2241347

HG00099 >hsa-mir-412 CACCTGGTTCACTGGCCGTCCG ACCTGGTTCACTGGCCGTCCGT CTGGCCGTCCGTATCCGCTGCA TCACCTGGTTCACTGGCCGTCC 207647981 rs2241347

HG00099 >hsa-mir-515-1 CAGAGTGCCTTCTTTTGGAGCA GAGTGCCTTCTTTTGGAGCATT TGCCTTCTTTTGGAGCATTACT GTGCCTTCTTTTGGAGCATTAC 104166902 rs35605502

HG00099 >hsa-mir-1227CATTTGACCCCGTGCCACCCTT ATTTGACCCCGTGCCACCCTTT AGGCATTTGACCCCGTGCCACC GACCCCGTGCCACCCTTTTCCC 18573360 rs13299349

HG00099 >hsa-mir-1908CCACCGGCCGCCGGCTCCGCCC CCGCCGGCTCCGCCCCGGCCCC GGCCGCCGGCTCCGCCCCGGCC CGGCCGCCGGCTCCGCCCCGGC 14830215 rs113478966

HG00099 >hsa-mir-3151CCACCTGATCCCACACCCCACC CACCTGATCCCACACCCCACCT CCCACCTGATCCCACACCCCAC TGATCCCACACCCCACCTGTCA 87909701 .

HG00099 >hsa-mir-1343CCCCTCCTGGGGCCCGCACTCT CCCTCCTGGGGCCCGCACTCTC CCTGGGGCCCGCACTCTCGCTC TGGGGCCCGCACTCTCGCTCTG 15248720 rs75000738

HG00099 >hsa-mir-3679CCCTTCCCCCCAGTAATCTTCA CCTTCCCCCCAGTAATCTTCAT CTTCCCCCCAGTAATCTTCATC TCCCCCCAGTAATCTTCATCAT 15248798 rs183853838

HG00099 >hsa-mir-4268CCTCTCAGGATGTGATGTCACC CTCCTCTCAGGATGTGATGTCA TCCTCTCAGGATGTGATGTCAC rs4674470 15248720 rs75000738 15248798 rs183853838

HG00099 >hsa-mir-3152CCTGTGTTAGAATAAGGGCAAT TTAGAATAAGGGCAATAACTCT AGAATAAGGGCAATAACTCTGC TGTGTTAGAATAAGGGCAATAA

HG00099 >hsa-mir-3117CTCATATAGTGCCAGGTGTTTT GACTCATATAGTGCCAGGTGTT
TCATATAGTGCCAGGTGTTTTG ATAAGACTCATATAGTGCCAGG

HG00099 >hsa-mir-196a-2 CTCGGCAACAAGAAACTGTCTG
CAAGAAACTGTCTGAGTTACAT CAACAAGAAACTGTCTGAGTTA
ACAAGAAACTGTCTGAGTTACA 18392913 rs7247767

HG00099 >hsa-mir-486-2 CTCGGCGCAGCTCAGTACAGGA
AGGGCCTCGGCGCAGCTCAGTA TCGGCGCAGCTCAGTACAGGAT
GGGCCTCGGCGCAGCTCAGTAC 19263542 .

HG00099 >hsa-mir-3615CTCTCTCGGCTCCTCGCGGCTC GGCTCCTCGCGGCTCGCGGCGG
CGGCTCCTCGCGGCTCGCGGCG TCGGCTCCTCGCGGCTCGCGGC

HG00099 >hsa-mir-888 CTCTTTGGGTGAAGGAAGGCTC CTGACACCTCTTTGGGTGAAGG
GACTGACACCTCTTTGGGTGAA CCTCTTTGGGTGAAGGAAGGCT 100774203
rs72631832
HG00099 >hsa-mir-1269a CTGGACTGAGCCATGCTACTGG
TGCCTGGACTGAGCCATGCTAC AATGCCTGGACTGAGCCATGCT
rs73239138 72744798 rs745666
HG00099 >hsa-mir-1304CTGTAGCATCGAACCCCTGGGC CTCACTGTAGCATCGAACCCCT
GAACCCCTGGGCTCAAGTGATT CGAACCCCTGGGCTCAAGTGAT 134884700
rs6430498
HG00099 >hsa-mir-2117CTGTTCTCTTTGCCAAGGACAG GCTGTTCTCTTTGCCAAGGACA
TCTCTTTGCCAAGGACAGATCT TGTTCTCTTTGCCAAGGACAGA 130496365
rs6997249
HG00099 >hsa-mir-940 GAAGGCAGGGCCCC-GCTCCCC G CCC-
GCTCCCCGGGCCTGACCC rs35356504 35731712 rs34874675

HG00099 >hsa-mir-4274GACCCAGCAGTCCCTCCCCCTG CCCAGCAGTCCCTCCCCCTGCA
TGACCCAGCAGTCCCTCCCCCT TCAGGTGACCCAGCAGTCCCTC 131701279
rs367805
HG00099 >hsa-mir-3188GAGGCTTTGTGCGGATACGGGG GAGAGGCTTTGTGCGGATACGG
GCGGATACGGGGCTGGAGGCCT rs7247237 101531849
.
HG00099 >hsa-mir-3188GAGGCTTTGTGCGGATACGGGG GGAGAGGCTTTGTGCGGATACG
GAGAGGCTTTGTGCGGATACGG rs7247767 101531854
rs61992671
HG00099 >hsa-mir-3188GAGGCTTTGTGCGGATACGGGG GAGAGGCTTTGTGCGGATACGG
GGAGAGGCTTTGTGCGGATACG rs7247237 101531849
. 101531854 rs61992671
HG00099 >hsa-mir-629 GAGGTTCTCCCAACGTAAGCCC AGGTTCTCCCAACGTAAGCCCA
TCTCCCAACGTAAGCCCAGCCC CAGGAGGTTCTCCCAACGTAAG 28444183
rs6505162
HG00099 >hsa-mir-3130-1 GCACCAGAGACTGGGTAAGACA
GAGACTGGGTAAGACATGACAA CCAGAGACTGGGTAAGACATGA

TGCACCAGAGACTGGGTAAGAC

HG00099 >hsa-mir-3130-2 GCACCGGAGACTGGGTAAGACA CTGCACCGGAGACTGGGTAAGA CCGGAGACTGGGTAAGACATGA TGCACCGGAGACTGGGTAAGAC 7461769 rs12512664

HG00099 >hsa-mir-222 GCAGCTACATCTGGCTACTGGG TACTGGGTCTCTGATGGCATCT GCTACTGGGTCTCTGATGGCAT CTGGCTACTGGGTCTCTGATGG 41518007 .

HG00099 >hsa-mir-3180-4 GCGGAGGGTGAAGCCTCCGGAT CGCTGGCCTGGTCGCGCTGTGG TCGCTGGCCTGGTCGCGCTGTG AAGCCTCCGGATGCCAGTCCCT 33578202 rs140486571

HG00099 >hsa-mir-2682GGACACCTCTTCAGCGCTGTCT CAGCGCTGTCTTCCCTGCCTCT TTCAGCGCTGTCTTCCCTGCCT CACCTCTTCAGCGCTGTCTTCC 33578251 rs3746444

HG00099 >hsa-mir-548a-3 GGCAAAACTGGCAGTTACTTTT GCAAAACTGGCAGTTACTTTTG AAAACTGGCAGTTACTTTTGCA AACTGGCAGTTACTTTTGCACC 33578202 rs140486571 33578251 rs3746444

HG00099 >hsa-mir-149 GGGAGGGAGGGACGGGGGCTGT GGAGGGACGGGGGCTGTGCTGG AGGGACGGGGGCTGTGCTGGGG GAGGAGGGAGGGAGGGACGGGG

HG00099 >hsa-mir-1197GTAGGACACATGGTCTACTTCT ACACATGGTCTACTTCTTCTCA ACATGGTCTACTTCTTCTCAAT TAGGACACATGGTCTACTTCTT 146271303 rs145416750

HG00099 >hsa-mir-3936TAAGGGGTGTATGGCAGATGCA TTCTGGTAAGGGGTGTATGGCA CACCCGACAGATGCACTTGGCA TGTATGGCAGATGCACCCGACA 54182326 rs374576826

HG00099 >hsa-mir-633 TAATAGTATCTACCACAATAAA CTAATAGTATCTACCACAATAA TAGTATCTACCACAATAAAATT rs17759989 54265670 .

HG00099 >hsa-mir-499aTCACAGCAAGTCTGTGCTGCTT CGTCCCTACGCTGCCTGGGCAG GCTGCTTCCCGTCCCTACGCTG TCCCTACGCTGCCTGGGCAGGG

HG00099 >hsa-mir-499aTCACAGCAAGTCTGTGCTGCTT ACAGCAAGTCTGTGCTGCTTCC CGTCACAGCAAGTCTGTGCTGC TCCCTACGCTGCCTGGGCAGGG 49767832 rs456615

HG00099 >hsa-mir-499aTCACAGCAAGTCTGTGCTGCTT CGTCCCTACGCTGCCTGGGCAG GCTGCTTCCCGTCCCTACGCTG TCCCTACGCTGCCTGGGCAGGG 49767835 rs456617

HG00099 >hsa-mir-412 TCACCTGGTTCACTAGCCGTCC TCACCTGGTTCACTAGCCGTCC ATGTACTTCACCTGGTTCACTA CTTCACCTGGTTCACTAGCCGT 49767832 rs456615 49767835 rs456617

HG00099 >hsa-mir-532 TCCCACACCCAAGGCTTGCAGA CTCCCACACCCAAGGCTTGCAG CCTCCCACACCCAAGGCTTGCA CACCCAAGGCTTGCAGAAGAGC 105496622 .

HG00099 >hsa-mir-532 TCCCACACCCAAGGCTTGCAGA CTCCCACACCCAAGGCTTGCAG
CCTCCCACACCCAAGGCTTGCA CACCCAAGGCTTGCAGAAGAGC

HG00099 >hsa-mir-532 TCCCACACCCAAGGCTTGCAGA CTCCCACACCCAAGGCTTGCAG
CCTCCCACACCCAAGGCTTGCA CACCCAAGGCTTGCAGAAGAGC

HG00099 >hsa-mir-892cTCCTTTCTAAGTGGAGTAGGGC CTTTCTAAGTGGAGTAGGGCTT
ACTGTTTCCTTTCTAAGTGGAG TTCCTTTCTAAGTGGAGTAGGG 86368959
rs4577031
HG00099 >hsa-mir-604 TGACACAGGCTGCGGAATTCAG CACAGGCTGCGGAATTCAGGAC
GACACAGGCTGCGGAATTCAGG TTTCTGACACAGGCTGCGGAAT

HG00099 >hsa-mir-604 TGACACAGGCTGCGGAATTCAG GACACAGGCTGCGGAATTCAGG
CACAGGCTGCGGAATTCAGGAC TTTCTGACACAGGCTGCGGAAT 36148057
rs115089112
HG00099 >hsa-mir-1273h TGCTGCAGACTCGACCTCCCAG
TGCAGACTCGACCTCCCAGGCT CTGCAGACTCGACCTCCCAGGC
AGACTCGACCTCCCAGGCTTAA 168690612 rs62376934

HG00099 >hsa-mir-3156-2 TGGCCCCCACTTCCAGATCTTT
CCCCCACTTCCAGATCTTTCTC ACTTCCAGATCTTTCTCTCTGT
CCCCACTTCCAGATCTTTCTCT 168690635 rs62376935

HG00099 >hsa-mir-345 TGGGCCCTGAACGAGGGGTCTG GTGGGCCCTGAACGAGGGGTCT
GCCCTGAACGAGGGGTCTGGAG GGCCCTGAACGAGGGGTCTGGA 168690612
rs62376934 168690635 rs62376935
HG00099 >hsa-mir-3909TGTCCTCTA-GGCCTGCAGTCT TA-GGCCTGCAGTCTCATGGGA
GGCCTGCAGTCTCATGGGAGA CCTGCAGTCTCATGGGAGAGTG 9599255
rs146125159
HG00099 >hsa-mir-2053TGTTAATTAAACCTCTATTTAC ACTTTAAGTGTTAATTAAACCT
TTTAAGTGTTAATTAAACCTCT TTAAGTGTTAATTAAACCTCTA 29833998
rs2368393
HG00099 >hsa-mir-585 TTGGACGTATCTGTATGCTAGG ACGTATCTGTATGCTAGGGCTG
TGGACGTATCTGTATGCTAGGG TATCTGTATGCTAGGGCTGCTG 29834003
rs2368392
HG00099 >hsa-mir-585 TTGGACGTATCTGTATGCTAGG TATCTGTATGCTAGGGCTGCCG
ACGTATCTGTATGCTAGGGCTG TGGACGTATCTGTATGCTAGGG 29833998
rs2368393 29834003 rs2368392
HG00099 >hsa-mir-585 TTGGGCGTATCTGTATGCTAGG TATCTGTATGCTAGGGCTGCCG
TGGGCGTATCTGTATGCTAGGG GCGTATCTGTATGCTAGGGCTG 70371761
rs377691713
HG00099 >hsa-mir-1303TTTAGAGACGGGGTCTTGCTCT TAGAGACGGGGTCTTGCTCTGT
ACGGGGTCTTGCTCTGTTGCCA GGGTCTTGCTCTGTTGCCAGGC 72879653
rs113971639
HG00099 >hsa-mir-1303TTTAGAGACGGGGTCTTGCTCT TAGAGACGGGGTCTTGCTCTGT
TTAGAGACGGGGTCTTGCTCTG ACGGGGTCTTGCTCTGTTGCCA

HG00099	>hsa-mir-1303TTTAGAGACGGGGTCTTGCTCT	TAGAGACGGGGTCTTGCTCTGT
TTAGAGACGGGGTCTTGCTCTG	ACGGGGTCTTGCTCTGTTGCCA

HG00099	>hsa-mir-580 TTTGAGAATGATGAATCATTAG	GATGAATCATTAGGTTCCGGTC
AATGATGAATCATTAGGTTCCG	AGAATGATGAATCATTAGGTTC	46178217
rs111664333
HG00099	>hsa-mir-133bTTTGGTCCCCTTCAACCAGCTA	TGGTCCCCTTCAACCAGCTACA
CCTTCAACCAGCTACAGCAGGG	AGAGGTTTGGTCCCCTTCAACC	46178217
rs111664333
HG00100	>hsa-mir-3125AGAATGGATAGAGGAAGCTGTG	GAGGAAGCTGTGGAGAGAACTC
AGAGGAAGCTGTGGAGAGAACT	GCTGTGGAGAGAACTCACGGTG
GGAAGCTGTGGAGAGAACTCAC	12877501	rs78852835

HG00100	>hsa-mir-577 AGTGAAGAGTAGATAAAATATT	GTAGATAAAATATTGGTACCTG
GAAGAGTAGATAAAATATTGGT	AAGAGTAGATAAAATATTGGTA
AGTAGATAAAATATTGGTACCT	115577997	rs34115976

HG00100	>hsa-mir-1908CCGCGGCGGGGACGGCGATTGG	GCGGCGGGGACGGCGATTGGTC
CGCGGCGGGGACGGCGATTGGT	CGGGGACGGCGATTGGTCCGTA
GGGATGCCGCGGCGGGGACGGC	61582708	rs174561

HG00100	>hsa-mir-1273h	CCTGGGAGGTCAAGGCTGTAGT
TGGGAGGTCAAGGCTGTAGTGT	GCCTGGGAGGTCAAGGCTGTAG
ATTGCTTGAGCCTGGGAGGTCA	TTGAGCCTGGGAGGTCAAGGCT	24214486	.

HG00100	>hsa-mir-3196GCGGGGCGGCAGGGGCCTCCCC	GGCGGGGCGGCAGGGGCCTCCC
GGGCGGGGCGGCAGGGGCCTCC	TGGGGGCGGGGCGGCAGGGGCC
GGGGCGGGGCGGCAGGGGCCTC	61870167	rs744591

HG00100	>hsa-mir-4326TCTGCTGTTCCTCTGTCTCCCA	TGGTCTGCTGTTCCTCTGTCTC
CTGGTCTGCTGTTCCTCTGTCT	GCTGTTCCTCTGTCTCCCAGAC
TGCTGTTCCTCTGTCTCCCAGA	61918164	rs6062431

HG00100	>hsa-mir-3199-1	TGCCTTAGGAGAAAGTTTCTGG
CCGGGACTGCCTTAGGAGAAAG	CTTAGGAGAAAGTTTCTGGAAG
GCCTTAGGAGAAAGTTTCTGGA	TGACTCCCGGGACTGCCTTAGG	28316591
rs78805657
HG00100	>hsa-mir-3199-1	TGCCTTAGGAGAAAGTTTCTGG
CCGGGACTGCCTTAGGAGAAAG	CTTAGGAGAAAGTTTCTGGAAG
GCCTTAGGAGAAAGTTTCTGGA	TGACTCCCGGGACTGCCTTAGG	28316513
rs118160653	28316591	rs78805657
HG00100	>hsa-mir-412 TGGGGTACGGGGATGGATGGTC	GGATGGATGGTCGACCAGTTGG
GATGGATGGTCGACCAGTTGGA	TCGACCAGTTGGAAAGTAATTG
ACGGGGATGGATGGTCGACCAG	101531854	rs61992671

HG00100	>hsa-mir-412 TGGGGTACGGGGATGGATGGTC	GGATGGATGGTCGACCAGTTGG
GGGTACGGGGATGGATGGTCGA	GATGGATGGTCGACCAGTTGGA

TCGACCAGTTGGAAAGTAATTG 101531849 . 101531854 rs61992671

HG00100 >hsa-mir-553 TTTTAAAACGGTGAGATTTTGT TATTTTAAAACGGTGAGATTTT
TTTTATTTTAAAACGGTGAGAT TTTAAAACGGTGAGATTTTGTT
ATTTTATTTTAAAACGGTGAGA 100746848 .

HG00100 >hsa-mir-553 TTTTAAAACGGTGAGATTTTGT TTTTATTTTAAAACGGTGAGAT
TTTAAAACGGTGAGATTTTGTT TATTTTAAAACGGTGAGATTTT
ATTTTATTTTAAAACGGTGAGA 100746848 . 100746855
rs112891767
HG00100 >hsa-mir-553 TTTTATTTTAAAACGGTGAGAT AATTTTATTTTAAAACGGTGAG
TTTTAAAACGGTGAGATTTTGT TTATTTTAAAACGGTGAGATTT
TTTAAAACGGTGAGATTTTGTT 100746855 rs112891767

HG00100 >hsa-mir-486-2 ACTGAGCTGCCCCGAGCTGGGC
CTGAGCTGCCCCGAGCTGGGCA CTGTACTGAGCTGCCCCGAGCT
CCTGTACTGAGCTGCCCCGAGC GTACTGAGCTGCCCCGAGCTGG 41518007 .

HG00100 >hsa-mir-1269b AGCCATGCTACGGGCTTCTCTG
ACTGAGCCATGCTACGGGCTTC AGGTTTCTGGACTGAGCCATGC
TGAGGTTTCTGGACTGAGCCAT TTTCTGGACTGAGCCATGCTAC 12820632
rs12451747
HG00100 >hsa-mir-3125AGCTGTGGAGAGAACTCACGGT AAGCTGTGGAGAGAACTCACGG
TAGAGGAAGCTGTGGAGAGAAC TGGATAGAGGAAGCTGTGGAGA
AGGAAGCTGTGGAGAGAACTCA 12877501 rs78852835

HG00100 >hsa-mir-1227AGGCGGTGGTGGGCACTGCTGG TGGTGGGCACTGCTGGGGTGGG
TGGGGCCAGGCGGTGGTGGGCA CGGTGGTGGGCACTGCTGGGGT
GGTGGGCACTGCTGGGGTGGGC 2234093 rs190788838

HG00100 >hsa-mir-548ap AGTAATTGCAGTCTTTGTCATT
AAGTAATTGCAGTCTTTGTCAT AAAGTAATTGCAGTCTTTGTCA
AAAAGTAATTGCAGTCTTTGTC CAAAAGTAATTGCAGTCTTTGT 86368898
rs4414449
HG00100 >hsa-mir-548ap AGTAATTGCAGTCTTTGTCATT
AAGTAATTGCAGTCTTTGTCAT AAAGTAATTGCAGTCTTTGTCA
AAAAGTAATTGCAGTCTTTGTC CAAAAGTAATTGCAGTCTTTGT 86368898
rs4414449 86368959 rs4577031
HG00100 >hsa-mir-548aj-2 AGTAATTGCAGTTTTTGCCATT
AAGTAATTGCAGTTTTTGCCAT AAAGTAATTGCAGTTTTTGCCA
TGCAAAAGTAATTGCAGTTTTT AAAAGTAATTGCAGTTTTTGCC 37883200
rs73463468
HG00100 >hsa-mir-548ap AGTAATTGCGGTCTTTGTCATT
AAGTAATTGCGGTCTTTGTCAT AAAGTAATTGCGGTCTTTGTCA
AAAAGTAATTGCGGTCTTTGTC CAAAAGTAATTGCGGTCTTTGT 86368959
rs4577031
HG00100 >hsa-mir-196a-2 AGTTTCATGTTGTTGGGATTGA

AGGTAGTTTCATGTTGTTGGGA TAGTTTCATGTTGTTGGGATTG
GTAGTTTCATGTTGTTGGGATT GGTAGTTTCATGTTGTTGGGAT 54385599
rs11614913

HG00100 >hsa-mir-500bATCCTTGCTACCTGGGTGAGAG CTTGCTACCTGGGTGAGAGTGC
AATCCTTGCTACCTGGGTGAGA CCTTGCTACCTGGGTGAGAGTG
TCCTTGCTACCTGGGTGAGAGT 49775351 rs151318590

HG00100 >hsa-mir-1257ATGGGTTCTGACCCCCATGCAC GTTCTGACCCCCATGCACCCCT
ATGATGGGTTCTGACCCCCATG GGTTCTGACCCCCATGCACCCC
AATGATGGGTTCTGACCCCCAT 60528617 rs367783642

HG00100 >hsa-mir-1265ATGTGGTCAAGTGTTGTTAAGG AGGATGTGGTCAAGTGTTGTTA
TGTGGTCAAGTGTTGTTAAGGC ACTCAGGATGTGGTCAAGTGTT
GTGGTCAAGTGTTGTTAAGGCA 14478618 rs11259096

HG00100 >hsa-mir-650 GAGGCAGCGCTCTCAGGACGTC GGAGGCAGCGCTCTCAGGACGT
TCAGGAGGCAGCGCTCTCAGGA TCTCAGGAGGCAGCGCTCTCAG
GTCTCAGGAGGCAGCGCTCTCA 23165340 rs5996397

HG00100 >hsa-mir-1908GGGGACGGCGATTGGTCCGTAT GCGGGGACGGCGATTGGTCCGT
GGCGGGGACGGCGATTGGTCCG CGGGGACGGCGATTGGTCCGTA
TGCCGCGGCGGGGACGGCGATT 61582708 rs174561

HG00100 >hsa-mir-573 GTGTAACTGATCAGGATCTACT TGTGTAACTGATCAGGATCTAC
GATGTGTAACTGATCAGGATCT TGATGTGTAACTGATCAGGATC
GTGATGTGTAACTGATCAGGAT 24521902 rs76014664

HG00100 >hsa-mir-3117TAAAGGGCCAGACACTATACGA GGGCCAGACACTATACGAGTCA
GCCAGACACTATACGAGTCATA GGCCAGACACTATACGAGTCAT
CCCTAAAGGGCCAGACACTATA 67094171 rs12402181

HG00100 >hsa-mir-629 TACGTTGGGAGAACTTTTATGG TTACGTTGGGAGAACTTTTATG
TTTACGTTGGGAGAACTTTTAT TGGGTTTACGTTGGGAGAACTT
GTTTACGTTGGGAGAACTTTTA 70371761 rs377691713

HG00100 >hsa-mir-888 TACTCAAAAAGCTTTCAGTCAC TCTACTCAAAAAGCTTTCAGTC
TGCTCTACTCAAAAAGCTTTCA GCTCTACTCAAAAAGCTTTCAG
GGCAGTGCTCTACTCAAAAAGC 145076355 rs143634721

HG00100 >hsa-mir-888 TACTCAAAAAGCTTTCAGTCAC TCTACTCAAAAAGCTTTCAGTC
TGCTCTACTCAAAAAGCTTTCA GCTCTACTCAAAAAGCTTTCAG
GTGCTCTACTCAAAAAGCTTTC 145076302 rs5965660 145076355
rs143634721

HG00100 >hsa-mir-3199-1 TAGGAGAAAGTTTCTGGAAGTT
TTAGGAGAAAGTTTCTGGAAGT TGCCTTAGGAGAAAGTTTCTGG
TCCAGGGACTGCCTTAGGAGAA TGACTCCAGGGACTGCCTTAGG 28316513
rs118160653

HG00100 >hsa-mir-3199-1 TAGGAGAAAGTTTCTGGAAGTT
TTAGGAGAAAGTTTCTGGAAGT TGCCTTAGGAGAAAGTTTCTGG
TCCCGGGACTGCCTTAGGAGAA TGACTCCCGGGACTGCCTTAGG 28316591
rs78805657
HG00100 >hsa-mir-3199-1 TAGGAGAAAGTTTCTGGAAGTT
TTAGGAGAAAGTTTCTGGAAGT TGCCTTAGGAGAAAGTTTCTGG
TCCCGGGACTGCCTTAGGAGAA TGACTCCCGGGACTGCCTTAGG 28316513
rs118160653 28316591 rs78805657
HG00100 >hsa-mir-3199-2 TAGGAGAAAGTTTCTGGAATGT
TTAGGAGAAAGTTTCTGGAATG TGCCTTAGGAGAAAGTTTCTGG
TCCCAGGGACTGCCTTAGGAGA TGACTCCCAGGGACTGCCTTAG 28316591
rs78805657
HG00100 >hsa-mir-3199-2 TAGGAGAAAGTTTCTGGAATGT
TTAGGAGAAAGTTTCTGGAATG TGCCTTAGGAGAAAGTTTCTGG
TCCCAGGGACTGCCTTAGGAGA TGACTCCCAGGGACTGCCTTAG 28316591
rs80166589
HG00100 >hsa-mir-3199-2 TAGGAGAAAGTTTCTGGAATGT
TTAGGAGAAAGTTTCTGGAATG TGCCTTAGGAGAAAGTTTCTGG
TCCCAGGGACTGCCTTAGGAGA TGACTCCCAGGGACTGCCTTAG 28316591
rs78805657 28316591 rs80166589
HG00100 >hsa-mir-449cTAGGCAGTGTATTGCTAGCGGC TCAGATAGGCAGTGTATTGCTA
TGTCAGATAGGCAGTGTATTGC TGTGTCAGATAGGCAGTGTATT
TGGGATGTGTCAGATAGGCAGT 54468166 rs75661995

HG00100 >hsa-mir-3922TCAAGGCCAGAGGTCCCACAAC TCAAGTCAAGGCCAGAGGTCCC
GCCAGAGGTCCCACAACAGGGC GGCCAGAGGTCCCACAACAGGG
GTCAAGGCCAGAGGTCCCACAA 104985443 rs61938575

HG00100 >hsa-mir-564 TCAGCAGGCAACATGGCCGAGA TGTCAGCAGGCAACATGGCCGA
GTCAGCAGGCAACATGGCCGAG GTGTCAGCAGGCAACATGGCCG
TGCCAGGCACGGTGTCAGCAGG 44903385 rs114636202

HG00100 >hsa-mir-222 TCAGTAGCCAGTGTAGATCCTG TGGCTCAGTAGCCAGTGTAGAT
TTGGCTCAGTAGCCAGTGTAGA TCATTGGCTCAGTAGCCAGTGT
TACCCTCATTGGCTCAGTAGCC 45606504 rs191727254

HG00100 >hsa-mir-515-1 TCCAAAAGAAAGCACTTTCTGT
TCTCCAAAAGAAAGCACTTTCT TTCTCCAAAAGAAAGCACTTTC
TCATTCTCCAAAAGAAAGCACT TGCAGTCATTCTCCAAAAGAAA 54182326
rs374576826
HG00100 >hsa-mir-663aTCCCAGGCGGGGCGCCGCGGGA TCCGGCGTCCCAGGCGGGGCGC
TTCCGGCGTCCCAGGCGGGGCG GCGCCGCGGGACCTCCCTCGTG
GGCGCCGCGGGACCTCCCTCGT 26188880 .

HG00100 >hsa-mir-149 TCCGTGTCTTCACTCCCGTGCT TGGCTCCGTGTCTTCACTCCCG
TCTGGCTCCGTGTCTTCACTCC CCGTGTCTTCACTCCCGTGCTT
AGCTCTGGCTCCGTGTCTTCAC 241395503 rs2292832

HG00100 >hsa-mir-618 TCCTTCTGAGTGTAATTACGTA TGTCCTTCTGAGTGTAATTACG TTGTCCTTCTGAGTGTAATTAC TACTTGTCCTTCTGAGTGTAAT GTCCTTCTGAGTGTAATTACGT 81329536 rs2682818

HG00100 >hsa-mir-492 TCGAGGACCTGCGGGACAAGAT TACAGGACCATCGAGGACCTGC TACTACAGGACCATCGAGGACC TCCAGCCACTACTACAGGACCA GACCTGCGGGACAAGATTCTTG 95228179 rs200816308

HG00100 >hsa-mir-520cTCTCAGGCTGTCGTCCTCTAGA TCAGGCTGTCGTCCTCTAGAGG TGTCGTCCTCTAGAGGGAAGCA TCGTCCTCTAGAGGGAAGCACT TCCTCTAGAGGGAAGCACTTTC 54210774 .

HG00100 >hsa-mir-4305TCTGGGTTCTTAGAGGCCTAAT TTCTGGGTTCTTAGAGGCCTAA GTTCTGGGTTCTTAGAGGCCTA TCCAGTTCTGGGTTCTTAGAGG CAGTTCTGGGTTCTTAGAGGCC 40238175 rs67976778

HG00100 >hsa-mir-3151TGATGGGTGGGGCAATGGGATC TGGGTGGGGCAATGGGATCAGG TGGGGCAATGGGATCAGGTGCC GGGGTGATGGGTGGGGCAATGG GGGTGATGGGTGGGGCAATGGG 104166902 rs35605502

HG00100 >hsa-mir-3156-3 TGCAGAAGAAAGATCTGGAAGT GCAGAAGAAAGATCTGGAAGTG GAAGAAAGATCTGGAAGTGGGA GAAAGATCTGGAAGTGGGAGAC AGAAGAAAGATCTGGAAGTGGG 14778721 rs2747232

HG00100 >hsa-mir-3152TGCCTCTGTTCTAACACAAGAC TTGCCTCTGTTCTAACACAAGA TATTGCCTCTGTTCTAACACAA TTATTGCCTCTGTTCTAACACA TGCAGAGTTATTGCCTCTGTTC 18573360 rs13299349

HG00100 >hsa-mir-1254-2 TGGAAGCTGGAGCCTGCAGTGA TGAGCCTGGAAGCTGGAGCCTG GAAGCTGGAGCCTGCAGTGAGC GGAAGCTGGAGCCTGCAGTGAG GCCTGGAAGCTGGAGCCTGCAG 23682383 rs200793185

HG00100 >hsa-mir-516b-2 TGGAGGTAAGAAGCACTTTGTG TCTGGAGGTAAGAAGCACTTTG TGACCATCTGGAGGTAAGAAGC TGTGACCATCTGGAGGTAAGAA TGATGTGACCATCTGGAGGTAA 54228742 rs10670323

HG00100 >hsa-mir-1273h TGGGAGGTCAAGGCTGTAGTGT TGAGCCTGGGAGGTCAAGGCTG TTGAGCCTGGGAGGTCAAGGCT TGCTTGAGCCTGGGAGGTCAAG TTGCTTGAGCCTGGGAGGTCAA 24214486 .

HG00100 >hsa-mir-412 TGGGGTACGGGGATGGATGGTC TCGACCAGTTGGAAAGTAATTG TGGTCGACCAGTTGGAAAGTAA TACGGGGATGGATGGTCGACCA TGGATGGTCGACCAGTTGGAAA 101531854 rs61992671

HG00100 >hsa-mir-412 TGGGGTACGGGGATGGATGGTC TCGACCAGTTGGAAAGTAATTG TGGTCGACCAGTTGGAAAGTAA TACGGGGATGGATGGTCGACCA

TGGATGGTCGACCAGTTGGAAA 101531849 . 101531854 rs61992671

HG00100 >hsa-mir-323bTGTCCGTGGTGAGTTCGCATTA TTGTCCGTGGTGAGTTCGCATT TACTCGGAGGGAGGTTGTCCGT TCGGAGGGAGGTTGTCCGTGGT AGGTTGTCCGTGGTGAGTTCGC 101522556 rs56103835

HG00100 >hsa-mir-642bTTCCCTCTCCAAATGTGTCTTG TTGGGAGGTTCCCTCTCCAAAT TGGGAGGTTCCCTCTCCAAATG GAGTTGGGAGGTTCCCTCTCCA GTTGGGAGGTTCCCTCTCCAAA 46178217 rs111664333

HG00100 >hsa-mir-4277TTCTGAGCACAGTACACTGGGC TCGAGGCAGTTCTGAGCACAGT TGGGTCGAGGCAGTTCTGAGCA GTTCTGAGCACAGTACACTGGG GCAGTTCTGAGCACAGTACACT 1708902 rs115200817

HG00100 >hsa-mir-4277TTCTGAGCACAGTACACTGGGC TCGAGGCAGTTCTGAGCACAGT TGGGTCGAGGCAGTTCTGAGCA TTGGGTCGAGGCAGTTCTGAGC GTTCTGAGCACAGTACACTGGG 1708983 rs12523324

HG00100 >hsa-mir-4277TTCTGAGCACAGTACACTGGGC TCGAGGCAGTTCTGAGCACAGT TGGGTCGAGGCAGTTCTGAGCA TTGGGTCGAGGCAGTTCTGAGC GTTCTGAGCACAGTACACTGGG 1708902 rs115200817 1708983 rs12523324

HG00100 >hsa-mir-553 TTTAAAACGGTGAGATTTTGTT TTTTAAAACGGTGAGATTTTGT ATTTTAAAACGGTGAGATTTTG TATTTTAAAACGGTGAGATTTT TTATTTTAAAACGGTGAGATTT 100746848 .

HG00100 >hsa-mir-553 TTTAAAACGGTGAGATTTTGTT TTTTAAAACGGTGAGATTTTGT ATTTTAAAACGGTGAGATTTTG TATTTTAAAACGGTGAGATTTT TTATTTTAAAACGGTGAGATTT 100746855 rs112891767

HG00100 >hsa-mir-553 TTTAAAACGGTGAGATTTTGTT TTTTAAAACGGTGAGATTTTGT ATTTTAAAACGGTGAGATTTTG TATTTTAAAACGGTGAGATTTT TTATTTTAAAACGGTGAGATTT 100746848 . 100746855 rs112891767

HG00100 >hsa-mir-548ad AAAACGACAATGACTTTTGCAC ACTTTTGCACCAATCTAATAC CTTTTGCACCAATCTAATAC GCAAAAACGACAATGACTTTTG ACAATGACTTTTGCACCAATCT 35696519 rs62143301

HG00100 >hsa-mir-1294ACAACAGTGCCAACCTCACGGG ACAGTGCCAACCTCACGGGACT AACAGTGCCAACCTCACGGGAC TACAACAGTGCCAACCTCACGG ATATACAACAGTGCCAACCTCA 153726769 rs13186787

HG00100 >hsa-mir-646 AGCAGCTGCCTCTGAGGCCTCA CTGAGGCCTCAGGCTCAGTGGC CTCTGAGGCCTCAGGCTCAGTG TCTGAGGCCTCAGGCTCAGTGG GCCTCTGAGGCCTCAGGCTCAG 58883534 rs6513496

HG00100 >hsa-mir-149 AGGGAGGGAGGGACGGGGGCTG GGGCTGTGCTGGGGCAGCCGGA

GGGACGGGGGCTGTGCTGGGGC GACGGGGGCTGTGCTGGGGCAG
GGGGCTGTGCTGGGGCAGCCGG 241395503 rs2292832

HG00100 >hsa-mir-658 AGGTCGGTTGGTCGGTCGGGAA GTCGGTTGGTCGGTCGGGAACG
TAGGTCGGTTGGTCGGTCGGGA . .

HG00100 >hsa-mir-604 CACAGGCTGCGGAATTCAGGAC ACACAGGCTGCGGAATTCAGGA
ACAGGCTGCGGAATTCAGGACA GGCTGCGGAATTCAGGACAGTG
CTGCGGAATTCAGGACAGTGCA 29833998 rs2368393

HG00100 >hsa-mir-604 CACAGGCTGCGGAATTCAGGAC ACACAGGCTGCGGAATTCAGGA
ACAGGCTGCGGAATTCAGGACA GGCTGCGGAATTCAGGACAGTG
CTGCGGAATTCAGGACAGTGCA 29833998 rs2368393 29834003
rs2368392
HG00100 >hsa-mir-216aCACAGTGGTCTCTGGGATTATG ACAGTGGTCTCTGGGATTATGC
CTCTGGGATTATGCTAAACAGA GATTATGCTAAACAGAGCAATT
TGGGATTATGCTAAACAGAGCA 56216090 rs41291179

HG00100 >hsa-mir-412 CGTCCGTATCCGCTGCAG CCGTCCGTATCCGCTGCAG
TCACCTGGTCCACTGGCCGTCC ACCTGGTCCACTGGCCGTCCGT
CACCTGGTCCACTGGCCGTCCG 101531854 rs61992671

HG00100 >hsa-mir-412 CGTCCGTATCCGCTGCAG CCGTCCGTATCCGCTGCAG
TCACCTGGTTCACTGGCCGTCC ACCTGGTTCACTGGCCGTCCGT
CACCTGGTTCACTGGCCGTCCG 101531849 . 101531854 rs61992671

HG00100 >hsa-mir-3180-4 CTCCGGATGCCAGTCCCTCATC
GGAGGGTGAAGCCTCCGGATGC CTGGCCTGGTCGCGCTGTGGCT
AGCGGAGGGTGAAGCCTCCGGA GAGCGGAGGGTGAAGCCTCCGG 15248720
rs75000738
HG00100 >hsa-mir-4268CTCTCAGGATGTGATGTCACCT CCTCTCAGGATGTGATGTCACC
GCTCCTCCTCTCAGGATGTGAT CTCCTCCTCTCAGGATGTGATG
TCCTCTCAGGATGTGATGTCAC 220771223 rs4674470

HG00100 >hsa-mir-3151CTGATCCCACACCCCACCTGTC TGATCCCACACCCCACCTGTCA
GATCCCACACCCCACCTGTCAC GGGCATCCCACCTGATCCCACA
TCCCACCTGATCCCACACCCCA 104166902 rs35605502

HG00100 >hsa-mir-412 CTTCACCTGGTTCACTAGCCGT ACCTGGTTCACTAGCCGTCCGT
TGTACTTCACCTGGTTCACTAG CTGGTTCACTAGCCGTCCGTAT
GTACTTCACCTGGTTCACTAGC 101531849 .

HG00100 >hsa-mir-320eGAAAAGCTGGGTTGAGAAGGT AAAAGCTGGGTTGAGAAGGT
GGAAAAGCTGGGTTGAGAAGGT GGGAAAAGCTGGGTTGAGAAGG rs10423365

HG00100 >hsa-mir-1343GCCCCTCCTGGGGCCCGCACTC CCCCTCCTGGGGCCCGCACTCT
GGGGCCCGCACTCTCGCTCTGG CCCTCCTGGGGCCCGCACTCTC

TGGGGCCCGCACTCTCGCTCTG 34963416 rs2986407

HG00100 >hsa-mir-3180-4 GGAGGGTGAAGCCTCCGGATGC
GGTGAAGCCTCCGGATGCCAGT AGCGGAGGGTGAAGCCTCCGGA
GCGGAGGGTGAAGCCTCCGGAT GCCTGGTCGCGCTGTGGCGAAG 15248798
rs183853838
HG00100 >hsa-mir-3180-4 GGAGGGTGAAGCCTCCGGATGC
GGTGAAGCCTCCGGATGCCAGT AGCGGAGGGTGAAGCCTCCGGA
GCGGAGGGTGAAGCCTCCGGAT CTGGCCTGGTCGCGCTGTGGCT 15248720
rs75000738 15248798 rs183853838
HG00100 >hsa-mir-1227GGCATTTGACCCCGTGCCACCC AGGCATTTGACCCCGTGCCACC
AGGCATTTGACCCCGTGCCACC TGACCCCGTGCCACCCTTTTCC
AGGCATTTGACCCCGTGCCACC 2234093 rs190788838

HG00100 >hsa-mir-580 TATTTGAGAATGATGAATCATT TGAATCATTAGGTTCCGGTCAG
ATGAATCATTAGGTTCCGGTCA GAGAATGATGAATCATTAGGTT
TTTGAGAATGATGAATCATTAG 36148057 rs115089112

HG00100 >hsa-mir-3118-1 TGAAAATTCTTCTAGTGTG ATGAAAATTCTTCTAGTGTG
TGCATTATGAAAATTCTTCTAG TTATGAAAATTCTTCTAGTGTG
ATTATGAAAATTCTTCTAGTGT 142667330 rs76132421

HG00100 >hsa-mir-558 TGAGCTGCTGTACCAAAATACC GCTGCTGTACCAAAATACCACA
TGCTGTACCAAAATACCACAAA CTGAGCTGCTGTACCAAAATAC
GAGCTGCTGTACCAAAATACCA 32757230 rs72089144

HG00100 >hsa-mir-516b-2 TGCTTCCTTTCAGAGGGTTACT
TCAGAGGGTTACTCTTTGAGA AAAGAAAGTGCTTCCTTTCAGA
CAGAGGGTTACTCTTTGAGA AAAAGAAAGTGCTTCCTTTCAG 54228742
rs10670323
HG00100 >hsa-mir-637 TGGCTAAGGTGTTGGCTCGGGC TGGCTAAGGTGTTGGCTCGGGC

HG00100 >hsa-mir-548aj-2 AAAAACTGCAATTACTTTTACA
TGGTAAAAACTGCAATTACTTT AACTGCAATTACTTTTACACCA
AATTACTTTTACACCAACCTAA 37883200 rs73463468

HG00100 >hsa-mir-320c-1 AAAAGCTGGGTTGAGAGGGTAG
AGCTGGGTTGAGAGGGTAGGAA CTGGGTTGAGAGGGTAGGAAAA
AGGGTAGGAAAAAAATGATGTA 19263542 .

HG00100 >hsa-mir-651 AAAAGGAAAGTGTATCCTAAAA GGAAAGTGTATCCTAAAAGGCA
TGTATCCTAAAAGGCAATGACA AAAGGAAAGTGTATCCTAAAAG 8095036
rs111336920
HG00100 >hsa-mir-499bAAACATCACTGCAAGTCTTAAC
rs3746444
HG00100 >hsa-mir-202 AAAGAGGTATAGGGCATGGGAA AAGAGGTATAGGGCATGGGAAA
GGGAAAACGGGGCGGTCGGGTC TAAAGAGGTATAGGGCATGGGA 135061112

rs12355840
HG00100 >hsa-mir-548ap AACAAAAACCACAATTACTTTT
CAAAAACCACAATTACTTTTTA CAATTACTTTTTACTGACCTAA
rs4414449
HG00100 >hsa-mir-548ap AACAAAAACCACAATTACTTTT
CAAAAACCACAATTACTTTTTA TTACTTTTTACTGACCTAAAGA
rs4577031
HG00100 >hsa-mir-548ap AACAAAAACCACAATTACTTTT
CAAAAACCACAATTACTTTTTA CAATTACTTTTTACTGACCTAA
rs4414449 86368959 rs4577031
HG00100 >hsa-mir-1294AACAGTGCCAACCTCACGGGAC TATACAACAGTGCCAACCTCAC
ATACAACAGTGCCAACCTCACG ACAACAGTGCCAACCTCACGGG 153726769
rs13186787
HG00100 >hsa-mir-1255b-2 AACCACTTTCTTTGCTCATCCG
CTTTCTTTGCTCATCCGTAAGG AAACCACTTTCTTTGCTCATCC
CTTTCTTTGCTCATCCGTAAGG 167967958 rs79639536

HG00100 >hsa-mir-548ad AACGACAATGACTTTTGCACCA
GGCAAAAACGACAATGACTTTT TGGCAAAAACGACAATGACTTT
AAAAACGACAATGACTTTTGCA 35696519 rs62143301

HG00100 >hsa-mir-516b-2 AAGAAAGTGCTTCCTTTCAGAG
AAAAGAAAGTGCTTCCTTTCAG AAGAAAAGAAAGTGCTTCCTTT
AAAGTGCTTCCTTTCAGAGGGT 54228742 rs10670323

HG00100 >hsa-mir-520cAAGAAAGTGCTTCCTTTGAGAG AAAAGAAAGTGCTTCCTTTGAG
GTGCTTCCTTTGAGAGGGTTAC TTTGAGAGGGTTACCGTTTGAG 54210774
.
HG00100 >hsa-mir-24-2ACACTGGCTCAGTTCAGCAGGA CACTGGCTCAGTTCAGCAGGAA
TGGCTCAGTTCAGCAGGAACAG CTGGCTCAGTTCAGCAGGAACA 13947170
.
HG00100 >hsa-mir-597 ACAGTGGTTCTCTTGTGGCTTA GGCTTAAGCGTAATGTAGAGTA
TGTACAGTGGTTCTCTTGTGGC AATGTACAGTGGTTCTCTTGTG 9599255
rs146125159
HG00100 >hsa-mir-449cACAGTTGCTAGTTGCACTCCTC AACAGTTGCTAGTTGCACTCCT
GTTGCTAGTTGCACTCCTCTCT GTTGCACTCCTCTCTGTTGCAT 54468166
rs75661995
HG00100 >hsa-mir-3118-1 ACTGCATTATGAAAATTCTTCT
ATTATGAAAATTCTTCTAGTGT GCATTATGAAAATTCTTCTAGT
CTGCATTATGAAAATTCTTCTA 142667330 rs76132421

HG00100 >hsa-mir-642bAGATACATTTGGAGAGGGACCC TTGGAGAGGGACCCTCCCAACT
TTTGGAGAGGGACCCTCCCAAC ATACATTTGGAGAGGGACCCTC 46178217
rs111664333
HG00100 >hsa-mir-3180-4 AGCGGAGGGTGAAGCCTCCGGA
GGAGGGTGAAGCCTCCGGATGC GAGCGGAGGGTGAAGCCTCCGG
GCGGAGGGTGAAGCCTCCGGAT 15248798 rs183853838

HG00100 >hsa-mir-3180-4 AGCGGAGGGTGAAGCCTCCGGA
GGAGGGTGAAGCCTCCGGATGC GAGCGGAGGGTGAAGCCTCCGG
GCGGAGGGTGAAGCCTCCGGAT 15248720 rs75000738 15248798
rs183853838

HG00100 >hsa-mir-30d AGCTTTCAGTCAGATGTTTGCT GGCTAAGCTTTCAGTCAGATGT
GCTAAGCTTTCAGTCAGATGTT TTCAGTCAGATGTTTGCTGCTA 135817150
.

HG00100 >hsa-mir-519a-2 AGGAAAGTGCATCCTTTTAGAG
AGTGCATCCTTTTAGAGGGTTA GGAAAGTGCATCCTTTTAGAGG
GAAAGGAAAGTGCATCCTTTTA 54265670 .

HG00100 >hsa-mir-646 AGGAAGCAGCTGCCTCTGAGGC GCTGCCTCTGAGGCCTCAGGCT
CTGAGGCCTCAGGCTCAGTGGC TCTGAGGCCTCAGGCTCAGTGG 58883534
rs6513496

HG00100 >hsa-mir-3167AGGATTTCAGAAATACTGGTGT GGATTTCAGAAATACTGGTGTC
CAGGAAGGATTTCAGAAATACT CTCAGGAAGGATTTCAGAAATA 126858392
rs670637

HG00100 >hsa-mir-3167AGGATTTCAGAAATACTGGTGT AAGGATTTCAGAAATACTGGTG
TTCAGGAAGGATTTCAGAAATA GGATTTCAGAAATACTGGTGTC 126858406
rs634171

HG00100 >hsa-mir-3167AGGATTTCAGAAATACTGGTGT AAGGATTTCAGAAATACTGGTG
CTCAGGAAGGATTTCAGAAATA GGATTTCAGAAATACTGGTGTC 126858392
rs670637 126858406 rs634171

HG00100 >hsa-mir-630 AGTATTCTGTACCAGGGAAGGT ACCTAGTATTCTGTACCAGGGA
CCAGGGAAGGTAGTTCTTAACT GGAAGGTAGTTCTTAACTATGT 72879653
rs113971639

HG00100 >hsa-mir-3686AGTGATCTGTAAGAGAAAGTAA TCTGTAAGAGAAAGTAAATGAA
GTAAGAGAAAGTAAATGAAAGA ACAGTGATCTGTAAGAGAAAGT 130496365
rs6997249

HG00100 >hsa-mir-513cATAAATTTCACCTTTCTGAGAA TTCTGAGAAGAGTAATGTACAG
CCTTTCTGAGAAGAGTAATGTA TTTCACCTTTCTGAGAAGAGTA 146271303
rs145416750

HG00100 >hsa-mir-323bATACACGGTCGACCTCTTTTCG TACACGGTCGACCTCTTTTCGG
ACACGGTCGACCTCTTTTCGGT rs56103835

HG00100 >hsa-mir-642aATTTGGAGAGGGAACCTCCCAA AGACACATTTGGAGAGGGAACC
ACACATTTGGAGAGGGAACCTC CACATTTGGAGAGGGAACCTCC 46178217
rs111664333

HG00100 >hsa-mir-604 CACAGGCTGCGGAATTCAGGAC TGACACAGGCTGCGGAATTCAG
GACACAGGCTGCGGAATTCAGG rs2368392

HG00100 >hsa-mir-412 CACCTGGTCCACTGGCCGTCCG ACCTGGTCCACTGGCCGTCCGT
CTGGCCGTCCGTATCCGCTGCA CCTGGTCCACTGGCCGTCCGTA 101531854
rs61992671

HG00100 >hsa-mir-412 CACCTGGTTCACTGGCCGTCCG ACCTGGTTCACTGGCCGTCCGT
CTGGCCGTCCGTATCCGCTGCA CCTGGTTCACTGGCCGTCCGTA 101531849

. 101531854 rs61992671
HG00100 >hsa-mir-515-1 CAGAGTGCCTTCTTTTGGAGCA
GAGTGCCTTCTTTTGGAGCATT TGCCTTCTTTTGGAGCATTACT
GTGCCTTCTTTTGGAGCATTAC 54182326 rs374576826

HG00100 >hsa-mir-1227CATTTGACCCCGTGCCACCCTT ATTTGACCCCGTGCCACCCTTT
AGGCATTTGACCCCGTGCCACC GACCCCGTGCCACCCTTTTCCC 2234093
rs190788838
HG00100 >hsa-mir-1908CCACCGGCCGCCGGCTCCGCCC CCGCCGGCTCCGCCCCGGCCCC
GGCCGCCGGCTCCGCCCCGGCC CGGCCGCCGGCTCCGCCCCGGC 61582708
rs174561
HG00100 >hsa-mir-3151CCACCTGATCCCACACCCCACC CACCTGATCCCACACCCCACCT
CCCACCTGATCCCACACCCCAC TGATCCCACACCCCACCTGTCA 104166902
rs35605502
HG00100 >hsa-mir-1343CCCCTCCTGGGGCCCGCACTCT CCTGGGGCCCGCACTCTCGCTC
CCCTCCTGGGGCCCGCACTCTC TGGGGCCCGCACTCTCGCTCTG 34963416
rs2986407
HG00100 >hsa-mir-3679CCCTTCCCCCCAGTAATCTTCA CCTTCCCCCCAGTAATCTTCAT
TCCCCCCAGTAATCTTCATCAT CTTCCCCCCAGTAATCTTCATC 134884700
rs6430498
HG00100 >hsa-mir-4268CCTCTCAGGATGTGATGTCACC CTCCTCTCAGGATGTGATGTCA
CTCTCAGGATGTGATGTCACCT rs4674470

HG00100 >hsa-mir-4254CCTGGAGATACTCCACCATCTC AGATACTCCACCATCTCCCCCA
GGAGATACTCCACCATCTCCCC rs12731294

HG00100 >hsa-mir-3152CCTGTGTTAGAATAAGGGCAAT TTAGAATAAGGGCAATAACTCT
AGAATAAGGGCAATAACTCTGC TGTGTTAGAATAAGGGCAATAA 18573360
rs13299349
HG00100 >hsa-mir-216aCTCACAGTGGTCTCTGGGATTA TCTCTGGGATTATGCTAAACAG
GTGGTCTCTGGGATTATGCTAA CTCTGGGATTATGCTAAACAGA 56216090
rs41291179
HG00100 >hsa-mir-3117CTCATATAGTGCCAGGTGTTTT GACTCATATAGTGCCAGGTGTT
TCATATAGTGCCAGGTGTTTTG ATAAGACTCATATAGTGCCAGG 67094171
rs12402181
HG00100 >hsa-mir-196a-2 CTCGGCAACAAGAAACTGTCTG
ACTCGGCAACAAGAAACTGTCT CAACAAGAAACTGTCTGAGTTA
ACAAGAAACTGTCTGAGTTACA 54385599 rs11614913

HG00100 >hsa-mir-486-2 CTCGGCGCAGCTCAGTACAGGA
AGGGCCTCGGCGCAGCTCAGTA TCGGCGCAGCTCAGTACAGGAT
GGGCCTCGGCGCAGCTCAGTAC 41518007 .

HG00100 >hsa-mir-3615CTCTCTCGGCTCCTCGCGGCTC GGCTCCTCGCGGCTCGCGGCGG
CGGCTCCTCGCGGCTCGCGGCG TCGGCTCCTCGCGGCTCGCGGC 72744798
rs745666
HG00100 >hsa-mir-888 CTCTTTGGGTGAAGGAAGGCTC CTGACACCTCTTTGGGTGAAGG

GACTGACACCTCTTTGGGTGAA CCTCTTTGGGTGAAGGAAGGCT 145076355 rs143634721
HG00100 >hsa-mir-888 CTCTTTGGGTGAAGGAAGGCTC CTGACACCTCTTTGGGTGAAGG GACTGACACCTCTTTGGGTGAA CCTCTTTGGGTGAAGGAAGGCT 145076302 rs5965660 145076355 rs143634721
HG00100 >hsa-mir-1269a CTGGACTGAGCCATGCTACTGG TGCCTGGACTGAGCCATGCTAC AATGCCTGGACTGAGCCATGCT rs73239138

HG00100 >hsa-mir-3922CTGTGGGACTTCTGGCCTTGAC ACCTGTGGGACTTCTGGCCTTG GGGACTTCTGGCCTTGACTTGA TGGGACTTCTGGCCTTGACTTG 104985443 rs61938575
HG00100 >hsa-mir-2117CTGTTCTCTTTGCCAAGGACAG GCTGTTCTCTTTGCCAAGGACA TCTCTTTGCCAAGGACAGATCT TGTTCTCTTTGCCAAGGACAGA 41522213 rs7207008
HG00100 >hsa-mir-4274GACCCAGCAGTCCCTCCCCCTG CCCAGCAGTCCCTCCCCCTGCA TGACCCAGCAGTCCCTCCCCCT TCAGGTGACCCAGCAGTCCCTC 7461769 rs12512664
HG00100 >hsa-mir-888 GACTGACACCTCTTTGGGTGAA GACACCTCTTTGGGTGAAGGAA TGACACCTCTTTGGGTGAAGGA CACCTCTTTGGGTGAAGGAAGG 145076302 rs5965660
HG00100 >hsa-mir-3188GAGGCTTTGTGCGGATACGGGG GAGAGGCTTTGTGCGGATACGG GCGGATACGGGGCTGGAGGCCT rs7247237

HG00100 >hsa-mir-3188GAGGCTTTGTGCGGATACGGGG GAGAGGCTTTGTGCGGATACGG GGAGAGGCTTTGTGCGGATACG rs7247767

HG00100 >hsa-mir-3188GAGGCTTTGTGCGGATACGGGG GAGAGGCTTTGTGCGGATACGG GGAGAGGCTTTGTGCGGATACG rs7247237 18392913 rs7247767
HG00100 >hsa-mir-629 GAGGTTCTCCCAACGTAAGCCC AGGTTCTCCCAACGTAAGCCCA TCTCCCAACGTAAGCCCAGCCC CAGGAGGTTCTCCCAACGTAAG 70371761 rs377691713
HG00100 >hsa-mir-222 GCAGCTACATCTGGCTACTGGG TACTGGGTCTCTGATGGCATCT GCTACTGGGTCTCTGATGGCAT CTGGCTACTGGGTCTCTGATGG 45606504 rs191727254
HG00100 >hsa-mir-500bGCAGTGCACCCAGGCAAGGATT CACCCAGGCAAGGATTCTGCGA TGCAGTGCACCCAGGCAAGGAT AGGCAAGGATTCTGCGAGGGGG 49775351 rs151318590
HG00100 >hsa-mir-3180-4 GCGGAGGGTGAAGCCTCCGGAT CGCTGGCCTGGTCGCGCTGTGG TCGCTGGCCTGGTCGCGCTGTG AAGCCTCCGGATGCCAGTCCCT 15248720 rs75000738

HG00100 >hsa-mir-3176GGGACTGGCCTGGGACTACCGG GCCTGGGACTACCGGGGGTGGC ACTGGCCTGGGACTACCGGGGG CAGGGACTGGCCTGGGACTACC 593277 rs8054514
HG00100 >hsa-mir-149 GGGAGGGAGGGACGGGGGCTGT GGAGGGACGGGGGCTGTGCTGG AGGGACGGGGGCTGTGCTGGGG GACGGGGGCTGTGCTGGGGCAG 241395503

rs2292832
HG00100 >hsa-mir-1197GTAGGACACATGGTCTACTTCT ACACATGGTCTACTTCTTCTCA ACATGGTCTACTTCTTCTCAAT TAGGACACATGGTCTACTTCTT 101491923 rs141611518
HG00100 >hsa-mir-3936TAAGGGGTGTATGGCAGATGCA CACCCGACAGATGCACTTGGCA GATGCACCCGACAGATGCACTT TGTATGGCAGATGCACCCGACA 131701279 rs367805
HG00100 >hsa-mir-466 TACACATACACGCAACACACCT ACATACACATACACGCAACACA ACACATACACGCAACACACCTA CATACACATACACGCAACACAC 31203207 rs148367480
HG00100 >hsa-mir-300 TATACAAGGGCAGACTCTCTCT TGATTATACAAGGGCAGACTCT ATTATACAAGGGCAGACTCTCT rs12894467

HG00100 >hsa-mir-499aTCACAGCAAGTCTGTGCTGCTT ACAGCAAGTCTGTGCTGCTTCC TCCCTACGCTGCCTGGGCAGGG CGTCACAGCAAGTCTGTGCTGC 33578251 rs3746444
HG00100 >hsa-mir-412 TCACCTGGTTCACTAGCCGTCC TCACCTGGTTCACTAGCCGTCC ATGTACTTCACCTGGTTCACTA CTTCACCTGGTTCACTAGCCGT 101531849 .
HG00100 >hsa-mir-532 TCCCACACCCAAGGCTTGCAGA CTCCCACACCCAAGGCTTGCAG CCTCCCACACCCAAGGCTTGCA CACCCAAGGCTTGCAGAAGAGC 49767832 rs456615
HG00100 >hsa-mir-532 TCCCACACCCAAGGCTTGCAGA CTCCCACACCCAAGGCTTGCAG CCTCCCACACCCAAGGCTTGCA CACCCAAGGCTTGCAGAAGAGC 49767835 rs456617
HG00100 >hsa-mir-532 TCCCACACCCAAGGCTTGCAGA CTCCCACACCCAAGGCTTGCAG CCTCCCACACCCAAGGCTTGCA CACCCAAGGCTTGCAGAAGAGC 49767832 rs456615 49767835 rs456617
HG00100 >hsa-mir-558 TCCTGAGCTGCTGTACCAAAAT TTCCTGAGCTGCTGTACCAAAA GAGCTGCTGTACCAAAATACCA CCTGAGCTGCTGTACCAAAATA 32757230 rs72089144
HG00100 >hsa-mir-1304TCTCACTGTAGCATCGAACCCC GAACCCCTGGGCTCAAGTGATT CGAACCCCTGGGCTCAAGTGAT CTCACTGTAGCATCGAACCCCT 93466866 rs2155248
HG00100 >hsa-mir-4308TCTTTCCCTGGAGTTTCTTCTT AATCTTTCCCTGGAGTTTCTTC GAAATCTTTCCCTGGAGTTTCT CCTGGAGTTTCTTCTTACCTTT 55344901 rs28477407
HG00100 >hsa-mir-595 TGAAGTGTGCCGTGGTGTGTCT GTGTGTCTGGAGGAAGCGCCTG GCCGTGGTGTGTCTGGAGGAAG rs4909237

HG00100 >hsa-mir-604 TGACACAGGCTGCGGAATTCAG CACAGGCTGCGGAATTCAGGAC GACACAGGCTGCGGAATTCAGG TTTCTGACACAGGCTGCGGAAT 29833998 rs2368393
HG00100 >hsa-mir-604 TGACACAGGCTGCGGAATTCAG GACACAGGCTGCGGAATTCAGG CACAGGCTGCGGAATTCAGGAC TTTCTGACACAGGCTGCGGAAT 29833998 rs2368393 29834003 rs2368392
HG00100 >hsa-mir-1273h TGCTGCAGACTCGACCTCCCAG

TGCAGACTCGACCTCCCAGGCT CTGCAGACTCGACCTCCCAGGC
AGACTCGACCTCCCAGGCTTAA 24214486 .

HG00100 >hsa-mir-3909TGTCCTCTAGGGCCTGCAGTCT TCCTCTAGGGCCTGCAGTCTCA
TGGGAGAGTGACATGCACCAGG TAGGGCCTGCAGTCTCATGGGA 35731697
rs9607265
HG00100 >hsa-mir-2053TGTTAATTAAACCTCTATTTAC ACTTTAAGTGTTAATTAAACCT
TTTAAGTGTTAATTAAACCTCT GTTAATTAAACCTCTATTTACA 113655752
rs10505168
HG00100 >hsa-mir-563 TTAGGTTGACATACGTTTCCCT AGGTTGACATACGTTTCCCTGG
ACATACGTTTCCCTGGTAGCCA

HG00100 >hsa-mir-585 TTGGGCGTATCTGTATGCTAGG TATCTGTATGCTAGGGCTGCCG
TGGGCGTATCTGTATGCTAGGG GCGTATCTGTATGCTAGGGCTG 168690612
rs62376934
HG00100 >hsa-mir-1303TTTAGAGACGGGGTCTTGCTCT TAGAGACGGGGTCTTGCTCTGT
GGGTCTTGCTCTGTTGCCAGGC ACGGGGTCTTGCTCTGTTGCCA 154065347
rs142414368
HG00100 >hsa-mir-1303TTTAGAGACGGGGTCTTGCTCT TAGAGACGGGGTCTTGCTCTGT
TTAGAGACGGGGTCTTGCTCTG ACGGGGTCTTGCTCTGTTGCCA 154065383
rs75538180
HG00100 >hsa-mir-1303TTTAGAGACGGGGTCTTGCTCT TAGAGACGGGGTCTTGCTCTGT
TTAGAGACGGGGTCTTGCTCTG GGGTCTTGCTCTGTTGCCAGGC 154065347
rs142414368 154065383 rs75538180
HG00100 >hsa-mir-580 TTTGAGAATGATGAATCATTAG GATGAATCATTAGGTTCCGGTC
AATGATGAATCATTAGGTTCCG AGAATGATGAATCATTAGGTTC 36148057
rs115089112
HG00100 >hsa-mir-133bTTTGGTCCCCTTCAACCAGCTA TGGTCCCCTTCAACCAGCTACA
CCTTCAACCAGCTACAGCAGGG AGAGGTTTGGTCCCCTTCAACC 52013832
rs374103744
HG00101 >hsa-mir-1273h CCTGGGAGGTCAAGGCTGTAGT
TGGGAGGTCAAGGCTGTAGTGT ATTGCTTGAGCCTGGGAGGTCA
GCCTGGGAGGTCAAGGCTGTAG TTGAGCCTGGGAGGTCAAGGCT 24214486 .

HG00101 >hsa-mir-3125AGAATGGATAGAGGAAGCTGTG GAGGAAGCTGTGGAGAGAACTC
AGAGGAAGCTGTGGAGAGAACT GCTGTGGAGAGAACTCACGGTG
GGAAGCTGTGGAGAGAACTCAC 12877501 rs78852835

HG00101 >hsa-mir-3141TCACCCGGTGAGGGCGGGTGGA CCGGTGAGGGCGGGTGGAGGAG
CGGTGAGGGCGGGTGGAGGAGG CACCCGGTGAGGGCGGGTGGAG
CCCGGTGAGGGCGGGTGGAGGA 153975576 rs936581

HG00101 >hsa-mir-3144AAGGGGACCAAAGAGATATATA TAAGGGGACCAAAGAGATATAT
TTAAGGGGACCAAAGAGATATA TTTAAGGGGACCAAAGAGATAT
ATACACTTTAAGGGGACCAAAG 120336327 rs68035463

HG00101 >hsa-mir-3144TAAGGGGACCAAAGAGATATAT TTAAGGGGACCAAAGAGATATA

AAGGGGACCAAAGAGATATATA CTACACTTTAAGGGGACCAAAG
TTTAAGGGGACCAAAGAGATAT 120336384 rs67106263

HG00101 >hsa-mir-3144TAAGGGGACCAAAGAGATATAT AAGGGGACCAAAGAGATATATA
TTAAGGGGACCAAAGAGATATA ATACACTTTAAGGGGACCAAAG
TTTAAGGGGACCAAAGAGATAT 120336327 rs68035463 120336384
rs67106263
HG00101 >hsa-mir-3196GCGGGGCGGCAGGGGCCTCCCC GGCGGGGCGGCAGGGGCCTCCC
GGGCGGGGCGGCAGGGGCCTCC TGGGGGCGGGGCGGCAGGGGCC
GGGGCGGGGCGGCAGGGGCCTC 61870167 rs744591

HG00101 >hsa-mir-4326TCTGCTGTTCCTCTGTCTCCCA TGGTCTGCTGTTCCTCTGTCTC
CTGGTCTGCTGTTCCTCTGTCT GCTGTTCCTCTGTCTCCCAGAC
TGCTGTTCCTCTGTCTCCCAGA 61918164 rs6062431

HG00101 >hsa-mir-553 TTTTAAAACGGTGAGATTTTGT TATTTTAAAACGGTGAGATTTT
TTTTATTTTAAAACGGTGAGAT TTTAAAACGGTGAGATTTTGTT
ATTTTATTTTAAAACGGTGAGA 100746848 .

HG00101 >hsa-mir-553 TTTTATTTTAAAACGGTGAGAT TTTAAAACGGTGAGATTTTGTT
TTTTAAAACGGTGAGATTTTGT AATTTTATTTTAAAACGGTGAG
TTATTTTAAAACGGTGAGATTT 100746855 rs112891767

HG00101 >hsa-mir-553 TTTTAAAACGGTGAGATTTTGT TTTTATTTTAAAACGGTGAGAT
TTTAAAACGGTGAGATTTTGTT TATTTTAAAACGGTGAGATTTT
ATTTTATTTTAAAACGGTGAGA 100746848 . 100746855
rs112891767
HG00101 >hsa-mir-744 TTGGGCAAGGTGCGGGGCTAGG TGGGCAAGGTGCGGGGCTAGGG
CGGGGCTAGGGCTAACAGCAGT GTGCGGGGCTAGGGCTAACAGC
CTAGGGCTAACAGCAGTCTTAC 11985275 .

HG00101 >hsa-mir-877 TAGAGGAGATGGCGCAGGGGAC GAGGAGATGGCGCAGGGGACAC
GAGATGGCGCAGGGGACACGGG TGGCGCAGGGGACACGGGCAAA
GGAGATGGCGCAGGGGACACGG 30552187 rs372113020

HG00101 >hsa-mir-3141ACCCGGTGAGGGCGGGTGGAGG CGGTGAGGGCGGGTGGAGGAGG
CCGGTGAGGGCGGGTGGAGGAG CCCGGTGAGGGCGGGTGGAGGA
CACCCGGTGAGGGCGGGTGGAG 153975576 rs936581

HG00101 >hsa-mir-1307ACCGGACCTCGACCGGCTCGTC CGGACCTCGACCGGCTCGTCTG
CCGGACCTCGACCGGCTCGTCT ATCTCGACCGGACCTCGACCGG
AATCTCGACCGGACCTCGACCG 105154089 rs7911488

HG00101 >hsa-mir-486-2 ACTGAGCTGCCCCGAGCTGGGC
CTGAGCTGCCCCGAGCTGGGCA CTGTACTGAGCTGCCCCGAGCT
CCTGTACTGAGCTGCCCCGAGC GTACTGAGCTGCCCCGAGCTGG 41518007 .

HG00101 >hsa-mir-1269b AGCCATGCTACGGGCTTCTCTG
ACTGAGCCATGCTACGGGCTTC AGGTTTCTGGACTGAGCCATGC
TGAGGTTTCTGGACTGAGCCAT TTTCTGGACTGAGCCATGCTAC 12820632
rs12451747
HG00101 >hsa-mir-3125AGCTGTGGAGAGAACTCACGGT AAGCTGTGGAGAGAACTCACGG
TAGAGGAAGCTGTGGAGAGAAC TGGATAGAGGAAGCTGTGGAGA
AGGAAGCTGTGGAGAGAACTCA 12877501 rs78852835

HG00101 >hsa-mir-612 AGGGCTTCTGAGCTCCTTAGCA GGGCTTCTGAGCTCCTTAGCAC
CAGGGCTTCTGAGCTCCTTAGC GCAGGGCTTCTGAGCTCCTTAG
GGCAGGGCTTCTGAGCTCCTTA 65211979 rs12803915

HG00101 >hsa-mir-548ap AGTAATTGCAGTCTTTGTCATT
AAGTAATTGCAGTCTTTGTCAT AAAGTAATTGCAGTCTTTGTCA
AAAAGTAATTGCAGTCTTTGTC CAAAAGTAATTGCAGTCTTTGT 86368898
rs4414449
HG00101 >hsa-mir-548ap AGTAATTGCAGTCTTTGTCATT
AAGTAATTGCAGTCTTTGTCAT AAAGTAATTGCAGTCTTTGTCA
AAAAGTAATTGCAGTCTTTGTC CAAAAGTAATTGCAGTCTTTGT 86368898
rs4414449 86368959 rs4577031
HG00101 >hsa-mir-548ap AGTAATTGCGGTCTTTGTCATT
AAGTAATTGCGGTCTTTGTCAT AAAGTAATTGCGGTCTTTGTCA
AAAAGTAATTGCGGTCTTTGTC CAAAAGTAATTGCGGTCTTTGT 86368959
rs4577031
HG00101 >hsa-mir-196a-2 AGTTTCATGTTGTTGGGATTGA
AGGTAGTTTCATGTTGTTGGGA TAGTTTCATGTTGTTGGGATTG
GTAGTTTCATGTTGTTGGGATT GGTAGTTTCATGTTGTTGGGAT 54385599
rs11614913
HG00101 >hsa-mir-500bATCCTTGCTACCTGGGTGAGAG CTTGCTACCTGGGTGAGAGTGC
AATCCTTGCTACCTGGGTGAGA CCTTGCTACCTGGGTGAGAGTG
TCCTTGCTACCTGGGTGAGAGT 49775351 rs151318590

HG00101 >hsa-mir-3622b ATGGGAGGTCAGGTGAGCTCAG
CATGGGAGGTCAGGTGAGCTCA GCATGGGAGGTCAGGTGAGCTC
AGGCATGGGAGGTCAGGTGAGC GGCATGGGAGGTCAGGTGAGCT 27559214
rs66683138
HG00101 >hsa-mir-744 GGGCTAACAGCAGTCTTACTGA GCTAGGGCTAACAGCAGTCTTA
TAGGGCTAACAGCAGTCTTACT GGCTAGGGCTAACAGCAGTCTT
CTAGGGCTAACAGCAGTCTTAC 11985275 .

HG00101 >hsa-mir-548h-3 GTAATCGTGGTTTTTGTCATTG
TGCAAAAGTAATCGTGGTTTTT AGTAATCGTGGTTTTTGTCATT
AAGTAATCGTGGTTTTTGTCAT TGGTGCAAAAGTAATCGTGGTT 13446924
rs9913045
HG00101 >hsa-mir-573 GTGTAACTGATCAGGATCTACT TGTGTAACTGATCAGGATCTAC
GATGTGTAACTGATCAGGATCT TGATGTGTAACTGATCAGGATC
GTGATGTGTAACTGATCAGGAT 24521902 rs76014664

HG00101 >hsa-mir-573 GTGTAACTGATCAGGATCTACT TGTGTAACTGATCAGGATCTAC
GATGTGTAACTGATCAGGATCT TGATGTGTAACTGATCAGGATC
GTGATGTGTAACTGATCAGGAT 24521904 rs78830737

HG00101 >hsa-mir-573 GTGTAACTGATCAGGATCTACT TGTGTAACTGATCAGGATCTAC
GATGTGTAACTGATCAGGATCT TGATGTGTAACTGATCAGGATC
GTGATGTGTAACTGATCAGGAT 24521902 rs76014664 24521904
rs78830737

HG00101 >hsa-mir-3117TAAAGGGCCAGACACTATACGA GGGCCAGACACTATACGAGTCA
GCCAGACACTATACGAGTCATA GGCCAGACACTATACGAGTCAT
CCCTAAAGGGCCAGACACTATA 67094171 rs12402181

HG00101 >hsa-mir-3144TAAGGGGACCAAAGAGATATAT TTAAGGGGACCAAAGAGATATA
TTTAAGGGGACCAAAGAGATAT TACACTTTAAGGGGACCAAAGA
AAATACACTTTAAGGGGACCAA 120336327 rs68035463

HG00101 >hsa-mir-3144TAAGGGGACCAAAGAGATATAT TTAAGGGGACCAAAGAGATATA
TTTAAGGGGACCAAAGAGATAT TACACTTTAAGGGGACCAAAGA
AACTACACTTTAAGGGGACCAA 120336384 rs67106263

HG00101 >hsa-mir-3144TAAGGGGACCAAAGAGATATAT TTAAGGGGACCAAAGAGATATA
TTTAAGGGGACCAAAGAGATAT TACACTTTAAGGGGACCAAAGA
AAATACACTTTAAGGGGACCAA 120336327 rs68035463 120336384
rs67106263

HG00101 >hsa-mir-629 TACGTTGGGAGAACTTTTACGG TTACGTTGGGAGAACTTTTACG
GTTTACGTTGGGAGAACTTTTA TTTACGTTGGGAGAACTTTTAC
GGTTTACGTTGGGAGAACTTTT 70371794 .

HG00101 >hsa-mir-629 TACGTTGGGAGAACTTTTATGG TTACGTTGGGAGAACTTTTATG
TTTACGTTGGGAGAACTTTTAT TGGGTTTACGTTGGGAGAACTT
GTTTACGTTGGGAGAACTTTTA 70371761 rs377691713

HG00101 >hsa-mir-629 TACGTTGGGAGAACTTTTATGG TTACGTTGGGAGAACTTTTATG
TTTACGTTGGGAGAACTTTTAT TGGGTTTACGTTGGGAGAACTT
GTTTACGTTGGGAGAACTTTTA 70371761 rs377691713 70371794 .

HG00101 >hsa-mir-888 TACTCAAAAAGCTTTCAGTCAC TCTACTCAAAAAGCTTTCAGTC
TGCTCTACTCAAAAAGCTTTCA GCTCTACTCAAAAAGCTTTCAG
GGCAGTGCTCTACTCAAAAAGC 145076355 rs143634721

HG00101 >hsa-mir-449cTAGGCAGTGTATTGCTAGCGGC TCAGATAGGCAGTGTATTGCTA
TGTCAGATAGGCAGTGTATTGC TGTGTCAGATAGGCAGTGTATT
TGGGATGTGTCAGATAGGCAGT 54468166 rs75661995

HG00101 >hsa-mir-590 TATTCATAAAAGTGCAGTATGG TTATTCATAAAAGTGCAGTATG
TTTATTCATAAAAGTGCAGTAT AGTTTATTCATAAAAGTGCAGT

TGAGTTTATTCATAAAAGTGCA 73605546 rs189727189

HG00101 >hsa-mir-564 TCAGCAGGCAACATGGCCGAGA TGTCAGCAGGCAACATGGCCGA
GTCAGCAGGCAACATGGCCGAG GTGTCAGCAGGCAACATGGCCG
TGCCAGGCACGGTGTCAGCAGG 44903385 rs114636202

HG00101 >hsa-mir-564 TCAGCAGGCAACATGGCCGAGA TGTCAGCAGGCAACATGGCCGA
GTCAGCAGGCAACATGGCCGAG TGCCAGGCACGGTGTCAGCAGG
GTGTCAGCAGGCAACATGGCCG 44903433 .

HG00101 >hsa-mir-564 TCAGCAGGCAACATGGCCGAGA TGTCAGCAGGCAACATGGCCGA
GTCAGCAGGCAACATGGCCGAG TGCCAGGCACGGTGTCAGCAGG
GTGTCAGCAGGCAACATGGCCG 44903385 rs114636202 44903433 .

HG00101 >hsa-mir-222 TCAGTAGCCAGTGTAGATCCTG TGGCTCAGTAGCCAGTGTAGAT
TTGGCTCAGTAGCCAGTGTAGA TCATTGGCTCAGTAGCCAGTGT
TACCCTCATTGGCTCAGTAGCC 45606504 rs191727254

HG00101 >hsa-mir-515-1 TCCAAAAGAAAGCACTTTCTGT
TCTCCAAAAGAAAGCACTTTCT TTCTCCAAAAGAAAGCACTTTC
TCATTCTCCAAAAGAAAGCACT TGCAGTCATTCTCCAAAAGAAA 54182326
rs374576826
HG00101 >hsa-mir-663aTCCCAGGCGGGGCGCCGCGGGA TCCGGCGTCCCAGGCGGGGCGC
TTCCGGCGTCCCAGGCGGGGCG GCGCCGCGGGACCTCCCTCGTG
GGCGCCGCGGGACCTCCCTCGT 26188880 .

HG00101 >hsa-mir-149 TCCGTGTCTTCACTCCCGTGCT TGGCTCCGTGTCTTCACTCCCG
TCTGGCTCCGTGTCTTCACTCC CCGTGTCTTCACTCCCGTGCTT
AGCTCTGGCTCCGTGTCTTCAC 241395503 rs2292832

HG00101 >hsa-mir-618 TCCTTCTGAGTGTAATTACGTA TGTCCTTCTGAGTGTAATTACG
TTGTCCTTCTGAGTGTAATTAC TACTTGTCCTTCTGAGTGTAAT
GTCCTTCTGAGTGTAATTACGT 81329527 rs145551269

HG00101 >hsa-mir-618 TCCTTCTGAGTGTAATTACGTA TGTCCTTCTGAGTGTAATTACG
TTGTCCTTCTGAGTGTAATTAC TACTTGTCCTTCTGAGTGTAAT
GTCCTTCTGAGTGTAATTACGT 81329536 rs2682818

HG00101 >hsa-mir-618 TCCTTCTGAGTGTAATTACGTA TGTCCTTCTGAGTGTAATTACG
TTGTCCTTCTGAGTGTAATTAC TACTTGTCCTTCTGAGTGTAAT
GTCCTTCTGAGTGTAATTACGT 81329527 rs145551269 81329536
rs2682818
HG00101 >hsa-mir-492 TCGAGGACCTGCGGGACAAGAT TACAGGACCATCGAGGACCTGC
TACTACAGGACCATCGAGGACC TCCAGCCACTACTACAGGACCA
GACCTGCGGGACAAGATTCTTG 95228179 rs200816308

HG00101 >hsa-mir-1200TGAGCCATTCTGAGCCTCAATC TCCTGAGCCATTCTGAGCCTCA

TCTCCTGAGCCATTCTGAGCCT TTCTCCTGAGCCATTCTGAGCC
TGCTACTTCTCCTGAGCCATTC 36958995 rs180826747

HG00101 >hsa-mir-27a TGAGGAGCAGGGCTTAGCTGCT TTAGCTGCTTGTGAGCAGGGTC
GAGGAGCAGGGCTTAGCTGCTT GGAGCAGGGCTTAGCTGCTTGT
GAGCAGGGCTTAGCTGCTTGTG 13947292 rs895819

HG00101 >hsa-mir-3151TGATGGGTGGGGCAATGGGATC TGGGTGGGGCAATGGGATCAGG
TGGGGCAATGGGATCAGGTGCC GGGGTGATGGGTGGGGCAATGG
GGGTGATGGGTGGGGCAATGGG 104166902 rs35605502

HG00101 >hsa-mir-3622a TGCACAGGCACAGGAGCTCAGG
TAGAGGGTGCACAGGCACAGGA GCACAGGAGCTCAGGTGAGGCA
GAGGGTGCACAGGCACAGGAGC GGCACAGGAGCTCAGGTGAGGC 27559214
rs66683138
HG00101 >hsa-mir-3156-3 TGCAGAAGAAAGATCTGGAAGT
GCAGAAGAAAGATCTGGAAGTG GAAGAAAGATCTGGAAGTGGGA
GAAAGATCTGGAAGTGGGAGAC AGAAGAAAGATCTGGAAGTGGG 14778721
rs2747232
HG00101 >hsa-mir-1254-2 TGGAAGCTGGAGCCTGCAGTGA
TGAGCCTGGAAGCTGGAGCCTG GAAGCTGGAGCCTGCAGTGAGC
GGAAGCTGGAGCCTGCAGTGAG GCCTGGAAGCTGGAGCCTGCAG 23682383
rs200793185
HG00101 >hsa-mir-378d-2 TGGACTTGGAGTCAGAAAACTT
GACTTGGAGTCAGAAAACTTTC GGACTTGGAGTCAGAAAACTTT
GAACACTGGACTTGGAGTCAGA TACAAGGAGAGAACACTGGACT 94928250
rs73692959
HG00101 >hsa-mir-877 TGGCGCAGGGGACACGGGCAAA GACACGGGCAAAGACTTGGGGG
GGACACGGGCAAAGACTTGGGG GGGACACGGGCAAAGACTTGGG
GGGGACACGGGCAAAGACTTGG 30552187 rs372113020

HG00101 >hsa-mir-1273h TGGGAGGTCAAGGCTGTAGTGT
TGAGCCTGGGAGGTCAAGGCTG TTGAGCCTGGGAGGTCAAGGCT
TGCTTGAGCCTGGGAGGTCAAG TTGCTTGAGCCTGGGAGGTCAA 24214486 .

HG00101 >hsa-mir-1227TGGTGGGCACTGCTGGGGTGGG AGGCGGTGGTGGGCACTGCTGG
TGGGGCCAGGCGGTGGTGGGCA GGTGGGCACTGCTGGGGTGGGC
GTGGTGGGCACTGCTGGGGTGG 2234093 rs190788838

HG00101 >hsa-mir-323bTGTCCGTGGTGAGTTCGCATTA TTGTCCGTGGTGAGTTCGCATT
TACTCGGAGGGAGGTTGTCCGT TCGGAGGGAGGTTGTCCGTGGT
AGGTTGTCCGTGGTGAGTTCGC 101522556 rs56103835

HG00101 >hsa-mir-642bTTCCCTCTCCAAATGTGTCTTG TTGGGAGGTTCCCTCTCCAAAT
TGGGAGGTTCCCTCTCCAAATG GAGTTGGGAGGTTCCCTCTCCA
GTTGGGAGGTTCCCTCTCCAAA 46178217 rs111664333

HG00101 >hsa-mir-4277TTCTGAGCACAGTACACTGGGC TCGAGGCAGTTCTGAGCACAGT TGGGTCGAGGCAGTTCTGAGCA GTTCTGAGCACAGTACACTGGG GCAGTTCTGAGCACAGTACACT 1708902 rs115200817

HG00101 >hsa-mir-4277TTCTGAGCACAGTACACTGGGC TCGAGGCAGTTCTGAGCACAGT TGGGTCGAGGCAGTTCTGAGCA TTGGGTCGAGGCAGTTCTGAGC GTTCTGAGCACAGTACACTGGG 1708983 rs12523324

HG00101 >hsa-mir-4277TTCTGAGCACAGTACACTGGGC TCGAGGCAGTTCTGAGCACAGT TGGGTCGAGGCAGTTCTGAGCA TTGGGTCGAGGCAGTTCTGAGC GTTCTGAGCACAGTACACTGGG 1708902 rs115200817 1708983 rs12523324

HG00101 >hsa-mir-553 TTTAAAACGGTGAGATTTTGTT TTTTAAAACGGTGAGATTTTGT ATTTTAAAACGGTGAGATTTTG TATTTTAAAACGGTGAGATTTT TTATTTTAAAACGGTGAGATTT 100746848 .

HG00101 >hsa-mir-553 TTTAAAACGGTGAGATTTTGTT TTTTAAAACGGTGAGATTTTGT ATTTTAAAACGGTGAGATTTTG TATTTTAAAACGGTGAGATTTT TTATTTTAAAACGGTGAGATTT 100746855 rs112891767

HG00101 >hsa-mir-553 TTTAAAACGGTGAGATTTTGTT TTTTAAAACGGTGAGATTTTGT ATTTTAAAACGGTGAGATTTTG TATTTTAAAACGGTGAGATTTT TTATTTTAAAACGGTGAGATTT 100746848 . 100746855 rs112891767

HG00101 >hsa-mir-1227GGCATTTGACCCCGTGCCACCC AGGCATTTGACCCCGTGCCACC AGGCATTTGACCCCGTGCCACC TGACCCCGTGCCACCCTTTTCC ATTTGACCCCGTGCCACCCTTT 2234093 rs190788838

HG00101 >hsa-mir-1343GCCCCTCCTGGGGCCCGCACTC CCCCTCCTGGGGCCCGCACTCT GGGGCCCGCACTCTCGCTCTGG CCCTCCTGGGGCCCGCACTCTC TGGGGCCCGCACTCTCGCTCTG 34963416 rs2986407

HG00101 >hsa-mir-149 AGGGAGGGAGGGACGGGGGCTG GGGCTGTGCTGGGGCAGCCGGA GAGGGAGGGAGGGACGGGGGCT GGGAGGGAGGGACGGGGGCTGT GGGACGGGGGCTGTGCTGGGGC 241395503 rs2292832

HG00101 >hsa-mir-3118-1 TGAAAATTCTTCTAGTGTG ATGAAAATTCTTCTAGTGTG TGCATTATGAAAATTCTTCTAG TTATGAAAATTCTTCTAGTGTG ATTATGAAAATTCTTCTAGTGT 142667330 rs76132421

HG00101 >hsa-mir-3151CTGATCCCACACCCCACCTGTC TGATCCCACACCCCACCTGTCA GATCCCACACCCCACCTGTCAC GGGCATCCCACCTGATCCCACA TCCCACCTGATCCCACACCCCA 104166902 rs35605502

HG00101 >hsa-mir-3180-4 CTCCGGATGCCAGTCCCTCATC GGAGGGTGAAGCCTCCGGATGC AGCGGAGGGTGAAGCCTCCGGA CTGGCCTGGTCGCGCTGTGGCT GAGCGGAGGGTGAAGCCTCCGG 15248720

rs75000738
HG00101 >hsa-mir-3180-4 GGAGGGTGAAGCCTCCGGATGC
GGTGAAGCCTCCGGATGCCAGT GCGGAGGGTGAAGCCTCCGGAT
AGCGGAGGGTGAAGCCTCCGGA GCCTGGTCGCGCTGTGGCGAAG 15248798
rs183853838
HG00101 >hsa-mir-3180-4 GGAGGGTGAAGCCTCCGGATGC
GGTGAAGCCTCCGGATGCCAGT GCGGAGGGTGAAGCCTCCGGAT
AGCGGAGGGTGAAGCCTCCGGA CTGGCCTGGTCGCGCTGTGGCT 15248720
rs75000738 15248798 rs183853838
HG00101 >hsa-mir-320eGAAAAGCTGGGTTGAGAAGGT AAAAGCTGGGTTGAGAAGGT
GGAAAAGCTGGGTTGAGAAGGT GGGAAAAGCTGGGTTGAGAAGG rs10423365

HG00101 >hsa-mir-3622a CACCTGACCTCCCATGCCTGTG
CATGCCTGTGCACCCTCTATT CCTGACCTCCCATGCCTGTGCA
ATGCCTGTGCACCCTCTATT ACCTGACCTCCCATGCCTGTGC 27559214
rs66683138
HG00101 >hsa-mir-412 CTTCACCTGGTTCACTAGCCGT ACCTGGTTCACTAGCCGTCCGT
TGTACTTCACCTGGTTCACTAG CTGGTTCACTAGCCGTCCGTAT
GTACTTCACCTGGTTCACTAGC 101531849 .

HG00101 >hsa-mir-4268CTCTCAGGATGTGATGTCACCT CCTCTCAGGATGTGATGTCACC
GCTCCTCCTCTCAGGATGTGAT TCCTCTCAGGATGTGATGTCAC
CTCCTCCTCTCAGGATGTGATG 220771223 rs4674470

HG00101 >hsa-mir-580 TATTTGAGAATGATGAATCATT TGAATCATTAGGTTCCGGTCAG
ATGAATCATTAGGTTCCGGTCA TTTGAGAATGATGAATCATTAG
GAGAATGATGAATCATTAGGTT 36148057 rs115089112

HG00101 >hsa-mir-604 CACAGGCTGCGGAATTCAGGAC ACACAGGCTGCGGAATTCAGGA
ACAGGCTGCGGAATTCAGGACA GGCTGCGGAATTCAGGACAGTG
CTGCGGAATTCAGGACAGTGCA 29833998 rs2368393

HG00101 >hsa-mir-604 CACAGGCTGCGGAATTCAGGAC ACACAGGCTGCGGAATTCAGGA
ACAGGCTGCGGAATTCAGGACA GGCTGCGGAATTCAGGACAGTG
CTGCGGAATTCAGGACAGTGCA 29833998 rs2368393 29834003
rs2368392
HG00101 >hsa-mir-637 TGGCTAAGGTGTTGGCTCGGGC TGGCTAAGGTGTTGGCTCGGGC
.
HG00101 >hsa-mir-658 TAGGTCGGTTGGTCGGTCGGGA

HG00101 >hsa-mir-658 GTCCGTTGGTCGGTCGGGAACG TCCGTTGGTCGGTCGGGAACGA
.
HG00101 >hsa-mir-658 AGGTCGGTTGGTCGGTCGGGAA GTCGGTTGGTCGGTCGGGAACG
TAGGTCGGTTGGTCGGTCGGGA .

HG00101 >hsa-mir-744 ATGCACATGCTGTTGCCACTAA TGTTGCCACTAACCTCAACCTT
GCTGTTGCCACTAACCTCAACC TGCACATGCTGTTGCCACTAAC

11985275 .

HG00101 >hsa-mir-320c-1 AAAAGCTGGGTTGAGAGGGTAG AGCTGGGTTGAGAGGGTAGGAA CTGGGTTGAGAGGGTAGGAAAA AGGGTAGGAAAAAAATGATGTA 19263542 .

HG00101 >hsa-mir-202 AAAGAGGTATAGGGCATGGGAA AAGAGGTATAGGGCATGGGAAA GGGAAAACGGGGCGGTCGGGTC TAAAGAGGTATAGGGCATGGGA 135061112 rs12355840

HG00101 >hsa-mir-548ap AACAAAAACCACAATTACTTTT CAAAAACCACAATTACTTTTTA CAATTACTTTTTACTGACCTAA rs4414449

HG00101 >hsa-mir-548ap AACAAAAACCACAATTACTTTT CAAAAACCACAATTACTTTTTA TTACTTTTTACTGACCTAAAGA rs4577031

HG00101 >hsa-mir-548ap AACAAAAACCACAATTACTTTT CAAAAACCACAATTACTTTTTA CAATTACTTTTTACTGACCTAA 86368959 rs4414449

HG00101 >hsa-mir-1255b-2 AACCACTTTCTTTGCTCATCCG AAACCACTTTCTTTGCTCATCC CTTTCTTTGCTCATCCGTAAGG rs79639536

HG00101 >hsa-mir-3166AACGCAGACAATGCCTACTGGC AGACAATGCCTACTGGCCTAAG ATGCCTACTGGCCTAAGAAAAA CAATGCCTACTGGCCTAAGAAA 87909673 rs35854553

HG00101 >hsa-mir-597 ACAGTGGTTCTCTTGTGGCTTA GGCTTAAGCGTAATGTAGAGTA AATGTACAGTGGTTCTCTTGTG TGTACAGTGGTTCTCTTGTGGC 9599255 rs146125159

HG00101 >hsa-mir-449cACAGTTGCTAGTTGCACTCCTC AACAGTTGCTAGTTGCACTCCT GTTGCTAGTTGCACTCCTCTCT GTTGCACTCCTCTCTGTTGCAT 54468166 rs75661995

HG00101 >hsa-mir-3118-1 ACTGCATTATGAAAATTCTTCT ATTATGAAAATTCTTCTAGTGT GCATTATGAAAATTCTTCTAGT CTGCATTATGAAAATTCTTCTA 142667330 rs76132421

HG00101 >hsa-mir-605 AGAGAAGGCACTATGAGATTTA GGCACTATGAGATTTAGAACCA CAGAGAAGGCACTATGAGATTT GAGAAGGCACTATGAGATTTAG 53059406 rs2043556

HG00101 >hsa-mir-642bAGATACATTTGGAGAGGGACCC TTGGAGAGGGACCCTCCCAACT TTTGGAGAGGGACCCTCCCAAC ATACATTTGGAGAGGGACCCTC 46178217 rs111664333

HG00101 >hsa-mir-3180-4 AGCGGAGGGTGAAGCCTCCGGA CGGAGGGTGAAGCCTCCGGATG GAGCGGAGGGTGAAGCCTCCGG GCGGAGGGTGAAGCCTCCGGAT 15248798 rs183853838

HG00101 >hsa-mir-3180-4 AGCGGAGGGTGAAGCCTCCGGA CGGAGGGTGAAGCCTCCGGATG GAGCGGAGGGTGAAGCCTCCGG GCGGAGGGTGAAGCCTCCGGAT 15248720 rs75000738 15248798 rs183853838

HG00101 >hsa-mir-30d AGCTTTCAGTCAGATGTTTGCT GGCTAAGCTTTCAGTCAGATGT
GCTAAGCTTTCAGTCAGATGTT TTCAGTCAGATGTTTGCTGCTA 135817150
.
HG00101 >hsa-mir-519a-2 AGGAAAGTGCATCCTTTTAGAG
AGTGCATCCTTTTAGAGGGTTA GGAAAGTGCATCCTTTTAGAGG
GAAAGGAAAGTGCATCCTTTTA 54265670 .

HG00101 >hsa-mir-630 AGTATTCTGTACCAGGGAAGGT ACCTAGTATTCTGTACCAGGGA
CCAGGGAAGGTAGTTCTTAACT CAGGGAAGGTAGTTCTTAACTA 72879653
rs113971639
HG00101 >hsa-mir-513cATAAATTTCACCTTTCTGAGAA TTTCTGAGAAGAGTAATGTACA
CCTTTCTGAGAAGAGTAATGTA TTTCACCTTTCTGAGAAGAGTA 146271303
rs145416750
HG00101 >hsa-mir-323bATACACGGTCGACCTCTTTTCG TACACGGTCGACCTCTTTTCGG
ACACGGTCGACCTCTTTTCGGT rs56103835

HG00101 >hsa-mir-3144ATACCTGTTCAGTCTCTTTAAA TTCAGTCTCTTTAAAGTGTAGT
CCTGTTCAGTCTCTTTAAAGTG TGTTCAGTCTCTTTAAAGTGTA 120336384
rs67106263
HG00101 >hsa-mir-3144ATACCTGTTCAGTCTCTTTAAA TTCAGTCTCTTTAAAGTGTAGT
CTGTTCAGTCTCTTTAAAGTGT TATACCTGTTCAGTCTCTTTAA 120336327
rs68035463 120336384 rs67106263
HG00101 >hsa-mir-3144ATACCTGTTCGGTCTCTTTAAA CTGTTCGGTCTCTTTAAAGTGT
GTTCGGTCTCTTTAAAGTGTAG TGTTCGGTCTCTTTAAAGTGTA 120336327
rs68035463
HG00101 >hsa-mir-642aATTTGGAGAGGGAACCTCCCAA AGACACATTTGGAGAGGGAACC
ACACATTTGGAGAGGGAACCTC CACATTTGGAGAGGGAACCTCC 46178217
rs111664333
HG00101 >hsa-mir-548ac CAAAAACCGGCAATTACTTTTG
GGCAAAAACCGGCAATTACTTT TTACTTTTGCACTAACCTAATA
ACCGGCAATTACTTTTGCACTA 117102649 rs1414273

HG00101 >hsa-mir-4293CACACCAGCCTGACAGGAACAG TCACACCAGCCTGACAGGAACA
CACCAGCCTGACAGGAACAGCC CAGCCTGACAGGAACAGCCTGT 14425204
rs12780876
HG00101 >hsa-mir-604 CACAGGCTGCGGAATTCAGGAC TGACACAGGCTGCGGAATTCAG
GACACAGGCTGCGGAATTCAGG TTTCTGACACAGGCTGCGGAAT 29834003
rs2368392
HG00101 >hsa-mir-515-1 CAGAGTGCCTTCTTTTGGAGCA
GAGTGCCTTCTTTTGGAGCATT TGCCTTCTTTTGGAGCATTACT
GTGCCTTCTTTTGGAGCATTAC 54182326 rs374576826

HG00101 >hsa-mir-744 CATGCTGTTGCCACTAACCTCA GCTGTTGCCACTAACCTCAACC
CACTAACCTCAACCTTACTCGG .

HG00101 >hsa-mir-1227CATTTGACCCCGTGCCACCCTT ATTTGACCCCGTGCCACCCTTT
AGGCATTTGACCCCGTGCCACC GACCCCGTGCCACCCTTTTCCC 2234093

rs190788838
HG00101 >hsa-mir-3151CCACCTGATCCCACACCCCACC CACCTGATCCCACACCCCACCT CCCACCTGATCCCACACCCCAC TGATCCCACACCCCACCTGTCA 104166902 rs35605502
HG00101 >hsa-mir-1343CCCCTCCTGGGGCCCGCACTCT CCCTCCTGGGGCCCGCACTCTC CCTGGGGCCCGCACTCTCGCTC TGGGGCCCGCACTCTCGCTCTG 34963416 rs2986407
HG00101 >hsa-mir-1343CCCTCCTGGGGCGCGCACTCTC CTCCTGGGGCGCGCACTCTCGC GGGGCGCGCACTCTCGCTCTGG TGGGGCGCGCACTCTCGCTCTG 34963445 .
HG00101 >hsa-mir-3622b CCTCACCTGAGCTCCTGTGCCT CCTGAGCTCCTGTGCCTGTGCA CTCCTGTGCCTGTGCACCCTCT CTGAGCTCCTGTGCCTGTGCAC 27559214 rs66683138

HG00101 >hsa-mir-4268CCTCTCAGGATGTGATGTCACC CTCCTCTCAGGATGTGATGTCA CTCCTCCTCTCAGGATGTGATG rs4674470

HG00101 >hsa-mir-3622a CTCACCTGACCTCCCATGCCTG CCTGACCTCCCATGCCTGTGCA GCTCACCTGACCTCCCATGCCT CTGACCTCCCATGCCTGTGCAC 27559214 rs66683138

HG00101 >hsa-mir-3117CTCATATAGTGCCAGGTGTTTT GACTCATATAGTGCCAGGTGTT TCATATAGTGCCAGGTGTTTTG AGACTCATATAGTGCCAGGTGT 67094171 rs12402181
HG00101 >hsa-mir-1343CTCCTGGGGCGCGCACTCTCGC CCCTCCTGGGGCGCGCACTCTC GGGGCGCGCACTCTCGCTCTGG TGGGGCGCGCACTCTCGCTCTG 34963416 rs2986407 34963445 .
HG00101 >hsa-mir-196a-2 CTCGGCAACAAGAAACTGTCTG CAAGAAACTGTCTGAGTTACAT CAACAAGAAACTGTCTGAGTTA ACAAGAAACTGTCTGAGTTACA 54385599 rs11614913

HG00101 >hsa-mir-486-2 CTCGGCGCAGCTCAGTACAGGA AGGGCCTCGGCGCAGCTCAGTA TCGGCGCAGCTCAGTACAGGAT GGGCCTCGGCGCAGCTCAGTAC 41518007 .

HG00101 >hsa-mir-888 CTCTTTGGGTGAAGGAAGGCTC CTGACACCTCTTTGGGTGAAGG GACTGACACCTCTTTGGGTGAA CCTCTTTGGGTGAAGGAAGGCT 145076355 rs143634721
HG00101 >hsa-mir-1304CTGTAGCATCGAACCCCTGGGC CTCACTGTAGCATCGAACCCCT GAACCCCTGGGCTCAAGTGATT CGAACCCCTGGGCTCAAGTGAT 93466866 rs2155248
HG00101 >hsa-mir-2117CTGTTCTCTTTGCCAAGGACAG TAGCTGTTCTCTTTGCCAAGGA TCTCTTTGCCAAGGACAGATCT TGTTCTCTTTGCCAAGGACAGA 41522213 rs7207008
HG00101 >hsa-mir-940 GAAGGCAGGGCCCCCGCTCCCC AGGAAGGCAGGGCCCCCGCTCC AGGGCCCCCGCTCCCCGGGCCT rs373947923

HG00101 >hsa-mir-940 GAAGGCAGGGCCCC-GCTCCCC G CCC-GCTCCCCGGGCCTGACCC rs35356504

HG00101 >hsa-mir-940 GAAGGCAGGGCCCC-GCTCCCC G GGGCCCC-GCTCCCCGGGCCTG rs373947923 2321820 rs35356504

HG00101 >hsa-mir-1307GACTCGGCGTGGCGTCGGTCGT CGTGGCGTCGGTCGTGGTAGAT ATCGACTCGGCGTGGCGTCGGT CGACTCGGCGTGGCGTCGGTCG 105154089 rs7911488
HG00101 >hsa-mir-629 GAGGTTCTCCCAACGTAAGCCC AGGTTCTCCCAACGTAAGCCCA TCTCCCAACGTAAGCCCAGCCC CAGGAGGTTCTCCCAACGTAAG 70371761 rs377691713
HG00101 >hsa-mir-629 GAGGTTCTCCCAACGTAAGCCC AGGAGGTTCTCCCAACGTAAGC GGAGGTTCTCCCAACGTAAGCC TCTCCCAACGTAAGCCCAGCCC 70371794 .
HG00101 >hsa-mir-629 GAGGTTCTCCCAACGTAAGCCC AGGTTCTCCCAACGTAAGCCCA TCTCCCAACGTAAGCCCAGCCC CCCAACGTAAGCCCAGCCCCTC 70371761 rs377691713 70371794 .
HG00101 >hsa-mir-222 GCAGCTACATCTGGCTACTGGG TACTGGGTCTCTGATGGCATCT GCTACTGGGTCTCTGATGGCAT CTGGCTACTGGGTCTCTGATGG 45606504 rs191727254
HG00101 >hsa-mir-500bGCAGTGCACCCAGGCAAGGATT CACCCAGGCAAGGATTCTGCGA AGGCAAGGATTCTGCGAGGGGG TGCAGTGCACCCAGGCAAGGAT 49775351 rs151318590
HG00101 >hsa-mir-3180-4 GCGGAGGGTGAAGCCTCCGGAT CGCTGGCCTGGTCGCGCTGTGG TCGCTGGCCTGGTCGCGCTGTG AAGCCTCCGGATGCCAGTCCCT 15248720 rs75000738

HG00101 >hsa-mir-3176GGGACTGGCCTGGGACTACCGG GCCTGGGACTACCGGGGGTGGC ACTGGCCTGGGACTACCGGGGG CAGGGACTGGCCTGGGACTACC 593277 rs8054514
HG00101 >hsa-mir-149 GGGAGGGAGGGACGGGGGCTGT GGAGGGACGGGGGCTGTGCTGG AGGGACGGGGGCTGTGCTGGGG GAGGAGGGAGGGAGGGACGGGG 241395503 rs2292832
HG00101 >hsa-mir-1197GTAGGACACATGGTCTACTTCT ACACATGGTCTACTTCTTCTCA ACATGGTCTACTTCTTCTCAAT TAGGACACATGGTCTACTTCTT 101491923 rs141611518
HG00101 >hsa-mir-877 GTCCTCTTCTCCCTTCTCCCAG TGGGACCCTCAGACGTGTGTCC AGACGTGTGTCCTCTTCTCCCT CTGGGACCCTCAGACGTGTGTC 30552187 rs372113020
HG00101 >hsa-mir-27a GTGTTCACAGTGGCTAAGTTCC TCGTGTTCACAGTGGCTAAGTT AGTGGCTAAGTTCCGCCCCCCA TGTTCACAGTGGCTAAGTTCCG 13947292 rs895819
HG00101 >hsa-mir-3936TAAGGGGTGTATGGCAGATGCA TTCTGGTAAGGGGTGTATGGCA CACCCGACAGATGCACTTGGCA TGTATGGCAGATGCACCCGACA 131701279 rs367805
HG00101 >hsa-mir-300 TATACAAGGGCAGACTCTCTCT TGATTATACAAGGGCAGACTCT

ATTATACAAGGGCAGACTCTCT rs12894467

HG00101 >hsa-mir-412 TCACCTGGTTCACTAGCCGTCC TCACCTGGTTCACTAGCCGTCC
ATGTACTTCACCTGGTTCACTA CTTCACCTGGTTCACTAGCCGT 101531849
.
HG00101 >hsa-mir-595 TGAAGTGTGCCGTGGTGTGTCT GTGTGTCTGGAGGAAGCGCCTG
GCCGTGGTGTGTCTGGAGGAAG rs4909237

HG00101 >hsa-mir-604 TGACACAGGCTGCGGAATTCAG CACAGGCTGCGGAATTCAGGAC
GACACAGGCTGCGGAATTCAGG TTTCTGACACAGGCTGCGGAAT 29833998
rs2368393
HG00101 >hsa-mir-604 TGACACAGGCTGCGGAATTCAG GACACAGGCTGCGGAATTCAGG
CACAGGCTGCGGAATTCAGGAC TTTCTGACACAGGCTGCGGAAT 29833998
rs2368393 29834003 rs2368392
HG00101 >hsa-mir-1273h TGCTGCAGACTCGACCTCCCAG
TGCAGACTCGACCTCCCAGGCT CTGCAGACTCGACCTCCCAGGC
AGACTCGACCTCCCAGGCTTAA 24214486 .

HG00101 >hsa-mir-590 TGTAATTTTATGTATAAGCTAG AATCTGTAATTTTATGTATAAG
GTATAAGCTAGTCTCTGATTGA TTTTATGTATAAGCTAGTCTCT 73605546
rs189727189
HG00101 >hsa-mir-3909TGTCCTCTAGGGCCTGCAGTCT TCCTCTAGGGCCTGCAGTCTCA
TGGGAGAGTGACATGCACCAGG TAGGGCCTGCAGTCTCATGGGA 35731697
rs9607265
HG00101 >hsa-mir-2053TGTTAATTAAACCTCTATTTAC ACTTTAAGTGTTAATTAAACCT
TTTAAGTGTTAATTAAACCTCT GTTAATTAAACCTCTATTTACA 113655752
rs10505168
HG00101 >hsa-mir-585 TTGGGCGTATCTGTATGCTAGG TATCTGTATGCTAGGGCTGCCG
TGGGCGTATCTGTATGCTAGGG GCGTATCTGTATGCTAGGGCTG 168690612
rs62376934
HG00101 >hsa-mir-1303TTTAGAGACGGGGTCTTGCTCT TAGAGACGGGGTCTTGCTCTGT
TTAGAGACGGGGTCTTGCTCTG ACGGGGTCTTGCTCTGTTGCCA 154065383
rs75538180
HG00101 >hsa-mir-580 TTTGAGAATGATGAATCATTAG GATGAATCATTAGGTTCCGGTC
AATGATGAATCATTAGGTTCCG AGAATGATGAATCATTAGGTTC 36148057
rs115089112
HG00101 >hsa-mir-133bTTTGGTCCCCTTCAACCAGCTA TGGTCCCCTTCAACCAGCTACA
CCTTCAACCAGCTACAGCAGGG AGAGGTTTGGTCCCCTTCAACC 52013832
rs374103744
HG00102 >hsa-mir-1206CCTGGGAGGTCAAGGCTGTAGT

HG00102 >hsa-mir-1273h AGAATGGATAGAGGAAGCTGTG
TGGGAGGTCAAGGCTGTAGTGT GCCTGGGAGGTCAAGGCTGTAG
ATTGCTTGAGCCTGGGAGGTCA TTGAGCCTGGGAGGTCAAGGCT 24214486 .

HG00102 >hsa-mir-187 TGGGGTACGGGGATGGATGGTC

HG00102 >hsa-mir-212 TGGGGTACGGGGATGGATGGTC

HG00102 >hsa-mir-3125GAGGTACCTGGGACAGGAGGAG GAGGAAGCTGTGGAGAGAACTC AGAGGAAGCTGTGGAGAGAACT GCTGTGGAGAGAACTCACGGTG GGAAGCTGTGGAGAGAACTCAC 12877501 rs78852835

HG00102 >hsa-mir-412 TCTGCTGTTCCTCTGTCTCCCA GGATGGATGGTCGACCAGTTGG GATGGATGGTCGACCAGTTGGA TCGACCAGTTGGAAAGTAATTG ACGGGGATGGATGGTCGACCAG 101531854 rs61992671

HG00102 >hsa-mir-412 TTTTAAAACGGTGAGATTTTGT GGATGGATGGTCGACCAGTTGG GATGGATGGTCGACCAGTTGGA GGGTACGGGGATGGATGGTCGA TCGACCAGTTGGAAAGTAATTG 101531849 . 101531854 rs61992671

HG00102 >hsa-mir-4298TTTTATTTTAAAACGGTGAGAT GGAGGTACCTGGGACAGGAGGA GGGGAGGTACCTGGGACAGGAG GGGAGGTACCTGGGACAGGAGG rs75966923

HG00102 >hsa-mir-4326TTTTAAAACGGTGAGATTTTGT TGGTCTGCTGTTCCTCTGTCTC CTGGTCTGCTGTTCCTCTGTCT GCTGTTCCTCTGTCTCCCAGAC TGCTGTTCCTCTGTCTCCCAGA 61918164 rs6062431

HG00102 >hsa-mir-553 GGCCAAGGTGGGCCAGGGGTGG TATTTTAAAACGGTGAGATTTT TTTTATTTTAAAACGGTGAGAT TTTAAAACGGTGAGATTTTGTT ATTTTATTTTAAAACGGTGAGA 100746848 .

HG00102 >hsa-mir-553 TAGAGGAGATGGCGCAGGGGAC AATTTTATTTTAAAACGGTGAG TTTTAAAACGGTGAGATTTTGT TTATTTTAAAACGGTGAGATTT TTTAAAACGGTGAGATTTTGTT 100746855 rs112891767

HG00102 >hsa-mir-553 TGCCCTATTCAGAAAGGTGCCA TTTTATTTTAAAACGGTGAGAT TATTTTAAAACGGTGAGATTTT TTTAAAACGGTGAGATTTTGTT ATTTTATTTTAAAACGGTGAGA 100746848 . 100746855 rs112891767

HG00102 >hsa-mir-608 AAGGTGGGCCAGGGGTGGTGTT GGGGTGGTGTTGGGACAGCTGC TGGTGTTGGGACAGCTGCGTTT GGTGGGCCAGGGGTGGTGTTGG 102734778 rs4919510

HG00102 >hsa-mir-877 GAGGAGATGGCGCAGGGGACAC GAGATGGCGCAGGGGACACGGG GGAGATGGCGCAGGGGACACGG TGGCGCAGGGGACACGGGCAAA 30552187 rs372113020

HG00102 >hsa-mir-892c TCAGAAAGGTGCCAGTCACTTA AGTGCCCTATTCAGAAAGGTGC GTGCCCTATTCAGAAAGGTGCC GCCCTATTCAGAAAGGTGCCAG 145074284 .

HG00102 >hsa-mir-1307ACCGGACCTCGACCGGCTCGTC CGGACCTCGACCGGCTCGTCTG CCGGACCTCGACCGGCTCGTCT ATCTCGACCGGACCTCGACCGG

AATCTCGACCGGACCTCGACCG 105154089 rs7911488

HG00102 >hsa-mir-486-2 ACTGAGCTGCCCCGAGCTGGGC
CTGAGCTGCCCCGAGCTGGGCA CTGTACTGAGCTGCCCCGAGCT
CCTGTACTGAGCTGCCCCGAGC GTACTGAGCTGCCCCGAGCTGG 41518007 .

HG00102 >hsa-mir-1269b AGCCATGCTACGGGCTTCTCTG
ACTGAGCCATGCTACGGGCTTC AGGTTTCTGGACTGAGCCATGC
TGAGGTTTCTGGACTGAGCCAT TTTCTGGACTGAGCCATGCTAC 12820632
rs12451747
HG00102 >hsa-mir-1269b AGCCATGCTACGGGCTTCTCTG
AGTGAGCCATGCTACGGGCTTC AGGTTTCTGGAGTGAGCCATGC
TGAGGTTTCTGGAGTGAGCCAT TTTCTGGAGTGAGCCATGCTAC 12820632
rs12451747 12820646 rs7210937
HG00102 >hsa-mir-3135b AGCGAGTGCAGTGGTGCAGTCA
AGGCTGGAGCGAGTGCAGTGGT CTGGAGCGAGTGCAGTGGTGCA
CAGGCTGGAGCGAGTGCAGTGG CCAGGCTGGAGCGAGTGCAGTG 32717702
rs4285314
HG00102 >hsa-mir-3125AGCTGTGGAGAGAACTCACGGT AAGCTGTGGAGAGAACTCACGG
TAGAGGAAGCTGTGGAGAGAAC TGGATAGAGGAAGCTGTGGAGA
AGGAAGCTGTGGAGAGAACTCA 12877501 rs78852835

HG00102 >hsa-mir-612 AGGGCTTCTGAGCTCCTTAGCA GGGCTTCTGAGCTCCTTAGCAC
CAGGGCTTCTGAGCTCCTTAGC GCAGGGCTTCTGAGCTCCTTAG
GGCAGGGCTTCTGAGCTCCTTA 65211979 rs12803915

HG00102 >hsa-mir-548ap AGTAATTGCAGTCTTTGTCATT
AAGTAATTGCAGTCTTTGTCAT AAAGTAATTGCAGTCTTTGTCA
AAAAGTAATTGCAGTCTTTGTC CAAAAGTAATTGCAGTCTTTGT 86368898
rs4414449
HG00102 >hsa-mir-548ap AGTAATTGCAGTCTTTGTCATT
AAGTAATTGCAGTCTTTGTCAT AAAGTAATTGCAGTCTTTGTCA
AAAAGTAATTGCAGTCTTTGTC CAAAAGTAATTGCAGTCTTTGT 86368898
rs4414449 86368959 rs4577031
HG00102 >hsa-mir-548ap AGTAATTGCGGTCTTTGTCATT
AAGTAATTGCGGTCTTTGTCAT AAAGTAATTGCGGTCTTTGTCA
AAAAGTAATTGCGGTCTTTGTC CAAAAGTAATTGCGGTCTTTGT 86368959
rs4577031
HG00102 >hsa-mir-2278AGTGTGTGTTGCCTGGGGACTG AGCAGTGTGTGTTGCCTGGGGA
AGAGCAGTGTGTGTTGCCTGGG AGGTGTTGGAGAGCAGTGTGTG
ATGCTGCAGGTGTTGGAGAGCA 97572244 rs356125

HG00102 >hsa-mir-4298GAGGTACCTGGGACAGGAGGAG GGAGGTACCTGGGACAGGAGGA
GGGAGGTACCTGGGACAGGAGG GGGGAGGTACCTGGGACAGGAG rs75966923

HG00102 >hsa-mir-573 GTGTAACTGATCAGGATCTACT TGTGTAACTGATCAGGATCTAC
GATGTGTAACTGATCAGGATCT TGATGTGTAACTGATCAGGATC

GTGATGTGTAACTGATCAGGAT 24521902 rs76014664

HG00102 >hsa-mir-573 GTGTAACTGATCAGGATCTACT TGTGTAACTGATCAGGATCTAC
GATGTGTAACTGATCAGGATCT TGATGTGTAACTGATCAGGATC
GTGATGTGTAACTGATCAGGAT 24521904 rs78830737

HG00102 >hsa-mir-573 GTGTAACTGATCAGGATCTACT TGTGTAACTGATCAGGATCTAC
GATGTGTAACTGATCAGGATCT TGATGTGTAACTGATCAGGATC
GTGATGTGTAACTGATCAGGAT 24521902 rs76014664 24521904
rs78830737
HG00102 >hsa-mir-629 TACGTTGGGAGAACTTTTACGG TTACGTTGGGAGAACTTTTACG
GTTTACGTTGGGAGAACTTTTA TTTACGTTGGGAGAACTTTTAC
GGTTTACGTTGGGAGAACTTTT 70371794 .

HG00102 >hsa-mir-629 TACGTTGGGAGAACTTTTATGG TTACGTTGGGAGAACTTTTATG
TTTACGTTGGGAGAACTTTTAT TGGGTTTACGTTGGGAGAACTT
GTTTACGTTGGGAGAACTTTTA 70371761 rs377691713

HG00102 >hsa-mir-629 TACGTTGGGAGAACTTTTATGG TTACGTTGGGAGAACTTTTATG
TTTACGTTGGGAGAACTTTTAT TGGGTTTACGTTGGGAGAACTT
GTTTACGTTGGGAGAACTTTTA 70371761 rs377691713 70371794 .

HG00102 >hsa-mir-888 TACTCAAAAAGCTTTCAGTCAC TCTACTCAAAAAGCTTTCAGTC
TGCTCTACTCAAAAAGCTTTCA GCTCTACTCAAAAAGCTTTCAG
GGCAGTGCTCTACTCAAAAAGC 145076355 rs143634721

HG00102 >hsa-mir-3199-1 TAGGAGAAAGTTTCTGGAAGTT
TTAGGAGAAAGTTTCTGGAAGT TGCCTTAGGAGAAAGTTTCTGG
TCCAGGGACTGCCTTAGGAGAA TGACTCCAGGGACTGCCTTAGG 28316513
rs118160653
HG00102 >hsa-mir-449cTAGGCAGTGTATTGCTAGCGGC TCAGATAGGCAGTGTATTGCTA
TGTCAGATAGGCAGTGTATTGC TGTGTCAGATAGGCAGTGTATT
TGGGATGTGTCAGATAGGCAGT 54468166 rs75661995

HG00102 >hsa-mir-892cTCAGAAAGGTGCCAGTCACTTA TTCAGAAAGGTGCCAGTCACTT
TATTCAGAAAGGTGCCAGTCAC TGCCCTATTCAGAAAGGTGCCA
GCCCTATTCAGAAAGGTGCCAG 145074284 .

HG00102 >hsa-mir-564 TCAGCAGGCAACATGGCCGAGA TGTCAGCAGGCAACATGGCCGA
GTCAGCAGGCAACATGGCCGAG TGCCAGGCACGGTGTCAGCAGG
GTGTCAGCAGGCAACATGGCCG 44903433 .

HG00102 >hsa-mir-937 TCAGGGTGGGGCTGGCCCCCTG TGAGTCAGGGTGGGGCTGGCCC
TGCCCCCGGTGAGTCAGGGTGG GTCAGGGTGGGGCTGGCCCCCT
GAGTCAGGGTGGGGCTGGCCCC 144895168 rs200654758

HG00102 >hsa-mir-222 TCAGTAGCCAGTGTAGATCCTG TGGCTCAGTAGCCAGTGTAGAT

TTGGCTCAGTAGCCAGTGTAGA TCATTGGCTCAGTAGCCAGTGT
TACCCTCATTGGCTCAGTAGCC 45606504 rs191727254

HG00102 >hsa-mir-515-1 TCCAAAAGAAAGCACTTTCTGT
TCTCCAAAAGAAAGCACTTTCT TTCTCCAAAAGAAAGCACTTTC
TCATTCTCCAAAAGAAAGCACT TGCAGTCATTCTCCAAAAGAAA 54182326
rs374576826
HG00102 >hsa-mir-663aTCCCAGGCGGGGCGCCGCGGGA TCCGGCGTCCCAGGCGGGGCGC
TTCCGGCGTCCCAGGCGGGGCG GCGCCGCGGGACCTCCCTCGTG
GGCGCCGCGGGACCTCCCTCGT 26188880 .

HG00102 >hsa-mir-618 TCCTTCTGAGTGTAATTACGTA TGTCCTTCTGAGTGTAATTACG
TTGTCCTTCTGAGTGTAATTAC TACTTGTCCTTCTGAGTGTAAT
GTCCTTCTGAGTGTAATTACGT 81329536 rs2682818

HG00102 >hsa-mir-3183TCGGAGTCGCTCGGAGCAGCCA TCTCGGAGTCGCTCGGAGCAGC
TCTCTCGGAGTCGCTCGGAGCA TCTGCCCTGCCTCTCTCGGAGT
TGCCCTGCCTCTCTCGGAGTCG 925764 rs2663345

HG00102 >hsa-mir-4305TCTGGGTTCTTAGAGGCCTAAT TTCTGGGTTCTTAGAGGCCTAA
GTTCTGGGTTCTTAGAGGCCTA TCCAGTTCTGGGTTCTTAGAGG
CAGTTCTGGGTTCTTAGAGGCC 40238175 rs67976778

HG00102 >hsa-mir-635 TGAAACAATGTCCATTAGGCTT GAAACAATGTCCATTAGGCTTT
ACAATGTCCATTAGGCTTTGTT AACAATGTCCATTAGGCTTTGT
CTGAAACAATGTCCATTAGGCT 66420592 rs77279010

HG00102 >hsa-mir-3612TGAGGAGGCATCTTGAGAAATG TGGGGATGAGGAGGCATCTTGA
GAGGCATCTTGAGAAATGGAAG GGAGGCATCTTGAGAAATGGAA
GAGGAGGCATCTTGAGAAATGG 128778703 rs1683709

HG00102 >hsa-mir-1269b TGAGGTTTCTGGAGTGAGCCAT
TTTCTGGAGTGAGCCATGCTAC TTCTGGAGTGAGCCATGCTACT
TCTGGAGTGAGCCATGCTACTG TGGAGTGAGCCATGCTACTGGC 12820646
rs7210937
HG00102 >hsa-mir-3151TGATGGGTGGGGCAATGGGATC TGGGTGGGGCAATGGGATCAGG
TGGGGCAATGGGATCAGGTGCC GGGGTGATGGGTGGGGCAATGG
GGGTGATGGGTGGGGCAATGGG 104166902 rs35605502

HG00102 >hsa-mir-3156-2 TGCAGAAGAAAGATCTGGAAGT
GAAAGATCTGGAAGTGGGAGAC GAAGAAAGATCTGGAAGTGGGA
GCAGAAGAAAGATCTGGAAGTG CAGAAGAAAGATCTGGAAGTGG 14830215
rs113478966
HG00102 >hsa-mir-3156-3 TGCAGAAGAAAGATCTGGAAGT
GCAGAAGAAAGATCTGGAAGTG GAAGAAAGATCTGGAAGTGGGA
GAAAGATCTGGAAGTGGGAGAC AGAAGAAAGATCTGGAAGTGGG 14778721
rs2747232

HG00102 >hsa-mir-1200TGCTACTTCTCCTGAGCCATTC TGAGCCATTCTGAGCCTCAGTC TACTTCTCCTGAGCCATTCTGA TCCTGAGCCATTCTGAGCCTCA TTCTCCTGAGCCATTCTGAGCC 36959006 .

HG00102 >hsa-mir-1254-2 TGGAAGCTGGAGCCTGCAGTGA TGAGCCTGGAAGCTGGAGCCTG GAAGCTGGAGCCTGCAGTGAGC GGAAGCTGGAGCCTGCAGTGAG GCCTGGAAGCTGGAGCCTGCAG 23682383 rs200793185

HG00102 >hsa-mir-877 TGGCGCAGGGGACACGGGCAAA GACACGGGCAAAGACTTGGGGG GGACACGGGCAAAGACTTGGGG GGGACACGGGCAAAGACTTGGG GGGGACACGGGCAAAGACTTGG 30552187 rs372113020

HG00102 >hsa-mir-608 TGGGACAGCTGCGTTTAAAAAG TTGGGACAGCTGCGTTTAAAAA GGACAGCTGCGTTTAAAAAGGC TGTTGGGACAGCTGCGTTTAAA GGGACAGCTGCGTTTAAAAAGG 102734778 rs4919510

HG00102 >hsa-mir-1273h TGGGAGGTCAAGGCTGTAGTGT TGAGCCTGGGAGGTCAAGGCTG TTGAGCCTGGGAGGTCAAGGCT TGCTTGAGCCTGGGAGGTCAAG TTGCTTGAGCCTGGGAGGTCAA 24214486 .

HG00102 >hsa-mir-3175TGGGGGGCGGGGAGAGAACGCA GAGAGAACGCAGTGACGTCTGG GGAGAGAACGCAGTGACGTCTG GGGAGAGAACGCAGTGACGTCT GGGGAGAGAACGCAGTGACGTC 93447700 .

HG00102 >hsa-mir-412 TGGGGTACGGGGATGGATGGTC TCGACCAGTTGGAAAGTAATTG TGGTCGACCAGTTGGAAAGTAA TACGGGGATGGATGGTCGACCA TGGATGGTCGACCAGTTGGAAA 101531854 rs61992671

HG00102 >hsa-mir-412 TGGGGTACGGGGATGGATGGTC TCGACCAGTTGGAAAGTAATTG TGGTCGACCAGTTGGAAAGTAA TACGGGGATGGATGGTCGACCA TGGATGGTCGACCAGTTGGAAA 101531849 . 101531854 rs61992671

HG00102 >hsa-mir-1227TGGTGGGCACTGCTGGGGTGGG TGGGGCCAGGCGGTGGTGGGCA AGGCGGTGGTGGGCACTGCTGG GGTGGGCACTGCTGGGGTGGGC GTGGTGGGCACTGCTGGGGTGG 2234093 rs190788838

HG00102 >hsa-mir-323bTGTCCGTGGTGAGTTCGCATTA TTGTCCGTGGTGAGTTCGCATT TACTCGGAGGGAGGTTGTCCGT TCGGAGGGAGGTTGTCCGTGGT AGGTTGTCCGTGGTGAGTTCGC 101522556 rs56103835

HG00102 >hsa-mir-642bTTCCCTCTCCAAATGTGTCTTG TTGGGAGGTTCCCTCTCCAAAT TGGGAGGTTCCCTCTCCAAATG GAGTTGGGAGGTTCCCTCTCCA GTTGGGAGGTTCCCTCTCCAAA 46178217 rs111664333

HG00102 >hsa-mir-4277TTCTGAGCACAGTACACTGGGC TCGAGGCAGTTCTGAGCACAGT TGGGTCGAGGCAGTTCTGAGCA GTTCTGAGCACAGTACACTGGG GCAGTTCTGAGCACAGTACACT 1708902 rs115200817

HG00102 >hsa-mir-4277TTCTGAGCACAGTACACTGGGC TCGAGGCAGTTCTGAGCACAGT TGGGTCGAGGCAGTTCTGAGCA TTGGGTCGAGGCAGTTCTGAGC GTTCTGAGCACAGTACACTGGG 1708983 rs12523324

HG00102 >hsa-mir-4277TTCTGAGCACAGTACACTGGGC TCGAGGCAGTTCTGAGCACAGT TGGGTCGAGGCAGTTCTGAGCA TTGGGTCGAGGCAGTTCTGAGC GTTCTGAGCACAGTACACTGGG 1708902 rs115200817 1708983 rs12523324

HG00102 >hsa-mir-553 TTTAAAACGGTGAGATTTTGTT TTTTAAAACGGTGAGATTTTGT ATTTTAAAACGGTGAGATTTTG TATTTTAAAACGGTGAGATTTT TTATTTTAAAACGGTGAGATTT 100746848 .

HG00102 >hsa-mir-553 TTTAAAACGGTGAGATTTTGTT TTTTAAAACGGTGAGATTTTGT ATTTTAAAACGGTGAGATTTTG TATTTTAAAACGGTGAGATTTT TTATTTTAAAACGGTGAGATTT 100746855 rs112891767

HG00102 >hsa-mir-553 TTTAAAACGGTGAGATTTTGTT TTTTAAAACGGTGAGATTTTGT ATTTTAAAACGGTGAGATTTTG TATTTTAAAACGGTGAGATTTT TTATTTTAAAACGGTGAGATTT 100746848 . 100746855 rs112891767

HG00102 >hsa-mir-661 ACTTTGGGGTGGCT GACTTTGGGGTGGCT TGGGTCTCTGGCCTGCGTGTGA CTGGGTCTCTGGCCTGCGTGTG CCTGGGTCTCTGGCCTGCGTGT 145019376 .

HG00102 >hsa-mir-646 AGCAGCTGCCTCGGAGGCCTCA CGGAGGCCTCAGGCTCAGTGGC TCGGAGGCCTCAGGCTCAGTGG GAGGAAGCAGCTGCCTCGGAGG AGGAAGCAGCTGCCTCGGAGGC 58883605 rs6513497

HG00102 >hsa-mir-646 AGCAGCTGCCTCGGAGGCCTCA CGGAGGCCTCAGGCTCAGTGGC CTCGGAGGCCTCAGGCTCAGTG TCGGAGGCCTCAGGCTCAGTGG GCCTCGGAGGCCTCAGGCTCAG 58883534 rs6513496 58883605 rs6513497

HG00102 >hsa-mir-646 AGCAGCTGCCTCTGAGGCCTCA CTGAGGCCTCAGGCTCAGTGGC CTCTGAGGCCTCAGGCTCAGTG TCTGAGGCCTCAGGCTCAGTGG GCCTCTGAGGCCTCAGGCTCAG 58883534 rs6513496

HG00102 >hsa-mir-658 AGGTCGGTTGGTCGGTCGGGAA GTCGGTTGGTCGGTCGGGAACG TAGGTCGGTTGGTCGGTCGGGA . .

HG00102 >hsa-mir-412 CGTCCGTATCCGCTGCAG CCGTCCGTATCCGCTGCAG TCACCTGGTCCACTGGCCGTCC ACCTGGTCCACTGGCCGTCCGT CACCTGGTCCACTGGCCGTCCG 101531854 rs61992671

HG00102 >hsa-mir-412 CGTCCGTATCCGCTGCAG CCGTCCGTATCCGCTGCAG TCACCTGGTTCACTGGCCGTCC ACCTGGTTCACTGGCCGTCCGT CACCTGGTTCACTGGCCGTCCG 101531849 . 101531854 rs61992671

HG00102 >hsa-mir-3180-4 CTCCGGATGCCAGTCCCTCATC
GGAGGGTGAAGCCTCCGGATGC CTGGCCTGGTCGCGCTGTGGCT
AGCGGAGGGTGAAGCCTCCGGA GAGCGGAGGGTGAAGCCTCCGG 15248720
rs75000738
HG00102 >hsa-mir-4268CTCTCAGGATGTGATGTCACCT CCTCTCAGGATGTGATGTCACC
GCTCCTCCTCTCAGGATGTGAT TCCTCTCAGGATGTGATGTCAC
CTCCTCCTCTCAGGATGTGATG 220771223 rs4674470

HG00102 >hsa-mir-3151CTGATCCCACACCCCACCTGTC TGATCCCACACCCCACCTGTCA
GATCCCACACCCCACCTGTCAC GGGCATCCCACCTGATCCCACA
TCCCACCTGATCCCACACCCCA 104166902 rs35605502

HG00102 >hsa-mir-412 CTTCACCTGGTTCACTAGCCGT ACCTGGTTCACTAGCCGTCCGT
CTGGTTCACTAGCCGTCCGTAT TGTACTTCACCTGGTTCACTAG
GTACTTCACCTGGTTCACTAGC 101531849 .

HG00102 >hsa-mir-658 TAGGTCGGTTGGTCGGTCGGGA

HG00102 >hsa-mir-658 GTCCGTTGGTCGGTCGGGAACG TCCGTTGGTCGGTCGGGAACGA
.
HG00102 >hsa-mir-320eGAAAAGCTGGGTTGAGAAGGT AAAAGCTGGGTTGAGAAGGT
GGAAAAGCTGGGTTGAGAAGGT GGGAAAAGCTGGGTTGAGAAGG rs10423365

HG00102 >hsa-mir-1343GCCCCTCCTGGGGCCCGCACTC CCCCTCCTGGGGCCCGCACTCT
GGGGCCCGCACTCTCGCTCTGG CCCTCCTGGGGCCCGCACTCTC
TGGGGCCCGCACTCTCGCTCTG 34963416 rs2986407

HG00102 >hsa-mir-3180-4 GGAGGGTGAAGCCTCCGGATGC
GGTGAAGCCTCCGGATGCCAGT AGCGGAGGGTGAAGCCTCCGGA
GCGGAGGGTGAAGCCTCCGGAT GCCTGGTCGCGCTGTGGCGAAG 15248798
rs183853838
HG00102 >hsa-mir-3180-4 GGAGGGTGAAGCCTCCGGATGC
GGTGAAGCCTCCGGATGCCAGT AGCGGAGGGTGAAGCCTCCGGA
GCGGAGGGTGAAGCCTCCGGAT CTGGCCTGGTCGCGCTGTGGCT 15248720
rs75000738 15248798 rs183853838
HG00102 >hsa-mir-1227GGCATTTGACCCCGTGCCACCC AGGCATTTGACCCCGTGCCACC
AGGCATTTGACCCCGTGCCACC TGACCCCGTGCCACCCTTTTCC
ATTTGACCCCGTGCCACCCTTT 2234093 rs190788838

HG00102 >hsa-mir-580 TATTTGAGAATGATGAATCATT TGAATCATTAGGTTCCGGTCAG
ATGAATCATTAGGTTCCGGTCA GAGAATGATGAATCATTAGGTT
TTTGAGAATGATGAATCATTAG 36148057 rs115089112

HG00102 >hsa-mir-3118-1 TGAAAATTCTTCTAGTGTG ATGAAAATTCTTCTAGTGTG
TGCATTATGAAAATTCTTCTAG ATTATGAAAATTCTTCTAGTGT
GCATTATGAAAATTCTTCTAGT 142667330 rs76132421

HG00102 >hsa-mir-558 TGAGCTGCTGTACCAAAATACC GCTGCTGTACCAAAATACCACA TGCTGTACCAAAATACCACAAA CTGAGCTGCTGTACCAAAATAC GAGCTGCTGTACCAAAATACCA 32757230 rs72089144

HG00102 >hsa-mir-637 TGGCTAAGGTGTTGGCTCGGGC TGGCTAAGGTGTTGGCTCGGGC .

HG00102 >hsa-mir-320c-1 AAAAGCTGGGTTGAGAGGGTAG AGCTGGGTTGAGAGGGTAGGAA CTGGGTTGAGAGGGTAGGAAAA AGGGTAGGAAAAAAATGATGTA 19263542 .

HG00102 >hsa-mir-202 AAAGAGGTATAGGGCATGGGAA AAGAGGTATAGGGCATGGGAAA GGGAAAACGGGGCGGTCGGGTC TGGGAAAACGGGGCGGTCGGGT 135061112 rs12355840

HG00102 >hsa-mir-520hAAAGTGCTTCCCTTTAGAGTTA rs148716001

HG00102 >hsa-mir-548ap AACAAAAACCACAATTACTTTT CAAAAACCACAATTACTTTTTA CAATTACTTTTTACTGACCTAA rs4414449

HG00102 >hsa-mir-548ap AACAAAAACCACAATTACTTTT CAAAAACCACAATTACTTTTTA TTACTTTTTACTGACCTAAAGA rs4577031

HG00102 >hsa-mir-548ap AACAAAAACCACAATTACTTTT CAAAAACCACAATTACTTTTTA CAATTACTTTTTACTGACCTAA rs4414449 86368959 rs4577031

HG00102 >hsa-mir-3166AACGCAGACAATGCCTACTGGC AGACAATGCCTACTGGCCTAAG ATGCCTACTGGCCTAAGAAAAA CAATGCCTACTGGCCTAAGAAA 87909673 rs35854553

HG00102 >hsa-mir-449cACAGTTGCTAGTTGCACTCCTC AACAGTTGCTAGTTGCACTCCT GTTGCTAGTTGCACTCCTCTCT GTTGCACTCCTCTCTGTTGCAT 54468166 rs75661995

HG00102 >hsa-mir-544bACCTGAGGTTGTGCATTTCTAA AGACCTGAGGTTGTGCATTTCT TAGACCTGAGGTTGTGCATTTC GAGGTTGTGCATTTCTAACAAA 124451312 rs10934682

HG00102 >hsa-mir-3118-1 ACTGCATTATGAAAATTCTTCT ATTATGAAAATTCTTCTAGTGT GCATTATGAAAATTCTTCTAGT CTGCATTATGAAAATTCTTCTA 142667330 rs76132421

HG00102 >hsa-mir-642bAGATACATTTGGAGAGGGACCC TTGGAGAGGGACCCTCCCAACT TTTGGAGAGGGACCCTCCCAAC ATACATTTGGAGAGGGACCCTC 46178217 rs111664333

HG00102 >hsa-mir-3180-4 AGCGGAGGGTGAAGCCTCCGGA CGGAGGGTGAAGCCTCCGGATG GAGCGGAGGGTGAAGCCTCCGG GCGGAGGGTGAAGCCTCCGGAT 15248798 rs183853838

HG00102 >hsa-mir-3180-4 AGCGGAGGGTGAAGCCTCCGGA CGGAGGGTGAAGCCTCCGGATG GAGCGGAGGGTGAAGCCTCCGG

GCGGAGGGTGAAGCCTCCGGAT 15248720 rs75000738 15248798 rs183853838
HG00102 >hsa-mir-30d AGCTTTCAGTCAGATGTTTGCT GGCTAAGCTTTCAGTCAGATGT GCTAAGCTTTCAGTCAGATGTT TTCAGTCAGATGTTTGCTGCTA 135817150 .
HG00102 >hsa-mir-519a-2 AGGAAAGTGCATCCTTTTAGAG AGTGCATCCTTTTAGAGGGTTA GGAAAGTGCATCCTTTTAGAGG GAAAGGAAAGTGCATCCTTTTA 54265670 .

HG00102 >hsa-mir-646 AGGAAGCAGCTGCCTCGGAGGC CCAGGAGAGGAAGCAGCTGCCT GCTGCCTCGGAGGCCTCAGGCT GAGAGGAAGCAGCTGCCTCGGA 58883605 rs6513497
HG00102 >hsa-mir-646 AGGAAGCAGCTGCCTCGGAGGC TGCCTCGGAGGCCTCAGGCTCA CGGAGGCCTCAGGCTCAGTGGC TCGGAGGCCTCAGGCTCAGTGG 58883534 rs6513496 58883605 rs6513497
HG00102 >hsa-mir-646 AGGAAGCAGCTGCCTCTGAGGC GCTGCCTCTGAGGCCTCAGGCT CTGAGGCCTCAGGCTCAGTGGC TCTGAGGCCTCAGGCTCAGTGG 58883534 rs6513496
HG00102 >hsa-mir-630 AGTATTCTGTACCAGGGAAGGT ACCTAGTATTCTGTACCAGGGA GGAAGGTAGTTCTTAACTATGT rs113971639

HG00102 >hsa-mir-3686AGTGATCTGTAAGAGAAAGTAA TCTGTAAGAGAAAGTAAATGAA GTAAGAGAAAGTAAATGAAAGA CAGTGATCTGTAAGAGAAAGTA 130496365 rs6997249
HG00102 >hsa-mir-513cATAAATTTCACCTTTCTGAGAA TTCTGAGAAGAGTAATGTACAG CCTTTCTGAGAAGAGTAATGTA TTTCACCTTTCTGAGAAGAGTA 146271303 rs145416750
HG00102 >hsa-mir-323bATACACGGTCGACCTCTTTTCG TACACGGTCGACCTCTTTTCGG ACACGGTCGACCTCTTTTCGGT rs56103835

HG00102 >hsa-mir-642aATTTGGAGAGGGAACCTCCCAA AGACACATTTGGAGAGGGAACC ACACATTTGGAGAGGGAACCTC CACATTTGGAGAGGGAACCTCC 46178217 rs111664333
HG00102 >hsa-mir-4293CACACCAGCCTGACAGGAACAG CACCAGCCTGACAGGAACAGCC TCACACCAGCCTGACAGGAACA CAGCCTGACAGGAACAGCCTGT 14425204 rs12780876
HG00102 >hsa-mir-412 CACCTGGTCCACTGGCCGTCCG ACCTGGTCCACTGGCCGTCCGT CTGGCCGTCCGTATCCGCTGCA CCTGGTCCACTGGCCGTCCGTA 101531854 rs61992671
HG00102 >hsa-mir-412 CACCTGGTTCACTGGCCGTCCG ACCTGGTTCACTGGCCGTCCGT CTGGCCGTCCGTATCCGCTGCA CCTGGTTCACTGGCCGTCCGTA 101531849 . 101531854 rs61992671
HG00102 >hsa-mir-515-1 CAGAGTGCCTTCTTTTGGAGCA GAGTGCCTTCTTTTGGAGCATT TGCCTTCTTTTGGAGCATTACT GTGCCTTCTTTTGGAGCATTAC 54182326 rs374576826

HG00102 >hsa-mir-2861CAGGGGGCCTGGCGGTGGGCGG GCCTGGCGGTGGGCGGCGGGCG

AGGGGGCCTGGCGGTGGGCGGC GGCCTGGCGGTGGGCGGCGGGC 130548199
.
HG00102 >hsa-mir-1227CATTTGACCCCGTGCCACCCTT ATTTGACCCCGTGCCACCCTTT
AGGCATTTGACCCCGTGCCACC GACCCCGTGCCACCCTTTTCCC 2234093
rs190788838
HG00102 >hsa-mir-3151CCACCTGATCCCACACCCCACC CACCTGATCCCACACCCCACCT
CCCACCTGATCCCACACCCCAC TGATCCCACACCCCACCTGTCA 104166902
rs35605502
HG00102 >hsa-mir-188 CCCCTCCCATATGCAGGGTTTG ACCCCTCCCATATGCAGGGTTT
CCATATGCAGGGTTTGCAGGAT CCCTCCCATATGCAGGGTTTGC 49768168
rs191840972
HG00102 >hsa-mir-1343CCCCTCCTGGGGCCCGCACTCT CCCTCCTGGGGCCCGCACTCTC
CCTGGGGCCCGCACTCTCGCTC TGGGGCCCGCACTCTCGCTCTG 34963416
rs2986407
HG00102 >hsa-mir-4254CCTGGAGATACTCCACCATCTC AGATACTCCACCATCTCCCCCA
GGAGATACTCCACCATCTCCCC rs12731294

HG00102 >hsa-mir-182 CGGTGGTTCTAGACTTGCCAAC GTGGTTCTAGACTTGCCAACTA
CCGGTGGTTCTAGACTTGCCAA GCCAACTATGGGGCAAGGACTC 129410239
rs80041074
HG00102 >hsa-mir-486-2 CTCGGCGCAGCTCAGTACAGGA
AGGGCCTCGGCGCAGCTCAGTA TCGGCGCAGCTCAGTACAGGAT
GGGCCTCGGCGCAGCTCAGTAC 41518007 .

HG00102 >hsa-mir-3615CTCTCTCGGCTCCTCGCGGCTC GGCTCCTCGCGGCTCGCGGCGG
CGGCTCCTCGCGGCTCGCGGCG TCGGCTCCTCGCGGCTCGCGGC 72744798
rs745666
HG00102 >hsa-mir-888 CTCTTTGGGTGAAGGAAGGCTC CTGACACCTCTTTGGGTGAAGG
GACTGACACCTCTTTGGGTGAA CCTCTTTGGGTGAAGGAAGGCT 145076355
rs143634721
HG00102 >hsa-mir-1269a CTGGACTGAGCCATGCTACTGG
TGCCTGGACTGAGCCATGCTAC AATGCCTGGACTGAGCCATGCT
rs73239138
HG00102 >hsa-mir-4274GACCCAGCAGTCCCTCCCCCTG CCCAGCAGTCCCTCCCCCTGCA
TGACCCAGCAGTCCCTCCCCCT TCAGGTGACCCAGCAGTCCCTC 7461769
rs12512664
HG00102 >hsa-mir-1307GACTCGGCGTGGCGTCGGTCGT CGTGGCGTCGGTCGTGGTAGAT
ATCGACTCGGCGTGGCGTCGGT CGACTCGGCGTGGCGTCGGTCG 105154089
rs7911488
HG00102 >hsa-mir-3188GAGGCTTTGTGCGGATACGGGG GAGAGGCTTTGTGCGGATACGG
GCGGATACGGGGCTGGAGGCCT rs7247237

HG00102 >hsa-mir-3188GAGGCTTTGTGCGGATACGGGG GAGAGGCTTTGTGCGGATACGG
AGAGGCTTTGTGCGGATACGGG rs7247767

HG00102 >hsa-mir-3188GAGGCTTTGTGCGGATACGGGG GAGAGGCTTTGTGCGGATACGG
GGAGAGGCTTTGTGCGGATACG rs7247237 18392913

rs7247767
HG00102	>hsa-mir-629 GAGGTTCTCCCAACGTAAGCCC	AGGTTCTCCCAACGTAAGCCCA
TCTCCCAACGTAAGCCCAGCCC	CAGGAGGTTCTCCCAACGTAAG	70371761
rs377691713
HG00102	>hsa-mir-629 GAGGTTCTCCCAACGTAAGCCC	AGGAGGTTCTCCCAACGTAAGC
GGAGGTTCTCCCAACGTAAGCC	TCTCCCAACGTAAGCCCAGCCC	70371794
.
HG00102	>hsa-mir-629 GAGGTTCTCCCAACGTAAGCCC	AGGTTCTCCCAACGTAAGCCCA
TCTCCCAACGTAAGCCCAGCCC	CCCAACGTAAGCCCAGCCCCTC	70371761
rs377691713 70371794	.
HG00102	>hsa-mir-222 GCAGCTACATCTGGCTACTGGG	TACTGGGTCTCTGATGGCATCT
GCTACTGGGTCTCTGATGGCAT	CTGGCTACTGGGTCTCTGATGG	45606504
rs191727254
HG00102	>hsa-mir-937 GCCCATCCGCGCTCTGACTCTC	TGCCCATCCGCGCTCTGACTCT
CTCTCTGCCCACCTGCAGGAGC	TCTGACTCTCTGCCCACCTGCA	144895168
rs200654758
HG00102	>hsa-mir-151bGCCCTCGAGGAGCTCACAGTCT	TCTAGACAAACAAACTCAGGGT
AGCTCACAGTCTAGACAAACAA

HG00102	>hsa-mir-3180-4	GCGGAGGGTGAAGCCTCCGGAT
CGCTGGCCTGGTCGCGCTGTGG	TCGCTGGCCTGGTCGCGCTGTG
AAGCCTCCGGATGCCAGTCCCT	15248720	rs75000738

HG00102	>hsa-mir-3176GGGACTGGCCTGGGACTACCGG	GCCTGGGACTACCGGGGGTGGC
ACTGGCCTGGGACTACCGGGGG	CAGGGACTGGCCTGGGACTACC	593277
rs8054514
HG00102	>hsa-mir-1197GTAGGACACATGGTCTACTTCT	ACACATGGTCTACTTCTTCTCA
TAGGACACATGGTCTACTTCTT	ACATGGTCTACTTCTTCTCAAT	101491923
rs141611518
HG00102	>hsa-mir-450a-2	GTATTGCGGACATTTTGCATTC
GCGGACATTTTGCATTCATAGT	TTGCGGACATTTTGCATTCATA
GACATTTTGCATTCATAGTTTT	133674576	rs185256782

HG00102	>hsa-mir-877 GTCCTCTTCTCCCTTCTCCCAG	TGGGACCCTCAGACGTGTGTCC
AGACGTGTGTCCTCTTCTCCCT	GTGTCCTCTTCTCCCTTCTCCC	30552187
rs372113020
HG00102	>hsa-mir-3936TAAGGGGTGTATGGCAGATGCA	CACCCGACAGATGCACTTGGCA
GATGCACCCGACAGATGCACTT	TGTATGGCAGATGCACCCGACA	131701279
rs367805
HG00102	>hsa-mir-300 TATACAAGGGCAGACTCTCTCT	TGATTATACAAGGGCAGACTCT
ATTATACAAGGGCAGACTCTCT	rs12894467

HG00102	>hsa-mir-412 TCACCTGGTTCACTAGCCGTCC	TCACCTGGTTCACTAGCCGTCC
ATGTACTTCACCTGGTTCACTA	CTTCACCTGGTTCACTAGCCGT	101531849
.
HG00102	>hsa-mir-589 TCAGAACAAATGCCGGTTCCCA	TGCCGGTTCCCAGATGCTGCCA
CAAATGCCGGTTCCCAGATGCT	TTCAGAACAAATGCCGGTTCCC	5535464

rs368679861
HG00102 >hsa-mir-532 TCCCACACCCAAGGCTTGCAGA CTCCCACACCCAAGGCTTGCAG CCTCCCACACCCAAGGCTTGCA CACCCAAGGCTTGCAGAAGAGC 49767832 rs456615
HG00102 >hsa-mir-532 TCCCACACCCAAGGCTTGCAGA CTCCCACACCCAAGGCTTGCAG CCTCCCACACCCAAGGCTTGCA CACCCAAGGCTTGCAGAAGAGC 49767835 rs456617
HG00102 >hsa-mir-532 TCCCACACCCAAGGCTTGCAGA CTCCCACACCCAAGGCTTGCAG CCTCCCACACCCAAGGCTTGCA CACCCAAGGCTTGCAGAAGAGC 49767832 rs456615 49767835 rs456617
HG00102 >hsa-mir-4268TCCTCCTCTCAGGATGTGATGT TCCTCCTCTCAGGATGTGATGT CTCCTCTCAGGATGTGATGTCA CTCCTCCTCTCAGGATGTGATG 220771223 rs4674470
HG00102 >hsa-mir-558 TCCTGAGCTGCTGTACCAAAAT TTCCTGAGCTGCTGTACCAAAA GAGCTGCTGTACCAAAATACCA CCTGAGCTGCTGTACCAAAATA 32757230 rs72089144
HG00102 >hsa-mir-892cTCCTTTCTAAGTGGAGTAGGGC CTTTCTAAGTGGAGTAGGGCTT ACTGTTTCCTTTCTAAGTGGAG TTCCTTTCTAAGTGGAGTAGGG 145074284 .
HG00102 >hsa-mir-1304TCTCACTGTAGCATCGAACCCC CTCACTGTAGCATCGAACCCCT GAACCCCTGGGCTCAAGTGATT CGAACCCCTGGGCTCAAGTGAT 93466866 rs2155248
HG00102 >hsa-mir-1273h TGCTGCAGACTCGACCTCCCAG TGCAGACTCGACCTCCCAGGCT CTGCAGACTCGACCTCCCAGGC AGACTCGACCTCCCAGGCTTAA 24214486 .

HG00102 >hsa-mir-3156-2 TGGCCCCCACTTCCAGATCTTT CCCCCACTTCCAGATCTTTCTC ACTTCCAGATCTTTCTCTCTGT CCCCACTTCCAGATCTTTCTCT 14830215 rs113478966

HG00102 >hsa-mir-661 TGGGTCTCTGGCCTGCGTGTGA GGCCTGCGTGTGACTTTGGGGT TGCGTGTGACTTTGGGGTGGCT .

HG00102 >hsa-mir-3909TGTCCTCTA-GGCCTGCAGTCT TA-GGCCTGCAGTCTCATGGGA GGCCTGCAGTCTCATGGGAGA CCTGCAGTCTCATGGGAGAGTG 35731712 rs34874675
HG00102 >hsa-mir-488 TGTTTGAAAGGCTATTTCTTGG TATTTCTTGGTCAGATGACTCT GAAAGGCTATTTCTTGGTCAGA TTGTTTGAAAGGCTATTTCTTG 176998548 rs181176070
HG00102 >hsa-mir-585 TTGGGCGTATCTGTATGCTAGG TGGGCGTATCTGTATGCTAGGG TATCTGTATGCTAGGGCTGCCG GCGTATCTGTATGCTAGGGCTG 168690612 rs62376934
HG00102 >hsa-mir-1303TTTAGAGACGGGGTCTTGCTCT TAGAGACGGGGTCTTGCTCTGT ACGGGGTCTTGCTCTGTTGCCA GGGTCTTGCTCTGTTGCCAGGC 154065347 rs142414368
HG00102 >hsa-mir-1303TTTAGAGACGGGGTCTTGCTCT TAGAGACGGGGTCTTGCTCTGT TTAGAGACGGGGTCTTGCTCTG ACGGGGTCTTGCTCTGTTGCCA 154065383

rs75538180
HG00102 >hsa-mir-1303TTTAGAGACGGGGTCTTGCTCT TAGAGACGGGGTCTTGCTCTGT TTAGAGACGGGGTCTTGCTCTG ACGGGGTCTTGCTCTGTTGCCA 154065347 rs142414368 154065383 rs75538180
HG00102 >hsa-mir-580 TTTGAGAATGATGAATCATTAG GATGAATCATTAGGTTCCGGTC AGAATGATGAATCATTAGGTTC AATGATGAATCATTAGGTTCCG 36148057 rs115089112
HG00102 >hsa-mir-133bTTTGGTCCCCTTCAACCAGCTA TGGTCCCCTTCAACCAGCTACA CCTTCAACCAGCTACAGCAGGG AGAGGTTTGGTCCCCTTCAACC 52013832 rs374103744
HG00105 >hsa-mir-3144AAGGGGACCAAAGAGATATATA TAAGGGGACCAAAGAGATATAT TTAAGGGGACCAAAGAGATATA TTTAAGGGGACCAAAGAGATAT ATACACTTTAAGGGGACCAAAG 120336327 rs68035463

HG00105 >hsa-mir-1908CCGCGGCGGGGACGGCGATTGG GCGGCGGGGACGGCGATTGGTC CGCGGCGGGGACGGCGATTGGT CGGGGACGGCGATTGGTCCGTA GGGATGCCGCGGCGGGGACGGC 61582708 rs174561

HG00105 >hsa-mir-1273h CCTGGGAGGTCAAGGCTGTAGT TGGGAGGTCAAGGCTGTAGTGT GCCTGGGAGGTCAAGGCTGTAG ATTGCTTGAGCCTGGGAGGTCA TTGAGCCTGGGAGGTCAAGGCT 24214486 .

HG00105 >hsa-mir-1178GAAGGGAAGGGTTCAGGGTCAG GAAGGGTTCAGGGTCAGCTGAG AAGGGAAGGGTTCAGGGTCAGC GTTGGCTGGCAGAGGAAGGGAA GGAAGGGTTCAGGGTCAGCTGA 120151501 rs74614893

HG00105 >hsa-mir-548ak GCAAAAGTAACTGCGGTTTTTG TGCAAAAGTAACTGCGGTTTTT CAAAAGTAACTGCGGTTTTTGA GTGCAAAAGTAACTGCGGTTTT rs7070684

HG00105 >hsa-mir-3196GCGGGGCGGCAGGGGCCTCCCC GGCGGGGCGGCAGGGGCCTCCC GGGCGGGGCGGCAGGGGCCTCC TGGGGGCGGGGCGGCAGGGGCC GGGGCGGGGCGGCAGGGGCCTC 61870167 rs744591

HG00105 >hsa-mir-3144TAAGGGGACCAAAGAGATATAT TTAAGGGGACCAAAGAGATATA AAGGGGACCAAAGAGATATATA CTACACTTTAAGGGGACCAAAG TTTAAGGGGACCAAAGAGATAT 120336384 rs67106263

HG00105 >hsa-mir-3144TAAGGGGACCAAAGAGATATAT AAGGGGACCAAAGAGATATATA TTAAGGGGACCAAAGAGATATA ATACACTTTAAGGGGACCAAAG TTTAAGGGGACCAAAGAGATAT 120336327 rs68035463 120336384 rs67106263
HG00105 >hsa-mir-3141TCACCCGGTGAGGGCGGGTGGA CCGGTGAGGGCGGGTGGAGGAG CGGTGAGGGCGGGTGGAGGAGG CACCCGGTGAGGGCGGGTGGAG CCCGGTGAGGGCGGGTGGAGGA 153975576 rs936581

HG00105 >hsa-mir-4326TCTGCTGTTCCTCTGTCTCCCA TGGTCTGCTGTTCCTCTGTCTC

CTGGTCTGCTGTTCCTCTGTCT   GCTGTTCCTCTGTCTCCCAGAC
TGCTGTTCCTCTGTCTCCCAGA   61918164   rs6062431

HG00105   >hsa-mir-412 TGGGGTACGGGGATGGATGGTC   GGATGGATGGTCGACCAGTTGG
GATGGATGGTCGACCAGTTGGA   TCGACCAGTTGGAAAGTAATTG
ACGGGGATGGATGGTCGACCAG   101531854   rs61992671

HG00105   >hsa-mir-412 TGGGGTACGGGGATGGATGGTC   GGATGGATGGTCGACCAGTTGG
GGGTACGGGGATGGATGGTCGA   GATGGATGGTCGACCAGTTGGA
TCGACCAGTTGGAAAGTAATTG   101531849   .   101531854   rs61992671

HG00105   >hsa-mir-553 TTTTAAAACGGTGAGATTTTGT   TATTTTAAAACGGTGAGATTTT
TTTTATTTTAAAACGGTGAGAT   TTTAAAACGGTGAGATTTTGTT
ATTTTATTTTAAAACGGTGAGA   100746848   .

HG00105   >hsa-mir-553 TTTTAAAACGGTGAGATTTTGT   TTTTATTTTAAAACGGTGAGAT
TATTTTAAAACGGTGAGATTTT   TTTAAAACGGTGAGATTTTGTT
ATTTTATTTTAAAACGGTGAGA   100746848   .   100746855
rs112891767
HG00105   >hsa-mir-553 TTTTATTTTAAAACGGTGAGAT   AATTTTATTTTAAAACGGTGAG
TTTTAAAACGGTGAGATTTTGT   TTATTTTAAAACGGTGAGATTT
TTTAAAACGGTGAGATTTTGTT   100746855   rs112891767

HG00105   >hsa-mir-3141ACCCGGTGAGGGCGGGTGGAGG   CGGTGAGGGCGGGTGGAGGAGG
CCGGTGAGGGCGGGTGGAGGAG   CCCGGTGAGGGCGGGTGGAGGA
CACCCGGTGAGGGCGGGTGGAG   153975576   rs936581

HG00105   >hsa-mir-486-2   ACTGAGCTGCCCCGAGCTGGGC
CTGAGCTGCCCCGAGCTGGGCA   CTGTACTGAGCTGCCCCGAGCT
CCTGTACTGAGCTGCCCCGAGC   GTACTGAGCTGCCCCGAGCTGG   41518007   .

HG00105   >hsa-mir-1269b   AGCCATGCTACGGGCTTCTCTG
ACTGAGCCATGCTACGGGCTTC   AGGTTTCTGGACTGAGCCATGC
TGAGGTTTCTGGACTGAGCCAT   TTTCTGGACTGAGCCATGCTAC   12820632
rs12451747
HG00105   >hsa-mir-3135b   AGCGAGTGCAGTGGTGCAGTCA
AGGCTGGAGCGAGTGCAGTGGT   CTGGAGCGAGTGCAGTGGTGCA
CAGGCTGGAGCGAGTGCAGTGG   CCAGGCTGGAGCGAGTGCAGTG   32717702
rs4285314
HG00105   >hsa-mir-3135b   AGCGAGTGCAGTGGTGCAGTCA
AGGCTGGAGCGAGTGCAGTGGT   CTGGAGCGAGTGCAGTGGTGCA
CAGGCTGGAGCGAGTGCAGTGG   CCAGGCTGGAGCGAGTGCAGTG   32717722
rs4351242
HG00105   >hsa-mir-3135b   AGCGAGTGCAGTGGTGCAGTCA
AGGCTGGAGCGAGTGCAGTGGT   CTGGAGCGAGTGCAGTGGTGCA
CAGGCTGGAGCGAGTGCAGTGG   CCAGGCTGGAGCGAGTGCAGTG   32717702
rs4285314   32717722   rs4351242

HG00105 >hsa-mir-1227AGGCGGTGGTGGGCACTGCTGG TGGTGGGCACTGCTGGGGTGGG
TGGGGCCAGGCGGTGGTGGGCA CGGTGGTGGGCACTGCTGGGGT
GGTGGGCACTGCTGGGGTGGGC 2234093 rs190788838

HG00105 >hsa-mir-612 AGGGCTTCTGAGCTCCTTAGCA GGGCTTCTGAGCTCCTTAGCAC
CAGGGCTTCTGAGCTCCTTAGC GCAGGGCTTCTGAGCTCCTTAG
GGCAGGGCTTCTGAGCTCCTTA 65211979 rs12803915

HG00105 >hsa-mir-548ap AGTAATTGCAGTCTTTGTCATT
AAGTAATTGCAGTCTTTGTCAT AAAGTAATTGCAGTCTTTGTCA
AAAAGTAATTGCAGTCTTTGTC CAAAAGTAATTGCAGTCTTTGT 86368898
rs4414449
HG00105 >hsa-mir-548ap AGTAATTGCAGTCTTTGTCATT
AAGTAATTGCAGTCTTTGTCAT AAAGTAATTGCAGTCTTTGTCA
AAAAGTAATTGCAGTCTTTGTC CAAAAGTAATTGCAGTCTTTGT 86368898
rs4414449 86368959 rs4577031
HG00105 >hsa-mir-548ap AGTAATTGCGGTCTTTGTCATT
AAGTAATTGCGGTCTTTGTCAT AAAGTAATTGCGGTCTTTGTCA
AAAAGTAATTGCGGTCTTTGTC CAAAAGTAATTGCGGTCTTTGT 86368922
rs76468441
HG00105 >hsa-mir-548ap AGTAATTGCGGTCTTTGTCATT
AAGTAATTGCGGTCTTTGTCAT AAAGTAATTGCGGTCTTTGTCA
AAAAGTAATTGCGGTCTTTGTC CAAAAGTAATTGCGGTCTTTGT 86368959
rs4577031
HG00105 >hsa-mir-548ap AGTAATTGCGGTCTTTGTCATT
AAGTAATTGCGGTCTTTGTCAT AAAGTAATTGCGGTCTTTGTCA
AAAAGTAATTGCGGTCTTTGTC CAAAAGTAATTGCGGTCTTTGT 86368922
rs76468441 86368959 rs4577031
HG00105 >hsa-mir-196a-2 AGTTTCATGTTGTTGGGATTGA
AGGTAGTTTCATGTTGTTGGGA TAGTTTCATGTTGTTGGGATTG
GTAGTTTCATGTTGTTGGGATT GGTAGTTTCATGTTGTTGGGAT 54385599
rs11614913
HG00105 >hsa-mir-1908GGGGACGGCGATTGGTCCGTAT GCGGGGACGGCGATTGGTCCGT
GGCGGGGACGGCGATTGGTCCG CGGGGACGGCGATTGGTCCGTA
TGCCGCGGCGGGGACGGCGATT 61582708 rs174561

HG00105 >hsa-mir-548h-3 GTAATCGTGGTTTTTGTCATTG
TGCAAAAGTAATCGTGGTTTTT AGTAATCGTGGTTTTTGTCATT
AAGTAATCGTGGTTTTTGTCAT TGGTGCAAAAGTAATCGTGGTT 13446924
rs9913045
HG00105 >hsa-mir-449bGTATCGTTAGCTGGCTGCTTGG AGTGTATCGTTAGCTGGCTGCT
CAGTGTATCGTTAGCTGGCTGC GTGTATCGTTAGCTGGCTGCTT
TGTATCGTTAGCTGGCTGCTTG 54466544 rs10061133

HG00105 >hsa-mir-573 GTGTAACTGATCAGGATCTACT TGTGTAACTGATCAGGATCTAC
GATGTGTAACTGATCAGGATCT TGATGTGTAACTGATCAGGATC
GTGATGTGTAACTGATCAGGAT 24521904 rs78830737

HG00105 >hsa-mir-3144TAAGGGGACCAAAGAGATATAT TTAAGGGGACCAAAGAGATATA TTTAAGGGGACCAAAGAGATAT TACACTTTAAGGGGACCAAAGA AAATACACTTTAAGGGGACCAA 120336327 rs68035463

HG00105 >hsa-mir-3144TAAGGGGACCAAAGAGATATAT TTAAGGGGACCAAAGAGATATA TTTAAGGGGACCAAAGAGATAT TACACTTTAAGGGGACCAAAGA AACTACACTTTAAGGGGACCAA 120336384 rs67106263

HG00105 >hsa-mir-3144TAAGGGGACCAAAGAGATATAT TTAAGGGGACCAAAGAGATATA TTTAAGGGGACCAAAGAGATAT TACACTTTAAGGGGACCAAAGA AAATACACTTTAAGGGGACCAA 120336327 rs68035463 120336384 rs67106263

HG00105 >hsa-mir-629 TACGTTGGGAGAACTTTTACGG TTACGTTGGGAGAACTTTTACG GTTTACGTTGGGAGAACTTTTA TTTACGTTGGGAGAACTTTTAC GGTTTACGTTGGGAGAACTTTT 70371794 .

HG00105 >hsa-mir-629 TACGTTGGGAGAACTTTTATGG TTACGTTGGGAGAACTTTTATG TTTACGTTGGGAGAACTTTTAT TGGGTTTACGTTGGGAGAACTT GTTTACGTTGGGAGAACTTTTA 70371761 rs377691713

HG00105 >hsa-mir-629 TACGTTGGGAGAACTTTTATGG TTACGTTGGGAGAACTTTTATG TTTACGTTGGGAGAACTTTTAT TGGGTTTACGTTGGGAGAACTT GTTTACGTTGGGAGAACTTTTA 70371761 rs377691713 70371794 .

HG00105 >hsa-mir-888 TACTCAAAAAGCTTTCAGTCAC TCTACTCAAAAAGCTTTCAGTC TGCTCTACTCAAAAAGCTTTCA GCTCTACTCAAAAAGCTTTCAG GGCAGTGCTCTACTCAAAAAGC 145076355 rs143634721

HG00105 >hsa-mir-449cTAGGCAGTGTATTGCTAGCGGC TCAGATAGGCAGTGTATTGCTA TGTCAGATAGGCAGTGTATTGC TGTGTCAGATAGGCAGTGTATT TGGGATGTGTCAGATAGGCAGT 54468166 rs75661995

HG00105 >hsa-mir-564 TCAGCAGGCAACATGGCCGAGA TGTCAGCAGGCAACATGGCCGA GTCAGCAGGCAACATGGCCGAG GTGTCAGCAGGCAACATGGCCG TGCCAGGCACGGTGTCAGCAGG 44903385 rs114636202

HG00105 >hsa-mir-222 TCAGTAGCCAGTGTAGATCCTG TGGCTCAGTAGCCAGTGTAGAT TTGGCTCAGTAGCCAGTGTAGA TCATTGGCTCAGTAGCCAGTGT TACCCTCATTGGCTCAGTAGCC 45606504 rs191727254

HG00105 >hsa-mir-515-1 TCCAAAAGAAAGCACTTTCTGT TCTCCAAAAGAAAGCACTTTCT TTCTCCAAAAGAAAGCACTTTC TCATTCTCCAAAAGAAAGCACT TGCAGTCATTCTCCAAAAGAAA 54182326 rs374576826

HG00105 >hsa-mir-663aTCCCAGGCGGGGCGCCGCGGGA TCCGGCGTCCCAGGCGGGGCGC TTCCGGCGTCCCAGGCGGGGCG GCGCCGCGGGACCTCCCTCGTG

GGCGCCGCGGGACCTCCCTCGT 26188880 .

HG00105 >hsa-mir-149 TCCGTGTCTTCACTCCCGTGCT TGGCTCCGTGTCTTCACTCCCG
TCTGGCTCCGTGTCTTCACTCC CCGTGTCTTCACTCCCGTGCTT
AGCTCTGGCTCCGTGTCTTCAC 241395503 rs2292832

HG00105 >hsa-mir-618 TCCTTCTGAGTGTAATTACGTA TGTCCTTCTGAGTGTAATTACG
TTGTCCTTCTGAGTGTAATTAC TACTTGTCCTTCTGAGTGTAAT
GTCCTTCTGAGTGTAATTACGT 81329536 rs2682818

HG00105 >hsa-mir-492 TCGAGGACCTGCGGGACAAGAT TACAGGACCATCGAGGACCTGC
TACTACAGGACCATCGAGGACC TCCAGCCACTACTACAGGACCA
GACCTGCGGGACAAGATTCTTG 95228179 rs200816308

HG00105 >hsa-mir-3183TCGGAGTCGCTCGGAGCAGCCA TCTCGGAGTCGCTCGGAGCAGC
TCTCTCGGAGTCGCTCGGAGCA TCTGCCCTGCCTCTCTCGGAGT
TGCCCTGCCTCTCTCGGAGTCG 925764 rs2663345

HG00105 >hsa-mir-4305TCTGGGTTCTTAGAGGCCTAAT TTCTGGGTTCTTAGAGGCCTAA
GTTCTGGGTTCTTAGAGGCCTA TCCAGTTCTGGGTTCTTAGAGG
CAGTTCTGGGTTCTTAGAGGCC 40238175 rs67976778

HG00105 >hsa-mir-1255a TGAGCAAAGAAAGTAGATTTTT
GCAAAGAAAGTAGATTTTTTAG TCAAGGATGAGCAAAGAAAGTA
GAGCAAAGAAAGTAGATTTTTT TCTCAAGGATGAGCAAAGAAAG 102251501
rs28664200
HG00105 >hsa-mir-1200TGAGCCATTCTGAGCCTCAATC TCCTGAGCCATTCTGAGCCTCA
TCTCCTGAGCCATTCTGAGCCT TTCTCCTGAGCCATTCTGAGCC
TGCTACTTCTCCTGAGCCATTC 36958995 rs180826747

HG00105 >hsa-mir-27a TGAGGAGCAGGGCTTAGCTGCT TTAGCTGCTTGTGAGCAGGGTC
GAGGAGCAGGGCTTAGCTGCTT GGAGCAGGGCTTAGCTGCTTGT
GAGCAGGGCTTAGCTGCTTGTG 13947292 rs895819

HG00105 >hsa-mir-3151TGATGGGTGGGGCAATGGGATC TGGGTGGGGCAATGGGATCAGG
TGGGGCAATGGGATCAGGTGCC GGGGTGATGGGTGGGGCAATGG
GGGTGATGGGTGGGGCAATGGG 104166902 rs35605502

HG00105 >hsa-mir-3156-2 TGCAGAAGAAAGATCTGGAAGT
GAAAGATCTGGAAGTGGGAGAC GAAGAAAGATCTGGAAGTGGGA
GCAGAAGAAAGATCTGGAAGTG CAGAAGAAAGATCTGGAAGTGG 14830215
rs113478966
HG00105 >hsa-mir-3156-3 TGCAGAAGAAAGATCTGGAAGT
GCAGAAGAAAGATCTGGAAGTG GAAGAAAGATCTGGAAGTGGGA
GAAAGATCTGGAAGTGGGAGAC AGAAGAAAGATCTGGAAGTGGG 14778721
rs2747232
HG00105 >hsa-mir-3152TGCCTCTGTTCTAACACAAGAC TTGCCTCTGTTCTAACACAAGA

TATTGCCTCTGTTCTAACACAA TTATTGCCTCTGTTCTAACACA
TGCAGAGTTATTGCCTCTGTTC 18573360 rs13299349

HG00105 >hsa-mir-1254-2 TGGAAGCTGGAGCCTGCAGTGA
TGAGCCTGGAAGCTGGAGCCTG GAAGCTGGAGCCTGCAGTGAGC
GGAAGCTGGAGCCTGCAGTGAG GCCTGGAAGCTGGAGCCTGCAG 23682383
rs200793185
HG00105 >hsa-mir-378d-2 TGGACTTGGAGTCAGAAAACTT
GACTTGGAGTCAGAAAACTTTC GGACTTGGAGTCAGAAAACTTT
GAACACTGGACTTGGAGTCAGA TACAAGGAGAGAACACTGGACT 94928250
rs73692959
HG00105 >hsa-mir-1273h TGGGAGGTCAAGGCTGTAGTGT
TGAGCCTGGGAGGTCAAGGCTG TTGAGCCTGGGAGGTCAAGGCT
TGCTTGAGCCTGGGAGGTCAAG TTGCTTGAGCCTGGGAGGTCAA 24214486 .

HG00105 >hsa-mir-412 TGGGGTACGGGGATGGATGGTC TCGACCAGTTGGAAAGTAATTG
TGGTCGACCAGTTGGAAAGTAA TACGGGGATGGATGGTCGACCA
TGGATGGTCGACCAGTTGGAAA 101531854 rs61992671

HG00105 >hsa-mir-412 TGGGGTACGGGGATGGATGGTC TCGACCAGTTGGAAAGTAATTG
TGGTCGACCAGTTGGAAAGTAA TACGGGGATGGATGGTCGACCA
TGGATGGTCGACCAGTTGGAAA 101531849 . 101531854 rs61992671

HG00105 >hsa-mir-323bTGTCCGTGGTGAGTTCGCATTA TTGTCCGTGGTGAGTTCGCATT
TACTCGGAGGGAGGTTGTCCGT TCGGAGGGAGGTTGTCCGTGGT
AGGTTGTCCGTGGTGAGTTCGC 101522556 rs56103835

HG00105 >hsa-mir-199bTTAGACTATCTGTTCAGGACTC AGTGTTTAGACTATCTGTTCAG
TTTAGACTATCTGTTCAGGACT TGTTTAGACTATCTGTTCAGGA
ACCCAGTGTTTAGACTATCTGT 131007001 rs146892675

HG00105 >hsa-mir-642bTTCCCTCTCCAAATGTGTCTTG TTGGGAGGTTCCCTCTCCAAAT
TGGGAGGTTCCCTCTCCAAATG GAGTTGGGAGGTTCCCTCTCCA
GTTGGGAGGTTCCCTCTCCAAA 46178217 rs111664333

HG00105 >hsa-mir-4277TTCTGAGCACAGTACACTGGGC TCGAGGCAGTTCTGAGCACAGT
TGGGTCGAGGCAGTTCTGAGCA GTTCTGAGCACAGTACACTGGG
GCAGTTCTGAGCACAGTACACT 1708902 rs115200817

HG00105 >hsa-mir-1178TTGGCTGGCAGAGGAAGGGAAG TGGCTGGCAGAGGAAGGGAAGG
TGGCAGAGGAAGGGAAGGGTTC GCGTTGGCTGGCAGAGGAAGGG
GTTGGCTGGCAGAGGAAGGGAA 120151501 rs74614893

HG00105 >hsa-mir-553 TTTAAAACGGTGAGATTTTGTT TTTTAAAACGGTGAGATTTTGT
ATTTTAAAACGGTGAGATTTTG TATTTTAAAACGGTGAGATTTT
TTATTTTAAAACGGTGAGATTT 100746848 .

HG00105 >hsa-mir-553 TTTAAAACGGTGAGATTTTGTT TTTTAAAACGGTGAGATTTTGT
ATTTTAAAACGGTGAGATTTTG TATTTTAAAACGGTGAGATTTT
TTATTTTAAAACGGTGAGATTT 100746855 rs112891767

HG00105 >hsa-mir-553 TTTAAAACGGTGAGATTTTGTT TTTTAAAACGGTGAGATTTTGT
ATTTTAAAACGGTGAGATTTTG TATTTTAAAACGGTGAGATTTT
TTATTTTAAAACGGTGAGATTT 100746848 . 100746855
rs112891767
HG00105 >hsa-mir-646 AGCAGCTGCCTCGGAGGCCTCA CGGAGGCCTCAGGCTCAGTGGC
TCGGAGGCCTCAGGCTCAGTGG GAGGAAGCAGCTGCCTCGGAGG
AGGAAGCAGCTGCCTCGGAGGC 58883605 rs6513497

HG00105 >hsa-mir-646 AGCAGCTGCCTCGGAGGCCTCA CGGAGGCCTCAGGCTCAGTGGC
CTCGGAGGCCTCAGGCTCAGTG TCGGAGGCCTCAGGCTCAGTGG
GCCTCGGAGGCCTCAGGCTCAG 58883534 rs6513496 58883605
rs6513497
HG00105 >hsa-mir-646 AGCAGCTGCCTCTGAGGCCTCA CTGAGGCCTCAGGCTCAGTGGC
CTCTGAGGCCTCAGGCTCAGTG TCTGAGGCCTCAGGCTCAGTGG
GCCTCTGAGGCCTCAGGCTCAG 58883534 rs6513496

HG00105 >hsa-mir-149 AGGGAGGGAGGGACGGGGGCTG GGGCTGTGCTGGGGCAGCCGGA
GGGACGGGGGCTGTGCTGGGGC GACGGGGGCTGTGCTGGGGCAG
GGGGCTGTGCTGGGGCAGCCGG 241395503 rs2292832

HG00105 >hsa-mir-658 AGGTCGGTTGGTCGGTCGGGAA GTCGGTTGGTCGGTCGGGAACG
TAGGTCGGTTGGTCGGTCGGGA . .

HG00105 >hsa-mir-412 CGTCCGTATCCGCTGCAG CCGTCCGTATCCGCTGCAG
TCACCTGGTCCACTGGCCGTCC ACCTGGTCCACTGGCCGTCCGT
CACCTGGTCCACTGGCCGTCCG 101531854 rs61992671

HG00105 >hsa-mir-412 CGTCCGTATCCGCTGCAG CCGTCCGTATCCGCTGCAG
TCACCTGGTTCACTGGCCGTCC ACCTGGTTCACTGGCCGTCCGT
CACCTGGTTCACTGGCCGTCCG 101531849 . 101531854 rs61992671

HG00105 >hsa-mir-3180-4 CTCCGGATGCCAGTCCCTCATC
GGAGGGTGAAGCCTCCGGATGC CTGGCCTGGTCGCGCTGTGGCT
AGCGGAGGGTGAAGCCTCCGGA GAGCGGAGGGTGAAGCCTCCGG 15248720
rs75000738
HG00105 >hsa-mir-4268CTCTCAGGATGTGATGTCACCT CCTCTCAGGATGTGATGTCACC
GCTCCTCCTCTCAGGATGTGAT CTCCTCCTCTCAGGATGTGATG
TCCTCTCAGGATGTGATGTCAC 220771223 rs4674470

HG00105 >hsa-mir-3151CTGATCCCACACCCCACCTGTC TGATCCCACACCCCACCTGTCA
GATCCCACACCCCACCTGTCAC GGGCATCCCACCTGATCCCACA
TCCCACCTGATCCCACACCCCA 104166902 rs35605502

HG00105 >hsa-mir-412 CTTCACCTGGTTCACTAGCCGT ACCTGGTTCACTAGCCGTCCGT
TGTACTTCACCTGGTTCACTAG CTGGTTCACTAGCCGTCCGTAT
GTACTTCACCTGGTTCACTAGC 101531849 .

HG00105 >hsa-mir-658 TAGGTCGGTTGGTCGGTCGGGA .

HG00105 >hsa-mir-658 GTCCGTTGGTCGGTCGGGAACG G
TCCGTTGGTCGGTCGGGAACGA .

HG00105 >hsa-mir-320eGAAAAGCTGGGTTGAGAAGGT AAAAGCTGGGTTGAGAAGGT
GGAAAAGCTGGGTTGAGAAGGT GGGAAAAGCTGGGTTGAGAAGG rs10423365

HG00105 >hsa-mir-1343GCCCCTCCTGGGGCCCGCACTC CCCCTCCTGGGGCCCGCACTCT
GGGGCCCGCACTCTCGCTCTGG CCCTCCTGGGGCCCGCACTCTC
TGGGGCCCGCACTCTCGCTCTG 34963416 rs2986407

HG00105 >hsa-mir-3180-4 GGAGGGTGAAGCCTCCGGATGC
GGTGAAGCCTCCGGATGCCAGT AGCGGAGGGTGAAGCCTCCGGA
GCGGAGGGTGAAGCCTCCGGAT GCCTGGTCGCGCTGTGGCGAAG 15248798
rs183853838
HG00105 >hsa-mir-3180-4 GGAGGGTGAAGCCTCCGGATGC
GGTGAAGCCTCCGGATGCCAGT AGCGGAGGGTGAAGCCTCCGGA
GCGGAGGGTGAAGCCTCCGGAT CTGGCCTGGTCGCGCTGTGGCT 15248720
rs75000738 15248798 rs183853838
HG00105 >hsa-mir-1227GGCATTTGACCCCGTGCCACCC AGGCATTTGACCCCGTGCCACC
AGGCATTTGACCCCGTGCCACC TGACCCCGTGCCACCCTTTTCC
ATTTGACCCCGTGCCACCCTTT 2234093 rs190788838

HG00105 >hsa-mir-580 TATTTGAGAATGATGAATCATT TGAATCATTAGGTTCCGGTCAG
ATGAATCATTAGGTTCCGGTCA TTTGAGAATGATGAATCATTAG
GAGAATGATGAATCATTAGGTT 36148057 rs115089112

HG00105 >hsa-mir-3118-1 TGAAAATTCTTCTAGTGTG ATGAAAATTCTTCTAGTGTG
TGCATTATGAAAATTCTTCTAG TTATGAAAATTCTTCTAGTGTG
ATTATGAAAATTCTTCTAGTGT 142667330 rs76132421

HG00105 >hsa-mir-637 TGGCTAAGGTGTTGGCTCGGGC TGGCTAAGGTGTTGGCTCGGGC
.
HG00105 >hsa-mir-320c-1 AAAAGCTGGGTTGAGAGGGTAG
AGCTGGGTTGAGAGGGTAGGAA CTGGGTTGAGAGGGTAGGAAAA
AGGGTAGGAAAAAAATGATGTA 101491923 rs141611518

HG00105 >hsa-mir-651 AAAAGGAAAGTGTATCCTAAAA GGAAAGTGTATCCTAAAAGGCA
TGTATCCTAAAAGGCAATGACA AAAGGAAAGTGTATCCTAAAAG 2234093
rs190788838
HG00105 >hsa-mir-202 AAAGAGGTATAGGGCATGGGAA AAGAGGTATAGGGCATGGGAAA
GGGAAAACGGGGCGGTCGGGTC TAAAGAGGTATAGGGCATGGGA 167967958

rs79639536
HG00105 >hsa-mir-548ap AACAAAAACCACAATTACTTTT
CAAAAACCACAATTACTTTTTA CAATTACTTTTTACTGACCTAA rs4414449
24214486 .
HG00105 >hsa-mir-548ap AACAAAAACCACAATTACTTTT
CAAAAACCACAATTACTTTTTA TTACTTTTTACTGACCTAAAGA rs4577031
154065347 rs142414368
HG00105 >hsa-mir-548ap AACAAAAACCACAATTACTTTT
CAAAAACCACAATTACTTTTTA CAATTACTTTTTACTGACCTAA rs4414449
154065383 rs75538180
HG00105 >hsa-mir-1255b-2 AACCACTTTCTTTGCTCATCCG
CTTTCTTTGCTCATCCGTAAGG AAACCACTTTCTTTGCTCATCC
CTTTCTTTGCTCATCCGTAAGG 154065347 rs142414368 154065383
rs75538180
HG00105 >hsa-mir-423 AAGCTCGGTCTGAGGCCCCTCA AGGCCCCTCAGTCTTGCTTCCT
TCTGAGGCCCCTCAGTCTTGCT GTCTGAGGCCCCTCAGTCTTGC 93466866
rs2155248
HG00105 >hsa-mir-548ap AATAAAAACCACAATTACTTTT
TAAAAACCACAATTACTTTTTA ATAAAAACCACAATTACTTTTT rs76468441
52013832 rs374103744
HG00105 >hsa-mir-548ap AATAAAAACCACAATTACTTTT
TAAAAACCACAATTACTTTTTA CAATTACTTTTTACTGACCTAA rs4414449
34963416 rs2986407
HG00105 >hsa-mir-548ap AATAAAAACCACAATTACTTTT
TAAAAACCACAATTACTTTTTA ATAAAAACCACAATTACTTTTT rs76468441
241395503 rs2292832
HG00105 >hsa-mir-548ap AATAAAAACCACAATTACTTTT
TAAAAACCACAATTACTTTTTA CAATTACTTTTTACTGACCTAA rs4414449

HG00105 >hsa-mir-449cACAGTTGCTAGTTGCACTCCTC AACAGTTGCTAGTTGCACTCCT
GTTGCTAGTTGCACTCCTCTCT GTTGCACTCCTCTCTGTTGCAT 61582708
rs174561
HG00105 >hsa-mir-3118-1 ACTGCATTATGAAAATTCTTCT
ATTATGAAAATTCTTCTAGTGT GCATTATGAAAATTCTTCTAGT
CTGCATTATGAAAATTCTTCTA 54385599 rs11614913

HG00105 >hsa-mir-642bAGATACATTTGGAGAGGGACCC TTGGAGAGGGACCCTCCCAACT
TTTGGAGAGGGACCCTCCCAAC ATACATTTGGAGAGGGACCCTC 131007001
rs146892675
HG00105 >hsa-mir-3180-4 AGCGGAGGGTGAAGCCTCCGGA
GGAGGGTGAAGCCTCCGGATGC GAGCGGAGGGTGAAGCCTCCGG
GCGGAGGGTGAAGCCTCCGGAT 135061112 rs12355840

HG00105 >hsa-mir-3180-4 AGCGGAGGGTGAAGCCTCCGGA
GGAGGGTGAAGCCTCCGGATGC GAGCGGAGGGTGAAGCCTCCGG
GCGGAGGGTGAAGCCTCCGGAT 41522213 rs7207008

HG00105 >hsa-mir-30d AGCTTTCAGTCAGATGTTTGCT GGCTAAGCTTTCAGTCAGATGT GCTAAGCTTTCAGTCAGATGTT TTCAGTCAGATGTTTGCTGCTA

HG00105 >hsa-mir-519a-2 AGGAAAGTGCATCCTTTTAGAG AGTGCATCCTTTTAGAGGGTTA GGAAAGTGCATCCTTTTAGAGG GAAAGGAAAGTGCATCCTTTTA 45606504 rs191727254

HG00105 >hsa-mir-646 AGGAAGCAGCTGCCTCGGAGGC CCAGGAGAGGAAGCAGCTGCCT GCTGCCTCGGAGGCCTCAGGCT GAGAGGAAGCAGCTGCCTCGGA 13947292 rs895819

HG00105 >hsa-mir-646 AGGAAGCAGCTGCCTCGGAGGC TGCCTCGGAGGCCTCAGGCTCA CGGAGGCCTCAGGCTCAGTGGC TCGGAGGCCTCAGGCTCAGTGG

HG00105 >hsa-mir-646 AGGAAGCAGCTGCCTCTGAGGC GCTGCCTCTGAGGCCTCAGGCT CTGAGGCCTCAGGCTCAGTGGC TCTGAGGCCTCAGGCTCAGTGG 135817150 .

HG00105 >hsa-mir-630 AGTATTCTGTACCAGGGAAGGT ACCTAGTATTCTGTACCAGGGA CCAGGGAAGGTAGTTCTTAACT GGAAGGTAGTTCTTAACTATGT 142667330 rs76132421

HG00105 >hsa-mir-513cATAAATTTCACCTTTCTGAGAA TTCTGAGAAGAGTAATGTACAG CCTTTCTGAGAAGAGTAATGTA TTTCACCTTTCTGAGAAGAGTA 207647981 rs2241347

HG00105 >hsa-mir-323bATACACGGTCGACCTCTTTTCG TACACGGTCGACCTCTTTTCGG ACACGGTCGACCTCTTTTCGGT rs56103835 207647981 rs2241347

HG00105 >hsa-mir-3144ATACCTGTTCAGTCTCTTTAAA TTCAGTCTCTTTAAAGTGTAGT CCTGTTCAGTCTCTTTAAAGTG TGTTCAGTCTCTTTAAAGTGTA 120336327 rs68035463

HG00105 >hsa-mir-3144ATACCTGTTCAGTCTCTTTAAA TTCAGTCTCTTTAAAGTGTAGT CTGTTCAGTCTCTTTAAAGTGT TATACCTGTTCAGTCTCTTTAA 120336384 rs67106263

HG00105 >hsa-mir-3144ATACCTGTTCGGTCTCTTTAAA CTGTTCGGTCTCTTTAAAGTGT GTTCGGTCTCTTTAAAGTGTAG TGTTCGGTCTCTTTAAAGTGTA 120336327 rs68035463 120336384 rs67106263

HG00105 >hsa-mir-642aATTTGGAGAGGGAACCTCCCAA AGACACATTTGGAGAGGGAACC ACACATTTGGAGAGGGAACCTC CACATTTGGAGAGGGAACCTCC 104166902 rs35605502

HG00105 >hsa-mir-548ac CAAAAACCGGCAATTACTTTTG GGCAAAAACCGGCAATTACTTT TTACTTTTGCACTAACCTAATA CAATTACTTTTGCACTAACCTA 18573360 rs13299349

HG00105 >hsa-mir-4293CACACCAGCCTGACAGGAACAG CACCAGCCTGACAGGAACAGCC TCACACCAGCCTGACAGGAACA CAGCCTGACAGGAACAGCCTGT 14830215 rs113478966

HG00105 >hsa-mir-412 CACCTGGTCCACTGGCCGTCCG ACCTGGTCCACTGGCCGTCCGT CTGGCCGTCCGTATCCGCTGCA CCTGGTCCACTGGCCGTCCGTA 15248720 rs75000738

HG00105 >hsa-mir-412 CACCTGGTTCACTGGCCGTCCG ACCTGGTTCACTGGCCGTCCGT
CTGGCCGTCCGTATCCGCTGCA CCTGGTTCACTGGCCGTCCGTA 15248798
rs183853838
HG00105 >hsa-mir-515-1 CAGAGTGCCTTCTTTTGGAGCA
GAGTGCCTTCTTTTGGAGCATT TGCCTTCTTTTGGAGCATTACT
GTGCCTTCTTTTGGAGCATTAC 15248720 rs75000738 15248798
rs183853838
HG00105 >hsa-mir-449bCAGCAGCCACAACTACCCTGCC CACAACTACCCTGCCACTTGCT
GCAGCCACAACTACCCTGCCAC AGCAGCCACAACTACCCTGCCA 19263542
.
HG00105 >hsa-mir-1227CATTTGACCCCGTGCCACCCTT ATTTGACCCCGTGCCACCCTTT
AGGCATTTGACCCCGTGCCACC GACCCCGTGCCACCCTTTTCCC

HG00105 >hsa-mir-1908CCACCGGCCGCCGGCTCCGCCC CCGCCGGCTCCGCCCCGGCCCC
GGCCGCCGGCTCCGCCCCGGCC CGGCCGCCGGCTCCGCCCCGGC

HG00105 >hsa-mir-3151CCACCTGATCCCACACCCCACC CACCTGATCCCACACCCCACCT
CCCACCTGATCCCACACCCCAC TGATCCCACACCCCACCTGTCA 72744798
rs745666
HG00105 >hsa-mir-1343CCCCTCCTGGGGCCCGCACTCT CCCTCCTGGGGCCCGCACTCTC
CCTGGGGCCCGCACTCTCGCTC TGGGGCCCGCACTCTCGCTCTG 35731697
rs9607265
HG00105 >hsa-mir-943 CCTGACTGTTGCCGTCCTCCAG CTGTTGCCGTCCTCCAGCCCCA
CTCCAGCCCCACTCAAAGGCAT GCCGTCCTCCAGCCCCACTCAA 35731712
rs34874675
HG00105 >hsa-mir-3152CCTGTGTTAGAATAAGGGCAAT TTAGAATAAGGGCAATAACTCT
AGAATAAGGGCAATAACTCTGC TGTGTTAGAATAAGGGCAATAA 35731697
rs9607265 35731712 rs34874675
HG00105 >hsa-mir-196a-2 CTCGGCAACAAGAAACTGTCTG
ACTCGGCAACAAGAAACTGTCT CAACAAGAAACTGTCTGAGTTA
ACAAGAAACTGTCTGAGTTACA

HG00105 >hsa-mir-486-2 CTCGGCGCAGCTCAGTACAGGA
AGGGCCTCGGCGCAGCTCAGTA TCGGCGCAGCTCAGTACAGGAT
GGGCCTCGGCGCAGCTCAGTAC 131701279 rs367805

HG00105 >hsa-mir-3615CTCTCTCGGCTCCTCGCGGCTC GGCTCCTCGCGGCTCGCGGCGG
CGGCTCCTCGCGGCTCGCGGCG TCGGCTCCTCGCGGCTCGCGGC 101531849
.
HG00105 >hsa-mir-888 CTCTTTGGGTGAAGGAAGGCTC CTGACACCTCTTTGGGTGAAGG
GACTGACACCTCTTTGGGTGAA CCTCTTTGGGTGAAGGAAGGCT 101531854
rs61992671
HG00105 >hsa-mir-2117CTGTTCTCTTTGCCAAGGACAG GCTGTTCTCTTTGCCAAGGACA
TCTCTTTGCCAAGGACAGATCT TGTTCTCTTTGCCAAGGACAGA 101531849
. 101531854 rs61992671
HG00105 >hsa-mir-4274GACCCAGCAGTCCCTCCCCCTG CCCAGCAGTCCCTCCCCCTGCA
TGACCCAGCAGTCCCTCCCCCT TCAGGTGACCCAGCAGTCCCTC 28444183

rs6505162
HG00105 >hsa-mir-629 GAGGTTCTCCCAACGTAAGCCC AGGTTCTCCCAACGTAAGCCCA TCTCCCAACGTAAGCCCAGCCC CAGGAGGTTCTCCCAACGTAAG 220771223 rs4674470
HG00105 >hsa-mir-629 GAGGTTCTCCCAACGTAAGCCC AGGAGGTTCTCCCAACGTAAGC GGAGGTTCTCCCAACGTAAGCC TCTCCCAACGTAAGCCCAGCCC 7461769 rs12512664
HG00105 >hsa-mir-629 GAGGTTCTCCCAACGTAAGCCC AGGTTCTCCCAACGTAAGCCCA TCTCCCAACGTAAGCCCAGCCC CCCAACGTAAGCCCAGCCCCTC 14425204 rs12780876
HG00105 >hsa-mir-3130-1 GCACCAGAGACTGGGTAAGACA GAGACTGGGTAAGACATGACAA CCAGAGACTGGGTAAGACATGA TGCACCAGAGACTGGGTAAGAC 54466544 rs10061133

HG00105 >hsa-mir-3130-2 GCACCGGAGACTGGGTAAGACA CTGCACCGGAGACTGGGTAAGA CCGGAGACTGGGTAAGACATGA TGCACCGGAGACTGGGTAAGAC 54468166 rs75661995

HG00105 >hsa-mir-222 GCAGCTACATCTGGCTACTGGG TACTGGGTCTCTGATGGCATCT GCTACTGGGTCTCTGATGGCAT CTGGCTACTGGGTCTCTGATGG 41518007 .
HG00105 >hsa-mir-3180-4 GCGGAGGGTGAAGCCTCCGGAT CGCTGGCCTGGTCGCGCTGTGG TCGCTGGCCTGGTCGCGCTGTG AAGCCTCCGGATGCCAGTCCCT 33578202 rs140486571

HG00105 >hsa-mir-675 GCTGTATGCCCTCACCACTCAG GTATGCCCTCACCACTCAGCCC GCCCTCACCACTCAGCCCCTGG TGTATGCCCTCACCACTCAGCC 146271303 rs145416750
HG00105 >hsa-mir-149 GGGAGGGAGGGACGGGGGCTGT GGAGGGACGGGGGCTGTGCTGG AGGGACGGGGGCTGTGCTGGGG GACGGGGGCTGTGCTGGGGCAG 54182326 rs374576826
HG00105 >hsa-mir-199bGTACAGTAGTCTGCACATTGGT ACAGTAGTCTGCACATTGGTTA ATTGGTTAGGCTGGGCTGGGTT TACAGTAGTCTGCACATTGGTT 54265670 .
HG00105 >hsa-mir-1197GTAGGACACATGGTCTACTTCT ACACATGGTCTACTTCTTCTCA ACATGGTCTACTTCTTCTCAAT TAGGACACATGGTCTACTTCTT 117102649 rs1414273
HG00105 >hsa-mir-27a GTGTTCACAGTGGCTAAGTTCC AGTGGCTAAGTTCCGCCCCCCA TCGTGTTCACAGTGGCTAAGTT CACAGTGGCTAAGTTCCGCCCC

HG00105 >hsa-mir-3936TAAGGGGTGTATGGCAGATGCA CACCCGACAGATGCACTTGGCA GATGCACCCGACAGATGCACTT TGTATGGCAGATGCACCCGACA

HG00105 >hsa-mir-300 TATACAAGGGCAGACTCTCTCT TGATTATACAAGGGCAGACTCT ATTATACAAGGGCAGACTCTCT rs12894467

HG00105 >hsa-mir-499aTCACAGCAAGTCTGTGCTGCTT CGTCCCTACGCTGCCTGGGCAG

GCTGCTTCCCGTCCCTACGCTG TCCCTACGCTGCCTGGCAGGG 86368922 rs76468441
HG00105 >hsa-mir-412 TCACCTGGTTCACTAGCCGTCC TCACCTGGTTCACTAGCCGTCC ATGTACTTCACCTGGTTCACTA CTTCACCTGGTTCACTAGCCGT 86368959 rs4577031
HG00105 >hsa-mir-4268TCCTCCTCTCAGGATGTGATGT TCCTCCTCTCAGGATGTGATGT CTCCTCTCAGGATGTGATGTCA CTCCTCCTCTCAGGATGTGATG 86368959 rs4577031
HG00105 >hsa-mir-1304TCTCACTGTAGCATCGAACCCC GAACCCCTGGGCTCAAGTGATT CTCACTGTAGCATCGAACCCCT CGAACCCCTGGGCTCAAGTGAT 86368922 rs76468441 86368959 rs4577031
HG00105 >hsa-mir-1273h TGCTGCAGACTCGACCTCCCAG TGCAGACTCGACCTCCCAGGCT CTGCAGACTCGACCTCCCAGGC AGACTCGACCTCCCAGGCTTAA

HG00105 >hsa-mir-3156-2 TGGCCCCCACTTCCAGATCTTT CCCCCACTTCCAGATCTTTCTC ACTTCCAGATCTTTCTCTCTGT CCCCACTTCCAGATCTTTCTCT 36148057 rs115089112

HG00105 >hsa-mir-3909TGTCCTCTA-GGCCTGCAGTCT TA-GGCCTGCAGTCTCATGGGA #NAME? CCTGCAGTCTCATGGGAGAGTG 70371761 rs377691713

HG00105 >hsa-mir-3909TGTCCTCTA-GGCCTGCAGTCT TA-GGCCTGCAGTCTCATGGGA #NAME? CCTGCAGTCTCATGGGAGAGTG 70371794 .

HG00105 >hsa-mir-3909TGTCCTCTAGGGCCTGCAGTCT TCCTCTAGGGCCTGCAGTCTCA TGGGAGAGTGACATGCACCAGG TAGGGCCTGCAGTCTCATGGGA 70371761 rs377691713 70371794 .
HG00105 >hsa-mir-1178TGTTCTTCCCTAGAATGTCAGG TTCTTCCCTAGAATGTCAGGTG GTTCTTCCCTAGAATGTCAGGT TCTTCCCTAGAATGTCAGGTGA 72879653 rs113971639
HG00105 >hsa-mir-1303TTTAGAGACGGGGTCTTGCTCT TAGAGACGGGGTCTTGCTCTGT GGGTCTTGCTCTGTTGCCAGGC ACGGGGTCTTGCTCTGTTGCCA

HG00105 >hsa-mir-1303TTTAGAGACGGGGTCTTGCTCT TAGAGACGGGGTCTTGCTCTGT TTAGAGACGGGGTCTTGCTCTG ACGGGGTCTTGCTCTGTTGCCA 46178217 rs111664333
HG00105 >hsa-mir-1303TTTAGAGACGGGGTCTTGCTCT TAGAGACGGGGTCTTGCTCTGT TTAGAGACGGGGTCTTGCTCTG GGGTCTTGCTCTGTTGCCAGGC 46178217 rs111664333
HG00105 >hsa-mir-580 TTTGAGAATGATGAATCATTAG GATGAATCATTAGGTTCCGGTC AATGATGAATCATTAGGTTCCG AGAATGATGAATCATTAGGTTC 58883534 rs6513496
HG00105 >hsa-mir-133bTTTGGTCCCCTTCAACCAGCTA TGGTCCCCTTCAACCAGCTACA CCTTCAACCAGCTACAGCAGGG AGAGGTTTGGTCCCCTTCAACC 58883605 rs6513497
HG00106 >hsa-mir-3144AAGGGGACCAAAGAGATATATA TAAGGGGACCAAAGAGATATAT

TTAAGGGGACCAAAGAGATATA TTTAAGGGGACCAAAGAGATAT
ATACACTTTAAGGGGACCAAAG 120336327 rs68035463

HG00106 >hsa-mir-3125AGAATGGATAGAGGAAGCTGTG GAGGAAGCTGTGGAGAGAACTC
AGAGGAAGCTGTGGAGAGAACT GCTGTGGAGAGAACTCACGGTG
GGAAGCTGTGGAGAGAACTCAC 12877501 rs78852835

HG00106 >hsa-mir-1273h CCTGGGAGGTCAAGGCTGTAGT
TGGGAGGTCAAGGCTGTAGTGT GCCTGGGAGGTCAAGGCTGTAG
ATTGCTTGAGCCTGGGAGGTCA TTGAGCCTGGGAGGTCAAGGCT 24214486 .

HG00106 >hsa-mir-1178GAAGGGAAGGGTCCAGGGTCGG TGGCAGAGGAAGGGAAGGGTCC
GAAGGGTCCAGGGTCGGCTGAG AAGGGAAGGGTCCAGGGTCGGC
GGAAGGGAAGGGTCCAGGGTCG 120151493 rs7311975

HG00106 >hsa-mir-548ak GCAAAAGTAACTGCGGTTTTTG
TGCAAAAGTAACTGCGGTTTTT CAAAAGTAACTGCGGTTTTTGA
GTGCAAAAGTAACTGCGGTTTT rs7070684

HG00106 >hsa-mir-3196GCGGGGCGGCAGGGGCCTCCCC GGCGGGGCGGCAGGGGCCTCCC
GGGCGGGGCGGCAGGGGCCTCC TGGGGGCGGGGCGGCAGGGGCC
GGGGCGGGGCGGCAGGGGCCTC 61870167 rs744591

HG00106 >hsa-mir-3620GGTGGGGGCCAGCAGGGAGTGG TGAGGTGGGGGCCAGCAGGGAG
GTGAGGTGGGGGCCAGCAGGGA GAGGTGGGGGCCAGCAGGGAGT
AGGTGGGGGCCAGCAGGGAGTG 228284991 rs2070960

HG00106 >hsa-mir-3144TAAGGGGACCAAAGAGATATAT TTAAGGGGACCAAAGAGATATA
AAGGGGACCAAAGAGATATATA CTACACTTTAAGGGGACCAAAG
TTTAAGGGGACCAAAGAGATAT 120336384 rs67106263

HG00106 >hsa-mir-3144TAAGGGGACCAAAGAGATATAT AAGGGGACCAAAGAGATATATA
TTAAGGGGACCAAAGAGATATA ATACACTTTAAGGGGACCAAAG
TTTAAGGGGACCAAAGAGATAT 120336327 rs68035463 120336384
rs67106263
HG00106 >hsa-mir-4326TCTGCTGTTCCTCTGTCTCCCA TGGTCTGCTGTTCCTCTGTCTC
CTGGTCTGCTGTTCCTCTGTCT GCTGTTCCTCTGTCTCCCAGAC
TGCTGTTCCTCTGTCTCCCAGA 61918164 rs6062431

HG00106 >hsa-mir-744 TTGGGCAAGGTGCGGGGCTAGG TGGGCAAGGTGCGGGGCTAGGG
CGGGGCTAGGGCTAACAGCAGT GTGCGGGGCTAGGGCTAACAGC
CTAGGGCTAACAGCAGTCTTAC 11985275 .

HG00106 >hsa-mir-553 TTTAAGACGGTGAGATTTTGTT TATTTTAAGACGGTGAGATTTT
TTATTTTAAGACGGTGAGATTT TTTTATTTTAAGACGGTGAGAT
AATTTTATTTTAAGACGGTGAG 100746814 rs190622705

HG00106 >hsa-mir-553 TTTAAGACGGTGAGATTTTGTT TTTTAAGACGGTGAGATTTTGT
TATTTTAAGACGGTGAGATTTT TTATTTTAAGACGGTGAGATTT
TTTTATTTTAAGACGGTGAGAT 100746814 rs190622705 100746835 .

HG00106 >hsa-mir-553 TTTTAAAACGGTGAGATTTTGT TTATTTTAAAACGGTGAGATTT
TTTTATTTTAAAACGGTGAGAT TTTAAAACGGTGAGATTTTGTT
TATTTTAAAACGGTGAGATTTT 100746835 .

HG00106 >hsa-mir-553 TTTTAAAACGGTGAGATTTTGT TATTTTAAAACGGTGAGATTTT
TTTTATTTTAAAACGGTGAGAT TTTAAAACGGTGAGATTTTGTT
ATTTTATTTTAAAACGGTGAGA 100746848 .

HG00106 >hsa-mir-553 TTTTAAAACGGTGAGATTTTGT TATTTTAAAACGGTGAGATTTT
TTTTATTTTAAAACGGTGAGAT TTTAAAACGGTGAGATTTTGTT
ATTTTATTTTAAAACGGTGAGA 100746835 . 100746848 .

HG00106 >hsa-mir-553 TTTTAAGACGGTGAGATTTTGT TATTTTAAGACGGTGAGATTTT
TTTTATTTTAAGACGGTGAGAT TTTAAGACGGTGAGATTTTGTT
ATTTTATTTTAAGACGGTGAGA 100746814 rs190622705 100746848 .

HG00106 >hsa-mir-553 TTTTAAGACGGTGAGATTTTGT TATTTTAAGACGGTGAGATTTT
TTTTATTTTAAGACGGTGAGAT TTTAAGACGGTGAGATTTTGTT
ATTTTATTTTAAGACGGTGAGA 100746814 rs190622705 100746835 .
100746848 .
HG00106 >hsa-mir-486-2 ACTGAGCTGCCCCGAGCTGGGC
CTGAGCTGCCCCGAGCTGGGCA CTGTACTGAGCTGCCCCGAGCT
GTACTGAGCTGCCCCGAGCTGG CCTGTACTGAGCTGCCCCGAGC 41518007 .

HG00106 >hsa-mir-3175AGAGAACGCAGTGACGTCTGGC GAGAGAACGCAGTGACGTCTGG
GGAGAGAACGCAGTGACGTCTG GGGAGAGAACGCAGTGACGTCT
GGGGAGAGAACGCAGTGACGTC 93447631 rs1439619

HG00106 >hsa-mir-1269b AGCCATGCTACGGGCTTCTCTG
ACTGAGCCATGCTACGGGCTTC AGGTTTCTGGACTGAGCCATGC
TGAGGTTTCTGGACTGAGCCAT TTTCTGGACTGAGCCATGCTAC 12820632
rs12451747
HG00106 >hsa-mir-548ap AGTAATTGCAGTCTTTGTCATT
AAGTAATTGCAGTCTTTGTCAT AAAGTAATTGCAGTCTTTGTCA
AAAAGTAATTGCAGTCTTTGTC CAAAAGTAATTGCAGTCTTTGT 86368898
rs4414449
HG00106 >hsa-mir-548ap AGTAATTGCAGTCTTTGTCATT
AAGTAATTGCAGTCTTTGTCAT AAAGTAATTGCAGTCTTTGTCA
AAAAGTAATTGCAGTCTTTGTC CAAAAGTAATTGCAGTCTTTGT 86368898
rs4414449 86368959 rs4577031
HG00106 >hsa-mir-548ap AGTAATTGCGGTCTTTGTCATT
AAGTAATTGCGGTCTTTGTCAT AAAGTAATTGCGGTCTTTGTCA
AAAAGTAATTGCGGTCTTTGTC CAAAAGTAATTGCGGTCTTTGT 86368959

rs4577031
HG00106 >hsa-mir-196a-2 AGTTTCATGTTGTTGGGATTGA
TAGTTTCATGTTGTTGGGATTG AGGTAGTTTCATGTTGTTGGGA
GTAGTTTCATGTTGTTGGGATT GGTAGTTTCATGTTGTTGGGAT 54385599
rs11614913
HG00106 >hsa-mir-3622b ATGGGAGGTCAGGTGAGCTCAG
CATGGGAGGTCAGGTGAGCTCA GCATGGGAGGTCAGGTGAGCTC
AGGCATGGGAGGTCAGGTGAGC GGCATGGGAGGTCAGGTGAGCT 27559214
rs66683138
HG00106 >hsa-mir-500bCTTGCTACCTGGGTGAGAGTGC ATCCTTGCTACCTGGGTGAGAG
AATCCTTGCTACCTGGGTGAGA CCTTGCTACCTGGGTGAGAGTG
TCCTTGCTACCTGGGTGAGAGT 49775351 rs151318590

HG00106 >hsa-mir-650 GAGGCAGCGCTCTCAGGACGTC GGAGGCAGCGCTCTCAGGACGT
TCAGGAGGCAGCGCTCTCAGGA TCTCAGGAGGCAGCGCTCTCAG
GTCTCAGGAGGCAGCGCTCTCA 23165340 rs5996397

HG00106 >hsa-mir-744 GGGCTAACAGCAGTCTTACTGA GCTAGGGCTAACAGCAGTCTTA
TAGGGCTAACAGCAGTCTTACT GGCTAGGGCTAACAGCAGTCTT
CTAGGGCTAACAGCAGTCTTAC 11985275 .

HG00106 >hsa-mir-548h-3 GTAATCGTGGTTTTTGTCATTG
TGCAAAAGTAATCGTGGTTTTT TGGTGCAAAAGTAATCGTGGTT
TTGGTGCAAAAGTAATCGTGGT AGTAATCGTGGTTTTTGTCATT 13446924
rs9913045
HG00106 >hsa-mir-573 GTGTAACTGATCAGGATCTACT TGTGTAACTGATCAGGATCTAC
GATGTGTAACTGATCAGGATCT TGATGTGTAACTGATCAGGATC
GTGATGTGTAACTGATCAGGAT 24521902 rs76014664

HG00106 >hsa-mir-573 GTGTAACTGATCAGGATCTACT TGTGTAACTGATCAGGATCTAC
GATGTGTAACTGATCAGGATCT TGATGTGTAACTGATCAGGATC
GTGATGTGTAACTGATCAGGAT 24521904 rs78830737

HG00106 >hsa-mir-573 GTGTAACTGATCAGGATCTACT TGTGTAACTGATCAGGATCTAC
GATGTGTAACTGATCAGGATCT TGATGTGTAACTGATCAGGATC
GTGATGTGTAACTGATCAGGAT 24521902 rs76014664 24521904
rs78830737
HG00106 >hsa-mir-3117TAAAGGGCCAGACACTATACGA GCCAGACACTATACGAGTCATA
GGGCCAGACACTATACGAGTCA GGCCAGACACTATACGAGTCAT
CCCTAAAGGGCCAGACACTATA 67094171 rs12402181

HG00106 >hsa-mir-3144TAAGGGGACCAAAGAGATATAT TTAAGGGGACCAAAGAGATATA
TTTAAGGGGACCAAAGAGATAT TACACTTTAAGGGGACCAAAGA
AAATACACTTTAAGGGGACCAA 120336327 rs68035463

HG00106 >hsa-mir-3144TAAGGGGACCAAAGAGATATAT TTAAGGGGACCAAAGAGATATA
TTTAAGGGGACCAAAGAGATAT TACACTTTAAGGGGACCAAAGA

AACTACACTTTAAGGGGACCAA 120336384 rs67106263

HG00106 >hsa-mir-3144TAAGGGGACCAAAGAGATATAT TTAAGGGGACCAAAGAGATATA TTTAAGGGGACCAAAGAGATAT TACACTTTAAGGGGACCAAAGA AAATACACTTTAAGGGGACCAA 120336327 rs68035463 120336384 rs67106263

HG00106 >hsa-mir-629 TACGTTGGGAGAACTTTTACGG TTACGTTGGGAGAACTTTTACG GTTTACGTTGGGAGAACTTTTA TTTACGTTGGGAGAACTTTTAC GGTTTACGTTGGGAGAACTTTT 70371794 .

HG00106 >hsa-mir-629 TACGTTGGGAGAACTTTTATGG TTACGTTGGGAGAACTTTTATG TTTACGTTGGGAGAACTTTTAT TGGGTTTACGTTGGGAGAACTT GTTTACGTTGGGAGAACTTTTA 70371761 rs377691713

HG00106 >hsa-mir-629 TACGTTGGGAGAACTTTTATGG TTACGTTGGGAGAACTTTTATG TTTACGTTGGGAGAACTTTTAT TGGGTTTACGTTGGGAGAACTT GTTTACGTTGGGAGAACTTTTA 70371761 rs377691713 70371794 .

HG00106 >hsa-mir-888 TACTCAAAAAGCTGTCAGTCAC TCTACTCAAAAAGCTGTCAGTC TGCTCTACTCAAAAAGCTGTCA GCTCTACTCAAAAAGCTGTCAG GTGCTCTACTCAAAAAGCTGTC 145076302 rs5965660

HG00106 >hsa-mir-888 TACTCAAAAAGCTTTCAGTCAC TCTACTCAAAAAGCTTTCAGTC TGCTCTACTCAAAAAGCTTTCA GCTCTACTCAAAAAGCTTTCAG GTGCTCTACTCAAAAAGCTTTC 145076355 rs143634721

HG00106 >hsa-mir-888 TACTCAAAAAGCTTTCAGTCAC TCTACTCAAAAAGCTTTCAGTC TGCTCTACTCAAAAAGCTTTCA GCTCTACTCAAAAAGCTTTCAG GTGCTCTACTCAAAAAGCTTTC 145076302 rs5965660 145076355 rs143634721

HG00106 >hsa-mir-3125TAGAGGAAGCTGTGGAGAGAAC AGCTGTGGAGAGAACTCACGGT TGGATAGAGGAAGCTGTGGAGA AAGCTGTGGAGAGAACTCACGG AGGAAGCTGTGGAGAGAACTCA 12877501 rs78852835

HG00106 >hsa-mir-3199-1 TAGGAGAAAGTTTCTGGAAGTT TTAGGAGAAAGTTTCTGGAAGT TGCCTTAGGAGAAAGTTTCTGG TCCAGGGACTGCCTTAGGAGAA TGACTCCAGGGACTGCCTTAGG 28316513 rs118160653

HG00106 >hsa-mir-449cTAGGCAGTGTATTGCTAGCGGC TCAGATAGGCAGTGTATTGCTA TGTCAGATAGGCAGTGTATTGC TGTGTCAGATAGGCAGTGTATT TGGGATGTGTCAGATAGGCAGT 54468166 rs75661995

HG00106 >hsa-mir-590 TATTCATAAAAGTGCAGTATGG TTATTCATAAAAGTGCAGTATG TTTATTCATAAAAGTGCAGTAT TGAGTTTATTCATAAAAGTGCA GTTTATTCATAAAAGTGCAGTA 73605546 rs189727189

HG00106 >hsa-mir-564 TCAGCAGGCAACATGGCCGAGA TGTCAGCAGGCAACATGGCCGA

GTCAGCAGGCAACATGGCCGAG GTGTCAGCAGGCAACATGGCCG
TGCCAGGCACGGTGTCAGCAGG 44903385 rs114636202

HG00106 >hsa-mir-222 TCAGTAGCCAGTGTAGATCCTG TGGCTCAGTAGCCAGTGTAGAT
TTGGCTCAGTAGCCAGTGTAGA TCATTGGCTCAGTAGCCAGTGT
TACCCTCATTGGCTCAGTAGCC 45606504 rs191727254

HG00106 >hsa-mir-663aTCCCAGGCGGGGCGCCGCGGGA TCCGGCGTCCCAGGCGGGGCGC
TTCCGGCGTCCCAGGCGGGGCG GCGCCGCGGGACCTCCCTCGTG
GGCGCCGCGGGACCTCCCTCGT 26188880 .

HG00106 >hsa-mir-149 TCCGTGTCTTCACTCCCGTGCT TGGCTCCGTGTCTTCACTCCCG
TCTGGCTCCGTGTCTTCACTCC CCGTGTCTTCACTCCCGTGCTT
CTCCGTGTCTTCACTCCCGTGC 241395503 rs2292832

HG00106 >hsa-mir-618 TCCTTCTGAGTGTAATTACGTA TGTCCTTCTGAGTGTAATTACG
TTGTCCTTCTGAGTGTAATTAC TACTTGTCCTTCTGAGTGTAAT
GTCCTTCTGAGTGTAATTACGT 81329527 rs145551269

HG00106 >hsa-mir-618 TCCTTCTGAGTGTAATTACGTA TGTCCTTCTGAGTGTAATTACG
TTGTCCTTCTGAGTGTAATTAC TACTTGTCCTTCTGAGTGTAAT
GTCCTTCTGAGTGTAATTACGT 81329536 rs2682818

HG00106 >hsa-mir-618 TCCTTCTGAGTGTAATTACGTA TGTCCTTCTGAGTGTAATTACG
TTGTCCTTCTGAGTGTAATTAC TACTTGTCCTTCTGAGTGTAAT
GTCCTTCTGAGTGTAATTACGT 81329527 rs145551269 81329536
rs2682818
HG00106 >hsa-mir-492 TCGAGGACCTGCGGGACAAGAT TACAGGACCATCGAGGACCTGC
TACTACAGGACCATCGAGGACC TCCAGCCACTACTACAGGACCA
GACCTGCGGGACAAGATTCTTG 95228179 rs200816308

HG00106 >hsa-mir-3183TCGGAGTCGCTCGGAGCAGTCA TCTCGGAGTCGCTCGGAGCAGT
TCTCTCGGAGTCGCTCGGAGCA TGCCTCTCTCGGAGTCGCTCGG
TGCCCTGCCTCTCTCGGAGTCG 925742 rs72812091

HG00106 >hsa-mir-1200TGAGCCATTCTGAGCCTCAATC TCCTGAGCCATTCTGAGCCTCA
TCTCCTGAGCCATTCTGAGCCT TTCTCCTGAGCCATTCTGAGCC
TACTTCTCCTGAGCCATTCTGA 36958995 rs180826747

HG00106 >hsa-mir-27a TGAGGAGCAGGGCTTAGCTGCT TTAGCTGCTTGTGAGCAGGGTC
GAGGAGCAGGGCTTAGCTGCTT GGAGCAGGGCTTAGCTGCTTGT
GAGCAGGGCTTAGCTGCTTGTG 13947292 rs895819

HG00106 >hsa-mir-3151TGATGGGTGGGGCAATGGGATC TGGGTGGGGCAATGGGATCAGG
TGGGGCAATGGGATCAGGTGCC GGGGTGATGGGTGGGGCAATGG
GGGTGATGGGTGGGGCAATGGG 104166902 rs35605502

HG00106 >hsa-mir-548kTGCAAAAGTACTTGAGGATTTT TGGTGCAAAAGTACTTGAGGAT
TTGGTGCAAAAGTACTTGAGGA GCAAAAGTACTTGAGGATTTTG
TAGGTTGGTGCAAAAGTACTTG 70130103 .

HG00106 >hsa-mir-3622a TGCACAGGCACAGGAGCTCAGG
TAGAGGGTGCACAGGCACAGGA GCACAGGAGCTCAGGTGAGGCA
GGCACAGGAGCTCAGGTGAGGC GAGGGTGCACAGGCACAGGAGC 27559214
rs66683138
HG00106 >hsa-mir-3156-3 TGCAGAAGAAAGATCTGGAAGT
GCAGAAGAAAGATCTGGAAGTG GAAAGATCTGGAAGTGGGAGAC
GAAGAAAGATCTGGAAGTGGGA AGAAGAAAGATCTGGAAGTGGG 14778721
rs2747232
HG00106 >hsa-mir-3152TGCCTCTGTTCTAACACAAGAC TTGCCTCTGTTCTAACACAAGA
TATTGCCTCTGTTCTAACACAA TTATTGCCTCTGTTCTAACACA
TGCAGAGTTATTGCCTCTGTTC 18573360 rs13299349

HG00106 >hsa-mir-1254-2 TGGAAGCTGGAGCCTGCAGTGA
TGAGCCTGGAAGCTGGAGCCTG GAAGCTGGAGCCTGCAGTGAGC
GGAAGCTGGAGCCTGCAGTGAG GCCTGGAAGCTGGAGCCTGCAG 23682383
rs200793185
HG00106 >hsa-mir-378d-2 TGGACTTGGAGTCAGAAAACTT
GACTTGGAGTCAGAAAACTTTC GGACTTGGAGTCAGAAAACTTT
GAACACTGGACTTGGAGTCAGA CTGGACTTGGAGTCAGAAAACT 94928250
rs73692959
HG00106 >hsa-mir-1273h TGGGAGGTCAAGGCTGTAGTGT
TGAGCCTGGGAGGTCAAGGCTG TTGAGCCTGGGAGGTCAAGGCT
TGCTTGAGCCTGGGAGGTCAAG TTGCTTGAGCCTGGGAGGTCAA 24214486 .

HG00106 >hsa-mir-3620TGGGGGCCAGCAGGGAGTGGGT TGAGGTGGGGGCCAGCAGGGAG
GGGGGCCAGCAGGGAGTGGGTT GTGGGGGCCAGCAGGGAGTGGG
GGTGGGGGCCAGCAGGGAGTGG 228284991 rs2070960

HG00106 >hsa-mir-1227TGGTGGGCACTGCTGGGGTGGG TGGGGCCAGGCGGTGGTGGGCA
AGGCGGTGGTGGGCACTGCTGG GGTGGGCACTGCTGGGGTGGGC
GTGGTGGGCACTGCTGGGGTGG 2234093 rs190788838

HG00106 >hsa-mir-323bTGTCCGTGGTGAGTTCGCATTA TTGTCCGTGGTGAGTTCGCATT
TACTCGGAGGGAGGTTGTCCGT TCGGAGGGAGGTTGTCCGTGGT
AGGTTGTCCGTGGTGAGTTCGC 101522556 rs56103835

HG00106 >hsa-mir-152 TGTGATACACTCCGACTCGGGC TCTGTGATACACTCCGACTCGG
TTCTGTGATACACTCCGACTCG TCCCCCCGGCCCAGGTTCTGTG
TTCCCCCCGGCCCAGGTTCTGT 46114610 .

HG00106 >hsa-mir-642bTTCCCTCTCCAAATGTGTCTTG TTGGGAGGTTCCCTCTCCAAAT
TGGGAGGTTCCCTCTCCAAATG GAGTTGGGAGGTTCCCTCTCCA
GTTGGGAGGTTCCCTCTCCAAA 46178217 rs111664333

HG00106 >hsa-mir-4277TTCTGAGCACAGTACACTGGGC TCGAGGCAGTTCTGAGCACAGT TGGGTCGAGGCAGTTCTGAGCA TTGGGTCGAGGCAGTTCTGAGC GTTCTGAGCACAGTACACTGGG 1708983 rs12523324

HG00106 >hsa-mir-1178TTGGCTGGCAGAGGAAGGGAAG TGGCTGGCAGAGGAAGGGAAGG TGGCAGAGGAAGGGAAGGGTCC GCGTTGGCTGGCAGAGGAAGGG GTTGGCTGGCAGAGGAAGGGAA 120151493 rs7311975

HG00106 >hsa-mir-553 TTTAAAACGGTGAGATTTTGTT TTTTAAAACGGTGAGATTTTGT ATTTTAAAACGGTGAGATTTTG TATTTTAAAACGGTGAGATTTT TTATTTTAAAACGGTGAGATTT 100746835 .

HG00106 >hsa-mir-553 TTTAAAACGGTGAGATTTTGTT TTTTAAAACGGTGAGATTTTGT ATTTTAAAACGGTGAGATTTTG TATTTTAAAACGGTGAGATTTT TTATTTTAAAACGGTGAGATTT 100746848 .

HG00106 >hsa-mir-553 TTTAAAACGGTGAGATTTTGTT TTTTAAAACGGTGAGATTTTGT ATTTTAAAACGGTGAGATTTTG TATTTTAAAACGGTGAGATTTT TTATTTTAAAACGGTGAGATTT 100746835 . 100746848 .

HG00106 >hsa-mir-553 TTTAAGACGGTGAGATTTTGTT TTTTAAGACGGTGAGATTTTGT ATTTTAAGACGGTGAGATTTTG TATTTTAAGACGGTGAGATTTT TTATTTTAAGACGGTGAGATTT 100746814 rs190622705

HG00106 >hsa-mir-553 TTTAAGACGGTGAGATTTTGTT TTTTAAGACGGTGAGATTTTGT ATTTTAAGACGGTGAGATTTTG TATTTTAAGACGGTGAGATTTT TTATTTTAAGACGGTGAGATTT 100746814 rs190622705 100746835 .

HG00106 >hsa-mir-553 TTTAAGACGGTGAGATTTTGTT TTTTAAGACGGTGAGATTTTGT ATTTTAAGACGGTGAGATTTTG TATTTTAAGACGGTGAGATTTT TTATTTTAAGACGGTGAGATTT 100746814 rs190622705 100746848 .

HG00106 >hsa-mir-553 TTTAAGACGGTGAGATTTTGTT TTTTAAGACGGTGAGATTTTGT ATTTTAAGACGGTGAGATTTTG TATTTTAAGACGGTGAGATTTT TTATTTTAAGACGGTGAGATTT 100746814 rs190622705 100746835 . 100746848 .

HG00106 >hsa-mir-149 AGGGAGGGAGGGACGGGGGCTG GGGCTGTGCTGGGGCAGCCGGA GGGACGGGGGCTGTGCTGGGGC GACGGGGGCTGTGCTGGGGCAG GGGGCTGTGCTGGGGCAGCCGG 241395503 rs2292832

HG00106 >hsa-mir-658 AGGTCGGTTGGTCGGTCGGGAA GTCGGTTGGTCGGTCGGGAACG TAGGTCGGTTGGTCGGTCGGGA . .

HG00106 >hsa-mir-744 ATGCACATGCTGTTGCCACTAA TGTTGCCACTAACCTCAACCTT GCTGTTGCCACTAACCTCAACC TGCACATGCTGTTGCCACTAAC C 11985275 .

HG00106 >hsa-mir-604 CACAGGCTGCGGAATTCAGGAC ACACAGGCTGCGGAATTCAGGA
ACAGGCTGCGGAATTCAGGACA GGCTGCGGAATTCAGGACAGTG
CTGCGGAATTCAGGACAGTGCA 29833998 rs2368393

HG00106 >hsa-mir-604 CACAGGCTGCGGAATTCAGGAC ACACAGGCTGCGGAATTCAGGA
ACAGGCTGCGGAATTCAGGACA GGCTGCGGAATTCAGGACAGTG
CTGCGGAATTCAGGACAGTGCA 29833998 rs2368393 29834003
rs2368392
HG00106 >hsa-mir-3622a CACCTGACCTCCCATGCCTGTG
CATGCCTGTGCACCCTCTATT CCTGACCTCCCATGCCTGTGCA
ATGCCTGTGCACCCTCTATT ACCTGACCTCCCATGCCTGTGC 27559214
rs66683138
HG00106 >hsa-mir-622 CAGTCTGCTGAGGTTGGAGCCG AGGTTGGAGCCGCTGAGATGAC
TCATCACACAGTCTGCTGAGGT GCTGAGGTTGGAGCCGCTGAGA
GAGGTTGGAGCCGCTGAGATGA 90883517 rs111371406

HG00106 >hsa-mir-3180-4 CTCCGGATGCCAGTCCCTCATC
GGAGGGTGAAGCCTCCGGATGC CTGGCCTGGTCGCGCTGTGGCT
AGCGGAGGGTGAAGCCTCCGGA GAGCGGAGGGTGAAGCCTCCGG 15248720
rs75000738
HG00106 >hsa-mir-4268CTCTCAGGATGTGATGTCACCT CCTCTCAGGATGTGATGTCACC
GCTCCTCCTCTCAGGATGTGAT CTCCTCCTCTCAGGATGTGATG
TCCTCTCAGGATGTGATGTCAC 220771223 rs4674470

HG00106 >hsa-mir-3151CTGATCCCACACCCCACCTGTC TGATCCCACACCCCACCTGTCA
GATCCCACACCCCACCTGTCAC GGGCATCCCACCTGATCCCACA
TCCCACCTGATCCCACACCCCA 104166902 rs35605502

HG00106 >hsa-mir-3620CTGCATCCCGCACCCAG CCTGCATCCCGCACCCAG
TCCCGCACCCAG ATCCCGCACCCAGCATCCCGCACCCAG 228284991 rs2070960

HG00106 >hsa-mir-412 CTTCACCTGGTTCACTAGCCGT ACCTGGTTCACTAGCCGTCCGT
TGTACTTCACCTGGTTCACTAG CTGGTTCACTAGCCGTCCGTAT
GTACTTCACCTGGTTCACTAGC 101531849 .

HG00106 >hsa-mir-658 TAGGTCGGTTGGTCGGTCGGGA .

HG00106 >hsa-mir-658 GTCCGTTGGTCGGTCGGGAACG G
TCCGTTGGTCGGTCGGGAACGA .

HG00106 >hsa-mir-320eGAAAAGCTGGGTTGAGAAGGT AAAAGCTGGGTTGAGAAGGT
GGAAAAGCTGGGTTGAGAAGGT GGGAAAAGCTGGGTTGAGAAGG rs10423365

HG00106 >hsa-mir-1343GCCCCTCCTGGGGCCCGCACTC CCCCTCCTGGGGCCCGCACTCT
GGGGCCCGCACTCTCGCTCTGG CCCTCCTGGGGCCCGCACTCTC
TGGGGCCCGCACTCTCGCTCTG 34963416 rs2986407

HG00106 >hsa-mir-3180-4 GGAGGGTGAAGCCTCCGGATGC
GGTGAAGCCTCCGGATGCCAGT AGCGGAGGGTGAAGCCTCCGGA
GCGGAGGGTGAAGCCTCCGGAT GCCTGGTCGCGCTGTGGCGAAG 15248798
rs183853838
HG00106 >hsa-mir-3180-4 GGAGGGTGAAGCCTCCGGATGC
GGTGAAGCCTCCGGATGCCAGT AGCGGAGGGTGAAGCCTCCGGA
GCGGAGGGTGAAGCCTCCGGAT CTGGCCTGGTCGCGCTGTGGCT 15248720
rs75000738 15248798 rs183853838
HG00106 >hsa-mir-1227GGCATTTGACCCCGTGCCACCC AGGCATTTGACCCCGTGCCACC
AGGCATTTGACCCCGTGCCACC TGACCCCGTGCCACCCTTTTCC
ATTTGACCCCGTGCCACCCTTT 2234093 rs190788838

HG00106 >hsa-mir-580 TATTTGAGAATGATGAATCATT TGAATCATTAGGTTCCGGTCAG
ATGAATCATTAGGTTCCGGTCA TTTGAGAATGATGAATCATTAG
GAGAATGATGAATCATTAGGTT 36148057 rs115089112

HG00106 >hsa-mir-3118-1 TGAAAATTCTTCTAGTGTG ATGAAAATTCTTCTAGTGTG
TGCATTATGAAAATTCTTCTAG TTATGAAAATTCTTCTAGTGTG
ATTATGAAAATTCTTCTAGTGT 142667330 rs76132421

HG00106 >hsa-mir-558 TGAGCTGCTGTACCAAAATACC GCTGCTGTACCAAAATACCACA
TGCTGTACCAAAATACCACAAA CTGAGCTGCTGTACCAAAATAC
GAGCTGCTGTACCAAAATACCA 32757230 rs72089144

HG00106 >hsa-mir-637 TGGCTAAGGTGTTGGCTCGGGC TGGCTAAGGTGTTGGCTCGGGC
.
HG00106 >hsa-mir-320c-1 AAAAGCTGGGTTGAGAGGGTAG
AGCTGGGTTGAGAGGGTAGGAA CTGGGTTGAGAGGGTAGGAAAA
AGGGTAGGAAAAAAATGATGTA 101491923 rs141611518

HG00106 >hsa-mir-202 AAAGAGGTATAGGGCATGGGAA AAGAGGTATAGGGCATGGGAAA
GGGAAAACGGGGCGGTCGGGTC TAAAGAGGTATAGGGCATGGGA 2234093
rs190788838
HG00106 >hsa-mir-520hAAAGTGCTTCCCTTTAGAGTTA rs148716001 74

HG00106 >hsa-mir-548ap AACAAAAACCACAATTACTTTT
CAAAAACCACAATTACTTTTTA CAATTACTTTTTACTGACCTAA rs4414449
24214486 .
HG00106 >hsa-mir-548ap AACAAAAACCACAATTACTTTT
CAAAAACCACAATTACTTTTTA TTACTTTTTACTGACCTAAAGA rs4577031
154065347 rs142414368
HG00106 >hsa-mir-548ap AACAAAAACCACAATTACTTTT
CAAAAACCACAATTACTTTTTA CAATTACTTTTTACTGACCTAA rs4414449
93466866 rs2155248
HG00106 >hsa-mir-548al AACGGCAGTGACTTTTGTACCA
TGGCAAAAACGGCAGTGACTTT TAAAAGTAATGGCAAAAACGGC
AAAAGTAATGGCAAAAACGGCA 52013832 rs374103744

HG00106 >hsa-mir-423 AAGCTCGGTCTGAGGCCCCTCA AGGCCCCTCAGTCTTGCTTCCT TCTGAGGCCCCTCAGTCTTGCT GTCTGAGGCCCCTCAGTCTTGC 34963416 rs2986407

HG00106 >hsa-mir-622 ACACAGTCTGCTGAGGTTGGAG CTGCTGAGGTTGGAGCCGCTGA ACACAGTCTGCTGAGGTTGGAG rs111371406 241395503 rs2292832

HG00106 >hsa-mir-597 ACAGTGGTTCTCTTGTGGCTCA GGCTCAAGCGTAATGTAGAGTA CAAGCGTAATGTAGAGTACTGA rs79397096 46114610 .

HG00106 >hsa-mir-449cACAGTTGCTAGTTGCACTCCTC AACAGTTGCTAGTTGCACTCCT GTTGCTAGTTGCACTCCTCTCT GTTGCACTCCTCTCTGTTGCAT

HG00106 >hsa-mir-3118-1 ACTGCATTATGAAAATTCTTCT ATTATGAAAATTCTTCTAGTGT GCATTATGAAAATTCTTCTAGT CTGCATTATGAAAATTCTTCTA 54385599 rs11614913

HG00106 >hsa-mir-3166AGACAATGCCTACTGGCCTAAG ATGCCTACTGGCCTAAGAAAAA AACGCAGACAATGCCTACTGGC CAATGCCTACTGGCCTAAGAAA 135061112 rs12355840

HG00106 >hsa-mir-605 AGAGAAGGCACTATGAGATTTA GGCACTATGAGATTTAGAACCA CAGAGAAGGCACTATGAGATTT GAGAAGGCACTATGAGATTTAG 113655752 rs10505168

HG00106 >hsa-mir-642bAGATACATTTGGAGAGGGACCC TTGGAGAGGGACCCTCCCAACT TTTGGAGAGGGACCCTCCCAAC ATACATTTGGAGAGGGACCCTC 41522213 rs7207008

HG00106 >hsa-mir-3180-4 AGCGGAGGGTGAAGCCTCCGGA GGAGGGTGAAGCCTCCGGATGC GAGCGGAGGGTGAAGCCTCCGG GCGGAGGGTGAAGCCTCCGGAT

HG00106 >hsa-mir-3180-4 AGCGGAGGGTGAAGCCTCCGGA GGAGGGTGAAGCCTCCGGATGC GAGCGGAGGGTGAAGCCTCCGG GCGGAGGGTGAAGCCTCCGGAT 45606504 rs191727254

HG00106 >hsa-mir-30d AGCTTTCAGTCAGATGTTTGCT GGCTAAGCTTTCAGTCAGATGT GCTAAGCTTTCAGTCAGATGTT TTCAGTCAGATGTTTGCTGCTA 13947292 rs895819

HG00106 >hsa-mir-519a-2 AGGAAAGTGCATCCTTTTAGAG AGTGCATCCTTTTAGAGGGTTA GGAAAGTGCATCCTTTTAGAGG GAAAGGAAAGTGCATCCTTTTA

HG00106 >hsa-mir-630 AGTATTCTGTACCAGGGAAGGT ACCTAGTATTCTGTACCAGGGA CCAGGGAAGGTAGTTCTTAACT GGAAGGTAGTTCTTAACTATGT 135817150 .

HG00106 >hsa-mir-513cATAAATTTCACCTTTCTGAGAA TTCTGAGAAGAGTAATGTACAG CCTTTCTGAGAAGAGTAATGTA TTTCACCTTTCTGAGAAGAGTA 67094171 rs12402181

HG00106 >hsa-mir-323bATACACGGTCGACCTCTTTTCG TACACGGTCGACCTCTTTTCGG
ACACGGTCGACCTCTTTTCGGT rs56103835 142667330 rs76132421

HG00106 >hsa-mir-3144ATACCTGTTCAGTCTCTTTAAA TTCAGTCTCTTTAAAGTGTAGT
CCTGTTCAGTCTCTTTAAAGTG TGTTCAGTCTCTTTAAAGTGTA 120336327
rs68035463
HG00106 >hsa-mir-3144ATACCTGTTCAGTCTCTTTAAA TTCAGTCTCTTTAAAGTGTAGT
CTGTTCAGTCTCTTTAAAGTGT TATACCTGTTCAGTCTCTTTAA 120336384
rs67106263
HG00106 >hsa-mir-3144ATACCTGTTCGGTCTCTTTAAA CTGTTCGGTCTCTTTAAAGTGT
GTTCGGTCTCTTTAAAGTGTAG TGTTCGGTCTCTTTAAAGTGTA 120336327
rs68035463 120336384 rs67106263
HG00106 >hsa-mir-642aATTTGGAGAGGGAACCTCCCAA AGACACATTTGGAGAGGGAACC
ACACATTTGGAGAGGGAACCTC CACATTTGGAGAGGGAACCTCC 104166902
rs35605502
HG00106 >hsa-mir-4293CACACCAGCCTGACAGGAACAG CACCAGCCTGACAGGAACAGCC
TCACACCAGCCTGACAGGAACA CAGCCTGACAGGAACAGCCTGT 18573360
rs13299349
HG00106 >hsa-mir-604 CACAGGCTGCGGAATTCAGGAC TGACACAGGCTGCGGAATTCAG
GACACAGGCTGCGGAATTCAGG rs2368392 87909701 .

HG00106 >hsa-mir-942 CACATGGCTGAAACAGAGAAGT AACAGAGAAGTTACTTTCCTAA
TGGCTGAAACAGAGAAGTTACT TGAAACAGAGAAGTTACTTTCC 15248720
rs75000738
HG00106 >hsa-mir-4265CAGCTGTGGGCTCAACTCTGGG ATCTCTGCAGCTGTGGGCTCAA
GATCTCTGCAGCTGTGGGCTCA CTGCAGCTGTGGGCTCAACTCT 15248798
rs183853838
HG00106 >hsa-mir-152 CAGTCAGTGCATGACAGAACTT GCAGTCAGTGCATGACAGAACT
GTGCATGACAGAACTTGGGCCC GCATGACAGAACTTGGGCCCGG 15248720
rs75000738 15248798 rs183853838
HG00106 >hsa-mir-744 CATGCTGTTGCCACTAACCTCA GCTGTTGCCACTAACCTCAACC
CACTAACCTCAACCTTACTCGG . 19263542 .

HG00106 >hsa-mir-1227CATTTGACCCCGTGCCACCCTT ATTTGACCCCGTGCCACCCTTT
AGGCATTTGACCCCGTGCCACC GACCCCGTGCCACCCTTTTCCC

HG00106 >hsa-mir-3151CCACCTGATCCCACACCCCACC CACCTGATCCCACACCCCACCT
CCCACCTGATCCCACACCCCAC TGATCCCACACCCCACCTGTCA

HG00106 >hsa-mir-1343CCCCTCCTGGGGCCCGCACTCT CCCTCCTGGGGCCCGCACTCTC
CCTGGGGCCCGCACTCTCGCTC TGGGGCCCGCACTCTCGCTCTG 72744798
rs745666
HG00106 >hsa-mir-3679CCCTTCCCCCCAGTAATCTTCA CCTTCCCCCCAGTAATCTTCAT
TCCCCCCAGTAATCTTCATCAT CTTCCCCCCAGTAATCTTCATC

HG00106 >hsa-mir-3622b CCTCACCTGAGCTCCTGTGCCT
CCTGAGCTCCTGTGCCTGTGCA CTCCTGTGCCTGTGCACCCTCT

CTGAGCTCCTGTGCCTGTGCAC 27559214 rs66683138

HG00106 >hsa-mir-4268CCTCTCAGGATGTGATGTCACC CTCCTCTCAGGATGTGATGTCA CTCTCAGGATGTGATGTCACCT rs4674470 27559214 rs66683138

HG00106 >hsa-mir-4254CCTGGAGATACTCCACCATCTC AGATACTCCACCATCTCCCCCA GGAGATACTCCACCATCTCCCC rs12731294 134884700 rs6430498

HG00106 >hsa-mir-3152CCTGTGTTAGAATAAGGGCAAT TTAGAATAAGGGCAATAACTCT AGAATAAGGGCAATAACTCTGC TGTGTTAGAATAAGGGCAATAA

HG00106 >hsa-mir-3620CTCACCCTGCATCCCGCACCCA CCTCACCCTGCATCCCGCACCC CTCACCCTGCATCCCGCACCCA rs2070960 131701279 rs367805

HG00106 >hsa-mir-3622a CTCACCTGACCTCCCATGCCTG CCTGACCTCCCATGCCTGTGCA GCTCACCTGACCTCCCATGCCT CTGACCTCCCATGCCTGTGCAC 101531849 .

HG00106 >hsa-mir-3117CTCATATAGTGCCAGGTGTTTT GACTCATATAGTGCCAGGTGTT TCATATAGTGCCAGGTGTTTTG ATAAGACTCATATAGTGCCAGG 28444183 rs6505162

HG00106 >hsa-mir-196a-2 CTCGGCAACAAGAAACTGTCTG ACTCGGCAACAAGAAACTGTCT CAACAAGAAACTGTCTGAGTTA ACAAGAAACTGTCTGAGTTACA

HG00106 >hsa-mir-486-2 CTCGGCGCAGCTCAGTACAGGA AGGGCCTCGGCGCAGCTCAGTA TCGGCGCAGCTCAGTACAGGAT GGGCCTCGGCGCAGCTCAGTAC 109757963 rs4676066

HG00106 >hsa-mir-3615CTCTCTCGGCTCCTCGCGGCTC GGCTCCTCGCGGCTCGCGGCGG CGGCTCCTCGCGGCTCGCGGCG TCGGCTCCTCGCGGCTCGCGGC

HG00106 >hsa-mir-888 CTCTTTGGGTGAAGGAAGGCTC CTGACACCTCTTTGGGTGAAGG GACTGACACCTCTTTGGGTGAA CCTCTTTGGGTGAAGGAAGGCT 14425204 rs12780876

HG00106 >hsa-mir-888 CTCTTTGGGTGAAGGAAGGCTC CTGACACCTCTTTGGGTGAAGG GACTGACACCTCTTTGGGTGAA CCTCTTTGGGTGAAGGAAGGCT 54468166 rs75661995

HG00106 >hsa-mir-1269a CTGGACTGAGCCATGCTACTGG TGCCTGGACTGAGCCATGCTAC AATGCCTGGACTGAGCCATGCT rs73239138 41518007 .

HG00106 >hsa-mir-2117CTGTTCTCTTTGCCAAGGACAG GCTGTTCTCTTTGCCAAGGACA TCTCTTTGCCAAGGACAGATCT TGTTCTCTTTGCCAAGGACAGA 49775351 rs151318590

HG00106 >hsa-mir-888 GACTGACACCTCTTTGGGTGAA GACACCTCTTTGGGTGAAGGAA TGACACCTCTTTGGGTGAAGGA CACCTCTTTGGGTGAAGGAAGG 146271303 rs145416750

HG00106 >hsa-mir-629 GAGGTTCTCCCAACGTAAGCCC AGGTTCTCCCAACGTAAGCCCA TCTCCCAACGTAAGCCCAGCCC CAGGAGGTTCTCCCAACGTAAG 54265670 .

HG00106 >hsa-mir-629 GAGGTTCTCCCAACGTAAGCCC AGGAGGTTCTCCCAACGTAAGC GGAGGTTCTCCCAACGTAAGCC TCTCCCAACGTAAGCCCAGCCC

HG00106 >hsa-mir-629 GAGGTTCTCCCAACGTAAGCCC AGGTTCTCCCAACGTAAGCCCA TCTCCCAACGTAAGCCCAGCCC CCCAACGTAAGCCCAGCCCCTC 49767832 rs456615

HG00106 >hsa-mir-222 GCAGCTACATCTGGCTACTGGG TACTGGGTCTCTGATGGCATCT GCTACTGGGTCTCTGATGGCAT CTGGCTACTGGGTCTCTGATGG 49767835 rs456617

HG00106 >hsa-mir-500bGCAGTGCACCCAGGCAAGGATT CACCCAGGCAAGGATTCTGCGA AGGCAAGGATTCTGCGAGGGGG TGCAGTGCACCCAGGCAAGGAT 49767832 rs456615 49767835 rs456617

HG00106 >hsa-mir-3180-4 GCGGAGGGTGAAGCCTCCGGAT CGCTGGCCTGGTCGCGCTGTGG TCGCTGGCCTGGTCGCGCTGTG AAGCCTCCGGATGCCAGTCCCT 74110353 rs515924

HG00106 >hsa-mir-149 GGGAGGGAGGGACGGGGGCTGT GGAGGGACGGGGGCTGTGCTGG AGGGACGGGGGCTGTGCTGGGG GACGGGGGCTGTGCTGGGGCAG

HG00106 >hsa-mir-1197GTAGGACACATGGTCTACTTCT ACACATGGTCTACTTCTTCTCA ACATGGTCTACTTCTTCTCAAT TAGGACACATGGTCTACTTCTT

HG00106 >hsa-mir-27a GTGTTCACAGTGGCTAAGTTCC AGTGGCTAAGTTCCGCCCCCCA TCGTGTTCACAGTGGCTAAGTT CACAGTGGCTAAGTTCCGCCCC 86368959 rs4577031

HG00106 >hsa-mir-606 TAAACTACTGAAAATCAAAGAT CCATCATAGTAAACTACTGAAA ACTACTGAAAATCAAAGATACA CCCATCATAGTAAACTACTGAA

HG00106 >hsa-mir-3936TAAGGGGTGTATGGCAGATGCA CACCCGACAGATGCACTTGGCA GATGCACCCGACAGATGCACTT TGTATGGCAGATGCACCCGACA 32757230 rs72089144

HG00106 >hsa-mir-300 TATACAAGGGCAGACTCTCTCT TGATTATACAAGGGCAGACTCT ATTATACAAGGGCAGACTCTCT rs12894467 36148057 rs115089112

HG00106 >hsa-mir-412 TCACCTGGTTCACTAGCCGTCC TCACCTGGTTCACTAGCCGTCC ATGTACTTCACCTGGTTCACTA CTTCACCTGGTTCACTAGCCGT 168690612 rs62376934

HG00106 >hsa-mir-1178TCACTGTTCTTCCCTAGAATGT TGCTCACTGTTCTTCCCTAGAA GTTGCTCACTGTTCTTCCCTAG CAGGTTGCTCACTGTTCTTCCC 168690635 rs62376935

HG00106 >hsa-mir-532 TCCCACACCCAAGGCTTGCAGA CTCCCACACCCAAGGCTTGCAG CCTCCCACACCCAAGGCTTGCA CACCCAAGGCTTGCAGAAGAGC 168690612 rs62376934 168690635 rs62376935

HG00106 >hsa-mir-532 TCCCACACCCAAGGCTTGCAGA CTCCCACACCCAAGGCTTGCAG

CCTCCCACACCCAAGGCTTGCA CACCCAAGGCTTGCAGAAGAGC 73605546 rs189727189
HG00106 >hsa-mir-532 TCCCACACCCAAGGCTTGCAGA CTCCCACACCCAAGGCTTGCAG CCTCCCACACCCAAGGCTTGCA CACCCAAGGCTTGCAGAAGAGC

HG00106 >hsa-mir-558 TCCTGAGCTGCTGTACCAAAAT TTCCTGAGCTGCTGTACCAAAA GAGCTGCTGTACCAAAATACCA CCTGAGCTGCTGTACCAAAATA 29833998 rs2368393
HG00106 >hsa-mir-1304TCTCACTGTAGCATCGAACCCC GAACCCCTGGGCTCAAGTGATT CGAACCCCTGGGCTCAAGTGAT CTCACTGTAGCATCGAACCCCT

HG00106 >hsa-mir-604 TGACACAGGCTGCGGAATTCAG CACAGGCTGCGGAATTCAGGAC GACACAGGCTGCGGAATTCAGG TTTCTGACACAGGCTGCGGAAT 29833998 rs2368393 29834003 rs2368392
HG00106 >hsa-mir-604 TGACACAGGCTGCGGAATTCAG GACACAGGCTGCGGAATTCAGG CACAGGCTGCGGAATTCAGGAC TTTCTGACACAGGCTGCGGAAT 53059406 rs2043556
HG00106 >hsa-mir-1273h TGCTGCAGACTCGACCTCCCAG TGCAGACTCGACCTCCCAGGCT CTGCAGACTCGACCTCCCAGGC AGACTCGACCTCCCAGGCTTAA 77312301 .

HG00106 >hsa-mir-590 TGTAATTTTATGTATAAGCTAG AATCTGTAATTTTATGTATAAG GTATAAGCTAGTCTCTGATTGA TTTTATGTATAAGCTAGTCTCT

HG00106 >hsa-mir-2053TGTTAATTAAACCTCTATTTAC ACTTTAAGTGTTAATTAAACCT TTTAAGTGTTAATTAAACCTCT GTTAATTAAACCTCTATTTACA 70371761 rs377691713
HG00106 >hsa-mir-585 TTGGACGTATCTGTATGCTAGG ACGTATCTGTATGCTAGGGCTG TGGACGTATCTGTATGCTAGGG TATCTGTATGCTAGGGCTGCTG 70371794 .
HG00106 >hsa-mir-585 TTGGACGTATCTGTATGCTAGG TATCTGTATGCTAGGGCTGCCG ACGTATCTGTATGCTAGGGCTG TGGACGTATCTGTATGCTAGGG 70371761 rs377691713 70371794 .
HG00106 >hsa-mir-585 TTGGGCGTATCTGTATGCTAGG TATCTGTATGCTAGGGCTGCCG TGGGCGTATCTGTATGCTAGGG GCGTATCTGTATGCTAGGGCTG 72879653 rs113971639
HG00106 >hsa-mir-1303TTTAGAGACGGGGTCTTGCTCT TAGAGACGGGGTCTTGCTCTGT GGGTCTTGCTCTGTTGCCAGGC ACGGGGTCTTGCTCTGTTGCCA

HG00106 >hsa-mir-580 TTTGAGAATGATGAATCATTAG GATGAATCATTAGGTTCCGGTC AATGATGAATCATTAGGTTCCG AGAATGATGAATCATTAGGTTC 46178217 rs111664333
HG00106 >hsa-mir-133bTTTGGTCCCCTTCAACCAGCTA TGGTCCCCTTCAACCAGCTACA CCTTCAACCAGCTACAGCAGGG AGAGGTTTGGTCCCCTTCAACC 46178217 rs111664333
HG00107 >hsa-mir-3144AAGGGGACCAAAGAGATATATA TAAGGGGACCAAAGAGATATAT TTAAGGGGACCAAAGAGATATA TTTAAGGGGACCAAAGAGATAT

ATACACTTTAAGGGGACCAAAG 120336327 rs68035463

HG00107 >hsa-mir-3125AGAATGGATAGAGGAAGCTGTG GAGGAAGCTGTGGAGAGAACTC
AGAGGAAGCTGTGGAGAGAACT GCTGTGGAGAGAACTCACGGTG
GGAAGCTGTGGAGAGAACTCAC 12877501 rs78852835

HG00107 >hsa-mir-1908CCGCGGCGGGGACGGCGATTGG GCGGCGGGGACGGCGATTGGTC
CGCGGCGGGGACGGCGATTGGT CGGGGACGGCGATTGGTCCGTA
GGGATGCCGCGGCGGGGACGGC 61582708 rs174561

HG00107 >hsa-mir-1273h CCTGGGAGGTCAAGGCTGTAGT
TGGGAGGTCAAGGCTGTAGTGT ATTGCTTGAGCCTGGGAGGTCA
GCCTGGGAGGTCAAGGCTGTAG TTGAGCCTGGGAGGTCAAGGCT 24214486 .

HG00107 >hsa-mir-1237CGCGGGCAGGGGTGTGGGTGGC CAGGGGTGTGGGTGGCAGAGCG
AGGCGCGGGCAGGGGTGTGGGT GGCCCAGGCGCGGGCAGGGGTG
GGGGTGTGGGTGGCAGAGCGCT 64136103 rs199916083

HG00107 >hsa-mir-1178GAAGGGAAGGGTTCAGGGTCAG GAAGGGTTCAGGGTCAGCTGAG
AAGGGAAGGGTTCAGGGTCAGC GGAAGGGTTCAGGGTCAGCTGA
GTTGGCTGGCAGAGGAAGGGAA 120151501 rs74614893

HG00107 >hsa-mir-3144TAAGGGGACCAAAGAGATATAT TTAAGGGGACCAAAGAGATATA
AAGGGGACCAAAGAGATATATA CTACACTTTAAGGGGACCAAAG
TTTAAGGGGACCAAAGAGATAT 120336384 rs67106263

HG00107 >hsa-mir-3144TAAGGGGACCAAAGAGATATAT AAGGGGACCAAAGAGATATATA
TTAAGGGGACCAAAGAGATATA ATACACTTTAAGGGGACCAAAG
TTTAAGGGGACCAAAGAGATAT 120336327 rs68035463 120336384
rs67106263
HG00107 >hsa-mir-3141TCACCCGGTGAGGGCGGGTGGA CCGGTGAGGGCGGGTGGAGGAG
CGGTGAGGGCGGGTGGAGGAGG CACCCGGTGAGGGCGGGTGGAG
CCCGGTGAGGGCGGGTGGAGGA 153975576 rs936581

HG00107 >hsa-mir-4326TCTGCTGTTCCTCTGTCTCCCA TGGTCTGCTGTTCCTCTGTCTC
CTGGTCTGCTGTTCCTCTGTCT GCTGTTCCTCTGTCTCCCAGAC
TGCTGTTCCTCTGTCTCCCAGA 61918164 rs6062431

HG00107 >hsa-mir-412 TGGGGTACGGGGATGGATGGTC GGATGGATGGTCGACCAGTTGG
GATGGATGGTCGACCAGTTGGA TCGACCAGTTGGAAAGTAATTG
ACGGGGATGGATGGTCGACCAG 101531854 rs61992671

HG00107 >hsa-mir-412 TGGGGTACGGGGATGGATGGTC GGATGGATGGTCGACCAGTTGG
GATGGATGGTCGACCAGTTGGA GGGTACGGGGATGGATGGTCGA
TCGACCAGTTGGAAAGTAATTG 101531849 . 101531854 rs61992671

HG00107 >hsa-mir-553 TTTAAGACGGTGAGATTTTGTT TATTTTAAGACGGTGAGATTTT

TTATTTTAAGACGGTGAGATTT TTTTATTTTAAGACGGTGAGAT
AATTTTATTTTAAGACGGTGAG 100746814 rs190622705

HG00107 >hsa-mir-553 TTTAAGACGGTGAGATTTTGTT TTTTAAGACGGTGAGATTTTGT
TATTTTAAGACGGTGAGATTTT TTATTTTAAGACGGTGAGATTT
TTTTATTTTAAGACGGTGAGAT 100746814 rs190622705 100746835 .

HG00107 >hsa-mir-553 TTTTAAAACGGTGAGATTTTGT TTTAAAACGGTGAGATTTTGTT
TATTTTAAAACGGTGAGATTTT TTATTTTAAAACGGTGAGATTT
TTTTATTTTAAAACGGTGAGAT 100746835 .

HG00107 >hsa-mir-553 TTTTAAAACGGTGAGATTTTGT TATTTTAAAACGGTGAGATTTT
TTTTATTTTAAAACGGTGAGAT TTTAAAACGGTGAGATTTTGTT
ATTTTATTTTAAAACGGTGAGA 100746848 .

HG00107 >hsa-mir-553 TTTTAAAACGGTGAGATTTTGT TATTTTAAAACGGTGAGATTTT
TTTTATTTTAAAACGGTGAGAT TTTAAAACGGTGAGATTTTGTT
ATTTTATTTTAAAACGGTGAGA 100746835 . 100746848 .

HG00107 >hsa-mir-553 TTTTAAGACGGTGAGATTTTGT TATTTTAAGACGGTGAGATTTT
TTTAAGACGGTGAGATTTTGTT TTTTATTTTAAGACGGTGAGAT
ATTTTATTTTAAGACGGTGAGA 100746814 rs190622705 100746848 .

HG00107 >hsa-mir-553 TTTTAAGACGGTGAGATTTTGT TATTTTAAGACGGTGAGATTTT
TTTTATTTTAAGACGGTGAGAT TTTAAGACGGTGAGATTTTGTT
ATTTTATTTTAAGACGGTGAGA 100746814 rs190622705 100746835 .
100746848 .
HG00107 >hsa-mir-3141ACCCGGTGAGGGCGGGTGGAGG CGGTGAGGGCGGGTGGAGGAGG
CCGGTGAGGGCGGGTGGAGGAG CCCGGTGAGGGCGGGTGGAGGA
CACCCGGTGAGGGCGGGTGGAG 153975576 rs936581

HG00107 >hsa-mir-486-2 ACTGAGCTGCCCCGAGCTGGGC
CTGAGCTGCCCCGAGCTGGGCA CTGTACTGAGCTGCCCCGAGCT
CCTGTACTGAGCTGCCCCGAGC GTACTGAGCTGCCCCGAGCTGG 41518007 .

HG00107 >hsa-mir-378hAGATGGGATGAGCCCTGGCTCT TCAGATGGGATGAGCCCTGGCT
TGTCAGATGGGATGAGCCCTGG GATGGGATGAGCCCTGGCTCTG
CAGATGGGATGAGCCCTGGCTC 154209024 rs702742

HG00107 >hsa-mir-1269b AGCCATGCTACGGGCTTCTCTG
ACTGAGCCATGCTACGGGCTTC AGGTTTCTGGACTGAGCCATGC
TGAGGTTTCTGGACTGAGCCAT TTTCTGGACTGAGCCATGCTAC 12820632
rs12451747
HG00107 >hsa-mir-3125AGCTGTGGAGAGAACTCACGGT AAGCTGTGGAGAGAACTCACGG
TAGAGGAAGCTGTGGAGAGAAC TGGATAGAGGAAGCTGTGGAGA
AGGAAGCTGTGGAGAGAACTCA 12877501 rs78852835

HG00107 >hsa-mir-612 AGGGCTTCTGAGCTCCTTAGCA CAGGGCTTCTGAGCTCCTTAGC
GGGCTTCTGAGCTCCTTAGCAC GCAGGGCTTCTGAGCTCCTTAG
GGCAGGGCTTCTGAGCTCCTTA 65211940 rs550894

HG00107 >hsa-mir-548ap AGTAATTGCAGTCTTTGTCATT
AAGTAATTGCAGTCTTTGTCAT AAAGTAATTGCAGTCTTTGTCA
AAAAGTAATTGCAGTCTTTGTC CAAAAGTAATTGCAGTCTTTGT 86368898
rs4414449
HG00107 >hsa-mir-548ap AGTAATTGCAGTCTTTGTCATT
AAGTAATTGCAGTCTTTGTCAT AAAGTAATTGCAGTCTTTGTCA
AAAAGTAATTGCAGTCTTTGTC CAAAAGTAATTGCAGTCTTTGT 86368898
rs4414449 86368959 rs4577031
HG00107 >hsa-mir-548ap AGTAATTGCGGTCTTTGTCATT
AAGTAATTGCGGTCTTTGTCAT AAAGTAATTGCGGTCTTTGTCA
AAAAGTAATTGCGGTCTTTGTC CAAAAGTAATTGCGGTCTTTGT 86368959
rs4577031
HG00107 >hsa-mir-196a-2 AGTTTCATGTTGTTGGGATTGA
AGGTAGTTTCATGTTGTTGGGA TAGTTTCATGTTGTTGGGATTG
GTAGTTTCATGTTGTTGGGATT GGTAGTTTCATGTTGTTGGGAT 54385599
rs11614913
HG00107 >hsa-mir-744 GGGCTAACAGCAGTCTTACTGA GCTAGGGCTAACAGCAGTCTTA
CTAGGGCTAACAGCAGTCTTAC TAGGGCTAACAGCAGTCTTACT
GGCTAGGGCTAACAGCAGTCTT 11985275 .

HG00107 >hsa-mir-1908GGGGACGGCGATTGGTCCGTAT GCGGGGACGGCGATTGGTCCGT
GGCGGGGACGGCGATTGGTCCG CGGGGACGGCGATTGGTCCGTA
TGCCGCGGCGGGGACGGCGATT 61582708 rs174561

HG00107 >hsa-mir-1237GTGGCAGAGCGCTGTCCCGGGG GGTGGCAGAGCGCTGTCCCGGG
GGGTGGCAGAGCGCTGTCCCGG TGGGTGGCAGAGCGCTGTCCCG
AGGGGTGTGGGTGGCAGAGCGC 64136103 rs199916083

HG00107 >hsa-mir-573 GTGTAACTGATCAGGATCTACT TGTGTAACTGATCAGGATCTAC
GATGTGTAACTGATCAGGATCT TGATGTGTAACTGATCAGGATC
GTGATGTGTAACTGATCAGGAT 24521902 rs76014664

HG00107 >hsa-mir-573 GTGTAACTGATCAGGATCTACT TGTGTAACTGATCAGGATCTAC
GATGTGTAACTGATCAGGATCT TGATGTGTAACTGATCAGGATC
GTGATGTGTAACTGATCAGGAT 24521904 rs78830737

HG00107 >hsa-mir-573 GTGTAACTGATCAGGATCTACT TGTGTAACTGATCAGGATCTAC
GATGTGTAACTGATCAGGATCT TGATGTGTAACTGATCAGGATC
GTGATGTGTAACTGATCAGGAT 24521902 rs76014664 24521904
rs78830737
HG00107 >hsa-mir-3117TAAAGGGCCAGACACTATACGA GGGCCAGACACTATACGAGTCA
GCCAGACACTATACGAGTCATA GGCCAGACACTATACGAGTCAT
CCCTAAAGGGCCAGACACTATA 67094171 rs12402181

HG00107 >hsa-mir-3144TAAGGGGACCAAAGAGATATAT TTAAGGGGACCAAAGAGATATA TTTAAGGGGACCAAAGAGATAT TACACTTTAAGGGGACCAAAGA AAATACACTTTAAGGGGACCAA 120336327 rs68035463

HG00107 >hsa-mir-3144TAAGGGGACCAAAGAGATATAT TTAAGGGGACCAAAGAGATATA TTTAAGGGGACCAAAGAGATAT TACACTTTAAGGGGACCAAAGA AACTACACTTTAAGGGGACCAA 120336384 rs67106263

HG00107 >hsa-mir-3144TAAGGGGACCAAAGAGATATAT TTAAGGGGACCAAAGAGATATA TTTAAGGGGACCAAAGAGATAT TACACTTTAAGGGGACCAAAGA AAATACACTTTAAGGGGACCAA 120336327 rs68035463 120336384 rs67106263
HG00107 >hsa-mir-629 TACGTTGGGAGAACTTTTACGG TTACGTTGGGAGAACTTTTACG GTTTACGTTGGGAGAACTTTTA TTTACGTTGGGAGAACTTTTAC GGTTTACGTTGGGAGAACTTTT 70371794 .

HG00107 >hsa-mir-629 TACGTTGGGAGAACTTTTATGG TTACGTTGGGAGAACTTTTATG TTTACGTTGGGAGAACTTTTAT TGGGTTTACGTTGGGAGAACTT GTTTACGTTGGGAGAACTTTTA 70371761 rs377691713

HG00107 >hsa-mir-629 TACGTTGGGAGAACTTTTATGG TTACGTTGGGAGAACTTTTATG TTTACGTTGGGAGAACTTTTAT TGGGTTTACGTTGGGAGAACTT GTTTACGTTGGGAGAACTTTTA 70371761 rs377691713 70371794 .

HG00107 >hsa-mir-888 TACTCAAAAAGCTTTCAGTCAC TCTACTCAAAAAGCTTTCAGTC TGCTCTACTCAAAAAGCTTTCA GCTCTACTCAAAAAGCTTTCAG GGCAGTGCTCTACTCAAAAAGC 145076355 rs143634721

HG00107 >hsa-mir-518dTAGAGGGAAGCACTTTCTGTTG TCTAGAGGGAAGCACTTTCTGT TGACCCTCTAGAGGGAAGCACT TGTGACCCTCTAGAGGGAAGCA TGCTGTGACCCTCTAGAGGGAA 54238208 rs74704964

HG00107 >hsa-mir-449cTAGGCAGTGTATTGCTAGCGGC TCAGATAGGCAGTGTATTGCTA TGTCAGATAGGCAGTGTATTGC TGTGTCAGATAGGCAGTGTATT TGGGATGTGTCAGATAGGCAGT 54468166 rs75661995

HG00107 >hsa-mir-564 TCAGCAGGCAACATGGCCGAGA TGTCAGCAGGCAACATGGCCGA GTCAGCAGGCAACATGGCCGAG GTGTCAGCAGGCAACATGGCCG TGCCAGGCACGGTGTCAGCAGG 44903385 rs114636202

HG00107 >hsa-mir-564 TCAGCAGGCAACATGGCCGAGA TGTCAGCAGGCAACATGGCCGA GTCAGCAGGCAACATGGCCGAG TGCCAGGCACGGTGTCAGCAGG GTGTCAGCAGGCAACATGGCCG 44903433 .

HG00107 >hsa-mir-564 TCAGCAGGCAACATGGCCGAGA TGTCAGCAGGCAACATGGCCGA GTCAGCAGGCAACATGGCCGAG TGCCAGGCACGGTGTCAGCAGG

GTGTCAGCAGGCAACATGGCCG 44903385 rs114636202 44903433 .

HG00107 >hsa-mir-222 TCAGTAGCCAGTGTAGATCCTG TGGCTCAGTAGCCAGTGTAGAT
TTGGCTCAGTAGCCAGTGTAGA TCATTGGCTCAGTAGCCAGTGT
TACCCTCATTGGCTCAGTAGCC 45606504 rs191727254

HG00107 >hsa-mir-515-1 TCCAAAAGAAAGCACTTTCTGT
TCTCCAAAAGAAAGCACTTTCT TTCTCCAAAAGAAAGCACTTTC
TCATTCTCCAAAAGAAAGCACT TGCAGTCATTCTCCAAAAGAAA 54182326
rs374576826

HG00107 >hsa-mir-149 TCCGTGTCTTCACTCCCGTGCT TGGCTCCGTGTCTTCACTCCCG
TCTGGCTCCGTGTCTTCACTCC CCGTGTCTTCACTCCCGTGCTT
AGCTCTGGCTCCGTGTCTTCAC 241395500 rs71428439

HG00107 >hsa-mir-149 TCCGTGTCTTCACTCCCGTGCT TGGCTCCGTGTCTTCACTCCCG
TCTGGCTCCGTGTCTTCACTCC CCGTGTCTTCACTCCCGTGCTT
AGCTCTGGCTCCGTGTCTTCAC 241395503 rs2292832

HG00107 >hsa-mir-149 TCCGTGTCTTCACTCCCGTGCT TGGCTCCGTGTCTTCACTCCCG
TCTGGCTCCGTGTCTTCACTCC CCGTGTCTTCACTCCCGTGCTT
AGCTCTGGCTCCGTGTCTTCAC 241395500 rs71428439 241395503
rs2292832

HG00107 >hsa-mir-618 TCCTTCTGAGTGTAATTACGTA TGTCCTTCTGAGTGTAATTACG
TTGTCCTTCTGAGTGTAATTAC TACTTGTCCTTCTGAGTGTAAT
GTCCTTCTGAGTGTAATTACGT 81329536 rs2682818

HG00107 >hsa-mir-3183TCGGAGTCGCTCGGAGCAGTCA TCTCGGAGTCGCTCGGAGCAGT
TCTCTCGGAGTCGCTCGGAGCA TGCCTCTCTCGGAGTCGCTCGG
TGCCCTGCCTCTCTCGGAGTCG 925742 rs72812091

HG00107 >hsa-mir-1200TGAGCCATTCTGAGCCTCAATC TCCTGAGCCATTCTGAGCCTCA
TCTCCTGAGCCATTCTGAGCCT TTCTCCTGAGCCATTCTGAGCC
TGCTACTTCTCCTGAGCCATTC 36958995 rs180826747

HG00107 >hsa-mir-3612TGAGGAGGCATCTTGAGAAATG TGGGGATGAGGAGGCATCTTGA
GAGGCATCTTGAGAAATGGAAG GGAGGCATCTTGAGAAATGGAA
GAGGAGGCATCTTGAGAAATGG 128778703 rs1683709

HG00107 >hsa-mir-3151TGATGGGTGGGGCAATGGGATC TGGGTGGGGCAATGGGATCAGG
TGGGGCAATGGGATCAGGTGCC GGGGTGATGGGTGGGGCAATGG
GGGTGATGGGTGGGGCAATGGG 104166902 rs35605502

HG00107 >hsa-mir-548kTGCAAAAGTACTTGAGGATTTT TGGTGCAAAAGTACTTGAGGAT
TTGGTGCAAAAGTACTTGAGGA GCAAAAGTACTTGAGGATTTTG
GTGCAAAAGTACTTGAGGATTT 70130103 .

HG00107 >hsa-mir-3156-3 TGCAGAAGAAAGATCTGGAAGT

GCAGAAGAAAGATCTGGAAGTG GAAGAAAGATCTGGAAGTGGGA
GAAAGATCTGGAAGTGGGAGAC AGAAGAAAGATCTGGAAGTGGG 14778721
rs2747232
HG00107 >hsa-mir-3152TGCCTCTGTTCTAACACAAGAC TTGCCTCTGTTCTAACACAAGA
TATTGCCTCTGTTCTAACACAA TTATTGCCTCTGTTCTAACACA
TGCAGAGTTATTGCCTCTGTTC 18573360 rs13299349

HG00107 >hsa-mir-1254-2 TGGAAGCTGGAGCCTGCAGTGA
TGAGCCTGGAAGCTGGAGCCTG GAAGCTGGAGCCTGCAGTGAGC
GGAAGCTGGAGCCTGCAGTGAG GCCTGGAAGCTGGAGCCTGCAG 23682383
rs200793185
HG00107 >hsa-mir-378d-2 TGGACTTGGAGTCAGAAAACTT
GACTTGGAGTCAGAAAACTTTC GGACTTGGAGTCAGAAAACTTT
GAACACTGGACTTGGAGTCAGA TACAAGGAGAGAACACTGGACT 94928250
rs73692959
HG00107 >hsa-mir-1273h TGGGAGGTCAAGGCTGTAGTGT
TGAGCCTGGGAGGTCAAGGCTG TTGAGCCTGGGAGGTCAAGGCT
TGCTTGAGCCTGGGAGGTCAAG TTGCTTGAGCCTGGGAGGTCAA 24214486 .

HG00107 >hsa-mir-1227TGGGGCCAGGCGGTGGTGGGCA TGGTGGGCACTGCTGGGGTGGG
AGGCGGTGGTGGGCACTGCTGG GTGGGGCCAGGCGGTGGTGGGC
GGGGCCAGGCGGTGGTGGGCAC 2234093 rs190788838

HG00107 >hsa-mir-412 TGGGGTACGGGGATGGATGGTC TCGACCAGTTGGAAAGTAATTG
TGGTCGACCAGTTGGAAAGTAA TACGGGGATGGATGGTCGACCA
TGGATGGTCGACCAGTTGGAAA 101531854 rs61992671

HG00107 >hsa-mir-412 TGGGGTACGGGGATGGATGGTC TCGACCAGTTGGAAAGTAATTG
TGGTCGACCAGTTGGAAAGTAA TACGGGGATGGATGGTCGACCA
TGGATGGTCGACCAGTTGGAAA 101531849 . 101531854 rs61992671

HG00107 >hsa-mir-642bTTCCCTCTCCAAATGTGTCTTG TTGGGAGGTTCCCTCTCCAAAT
TGGGAGGTTCCCTCTCCAAATG GAGTTGGGAGGTTCCCTCTCCA
GTTGGGAGGTTCCCTCTCCAAA 46178217 rs111664333

HG00107 >hsa-mir-4277TTCTGAGCACAGTACACTGGGC TCGAGGCAGTTCTGAGCACAGT
TGGGTCGAGGCAGTTCTGAGCA GTTCTGAGCACAGTACACTGGG
GCAGTTCTGAGCACAGTACACT 1708902 rs115200817

HG00107 >hsa-mir-4277TTCTGAGCACAGTACACTGGGC TCGAGGCAGTTCTGAGCACAGT
TGGGTCGAGGCAGTTCTGAGCA TTGGGTCGAGGCAGTTCTGAGC
GTTCTGAGCACAGTACACTGGG 1708983 rs12523324

HG00107 >hsa-mir-4277TTCTGAGCACAGTACACTGGGC TCGAGGCAGTTCTGAGCACAGT
TGGGTCGAGGCAGTTCTGAGCA TTGGGTCGAGGCAGTTCTGAGC
GTTCTGAGCACAGTACACTGGG 1708902 rs115200817 1708983
rs12523324

HG00107 >hsa-mir-1178TTGGCTGGCAGAGGAAGGGAAG TGGCTGGCAGAGGAAGGGAAGG
TGGCAGAGGAAGGGAAGGGTTC GCGTTGGCTGGCAGAGGAAGGG
GTTGGCTGGCAGAGGAAGGGAA 120151501 rs74614893

HG00107 >hsa-mir-553 TTTAAAACGGTGAGATTTTGTT TTTTAAAACGGTGAGATTTTGT
ATTTTAAAACGGTGAGATTTTG TATTTTAAAACGGTGAGATTTT
TTATTTTAAAACGGTGAGATTT 100746835 .

HG00107 >hsa-mir-553 TTTAAAACGGTGAGATTTTGTT TTTTAAAACGGTGAGATTTTGT
ATTTTAAAACGGTGAGATTTTG TATTTTAAAACGGTGAGATTTT
TTATTTTAAAACGGTGAGATTT 100746848 .

HG00107 >hsa-mir-553 TTTAAAACGGTGAGATTTTGTT TTTTAAAACGGTGAGATTTTGT
ATTTTAAAACGGTGAGATTTTG TATTTTAAAACGGTGAGATTTT
TTATTTTAAAACGGTGAGATTT 100746835 . 100746848 .

HG00107 >hsa-mir-553 TTTAAGACGGTGAGATTTTGTT TTTTAAGACGGTGAGATTTTGT
ATTTTAAGACGGTGAGATTTTG TATTTTAAGACGGTGAGATTTT
TTATTTTAAGACGGTGAGATTT 100746814 rs190622705

HG00107 >hsa-mir-553 TTTAAGACGGTGAGATTTTGTT TTTTAAGACGGTGAGATTTTGT
ATTTTAAGACGGTGAGATTTTG TATTTTAAGACGGTGAGATTTT
TTATTTTAAGACGGTGAGATTT 100746814 rs190622705 100746835 .

HG00107 >hsa-mir-553 TTTAAGACGGTGAGATTTTGTT TTTTAAGACGGTGAGATTTTGT
ATTTTAAGACGGTGAGATTTTG TATTTTAAGACGGTGAGATTTT
TTATTTTAAGACGGTGAGATTT 100746814 rs190622705 100746848 .

HG00107 >hsa-mir-553 TTTAAGACGGTGAGATTTTGTT TTTTAAGACGGTGAGATTTTGT
ATTTTAAGACGGTGAGATTTTG TATTTTAAGACGGTGAGATTTT
TTATTTTAAGACGGTGAGATTT 100746814 rs190622705 100746835 .
100746848 .

HG00108 >hsa-mir-577 AGTGAAGAGTAGATAAAATATT GTAGATAAAATATTGGTACCTG
GAAGAGTAGATAAAATATTGGT AAGAGTAGATAAAATATTGGTA
AGTAGATAAAATATTGGTACCT 115577997 rs34115976

HG00108 >hsa-mir-1908CCGCGGCGGGGACGGCGATTGG GCGGCGGGGACGGCGATTGGTC
CGCGGCGGGGACGGCGATTGGT CGGGGACGGCGATTGGTCCGTA
GGGGACGGCGATTGGTCCGTAT 61582708 rs174561

HG00108 >hsa-mir-1273h CCTGGGAGGTCAAGGCTGTAGT
TGGGAGGTCAAGGCTGTAGTGT ATTGCTTGAGCCTGGGAGGTCA
GCCTGGGAGGTCAAGGCTGTAG TTGAGCCTGGGAGGTCAAGGCT 24214486 .

HG00108 >hsa-mir-608 GGCCAAGGTGGGCCAGGGGTGG AAGGTGGGCCAGGGGTGGTGTT
GGGGTGGTGTTGGGACAGCTGC TGGTGTTGGGACAGCTGCGTTT
GGTGGGCCAGGGGTGGTGTTGG 102734778 rs4919510

HG00108 >hsa-mir-3620GGTGGGGGCCAGCAGGGAGTGG TGAGGTGGGGGCCAGCAGGGAG GTGAGGTGGGGGCCAGCAGGGA GAGGTGGGGGCCAGCAGGGAGT AGGTGGGGGCCAGCAGGGAGTG 228284991 rs2070960

HG00108 >hsa-mir-412 TGGGGTACGGGGATGGATGGTC GGATGGATGGTCGACCAGTTGG GATGGATGGTCGACCAGTTGGA TCGACCAGTTGGAAAGTAATTG ACGGGGATGGATGGTCGACCAG 101531854 rs61992671

HG00108 >hsa-mir-412 TGGGGTACGGGGATGGATGGTC GGATGGATGGTCGACCAGTTGG GGGTACGGGGATGGATGGTCGA GATGGATGGTCGACCAGTTGGA TCGACCAGTTGGAAAGTAATTG 101531849 . 101531854 rs61992671

HG00108 >hsa-mir-553 TTTTAAAACGGTGAGATTTTGT TTTAAAACGGTGAGATTTTGTT TTATTTTAAAACGGTGAGATTT TATTTTAAAACGGTGAGATTTT TTTTATTTTAAAACGGTGAGAT 100746835 .

HG00108 >hsa-mir-553 TTTTAAAACGGTGAGATTTTGT TATTTTAAAACGGTGAGATTTT TTTTATTTTAAAACGGTGAGAT TTTAAAACGGTGAGATTTTGTT ATTTTATTTTAAAACGGTGAGA 100746848 .

HG00108 >hsa-mir-553 TTTTAAAACGGTGAGATTTTGT TATTTTAAAACGGTGAGATTTT TTTTATTTTAAAACGGTGAGAT TTTAAAACGGTGAGATTTTGTT ATTTTATTTTAAAACGGTGAGA 100746835 . 100746848 .

HG00108 >hsa-mir-1307ACCGGACCTCGACCGGCTCGTC CGGACCTCGACCGGCTCGTCTG CCGGACCTCGACCGGCTCGTCT ATCTCGACCGGACCTCGACCGG AATCTCGACCGGACCTCGACCG 105154089 rs7911488

HG00108 >hsa-mir-3175AGAGAACGCAGTGACGTCTGGC GAGAGAACGCAGTGACGTCTGG GGAGAGAACGCAGTGACGTCTG GGGAGAGAACGCAGTGACGTCT GGGGAGAGAACGCAGTGACGTC 93447631 rs1439619

HG00108 >hsa-mir-378hAGATGGGATGAGCCCTGGCTCT TCAGATGGGATGAGCCCTGGCT TGTCAGATGGGATGAGCCCTGG GATGGGATGAGCCCTGGCTCTG TGGTGTCAGATGGGATGAGCCC 154209024 rs702742

HG00108 >hsa-mir-1269b AGCCATGCTACGGGCTTCTCTG ACTGAGCCATGCTACGGGCTTC AGGTTTCTGGACTGAGCCATGC TGAGGTTTCTGGACTGAGCCAT TTTCTGGACTGAGCCATGCTAC 12820632 rs12451747
HG00108 >hsa-mir-3135b AGCGAGTGCAGTGGTGCAGTCA AGGCTGGAGCGAGTGCAGTGGT CTGGAGCGAGTGCAGTGGTGCA CAGGCTGGAGCGAGTGCAGTGG TGGAGCGAGTGCAGTGGTGCAG 32717702 rs4285314
HG00108 >hsa-mir-548ap AGTAATTGCAGTCTTTGTCATT AAGTAATTGCAGTCTTTGTCAT AAAGTAATTGCAGTCTTTGTCA

AAAAGTAATTGCAGTCTTTGTC CAAAAGTAATTGCAGTCTTTGT 86368898
rs4414449
HG00108 >hsa-mir-548ap AGTAATTGCAGTCTTTGTCATT
AAGTAATTGCAGTCTTTGTCAT AAAGTAATTGCAGTCTTTGTCA
AAAAGTAATTGCAGTCTTTGTC CAAAAGTAATTGCAGTCTTTGT 86368898
rs4414449 86368959 rs4577031
HG00108 >hsa-mir-548ap AGTAATTGCGGTCTTTGTCATT
AAGTAATTGCGGTCTTTGTCAT AAAGTAATTGCGGTCTTTGTCA
AAAAGTAATTGCGGTCTTTGTC CAAAAGTAATTGCGGTCTTTGT 86368959
rs4577031
HG00108 >hsa-mir-2278AGTGTGTGTTGCCTGGGGACTG AGCAGTGTGTGTTGCCTGGGGA
AGAGCAGTGTGTGTTGCCTGGG AGGTGTTGGAGAGCAGTGTGTG
ATGCTGCAGGTGTTGGAGAGCA 97572244 rs356125

HG00108 >hsa-mir-196a-2 AGTTTCATGTTGTTGGGATTGA
TAGTTTCATGTTGTTGGGATTG AGGTAGTTTCATGTTGTTGGGA
GTAGTTTCATGTTGTTGGGATT GGTAGTTTCATGTTGTTGGGAT 54385599
rs11614913
HG00108 >hsa-mir-3622b ATGGGAGGTCAGGTGAGCTCAG
CATGGGAGGTCAGGTGAGCTCA GCATGGGAGGTCAGGTGAGCTC
AGGCATGGGAGGTCAGGTGAGC GGCATGGGAGGTCAGGTGAGCT 27559214
rs66683138
HG00108 >hsa-mir-500bCTTGCTACCTGGGTGAGAGTGC ATCCTTGCTACCTGGGTGAGAG
AATCCTTGCTACCTGGGTGAGA CCTTGCTACCTGGGTGAGAGTG
TCCTTGCTACCTGGGTGAGAGT 49775351 rs151318590

HG00108 >hsa-mir-650 GAGGCAGCGCTCTCAGGACGTC GGAGGCAGCGCTCTCAGGACGT
TCAGGAGGCAGCGCTCTCAGGA TCTCAGGAGGCAGCGCTCTCAG
GTCTCAGGAGGCAGCGCTCTCA 23165340 rs5996397

HG00108 >hsa-mir-612 GGGCTTCTGAGCTCCTTAGCAC AGGGCTTCTGAGCTCCTTAGCA
CAGGGCTTCTGAGCTCCTTAGC GCAGGGCTTCTGAGCTCCTTAG
GGCAGGGCTTCTGAGCTCCTTA 65211979 rs12803915

HG00108 >hsa-mir-1908GGGGACGGCGATTGGTCCGTAT GCGGGGACGGCGATTGGTCCGT
TGCCGCGGCGGGGACGGCGATT GGCGGGGACGGCGATTGGTCCG
TGCCGCGGCGGGGACGGCGATT 61582708 rs174561

HG00108 >hsa-mir-548h-3 GTAATCGTGGTTTTTGTCATTG
TGCAAAAGTAATCGTGGTTTTT TGGTGCAAAAGTAATCGTGGTT
TTGGTGCAAAAGTAATCGTGGT AGTAATCGTGGTTTTTGTCATT 13446924
rs9913045
HG00108 >hsa-mir-573 GTGTAACTGATCAGGATCTACT TGTGTAACTGATCAGGATCTAC
GATGTGTAACTGATCAGGATCT TGATGTGTAACTGATCAGGATC
GTGATGTGTAACTGATCAGGAT 24521902 rs76014664

HG00108 >hsa-mir-573 GTGTAACTGATCAGGATCTACT TGTGTAACTGATCAGGATCTAC

GATGTGTAACTGATCAGGATCT TGATGTGTAACTGATCAGGATC
GTGATGTGTAACTGATCAGGAT 24521904 rs78830737

HG00108 >hsa-mir-573 GTGTAACTGATCAGGATCTACT TGTGTAACTGATCAGGATCTAC
GATGTGTAACTGATCAGGATCT TGATGTGTAACTGATCAGGATC
GTGATGTGTAACTGATCAGGAT 24521902 rs76014664 24521904
rs78830737
HG00108 >hsa-mir-3117TAAAGGGCCAGACACTATACGA GCCAGACACTATACGAGTCATA
GGGCCAGACACTATACGAGTCA GGCCAGACACTATACGAGTCAT
CCCTAAAGGGCCAGACACTATA 67094171 rs12402181

HG00108 >hsa-mir-559 TAAATATGCACCAAAATTACTT ATATGCACCAAAATTACTTCTG
AATATGCACCAAAATTACTTCT AAATATGCACCAAAATTACTTC
TAAAGTAAATATGCACCAAAAT 47604856 rs114803590

HG00108 >hsa-mir-576 TAATTTCTCCACGTCTTTGGTA TCTAATTTCTCCACGTCTTTGG
TTCTAATTTCTCCACGTCTTTG CTAATTTCTCCACGTCTTTGGT
GATTCTAATTTCTCCACGTCTT 110409933 rs77639117

HG00108 >hsa-mir-629 TACGTTGGGAGAACTTTTATGG TTACGTTGGGAGAACTTTTATG
TTTACGTTGGGAGAACTTTTAT TGGGTTTACGTTGGGAGAACTT
GTTTACGTTGGGAGAACTTTTA 70371761 rs377691713

HG00108 >hsa-mir-936 TAGAGGGAGGAATCGCAGAAAT TGGGACAGTAGAGGGAGGAATC
TCAAGGCCACTGGGACAGTAGA GGAGGAATCGCAGAAATCACTC
GGGAGGAATCGCAGAAATCACT 105807858 rs145823228

HG00108 >hsa-mir-449cTAGGCAGTGTATTGCTAGCGGC TCAGATAGGCAGTGTATTGCTA
TGTCAGATAGGCAGTGTATTGC TGTGTCAGATAGGCAGTGTATT
TGGGATGTGTCAGATAGGCAGT 54468166 rs75661995

HG00108 >hsa-mir-590 TATTCATAAAAGTGCAGTATGG TTATTCATAAAAGTGCAGTATG
TTTATTCATAAAAGTGCAGTAT TGAGTTTATTCATAAAAGTGCA
GTTTATTCATAAAAGTGCAGTA 73605546 rs189727189

HG00108 >hsa-mir-222 TCAGTAGCCAGTGTAGATCCTG TGGCTCAGTAGCCAGTGTAGAT
TTGGCTCAGTAGCCAGTGTAGA TCATTGGCTCAGTAGCCAGTGT
TACCCTCATTGGCTCAGTAGCC 45606504 rs191727254

HG00108 >hsa-mir-515-1 TCCAAAAGAAAGCACTTTCTGT
TCTCCAAAAGAAAGCACTTTCT TTCTCCAAAAGAAAGCACTTTC
TCATTCTCCAAAAGAAAGCACT TGCAGTCATTCTCCAAAAGAAA 54182326
rs374576826
HG00108 >hsa-mir-663aTCCCAGGCGGGGCGCCGCGGGA TCCGGCGTCCCAGGCGGGGCGC
TTCCGGCGTCCCAGGCGGGGCG GCGCCGCGGGACCTCCCTCGTG
GGCGCCGCGGGACCTCCCTCGT 26188880 .

HG00108 >hsa-mir-596 TCCGAAGCCTGCCCGGCCCCTC GCCTGCCCGGCCCCTCGGGAAC TCTCCGAAGCCTGCCCGGCCCC CTGCCCGGCCCCTCGGGAACCT CCTGCCCGGCCCCTCGGGAACC 1765425 rs61388742

HG00108 >hsa-mir-149 TCCGTGTCTTCACTCCCGTGCT TGGCTCCGTGTCTTCACTCCCG TCTGGCTCCGTGTCTTCACTCC CCGTGTCTTCACTCCCGTGCTT CTCCGTGTCTTCACTCCCGTGC 241395503 rs2292832

HG00108 >hsa-mir-618 TCCTTCTGAGTGTAATTACGTA TGTCCTTCTGAGTGTAATTACG TTGTCCTTCTGAGTGTAATTAC TACTTGTCCTTCTGAGTGTAAT GTCCTTCTGAGTGTAATTACGT 81329536 rs2682818

HG00108 >hsa-mir-412 TCGACCAGTTGGAAAGTAATTG TGGTCGACCAGTTGGAAAGTAA TGGGGTACGGGGATGGATGGTC TGGATGGTCGACCAGTTGGAAA TACGGGGATGGATGGTCGACCA 101531854 rs61992671

HG00108 >hsa-mir-412 TCGACCAGTTGGAAAGTAATTG TGGTCGACCAGTTGGAAAGTAA TGGGGTACGGGGATGGATGGTC TGGATGGTCGACCAGTTGGAAA TACGGGGATGGATGGTCGACCA 101531849 . 101531854 rs61992671

HG00108 >hsa-mir-3183TCGGAGTCGCTCGGAGCAGTCA TCTCGGAGTCGCTCGGAGCAGT TCTCTCGGAGTCGCTCGGAGCA TGCCTCTCTCGGAGTCGCTCGG TGCCCTGCCTCTCTCGGAGTCG 925742 rs72812091

HG00108 >hsa-mir-520fTCTCAGGCTGTGACCCTCTAAA TCAGGCTGTGACCCTCTAAAGG TAAAGGGAAGCGCTTTCTGTGG TCTAAAGGGAAGCGCTTTCTGT TGTGACCCTCTAAAGGGAAGCG 54185492 rs75598818

HG00108 >hsa-mir-4305TCTGGGTTCTTAGAGGCCTAAT TTCTGGGTTCTTAGAGGCCTAA GTTCTGGGTTCTTAGAGGCCTA TCCAGTTCTGGGTTCTTAGAGG CAGTTCTGGGTTCTTAGAGGCC 40238175 rs67976778

HG00108 >hsa-mir-27a TGAGGAGCAGGGCTTAGCTGCT TTAGCTGCTTGTGAGCAGGGTC GAGGAGCAGGGCTTAGCTGCTT GGAGCAGGGCTTAGCTGCTTGT GAGCAGGGCTTAGCTGCTTGTG 13947292 rs895819

HG00108 >hsa-mir-3612TGAGGAGGCATCTTGAGAAATG TGGGGATGAGGAGGCATCTTGA GAGGCATCTTGAGAAATGGAAG GGAGGCATCTTGAGAAATGGAA GAGGAGGCATCTTGAGAAATGG 128778703 rs1683709

HG00108 >hsa-mir-3151TGATGGGTGGGGCAATGGGATC TGGGTGGGGCAATGGGATCAGG TGGGGCAATGGGATCAGGTGCC GGGGTGATGGGTGGGGCAATGG GGGTGATGGGTGGGGCAATGGG 104166902 rs35605502

HG00108 >hsa-mir-3622a TGCACAGGCACAGGAGCTCAGG TAGAGGGTGCACAGGCACAGGA GCACAGGAGCTCAGGTGAGGCA GGCACAGGAGCTCAGGTGAGGC GAGGGTGCACAGGCACAGGAGC 27559214

rs66683138
HG00108 >hsa-mir-3156-3 TGCAGAAGAAAGATCTGGAAGT
GCAGAAGAAAGATCTGGAAGTG GAAAGATCTGGAAGTGGGAGAC
GAAGAAAGATCTGGAAGTGGGA AGAAGAAAGATCTGGAAGTGGG 14778721
rs2747232
HG00108 >hsa-mir-4327TGCAGGGGGGACTGGGAAGAGA TTGCAGGGGGGACTGGGAAGAG
GGGGGACTGGGAAGAGACCATG GGGGGGACTGGGAAGAGACCAT
TAGGCTTGCAGGGGGGACTGGG 31747678 rs115477019

HG00108 >hsa-mir-3152TGCCTCTGTTCTAACACAAGAC TTGCCTCTGTTCTAACACAAGA
TATTGCCTCTGTTCTAACACAA TTATTGCCTCTGTTCTAACACA
TGCAGAGTTATTGCCTCTGTTC 18573360 rs13299349

HG00108 >hsa-mir-1254-2 TGGAAGCTGGAGCCTGCAGTGA
TGAGCCTGGAAGCTGGAGCCTG GAAGCTGGAGCCTGCAGTGAGC
GGAAGCTGGAGCCTGCAGTGAG GCCTGGAAGCTGGAGCCTGCAG 23682383
rs200793185
HG00108 >hsa-mir-608 TGGGACAGCTGCGTTTAAAAAG TTGGGACAGCTGCGTTTAAAAA
GGACAGCTGCGTTTAAAAAGGC TGTTGGGACAGCTGCGTTTAAA
GGGACAGCTGCGTTTAAAAAGG 102734778 rs4919510

HG00108 >hsa-mir-1273h TGGGAGGTCAAGGCTGTAGTGT
TGAGCCTGGGAGGTCAAGGCTG TTGAGCCTGGGAGGTCAAGGCT
TGCTTGAGCCTGGGAGGTCAAG TTGCTTGAGCCTGGGAGGTCAA 24214486 .

HG00108 >hsa-mir-1227TGGGGCCAGGCGGTGGTGGGCA TGGTGGGCACTGCTGGGGTGGG
AGGCGGTGGTGGGCACTGCTGG GTGGGGCCAGGCGGTGGTGGGC
GGGGCCAGGCGGTGGTGGGCAC 2234093 rs190788838

HG00108 >hsa-mir-3620TGGGGGCCAGCAGGGAGTGGGT TGAGGTGGGGGCCAGCAGGGAG
GGGGGCCAGCAGGGAGTGGGTT GTGGGGGCCAGCAGGGAGTGGG
GTGAGGTGGGGGCCAGCAGGGA 228284991 rs2070960

HG00108 >hsa-mir-642bTTCCCTCTCCAAATGTGTCTTG TTGGGAGGTTCCCTCTCCAAAT
TGGGAGGTTCCCTCTCCAAATG GAGTTGGGAGGTTCCCTCTCCA
GTTGGGAGGTTCCCTCTCCAAA 46178217 rs111664333

HG00108 >hsa-mir-4277TTCTGAGCACAGTACACTGGGC TCGAGGCAGTTCTGAGCACAGT
TGGGTCGAGGCAGTTCTGAGCA GTTCTGAGCACAGTACACTGGG
GCAGTTCTGAGCACAGTACACT 1708902 rs115200817

HG00108 >hsa-mir-4277TTCTGAGCACAGTACACTGGGC TCGAGGCAGTTCTGAGCACAGT
TGGGTCGAGGCAGTTCTGAGCA TTGGGTCGAGGCAGTTCTGAGC
GTTCTGAGCACAGTACACTGGG 1708983 rs12523324

HG00108 >hsa-mir-4277TTCTGAGCACAGTACACTGGGC TCGAGGCAGTTCTGAGCACAGT
TGGGTCGAGGCAGTTCTGAGCA TTGGGTCGAGGCAGTTCTGAGC

GTTCTGAGCACAGTACACTGGG 1708902 rs115200817 1708983
rs12523324
HG00108 >hsa-mir-553 TTTAAAACGGTGAGATTTTGTT TTTTAAAACGGTGAGATTTTGT
ATTTTAAAACGGTGAGATTTTG TATTTTAAAACGGTGAGATTTT
TTATTTTAAAACGGTGAGATTT 100746835 .

HG00108 >hsa-mir-553 TTTAAAACGGTGAGATTTTGTT TTTTAAAACGGTGAGATTTTGT
ATTTTAAAACGGTGAGATTTTG TATTTTAAAACGGTGAGATTTT
TTATTTTAAAACGGTGAGATTT 100746848 .

HG00108 >hsa-mir-553 TTTAAAACGGTGAGATTTTGTT TTTTAAAACGGTGAGATTTTGT
ATTTTAAAACGGTGAGATTTTG TATTTTAAAACGGTGAGATTTT
TTATTTTAAAACGGTGAGATTT 100746835 . 100746848 .

HG00108 >hsa-mir-3671AATAAGGACTAGTCTGCAGTGA TTTATTTCTATCAAATAAGGAC
TTTTATTTCTATCAAATAAGGA GGACTAGTCTGCAGTGATAT
AAATAAGGACTAGTCTGCAGTG 65523519 rs521188

HG00108 >hsa-mir-149 AGGGAGGGAGGGACGGGGGCTG GGGCTGTGCTGGGGCAGCCGGA
GAGGGAGGGAGGGACGGGGGCT GGGACGGGGGCTGTGCTGGGGC
GGGAGGGAGGGACGGGGGCTGT 241395503 rs2292832

HG00108 >hsa-mir-658 AGGTCGGTTGGTCGGTCGGGAA GTCGGTTGGTCGGTCGGGAACG
TAGGTCGGTTGGTCGGTCGGGA . .

HG00108 >hsa-mir-520fCAAGTGCTTCCTTTTAGAGGGT GCAAGTGCTTCCTTTTAGAGGG
GTGCTTCCTTTTAGAGGGTTAC GCTTCCTTTTAGAGGGTTACCA
AAGCAAGTGCTTCCTTTTAGAG 54185492 rs75598818

HG00108 >hsa-mir-604 CACAGGCTGCGGAATTCAGGAC ACACAGGCTGCGGAATTCAGGA
ACAGGCTGCGGAATTCAGGACA GGCTGCGGAATTCAGGACAGTG
CTGCGGAATTCAGGACAGTGCA 29833998 rs2368393

HG00108 >hsa-mir-604 CACAGGCTGCGGAATTCAGGAC ACACAGGCTGCGGAATTCAGGA
ACAGGCTGCGGAATTCAGGACA GGCTGCGGAATTCAGGACAGTG
CTGCGGAATTCAGGACAGTGCA 29833998 rs2368393 29834003
rs2368392
HG00108 >hsa-mir-3622a CACCTGACCTCCCATGCCTGTG
CATGCCTGTGCACCCTCTATT CCTGACCTCCCATGCCTGTGCA
ATGCCTGTGCACCCTCTATT ACCTGACCTCCCATGCCTGTGC 27559214
rs66683138
HG00108 >hsa-mir-412 CGTCCGTATCCGCTGCAG CCGTCCGTATCCGCTGCAG
TCACCTGGTCCACTGGCCGTCC ACCTGGTCCACTGGCCGTCCGT
CACCTGGTCCACTGGCCGTCCG 101531854 rs61992671

HG00108 >hsa-mir-412 CGTCCGTATCCGCTGCAG CCGTCCGTATCCGCTGCAG
TCACCTGGTTCACTGGCCGTCC ACCTGGTTCACTGGCCGTCCGT

CACCTGGTTCACTGGCCGTCCG 101531849 . 101531854 rs61992671

HG00108 >hsa-mir-3180-4 CTCCGGATGCCAGTCCCTCATC
GGAGGGTGAAGCCTCCGGATGC AGCGGAGGGTGAAGCCTCCGGA
CTGGCCTGGTCGCGCTGTGGCT GAGCGGAGGGTGAAGCCTCCGG 15248720
rs75000738
HG00108 >hsa-mir-4268CTCTCAGGATGTGATGTCACCT CCTCTCAGGATGTGATGTCACC
GCTCCTCCTCTCAGGATGTGAT TCCTCTCAGGATGTGATGTCAC
CTCCTCCTCTCAGGATGTGATG 220771223 rs4674470

HG00108 >hsa-mir-3151CTGATCCCACACCCCACCTGTC TGATCCCACACCCCACCTGTCA
GATCCCACACCCCACCTGTCAC GGGCATCCCACCTGATCCCACA
TCCCACCTGATCCCACACCCCA 104166902 rs35605502

HG00108 >hsa-mir-3620CTGCATCCCGCACCCAG CCTGCATCCCGCACCCAG
TCCCGCACCCAG ATCCCGCACCCAGCATCCCGCACCCAG 228284991 rs2070960

HG00108 >hsa-mir-412 CTTCACCTGGTTCACTAGCCGT ACCTGGTTCACTAGCCGTCCGT
TGTACTTCACCTGGTTCACTAG CTGGTTCACTAGCCGTCCGTAT
GTACTTCACCTGGTTCACTAGC 101531849 .

HG00108 >hsa-mir-658 TAGGTCGGTTGGTCGGTCGGGA .

HG00108 >hsa-mir-658 GTCCGTTGGTCGGTCGGGAACG G
TCCGTTGGTCGGTCGGGAACGA .

HG00108 >hsa-mir-1343GCCCCTCCTGGGGCCCGCACTC CCCCTCCTGGGGCCCGCACTCT
GGGGCCCGCACTCTCGCTCTGG CCCTCCTGGGGCCCGCACTCTC
TGGGGCCCGCACTCTCGCTCTG 34963416 rs2986407

HG00108 >hsa-mir-3180-4 GGAGGGTGAAGCCTCCGGATGC
GGTGAAGCCTCCGGATGCCAGT GCGGAGGGTGAAGCCTCCGGAT
AGCGGAGGGTGAAGCCTCCGGA GCCTGGTCGCGCTGTGGCGAAG 15248798
rs183853838
HG00108 >hsa-mir-3180-4 GGAGGGTGAAGCCTCCGGATGC
GGTGAAGCCTCCGGATGCCAGT GCGGAGGGTGAAGCCTCCGGAT
AGCGGAGGGTGAAGCCTCCGGA CTGGCCTGGTCGCGCTGTGGCT 15248720
rs75000738 15248798 rs183853838
HG00108 >hsa-mir-1227GGCATTTGACCCCGTGCCACCC AGGCATTTGACCCCGTGCCACC
AGGCATTTGACCCCGTGCCACC TGACCCCGTGCCACCCTTTTCC
ATTTGACCCCGTGCCACCCTTT 2234093 rs190788838

HG00108 >hsa-mir-580 TATTTGAGAATGATGAATCATT TGAATCATTAGGTTCCGGTCAG
ATGAATCATTAGGTTCCGGTCA TTTGAGAATGATGAATCATTAG
GAGAATGATGAATCATTAGGTT 36148057 rs115089112

HG00108 >hsa-mir-3118-1 TGAAAATTCTTCTAGTGTG ATGAAAATTCTTCTAGTGTG

TGCATTATGAAAATTCTTCTAG TTATGAAAATTCTTCTAGTGTG
ATTATGAAAATTCTTCTAGTGT 142667330 rs76132421

HG00108 >hsa-mir-320c-1 AAAAGCTGGGTTGAGAGGGTAG
AGCTGGGTTGAGAGGGTAGGAA CTGGGTTGAGAGGGTAGGAAAA
AGGGTAGGAAAAAAATGATGTA 19263542 .

HG00108 >hsa-mir-202 AAAGAGGTATAGGGCATGGGAA AAGAGGTATAGGGCATGGGAAA
GGGAAAACGGGGCGGTCGGGTC TAAAGAGGTATAGGGCATGGGA 135061112
rs12355840
HG00108 >hsa-mir-576 AAAGATGTGGAAAAATTGGAAT AAGATGTGGAAAAATTGGAATC
TGGAAAAATTGGAATCCTCTTT GATGTGGAAAAATTGGAATCCT 110409933
rs77639117
HG00108 >hsa-mir-520hAAAGTGCTTCCCTTTAGAGTTA rs148716001 74

HG00108 >hsa-mir-944 AAATTATTGTACATCGGATGAG GTACATCGGATGAGCTGTGTCT
GAAATTATTGTACATCGGATGA CATCGGATGAGCTGTGTCTGGG 189547735
rs75715827
HG00108 >hsa-mir-548ap AACAAAAACCACAATTACTTTT
CAAAAACCACAATTACTTTTTA CAATTACTTTTTACTGACCTAA rs4414449

HG00108 >hsa-mir-548ap AACAAAAACCACAATTACTTTT
CAAAAACCACAATTACTTTTTA TTACTTTTTACTGACCTAAAGA rs4577031

HG00108 >hsa-mir-548ap AACAAAAACCACAATTACTTTT
CAAAAACCACAATTACTTTTTA CAATTACTTTTTACTGACCTAA rs4414449
86368959 rs4577031
HG00108 >hsa-mir-1255b-2 AACCACTTTCTTTGCTCATCCG
AAACCACTTTCTTTGCTCATCC CTTTCTTTGCTCATCCGTAAGG rs79639536

HG00108 >hsa-mir-548al AACGGCAGTGACTTTTGTACCA
TGGCAAAAACGGCAGTGACTTT TAAAAGTAATGGCAAAAACGGC
AAAAGTAATGGCAAAAACGGCA 74110353 rs515924

HG00108 >hsa-mir-520fAAGCAAGTGCTTCCTTTTAGAG AGCAAGTGCTTCCTTTTAGAGG
GTGCTTCCTTTTAGAGGGTTAC TGCTTCCTTTTAGAGGGTTACC 54185492
rs75598818
HG00108 >hsa-mir-423 AAGCTCGGTCTGAGGCCCCTCA AGGCCCCTCAGTCTTGCTTCCT
TCTGAGGCCCCTCAGTCTTGCT GTCTGAGGCCCCTCAGTCTTGC 28444183
rs6505162
HG00108 >hsa-mir-3938AATTCCCTTGTAGATAACCCGG ATAACCCGGTGGTCAGGTTGGA
TAGATAACCCGGTGGTCAGGTT CCTTGTAGATAACCCGGTGGTC 55886574
rs59684995
HG00108 >hsa-mir-449cACAGTTGCTAGTTGCACTCCTC AACAGTTGCTAGTTGCACTCCT
GTTGCTAGTTGCACTCCTCTCT GTTGCACTCCTCTCTGTTGCAT 54468166
rs75661995
HG00108 >hsa-mir-3118-1 ACTGCATTATGAAAATTCTTCT

ATTATGAAAATTCTTCTAGTGT GCATTATGAAAATTCTTCTAGT
CTGCATTATGAAAATTCTTCTA 142667330 rs76132421

HG00108 >hsa-mir-642bAGATACATTTGGAGAGGGACCC TTGGAGAGGGACCCTCCCAACT
TTTGGAGAGGGACCCTCCCAAC ATACATTTGGAGAGGGACCCTC 46178217
rs111664333
HG00108 >hsa-mir-3180-4 AGCGGAGGGTGAAGCCTCCGGA
CGGAGGGTGAAGCCTCCGGATG GAGCGGAGGGTGAAGCCTCCGG
GCGGAGGGTGAAGCCTCCGGAT 15248798 rs183853838

HG00108 >hsa-mir-3180-4 AGCGGAGGGTGAAGCCTCCGGA
CGGAGGGTGAAGCCTCCGGATG GAGCGGAGGGTGAAGCCTCCGG
GCGGAGGGTGAAGCCTCCGGAT 15248720 rs75000738 15248798
rs183853838
HG00108 >hsa-mir-30d AGCTTTCAGTCAGATGTTTGCT GGCTAAGCTTTCAGTCAGATGT
GCTAAGCTTTCAGTCAGATGTT TTCAGTCAGATGTTTGCTGCTA 135817150
.
HG00108 >hsa-mir-519a-2 AGGAAAGTGCATCCTTTTAGAG
AGTGCATCCTTTTAGAGGGTTA GGAAAGTGCATCCTTTTAGAGG
GAAAGGAAAGTGCATCCTTTTA 54265670 .

HG00108 >hsa-mir-630 AGTATTCTGTACCAGGGAAGGT ACCTAGTATTCTGTACCAGGGA
CCAGGGAAGGTAGTTCTTAACT CAGGGAAGGTAGTTCTTAACTA 72879653
rs113971639
HG00108 >hsa-mir-3686AGTGATCTGTAAGAGAAAGTAA TCTGTAAGAGAAAGTAAATGAA
GTAAGAGAAAGTAAATGAAAGA ACAGTGATCTGTAAGAGAAAGT 130496365
rs6997249
HG00108 >hsa-mir-642aATTTGGAGAGGGAACCTCCCAA AGACACATTTGGAGAGGGAACC
ACACATTTGGAGAGGGAACCTC CACATTTGGAGAGGGAACCTCC 46178217
rs111664333
HG00108 >hsa-mir-548ac CAAAAACCGGCAATTACTTTTG
GGCAAAAACCGGCAATTACTTT TTACTTTTGCACTAACCTAATA
ACCGGCAATTACTTTTGCACTA 117102649 rs1414273

HG00108 >hsa-mir-4293CACACCAGCCTGACAGGAACAG TCACACCAGCCTGACAGGAACA
CACCAGCCTGACAGGAACAGCC CAGCCTGACAGGAACAGCCTGT 14425204
rs12780876
HG00108 >hsa-mir-604 CACAGGCTGCGGAATTCAGGAC TGACACAGGCTGCGGAATTCAG
GACACAGGCTGCGGAATTCAGG TTTCTGACACAGGCTGCGGAAT 29834003
rs2368392
HG00108 >hsa-mir-412 CACCTGGTCCACTGGCCGTCCG ACCTGGTCCACTGGCCGTCCGT
CTGGCCGTCCGTATCCGCTGCA TCACCTGGTCCACTGGCCGTCC 101531854
rs61992671
HG00108 >hsa-mir-412 CACCTGGTTCACTGGCCGTCCG ACCTGGTTCACTGGCCGTCCGT
CTGGCCGTCCGTATCCGCTGCA TCACCTGGTTCACTGGCCGTCC 101531849
. 101531854 rs61992671
HG00108 >hsa-mir-515-1 CAGAGTGCCTTCTTTTGGAGCA

GAGTGCCTTCTTTTGGAGCATT	TGCCTTCTTTTGGAGCATTACT
GTGCCTTCTTTTGGAGCATTAC	54182326	rs374576826

HG00108	>hsa-mir-4265CAGCTGTGGGCTCAACTCTGGG	ATCTCTGCAGCTGTGGGCTCAA
GATCTCTGCAGCTGTGGGCTCA	CTGCAGCTGTGGGCTCAACTCT	109757963
rs4676066
HG00108	>hsa-mir-1227CATTTGACCCCGTGCCACCCTT	ATTTGACCCCGTGCCACCCTTT
AGGCATTTGACCCCGTGCCACC	GACCCCGTGCCACCCTTTTCCC	2234093
rs190788838
HG00108	>hsa-mir-1908CCACCGGCCGCCGGCTCCGCCC	CCGCCGGCTCCGCCCCGGCCCC
GGCCGCCGGCTCCGCCCCGGCC	CGGCCGCCGGCTCCGCCCCGGC	61582708
rs174561
HG00108	>hsa-mir-3151CCACCTGATCCCACACCCCACC	CACCTGATCCCACACCCCACCT
CCCACCTGATCCCACACCCCAC	TGATCCCACACCCCACCTGTCA	104166902
rs35605502
HG00108	>hsa-mir-1343CCCCTCCTGGGGCCCGCACTCT	CCCTCCTGGGGCCCGCACTCTC
CCTGGGGCCCGCACTCTCGCTC	TGGGGCCCGCACTCTCGCTCTG	34963416
rs2986407
HG00108	>hsa-mir-3622b	CCTCACCTGAGCTCCTGTGCCT
CCTGAGCTCCTGTGCCTGTGCA	CTCCTGTGCCTGTGCACCCTCT
CTGAGCTCCTGTGCCTGTGCAC	27559214	rs66683138

HG00108	>hsa-mir-4268CCTCTCAGGATGTGATGTCACC	CTCCTCTCAGGATGTGATGTCA
CTCCTCCTCTCAGGATGTGATG	rs4674470

HG00108	>hsa-mir-943 CCTGACTGTTGCCGTCCTCCAG	CTGTTGCCGTCCTCCAGCCCCA
CTCCAGCCCCACTCAAAGGCAT	GCCGTCCTCCAGCCCCACTCAA	1988193
rs1077020
HG00108	>hsa-mir-4254CCTGGAGATACTCCACCATCTC	AGATACTCCACCATCTCCCCCA
GGAGATACTCCACCATCTCCCC	rs12731294

HG00108	>hsa-mir-3152CCTGTGTTAGAATAAGGGCAAT	TTAGAATAAGGGCAATAACTCT
AGAATAAGGGCAATAACTCTGC	TGTGTTAGAATAAGGGCAATAA	18573360
rs13299349
HG00108	>hsa-mir-182 CGGTGGTTCTAGACTTGCCAAC	CCGGTGGTTCTAGACTTGCCAA
TCCGGTGGTTCTAGACTTGCCA	GCCAACTATGGGGCGAGGACTC	129410227
rs76481776
HG00108	>hsa-mir-3620CTCACCCTGCATCCCGCACCCA	CCTCACCCTGCATCCCGCACCC
CTCACCCTGCATCCCGCACCCA	rs2070960

HG00108	>hsa-mir-3622a	CTCACCTGACCTCCCATGCCTG
CCTGACCTCCCATGCCTGTGCA	GCTCACCTGACCTCCCATGCCT
CTGACCTCCCATGCCTGTGCAC	27559214	rs66683138

HG00108	>hsa-mir-3117CTCATATAGTGCCAGGTGTTTT	GACTCATATAGTGCCAGGTGTT
TCATATAGTGCCAGGTGTTTTG	ATAAGACTCATATAGTGCCAGG	67094171
rs12402181

HG00108 >hsa-mir-196a-2 CTCGGCAACAAGAAACTGTCTG
CAAGAAACTGTCTGAGTTACAT CAACAAGAAACTGTCTGAGTTA
ACAAGAAACTGTCTGAGTTACA 54385599 rs11614913

HG00108 >hsa-mir-3615CTCTCTCGGCTCCTCGCGGCTC GGCTCCTCGCGGCTCGCGGCGG
CGGCTCCTCGCGGCTCGCGGCG TCGGCTCCTCGCGGCTCGCGGC 72744798
rs745666
HG00108 >hsa-mir-1304CTGTAGCATCGAACCCCTGGGC GAACCCCTGGGCTCAAGTGATT
CTCACTGTAGCATCGAACCCCT CGAACCCCTGGGCTCAAGTGAT 93466866
rs2155248
HG00108 >hsa-mir-2117CTGTTCTCTTTGCCAAGGACAG GCTGTTCTCTTTGCCAAGGACA
TCTCTTTGCCAAGGACAGATCT TGTTCTCTTTGCCAAGGACAGA 41522213
rs7207008
HG00108 >hsa-mir-940 GAAGGCAGGGCCCC-GCTCCCC G CCC-
GCTCCCCGGGCCTGACCC rs35356504

HG00108 >hsa-mir-4274GACCCAGCAGTCCCTCCCCCTG CCCAGCAGTCCCTCCCCCTGCA
TGACCCAGCAGTCCCTCCCCCT TCAGGTGACCCAGCAGTCCCTC 7461769
rs12512664
HG00108 >hsa-mir-1307GACTCGGCGTGGCGTCGGTCGT CGTGGCGTCGGTCGTGGTAGAT
ATCGACTCGGCGTGGCGTCGGT GTGGCGTCGGTCGTGGTAGATA 105154089
rs7911488
HG00108 >hsa-mir-629 GAGGTTCTCCCAACGTAAGCCC AGGTTCTCCCAACGTAAGCCCA
TCTCCCAACGTAAGCCCAGCCC CAGGAGGTTCTCCCAACGTAAG 70371761
rs377691713
HG00108 >hsa-mir-222 GCAGCTACATCTGGCTACTGGG TACTGGGTCTCTGATGGCATCT
GCTACTGGGTCTCTGATGGCAT CTGGCTACTGGGTCTCTGATGG 45606504
rs191727254
HG00108 >hsa-mir-500bGCAGTGCACCCAGGCAAGGATT CACCCAGGCAAGGATTCTGCGA
AGGCAAGGATTCTGCGAGGGGG TGCAGTGCACCCAGGCAAGGAT 49775351
rs151318590
HG00108 >hsa-mir-3180-4 GCGGAGGGTGAAGCCTCCGGAT
CGCTGGCCTGGTCGCGCTGTGG TCGCTGGCCTGGTCGCGCTGTG
AAGCCTCCGGATGCCAGTCCCT 15248720 rs75000738

HG00108 >hsa-mir-30a GGCTTTCAGTCGGATGTTTGCA AGATGGGCTTTCAGTCGGATGT
ATGGGCTTTCAGTCGGATGTTT TTTCAGTCGGATGTTTGCAGCT 72113306
rs190842689
HG00108 >hsa-mir-3176GGGACTGGCCTGGGACTACCGG GCCTGGGACTACCGGGGGTGGC
ACTGGCCTGGGACTACCGGGGG CAGGGACTGGCCTGGGACTACC 593277
rs8054514
HG00108 >hsa-mir-149 GGGAGGGAGGGACGGGGGCTGT GGAGGGACGGGGGCTGTGCTGG
AGGGACGGGGGCTGTGCTGGGG GAGGAGGGAGGGAGGGACGGGG 241395503
rs2292832
HG00108 >hsa-mir-1197GTAGGACACATGGTCTACTTCT ACACATGGTCTACTTCTTCTCA
ACATGGTCTACTTCTTCTCAAT TAGGACACATGGTCTACTTCTT 101491923
rs141611518

HG00108 >hsa-mir-27a GTGTTCACAGTGGCTAAGTTCC TCGTGTTCACAGTGGCTAAGTT AGTGGCTAAGTTCCGCCCCCCA CACAGTGGCTAAGTTCCGCCCC 13947292 rs895819
HG00108 >hsa-mir-3936TAAGGGGTGTATGGCAGATGCA TTCTGGTAAGGGGTGTATGGCA CACCCGACAGATGCACTTGGCA TGTATGGCAGATGCACCCGACA 131701279 rs367805
HG00108 >hsa-mir-412 TCACCTGGTTCACTAGCCGTCC TCACCTGGTTCACTAGCCGTCC ATGTACTTCACCTGGTTCACTA CTTCACCTGGTTCACTAGCCGT 101531849 .
HG00108 >hsa-mir-3671TCTATCAAATAAGGACTAGTCT AAATAAGGACTAGTCTGCAGTG TTTATTTCTATCAAATAAGGAC CAAATAAGGACTAGTCTGCAGT 65523519 rs521188
HG00108 >hsa-mir-4308TCTTTCCCTGGAGTTTCTTCTT AATCTTTCCCTGGAGTTTCTTC GAAATCTTTCCCTGGAGTTTCT CCTGGAGTTTCTTCTTACCTTT 55344901 rs28477407
HG00108 >hsa-mir-595 TGAAGTGTGCCGTGGTGTGTCT GTGTGTCTGGAGGAAGCGCCTG GCCGTGGTGTGTCTGGAGGAAG rs4909237

HG00108 >hsa-mir-604 TGACACAGGCTGCGGAATTCAG CACAGGCTGCGGAATTCAGGAC GACACAGGCTGCGGAATTCAGG TTTCTGACACAGGCTGCGGAAT 29833998 rs2368393
HG00108 >hsa-mir-604 TGACACAGGCTGCGGAATTCAG GACACAGGCTGCGGAATTCAGG CACAGGCTGCGGAATTCAGGAC TTTCTGACACAGGCTGCGGAAT 29833998 rs2368393 29834003 rs2368392
HG00108 >hsa-mir-1273h TGCTGCAGACTCGACCTCCCAG TGCAGACTCGACCTCCCAGGCT CTGCAGACTCGACCTCCCAGGC AGACTCGACCTCCCAGGCTTAA 24214486 .

HG00108 >hsa-mir-590 TGTAATTTTATGTATAAGCTAG AATCTGTAATTTTATGTATAAG GTATAAGCTAGTCTCTGATTGA TTTTATGTATAAGCTAGTCTCT 73605546 rs189727189
HG00108 >hsa-mir-2053TGTTAATTAAACCTCTATTTAC ACTTTAAGTGTTAATTAAACCT TTTAAGTGTTAATTAAACCTCT TTAAGTGTTAATTAAACCTCTA 113655752 rs10505168
HG00108 >hsa-mir-585 TTGGGCGTATCTGTATGCTAGG TATCTGTATGCTAGGGCTGCCG TGGGCGTATCTGTATGCTAGGG GCGTATCTGTATGCTAGGGCTG 168690612 rs62376934
HG00108 >hsa-mir-1303TTTAGAGACGGGGTCTTGCTCT TAGAGACGGGGTCTTGCTCTGT TTAGAGACGGGGTCTTGCTCTG ACGGGGTCTTGCTCTGTTGCCA 154065383 rs75538180
HG00108 >hsa-mir-580 TTTGAGAATGATGAATCATTAG GATGAATCATTAGGTTCCGGTC AATGATGAATCATTAGGTTCCG AGAATGATGAATCATTAGGTTC 36148057 rs115089112
HG00109 >hsa-mir-492 AACTACAGCCACTACTACAGGA CAACTACAGCCACTACTACAGG ACAGCCACTACTACAGGACCAT ACTACAGCCACTACTACAGGAC GCCACTACTACAGGACCATCGA 95228209 .

HG00109 >hsa-mir-492 AACTCCAGCCACTACTACAGGA CAACTCCAGCCACTACTACAGG CCAGCCACTACTACAGGACCAT GCCACTACTACAGGACCATCGA ACTCCAGCCACTACTACAGGAC 95228179 rs200816308 95228209 .

HG00109 >hsa-mir-3125AGAATGGATAGAGGAAGCTGTG GAGGAAGCTGTGGAGAGAACTC AGAGGAAGCTGTGGAGAGAACT GCTGTGGAGAGAACTCACGGTG GGAAGCTGTGGAGAGAACTCAC 12877501 rs78852835

HG00109 >hsa-mir-577 AGTGAAGAGTAGATAAAATATT GTAGATAAAATATTGGTACCTG GAAGAGTAGATAAAATATTGGT AAGAGTAGATAAAATATTGGTA AGTAGATAAAATATTGGTACCT 115577997 rs34115976

HG00109 >hsa-mir-1908CCGCGGCGGGGACGGCGATTGG GCGGCGGGGACGGCGATTGGTC CGCGGCGGGGACGGCGATTGGT CGGGGACGGCGATTGGTCCGTA GGGGACGGCGATTGGTCCGTAT 61582708 rs174561

HG00109 >hsa-mir-1273h CCTGGGAGGTCAAGGCTGTAGT TGGGAGGTCAAGGCTGTAGTGT ATTGCTTGAGCCTGGGAGGTCA GCCTGGGAGGTCAAGGCTGTAG TTGAGCCTGGGAGGTCAAGGCT 24214486 .

HG00109 >hsa-mir-4309CTGGGGGTTCTGGAGTCTAGGA TGGAGTCTAGGATTCCAGGATC GGAGTCTAGGATTCCAGGATCT GGGGGTTCTGGAGTCTAGGATT GGGTTCTGGAGTCTAGGATTCC 103006047 rs12879262

HG00109 >hsa-mir-548ak GCAAAAGTAACTGCGGTTTTTG TGCAAAAGTAACTGCGGTTTTT CAAAAGTAACTGCGGTTTTTGA GTGCAAAAGTAACTGCGGTTTT rs7070684

HG00109 >hsa-mir-3196GCGGGGCGGCAGGGGCCTCCCC GGCGGGGCGGCAGGGGCCTCCC GGGCGGGGCGGCAGGGGCCTCC TGGGGGCGGGGCGGCAGGGGCC GGGGCGGGGCGGCAGGGGCCTC 61870167 rs744591

HG00109 >hsa-mir-577 GTAGATAAAATATTGGTACCTG GGTGAAGAGTAGATAAAATATT AGTAGATAAAATATTGGTACCT AAGAGTAGATAAAATATTGGTA GTGAAGAGTAGATAAAATATTG 115577921 rs79560193

HG00109 >hsa-mir-577 GTAGATAAAATATTGGTACCTG GGTGAAGAGTAGATAAAATATT AGTAGATAAAATATTGGTACCT AAGAGTAGATAAAATATTGGTA AGAGTAGATAAAATATTGGTAC 115577921 rs79560193 115577997 rs34115976

HG00109 >hsa-mir-892cTGCCCTATTCAGAAAGGTGCCA TCAGAAAGGTGCCAGTCACTTA AGTGCCCTATTCAGAAAGGTGC GCCCTATTCAGAAAGGTGCCAG GTGCCCTATTCAGAAAGGTGCC 145074284 .

HG00109 >hsa-mir-553 TTTTAAAACGGTGAGATTTTGT TATTTTAAAACGGTGAGATTTT TTTTATTTTAAAACGGTGAGAT TTTAAAACGGTGAGATTTTGTT ATTTTATTTTAAAACGGTGAGA 100746848 .

HG00109 >hsa-mir-553 TTTTAAAACGGTGAGATTTTGT TTTTATTTTAAAACGGTGAGAT TTTAAAACGGTGAGATTTTGTT TATTTTAAAACGGTGAGATTTT ATTTTATTTTAAAACGGTGAGA 100746848 . 100746855 rs112891767

HG00109 >hsa-mir-553 TTTTATTTTAAAACGGTGAGAT TTTAAAACGGTGAGATTTTGTT TTTTAAAACGGTGAGATTTTGT AATTTTATTTTAAAACGGTGAG TTATTTTAAAACGGTGAGATTT 100746855 rs112891767

HG00109 >hsa-mir-492 AACTACAGCCACTACTACAGGA AGCCACTACTACAGGACCATCG ACAGCCACTACTACAGGACCAT ACTACAGCCACTACTACAGGAC AACTACAGCCACTACTACAGGA 95228209 .

HG00109 >hsa-mir-492 AACTCCAGCCACTACTACAGGA AGCCACTACTACAGGACCATCG ACTCCAGCCACTACTACAGGAC AACTCCAGCCACTACTACAGGA CAACTCCAGCCACTACTACAGG 95228179 rs200816308 95228209 .

HG00109 >hsa-mir-1307ACCGGACCTCGACCGGCTCGTC CGGACCTCGACCGGCTCGTCTG CCGGACCTCGACCGGCTCGTCT ATCTCGACCGGACCTCGACCGG AATCTCGACCGGACCTCGACCG 105154089 rs7911488

HG00109 >hsa-mir-486-2 ACTGAGCTGCCCCGAGCTGGGC CTGAGCTGCCCCGAGCTGGGCA CTGTACTGAGCTGCCCCGAGCT CCTGTACTGAGCTGCCCCGAGC GTACTGAGCTGCCCCGAGCTGG 41518007 .

HG00109 >hsa-mir-1269b AGCCATGCTACGGGCTTCTCTG ACTGAGCCATGCTACGGGCTTC AGGTTTCTGGACTGAGCCATGC TGAGGTTTCTGGACTGAGCCAT TTTCTGGACTGAGCCATGCTAC 12820632 rs12451747

HG00109 >hsa-mir-3125AGCTGTGGAGAGAACTCACGGT AAGCTGTGGAGAGAACTCACGG TAGAGGAAGCTGTGGAGAGAAC TGGATAGAGGAAGCTGTGGAGA AGGAAGCTGTGGAGAGAACTCA 12877501 rs78852835

HG00109 >hsa-mir-612 AGGGCTTCTGAGCTCCTTAGCA GGGCTTCTGAGCTCCTTAGCAC CAGGGCTTCTGAGCTCCTTAGC GCAGGGCTTCTGAGCTCCTTAG GGCAGGGCTTCTGAGCTCCTTA 65211979 rs12803915

HG00109 >hsa-mir-548ap AGTAATTGCAGTCTTTGTCATT AAGTAATTGCAGTCTTTGTCAT AAAGTAATTGCAGTCTTTGTCA AAAAGTAATTGCAGTCTTTGTC CAAAAGTAATTGCAGTCTTTGT 86368898 rs4414449

HG00109 >hsa-mir-548ap AGTAATTGCAGTCTTTGTCATT AAGTAATTGCAGTCTTTGTCAT AAAGTAATTGCAGTCTTTGTCA AAAAGTAATTGCAGTCTTTGTC CAAAAGTAATTGCAGTCTTTGT 86368898 rs4414449 86368959 rs4577031

HG00109 >hsa-mir-548ap AGTAATTGCGGTCTTTGTCATT AAGTAATTGCGGTCTTTGTCAT AAAGTAATTGCGGTCTTTGTCA

AAAAGTAATTGCGGTCTTTGTC CAAAAGTAATTGCGGTCTTTGT 86368959
rs4577031
HG00109 >hsa-mir-196a-2 AGTTTCATGTTGTTGGGATTGA
AGGTAGTTTCATGTTGTTGGGA TAGTTTCATGTTGTTGGGATTG
GTAGTTTCATGTTGTTGGGATT GGTAGTTTCATGTTGTTGGGAT 54385599
rs11614913
HG00109 >hsa-mir-500bATCCTTGCTACCTGGGTGAGAG CTTGCTACCTGGGTGAGAGTGC
AATCCTTGCTACCTGGGTGAGA CCTTGCTACCTGGGTGAGAGTG
TCCTTGCTACCTGGGTGAGAGT 49775351 rs151318590

HG00109 >hsa-mir-650 GAGGCAGCGCTCTCAGGACGTC GGAGGCAGCGCTCTCAGGACGT
TCAGGAGGCAGCGCTCTCAGGA TCTCAGGAGGCAGCGCTCTCAG
GTCTCAGGAGGCAGCGCTCTCA 23165340 rs5996397

HG00109 >hsa-mir-1273d GAGGTTGAGGCTGCAGTGAGCC
TGAGGTTGAGGCTGCAGTGAGC CATGAGGTTGAGGCTGCAGTGA
CCATGAGGTTGAGGCTGCAGTG ATGAGGTTGAGGCTGCAGTGAG 10287824
rs150712502
HG00109 >hsa-mir-1908GGGGACGGCGATTGGTCCGTAT GCGGGGACGGCGATTGGTCCGT
GGCGGGGACGGCGATTGGTCCG CGGGGACGGCGATTGGTCCGTA
TGCCGCGGCGGGGACGGCGATT 61582708 rs174561

HG00109 >hsa-mir-548h-3 GTAATCGTGGTTTTTGTCATTG
TGCAAAAGTAATCGTGGTTTTT AGTAATCGTGGTTTTTGTCATT
AAGTAATCGTGGTTTTTGTCAT TGGTGCAAAAGTAATCGTGGTT 13446924
rs9913045
HG00109 >hsa-mir-573 GTGTAACTGATCAGGATCTACT TGTGTAACTGATCAGGATCTAC
GATGTGTAACTGATCAGGATCT TGATGTGTAACTGATCAGGATC
GTGATGTGTAACTGATCAGGAT 24521902 rs76014664

HG00109 >hsa-mir-573 GTGTAACTGATCAGGATCTACT TGTGTAACTGATCAGGATCTAC
GATGTGTAACTGATCAGGATCT TGATGTGTAACTGATCAGGATC
GTGATGTGTAACTGATCAGGAT 24521904 rs78830737

HG00109 >hsa-mir-573 GTGTAACTGATCAGGATCTACT TGTGTAACTGATCAGGATCTAC
GATGTGTAACTGATCAGGATCT TGATGTGTAACTGATCAGGATC
GTGATGTGTAACTGATCAGGAT 24521902 rs76014664 24521904
rs78830737
HG00109 >hsa-mir-3117TAAAGGGCCAGACACTATACGA GGGCCAGACACTATACGAGTCA
GCCAGACACTATACGAGTCATA GGCCAGACACTATACGAGTCAT
CCCTAAAGGGCCAGACACTATA 67094171 rs12402181

HG00109 >hsa-mir-629 TACGTTGGGAGAACTTTTACGG TTACGTTGGGAGAACTTTTACG
GTTTACGTTGGGAGAACTTTTA TTTACGTTGGGAGAACTTTTAC
GGTTTACGTTGGGAGAACTTTT 70371794 .

HG00109 >hsa-mir-629 TACGTTGGGAGAACTTTTATGG TTACGTTGGGAGAACTTTTATG

TTTACGTTGGGAGAACTTTTAT TGGGTTTACGTTGGGAGAACTT
GTTTACGTTGGGAGAACTTTTA 70371761 rs377691713

HG00109 >hsa-mir-629 TACGTTGGGAGAACTTTTATGG TTACGTTGGGAGAACTTTTATG
TTTACGTTGGGAGAACTTTTAT TGGGTTTACGTTGGGAGAACTT
GTTTACGTTGGGAGAACTTTTA 70371761 rs377691713 70371794 .

HG00109 >hsa-mir-888 TACTCAAAAAGCCGTCAGTCAC TCTACTCAAAAAGCCGTCAGTC
TGCTCTACTCAAAAAGCCGTCA GCTCTACTCAAAAAGCCGTCAG
AGTGCTCTACTCAAAAAGCCGT 145076356 rs112850228

HG00109 >hsa-mir-888 TACTCAAAAAGCCTTCAGTCAC TCTACTCAAAAAGCCTTCAGTC
TGCTCTACTCAAAAAGCCTTCA GCTCTACTCAAAAAGCCTTCAG
GTGCTCTACTCAAAAAGCCTTC 145076355 rs143634721 145076356
rs112850228
HG00109 >hsa-mir-888 TACTCAAAAAGCTTTCAGTCAC TCTACTCAAAAAGCTTTCAGTC
TGCTCTACTCAAAAAGCTTTCA GCTCTACTCAAAAAGCTTTCAG
GGCAGTGCTCTACTCAAAAAGC 145076355 rs143634721

HG00109 >hsa-mir-3199-1 TAGGAGAAAGTTTCTGGAAGTT
TTAGGAGAAAGTTTCTGGAAGT TGCCTTAGGAGAAAGTTTCTGG
TCCAGGGACTGCCTTAGGAGAA TGACTCCAGGGACTGCCTTAGG 28316513
rs118160653
HG00109 >hsa-mir-449cTAGGCAGTGTATTGCTAGCGGC TCAGATAGGCAGTGTATTGCTA
TGTCAGATAGGCAGTGTATTGC TGTGTCAGATAGGCAGTGTATT
TGGGATGTGTCAGATAGGCAGT 54468166 rs75661995

HG00109 >hsa-mir-3922TCAAGGCCAGAGGTCCCACAAC TCAAGTCAAGGCCAGAGGTCCC
GCCAGAGGTCCCACAACAGGGC GGCCAGAGGTCCCACAACAGGG
GTCAAGGCCAGAGGTCCCACAA 104985443 rs61938575

HG00109 >hsa-mir-892cTCAGAAAGGTGCCAGTCACTTA TTCAGAAAGGTGCCAGTCACTT
TATTCAGAAAGGTGCCAGTCAC TGCCCTATTCAGAAAGGTGCCA
GCCCTATTCAGAAAGGTGCCAG 145074284 .

HG00109 >hsa-mir-222 TCAGTAGCCAGTGTAGATCCTG TGGCTCAGTAGCCAGTGTAGAT
TTGGCTCAGTAGCCAGTGTAGA TCATTGGCTCAGTAGCCAGTGT
TACCCTCATTGGCTCAGTAGCC 45606504 rs191727254

HG00109 >hsa-mir-515-1 TCCAAAAGAAAGCACTTTCTGT
TCTCCAAAAGAAAGCACTTTCT TTCTCCAAAAGAAAGCACTTTC
TCATTCTCCAAAAGAAAGCACT TGCAGTCATTCTCCAAAAGAAA 54182326
rs374576826
HG00109 >hsa-mir-663aTCCCAGGCGGGGCGCCGCGGGA TCCGGCGTCCCAGGCGGGGCGC
TTCCGGCGTCCCAGGCGGGGCG GCGCCGCGGGACCTCCCTCGTG
GGCGCCGCGGGACCTCCCTCGT 26188880 .

HG00109 >hsa-mir-149 TCCGTGTCTTCACTCCCGTGCT TGGCTCCGTGTCTTCACTCCCG
TCTGGCTCCGTGTCTTCACTCC CCGTGTCTTCACTCCCGTGCTT
AGCTCTGGCTCCGTGTCTTCAC 241395503 rs2292832

HG00109 >hsa-mir-618 TCCTTCTGAGTGTAATTACGTA TGTCCTTCTGAGTGTAATTACG
TTGTCCTTCTGAGTGTAATTAC TACTTGTCCTTCTGAGTGTAAT
GTCCTTCTGAGTGTAATTACGT 81329536 rs2682818

HG00109 >hsa-mir-492 TCGAGGACCTGCGGGACAAGAT TACAGGACCATCGAGGACCTGC
TACTACAGGACCATCGAGGACC TCCAGCCACTACTACAGGACCA
GACCTGCGGGACAAGATTCTTG 95228179 rs200816308

HG00109 >hsa-mir-3183TCGGAGTCGCTCGGAGCAGCCA TCTCGGAGTCGCTCGGAGCAGC
TCTCTCGGAGTCGCTCGGAGCA TCTGCCCTGCCTCTCTCGGAGT
TGCCCTGCCTCTCTCGGAGTCG 925764 rs2663345

HG00109 >hsa-mir-520fTCTCAGGCTGTGACCCTCTAAA TCAGGCTGTGACCCTCTAAAGG
TGTGACCCTCTAAAGGGAAGCG TAAAGGGAAGCGCTTTCTGTGG
TGACCCTCTAAAGGGAAGCGCT 54185492 rs75598818

HG00109 >hsa-mir-4305TCTGGGTTCTTAGAGGCCTAAT TTCTGGGTTCTTAGAGGCCTAA
GTTCTGGGTTCTTAGAGGCCTA TCCAGTTCTGGGTTCTTAGAGG
CAGTTCTGGGTTCTTAGAGGCC 40238175 rs67976778

HG00109 >hsa-mir-1200TGAGCCATTCTGAGCCTCAATC TCCTGAGCCATTCTGAGCCTCA
TCTCCTGAGCCATTCTGAGCCT TTCTCCTGAGCCATTCTGAGCC
TGCTACTTCTCCTGAGCCATTC 36958995 rs180826747

HG00109 >hsa-mir-3151TGATGGGTGGGGCAATGGGATC TGGGTGGGGCAATGGGATCAGG
TGGGGCAATGGGATCAGGTGCC GGGGTGATGGGTGGGGCAATGG
GGGTGATGGGTGGGGCAATGGG 104166902 rs35605502

HG00109 >hsa-mir-570 TGCAAAGGTAATCGCAGTTTTT TGGGTGCAAAGGTAATCGCAGT
GTAATCGCAGTTTTTCCCATTA GGTAATCGCAGTTTTTCCCATT
TAGGTGGGTGCAAAGGTAATCG 195426305 rs9860655

HG00109 >hsa-mir-3156-2 TGCAGAAGAAAGATCTGGAAGT
GAAAGATCTGGAAGTGGGAGAC GAAGAAAGATCTGGAAGTGGGA
GCAGAAGAAAGATCTGGAAGTG CAGAAGAAAGATCTGGAAGTGG 14830215
rs113478966
HG00109 >hsa-mir-3156-3 TGCAGAAGAAAGATCTGGAAGT
GCAGAAGAAAGATCTGGAAGTG GAAGAAAGATCTGGAAGTGGGA
GAAAGATCTGGAAGTGGGAGAC AGAAGAAAGATCTGGAAGTGGG 14778721
rs2747232
HG00109 >hsa-mir-4327TGCAGGGGGGACTGGGAAGAGA TTGCAGGGGGGACTGGGAAGAG
GGGGGACTGGGAAGAGACCATG GGGGGGACTGGGAAGAGACCAT
TAGGCTTGCAGGGGGGACTGGG 31747678 rs115477019

HG00109 >hsa-mir-3152TGCCTCTGTTCTAACACAAGAC TTGCCTCTGTTCTAACACAAGA TATTGCCTCTGTTCTAACACAA TTATTGCCTCTGTTCTAACACA TGCAGAGTTATTGCCTCTGTTC 18573360 rs13299349

HG00109 >hsa-mir-1200TGCTACTTCTCCTGAGCCATTC TGAGCCATTCTGAGCCTCAGTC TACTTCTCCTGAGCCATTCTGA TCCTGAGCCATTCTGAGCCTCA TTCTCCTGAGCCATTCTGAGCC 36959006 .

HG00109 >hsa-mir-1200TGCTACTTCTCCTGAGCCATTC TGAGCCATTCTGAGCCTCAGTC TACTTCTCCTGAGCCATTCTGA TCCTGAGCCATTCTGAGCCTCA TTCTCCTGAGCCATTCTGAGCC 36958995 rs180826747 36959006 .

HG00109 >hsa-mir-378d-2 TGGACTTGGAGTCAGAAAACTT GACTTGGAGTCAGAAAACTTTC GGACTTGGAGTCAGAAAACTTT GAACACTGGACTTGGAGTCAGA TACAAGGAGAGAACACTGGACT 94928250 rs73692959

HG00109 >hsa-mir-4309TGGAGTCTAGGATTCCAGGATC GGAGTCTAGGATTCCAGGATCT TCTGGGGGTTCTGGAGTCTAGG TTCTGGAGTCTAGGATTCCAGG TGGGGGTTCTGGAGTCTAGGAT 103006047 rs12879262

HG00109 >hsa-mir-1273h TGGGAGGTCAAGGCTGTAGTGT TGAGCCTGGGAGGTCAAGGCTG TTGAGCCTGGGAGGTCAAGGCT TGCTTGAGCCTGGGAGGTCAAG TTGCTTGAGCCTGGGAGGTCAA 24214486 .

HG00109 >hsa-mir-1227TGGTGGGCACTGCTGGGGTGGG TGGGGCCAGGCGGTGGTGGGCA AGGCGGTGGTGGGCACTGCTGG GGTGGGCACTGCTGGGGTGGGC GTGGTGGGCACTGCTGGGGTGG 2234093 rs190788838

HG00109 >hsa-mir-323bTGTCCGTGGTGAGTTCGCATTA TTGTCCGTGGTGAGTTCGCATT TACTCGGAGGGAGGTTGTCCGT TCGGAGGGAGGTTGTCCGTGGT AGGTTGTCCGTGGTGAGTTCGC 101522556 rs56103835

HG00109 >hsa-mir-642bTTCCCTCTCCAAATGTGTCTTG TTGGGAGGTTCCCTCTCCAAAT TGGGAGGTTCCCTCTCCAAATG GAGTTGGGAGGTTCCCTCTCCA GTTGGGAGGTTCCCTCTCCAAA 46178217 rs111664333

HG00109 >hsa-mir-4277TTCTGAGCACAGTACACTGGGC TCGAGGCAGTTCTGAGCACAGT TGGGTCGAGGCAGTTCTGAGCA GTTCTGAGCACAGTACACTGGG GCAGTTCTGAGCACAGTACACT 1708902 rs115200817

HG00109 >hsa-mir-4277TTCTGAGCACAGTACACTGGGC TCGAGGCAGTTCTGAGCACAGT TGGGTCGAGGCAGTTCTGAGCA TTGGGTCGAGGCAGTTCTGAGC GTTCTGAGCACAGTACACTGGG 1708983 rs12523324

HG00109 >hsa-mir-4277TTCTGAGCACAGTACACTGGGC TCGAGGCAGTTCTGAGCACAGT TGGGTCGAGGCAGTTCTGAGCA TTGGGTCGAGGCAGTTCTGAGC

GTTCTGAGCACAGTACACTGGG 1708902 rs115200817 1708983 rs12523324

HG00109 >hsa-mir-553 TTTAAAACGGTGAGATTTTGTT TTTTAAAACGGTGAGATTTTGT ATTTTAAAACGGTGAGATTTTG TATTTTAAAACGGTGAGATTTT TTATTTTAAAACGGTGAGATTT 100746848 .

HG00109 >hsa-mir-553 TTTAAAACGGTGAGATTTTGTT TTTTAAAACGGTGAGATTTTGT ATTTTAAAACGGTGAGATTTTG TATTTTAAAACGGTGAGATTTT TTATTTTAAAACGGTGAGATTT 100746855 rs112891767

HG00109 >hsa-mir-553 TTTAAAACGGTGAGATTTTGTT TTTTAAAACGGTGAGATTTTGT ATTTTAAAACGGTGAGATTTTG TATTTTAAAACGGTGAGATTTT TTATTTTAAAACGGTGAGATTT 100746848 . 100746855 rs112891767

HG00109 >hsa-mir-1227GGCATTTGACCCCGTGCCACCC AGGCATTTGACCCCGTGCCACC AGGCATTTGACCCCGTGCCACC TGACCCCGTGCCACCCTTTTCC ATTTGACCCCGTGCCACCCTTT 2234093 rs190788838

HG00109 >hsa-mir-1343GCCCCTCCTGGGGCCCGCACTC CCCCTCCTGGGGCCCGCACTCT GGGGCCCGCACTCTCGCTCTGG CCCTCCTGGGGCCCGCACTCTC TGGGGCCCGCACTCTCGCTCTG 34963416 rs2986407

HG00109 >hsa-mir-149 AGGGAGGGAGGGACGGGGGCTG GGGCTGTGCTGGGGCAGCCGGA GAGGGAGGGAGGGACGGGGGCT GGGAGGGAGGGACGGGGGCTGT GGGACGGGGGCTGTGCTGGGGC 241395503 rs2292832

HG00109 >hsa-mir-3118-1 TGAAAATTCTTCTAGTGTG ATGAAAATTCTTCTAGTGTG TGCATTATGAAAATTCTTCTAG TTATGAAAATTCTTCTAGTGTG ATTATGAAAATTCTTCTAGTGT 142667330 rs76132421

HG00109 >hsa-mir-3151CTGATCCCACACCCCACCTGTC TGATCCCACACCCCACCTGTCA GATCCCACACCCCACCTGTCAC GGGCATCCCACCTGATCCCACA TCCCACCTGATCCCACACCCCA 104166902 rs35605502

HG00109 >hsa-mir-3180-4 CTCCGGATGCCAGTCCCTCATC GGAGGGTGAAGCCTCCGGATGC AGCGGAGGGTGAAGCCTCCGGA CTGGCCTGGTCGCGCTGTGGCT GAGCGGAGGGTGAAGCCTCCGG 15248720 rs75000738

HG00109 >hsa-mir-3180-4 GGAGGGTGAAGCCTCCGGATGC GGTGAAGCCTCCGGATGCCAGT GCGGAGGGTGAAGCCTCCGGAT AGCGGAGGGTGAAGCCTCCGGA GCCTGGTCGCGCTGTGGCGAAG 15248798 rs183853838

HG00109 >hsa-mir-3180-4 GGAGGGTGAAGCCTCCGGATGC GGTGAAGCCTCCGGATGCCAGT GCGGAGGGTGAAGCCTCCGGAT AGCGGAGGGTGAAGCCTCCGGA CTGGCCTGGTCGCGCTGTGGCT 15248720 rs75000738 15248798 rs183853838

HG00109 >hsa-mir-320eGAAAAGCTGGGTTGAGAAGGT AAAAGCTGGGTTGAGAAGGT

GGAAAAGCTGGGTTGAGAAGGT GGGAAAAGCTGGGTTGAGAAGG rs10423365

HG00109 >hsa-mir-412 CTTCACCTGGTTCACTAGCCGT ACCTGGTTCACTAGCCGTCCGT TGTACTTCACCTGGTTCACTAG CTGGTTCACTAGCCGTCCGTAT GTACTTCACCTGGTTCACTAGC 101531849 .

HG00109 >hsa-mir-4268CTCTCAGGATGTGATGTCACCT CCTCTCAGGATGTGATGTCACC GCTCCTCCTCTCAGGATGTGAT TCCTCTCAGGATGTGATGTCAC CTCCTCCTCTCAGGATGTGATG 220771223 rs4674470

HG00109 >hsa-mir-520fCAAGTGCTTCCTTTTAGAGGGT GCAAGTGCTTCCTTTTAGAGGG GTGCTTCCTTTTAGAGGGTTAC GCTTCCTTTTAGAGGGTTACCA AAGCAAGTGCTTCCTTTTAGAG 54185492 rs75598818

HG00109 >hsa-mir-580 TATTTGAGAATGATGAATCATT TGAATCATTAGGTTCCGGTCAG ATGAATCATTAGGTTCCGGTCA TTTGAGAATGATGAATCATTAG GAGAATGATGAATCATTAGGTT 36148057 rs115089112

HG00109 >hsa-mir-637 TGGCTAAGGTGTTGGCTCGGGC TGGCTAAGGTGTTGGCTCGGGC .

HG00109 >hsa-mir-646 AGCAGCTGCCTCTGAGGCCTCA CTGAGGCCTCAGGCTCAGTGGC CTCTGAGGCCTCAGGCTCAGTG GCCTCTGAGGCCTCAGGCTCAG TCTGAGGCCTCAGGCTCAGTGG 58883534 rs6513496

HG00109 >hsa-mir-646 AGCAGCTGCCTCGGAGGCCTCA CGGAGGCCTCAGGCTCAGTGGC TCGGAGGCCTCAGGCTCAGTGG GAGGAAGCAGCTGCCTCGGAGG AGGAAGCAGCTGCCTCGGAGGC 58883605 rs6513497

HG00109 >hsa-mir-646 AGCAGCTGCCTCGGAGGCCTCA CGGAGGCCTCAGGCTCAGTGGC CTCGGAGGCCTCAGGCTCAGTG GCCTCGGAGGCCTCAGGCTCAG TCGGAGGCCTCAGGCTCAGTGG 58883534 rs6513496 58883605 rs6513497

HG00109 >hsa-mir-658 TAGGTCGGTTGGTCGGTCGGGA .

HG00109 >hsa-mir-658 GTCCGTTGGTCGGTCGGGAACG G TCCGTTGGTCGGTCGGGAACGA .

HG00109 >hsa-mir-658 AGGTCGGTTGGTCGGTCGGGAA GTCGGTTGGTCGGTCGGGAACG TAGGTCGGTTGGTCGGTCGGGA . .

HG00109 >hsa-mir-1197GTAGGACACATGGTCTACTTCT ACACATGGTCTACTTCTTCTCA ACATGGTCTACTTCTTCTCAAT TAGGACACATGGTCTACTTCTT 101491923 rs141611518

HG00109 >hsa-mir-1227CATTTGACCCCGTGCCACCCTT ATTTGACCCCGTGCCACCCTTT AGGCATTTGACCCCGTGCCACC GACCCCGTGCCACCCTTTTCCC 2234093 rs190788838

HG00109 >hsa-mir-658 38240300

HG00109 >hsa-mir-320c-1 AAAAGCTGGGTTGAGAGGGTAG AGCTGGGTTGAGAGGGTAGGAA CTGGGTTGAGAGGGTAGGAAAA AGGGTAGGAAAAAAATGATGTA

HG00109 >hsa-mir-651 AAAAGGAAAGTGTATCCTAAAA GGAAAGTGTATCCTAAAAGGCA TGTATCCTAAAAGGCAATGACA AAAGGAAAGTGTATCCTAAAAG 24214486 .

HG00109 >hsa-mir-202 AAAGAGGTATAGGGCATGGGAA AAGAGGTATAGGGCATGGGAAA GGGAAAACGGGGCGGTCGGGTC TAAAGAGGTATAGGGCATGGGA 154065347 rs142414368

HG00109 >hsa-mir-548ap AACAAAAACCACAATTACTTTT CAAAAACCACAATTACTTTTTA CAATTACTTTTTACTGACCTAA rs4414449 154065383 rs75538180

HG00109 >hsa-mir-548ap AACAAAAACCACAATTACTTTT CAAAAACCACAATTACTTTTTA TTACTTTTTACTGACCTAAAGA rs4577031 154065347 rs142414368 154065383 rs75538180

HG00109 >hsa-mir-548ap AACAAAAACCACAATTACTTTT CAAAAACCACAATTACTTTTTA CAATTACTTTTTACTGACCTAA rs4414449 93466866 rs2155248

HG00109 >hsa-mir-1255b-2 AACCACTTTCTTTGCTCATCCG AAACCACTTTCTTTGCTCATCC CTTTCTTTGCTCATCCGTAAGG rs79639536 105154089 rs7911488

HG00109 >hsa-mir-520fAAGCAAGTGCTTCCTTTTAGAG AGCAAGTGCTTCCTTTTAGAGG GTGCTTCCTTTTAGAGGGTTAC TGCTTCCTTTTAGAGGGTTACC 52013832 rs374103744

HG00109 >hsa-mir-3938AATTCCCTTGTAGATAACCCGG ATAACCCGGTGGTCAGGTTGGA TAGATAACCCGGTGGTCAGGTT CCTTGTAGATAACCCGGTGGTC 34963416 rs2986407

HG00109 >hsa-mir-597 ACAGTGGTTCTCTTGTGGCTTA GGCTTAAGCGTAATGTAGAGTA AATGTACAGTGGTTCTCTTGTG TGTACAGTGGTTCTCTTGTGGC 241395503 rs2292832

HG00109 >hsa-mir-449cACAGTTGCTAGTTGCACTCCTC AACAGTTGCTAGTTGCACTCCT GTTGCTAGTTGCACTCCTCTCT GTTGCACTCCTCTCTGTTGCAT 129410227 rs76481776

HG00109 >hsa-mir-3118-1 ACTGCATTATGAAAATTCTTCT ATTATGAAAATTCTTCTAGTGT GCATTATGAAAATTCTTCTAGT CTGCATTATGAAAATTCTTCTA

HG00109 >hsa-mir-3166AGACAATGCCTACTGGCCTAAG AACGCAGACAATGCCTACTGGC ATGCCTACTGGCCTAAGAAAAA CAATGCCTACTGGCCTAAGAAA 49768168 rs191840972

HG00109 >hsa-mir-642bAGATACATTTGGAGAGGGACCC TTGGAGAGGGACCCTCCCAACT TTTGGAGAGGGACCCTCCCAAC ATACATTTGGAGAGGGACCCTC 61582708 rs174561

HG00109 >hsa-mir-3180-4 AGCGGAGGGTGAAGCCTCCGGA

CGGAGGGTGAAGCCTCCGGATG GAGCGGAGGGTGAAGCCTCCGG
GCGGAGGGTGAAGCCTCCGGAT 54385599 rs11614913

HG00109 >hsa-mir-3180-4 AGCGGAGGGTGAAGCCTCCGGA
CGGAGGGTGAAGCCTCCGGATG GAGCGGAGGGTGAAGCCTCCGG
GCGGAGGGTGAAGCCTCCGGAT 135061112 rs12355840

HG00109 >hsa-mir-30d AGCTTTCAGTCAGATGTTTGCT GGCTAAGCTTTCAGTCAGATGT
GCTAAGCTTTCAGTCAGATGTT TTCAGTCAGATGTTTGCTGCTA 41522213
rs7207008
HG00109 >hsa-mir-519a-2 AGGAAAGTGCATCCTTTTAGAG
AGTGCATCCTTTTAGAGGGTTA GGAAAGTGCATCCTTTTAGAGG
GAAAGGAAAGTGCATCCTTTTA

HG00109 >hsa-mir-646 AGGAAGCAGCTGCCTCGGAGGC CCAGGAGAGGAAGCAGCTGCCT
GCTGCCTCGGAGGCCTCAGGCT GAGAGGAAGCAGCTGCCTCGGA 45606504
rs191727254
HG00109 >hsa-mir-646 AGGAAGCAGCTGCCTCGGAGGC TGCCTCGGAGGCCTCAGGCTCA
CGGAGGCCTCAGGCTCAGTGGC CTGCCTCGGAGGCCTCAGGCTC

HG00109 >hsa-mir-646 AGGAAGCAGCTGCCTCTGAGGC GCTGCCTCTGAGGCCTCAGGCT
CTGAGGCCTCAGGCTCAGTGGC TCTGAGGCCTCAGGCTCAGTGG

HG00109 >hsa-mir-630 AGTATTCTGTACCAGGGAAGGT ACCTAGTATTCTGTACCAGGGA
CCAGGGAAGGTAGTTCTTAACT CAGGGAAGGTAGTTCTTAACTA

HG00109 >hsa-mir-3686AGTGATCTGTAAGAGAAAGTAA TCTGTAAGAGAAAGTAAATGAA
GTAAGAGAAAGTAAATGAAAGA CAGTGATCTGTAAGAGAAAGTA 135817150
.
HG00109 >hsa-mir-323bATACACGGTCGACCTCTTTTCG TACACGGTCGACCTCTTTTCGG
ACACGGTCGACCTCTTTTCGGT rs56103835 67094171 rs12402181

HG00109 >hsa-mir-642aATTTGGAGAGGGAACCTCCCAA AGACACATTTGGAGAGGGAACC
ACACATTTGGAGAGGGAACCTC CACATTTGGAGAGGGAACCTCC 142667330
rs76132421
HG00109 >hsa-mir-4293CACACCAGCCTGACAGGAACAG TCACACCAGCCTGACAGGAACA
CACCAGCCTGACAGGAACAGCC CAGCCTGACAGGAACAGCCTGT 104166902
rs35605502
HG00109 >hsa-mir-515-1 CAGAGTGCCTTCTTTTGGAGCA
GAGTGCCTTCTTTTGGAGCATT TGCCTTCTTTTGGAGCATTACT
GTGCCTTCTTTTGGAGCATTAC 18573360 rs13299349

HG00109 >hsa-mir-1908CCACCGGCCGCCGGCTCCGCCC CCGCCGGCTCCGCCCCGGCCCC
GGCCGCCGGCTCCGCCCCGGCC CGGCCGCCGGCTCCGCCCCGGC 14830215
rs113478966
HG00109 >hsa-mir-3151CCACCTGATCCCACACCCCACC CACCTGATCCCACACCCCACCT
CCCACCTGATCCCACACCCCAC TGATCCCACACCCCACCTGTCA 87909701

.
HG00109 >hsa-mir-188 CCCCTCCCATATGCAGGGTTTG ACCCCTCCCATATGCAGGGTTT CCCTCCCATATGCAGGGTTTGC CCATATGCAGGGTTTGCAGGAT 15248720 rs75000738
HG00109 >hsa-mir-1343CCCCTCCTGGGGCCCGCACTCT CCCTCCTGGGGCCCGCACTCTC CCTGGGGCCCGCACTCTCGCTC TGGGGCCCGCACTCTCGCTCTG 15248798 rs183853838
HG00109 >hsa-mir-3679CCCTTCCCCCCAGTAATCTTCA CCTTCCCCCCAGTAATCTTCAT CTTCCCCCCAGTAATCTTCATC TCCCCCCAGTAATCTTCATCAT 15248720 rs75000738 15248798 rs183853838
HG00109 >hsa-mir-4268CCTCTCAGGATGTGATGTCACC CTCCTCTCAGGATGTGATGTCA CTCCTCCTCTCAGGATGTGATG rs4674470

HG00109 >hsa-mir-4254CCTGGAGATACTCCACCATCTC AGATACTCCACCATCTCCCCCA GGAGATACTCCACCATCTCCCC rs12731294

HG00109 >hsa-mir-3152CCTGTGTTAGAATAAGGGCAAT TTAGAATAAGGGCAATAACTCT AGAATAAGGGCAATAACTCTGC TGTGTTAGAATAAGGGCAATAA 18392913 rs7247767
HG00109 >hsa-mir-182 CGGTGGTTCTAGACTTGCCAAC CCGGTGGTTCTAGACTTGCCAA TCCGGTGGTTCTAGACTTGCCA GCCAACTATGGGGCGAGGACTC 19263542 .
HG00109 >hsa-mir-3117CTCATATAGTGCCAGGTGTTTT GACTCATATAGTGCCAGGTGTT TCATATAGTGCCAGGTGTTTTG AGACTCATATAGTGCCAGGTGT

HG00109 >hsa-mir-196a-2 CTCGGCAACAAGAAACTGTCTG CAAGAAACTGTCTGAGTTACAT CAACAAGAAACTGTCTGAGTTA ACAAGAAACTGTCTGAGTTACA

HG00109 >hsa-mir-486-2 CTCGGCGCAGCTCAGTACAGGA AGGGCCTCGGCGCAGCTCAGTA TCGGCGCAGCTCAGTACAGGAT GGGCCTCGGCGCAGCTCAGTAC 72744798 rs745666

HG00109 >hsa-mir-3615CTCTCTCGGCTCCTCGCGGCTC GGCTCCTCGCGGCTCGCGGCGG CGGCTCCTCGCGGCTCGCGGCG TCGGCTCCTCGCGGCTCGCGGC 134884700 rs6430498
HG00109 >hsa-mir-888 CTCTTTGGGTGAAGGAAGGCTC CTGACACCTCTTTGGGTGAAGG GACTGACACCTCTTTGGGTGAA CCTCTTTGGGTGAAGGAAGGCT 130496365 rs6997249
HG00109 >hsa-mir-888 CTCTTTGGGTGAAGGAAGGCTC GACTGACACCTCTTTGGGTGAA CTGACACCTCTTTGGGTGAAGG TGACACCTCTTTGGGTGAAGGA 104985443 rs61938575
HG00109 >hsa-mir-1269a CTGGACTGAGCCATGCTACTGG TGCCTGGACTGAGCCATGCTAC AATGCCTGGACTGAGCCATGCT rs73239138

HG00109 >hsa-mir-1304CTGTAGCATCGAACCCCTGGGC CTCACTGTAGCATCGAACCCCT GAACCCCTGGGCTCAAGTGATT CGAACCCCTGGGCTCAAGTGAT 131701279

rs367805
HG00109 >hsa-mir-3922CTGTGGGACTTCTGGCCTTGAC ACCTGTGGGACTTCTGGCCTTG GGGACTTCTGGCCTTGACTTGA TGGGACTTCTGGCCTTGACTTG 55886574 rs59684995
HG00109 >hsa-mir-2117CTGTTCTCTTTGCCAAGGACAG TAGCTGTTCTCTTTGCCAAGGA TCTCTTTGCCAAGGACAGATCT TGTTCTCTTTGCCAAGGACAGA 101531849 .
HG00109 >hsa-mir-940 GAAGGCAGGGCCCC-GCTCCCC G CCC-GCTCCCCGGGCCTGACCC rs35356504

HG00109 >hsa-mir-888 GACACCTCTTTGGGTGAAGGAA ACTGACACCTCTTTGGGTGAAG CTGACACCTCTTTGGGTGAAGG GACTGACACCTCTTTGGGTGAA

HG00109 >hsa-mir-4274GACCCAGCAGTCCCTCCCCCTG CCCAGCAGTCCCTCCCCCTGCA TGACCCAGCAGTCCCTCCCCCT TCAGGTGACCCAGCAGTCCCTC 7461769 rs12512664
HG00109 >hsa-mir-1307GACTCGGCGTGGCGTCGGTCGT CGTGGCGTCGGTCGTGGTAGAT ATCGACTCGGCGTGGCGTCGGT CGACTCGGCGTGGCGTCGGTCG 14425204 rs12780876
HG00109 >hsa-mir-3188GAGGCTTTGTGCGGATACGGGG GAGAGGCTTTGTGCGGATACGG GCGGATACGGGGCTGGAGGCCT rs7247237 54468166 rs75661995

HG00109 >hsa-mir-3188GAGGCTTTGTGCGGATACGGGG GGAGAGGCTTTGTGCGGATACG GAGAGGCTTTGTGCGGATACGG rs7247767 41518007 .

HG00109 >hsa-mir-3188GAGGCTTTGTGCGGATACGGGG GAGAGGCTTTGTGCGGATACGG GGAGAGGCTTTGTGCGGATACG rs7247237 49775351 rs151318590
HG00109 >hsa-mir-629 GAGGTTCTCCCAACGTAAGCCC AGGTTCTCCCAACGTAAGCCCA TCTCCCAACGTAAGCCCAGCCC CAGGAGGTTCTCCCAACGTAAG 54182326 rs374576826
HG00109 >hsa-mir-629 GAGGTTCTCCCAACGTAAGCCC AGGAGGTTCTCCCAACGTAAGC GGAGGTTCTCCCAACGTAAGCC TCTCCCAACGTAAGCCCAGCCC 54265670 .
HG00109 >hsa-mir-629 GAGGTTCTCCCAACGTAAGCCC AGGTTCTCCCAACGTAAGCCCA TCTCCCAACGTAAGCCCAGCCC CCCAACGTAAGCCCAGCCCCTC 54185492 rs75598818
HG00109 >hsa-mir-222 GCAGCTACATCTGGCTACTGGG TACTGGGTCTCTGATGGCATCT GCTACTGGGTCTCTGATGGCAT CTGGCTACTGGGTCTCTGATGG

HG00109 >hsa-mir-500bGCAGTGCACCCAGGCAAGGATT CACCCAGGCAAGGATTCTGCGA AGGCAAGGATTCTGCGAGGGGG TGCAGTGCACCCAGGCAAGGAT

HG00109 >hsa-mir-3180-4 GCGGAGGGTGAAGCCTCCGGAT CGCTGGCCTGGTCGCGCTGTGG TCGCTGGCCTGGTCGCGCTGTG AAGCCTCCGGATGCCAGTCCCT 86368959 rs4577031

HG00109 >hsa-mir-149 GGGAGGGAGGGACGGGGGCTGT GGAGGGACGGGGGCTGTGCTGG AGGGACGGGGGCTGTGCTGGGG GAGGAGGGAGGGAGGGACGGGG 195426305 rs9860655

HG00109 >hsa-mir-3936TAAGGGGTGTATGGCAGATGCA TTCTGGTAAGGGGTGTATGGCA CACCCGACAGATGCACTTGGCA TGTATGGCAGATGCACCCGACA 36148057 rs115089112

HG00109 >hsa-mir-300 TATACAAGGGCAGACTCTCTCT TGATTATACAAGGGCAGACTCT ATTATACAAGGGCAGACTCTCT rs12894467 9599255 rs146125159

HG00109 >hsa-mir-412 TCACCTGGTTCACTAGCCGTCC TCACCTGGTTCACTAGCCGTCC ATGTACTTCACCTGGTTCACTA CTTCACCTGGTTCACTAGCCGT 70371761 rs377691713

HG00109 >hsa-mir-892cTCCTTTCTAAGTGGAGTAGGGC CTTTCTAAGTGGAGTAGGGCTT ACTGTTTCCTTTCTAAGTGGAG TTCCTTTCTAAGTGGAGTAGGG 70371794 .

HG00109 >hsa-mir-656 TGAATATTATACAGTCAACCTC ATTATACAGTCAACCTCTTTCC AATATTATACAGTCAACCTCTT ATATTATACAGTCAACCTCTTT 70371761 rs377691713 70371794 .

HG00109 >hsa-mir-570 TGCGAAAACAGCAATTACCTTT GAAAACAGCAATTACCTTTGCA ACAGCAATTACCTTTGCACCAA AAACAGCAATTACCTTTGCACC 72879653 rs113971639

HG00109 >hsa-mir-1273h TGCTGCAGACTCGACCTCCCAG TGCAGACTCGACCTCCCAGGCT CTGCAGACTCGACCTCCCAGGC AGACTCGACCTCCCAGGCTTAA

HG00109 >hsa-mir-3156-2 TGGCCCCCACTTCCAGATCTTT CCCCCACTTCCAGATCTTTCTC ACTTCCAGATCTTTCTCTCTGT CCCCACTTCCAGATCTTTCTCT 46178217 rs111664333

HG00109 >hsa-mir-1303TTTAGAGACGGGGTCTTGCTCT TAGAGACGGGGTCTTGCTCTGT ACGGGGTCTTGCTCTGTTGCCA GGGTCTTGCTCTGTTGCCAGGC 46178217 rs111664333

HG00109 >hsa-mir-1303TTTAGAGACGGGGTCTTGCTCT TAGAGACGGGGTCTTGCTCTGT TTAGAGACGGGGTCTTGCTCTG ACGGGGTCTTGCTCTGTTGCCA 58883534 rs6513496

HG00109 >hsa-mir-1303TTTAGAGACGGGGTCTTGCTCT TAGAGACGGGGTCTTGCTCTGT TTAGAGACGGGGTCTTGCTCTG ACGGGGTCTTGCTCTGTTGCCA 58883605 rs6513497

HG00109 >hsa-mir-580 TTTGAGAATGATGAATCATTAG GATGAATCATTAGGTTCCGGTC AATGATGAATCATTAGGTTCCG AGAATGATGAATCATTAGGTTC 58883534 rs6513496 58883605 rs6513497

HG00109 >hsa-mir-133bTTTGGTCCCCTTCAACCAGCTA TGGTCCCCTTCAACCAGCTACA CCTTCAACCAGCTACAGCAGGG AGAGGTTTGGTCCCCTTCAACC 8095036 rs111336920

HG00110 >hsa-mir-3125AGAATGGATAGAGGAAGCTGTG GAGGAAGCTGTGGAGAGAACTC AGAGGAAGCTGTGGAGAGAACT GCTGTGGAGAGAACTCACGGTG GGAAGCTGTGGAGAGAACTCAC 12877501 rs78852835

HG00110 >hsa-mir-1255b-1 ATGAGCAAAGAAAGTGGTTTCT
GATGAGCAAAGAAAGTGGTTTC TATGGATGAGCAAAGAAAGTGG
GGATGAGCAAAGAAAGTGGTTT ATGGATGAGCAAAGAAAGTGGT 36428048
rs6841938
HG00110 >hsa-mir-1273h CCTGGGAGGTCAAGGCTGTAGT
TGGGAGGTCAAGGCTGTAGTGT GCCTGGGAGGTCAAGGCTGTAG
ATTGCTTGAGCCTGGGAGGTCA TTGAGCCTGGGAGGTCAAGGCT 24214486 .

HG00110 >hsa-mir-3683CTGCGACATTGGAAGTAGTATC CCCCTGCGACATTGGAAGTAGT
TGTACACCCCCTGCGACATTGG GTGTACACCCCCTGCGACATTG
GTACACCCCCTGCGACATTGGA 7106636 rs6977967

HG00110 >hsa-mir-548ak GCAAAAGTAACTGCGGTTTTTG
TGCAAAAGTAACTGCGGTTTTT CAAAAGTAACTGCGGTTTTTGA
GTGCAAAAGTAACTGCGGTTTT rs7070684

HG00110 >hsa-mir-608 GGCCAAGGTGGGCCAGGGGTGG AAGGTGGGCCAGGGGTGGTGTT
GGGGTGGTGTTGGGACAGCTGC TGGTGTTGGGACAGCTGCGTTT
GGTGGGCCAGGGGTGGTGTTGG 102734778 rs4919510

HG00110 >hsa-mir-3620GGTGGGGGCCAGCAGGGAGTGG TGAGGTGGGGGCCAGCAGGGAG
GTGAGGTGGGGGCCAGCAGGGA GAGGTGGGGGCCAGCAGGGAGT
AGGTGGGGGCCAGCAGGGAGTG 228284991 rs2070960

HG00110 >hsa-mir-877 TAGAGGAGATGGCGCAGGGGAC GAGGAGATGGCGCAGGGGACAC
GAGATGGCGCAGGGGACACGGG GGAGATGGCGCAGGGGACACGG
TGGCGCAGGGGACACGGGCAAA 30552187 rs372113020

HG00110 >hsa-mir-4326TCTGCTGTTCCTCTGTCTCCCA TGGTCTGCTGTTCCTCTGTCTC
CTGGTCTGCTGTTCCTCTGTCT GCTGTTCCTCTGTCTCCCAGAC
TGCTGTTCCTCTGTCTCCCAGA 61918164 rs6062431

HG00110 >hsa-mir-553 TTTTAAAACGGTGAGATTTTGT TTATTTTAAAACGGTGAGATTT
TTTTATTTTAAAACGGTGAGAT TATTTTAAAACGGTGAGATTTT
TTTAAAACGGTGAGATTTTGTT 100746835 .

HG00110 >hsa-mir-553 TTTTAAAACGGTGAGATTTTGT TATTTTAAAACGGTGAGATTTT
TTTTATTTTAAAACGGTGAGAT TTTAAAACGGTGAGATTTTGTT
ATTTTATTTTAAAACGGTGAGA 100746848 .

HG00110 >hsa-mir-553 TTTTAAAACGGTGAGATTTTGT TATTTTAAAACGGTGAGATTTT
TTTTATTTTAAAACGGTGAGAT TTTAAAACGGTGAGATTTTGTT
ATTTTATTTTAAAACGGTGAGA 100746835 . 100746848 .

HG00110 >hsa-mir-553 TTTTAAAACGGTGAGATTTTGT TTTTATTTTAAAACGGTGAGAT
AATTTTATTTTAAAACGGTGAG TTATTTTAAAACGGTGAGATTT

TTTATTTTAAAACGGTGAGATT 100746835 . 100746855
rs112891767
HG00110 >hsa-mir-553 TTTTAAAACGGTGAGATTTTGT TTTTATTTTAAAACGGTGAGAT
TATTTTAAAACGGTGAGATTTT TTTAAAACGGTGAGATTTTGTT
ATTTTATTTTAAAACGGTGAGA 100746848 . 100746855
rs112891767
HG00110 >hsa-mir-553 TTTTAAAACGGTGAGATTTTGT TTTTATTTTAAAACGGTGAGAT
TATTTTAAAACGGTGAGATTTT TTTAAAACGGTGAGATTTTGTT
ATTTTATTTTAAAACGGTGAGA 100746835 . 100746848 .
100746855 rs112891767
HG00110 >hsa-mir-553 TTTATTTTAAAACGGTGAGAT AATTTTATTTTAAAACGGTGAG
TTTTAAAACGGTGAGATTTTGT TTATTTTAAAACGGTGAGATTT
TTTAAAACGGTGAGATTTTGTT 100746855 rs112891767

HG00110 >hsa-mir-3175AGAGAACGCAGTGACGTCTGGC GAGAGAACGCAGTGACGTCTGG
GGAGAGAACGCAGTGACGTCTG GGGAGAGAACGCAGTGACGTCT
GGGGAGAGAACGCAGTGACGTC 93447631 rs1439619

HG00110 >hsa-mir-378hAGATGGGATGAGCCCTGGCTCT TCAGATGGGATGAGCCCTGGCT
TGTCAGATGGGATGAGCCCTGG GATGGGATGAGCCCTGGCTCTG
TGGTGTCAGATGGGATGAGCCC 154209024 rs702742

HG00110 >hsa-mir-3135b AGCGAGTGCAGTGGTGCAGTCA
AGGCTGGAGCGAGTGCAGTGGT CTGGAGCGAGTGCAGTGGTGCA
CAGGCTGGAGCGAGTGCAGTGG TGGAGCGAGTGCAGTGGTGCAG 32717702
rs4285314
HG00110 >hsa-mir-548ap AGTAATTGCAGTCTTTGTCATT
AAGTAATTGCAGTCTTTGTCAT AAAGTAATTGCAGTCTTTGTCA
AAAAGTAATTGCAGTCTTTGTC CAAAAGTAATTGCAGTCTTTGT 86368898
rs4414449
HG00110 >hsa-mir-548ap AGTAATTGCAGTCTTTGTCATT
AAGTAATTGCAGTCTTTGTCAT AAAGTAATTGCAGTCTTTGTCA
AAAAGTAATTGCAGTCTTTGTC CAAAAGTAATTGCAGTCTTTGT 86368898
rs4414449 86368959 rs4577031
HG00110 >hsa-mir-548ap AGTAATTGCGGTCTTTGTCATT
AAGTAATTGCGGTCTTTGTCAT AAAGTAATTGCGGTCTTTGTCA
AAAAGTAATTGCGGTCTTTGTC CAAAAGTAATTGCGGTCTTTGT 86368959
rs4577031
HG00110 >hsa-mir-612 GGGCTTCTGAGCTCCTTAGCAC AGGGCTTCTGAGCTCCTTAGCA
CAGGGCTTCTGAGCTCCTTAGC GCAGGGCTTCTGAGCTCCTTAG
GGCAGGGCTTCTGAGCTCCTTA 65211979 rs12803915

HG00110 >hsa-mir-548h-3 GTAATCGTGGTTTTTGTCATTG
TGCAAAAGTAATCGTGGTTTTT TGGTGCAAAAGTAATCGTGGTT
TTGGTGCAAAAGTAATCGTGGT AGTAATCGTGGTTTTTGTCATT 13446924
rs9913045
HG00110 >hsa-mir-573 GTGTAACTGATCAGGATCTACT TGTGTAACTGATCAGGATCTAC

GATGTGTAACTGATCAGGATCT TGATGTGTAACTGATCAGGATC
GTGATGTGTAACTGATCAGGAT 24521902 rs76014664

HG00110 >hsa-mir-573 GTGTAACTGATCAGGATCTACT TGTGTAACTGATCAGGATCTAC
GATGTGTAACTGATCAGGATCT TGATGTGTAACTGATCAGGATC
GTGATGTGTAACTGATCAGGAT 24521904 rs78830737

HG00110 >hsa-mir-573 GTGTAACTGATCAGGATCTACT TGTGTAACTGATCAGGATCTAC
GATGTGTAACTGATCAGGATCT TGATGTGTAACTGATCAGGATC
GTGATGTGTAACTGATCAGGAT 24521902 rs76014664 24521904
rs78830737

HG00110 >hsa-mir-3117TAAAGGGCCAGACACTATACGA GCCAGACACTATACGAGTCATA
GGGCCAGACACTATACGAGTCA GGCCAGACACTATACGAGTCAT
CCCTAAAGGGCCAGACACTATA 67094171 rs12402181

HG00110 >hsa-mir-3683TACACCCCCTGCGACATTGGAA TGTACACCCCCTGCGACATTGG
GGGTGTACACCCCCTGCGACAT GTACACCCCCTGCGACATTGGA
GGTGTACACCCCCTGCGACATT 7106636 rs6977967

HG00110 >hsa-mir-629 TACGTTGGGAGAACTTTTACGG TTACGTTGGGAGAACTTTTACG
GTTTACGTTGGGAGAACTTTTA TTTACGTTGGGAGAACTTTTAC
GGTTTACGTTGGGAGAACTTTT 70371794 .

HG00110 >hsa-mir-629 TACGTTGGGAGAACTTTTATGG TTACGTTGGGAGAACTTTTATG
TTTACGTTGGGAGAACTTTTAT TGGGTTTACGTTGGGAGAACTT
GTTTACGTTGGGAGAACTTTTA 70371761 rs377691713

HG00110 >hsa-mir-629 TACGTTGGGAGAACTTTTATGG TTACGTTGGGAGAACTTTTATG
TTTACGTTGGGAGAACTTTTAT TGGGTTTACGTTGGGAGAACTT
GTTTACGTTGGGAGAACTTTTA 70371761 rs377691713 70371794 .

HG00110 >hsa-mir-888 TACTCAAAAAGCCGTCAGTCAC TCTACTCAAAAAGCCGTCAGTC
TGCTCTACTCAAAAAGCCGTCA GCTCTACTCAAAAAGCCGTCAG
GTGCTCTACTCAAAAAGCCGTC 145076356 rs112850228

HG00110 >hsa-mir-888 TACTCAAAAAGCCGTCAGTCAC TCTACTCAAAAAGCCGTCAGTC
TGCTCTACTCAAAAAGCCGTCA GCTCTACTCAAAAAGCCGTCAG
GTGCTCTACTCAAAAAGCCGTC 145076302 rs5965660 145076356
rs112850228

HG00110 >hsa-mir-888 TACTCAAAAAGCCTTCAGTCAC TCTACTCAAAAAGCCTTCAGTC
TGCTCTACTCAAAAAGCCTTCA GCTCTACTCAAAAAGCCTTCAG
GTGCTCTACTCAAAAAGCCTTC 145076355 rs143634721 145076356
rs112850228

HG00110 >hsa-mir-888 TACTCAAAAAGCCTTCAGTCAC TCTACTCAAAAAGCCTTCAGTC
TGCTCTACTCAAAAAGCCTTCA GCTCTACTCAAAAAGCCTTCAG
GTGCTCTACTCAAAAAGCCTTC 145076302 rs5965660 145076355
rs143634721 145076356 rs112850228

HG00110 >hsa-mir-888 TACTCAAAAAGCTGTCAGTCAC TCTACTCAAAAAGCTGTCAGTC
TGCTCTACTCAAAAAGCTGTCA GCTCTACTCAAAAAGCTGTCAG
GTGCTCTACTCAAAAAGCTGTC 145076302 rs5965660

HG00110 >hsa-mir-888 TACTCAAAAAGCTTTCAGTCAC TCTACTCAAAAAGCTTTCAGTC
TGCTCTACTCAAAAAGCTTTCA GCTCTACTCAAAAAGCTTTCAG
GTGCTCTACTCAAAAAGCTTTC 145076355 rs143634721

HG00110 >hsa-mir-888 TACTCAAAAAGCTTTCAGTCAC TCTACTCAAAAAGCTTTCAGTC
TGCTCTACTCAAAAAGCTTTCA GCTCTACTCAAAAAGCTTTCAG
GTGCTCTACTCAAAAAGCTTTC 145076302 rs5965660 145076355
rs143634721
HG00110 >hsa-mir-3125TAGAGGAAGCTGTGGAGAGAAC AGCTGTGGAGAGAACTCACGGT
TGGATAGAGGAAGCTGTGGAGA AAGCTGTGGAGAGAACTCACGG
AGGAAGCTGTGGAGAGAACTCA 12877501 rs78852835

HG00110 >hsa-mir-449cTAGGCAGTGTATTGCTAGCGGC TCAGATAGGCAGTGTATTGCTA
TGTCAGATAGGCAGTGTATTGC TGTGTCAGATAGGCAGTGTATT
TGGGATGTGTCAGATAGGCAGT 54468166 rs75661995

HG00110 >hsa-mir-590 TATTCATAAAAGTGCAGTATGG TTATTCATAAAAGTGCAGTATG
TTTATTCATAAAAGTGCAGTAT TGAGTTTATTCATAAAAGTGCA
GTTTATTCATAAAAGTGCAGTA 73605546 rs189727189

HG00110 >hsa-mir-564 TCAGCAGGCAACATGGCCGAGA TGTCAGCAGGCAACATGGCCGA
GTCAGCAGGCAACATGGCCGAG TGCCAGGCACGGTGTCAGCAGG
GTGTCAGCAGGCAACATGGCCG 44903433 .

HG00110 >hsa-mir-222 TCAGTAGCCAGTGTAGATCCTG TGGCTCAGTAGCCAGTGTAGAT
TTGGCTCAGTAGCCAGTGTAGA TCATTGGCTCAGTAGCCAGTGT
TACCCTCATTGGCTCAGTAGCC 45606504 rs191727254

HG00110 >hsa-mir-515-1 TCCAAAAGAAAGCACTTTCTGT
TCTCCAAAAGAAAGCACTTTCT TTCTCCAAAAGAAAGCACTTTC
TCATTCTCCAAAAGAAAGCACT TGCAGTCATTCTCCAAAAGAAA 54182326
rs374576826
HG00110 >hsa-mir-663aTCCCAGGCGGGGCGCCGCGGGA TCCGGCGTCCCAGGCGGGGCGC
TTCCGGCGTCCCAGGCGGGGCG GCGCCGCGGGACCTCCCTCGTG
GGCGCCGCGGGACCTCCCTCGT 26188880 .

HG00110 >hsa-mir-149 TCCGTGTCTTCACTCCCGTGCT TGGCTCCGTGTCTTCACTCCCG
TCTGGCTCCGTGTCTTCACTCC CCGTGTCTTCACTCCCGTGCTT
CTCCGTGTCTTCACTCCCGTGC 241395503 rs2292832

HG00110 >hsa-mir-618 TCCTTCTGAGTGTAATTACGTA TGTCCTTCTGAGTGTAATTACG
TTGTCCTTCTGAGTGTAATTAC TACTTGTCCTTCTGAGTGTAAT
GTCCTTCTGAGTGTAATTACGT 81329527 rs145551269

HG00110 >hsa-mir-618 TCCTTCTGAGTGTAATTACGTA TGTCCTTCTGAGTGTAATTACG TTGTCCTTCTGAGTGTAATTAC TACTTGTCCTTCTGAGTGTAAT GTCCTTCTGAGTGTAATTACGT 81329536 rs2682818

HG00110 >hsa-mir-618 TCCTTCTGAGTGTAATTACGTA TGTCCTTCTGAGTGTAATTACG TTGTCCTTCTGAGTGTAATTAC TACTTGTCCTTCTGAGTGTAAT GTCCTTCTGAGTGTAATTACGT 81329527 rs145551269 81329536 rs2682818

HG00110 >hsa-mir-492 TCGAGGACCTGCGGGACAAGAT TACAGGACCATCGAGGACCTGC TACTACAGGACCATCGAGGACC TCCAGCCACTACTACAGGACCA GACCTGCGGGACAAGATTCTTG 95228179 rs200816308

HG00110 >hsa-mir-3183TCGGAGTCGCTCGGAGCAGCCA TCTCGGAGTCGCTCGGAGCAGC TCTCTCGGAGTCGCTCGGAGCA TCTGCCCTGCCTCTCTCGGAGT TGCCTCTCTCGGAGTCGCTCGG 925764 rs2663345

HG00110 >hsa-mir-635 TGAAACAATGTCCATTAGGCTT GAAACAATGTCCATTAGGCTTT ACAATGTCCATTAGGCTTTGTT AACAATGTCCATTAGGCTTTGT CTGAAACAATGTCCATTAGGCT 66420592 rs77279010

HG00110 >hsa-mir-1255a TGAGCAAAGAAAGTAGATTTTT GCAAAGAAAGTAGATTTTTTAG TCAAGGATGAGCAAAGAAAGTA GAGCAAAGAAAGTAGATTTTTT TCTCAAGGATGAGCAAAGAAAG 102251501 rs28664200

HG00110 >hsa-mir-1200TGAGCCATTCTGAGCCTCAATC TCCTGAGCCATTCTGAGCCTCA TCTCCTGAGCCATTCTGAGCCT TTCTCCTGAGCCATTCTGAGCC TACTTCTCCTGAGCCATTCTGA 36958995 rs180826747

HG00110 >hsa-mir-1200TGAGCCATTCTGAGCCTCAGTC TCCTGAGCCATTCTGAGCCTCA TGCTACTTCTCCTGAGCCATTC TCTCCTGAGCCATTCTGAGCCT TTCTCCTGAGCCATTCTGAGCC 36959006 .

HG00110 >hsa-mir-1200TGAGCCATTCTGAGCCTCAGTC TCCTGAGCCATTCTGAGCCTCA TGCTACTTCTCCTGAGCCATTC TCTCCTGAGCCATTCTGAGCCT TTCTCCTGAGCCATTCTGAGCC 36958995 rs180826747 36959006 .

HG00110 >hsa-mir-3151TGATGGGTGGGGCAATGGGATC TGGGTGGGGCAATGGGATCAGG TGGGGCAATGGGATCAGGTGCC GGGGTGATGGGTGGGGCAATGG GGGTGATGGGTGGGGCAATGGG 104166902 rs35605502

HG00110 >hsa-mir-570 TGCAAAGGTAATCGCAGTTTTT TGGGTGCAAAGGTAATCGCAGT GTAATCGCAGTTTTTCCCATTA GGTAATCGCAGTTTTTCCCATT TAGGTGGGTGCAAAGGTAATCG 195426305 rs9860655

HG00110 >hsa-mir-3156-3 TGCAGAAGAAAGATCTGGAAGT GCAGAAGAAAGATCTGGAAGTG GAAAGATCTGGAAGTGGGAGAC

GAAGAAAGATCTGGAAGTGGGA   AGAAGAAAGATCTGGAAGTGGG   14778721
rs2747232
HG00110   >hsa-mir-1254-2   TGGAAGCTGGAGCCTGCAGTGA
TGAGCCTGGAAGCTGGAGCCTG   GAAGCTGGAGCCTGCAGTGAGC
GGAAGCTGGAGCCTGCAGTGAG   GCCTGGAAGCTGGAGCCTGCAG   23682383
rs200793185
HG00110   >hsa-mir-378d-2   TGGACTTGGAGTCAGAAAACTT
GACTTGGAGTCAGAAAACTTTC   GGACTTGGAGTCAGAAAACTTT
GAACACTGGACTTGGAGTCAGA   CTGGACTTGGAGTCAGAAAACT   94928250
rs73692959
HG00110   >hsa-mir-3652TGGAGGCGGCTCCTGCGATCGA   GGAGGCGGCTCCTGCGATCGAA
TGGGGGGTGGAGGCGGCTCCTG   GTGGAGGCGGCTCCTGCGATCG
GGTGGAGGCGGCTCCTGCGATC   104324266   rs17797090

HG00110   >hsa-mir-877 TGGCGCAGGGGACACGGGCAAA   GACACGGGCAAAGACTTGGGGG
GGACACGGGCAAAGACTTGGGG   GGGACACGGGCAAAGACTTGGG
GGGGACACGGGCAAAGACTTGG   30552187   rs372113020

HG00110   >hsa-mir-608 TGGGACAGCTGCGTTTAAAAAG   TTGGGACAGCTGCGTTTAAAAA
GGACAGCTGCGTTTAAAAAGGC   TGTTGGGACAGCTGCGTTTAAA
GGGACAGCTGCGTTTAAAAAGG   102734778   rs4919510

HG00110   >hsa-mir-1273h   TGGGAGGTCAAGGCTGTAGTGT
TGAGCCTGGGAGGTCAAGGCTG   TTGAGCCTGGGAGGTCAAGGCT
TGCTTGAGCCTGGGAGGTCAAG   TTGCTTGAGCCTGGGAGGTCAA   24214486   .

HG00110   >hsa-mir-1227TGGGGCCAGGCGGTGGTGGGCA   TGGTGGGCACTGCTGGGGTGGG
AGGCGGTGGTGGGCACTGCTGG   GTGGGGCCAGGCGGTGGTGGGC
GGGGCCAGGCGGTGGTGGGCAC   2234093   rs190788838

HG00110   >hsa-mir-3620TGGGGGCCAGCAGGGAGTGGGT   TGAGGTGGGGGCCAGCAGGGAG
GGGGGCCAGCAGGGAGTGGGTT   GTGGGGGCCAGCAGGGAGTGGG
GTGAGGTGGGGGCCAGCAGGGA   228284991   rs2070960

HG00110   >hsa-mir-323bTGTCCGTGGTGAGTTCGCATTA   TTGTCCGTGGTGAGTTCGCATT
TACTCGGAGGGAGGTTGTCCGT   TCGGAGGGAGGTTGTCCGTGGT
AGGTTGTCCGTGGTGAGTTCGC   101522556   rs56103835

HG00110   >hsa-mir-4277TTCTGAGCACAGTACACTGGGC   TCGAGGCAGTTCTGAGCACAGT
TGGGTCGAGGCAGTTCTGAGCA   GTTCTGAGCACAGTACACTGGG
GCAGTTCTGAGCACAGTACACT   1708902   rs115200817

HG00110   >hsa-mir-4277TTCTGAGCACAGTACACTGGGC   TCGAGGCAGTTCTGAGCACAGT
TGGGTCGAGGCAGTTCTGAGCA   TTGGGTCGAGGCAGTTCTGAGC
GTTCTGAGCACAGTACACTGGG   1708983   rs12523324

HG00110   >hsa-mir-4277TTCTGAGCACAGTACACTGGGC   TCGAGGCAGTTCTGAGCACAGT

TGGGTCGAGGCAGTTCTGAGCA TTGGGTCGAGGCAGTTCTGAGC
GTTCTGAGCACAGTACACTGGG 1708902 rs115200817 1708983
rs12523324

HG00110 >hsa-mir-553 TTTAAAACGGTGAGATTTTGTT TTTTAAAACGGTGAGATTTTGT
ATTTTAAAACGGTGAGATTTTG TATTTTAAAACGGTGAGATTTT
TTATTTTAAAACGGTGAGATTT 100746835 .

HG00110 >hsa-mir-553 TTTAAAACGGTGAGATTTTGTT TTTTAAAACGGTGAGATTTTGT
ATTTTAAAACGGTGAGATTTTG TATTTTAAAACGGTGAGATTTT
TTATTTTAAAACGGTGAGATTT 100746848 .

HG00110 >hsa-mir-553 TTTAAAACGGTGAGATTTTGTT TTTTAAAACGGTGAGATTTTGT
ATTTTAAAACGGTGAGATTTTG TATTTTAAAACGGTGAGATTTT
TTATTTTAAAACGGTGAGATTT 100746855 rs112891767

HG00110 >hsa-mir-553 TTTAAAACGGTGAGATTTTGTT TTTTAAAACGGTGAGATTTTGT
ATTTTAAAACGGTGAGATTTTG TATTTTAAAACGGTGAGATTTT
TTATTTTAAAACGGTGAGATTT 100746835 . 100746848 .

HG00110 >hsa-mir-553 TTTAAAACGGTGAGATTTTGTT TTTTAAAACGGTGAGATTTTGT
ATTTTAAAACGGTGAGATTTTG TATTTTAAAACGGTGAGATTTT
TTATTTTAAAACGGTGAGATTT 100746835 . 100746855
rs112891767

HG00110 >hsa-mir-553 TTTAAAACGGTGAGATTTTGTT TTTTAAAACGGTGAGATTTTGT
ATTTTAAAACGGTGAGATTTTG TATTTTAAAACGGTGAGATTTT
TTATTTTAAAACGGTGAGATTT 100746848 . 100746855
rs112891767

HG00110 >hsa-mir-553 TTTAAAACGGTGAGATTTTGTT TTTTAAAACGGTGAGATTTTGT
ATTTTAAAACGGTGAGATTTTG TATTTTAAAACGGTGAGATTTT
TTATTTTAAAACGGTGAGATTT 100746835 . 100746848 .
100746855 rs112891767

HG00110 >hsa-mir-548ad AAAACGACAATGACTTTTGCAC
ACTTTTGCACCAATCTAATAC CTTTTGCACCAATCTAATAC
GCAAAAACGACAATGACTTTTG ACAATGACTTTTGCACCAATCT 35696519
rs62143301

HG00110 >hsa-mir-149 AGGGAGGGAGGGACGGGGGCTG GGGCTGTGCTGGGGCAGCCGGA
GGGACGGGGGCTGTGCTGGGGC GACGGGGGCTGTGCTGGGGCAG
GGGGCTGTGCTGGGGCAGCCGG 241395503 rs2292832

HG00110 >hsa-mir-658 AGGTCGGTTGGTCGGTCGGGAA GTCGGTTGGTCGGTCGGGAACG
TAGGTCGGTTGGTCGGTCGGGA . .

HG00110 >hsa-mir-604 CACAGGCTGCGGAATTCAGGAC ACACAGGCTGCGGAATTCAGGA
ACAGGCTGCGGAATTCAGGACA GGCTGCGGAATTCAGGACAGTG
CTGCGGAATTCAGGACAGTGCA 29833998 rs2368393

HG00110 >hsa-mir-604 CACAGGCTGCGGAATTCAGGAC ACACAGGCTGCGGAATTCAGGA

ACAGGCTGCGGAATTCAGGACA GGCTGCGGAATTCAGGACAGTG
CTGCGGAATTCAGGACAGTGCA 29833998 rs2368393 29834003
rs2368392
HG00110 >hsa-mir-3180-4 CTCCGGATGCCAGTCCCTCATC
GGAGGGTGAAGCCTCCGGATGC CTGGCCTGGTCGCGCTGTGGCT
AGCGGAGGGTGAAGCCTCCGGA GAGCGGAGGGTGAAGCCTCCGG 15248720
rs75000738
HG00110 >hsa-mir-4268CTCTCAGGATGTGATGTCACCT CCTCTCAGGATGTGATGTCACC
GCTCCTCCTCTCAGGATGTGAT CTCCTCCTCTCAGGATGTGATG
TCCTCTCAGGATGTGATGTCAC 220771223 rs4674470

HG00110 >hsa-mir-3151CTGATCCCACACCCCACCTGTC TGATCCCACACCCCACCTGTCA
GATCCCACACCCCACCTGTCAC GGGCATCCCACCTGATCCCACA
TCCCACCTGATCCCACACCCCA 104166902 rs35605502

HG00110 >hsa-mir-3620CTGCATCCCGCACCCAG CCTGCATCCCGCACCCAG
TCCCGCACCCAG ATCCCGCACCCAGCATCCCGCACCCAG 228284991 rs2070960

HG00110 >hsa-mir-412 CTTCACCTGGTTCACTAGCCGT ACCTGGTTCACTAGCCGTCCGT
TGTACTTCACCTGGTTCACTAG CTGGTTCACTAGCCGTCCGTAT
GTACTTCACCTGGTTCACTAGC 101531849 .

HG00110 >hsa-mir-658 TAGGTCGGTTGGTCGGTCGGGA .

HG00110 >hsa-mir-658 GTCCGTTGGTCGGTCGGGAACG G
TCCGTTGGTCGGTCGGGAACGA .

HG00110 >hsa-mir-320eGAAAAGCTGGGTTGAGAAGGT AAAAGCTGGGTTGAGAAGGT
GGAAAAGCTGGGTTGAGAAGGT GGGAAAAGCTGGGTTGAGAAGG rs10423365

HG00110 >hsa-mir-1343GCCCCTCCTGGGGCCCGCACTC CCCCTCCTGGGGCCCGCACTCT
GGGGCCCGCACTCTCGCTCTGG CCCTCCTGGGGCCCGCACTCTC
TGGGGCCCGCACTCTCGCTCTG 34963416 rs2986407

HG00110 >hsa-mir-3180-4 GGAGGGTGAAGCCTCCGGATGC
GGTGAAGCCTCCGGATGCCAGT AGCGGAGGGTGAAGCCTCCGGA
GCGGAGGGTGAAGCCTCCGGAT GCCTGGTCGCGCTGTGGCGAAG 15248798
rs183853838
HG00110 >hsa-mir-3180-4 GGAGGGTGAAGCCTCCGGATGC
GGTGAAGCCTCCGGATGCCAGT AGCGGAGGGTGAAGCCTCCGGA
GCGGAGGGTGAAGCCTCCGGAT CTGGCCTGGTCGCGCTGTGGCT 15248720
rs75000738 15248798 rs183853838
HG00110 >hsa-mir-1227GGCATTTGACCCCGTGCCACCC AGGCATTTGACCCCGTGCCACC
AGGCATTTGACCCCGTGCCACC TGACCCCGTGCCACCCTTTTCC
ATTTGACCCCGTGCCACCCTTT 2234093 rs190788838

HG00110 >hsa-mir-580 TATTTGAGAATGATGAATCATT TGAATCATTAGGTTCCGGTCAG

ATGAATCATTAGGTTCCGGTCA TTTGAGAATGATGAATCATTAG
GAGAATGATGAATCATTAGGTT 36148057 rs115089112

HG00110 >hsa-mir-3118-1 TGAAAATTCTTCTAGTGTG ATGAAAATTCTTCTAGTGTG
TGCATTATGAAAATTCTTCTAG TTATGAAAATTCTTCTAGTGTG
ATTATGAAAATTCTTCTAGTGT 142667330 rs76132421

HG00110 >hsa-mir-320c-1 AAAAGCTGGGTTGAGAGGGTAG
AGCTGGGTTGAGAGGGTAGGAA CTGGGTTGAGAGGGTAGGAAAA
AGGGTAGGAAAAAAATGATGTA 19263542 .

HG00110 >hsa-mir-202 AAAGAGGTATAGGGCATGGGAA AAGAGGTATAGGGCATGGGAAA
GGGAAAACGGGGCGGTCGGGTC TAAAGAGGTATAGGGCATGGGA 135061112
rs12355840
HG00110 >hsa-mir-520hAAAGTGCTTCCCTTTAGAGTTA rs148716001 74

HG00110 >hsa-mir-944 AAATTATTGTACATCGGATGAG GTACATCGGATGAGCTGTGTCT
GAAATTATTGTACATCGGATGA CATCGGATGAGCTGTGTCTGGG 189547735
rs75715827
HG00110 >hsa-mir-548ap AACAAAAACCACAATTACTTTT
CAAAAACCACAATTACTTTTTA CAATTACTTTTTACTGACCTAA rs4414449

HG00110 >hsa-mir-548ap AACAAAAACCACAATTACTTTT
CAAAAACCACAATTACTTTTTA TTACTTTTTACTGACCTAAAGA rs4577031

HG00110 >hsa-mir-548ap AACAAAAACCACAATTACTTTT
CAAAAACCACAATTACTTTTTA CAATTACTTTTTACTGACCTAA rs4414449
86368959 rs4577031
HG00110 >hsa-mir-548ad AACGACAATGACTTTTGCACCA
GGCAAAAACGACAATGACTTTT TGGCAAAAACGACAATGACTTT
AAAAACGACAATGACTTTTGCA 35696519 rs62143301

HG00110 >hsa-mir-3938AATTCCCTTGTAGATAACCCGG ATAACCCGGTGGTCAGGTTGGA
TAGATAACCCGGTGGTCAGGTT CCTTGTAGATAACCCGGTGGTC 55886574
rs59684995
HG00110 >hsa-mir-449cACAGTTGCTAGTTGCACTCCTC AACAGTTGCTAGTTGCACTCCT
GTTGCTAGTTGCACTCCTCTCT GTTGCACTCCTCTCTGTTGCAT 54468166
rs75661995
HG00110 >hsa-mir-3118-1 ACTGCATTATGAAAATTCTTCT
ATTATGAAAATTCTTCTAGTGT GCATTATGAAAATTCTTCTAGT
CTGCATTATGAAAATTCTTCTA 142667330 rs76132421

HG00110 >hsa-mir-605 AGAGAAGGCACTATGAGATTTA GGCACTATGAGATTTAGAACCA
CAGAGAAGGCACTATGAGATTT GAGAAGGCACTATGAGATTTAG 53059406
rs2043556
HG00110 >hsa-mir-3180-4 AGCGGAGGGTGAAGCCTCCGGA
GGAGGGTGAAGCCTCCGGATGC GAGCGGAGGGTGAAGCCTCCGG

GCGGAGGGTGAAGCCTCCGGAT 15248798 rs183853838

HG00110 >hsa-mir-3180-4 AGCGGAGGGTGAAGCCTCCGGA
GGAGGGTGAAGCCTCCGGATGC GAGCGGAGGGTGAAGCCTCCGG
GCGGAGGGTGAAGCCTCCGGAT 15248720 rs75000738 15248798
rs183853838
HG00110 >hsa-mir-30d AGCTTTCAGTCAGATGTTTGCT GGCTAAGCTTTCAGTCAGATGT
GCTAAGCTTTCAGTCAGATGTT TTCAGTCAGATGTTTGCTGCTA 135817150
.
HG00110 >hsa-mir-519a-2 AGGAAAGTGCATCCTTTTAGAG
AGTGCATCCTTTTAGAGGGTTA GGAAAGTGCATCCTTTTAGAGG
GAAAGGAAAGTGCATCCTTTTA 54265670 .

HG00110 >hsa-mir-630 AGTATTCTGTACCAGGGAAGGT ACCTAGTATTCTGTACCAGGGA
CCAGGGAAGGTAGTTCTTAACT GGAAGGTAGTTCTTAACTATGT 72879653
rs113971639
HG00110 >hsa-mir-513cATAAATTTCACCTTTCTGAGAA TTCTGAGAAGAGTAATGTACAG
CCTTTCTGAGAAGAGTAATGTA TTTCACCTTTCTGAGAAGAGTA 146271303
rs145416750
HG00110 >hsa-mir-323bATACACGGTCGACCTCTTTTCG TACACGGTCGACCTCTTTTCGG
ACACGGTCGACCTCTTTTCGGT rs56103835

HG00110 >hsa-mir-4293CACACCAGCCTGACAGGAACAG CACCAGCCTGACAGGAACAGCC
TCACACCAGCCTGACAGGAACA CAGCCTGACAGGAACAGCCTGT 14425204
rs12780876
HG00110 >hsa-mir-604 CACAGGCTGCGGAATTCAGGAC TGACACAGGCTGCGGAATTCAG
GACACAGGCTGCGGAATTCAGG rs2368392

HG00110 >hsa-mir-515-1 CAGAGTGCCTTCTTTTGGAGCA
GAGTGCCTTCTTTTGGAGCATT TGCCTTCTTTTGGAGCATTACT
GTGCCTTCTTTTGGAGCATTAC 54182326 rs374576826

HG00110 >hsa-mir-4265CAGCTGTGGGCTCAACTCTGGG ATCTCTGCAGCTGTGGGCTCAA
GATCTCTGCAGCTGTGGGCTCA CTGCAGCTGTGGGCTCAACTCT 109757963
rs4676066
HG00110 >hsa-mir-1227CATTTGACCCCGTGCCACCCTT ATTTGACCCCGTGCCACCCTTT
AGGCATTTGACCCCGTGCCACC GACCCCGTGCCACCCTTTTCCC 2234093
rs190788838
HG00110 >hsa-mir-3151CCACCTGATCCCACACCCCACC CACCTGATCCCACACCCCACCT
CCCACCTGATCCCACACCCCAC TGATCCCACACCCCACCTGTCA 104166902
rs35605502
HG00110 >hsa-mir-1343CCCCTCCTGGGGCCCGCACTCT CCCTCCTGGGGCCCGCACTCTC
CCTGGGGCCCGCACTCTCGCTC TGGGGCCCGCACTCTCGCTCTG 34963416
rs2986407
HG00110 >hsa-mir-1343CCCTCCTGGGGCGCGCACTCTC CTCCTGGGGCGCGCACTCTCGC
GGGGCGCGCACTCTCGCTCTGG TGGGGCGCGCACTCTCGCTCTG 34963445
.

HG00110 >hsa-mir-3679CCCTTCCCCCCAGTAATCTTCA CCTTCCCCCCAGTAATCTTCAT TCCCCCCAGTAATCTTCATCAT CTTCCCCCCAGTAATCTTCATC 134884700 rs6430498
HG00110 >hsa-mir-4254CCTGGAGATACTCCACCATCTC AGATACTCCACCATCTCCCCCA GGAGATACTCCACCATCTCCCC rs12731294

HG00110 >hsa-mir-3620CTCACCCTGCATCCCGCACCCA CCTCACCCTGCATCCCGCACCC CTCACCCTGCATCCCGCACCCA rs2070960

HG00110 >hsa-mir-3117CTCATATAGTGCCAGGTGTTTT GACTCATATAGTGCCAGGTGTT TCATATAGTGCCAGGTGTTTTG AGACTCATATAGTGCCAGGTGT 67094171 rs12402181
HG00110 >hsa-mir-1343CTCCTGGGGCGCGCACTCTCGC CCCTCCTGGGGCGCGCACTCTC GGGGCGCGCACTCTCGCTCTGG TGGGGCGCGCACTCTCGCTCTG 34963416 rs2986407 34963445 .
HG00110 >hsa-mir-888 CTCTTTGGGTGAAGGAAGGCTC CTGACACCTCTTTGGGTGAAGG GACTGACACCTCTTTGGGTGAA CCTCTTTGGGTGAAGGAAGGCT 145076355 rs143634721
HG00110 >hsa-mir-888 CTCTTTGGGTGAAGGAAGGCTC GACTGACACCTCTTTGGGTGAA CTGACACCTCTTTGGGTGAAGG TGACACCTCTTTGGGTGAAGGA 145076356 rs112850228
HG00110 >hsa-mir-888 CTCTTTGGGTGAAGGAAGGCTC CTGACACCTCTTTGGGTGAAGG GACTGACACCTCTTTGGGTGAA CCTCTTTGGGTGAAGGAAGGCT 145076302 rs5965660 145076355 rs143634721
HG00110 >hsa-mir-888 CTCTTTGGGTGAAGGAAGGCTC GACTGACACCTCTTTGGGTGAA CTGACACCTCTTTGGGTGAAGG CCTCTTTGGGTGAAGGAAGGCT 145076302 rs5965660 145076356 rs112850228
HG00110 >hsa-mir-2117CTGTTCTCTTTGCCAAGGACAG GCTGTTCTCTTTGCCAAGGACA TCTCTTTGCCAAGGACAGATCT TGTTCTCTTTGCCAAGGACAGA 41522213 rs7207008
HG00110 >hsa-mir-888 GACACCTCTTTGGGTGAAGGAA ACTGACACCTCTTTGGGTGAAG CTGACACCTCTTTGGGTGAAGG GACTGACACCTCTTTGGGTGAA 145076355 rs143634721 145076356 rs112850228
HG00110 >hsa-mir-888 GACACCTCTTTGGGTGAAGGAA ACTGACACCTCTTTGGGTGAAG CTGACACCTCTTTGGGTGAAGG GACTGACACCTCTTTGGGTGAA 145076302 rs5965660 145076355 rs143634721 145076356
HG00110 >hsa-mir-4274GACCCAGCAGTCCCTCCCCCTG CCCAGCAGTCCCTCCCCCTGCA TGACCCAGCAGTCCCTCCCCCT TCAGGTGACCCAGCAGTCCCTC 7461769 rs12512664
HG00110 >hsa-mir-888 GACTGACACCTCTTTGGGTGAA GACACCTCTTTGGGTGAAGGAA TGACACCTCTTTGGGTGAAGGA CACCTCTTTGGGTGAAGGAAGG 145076302 rs5965660
HG00110 >hsa-mir-629 GAGGTTCTCCCAACGTAAGCCC AGGTTCTCCCAACGTAAGCCCA TCTCCCAACGTAAGCCCAGCCC CAGGAGGTTCTCCCAACGTAAG 70371761 rs377691713
HG00110 >hsa-mir-629 GAGGTTCTCCCAACGTAAGCCC AGGAGGTTCTCCCAACGTAAGC GGAGGTTCTCCCAACGTAAGCC TCTCCCAACGTAAGCCCAGCCC 70371794

.
HG00110 >hsa-mir-629 GAGGTTCTCCCAACGTAAGCCC AGGTTCTCCCAACGTAAGCCCA TCTCCCAACGTAAGCCCAGCCC CCCAACGTAAGCCCAGCCCCTC 70371761 rs377691713 70371794 .
HG00110 >hsa-mir-3130-1 GCACCAGAGACTGGGTAAGACA GAGACTGGGTAAGACATGACAA CCAGAGACTGGGTAAGACATGA TGCACCAGAGACTGGGTAAGAC 207647981 rs2241347

HG00110 >hsa-mir-3130-2 GCACCGGAGACTGGGTAAGACA CTGCACCGGAGACTGGGTAAGA CCGGAGACTGGGTAAGACATGA TGCACCGGAGACTGGGTAAGAC 207647981 rs2241347

HG00110 >hsa-mir-222 GCAGCTACATCTGGCTACTGGG TACTGGGTCTCTGATGGCATCT GCTACTGGGTCTCTGATGGCAT CTGGCTACTGGGTCTCTGATGG 45606504 rs191727254
HG00110 >hsa-mir-3180-4 GCGGAGGGTGAAGCCTCCGGAT CGCTGGCCTGGTCGCGCTGTGG TCGCTGGCCTGGTCGCGCTGTG AAGCCTCCGGATGCCAGTCCCT 15248720 rs75000738

HG00110 >hsa-mir-675 GCTGTATGCCCTCACCACTCAG GTATGCCCTCACCACTCAGCCC GCCCTCACCACTCAGCCCCTGG TGTATGCCCTCACCACTCAGCC 2018002 rs376650475
HG00110 >hsa-mir-3176GGGACTGGCCTGGGACTACCGG GCCTGGGACTACCGGGGGTGGC ACTGGCCTGGGACTACCGGGGG CAGGGACTGGCCTGGGACTACC 593277 rs8054514
HG00110 >hsa-mir-149 GGGAGGGAGGGACGGGGGCTGT GGAGGGACGGGGGCTGTGCTGG AGGGACGGGGGCTGTGCTGGGG GACGGGGGCTGTGCTGGGGCAG 241395503 rs2292832
HG00110 >hsa-mir-1244-3 GTACTATAAGTAGTTGGTTTGT TAAGTAGTTGGTTTGTATGAGA GTACTATAAGTAGTTGGTTTGT ACTATAAGTAGTTGGTTTGTAT 9392072 rs367702648

HG00110 >hsa-mir-1197GTAGGACACATGGTCTACTTCT ACACATGGTCTACTTCTTCTCA ACATGGTCTACTTCTTCTCAAT TAGGACACATGGTCTACTTCTT 101491923 rs141611518
HG00110 >hsa-mir-877 GTCCTCTTCTCCCTTCTCCCAG TGGGACCCTCAGACGTGTGTCC AGACGTGTGTCCTCTTCTCCCT CTGGGACCCTCAGACGTGTGTC 30552187 rs372113020
HG00110 >hsa-mir-606 TAAACTACTGAAAATCAAAGAT CCATCATAGTAAACTACTGAAA ACTACTGAAAATCAAAGATACA CCCATCATAGTAAACTACTGAA 77312301 .
HG00110 >hsa-mir-3936TAAGGGGTGTATGGCAGATGCA CACCCGACAGATGCACTTGGCA GATGCACCCGACAGATGCACTT TGTATGGCAGATGCACCCGACA 131701279 rs367805
HG00110 >hsa-mir-300 TATACAAGGGCAGACTCTCTCT TGATTATACAAGGGCAGACTCT ATTATACAAGGGCAGACTCTCT rs12894467

HG00110 >hsa-mir-499aTCACAGCAAGTCTGTGCTGCTT ACAGCAAGTCTGTGCTGCTTCC
CGTCACAGCAAGTCTGTGCTGC TCCCTACGCTGCCTGGGCAGGG 33578251
rs3746444
HG00110 >hsa-mir-412 TCACCTGGTTCACTAGCCGTCC TCACCTGGTTCACTAGCCGTCC
ATGTACTTCACCTGGTTCACTA CTTCACCTGGTTCACTAGCCGT 101531849
.
HG00110 >hsa-mir-499bTCACTGCAAGTCTTAACAGCCG rs3746444 73

HG00110 >hsa-mir-4312TCAGGCCTTGTTCCTGTCCCCA CCTTGTTCCTGTCCCCATTCCT
CTTGTTCCTGTCCCCATTCCTC AGGCCTTGTTCCTGTCCCCATT 69094259
rs79711957
HG00110 >hsa-mir-532 TCCCACACCCAAGGCTTGCAGA CTCCCACACCCAAGGCTTGCAG
CCTCCCACACCCAAGGCTTGCA CACCCAAGGCTTGCAGAAGAGC 49767832
rs456615
HG00110 >hsa-mir-532 TCCCACACCCAAGGCTTGCAGA CTCCCACACCCAAGGCTTGCAG
CCTCCCACACCCAAGGCTTGCA CACCCAAGGCTTGCAGAAGAGC 49767835
rs456617
HG00110 >hsa-mir-532 TCCCACACCCAAGGCTTGCAGA CTCCCACACCCAAGGCTTGCAG
CCTCCCACACCCAAGGCTTGCA CACCCAAGGCTTGCAGAAGAGC 49767832
rs456615 49767835 rs456617
HG00110 >hsa-mir-4268TCCTCCTCTCAGGATGTGATGT TCCTCCTCTCAGGATGTGATGT
CTCCTCTCAGGATGTGATGTCA CTCCTCCTCTCAGGATGTGATG 220771223
rs4674470
HG00110 >hsa-mir-1304TCTCACTGTAGCATCGAACCCC GAACCCCTGGGCTCAAGTGATT
CTCACTGTAGCATCGAACCCCT CGAACCCCTGGGCTCAAGTGAT 93466866
rs2155248
HG00110 >hsa-mir-595 TGAAGTGTGCCGTGGTGTGTCT GTGTGTCTGGAGGAAGCGCCTG
GCCGTGGTGTGTCTGGAGGAAG rs4909237

HG00110 >hsa-mir-604 TGACACAGGCTGCGGAATTCAG CACAGGCTGCGGAATTCAGGAC
GACACAGGCTGCGGAATTCAGG TTTCTGACACAGGCTGCGGAAT 29833998
rs2368393
HG00110 >hsa-mir-604 TGACACAGGCTGCGGAATTCAG GACACAGGCTGCGGAATTCAGG
CACAGGCTGCGGAATTCAGGAC TTTCTGACACAGGCTGCGGAAT 29833998
rs2368393 29834003 rs2368392
HG00110 >hsa-mir-570 TGCGAAAACAGCAATTACCTTT GAAAACAGCAATTACCTTTGCA
ACAGCAATTACCTTTGCACCAA CGAAAACAGCAATTACCTTTGC 195426305
rs9860655
HG00110 >hsa-mir-1273h TGCTGCAGACTCGACCTCCCAG
TGCAGACTCGACCTCCCAGGCT CTGCAGACTCGACCTCCCAGGC
AGACTCGACCTCCCAGGCTTAA 24214486 .

HG00110 >hsa-mir-590 TGTAATTTTATGTATAAGCTAG AATCTGTAATTTTATGTATAAG
GTATAAGCTAGTCTCTGATTGA TTTTATGTATAAGCTAGTCTCT 73605546
rs189727189
HG00110 >hsa-mir-585 TTGGGCGTATCTGTATGCTAGG TGGGCGTATCTGTATGCTAGGG
TATCTGTATGCTAGGGCTGCCG GCGTATCTGTATGCTAGGGCTG 168690612

rs62376934
HG00110 >hsa-mir-1303TTTAGAGACGGGGTCTTGCTCT TAGAGACGGGGTCTTGCTCTGT
TTAGAGACGGGGTCTTGCTCTG ACGGGGTCTTGCTCTGTTGCCA 154065383
rs75538180
HG00110 >hsa-mir-580 TTTGAGAATGATGAATCATTAG GATGAATCATTAGGTTCCGGTC
AATGATGAATCATTAGGTTCCG AGAATGATGAATCATTAGGTTC 36148057
rs115089112
HG00110 >hsa-mir-133bTTTGGTCCCCTTCAACCAGCTA TGGTCCCCTTCAACCAGCTACA
CCTTCAACCAGCTACAGCAGGG AGAGGTTTGGTCCCCTTCAACC 52013832
rs374103744
HG00111 >hsa-mir-648 AGCGTGCAGGGCACTGGTGGGG CGTGCAGGGCACTGGTGGGGGC
GGGCACTGGTGGGGGCCGGGGC GCGTGCAGGGCACTGGTGGGGG
CTCCAAGCGTGCAGGGCACTGG 18463709 .

HG00111 >hsa-mir-1255b-1 ATGAGCAAAGAAAGTGGTTTCT
GATGAGCAAAGAAAGTGGTTTC TATGGATGAGCAAAGAAAGTGG
GGATGAGCAAAGAAAGTGGTTT ATGGATGAGCAAAGAAAGTGGT 36428048
rs6841938
HG00111 >hsa-mir-1273h CCTGGGAGGTCAAGGCTGTAGT
TGGGAGGTCAAGGCTGTAGTGT GCCTGGGAGGTCAAGGCTGTAG
ATTGCTTGAGCCTGGGAGGTCA TTGAGCCTGGGAGGTCAAGGCT 24214486 .

HG00111 >hsa-mir-3683CTGCGACATTGGAAGTAGTATC CCCCTGCGACATTGGAAGTAGT
TGTACACCCCCTGCGACATTGG GTGTACACCCCCTGCGACATTG
CCTGCGACATTGGAAGTAGTAT 7106636 rs6977967

HG00111 >hsa-mir-548ak GCAAAAGTAACTGCGGTTTTTG
TGCAAAAGTAACTGCGGTTTTT CAAAAGTAACTGCGGTTTTTGA
GTGCAAAAGTAACTGCGGTTTT rs7070684

HG00111 >hsa-mir-3196GCGGGGCGGCAGGGGCCTCCCC GGCGGGGCGGCAGGGGCCTCCC
GGGCGGGGCGGCAGGGGCCTCC TGGGGGCGGGGCGGCAGGGGCC
GGGGCGGGGCGGCAGGGGCCTC 61870167 rs744591

HG00111 >hsa-mir-608 GGCCAAGGTGGGCCAGGGGTGG AAGGTGGGCCAGGGGTGGTGTT
GGGGTGGTGTTGGGACAGCTGC TGGTGTTGGGACAGCTGCGTTT
GGTGGGCCAGGGGTGGTGTTGG 102734778 rs4919510

HG00111 >hsa-mir-3199-1 TGCCTTAGGAGAAAGTTTCTGG
CCGGGACTGCCTTAGGAGAAAG CTTAGGAGAAAGTTTCTGGAAG
GCCTTAGGAGAAAGTTTCTGGA TGACTCCCGGGACTGCCTTAGG 28316591
rs78805657
HG00111 >hsa-mir-744 TTGGGCAAGGTGCGGGGCTAGG TGGGCAAGGTGCGGGGCTAGGG
CGGGGCTAGGGCTAACAGCAGT GTGCGGGGCTAGGGCTAACAGC
CTAGGGCTAACAGCAGTCTTAC 11985275 .

HG00111 >hsa-mir-553 TTTTAAAACGGTGAGATTTTGT TATTTTAAAACGGTGAGATTTT

TTTTATTTTAAAACGGTGAGAT TTTAAAACGGTGAGATTTTGTT
ATTTTATTTTAAAACGGTGAGA 100746848 .

HG00111 >hsa-mir-553 TTTTAAAACGGTGAGATTTTGT TTTTATTTTAAAACGGTGAGAT
TATTTTAAAACGGTGAGATTTT TTTAAAACGGTGAGATTTTGTT
ATTTTATTTTAAAACGGTGAGA 100746848 . 100746855
rs112891767
HG00111 >hsa-mir-553 TTTTATTTTAAAACGGTGAGAT AATTTTATTTTAAAACGGTGAG
TTTTAAAACGGTGAGATTTTGT TTATTTTAAAACGGTGAGATTT
TTTAAAACGGTGAGATTTTGTT 100746855 rs112891767

HG00111 >hsa-mir-1283-1 AAAGGAAAGCGCTTTCTGTTGT
ACAAAGGAAAGCGCTTTCTGTT AGTCTACAAAGGAAAGCGCTTT
CAAAGGAAAGCGCTTTCTGTTG CTACAAAGGAAAGCGCTTTCTG 54191743
rs57111412
HG00111 >hsa-mir-486-2 ACTGAGCTGCCCCGAGCTGGGC
CTGAGCTGCCCCGAGCTGGGCA CTGTACTGAGCTGCCCCGAGCT
CCTGTACTGAGCTGCCCCGAGC GTACTGAGCTGCCCCGAGCTGG 41518007 .

HG00111 >hsa-mir-1269b AGCCATGCTACGGGCTTCTCTG
ACTGAGCCATGCTACGGGCTTC AGGTTTCTGGACTGAGCCATGC
TGAGGTTTCTGGACTGAGCCAT TTTCTGGACTGAGCCATGCTAC 12820632
rs12451747
HG00111 >hsa-mir-3135b AGCGAGTGCAGTGGTGCAGTCA
AGGCTGGAGCGAGTGCAGTGGT CTGGAGCGAGTGCAGTGGTGCA
CAGGCTGGAGCGAGTGCAGTGG CCAGGCTGGAGCGAGTGCAGTG 32717702
rs4285314
HG00111 >hsa-mir-548ap AGTAATTGCAGTCTTTGTCATT
AAGTAATTGCAGTCTTTGTCAT AAAGTAATTGCAGTCTTTGTCA
AAAAGTAATTGCAGTCTTTGTC CAAAAGTAATTGCAGTCTTTGT 86368898
rs4414449
HG00111 >hsa-mir-548ap AGTAATTGCAGTCTTTGTCATT
AAGTAATTGCAGTCTTTGTCAT AAAGTAATTGCAGTCTTTGTCA
AAAAGTAATTGCAGTCTTTGTC CAAAAGTAATTGCAGTCTTTGT 86368898
rs4414449 86368959 rs4577031
HG00111 >hsa-mir-548aj-2 AGTAATTGCAGTTTTTGCCATT
AAGTAATTGCAGTTTTTGCCAT AAAGTAATTGCAGTTTTTGCCA
TGCAAAAGTAATTGCAGTTTTT AAAAGTAATTGCAGTTTTTGCC 37883200
rs73463468
HG00111 >hsa-mir-548ap AGTAATTGCGGTCTTTGTCATT
AAGTAATTGCGGTCTTTGTCAT AAAGTAATTGCGGTCTTTGTCA
AAAAGTAATTGCGGTCTTTGTC CAAAAGTAATTGCGGTCTTTGT 86368959
rs4577031
HG00111 >hsa-mir-196a-2 AGTTTCATGTTGTTGGGATTGA
AGGTAGTTTCATGTTGTTGGGA TAGTTTCATGTTGTTGGGATTG
GTAGTTTCATGTTGTTGGGATT GGTAGTTTCATGTTGTTGGGAT 54385599
rs11614913

HG00111 >hsa-mir-500bATCCTTGCTACCTGGGTGAGAG CTTGCTACCTGGGTGAGAGTGC AATCCTTGCTACCTGGGTGAGA CCTTGCTACCTGGGTGAGAGTG TCCTTGCTACCTGGGTGAGAGT 49775351 rs151318590

HG00111 >hsa-mir-744 GGGCTAACAGCAGTCTTACTGA GCTAGGGCTAACAGCAGTCTTA CTAGGGCTAACAGCAGTCTTAC GGCTAGGGCTAACAGCAGTCTT TAGGGCTAACAGCAGTCTTACT 11985275 .

HG00111 >hsa-mir-548h-3 GTAATCGTGGTTTTTGTCATTG TGCAAAAGTAATCGTGGTTTTT AGTAATCGTGGTTTTTGTCATT AAGTAATCGTGGTTTTTGTCAT TGGTGCAAAAGTAATCGTGGTT 13446924 rs9913045

HG00111 >hsa-mir-573 GTGTAACTGATCAGGATCTACT TGTGTAACTGATCAGGATCTAC GATGTGTAACTGATCAGGATCT TGATGTGTAACTGATCAGGATC GTGATGTGTAACTGATCAGGAT 24521902 rs76014664

HG00111 >hsa-mir-573 GTGTAACTGATCAGGATCTACT TGTGTAACTGATCAGGATCTAC GATGTGTAACTGATCAGGATCT TGATGTGTAACTGATCAGGATC GTGATGTGTAACTGATCAGGAT 24521904 rs78830737

HG00111 >hsa-mir-573 GTGTAACTGATCAGGATCTACT TGTGTAACTGATCAGGATCTAC GATGTGTAACTGATCAGGATCT TGATGTGTAACTGATCAGGATC GTGATGTGTAACTGATCAGGAT 24521902 rs76014664 24521904 rs78830737

HG00111 >hsa-mir-3117TAAAGGGCCAGACACTATACGA GGGCCAGACACTATACGAGTCA GCCAGACACTATACGAGTCATA GGCCAGACACTATACGAGTCAT CCCTAAAGGGCCAGACACTATA 67094171 rs12402181

HG00111 >hsa-mir-3683TACACCCCCTGCGACATTGGAA TGTACACCCCCTGCGACATTGG GGGTGTACACCCCCTGCGACAT GGTGTACACCCCCTGCGACATT GTGTACACCCCCTGCGACATTG 7106636 rs6977967

HG00111 >hsa-mir-629 TACGTTGGGAGAACTTTTACGG TTACGTTGGGAGAACTTTTACG GTTTACGTTGGGAGAACTTTTA TTTACGTTGGGAGAACTTTTAC GGTTTACGTTGGGAGAACTTTT 70371794 .

HG00111 >hsa-mir-629 TACGTTGGGAGAACTTTTATGG TTACGTTGGGAGAACTTTTATG TTTACGTTGGGAGAACTTTTAT TGGGTTTACGTTGGGAGAACTT GTTTACGTTGGGAGAACTTTTA 70371761 rs377691713

HG00111 >hsa-mir-629 TACGTTGGGAGAACTTTTATGG TTACGTTGGGAGAACTTTTATG TTTACGTTGGGAGAACTTTTAT TGGGTTTACGTTGGGAGAACTT GTTTACGTTGGGAGAACTTTTA 70371761 rs377691713 70371794 .

HG00111 >hsa-mir-888 TACTCAAAAAGCTTTCAGTCAC TCTACTCAAAAAGCTTTCAGTC TGCTCTACTCAAAAAGCTTTCA GCTCTACTCAAAAAGCTTTCAG GGCAGTGCTCTACTCAAAAAGC 145076355 rs143634721

HG00111 >hsa-mir-888 TACTCAAAAAGCTTTCAGTCAC TCTACTCAAAAAGCTTTCAGTC TGCTCTACTCAAAAAGCTTTCA GCTCTACTCAAAAAGCTTTCAG GTGCTCTACTCAAAAAGCTTTC 145076302 rs5965660 145076355 rs143634721
HG00111 >hsa-mir-520gTAGAGGAAGCACTTTCTGTTTG TCTAGAGGAAGCACTTTCTGTT TGACCCTCTAGAGGAAGCACTT TGTGACCCTCTAGAGGAAGCAC TCCCATGCTGTGACCCTCTAGA 54225460 rs375062679

HG00111 >hsa-mir-3199-1 TAGGAGAAAGTTTCTGGAAGTT TTAGGAGAAAGTTTCTGGAAGT TGCCTTAGGAGAAAGTTTCTGG TCCCGGGACTGCCTTAGGAGAA TGACTCCCGGGACTGCCTTAGG 28316591 rs78805657
HG00111 >hsa-mir-3199-2 TAGGAGAAAGTTTCTGGAATGT TTAGGAGAAAGTTTCTGGAATG TGCCTTAGGAGAAAGTTTCTGG TCCCAGGGACTGCCTTAGGAGA TGACTCCCAGGGACTGCCTTAG 28316591 rs78805657
HG00111 >hsa-mir-3199-2 TAGGAGAAAGTTTCTGGAATGT TTAGGAGAAAGTTTCTGGAATG TGCCTTAGGAGAAAGTTTCTGG TCCCAGGGACTGCCTTAGGAGA TGACTCCCAGGGACTGCCTTAG 28316591 rs80166589
HG00111 >hsa-mir-3199-2 TAGGAGAAAGTTTCTGGAATGT TTAGGAGAAAGTTTCTGGAATG TGCCTTAGGAGAAAGTTTCTGG TCCCAGGGACTGCCTTAGGAGA TGACTCCCAGGGACTGCCTTAG 28316591 rs78805657 28316591 rs80166589
HG00111 >hsa-mir-449cTAGGCAGTGTATTGCTAGCGGC TCAGATAGGCAGTGTATTGCTA TGTCAGATAGGCAGTGTATTGC TGTGTCAGATAGGCAGTGTATT TGGGATGTGTCAGATAGGCAGT 54468166 rs75661995

HG00111 >hsa-mir-590 TATACATAAAAGTGCAGTATGG TTATACATAAAAGTGCAGTATG AGCTTATACATAAAAGTGCAGT CTTATACATAAAAGTGCAGTAT TGAGCTTATACATAAAAGTGCA 73605551 rs192756765

HG00111 >hsa-mir-590 TATACATAAAAGTGCAGTATGG TTATACATAAAAGTGCAGTATG TTTATACATAAAAGTGCAGTAT AGTTTATACATAAAAGTGCAGT TGAGTTTATACATAAAAGTGCA 73605546 rs189727189 73605551 rs192756765
HG00111 >hsa-mir-590 TATTCATAAAAGTGCAGTATGG TTATTCATAAAAGTGCAGTATG TTTATTCATAAAAGTGCAGTAT AGTTTATTCATAAAAGTGCAGT TGAGTTTATTCATAAAAGTGCA 73605546 rs189727189

HG00111 >hsa-mir-3922TCAAGGCCAGAGGTCCCACAAC TCAAGTCAAGGCCAGAGGTCCC GCCAGAGGTCCCACAACAGGGC GGCCAGAGGTCCCACAACAGGG GTCAAGGCCAGAGGTCCCACAA 104985443 rs61938575

HG00111 >hsa-mir-222 TCAGTAGCCAGTGTAGATCCTG TGGCTCAGTAGCCAGTGTAGAT TTGGCTCAGTAGCCAGTGTAGA TCATTGGCTCAGTAGCCAGTGT

TACCCTCATTGGCTCAGTAGCC 45606504 rs191727254

HG00111 >hsa-mir-515-1 TCCAAAAGAAAGCACTTTCTGT TCTCCAAAAGAAAGCACTTTCT TTCTCCAAAAGAAAGCACTTTC TCATTCTCCAAAAGAAAGCACT TGCAGTCATTCTCCAAAAGAAA 54182326 rs374576826

HG00111 >hsa-mir-663aTCCCAGGCGGGGCGCCGCGGGA TCCGGCGTCCCAGGCGGGGCGC TTCCGGCGTCCCAGGCGGGGCG GCGCCGCGGGACCTCCCTCGTG GGCGCCGCGGGACCTCCCTCGT 26188880 .

HG00111 >hsa-mir-149 TCCGTGTCTTCACTCCCGTGCT TGGCTCCGTGTCTTCACTCCCG TCTGGCTCCGTGTCTTCACTCC CCGTGTCTTCACTCCCGTGCTT AGCTCTGGCTCCGTGTCTTCAC 241395500 rs71428439

HG00111 >hsa-mir-149 TCCGTGTCTTCACTCCCGTGCT TGGCTCCGTGTCTTCACTCCCG TCTGGCTCCGTGTCTTCACTCC CCGTGTCTTCACTCCCGTGCTT AGCTCTGGCTCCGTGTCTTCAC 241395503 rs2292832

HG00111 >hsa-mir-149 TCCGTGTCTTCACTCCCGTGCT TGGCTCCGTGTCTTCACTCCCG TCTGGCTCCGTGTCTTCACTCC CCGTGTCTTCACTCCCGTGCTT AGCTCTGGCTCCGTGTCTTCAC 241395500 rs71428439 241395503 rs2292832

HG00111 >hsa-mir-618 TCCTTCTGAGTGTAATTACGTA TGTCCTTCTGAGTGTAATTACG TTGTCCTTCTGAGTGTAATTAC TACTTGTCCTTCTGAGTGTAAT GTCCTTCTGAGTGTAATTACGT 81329536 rs2682818

HG00111 >hsa-mir-3183TCGGAGTCGCTCGGAGCAGCCA TCTCGGAGTCGCTCGGAGCAGC TCTCTCGGAGTCGCTCGGAGCA TCTGCCCTGCCTCTCTCGGAGT TGCCCTGCCTCTCTCGGAGTCG 925764 rs2663345

HG00111 >hsa-mir-520cTCTCAGGCTGTCGTCCTCTAGA TCAGGCTGTCGTCCTCTAGAGG TGTCGTCCTCTAGAGGGAAGCA TCGTCCTCTAGAGGGAAGCACT TCCTCTAGAGGGAAGCACTTTC 54210774 .

HG00111 >hsa-mir-635 TGAAACAATGTCCATTAGGCTT GAAACAATGTCCATTAGGCTTT ACAATGTCCATTAGGCTTTGTT AACAATGTCCATTAGGCTTTGT CTGAAACAATGTCCATTAGGCT 66420592 rs77279010

HG00111 >hsa-mir-1255a TGAGCAAAGAAAGTAGATTTTT GCAAAGAAAGTAGATTTTTTAG TCAAGGATGAGCAAAGAAAGTA GAGCAAAGAAAGTAGATTTTTT TCTCAAGGATGAGCAAAGAAAG 102251501 rs28664200

HG00111 >hsa-mir-3151TGATGGGTGGGGCAATGGGATC TGGGTGGGGCAATGGGATCAGG TGGGGCAATGGGATCAGGTGCC GGGGTGATGGGTGGGGCAATGG GGGTGATGGGTGGGGCAATGGG 104166902 rs35605502

HG00111 >hsa-mir-3156-3 TGCAGAAGAAAGATCTGGAAGT

GCAGAAGAAAGATCTGGAAGTG GAAGAAAGATCTGGAAGTGGGA
GAAAGATCTGGAAGTGGGAGAC AGAAGAAAGATCTGGAAGTGGG 14778721
rs2747232
HG00111 >hsa-mir-378d-2 TGGACTTGGAGTCAGAAAACTT
GACTTGGAGTCAGAAAACTTTC GGACTTGGAGTCAGAAAACTTT
GAACACTGGACTTGGAGTCAGA TACAAGGAGAGAACACTGGACT 94928250
rs73692959
HG00111 >hsa-mir-3652TGGAGGCGGCTCCTGCGATCGA GGAGGCGGCTCCTGCGATCGAA
TGGGGGGTGGAGGCGGCTCCTG GTGGAGGCGGCTCCTGCGATCG
GGTGGAGGCGGCTCCTGCGATC 104324266 rs17797090

HG00111 >hsa-mir-516b-2 TGGAGGTAAGAAGCACTTTGTG
TCTGGAGGTAAGAAGCACTTTG TGACCATCTGGAGGTAAGAAGC
TGTGACCATCTGGAGGTAAGAA TGATGTGACCATCTGGAGGTAA 54228742
rs10670323
HG00111 >hsa-mir-608 TGGGACAGCTGCGTTTAAAAAG TTGGGACAGCTGCGTTTAAAAA
GGACAGCTGCGTTTAAAAAGGC TGTTGGGACAGCTGCGTTTAAA
GGGACAGCTGCGTTTAAAAAGG 102734778 rs4919510

HG00111 >hsa-mir-1273h TGGGAGGTCAAGGCTGTAGTGT
TGAGCCTGGGAGGTCAAGGCTG TTGAGCCTGGGAGGTCAAGGCT
TGCTTGAGCCTGGGAGGTCAAG TTGCTTGAGCCTGGGAGGTCAA 24214486 .

HG00111 >hsa-mir-1227TGGTGGGCACTGCTGGGGTGGG TGGGGCCAGGCGGTGGTGGGCA
AGGCGGTGGTGGGCACTGCTGG GGTGGGCACTGCTGGGGTGGGC
GTGGTGGGCACTGCTGGGGTGG 2234093 rs190788838

HG00111 >hsa-mir-323bTGTCCGTGGTGAGTTCGCATTA TTGTCCGTGGTGAGTTCGCATT
TACTCGGAGGGAGGTTGTCCGT TCGGAGGGAGGTTGTCCGTGGT
AGGTTGTCCGTGGTGAGTTCGC 101522556 rs56103835

HG00111 >hsa-mir-181dTGTTGTCGGTGGGTTGTGAGGA TTGTTGTCGGTGGGTTGTGAGG
TCATTGTTGTCGGTGGGTTGTG TTCATTGTTGTCGGTGGGTTGT
TCAACATTCATTGTTGTCGGTG 13985772 rs369928561

HG00111 >hsa-mir-642bTTCCCTCTCCAAATGTGTCTTG TTGGGAGGTTCCCTCTCCAAAT
TGGGAGGTTCCCTCTCCAAATG GAGTTGGGAGGTTCCCTCTCCA
GTTGGGAGGTTCCCTCTCCAAA 46178217 rs111664333

HG00111 >hsa-mir-4277TTCTGAGCACAGTACACTGGGC TCGAGGCAGTTCTGAGCACAGT
TGGGTCGAGGCAGTTCTGAGCA TTGGGTCGAGGCAGTTCTGAGC
GTTCTGAGCACAGTACACTGGG 1708983 rs12523324

HG00111 >hsa-mir-553 TTTAAAACGGTGAGATTTTGTT TTTTAAAACGGTGAGATTTTGT
ATTTTAAAACGGTGAGATTTTG TATTTTAAAACGGTGAGATTTT
TTATTTTAAAACGGTGAGATTT 100746848 .

HG00111 >hsa-mir-553 TTTAAAACGGTGAGATTTTGTT TTTTAAAACGGTGAGATTTTGT ATTTTAAAACGGTGAGATTTTG TATTTTAAAACGGTGAGATTTT TTATTTTAAAACGGTGAGATTT 100746855 rs112891767

HG00111 >hsa-mir-553 TTTAAAACGGTGAGATTTTGTT TTTTAAAACGGTGAGATTTTGT ATTTTAAAACGGTGAGATTTTG TATTTTAAAACGGTGAGATTTT TTATTTTAAAACGGTGAGATTT 100746848 . 100746855 rs112891767

HG00111 >hsa-mir-3671AATAAGGACTAGTCTGCAGTGA TTTATTTCTATCAAATAAGGAC TTTTATTTCTATCAAATAAGGA GGACTAGTCTGCAGTGATAT AAATAAGGACTAGTCTGCAGTG 65523519 rs521188

HG00111 >hsa-mir-149 AGGGAGGGAGGGACGGGGGCTG GGGCTGTGCTGGGGCGGCTGGA GGGAGGGACGGGGGCTGTGCTG ACGGGGGCTGTGCTGGGGCGGC GAGGGAGGGAGGGACGGGGGCT 241395500 rs71428439

HG00111 >hsa-mir-149 AGGGAGGGAGGGACGGGGGCTG GGGCTGTGCTGGGGCAGCCGGA GGGACGGGGGCTGTGCTGGGGC GACGGGGGCTGTGCTGGGGCAG GGGGCTGTGCTGGGGCAGCCGG 241395503 rs2292832

HG00111 >hsa-mir-149 AGGGAGGGAGGGACGGGGGCTG GGGCTGTGCTGGGGCGGCCGGA ACGGGGGCTGTGCTGGGGCGGC GGGAGGGACGGGGGCTGTGCTG GGGGCTGTGCTGGGGCGGCCGG 241395500 rs71428439 241395503 rs2292832

HG00111 >hsa-mir-658 AGGTCGGTTGGTCGGTCGGGAA GTCGGTTGGTCGGTCGGGAACG TAGGTCGGTTGGTCGGTCGGGA . .

HG00111 >hsa-mir-744 ATGCACATGCTGTTGCCACTAA TGTTGCCACTAACCTCAACCTT GCTGTTGCCACTAACCTCAACC TGCACATGCTGTTGCCACTAAC C 11985275 .

HG00111 >hsa-mir-3180-4 CTCCGGATGCCAGTCCCTCATC GGAGGGTGAAGCCTCCGGATGC CTGGCCTGGTCGCGCTGTGGCT AGCGGAGGGTGAAGCCTCCGGA GAGCGGAGGGTGAAGCCTCCGG 15248720 rs75000738

HG00111 >hsa-mir-4268CTCTCAGGATGTGATGTCACCT CCTCTCAGGATGTGATGTCACC GCTCCTCCTCTCAGGATGTGAT CTCCTCCTCTCAGGATGTGATG TCCTCTCAGGATGTGATGTCAC 220771223 rs4674470

HG00111 >hsa-mir-3151CTGATCCCACACCCCACCTGTC TGATCCCACACCCCACCTGTCA GATCCCACACCCCACCTGTCAC GGGCATCCCACCTGATCCCACA TCCCACCTGATCCCACACCCCA 104166902 rs35605502

HG00111 >hsa-mir-412 CTTCACCTGGTTCACTAGCCGT ACCTGGTTCACTAGCCGTCCGT TGTACTTCACCTGGTTCACTAG CTGGTTCACTAGCCGTCCGTAT GTACTTCACCTGGTTCACTAGC 101531849 .

HG00111 >hsa-mir-658 TAGGTCGGTTGGTCGGTCGGGA .

HG00111 >hsa-mir-658 GTCCGTTGGTCGGTCGGGAACG G
TCCGTTGGTCGGTCGGGAACGA .

HG00111 >hsa-mir-658 G
rs141002682
HG00111 >hsa-mir-658 GTAGGTCGGTTGGTCGGTCGGG G
TAGGTCGGTTGGTCGGTCGGGA . 38240368 rs141002682

HG00111 >hsa-mir-658 G GTCCGTTGGTCGGTCGGGAACG .
rs141002682
HG00111 >hsa-mir-658 G GTAGGTCGGTTGGTCGGTCGGG
TAGGTCGGTTGGTCGGTCGGGA . 38240315 . 38240368
rs141002682
HG00111 >hsa-mir-320eGAAAAGCTGGGTTGAGAAGGT AAAAGCTGGGTTGAGAAGGT
GGAAAAGCTGGGTTGAGAAGGT GGGAAAAGCTGGGTTGAGAAGG
rs10423365
HG00111 >hsa-mir-1343GCCCCTCCTGGGGCCCGCACTC CCCCTCCTGGGGCCCGCACTCT
GGGGCCCGCACTCTCGCTCTGG CCCTCCTGGGGCCCGCACTCTC
TGGGGCCCGCACTCTCGCTCTG 34963416 rs2986407

HG00111 >hsa-mir-1234GCCTAGTCGGCCTGACCACCCA TGCCTAGTCGGCCTGACCACCC
CTGACCACCCACCCCACAG GGCCTGCCTAGTCGGCCTGACC AGTCGGCCTGACCACCCACCCC
145625537 rs66769762
HG00111 >hsa-mir-3180-4 GGAGGGTGAAGCCTCCGGATGC
GGTGAAGCCTCCGGATGCCAGT AGCGGAGGGTGAAGCCTCCGGA
GCGGAGGGTGAAGCCTCCGGAT GCCTGGTCGCGCTGTGGCGAAG 15248798
rs183853838
HG00111 >hsa-mir-3180-4 GGAGGGTGAAGCCTCCGGATGC
GGTGAAGCCTCCGGATGCCAGT AGCGGAGGGTGAAGCCTCCGGA
GCGGAGGGTGAAGCCTCCGGAT CTGGCCTGGTCGCGCTGTGGCT 15248720
rs75000738 15248798 rs183853838
HG00111 >hsa-mir-1227GGCATTTGACCCCGTGCCACCC AGGCATTTGACCCCGTGCCACC
AGGCATTTGACCCCGTGCCACC TGACCCCGTGCCACCCTTTTCC
AGGCATTTGACCCCGTGCCACC 2234093 rs190788838

HG00111 >hsa-mir-580 TATTTGAGAATGATGAATCATT TGAATCATTAGGTTCCGGTCAG
ATGAATCATTAGGTTCCGGTCA TTTGAGAATGATGAATCATTAG
GAGAATGATGAATCATTAGGTT 36148057 rs115089112

HG00111 >hsa-mir-3118-1 TGAAAATTCTTCTAGTGTG ATGAAAATTCTTCTAGTGTG
TGCATTATGAAAATTCTTCTAG TTATGAAAATTCTTCTAGTGTG
ATTATGAAAATTCTTCTAGTGT 142667330 rs76132421

HG00111 >hsa-mir-558 TGAGCTGCTGTACCAAAATACC GCTGCTGTACCAAAATACCACA
TGCTGTACCAAAATACCACAAA CTGAGCTGCTGTACCAAAATAC
GAGCTGCTGTACCAAAATACCA 32757230 rs72089144

HG00111 >hsa-mir-516b-2 TGCTTCCTTTCAGAGGGTTACT
TCAGAGGGTTACTCTTTGAGA AAAGAAAGTGCTTCCTTTCAGA
CAGAGGGTTACTCTTTGAGA AAAAGAAAGTGCTTCCTTTCAG 54228742
rs10670323
HG00111 >hsa-mir-637 TGGCTAAGGTGTTGGCTCGGGC TGGCTAAGGTGTTGGCTCGGGC
.
HG00111 >hsa-mir-548aj-2 AAAAACTGCAATTACTTTTACA
TGGTAAAAACTGCAATTACTTT AACTGCAATTACTTTTACACCA
AATTACTTTTACACCAACCTAA 37883200 rs73463468

HG00111 >hsa-mir-320c-1 AAAAGCTGGGTTGAGAGGGTAG
AGCTGGGTTGAGAGGGTAGGAA CTGGGTTGAGAGGGTAGGAAAA
AGGGTAGGAAAAAAATGATGTA 19263542 .

HG00111 >hsa-mir-651 AAAAGGAAAGTGTATCCTAAAA GGAAAGTGTATCCTAAAAGGCA
TGTATCCTAAAAGGCAATGACA AAAGGAAAGTGTATCCTAAAAG 8095036
rs111336920
HG00111 >hsa-mir-202 AAAGAGGTATAGGGCATGGGAA AAGAGGTATAGGGCATGGGAAA
GGGAAAACGGGGCGGTCGGGTC TAAAGAGGTATAGGGCATGGGA 135061112
rs12355840
HG00111 >hsa-mir-520gAAAGTGCTTCCCTTTAGAGTGT CAAAGTGCTTCCCTTTAGAGTG
AAACAAAGTGCTTCCCTTTAGA AAGTGCTTCCCTTTAGAGTGTT 54225460
rs375062679
HG00111 >hsa-mir-520hAAAGTGCTTCCCTTTAGAGTTA
rs148716001
HG00111 >hsa-mir-548ap AACAAAAACCACAATTACTTTT
CAAAAACCACAATTACTTTTTA CAATTACTTTTTACTGACCTAA
rs4414449
HG00111 >hsa-mir-548ap AACAAAAACCACAATTACTTTT
CAAAAACCACAATTACTTTTTA TTACTTTTTACTGACCTAAAGA
rs4577031
HG00111 >hsa-mir-548ap AACAAAAACCACAATTACTTTT
CAAAAACCACAATTACTTTTTA CAATTACTTTTTACTGACCTAA
rs4414449 86368959 rs4577031
HG00111 >hsa-mir-1255b-2 AACCACTTTCTTTGCTCATCCG
CTTTCTTTGCTCATCCGTAAGG AAACCACTTTCTTTGCTCATCC
CTTTCTTTGCTCATCCGTAAGG 167967958 rs79639536

HG00111 >hsa-mir-516b-2 AAGAAAGTGCTTCCTTTCAGAG
AAAAGAAAGTGCTTCCTTTCAG AAGAAAAGAAAGTGCTTCCTTT
AAAGTGCTTCCTTTCAGAGGGT 54228742 rs10670323

HG00111 >hsa-mir-520cAAGAAAGTGCTTCCTTTGAGAG AGTGCTTCCTTTGAGAGGGTTA
GTGCTTCCTTTGAGAGGGTTAC TTTGAGAGGGTTACCGTTTGAG 54210774
.
HG00111 >hsa-mir-423 AAGCTCGGTCTGAGGCCCCTCA AGGCCCCTCAGTCTTGCTTCCT

TCTGAGGCCCCTCAGTCTTGCT GTCTGAGGCCCCTCAGTCTTGC 28444183 rs6505162

HG00111 >hsa-mir-449cACAGTTGCTAGTTGCACTCCTC GTTGCTAGTTGCACTCCTCTCT AACAGTTGCTAGTTGCACTCCT GTTGCACTCCTCTCTGTTGCAT 54468166 rs75661995

HG00111 >hsa-mir-3118-1 ACTGCATTATGAAAATTCTTCT ATTATGAAAATTCTTCTAGTGT GCATTATGAAAATTCTTCTAGT CTGCATTATGAAAATTCTTCTA 142667330 rs76132421

HG00111 >hsa-mir-605 AGAGAAGGCACTATGAGATTTA GGCACTATGAGATTTAGAACCA CAGAGAAGGCACTATGAGATTT GAGAAGGCACTATGAGATTTAG 53059406 rs2043556

HG00111 >hsa-mir-642bAGATACATTTGGAGAGGGACCC TTGGAGAGGGACCCTCCCAACT TTTGGAGAGGGACCCTCCCAAC ATACATTTGGAGAGGGACCCTC 46178217 rs111664333

HG00111 >hsa-mir-3180-4 AGCGGAGGGTGAAGCCTCCGGA GGAGGGTGAAGCCTCCGGATGC GAGCGGAGGGTGAAGCCTCCGG GCGGAGGGTGAAGCCTCCGGAT 15248798 rs183853838

HG00111 >hsa-mir-3180-4 AGCGGAGGGTGAAGCCTCCGGA GGAGGGTGAAGCCTCCGGATGC GAGCGGAGGGTGAAGCCTCCGG GCGGAGGGTGAAGCCTCCGGAT 15248720 rs75000738 15248798 rs183853838

HG00111 >hsa-mir-30d AGCTTTCAGTCAGATGTTTGCT GGCTAAGCTTTCAGTCAGATGT GCTAAGCTTTCAGTCAGATGTT TTCAGTCAGATGTTTGCTGCTA 135817150 .

HG00111 >hsa-mir-519a-2 AGGAAAGTGCATCCTTTTAGAG AGTGCATCCTTTTAGAGGGTTA GGAAAGTGCATCCTTTTAGAGG GAAAGGAAAGTGCATCCTTTTA 54265670 .

HG00111 >hsa-mir-630 AGTATTCTGTACCAGGGAAGGT ACCTAGTATTCTGTACCAGGGA CCAGGGAAGGTAGTTCTTAACT GGAAGGTAGTTCTTAACTATGT 72879653 rs113971639

HG00111 >hsa-mir-3686AGTGATCTGTAAGAGAAAGTAA TCTGTAAGAGAAAGTAAATGAA GTAAGAGAAAGTAAATGAAAGA ACAGTGATCTGTAAGAGAAAGT 130496365 rs6997249

HG00111 >hsa-mir-513cATAAATTTCACCTTTCTGAGAA TTCTGAGAAGAGTAATGTACAG CCTTTCTGAGAAGAGTAATGTA TTTCACCTTTCTGAGAAGAGTA 146271303 rs145416750

HG00111 >hsa-mir-323bATACACGGTCGACCTCTTTTCG TACACGGTCGACCTCTTTTCGG ACACGGTCGACCTCTTTTCGGT rs56103835

HG00111 >hsa-mir-642aATTTGGAGAGGGAACCTCCCAA AGACACATTTGGAGAGGGAACC ACACATTTGGAGAGGGAACCTC CACATTTGGAGAGGGAACCTCC 46178217 rs111664333

HG00111 >hsa-mir-515-1 CAGAGTGCCTTCTTTTGGAGCA GAGTGCCTTCTTTTGGAGCATT TGCCTTCTTTTGGAGCATTACT

GTGCCTTCTTTTGGAGCATTAC 54182326 rs374576826

HG00111 >hsa-mir-4265CAGCTGTGGGCTCAACTCTGGG ATCTCTGCAGCTGTGGGCTCAA GATCTCTGCAGCTGTGGGCTCA CTGCAGCTGTGGGCTCAACTCT 109757963 rs4676066

HG00111 >hsa-mir-744 CATGCTGTTGCCACTAACCTCA GCTGTTGCCACTAACCTCAACC CACTAACCTCAACCTTACTCGG .

HG00111 >hsa-mir-1227CATTTGACCCCGTGCCACCCTT ATTTGACCCCGTGCCACCCTTT AGGCATTTGACCCCGTGCCACC GACCCCGTGCCACCCTTTTCCC 2234093 rs190788838

HG00111 >hsa-mir-3151CCACCTGATCCCACACCCCACC CACCTGATCCCACACCCCACCT CCCACCTGATCCCACACCCCAC TGATCCCACACCCCACCTGTCA 104166902 rs35605502

HG00111 >hsa-mir-1343CCCCTCCTGGGGCCCGCACTCT CCCTCCTGGGGCCCGCACTCTC CCTGGGGCCCGCACTCTCGCTC TGGGGCCCGCACTCTCGCTCTG 34963416 rs2986407

HG00111 >hsa-mir-4268CCTCTCAGGATGTGATGTCACC CTCCTCTCAGGATGTGATGTCA CTCTCAGGATGTGATGTCACCT rs4674470

HG00111 >hsa-mir-4254CCTGGAGATACTCCACCATCTC AGATACTCCACCATCTCCCCCA GGAGATACTCCACCATCTCCCC rs12731294

HG00111 >hsa-mir-3117CTCATATAGTGCCAGGTGTTTT GACTCATATAGTGCCAGGTGTT TCATATAGTGCCAGGTGTTTTG ATAAGACTCATATAGTGCCAGG 67094171 rs12402181

HG00111 >hsa-mir-196a-2 CTCGGCAACAAGAAACTGTCTG ACTCGGCAACAAGAAACTGTCT CAACAAGAAACTGTCTGAGTTA ACAAGAAACTGTCTGAGTTACA 54385599 rs11614913

HG00111 >hsa-mir-486-2 CTCGGCGCAGCTCAGTACAGGA AGGGCCTCGGCGCAGCTCAGTA TCGGCGCAGCTCAGTACAGGAT GGGCCTCGGCGCAGCTCAGTAC 41518007 .

HG00111 >hsa-mir-3615CTCTCTCGGCTCCTCGCGGCTC GGCTCCTCGCGGCTCGCGGCGG CGGCTCCTCGCGGCTCGCGGCG TCGGCTCCTCGCGGCTCGCGGC 72744798 rs745666

HG00111 >hsa-mir-888 CTCTTTGGGTGAAGGAAGGCTC CTGACACCTCTTTGGGTGAAGG GACTGACACCTCTTTGGGTGAA CCTCTTTGGGTGAAGGAAGGCT 145076355 rs143634721

HG00111 >hsa-mir-888 CTCTTTGGGTGAAGGAAGGCTC CTGACACCTCTTTGGGTGAAGG GACTGACACCTCTTTGGGTGAA CCTCTTTGGGTGAAGGAAGGCT 145076302 rs5965660 145076355 rs143634721

HG00111 >hsa-mir-3922CTGTGGGACTTCTGGCCTTGAC ACCTGTGGGACTTCTGGCCTTG GGGACTTCTGGCCTTGACTTGA TGGGACTTCTGGCCTTGACTTG 104985443 rs61938575

HG00111 >hsa-mir-2117CTGTTCTCTTTGCCAAGGACAG GCTGTTCTCTTTGCCAAGGACA

TCTCTTTGCCAAGGACAGATCT TGTTCTCTTTGCCAAGGACAGA 41522213 rs7207008

HG00111 >hsa-mir-599 CTGTTGTGTCAGTTTATCAAAC ACTGTTGTGTCAGTTTATCAAA TCAGTTTATCAAACCCATACTT ATCAAACCCATACTTGGATGAC 100548917 rs186714401

HG00111 >hsa-mir-181dGACCCACCAGGGGATGAATGTC GGGATGAATGTCACTGTGGCTG GGGGATGAATGTCACTGTGGCT rs369928561

HG00111 >hsa-mir-888 GACTGACACCTCTTTGGGTGAA GACACCTCTTTGGGTGAAGGAA TGACACCTCTTTGGGTGAAGGA CACCTCTTTGGGTGAAGGAAGG 145076302 rs5965660

HG00111 >hsa-mir-3188GAGGCTTTGTGCGGATACGGGG GAGAGGCTTTGTGCGGATACGG GCGGATACGGGGCTGGAGGCCT rs7247237

HG00111 >hsa-mir-3188GAGGCTTTGTGCGGATACGGGG GAGAGGCTTTGTGCGGATACGG GGAGAGGCTTTGTGCGGATACG rs7247767

HG00111 >hsa-mir-3188GAGGCTTTGTGCGGATACGGGG GAGAGGCTTTGTGCGGATACGG GGAGAGGCTTTGTGCGGATACG rs7247237 18392913 rs7247767

HG00111 >hsa-mir-629 GAGGTTCTCCCAACGTAAGCCC AGGTTCTCCCAACGTAAGCCCA TCTCCCAACGTAAGCCCAGCCC CAGGAGGTTCTCCCAACGTAAG 70371761 rs377691713

HG00111 >hsa-mir-629 GAGGTTCTCCCAACGTAAGCCC AGGAGGTTCTCCCAACGTAAGC GGAGGTTCTCCCAACGTAAGCC TCTCCCAACGTAAGCCCAGCCC 70371794 .

HG00111 >hsa-mir-629 GAGGTTCTCCCAACGTAAGCCC AGGTTCTCCCAACGTAAGCCCA TCTCCCAACGTAAGCCCAGCCC CCCAACGTAAGCCCAGCCCCTC 70371761 rs377691713 70371794 .

HG00111 >hsa-mir-222 GCAGCTACATCTGGCTACTGGG TACTGGGTCTCTGATGGCATCT GCTACTGGGTCTCTGATGGCAT CTGGCTACTGGGTCTCTGATGG 45606504 rs191727254

HG00111 >hsa-mir-500bGCAGTGCACCCAGGCAAGGATT CACCCAGGCAAGGATTCTGCGA AGGCAAGGATTCTGCGAGGGGG TGCAGTGCACCCAGGCAAGGAT 49775351 rs151318590

HG00111 >hsa-mir-3180-4 GCGGAGGGTGAAGCCTCCGGAT CGCTGGCCTGGTCGCGCTGTGG TCGCTGGCCTGGTCGCGCTGTG AAGCCTCCGGATGCCAGTCCCT 15248720 rs75000738

HG00111 >hsa-mir-149 GGGAGGGAGGGACGGGGGCTGT GGAGGGACGGGGGCTGTGCTGG GACGGGGGCTGTGCTGGGGCGG AGGGACGGGGGCTGTGCTGGGG 241395500 rs71428439

HG00111 >hsa-mir-149 GGGAGGGAGGGACGGGGGCTGT GGAGGGACGGGGGCTGTGCTGG AGGGACGGGGGCTGTGCTGGGG GACGGGGGCTGTGCTGGGGCAG 241395503 rs2292832

HG00111 >hsa-mir-149 GGGAGGGAGGGACGGGGGCTGT GGAGGGACGGGGGCTGTGCTGG AGGGACGGGGGCTGTGCTGGGG GACGGGGGCTGTGCTGGGGCGG 241395500

rs71428439 241395503 rs2292832
HG00111 >hsa-mir-1244-3 GTACTATAAGTAGTTGGTTTGT
GTACTATAAGTAGTTGGTTTGT TAAGTAGTTGGTTTGTATGAGA
ACTATAAGTAGTTGGTTTGTAT 9392072 rs367702648

HG00111 >hsa-mir-1197GTAGGACACATGGTCTACTTCT ACACATGGTCTACTTCTTCTCA
ACATGGTCTACTTCTTCTCAAT TAGGACACATGGTCTACTTCTT 101491923
rs141611518
HG00111 >hsa-mir-3936TAAGGGGTGTATGGCAGATGCA CACCCGACAGATGCACTTGGCA
GATGCACCCGACAGATGCACTT TGTATGGCAGATGCACCCGACA 131701279
rs367805
HG00111 >hsa-mir-300 TATACAAGGGCAGACTCTCTCT TGATTATACAAGGGCAGACTCT
ATTATACAAGGGCAGACTCTCT rs12894467

HG00111 >hsa-mir-125b-2 TCACAGGTCAGGCTCTTGGGAC
CAGGTCAGGCTCTTGGGACCTA GCTCTTGGGACCTAGGCGGAGG
CAGGCTCTTGGGACCTAGGCGG 17962615 .

HG00111 >hsa-mir-412 TCACCTGGTTCACTAGCCGTCC TCACCTGGTTCACTAGCCGTCC
ATGTACTTCACCTGGTTCACTA CTTCACCTGGTTCACTAGCCGT 101531849
.
HG00111 >hsa-mir-1972-2 TCAGGCCAGGCACAGTGGCTCA
CAGGCACAGTGGCTCATGCCTG CCAGGCACAGTGGCTCATGCCT
AGGCACAGTGGCTCATGCCTGT 70064261 rs57629257

HG00111 >hsa-mir-532 TCCCACACCCAAGGCTTGCAGA CCTCCCACACCCAAGGCTTGCA
CTCCCACACCCAAGGCTTGCAG CACCCAAGGCTTGCAGAAGAGC 49767832
rs456615
HG00111 >hsa-mir-532 TCCCACACCCAAGGCTTGCAGA CCTCCCACACCCAAGGCTTGCA
CTCCCACACCCAAGGCTTGCAG CACCCAAGGCTTGCAGAAGAGC 49767835
rs456617
HG00111 >hsa-mir-532 TCCCACACCCAAGGCTTGCAGA CCTCCCACACCCAAGGCTTGCA
CTCCCACACCCAAGGCTTGCAG CACCCAAGGCTTGCAGAAGAGC 49767832
rs456615 49767835 rs456617
HG00111 >hsa-mir-558 TCCTGAGCTGCTGTACCAAAAT TTCCTGAGCTGCTGTACCAAAA
GAGCTGCTGTACCAAAATACCA CCTGAGCTGCTGTACCAAAATA 32757230
rs72089144
HG00111 >hsa-mir-639 TCGCTGCGGTTGCGAGCGCTGT CTATCGCTGCGGTTGCGAGCGC
GCTGCGGTTGCGAGCGCTGTAA TTTAGTCTATCGCTGCGGTTGC 14640439
rs35149836
HG00111 >hsa-mir-1234TCGGCCTGACCACCCACCCCAC GTCGGCCTGACCACCCACCCCA
CTAGTCGGCCTGACCACCCACC rs66769762

HG00111 >hsa-mir-3671TCTATCAAATAAGGACTAGTCT AAATAAGGACTAGTCTGCAGTG
TTTATTTCTATCAAATAAGGAC CAAATAAGGACTAGTCTGCAGT 65523519
rs521188
HG00111 >hsa-mir-1273h TGCTGCAGACTCGACCTCCCAG

TGCAGACTCGACCTCCCAGGCT CTGCAGACTCGACCTCCCAGGC
AGACTCGACCTCCCAGGCTTAA 24214486 .

HG00111 >hsa-mir-590 TGTAATTTTATGTATAAGCTAG AATCTGTAATTTTATGTATAAG
GTATAAGCTAGTCTCTGATTGA TTTTATGTATAAGCTAGTCTCT 73605546
rs189727189
HG00111 >hsa-mir-590 TGTAATTTTATGTATAAGCTAG TTTTATGTATAAGCTAGTCTCT
GTAATTTTATGTATAAGCTAGT GTATAAGCTAGTCTCTGATTGA 73605551
rs192756765
HG00111 >hsa-mir-590 TGTAATTTTATGTATAAGCTAG TTTTATGTATAAGCTAGTCTCT
GTAATTTTATGTATAAGCTAGT GTATAAGCTAGTCTCTGATTGA 73605546
rs189727189 73605551 rs192756765
HG00111 >hsa-mir-3909TGTCCTCTA-GGCCTGCAGTCT TA-GGCCTGCAGTCTCATGGGA
CCTGCAGTCTCATGGGAGAGTG 35731712 rs34874675

HG00111 >hsa-mir-3909TGTCCTCTA-GGCCTGCAGTCT TA-GGCCTGCAGTCTCATGGGA
GGCCTGCAGTCTCATGGGAGA CCTGCAGTCTCATGGGAGAGTG 35731697
rs9607265 35731712 rs34874675
HG00111 >hsa-mir-3909TGTCCTCTAGGGCCTGCAGTCT TCCTCTAGGGCCTGCAGTCTCA
TGGGAGAGTGACATGCACCAGG TAGGGCCTGCAGTCTCATGGGA 35731697
rs9607265
HG00111 >hsa-mir-563 TTAGGTTGACATACGTTTCCCT AGGTTGACATACGTTTCCCTGG
ACATACGTTTCCCTGGTAGCCA .

HG00111 >hsa-mir-585 TTGGGCGTATCTGTATGCTAGG TATCTGTATGCTAGGGCTGCCG
TGGGCGTATCTGTATGCTAGGG GCGTATCTGTATGCTAGGGCTG 168690612
rs62376934
HG00111 >hsa-mir-1303TTTAGAGACGGGGTCTTGCTCT TAGAGACGGGGTCTTGCTCTGT
TTAGAGACGGGGTCTTGCTCTG ACGGGGTCTTGCTCTGTTGCCA 154065383
rs75538180
HG00111 >hsa-mir-580 TTTGAGAATGATGAATCATTAG GATGAATCATTAGGTTCCGGTC
AATGATGAATCATTAGGTTCCG AGAATGATGAATCATTAGGTTC 36148057
rs115089112
HG00111 >hsa-mir-133bTTTGGTCCCCTTCAACCAGCTA TGGTCCCCTTCAACCAGCTACA
CCTTCAACCAGCTACAGCAGGG AGAGGTTTGGTCCCCTTCAACC 52013832
rs374103744
HG00112 >hsa-mir-3125AGAATGGATAGAGGAAGCTGTG GAGGAAGCTGTGGAGAGAACTC
AGAGGAAGCTGTGGAGAGAACT GCTGTGGAGAGAACTCACGGTG
GGAAGCTGTGGAGAGAACTCAC 12877501 rs78852835

HG00112 >hsa-mir-1273h CCTGGGAGGTCAAGGCTGTAGT
TGGGAGGTCAAGGCTGTAGTGT ATTGCTTGAGCCTGGGAGGTCA
GCCTGGGAGGTCAAGGCTGTAG TTGAGCCTGGGAGGTCAAGGCT 24214486 .

HG00112 >hsa-mir-548ak GCAAAAGTAACTGCGGTTTTTG
TGCAAAAGTAACTGCGGTTTTT CAAAAGTAACTGCGGTTTTTGA
GTGCAAAAGTAACTGCGGTTTT rs7070684

HG00112 >hsa-mir-3196GCGGGGCGGCAGGGGCCTCCCC GGCGGGGCGGCAGGGGCCTCCC
GGGCGGGGCGGCAGGGGCCTCC TGGGGGCGGGGCGGCAGGGGCC
GGGGCGGGGCGGCAGGGGCCTC 61870167 rs744591

HG00112 >hsa-mir-3620GGTGGGGGCCAGCAGGGAGTGG TGAGGTGGGGGCCAGCAGGGAG
GTGAGGTGGGGGCCAGCAGGGA GAGGTGGGGGCCAGCAGGGAGT
AGGTGGGGGCCAGCAGGGAGTG 228284991 rs2070960

HG00112 >hsa-mir-3141TCACCCGGTGAGGGCGGGTGGA CCGGTGAGGGCGGGTGGAGGAG
CGGTGAGGGCGGGTGGAGGAGG CACCCGGTGAGGGCGGGTGGAG
CCCGGTGAGGGCGGGTGGAGGA 153975576 rs936581

HG00112 >hsa-mir-412 TGGGGTACGGGGATGGATGGTC GGATGGATGGTCGACCAGTTGG
GATGGATGGTCGACCAGTTGGA TCGACCAGTTGGAAAGTAATTG
ACGGGGATGGATGGTCGACCAG 101531854 rs61992671

HG00112 >hsa-mir-412 TGGGGTACGGGGATGGATGGTC GGATGGATGGTCGACCAGTTGG
GATGGATGGTCGACCAGTTGGA GGGTACGGGGATGGATGGTCGA
TCGACCAGTTGGAAAGTAATTG 101531849 . 101531854 rs61992671

HG00112 >hsa-mir-553 TTTAAGACGGTGAGATTTTGTT TATTTTAAGACGGTGAGATTTT
TTATTTTAAGACGGTGAGATTT TTTTATTTTAAGACGGTGAGAT
AATTTTATTTTAAGACGGTGAG 100746814 rs190622705

HG00112 >hsa-mir-553 TTTTAAAACGGTGAGATTTTGT TATTTTAAAACGGTGAGATTTT
TTTTATTTTAAAACGGTGAGAT TTTAAAACGGTGAGATTTTGTT
ATTTTATTTTAAAACGGTGAGA 100746848 .

HG00112 >hsa-mir-553 TTTTAAGACGGTGAGATTTTGT TATTTTAAGACGGTGAGATTTT
TTTAAGACGGTGAGATTTTGTT TTTTATTTTAAGACGGTGAGAT
ATTTTATTTTAAGACGGTGAGA 100746814 rs190622705 100746848 .

HG00112 >hsa-mir-3141ACCCGGTGAGGGCGGGTGGAGG CGGTGAGGGCGGGTGGAGGAGG
CCGGTGAGGGCGGGTGGAGGAG CCCGGTGAGGGCGGGTGGAGGA
CACCCGGTGAGGGCGGGTGGAG 153975576 rs936581

HG00112 >hsa-mir-1307ACCGGACCTCGACCGGCTCGTC CGGACCTCGACCGGCTCGTCTG
CCGGACCTCGACCGGCTCGTCT ATCTCGACCGGACCTCGACCGG
AATCTCGACCGGACCTCGACCG 105154089 rs7911488

HG00112 >hsa-mir-486-2 ACTGAGCTGCCCCGAGCTGGGC
CTGAGCTGCCCCGAGCTGGGCA CTGTACTGAGCTGCCCCGAGCT
GTACTGAGCTGCCCCGAGCTGG CCTGTACTGAGCTGCCCCGAGC 41518007 .

HG00112 >hsa-mir-3175AGAGAACGCAGTGACGTCTGGC GAGAGAACGCAGTGACGTCTGG
GGAGAGAACGCAGTGACGTCTG GGGAGAGAACGCAGTGACGTCT

GGGGAGAGAACGCAGTGACGTC 93447631 rs1439619

HG00112 >hsa-mir-1269b AGCCATGCTACGGGCTTCTCTG
ACTGAGCCATGCTACGGGCTTC AGGTTTCTGGACTGAGCCATGC
TGAGGTTTCTGGACTGAGCCAT TTTCTGGACTGAGCCATGCTAC 12820632
rs12451747
HG00112 >hsa-mir-3135b AGCGAGTGCAGTGGTGCAGTCA
AGGCTGGAGCGAGTGCAGTGGT CTGGAGCGAGTGCAGTGGTGCA
CAGGCTGGAGCGAGTGCAGTGG TGGAGCGAGTGCAGTGGTGCAG 32717702
rs4285314
HG00112 >hsa-mir-1265ATGTGGTCAAGTGTTGTTAAGG AGGATGTGGTCAAGTGTTGTTA
TGTGGTCAAGTGTTGTTAAGGC GTGGTCAAGTGTTGTTAAGGCA
ACTCAGGATGTGGTCAAGTGTT 14478618 rs11259096

HG00112 >hsa-mir-500bCTTGCTACCTGGGTGAGAGTGC ATCCTTGCTACCTGGGTGAGAG
AATCCTTGCTACCTGGGTGAGA CCTTGCTACCTGGGTGAGAGTG
TCCTTGCTACCTGGGTGAGAGT 49775351 rs151318590

HG00112 >hsa-mir-573 GTGTAACTGATCAGGATCTACT TGTGTAACTGATCAGGATCTAC
GATGTGTAACTGATCAGGATCT TGATGTGTAACTGATCAGGATC
GTGATGTGTAACTGATCAGGAT 24521902 rs76014664

HG00112 >hsa-mir-573 GTGTAACTGATCAGGATCTACT TGTGTAACTGATCAGGATCTAC
GATGTGTAACTGATCAGGATCT TGATGTGTAACTGATCAGGATC
GTGATGTGTAACTGATCAGGAT 24521904 rs78830737

HG00112 >hsa-mir-573 GTGTAACTGATCAGGATCTACT TGTGTAACTGATCAGGATCTAC
GATGTGTAACTGATCAGGATCT TGATGTGTAACTGATCAGGATC
GTGATGTGTAACTGATCAGGAT 24521902 rs76014664 24521904
rs78830737
HG00112 >hsa-mir-573 GTGTAACTGATCAGGATTTACT TGTAACTGATCAGGATTTACTC
ATGTGTAACTGATCAGGATTTA TGTGTAACTGATCAGGATTTAC
GATGTGTAACTGATCAGGATTT 24521871 rs192259662

HG00112 >hsa-mir-573 GTGTAACTGATCAGGATTTACT TGTAACTGATCAGGATTTACTC
ATGTGTAACTGATCAGGATTTA TGTGTAACTGATCAGGATTTAC
GATGTGTAACTGATCAGGATTT 24521871 rs192259662 24521902
rs76014664
HG00112 >hsa-mir-573 GTGTAACTGATCAGGATTTACT TGTAACTGATCAGGATTTACTC
ATGTGTAACTGATCAGGATTTA TGTGTAACTGATCAGGATTTAC
GATGTGTAACTGATCAGGATTT 24521871 rs192259662 24521904
rs78830737
HG00112 >hsa-mir-573 GTGTAACTGATCAGGATTTACT TGTAACTGATCAGGATTTACTC
ATGTGTAACTGATCAGGATTTA TGTGTAACTGATCAGGATTTAC
GATGTGTAACTGATCAGGATTT 24521871 rs192259662 24521902
rs76014664 24521904 rs78830737
HG00112 >hsa-mir-3117TAAAGGGCCAGACACTATACGA GCCAGACACTATACGAGTCATA

GGGCCAGACACTATACGAGTCA GGCCAGACACTATACGAGTCAT
CCCTAAAGGGCCAGACACTATA 67094171 rs12402181

HG00112 >hsa-mir-629 TACGTTGGGAGAACTTTTATGG TTACGTTGGGAGAACTTTTATG
TTTACGTTGGGAGAACTTTTAT TGGGTTTACGTTGGGAGAACTT
GTTTACGTTGGGAGAACTTTTA 70371761 rs377691713

HG00112 >hsa-mir-888 TACTCAAAAAGCTTTCAGTCAC TCTACTCAAAAAGCTTTCAGTC
TGCTCTACTCAAAAAGCTTTCA GCTCTACTCAAAAAGCTTTCAG
GTGCTCTACTCAAAAAGCTTTC 145076355 rs143634721

HG00112 >hsa-mir-3125TAGAGGAAGCTGTGGAGAGAAC AGCTGTGGAGAGAACTCACGGT
TGGATAGAGGAAGCTGTGGAGA AAGCTGTGGAGAGAACTCACGG
AGGAAGCTGTGGAGAGAACTCA 12877501 rs78852835

HG00112 >hsa-mir-518dTAGAGGGAAGCACTTTCTGTTG TCTAGAGGGAAGCACTTTCTGT
TGACCCTCTAGAGGGAAGCACT TGTGACCCTCTAGAGGGAAGCA
TGCTGTGACCCTCTAGAGGGAA 54238208 rs74704964

HG00112 >hsa-mir-3199-1 TAGGAGAAAGTTTCTGGAAGTT
TTAGGAGAAAGTTTCTGGAAGT TGCCTTAGGAGAAAGTTTCTGG
TCCAGGGACTGCCTTAGGAGAA TGACTCCAGGGACTGCCTTAGG 28316513
rs118160653
HG00112 >hsa-mir-590 TATTCATAAAAGTGCAGTATGG TTATTCATAAAAGTGCAGTATG
TTTATTCATAAAAGTGCAGTAT TGAGTTTATTCATAAAAGTGCA
GTTTATTCATAAAAGTGCAGTA 73605546 rs189727189

HG00112 >hsa-mir-564 TCAGCAGGCAACATGGCCGAGA TGTCAGCAGGCAACATGGCCGA
GTCAGCAGGCAACATGGCCGAG TGCCAGGCACGGTGTCAGCAGG
GTGTCAGCAGGCAACATGGCCG 44903433 .

HG00112 >hsa-mir-222 TCAGTAGCCAGTGTAGATCCTG TGGCTCAGTAGCCAGTGTAGAT
TTGGCTCAGTAGCCAGTGTAGA TCATTGGCTCAGTAGCCAGTGT
TACCCTCATTGGCTCAGTAGCC 45606504 rs191727254

HG00112 >hsa-mir-515-1 TCCAAAAGAAAGCACTTTCTGT
TCTCCAAAAGAAAGCACTTTCT TTCTCCAAAAGAAAGCACTTTC
TCATTCTCCAAAAGAAAGCACT TGCAGTCATTCTCCAAAAGAAA 54182326
rs374576826
HG00112 >hsa-mir-663aTCCCAGGCGGGGCGCCGCGGGA TCCGGCGTCCCAGGCGGGGCGC
TTCCGGCGTCCCAGGCGGGGCG GCGCCGCGGGACCTCCCTCGTG
GGCGCCGCGGGACCTCCCTCGT 26188880 .

HG00112 >hsa-mir-618 TCCTTCTGAGTGTAATTACGTA TGTCCTTCTGAGTGTAATTACG
TTGTCCTTCTGAGTGTAATTAC TACTTGTCCTTCTGAGTGTAAT
GTCCTTCTGAGTGTAATTACGT 81329536 rs2682818

HG00112 >hsa-mir-412 TCGACCAGTTGGAAAGTAATTG TGGTCGACCAGTTGGAAAGTAA TGGGGTACGGGGATGGATGGTC TGGATGGTCGACCAGTTGGAAA TACGGGGATGGATGGTCGACCA 101531854 rs61992671

HG00112 >hsa-mir-412 TCGACCAGTTGGAAAGTAATTG TGGTCGACCAGTTGGAAAGTAA TGGGGTACGGGGATGGATGGTC TGGATGGTCGACCAGTTGGAAA TACGGGGATGGATGGTCGACCA 101531849 . 101531854 rs61992671

HG00112 >hsa-mir-3183TCGGAGTCGCTCGGAGCAGCCA TCTCGGAGTCGCTCGGAGCAGC TCTCTCGGAGTCGCTCGGAGCA TCTGCCCTGCCTCTCTCGGAGT TGCCTCTCTCGGAGTCGCTCGG 925764 rs2663345

HG00112 >hsa-mir-27a TGAGGAGCAGGGCTTAGCTGCT TTAGCTGCTTGTGAGCAGGGTC GAGGAGCAGGGCTTAGCTGCTT GGAGCAGGGCTTAGCTGCTTGT GAGCAGGGCTTAGCTGCTTGTG 13947292 rs895819

HG00112 >hsa-mir-3151TGATGGGTGGGGCAATGGGATC TGGGTGGGGCAATGGGATCAGG TGGGGCAATGGGATCAGGTGCC GGGGTGATGGGTGGGGCAATGG GGGTGATGGGTGGGGCAATGGG 104166902 rs35605502

HG00112 >hsa-mir-548h-2 TGCAAAAGTAATCGCGGTTTTT TGGTGCAAAAGTAATCGCGGTT TTGGTGCAAAAGTAATCGCGGT GCAAAAGTAATCGCGGTTTTTG TAGGTTGGTGCAAAAGTAATCG 11400303 .

HG00112 >hsa-mir-548jTGCAAAAGTAATTGCGGTCTTT TGGTGCAAAAGTAATTGCGGTC GCAAAAGTAATTGCGGTCTTTG GTGCAAAAGTAATTGCGGTCTT TAGCTGGTGCAAAAGTAATTGC 26951185 rs4822739

HG00112 >hsa-mir-3156-3 TGCAGAAGAAAGATCTGGAAGT GCAGAAGAAAGATCTGGAAGTG GAAAGATCTGGAAGTGGGAGAC GAAGAAAGATCTGGAAGTGGGA AGAAGAAAGATCTGGAAGTGGG 14778721 rs2747232

HG00112 >hsa-mir-1254-2 TGGAAGCTGGAGCCTGCAGTGA TGAGCCTGGAAGCTGGAGCCTG GAAGCTGGAGCCTGCAGTGAGC GGAAGCTGGAGCCTGCAGTGAG GCCTGGAAGCTGGAGCCTGCAG 23682383 rs200793185

HG00112 >hsa-mir-1273h TGGGAGGTCAAGGCTGTAGTGT TGAGCCTGGGAGGTCAAGGCTG TTGAGCCTGGGAGGTCAAGGCT TGCTTGAGCCTGGGAGGTCAAG TTGCTTGAGCCTGGGAGGTCAA 24214486 .

HG00112 >hsa-mir-3620TGGGGGCCAGCAGGGAGTGGGT TGAGGTGGGGGCCAGCAGGGAG GGGGGCCAGCAGGGAGTGGGTT GTGGGGGCCAGCAGGGAGTGGG GGTGGGGGCCAGCAGGGAGTGG 228284991 rs2070960

HG00112 >hsa-mir-642bTTCCCTCTCCAAATGTGTCTTG TTGGGAGGTTCCCTCTCCAAAT TGGGAGGTTCCCTCTCCAAATG GAGTTGGGAGGTTCCCTCTCCA GTTGGGAGGTTCCCTCTCCAAA 46178217 rs111664333

HG00112 >hsa-mir-4277TTCTGAGCACAGTACACTGGGC TCGAGGCAGTTCTGAGCACAGT TGGGTCGAGGCAGTTCTGAGCA GTTCTGAGCACAGTACACTGGG GCAGTTCTGAGCACAGTACACT 1708902 rs115200817

HG00112 >hsa-mir-553 TTTAAAACGGTGAGATTTTGTT TTTTAAAACGGTGAGATTTTGT ATTTTAAAACGGTGAGATTTTG TATTTTAAAACGGTGAGATTTT TTATTTTAAAACGGTGAGATTT 100746848 .

HG00112 >hsa-mir-553 TTTAAGACGGTGAGATTTTGTT TTTTAAGACGGTGAGATTTTGT ATTTTAAGACGGTGAGATTTTG TATTTTAAGACGGTGAGATTTT TTATTTTAAGACGGTGAGATTT 100746814 rs190622705

HG00112 >hsa-mir-553 TTTAAGACGGTGAGATTTTGTT TTTTAAGACGGTGAGATTTTGT ATTTTAAGACGGTGAGATTTTG TATTTTAAGACGGTGAGATTTT TTATTTTAAGACGGTGAGATTT 100746814 rs190622705 100746848 .

HG00112 >hsa-mir-1229TTTGGGGGAGAGTGTGGGCTGG TAGGGTTTGGGGGAGAGTGTGG AGGGTTTGGGGGAGAGTGTGGG GTTTGGGGGAGAGTGTGGGCTG GGTTTGGGGGAGAGTGTGGGCT 179225324 rs2291418

HG00112 >hsa-mir-320c-1 AAAAGCTGGGTTGAGAGGGTAG AGCTGGGTTGAGAGGGTAGGAA CTGGGTTGAGAGGGTAGGAAAA AGGGTAGGAAAAAAATGATGTA 19263542 .

HG00112 >hsa-mir-202 AAAGAGGTATAGGGCATGGGAA AAGAGGTATAGGGCATGGGAAA GGGAAAACGGGGCGGTCGGGTC TAAAGAGGTATAGGGCATGGGA 135061112 rs12355840
HG00112 >hsa-mir-1255b-2 AACCACTTTCTTTGCTCATCCG AAACCACTTTCTTTGCTCATCC CTTTCTTTGCTCATCCGTAAGG rs79639536

HG00112 >hsa-mir-423 AAGCTCGGTCTGAGGCCCCTCA AGGCCCCTCAGTCTTGCTTCCT TCTGAGGCCCCTCAGTCTTGCT GTCTGAGGCCCCTCAGTCTTGC 28444183 rs6505162
HG00112 >hsa-mir-24-2ACACTGGCTCAGTTCAGCAGGA CACTGGCTCAGTTCAGCAGGAA TGGCTCAGTTCAGCAGGAACAG CTGGCTCAGTTCAGCAGGAACA 13947170 .
HG00112 >hsa-mir-518dACCAAAGCGCTTCCCTTTGGAG AAACCAAAGCGCTTCCCTTTGG AACCAAAGCGCTTCCCTTTGGA AGCGCTTCCCTTTGGAGCGTTA 54238208 rs74704964
HG00112 >hsa-mir-3118-1 ACTGCATTATGAAAATTCTTCT ATTATGAAAATTCTTCTAGTGT GCATTATGAAAATTCTTCTAGT CTGCATTATGAAAATTCTTCTA 142667330 rs76132421

HG00112 >hsa-mir-605 AGAGAAGGCACTATGAGATTTA GGCACTATGAGATTTAGAACCA CAGAGAAGGCACTATGAGATTT GAGAAGGCACTATGAGATTTAG 53059406 rs2043556

HG00112 >hsa-mir-642bAGATACATTTGGAGAGGGACCC TTGGAGAGGGACCCTCCCAACT TTTGGAGAGGGACCCTCCCAAC ATACATTTGGAGAGGGACCCTC 46178217 rs111664333

HG00112 >hsa-mir-3180-4 AGCGGAGGGTGAAGCCTCCGGA CGGAGGGTGAAGCCTCCGGATG GAGCGGAGGGTGAAGCCTCCGG GCGGAGGGTGAAGCCTCCGGAT 15248798 rs183853838

HG00112 >hsa-mir-3180-4 AGCGGAGGGTGAAGCCTCCGGA CGGAGGGTGAAGCCTCCGGATG GAGCGGAGGGTGAAGCCTCCGG GCGGAGGGTGAAGCCTCCGGAT 15248720 rs75000738 15248798 rs183853838

HG00112 >hsa-mir-30d AGCTTTCAGTCAGATGTTTGCT GGCTAAGCTTTCAGTCAGATGT GCTAAGCTTTCAGTCAGATGTT TTCAGTCAGATGTTTGCTGCTA 135817150 .

HG00112 >hsa-mir-519a-2 AGGAAAGTGCATCCTTTTAGAG AGTGCATCCTTTTAGAGGGTTA GGAAAGTGCATCCTTTTAGAGG GAAAGGAAAGTGCATCCTTTTA 54265670 .

HG00112 >hsa-mir-646 AGGAAGCAGCTGCCTCGGAGGC CCAGGAGAGGAAGCAGCTGCCT GCTGCCTCGGAGGCCTCAGGCT GAGAGGAAGCAGCTGCCTCGGA 58883605 rs6513497

HG00112 >hsa-mir-646 AGGAAGCAGCTGCCTCGGAGGC TGCCTCGGAGGCCTCAGGCTCA CGGAGGCCTCAGGCTCAGTGGC CTGCCTCGGAGGCCTCAGGCTC 58883534 rs6513496 58883605 rs6513497

HG00112 >hsa-mir-646 AGGAAGCAGCTGCCTCTGAGGC GCTGCCTCTGAGGCCTCAGGCT CTGAGGCCTCAGGCTCAGTGGC TCTGAGGCCTCAGGCTCAGTGG 58883534 rs6513496

HG00112 >hsa-mir-630 AGTATTCTGTACCAGGGAAGGT ACCTAGTATTCTGTACCAGGGA CCAGGGAAGGTAGTTCTTAACT GGAAGGTAGTTCTTAACTATGT 72879653 rs113971639

HG00112 >hsa-mir-642aATTTGGAGAGGGAACCTCCCAA AGACACATTTGGAGAGGGAACC ACACATTTGGAGAGGGAACCTC CACATTTGGAGAGGGAACCTCC 46178217 rs111664333

HG00112 >hsa-mir-603 CACACACTGCAATTACTTTTGC TGGCACACACTGCAATTACTTT CACTGCAATTACTTTTGCTCCA ACACTGCAATTACTTTTGCTCC 24564653 rs11014002

HG00112 >hsa-mir-412 CACCTGGTCCACTGGCCGTCCG ACCTGGTCCACTGGCCGTCCGT CTGGCCGTCCGTATCCGCTGCA CCTGGTCCACTGGCCGTCCGTA 101531854 rs61992671

HG00112 >hsa-mir-412 CACCTGGTTCACTGGCCGTCCG ACCTGGTTCACTGGCCGTCCGT CTGGCCGTCCGTATCCGCTGCA CCTGGTTCACTGGCCGTCCGTA 101531849 . 101531854 rs61992671

HG00112 >hsa-mir-515-1 CAGAGTGCCTTCTTTTGGAGCA GAGTGCCTTCTTTTGGAGCATT TGCCTTCTTTTGGAGCATTACT GTGCCTTCTTTTGGAGCATTAC 54182326 rs374576826

HG00112 >hsa-mir-4265CAGCTGTGGGCTCAACTCTGGG ATCTCTGCAGCTGTGGGCTCAA

GATCTCTGCAGCTGTGGGCTCA CTGCAGCTGTGGGCTCAACTCT 109757963 rs4676066
HG00112 >hsa-mir-3151CCACCTGATCCCACACCCCACC CACCTGATCCCACACCCCACCT CCCACCTGATCCCACACCCCAC TGATCCCACACCCCACCTGTCA 104166902 rs35605502
HG00112 >hsa-mir-1229CCTCTCACCACTGCCCTCCCAC GACACCCTCTCACCACTGCCCT ACCCTCTCACCACTGCCCTCCC CTCTCACCACTGCCCTCCCACA 179225324 rs2291418
HG00112 >hsa-mir-3620CTCACCCTGCATCCCGCACCCA CCTCACCCTGCATCCCGCACCC CTCACCCTGCATCCCGCACCCA rs2070960

HG00112 >hsa-mir-3117CTCATATAGTGCCAGGTGTTTT GACTCATATAGTGCCAGGTGTT TCATATAGTGCCAGGTGTTTTG AGACTCATATAGTGCCAGGTGT 67094171 rs12402181
HG00112 >hsa-mir-486-2 CTCGGCGCAGCTCAGTACAGGA AGGGCCTCGGCGCAGCTCAGTA GGGCCTCGGCGCAGCTCAGTAC TCGGCGCAGCTCAGTACAGGAT 41518007 .

HG00112 >hsa-mir-3615CTCTCTCGGCTCCTCGCGGCTC GGCTCCTCGCGGCTCGCGGCGG CGGCTCCTCGCGGCTCGCGGCG TCGGCTCCTCGCGGCTCGCGGC 72744798 rs745666
HG00112 >hsa-mir-888 CTCTTTGGGTGAAGGAAGGCTC CTGACACCTCTTTGGGTGAAGG GACTGACACCTCTTTGGGTGAA CCTCTTTGGGTGAAGGAAGGCT 145076355 rs143634721
HG00112 >hsa-mir-1269a CTGGACTGAGCCATGCTACTGG TGCCTGGACTGAGCCATGCTAC AATGCCTGGACTGAGCCATGCT rs73239138

HG00112 >hsa-mir-940 GAAGGCAGGGCCCC-GCTCCCC G CCC-GCTCCCCGGGCCTGACCC rs35356504

HG00112 >hsa-mir-4274GACCCAGCAGTCCCTCCCCCTG CCCAGCAGTCCCTCCCCCTGCA TGACCCAGCAGTCCCTCCCCCT TCAGGTGACCCAGCAGTCCCTC 7461769 rs12512664
HG00112 >hsa-mir-1307GACTCGGCGTGGCGTCGGTCGT CGTGGCGTCGGTCGTGGTAGAT ATCGACTCGGCGTGGCGTCGGT CGACTCGGCGTGGCGTCGGTCG 105154089 rs7911488
HG00112 >hsa-mir-3188GAGGCTTTGTGCGGATACGGGG GAGAGGCTTTGTGCGGATACGG GCGGATACGGGGCTGGAGGCCT rs7247237

HG00112 >hsa-mir-3188GAGGCTTTGTGCGGATACGGGG GGAGAGGCTTTGTGCGGATACG GAGAGGCTTTGTGCGGATACGG rs7247767

HG00112 >hsa-mir-3188GAGGCTTTGTGCGGATACGGGG GAGAGGCTTTGTGCGGATACGG GGAGAGGCTTTGTGCGGATACG rs7247237 18392913 rs7247767

HG00112 >hsa-mir-629 GAGGTTCTCCCAACGTAAGCCC AGGTTCTCCCAACGTAAGCCCA TCTCCCAACGTAAGCCCAGCCC CAGGAGGTTCTCCCAACGTAAG 70371761

rs377691713
HG00112 >hsa-mir-222 GCAGCTACATCTGGCTACTGGG TACTGGGTCTCTGATGGCATCT GCTACTGGGTCTCTGATGGCAT CTGGCTACTGGGTCTCTGATGG 45606504 rs191727254
HG00112 >hsa-mir-500bGCAGTGCACCCAGGCAAGGATT CACCCAGGCAAGGATTCTGCGA AGGCAAGGATTCTGCGAGGGGG TGCAGTGCACCCAGGCAAGGAT 49775351 rs151318590
HG00112 >hsa-mir-3180-4 GCGGAGGGTGAAGCCTCCGGAT CGCTGGCCTGGTCGCGCTGTGG TCGCTGGCCTGGTCGCGCTGTG AAGCCTCCGGATGCCAGTCCCT 15248720 rs75000738

HG00112 >hsa-mir-1197GTAGGACACATGGTCTACTTCT ACACATGGTCTACTTCTTCTCA TAGGACACATGGTCTACTTCTT ACATGGTCTACTTCTTCTCAAT 101491923 rs141611518
HG00112 >hsa-mir-27a GTGTTCACAGTGGCTAAGTTCC TCGTGTTCACAGTGGCTAAGTT TGTTCACAGTGGCTAAGTTCCG AGTGGCTAAGTTCCGCCCCCCA 13947292 rs895819
HG00112 >hsa-mir-3936TAAGGGGTGTATGGCAGATGCA CACCCGACAGATGCACTTGGCA TTCTGGTAAGGGGTGTATGGCA GATGCACCCGACAGATGCACTT 131701279 rs367805
HG00112 >hsa-mir-499aTCACAGCAAGTCTGTGCTGCTT ACAGCAAGTCTGTGCTGCTTCC CGTCACAGCAAGTCTGTGCTGC GTGCTGCTTCCCGTCCCTACGC 33578251 rs3746444
HG00112 >hsa-mir-412 TCACCTGGTTCACTAGCCGTCC TCACCTGGTTCACTAGCCGTCC ATGTACTTCACCTGGTTCACTA CTTCACCTGGTTCACTAGCCGT 101531849 .
HG00112 >hsa-mir-499bTCACTGCAAGTCTTAACAGCCG rs3746444 73

HG00112 >hsa-mir-1972-2 TCAGGCCAGGCACAGTGGCTCA CAGGCACAGTGGCTCATGCCTG CCAGGCACAGTGGCTCATGCCT AGGCACAGTGGCTCATGCCTGT 70064261 rs57629257

HG00112 >hsa-mir-595 TGAAGTGTGCCGTGGTGTGTCT GTGTGTCTGGAGGAAGCGCCTG GCCGTGGTGTGTCTGGAGGAAG rs4909237

HG00112 >hsa-mir-1273h TGCTGCAGACTCGACCTCCCAG TGCAGACTCGACCTCCCAGGCT CTGCAGACTCGACCTCCCAGGC AGACTCGACCTCCCAGGCTTAA 24214486 .

HG00112 >hsa-mir-548jTGGCAAAAACTGCATTACTTTT GGCAAAAACTGCATTACTTTTG CAAAAACTGCATTACTTTTGCA AAACTGCATTACTTTTGCACCA 26951185 rs4822739
HG00112 >hsa-mir-590 TGTAATTTTATGTATAAGCTAG AATCTGTAATTTTATGTATAAG GTATAAGCTAGTCTCTGATTGA TTTTATGTATAAGCTAGTCTCT 73605546 rs189727189
HG00112 >hsa-mir-2053TGTTAATTAAACCTCTATTTAC TTTAAGTGTTAATTAAACCTCT ACTTTAAGTGTTAATTAAACCT GTTAATTAAACCTCTATTTACA 113655752

rs10505168
HG00112 >hsa-mir-585 TTGGGCGTATCTGTATGCTAGG TGGGCGTATCTGTATGCTAGGG TATCTGTATGCTAGGGCTGCCG GCGTATCTGTATGCTAGGGCTG 168690612 rs62376934
HG00112 >hsa-mir-1303TTTAGAGACGGGGTCTTGCTCT TAGAGACGGGGTCTTGCTCTGT ACGGGGTCTTGCTCTGTTGCCA GGGTCTTGCTCTGTTGCCAGGC 154065347 rs142414368
HG00112 >hsa-mir-580 TTTGAGAATGATGAATCATTAG GATGAATCATTAGGTTCCGGTC AATGATGAATCATTAGGTTCCG AGAATGATGAATCATTAGGTTC 36148057 rs115089112
HG00112 >hsa-mir-133bTTTGGTCCCCTTCAACCAGCTA TGGTCCCCTTCAACCAGCTACA CCTTCAACCAGCTACAGCAGGG AGAGGTTTGGTCCCCTTCAACC 52013832 rs374103744
HG00114 >hsa-mir-3144AAGGGGACCAAAGAGATATATA TAAGGGGACCAAAGAGATATAT TTAAGGGGACCAAAGAGATATA TTTAAGGGGACCAAAGAGATAT ATACACTTTAAGGGGACCAAAG 120336327 rs68035463

HG00114 >hsa-mir-3125AGAATGGATAGAGGAAGCTGTG GAGGAAGCTGTGGAGAGAACTC AGAGGAAGCTGTGGAGAGAACT GCTGTGGAGAGAACTCACGGTG GGAAGCTGTGGAGAGAACTCAC 12877501 rs78852835

HG00114 >hsa-mir-1908CCGCGGCGGGGACGGCGATTGG GCGGCGGGGACGGCGATTGGTC CGCGGCGGGGACGGCGATTGGT CGGGGACGGCGATTGGTCCGTA GGGGACGGCGATTGGTCCGTAT 61582708 rs174561

HG00114 >hsa-mir-1273h CCTGGGAGGTCAAGGCTGTAGT TGGGAGGTCAAGGCTGTAGTGT GCCTGGGAGGTCAAGGCTGTAG ATTGCTTGAGCCTGGGAGGTCA TTGAGCCTGGGAGGTCAAGGCT 24214486 .

HG00114 >hsa-mir-3196GCGGGGCGGCAGGGGCCTCCCC GGCGGGGCGGCAGGGGCCTCCC GGGCGGGGCGGCAGGGGCCTCC TGGGGGCGGGGCGGCAGGGGCC GGGGCGGGGCGGCAGGGGCCTC 61870167 rs744591

HG00114 >hsa-mir-3144TAAGGGGACCAAAGAGATATAT TTAAGGGGACCAAAGAGATATA AAGGGGACCAAAGAGATATATA CTACACTTTAAGGGGACCAAAG TTTAAGGGGACCAAAGAGATAT 120336384 rs67106263

HG00114 >hsa-mir-3144TAAGGGGACCAAAGAGATATAT AAGGGGACCAAAGAGATATATA TTAAGGGGACCAAAGAGATATA ATACACTTTAAGGGGACCAAAG TTTAAGGGGACCAAAGAGATAT 120336327 rs68035463 120336384 rs67106263
HG00114 >hsa-mir-1268a TAGCCAGGCGTGGTGGTGGGGG GCCAGGCGTGGTGGTGGGGGCC AGCCAGGCGTGGTGGTGGGGGC

HG00114 >hsa-mir-4326TCTGCTGTTCCTCTGTCTCCCA TGGTCTGCTGTTCCTCTGTCTC CTGGTCTGCTGTTCCTCTGTCT GCTGTTCCTCTGTCTCCCAGAC TGCTGTTCCTCTGTCTCCCAGA 61918164 rs6062431

HG00114 >hsa-mir-412 TGGGGTACGGGGATGGATGGTC GGATGGATGGTCGACCAGTTGG GATGGATGGTCGACCAGTTGGA TCGACCAGTTGGAAAGTAATTG ACGGGGATGGATGGTCGACCAG 101531854 rs61992671

HG00114 >hsa-mir-553 TTTAAGACGGTGAGATTTTGTT TTATTTTAAGACGGTGAGATTT TATTTTAAGACGGTGAGATTTT ATTTTAAGACGGTGAGATTTTG TTTTATTTTAAGACGGTGAGAT 100746814 rs190622705

HG00114 >hsa-mir-553 TTTAAGACGGTGAGATTTTGTT TTTTATTTTAAGACGGTGAGAT TTTTAAGACGGTGAGATTTTGT AATTTTATTTTAAGACGGTGAG TTATTTTAAGACGGTGAGATTT 100746814 rs190622705 100746855 rs112891767

HG00114 >hsa-mir-553 TTTTAAAACGGTGAGATTTTGT TATTTTAAAACGGTGAGATTTT TTTTATTTTAAAACGGTGAGAT TTTAAAACGGTGAGATTTTGTT ATTTTATTTTAAAACGGTGAGA 100746848 .

HG00114 >hsa-mir-553 TTTTAAAACGGTGAGATTTTGT TTTTATTTTAAAACGGTGAGAT TTTAAAACGGTGAGATTTTGTT TATTTTAAAACGGTGAGATTTT ATTTTATTTTAAAACGGTGAGA 100746848 . 100746855 rs112891767

HG00114 >hsa-mir-553 TTTTAAGACGGTGAGATTTTGT TATTTTAAGACGGTGAGATTTT TTTAAGACGGTGAGATTTTGTT TTTTATTTTAAGACGGTGAGAT ATTTTATTTTAAGACGGTGAGA 100746814 rs190622705 100746848 .

HG00114 >hsa-mir-553 TTTTAAGACGGTGAGATTTTGT TTTTATTTTAAGACGGTGAGAT TTTAAGACGGTGAGATTTTGTT TATTTTAAGACGGTGAGATTTT ATTTTATTTTAAGACGGTGAGA 100746814 rs190622705 100746848 . 100746855 rs112891767

HG00114 >hsa-mir-553 TTTTATTTTAAAACGGTGAGAT TTTAAAACGGTGAGATTTTGTT TTTTAAAACGGTGAGATTTTGT TTATTTTAAAACGGTGAGATTT AATTTTATTTTAAAACGGTGAG 100746855 rs112891767

HG00114 >hsa-mir-486-2 ACTGAGCTGCCCCGAGCTGGGC CTGAGCTGCCCCGAGCTGGGCA CTGTACTGAGCTGCCCCGAGCT CCTGTACTGAGCTGCCCCGAGC GTACTGAGCTGCCCCGAGCTGG 41518007 .

HG00114 >hsa-mir-1269b AGCCATGCTACGGGCTTCTCTG ACTGAGCCATGCTACGGGCTTC AGGTTTCTGGACTGAGCCATGC TGAGGTTTCTGGACTGAGCCAT TTTCTGGACTGAGCCATGCTAC 12820632 rs12451747

HG00114 >hsa-mir-1269b AGCCATGCTACGGGCTTCTCTG AGTGAGCCATGCTACGGGCTTC AGGTTTCTGGAGTGAGCCATGC TGAGGTTTCTGGAGTGAGCCAT TTTCTGGAGTGAGCCATGCTAC 12820632 rs12451747 12820646 rs7210937

HG00114 >hsa-mir-3125AGCTGTGGAGAGAACTCACGGT AAGCTGTGGAGAGAACTCACGG TAGAGGAAGCTGTGGAGAGAAC TGGATAGAGGAAGCTGTGGAGA

AGGAAGCTGTGGAGAGAACTCA 12877501 rs78852835

HG00114 >hsa-mir-548ap AGTAATTGCAGTCTTTGTCATT
AAGTAATTGCAGTCTTTGTCAT AAAGTAATTGCAGTCTTTGTCA
AAAAGTAATTGCAGTCTTTGTC CAAAAGTAATTGCAGTCTTTGT 86368898
rs4414449
HG00114 >hsa-mir-548ap AGTAATTGCAGTCTTTGTCATT
AAGTAATTGCAGTCTTTGTCAT AAAGTAATTGCAGTCTTTGTCA
AAAAGTAATTGCAGTCTTTGTC CAAAAGTAATTGCAGTCTTTGT 86368898
rs4414449 86368959 rs4577031
HG00114 >hsa-mir-548ap AGTAATTGCGGTCTTTGTCATT
AAGTAATTGCGGTCTTTGTCAT AAAGTAATTGCGGTCTTTGTCA
AAAAGTAATTGCGGTCTTTGTC CAAAAGTAATTGCGGTCTTTGT 86368959
rs4577031
HG00114 >hsa-mir-196a-2 AGTTTCATGTTGTTGGGATTGA
AGGTAGTTTCATGTTGTTGGGA TAGTTTCATGTTGTTGGGATTG
GTAGTTTCATGTTGTTGGGATT GGTAGTTTCATGTTGTTGGGAT 54385599
rs11614913
HG00114 >hsa-mir-500bATCCTTGCTACCTGGGTGAGAG CTTGCTACCTGGGTGAGAGTGC
AATCCTTGCTACCTGGGTGAGA CCTTGCTACCTGGGTGAGAGTG
TCCTTGCTACCTGGGTGAGAGT 49775351 rs151318590

HG00114 >hsa-mir-650 GAGGCAGCGCTCTCAGGACGTC GGAGGCAGCGCTCTCAGGACGT
TCAGGAGGCAGCGCTCTCAGGA TCTCAGGAGGCAGCGCTCTCAG
GTCTCAGGAGGCAGCGCTCTCA 23165340 rs5996397

HG00114 >hsa-mir-1908GGGGACGGCGATTGGTCCGTAT GCGGGGACGGCGATTGGTCCGT
GGCGGGGACGGCGATTGGTCCG CGGGGACGGCGATTGGTCCGTA
TGCCGCGGCGGGGACGGCGATT 61582708 rs174561

HG00114 >hsa-mir-573 GTGTAACTGATCAGGATCTACT TGTGTAACTGATCAGGATCTAC
GATGTGTAACTGATCAGGATCT TGATGTGTAACTGATCAGGATC
GTGATGTGTAACTGATCAGGAT 24521902 rs76014664

HG00114 >hsa-mir-3117TAAAGGGCCAGACACTATACGA GGGCCAGACACTATACGAGTCA
GCCAGACACTATACGAGTCATA GGCCAGACACTATACGAGTCAT
CCCTAAAGGGCCAGACACTATA 67094171 rs12402181

HG00114 >hsa-mir-3144TAAGGGGACCAAAGAGATATAT TTAAGGGGACCAAAGAGATATA
TTTAAGGGGACCAAAGAGATAT TACACTTTAAGGGGACCAAAGA
AAATACACTTTAAGGGGACCAA 120336327 rs68035463

HG00114 >hsa-mir-3144TAAGGGGACCAAAGAGATATAT TTAAGGGGACCAAAGAGATATA
TTTAAGGGGACCAAAGAGATAT TACACTTTAAGGGGACCAAAGA
AACTACACTTTAAGGGGACCAA 120336384 rs67106263

HG00114 >hsa-mir-3144TAAGGGGACCAAAGAGATATAT TTAAGGGGACCAAAGAGATATA

TTTAAGGGGACCAAAGAGATAT TACACTTTAAGGGGACCAAAGA
AAATACACTTTAAGGGGACCAA 120336327 rs68035463 120336384
rs67106263
HG00114 >hsa-mir-629 TACGTTGGGAGAACTTTTATGG TTACGTTGGGAGAACTTTTATG
TTTACGTTGGGAGAACTTTTAT TGGGTTTACGTTGGGAGAACTT
GTTTACGTTGGGAGAACTTTTA 70371761 rs377691713

HG00114 >hsa-mir-888 TACTCAAAAAGCTTTCAGTCAC TCTACTCAAAAAGCTTTCAGTC
TGCTCTACTCAAAAAGCTTTCA GCTCTACTCAAAAAGCTTTCAG
GGCAGTGCTCTACTCAAAAAGC 145076355 rs143634721

HG00114 >hsa-mir-936 TAGAGGGAGGAATCGCAGAAAT TCAAGGCCACTGGGACAGTAGA
TGGGACAGTAGAGGGAGGAATC GGAGGAATCGCAGAAATCACTC
GGGAGGAATCGCAGAAATCACT 105807858 rs145823228

HG00114 >hsa-mir-3199-1 TAGGAGAAAGTTTCTGGAAGTT
TTAGGAGAAAGTTTCTGGAAGT TGCCTTAGGAGAAAGTTTCTGG
TCCAGGGACTGCCTTAGGAGAA TGACTCCAGGGACTGCCTTAGG 28316513
rs118160653
HG00114 >hsa-mir-590 TATTCATAAAAGTGCAGTATGG TTATTCATAAAAGTGCAGTATG
TTTATTCATAAAAGTGCAGTAT AGTTTATTCATAAAAGTGCAGT
TGAGTTTATTCATAAAAGTGCA 73605546 rs189727189

HG00114 >hsa-mir-3922TCAAGGCCAGAGGTCCCACAAC TCAAGTCAAGGCCAGAGGTCCC
GCCAGAGGTCCCACAACAGGGC GGCCAGAGGTCCCACAACAGGG
GTCAAGGCCAGAGGTCCCACAA 104985443 rs61938575

HG00114 >hsa-mir-222 TCAGTAGCCAGTGTAGATCCTG TGGCTCAGTAGCCAGTGTAGAT
TTGGCTCAGTAGCCAGTGTAGA TCATTGGCTCAGTAGCCAGTGT
TACCCTCATTGGCTCAGTAGCC 45606504 rs191727254

HG00114 >hsa-mir-515-1 TCCAAAAGAAAGCACTTTCTGT
TCTCCAAAAGAAAGCACTTTCT TTCTCCAAAAGAAAGCACTTTC
TCATTCTCCAAAAGAAAGCACT TGCAGTCATTCTCCAAAAGAAA 54182326
rs374576826
HG00114 >hsa-mir-663aTCCCAGGCGGGGCGCCGCGGGA TCCGGCGTCCCAGGCGGGGCGC
TTCCGGCGTCCCAGGCGGGGCG GCGCCGCGGGACCTCCCTCGTG
GGCGCCGCGGGACCTCCCTCGT 26188880 .

HG00114 >hsa-mir-149 TCCGTGTCTTCACTCCCGTGCT TGGCTCCGTGTCTTCACTCCCG
TCTGGCTCCGTGTCTTCACTCC CCGTGTCTTCACTCCCGTGCTT
AGCTCTGGCTCCGTGTCTTCAC 241395503 rs2292832

HG00114 >hsa-mir-618 TCCTTCTGAGTGTAATTACGTA TGTCCTTCTGAGTGTAATTACG
TTGTCCTTCTGAGTGTAATTAC TACTTGTCCTTCTGAGTGTAAT
GTCCTTCTGAGTGTAATTACGT 81329527 rs145551269

HG00114 >hsa-mir-618 TCCTTCTGAGTGTAATTACGTA TGTCCTTCTGAGTGTAATTACG TTGTCCTTCTGAGTGTAATTAC TACTTGTCCTTCTGAGTGTAAT GTCCTTCTGAGTGTAATTACGT 81329536 rs2682818

HG00114 >hsa-mir-618 TCCTTCTGAGTGTAATTACGTA TGTCCTTCTGAGTGTAATTACG TTGTCCTTCTGAGTGTAATTAC TACTTGTCCTTCTGAGTGTAAT GTCCTTCTGAGTGTAATTACGT 81329527 rs145551269 81329536 rs2682818

HG00114 >hsa-mir-3183TCGGAGTCGCTCGGAGCAGCCA TCTCGGAGTCGCTCGGAGCAGC TCTCTCGGAGTCGCTCGGAGCA TCTGCCCTGCCTCTCTCGGAGT TGCCCTGCCTCTCTCGGAGTCG 925764 rs2663345

HG00114 >hsa-mir-4305TCTGGGTTCTTAGAGGCCTAAT TTCTGGGTTCTTAGAGGCCTAA GTTCTGGGTTCTTAGAGGCCTA TCCAGTTCTGGGTTCTTAGAGG CAGTTCTGGGTTCTTAGAGGCC 40238175 rs67976778

HG00114 >hsa-mir-635 TGAAACAATGTCCATTAGGCTT GAAACAATGTCCATTAGGCTTT ACAATGTCCATTAGGCTTTGTT AACAATGTCCATTAGGCTTTGT CTGAAACAATGTCCATTAGGCT 66420592 rs77279010

HG00114 >hsa-mir-1200TGAGCCATTCTGAGCCTCAATC TCCTGAGCCATTCTGAGCCTCA TCTCCTGAGCCATTCTGAGCCT TTCTCCTGAGCCATTCTGAGCC TGCTACTTCTCCTGAGCCATTC 36958995 rs180826747

HG00114 >hsa-mir-27a TGAGGAGCAGGGCTTAGCTGCT TTAGCTGCTTGTGAGCAGGGTC GAGGAGCAGGGCTTAGCTGCTT GGAGCAGGGCTTAGCTGCTTGT GAGCAGGGCTTAGCTGCTTGTG 13947292 rs895819

HG00114 >hsa-mir-1269b TGAGGTTTCTGGAGTGAGCCAT TTTCTGGAGTGAGCCATGCTAC TTCTGGAGTGAGCCATGCTACT TCTGGAGTGAGCCATGCTACTG TGGAGTGAGCCATGCTACTGGC 12820646 rs7210937

HG00114 >hsa-mir-3151TGATGGGTGGGGCAATGGGATC TGGGTGGGGCAATGGGATCAGG TGGGGCAATGGGATCAGGTGCC GGGGTGATGGGTGGGGCAATGG GGGTGATGGGTGGGGCAATGGG 104166902 rs35605502

HG00114 >hsa-mir-548kTGCAAAAGTACTTGAGGATTTT TGGTGCAAAAGTACTTGAGGAT TTGGTGCAAAAGTACTTGAGGA GCAAAAGTACTTGAGGATTTTG GTGCAAAAGTACTTGAGGATTT 70130103 .

HG00114 >hsa-mir-3156-3 TGCAGAAGAAAGATCTGGAAGT GCAGAAGAAAGATCTGGAAGTG GAAGAAAGATCTGGAAGTGGGA GAAAGATCTGGAAGTGGGAGAC AGAAGAAAGATCTGGAAGTGGG 14778721 rs2747232

HG00114 >hsa-mir-1254-2 TGGAAGCTGGAGCCTGCAGTGA TGAGCCTGGAAGCTGGAGCCTG GAAGCTGGAGCCTGCAGTGAGC GGAAGCTGGAGCCTGCAGTGAG GCCTGGAAGCTGGAGCCTGCAG 23682383

rs200793185
HG00114 >hsa-mir-378d-2 TGGACTTGGAGTCAGAAAACTT GACTTGGAGTCAGAAAACTTTC GGACTTGGAGTCAGAAAACTTT GAACACTGGACTTGGAGTCAGA TACAAGGAGAGAACACTGGACT 94928250 rs73692959
HG00114 >hsa-mir-1273h TGGGAGGTCAAGGCTGTAGTGT TGAGCCTGGGAGGTCAAGGCTG TTGAGCCTGGGAGGTCAAGGCT TGCTTGAGCCTGGGAGGTCAAG TTGCTTGAGCCTGGGAGGTCAA 24214486 .

HG00114 >hsa-mir-2682TGGGGCAGGCAGTGACTGTTCA TTGGGGCAGGCAGTGACTGTTC GGCAGTGACTGTTCAGACGTCC TGAAAGAGGTTGGGGCAGGCAG GCAGGCAGTGACTGTTCAGACG 98510847 rs74904371

HG00114 >hsa-mir-1227TGGGGCCAGGCGGTGGTGGGCA TGGTGGGCACTGCTGGGGTGGG AGGCGGTGGTGGGCACTGCTGG GTGGGGCCAGGCGGTGGTGGGC GGGGCCAGGCGGTGGTGGGCAC 2234093 rs190788838

HG00114 >hsa-mir-412 TGGGGTACGGGGATGGATGGTC TCGACCAGTTGGAAAGTAATTG TGGTCGACCAGTTGGAAAGTAA TACGGGGATGGATGGTCGACCA TGGATGGTCGACCAGTTGGAAA 101531854 rs61992671

HG00114 >hsa-mir-642bTTCCCTCTCCAAATGTGTCTTG TTGGGAGGTTCCCTCTCCAAAT TGGGAGGTTCCCTCTCCAAATG GAGTTGGGAGGTTCCCTCTCCA GTTGGGAGGTTCCCTCTCCAAA 46178217 rs111664333

HG00114 >hsa-mir-4277TTCTGAGCACAGTACACTGGGC TCGAGGCAGTTCTGAGCACAGT TGGGTCGAGGCAGTTCTGAGCA GTTCTGAGCACAGTACACTGGG GCAGTTCTGAGCACAGTACACT 1708902 rs115200817

HG00114 >hsa-mir-4277TTCTGAGCACAGTACACTGGGC TCGAGGCAGTTCTGAGCACAGT TGGGTCGAGGCAGTTCTGAGCA TTGGGTCGAGGCAGTTCTGAGC GTTCTGAGCACAGTACACTGGG 1708983 rs12523324

HG00114 >hsa-mir-4277TTCTGAGCACAGTACACTGGGC TCGAGGCAGTTCTGAGCACAGT TGGGTCGAGGCAGTTCTGAGCA TTGGGTCGAGGCAGTTCTGAGC GTTCTGAGCACAGTACACTGGG 1708902 rs115200817 1708983 rs12523324
HG00114 >hsa-mir-553 TTTAAAACGGTGAGATTTTGTT TTTTAAAACGGTGAGATTTTGT ATTTTAAAACGGTGAGATTTTG TATTTTAAAACGGTGAGATTTT TTATTTTAAAACGGTGAGATTT 100746848 .

HG00114 >hsa-mir-553 TTTAAAACGGTGAGATTTTGTT TTTTAAAACGGTGAGATTTTGT ATTTTAAAACGGTGAGATTTTG TATTTTAAAACGGTGAGATTTT TTATTTTAAAACGGTGAGATTT 100746855 rs112891767

HG00114 >hsa-mir-553 TTTAAAACGGTGAGATTTTGTT TTTTAAAACGGTGAGATTTTGT ATTTTAAAACGGTGAGATTTTG TATTTTAAAACGGTGAGATTTT

TTATTTTAAAACGGTGAGATTT 100746848 . 100746855
rs112891767
HG00114 >hsa-mir-553 TTTAAGACGGTGAGATTTTGTT TTTTAAGACGGTGAGATTTTGT
ATTTTAAGACGGTGAGATTTTG TATTTTAAGACGGTGAGATTTT
TTATTTTAAGACGGTGAGATTT 100746814 rs190622705

HG00114 >hsa-mir-553 TTTAAGACGGTGAGATTTTGTT TTTTAAGACGGTGAGATTTTGT
ATTTTAAGACGGTGAGATTTTG TATTTTAAGACGGTGAGATTTT
TTATTTTAAGACGGTGAGATTT 100746814 rs190622705 100746848 .

HG00114 >hsa-mir-553 TTTAAGACGGTGAGATTTTGTT TTTTAAGACGGTGAGATTTTGT
ATTTTAAGACGGTGAGATTTTG TATTTTAAGACGGTGAGATTTT
TTATTTTAAGACGGTGAGATTT 100746814 rs190622705 100746855
rs112891767
HG00114 >hsa-mir-553 TTTAAGACGGTGAGATTTTGTT TTTTAAGACGGTGAGATTTTGT
ATTTTAAGACGGTGAGATTTTG TATTTTAAGACGGTGAGATTTT
TTATTTTAAGACGGTGAGATTT 100746814 rs190622705 100746848 .
100746855 rs112891767
HG00114 >hsa-mir-1229TTTGGGGGAGAGTGTGGGCTGG AGGGTTTGGGGGAGAGTGTGGG
TAGGGTTTGGGGGAGAGTGTGG GTTTGGGGGAGAGTGTGGGCTG
GGTTTGGGGGAGAGTGTGGGCT 179225324 rs2291418

HG00114 >hsa-mir-548ad AAAACGACAATGACTTTTGCAC
ACTTTTGCACCAATCTAATAC CTTTTGCACCAATCTAATAC
GCAAAAACGACAATGACTTTTG CAAAAACGACAATGACTTTTGC 35696519
rs62143301
HG00114 >hsa-mir-3671AATAAGGACTAGTCTGCAGTGA TTTATTTCTATCAAATAAGGAC
TTTTATTTCTATCAAATAAGGA GGACTAGTCTGCAGTGATAT
AAATAAGGACTAGTCTGCAGTG 65523519 rs521188

HG00114 >hsa-mir-646 AGCAGCTGCCTCTGAGGCCTCA CTGAGGCCTCAGGCTCAGTGGC
CTCTGAGGCCTCAGGCTCAGTG GCCTCTGAGGCCTCAGGCTCAG
TCTGAGGCCTCAGGCTCAGTGG 58883534 rs6513496

HG00114 >hsa-mir-149 AGGGAGGGAGGGACGGGGGCTG GGGCTGTGCTGGGGCAGCCGGA
GAGGGAGGGAGGGACGGGGGCT GGGACGGGGGCTGTGCTGGGGC
GGGAGGGAGGGACGGGGGCTGT 241395503 rs2292832

HG00114 >hsa-mir-658 AGGTCGGTTGGTCGGTCGGGAA GTCGGTTGGTCGGTCGGGAACG
TAGGTCGGTTGGTCGGTCGGGA . .

HG00114 >hsa-mir-412 CGTCCGTATCCGCTGCAG CCGTCCGTATCCGCTGCAG
TCACCTGGTCCACTGGCCGTCC ACCTGGTCCACTGGCCGTCCGT
CACCTGGTCCACTGGCCGTCCG 101531854 rs61992671

HG00114 >hsa-mir-3180-4 CTCCGGATGCCAGTCCCTCATC
GGAGGGTGAAGCCTCCGGATGC AGCGGAGGGTGAAGCCTCCGGA

CTGGCCTGGTCGCGCTGTGGCT GAGCGGAGGGTGAAGCCTCCGG 15248720
rs75000738
HG00114 >hsa-mir-4268CTCTCAGGATGTGATGTCACCT CCTCTCAGGATGTGATGTCACC
GCTCCTCCTCTCAGGATGTGAT TCCTCTCAGGATGTGATGTCAC
CTCCTCCTCTCAGGATGTGATG 220771223 rs4674470

HG00114 >hsa-mir-3151CTGATCCCACACCCCACCTGTC TGATCCCACACCCCACCTGTCA
GATCCCACACCCCACCTGTCAC GGGCATCCCACCTGATCCCACA
TCCCACCTGATCCCACACCCCA 104166902 rs35605502

HG00114 >hsa-mir-658 G TAGGTCGGTTGGTCGGTCGGGA .

HG00114 >hsa-mir-658 G GTCCGTTGGTCGGTCGGGAACG G
TCCGTTGGTCGGTCGGGAACGA .

HG00114 >hsa-mir-320eGAAAAGCTGGGTTGAGAAGGT AAAAGCTGGGTTGAGAAGGT
GGAAAAGCTGGGTTGAGAAGGT GGGAAAAGCTGGGTTGAGAAGG rs10423365

HG00114 >hsa-mir-1343GCCCCTCCTGGGGCCCGCACTC CCCCTCCTGGGGCCCGCACTCT
GGGGCCCGCACTCTCGCTCTGG CCCTCCTGGGGCCCGCACTCTC
TGGGGCCCGCACTCTCGCTCTG 34963416 rs2986407

HG00114 >hsa-mir-3180-4 GGAGGGTGAAGCCTCCGGATGC
GGTGAAGCCTCCGGATGCCAGT GCGGAGGGTGAAGCCTCCGGAT
AGCGGAGGGTGAAGCCTCCGGA GCCTGGTCGCGCTGTGGCGAAG 15248798
rs183853838
HG00114 >hsa-mir-3180-4 GGAGGGTGAAGCCTCCGGATGC
GGTGAAGCCTCCGGATGCCAGT GCGGAGGGTGAAGCCTCCGGAT
AGCGGAGGGTGAAGCCTCCGGA CTGGCCTGGTCGCGCTGTGGCT 15248720
rs75000738 15248798 rs183853838
HG00114 >hsa-mir-1227GGCATTTGACCCCGTGCCACCC AGGCATTTGACCCCGTGCCACC
AGGCATTTGACCCCGTGCCACC TGACCCCGTGCCACCCTTTTCC
ATTTGACCCCGTGCCACCCTTT 2234093 rs190788838

HG00114 >hsa-mir-580 TATTTGAGAATGATGAATCATT TGAATCATTAGGTTCCGGTCAG
ATGAATCATTAGGTTCCGGTCA TTTGAGAATGATGAATCATTAG
GAGAATGATGAATCATTAGGTT 36148057 rs115089112

HG00114 >hsa-mir-3118-1 TGAAAATTCTTCTAGTGTG ATGAAAATTCTTCTAGTGTG
TGCATTATGAAAATTCTTCTAG TTATGAAAATTCTTCTAGTGTG
CTGCATTATGAAAATTCTTCTA 142667330 rs76132421

HG00114 >hsa-mir-558 TGAGCTGCTGTACCAAAATACC GCTGCTGTACCAAAATACCACA
TGCTGTACCAAAATACCACAAA CTGAGCTGCTGTACCAAAATAC
GAGCTGCTGTACCAAAATACCA 32757230 rs72089144

HG00114 >hsa-mir-320c-1 AAAAGCTGGGTTGAGAGGGTAG

AGCTGGGTTGAGAGGGTAGGAA CTGGGTTGAGAGGGTAGGAAAA
AGGGTAGGAAAAAAATGATGTA 19263542 .

HG00114 >hsa-mir-202 AAAGAGGTATAGGGCATGGGAA AAGAGGTATAGGGCATGGGAAA
GGGAAAACGGGGCGGTCGGGTC TAAAGAGGTATAGGGCATGGGA 135061112
rs12355840
HG00114 >hsa-mir-520hAAAGTGCTTCCCTTTAGAGTTA rs148716001 74

HG00114 >hsa-mir-548ap AACAAAAACCACAATTACTTTT
CAAAAACCACAATTACTTTTTA CAATTACTTTTTACTGACCTAA rs4414449

HG00114 >hsa-mir-548ap AACAAAAACCACAATTACTTTT
CAAAAACCACAATTACTTTTTA TTACTTTTTACTGACCTAAAGA rs4577031

HG00114 >hsa-mir-548ap AACAAAAACCACAATTACTTTT
CAAAAACCACAATTACTTTTTA CAATTACTTTTTACTGACCTAA rs4414449
86368959 rs4577031
HG00114 >hsa-mir-1255b-2 AACCACTTTCTTTGCTCATCCG
AAACCACTTTCTTTGCTCATCC CTTTCTTTGCTCATCCGTAAGG rs79639536

HG00114 >hsa-mir-548ad AACGACAATGACTTTTGCACCA
GGCAAAAACGACAATGACTTTT TGGCAAAAACGACAATGACTTT
AAAAACGACAATGACTTTTGCA 35696519 rs62143301

HG00114 >hsa-mir-3166AACGCAGACAATGCCTACTGGC AGACAATGCCTACTGGCCTAAG
ATGCCTACTGGCCTAAGAAAAA CAATGCCTACTGGCCTAAGAAA 87909673
rs35854553
HG00114 >hsa-mir-3118-1 ACTGCATTATGAAAATTCTTCT
ATTATGAAAATTCTTCTAGTGT GCATTATGAAAATTCTTCTAGT
CTGCATTATGAAAATTCTTCTA 142667330 rs76132421

HG00114 >hsa-mir-605 AGAGAAGGCACTATGAGATTTA GGCACTATGAGATTTAGAACCA
CAGAGAAGGCACTATGAGATTT GAGAAGGCACTATGAGATTTAG 53059406
rs2043556
HG00114 >hsa-mir-642bAGATACATTTGGAGAGGGACCC TTGGAGAGGGACCCTCCCAACT
TTTGGAGAGGGACCCTCCCAAC ATACATTTGGAGAGGGACCCTC 46178217
rs111664333
HG00114 >hsa-mir-3180-4 AGCGGAGGGTGAAGCCTCCGGA
CGGAGGGTGAAGCCTCCGGATG GAGCGGAGGGTGAAGCCTCCGG
GCGGAGGGTGAAGCCTCCGGAT 15248798 rs183853838

HG00114 >hsa-mir-3180-4 AGCGGAGGGTGAAGCCTCCGGA
CGGAGGGTGAAGCCTCCGGATG GAGCGGAGGGTGAAGCCTCCGG
GCGGAGGGTGAAGCCTCCGGAT 15248720 rs75000738 15248798

HG00114 >hsa-mir-30d AGCTTTCAGTCAGATGTTTGCT GGCTAAGCTTTCAGTCAGATGT
GCTAAGCTTTCAGTCAGATGTT TTCAGTCAGATGTTTGCTGCTA 135817150

.
HG00114 >hsa-mir-519a-2 AGGAAAGTGCATCCTTTTAGAG
AGTGCATCCTTTTAGAGGGTTA GGAAAGTGCATCCTTTTAGAGG
GAAAGGAAAGTGCATCCTTTTA 54265670 .

HG00114 >hsa-mir-646 AGGAAGCAGCTGCCTCTGAGGC GCTGCCTCTGAGGCCTCAGGCT
CTGAGGCCTCAGGCTCAGTGGC TCTGAGGCCTCAGGCTCAGTGG 58883534
rs6513496
HG00114 >hsa-mir-630 AGTATTCTGTACCAGGGAAGGT ACCTAGTATTCTGTACCAGGGA
CCAGGGAAGGTAGTTCTTAACT CAGGGAAGGTAGTTCTTAACTA 72879653
rs113971639
HG00114 >hsa-mir-3686AGTGATCTGTAAGAGAAAGTAA TCTGTAAGAGAAAGTAAATGAA
GTAAGAGAAAGTAAATGAAAGA ACAGTGATCTGTAAGAGAAAGT 130496365
rs6997249
HG00114 >hsa-mir-513cATAAATTTCACCTTTCTGAGAA TTTCTGAGAAGAGTAATGTACA
CCTTTCTGAGAAGAGTAATGTA TTTCACCTTTCTGAGAAGAGTA 146271303
rs145416750
HG00114 >hsa-mir-3144ATACCTGTTCAGTCTCTTTAAA TTCAGTCTCTTTAAAGTGTAGT
CCTGTTCAGTCTCTTTAAAGTG TGTTCAGTCTCTTTAAAGTGTA 120336384
rs67106263
HG00114 >hsa-mir-3144ATACCTGTTCAGTCTCTTTAAA TTCAGTCTCTTTAAAGTGTAGT
CTGTTCAGTCTCTTTAAAGTGT TATACCTGTTCAGTCTCTTTAA 120336327
rs68035463 120336384
HG00114 >hsa-mir-3144ATACCTGTTCGGTCTCTTTAAA CTGTTCGGTCTCTTTAAAGTGT
GTTCGGTCTCTTTAAAGTGTAG TGTTCGGTCTCTTTAAAGTGTA 120336327
rs68035463
HG00114 >hsa-mir-642aATTTGGAGAGGGAACCTCCCAA AGACACATTTGGAGAGGGAACC
ACACATTTGGAGAGGGAACCTC CACATTTGGAGAGGGAACCTCC 46178217
rs111664333
HG00114 >hsa-mir-548ac CAAAAACCGGCAATTACTTTTG
GGCAAAAACCGGCAATTACTTT TTACTTTTGCACTAACCTAATA
ACCGGCAATTACTTTTGCACTA 117102649 rs1414273

HG00114 >hsa-mir-412 CACCTGGTCCACTGGCCGTCCG ACCTGGTCCACTGGCCGTCCGT
CTGGCCGTCCGTATCCGCTGCA TCACCTGGTCCACTGGCCGTCC 101531854
rs61992671
HG00114 >hsa-mir-515-1 CAGAGTGCCTTCTTTTGGAGCA
GAGTGCCTTCTTTTGGAGCATT TGCCTTCTTTTGGAGCATTACT
GTGCCTTCTTTTGGAGCATTAC 54182326 rs374576826

HG00114 >hsa-mir-1227CATTTGACCCCGTGCCACCCTT ATTTGACCCCGTGCCACCCTTT
AGGCATTTGACCCCGTGCCACC GACCCCGTGCCACCCTTTTCCC 2234093
rs190788838
HG00114 >hsa-mir-1908CCACCGGCCGCCGGCTCCGCCC CCGCCGGCTCCGCCCCGGCCCC
GGCCGCCGGCTCCGCCCCGGCC CGGCCGCCGGCTCCGCCCCGGC 61582708
rs174561
HG00114 >hsa-mir-3151CCACCTGATCCCACACCCCACC CACCTGATCCCACACCCCACCT

CCCACCTGATCCCACACCCCAC TGATCCCACACCCCACCTGTCA 104166902 rs35605502
HG00114 >hsa-mir-1343CCCCTCCTGGGGCCCGCACTCT CCCTCCTGGGGCCCGCACTCTC CCTGGGGCCCGCACTCTCGCTC TGGGGCCCGCACTCTCGCTCTG 34963416 rs2986407
HG00114 >hsa-mir-1229CCTCTCACCACTGCCCTCCCAC GACACCCTCTCACCACTGCCCT ACCCTCTCACCACTGCCCTCCC CTCTCACCACTGCCCTCCCACA 179225324 rs2291418
HG00114 >hsa-mir-4268CCTCTCAGGATGTGATGTCACC CTCCTCTCAGGATGTGATGTCA CTCCTCCTCTCAGGATGTGATG rs4674470

HG00114 >hsa-mir-4254CCTGGAGATACTCCACCATCTC AGATACTCCACCATCTCCCCCA GGAGATACTCCACCATCTCCCC rs12731294

HG00114 >hsa-mir-3117CTCATATAGTGCCAGGTGTTTT GACTCATATAGTGCCAGGTGTT TCATATAGTGCCAGGTGTTTTG ATAAGACTCATATAGTGCCAGG 67094171 rs12402181
HG00114 >hsa-mir-196a-2 CTCGGCAACAAGAAACTGTCTG CAAGAAACTGTCTGAGTTACAT CAACAAGAAACTGTCTGAGTTA ACAAGAAACTGTCTGAGTTACA 54385599 rs11614913

HG00114 >hsa-mir-486-2 CTCGGCGCAGCTCAGTACAGGA AGGGCCTCGGCGCAGCTCAGTA TCGGCGCAGCTCAGTACAGGAT GGGCCTCGGCGCAGCTCAGTAC 41518007 .

HG00114 >hsa-mir-3615CTCTCTCGGCTCCTCGCGGCTC GGCTCCTCGCGGCTCGCGGCGG CGGCTCCTCGCGGCTCGCGGCG TCGGCTCCTCGCGGCTCGCGGC 72744798 rs745666
HG00114 >hsa-mir-888 CTCTTTGGGTGAAGGAAGGCTC CTGACACCTCTTTGGGTGAAGG GACTGACACCTCTTTGGGTGAA CCTCTTTGGGTGAAGGAAGGCT 145076355 rs143634721
HG00114 >hsa-mir-1304CTGTAGCATCGAACCCCTGGGC GAACCCCTGGGCTCAAGTGATT CTCACTGTAGCATCGAACCCCT CGAACCCCTGGGCTCAAGTGAT 93466866 rs2155248
HG00114 >hsa-mir-3922CTGTGGGACTTCTGGCCTTGAC ACCTGTGGGACTTCTGGCCTTG GGGACTTCTGGCCTTGACTTGA TGGGACTTCTGGCCTTGACTTG 104985443 rs61938575
HG00114 >hsa-mir-2117CTGTTCTCTTTGCCAAGGACAG GCTGTTCTCTTTGCCAAGGACA TCTCTTTGCCAAGGACAGATCT TGTTCTCTTTGCCAAGGACAGA 41522213 rs7207008
HG00114 >hsa-mir-940 GAAGGCAGGGCCCC-GCTCCCC G CCC-GCTCCCCGGGCCTGACCC rs35356504

HG00114 >hsa-mir-4274GACCCAGCAGTCCCTCCCCCTG CCCAGCAGTCCCTCCCCCTGCA TGACCCAGCAGTCCCTCCCCCT TCAGGTGACCCAGCAGTCCCTC 7461769 rs12512664
HG00114 >hsa-mir-3188GAGGCTTTGTGCGGATACGGGG GAGAGGCTTTGTGCGGATACGG

GCGGATACGGGGCTGGAGGCCT rs7247237

HG00114 >hsa-mir-3188GAGGCTTTGTGCGGATACGGGG GGAGAGGCTTTGTGCGGATACG GAGAGGCTTTGTGCGGATACGG rs7247767

HG00114 >hsa-mir-3188GAGGCTTTGTGCGGATACGGGG GAGAGGCTTTGTGCGGATACGG GGAGAGGCTTTGTGCGGATACG rs7247237 18392913 rs7247767

HG00114 >hsa-mir-629 GAGGTTCTCCCAACGTAAGCCC AGGTTCTCCCAACGTAAGCCCA TCTCCCAACGTAAGCCCAGCCC CAGGAGGTTCTCCCAACGTAAG 70371761 rs377691713

HG00114 >hsa-mir-222 GCAGCTACATCTGGCTACTGGG TACTGGGTCTCTGATGGCATCT GCTACTGGGTCTCTGATGGCAT CTGGCTACTGGGTCTCTGATGG 45606504 rs191727254

HG00114 >hsa-mir-500bGCAGTGCACCCAGGCAAGGATT CACCCAGGCAAGGATTCTGCGA AGGCAAGGATTCTGCGAGGGGG TGCAGTGCACCCAGGCAAGGAT 49775351 rs151318590

HG00114 >hsa-mir-3180-4 GCGGAGGGTGAAGCCTCCGGAT CGCTGGCCTGGTCGCGCTGTGG TCGCTGGCCTGGTCGCGCTGTG AAGCCTCCGGATGCCAGTCCCT 15248720 rs75000738

HG00114 >hsa-mir-2682GGACACCTCTTCAGCGCTGTCT CAGCGCTGTCTTCCCTGCCTCT TTCAGCGCTGTCTTCCCTGCCT CACCTCTTCAGCGCTGTCTTCC 98510847 rs74904371

HG00114 >hsa-mir-149 GGGAGGGAGGGACGGGGGCTGT GGAGGGACGGGGGCTGTGCTGG AGGGACGGGGGCTGTGCTGGGG GAGGAGGGAGGGAGGGACGGGG 241395503 rs2292832

HG00114 >hsa-mir-1197GTAGGACACATGGTCTACTTCT ACACATGGTCTACTTCTTCTCA ACATGGTCTACTTCTTCTCAAT TAGGACACATGGTCTACTTCTT 101491923 rs141611518

HG00114 >hsa-mir-27a GTGTTCACAGTGGCTAAGTTCC TCGTGTTCACAGTGGCTAAGTT AGTGGCTAAGTTCCGCCCCCCA CACAGTGGCTAAGTTCCGCCCC 13947292 rs895819

HG00114 >hsa-mir-3936TAAGGGGTGTATGGCAGATGCA TTCTGGTAAGGGGTGTATGGCA CACCCGACAGATGCACTTGGCA TGTATGGCAGATGCACCCGACA 131701279 rs367805

HG00114 >hsa-mir-644aTATAGTGTGGCTTTCTTAGAGC TTAGAGCAAAGATGGTTCCCTA TGTGGCTTTCTTAGAGCAAAGA CTTAGAGCAAAGATGGTTCCCT 33054160 rs181082862

HG00114 >hsa-mir-499aTCACAGCAAGTCTGTGCTGCTT CGTCCCTACGCTGCCTGGGCAG GCTGCTTCCCGTCCCTACGCTG TCCCTACGCTGCCTGGGCAGGG 33578202 rs140486571

HG00114 >hsa-mir-558 TCCTGAGCTGCTGTACCAAAAT TTCCTGAGCTGCTGTACCAAAA GAGCTGCTGTACCAAAATACCA CCTGAGCTGCTGTACCAAAATA 32757230 rs72089144

HG00114 >hsa-mir-3671TCTATCAAATAAGGACTAGTCT AAATAAGGACTAGTCTGCAGTG TTTATTTCTATCAAATAAGGAC CAAATAAGGACTAGTCTGCAGT 65523519

rs521188
HG00114 >hsa-mir-1273h TGCTGCAGACTCGACCTCCCAG
TGCAGACTCGACCTCCCAGGCT CTGCAGACTCGACCTCCCAGGC
AGACTCGACCTCCCAGGCTTAA 24214486 .

HG00114 >hsa-mir-590 TGTAATTTTATGTATAAGCTAG AATCTGTAATTTTATGTATAAG
GTATAAGCTAGTCTCTGATTGA TTTTATGTATAAGCTAGTCTCT 73605546
rs189727189
HG00114 >hsa-mir-3909TGTCCTCTA-GGCCTGCAGTCT TA-GGCCTGCAGTCTCATGGGA
#NAME? CCTGCAGTCTCATGGGAGAGTG 35731712 rs34874675

HG00114 >hsa-mir-2053TGTTAATTAAACCTCTATTTAC ACTTTAAGTGTTAATTAAACCT
TTTAAGTGTTAATTAAACCTCT TTAAGTGTTAATTAAACCTCTA 113655752
rs10505168
HG00114 >hsa-mir-585 TTGGGCGTATCTGTATGCTAGG TATCTGTATGCTAGGGCTGCCG
TGGGCGTATCTGTATGCTAGGG GCGTATCTGTATGCTAGGGCTG 168690612
rs62376934
HG00114 >hsa-mir-1303TTTAGAGACGGGGTCTTGCTCT TAGAGACGGGGTCTTGCTCTGT
TTAGAGACGGGGTCTTGCTCTG ACGGGGTCTTGCTCTGTTGCCA 154065383
rs75538180
HG00114 >hsa-mir-580 TTTGAGAATGATGAATCATTAG GATGAATCATTAGGTTCCGGTC
AATGATGAATCATTAGGTTCCG AGAATGATGAATCATTAGGTTC 36148057
rs115089112
HG00115 >hsa-mir-3144AAGGGGACCAAAGAGATATATA TAAGGGGACCAAAGAGATATAT
TTAAGGGGACCAAAGAGATATA TTTAAGGGGACCAAAGAGATAT
ATACACTTTAAGGGGACCAAAG 120336327 rs68035463

HG00115 >hsa-mir-3125AGAATGGATAGAGGAAGCTGTG GAGGAAGCTGTGGAGAGAACTC
AGAGGAAGCTGTGGAGAGAACT GCTGTGGAGAGAACTCACGGTG
GGAAGCTGTGGAGAGAACTCAC 12877501 rs78852835

HG00115 >hsa-mir-577 AGTGAAGAGTAGATAAAATATT GTAGATAAAATATTGGTACCTG
GAAGAGTAGATAAAATATTGGT AAGAGTAGATAAAATATTGGTA
AGTAGATAAAATATTGGTACCT 115577997 rs34115976

HG00115 >hsa-mir-1908CCGCGGCGGGGACGGCGATTGG GCGGCGGGGACGGCGATTGGTC
CGCGGCGGGGACGGCGATTGGT CGGGGACGGCGATTGGTCCGTA
GGGATGCCGCGGCGGGGACGGC 61582708 rs174561

HG00115 >hsa-mir-1273h CCTGGGAGGTCAAGGCTGTAGT
TGGGAGGTCAAGGCTGTAGTGT ATTGCTTGAGCCTGGGAGGTCA
GCCTGGGAGGTCAAGGCTGTAG TTGAGCCTGGGAGGTCAAGGCT 24214486 .

HG00115 >hsa-mir-3144TAAGGGGACCAAAGAGATATAT TTAAGGGGACCAAAGAGATATA
AAGGGGACCAAAGAGATATATA CTACACTTTAAGGGGACCAAAG
TTTAAGGGGACCAAAGAGATAT 120336384 rs67106263

HG00115 >hsa-mir-3144TAAGGGGACCAAAGAGATATAT AAGGGGACCAAAGAGATATATA TTAAGGGGACCAAAGAGATATA ATACACTTTAAGGGGACCAAAG TTTAAGGGGACCAAAGAGATAT 120336327 rs68035463 120336384 rs67106263

HG00115 >hsa-mir-3199-1 TGCCTTAGGAGAAAGTTTCTGG CCGGGACTGCCTTAGGAGAAAG CTTAGGAGAAAGTTTCTGGAAG GCCTTAGGAGAAAGTTTCTGGA TGACTCCCGGGACTGCCTTAGG 28316591 rs78805657

HG00115 >hsa-mir-3199-1 TGCCTTAGGAGAAAGTTTCTGG CCGGGACTGCCTTAGGAGAAAG CTTAGGAGAAAGTTTCTGGAAG GCCTTAGGAGAAAGTTTCTGGA TGACTCCCGGGACTGCCTTAGG 28316513 rs118160653 28316591 rs78805657

HG00115 >hsa-mir-553 TTTAAGACGGTGAGATTTTGTT TATTTTAAGACGGTGAGATTTT TTATTTTAAGACGGTGAGATTT TTTTATTTTAAGACGGTGAGAT AATTTTATTTTAAGACGGTGAG 100746814 rs190622705

HG00115 >hsa-mir-553 TTTAAGACGGTGAGATTTTGTT TTTTATTTTAAGACGGTGAGAT TTTTAAGACGGTGAGATTTTGT AATTTTATTTTAAGACGGTGAG TTATTTTAAGACGGTGAGATTT 100746814 rs190622705 100746855 rs112891767

HG00115 >hsa-mir-553 TTTTAAAACGGTGAGATTTTGT TATTTTAAAACGGTGAGATTTT TTTTATTTTAAAACGGTGAGAT TTTAAAACGGTGAGATTTTGTT ATTTTATTTTAAAACGGTGAGA 100746848 .

HG00115 >hsa-mir-553 TTTTAAAACGGTGAGATTTTGT TTTTATTTTAAAACGGTGAGAT TTTAAAACGGTGAGATTTTGTT TATTTTAAAACGGTGAGATTTT ATTTTATTTTAAAACGGTGAGA 100746848 . 100746855 rs112891767

HG00115 >hsa-mir-553 TTTTAAGACGGTGAGATTTTGT TATTTTAAGACGGTGAGATTTT TTTAAGACGGTGAGATTTTGTT TTTTATTTTAAGACGGTGAGAT ATTTTATTTTAAGACGGTGAGA 100746814 rs190622705 100746848 .

HG00115 >hsa-mir-553 TTTTAAGACGGTGAGATTTTGT TTTTATTTTAAGACGGTGAGAT TTTAAGACGGTGAGATTTTGTT TATTTTAAGACGGTGAGATTTT ATTTTATTTTAAGACGGTGAGA 100746814 rs190622705 100746848 . 100746855 rs112891767

HG00115 >hsa-mir-553 TTTTATTTTAAAACGGTGAGAT TTTTAAAACGGTGAGATTTTGT TTTAAAACGGTGAGATTTTGTT AATTTTATTTTAAAACGGTGAG TTATTTTAAAACGGTGAGATTT 100746855 rs112891767

HG00115 >hsa-mir-1307ACCGGACCTCGACCGGCTCGTC CGGACCTCGACCGGCTCGTCTG CCGGACCTCGACCGGCTCGTCT ATCTCGACCGGACCTCGACCGG AATCTCGACCGGACCTCGACCG 105154089 rs7911488

HG00115 >hsa-mir-486-2 ACTGAGCTGCCCCGAGCTGGGC CTGAGCTGCCCCGAGCTGGGCA CTGTACTGAGCTGCCCCGAGCT CCTGTACTGAGCTGCCCCGAGC GTACTGAGCTGCCCCGAGCTGG 41518007 .

HG00115 >hsa-mir-1269b AGCCATGCTACGGGCTTCTCTG
ACTGAGCCATGCTACGGGCTTC AGGTTTCTGGACTGAGCCATGC
TGAGGTTTCTGGACTGAGCCAT TTTCTGGACTGAGCCATGCTAC 12820632
rs12451747
HG00115 >hsa-mir-3135b AGCGAGTGCAGTGGTGCAGTCA
AGGCTGGAGCGAGTGCAGTGGT CTGGAGCGAGTGCAGTGGTGCA
CAGGCTGGAGCGAGTGCAGTGG CCAGGCTGGAGCGAGTGCAGTG 32717702
rs4285314
HG00115 >hsa-mir-3125AGCTGTGGAGAGAACTCACGGT AAGCTGTGGAGAGAACTCACGG
TAGAGGAAGCTGTGGAGAGAAC TGGATAGAGGAAGCTGTGGAGA
AGGAAGCTGTGGAGAGAACTCA 12877501 rs78852835

HG00115 >hsa-mir-1227AGGCGGTGGTGGGCACTGCTGG TGGTGGGCACTGCTGGGGTGGG
TGGGGCCAGGCGGTGGTGGGCA CGGTGGTGGGCACTGCTGGGGT
GGTGGGCACTGCTGGGGTGGGC 2234093 rs190788838

HG00115 >hsa-mir-548ap AGTAATTGCAGTCTTTGTCATT
AAGTAATTGCAGTCTTTGTCAT AAAGTAATTGCAGTCTTTGTCA
AAAAGTAATTGCAGTCTTTGTC CAAAAGTAATTGCAGTCTTTGT 86368898
rs4414449
HG00115 >hsa-mir-548ap AGTAATTGCAGTCTTTGTCATT
AAGTAATTGCAGTCTTTGTCAT AAAGTAATTGCAGTCTTTGTCA
AAAAGTAATTGCAGTCTTTGTC CAAAAGTAATTGCAGTCTTTGT 86368898
rs4414449 86368959 rs4577031
HG00115 >hsa-mir-548ap AGTAATTGCGGTCTTTGTCATT
AAGTAATTGCGGTCTTTGTCAT AAAGTAATTGCGGTCTTTGTCA
AAAAGTAATTGCGGTCTTTGTC CAAAAGTAATTGCGGTCTTTGT 86368959
rs4577031
HG00115 >hsa-mir-196a-2 AGTTTCATGTTGTTGGGATTGA
AGGTAGTTTCATGTTGTTGGGA TAGTTTCATGTTGTTGGGATTG
GTAGTTTCATGTTGTTGGGATT GGTAGTTTCATGTTGTTGGGAT 54385599
rs11614913
HG00115 >hsa-mir-1908GGGGACGGCGATTGGTCCGTAT GCGGGGACGGCGATTGGTCCGT
GGCGGGGACGGCGATTGGTCCG CGGGGACGGCGATTGGTCCGTA
TGCCGCGGCGGGGACGGCGATT 61582708 rs174561

HG00115 >hsa-mir-548h-3 GTAATCGTGGTTTTTGTCATTG
TGCAAAAGTAATCGTGGTTTTT AGTAATCGTGGTTTTTGTCATT
AAGTAATCGTGGTTTTTGTCAT TGGTGCAAAAGTAATCGTGGTT 13446924
rs9913045
HG00115 >hsa-mir-3144TAAGGGGACCAAAGAGATATAT TTAAGGGGACCAAAGAGATATA
TTTAAGGGGACCAAAGAGATAT TACACTTTAAGGGGACCAAAGA
AAATACACTTTAAGGGGACCAA 120336327 rs68035463

HG00115 >hsa-mir-3144TAAGGGGACCAAAGAGATATAT TTAAGGGGACCAAAGAGATATA
TTTAAGGGGACCAAAGAGATAT TACACTTTAAGGGGACCAAAGA

AACTACACTTTAAGGGGACCAA 120336384 rs67106263

HG00115 >hsa-mir-3144TAAGGGGACCAAAGAGATATAT TTAAGGGGACCAAAGAGATATA TTTAAGGGGACCAAAGAGATAT TACACTTTAAGGGGACCAAAGA AAATACACTTTAAGGGGACCAA 120336327 rs68035463 120336384 rs67106263

HG00115 >hsa-mir-629 TACGTTGGGAGAACTTTTACGG TTACGTTGGGAGAACTTTTACG GTTTACGTTGGGAGAACTTTTA TTTACGTTGGGAGAACTTTTAC GGTTTACGTTGGGAGAACTTTT 70371794 .

HG00115 >hsa-mir-629 TACGTTGGGAGAACTTTTATGG TTACGTTGGGAGAACTTTTATG TTTACGTTGGGAGAACTTTTAT TGGGTTTACGTTGGGAGAACTT GTTTACGTTGGGAGAACTTTTA 70371761 rs377691713

HG00115 >hsa-mir-629 TACGTTGGGAGAACTTTTATGG TTACGTTGGGAGAACTTTTATG TTTACGTTGGGAGAACTTTTAT TGGGTTTACGTTGGGAGAACTT GTTTACGTTGGGAGAACTTTTA 70371761 rs377691713 70371794 .

HG00115 >hsa-mir-888 TACTCAAAAAGCTTTCAGTCAC TCTACTCAAAAAGCTTTCAGTC TGCTCTACTCAAAAAGCTTTCA GCTCTACTCAAAAAGCTTTCAG GGCAGTGCTCTACTCAAAAAGC 145076355 rs143634721

HG00115 >hsa-mir-3199-1 TAGGAGAAAGTTTCTGGAAGTT TTAGGAGAAAGTTTCTGGAAGT TGCCTTAGGAGAAAGTTTCTGG TCCAGGGACTGCCTTAGGAGAA TGACTCCAGGGACTGCCTTAGG 28316513 rs118160653

HG00115 >hsa-mir-3199-1 TAGGAGAAAGTTTCTGGAAGTT TTAGGAGAAAGTTTCTGGAAGT TGCCTTAGGAGAAAGTTTCTGG TCCCGGGACTGCCTTAGGAGAA TGACTCCCGGGACTGCCTTAGG 28316591 rs78805657

HG00115 >hsa-mir-3199-1 TAGGAGAAAGTTTCTGGAAGTT TTAGGAGAAAGTTTCTGGAAGT TGCCTTAGGAGAAAGTTTCTGG TCCCGGGACTGCCTTAGGAGAA TGACTCCCGGGACTGCCTTAGG 28316513 rs118160653 28316591 rs78805657

HG00115 >hsa-mir-3199-2 TAGGAGAAAGTTTCTGGAATGT TTAGGAGAAAGTTTCTGGAATG TGCCTTAGGAGAAAGTTTCTGG TCCCAGGGACTGCCTTAGGAGA TGACTCCCAGGGACTGCCTTAG 28316591 rs78805657

HG00115 >hsa-mir-3199-2 TAGGAGAAAGTTTCTGGAATGT TTAGGAGAAAGTTTCTGGAATG TGCCTTAGGAGAAAGTTTCTGG TCCCAGGGACTGCCTTAGGAGA TGACTCCCAGGGACTGCCTTAG 28316591 rs80166589

HG00115 >hsa-mir-3199-2 TAGGAGAAAGTTTCTGGAATGT TTAGGAGAAAGTTTCTGGAATG TGCCTTAGGAGAAAGTTTCTGG TCCCAGGGACTGCCTTAGGAGA TGACTCCCAGGGACTGCCTTAG 28316591 rs78805657 28316591 rs80166589

HG00115 >hsa-mir-449cTAGGCAGTGTATTGCTAGCGGC TCAGATAGGCAGTGTATTGCTA

TGTCAGATAGGCAGTGTATTGC TGTGTCAGATAGGCAGTGTATT
TGGGATGTGTCAGATAGGCAGT 54468166 rs75661995

HG00115 >hsa-mir-564 TCAGCAGGCAACATGGCCGAGA TGTCAGCAGGCAACATGGCCGA
GTCAGCAGGCAACATGGCCGAG TGCCAGGCACGGTGTCAGCAGG
GTGTCAGCAGGCAACATGGCCG 44903433 .

HG00115 >hsa-mir-564 TCAGCAGGCAACATGGCCGAGA TGTCAGCAGGCAACATGGCCGA
GTCAGCAGGCAACATGGCCGAG GTGTCAGCAGGCAACATGGCCG
GGTGTCAGCAGGCAACATGGCC 44903434 rs2292181

HG00115 >hsa-mir-222 TCAGTAGCCAGTGTAGATCCTG TGGCTCAGTAGCCAGTGTAGAT
TTGGCTCAGTAGCCAGTGTAGA TCATTGGCTCAGTAGCCAGTGT
TACCCTCATTGGCTCAGTAGCC 45606504 rs191727254

HG00115 >hsa-mir-515-1 TCCAAAAGAAAGCACTTTCTGT
TCTCCAAAAGAAAGCACTTTCT TTCTCCAAAAGAAAGCACTTTC
TCATTCTCCAAAAGAAAGCACT TGCAGTCATTCTCCAAAAGAAA 54182326
rs374576826
HG00115 >hsa-mir-663aTCCCAGGCGGGGCGCCGCGGGA TCCGGCGTCCCAGGCGGGGCGC
TTCCGGCGTCCCAGGCGGGGCG GCGCCGCGGGACCTCCCTCGTG
GGCGCCGCGGGACCTCCCTCGT 26188880 .

HG00115 >hsa-mir-149 TCCGTGTCTTCACTCCCGTGCT TGGCTCCGTGTCTTCACTCCCG
TCTGGCTCCGTGTCTTCACTCC CCGTGTCTTCACTCCCGTGCTT
AGCTCTGGCTCCGTGTCTTCAC 241395503 rs2292832

HG00115 >hsa-mir-618 TCCTTCTGAGTGTAATTACGTA TGTCCTTCTGAGTGTAATTACG
TTGTCCTTCTGAGTGTAATTAC TACTTGTCCTTCTGAGTGTAAT
GTCCTTCTGAGTGTAATTACGT 81329536 rs2682818

HG00115 >hsa-mir-3183TCGGAGTCGCTCGGAGCAGCCA TCTCGGAGTCGCTCGGAGCAGC
TCTCTCGGAGTCGCTCGGAGCA TCTGCCCTGCCTCTCTCGGAGT
TGCCCTGCCTCTCTCGGAGTCG 925764 rs2663345

HG00115 >hsa-mir-4305TCTGGGTTCTTAGAGGCCTAAT TTCTGGGTTCTTAGAGGCCTAA
GTTCTGGGTTCTTAGAGGCCTA TCCAGTTCTGGGTTCTTAGAGG
CAGTTCTGGGTTCTTAGAGGCC 40238175 rs67976778

HG00115 >hsa-mir-1255a TGAGCAAAGAAAGTAGATTTTT
GCAAAGAAAGTAGATTTTTTAG TCAAGGATGAGCAAAGAAAGTA
GAGCAAAGAAAGTAGATTTTTT TCTCAAGGATGAGCAAAGAAAG 102251501
rs28664200
HG00115 >hsa-mir-3151TGATGGGTGGGGCAATGGGATC TGGGTGGGGCAATGGGATCAGG
TGGGGCAATGGGATCAGGTGCC GGGGTGATGGGTGGGGCAATGG
GGGTGATGGGTGGGGCAATGGG 104166902 rs35605502

HG00115 >hsa-mir-3156-2 TGCAGAAGAAAGATCTGGAAGT
GAAAGATCTGGAAGTGGGAGAC GAAGAAAGATCTGGAAGTGGGA
GCAGAAGAAAGATCTGGAAGTG CAGAAGAAAGATCTGGAAGTGG 14830215
rs113478966
HG00115 >hsa-mir-3156-3 TGCAGAAGAAAGATCTGGAAGT
GCAGAAGAAAGATCTGGAAGTG GAAGAAAGATCTGGAAGTGGGA
GAAAGATCTGGAAGTGGGAGAC AGAAGAAAGATCTGGAAGTGGG 14778721
rs2747232
HG00115 >hsa-mir-1254-2 TGGAAGCTGGAGCCTGCAGTGA
TGAGCCTGGAAGCTGGAGCCTG GAAGCTGGAGCCTGCAGTGAGC
GGAAGCTGGAGCCTGCAGTGAG GCCTGGAAGCTGGAGCCTGCAG 23682383
rs200793185
HG00115 >hsa-mir-3652TGGAGGCGGCTCCTGCGATCGA GGAGGCGGCTCCTGCGATCGAA
TGGGGGGTGGAGGCGGCTCCTG GTGGAGGCGGCTCCTGCGATCG
GGTGGAGGCGGCTCCTGCGATC 104324266 rs17797090

HG00115 >hsa-mir-1273h TGGGAGGTCAAGGCTGTAGTGT
TGAGCCTGGGAGGTCAAGGCTG TTGAGCCTGGGAGGTCAAGGCT
TGCTTGAGCCTGGGAGGTCAAG TTGCTTGAGCCTGGGAGGTCAA 24214486 .

HG00115 >hsa-mir-564 TGTCAGCAGGCAACATGGCCGA TGCCAGGCACGGTGTCAGCAGG
GTGTCAGCAGGCAACATGGCCG GGTGTCAGCAGGCAACATGGCC
GCACGGTGTCAGCAGGCAACAT 44903433 . 44903434 rs2292181

HG00115 >hsa-mir-323bTGTCCGTGGTGAGTTCGCATTA TTGTCCGTGGTGAGTTCGCATT
TACTCGGAGGGAGGTTGTCCGT TCGGAGGGAGGTTGTCCGTGGT
AGGTTGTCCGTGGTGAGTTCGC 101522556 rs56103835

HG00115 >hsa-mir-27a TTAGCTGCTTGTGAGCAGGGTT TGAGGAGCAGGGCTTAGCTGCT
GCTTAGCTGCTTGTGAGCAGGG GGCTTAGCTGCTTGTGAGCAGG
GAGGAGCAGGGCTTAGCTGCTT 13947296 rs11671784

HG00115 >hsa-mir-642bTTCCCTCTCCAAATGTGTCTTG TTGGGAGGTTCCCTCTCCAAAT
TGGGAGGTTCCCTCTCCAAATG GAGTTGGGAGGTTCCCTCTCCA
GTTGGGAGGTTCCCTCTCCAAA 46178217 rs111664333

HG00115 >hsa-mir-4277TTCTGAGCACAGTACACTGGGC TCGAGGCAGTTCTGAGCACAGT
TGGGTCGAGGCAGTTCTGAGCA GTTCTGAGCACAGTACACTGGG
GCAGTTCTGAGCACAGTACACT 1708902 rs115200817

HG00115 >hsa-mir-4277TTCTGAGCACAGTACACTGGGC TCGAGGCAGTTCTGAGCACAGT
TGGGTCGAGGCAGTTCTGAGCA TTGGGTCGAGGCAGTTCTGAGC
GTTCTGAGCACAGTACACTGGG 1708983 rs12523324

HG00115 >hsa-mir-4277TTCTGAGCACAGTACACTGGGC TCGAGGCAGTTCTGAGCACAGT
TGGGTCGAGGCAGTTCTGAGCA TTGGGTCGAGGCAGTTCTGAGC
GTTCTGAGCACAGTACACTGGG 1708902 rs115200817 1708983

rs12523324
HG00115 >hsa-mir-138-1 TTGTGAATCAGGCCGCTGCCAA TGTTGTGAATCAGGCCGCTGCC TGGTGTTGTGAATCAGGCCGCT TGGGGCAGCTGGTGTTGTGAAT TGGCATGGTGTGGTGGGGCAGC 44155749 rs374104338
HG00115 >hsa-mir-553 TTTAAAACGGTGAGATTTTGTT TTTTAAAACGGTGAGATTTTGT ATTTTAAAACGGTGAGATTTTG TATTTTAAAACGGTGAGATTTT TTATTTTAAAACGGTGAGATTT 100746848 .

HG00115 >hsa-mir-553 TTTAAAACGGTGAGATTTTGTT TTTTAAAACGGTGAGATTTTGT ATTTTAAAACGGTGAGATTTTG TATTTTAAAACGGTGAGATTTT TTATTTTAAAACGGTGAGATTT 100746855 rs112891767

HG00115 >hsa-mir-553 TTTAAAACGGTGAGATTTTGTT TTTTAAAACGGTGAGATTTTGT ATTTTAAAACGGTGAGATTTTG TATTTTAAAACGGTGAGATTTT TTATTTTAAAACGGTGAGATTT 100746848 . 100746855 rs112891767
HG00115 >hsa-mir-553 TTTAAGACGGTGAGATTTTGTT TTTTAAGACGGTGAGATTTTGT ATTTTAAGACGGTGAGATTTTG TATTTTAAGACGGTGAGATTTT TTATTTTAAGACGGTGAGATTT 100746814 rs190622705

HG00115 >hsa-mir-553 TTTAAGACGGTGAGATTTTGTT TTTTAAGACGGTGAGATTTTGT ATTTTAAGACGGTGAGATTTTG TATTTTAAGACGGTGAGATTTT TTATTTTAAGACGGTGAGATTT 100746814 rs190622705 100746848 .

HG00115 >hsa-mir-553 TTTAAGACGGTGAGATTTTGTT TTTTAAGACGGTGAGATTTTGT ATTTTAAGACGGTGAGATTTTG TATTTTAAGACGGTGAGATTTT TTATTTTAAGACGGTGAGATTT 100746814 rs190622705 100746855 rs112891767
HG00115 >hsa-mir-553 TTTAAGACGGTGAGATTTTGTT TTTTAAGACGGTGAGATTTTGT ATTTTAAGACGGTGAGATTTTG TATTTTAAGACGGTGAGATTTT TTATTTTAAGACGGTGAGATTT 100746814 rs190622705 100746848 . 100746855 rs112891767
HG00115 >hsa-mir-149 AGGGAGGGAGGGACGGGGGCTG GGGCTGTGCTGGGGCAGCCGGA GAGGGAGGGAGGGACGGGGGCT GGGAGGGAGGGACGGGGGCTGT GGACGGGGGCTGTGCTGGGGCA 241395503 rs2292832

HG00115 >hsa-mir-658 AGGTCGGTTGGTCGGTCGGGAA GTCGGTTGGTCGGTCGGGAACG TAGGTCGGTTGGTCGGTCGGGA . .

HG00115 >hsa-mir-3180-4 CTCCGGATGCCAGTCCCTCATC GGAGGGTGAAGCCTCCGGATGC AGCGGAGGGTGAAGCCTCCGGA CTGGCCTGGTCGCGCTGTGGCT GAGCGGAGGGTGAAGCCTCCGG 15248720 rs75000738
HG00115 >hsa-mir-4268CTCTCAGGATGTGATGTCACCT CCTCTCAGGATGTGATGTCACC GCTCCTCCTCTCAGGATGTGAT TCCTCTCAGGATGTGATGTCAC TCCTCCTCTCAGGATGTGATGT 220771223 rs4674470

HG00115 >hsa-mir-3151CTGATCCCACACCCCACCTGTC TGATCCCACACCCCACCTGTCA
GATCCCACACCCCACCTGTCAC GGGCATCCCACCTGATCCCACA
TCCCACCTGATCCCACACCCCA 104166902 rs35605502

HG00115 >hsa-mir-412 CTTCACCTGGTTCACTAGCCGT ACCTGGTTCACTAGCCGTCCGT
CTGGTTCACTAGCCGTCCGTAT TGTACTTCACCTGGTTCACTAG
GTACTTCACCTGGTTCACTAGC 101531849 .

HG00115 >hsa-mir-658 TAGGTCGGTTGGTCGGTCGGGA .

HG00115 >hsa-mir-658 GTCCGTTGGTCGGTCGGGAACG G
TCCGTTGGTCGGTCGGGAACGA .

HG00115 >hsa-mir-320eGAAAAGCTGGGTTGAGAAGGT AAAAGCTGGGTTGAGAAGGT
GGAAAAGCTGGGTTGAGAAGGT GGGAAAAGCTGGGTTGAGAAGG rs10423365

HG00115 >hsa-mir-1343GCCCCTCCTGGGGCCCGCACTC CCCCTCCTGGGGCCCGCACTCT
GGGGCCCGCACTCTCGCTCTGG CCCTCCTGGGGCCCGCACTCTC
TGGGGCCCGCACTCTCGCTCTG 34963416 rs2986407

HG00115 >hsa-mir-3180-4 GGAGGGTGAAGCCTCCGGATGC
GGTGAAGCCTCCGGATGCCAGT GCGGAGGGTGAAGCCTCCGGAT
AGCGGAGGGTGAAGCCTCCGGA GCCTGGTCGCGCTGTGGCGAAG 15248798
rs183853838
HG00115 >hsa-mir-3180-4 GGAGGGTGAAGCCTCCGGATGC
GGTGAAGCCTCCGGATGCCAGT GCGGAGGGTGAAGCCTCCGGAT
AGCGGAGGGTGAAGCCTCCGGA CTGGCCTGGTCGCGCTGTGGCT 15248720
rs75000738 15248798 rs183853838
HG00115 >hsa-mir-1227GGCATTTGACCCCGTGCCACCC AGGCATTTGACCCCGTGCCACC
AGGCATTTGACCCCGTGCCACC TGACCCCGTGCCACCCTTTTCC
ATTTGACCCCGTGCCACCCTTT 2234093 rs190788838

HG00115 >hsa-mir-580 TATTTGAGAATGATGAATCATT TGAATCATTAGGTTCCGGTCAG
ATGAATCATTAGGTTCCGGTCA TTTGAGAATGATGAATCATTAG
GAGAATGATGAATCATTAGGTT 36148057 rs115089112

HG00115 >hsa-mir-3118-1 TGAAAATTCTTCTAGTGTG ATGAAAATTCTTCTAGTGTG
TGCATTATGAAAATTCTTCTAG ATTATGAAAATTCTTCTAGTGT
TTATGAAAATTCTTCTAGTGTG 142667330 rs76132421

HG00115 >hsa-mir-558 TGAGCTGCTGTACCAAAATACC GCTGCTGTACCAAAATACCACA
TGCTGTACCAAAATACCACAAA CTGAGCTGCTGTACCAAAATAC
GAGCTGCTGTACCAAAATACCA 32757230 rs72089144

HG00115 >hsa-mir-637 TGGCTAAGGTGTTGGCTCGGGC TGGCTAAGGTGTTGGCTCGGGC
.

HG00115 >hsa-mir-548a-1 AAAACTGGCAATTACTTTTGCA
AAACTGGCAATTACTTTTGCAC TGGCAATTACTTTTGCACCAAA
AACTGGCAATTACTTTTGCACC 18572056 rs12197631

HG00115 >hsa-mir-320c-1 AAAAGCTGGGTTGAGAGGGTAG
AGCTGGGTTGAGAGGGTAGGAA CTGGGTTGAGAGGGTAGGAAAA
AGGGTAGGAAAAAAATGATGTA 19263542 .

HG00115 >hsa-mir-651 AAAAGGAAAGTGTATCCTAAAA GGAAAGTGTATCCTAAAAGGCA
TGTATCCTAAAAGGCAATGACA AAAGGAAAGTGTATCCTAAAAG 8095036
rs111336920
HG00115 >hsa-mir-202 AAAGAGGTATAGGGCATGGGAA AAGAGGTATAGGGCATGGGAAA
GGGAAAACGGGGCGGTCGGGTC TAAAGAGGTATAGGGCATGGGA 135061112
rs12355840
HG00115 >hsa-mir-520hAAAGTGCTTCCCTTTAGAGTTA rs148716001 74

HG00115 >hsa-mir-944 AAATTATTGTACATCGGATGAG GTACATCGGATGAGCTGTGTCT
GAAATTATTGTACATCGGATGA ATTATTGTACATCGGATGAGCT 189547735
rs75715827
HG00115 >hsa-mir-548ap AACAAAAACCACAATTACTTTT
CAAAAACCACAATTACTTTTTA CAATTACTTTTTACTGACCTAA rs4414449

HG00115 >hsa-mir-548ap AACAAAAACCACAATTACTTTT
CAAAAACCACAATTACTTTTTA TTACTTTTTACTGACCTAAAGA rs4577031

HG00115 >hsa-mir-548ap AACAAAAACCACAATTACTTTT
CAAAAACCACAATTACTTTTTA CAATTACTTTTTACTGACCTAA rs4414449
86368959 rs4577031
HG00115 >hsa-mir-1255b-2 AACCACTTTCTTTGCTCATCCG
AAACCACTTTCTTTGCTCATCC CTTTCTTTGCTCATCCGTAAGG rs79639536

HG00115 >hsa-mir-3166AACGCAGACAATGCCTACTGGC AGACAATGCCTACTGGCCTAAG
ATGCCTACTGGCCTAAGAAAAA CAATGCCTACTGGCCTAAGAAA 87909673
rs35854553
HG00115 >hsa-mir-548al AACGGCAGTGACTTTTGTACCA
TGGCAAAAACGGCAGTGACTTT TAAAAGTAATGGCAAAAACGGC
AAAAGTAATGGCAAAAACGGCA 74110353 rs515924

HG00115 >hsa-mir-423 AAGCTCGGTCTGAGGCCCCTCA AGGCCCCTCAGTCTTGCTTCCT
TCTGAGGCCCCTCAGTCTTGCT GTCTGAGGCCCCTCAGTCTTGC 28444183
rs6505162
HG00115 >hsa-mir-449cACAGTTGCTAGTTGCACTCCTC AACAGTTGCTAGTTGCACTCCT
GTTGCTAGTTGCACTCCTCTCT GTTGCACTCCTCTCTGTTGCAT 54468166
rs75661995
HG00115 >hsa-mir-138-1 ACGGCTACTTCACAACACCAGG
ACTTCACAACACCAGGGCCACA GGCTACTTCACAACACCAGGGC rs374104338

HG00115 >hsa-mir-3118-1 ACTGCATTATGAAAATTCTTCT
ATTATGAAAATTCTTCTAGTGT GCATTATGAAAATTCTTCTAGT
CTGCATTATGAAAATTCTTCTA 142667330 rs76132421

HG00115 >hsa-mir-605 AGAGAAGGCACTATGAGATTTA GGCACTATGAGATTTAGAACCA
CAGAGAAGGCACTATGAGATTT GAGAAGGCACTATGAGATTTAG 53059406
rs2043556
HG00115 >hsa-mir-642bAGATACATTTGGAGAGGGACCC TTGGAGAGGGACCCTCCCAACT
TTTGGAGAGGGACCCTCCCAAC ATACATTTGGAGAGGGACCCTC 46178217
rs111664333
HG00115 >hsa-mir-3180-4 AGCGGAGGGTGAAGCCTCCGGA
CGGAGGGTGAAGCCTCCGGATG GAGCGGAGGGTGAAGCCTCCGG
GCGGAGGGTGAAGCCTCCGGAT 15248798 rs183853838

HG00115 >hsa-mir-3180-4 AGCGGAGGGTGAAGCCTCCGGA
CGGAGGGTGAAGCCTCCGGATG GAGCGGAGGGTGAAGCCTCCGG
GCGGAGGGTGAAGCCTCCGGAT 15248720 rs75000738 15248798
rs183853838
HG00115 >hsa-mir-30d AGCTTTCAGTCAGATGTTTGCT GGCTAAGCTTTCAGTCAGATGT
GCTAAGCTTTCAGTCAGATGTT TTCAGTCAGATGTTTGCTGCTA 135817150
.
HG00115 >hsa-mir-519a-2 AGGAAAGTGCATCCTTTTAGAG
AGTGCATCCTTTTAGAGGGTTA GGAAAGTGCATCCTTTTAGAGG
GAAAGGAAAGTGCATCCTTTTA 54265670 .

HG00115 >hsa-mir-3167AGGATTTCAGAAATACTGGTGT GGATTTCAGAAATACTGGTGTC
CAGGAAGGATTTCAGAAATACT AAGGATTTCAGAAATACTGGTG 126858392
rs670637
HG00115 >hsa-mir-3167AGGATTTCAGAAATACTGGTGT AAGGATTTCAGAAATACTGGTG
TTCAGGAAGGATTTCAGAAATA GGATTTCAGAAATACTGGTGTC 126858406
rs634171
HG00115 >hsa-mir-3167AGGATTTCAGAAATACTGGTGT AAGGATTTCAGAAATACTGGTG
CTCAGGAAGGATTTCAGAAATA GGATTTCAGAAATACTGGTGTC 126858392
rs670637 126858406 rs634171
HG00115 >hsa-mir-1250AGGCCACATTTTCCAGCCCATT CTTCCAGAACCCTCTGAAGTGG
CCATTCAACCTTCCAGAACCCT AGCCCATTCAACCTTCCAGAAC 79107017
.
HG00115 >hsa-mir-630 AGTATTCTGTACCAGGGAAGGT ACCTAGTATTCTGTACCAGGGA
CCAGGGAAGGTAGTTCTTAACT GGAAGGTAGTTCTTAACTATGT 72879653
rs113971639
HG00115 >hsa-mir-3686AGTGATCTGTAAGAGAAAGTAA TCTGTAAGAGAAAGTAAATGAA
GTAAGAGAAAGTAAATGAAAGA CAGTGATCTGTAAGAGAAAGTA 130496365
rs6997249
HG00115 >hsa-mir-513cATAAATTTCACCTTTCTGAGAA TTTCTGAGAAGAGTAATGTACA
CCTTTCTGAGAAGAGTAATGTA TTTCACCTTTCTGAGAAGAGTA 146271303
rs145416750
HG00115 >hsa-mir-323bATACACGGTCGACCTCTTTTCG TACACGGTCGACCTCTTTTCGG

ACACGGTCGACCTCTTTTCGGT rs56103835

HG00115 >hsa-mir-3144ATACCTGTTCAGTCTCTTTAAA TTCAGTCTCTTTAAAGTGTAGT CCTGTTCAGTCTCTTTAAAGTG TGTTCAGTCTCTTTAAAGTGTA 120336384 rs67106263

HG00115 >hsa-mir-3144ATACCTGTTCAGTCTCTTTAAA TTCAGTCTCTTTAAAGTGTAGT CTGTTCAGTCTCTTTAAAGTGT TATACCTGTTCAGTCTCTTTAA 120336327 rs68035463 120336384 rs67106263

HG00115 >hsa-mir-3144ATACCTGTTCGGTCTCTTTAAA CTGTTCGGTCTCTTTAAAGTGT GTTCGGTCTCTTTAAAGTGTAG TGTTCGGTCTCTTTAAAGTGTA 120336327 rs68035463

HG00115 >hsa-mir-642aATTTGGAGAGGGAACCTCCCAA AGACACATTTGGAGAGGGAACC ACACATTTGGAGAGGGAACCTC CACATTTGGAGAGGGAACCTCC 46178217 rs111664333

HG00115 >hsa-mir-122 CAAATGCCATTATCACACTAAA AATGCCATTATCACACTAAATA CCATTATCACACTAAATAGCTA TATCAAATGCCATTATCACACT 56118358 rs41292412

HG00115 >hsa-mir-515-1 CAGAGTGCCTTCTTTTGGAGCA GAGTGCCTTCTTTTGGAGCATT TGCCTTCTTTTGGAGCATTACT GTGCCTTCTTTTGGAGCATTAC 54182326 rs374576826

HG00115 >hsa-mir-4265CAGCTGTGGGCTCAACTCTGGG ATCTCTGCAGCTGTGGGCTCAA GATCTCTGCAGCTGTGGGCTCA CTGCAGCTGTGGGCTCAACTCT 109757963 rs4676066

HG00115 >hsa-mir-1227CATTTGACCCCGTGCCACCCTT ATTTGACCCCGTGCCACCCTTT AGGCATTTGACCCCGTGCCACC GACCCCGTGCCACCCTTTTCCC 2234093 rs190788838

HG00115 >hsa-mir-1908CCACCGGCCGCCGGCTCCGCCC CCGCCGGCTCCGCCCCGGCCCC GGCCGCCGGCTCCGCCCCGGCC CGGCCGCCGGCTCCGCCCCGGC 61582708 rs174561

HG00115 >hsa-mir-3151CCACCTGATCCCACACCCCACC CACCTGATCCCACACCCCACCT CCCACCTGATCCCACACCCCAC TGATCCCACACCCCACCTGTCA 104166902 rs35605502

HG00115 >hsa-mir-1343CCCCTCCTGGGGCCCGCACTCT CCCTCCTGGGGCCCGCACTCTC CCTGGGGCCCGCACTCTCGCTC TGGGGCCCGCACTCTCGCTCTG 34963416 rs2986407

HG00115 >hsa-mir-1343CCCTCCTGGGGCGCGCACTCTC CTCCTGGGGCGCGCACTCTCGC GGGGCGCGCACTCTCGCTCTGG TGGGGCGCGCACTCTCGCTCTG 34963445 .

HG00115 >hsa-mir-4254CCTGGAGATACTCCACCATCTC AGATACTCCACCATCTCCCCCA GGAGATACTCCACCATCTCCCC rs12731294

HG00115 >hsa-mir-1343CTCCTGGGGCGCGCACTCTCGC CCCTCCTGGGGCGCGCACTCTC GGGGCGCGCACTCTCGCTCTGG TGGGGCGCGCACTCTCGCTCTG 34963416 rs2986407 34963445 .

HG00115 >hsa-mir-196a-2 CTCGGCAACAAGAAACTGTCTG ACTCGGCAACAAGAAACTGTCT CAACAAGAAACTGTCTGAGTTA

ACAAGAAACTGTCTGAGTTACA 54385599 rs11614913

HG00115 >hsa-mir-486-2 CTCGGCGCAGCTCAGTACAGGA
AGGGCCTCGGCGCAGCTCAGTA GGGCCTCGGCGCAGCTCAGTAC
TCGGCGCAGCTCAGTACAGGAT 41518007 .

HG00115 >hsa-mir-3615CTCTCTCGGCTCCTCGCGGCTC GGCTCCTCGCGGCTCGCGGCGG
CGGCTCCTCGCGGCTCGCGGCG TCGGCTCCTCGCGGCTCGCGGC 72744798
rs745666
HG00115 >hsa-mir-888 CTCTTTGGGTGAAGGAAGGCTC CTGACACCTCTTTGGGTGAAGG
GACTGACACCTCTTTGGGTGAA CCTCTTTGGGTGAAGGAAGGCT 145076355
rs143634721
HG00115 >hsa-mir-2117CTGTTCTCTTTGCCAAGGACAG TAGCTGTTCTCTTTGCCAAGGA
TCTCTTTGCCAAGGACAGATCT TGTTCTCTTTGCCAAGGACAGA 41522213
rs7207008
HG00115 >hsa-mir-940 GAAGGCAGGGCCCC-GCTCCCC G CCC-
GCTCCCCGGGCCTGACCC rs35356504

HG00115 >hsa-mir-1307GACTCGGCGTGGCGTCGGTCGT CGTGGCGTCGGTCGTGGTAGAT
ATCGACTCGGCGTGGCGTCGGT CGACTCGGCGTGGCGTCGGTCG 105154089
rs7911488
HG00115 >hsa-mir-3188GAGGCTTTGTGCGGATACGGGG GAGAGGCTTTGTGCGGATACGG
GCGGATACGGGGCTGGAGGCCT rs7247237

HG00115 >hsa-mir-3188GAGGCTTTGTGCGGATACGGGG GAGAGGCTTTGTGCGGATACGG
GGAGAGGCTTTGTGCGGATACG rs7247767

HG00115 >hsa-mir-3188GAGGCTTTGTGCGGATACGGGG GAGAGGCTTTGTGCGGATACGG
GGAGAGGCTTTGTGCGGATACG rs7247237 18392913 rs7247767

HG00115 >hsa-mir-629 GAGGTTCTCCCAACGTAAGCCC AGGTTCTCCCAACGTAAGCCCA
TCTCCCAACGTAAGCCCAGCCC CAGGAGGTTCTCCCAACGTAAG 70371761
rs377691713
HG00115 >hsa-mir-629 GAGGTTCTCCCAACGTAAGCCC AGGAGGTTCTCCCAACGTAAGC
GGAGGTTCTCCCAACGTAAGCC TCTCCCAACGTAAGCCCAGCCC 70371794
.
HG00115 >hsa-mir-629 GAGGTTCTCCCAACGTAAGCCC AGGTTCTCCCAACGTAAGCCCA
TCTCCCAACGTAAGCCCAGCCC CCCAACGTAAGCCCAGCCCCTC 70371761
rs377691713 70371794 .
HG00115 >hsa-mir-222 GCAGCTACATCTGGCTACTGGG TACTGGGTCTCTGATGGCATCT
GCTACTGGGTCTCTGATGGCAT CTGGCTACTGGGTCTCTGATGG 45606504
rs191727254
HG00115 >hsa-mir-151bGCCCTCGAGGAGCTCACAGTCT GAGCTCACAGTCTAGACAAACA
GGAGCTCACAGTCTAGACAAAC TGCCCTCGAGGAGCTCACAGTC 100575818
.
HG00115 >hsa-mir-3180-4 GCGGAGGGTGAAGCCTCCGGAT
CGCTGGCCTGGTCGCGCTGTGG TCGCTGGCCTGGTCGCGCTGTG

AAGCCTCCGGATGCCAGTCCCT 15248720 rs75000738

HG00115 >hsa-mir-149 GGGAGGGAGGGACGGGGGCTGT GGAGGGACGGGGGCTGTGCTGG AGGGACGGGGGCTGTGCTGGGG GAGGAGGGAGGGAGGGACGGGG 241395503 rs2292832

HG00115 >hsa-mir-1197GTAGGACACATGGTCTACTTCT ACACATGGTCTACTTCTTCTCA TAGGACACATGGTCTACTTCTT ACATGGTCTACTTCTTCTCAAT 101491923 rs141611518

HG00115 >hsa-mir-27a GTGTTCACAGTGGCTAAGTTCC CGTGTTCACAGTGGCTAAGTTC TGTTCACAGTGGCTAAGTTCCG GTCGTGTTCACAGTGGCTAAGT 13947296 rs11671784

HG00115 >hsa-mir-3936TAAGGGGTGTATGGCAGATGCA CACCCGACAGATGCACTTGGCA GATGCACCCGACAGATGCACTT TTCTGGTAAGGGGTGTATGGCA 131701279 rs367805

HG00115 >hsa-mir-300 TATACAAGGGCAGACTCTCTCT TGATTATACAAGGGCAGACTCT ATTATACAAGGGCAGACTCTCT rs12894467

HG00115 >hsa-mir-412 TCACCTGGTTCACTAGCCGTCC TCACCTGGTTCACTAGCCGTCC ATGTACTTCACCTGGTTCACTA CTTCACCTGGTTCACTAGCCGT 101531849 .

HG00115 >hsa-mir-4268TCCTCCTCTCAGGATGTGATGT TCCTCCTCTCAGGATGTGATGT CTCCTCTCAGGATGTGATGTCA CTCCTCCTCTCAGGATGTGATG 220771223 rs4674470

HG00115 >hsa-mir-558 TCCTGAGCTGCTGTACCAAAAT TTCCTGAGCTGCTGTACCAAAA GAGCTGCTGTACCAAAATACCA CCTGAGCTGCTGTACCAAAATA 32757230 rs72089144

HG00115 >hsa-mir-639 TCGCTGCGGTTGCGAGCGCTGT CTATCGCTGCGGTTGCGAGCGC ATCGCTGCGGTTGCGAGCGCTG TAGTCTATCGCTGCGGTTGCGA 14640439 rs35149836

HG00115 >hsa-mir-1304TCTCACTGTAGCATCGAACCCC CTCACTGTAGCATCGAACCCCT TCGAACCCCTGGGCTCAAGTGA GAACCCCTGGGCTCAAGTGATT 93466866 rs2155248

HG00115 >hsa-mir-1273h TGCTGCAGACTCGACCTCCCAG TGCAGACTCGACCTCCCAGGCT CTGCAGACTCGACCTCCCAGGC AGACTCGACCTCCCAGGCTTAA 24214486 .

HG00115 >hsa-mir-3156-2 TGGCCCCCACTTCCAGATCTTT CCCCCACTTCCAGATCTTTCTC ACTTCCAGATCTTTCTCTCTGT CCCCACTTCCAGATCTTTCTCT 14830215 rs113478966

HG00115 >hsa-mir-2053TGTTAATTAAACCTCTATTTAC TTTAAGTGTTAATTAAACCTCT ACTTTAAGTGTTAATTAAACCT GTTAATTAAACCTCTATTTACA 113655752 rs10505168

HG00115 >hsa-mir-585 TTGGGCGTATCTGTATGCTAGG TGGGCGTATCTGTATGCTAGGG TATCTGTATGCTAGGGCTGCCG GCGTATCTGTATGCTAGGGCTG 168690612 rs62376934

HG00115 >hsa-mir-1303TTTAGAGACGGGGTCTTGCTCT TAGAGACGGGGTCTTGCTCTGT

ACGGGGTCTTGCTCTGTTGCCA GGGTCTTGCTCTGTTGCCAGGC 154065347 rs142414368
HG00115 >hsa-mir-1303TTTAGAGACGGGGTCTTGCTCT TAGAGACGGGGTCTTGCTCTGT TTAGAGACGGGGTCTTGCTCTG ACGGGGTCTTGCTCTGTTGCCA 154065383 rs75538180
HG00115 >hsa-mir-1303TTTAGAGACGGGGTCTTGCTCT TAGAGACGGGGTCTTGCTCTGT TTAGAGACGGGGTCTTGCTCTG ACGGGGTCTTGCTCTGTTGCCA 154065347 rs142414368 154065383 rs75538180
HG00115 >hsa-mir-580 TTTGAGAATGATGAATCATTAG GATGAATCATTAGGTTCCGGTC AATGATGAATCATTAGGTTCCG AGAATGATGAATCATTAGGTTC 36148057 rs115089112
HG00115 >hsa-mir-133bTTTGGTCCCCTTCAACCAGCTA TGGTCCCCTTCAACCAGCTACA CCTTCAACCAGCTACAGCAGGG AGAGGTTTGGTCCCCTTCAACC 52013832 rs374103744
HG00116 >hsa-mir-3144AAGGGGACCAAAGAGATATATA TAAGGGGACCAAAGAGATATAT TTAAGGGGACCAAAGAGATATA TTTAAGGGGACCAAAGAGATAT ATACACTTTAAGGGGACCAAAG 120336327 rs68035463

HG00116 >hsa-mir-3125AGAATGGATAGAGGAAGCTGTG GAGGAAGCTGTGGAGAGAACTC AGAGGAAGCTGTGGAGAGAACT GCTGTGGAGAGAACTCACGGTG GGAAGCTGTGGAGAGAACTCAC 12877501 rs78852835

HG00116 >hsa-mir-648 AGCGTGCAGGGCACTGGTGGGG CGTGCAGGGCACTGGTGGGGGC GCGTGCAGGGCACTGGTGGGGG GGGCACTGGTGGGGGCCGGGGC CTCCAAGCGTGCAGGGCACTGG 18463709 .

HG00116 >hsa-mir-577 AGTGAAGAGTAGATAAAATATT GTAGATAAAATATTGGTACCTG GAAGAGTAGATAAAATATTGGT AAGAGTAGATAAAATATTGGTA AGTAGATAAAATATTGGTACCT 115577997 rs34115976

HG00116 >hsa-mir-1908CCGCGGCGGGGACGGCGATTGG GCGGCGGGGACGGCGATTGGTC CGCGGCGGGGACGGCGATTGGT CGGGGACGGCGATTGGTCCGTA GGGGACGGCGATTGGTCCGTAT 61582708 rs174561

HG00116 >hsa-mir-1273h CCTGGGAGGTCAAGGCTGTAGT TGGGAGGTCAAGGCTGTAGTGT ATTGCTTGAGCCTGGGAGGTCA GCCTGGGAGGTCAAGGCTGTAG TTGAGCCTGGGAGGTCAAGGCT 24214486 .

HG00116 >hsa-mir-548ak GCAAAAGTAACTGCGGTTTTTG TGCAAAAGTAACTGCGGTTTTT CAAAAGTAACTGCGGTTTTTGA GTGCAAAAGTAACTGCGGTTTT rs7070684

HG00116 >hsa-mir-1268a GCCAGGCGTGGTGGTGGGGGCC AGCCAGGCGTGGTGGTGGGGGC .

HG00116 >hsa-mir-3144TAAGGGGACCAAAGAGATATAT TTAAGGGGACCAAAGAGATATA AAGGGGACCAAAGAGATATATA CTACACTTTAAGGGGACCAAAG

TTTAAGGGGACCAAAGAGATAT 120336384 rs67106263

HG00116 >hsa-mir-3144TAAGGGGACCAAAGAGATATAT AAGGGGACCAAAGAGATATATA
TTAAGGGGACCAAAGAGATATA ATACACTTTAAGGGGACCAAAG
TTTAAGGGGACCAAAGAGATAT 120336327 rs68035463 120336384
rs67106263

HG00116 >hsa-mir-4326TCTGCTGTTCCTCTGTCTCCCA TGGTCTGCTGTTCCTCTGTCTC
CTGGTCTGCTGTTCCTCTGTCT GCTGTTCCTCTGTCTCCCAGAC
TGCTGTTCCTCTGTCTCCCAGA 61918164 rs6062431

HG00116 >hsa-mir-412 TGGGGTACGGGGATGGATGGTC GGATGGATGGTCGACCAGTTGG
GATGGATGGTCGACCAGTTGGA TCGACCAGTTGGAAAGTAATTG
ACGGGGATGGATGGTCGACCAG 101531854 rs61992671

HG00116 >hsa-mir-412 TGGGGTACGGGGATGGATGGTC GGATGGATGGTCGACCAGTTGG
GGGTACGGGGATGGATGGTCGA GATGGATGGTCGACCAGTTGGA
TCGACCAGTTGGAAAGTAATTG 101531849 . 101531854 rs61992671

HG00116 >hsa-mir-744 TTGGGCAAGGTGCGGGGCTAGG TGGGCAAGGTGCGGGGCTAGGG
CGGGGCTAGGGCTAACAGCAGT GTGCGGGGCTAGGGCTAACAGC
CTAGGGCTAACAGCAGTCTTAC 11985275 .

HG00116 >hsa-mir-553 TTTAAGACGGTGAGATTTTGTT TATTTTAAGACGGTGAGATTTT
TTATTTTAAGACGGTGAGATTT TTTTATTTTAAGACGGTGAGAT
AATTTTATTTTAAGACGGTGAG 100746814 rs190622705

HG00116 >hsa-mir-553 TTTTAAAACGGTGAGATTTTGT TATTTTAAAACGGTGAGATTTT
TTTTATTTTAAAACGGTGAGAT TTTAAAACGGTGAGATTTTGTT
ATTTTATTTTAAAACGGTGAGA 100746848 .

HG00116 >hsa-mir-553 TTTTAAGACGGTGAGATTTTGT TATTTTAAGACGGTGAGATTTT
TTTAAGACGGTGAGATTTTGTT TTTTATTTTAAGACGGTGAGAT
ATTTTATTTTAAGACGGTGAGA 100746814 rs190622705 100746848 .

HG00116 >hsa-mir-486-2 ACTGAGCTGCCCCGAGCTGGGC
CTGAGCTGCCCCGAGCTGGGCA CTGTACTGAGCTGCCCCGAGCT
CCTGTACTGAGCTGCCCCGAGC GTACTGAGCTGCCCCGAGCTGG 41518007 .

HG00116 >hsa-mir-1269b AGCCATGCTACGGGCTTCTCTG
ACTGAGCCATGCTACGGGCTTC AGGTTTCTGGACTGAGCCATGC
TGAGGTTTCTGGACTGAGCCAT TTTCTGGACTGAGCCATGCTAC 12820632
rs12451747

HG00116 >hsa-mir-3135b AGCGAGTGCAGTGGTGCAGTCA
AGGCTGGAGCGAGTGCAGTGGT CTGGAGCGAGTGCAGTGGTGCA
CAGGCTGGAGCGAGTGCAGTGG CCAGGCTGGAGCGAGTGCAGTG 32717702
rs4285314

HG00116 >hsa-mir-3125AGCTGTGGAGAGAACTCACGGT AAGCTGTGGAGAGAACTCACGG

TAGAGGAAGCTGTGGAGAGAAC TGGATAGAGGAAGCTGTGGAGA
AGGAAGCTGTGGAGAGAACTCA 12877501 rs78852835

HG00116 >hsa-mir-612 AGGGCTTCTGAGCTCCTTAGCA GGGCTTCTGAGCTCCTTAGCAC
CAGGGCTTCTGAGCTCCTTAGC GCAGGGCTTCTGAGCTCCTTAG
GGCAGGGCTTCTGAGCTCCTTA 65211979 rs12803915

HG00116 >hsa-mir-548ap AGTAATTGCAGTCTTTGTCATT
AAGTAATTGCAGTCTTTGTCAT AAAGTAATTGCAGTCTTTGTCA
AAAAGTAATTGCAGTCTTTGTC CAAAAGTAATTGCAGTCTTTGT 86368898
rs4414449
HG00116 >hsa-mir-548ap AGTAATTGCAGTCTTTGTCATT
AAGTAATTGCAGTCTTTGTCAT AAAGTAATTGCAGTCTTTGTCA
AAAAGTAATTGCAGTCTTTGTC CAAAAGTAATTGCAGTCTTTGT 86368898
rs4414449 86368959 rs4577031
HG00116 >hsa-mir-548ap AGTAATTGCGGTCTTTGTCATT
AAGTAATTGCGGTCTTTGTCAT AAAGTAATTGCGGTCTTTGTCA
AAAAGTAATTGCGGTCTTTGTC CAAAAGTAATTGCGGTCTTTGT 86368959
rs4577031
HG00116 >hsa-mir-196a-2 AGTTTCATGTTGTTGGGATTGA
AGGTAGTTTCATGTTGTTGGGA TAGTTTCATGTTGTTGGGATTG
GTAGTTTCATGTTGTTGGGATT GGTAGTTTCATGTTGTTGGGAT 54385599
rs11614913
HG00116 >hsa-mir-548h-4 ATCGCGGTTTTTGTCATTACCT
CGGTTTTTGTCATTACCTTAAT AATCGCGGTTTTTGTCATTACC
CGCGGTTTTTGTCATTACCTTA AGTAATCGCGGTTTTTGTCATT 26906437
rs184537764
HG00116 >hsa-mir-548h-4 ATCGCGGTTTTTGTCATTACCT
CGGTTTTTGTCATTACCTTAAT AATCGCGGTTTTTGTCATTACC
CGCGGTTTTTGTCATTACCTTA AGTAATCGCGGTTTTTGTCATT 26906402
rs73235381 26906437 rs184537764
HG00116 >hsa-mir-548h-4 ATCGCGGTTTTTGTCATTACTT
CGGTTTTTGTCATTACTTTAAT AATCGCGGTTTTTGTCATTACT
CGCGGTTTTTGTCATTACTTTA AGTAATCGCGGTTTTTGTCATT 26906402
rs73235381
HG00116 >hsa-mir-1273d GAGGTTGAGGCTGCAGTGAGCC
TGAGGTTGAGGCTGCAGTGAGC CATGAGGTTGAGGCTGCAGTGA
CCATGAGGTTGAGGCTGCAGTG ATGAGGTTGAGGCTGCAGTGAG 10287824
rs150712502
HG00116 >hsa-mir-744 GGGCTAACAGCAGTCTTACTGA GCTAGGGCTAACAGCAGTCTTA
CTAGGGCTAACAGCAGTCTTAC GCTAGGGCTAACAGCAGTCTTA
CTAGGGCTAACAGCAGTCTTAC 11985275 .

HG00116 >hsa-mir-1908GGGGACGGCGATTGGTCCGTAT GCGGGGACGGCGATTGGTCCGT
GGCGGGGACGGCGATTGGTCCG CGGGGACGGCGATTGGTCCGTA
TGCCGCGGCGGGGACGGCGATT 61582708 rs174561

HG00116 >hsa-mir-548h-3 GTAATCGTGGTTTTTGTCATTG TGCAAAAGTAATCGTGGTTTTT AGTAATCGTGGTTTTTGTCATT AAGTAATCGTGGTTTTTGTCAT TGGTGCAAAAGTAATCGTGGTT 13446924 rs9913045

HG00116 >hsa-mir-573 GTGTAACTGATCAGGATCTACT TGTGTAACTGATCAGGATCTAC GATGTGTAACTGATCAGGATCT TGATGTGTAACTGATCAGGATC GTGATGTGTAACTGATCAGGAT 24521902 rs76014664

HG00116 >hsa-mir-3144TAAGGGGACCAAAGAGATATAT TTAAGGGGACCAAAGAGATATA TTTAAGGGGACCAAAGAGATAT TACACTTTAAGGGGACCAAAGA AAATACACTTTAAGGGGACCAA 120336327 rs68035463

HG00116 >hsa-mir-3144TAAGGGGACCAAAGAGATATAT TTAAGGGGACCAAAGAGATATA TTTAAGGGGACCAAAGAGATAT TACACTTTAAGGGGACCAAAGA AACTACACTTTAAGGGGACCAA 120336384 rs67106263

HG00116 >hsa-mir-3144TAAGGGGACCAAAGAGATATAT TTAAGGGGACCAAAGAGATATA TTTAAGGGGACCAAAGAGATAT TACACTTTAAGGGGACCAAAGA AAATACACTTTAAGGGGACCAA 120336327 rs68035463 120336384 rs67106263

HG00116 >hsa-mir-629 TACGTTGGGAGAACTTTTATGG TTACGTTGGGAGAACTTTTATG TTTACGTTGGGAGAACTTTTAT TGGGTTTACGTTGGGAGAACTT GTTTACGTTGGGAGAACTTTTA 70371761 rs377691713

HG00116 >hsa-mir-888 TACTCAAAAAGCTTTCAGTCAC TCTACTCAAAAAGCTTTCAGTC TGCTCTACTCAAAAAGCTTTCA GCTCTACTCAAAAAGCTTTCAG GGCAGTGCTCTACTCAAAAAGC 145076355 rs143634721

HG00116 >hsa-mir-3199-1 TAGGAGAAAGTTTCTGGAAGTT TTAGGAGAAAGTTTCTGGAAGT TGCCTTAGGAGAAAGTTTCTGG TCCAGGGACTGCCTTAGGAGAA TGACTCCAGGGACTGCCTTAGG 28316513 rs118160653

HG00116 >hsa-mir-449cTAGGCAGTGTATTGCTAGCGGC TCAGATAGGCAGTGTATTGCTA TGTCAGATAGGCAGTGTATTGC TGTGTCAGATAGGCAGTGTATT TGGGATGTGTCAGATAGGCAGT 54468166 rs75661995

HG00116 >hsa-mir-3922TCAAGGCCAGAGGTCCCACAAC TCAAGTCAAGGCCAGAGGTCCC GCCAGAGGTCCCACAACAGGGC GGCCAGAGGTCCCACAACAGGG GTCAAGGCCAGAGGTCCCACAA 104985443 rs61938575

HG00116 >hsa-mir-222 TCAGTAGCCAGTGTAGATCCTG TGGCTCAGTAGCCAGTGTAGAT TTGGCTCAGTAGCCAGTGTAGA TCATTGGCTCAGTAGCCAGTGT TACCCTCATTGGCTCAGTAGCC 45606504 rs191727254

HG00116 >hsa-mir-663aTCCCAGGCGGGGCGCCGCGGGA TCCGGCGTCCCAGGCGGGGCGC TTCCGGCGTCCCAGGCGGGGCG GCGCCGCGGGACCTCCCTCGTG GGCGCCGCGGGACCTCCCTCGT 26188880 .

HG00116 >hsa-mir-596 TCCGAAGCCTGCCCGGCCCCTC GCCTGCCCGGCCCCTCGGGAAC TCTCCGAAGCCTGCCCGGCCCC CTGCCCGGCCCCTCGGGAACCT CCTGCCCGGCCCCTCGGGAACC 1765425 rs61388742

HG00116 >hsa-mir-149 TCCGTGTCTTCACTCCCGTGCT TGGCTCCGTGTCTTCACTCCCG TCTGGCTCCGTGTCTTCACTCC CCGTGTCTTCACTCCCGTGCTT AGCTCTGGCTCCGTGTCTTCAC 241395500 rs71428439

HG00116 >hsa-mir-149 TCCGTGTCTTCACTCCCGTGCT TGGCTCCGTGTCTTCACTCCCG TCTGGCTCCGTGTCTTCACTCC CCGTGTCTTCACTCCCGTGCTT AGCTCTGGCTCCGTGTCTTCAC 241395503 rs2292832

HG00116 >hsa-mir-149 TCCGTGTCTTCACTCCCGTGCT TGGCTCCGTGTCTTCACTCCCG TCTGGCTCCGTGTCTTCACTCC CCGTGTCTTCACTCCCGTGCTT AGCTCTGGCTCCGTGTCTTCAC 241395500 rs71428439 241395503 rs2292832

HG00116 >hsa-mir-618 TCCTTCTGAGTGTAATTACGTA TGTCCTTCTGAGTGTAATTACG TTGTCCTTCTGAGTGTAATTAC TACTTGTCCTTCTGAGTGTAAT GTCCTTCTGAGTGTAATTACGT 81329536 rs2682818

HG00116 >hsa-mir-492 TCGAGGACCTGCGGGACAAGAT TACAGGACCATCGAGGACCTGC TACTACAGGACCATCGAGGACC TCCAGCCACTACTACAGGACCA GACCTGCGGGACAAGATTCTTG 95228179 rs200816308

HG00116 >hsa-mir-3183TCGGAGTCGCTCGGAGCAGCCA TCTCGGAGTCGCTCGGAGCAGC TCTCTCGGAGTCGCTCGGAGCA TCTGCCCTGCCTCTCTCGGAGT TGCCCTGCCTCTCTCGGAGTCG 925764 rs2663345

HG00116 >hsa-mir-4305TCTGGGTTCTTAGAGGCCTAAT TTCTGGGTTCTTAGAGGCCTAA GTTCTGGGTTCTTAGAGGCCTA TCCAGTTCTGGGTTCTTAGAGG CAGTTCTGGGTTCTTAGAGGCC 40238175 rs67976778

HG00116 >hsa-mir-1255a TGAGCAAAGAAAGTAGATTTTT GCAAAGAAAGTAGATTTTTTAG TCAAGGATGAGCAAAGAAAGTA GAGCAAAGAAAGTAGATTTTTT TCTCAAGGATGAGCAAAGAAAG 102251501 rs28664200

HG00116 >hsa-mir-1200TGAGCCATTCTGAGCCTCAATC TCCTGAGCCATTCTGAGCCTCA TCTCCTGAGCCATTCTGAGCCT TTCTCCTGAGCCATTCTGAGCC TGCTACTTCTCCTGAGCCATTC 36958995 rs180826747

HG00116 >hsa-mir-27a TGAGGAGCAGGGCTTAGCTGCT TTAGCTGCTTGTGAGCAGGGTC GAGGAGCAGGGCTTAGCTGCTT GGAGCAGGGCTTAGCTGCTTGT GAGCAGGGCTTAGCTGCTTGTG 13947292 rs895819

HG00116 >hsa-mir-3151TGATGGGTGGGGCAATGGGATC TGGGTGGGGCAATGGGATCAGG TGGGGCAATGGGATCAGGTGCC GGGGTGATGGGTGGGGCAATGG

GGGTGATGGGTGGGGCAATGGG 104166902 rs35605502

HG00116 >hsa-mir-548a-3 TGCAAAAGTAATTGCGAGTTTT
TCGGTGCAAAAGTAATTGCGAG TAGGTCGGTGCAAAAGTAATTG
TTAGGTCGGTGCAAAAGTAATT TATTAGGTCGGTGCAAAAGTAA 105496622 .

HG00116 >hsa-mir-548jTGCAAAAGTAATTGCGGTCTTT TGGTGCAAAAGTAATTGCGGTC
GCAAAAGTAATTGCGGTCTTTG GTGCAAAAGTAATTGCGGTCTT
TAGCTGGTGCAAAAGTAATTGC 26951185 rs4822739

HG00116 >hsa-mir-3156-3 TGCAGAAGAAAGATCTGGAAGT
GCAGAAGAAAGATCTGGAAGTG GAAGAAAGATCTGGAAGTGGGA
GAAAGATCTGGAAGTGGGAGAC AGAAGAAAGATCTGGAAGTGGG 14778721
rs2747232
HG00116 >hsa-mir-1200TGCTACTTCTCCTGAGCCATTC TGAGCCATTCTGAGCCTCAGTC
TACTTCTCCTGAGCCATTCTGA TCCTGAGCCATTCTGAGCCTCA
TTCTCCTGAGCCATTCTGAGCC 36959006 .

HG00116 >hsa-mir-1200TGCTACTTCTCCTGAGCCATTC TGAGCCATTCTGAGCCTCAGTC
TACTTCTCCTGAGCCATTCTGA TCCTGAGCCATTCTGAGCCTCA
TTCTCCTGAGCCATTCTGAGCC 36958995 rs180826747 36959006 .

HG00116 >hsa-mir-1254-2 TGGAAGCTGGAGCCTGCAGTGA
TGAGCCTGGAAGCTGGAGCCTG GAAGCTGGAGCCTGCAGTGAGC
GGAAGCTGGAGCCTGCAGTGAG GCCTGGAAGCTGGAGCCTGCAG 23682383
rs200793185
HG00116 >hsa-mir-378d-2 TGGACTTGGAGTCAGAAAACTT
GACTTGGAGTCAGAAAACTTTC GGACTTGGAGTCAGAAAACTTT
GAACACTGGACTTGGAGTCAGA TACAAGGAGAGAACACTGGACT 94928250
rs73692959
HG00116 >hsa-mir-1273h TGGGAGGTCAAGGCTGTAGTGT
TGAGCCTGGGAGGTCAAGGCTG TTGAGCCTGGGAGGTCAAGGCT
TGCTTGAGCCTGGGAGGTCAAG TTGCTTGAGCCTGGGAGGTCAA 24214486 .

HG00116 >hsa-mir-412 TGGGGTACGGGGATGGATGGTC TCGACCAGTTGGAAAGTAATTG
TGGTCGACCAGTTGGAAAGTAA TACGGGGATGGATGGTCGACCA
TGGATGGTCGACCAGTTGGAAA 101531854 rs61992671

HG00116 >hsa-mir-412 TGGGGTACGGGGATGGATGGTC TCGACCAGTTGGAAAGTAATTG
TGGTCGACCAGTTGGAAAGTAA TACGGGGATGGATGGTCGACCA
TGGATGGTCGACCAGTTGGAAA 101531849 . 101531854 rs61992671

HG00116 >hsa-mir-1227TGGTGGGCACTGCTGGGGTGGG TGGGGCCAGGCGGTGGTGGGCA
AGGCGGTGGTGGGCACTGCTGG GGTGGGCACTGCTGGGGTGGGC
GTGGTGGGCACTGCTGGGGTGG 2234093 rs190788838

HG00116 >hsa-mir-323bTGTCCGTGGTGAGTTCGCATTA TTGTCCGTGGTGAGTTCGCATT

TACTCGGAGGGAGGTTGTCCGT TCGGAGGGAGGTTGTCCGTGGT
AGGTTGTCCGTGGTGAGTTCGC 101522556 rs56103835

HG00116 >hsa-mir-642bTTCCCTCTCCAAATGTGTCTTG TTGGGAGGTTCCCTCTCCAAAT
TGGGAGGTTCCCTCTCCAAATG GAGTTGGGAGGTTCCCTCTCCA
GTTGGGAGGTTCCCTCTCCAAA 46178217 rs111664333

HG00116 >hsa-mir-4277TTCTGAGCACAGTACACTGGGC TCGAGGCAGTTCTGAGCACAGT
TGGGTCGAGGCAGTTCTGAGCA GTTCTGAGCACAGTACACTGGG
GCAGTTCTGAGCACAGTACACT 1708902 rs115200817

HG00116 >hsa-mir-4277TTCTGAGCACAGTACACTGGGC TCGAGGCAGTTCTGAGCACAGT
TGGGTCGAGGCAGTTCTGAGCA TTGGGTCGAGGCAGTTCTGAGC
GTTCTGAGCACAGTACACTGGG 1708983 rs12523324

HG00116 >hsa-mir-4277TTCTGAGCACAGTACACTGGGC TCGAGGCAGTTCTGAGCACAGT
TGGGTCGAGGCAGTTCTGAGCA TTGGGTCGAGGCAGTTCTGAGC
GTTCTGAGCACAGTACACTGGG 1708902 rs115200817 1708983
rs12523324
HG00116 >hsa-mir-553 TTTAAAACGGTGAGATTTTGTT TTTTAAAACGGTGAGATTTTGT
ATTTTAAAACGGTGAGATTTTG TATTTTAAAACGGTGAGATTTT
TTATTTTAAAACGGTGAGATTT 100746848 .

HG00116 >hsa-mir-553 TTTAAGACGGTGAGATTTTGTT TTTTAAGACGGTGAGATTTTGT
ATTTTAAGACGGTGAGATTTTG TATTTTAAGACGGTGAGATTTT
TTATTTTAAGACGGTGAGATTT 100746814 rs190622705

HG00116 >hsa-mir-553 TTTAAGACGGTGAGATTTTGTT TTTTAAGACGGTGAGATTTTGT
ATTTTAAGACGGTGAGATTTTG TATTTTAAGACGGTGAGATTTT
TTATTTTAAGACGGTGAGATTT 100746814 rs190622705 100746848 .

HG00116 >hsa-mir-1229TTTGGGGGAGAGTGTGGGCTGG AGGGTTTGGGGGAGAGTGTGGG
TAGGGTTTGGGGGAGAGTGTGG GTTTGGGGGAGAGTGTGGGCTG
GGTTTGGGGGAGAGTGTGGGCT 179225324 rs2291418

HG00116 >hsa-mir-149 AGGGAGGGAGGGACGGGGGCTG GGGCTGTGCTGGGGCGGCTGGA
GAGGGAGGGAGGGACGGGGGCT GGGAGGGAGGGACGGGGGCTGT
GGGAGGGACGGGGGCTGTGCTG 241395500 rs71428439

HG00116 >hsa-mir-149 AGGGAGGGAGGGACGGGGGCTG GGGCTGTGCTGGGGCAGCCGGA
GAGGGAGGGAGGGACGGGGGCT GGGAGGGAGGGACGGGGGCTGT
GGGACGGGGGCTGTGCTGGGGC 241395503 rs2292832

HG00116 >hsa-mir-149 AGGGAGGGAGGGACGGGGGCTG GGGCTGTGCTGGGGCGGCCGGA
GAGGGAGGGAGGGACGGGGGCT ACGGGGGCTGTGCTGGGGCGGC
GGGAGGGAGGGACGGGGGCTGT 241395500 rs71428439 241395503
rs2292832

HG00116 >hsa-mir-658 AGGTCGGTTGGTCGGTCGGGAA GTCGGTTGGTCGGTCGGGAACG TAGGTCGGTTGGTCGGTCGGGA . .

HG00116 >hsa-mir-744 ATGCACATGCTGTTGCCACTAA TGTTGCCACTAACCTCAACCTT GCTGTTGCCACTAACCTCAACC TGCACATGCTGTTGCCACTAAC C 11985275 .

HG00116 >hsa-mir-412 CGTCCGTATCCGCTGCAG CCGTCCGTATCCGCTGCAG TCACCTGGTCCACTGGCCGTCC ACCTGGTCCACTGGCCGTCCGT CACCTGGTCCACTGGCCGTCCG 101531854 rs61992671

HG00116 >hsa-mir-412 CGTCCGTATCCGCTGCAG CCGTCCGTATCCGCTGCAG TCACCTGGTTCACTGGCCGTCC ACCTGGTTCACTGGCCGTCCGT CACCTGGTTCACTGGCCGTCCG 101531849 . 101531854 rs61992671

HG00116 >hsa-mir-3180-4 CTCCGGATGCCAGTCCCTCATC GGAGGGTGAAGCCTCCGGATGC AGCGGAGGGTGAAGCCTCCGGA CTGGCCTGGTCGCGCTGTGGCT GAGCGGAGGGTGAAGCCTCCGG 15248720 rs75000738

HG00116 >hsa-mir-4268CTCTCAGGATGTGATGTCACCT CCTCTCAGGATGTGATGTCACC GCTCCTCCTCTCAGGATGTGAT TCCTCTCAGGATGTGATGTCAC CTCCTCCTCTCAGGATGTGATG 220771223 rs4674470

HG00116 >hsa-mir-3151CTGATCCCACACCCCACCTGTC TGATCCCACACCCCACCTGTCA GATCCCACACCCCACCTGTCAC GGGCATCCCACCTGATCCCACA TCCCACCTGATCCCACACCCCA 104166902 rs35605502

HG00116 >hsa-mir-412 CTTCACCTGGTTCACTAGCCGT ACCTGGTTCACTAGCCGTCCGT TGTACTTCACCTGGTTCACTAG CTGGTTCACTAGCCGTCCGTAT GTACTTCACCTGGTTCACTAGC 101531849 .

HG00116 >hsa-mir-658 TAGGTCGGTTGGTCGGTCGGGA .

HG00116 >hsa-mir-658 GTCCGTTGGTCGGTCGGGAACG G TCCGTTGGTCGGTCGGGAACGA .

HG00116 >hsa-mir-658 G rs141002682

HG00116 >hsa-mir-658 GTAGGTCGGTTGGTCGGTCGGG G TAGGTCGGTTGGTCGGTCGGGA . 38240368 rs141002682

HG00116 >hsa-mir-658 G GTCCGTTGGTCGGTCGGGAACG . rs141002682

HG00116 >hsa-mir-658 G GTAGGTCGGTTGGTCGGTCGGG TAGGTCGGTTGGTCGGTCGGGA . 38240315 . 38240368 rs141002682

HG00116 >hsa-mir-320eGAAAAGCTGGGTTGAGAAGGT AAAAGCTGGGTTGAGAAGGT GGAAAAGCTGGGTTGAGAAGGT GGGAAAAGCTGGGTTGAGAAGG rs10423365

HG00116 >hsa-mir-1343GCCCCTCCTGGGGCCCGCACTC CCCCTCCTGGGGCCCGCACTCT GGGGCCCGCACTCTCGCTCTGG CCCTCCTGGGGCCCGCACTCTC TGGGGCCCGCACTCTCGCTCTG 34963416 rs2986407

HG00116 >hsa-mir-3180-4 GGAGGGTGAAGCCTCCGGATGC GGTGAAGCCTCCGGATGCCAGT GCGGAGGGTGAAGCCTCCGGAT AGCGGAGGGTGAAGCCTCCGGA GCCTGGTCGCGCTGTGGCGAAG 15248798 rs183853838

HG00116 >hsa-mir-3180-4 GGAGGGTGAAGCCTCCGGATGC GGTGAAGCCTCCGGATGCCAGT GCGGAGGGTGAAGCCTCCGGAT AGCGGAGGGTGAAGCCTCCGGA CTGGCCTGGTCGCGCTGTGGCT 15248720 rs75000738 15248798 rs183853838

HG00116 >hsa-mir-1227GGCATTTGACCCCGTGCCACCC AGGCATTTGACCCCGTGCCACC AGGCATTTGACCCCGTGCCACC TGACCCCGTGCCACCCTTTTCC ATTTGACCCCGTGCCACCCTTT 2234093 rs190788838

HG00116 >hsa-mir-580 TATTTGAGAATGATGAATCATT TGAATCATTAGGTTCCGGTCAG ATGAATCATTAGGTTCCGGTCA TTTGAGAATGATGAATCATTAG GAGAATGATGAATCATTAGGTT 36148057 rs115089112

HG00116 >hsa-mir-3118-1 TGAAAATTCTTCTAGTGTG ATGAAAATTCTTCTAGTGTG TGCATTATGAAAATTCTTCTAG TTATGAAAATTCTTCTAGTGTG ATTATGAAAATTCTTCTAGTGT 142667330 rs76132421

HG00116 >hsa-mir-320c-1 AAAAGCTGGGTTGAGAGGGTAG AGCTGGGTTGAGAGGGTAGGAA CTGGGTTGAGAGGGTAGGAAAA AGGGTAGGAAAAAAATGATGTA

HG00116 >hsa-mir-499bAAACATCACTGCAAGTCTTAAC rs3746444 73 24214486 .

HG00116 >hsa-mir-202 AAAGAGGTATAGGGCATGGGAA AAGAGGTATAGGGCATGGGAAA GGGAAAACGGGGCGGTCGGGTC TAAAGAGGTATAGGGCATGGGA 154065347 rs142414368

HG00116 >hsa-mir-520hAAAGTGCTTCCCTTTAGAGTTA rs148716001 74 154065383 rs75538180

HG00116 >hsa-mir-548ap AACAAAAACCACAATTACTTTT CAAAAACCACAATTACTTTTTA CAATTACTTTTTACTGACCTAA rs4414449 154065347 rs142414368 154065383 rs75538180

HG00116 >hsa-mir-548ap AACAAAAACCACAATTACTTTT CAAAAACCACAATTACTTTTTA TTACTTTTTACTGACCTAAAGA rs4577031 93466866 rs2155248

HG00116 >hsa-mir-548ap AACAAAAACCACAATTACTTTT CAAAAACCACAATTACTTTTTA CAATTACTTTTTACTGACCTAA rs4414449 52013832 rs374103744

HG00116 >hsa-mir-423 AAGCTCGGTCTGAGGCCCCTCA AGGCCCCTCAGTCTTGCTTCCT

TCTGAGGCCCCTCAGTCTTGCT GTCTGAGGCCCCTCAGTCTTGC 34963416 rs2986407
HG00116 >hsa-mir-3910-1 AAGGCATAAAACCAAGACAAGC CATAAAACCAAGACAAGCAACA GGCATAAAACCAAGACAAGCAA GCATAAAACCAAGACAAGCAAC 241395500 rs71428439

HG00116 >hsa-mir-520hACAAAGTGCTTCCCTTTAGAGT AGTGCTTCCCTTTAGAGTTACT GTGCTTCCCTTTAGAGTTACTG rs56013413 241395503 rs2292832

HG00116 >hsa-mir-520hACAAAGTGCTTCCCTTTAGAGT AGTGCTTCCCTTTAGAGTTACT CTTCCCTTTAGAGTTACTGTTT rs148716001 241395500 rs71428439 241395503 rs2292832
HG00116 >hsa-mir-449cACAGTTGCTAGTTGCACTCCTC AACAGTTGCTAGTTGCACTCCT GTTGCTAGTTGCACTCCTCTCT GTTGCACTCCTCTCTGTTGCAT

HG00116 >hsa-mir-544bACCTGAGGTTGTGCATTTCTAA AGACCTGAGGTTGTGCATTTCT TAGACCTGAGGTTGTGCATTTC GAGGTTGTGCATTTCTAACAAA 61582708 rs174561
HG00116 >hsa-mir-3118-1 ACTGCATTATGAAAATTCTTCT ATTATGAAAATTCTTCTAGTGT GCATTATGAAAATTCTTCTAGT CTGCATTATGAAAATTCTTCTA 54385599 rs11614913

HG00116 >hsa-mir-642bAGATACATTTGGAGAGGGACCC TTGGAGAGGGACCCTCCCAACT TTTGGAGAGGGACCCTCCCAAC ATACATTTGGAGAGGGACCCTC 135061112 rs12355840
HG00116 >hsa-mir-3180-4 AGCGGAGGGTGAAGCCTCCGGA CGGAGGGTGAAGCCTCCGGATG GAGCGGAGGGTGAAGCCTCCGG GCGGAGGGTGAAGCCTCCGGAT

HG00116 >hsa-mir-3180-4 AGCGGAGGGTGAAGCCTCCGGA CGGAGGGTGAAGCCTCCGGATG GAGCGGAGGGTGAAGCCTCCGG GCGGAGGGTGAAGCCTCCGGAT 45606504 rs191727254

HG00116 >hsa-mir-30d AGCTTTCAGTCAGATGTTTGCT GGCTAAGCTTTCAGTCAGATGT GCTAAGCTTTCAGTCAGATGTT TTCAGTCAGATGTTTGCTGCTA 13947292 rs895819
HG00116 >hsa-mir-519a-2 AGGAAAGTGCATCCTTTTAGAG AGTGCATCCTTTTAGAGGGTTA GGAAAGTGCATCCTTTTAGAGG GAAAGGAAAGTGCATCCTTTTA

HG00116 >hsa-mir-630 AGTATTCTGTACCAGGGAAGGT ACCTAGTATTCTGTACCAGGGA CCAGGGAAGGTAGTTCTTAACT CAGGGAAGGTAGTTCTTAACTA 135817150 .
HG00116 >hsa-mir-513cATAAATTTCACCTTTCTGAGAA TTTCTGAGAAGAGTAATGTACA CCTTTCTGAGAAGAGTAATGTA TTTCACCTTTCTGAGAAGAGTA 142667330 rs76132421
HG00116 >hsa-mir-323bATACACGGTCGACCTCTTTTCG TACACGGTCGACCTCTTTTCGG

ACACGGTCGACCTCTTTTCGGT rs56103835 120336327 rs68035463

HG00116 >hsa-mir-3144ATACCTGTTCAGTCTCTTTAAA TTCAGTCTCTTTAAAGTGTAGT CCTGTTCAGTCTCTTTAAAGTG TGTTCAGTCTCTTTAAAGTGTA 120336384 rs67106263

HG00116 >hsa-mir-3144ATACCTGTTCAGTCTCTTTAAA TTCAGTCTCTTTAAAGTGTAGT CTGTTCAGTCTCTTTAAAGTGT TATACCTGTTCAGTCTCTTTAA 120336327 rs68035463 120336384 rs67106263

HG00116 >hsa-mir-3144ATACCTGTTCGGTCTCTTTAAA CTGTTCGGTCTCTTTAAAGTGT GTTCGGTCTCTTTAAAGTGTAG TGTTCGGTCTCTTTAAAGTGTA 104166902 rs35605502

HG00116 >hsa-mir-642aATTTGGAGAGGGAACCTCCCAA AGACACATTTGGAGAGGGAACC ACACATTTGGAGAGGGAACCTC CACATTTGGAGAGGGAACCTCC 15248720 rs75000738

HG00116 >hsa-mir-412 CACCTGGTCCACTGGCCGTCCG ACCTGGTCCACTGGCCGTCCGT CTGGCCGTCCGTATCCGCTGCA TCACCTGGTCCACTGGCCGTCC 15248798 rs183853838

HG00116 >hsa-mir-412 CACCTGGTTCACTGGCCGTCCG ACCTGGTTCACTGGCCGTCCGT CTGGCCGTCCGTATCCGCTGCA CCTGGTTCACTGGCCGTCCGTA 15248720 rs75000738 15248798 rs183853838

HG00116 >hsa-mir-744 CATGCTGTTGCCACTAACCTCA GCTGTTGCCACTAACCTCAACC CACTAACCTCAACCTTACTCGG .

HG00116 >hsa-mir-1227CATTTGACCCCGTGCCACCCTT ATTTGACCCCGTGCCACCCTTT AGGCATTTGACCCCGTGCCACC GACCCCGTGCCACCCTTTTCCC

HG00116 >hsa-mir-1908CCACCGGCCGCCGGCTCCGCCC CCGCCGGCTCCGCCCCGGCCCC GGCCGCCGGCTCCGCCCCGGCC CGGCCGCCGGCTCCGCCCCGGC 18392913 rs7247767

HG00116 >hsa-mir-3151CCACCTGATCCCACACCCCACC CACCTGATCCCACACCCCACCT CCCACCTGATCCCACACCCCAC TGATCCCACACCCCACCTGTCA 19263542 .

HG00116 >hsa-mir-1343CCCCTCCTGGGGCCCGCACTCT CCCTCCTGGGGCCCGCACTCTC CCTGGGGCCCGCACTCTCGCTC TGGGGCCCGCACTCTCGCTCTG

HG00116 >hsa-mir-1229CCTCTCACCACTGCCCTCCCAC GACACCCTCTCACCACTGCCCT ACCCTCTCACCACTGCCCTCCC CTCTCACCACTGCCCTCCCACA

HG00116 >hsa-mir-4268CCTCTCAGGATGTGATGTCACC CTCCTCTCAGGATGTGATGTCA CTCCTCCTCTCAGGATGTGATG rs4674470 94398581 rs67339585

HG00116 >hsa-mir-943 CCTGACTGTTGCCGTCCTCCAG CTGTTGCCGTCCTCCAGCCCCA CTCCAGCCCCACTCAAAGGCAT GCCGTCCTCCAGCCCCACTCAA 94398581 rs67339585

HG00116 >hsa-mir-196a-2 CTCGGCAACAAGAAACTGTCTG CAAGAAACTGTCTGAGTTACAT CAACAAGAAACTGTCTGAGTTA ACAAGAAACTGTCTGAGTTACA 104985443 rs61938575

HG00116 >hsa-mir-486-2 CTCGGCGCAGCTCAGTACAGGA
AGGGCCTCGGCGCAGCTCAGTA TCGGCGCAGCTCAGTACAGGAT
GGGCCTCGGCGCAGCTCAGTAC

HG00116 >hsa-mir-888 CTCTTTGGGTGAAGGAAGGCTC CTGACACCTCTTTGGGTGAAGG
GACTGACACCTCTTTGGGTGAA CCTCTTTGGGTGAAGGAAGGCT 131701279
rs367805
HG00116 >hsa-mir-1269a CTGGACTGAGCCATGCTACTGG
TGCCTGGACTGAGCCATGCTAC AATGCCTGGACTGAGCCATGCT rs73239138
101531849 .
HG00116 >hsa-mir-1304CTGTAGCATCGAACCCCTGGGC CTCACTGTAGCATCGAACCCCT
GAACCCCTGGGCTCAAGTGATT CGAACCCCTGGGCTCAAGTGAT 101531854
rs61992671
HG00116 >hsa-mir-3922CTGTGGGACTTCTGGCCTTGAC ACCTGTGGGACTTCTGGCCTTG
GGGACTTCTGGCCTTGACTTGA TGGGACTTCTGGCCTTGACTTG 101531849
. 101531854 rs61992671
HG00116 >hsa-mir-940 GAAGGCAGGGCCCCCGCTCCCC AGGAAGGCAGGGCCCCCGCTCC
AGGGCCCCCGCTCCCCGGGCCT 28444183 rs6505162
rs373947923
HG00116 >hsa-mir-940 GAAGGCAGGGCCCC-GCTCCCC G CCC-
GCTCCCCGGGCCTGACCC rs35356504

HG00116 >hsa-mir-940 GAAGGCAGGGCCCC-GCTCCCC G GGGCCCC-
GCTCCCCGGGCCTG 54468166 rs75661995
rs373947923
HG00116 >hsa-mir-548h-4 GACAAAAACCACAATTACTTTT
AAAAACCACAATTACTTTTGCA TGACAAAAACCACAATTACTTT
ACAAAAACCACAATTACTTTTG 41518007 .

HG00116 >hsa-mir-548h-4 GACAAAAACCACAATTACTTTT
AAAAACCACAATTACTTTTGCA TGACAAAAACCACAATTACTTT
ACAAAAACCACAATTACTTTTG 33578251 rs3746444

HG00116 >hsa-mir-548h-4 GACAAAAACCGCAATTACTTTT
TGACAAAAACCGCAATTACTTT AAAAACCGCAATTACTTTTGCA
AATGACAAAAACCGCAATTACT

HG00116 >hsa-mir-3188GAGGCTTTGTGCGGATACGGGG GAGAGGCTTTGTGCGGATACGG
GCGGATACGGGGCTGGAGGCCT 146271303 rs145416750
rs7247237
HG00116 >hsa-mir-3188GAGGCTTTGTGCGGATACGGGG GGAGAGGCTTTGTGCGGATACG
GAGAGGCTTTGTGCGGATACGG 54265670 . rs7247767

HG00116 >hsa-mir-3188GAGGCTTTGTGCGGATACGGGG GAGAGGCTTTGTGCGGATACGG
GGAGAGGCTTTGTGCGGATACG rs7247237

HG00116 >hsa-mir-629 GAGGTTCTCCCAACGTAAGCCC AGGTTCTCCCAACGTAAGCCCA TCTCCCAACGTAAGCCCAGCCC CAGGAGGTTCTCCCAACGTAAG

HG00116 >hsa-mir-222 GCAGCTACATCTGGCTACTGGG TACTGGGTCTCTGATGGCATCT GCTACTGGGTCTCTGATGGCAT CTGGCTACTGGGTCTCTGATGG 54245788 rs56013413
HG00116 >hsa-mir-3180-4 GCGGAGGGTGAAGCCTCCGGAT CGCTGGCCTGGTCGCGCTGTGG TCGCTGGCCTGGTCGCGCTGTG AAGCCTCCGGATGCCAGTCCCT 124451312 rs10934682

HG00116 >hsa-mir-548a-3 GGCAAAACTGGCAGTTACTTTT GCAAAACTGGCAGTTACTTTTG AAAACTGGCAGTTACTTTTGCA AACTGGCAGTTACTTTTGCACC 105496622 .

HG00116 >hsa-mir-149 GGGAGGGAGGGACGGGGGCTGT GGAGGGACGGGGGCTGTGCTGG GAGGAGGGAGGGAGGGACGGGG AGGGACGGGGGCTGTGCTGGGG

HG00116 >hsa-mir-149 GGGAGGGAGGGACGGGGGCTGT GGAGGGACGGGGGCTGTGCTGG AGGGACGGGGGCTGTGCTGGGG GAGGAGGGAGGGAGGGACGGGG

HG00116 >hsa-mir-149 GGGAGGGAGGGACGGGGGCTGT GGAGGGACGGGGGCTGTGCTGG AGGGACGGGGGCTGTGCTGGGG GAGGAGGGAGGGAGGGACGGGG 86368959 rs4577031 rs367702648
HG00116 >hsa-mir-1244-3 GTACTATAAGTAGTTGGTTTGT GTAGTTGGTTTGTATGAGACGG TAAGTAGTTGGTTTGTATGAGA

HG00116 >hsa-mir-1197GTAGGACACATGGTCTACTTCT ACACATGGTCTACTTCTTCTCA ACATGGTCTACTTCTTCTCAAT TAGGACACATGGTCTACTTCTT

HG00116 >hsa-mir-27a GTGTTCACAGTGGCTAAGTTCC TCGTGTTCACAGTGGCTAAGTT AGTGGCTAAGTTCCGCCCCCCA TGTTCACAGTGGCTAAGTTCCG 26906402 rs73235381
HG00116 >hsa-mir-3910-2 TAAAAGGCATAAAACCAAGACA TAAAACCAAGACAAGCAACAGA AGGCATAAAACCAAGACAAGCA GGCATAAAACCAAGACAAGCAA 26906437 rs184537764

HG00116 >hsa-mir-3936TAAGGGGTGTATGGCAGATGCA TTCTGGTAAGGGGTGTATGGCA CACCCGACAGATGCACTTGGCA TGTATGGCAGATGCACCCGACA 26906402 rs73235381 26906437 rs184537764
HG00116 >hsa-mir-300 TATACAAGGGCAGACTCTCTCT TGATTATACAAGGGCAGACTCT ATTATACAAGGGCAGACTCTCT 26951185 rs4822739 rs12894467
HG00116 >hsa-mir-499aTCACAGCAAGTCTGTGCTGCTT ACAGCAAGTCTGTGCTGCTTCC CGTCACAGCAAGTCTGTGCTGC TCCCTACGCTGCCTGGGCAGGG 36148057 rs115089112
HG00116 >hsa-mir-412 TCACCTGGTTCACTAGCCGTCC TCACCTGGTTCACTAGCCGTCC ATGTACTTCACCTGGTTCACTA CTTCACCTGGTTCACTAGCCGT 168690612

rs62376934
HG00116 >hsa-mir-1273h TGCTGCAGACTCGACCTCCCAG
TGCAGACTCGACCTCCCAGGCT CTGCAGACTCGACCTCCCAGGC
AGACTCGACCTCCCAGGCTTAA 168690664 rs140379047

HG00116 >hsa-mir-548jTGGCAAAAACTGCATTACTTTT GGCAAAAACTGCATTACTTTTG
CAAAAACTGCATTACTTTTGCA AAACTGCATTACTTTTGCACCA 168690612
rs62376934 168690664 rs140379047
HG00116 >hsa-mir-585 TTGGGCGTATCTGTATGCTAGG TATCTGTATGCTAGGGCTGCCG
TGGGCGTATCTGTATGCTAGGG GCGTATCTGTATGCTAGGGCTG 70371761
rs377691713
HG00116 >hsa-mir-585 TTGGGCGTATCTGTATGCTAGG GGGCGTATCTGTATGCTAGGGC
GCGTATCTGTATGCTAGGGCTG TATCTGTATGCTAGGGCTGCTG 72879653
rs113971639
HG00116 >hsa-mir-585 TTGGGCGTATCTGTATGCTAGG GGGCGTATCTGTATGCTAGGGC
GCGTATCTGTATGCTAGGGCTG TATCTGTATGCTAGGGCTGCCG 46178217
rs111664333
HG00116 >hsa-mir-1303TTTAGAGACGGGGTCTTGCTCT TAGAGACGGGGTCTTGCTCTGT
ACGGGGTCTTGCTCTGTTGCCA GGGTCTTGCTCTGTTGCCAGGC 46178217
rs111664333
HG00116 >hsa-mir-1303TTTAGAGACGGGGTCTTGCTCT TAGAGACGGGGTCTTGCTCTGT
TTAGAGACGGGGTCTTGCTCTG ACGGGGTCTTGCTCTGTTGCCA

HG00116 >hsa-mir-1303TTTAGAGACGGGGTCTTGCTCT TAGAGACGGGGTCTTGCTCTGT
TTAGAGACGGGGTCTTGCTCTG ACGGGGTCTTGCTCTGTTGCCA

HG00116 >hsa-mir-580 TTTGAGAATGATGAATCATTAG GATGAATCATTAGGTTCCGGTC
AATGATGAATCATTAGGTTCCG AGAATGATGAATCATTAGGTTC

HG00116 >hsa-mir-133bTTTGGTCCCCTTCAACCAGCTA TGGTCCCCTTCAACCAGCTACA
CCTTCAACCAGCTACAGCAGGG AGAGGTTTGGTCCCCTTCAACC

HG00117 >hsa-mir-3620GGTGGGGGCCAGCAGGGAGTGG TGAGGTGGGGGCCAGCAGGGAG
GTGAGGTGGGGGCCAGCAGGGA GAGGTGGGGGCCAGCAGGGAGT
AGGTGGGGGCCAGCAGGGAGTG 228284991 rs2070960

HG00117 >hsa-mir-412 TGGGGTACGGGGATGGATGGTC GGATGGATGGTCGACCAGTTGG
GATGGATGGTCGACCAGTTGGA TCGACCAGTTGGAAAGTAATTG
ACGGGGATGGATGGTCGACCAG 101531854 rs61992671

HG00117 >hsa-mir-553 TTTTAAAACGGTGAGATTTTGT TATTTTAAAACGGTGAGATTTT
TTTTATTTTAAAACGGTGAGAT TTTAAAACGGTGAGATTTTGTT
ATTTTATTTTAAAACGGTGAGA 100746848 .

HG00117 >hsa-mir-553 TTTTAAGACGGTGAGATTTTGT TATTTTAAGACGGTGAGATTTT
TTTAAGACGGTGAGATTTTGTT TTTTATTTTAAGACGGTGAGAT
ATTTTATTTTAAGACGGTGAGA 100746814 rs190622705 100746848 .

HG00117	>hsa-mir-553 TTTTAAAACGGTGAGATTTTGT	TATTTTAAAACGGTGAGATTTT
TTTTATTTTAAAACGGTGAGAT	TTTAAAACGGTGAGATTTTGTT
ATTTTATTTTAAAACGGTGAGA	100746835	.	100746848	.

HG00117	>hsa-mir-553 TTTTAAGACGGTGAGATTTTGT	TATTTTAAGACGGTGAGATTTT
TTTTATTTTAAGACGGTGAGAT	TTTAAGACGGTGAGATTTTGTT
ATTTTATTTTAAGACGGTGAGA	100746814	rs190622705	100746835	.
100746848

HG00117	>hsa-mir-553 TTTTAAAACGGTGAGATTTTGT	TTTAAAACGGTGAGATTTTGTT
TTATTTTAAAACGGTGAGATTT	TATTTTAAAACGGTGAGATTTT
TTTTATTTTAAAACGGTGAGAT	100746835	.

HG00117	>hsa-mir-553 TTTAAGACGGTGAGATTTTGTT	TTTTAAGACGGTGAGATTTTGT
TATTTTAAGACGGTGAGATTTT	TTATTTTAAGACGGTGAGATTT
TTTTATTTTAAGACGGTGAGAT	100746814	rs190622705	100746835	.

HG00117	>hsa-mir-1273h	CCTGGGAGGTCAAGGCTGTAGT
TGGGAGGTCAAGGCTGTAGTGT	ATTGCTTGAGCCTGGGAGGTCA
GCCTGGGAGGTCAAGGCTGTAG	TTGAGCCTGGGAGGTCAAGGCT	24214486	.

HG00117	>hsa-mir-553 TTTAAGACGGTGAGATTTTGTT	TATTTTAAGACGGTGAGATTTT
TTATTTTAAGACGGTGAGATTT	TTTTATTTTAAGACGGTGAGAT
ATTTTAAGACGGTGAGATTTTG	100746814	rs190622705

HG00117	>hsa-mir-4326TCTGCTGTTCCTCTGTCTCCCA	TGGTCTGCTGTTCCTCTGTCTC
CTGGTCTGCTGTTCCTCTGTCT	GCTGTTCCTCTGTCTCCCAGAC
TGCTGTTCCTCTGTCTCCCAGA	61918164	rs6062431

HG00117	>hsa-mir-577 AGTGAAGAGTAGATAAAATATT	GTAGATAAAATATTGGTACCTG
GAAGAGTAGATAAAATATTGGT	AAGAGTAGATAAAATATTGGTA
AGTAGATAAAATATTGGTACCT	115577997	rs34115976

HG00117	>hsa-mir-412 TGGGGTACGGGGATGGATGGTC	GGATGGATGGTCGACCAGTTGG
GGGTACGGGGATGGATGGTCGA	GATGGATGGTCGACCAGTTGGA
TCGACCAGTTGGAAAGTAATTG	101531849	.	101531854	rs61992671

HG00117	>hsa-mir-3125AGAATGGATAGAGGAAGCTGTG	GAGGAAGCTGTGGAGAGAACTC
AGAGGAAGCTGTGGAGAGAACT	GCTGTGGAGAGAACTCACGGTG
GGAAGCTGTGGAGAGAACTCAC	12877501	rs78852835

HG00117	>hsa-mir-877 TAGAGGAGATGGCGCAGGGGAC	GAGGAGATGGCGCAGGGGACAC
GAGATGGCGCAGGGGACACGGG	TGGCGCAGGGGACACGGGCAAA
GGAGATGGCGCAGGGGACACGG	30552187	rs372113020

HG00117	>hsa-mir-548ak	GCAAAAGTAACTGCGGTTTTTG
TGCAAAAGTAACTGCGGTTTTT	CAAAAGTAACTGCGGTTTTTGA

GTGCAAAAGTAACTGCGGTTTT rs7070684

HG00117 >hsa-mir-1268a GCCAGGCGTGGTGGTGGGGGCC
AGCCAGGCGTGGTGGTGGGGGC

HG00117 >hsa-mir-1283-1 AAAGGAAAGCGCTTTCTGTTGT
ACAAAGGAAAGCGCTTTCTGTT CAAAGGAAAGCGCTTTCTGTTG
AGTCTACAAAGGAAAGCGCTTT TACAAAGGAAAGCGCTTTCTGT 54191743
rs57111412
HG00117 >hsa-mir-559 AAATATGCACCAAAATTACTTT AGTAAATATGCACCAAAATTAC
AAGTAAATATGCACCAAAATTA AAAGTAAATATGCACCAAAATT
GTAAATATGCACCAAAATTACT 47604866 rs58450758

HG00117 >hsa-mir-3175AGAGAACGCAGTGACGTCTGGC GAGAGAACGCAGTGACGTCTGG
GGAGAGAACGCAGTGACGTCTG GGGAGAGAACGCAGTGACGTCT
GGGGAGAGAACGCAGTGACGTC 93447631 rs1439619

HG00117 >hsa-mir-378hAGATGGGATGAGCCCTGGCTCT TCAGATGGGATGAGCCCTGGCT
TGTCAGATGGGATGAGCCCTGG GATGGGATGAGCCCTGGCTCTG
TGGTGTCAGATGGGATGAGCCC 154209024 rs702742

HG00117 >hsa-mir-1269b AGCCATGCTACGGGCTTCTCTG
ACTGAGCCATGCTACGGGCTTC AGGTTTCTGGACTGAGCCATGC
TGAGGTTTCTGGACTGAGCCAT TTTCTGGACTGAGCCATGCTAC 12820632
rs12451747
HG00117 >hsa-mir-3135b AGCGAGTGCAGTGGTGCAGTCA
AGGCTGGAGCGAGTGCAGTGGT CTGGAGCGAGTGCAGTGGTGCA
CAGGCTGGAGCGAGTGCAGTGG TGGAGCGAGTGCAGTGGTGCAG 32717702
rs4285314
HG00117 >hsa-mir-612 AGGGCTTCTGAGCTCCTTAGCA GGGCTTCTGAGCTCCTTAGCAC
CAGGGCTTCTGAGCTCCTTAGC GCAGGGCTTCTGAGCTCCTTAG
GGCAGGGCTTCTGAGCTCCTTA 65211940 rs550894

HG00117 >hsa-mir-548ap AGTAATTGCAGTCTTTGTCATT
AAGTAATTGCAGTCTTTGTCAT AAAGTAATTGCAGTCTTTGTCA
AAAAGTAATTGCAGTCTTTGTC CAAAAGTAATTGCAGTCTTTGT 86368898
rs4414449
HG00117 >hsa-mir-548ap AGTAATTGCAGTCTTTGTCATT
AAGTAATTGCAGTCTTTGTCAT AAAGTAATTGCAGTCTTTGTCA
AAAAGTAATTGCAGTCTTTGTC CAAAAGTAATTGCAGTCTTTGT 86368898
rs4414449 86368959 rs4577031
HG00117 >hsa-mir-548ap AGTAATTGCGGTCTTTGTCATT
AAGTAATTGCGGTCTTTGTCAT AAAGTAATTGCGGTCTTTGTCA
AAAAGTAATTGCGGTCTTTGTC CAAAAGTAATTGCGGTCTTTGT 86368959
rs4577031
HG00117 >hsa-mir-196a-2 AGTTTCATGTTGTTGGGATTGA
TAGTTTCATGTTGTTGGGATTG AGGTAGTTTCATGTTGTTGGGA

GTAGTTTCATGTTGTTGGGATT GGTAGTTTCATGTTGTTGGGAT 54385599
rs11614913
HG00117 >hsa-mir-3622b ATGGGAGGTCAGGTGAGCTCAG
CATGGGAGGTCAGGTGAGCTCA GCATGGGAGGTCAGGTGAGCTC
AGGCATGGGAGGTCAGGTGAGC GGCATGGGAGGTCAGGTGAGCT 27559214
rs66683138
HG00117 >hsa-mir-500bCTTGCTACCTGGGTGAGAGTGC ATCCTTGCTACCTGGGTGAGAG
AATCCTTGCTACCTGGGTGAGA CCTTGCTACCTGGGTGAGAGTG
TCCTTGCTACCTGGGTGAGAGT 49775351 rs151318590

HG00117 >hsa-mir-612 GGGCTTCTGAGCTCCTTAGCAC AGGGCTTCTGAGCTCCTTAGCA
CAGGGCTTCTGAGCTCCTTAGC GCAGGGCTTCTGAGCTCCTTAG
GGCAGGGCTTCTGAGCTCCTTA 65211979 rs12803915

HG00117 >hsa-mir-612 GGGCTTCTGAGCTCCTTAGCAC AGGGCTTCTGAGCTCCTTAGCA
CAGGGCTTCTGAGCTCCTTAGC GCAGGGCTTCTGAGCTCCTTAG
GGCAGGGCTTCTGAGCTCCTTA 65211940 rs550894 65211979
rs12803915
HG00117 >hsa-mir-548h-3 GTAATCGTGGTTTTTGTCATTG
TGCAAAAGTAATCGTGGTTTTT TGGTGCAAAAGTAATCGTGGTT
TTGGTGCAAAAGTAATCGTGGT AGTAATCGTGGTTTTTGTCATT 13446924
rs9913045
HG00117 >hsa-mir-573 GTGTAACTGATCAGGATCTACT TGTGTAACTGATCAGGATCTAC
GATGTGTAACTGATCAGGATCT TGATGTGTAACTGATCAGGATC
GTGATGTGTAACTGATCAGGAT 24521902 rs76014664

HG00117 >hsa-mir-573 GTGTAACTGATCAGGATTTACT TGTAACTGATCAGGATTTACTC
ATGTGTAACTGATCAGGATTTA TGTGTAACTGATCAGGATTTAC
GATGTGTAACTGATCAGGATTT 24521871 rs192259662

HG00117 >hsa-mir-573 GTGTAACTGATCAGGATTTACT TGTAACTGATCAGGATTTACTC
ATGTGTAACTGATCAGGATTTA TGTGTAACTGATCAGGATTTAC
GATGTGTAACTGATCAGGATTT 24521871 rs192259662 24521902
rs76014664
HG00117 >hsa-mir-3117TAAAGGGCCAGACACTATACGA GCCAGACACTATACGAGTCATA
GGGCCAGACACTATACGAGTCA GGCCAGACACTATACGAGTCAT
CCCTAAAGGGCCAGACACTATA 67094171 rs12402181

HG00117 >hsa-mir-576 TAATTTCTCCACGTCTTTGGTA TCTAATTTCTCCACGTCTTTGG
TTCTAATTTCTCCACGTCTTTG CTAATTTCTCCACGTCTTTGGT
GATTCTAATTTCTCCACGTCTT 110409933 rs77639117

HG00117 >hsa-mir-629 TACGTTGGGAGAACTTTTACGG TTACGTTGGGAGAACTTTTACG
GTTTACGTTGGGAGAACTTTTA TTTACGTTGGGAGAACTTTTAC
GGTTTACGTTGGGAGAACTTTT 70371794 .

HG00117 >hsa-mir-629 TACGTTGGGAGAACTTTTATGG TTACGTTGGGAGAACTTTTATG

TTTACGTTGGGAGAACTTTTAT TGGGTTTACGTTGGGAGAACTT
GTTTACGTTGGGAGAACTTTTA 70371761 rs377691713

HG00117 >hsa-mir-629 TACGTTGGGAGAACTTTTATGG TTACGTTGGGAGAACTTTTATG
TTTACGTTGGGAGAACTTTTAT TGGGTTTACGTTGGGAGAACTT
GTTTACGTTGGGAGAACTTTTA 70371761 rs377691713 70371794 .

HG00117 >hsa-mir-888 TACTCAAAAAGCTTTCAGTCAC TCTACTCAAAAAGCTTTCAGTC
TGCTCTACTCAAAAAGCTTTCA GCTCTACTCAAAAAGCTTTCAG
GTGCTCTACTCAAAAAGCTTTC 145076355 rs143634721

HG00117 >hsa-mir-520gTAGAGGAAGCACTTTCTGTTTG TCTAGAGGAAGCACTTTCTGTT
TGACCCTCTAGAGGAAGCACTT TGTGACCCTCTAGAGGAAGCAC
TGCTGTGACCCTCTAGAGGAAG 54225460 rs375062679

HG00117 >hsa-mir-3125TAGAGGAAGCTGTGGAGAGAAC AGCTGTGGAGAGAACTCACGGT
TGGATAGAGGAAGCTGTGGAGA AAGCTGTGGAGAGAACTCACGG
AGGAAGCTGTGGAGAGAACTCA 12877501 rs78852835

HG00117 >hsa-mir-3199-1 TAGGAGAAAGTTTCTGGAAGTT
TTAGGAGAAAGTTTCTGGAAGT TGCCTTAGGAGAAAGTTTCTGG
TCCAGGGACTGCCTTAGGAGAA TGACTCCAGGGACTGCCTTAGG 28316513
rs118160653
HG00117 >hsa-mir-449cTAGGCAGTGTATTGCTAGCGGC TCAGATAGGCAGTGTATTGCTA
TGTCAGATAGGCAGTGTATTGC TGTGTCAGATAGGCAGTGTATT
TGGGATGTGTCAGATAGGCAGT 54468166 rs75661995

HG00117 >hsa-mir-590 TATTCATAAAAGTGCAGTATGG TTATTCATAAAAGTGCAGTATG
TTTATTCATAAAAGTGCAGTAT TGAGTTTATTCATAAAAGTGCA
GTTTATTCATAAAAGTGCAGTA 73605546 rs189727189

HG00117 >hsa-mir-3922TCAAGGCCAGAGGTCCCACAAC TCAAGTCAAGGCCAGAGGTCCC
GCCAGAGGTCCCACAACAGGGC GGCCAGAGGTCCCACAACAGGG
GTCAAGGCCAGAGGTCCCACAA 104985443 rs61938575

HG00117 >hsa-mir-564 TCAGCAGGCAACATGGCCGAGA TGTCAGCAGGCAACATGGCCGA
GTCAGCAGGCAACATGGCCGAG TGCCAGGCACGGTGTCAGCAGG
GTGTCAGCAGGCAACATGGCCG 44903433 .

HG00117 >hsa-mir-222 TCAGTAGCCAGTGTAGATCCTG TGGCTCAGTAGCCAGTGTAGAT
TTGGCTCAGTAGCCAGTGTAGA TCATTGGCTCAGTAGCCAGTGT
TACCCTCATTGGCTCAGTAGCC 45606504 rs191727254

HG00117 >hsa-mir-515-1 TCCAAAAGAAAGCACTTTCTGT
TCTCCAAAAGAAAGCACTTTCT TTCTCCAAAAGAAAGCACTTTC
TCATTCTCCAAAAGAAAGCACT TGCAGTCATTCTCCAAAAGAAA 54182326
rs374576826

HG00117 >hsa-mir-663aTCCCAGGCGGGGCGCCGCGGGA TCCGGCGTCCCAGGCGGGGCGC
TTCCGGCGTCCCAGGCGGGGCG GCGCCGCGGGACCTCCCTCGTG
GGCGCCGCGGGACCTCCCTCGT 26188880 .

HG00117 >hsa-mir-149 TCCGTGTCTTCACTCCCGTGCT TGGCTCCGTGTCTTCACTCCCG
TCTGGCTCCGTGTCTTCACTCC CCGTGTCTTCACTCCCGTGCTT
CTCCGTGTCTTCACTCCCGTGC 241395503 rs2292832

HG00117 >hsa-mir-618 TCCTTCTGAGTGTAATTACGTA TGTCCTTCTGAGTGTAATTACG
TTGTCCTTCTGAGTGTAATTAC TACTTGTCCTTCTGAGTGTAAT
GTCCTTCTGAGTGTAATTACGT 81329536 rs2682818

HG00117 >hsa-mir-412 TCGACCAGTTGGAAAGTAATTG TGGTCGACCAGTTGGAAAGTAA
TGGGGTACGGGGATGGATGGTC TGGATGGTCGACCAGTTGGAAA
TACGGGGATGGATGGTCGACCA 101531854 rs61992671

HG00117 >hsa-mir-412 TCGACCAGTTGGAAAGTAATTG TGGTCGACCAGTTGGAAAGTAA
TGGGGTACGGGGATGGATGGTC TGGATGGTCGACCAGTTGGAAA
TACGGGGATGGATGGTCGACCA 101531849 . 101531854 rs61992671

HG00117 >hsa-mir-492 TCGAGGACCTGCGGGACAAGAT TACAGGACCATCGAGGACCTGC
TACTACAGGACCATCGAGGACC TCCAGCCACTACTACAGGACCA
GACCTGCGGGACAAGATTCTTG 95228179 rs200816308

HG00117 >hsa-mir-3183TCGGAGTCGCTCGGAGCAGTCA TCTCGGAGTCGCTCGGAGCAGT
TCTCTCGGAGTCGCTCGGAGCA TGCCTCTCTCGGAGTCGCTCGG
TGCCCTGCCTCTCTCGGAGTCG 925742 rs72812091

HG00117 >hsa-mir-4305TCTGGGTTCTTAGAGGCCTAAT TTCTGGGTTCTTAGAGGCCTAA
GTTCTGGGTTCTTAGAGGCCTA TCCAGTTCTGGGTTCTTAGAGG
CAGTTCTGGGTTCTTAGAGGCC 40238175 rs67976778

HG00117 >hsa-mir-1200TGAGCCATTCTGAGCCTCAGTC TCCTGAGCCATTCTGAGCCTCA
TGCTACTTCTCCTGAGCCATTC TCTCCTGAGCCATTCTGAGCCT
TTCTCCTGAGCCATTCTGAGCC 36959006 .

HG00117 >hsa-mir-27a TGAGGAGCAGGGCTTAGCTGCT TTAGCTGCTTGTGAGCAGGGTC
GAGGAGCAGGGCTTAGCTGCTT GGAGCAGGGCTTAGCTGCTTGT
GAGCAGGGCTTAGCTGCTTGTG 13947292 rs895819

HG00117 >hsa-mir-3151TGATGGGTGGGGCAATGGGATC TGGGTGGGGCAATGGGATCAGG
TGGGGCAATGGGATCAGGTGCC GGGGTGATGGGTGGGGCAATGG
GGGTGATGGGTGGGGCAATGGG 104166902 rs35605502

HG00117 >hsa-mir-3622a TGCACAGGCACAGGAGCTCAGG
TAGAGGGTGCACAGGCACAGGA GCACAGGAGCTCAGGTGAGGCA
GGCACAGGAGCTCAGGTGAGGC GAGGGTGCACAGGCACAGGAGC 27559214

rs66683138
HG00117 >hsa-mir-3156-3 TGCAGAAGAAAGATCTGGAAGT
GCAGAAGAAAGATCTGGAAGTG GAAAGATCTGGAAGTGGGAGAC
GAAGAAAGATCTGGAAGTGGGA AGAAGAAAGATCTGGAAGTGGG 14778721
rs2747232
HG00117 >hsa-mir-3152TGCCTCTGTTCTAACACAAGAC TTGCCTCTGTTCTAACACAAGA
TATTGCCTCTGTTCTAACACAA TTATTGCCTCTGTTCTAACACA
TGCAGAGTTATTGCCTCTGTTC 18573360 rs13299349

HG00117 >hsa-mir-1254-2 TGGAAGCTGGAGCCTGCAGTGA
TGAGCCTGGAAGCTGGAGCCTG GAAGCTGGAGCCTGCAGTGAGC
GGAAGCTGGAGCCTGCAGTGAG GCCTGGAAGCTGGAGCCTGCAG 23682383
rs200793185
HG00117 >hsa-mir-516b-2 TGGAGGTAAGAAGCACTTTGTG
TCTGGAGGTAAGAAGCACTTTG TGACCATCTGGAGGTAAGAAGC
TGTGACCATCTGGAGGTAAGAA TGATGTGACCATCTGGAGGTAA 54228742
rs10670323
HG00117 >hsa-mir-877 TGGCGCAGGGGACACGGGCAAA GACACGGGCAAAGACTTGGGGG
GGACACGGGCAAAGACTTGGGG GGGACACGGGCAAAGACTTGGG
GGGGACACGGGCAAAGACTTGG 30552187 rs372113020

HG00117 >hsa-mir-1273h TGGGAGGTCAAGGCTGTAGTGT
TGAGCCTGGGAGGTCAAGGCTG TTGAGCCTGGGAGGTCAAGGCT
TGCTTGAGCCTGGGAGGTCAAG TTGCTTGAGCCTGGGAGGTCAA 24214486 .

HG00117 >hsa-mir-3620TGGGGGCCAGCAGGGAGTGGGT TGAGGTGGGGGCCAGCAGGGAG
GGGGGCCAGCAGGGAGTGGGTT GTGGGGGCCAGCAGGGAGTGGG
GGTGGGGGCCAGCAGGGAGTGG 228284991 rs2070960

HG00117 >hsa-mir-1227TGGTGGGCACTGCTGGGGTGGG AGGCGGTGGTGGGCACTGCTGG
TGGGGCCAGGCGGTGGTGGGCA GGTGGGCACTGCTGGGGTGGGC
GTGGTGGGCACTGCTGGGGTGG 2234093 rs190788838

HG00117 >hsa-mir-323bTGTCCGTGGTGAGTTCGCATTA TTGTCCGTGGTGAGTTCGCATT
TACTCGGAGGGAGGTTGTCCGT TCGGAGGGAGGTTGTCCGTGGT
AGGTTGTCCGTGGTGAGTTCGC 101522556 rs56103835

HG00117 >hsa-mir-642bTTCCCTCTCCAAATGTGTCTTG TTGGGAGGTTCCCTCTCCAAAT
TGGGAGGTTCCCTCTCCAAATG GAGTTGGGAGGTTCCCTCTCCA
GTTGGGAGGTTCCCTCTCCAAA 46178217 rs111664333

HG00117 >hsa-mir-4277TTCTGAGCACAGTACACTGGGC TCGAGGCAGTTCTGAGCACAGT
TGGGTCGAGGCAGTTCTGAGCA GTTCTGAGCACAGTACACTGGG
GCAGTTCTGAGCACAGTACACT 1708902 rs115200817

HG00117 >hsa-mir-553 TTTAAAACGGTGAGATTTTGTT TTTTAAAACGGTGAGATTTTGT
ATTTTAAAACGGTGAGATTTTG TATTTTAAAACGGTGAGATTTT

TTATTTTAAAACGGTGAGATTT 100746835 .

HG00117 >hsa-mir-553 TTTAAAACGGTGAGATTTTGTT TTTTAAAACGGTGAGATTTTGT
ATTTTAAAACGGTGAGATTTTG TATTTTAAAACGGTGAGATTTT
TTATTTTAAAACGGTGAGATTT 100746848 .

HG00117 >hsa-mir-553 TTTAAAACGGTGAGATTTTGTT TTTTAAAACGGTGAGATTTTGT
ATTTTAAAACGGTGAGATTTTG TATTTTAAAACGGTGAGATTTT
TTATTTTAAAACGGTGAGATTT 100746835 . 100746848 .

HG00117 >hsa-mir-553 TTTAAGACGGTGAGATTTTGTT TTTTAAGACGGTGAGATTTTGT
ATTTTAAGACGGTGAGATTTTG TATTTTAAGACGGTGAGATTTT
TTATTTTAAGACGGTGAGATTT 100746814 rs190622705

HG00117 >hsa-mir-553 TTTAAGACGGTGAGATTTTGTT TTTTAAGACGGTGAGATTTTGT
ATTTTAAGACGGTGAGATTTTG TATTTTAAGACGGTGAGATTTT
TTATTTTAAGACGGTGAGATTT 100746814 rs190622705 100746835 .

HG00117 >hsa-mir-553 TTTAAGACGGTGAGATTTTGTT TTTTAAGACGGTGAGATTTTGT
ATTTTAAGACGGTGAGATTTTG TATTTTAAGACGGTGAGATTTT
TTATTTTAAGACGGTGAGATTT 100746814 rs190622705 100746848 .

HG00117 >hsa-mir-553 TTTAAGACGGTGAGATTTTGTT TTTTAAGACGGTGAGATTTTGT
ATTTTAAGACGGTGAGATTTTG TATTTTAAGACGGTGAGATTTT
TTATTTTAAGACGGTGAGATTT 100746814 rs190622705 100746835 .
100746848 .
HG00117 >hsa-mir-1283-1 AAAGGAAAGCGCTTTCTGTTGT
ACAAAGGAAAGCGCTTTCTGTT CAAAGGAAAGCGCTTTCTGTTG
AGTCTACAAAGGAAAGCGCTTT TACAAAGGAAAGCGCTTTCTGT 54191743
rs57111412
HG00117 >hsa-mir-559 AAATATGCACCAAAATTACTTT AGTAAATATGCACCAAAATTAC
AAGTAAATATGCACCAAAATTA AAAGTAAATATGCACCAAAATT
GTAAATATGCACCAAAATTACT 47604866 rs58450758

HG00117 >hsa-mir-3175AGAGAACGCAGTGACGTCTGGC GAGAGAACGCAGTGACGTCTGG
GGAGAGAACGCAGTGACGTCTG GGGAGAGAACGCAGTGACGTCT
GGGGAGAGAACGCAGTGACGTC 93447631 rs1439619

HG00117 >hsa-mir-378hAGATGGGATGAGCCCTGGCTCT TCAGATGGGATGAGCCCTGGCT
TGTCAGATGGGATGAGCCCTGG GATGGGATGAGCCCTGGCTCTG
TGGTGTCAGATGGGATGAGCCC 154209024 rs702742

HG00117 >hsa-mir-1269b AGCCATGCTACGGGCTTCTCTG
ACTGAGCCATGCTACGGGCTTC AGGTTTCTGGACTGAGCCATGC
TGAGGTTTCTGGACTGAGCCAT TTTCTGGACTGAGCCATGCTAC 12820632
rs12451747
HG00117 >hsa-mir-3135b AGCGAGTGCAGTGGTGCAGTCA

AGGCTGGAGCGAGTGCAGTGGT CTGGAGCGAGTGCAGTGGTGCA
CAGGCTGGAGCGAGTGCAGTGG TGGAGCGAGTGCAGTGGTGCAG 32717702
rs4285314
HG00117 >hsa-mir-612 AGGGCTTCTGAGCTCCTTAGCA GGGCTTCTGAGCTCCTTAGCAC
CAGGGCTTCTGAGCTCCTTAGC GCAGGGCTTCTGAGCTCCTTAG
GGCAGGGCTTCTGAGCTCCTTA 65211940 rs550894

HG00117 >hsa-mir-548ap AGTAATTGCAGTCTTTGTCATT
AAGTAATTGCAGTCTTTGTCAT AAAGTAATTGCAGTCTTTGTCA
AAAAGTAATTGCAGTCTTTGTC CAAAAGTAATTGCAGTCTTTGT 86368898
rs4414449
HG00117 >hsa-mir-548ap AGTAATTGCAGTCTTTGTCATT
AAGTAATTGCAGTCTTTGTCAT AAAGTAATTGCAGTCTTTGTCA
AAAAGTAATTGCAGTCTTTGTC CAAAAGTAATTGCAGTCTTTGT 86368898
rs4414449 86368959 rs4577031
HG00117 >hsa-mir-548ap AGTAATTGCGGTCTTTGTCATT
AAGTAATTGCGGTCTTTGTCAT AAAGTAATTGCGGTCTTTGTCA
AAAAGTAATTGCGGTCTTTGTC CAAAAGTAATTGCGGTCTTTGT 86368959
rs4577031
HG00117 >hsa-mir-196a-2 AGTTTCATGTTGTTGGGATTGA
TAGTTTCATGTTGTTGGGATTG AGGTAGTTTCATGTTGTTGGGA
GTAGTTTCATGTTGTTGGGATT GGTAGTTTCATGTTGTTGGGAT 54385599
rs11614913
HG00117 >hsa-mir-3622b ATGGGAGGTCAGGTGAGCTCAG
CATGGGAGGTCAGGTGAGCTCA GCATGGGAGGTCAGGTGAGCTC
AGGCATGGGAGGTCAGGTGAGC GGCATGGGAGGTCAGGTGAGCT 27559214
rs66683138
HG00117 >hsa-mir-500bCTTGCTACCTGGGTGAGAGTGC ATCCTTGCTACCTGGGTGAGAG
AATCCTTGCTACCTGGGTGAGA CCTTGCTACCTGGGTGAGAGTG
TCCTTGCTACCTGGGTGAGAGT 49775351 rs151318590

HG00117 >hsa-mir-612 GGGCTTCTGAGCTCCTTAGCAC AGGGCTTCTGAGCTCCTTAGCA
CAGGGCTTCTGAGCTCCTTAGC GCAGGGCTTCTGAGCTCCTTAG
GGCAGGGCTTCTGAGCTCCTTA 65211979 rs12803915

HG00117 >hsa-mir-612 GGGCTTCTGAGCTCCTTAGCAC AGGGCTTCTGAGCTCCTTAGCA
CAGGGCTTCTGAGCTCCTTAGC GCAGGGCTTCTGAGCTCCTTAG
GGCAGGGCTTCTGAGCTCCTTA 65211940 rs550894 65211979
rs12803915
HG00117 >hsa-mir-548h-3 GTAATCGTGGTTTTTGTCATTG
TGCAAAAGTAATCGTGGTTTTT TGGTGCAAAAGTAATCGTGGTT
TTGGTGCAAAAGTAATCGTGGT AGTAATCGTGGTTTTTGTCATT 13446924
rs9913045
HG00117 >hsa-mir-573 GTGTAACTGATCAGGATCTACT TGTGTAACTGATCAGGATCTAC
GATGTGTAACTGATCAGGATCT TGATGTGTAACTGATCAGGATC
GTGATGTGTAACTGATCAGGAT 24521902 rs76014664

HG00117 >hsa-mir-573 GTGTAACTGATCAGGATTTACT TGTAACTGATCAGGATTTACTC
ATGTGTAACTGATCAGGATTTA TGTGTAACTGATCAGGATTTAC
GATGTGTAACTGATCAGGATTT 24521871 rs192259662

HG00117 >hsa-mir-573 GTGTAACTGATCAGGATTTACT TGTAACTGATCAGGATTTACTC
ATGTGTAACTGATCAGGATTTA TGTGTAACTGATCAGGATTTAC
GATGTGTAACTGATCAGGATTT 24521871 rs192259662 24521902
rs76014664

HG00117 >hsa-mir-3117TAAAGGGCCAGACACTATACGA GCCAGACACTATACGAGTCATA
GGGCCAGACACTATACGAGTCA GGCCAGACACTATACGAGTCAT
CCCTAAAGGGCCAGACACTATA 67094171 rs12402181

HG00117 >hsa-mir-576 TAATTTCTCCACGTCTTTGGTA TCTAATTTCTCCACGTCTTTGG
TTCTAATTTCTCCACGTCTTTG CTAATTTCTCCACGTCTTTGGT
GATTCTAATTTCTCCACGTCTT 110409933 rs77639117

HG00117 >hsa-mir-629 TACGTTGGGAGAACTTTTACGG TTACGTTGGGAGAACTTTTACG
GTTTACGTTGGGAGAACTTTTA TTTACGTTGGGAGAACTTTTAC
GGTTTACGTTGGGAGAACTTTT 70371794 .

HG00117 >hsa-mir-629 TACGTTGGGAGAACTTTTATGG TTACGTTGGGAGAACTTTTATG
TTTACGTTGGGAGAACTTTTAT TGGGTTTACGTTGGGAGAACTT
GTTTACGTTGGGAGAACTTTTA 70371761 rs377691713

HG00117 >hsa-mir-629 TACGTTGGGAGAACTTTTATGG TTACGTTGGGAGAACTTTTATG
TTTACGTTGGGAGAACTTTTAT TGGGTTTACGTTGGGAGAACTT
GTTTACGTTGGGAGAACTTTTA 70371761 rs377691713 70371794 .

HG00117 >hsa-mir-888 TACTCAAAAAGCTTTCAGTCAC TCTACTCAAAAAGCTTTCAGTC
TGCTCTACTCAAAAAGCTTTCA GCTCTACTCAAAAAGCTTTCAG
GTGCTCTACTCAAAAAGCTTTC 145076355 rs143634721

HG00117 >hsa-mir-520gTAGAGGAAGCACTTTCTGTTTG TCTAGAGGAAGCACTTTCTGTT
TGACCCTCTAGAGGAAGCACTT TGTGACCCTCTAGAGGAAGCAC
TGCTGTGACCCTCTAGAGGAAG 54225460 rs375062679

HG00117 >hsa-mir-3125TAGAGGAAGCTGTGGAGAGAAC AGCTGTGGAGAGAACTCACGGT
TGGATAGAGGAAGCTGTGGAGA AAGCTGTGGAGAGAACTCACGG
AGGAAGCTGTGGAGAGAACTCA 12877501 rs78852835

HG00117 >hsa-mir-3199-1 TAGGAGAAAGTTTCTGGAAGTT
TTAGGAGAAAGTTTCTGGAAGT TGCCTTAGGAGAAAGTTTCTGG
TCCAGGGACTGCCTTAGGAGAA TGACTCCAGGGACTGCCTTAGG 28316513
rs118160653

HG00117 >hsa-mir-449cTAGGCAGTGTATTGCTAGCGGC TCAGATAGGCAGTGTATTGCTA
TGTCAGATAGGCAGTGTATTGC TGTGTCAGATAGGCAGTGTATT
TGGGATGTGTCAGATAGGCAGT 54468166 rs75661995

HG00117 >hsa-mir-590 TATTCATAAAAGTGCAGTATGG TTATTCATAAAAGTGCAGTATG TTTATTCATAAAAGTGCAGTAT TGAGTTTATTCATAAAAGTGCA GTTTATTCATAAAAGTGCAGTA 73605546 rs189727189

HG00117 >hsa-mir-3922TCAAGGCCAGAGGTCCCACAAC TCAAGTCAAGGCCAGAGGTCCC GCCAGAGGTCCCACAACAGGGC GGCCAGAGGTCCCACAACAGGG GTCAAGGCCAGAGGTCCCACAA 104985443 rs61938575

HG00117 >hsa-mir-564 TCAGCAGGCAACATGGCCGAGA TGTCAGCAGGCAACATGGCCGA GTCAGCAGGCAACATGGCCGAG TGCCAGGCACGGTGTCAGCAGG GTGTCAGCAGGCAACATGGCCG 44903433 .

HG00117 >hsa-mir-222 TCAGTAGCCAGTGTAGATCCTG TGGCTCAGTAGCCAGTGTAGAT TTGGCTCAGTAGCCAGTGTAGA TCATTGGCTCAGTAGCCAGTGT TACCCTCATTGGCTCAGTAGCC 45606504 rs191727254

HG00117 >hsa-mir-515-1 TCCAAAAGAAAGCACTTTCTGT TCTCCAAAAGAAAGCACTTTCT TTCTCCAAAAGAAAGCACTTTC TCATTCTCCAAAAGAAAGCACT TGCAGTCATTCTCCAAAAGAAA 54182326 rs374576826

HG00117 >hsa-mir-663aTCCCAGGCGGGGCGCCGCGGGA TCCGGCGTCCCAGGCGGGGCGC TTCCGGCGTCCCAGGCGGGGCG GCGCCGCGGGACCTCCCTCGTG GGCGCCGCGGGACCTCCCTCGT 26188880 .

HG00117 >hsa-mir-149 TCCGTGTCTTCACTCCCGTGCT TGGCTCCGTGTCTTCACTCCCG TCTGGCTCCGTGTCTTCACTCC CCGTGTCTTCACTCCCGTGCTT CTCCGTGTCTTCACTCCCGTGC 241395503 rs2292832

HG00117 >hsa-mir-618 TCCTTCTGAGTGTAATTACGTA TGTCCTTCTGAGTGTAATTACG TTGTCCTTCTGAGTGTAATTAC TACTTGTCCTTCTGAGTGTAAT GTCCTTCTGAGTGTAATTACGT 81329536 rs2682818

HG00117 >hsa-mir-412 TCGACCAGTTGGAAAGTAATTG TGGTCGACCAGTTGGAAAGTAA TGGGGTACGGGGATGGATGGTC TGGATGGTCGACCAGTTGGAAA TACGGGGATGGATGGTCGACCA 101531854 rs61992671

HG00117 >hsa-mir-412 TCGACCAGTTGGAAAGTAATTG TGGTCGACCAGTTGGAAAGTAA TGGGGTACGGGGATGGATGGTC TGGATGGTCGACCAGTTGGAAA TACGGGGATGGATGGTCGACCA 101531849 . 101531854 rs61992671

HG00117 >hsa-mir-492 TCGAGGACCTGCGGGACAAGAT TACAGGACCATCGAGGACCTGC TACTACAGGACCATCGAGGACC TCCAGCCACTACTACAGGACCA GACCTGCGGGACAAGATTCTTG 95228179 rs200816308

HG00117 >hsa-mir-3183TCGGAGTCGCTCGGAGCAGTCA TCTCGGAGTCGCTCGGAGCAGT TCTCTCGGAGTCGCTCGGAGCA TGCCTCTCTCGGAGTCGCTCGG

TGCCCTGCCTCTCTCGGAGTCG 925742 rs72812091

HG00117 >hsa-mir-4305TCTGGGTTCTTAGAGGCCTAAT TTCTGGGTTCTTAGAGGCCTAA GTTCTGGGTTCTTAGAGGCCTA TCCAGTTCTGGGTTCTTAGAGG CAGTTCTGGGTTCTTAGAGGCC 40238175 rs67976778

HG00117 >hsa-mir-1200TGAGCCATTCTGAGCCTCAGTC TCCTGAGCCATTCTGAGCCTCA TGCTACTTCTCCTGAGCCATTC TCTCCTGAGCCATTCTGAGCCT TTCTCCTGAGCCATTCTGAGCC 36959006 .

HG00117 >hsa-mir-27a TGAGGAGCAGGGCTTAGCTGCT TTAGCTGCTTGTGAGCAGGGTC GAGGAGCAGGGCTTAGCTGCTT GGAGCAGGGCTTAGCTGCTTGT GAGCAGGGCTTAGCTGCTTGTG 13947292 rs895819

HG00117 >hsa-mir-3151TGATGGGTGGGGCAATGGGATC TGGGTGGGGCAATGGGATCAGG TGGGGCAATGGGATCAGGTGCC GGGGTGATGGGTGGGGCAATGG GGGTGATGGGTGGGGCAATGGG 104166902 rs35605502

HG00117 >hsa-mir-3622a TGCACAGGCACAGGAGCTCAGG TAGAGGGTGCACAGGCACAGGA GCACAGGAGCTCAGGTGAGGCA GGCACAGGAGCTCAGGTGAGGC GAGGGTGCACAGGCACAGGAGC 27559214 rs66683138

HG00117 >hsa-mir-3156-3 TGCAGAAGAAAGATCTGGAAGT GCAGAAGAAAGATCTGGAAGTG GAAAGATCTGGAAGTGGGAGAC GAAGAAAGATCTGGAAGTGGGA AGAAGAAAGATCTGGAAGTGGG 14778721 rs2747232

HG00117 >hsa-mir-3152TGCCTCTGTTCTAACACAAGAC TTGCCTCTGTTCTAACACAAGA TATTGCCTCTGTTCTAACACAA TTATTGCCTCTGTTCTAACACA TGCAGAGTTATTGCCTCTGTTC 18573360 rs13299349

HG00117 >hsa-mir-1254-2 TGGAAGCTGGAGCCTGCAGTGA TGAGCCTGGAAGCTGGAGCCTG GAAGCTGGAGCCTGCAGTGAGC GGAAGCTGGAGCCTGCAGTGAG GCCTGGAAGCTGGAGCCTGCAG 23682383 rs200793185

HG00117 >hsa-mir-516b-2 TGGAGGTAAGAAGCACTTTGTG TCTGGAGGTAAGAAGCACTTTG TGACCATCTGGAGGTAAGAAGC TGTGACCATCTGGAGGTAAGAA TGATGTGACCATCTGGAGGTAA 54228742 rs10670323

HG00117 >hsa-mir-877 TGGCGCAGGGGACACGGGCAAA GACACGGGCAAAGACTTGGGGG GGACACGGGCAAAGACTTGGGG GGGACACGGGCAAAGACTTGGG GGGGACACGGGCAAAGACTTGG 30552187 rs372113020

HG00117 >hsa-mir-1273h TGGGAGGTCAAGGCTGTAGTGT TGAGCCTGGGAGGTCAAGGCTG TTGAGCCTGGGAGGTCAAGGCT TGCTTGAGCCTGGGAGGTCAAG TTGCTTGAGCCTGGGAGGTCAA 24214486 .

HG00117 >hsa-mir-3620TGGGGGCCAGCAGGGAGTGGGT TGAGGTGGGGGCCAGCAGGGAG

GGGGGCCAGCAGGGAGTGGGTT GTGGGGGCCAGCAGGGAGTGGG
GGTGGGGGCCAGCAGGGAGTGG 228284991 rs2070960

HG00117 >hsa-mir-1227TGGTGGGCACTGCTGGGGTGGG AGGCGGTGGTGGGCACTGCTGG
TGGGGCCAGGCGGTGGTGGGCA GGTGGGCACTGCTGGGGTGGGC
GTGGTGGGCACTGCTGGGGTGG 2234093 rs190788838

HG00117 >hsa-mir-323bTGTCCGTGGTGAGTTCGCATTA TTGTCCGTGGTGAGTTCGCATT
TACTCGGAGGGAGGTTGTCCGT TCGGAGGGAGGTTGTCCGTGGT
AGGTTGTCCGTGGTGAGTTCGC 101522556 rs56103835

HG00117 >hsa-mir-642bTTCCCTCTCCAAATGTGTCTTG TTGGGAGGTTCCCTCTCCAAAT
TGGGAGGTTCCCTCTCCAAATG GAGTTGGGAGGTTCCCTCTCCA
GTTGGGAGGTTCCCTCTCCAAA 46178217 rs111664333

HG00117 >hsa-mir-4277TTCTGAGCACAGTACACTGGGC TCGAGGCAGTTCTGAGCACAGT
TGGGTCGAGGCAGTTCTGAGCA GTTCTGAGCACAGTACACTGGG
GCAGTTCTGAGCACAGTACACT 1708902 rs115200817

HG00117 >hsa-mir-553 TTTAAAACGGTGAGATTTTGTT TTTTAAAACGGTGAGATTTTGT
ATTTTAAAACGGTGAGATTTTG TATTTTAAAACGGTGAGATTTT
TTATTTTAAAACGGTGAGATTT 100746835 .

HG00117 >hsa-mir-553 TTTAAAACGGTGAGATTTTGTT TTTTAAAACGGTGAGATTTTGT
ATTTTAAAACGGTGAGATTTTG TATTTTAAAACGGTGAGATTTT
TTATTTTAAAACGGTGAGATTT 100746848 .

HG00117 >hsa-mir-553 TTTAAAACGGTGAGATTTTGTT TTTTAAAACGGTGAGATTTTGT
ATTTTAAAACGGTGAGATTTTG TATTTTAAAACGGTGAGATTTT
TTATTTTAAAACGGTGAGATTT 100746835 . 100746848 .

HG00117 >hsa-mir-553 TTTAAGACGGTGAGATTTTGTT TTTTAAGACGGTGAGATTTTGT
ATTTTAAGACGGTGAGATTTTG TATTTTAAGACGGTGAGATTTT
TTATTTTAAGACGGTGAGATTT 100746814 rs190622705

HG00117 >hsa-mir-553 TTTAAGACGGTGAGATTTTGTT TTTTAAGACGGTGAGATTTTGT
ATTTTAAGACGGTGAGATTTTG TATTTTAAGACGGTGAGATTTT
TTATTTTAAGACGGTGAGATTT 100746814 rs190622705 100746835 .

HG00117 >hsa-mir-553 TTTAAGACGGTGAGATTTTGTT TTTTAAGACGGTGAGATTTTGT
ATTTTAAGACGGTGAGATTTTG TATTTTAAGACGGTGAGATTTT
TTATTTTAAGACGGTGAGATTT 100746814 rs190622705 100746848 .

HG00117 >hsa-mir-553 TTTAAGACGGTGAGATTTTGTT TTTTAAGACGGTGAGATTTTGT
ATTTTAAGACGGTGAGATTTTG TATTTTAAGACGGTGAGATTTT
TTATTTTAAGACGGTGAGATTT 100746814 rs190622705 100746835 .
100746848 .

HG00117 >hsa-mir-548a-1 AAAACTGGCAATTACTTTTGCA
AAACTGGCAATTACTTTTGCAC TGGCAATTACTTTTGCACCAAA
AACTGGCAATTACTTTTGCACC 24214486 .

HG00117 >hsa-mir-320c-1 AAAAGCTGGGTTGAGAGGGTAG
AGCTGGGTTGAGAGGGTAGGAA CTGGGTTGAGAGGGTAGGAAAA
AGGGTAGGAAAAAAATGATGTA 154065347 rs142414368 rs3746444

HG00117 >hsa-mir-499bAAACATCACTGCAAGTCTTAAC
154065383 rs75538180
HG00117 >hsa-mir-202 AAAGAGGTATAGGGCATGGGAA AAGAGGTATAGGGCATGGGAAA
GGGAAAACGGGGCGGTCGGGTC TAAAGAGGTATAGGGCATGGGA 154065347
rs142414368 154065383 rs75538180
HG00117 >hsa-mir-576 AAAGATGTGGAAAAATTGGAAT AAGATGTGGAAAAATTGGAATC
TGGAAAAATTGGAATCCTCTTT GATGTGGAAAAATTGGAATCCT 93466866
rs2155248
HG00117 >hsa-mir-520gAAAGTGCTTCCCTTTAGAGTGT CAAAGTGCTTCCCTTTAGAGTG
AAACAAAGTGCTTCCCTTTAGA AAGTGCTTCCCTTTAGAGTGTT 52013832
rs374103744
HG00117 >hsa-mir-548ap AACAAAAACCACAATTACTTTT
CAAAAACCACAATTACTTTTTA CAATTACTTTTTACTGACCTAA
34963416 rs2986407
HG00117 >hsa-mir-548ap AACAAAAACCACAATTACTTTT
CAAAAACCACAATTACTTTTTA TTACTTTTTACTGACCTAAAGA
34963459 rs11032942
HG00117 >hsa-mir-548ap AACAAAAACCACAATTACTTTT
CAAAAACCACAATTACTTTTTA CAATTACTTTTTACTGACCTAA
34963416 rs2986407 34963459 rs11032942

HG00117 >hsa-mir-548ad AACGACAATGACTTTTGCACCA
GGCAAAAACGACAATGACTTTT TGGCAAAAACGACAATGACTTT
AAAAACGACAATGACTTTTGCA 241395503 rs2292832

HG00117 >hsa-mir-548al AACGGCAGTGACTTTTGTACCA
TGGCAAAAACGGCAGTGACTTT TAAAAGTAATGGCAAAAACGGC
AAAAGTAATGGCAAAAACGGCA

HG00117 >hsa-mir-516b-2 AAGAAAGTGCTTCCTTTCAGAG
AAAGAAAGTGCTTCCTTTCAGA AAGAAAAGAAAGTGCTTCCTTT
AAAGTGCTTCCTTTCAGAGGGT

HG00117 >hsa-mir-622 ACACAGTCTGCTGAGGTTGGAG CTGCTGAGGTTGGAGCCGCTGA
ACACAGTCTGCTGAGGTTGGAG 54385599 rs11614913
rs111371406
HG00117 >hsa-mir-24-2ACACTGGCTCAGTTCAGCAGGA CACTGGCTCAGTTCAGCAGGAA
TGGCTCAGTTCAGCAGGAACAG CTGGCTCAGTTCAGCAGGAACA 135061112
rs12355840

HG00117 >hsa-mir-449cACAGTTGCTAGTTGCACTCCTC AACAGTTGCTAGTTGCACTCCT GTTGCTAGTTGCACTCCTCTCT GTTGCACTCCTCTCTGTTGCAT 113655752 rs10505168
HG00117 >hsa-mir-544bACCTGAGGTTGTGCATTTCTAA AGACCTGAGGTTGTGCATTTCT TAGACCTGAGGTTGTGCATTTC GAGGTTGTGCATTTCTAACAAA

HG00117 >hsa-mir-3118-1 ACTGCATTATGAAAATTCTTCT ATTATGAAAATTCTTCTAGTGT GCATTATGAAAATTCTTCTAGT CTGCATTATGAAAATTCTTCTA

HG00117 >hsa-mir-642bAGATACATTTGGAGAGGGACCC TTGGAGAGGGACCCTCCCAACT TTTGGAGAGGGACCCTCCCAAC ATACATTTGGAGAGGGACCCTC 45606504 rs191727254
HG00117 >hsa-mir-3180-4 AGCGGAGGGTGAAGCCTCCGGA CGGAGGGTGAAGCCTCCGGATG GAGCGGAGGGTGAAGCCTCCGG GCGGAGGGTGAAGCCTCCGGAT 13947170 .

HG00117 >hsa-mir-3180-4 AGCGGAGGGTGAAGCCTCCGGA CGGAGGGTGAAGCCTCCGGATG GAGCGGAGGGTGAAGCCTCCGG GCGGAGGGTGAAGCCTCCGGAT 13947292 rs895819

HG00117 >hsa-mir-30d AGCTTTCAGTCAGATGTTTGCT GGCTAAGCTTTCAGTCAGATGT GCTAAGCTTTCAGTCAGATGTT TTCAGTCAGATGTTTGCTGCTA

HG00117 >hsa-mir-519a-2 AGGAAAGTGCATCCTTTTAGAG AGTGCATCCTTTTAGAGGGTTA GGAAAGTGCATCCTTTTAGAGG GAAAGGAAAGTGCATCCTTTTA 135817150 .

HG00117 >hsa-mir-630 AGTATTCTGTACCAGGGAAGGT ACCTAGTATTCTGTACCAGGGA CCAGGGAAGGTAGTTCTTAACT CAGGGAAGGTAGTTCTTAACTA 67094171 rs12402181
HG00117 >hsa-mir-3686AGTGATCTGTAAGAGAAAGTAA TCTGTAAGAGAAAGTAAATGAA GTAAGAGAAAGTAAATGAAAGA ACAGTGATCTGTAAGAGAAAGT 142667330 rs76132421
HG00117 >hsa-mir-323bATACACGGTCGACCTCTTTTCG TACACGGTCGACCTCTTTTCGG ACACGGTCGACCTCTTTTCGGT 104166902 rs35605502 rs56103835
HG00117 >hsa-mir-642aATTTGGAGAGGGAACCTCCCAA AGACACATTTGGAGAGGGAACC ACACATTTGGAGAGGGAACCTC CACATTTGGAGAGGGAACCTCC 18573360 rs13299349
HG00117 >hsa-mir-412 CACCTGGTCCACTGGCCGTCCG ACCTGGTCCACTGGCCGTCCGT CTGGCCGTCCGTATCCGCTGCA TCACCTGGTCCACTGGCCGTCC 15248720 rs75000738
HG00117 >hsa-mir-412 CACCTGGTTCACTGGCCGTCCG ACCTGGTTCACTGGCCGTCCGT CTGGCCGTCCGTATCCGCTGCA TCACCTGGTTCACTGGCCGTCC 15248798 rs183853838
HG00117 >hsa-mir-515-1 CAGAGTGCCTTCTTTTGGAGCA

GAGTGCCTTCTTTTGGAGCATT TGCCTTCTTTTGGAGCATTACT
GTGCCTTCTTTTGGAGCATTAC 15248720 rs75000738 15248798
rs183853838
HG00117 >hsa-mir-1227CATTTGACCCCGTGCCACCCTT ATTTGACCCCGTGCCACCCTTT
AGGCATTTGACCCCGTGCCACC GACCCCGTGCCACCCTTTTCCC

HG00117 >hsa-mir-3151CCACCTGATCCCACACCCCACC CACCTGATCCCACACCCCACCT
CCCACCTGATCCCACACCCCAC TGATCCCACACCCCACCTGTCA

HG00117 >hsa-mir-1343CCCCTCCTGGGGCCCGCACTCT CCCTCCTGGGGCCCGCACTCTC
CCTGGGGCCCGCACTCTCGCTC TGGGGCCCGCACTCTCGCTCTG 18392913
rs7247767
HG00117 >hsa-mir-1343CCCCTCCTGGGGCCCGCACTCT CCTGGGGCCCGCACTCTCGCTC
CCCTCCTGGGGCCCGCACTCTC TGGGGCCCGCACTCTCGCTCCG 19263542
.
HG00117 >hsa-mir-3679CCCTTCCCCCCAGTAATCTTCA CCTTCCCCCCAGTAATCTTCAT
CTTCCCCCCAGTAATCTTCATC TCCCCCCAGTAATCTTCATCAT

HG00117 >hsa-mir-3622b CCTCACCTGAGCTCCTGTGCCT
CCTGAGCTCCTGTGCCTGTGCA CTCCTGTGCCTGTGCACCCTCT
CTGAGCTCCTGTGCCTGTGCAC

HG00117 >hsa-mir-4268CCTCTCAGGATGTGATGTCACC CTCCTCTCAGGATGTGATGTCA
CTCCTCCTCTCAGGATGTGATG

HG00117 >hsa-mir-4254CCTGGAGATACTCCACCATCTC AGATACTCCACCATCTCCCCCA
GGAGATACTCCACCATCTCCCC 72744798 rs745666
rs4674470
HG00117 >hsa-mir-3152CCTGTGTTAGAATAAGGGCAAT TTAGAATAAGGGCAATAACTCT
AGAATAAGGGCAATAACTCTGC TGTGTTAGAATAAGGGCAATAA
rs12731294
HG00117 >hsa-mir-3620CTCACCCTGCATCCCGCACCCA CCTCACCCTGCATCCCGCACCC
CTCACCCTGCATCCCGCACCCA 27559214 rs66683138
rs2070960
HG00117 >hsa-mir-3622a CTCACCTGACCTCCCATGCCTG
CCTGACCTCCCATGCCTGTGCA GCTCACCTGACCTCCCATGCCT
CTGACCTCCCATGCCTGTGCAC 27559214 rs66683138

HG00117 >hsa-mir-3117CTCATATAGTGCCAGGTGTTTT GACTCATATAGTGCCAGGTGTT
TCATATAGTGCCAGGTGTTTTG ATAAGACTCATATAGTGCCAGG 134884700
rs6430498
HG00117 >hsa-mir-1343CTCCTGGGGCCCGCACTCTCGC CCCTCCTGGGGCCCGCACTCTC
CCTGGGGCCCGCACTCTCGCTC TGGGGCCCGCACTCTCGCTCCG 130496365
rs6997249
HG00117 >hsa-mir-196a-2 CTCGGCAACAAGAAACTGTCTG
CAAGAAACTGTCTGAGTTACAT CAACAAGAAACTGTCTGAGTTA
ACAAGAAACTGTCTGAGTTACA 35731712 rs34874675

HG00117 >hsa-mir-3615CTCTCTCGGCTCCTCGCGGCTC GGCTCCTCGCGGCTCGCGGCGG CGGCTCCTCGCGGCTCGCGGCG TCGGCTCCTCGCGGCTCGCGGC 104985443 rs61938575
HG00117 >hsa-mir-888 CTCTTTGGGTGAAGGAAGGCTC CTGACACCTCTTTGGGTGAAGG GACTGACACCTCTTTGGGTGAA CCTCTTTGGGTGAAGGAAGGCT

HG00117 >hsa-mir-1304CTGTAGCATCGAACCCCTGGGC CTCACTGTAGCATCGAACCCCT GAACCCCTGGGCTCAAGTGATT CGAACCCCTGGGCTCAAGTGAT 131701279 rs367805
HG00117 >hsa-mir-3922CTGTGGGACTTCTGGCCTTGAC ACCTGTGGGACTTCTGGCCTTG GGGACTTCTGGCCTTGACTTGA TGGGACTTCTGGCCTTGACTTG 101531849 .
HG00117 >hsa-mir-940 GAAGGCAGGGCCCC-GCTCCCC G CCC-GCTCCCCGGGCCTGACCC 101531854 rs61992671 rs35356504

HG00117 >hsa-mir-4274GACCCAGCAGTCCCTCCCCCTG CCCAGCAGTCCCTCCCCCTGCA TGACCCAGCAGTCCCTCCCCCT TCAGGTGACCCAGCAGTCCCTC 101531849 . 101531854 rs61992671
HG00117 >hsa-mir-3188GAGGCTTTGTGCGGATACGGGG GAGAGGCTTTGTGCGGATACGG GCGGATACGGGGCTGGAGGCCT rs7247237

HG00117 >hsa-mir-3188GAGGCTTTGTGCGGATACGGGG GGAGAGGCTTTGTGCGGATACG GAGAGGCTTTGTGCGGATACGG rs7247767

HG00117 >hsa-mir-3188GAGGCTTTGTGCGGATACGGGG GAGAGGCTTTGTGCGGATACGG GGAGAGGCTTTGTGCGGATACG 7461769 rs12512664 rs7247237
HG00117 >hsa-mir-629 GAGGTTCTCCCAACGTAAGCCC AGGTTCTCCCAACGTAAGCCCA TCTCCCAACGTAAGCCCAGCCC CAGGAGGTTCTCCCAACGTAAG 54468166 rs75661995
HG00117 >hsa-mir-629 GAGGTTCTCCCAACGTAAGCCC AGGAGGTTCTCCCAACGTAAGC GGAGGTTCTCCCAACGTAAGCC TCTCCCAACGTAAGCCCAGCCC 33578251 rs3746444
HG00117 >hsa-mir-629 GAGGTTCTCCCAACGTAAGCCC AGGTTCTCCCAACGTAAGCCCA TCTCCCAACGTAAGCCCAGCCC CCCAACGTAAGCCCAGCCCCTC

HG00117 >hsa-mir-222 GCAGCTACATCTGGCTACTGGG TACTGGGTCTCTGATGGCATCT GCTACTGGGTCTCTGATGGCAT CTGGCTACTGGGTCTCTGATGG 49775351 rs151318590
HG00117 >hsa-mir-500bGCAGTGCACCCAGGCAAGGATT CACCCAGGCAAGGATTCTGCGA AGGCAAGGATTCTGCGAGGGGG TGCAGTGCACCCAGGCAAGGAT 54182326 rs374576826
HG00117 >hsa-mir-151bGCCCTCGAGGAGCTCACAGTCT TCTAGACAAACAAACTCAGGGT AGCTCACAGTCTAGACAAACAA . 54228742 rs10670323

HG00117 >hsa-mir-3180-4 GCGGAGGGTGAAGCCTCCGGAT

CGCTGGCCTGGTCGCGCTGTGG TCGCTGGCCTGGTCGCGCTGTG
AAGCCTCCGGATGCCAGTCCCT 54265670 .

HG00117 >hsa-mir-149 GGGAGGGAGGGACGGGGGCTGT GGAGGGACGGGGGCTGTGCTGG
AGGGACGGGGGCTGTGCTGGGG GAGGAGGGAGGGAGGGACGGGG 54225460
rs375062679
HG00117 >hsa-mir-877 GTCCTCTTCTCCCTTCTCCCAG TGGGACCCTCAGACGTGTGTCC
AGACGTGTGTCCTCTTCTCCCT CTGGGACCCTCAGACGTGTGTC 124451312
rs10934682
HG00117 >hsa-mir-27a GTGTTCACAGTGGCTAAGTTCC TCGTGTTCACAGTGGCTAAGTT
AGTGGCTAAGTTCCGCCCCCCA CACAGTGGCTAAGTTCCGCCCC 18572056
rs12197631
HG00117 >hsa-mir-3936TAAGGGGTGTATGGCAGATGCA TTCTGGTAAGGGGTGTATGGCA
CACCCGACAGATGCACTTGGCA TGTATGGCAGATGCACCCGACA 35696519
rs62143301 rs12894467
HG00117 >hsa-mir-300 TATACAAGGGCAGACTCTCTCT TGATTATACAAGGGCAGACTCT
ATTATACAAGGGCAGACTCTCT 74110353 rs515924

HG00117 >hsa-mir-499aTCACAGCAAGTCTGTGCTGCTT ACAGCAAGTCTGTGCTGCTTCC
CGTCACAGCAAGTCTGTGCTGC TCCCTACGCTGCCTGGGCAGGG

HG00117 >hsa-mir-125b-2 TCACAGGTCAGGCTCTTGGGAC
CAGGTCAGGCTCTTGGGACCTA GCTCTTGGGACCTAGGCGGAGG
CAGGCTCTTGGGACCTAGGCGG

HG00117 >hsa-mir-412 TCACCTGGTTCACTAGCCGTCC TCACCTGGTTCACTAGCCGTCC
ATGTACTTCACCTGGTTCACTA CTTCACCTGGTTCACTAGCCGT 86368959
rs4577031
HG00117 >hsa-mir-558 TCCTGAGCTGCTGTACCAAAAT TTCCTGAGCTGCTGTACCAAAA
GAGCTGCTGTACCAAAATACCA CCTGAGCTGCTGTACCAAAATA

HG00117 >hsa-mir-1273h TGCTGCAGACTCGACCTCCCAG
TGCAGACTCGACCTCCCAGGCT CTGCAGACTCGACCTCCCAGGC
AGACTCGACCTCCCAGGCTTAA

HG00117 >hsa-mir-590 TGTAATTTTATGTATAAGCTAG AATCTGTAATTTTATGTATAAG
GTATAAGCTAGTCTCTGATTGA TTTTATGTATAAGCTAGTCTCT

HG00117 >hsa-mir-3909TGTCCTCTA-GGCCTGCAGTCT TA-GGCCTGCAGTCTCATGGGA
GGCCTGCAGTCTCATGGGAGA CCTGCAGTCTCATGGGAGAGTG 32757230
rs72089144
HG00117 >hsa-mir-2053TGTTAATTAAACCTCTATTTAC ACTTTAAGTGTTAATTAAACCT
TTTAAGTGTTAATTAAACCTCT GTTAATTAAACCTCTATTTACA 110409933
rs77639117
HG00117 >hsa-mir-585 TTGGACGTATCTGTATGCTAGG ACGTATCTGTATGCTAGGGCTG
TGGACGTATCTGTATGCTAGGG TATCTGTATGCTAGGGCTGCTG 36148057
rs115089112

HG00117 >hsa-mir-585 TTGGACGTATCTGTATGCTAGG TATCTGTATGCTAGGGCTGCCG ACGTATCTGTATGCTAGGGCTG TGGACGTATCTGTATGCTAGGG 168690612 rs62376934

HG00117 >hsa-mir-585 TTGGGCGTATCTGTATGCTAGG TATCTGTATGCTAGGGCTGCCG TGGGCGTATCTGTATGCTAGGG GCGTATCTGTATGCTAGGGCTG 168690635 rs62376935

HG00117 >hsa-mir-1303TTTAGAGACGGGGTCTTGCTCT TAGAGACGGGGTCTTGCTCTGT ACGGGGTCTTGCTCTGTTGCCA GGGTCTTGCTCTGTTGCCAGGC 168690612 rs62376934 168690635 rs62376935

HG00117 >hsa-mir-1303TTTAGAGACGGGGTCTTGCTCT TAGAGACGGGGTCTTGCTCTGT TTAGAGACGGGGTCTTGCTCTG ACGGGGTCTTGCTCTGTTGCCA 73605546 rs189727189

HG00117 >hsa-mir-1303TTTAGAGACGGGGTCTTGCTCT TAGAGACGGGGTCTTGCTCTGT TTAGAGACGGGGTCTTGCTCTG ACGGGGTCTTGCTCTGTTGCCA

HG00117 >hsa-mir-580 TTTGAGAATGATGAATCATTAG GATGAATCATTAGGTTCCGGTC AATGATGAATCATTAGGTTCCG AGAATGATGAATCATTAGGTTC 70371761 rs377691713

HG00117 >hsa-mir-133bTTTGGTCCCCTTCAACCAGCTA TGGTCCCCTTCAACCAGCTACA CCTTCAACCAGCTACAGCAGGG AGAGGTTTGGTCCCCTTCAACC 70371794 .
