# Supplementary material for: Role of SNPs in the Biogenesis of Mature miRNAs
Source: Biomed Res Int. 2021 Jun 17;2021:2403418. doi: 10.1155/2021/2403418 (PMC8233088; doi:10.1155/2021/2403418)
Supplement: Supplementary Materials — Additional file 1 Table S1: the database miRSNPBase (xls). Additional file 2 Table S2: the list of pre-miRNAs in miRSNPBase which is classified based on mature miRNA in the 5′ arm or 3′ arm (xls). Additional file 3 Table S3: all the iso-pre-miRNAs, nor-pre-miRNAs, nor-SNPs, and iso-SNPs associated with four splicing sites (xls). Additional file 4 Table S4: the pre-miRNAs and SNPs associated with the normal and isomiRs (xls). Additional file 5 Table S5: the pre-miRNAs, iso-SNPs, and isomiRs of HG00097 (xls). Additional file 6 Table S6: the isomiRs and iso-SNP of 18 GBR populations (xls). Additional file 7 Table S7: the verified isomiRs of 18 GBR (xls). Additional file 8 Table S8: the iso-pre-miRNA candidates and the verified iso-pre-miRNAs of 18 GBR samples (xls). [file 2403418.f1.zip › 2403418.f1/Supp Tab S8.pdf]

Samples iso-pre-miRNA isomiR read count isomiR read count isomiR
read count isomiR read count isomiR read count

HG000100 >hsa-mir-3188GAGGCTTTGTGCGGATACGGGG 6
CTTGCTACCTGGGTGAGAGTGC 1 AATCCTTGCTACCTGGGTGAGA 8

HG000100 >hsa-mir-486-2
GCCTGGGAGGTCAAGGCTGTAG 1 ATTGCTTGAGCCTGGGAGGTCA 1

HG000100 >hsa-mir-3679 GCTAAGCTTTCAGTCAGATGTT
1 GACCCCGTGCCACCCTTTTCCC 1

HG000100 >hsa-mir-3199-2 TAGGAGAAAGTTTCTGGAATGT 6
TAGTTTCATGTTGTTGGGATTG 1 AGGTAGTTTCATGTTGTTGGGA 1
GTACTGAGCTGCCCCGAGCTGG 1

HG000100 >hsa-mir-1294 GCTAAGCTTTCAGTCAGATGTT
1 GTAGTTTCATGTTGTTGGGATT 1

HG000100 >hsa-mir-24-2 ATCGACTCGGCGTGGCGTCGGT
2 TGGGACTTCTGGCCTTGACTTG 4

HG000100 >hsa-mir-650 GAGGCAGCGCTCTCAGGACGTC 2
CTTGCTACCTGGGTGAGAGTGC 1 AATCCTTGCTACCTGGGTGAGA 7
TGGGTTTACGTTGGGAGAACTT 178

HG000100 >hsa-mir-30d AATCCTTGCTACCTGGGTGAGA
4 TGGGTTTACGTTGGGAGAACTT 42

HG000100 >hsa-mir-650 GAGGCAGCGCTCTCAGGACGTC 2
CTTGCTACCTGGGTGAGAGTGC 1 AATCCTTGCTACCTGGGTGAGA 7
TTTACGTTGGGAGAACTTTTAC 157 TTGAGCCTGGGAGGTCAAGGCT

HG000100 >hsa-mir-629 AATCCTTGCTACCTGGGTGAGA
6 TTTACGTTGGGAGAACTTTTAC 84

HG000100 >hsa-mir-1304TCTCACTGTAGCATCGAACCCC 29
TGCAGACTCGACCTCCCAGGCT 75 CTGCAGACTCGACCTCCCAGGC 76

HG000100 >hsa-mir-3909TGTCCTCTAGGGCCTGCAGTCT 4
TGCAGACTCGACCTCCCAGGCT 46 CTGCAGACTCGACCTCCCAGGC 49

HG000100 >hsa-mir-1294ACAACAGTGCCAACCTCACGGG 1
AGACACATTTGGAGAGGGAACC 1
TCCTTGCTACCTGGGTGAGAGT 3

HG000100 >hsa-mir-500bATCCTTGCTACCTGGGTGAGAG 3
AGGTAGTTTCATGTTGTTGGGA 2
GCCTGGAAGCTGGAGCCTGCAG 10

HG000100 >hsa-mir-500bATCCTTGCTACCTGGGTGAGAG 3
AGGTAGTTTCATGTTGTTGGGA 1
TCCTTGCTACCTGGGTGAGAGT 3

HG000100 >hsa-mir-1227CATTTGACCCCGTGCCACCCTT 1
ATTTGACCCCGTGCCACCCTTT 1
GTGATGTGTAACTGATCAGGAT 6

HG000100 >hsa-mir-3922CTGTGGGACTTCTGGCCTTGAC 2
CTCACTGTAGCATCGAACCCCT 29

HG000100 >hsa-mir-3199-2 TAGGAGAAAGTTTCTGGAATGT 6
TCCGGCGTCCCAGGCGGGGCGC 1
TCCTTGCTACCTGGGTGAGAGT 5
HG000100 >hsa-mir-449cTAGGCAGTGTATTGCTAGCGGC 2
TCCGGCGTCCCAGGCGGGGCGC 1
GTACTGAGCTGCCCCGAGCTGG 6
HG000100 >hsa-mir-449cTAGGCAGTGTATTGCTAGCGGC 2
TCCGGCGTCCCAGGCGGGGCGC 2
GCCTGGAAGCTGGAGCCTGCAG 16
HG000100 >hsa-mir-222 TCAGTAGCCAGTGTAGATCCTG 5
TCCGGCGTCCCAGGCGGGGCGC 1
GTGATGTGTAACTGATCAGGAT 3
HG000100 >hsa-mir-222 TCAGTAGCCAGTGTAGATCCTG 5
TCCGGCGTCCCAGGCGGGGCGC 1
TTGCTTGAGCCTGGGAGGTCAA 2
HG000100 >hsa-mir-1303TTTAGAGACGGGGTCTTGCTCT 3
TGTTGCCACTAACCTCAACCTT 1
HG000100 >hsa-mir-1254-2
GGTTTACGTTGGGAGAACTTTT 1
HG000100 >hsa-mir-573
GCCTGGAAGCTGGAGCCTGCAG 2
HG000101 >hsa-mir-744 AGAGGCTTTGTGCGGATACGGG
2 CCTGTACTGAGCTGCCCCGAGC 4
HG000101 >hsa-mir-1303TTTAGAGACGGGGTCTTGCTCT 7
TTACGTTGGGAGAACTTTTACG 3 GTTTACGTTGGGAGAACTTTTA 2
CCTGTACTGAGCTGCCCCGAGC 6
HG000101 >hsa-mir-30d AGCTTTCAGTCAGATGTTTGCT 1
AGGCCCCTCAGTCTTGCTTCCT 1 TCTGAGGCCCCTCAGTCTTGCT 1
CCTTGCTACCTGGGTGAGAGTG 2
HG000101 >hsa-mir-548ac CAAAAACCGGCAATTACTTTTG 1
ATCCTTGCTACCTGGGTGAGAG 9 AATCCTTGCTACCTGGGTGAGA 14
CTGGCTACTGGGTCTCTGATGG
HG000101 >hsa-mir-3909TGTCCTCTAGGGCCTGCAGTCT 7
TGCAGACTCGACCTCCCAGGCT 203 CTGCAGACTCGACCTCCCAGGC 206

HG000101 >hsa-mir-1307ACCGGACCTCGACCGGCTCGTC 1
AGACACATTTGGAGAGGGAACC 4
HG000101 >hsa-mir-500bATCCTTGCTACCTGGGTGAGAG 7
AGGTAGTTTCATGTTGTTGGGA 2
GCCTGGAAGCTGGAGCCTGCAG 14
HG000101 >hsa-mir-500bGCAGTGCACCCAGGCAAGGATT 1
GGGCTTCTGAGCTCCTTAGCAC 3
HG000101 >hsa-mir-27a GTGTTCACAGTGGCTAAGTTCC 1
GTAGATAAAATATTGGTACCTG 213
HG000101 >hsa-mir-877 TAGAGGAGATGGCGCAGGGGAC 19
TAGAGACGGGGTCTTGCTCTGT 1
TTGCTTGAGCCTGGGAGGTCAA 2

HG000101 >hsa-mir-580 TTTGAGAATGATGAATCATTAG 1
GGTTTACGTTGGGAGAACTTTT 1
HG000101 >hsa-mir-1254-2
TTTGAGAATGATGAATCATTAG 1
HG000101 >hsa-mir-744

HG000101 >hsa-mir-580
GTGATGTGTAACTGATCAGGAT 1
HG000101 >hsa-mir-486-2

HG000101 >hsa-mir-629
TTGCTTGAGCCTGGGAGGTCAA 1
HG000101 >hsa-mir-27a

HG000101 >hsa-mir-642a
GCCTGGAAGCTGGAGCCTGCAG 1
HG000101 >hsa-mir-1304
GGTAGTTTCATGTTGTTGGGAT 1
HG000101 >hsa-mir-744
TTGCTTGAGCCTGGGAGGTCAA 1
HG000101 >hsa-mir-1200
TTGCTTGAGCCTGGGAGGTCAA 1
HG000101 >hsa-mir-3176
TTGCTTGAGCCTGGGAGGTCAA 1
HG000101 >hsa-mir-1273h

HG000102 >hsa-mir-222 GCAGCTACATCTGGCTACTGGG 1
GGCTCCTCGCGGCTCGCGGCGG 2 CGGCTCCTCGCGGCTCGCGGCG 2
CCTGTACTGAGCTGCCCCGAGC 5
HG000102 >hsa-mir-3188GAGGCTTTGTGCGGATACGGGG 6
GAAACAATGTCCATTAGGCTTT 1 ACAATGTCCATTAGGCTTTGTT 1
TTCAGTCAGATGTTTGCTGCTA 2
HG000102 >hsa-mir-663aTCCCAGGCGGGGCGCCGCGGGA 1
TGAGCCTGGGAGGTCAAGGCTG 5 TTGAGCCTGGGAGGTCAAGGCT 4
TTTACGTTGGGAGAACTTTTAC 65
HG000102 >hsa-mir-1304TCTCACTGTAGCATCGAACCCC 13
TGCAGACTCGACCTCCCAGGCT 42 CTGCAGACTCGACCTCCCAGGC 39

HG000102 >hsa-mir-877 TAGAGGAGATGGCGCAGGGGAC 25
TAGAGACGGGGTCTTGCTCTGT 1
GTCTCAGGAGGCAGCGCTCTCA 3
HG000102 >hsa-mir-449cTAGGCAGTGTATTGCTAGCGGC 1
TCCGGCGTCCCAGGCGGGGCGC 1
CATCCCGCACCCAG 4
HG000102 >hsa-mir-589 TCAGAACAAATGCCGGTTCCCA 1
TCCGGCGTCCCAGGCGGGGCGC 2 C
6249548

HG000102 >hsa-mir-222 TCAGTAGCCAGTGTAGATCCTG 4
TCGGTGCAAAAGTAATTGCGAG 1
GTACTGAGCTGCCCCGAGCTGG 2
HG000102 >hsa-mir-1303TTTAGAGACGGGGTCTTGCTCT 9
TTACGTTGGGAGAACTTTTACG 2
GCCTGGAAGCTGGAGCCTGCAG 15
HG000102 >hsa-mir-580 TTTGAGAATGATGAATCATTAG 1
GTACTGAGCTGCCCCGAGCTGG 1
HG000105 >hsa-mir-1254-2 TGGAAGCTGGAGCCTGCAGTGA 1
TGCAGACTCGACCTCCCAGGCT 34 CTGCAGACTCGACCTCCCAGGC 38
AAGAGTAGATAAAATATTGGTA
HG000105 >hsa-mir-3909TGTCCTCTAGGGCCTGCAGTCT 2
TGTCAGCAGGCAACATGGCCGA 1 GTCAGCAGGCAACATGGCCGAG 1
ATCCCGCACCCAG1
HG000105 >hsa-mir-3615CTCTCTCGGCTCCTCGCGGCTC 1
CCTGCATCCCGCACCCAG 1 TCCCGCACCCAG 1 TCGGCTCCTCGCGGCTCGCGGC
2
HG000105 >hsa-mir-1304TCTCACTGTAGCATCGAACCCC 3
TGCAGACTCGACCTCCCAGGCT 18 CTGCAGACTCGACCTCCCAGGC 16

HG000105 >hsa-mir-1255b-2 AACCACTTTCTTTGCTCATCCG 1
AAACCACTTTCTTTGCTCATCC 1
TCCTTGCTACCTGGGTGAGAGT 2
HG000105 >hsa-mir-423 AAGCTCGGTCTGAGGCCCCTCA 98
AAACCACTTTCTTTGCTCATCC 2
GTACTGAGCTGCCCCGAGCTGG 8
HG000105 >hsa-mir-573
GTGATGTGTAACTGATCAGGAT 1
HG000105 >hsa-mir-629
GTGATGTGTAACTGATCAGGAT 1
HG000105 >hsa-mir-642a

HG000105 >hsa-mir-149
TTGAGCCTGGGAGGTCAAGGCT 1
HG000106 >hsa-mir-486-2
CTCACTGTAGCATCGAACCCCT 51 CGAACCCCTGGGCTCAAGTGAT

HG000106 >hsa-mir-3620CTGCATCCCGCACCCAG 1 CGTGTTCACAGTGGCTAAGTTC
1 TGTTCACAGTGGCTAAGTTCCG 1 CGACTCGGCGTGGCGTCGGTCG 2

HG000106 >hsa-mir-663aTCCCAGGCGGGGCGCCGCGGGA 3
TGAGCCTGGGAGGTCAAGGCTG 5 TTGAGCCTGGGAGGTCAAGGCT 4
TGGGTTTACGTTGGGAGAACTT 66
HG000106 >hsa-mir-1273h
CTCACTGTAGCATCGAACCCCT 9 TTCAGTCAGATGTTTGCTGCTA

HG000106 >hsa-mir-449cTAGGCAGTGTATTGCTAGCGGC 1

TCCGGCGTCCCAGGCGGGGCGC 1
CATCCCGCACCCAG 4
HG000106 >hsa-mir-222 TCAGTAGCCAGTGTAGATCCTG 5
TCGTGTTCACAGTGGCTAAGTT 1
GCCTGGAAGCTGGAGCCTGCAG 5
HG000106 >hsa-mir-1254-2
TTGAGCCTGGGAGGTCAAGGCT 1
HG000106 >hsa-mir-573
GTGATGTGTAACTGATCAGGAT 1
HG000106 >hsa-mir-744
GTGTCAGCAGGCAACATGGCCG 1
HG000106 >hsa-mir-629
CAAAAGTAATTGCGGTCTTTGT 1
HG000106 >hsa-mir-196a-2

HG000106 >hsa-mir-1273h
GGTAGTTTCATGTTGTTGGGAT 1
HG000106 >hsa-mir-500b

HG000107 >hsa-mir-449cTAGGCAGTGTATTGCTAGCGGC 1
TCCGGCGTCCCAGGCGGGGCGC 1
GGTTTACGTTGGGAGAACTTTT 4
HG000107 >hsa-mir-222 TCAGTAGCCAGTGTAGATCCTG 3
TCGTGTTCACAGTGGCTAAGTT 2 C
7602052
HG000107 >hsa-mir-1254-2

HG000107 >hsa-mir-573

HG000107 >hsa-mir-629
TTGCTTGAGCCTGGGAGGTCAA 1
HG000107 >hsa-mir-486-2

HG000108 >hsa-mir-196a-2
TTAGAGACGGGGTCTTGCTCTG 1 AACAATGTCCATTAGGCTTTGT 1

HG000108 >hsa-mir-943 CCTGACTGTTGCCGTCCTCCAG 3
CACTGGCTCAGTTCAGCAGGAA 1 TGGCTCAGTTCAGCAGGAACAG 2863
CACCCAAGGCTTGCAGAAGAGC
HG000108 >hsa-mir-629 TTAGAGACGGGGTCTTGCTCTG
3 CCTGTACTGAGCTGCCCCGAGC 8
HG000108 >hsa-mir-576 TTAGAGACGGGGTCTTGCTCTG
2 CCTGTACTGAGCTGCCCCGAGC 4
HG000108 >hsa-mir-3620CTGCATCCCGCACCCAG 4 CTCACTGTAGCATCGAACCCCT
195 GAACCCCTGGGCTCAAGTGATT 1 CCTTGCTACCTGGGTGAGAGTG 2

HG000108 >hsa-mir-577 TTAGAGACGGGGTCTTGCTCTG

2 GATGTGGAAAAATTGGAATCCT 4 91
HG000108 >hsa-mir-944 AAATTATTGTACATCGGATGAG 20
AAAAGCTGGGTTGAGAAGGT 1 GGAAAAGCTGGGTTGAGAAGGT 1
TGGGACTTCTGGCCTTGACTTG 2
HG000108 >hsa-mir-222 TCAGTAGCCAGTGTAGATCCTG 2
TCTAATTTCTCCACGTCTTTGG 5 TTCTAATTTCTCCACGTCTTTG 34

HG000108 >hsa-mir-423 AAGCTCGGTCTGAGGCCCCTCA 274
AACGCAGACAATGCCTACTGGC 1
GTACTGAGCTGCCCCGAGCTGG 3
HG000108 >hsa-mir-1307ACCGGACCTCGACCGGCTCGTC 1
AGACACATTTGGAGAGGGAACC 8
HG000108 >hsa-mir-3620CTCACCCTGCATCCCGCACCCA 1
CCTCCCACACCCAAGGCTTGCA 15
HG000108 >hsa-mir-612 GGGCTTCTGAGCTCCTTAGCAC 1
GTAGATAAAATATTGGTACCTG 94
HG000108 >hsa-mir-1303TTTAGAGACGGGGTCTTGCTCT 5
TTACGTTGGGAGAACTTTTACG 1
GTACTGAGCTGCCCCGAGCTGG 3
HG000108 >hsa-mir-1254-2

HG000108 >hsa-mir-27a

HG000108 >hsa-mir-663a
GGTAGTTTCATGTTGTTGGGAT 1
HG000108 >hsa-mir-642a
ATGGATGAGCAAAGAAAGTGGT 1
HG000108 >hsa-mir-500b
GGTAGTTTCATGTTGTTGGGAT 1
HG000108 >hsa-mir-3176

HG000108 >hsa-mir-1304
TTGAGCCTGGGAGGTCAAGGCT 1
HG000108 >hsa-mir-576
CTGAAACAATGTCCATTAGGCT 1
HG000108 >hsa-mir-1273h
GGTAGTTTCATGTTGTTGGGAT 1
HG000109 >hsa-mir-222 TCAGTAGCCAGTGTAGATCCTG 1
TCTAATTTCTCCACGTCTTTGG 17 TTCTAATTTCTCCACGTCTTTG 62

HG000109 >hsa-mir-1307ACCGGACCTCGACCGGCTCGTC 1
AGACACATTTGGAGAGGGAACC 12
HG000109 >hsa-mir-577 AGTGAAGAGTAGATAAAATATT 62
AGGTAGTTTCATGTTGTTGGGA 1
GGTTTACGTTGGGAGAACTTTT 2
HG000109 >hsa-mir-500bATCCTTGCTACCTGGGTGAGAG 5
AGGTAGTTTCATGTTGTTGGGA 1

TCCTTGCTACCTGGGTGAGAGT 3
HG000109 >hsa-mir-3188GAGGCTTTGTGCGGATACGGGG 3
GAGGAGATGGCGCAGGGGACAC 2
GCCTGGAAGCTGGAGCCTGCAG
HG000109 >hsa-mir-573 GTGTAACTGATCAGGATCTACT 3
GTAGATAAAATATTGGTACCTG 68
HG000109 >hsa-mir-1303TTTAGAGACGGGGTCTTGCTCT 12
TTAGCTGCTTGTGAGCAGGGTC 2
TCCTTGCTACCTGGGTGAGAGT 9
HG000110 >hsa-mir-1273h
ACTGGCCTGGGACTACCGGGGG 29 GTCTGAGGCCCCTCAGTCTTGC

HG000110 >hsa-mir-3620 CTGTACTGAGCTGCCCCGAGCT
2 TCGGCTCCTCGCGGCTCGCGGC 3 74
HG000110 >hsa-mir-532 CTGTACTGAGCTGCCCCGAGCT
2 TGATGTGTAACTGATCAGGATC 3
HG000110 >hsa-mir-1304TCTCACTGTAGCATCGAACCCC 24
TGCAGACTCGACCTCCCAGGCT 47 CTGCAGACTCGACCTCCCAGGC 50

HG000110 >hsa-mir-944 AAATTATTGTACATCGGATGAG 3
AAACCACTTTCTTTGCTCATCC 1
GGTAGTTTCATGTTGTTGGGAT 2
HG000110 >hsa-mir-30d AGCTTTCAGTCAGATGTTTGCT 1
AGGCCCCTCAGTCTTGCTTCCT 1
TTGCTTGAGCCTGGGAGGTCAA 2
HG000110 >hsa-mir-3176GGGACTGGCCTGGGACTACCGG 1
GTAGATAAAATATTGGTACCTG 14
HG000110 >hsa-mir-877 TAGAGGAGATGGCGCAGGGGAC 27
TAGAGACGGGGTCTTGCTCTGT 1
GTCTCAGGAGGCAGCGCTCTCA 3
HG000110 >hsa-mir-222 TCAGTAGCCAGTGTAGATCCTG 3
TGAGCCTGGAAGCTGGAGCCTG 1
GGTTTACGTTGGGAGAACTTTT 4
HG000110 >hsa-mir-1303TTTAGAGACGGGGTCTTGCTCT 3
TTAGCTGCTTGTGAGCAGGGTC 1
GTGATGTGTAACTGATCAGGAT 3
HG000111 >hsa-mir-30d TGGCTCAGTTCAGCAGGAACAG
387 AGACTCGACCTCCCAGGCTTAA
HG000111 >hsa-mir-222 TCAGTAGCCAGTGTAGATCCTG 3
TGAGCCTGGGAGGTCAAGGCTG 1 TTGAGCCTGGGAGGTCAAGGCT 1
ATCTCGACCGGACCTCGACCGG 1
HG000111 >hsa-mir-3909TGTCCTCTAGGGCCTGCAGTCT 2
TGTGTAACTGATCAGGATCTAC 3 GATGTGTAACTGATCAGGATCT 3
CCTGTACTGAGCTGCCCCGAGC 16
HG000111 >hsa-mir-590 CGGGGCTAGGGCTAACAGCAGT
1 GGGTCTTGCTCTGTTGCCAGGC 1
HG000111 >hsa-mir-500bATCCTTGCTACCTGGGTGAGAG 2

AGTGTTTAGACTATCTGTTCAG 1 TTTAGACTATCTGTTCAGGACT 1
GTCTGAGGCCCCTCAGTCTTGC 1
HG000111 >hsa-mir-744 CATGCTGTTGCCACTAACCTCA 1
ATCCTTGCTACCTGGGTGAGAG 3 AATCCTTGCTACCTGGGTGAGA 6
TTTACGTTGGGAGAACTTTTAC 104
HG000111 >hsa-mir-1273h
TCTCCTGAGCCATTCTGAGCCT 1 TTTGAGAATGATGAATCATTAG 1

HG000111 >hsa-mir-423 AAGCTCGGTCTGAGGCCCCTCA 186
AAGATGTGGAAAAATTGGAATC 1
GCCTGGAAGCTGGAGCCTGCAG 3
HG000111 >hsa-mir-1303TTTAGAGACGGGGTCTTGCTCT 2
TTAGGAGAAAGTTTCTGGAATG 6
HG000112 >hsa-mir-24-2 CTGTACTGAGCTGCCCCGAGCT
2 CCTTGCTACCTGGGTGAGAGTG 3
HG000112 >hsa-mir-658 ACTGGCCTGGGACTACCGGGGG
26 GGGTCTTGCTCTGTTGCCAGGC
HG000112 >hsa-mir-1307GACTCGGCGTGGCGTCGGTCGT 3
CTCCCACACCCAAGGCTTGCAG 3 CCTCCCACACCCAAGGCTTGCA 30

HG000112 >hsa-mir-1273h TGCTGCAGACTCGACCTCCCAG 1
TGCAGACTCGACCTCCCAGGCT 25 CTGCAGACTCGACCTCCCAGGC 26

HG000112 >hsa-mir-590 TGTAATTTTATGTATAAGCTAG 1
TGCAGACTCGACCTCCCAGGCT 67 CTGCAGACTCGACCTCCCAGGC 60

HG000112 >hsa-mir-423 AAGCTCGGTCTGAGGCCCCTCA 291
AAGATGTGGAAAAATTGGAATC 19
TACCCTCATTGGCTCAGTAGCC
HG000112 >hsa-mir-30d AGCTTTCAGTCAGATGTTTGCT 1
AGGGCTTCTGAGCTCCTTAGCA 1
GCCTGGAAGCTGGAGCCTGCAG 6
HG000112 >hsa-mir-3620CTGCATCCCGCACCCAG 1 CTCACTGTAGCATCGAACCCCT
17
HG000112 >hsa-mir-3188GAGGCTTTGTGCGGATACGGGG 6
GAGGAGATGGCGCAGGGGACAC 4
HG000112 >hsa-mir-500bGCAGTGCACCCAGGCAAGGATT 1
GTAGATAAAATATTGGTACCTG 31
HG000112 >hsa-mir-1303TTTAGAGACGGGGTCTTGCTCT 7
TTAGGAGAAAGTTTCTGGAATG 6
HG000114 >hsa-mir-1303TTTAGAGACGGGGTCTTGCTCT 1
GAAATTATTGTACATCGGATGA 2 CCTGTACTGAGCTGCCCCGAGC 4

HG000114 >hsa-mir-1254-2
CCTCCCACACCCAAGGCTTGCA 3 CCTTGCTACCTGGGTGAGAGTG 6

HG000114 >hsa-mir-196a-2

CTGTACTGAGCTGCCCCGAGCT 3 CTGGCTCAGTTCAGCAGGAACA 6

HG000114 >hsa-mir-222 TCAGTAGCCAGTGTAGATCCTG 4
TGAGCCTGGGAGGTCAAGGCTG 1 TTGAGCCTGGGAGGTCAAGGCT 1
GTCGTGTTCACAGTGGCTAAGT 1
HG000114 >hsa-mir-629 TCTGGCTCCGTGTCTTCACTCC
1 TGACCCCGTGCCACCCTTTTCC 1 77
HG000114 >hsa-mir-573 ACTGGCCTGGGACTACCGGGGG
7 TGGGTTTACGTTGGGAGAACTT 110
HG000114 >hsa-mir-486-2
TAGGTCGGTTGGTCGGTCGGGA
HG000114 >hsa-mir-30d

HG000114 >hsa-mir-1304

HG000114 >hsa-mir-1273h

HG000115 >hsa-mir-629 AGGTAGTTTCATGTTGTTGGGA
1 ACAACAGTGCCAACCTCACGGG 1
HG000115 >hsa-mir-222 TCAGTAGCCAGTGTAGATCCTG 6
TGAGCCTGGGAGGTCAAGGCTG 3 TTGAGCCTGGGAGGTCAAGGCT 1
CCTTGCTACCTGGGTGAGAGTG 2 81
HG000115 >hsa-mir-27a GTGTTCACAGTGGCTAAGTTCC 1
TAAGGGGACCAAAGAGATATAT 1 TTAAGGGGACCAAAGAGATATA 1
GGCTGCGGAATTCAGGACAGTG 2
HG000115 >hsa-mir-3652 CTGTACTGAGCTGCCCCGAGCT
1 TCGGCTCCTCGCGGCTCGCGGC 1
HG000115 >hsa-mir-1303TTTAGAGACGGGGTCTTGCTCT 24
GAAATTATTGTACATCGGATGA 1 TCGGCTCCTCGCGGCTCGCGGC 1

HG000115 >hsa-mir-196a-2
GCCTGGGAGGTCAAGGCTGTAG 1 TGCAGTGCACCCAGGCAAGGAT 1

HG000115 >hsa-mir-642a CTGTACTGAGCTGCCCCGAGCT
2 TGGGACTTCTGGCCTTGACTTG 3
HG000115 >hsa-mir-663aTCCCAGGCGGGGCGCCGCGGGA 2
TGAGCCTGGGAGGTCAAGGCTG 9 TTGAGCCTGGGAGGTCAAGGCT 7
TTTACGTTGGGAGAACTTTTAC 389 GGTTTACGTTGGGAGAACTTTT
HG000115 >hsa-mir-1304TCTCACTGTAGCATCGAACCCC 8
TGCAGACTCGACCTCCCAGGCT 53 CTGCAGACTCGACCTCCCAGGC 46

HG000115 >hsa-mir-1254-2 TGGAAGCTGGAGCCTGCAGTGA 1
TGCAGACTCGACCTCCCAGGCT 52 CTGCAGACTCGACCTCCCAGGC 60

HG000115 >hsa-mir-944 AAATTATTGTACATCGGATGAG 2
AAACCACTTTCTTTGCTCATCC 1
GGTAGTTTCATGTTGTTGGGAT 2

HG000115 >hsa-mir-423 AAGCTCGGTCTGAGGCCCCTCA 267
AATCTGTAATTTTATGTATAAG 1
GCCTGGAAGCTGGAGCCTGCAG 6
HG000115 >hsa-mir-3144AAGGGGACCAAAGAGATATATA 1
AGACACATTTGGAGAGGGAACC 2
GCCTGGAAGCTGGAGCCTGCAG 16
HG000115 >hsa-mir-1307ACCGGACCTCGACCGGCTCGTC 2
AGACACATTTGGAGAGGGAACC 4
TGGGATGTGTCAGATAGGCAGT
HG000115 >hsa-mir-3188GAGGCTTTGTGCGGATACGGGG 3
GAGGAGATGGCGCAGGGGACAC 1
GCCTGGAAGCTGGAGCCTGCAG 8
HG000116 >hsa-mir-744 CCTCCCACACCCAAGGCTTGCA
33 CACATTTGGAGAGGGAACCTCC
HG000116 >hsa-mir-943 CCTGACTGTTGCCGTCCTCCAG 1
CCTCACCCTGCATCCCGCACCC 1 CTCACCCTGCATCCCGCACCCA 1
CTGGCTCAGTTCAGCAGGAACA 1 TTGCTTGAGCCTGGGAGGTCAA
HG000116 >hsa-mir-629 ATTGCTTGAGCCTGGGAGGTCA
1 CTTCCCCCCAGTAATCTTCATC 1
HG000116 >hsa-mir-196a-2
CTTCCCCCCAGTAATCTTCATC 1 GGCTAGGGCTAACAGCAGTCTT 1

HG000116 >hsa-mir-1303TTTAGAGACGGGGTCTTGCTCT 42
GAAATTATTGTACATCGGATGA 1 GGGAAAAGCTGGGTTGAGAAGG 1

HG000116 >hsa-mir-1273h TCCCGCACCCAG 2
TCTCAGGAGGCAGCGCTCTCAG 3
HG000116 >hsa-mir-222 TCAGTAGCCAGTGTAGATCCTG 16
TGAGCCTGGGAGGTCAAGGCTG 3 TTGAGCCTGGGAGGTCAAGGCT 2
TGGGACTTCTGGCCTTGACTTG 6
HG000116 >hsa-mir-744 CATGCTGTTGCCACTAACCTCA 2
ATCCTTGCTACCTGGGTGAGAG 3 AATCCTTGCTACCTGGGTGAGA 6
TTTACGTTGGGAGAACTTTTAC 100
HG000116 >hsa-mir-744 TATGGATGAGCAAAGAAAGTGG
1 TTTGAGAATGATGAATCATTAG 1
HG000116 >hsa-mir-1273d GAGGTTGAGGCTGCAGTGAGCC 1
GCCTGGGACTACCGGGGGTGGC 1 ACTGGCCTGGGACTACCGGGGG 12

HG000116 >hsa-mir-1273h
ACTGGCCTGGGACTACCGGGGG 11
HG000116 >hsa-mir-486-2
CTCACTGTAGCATCGAACCCCT 13
HG000116 >hsa-mir-423 AAGCTCGGTCTGAGGCCCCTCA 841
AGACACATTTGGAGAGGGAACC 4
HG000117 >hsa-mir-3620CTCACCCTGCATCCCGCACCCA 2
CCTGCATCCCGCACCCAG 1 TCCCGCACCCAG 2 ATCCCGCACCCAG4

HG000117 >hsa-mir-1254-2
TAAGTAGTTGGTTTGTATGAGA 1 CACAGTGGCTAAGTTCCGCCCC 1

HG000117 >hsa-mir-27a CCAGGCACAGTGGCTCATGCCT
1 CCTGTACTGAGCTGCCCCGAGC 1 GGCGCCGCGGGACCTCCCTCGT
HG000117 >hsa-mir-222 TCAGTAGCCAGTGTAGATCCTG 6
TGAGCCTGGGAGGTCAAGGCTG 3 TTGAGCCTGGGAGGTCAAGGCT 3
CCTGTACTGAGCTGCCCCGAGC 10
HG000117 >hsa-mir-877 TAGAGGAGATGGCGCAGGGGAC 50
TAGAGACGGGGTCTTGCTCTGT 1 ACGGGGTCTTGCTCTGTTGCCA 1
TCGGCTCCTCGCGGCTCGCGGC 1
HG000117 >hsa-mir-1273h
CTGTACTGAGCTGCCCCGAGCT 2 TCTCAGGAGGCAGCGCTCTCAG 3

HG000117 >hsa-mir-642a TGTTCACAGTGGCTAAGTTCCG
1 TGCTTGAGCCTGGGAGGTCAAG 1
HG000117 >hsa-mir-576 AATCCTTGCTACCTGGGTGAGA
4 TGGGTTTACGTTGGGAGAACTT 33
HG000117 >hsa-mir-1303TTTAGAGACGGGGTCTTGCTCT 9
AAACCACTTTCTTTGCTCATCC 1 TGTTTAGACTATCTGTTCAGGA 1

HG000117 >hsa-mir-3922 ATTGCTTGAGCCTGGGAGGTCA
1 TTCAGTCAGATGTTTGCTGCTA 1
HG000117 >hsa-mir-1343CTCCTGGGGCCCGCACTCTCGC 2
CCTGCATCCCGCACCCAG 4 TCCCGCACCCAG 5 TTTACGTTGGGAGAACTTTTAC
72
HG000117 >hsa-mir-580 GTAATTTTATGTATAAGCTAGT
10
HG000117 >hsa-mir-576 TGGCTCAGTTCAGCAGGAACAG
69
HG000117 >hsa-mir-24-2ACACTGGCTCAGTTCAGCAGGA 1
AGACACATTTGGAGAGGGAACC 4
HG000117 >hsa-mir-612 AGGGCTTCTGAGCTCCTTAGCA 3
AGGTAGTTTCATGTTGTTGGGA 1
GGTTTACGTTGGGAGAACTTTT 2
HG000117 >hsa-mir-3620CTGCATCCCGCACCCAG 4 CTCACTGTAGCATCGAACCCCT
21
HG000117 >hsa-mir-500bCTTGCTACCTGGGTGAGAGTGC 3
CTCACTGTAGCATCGAACCCCT 49
HG000117 >hsa-mir-3188GAGGCTTTGTGCGGATACGGGG 6
GAGGAGATGGCGCAGGGGACAC 3
HG000117 >hsa-mir-500bGCAGTGCACCCAGGCAAGGATT 1
GTAGATAAAATATTGGTACCTG 64
HG000117 >hsa-mir-27a GTGTTCACAGTGGCTAAGTTCC 8
TAGAGACGGGGTCTTGCTCTGT 1
CTGCGGAATTCAGGACAGTGCA 2
HG000117 >hsa-mir-629 TACGTTGGGAGAACTTTTACGG 2

TAGAGACGGGGTCTTGCTCTGT 1
TTGCTTGAGCCTGGGAGGTCAA 2
HG000117 >hsa-mir-580 TTTGAGAATGATGAATCATTAG 1
GTACTGAGCTGCCCCGAGCTGG 1
HG00096 >hsa-mir-1303 CTGTACTGAGCTGCCCCGAGCT
8
HG00096 >hsa-mir-1273h
ATTGCTTGAGCCTGGGAGGTCA 1 ACGGGGTCTTGCTCTGTTGCCA 1

HG00096 >hsa-mir-1273h CCTGGGAGGTCAAGGCTGTAGT 1
CCTCACCCTGCATCCCGCACCC 2 CTCACCCTGCATCCCGCACCCA 2
ATCCCGCACCCAG4
HG00096 >hsa-mir-1303 TGGGGGGTGGAGGCGGCTCCTG
2 CCTGTACTGAGCTGCCCCGAGC 3
HG00096 >hsa-mir-1273h
TCTGGCTCCGTGTCTTCACTCC 3 CCTTGCTACCTGGGTGAGAGTG 6

HG00096 >hsa-mir-3615CTCTCTCGGCTCCTCGCGGCTC 3
CCTGCATCCCGCACCCAG 4 TCCCGCACCCAG 4 CTCACTGTAGCATCGAACCCCT
47
HG00096 >hsa-mir-1273d GAGGTTGAGGCTGCAGTGAGCC 12
GGCTCCTCGCGGCTCGCGGCGG 1 CGGCTCCTCGCGGCTCGCGGCG 1
CTCTCACCACTGCCCTCCCACA 2
HG00096 >hsa-mir-3615CTCTCTCGGCTCCTCGCGGCTC 2
CGGACCTCGACCGGCTCGTCTG 1 CCGGACCTCGACCGGCTCGTCT 1
CTGGCTCAGTTCAGCAGGAACA 1
HG00096 >hsa-mir-635 TGAAACAATGTCCATTAGGCTT 1
TGCAGACTCGACCTCCCAGGCT 39 CTGCAGACTCGACCTCCCAGGC 41
GCCTGGGAGGTCAAGGCTGTAG
HG00096 >hsa-mir-3615CTCTCTCGGCTCCTCGCGGCTC 1
CGGACCTCGACCGGCTCGTCTG 1 CCGGACCTCGACCGGCTCGTCT 1
GGCTTAGCTGCTTGTGAGCAGG 2
HG00096 >hsa-mir-1273d GAGGTTGAGGCTGCAGTGAGCC 10
GCGGCGGGGACGGCGATTGGTC 1 CGCGGCGGGGACGGCGATTGGT 1
TCGGCTCCTCGCGGCTCGCGGC 1
HG00096 >hsa-mir-1303 GCTTAGCTGCTTGTGAGCAGGG
1 TGCAGTGCACCCAGGCAAGGAT 1
HG00096 >hsa-mir-1273h
TAAGTAGTTGGTTTGTATGAGA 1 TGCAGTGCACCCAGGCAAGGAT 1

HG00096 >hsa-mir-3615CTCTCTCGGCTCCTCGCGGCTC 1
CGGACCTCGACCGGCTCGTCTG 1 CCGGACCTCGACCGGCTCGTCT 1
TGCTTGAGCCTGGGAGGTCAAG 1
HG00096 >hsa-mir-3615CTCTCTCGGCTCCTCGCGGCTC 3
CGGACCTCGACCGGCTCGTCTG 2 CCGGACCTCGACCGGCTCGTCT 2
TGCTTGAGCCTGGGAGGTCAAG 4
HG00096 >hsa-mir-3615CTCTCTCGGCTCCTCGCGGCTC 2

CGGACCTCGACCGGCTCGTCTG 1 CCGGACCTCGACCGGCTCGTCT 1
TGCTTGAGCCTGGGAGGTCAAG 1
HG00096 >hsa-mir-1273d GAGGTTGAGGCTGCAGTGAGCC 15
GGCTCCTCGCGGCTCGCGGCGG 1 CGGCTCCTCGCGGCTCGCGGCG 1
TGCTTGAGCCTGGGAGGTCAAG 2
HG00096 >hsa-mir-663aTCCCAGGCGGGGCGCCGCGGGA 3
TGCAGACTCGACCTCCCAGGCT 33 CTGCAGACTCGACCTCCCAGGC 31
TGGCGCAGGGGACACGGGCAAA
HG00096 >hsa-mir-1273h
CGGGGCTAGGGCTAACAGCAGT 2 TGGGACTTCTGGCCTTGACTTG 4

HG00096 >hsa-mir-1303 CTGTACTGAGCTGCCCCGAGCT
6 TGGGTTTACGTTGGGAGAACTT 77
HG00096 >hsa-mir-1273d GAGGTTGAGGCTGCAGTGAGCC 10
GGAGGCAGCGCTCTCAGGACGT 4 TCAGGAGGCAGCGCTCTCAGGA 6
TGGGTTTACGTTGGGAGAACTT 107
HG00096 >hsa-mir-1273d GAGGTTGAGGCTGCAGTGAGCC 13
GGAGGCAGCGCTCTCAGGACGT 4 TCAGGAGGCAGCGCTCTCAGGA 6
TGGGTTTACGTTGGGAGAACTT 110 GTACTGAGCTGCCCCGAGCTGG
HG00096 >hsa-mir-1273d GAGGTTGAGGCTGCAGTGAGCC 18
GGCTCCTCGCGGCTCGCGGCGG 1 CGGCTCCTCGCGGCTCGCGGCG 1
TTCAGTCAGATGTTTGCTGCTA 2
HG00096 >hsa-mir-1273h
GCAAAAGTAATTGCGGTCTTTG 1 TTCAGTCAGATGTTTGCTGCTA 1

HG00096 >hsa-mir-423 GGCTACTTCACAACACCAGGGC
1 TTTGAGAATGATGAATCATTAG 1
HG00096 >hsa-mir-1255b-2
CTCACTGTAGCATCGAACCCCT 21
HG00096 >hsa-mir-663aTCCCAGGCGGGGCGCCGCGGGA 2
TGCAGACTCGACCTCCCAGGCT 57 CTGCAGACTCGACCTCCCAGGC 44

HG00096 >hsa-mir-1254-2 TGGAAGCTGGAGCCTGCAGTGA 1
TGCAGACTCGACCTCCCAGGCT 92 CTGCAGACTCGACCTCCCAGGC 84

HG00096 >hsa-mir-320eGAAAAGCTGGGTTGAGAAGGT 1
CTCACTGTAGCATCGAACCCCT 25
HG00096 >hsa-mir-1273d GAGGTTGAGGCTGCAGTGAGCC 17
GGAGGCAGCGCTCTCAGGACGT 1
CCTGTACTGAGCTGCCCCGAGC 3
HG00096 >hsa-mir-564 TCAGCAGGCAACATGGCCGAGA 1
TCCGGCGTCCCAGGCGGGGCGC 3
HG00096 >hsa-mir-663aTCCCAGGCGGGGCGCCGCGGGA 1
TGAGCCTGGGAGGTCAAGGCTG 1
GTACTGAGCTGCCCCGAGCTGG 2
HG00096 >hsa-mir-663aTCCCAGGCGGGGCGCCGCGGGA 1
TGAGGTTGAGGCTGCAGTGAGC 1

TCCTTGCTACCTGGGTGAGAGT 7
HG00096 >hsa-mir-663aTCCCAGGCGGGGCGCCGCGGGA 1
TGAGGTTGAGGCTGCAGTGAGC 1
GCCTGGAAGCTGGAGCCTGCAG 8
HG00097 >hsa-mir-149 ATTGCTTGAGCCTGGGAGGTCA
1 ACGGGGTCTTGCTCTGTTGCCA 1
HG00097 >hsa-mir-637 CCTCCCACACCCAAGGCTTGCA
15 AGAATGATGAATCATTAGGTTC
HG00097 >hsa-mir-658 CTCACTGTAGCATCGAACCCCT
30 CAATGCCTACTGGCCTAAGAAA
HG00097 >hsa-mir-24-2 CTGTACTGAGCTGCCCCGAGCT
3 CCTGTACTGAGCTGCCCCGAGC 7
HG00097 >hsa-mir-1273h
ATTGCTTGAGCCTGGGAGGTCA 2 TGCTTGAGCCTGGGAGGTCAAG 2

HG00097 >hsa-mir-222 TCAGTAGCCAGTGTAGATCCTG 1
TGAGCCTGGGAGGTCAAGGCTG 4 TTGAGCCTGGGAGGTCAAGGCT 3
TTTACGTTGGGAGAACTTTTAC 28
HG00097 >hsa-mir-1304 TCTGGCTCCGTGTCTTCACTCC
1
HG00097 >hsa-mir-423 AAGCTCGGTCTGAGGCCCCTCA 391
AGACACATTTGGAGAGGGAACC 4
HG00097 >hsa-mir-1307ACCGGACCTCGACCGGCTCGTC 1
AGACACATTTGGAGAGGGAACC 5
HG00099 >hsa-mir-222 GCAGCTACATCTGGCTACTGGG 1
GGCTCCTCGCGGCTCGCGGCGG 3 CGGCTCCTCGCGGCTCGCGGCG 3
ATTGCTTGAGCCTGGGAGGTCA 11
HG00099 >hsa-mir-532 CCTCCCACACCCAAGGCTTGCA
207
HG00099 >hsa-mir-658 TAGGTCGGTTGGTCGGTCGGGA

HG00099 >hsa-mir-1273h
TGGCTCAGTTCAGCAGGAACAG 211
HG00099 >hsa-mir-423 AAGCTCGGTCTGAGGCCCCTCA 1936
AGACACATTTGGAGAGGGAACC 1
GGTTTACGTTGGGAGAACTTTT 2
HG00099 >hsa-mir-1294ACAACAGTGCCAACCTCACGGG 3
AGACACATTTGGAGAGGGAACC 1
TCCTTGCTACCTGGGTGAGAGT 3
HG00099 >hsa-mir-1304CTGTAGCATCGAACCCCTGGGC 1
CTCACTGTAGCATCGAACCCCT 28
HG00099 >hsa-mir-1303TTTAGAGACGGGGTCTTGCTCT 63
TTGCTTGAGCCTGGGAGGTCAA 1
HG00099 >hsa-mir-580 TTTGAGAATGATGAATCATTAG 1
GCCTGGAAGCTGGAGCCTGCAG 2
